# Forecasting dengue fever in Brazil: An assessment of climate conditions

**DOI:** 10.1371/journal.pone.0220106

**Published:** 2019-08-08

**Authors:** Lucas M. Stolerman, Pedro D. Maia, J. Nathan Kutz

**Affiliations:** 1 Programa de Computação Científica (PROCC)/Fiocruz, Rio de Janeiro, RJ, Brazil; 2 IMPA, Rio de Janeiro, RJ, Brazil; 3 Department of Applied Mathematics, University of Washington, Seattle, Washington, United States of America; Faculty of Science, Ain Shams University (ASU), EGYPT

## Abstract

Local climate conditions play a major role in the biology of the *Aedes aegypti* mosquito, the main vector responsible for transmitting dengue, zika, chikungunya and yellow fever in urban centers. For this reason, a detailed assessment of periods in which changes in climate conditions affect the number of human cases may improve the timing of vector-control efforts. In this work, we develop new machine-learning algorithms to analyze climate time series and their connection to the occurrence of dengue epidemic years for seven Brazilian state capitals. Our method explores the impact of two key variables—frequency of precipitation and average temperature—during a wide range of time windows in the annual cycle. Our results indicate that each Brazilian state capital considered has its own climate signatures that correlate with the overall number of human dengue-cases. However, for most of the studied cities, the winter preceding an epidemic year shows a strong predictive power. Understanding such climate contributions to the vector’s biology could lead to more accurate prediction models and early warning systems.

## Introduction

Dengue Fever is a tropical mosquito-borne viral disease present in more than 110 countries and a current threat to half of the world population [1, 2]. The dengue virus is primarily transmitted to humans through infected *Aedes aegypti* mosquitoes. This main disease vector is well adapted to urban environments, which allow viruses to spread easily through cities. In addition, local climate conditions play a critical role in the development of vector populations in major urban centers.

The first cases of dengue in Brazil date from the end of the 19^*th*^ century, and despite the elimination of the *Aedes aegypti* in 1955, the mosquito was reintroduced in the country in the 70s. A historically important outbreak occurred in 1981 in Boa Vista, in the state of Roraima, following several outbreaks in Central America involving the DENV-1 and DENV-4 serotypes [3, 4]. Since then, dengue has become one of the major public health problems in Brazil, with several epidemics reported yearly across the country. While dengue symptoms are usually limited to fever and muscle/joint pain, some develop more severe forms of the disease such as hemorrhagic fever or shock syndrome.

The proliferation of *Aedes aegypti* and the sustained transmission of dengue are influenced by a complex interplay of multi-scale factors such as the circulation of different serotypes [5, 6], the movement of infected and susceptible humans within a city [7, 8], and mosquito population size. There is also a growing body of evidence showing that local climate conditions such as temperature and precipitation may highly influence the biology of the mosquito [9–12]. Complicating our understanding is the fact that several cities exhibit an intricate alternation between epidemic and non-epidemic years. This suggests that climate conditions that favor dengue transmission are more complex than generally appreciated [13, 14].

**Fig 1:**
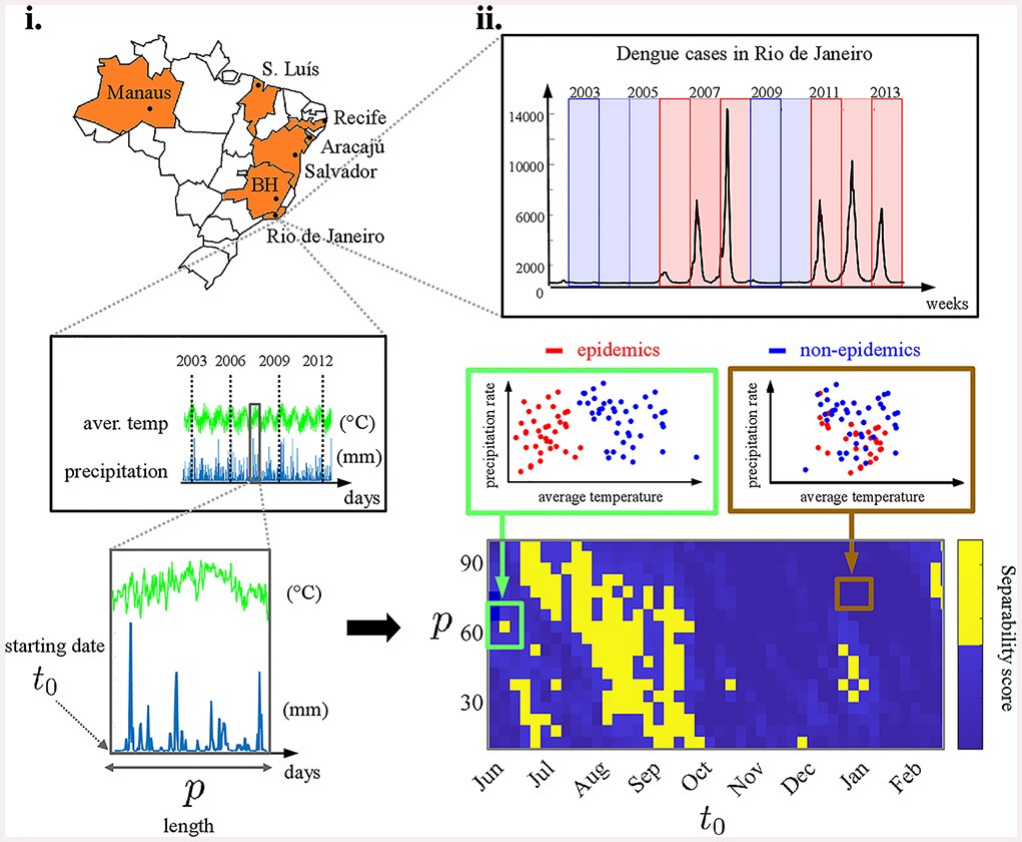
Schematic overview. We analyze time series data for climate variables from seven Brazilian state capitals (Aracajú, Belo Horizonte, Manaus, Recife, Rio de Janeiro, Salvador, and São Luís) and their connection to dengue epidemic years. **(i)** Illustrative example showing data from Rio de Janeiro. Two parameters define the epochs in which climate conditions are considered: the starting date *t*_0_ (month/day) and period length of *p* (days). **(ii)** We locate periods along the year where the separability between epidemic and non-epidemic climate is higher. Keeping track of signature differences at key epochs may significantly improve dengue forecasting in the upcoming years.

In this work, we analyze climate and epidemiological data from seven major Brazilian cities (Aracajú, Belo Horizonte, Manaus, Recife, Rio de Janeiro, Salvador and São Luís), which had epidemic and non-epidemic years in the recent past. Fig 1 is a schematic overview of our method. We estimate the correlation of climate conditions in different epochs preceding epidemic periods using a data-driven methodology based on machine learning algorithms for clustering and classification [15, 16] known as Support Vector Machines (SVM) [17, 18], which were applied to climate variables that are key to the life cycle of the mosquito. We also explore the predictability of our method combining periods of high association between climate conditions and dengue epidemics with different prediction approaches. The insights of this work may help tailor public health policies for each different city by increasing vector control measures during neglected critical epochs and ultimately improving the forecasting of dengue epidemic years—which would allow the public health system to make earlier logistic preparations or mosquito eradication programs.

## Materials and methods

### Description of epidemiological and climate datasets

All epidemiological data utilized in this work were taken from the publicly available datasets of the Brazilian Notifiable Diseases Information System (SINAN, [19]). This includes the total number of dengue cases per year (from 2002 to 2017) for all Brazilian state capitals. While we cannot be sure that all dengue cases occurred within the area measured by the climate variables, we are confident that the numbers reported are sufficient for disambiguating between a dengue and non-dengue year. A year is conventionally classified as an *epidemic* year for a given city if the incidence of dengue is above 100 cases per 100,000 inhabitants in the period January–December and classified as a *non-epidemic* year otherwise, based on the Brazilian Ministry of Health classifications of dengue incidences [20]. In order to find critical climate signatures that may have contributed to the epidemic outcomes, we restrict ourselves to seven state capitals that displayed at least 3 epidemic years and 3 non-epidemic years in the period 2002–2012. This allowed us to investigate the correlation between distinct climate conditions and the complicated alternations between epidemic and non-epidemic years over time. The climate data utilized in this work was obtained from the National Institute of Meteorology (INMET) [21] and included time series for the average temperature (in Celsius) and precipitation (in millimeters) for the state capitals Aracajú, Belo Horizonte, Manaus, Recife, Salvador, and São Luís (from 1/1/2001 to 12/31/2012) and for Rio de Janeiro (from 1/1/2002 to 12/31/2013).

### Defining periods of critical climate conditions for dengue

In this work, we investigate the correlation of climate conditions on dengue epidemics at different periods along the yearly cycle. We let (*t*_0_, *p*) denote a sampling period of *p* days starting at the date *t*_0_. Then, for a fixed period, we evaluate a score quantifying the discrepancy between climate conditions in epidemic years and non-epidemic years. See Fig 1 for an illustrative example using data from the city of Rio de Janeiro: periods with high climate *separability* between epidemic years (red dots) and non-epidemic years (blue dots) might be of critical importance to the cycle of the urban mosquito population and consequently, to the occurrence of dengue in the following year.

In what follows, we define the *SVM scores* as a proxy for the cluster separability. Our method highlights potentially critical periods for the occurrence of dengue. Finally, since dengue outbreaks in Brazil typically take place between March–May in a given year, we limit the range of (*t*_0_, *p*) from June (of the previous year) to May.

### SVM scores for cluster separability

**Fig 2:**
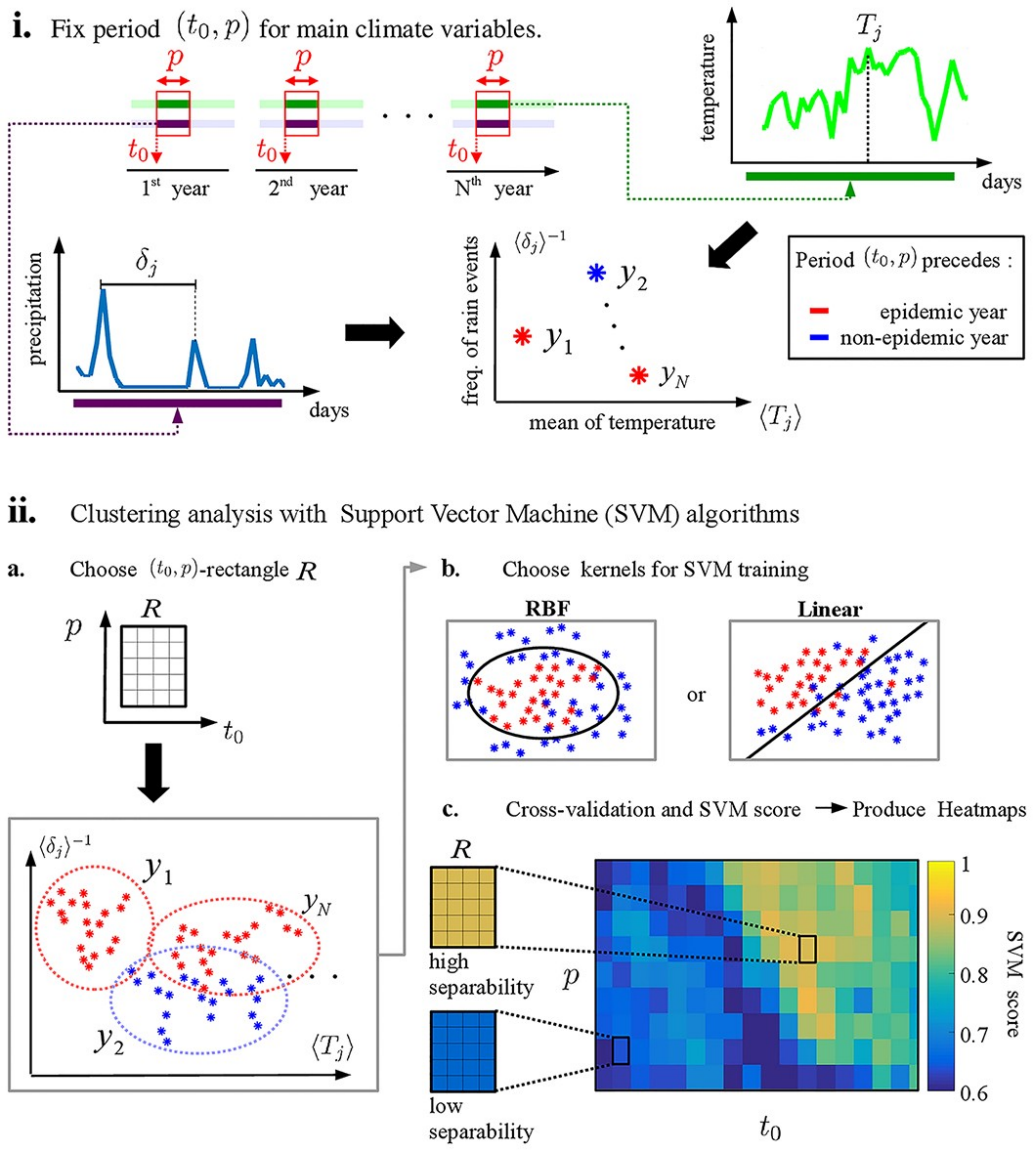
Outline of SVM methodology. A supervised learning technique for classification: **(i)** We calculate and plot mean of average temperature 〈*T*_*j*_〉 and frequency of rain events 〈*δ*_*j*_〉^−1^ for a fixed (*t*_0_, *p*) interval of all years, using red and blue colors or periods preceding epidemic and non-epidemic years respectively. **(ii)(a)** For each (*t*_0_, *p*) interval of the rectangle *R* (called (*t*_0_, *p*)-rectangle), we apply (i) to obtain a *cloud* (dashed circles) of points in the plane, for each year. **(b)** Linear and RBF kernels are used to execute the SVM train/test and cross-validation routines. **(c)** the SVM score for *R* is obtained. We plot *t*_0_ × *p* heatmaps with Regions of High and Low SVM scores, which indicates where temperature and precipitation are better correlated with the occurrence of dengue.

Our SVM score for measuring discrepancies between climate conditions in epidemic/non-epidemic years is based on a supervised learning technique for classification. Fig 2 outlines the main steps of our SVM algorithm: (i) For a fixed (*t*_0_, *p*) interval, we evaluate two climate indicators—the arithmetic mean of the average temperature 〈*T*_*j*_〉 and average frequency of rain events 〈*δ*_*j*_〉^−1^, where *δ*_*j*_ represents time intervals between consecutive peaks on precipitation data (see Fig 2i). We find the precipitation local maxima in the time series using Matlab’s *findpeaks* function, calculate the time intervals *δ*_*j*_ between them, and define the precipitation rate as the average peak interval. No specific thresholds were used in this step. (ii) We label the climate indicators in a 2D plot as an epidemic year (red) or as a non-epidemic year (blue) according to our dengue outbreak criteria. (iii) We repeat the process for *t*_0_ and *p* within a rectangular range *R* in the parameter space. Then we have a collection of red/blue points (dashed ellipses in Fig 2iia). In our simulations, the rectangular range R was 5 × 6, i.e, spanning 5 consecutive starting dates (*t*_0_) and 6 consecutive duration lengths (*p*). In this work we use both Linear and Radial Basis Function (RBF) kernels for the SVM training step on the (*t*_0_, *p*)-rectangles *R*. We cross-validated the climate indicators (red/blue dots) in the *t*_0_ × *p* period by subsampling 80% of the dataset and testing the classification accuracy in the remaining 20%. We evaluated the percentage of correctly classified test points and define the *SVM score* as the average accuracy after re-sampling the training/test data for 100 trials. No normalizations steps were used within the SVM steps, i.e., the climate indicators were simply the average temperature values and precipitation rates. Finally, we plot heatmaps (Fig 2iic) of the SVM scores for different (*t*_0_, *p*)-rectangles within a range of *t*_0_ and *p* values. We remark that high/low SVM scores are consistently associated with separable/overlapping clusters of epidemic vs non-epidemic points (red vs blue dots). Thus, the SVM score is a good proxy for the geometrical separability of the clusters. We postulate that periods with high SVM scores might be of critical importance to the cycle of the urban mosquito population and consequently, to predict the occurrence of dengue in a given out-of-sample test year. The *t*_0_ values range from June 1^*st*^ to February 21^*st*^ and *p* ranges from 10–100 days (except for Rio de Janeiro, ranging from 5–100 days), which completely covers (from June 1^*st*^ to May 31^*st*^) the periods that may influence dengue outbreaks.

The down-selection to the two parsimonious variables is consistent with well established and commonly used techniques such as LASSO and model/variable selection through information criteria such as AIC (Akaike Information Criteria) and BIC (Bayesian Information Criteria). These methods specifically penalize the number of predictive terms so that a parsimonious model is selected. In the application here, the two variables selected generalize their predictive power across all the different cities despite the different specific patterns of clustering (See Supplementary Information). More broadly, the down selection is consistent with the philosophy of the Pareto optimal solution, or Occam’s razor: explain the majority of observed data with an interpretable, parsimonious model. See S1 Appendix for details.

### Out-of-sample prediction

**Fig 3:**
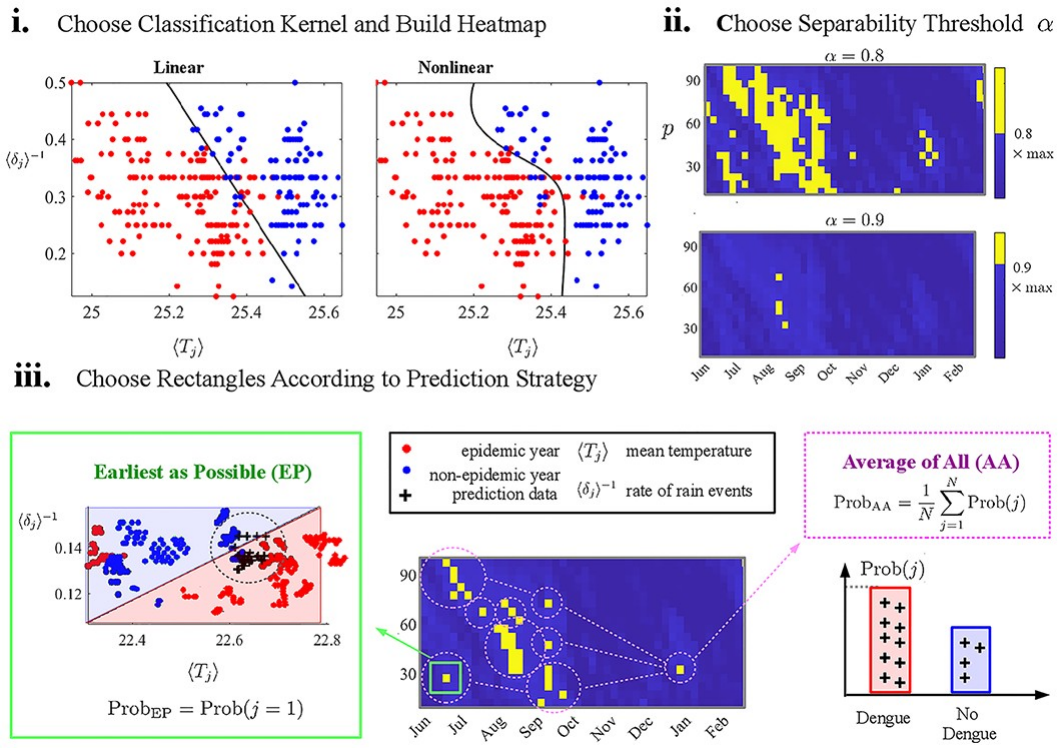
Outline of the prediction method. For each state capital, we calculate the dengue probability for an out-of-sample year using the remaining 10 years as a training set: the user (**i**) chooses between a linear/nonlinear (RBF) classification kernel to build a heatmap of SVM_score_ for a wide range of *t*_0_ and *p* values, (**ii**) selects (*t*_0_, *p*) rectangles with SVM_score_ ≥ *α* × max(SVM_score_) for a threshold parameter *α*, and (**iii**) computes the probability of dengue occurrence in the testing year using the *Earliest as Possible (EP)* strategy or the *Average of All (AA)* strategy. EP uses only the first selected rectangle (boxed in green) while AA takes an average of the probabilities of all selected rectangles (circled in magenta). See text for details.

Our training dataset for each state capital consists of 11 years (2002–2011) of temperature and precipitation time series. Due to the small number of years available and due to methodological constraints (that require a certain number of both epidemic and non-epidemic years in the training set), we can select 10 years for training and test/predict the remaining out-of-sample year with a few different strategies. This is effectively a *leave-one-out* cross-validation procedure enforced by the limited number of years in the dataset. Ideally, one would like to use a more sophisticated cross-validation procedure, but most other methods require substantially more data, i.e. number of dengue versus non-dengue years. Fig 3 illustrates the steps below:

1. **Choose SVM kernel and compute heatmap:** The user should choose between a linear/nonlinear (RBF) kernel to classify the climate data in the 〈*T*_*j*_〉 × 〈*δ*_*j*_〉^−1^ plane. See Fig 3i for an example. This classifier will provide an SVM score (color-coded in the heatmap) for each (*t*_0_, *p*)-rectangle.
2. **Choose the SVM threshold** *α*: Once the SVM heatmap is ready, we must select the (*t*_0_, *p*) rectangles that will be used to predict the testing year. We introduce a threshold parameter *α* ∈ [0, 1] and pick rectangles with SVM score ≥ *α* × max(SVM_score_). Fig 3ii shows that higher values of *α* diminishes the number of selected rectangles in the *t*_0_ × *p* plane.
3. **Choose a prediction strategy:** Fig 3iii illustrates the last choice needed to compute the probability of dengue occurrence in the testing year.
  - *Earliest as Possible (EP)*: this strategy uses the rectangle in the *t*0 × *p* plane with earliest *t*_0_, and in case of a tie, it chooses the one with the lowest *p*. We denote the index of this rectangle as j = 1 (see Fig 3). It then computes the dengue probability, denoted by Prob(*j* = 1), as the fraction of those test climate data points that fall into the dengue 〈*T*_*j*_〉 × 〈*δ*_*j*_〉^−1^ semi-space. We address the evaluated quantity as the EP probability
    $$\begin{array}{c} {\text{Prob}}_{\text{EP}}=\text{Prob}\left(j=1\right). \end{array}$$ (1)
  - *Average of All* (AA): this strategy computes the probability of dengue occurrence in the testing year using all *N* selected (*t*_0_, *p*) rectangles in step (ii) and taking an average of their probabilities. We address the AA probability as
    $$\begin{array}{c} {\text{Prob}}_{\text{AA}}=\frac{1}{N}\sum_{j=1}^{N}\text{Prob}\left(j\right). \end{array}$$ (2)

Predicting the dengue outcome of an out-of-sample year requires choosing (i) a classification kernel (linear vs nonlinear), (ii) a threshold *α* value (0.9, 0.95 or 1), and (iii) a strategy for calculating the probability of dengue occurrence (EP vs AA). Probability values above/below 0.5 led to epidemic/ non-epidemic predictions, respectively.

The results were then summarized in *confusion matrices* containing all four types of correct/wrong predictions: True Positives (TP), True Negatives (TN)/False Positives (FP), and False Negatives (FN). Our prediction accuracy

$$\begin{array}{c} \text{Accuracy}=\frac{\text{TP}+\text{TN}}{\text{TP}+\text{FP}+\text{FN}+\text{TN}} \end{array}$$ (3)

was the outcome was the outcome measure by which we compared the different prediction methods.

## Results

**Fig 4:**
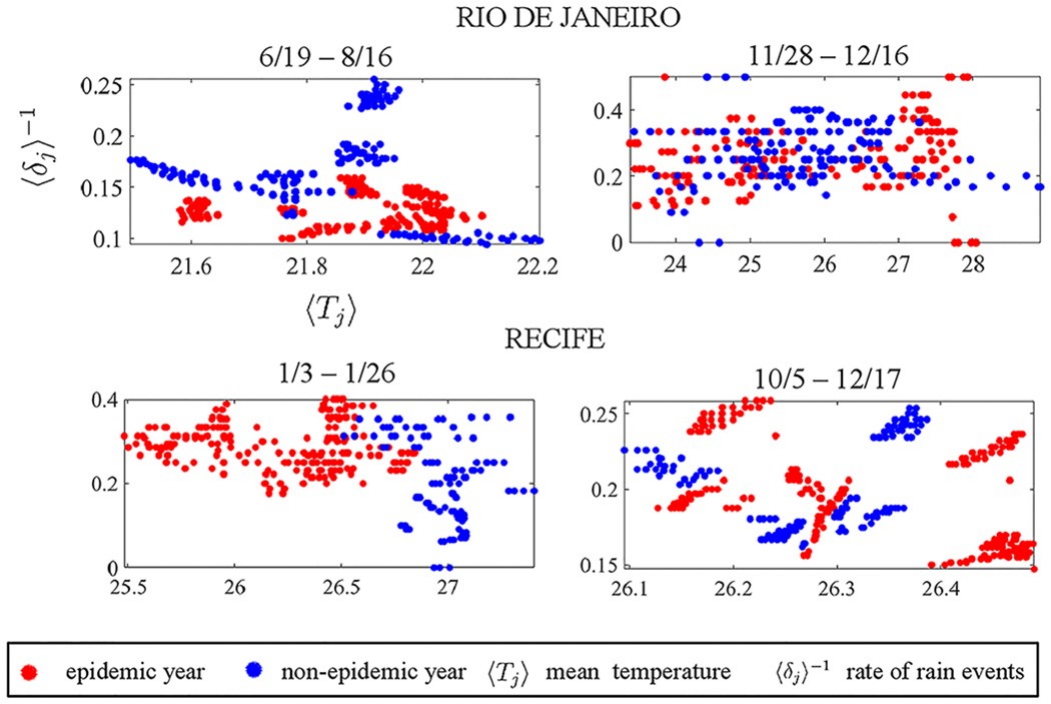
Examples of high and low cluster separability plots with the full training dataset. For each state Capital, we selected special time windows in which there was a clear separation between climate signatures preceding epidemic and non-epidemic years. This picture illustrates the cases of Rio de Janeiro and Recife. The Left side of the panel shows distinct data separation, while in the right side the climate variables seem to be poorly distinguishable, therefore not suitable for dengue prediction. This separability notion is made quantitatively precise by the SVM scores (see text for details). Examples for the other capitals can be found in the S1 Appendix.

In this section, we highlight significant differences between climate conditions during epidemic/non-epidemic years for a period starting at day *t*_0_ and duration of *p* days along the yearly cycle. Before delving into our dengue prediction results, it is highly informative to interpret high/low SVM scores for distinguishing epidemic and non-epidemic correlations. Fig 4 demonstrates the clustering of data, or lack thereof, for Rio de Janeiro and Recife, considering all 11 years of training data (Figs C and D in the S1 Appendix show similar results for all state Capitals). The left side of the panel shows representative data for time windows achieving a high correlation score. Remarkably, the red (epidemic) and blue (non-epidemic) dots are well separated and distinguishable by visual inspection. On the other hand, the right side shows data structures with low correlation scores. Note the significant overlap between the red and blue dots, suggesting that using this region for prediction of an epidemic is highly suspect. This result illustrates that each city has a unique pattern of clustering that can be capitalized on in order to provide predictive metrics for dengue epidemic years.

### Validation of the training dataset (2002–2012)

The different choices in (i) SVM kernel (linear or RBF), (ii) SVM threshold *α* (0.9, 0.95 or 1), and (iii) prediction strategy (EP or AA) lead to 12 possible confusion matrices for each state capital. We report the best choices for each city in Table 1 and leave the full report of our results for the SI. For the 5 state capitals where the EP strategy had best results, we found their respective *EP-windows*, i.e., a median date-range that comprises all EP-chosen rectangles used in the prediction. For state capitals where the AA strategy performed better, we highlighted the *AA-months* that were common to all out-of-sample predictions. Finally, we showed specific climate signatures for state capitals with good EP predictions. In what follows, we compute the heatmaps as described in the methods section (see also Fig 2). Here we present the best prediction results for Rio de Janeiro and Salvador and leave the details for the other capitals in the S1 Appendix.

**Table 1:** Best prediction results: Training set. We report the choices of SVM kernel, threshold, and strategy that resulted in highest prediction accuracy for each state capital, along with their respective EP-Windows or AA-months. *Similar results were found with the AA-strategy for Belo Horizonte. **Both strategies gave good results for Salvador. See text and S1 Appendix for details.

Capital	Kernel	*α*	Strategy	Accuracy	EP-Window/AA-months
Aracajú	RBF	0.9	EP	91%	Jun 1–19
Belo Horizonte	RBF	1	EP	73%*	Jun 13–Aug 25
Manaus	Linear	0.95	AA	64%	Aug–Oct
Recife	Linear	1	AA	82%	Dec–Jan
Rio de Janeiro	RBF	1	EP	82%	Jun 19–Sept 25
Salvador	Linear/RBF	0.9/0.95	EP/AA	73%/82%	Aug 30–Dec 11**
São Luís	RBF	1	AA	82%	Dec–Mar

#### Rio de Janeiro

Fig H in the S1 Appendix shows the best prediction result for Rio de Janeiro using (i) an RBF kernel, (ii) an SVM threshold of *α* = 1, and (iii) the EP-strategy to calculate the outbreak probability. Most EP-chosen rectangles occurred in the winter and in the spring. The corresponding *EP*-window ranged between June 19^*th*^ and September 25^*th*^, when most Epidemic years (all except 2012) had average temperatures above 23 Celsius and precipitation rates below 0.15 (see prediction tables at the S1 Appendix for details). All years except 2010 (FP) and 2012 (FN) were correctly predicted (82% accuracy).

#### Salvador

Fig I (**top**) in the S1 Appendix shows the best prediction result for the city of Salvador using (i) an RBF kernel, (ii) an SVM threshold of *α* = 0.95, and (iii) the AA-strategy to calculate the outbreak probability. The (*t*_0_, *p*) rectangles used in the prediction covered most of the year but were especially clustered around December-February (boxed in magenta). All years except 2002 (FN) and 2010 (FN) were correctly predicted (82% of accuracy).

Predictions using (i) a linear kernel, (ii) *α* = 0.9, and (iii) the EP-strategy also gave good results (highlighted in Fig I (**bottom**) in the SI appendix). Eight years were correctly predicted (73% accuracy) but the years of 2008 (FP), 2010 (FN) and 2012 (FN) were not. The EP strategy was just slightly less accurate than the AA strategy, yielding EP-windows within August 30^*th*^ and December 11^*th*^ (spring and summer). The epidemic years typically showed lower precipitation rates in the selected EP-rectangles.

#### Other Capitals

Each state Capital has an optimal choice of SVM kernel, *α* value and prediction strategy, as Table 1 shows. In the S1 Appendix, we present the different prediction results for Aracajú, Belo Horizonte, Manaus, Recife and, São Luís. See Figs E, F, G, and J in the S1 appendix for a comprehensive description of their prediction outcomes.

#### SVM classification and climate signatures

**Fig 5:**
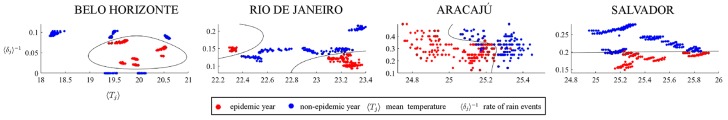
Favorable climate conditions for epidemics in predictive EP-periods. The EP strategy uses the rectangle in the *t*_0_ × *p* plane with earliest *t*_0_. Four capitals exhibited highly predictive EP rectangles, and we show the corresponding epidemic vs non-epidemic climate conditions. Belo Horizonte: EP-window from June 13th to August 25th. Most epidemic years had a precipitation rate in the interval [0.02,0.08] and different clusters were separated by an ovoid-shape kernel. Rio de Janeiro: EP-window ranged between June 19th and September 25th. Most epidemic years had average temperatures above 23° Celsius and precipitation rates below 0.15. Clusters were separated by an hyperboloid-shape kernel. Aracajú: EP-window from June 1st–19th. There is a clear separability between dengue and no-dengue regarding a temperature threshold around 25.2° Celsius. Clusters were separated by an S-shape kernel. Salvador: EP-windows from August 30th–December 11th. Clusters were separated by a single linear threshold of 〈*δ*_*i*_〉^−1^ below 0.2. The picture shows climate signatures considering training years 2003–2012 for Rio de Janeiro and 2002–2011 for the other capitals.

Fig 5 shows the corresponding favorable climate conditions for all capitals with predictive EP-periods. The EP prediction strategy uses only one rectangle from the *t*_0_ × *p* heatmap, i.e., the one with the lowest *t*_0_. This allows us to show the specific temperature and rain values that distinguished epidemics and non-epidemic years in that EP window. Contrastingly, the AA strategy averages over several rectangles throughout the entire year, making the analysis of specific climate conditions for each window impractical. The EP rectangles occur in June (winter) for the first three capitals and in the spring/summer for Salvador. The classifiers (curves in black) take very distinct shapes for the different cities. For Belo Horizonte, the different clusters were separated by an ovoid-shape kernel and most epidemic years had a precipitation rate between [0.02,0.08]. For Rio de Janeiro, most epidemic years have average temperatures above 23° C and the clusters are separated by an hyperboloid-shape kernel. In Aracajú, an S-shape kernel separates the clusters around a temperature threshold of 25.2° C. Finally, favorable climate conditions for dengue epidemics occur in Salvador during the spring for a frequency of rain events below 0.2. It is hard to infer specific relationships between optimal temperature and rain events however, because the cities have significantly different sizes, geography, vegetation, topography and other factors that might impact the mosquito development. Rio de Janeiro, for instance, exhibits a vast array of sub-regions ranging from highly-populated urban centers to forests [22].

### Predictions for the holdout dataset (2013–2017)

We used the model trained with data from earlier years 2002-2012 to predict dengue outcomes in a holdout dataset (usually from 2013-2017, but may vary depending on data availability). See S1 Appendix for details. It should be noted that approximately seven months after submission of the manuscript, SINAN released new dengue data for the years 2013-2017 [19]. Table 2 shows the accuracy for each state capital using the corresponding kernel, parameters, and strategy defined in the training step. The state capital of São Luís exhibited the best accuracy (100% corresponding to 3 correct predictions from a total of 3 test years), followed by Manaus and Salvador (80% accuracy corresponding to 4 correct predictions from a total of 5 test years). For Rio de Janeiro, Aracajú, Belo Horizonte and Recife, we obtained accuracies below 70%. Overall, we obtained a 74% accuracy considering the predictions from the 7 state capitals, correctly predicting the outcome of 23 out of 31 experiments.

**Table 2:** Best prediction results: Holdout dataset. *We obtained 80% accuracy (4/5) with *α* = 0.9 for both EP and AA strategies and both RBF and linear kernels. ** We obtained 100% accuracy (5/5) with *α* = 0.95, EP strategy and RBF kernel.

Capital	Accuracy
Aracajú	60% (3/5)
Belo Horizonte	60% (3/5)
Manaus	80% (4/5)
Recife	60% * (3/5)
Rio de Janeiro	67% (2/3)
Salvador	80%** (5/5)
São Luís	100% (3/3)
**TOTAL**	**74% (23/31)**

## Discussion

Understanding how *Ae. aegypti* mosquitoes respond to climate conditions is crucial for developing climate-based early warning systems for dengue prediction. While several works report and quantify how climate may influence the mosquito development on a weekly scale [23–25], we suggest that long-term effects occurring even months before the outbreaks may also play an important role. We developed a new data-driven method, based on Support Vector Machine (SVM) algorithms to identify, in a systematic manner, a set of critical periods and climate signatures in the annual cycle that may be decisive for the development of dengue epidemic years. We applied our methods to temperature and precipitation time series data for seven state capitals in Brazil where there was a significant alternation between epidemic and non-epidemic years in the recent past. We explored a few strategies to estimate the predictive power of our method, and the most accurate results for each state capital led to interesting time periods and climate patterns associated with the occurrence of dengue epidemics.

### Critical seasons for each state capital

In accordance to other reported studies [13, 26, 27], we obtained strong evidence that the correlation between climate and epidemics varies significantly across different state capitals, thus rejecting simplistic or universal explanations involving temperature and rain precipitation in urban centers. Remarkably, the average temperature and the frequency of precipitation showed a strong predictive power throughout the winter season for the cities of Aracajú, Belo Horizonte, Manaus and Rio de Janeiro (see Table 3). As a consequence, intensifying mosquito control campaigns during the winter season may prove an interesting epidemic control strategy, especially due to the smaller size of the vector populations during that period. In Brazil, the national and local campaigns are usually restricted to spring and summer periods [28, 29]. In fact, the Brazilian government announced that a special task force for fighting mosquitoes was to be formed November 3^*rd*^, 2016 [30]. We believe this starting date to be too late since critical climate conditions were detected in some cities even 9 months prior to epochs with higher dengue incidence.

**Table 3:** Season highlights. We found that each state capital has its own preferred seasons in which climate signatures may impact Dengue occurrence. Peaks of dengue happen typically during the fall (March–May).

Capital	Winter	Spring	Summer
Aracajú	×		
Belo Horizonte	×		
Manaus	×	×	
Recife			×
Rio de Janeiro	×	×	
Salvador		×	×
São Luís			×

### Appending new data and updating our method

**Fig 6:**
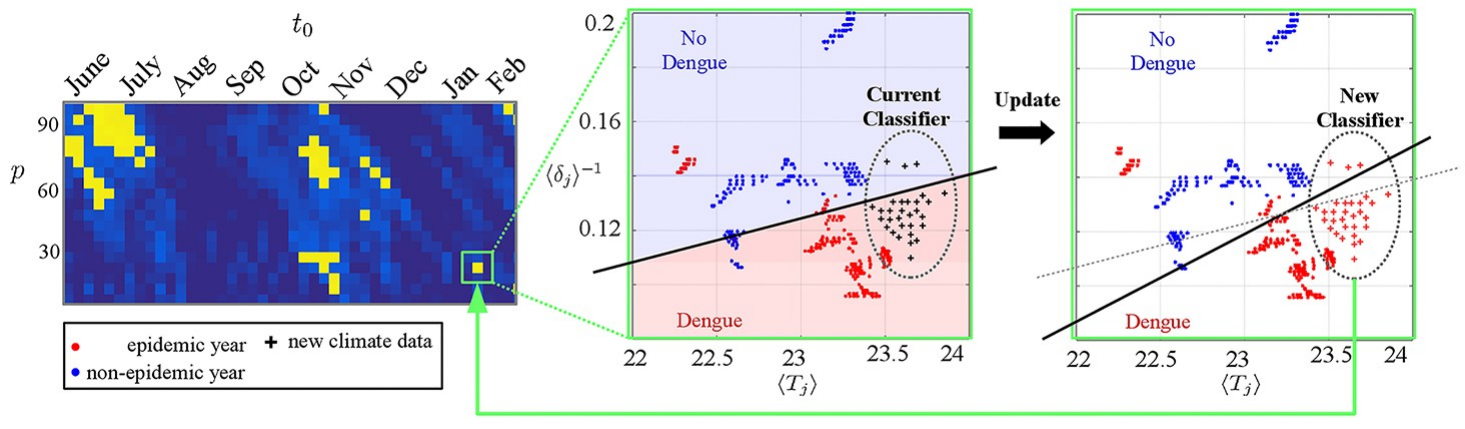
Appending data for further analysis. For a high scored (*t*_0_, *p*)-rectangle (green box), we plot the respective climate indicators with their epidemic/non-epidemic (red/blue) labels. A classifier is used to predict the outcome of newly available climate data (black crosses). Depending on the outcome, the new data is appended to the SVM-training set. This procedure will also update the SVM score and the importance of the chosen (*t*_0_, *p*)-rectangle for dengue prediction.

Our training sets and our classifiers used in intermediate methodological steps should be *updated* as new climate/epidemic data are made available. See Fig 6 for a schematic representation of how new climate data (black crosses) should be assimilated by the training dataset to improve the separability within the SVM heatmap and increase the statistical robustness of our prediction method. Thus, our method and its accuracy should be continuously updated in time to provide more reliable separability regions and accurate climate-based forecasts.

### Impacts of climate variables on the *Aedes aegypti* life cycle

Temperature and precipitation are important environmental factors affecting all biological processes of the *Ae. aegypti*. In fact, there are even precise mathematical expressions relating developmental rates with temperature [12, 31]. The rates at which mosquitoes acquire and transmit viruses are also temperature-dependent [32–35]. Precipitation events in their turn are extremely important for dengue transmission [36, 37]. The abundance of *Ae. aegypti* is regulated by rainfall during the water-dependent stages (egg, larva and, pupa), which provides breeding sites and stimulates egg hatching [38, 39].

The relations between lower temperatures, rain, and size of the mosquito population are usually studied in countries with temperate climates, where excessive rain propitiate egg hatching but the lower temperature might prove fatal for the larvae [40, 41]. The Brazilian tropical climate, however, may present adequate temperatures for vector proliferation even in the winter. Thus, we conjecture that winter rain-events may play an important role in the first mosquito generation in that year. A larger initial population, when compounded over several reproductive cycles, could lead to an epidemic outbreak in the summer. As shown in Fig 5, favorable climate conditions in Belo Horizonte, Rio de Janeiro, and Salvador are mostly a function of the rain events. The development of the mosquito population in the winter season is not a central subject of epidemic studies in tropical countries, and our work suggests that this can be a promising avenue for future studies.

Kesorn et al. (2015) [42] recently addressed a decade-long limitation of dengue surveillance systems, namely, that environmental factors can be unreliable and degrade the predictions when applied to areas with similar climate. The prediction accuracy of their model increased dramatically when, instead of using climate parameters in a classical framework, they utilized the *Ae. aegypti* female and larvae mosquito infection rates. Our work, on the other hand, was able to successfully predict dengue years using solely climate variables. This raises an important question: how reliable are climate parameters for dengue prediction? One possible explanation is that these parameters are reliable only at coarser spatial scales, and the large distances between cities in a continental country such as Brazil lead to meaningful climate differences. Another explanation is that our methodological innovations did improve the reliability of local climate factors; Kesorn et al. (2015) dismissed temperature as a good predictor by visual inspection of its time series, while we allow a wide range of time-lags linking temperature and future outcomes. It would be interesting to see if our approach could improve the reliability of climate signatures in other contexts.

Daily changes in temperature are known to affect the efficiency of the *Ae. aegypti* [43, 44]. Kesorn et al. (2015) also showed that the infection rates for the female *Ae. aegypti* and larvae correlate strongly with the number of human-reported dengue cases. On this regard, our method is agnostic as to which specific mechanisms led to an increase in the number of human cases. The factors above may be the missing link between climate variables and observed human cases. However, as also pointed out by the authors, it is not always possible to obtain data on mosquito infection rates. To the best of our knowledge, there are no available data on female and larvae mosquito infection rates for the Brazilian cities that we studied. Moreover, it would be extremely challenging to obtain a single infection rate on our spatial scale, especially for large capitals such as Rio de Janeiro and Salvador.

### Machine learning for dengue prediction

There is a broad array of methods to examine the influence of climate variables on dengue outbreaks: wavelet-analysis for time series [26], autoregressive integrated moving average (ARIMA) models [45], fuzzy association rule mining techniques [14], rule-based classifiers [46], Bayesian methods [47, 48] and others. See Racloz et al. [49] for a systematic literature review. More recently, a number of machine learning methods emerged to address the prediction of dengue outbreaks. Baqueiro et al. [50] produced a comprehensive comparison of generalized additive models (GAMs), artificial neural networks (ANNs) and seasonal autoregressive integrated moving average models (SARIMA) for the city of São Paulo. They obtained accurate predictions for dengue within a one-month time window. Our method provides larger time windows, and thus more time for implementing disease surveillance or outbreak prevention measures. In a similar study, Guo et al. [51] analyzed climate data from Guangdong, China, to forecast dengue outbreaks using support vector regression (SVR) algorithm, step-down linear regression model, gradient boosted regression tree algorithm (GBM), negative binomial regression model (NBM), least absolute shrinkage and selection operator (LASSO) linear regression models. The authors explored a four-year time series of weekly dengue cases, which can be a major issue for their prediction routine if the data is not reported in a timely fashion. Their SVR algorithm exhibited the best prediction performance with a 12-week time-window, which was also effective in other regions of China. Their results reported SVM-based models as highly predictive tools for dengue epidemics, but we provide additional plots for key climate signatures (see Fig 5). Our major methodological innovation is to frame the dengue-forecasting problem within an SVM setting that localizes the important periods for dengue prediction and their associated climate patterns. Other machine learning methods used recently include C-Support Vector Classification (C-SVC) [52], Random Forests [53], Decision Tree-Based Approaches [54] and even the curious combination of ARIMA models with Google Trends data [55]. Due to the nuanced and complex differences between the specific settings, we will leave a more detailed comparison of the methods for future works.

### Limitations of our methods

There are several limitations to our work and all of our results must be interpreted with caution and parsimony. We also acknowledge that using a binary threshold for classifying a year as epidemic/non-epidemic is somewhat arbitrary, but we decided to abide by the convention established by the Brazilian Ministry of Health. Moreover, we did not consider several other factors believed to be important for explaining dengue dynamics in details, such as circulation of different strains of the dengue virus [5, 6], human mobility within and among the cities [7, 8, 56, 57], human demographic dynamics [58, 59] and global warming and climate changes [60, 61]. Therefore it is important to acknowledge that there might be potential confounding between epidemic years and the coincidence of favorable climate conditions, given that other processes are not represented in the model.

Finally, we acknowledge that our machine learning method is agnostic as to which sequence of events was responsible for increasing/decreasing the number of human dengue cases from year to year. To be an effective vector, mosquitoes must have a high vector competence and vectorial capacity. The first refers to their ability to receive a disease agent microorganism from the reservoir host and then later transmit the infectious agent to another susceptible host. The vectorial capacity includes a number of factors like vector competence, mosquito population density, host preferences, biting rate, immunity of the mosquitoes, and others. All these factors may have been affected by the climatic differences from year to year. While we cannot disambiguate *which* changes occurred, our predictive windows along the yearly cycle may provide insight as to *when* they occurred.

### Future work

Since our methods led to promising forecasting results for various capitals of Brazil, in the future we would like to apply the same approach to other cities and climate-datasets worldwide. We also hope to better compare our methods with other machine learning techniques in future works. With respect to the definition of epidemic and non-epidemic years, we acknowledge that labeling years as dengue vs non-dengue might be too coarse and further insight might be gained by a richer categorization of epidemic years. Exploring the use of more categories or classes (such as high/medium/low years) may be an interesting approach for future studies.

## Conclusion

Epidemic control of dengue is one of the most urgent public health challenges in tropical countries such as Brazil. A better understanding of the multi-scale and long-term effects of climate conditions on the development of *Aedes aegypti* populations is crucial for improving the timing of vector-control efforts and other policies. In this work, we show that two specific climate variables—mean of temperature and frequency of precipitation—may be crucial for dengue prediction in Brazil. Remarkably, for Aracajú, Belo Horizonte, Manaus, and, Rio de Janeiro, a prediction can be made approximately six to nine months before the epidemic outbreak, which usually takes place in the months of March-May. Interestingly, the summer season in Rio de Janeiro offers little insight into this matter, since the data of years with and without dengue are qualitatively similar from a climate perspective. Yet public strategies have typically been enacted and decided during this time period, which is both too late and does not leverage the predictive capabilities of the climate data. This work also highlights that climate patterns with predictive success are quite distinct from city to city. This is large to be expected as climatic effects, such as proximity to the ocean, to the jungle/forest, dense populations, etc will likely play a significant role in how precipitation and temperature affect the growth of the disease vector *Aedes aegypti*.

## Supporting information

S1 Appendix Contains supplemental data and additional information regarding the article. (PDF)

S2 Appendix Contains supplemental data regarding Dengue incidence and missing climate data between 2013 and 2017. (PDF)

S1 File Contains data and Matlab codes. (ZIP)
