# Supplementary material for: Forecasting dengue fever in Brazil: An assessment of climate conditions
Source: PLoS One. 2019 Aug 8;14(8):e0220106. doi: 10.1371/journal.pone.0220106 (PMC6687106; doi:10.1371/journal.pone.0220106)
Supplement: S1 Appendix — (PDF) [file pone.0220106.s001.pdf]

SUPPORTING INFORMATION – Forecasting Dengue Outbreaks in Brazil: An Assessment of Climate Conditions.

Lucas M. Stolerman[‡*] Pedro D. Maia[†] J. Nathan Kutz[†]

Details about the choice of the seven capitals

As explained in our Methods section, we chose capitals that had at least 3 years with Dengue Epidemics (DE) and at least 3 years without DE in the recent past. The following 9 capitals passed this criterium: Aracajú, Belo Horizonte, Cuiabá, João Pessoa, Manaus, Recife, Rio de Janeiro, Salvador and São Luís. We completed missing data through linear interpolation and/or usage of alternative sources for precipitation time series given that the CVX routine does not work well for episodical data events. From the 9 capitals, the following 6 had only single precipitation gaps: Aracajú, Belo Horizonte, Manaus, Recife, Salvador and São Luís. The cities of Cuiabá, João Pessoa and Rio de Janeiro had big missing data epochs. For Rio de Janeiro we found an alternative source of precipitation data, but the other two capitals had to be discarded from our analysis.

Completing missing climate data via compressive sensing

The time series of our selected climate dataset contain episodic gaps on days where variables (temperature and precipitation) were not recorded. To fill in the missing data gaps, we employ two different methods: compressive sensing and interpolation (see Fig. A for illustrative examples). For temperature time series data with 2 or more consecutive missing recordings, we use a recently developed compressive sensing method based upon $\mathcal{L}^1$-convex optimization for approximating the missing data [1–5]. The compressive sensing method attempts to reconstruct a signal from a sparse, subsampling of the time series data. In this case, the sparse subsampling occurs from the fact that we have missing data. We have chosen to fill in the missing data through this *matrix completion* procedure for two reasons: (i) the mathematical methods for handling missing in this way have matured significantly over the past decade, and (ii) mixing data from two different recording devices (satellite data and ground recording data) is statistically unjustified. Specifically, the data collected by ground stations at fixed locations are the most reliable sources of climate information. Satellite data is typically noisier and has less precision, in part, due to the limitations of its resolution in comparison to ground recordings. Ultimately, using one data source as a proxy for another is its own statistically interesting, yet challenging, data science problem [6–8].

The signal reconstruction problem is nothing more than a large underdetermined system of linear equations. To be more precise, consider the conversion of a time series data to the frequency domain via the discrete cosine transform (DCT)

$$\psi \mathbf{c} = \mathbf{f} \tag{1}$$

where $\mathbf{f}$ is the signal vector in the time domain and $\mathbf{c}$ are the cosine transform coefficients representing the signal in the DCT domain. The matrix ψ represents the DCT transform itself. The key observation is that most of the coefficients of the vector $\mathbf{c}$ are zero, i.e. the time series is sparse in the Fourier domain. Note that the matrix ψ is of size $n \times n$ while $\mathbf{f}$ and $\mathbf{c}$ are $n \times 1$ vectors. The choice of basis functions is critical in carrying out the compressed sensing protocol. In particular, the signal must be sparse in the chosen basis. For the example here of a cosine basis,

*Programa de Computação Científifica, PROCC/ Fiocruz, Rio de Janeiro - RJ, Brazil.[‡] (lstolerman@eng.ucsd.edu). Questions, comments, or corrections to this document may be directed to that email address.

[†]Department of Applied Mathematics, University of Washington, Seattle, WA. 98195-2420.

the signal is clearly sparse, allowing us to accurately reconstruct the signal using sparse sampling. The idea is to now sample the signal randomly (and sparsely) so that

$$\mathbf{b} = \phi \mathbf{f} \tag{2}$$

where $\mathbf{b}$ is a few (m) random samples of the original signal $\mathbf{f}$ (ideally $m \ll n$). Thus ϕ is a subset of randomly permuted rows of the identity operator. More complicated sampling can be performed, but this is a simple example that will illustrate all the key features. Note here that $\mathbf{b}$ is an $m \times 1$ vector while the matrix ϕ is of size $m \times n$.

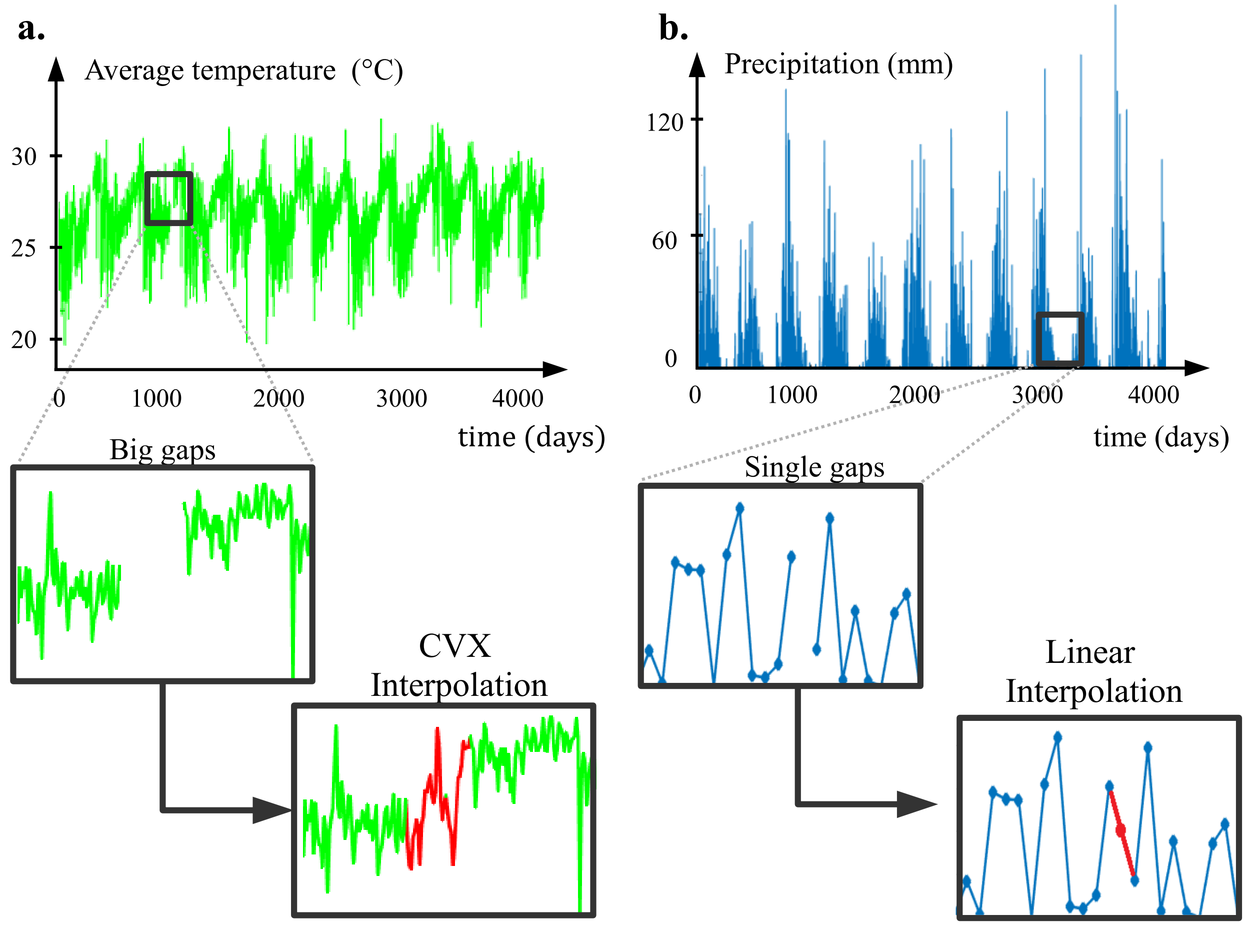

Figure A: **Completing missing data.** The daily measurements of climate variables for Brazilian state capitals from the National Institute of Meteorology (INMET) contain episodical gaps. **a.** We reconstruct larger portions of lacking data with compressed sensing ($\mathcal{L}^1$-convex optimization routines). **b.** Data values at minor holes were estimated by simpler interpolation protocols. Capitals with intractable missing portions of data were not considered.

Approximate signal reconstruction can then be performed by solving the linear system

$$\mathbf{Ax} = \mathbf{b} \tag{3}$$

where $\mathbf{b}$ is an $m \times 1$ vector, $\mathbf{x}$ is $n \times 1$ vector and

$$\mathbf{A} = \phi\psi \tag{4}$$

is a matrix of size $m \times n$. Here the $\mathbf{x}$ is the sparse approximation to the full DCT coefficient vector. Thus for $m \ll n$, the resulting linear algebra problem is highly under-determined. The idea is then to solve the underdetermined system using an appropriate norm constraint that best reconstructs the original signal, i.e. the sparsity promoting $\mathcal{L}^1$ is highly appropriate. The signal reconstruction is performed by using

$$\mathbf{f} \approx \psi \mathbf{x}\,. \tag{5}$$

If the original signal had exactly m non-zero coefficients, the reconstruction could be made exact (See Ref. [1], Ch. 18).

We applied this technique specifically to the climate series of Rio de Janeiro, Salvador and São Luís. For the other state capitals, we just linearly interpolate the time series whenever a single daily recording is missing. We note that there were intractable large gaps for the INMET precipitation series for Rio de Janeiro, which forced us to use alternative data sources made available by the city's alert system of rain events [9].

Variable Selection

Here we present a simple method based on the Singular Value Decomposition (SVD) [1] to utilize our climate date to find periods of high-separability between dengue vs non-dengue years. Our original dataset consists of the following daily measurements: (1) maximum temperature, (2) minimum temperature, (3) mean temperature, (4) humidity and (5) precipitation. These variables were not pre-selected, but instead, were the ones made available by INMET [10]. Our SVD algorithm works as follows:

1. We select climate data over the same period (t_0, p) for different years and build a corresponding matrix $X(t_0, p)$ that allows for an SVD decomposition.

2. We select data from k climate variables over the years, always starting at t0 and ending p days later.

3. We stack and normalize the data associated with year j in a block matrix $B_j(t_0, p)$, for $j = 1, \ldots, N$. All blocks are reshaped into column vectors, forming a new matrix $X = X(t_0, p)$, which yields

$$X(t_0, p) = U \Sigma V^T(t_0, p)$$

The columns of U – the SVD modes – form an orthogonal basis for the space generated by the columns of X and the projections of the principal components are given by the $\Sigma V^T(t0, p)$ matrix. For details see [11]

Figure B shows a panel with SVD-heatmaps from all state capitals considered in this study, for the same range of t0 and p values that we used in the SVM method. In the top row, we find the critical periods (highest separability between dengue vs non-dengue years) using all variables. On the bottom row, we find critical periods selecting only average temperature and precipitation. For most cities – and especially to the city of Rio de Janeiro – we can observe that these two variables detect almost the same periods of high separability in the $[t_0 \times p]$ heatmaps as detected by all variables. We highlight a few of the similar high-separability periods with green boxes (by visual inspection) to illustrate this fact. This result helps illustrate our choice of a sparse and generalizable variable set that gives a regression performance on part with using all variables.

Such an approach to a selection of a parsimonious variable set is consistent with commonly used regression techniques such as the LASSO, or with information criteria such as AIC (Akaike information criteria) and BIC (Bayes information criteria), whereby the selection of variables and goodness-of-fit is penalized by the number of total variables. Thus our methodology is consistent with well-known and commonly used techniques in the statistical sciences for variable selection.

In addition, there is the issue of generalizability. While it is true that for some cities, there might exist some particular combinations of variables that gives equal or better outcome. The city of Manaus, for example, yields a better result if one considers humidity instead of precipitation. These particular choices, however, do not generalize, thus, we decided to keep the two that are collectively a better representative of the climate dataset across all cities. Indeed, the two variables we have kept are the only two that generalize across all the cities, despite their distinct climates and clustering patterns. This generalizability argument is also consistent with LASSO and BIC/AIC model/variable selection theory [12, 13]

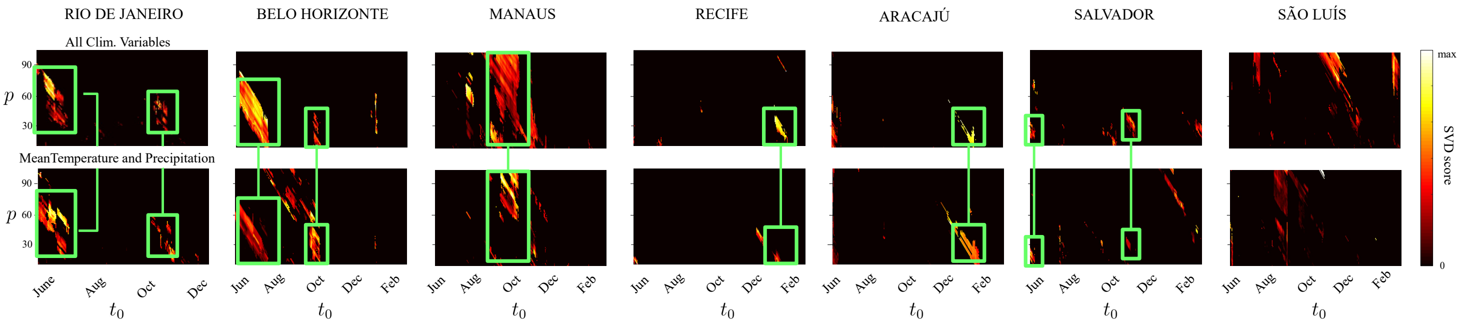

Figure B: Comparison between the use of all climate variables to detect high-separability periods (top row) vs using average temperature & precipitation alone (bottom row). Rio de Janeiro, Belo Horizonte, Aracajú, and Salvador detected very similar separability periods using this widely available and easily accessible environmental data (as highlighted by the green boxes to facilitate visual inspection). Different combinations of variables - even if they might perform better for a particular city - do not generalize to all others.

Examples of periods with high and low separability of the climate signatures

For each state Capital we selected special time windows in which there is a clear separation between climate signatures preceding epidemic and non-epidemic years. Figure C illustrates the distinct separation of the data for each individual city, suggesting that a universal model for climate effects across all cities may be unattainable. The separability of data further suggests that epidemics may be accurately predicted in a given state capital six to nine months in advance of their outbreak. This separability notion is made quantitatively precise by the SVM scores.

Figure D shows specific time windows in which the epidemic and non-epidemic climate variables seem to be poorly distinguishable, therefore not suitable for dengue prediction. Unlike Fig. C, the mixing of data suggests poor predictability across all cities. This separability notion is made quantitatively precise by the SVM scores.

Results for each Capital

Aracajú

The most accurate predictions were obtained with (i) a nonlinear RBF kernel, (ii) an SVM threshold of $\alpha = 0.9$, and (iii) using the EP-strategy to calculate the outbreak probability. Surprisingly, the *same* EP-rectangle was used in all out-of-sample predictions, giving an EP-window within June 1st – 19th (winter). Fig E highlights this rectangle (green box) and the respective results in the $\langle T_j \rangle \times \langle \delta_j \rangle^{-1}$ plane for each year. Only the year of 2006 was wrongly predicted (FP). There is a clear separability between dengue and No-dengue regarding a temperature threshold around 26°Celsius.

Belo Horizonte

Figure F highlights our best prediction results with (i) an RBF kernel, (ii) $\alpha = 1$, and (iii) using the EP-strategy. Most EP rectangles occurred during winter, yielding an EP-window within June 13th to August 25th. A total of 8 years were correctly predicted (73% of accuracy), but 2003 (FP), 2006 (FP), and 2007 (FN) were not. Most epidemic years had a precipitation rate in the interval [0.02,0.08] within the EP- chosen rectangles. The AA-strategy with the same kernel and α value also yielded 73% accuracy.

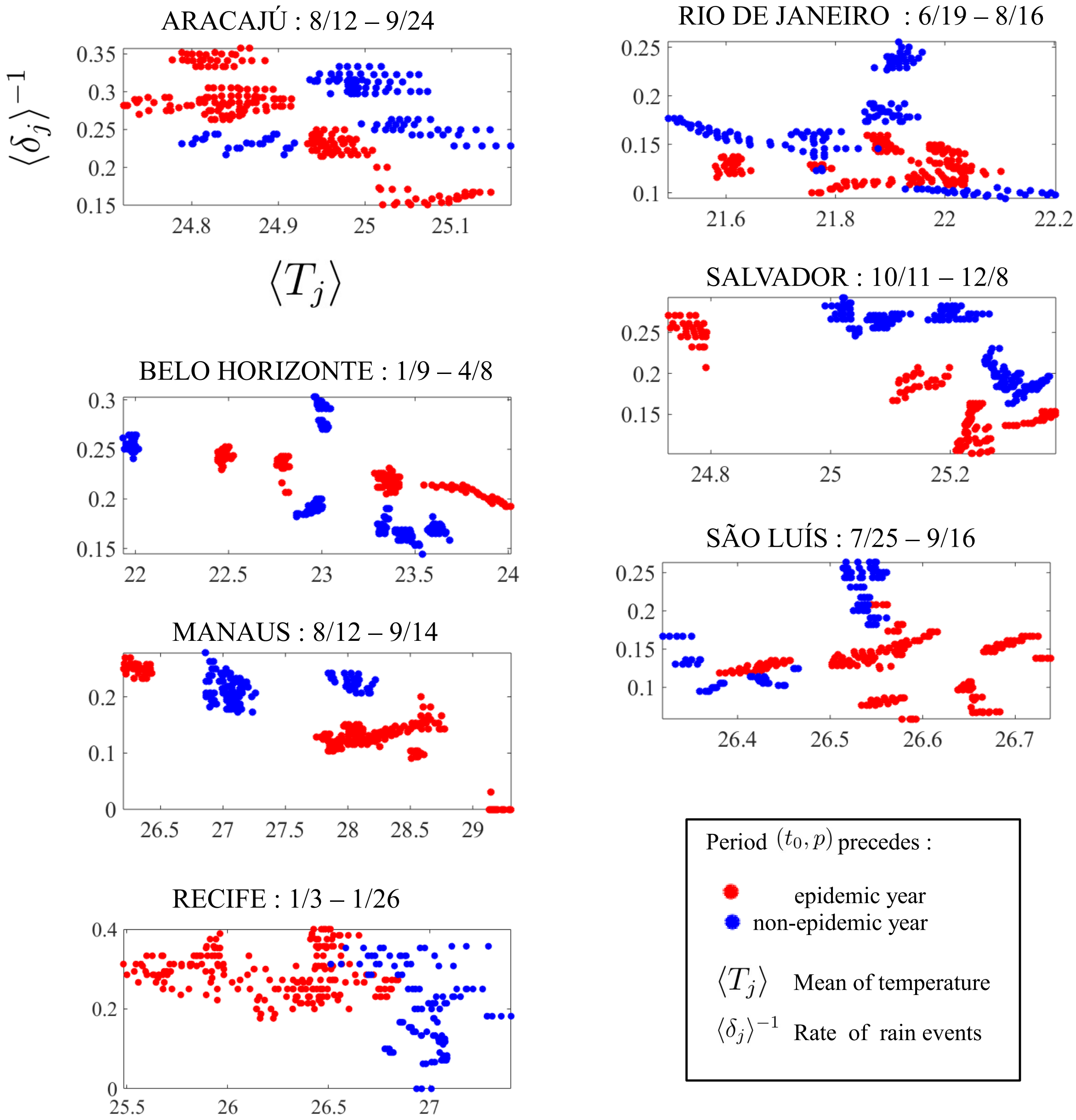

Figure C: **Examples of high separability plots with the full data sets.**

Manaus

Figure **Jb** shows the best prediction results for Manaus using (i) a linear kernel, (ii) $\alpha = 0.95$, and (iii) the AA strategy. Seven years were correctly predicted (64% accuracy) but the years 2002 (FN), 2004 (FP), 2006 (FN), and 2008 (FP) were not. Once again, we leave for the SI section the full list of time periods corresponding to the (t_0, p) rectangles found in all out-of-sample predictions. The selected *AA-months* were August, September and October. The EP strategy gave poor results, with a maximum accuracy of 54%.

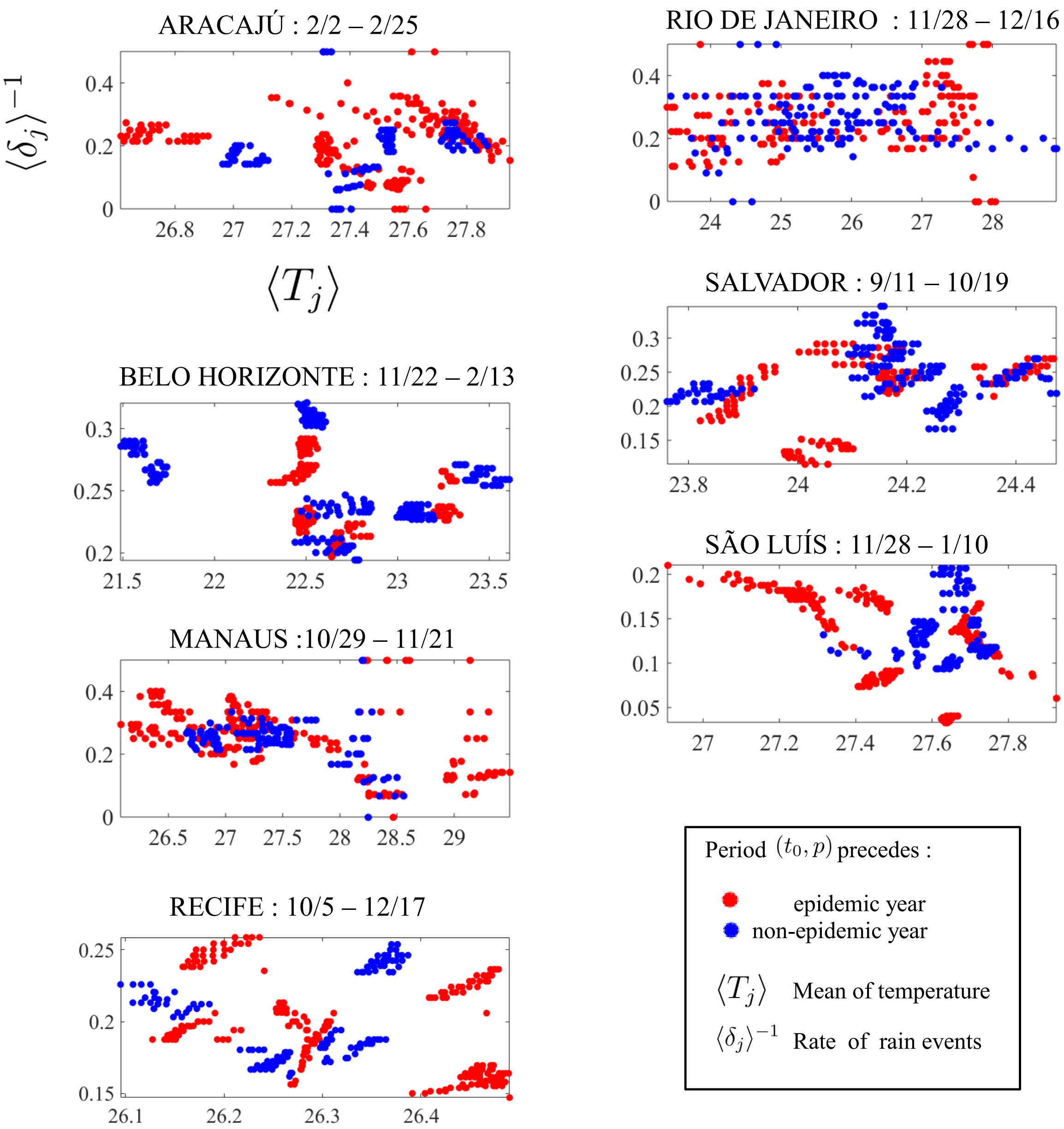

Figure D: **Examples of low separability plots with full data set.**

Recife

Prediction highlights for the city of Recife are shown in Fig. G. Best results used (i) a linear kernel, (ii) $\alpha = 1$, and (iii) AA strategy. A total of 9 years were correctly predicted (82% accuracy) and the years of 2003 and 2003 (both FP) were wrongly predicted. A single (t_0, p)-rectangle was chosen in all out-of-sample predictions, with climate data from December 28th to January 11th during the summer.

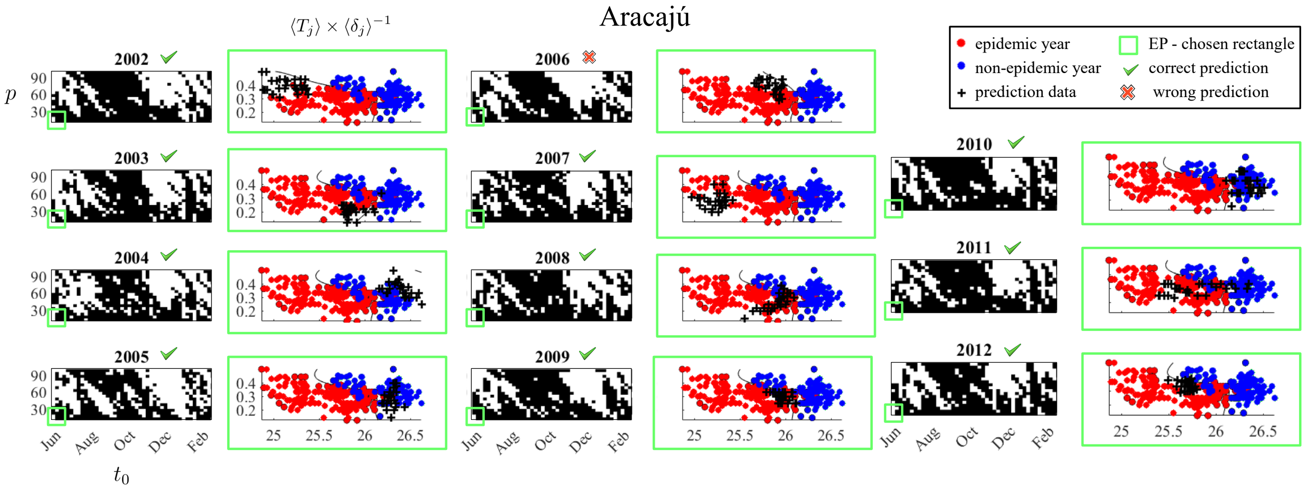

Figure E: **Prediction results for Aracajú.** We predicted the outcome of each target year using the others as a training set. Best results occurred choosing (i) a nonlinear RBF kernel, (ii) an SVM threshold of $\alpha = 0.9$, and (iii) the *Earliest as Possible* (EP) strategy (see main text), which takes only the earliest (t_0, p)-rectangle (called EP-chosen rectangle) and calculate the dengue probability Prob$(j = 1)$ in the $\langle T_j \rangle \times \langle \delta_j \rangle^{-1}$ plane. Most EP-chosen rectangles (boxed in green) occurred within June 1st – 19th, i.e., during the early winter preceding the epidemic outbreak. All years were correctly predicted except 2006 (false positive).

Rio de Janeiro

Figure H shows the best prediction result for Rio de Janeiro using (i) an RBF kernel, (ii) an SVM threshold of $\alpha = 1$, and (iii) the EP-strategy to calculate the outbreak probability. Most EP-chosen rectangles occurred in the winter and in the spring. The corresponding EP-window ranged between June 19th and September 25th, when most Epidemic years (all except 2012) had average temperatures above 23 Celsius and precipitation rates below 0.15. All years except 2010 (FP) and 2012 (FN) were correctly predicted (82% accuracy).

Salvador

Figure I (**top**) shows the best prediction result for the city of Salvador using (i) an RBF kernel, (ii) an SVM threshold of $\alpha = 0.95$, and (iii) the AA-strategy to calculate the outbreak probability. The (t_0, p) rectangles used in the prediction covered most of the year but were especially clustered around December-February (boxed in magenta). All years except 2002 (FN) and 2010 (FN) were correctly predicted (82% of accuracy).

Predictions using (i) a linear kernel, (ii) $\alpha = 0.9$, and (iii) the EP-strategy also gave good results (highlighted in Fig I (**bottom**)). Eight years were correctly predicted (73% accuracy) but the years of 2008 (FP), 2010 (FN) and 2012 (FN) were not. The EP strategy was just slightly less accurate than the AA strategy, yielding EP-windows within August 30th and December 11th (spring and summer). The epidemic years typically showed lower precipitation rates in the selected EP-rectangles.

São Luís

Fig **Ja** highlights the best prediction results for the city of São Luís using (i) an RBF kernel, (ii) an SVM threshold of $\alpha = 1$, and (iii) the AA strategy to calculate the outbreak probability. All years except 2003 (FP) and 2006 (FN) were correctly predicted (82% accuracy). See the SI material for a full list of (t_0, p) rectangles used in all out-of-sample predictions. The selected *AA-months* were December, January, February and March. This AA-strategy was significantly more accurate than all possible choices of EP-strategies.

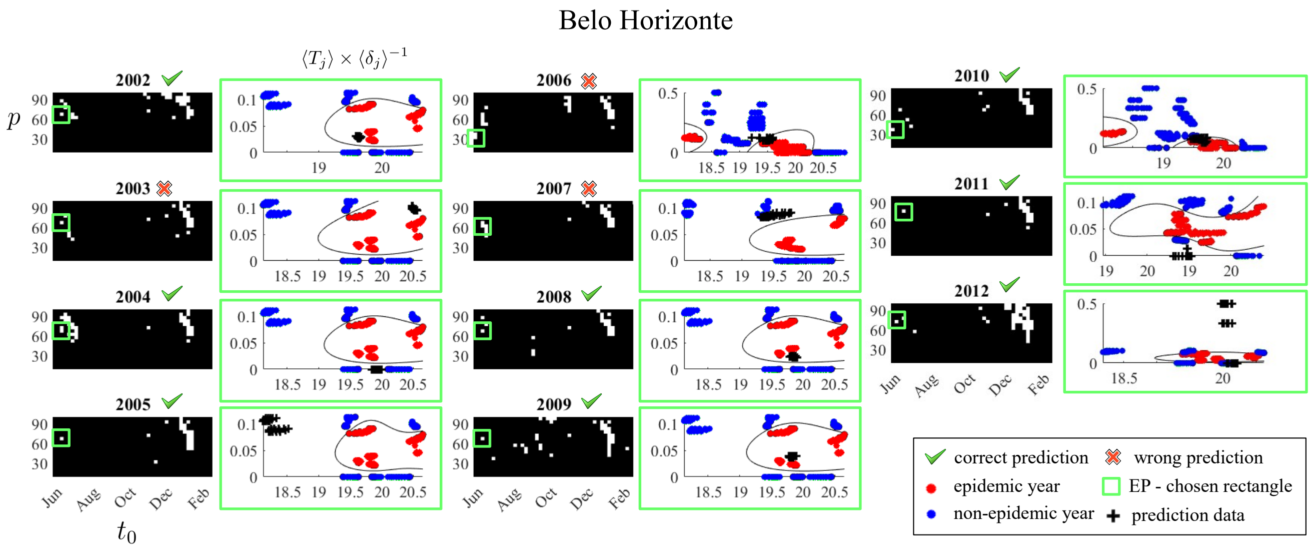

Figure F: **Prediction results for Belo Horizonte.** Best choice used (i) an RBF kernel, (ii) $\alpha = 1$ (maximum separability score), and (iii) the EP strategy. The EP-window occurred within June 13th to August 25th, during winter. Eight years were correctly predicted (73% of accuracy) while 2003 (false positive), 2006 (false positive) and 2007 (false negative) were not.

Additional Practical Considerations

Throughout the manuscript, we refer to a "user" as someone with access to new/different data (both climate and epidemiological) willing to calculate Dengue outbreak probabilities using our same methodology.

The user's first task is to (i) split the yearly climate data between a training set and a testing/prediction set. Then, he/she must choose between (ii) SVM kernels (linear or nonlinear), (iii) alpha values, (iv) rectangle sizes and (v) prediction strategies. If the user is simply appending new climate data to the Brazilian Capitals analyzed in this work, they could leverage our best parameter/strategy choices shown in Table 1. For other cities, the user might want to follow our work as a guideline. Ultimately, one should compute prediction routines for each combination of parameters, build confusion matrices, and compare the distinct choices listed above regarding their accuracy. Table 1 also demonstrates that cities may require significantly different choices. Thus, we recommend the user trying to predict a dengue outbreak in a novel city to systematically test all sensible combinations. We point out that the user should be careful when choosing rectangle sizes: smaller rectangles will increase the heatmap resolution but will contain fewer (t0, p) points. Our choice of dimensions 5 and 6 was reasonable for our specific dataset, but will likely not generalize to others. It is definitely worth to investigate in future studies this tradeoff between resolution vs content in rectangle sizes and how to control it to maximize prediction accuracy.

Tables with epidemic/non-epidemic years and missing climate data gaps

We provide tables with estimated population, total number of Dengue cases, incidence per $100,000$ inhabitants, and details of our climate data completing protocols (if any). For Rio de Janeiro, we consider the time period from 2003 to 2013 and we use epidemic data from municipal webpage (link). For the other capitals, the analyzed period ranges from 2002 to 2012 and data was collected from the Ministry of Health's Notifiable Diseases Information System. (SINAN)

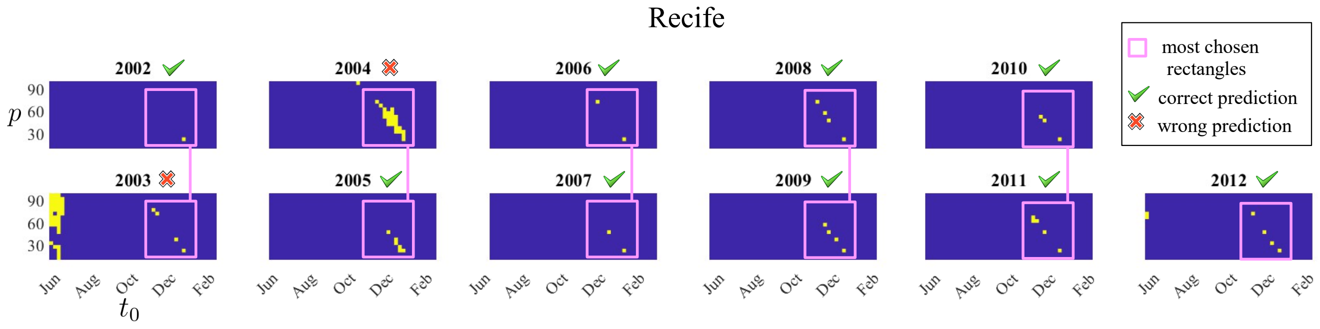

Figure G: **Prediction results for Recife.** Best results occurred choosing (i) a linear kernel, (ii) $\alpha = 1$, and (iii) the AA strategy. A total of 9 out of 11 years were correctly predicted, with most chosen rectangles (boxed in magenta) occurring in the summer season.

Table 1: **Aracajú.**

**Year**	**Pop.**	**Cases**	**Incidence**
2002	473, 991	1, 933	407.81
2003	479, 767	1, 301	271.17
2004	491, 898	166	33.75
2005	498, 619	271	54.35
2006	505, 286	355	70.26
2007	520, 303	728	139.92
2008	536, 785	10, 702	1,993.72
2009	544, 039	1, 232	226.45
2010	571, 149	302	52.88
2011	579, 563	1, 399	241.39
2012	587, 701	2, 656	451.93

Incidence = Cases per 100, 000 inhabitants. Single gaps of missing climate data were filled by linear interpolation; temperature on 12/21/2006 and precipitation on 7/24/2010.

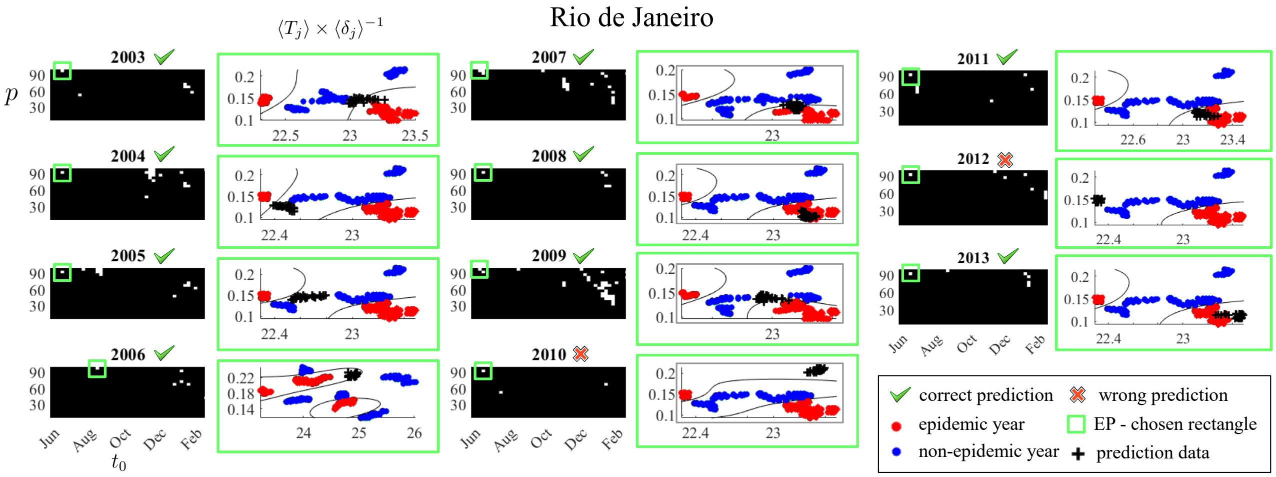

Figure H: **Prediction results for Rio de Janeiro.** Best results occurred choosing (i) a nonlinear RBF kernel, (ii) an SVM threshold of $\alpha = 1$ (maximum separability score), and (iii) the EP-strategy. Most EP-rectangles occurred in winter and spring, leading to an EP-window within June 19th and September 25th. A total of 9 years were correctly predicted (82% accuracy), but 2010 (false positive) and 2012 (false negative) were not.

Table 2: **Belo Horizonte.**

Year	Pop.	Cases	Incidence	Temp (L.I)	Precip (L.I)
2001	–	–	–	8/9	–
2002	2,284,468	4,749	207.88	8/31	10/1<br>10/3 – 10/4
2003	2,305,812	1,800	78.06	–	–
2004	2,350,564	472	20.08	–	–
2005	2,375,329	149	6.27	–	–
2006	2,399,920	872	36.33	–	–
2007	2,412,937	5278	218.74	12/31	–
2008	2,434,642	12,967	532.60	1/1	1/1
2009	2,452,617	14,494	590.96	12/12	–
2010	2,375,151	52,315	2,202.60	–	–
2011	2,385,640	1,749	73.31	–	–
2012	2,395,785	635	26.50	–	–

Incidence = Cases per 100,000 inhabitants. L.I stands for Linear Interpolation. We exceptionally used linear interpolation in the precipitation time series between 10/3 and 10/4 of 2002 due to the lack of other data sources.

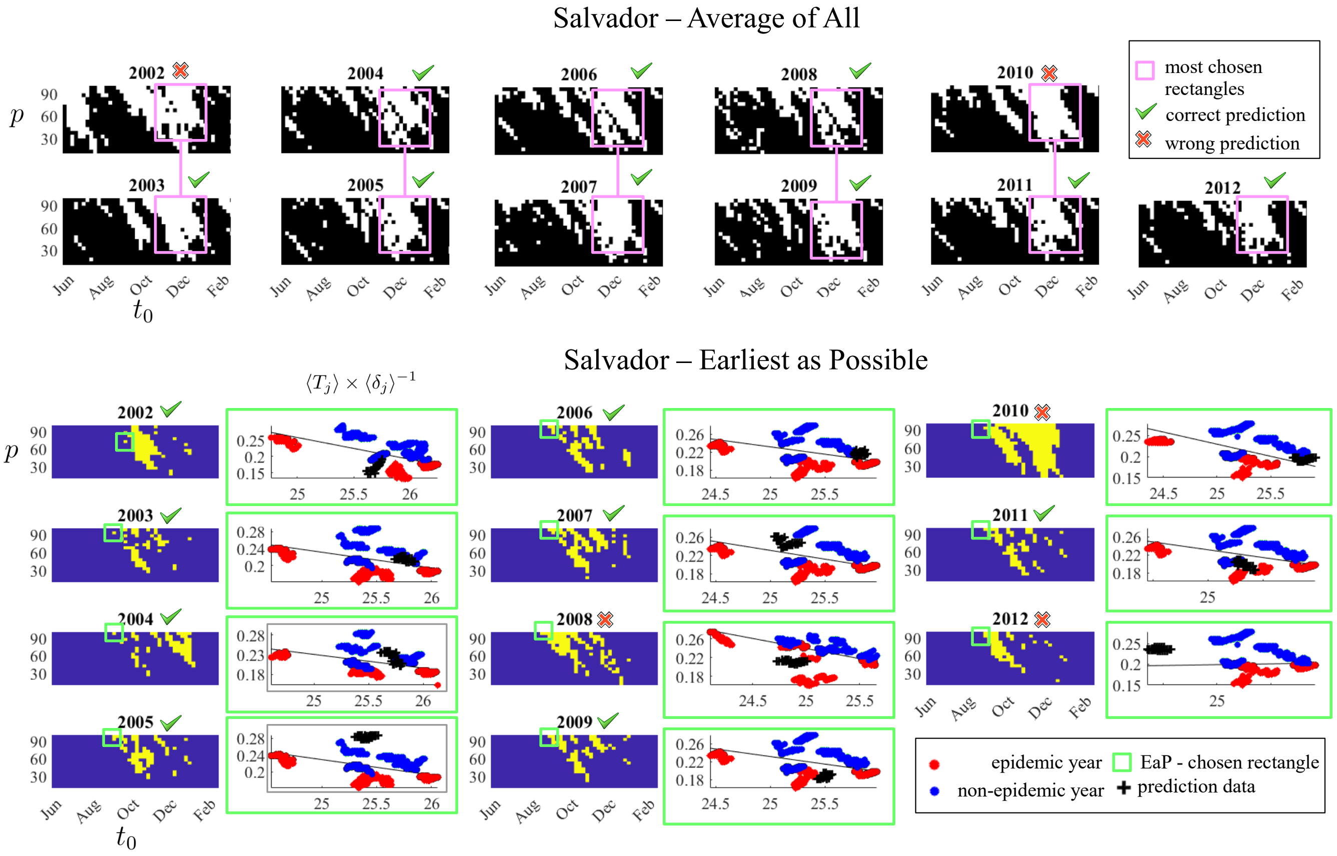

Figure I: **Prediction results for Salvador.** Two sets of parameters led to good results: **top.** choosing (i) a nonlinear RBF kernel, (ii) an SVM threshold of $\alpha = 0.95$, and (iii) the *Average of All* (AA) strategy. Most rectangles (boxed in magenta) used by the AA-strategy remained within December-February, suggesting that the summer season has a good predictive power. A total of 9 years were correctly predicted (82% of accuracy), but 2002 and 2010 were not (false negatives). **bottom.** Choosing (i) a linear kernel, (ii) $\alpha = 0.9$, and (iii) the EP strategy gave 8 correct predictions and 3 wrong predictions: 2008 (false positive), 2010, and 2012 (false negatives). This resulted in 73% of accuracy. This EP-choice performed slightly worse than the AA-strategy and led to EP-windows within August 30th - December 11th (spring-summer seasons).

Table 3: **Manaus.**

Year	Pop.	Cases	Incidence
2002	1,488,805	1,855	124.60
2003	1,527,314	3,731	244.29
2004	1,592,555	789	49.54
2005	1,644,690	915	55.63
2006	1,688,524	495	29.32
2007	1,646,602	1,989	120.79
2008	1,709,010	5,975	349.62
2009	1,738,641	623	35.83
2010	1,802,014	3,748	207.99
2011	1,832,424	54,342	2,965.58
2012	1,861,838	3,703	198.89

Incidence = Cases per 100,000 inhabit. Single gaps of missing climate data were filled by linear interpolation; temperature on 12/23/2005 and precipitation on 2/11/2005.

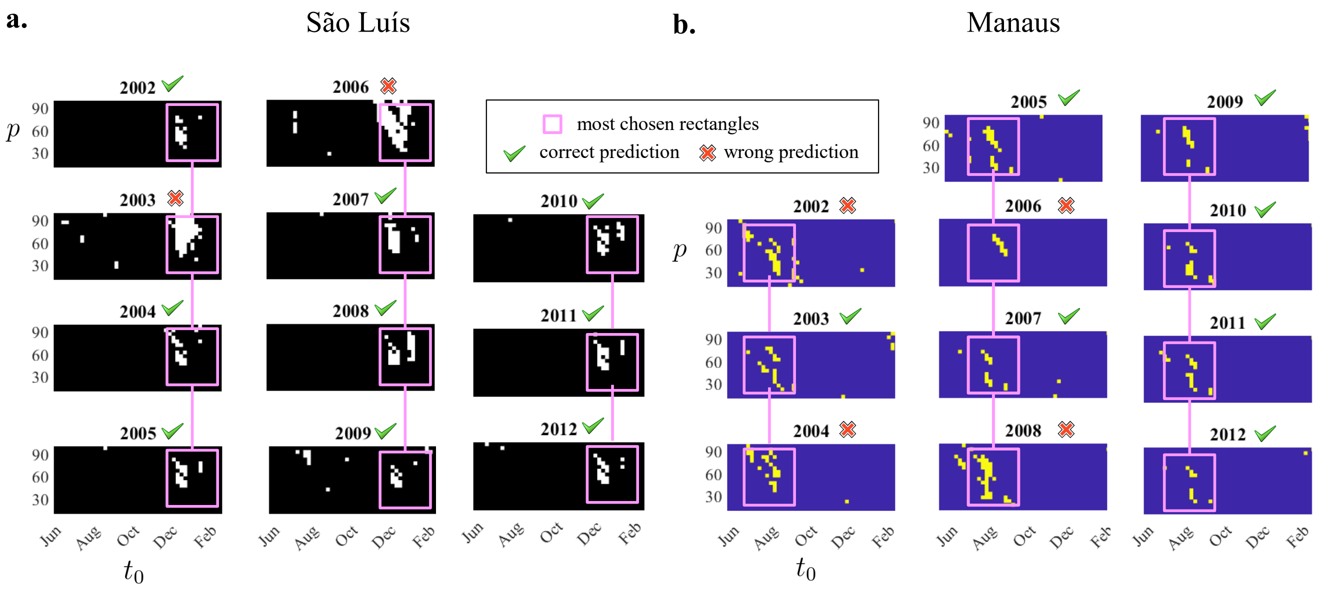

Figure J: **Best Prediction results for São Luís and Manaus. a.** For the city of São Luís, the best prediction results were found choosing (i) an RBF kernel, (ii) $\alpha = 1$ (maximum separability score), and (iii) the AA strategy. In total, there were 9 correct predictions (82 % of accuracy) but wrong predictions for 2003 (false positive) and 2006 (false negative). The AA-months spanned December – March. **b.** For the city of Manaus, best results were found using (i) a linear kernel, (ii) $\alpha = 0.95$, and (iii) the AA strategy. From a total of 11 years, 7 were correctly predicted (%64 of accuracy). The AA-months remained usually within the months of August – October.

Table 4: **Recife**

**Year**	**Pop.**	**Cases**	**Incidence**	**Temp (L.I)**	**Precip(L.I)**
2001	–	–	–	–	–
2002	1,449,135	42,791	2,952.86	–	–
2003	1,461,320	449	30.73	–	–
2004	1,486,869	241	16.21	–	–
2005	1,501,008	830	55.30	–	–
2006	1,515,052	1,443	95.24*	11/4<br>12/2	–
2007	1,533,580	1,503	98.01*	–	–
2008	1,549,980	4,771	307.81	4/28	–
2009	1,561,659	578	37.01	4/30<br>7/31<br>11/19	–
2010	1,537,704	11,494	747.48	9/8	–
2011	1,546,516	5,471	353.76	–	–
2012	1,555,039	11,444	735.93	1/1<br>5/2<br>6/14<br>8/14	1/1<br>5/2<br>8/14

Incidence = Cases per 100,000 inhabitants. L.I stands for Linear Interpolation. *Years 2006 and 2007 were particularly considered epidemic because their incidence values were almost the same as the classification threshold.

Table 5: **Rio de Janeiro.**

Year	Pop.	Cases	Incidence	Temp (CVX)	Precip (subst)
2002	–	–	–	8/31	–
2003	5,974,081	1,610	26.95	3/1 – 3/2<br>6/20 – 6/30	6/20 – 6/30
2004	6,051,399	607	10.03	–	–
2005	6,094,183	980	16.08	–	–
2006	6,136,652	14,435	235.23	–	12/13 – 12/31
2007	6,093,472	26,507	435.01	1/1 – 2/1	1/1 – 1/10
2008	6,161,047	110,861	1799.39	–	–
2009	6,186,710	2,961	47.86	2/11	–
2010	6,320,446	3,000	47.47	–	–
2011	6,355,949	78,645	1237.34	–	–
2012	6,390,290	137,505	2151.78	12/8<br>12/26 – 12/27	–
2013	6,429,923	66,278	1030.77	6/13 – 6/21	6/14 – 6/19

Incidence = Cases per 100,000 inhabitants. Number of Dengue cases in 2013 taken from the City's hall health department, because data from SINAN is not available for that year. For the larger gaps of missing data on precipitation time series, we have used data of the *Alerta Rio* system from Saúde neighborhood, the closest to the Santos Dumont airport where INMET's rain collectors are located.

Table 6: **Salvador.**

Year	Pop.	Cases	Incidence	Temp (CVX)	Precip (L.I)
2001	–	–	–	–	–
2002	2,520,504	26,838	1,064.79	10/9 – 10/21	–
2003	2,556,429	908	35.52	–	–
2004	2,631,831	154	5.85	–	–
2005	2,673,560	270	10.10	10/21 – 10/31	
2006	2,714,018	377	13.89	–	–
2007	2,892,625	1,349	46.64	10/6 – 10/7	10/7
2008	2,948,733	2,476	83.97	–	–
2009	2,998,056	6,819	227.45	6/9<br>12/27	–
2010	2,675,656	6,159	230.19	–	–
2011	2,693,606	5,321	197.54	–	–
2012	2,710,968	5,161	190.37	–	–

Incidence = Cases per 100,000 inhabitants. L.I stands for Linear Interpolation.

Table 7: **São Luís .**

Year	Pop.	Cases	Incidence	Temp (CVX)	Precip (L.I)
2001	–	–	–	10/1 – 10/31<br>11/14<br>11/21	–
2002	$906,567$	448	49.42	4/30	–
2003	$923,526$	567	61.40	9/5 – 9/26<br>9/28 – 10/10	–
2004	$959,124$	154	16.06	–	–
2005	$978,824$	$2,580$	263.58	–	–
2006	$998,385$	$1,395$	139.73	–	–
2007	$957,515$	$3,827$	399.68	–	–
2008	$986,826$	$1,183$	119.88	–	–
2009	$997,098$	100	10.03	–	5/31
2010	$1,014,837$	$2,731$	269.11	–	–
2011	$1,027,430$	$5,229$	508.94	10/20	–
2012	$1,039,610$	$1,315$	126.49	6/8 – 6/9<br>6/12 – 6/13<br>7/24 – 7/25<br>7/29	–

Incidence = Cases per $100,000$ inhabitants. L.I stands for Linear Interpolation.

Prediction Results for each state capital

Here the reader can find the best prediction results for all 7 state capitals considered in our work. We have chosen a criterion based on highest prediction accuracy (see manuscript for details) for selecting the SVM kernel, threshold α and prediction strategy. The following tables contain the evaluated probabilities of dengue outbreaks for each test year. For those state capitals where the EP-strategy performed the best results, we could also exhibit the correspondent dates of the EP-chosen rectangles of each out-of-sample prediction. For those capitals where the AA-strategy had best results, we provide a full list of the time windows ((t_0, p)-rectangles) that were common in all out-of-sample predictions. The *AA-months* are those months containing all time windows that were found.

Table 8: **Aracajú: Prediction Results – EP strategy, RBF Kernel,** $\alpha = 0.9$

Year	Data	Dengue Prob.	Result	EP-chosen rectangle
2002	D	0.97	✓	Jun 1 – 19
2003	D	0.87	✓	Jun 1 – 19
2004	ND	0	✓	Jun 1 – 19
2005	ND	0	✓	Jun 1 – 19
2006	ND	0.93	×	Jun 1 – 19
2007	D	1	✓	Jun 1 – 19
2008	D	0.87	✓	Jun 1 – 19
2009	D	0.73	✓	Jun 1 – 19
2010	ND	0	✓	Jun 1 – 19
2011	D	0.87	✓	Jun 1 – 19
2012	D	0.9	✓	Jun 1 – 19

Remark: "D" represents epidemic years and "ND" non-epidemic years.

Table 9: **Belo Horizonte: Prediction Results - EP strategy, RBF Kernel,** $\alpha = 1$

Year	Data	Dengue Prob.	Result	EP-chosen rectangle (dates)
2002	D	1	✓	Jun 13 – Aug 25
2003	ND	1	×	Jun 13 – Aug 25
2004	ND	0	✓	Jun 13 – Aug 25
2005	ND	0	✓	Jun 13 – Aug 25
2006	ND	0.9	×	Jun 1 – Jul 9
2007	D	0	×	Jun 13 – Aug 20
2008	D	1	✓	Jun 13 – Aug 25
2009	D	1	✓	Jun 13 – Aug 25
2010	D	0.87	✓	Jun 1 – Jul 14
2011	ND	0	✓	Jun 19 – Sept 10
2012	ND	0	✓	Jun 7 – Aug 24

Remark: "D" represents epidemic years and "ND" non-epidemic years.

Table 10: **Manaus: Prediction Results – AA strategy, Linear Kernel,** $\alpha = 0.95$

Year	Data	Dengue Prob.	Result
2002	D	0.3	×
2003	D	0.65	✓
2004	ND	0.88	×
2005	ND	0.27	✓
2006	ND	1	×
2007	D	0.9	✓
2008	D	0	×
2009	ND	0.2	✓
2010	D	0.73	✓
2011	D	0.93	✓
2012	D	0.87	✓

Remark: "D" represents epidemic years and "ND" non-epidemic years.
We find the following time windows (in the format DD/MM) common to all out-of-sample predictions: 11/09-9/10, 11/09-14/10, 12/08-24/09, 12/08-24/10, 12/08-29/09, 17/09-15/10, 18/08-25/10 and 23/09-16/10.

Table 11: **Recife: Prediction Results for AA strategy, linear Kernel, $\alpha = 1$**

Year	Data	Dengue Prob.	Result
2002	D	1	✓
2003	ND	0.92	×
2004	ND	0.78	×
2005	ND	0.23	✓
2006	D*	1.00	✓
2007	D*	1.00	✓
2008	D	1.00	✓
2009	ND	0	✓
2010	D	0.99	✓
2011	D	0.67	✓
2012	D	0.64	✓

Remarks: "D" represents epidemic years and "ND" non-epidemic years. *Years 2006 and 2007 were particularly considered epidemic because their incidence values were almost the same as the classification threshold.
We find the following time window (in the format DD/MM) common to all out-of-sample predictions: 28/12-11/01.

Table 12: **Rio de Janeiro: Prediction Results – EP strategy, RBF Kernel, $\alpha = 1$**

Year	Data	Dengue Prob.	Result	EP-chosen rectangle (dates)
2003	ND	0.27	✓	Jun 19 – Sept 30
2004	ND	0	✓	Jun 19 – Sept 25
2005	ND	0	✓	Jun 19 – Sept 25
2006	D	1	✓	Aug 18 – Nov 29
2007	D	0.5	✓	Jun 13 – Sept 24
2008	D	1	✓	Jun 19 – Sept 25
2009	ND	0.03	✓	Jun 13 – Sept 24
2010	ND	1	×	Jun 19 – Sept 25
2011	D	1	✓	Jun 19 – Sept 25
2012	D	0	×	Jun 19 – Sept 25
2013	D	1	✓	Jun 19 – Sept 25

Remark: "D" represents epidemic years and "ND" non-epidemic years.

Table 13: **Salvador: Prediction Results – EP strategy, Linear Kernel,** $\alpha = 0.9$

Year	Data	Dengue Prob.	Result	EP-chosen rectangle (dates)
2002	D	1	✓	Sept 23 – Dec 10
2003	ND	0	✓	Sept 5 – Dec 12
2004	ND	0.06	✓	Sept 5 – Dec 17
2005	ND	0	✓	Sept 5 – Dec 12
2006	ND	0	✓	Aug 30 – Dec 11
2007	ND	0	✓	Aug 30 – Dec 11
2008	ND	1	×	Aug 18 – Nov 29
2009	D	1	✓	Aug 30 – Dec 11
2010	D	0	×	Aug 30 – Dec 6
2011	D	1	✓	Aug 30 – Dec 11
2012	D	0	×	Aug 30 – Dec 6

Remark: "D" represents epidemic years and "ND" non-epidemic years.

Table 14: **Salvador: Prediction Results – AA strategy, RBF Kernel,** $\alpha = 0.95$

Year	Data	Dengue Prob.	Result
2002	D	0.37	×
2003	ND	0.49	✓
2004	ND	0.44	✓
2005	ND	0.20	✓
2006	ND	0.32	✓
2007	ND	0.34	✓
2008	ND	0.40	✓
2009	D	0.64	✓
2010	D	0.05	×
2011	D	0.72	✓
2012	D	0.78	✓

Remark: "D" represents epidemic years and "ND" non-epidemic years.
We find a total of 168 time windows common to all out-of-sample predictions, covered by almost all months of the year.

Table 15: **São Luís: Prediction Results – AA strategy, RBF Kernel,** $\alpha = 1$

Year	Data	Dengue Prob.	Result
2002	ND	0.038	✓
2003	ND	0.64	×
2004	ND	0.33	✓
2005	D	0.95	✓
2006	D	0.35	×
2007	D	0.68	✓
2008	D	0.61	✓
2009	ND	0.49	✓
2010	D	0.72	✓
2011	D	0.83	✓
2012	D	0.93	✓

Remark: "D" represents epidemic years and "ND" non-epidemic years.
We find the following time windows (in the format DD/MM) common to all out-of-sample predictions: 10/12-3/03, 10/12-6/02, 10/12-11/02, 10/12-16/02, 10/12-26/02, 16/12-4/03, 16/12-7/02, 16/12-12/02, 16/12-17/02, 22/12-13/02, 22/12-18/02 and 22/12-28/02

Confusion Matrices

We provide all confusion matrices. See manuscript for details on how we compute them. The best results are highlighted in bold-red color. For those capitals where we find the same accuracy for different α values, we choose the highest α in order to promote the best separability scores. The file can be downloaded here

References

[1] Kutz JN. Data-Driven Modeling & Scientific Computation: Methods for Complex Systems & Big Data. Oxford University Press; 2013.

[2] Candés EJ and Wakin MB. An introduction to compressive sampling. IEEE signal processing magazine 25.2 (2008): 21-30.

[3] Gemmeke JF, Van Hamme H, Cranen B, Boves L. Compressive sensing for missing data imputation in noise robust speech recognition. IEEE Journal of selected topics in Signal Processing 4.2 (2010): 272-287.

[4] Stankovic L, Stankovic S, and Amin M. Missing samples analysis in signals for applications to L-estimation and compressive sensing. Signal Processing 94 (2014): 401-408.

[5] Zhang Y When is missing data recoverable?. Technical Report, 2006.

[6] Dinku, T., Ceccato, P., Connor, S.J. (2011). Challenges to Satellite Rainfall Estimation over Mountainous and Arid Parts of East Africa. International Journal of Remote Sensing, 32:(21), 5965-5979

[7] Roseghini, W.F.F., Mendonca, F., Ceccato, P., Fernandes, K. (2011). Dengue epidemics in Middle-South of Brazil: Climate constraints (?) and some social aspects. Revista Brasileira de Climatologia 9: 94-101, ISSN: 1980-055x

[8] Dinku, T., Connor, S.J., Ceccato, P. (2011). Evaluation of Satellite Rainfall Estimates and Gridded Gauge Products over the Upper Blue Nile Region. In Melesse, A.M. (Ed.) Nile River Basin, Part II, Springer DOI: 10.1007/978-94-007-0689-7_5, 109-127

[9] Alert system of rain events, city Hall. Precipitation time series of Rio de Janeiro. Available from (website in Portuguese): http://alertario.rio.rj.gov.br/

[10] Brazilian National Institute of Meteorology (INMET) Available from (website in Portuguese): http://www.inmet.gov.br/projetos/rede/pesquisa/

[11] Stolerman, L. M. (2017).Dimensionality Reduction in Neuroscience and Epidemiology. Phd Thesis– IMPA

[12] Burnham KP, Anderson DR. (2003). Model selection and multimodel inference: a practical information-theoretic approach. Springer Science & Business Media.

[13] Tibshirani R (1996). Regression shrinkage and selection via the lasso. Journal of the Royal Statistical Society. Series B (Methodological), 267-288.
