# Supplementary material for: Forecasting dengue fever in Brazil: An assessment of climate conditions
Source: PLoS One. 2019 Aug 8;14(8):e0220106. doi: 10.1371/journal.pone.0220106 (PMC6687106; doi:10.1371/journal.pone.0220106)
Supplement: S2 Appendix — (PDF) [file pone.0220106.s002.pdf]

SUPPORTING INFORMATION - Forecasting Dengue Fever in Brazil: An Assessment of Climate Conditions.

Dengue Incidence and missing climate data between 2013 and 2017

Aracaju
Population (census 2010) = 571,149

Year	Cases	Incidence	Temperature
2013	424	742.363	-
2014	978	171.2338	7/7<br>7/9<br>7/10<br>8/24
2015	2,321	406.3738	-
2016	787	137.7924	7/8<br>8/19
2017	102	17.8587	9/28

Single gaps of missing precipitation data were filled by linear interpolation on 9/28/2017

Belo Horizonte
Population (census 2010) = 2,375,151

Year	Cases	Incidence	Temperature
2013	103,358	4.3516e+03	-
2014	3,550	149.4642	5/26
2015	19,457	819.1900	-
2016	161,296	6.7910e+03	-
2017	1,547	65.1327	6/4<br>8/23

Manaus

Population (census 2010) = 1,802,014

Year	Cases	Incidence	Temperature
2013	13,627	756.2094	-
2014	2,619	145.3374	12/22
2015	1,643	91.1758	8/29<br>10/22
2016	5,302	294.2263	3/28<br>5/16
2017	2,537	140.7869	2/2<br>2/16<br>3/9<br>8/5<br>9/9<br>10/7<br>11/27

Single gaps of missing precipitation data were filled by linear interpolation on 11/27/2017

Recife
Population (census 2010) =1,537,704

Year	Cases	Incidence	Temperature	Precipitation
2013	2,215	144.0459	5/17	-
2014	2,246	146.0619	1/8	-
2015	32,628	2.1219e+03	1/7<br>1/29<br>7/14<br>8/5	1/7<br>1/29<br>7/14<br>6/1<br>1/26
2016	18,008	1.1711e+03	2/24<br>2/25<br>6/1	6/1
2017	1,686	109.6440	1/26<br>8/28<br>10/3	1/26

Rio de Janeiro
Population (census 2010) = 6,320,446

Year	Cases	Incidence	Temperature
2014	2,738	43.3197	8/23<br>8/24<br>12/22
2015	18,.444	291.8148	-
2016	26,008	411.4899	7/6<br>8/4
2017*	3,679	58.2079	4/1<br>4/3<br>4/4

* Official climate date from INMET was only made available until 4/4 of 2017. For this reason, we did not have access to data until 5/31 of 2017, which would be required for the complete prediction of 2017. Therefore, we could not perform the prediction method for 201

Salvador

Population (census 2010) = 2,675,656

Year	Cases	Incidence	Temperature
2013	1,875	70.0763	6/23<br>6/24<br>6/25<br>8/23<br>11/3
2014	3,687	137.7980	7/19<br>7/20<br>7/21<br>7/22<br>8/8
2015	5,836	218.1147	12/30
2016	3,279	122.5494	2/15<br>5/17
2017	2,000	74.7480	-

We exceptionally used linear interpolation in the precipitation time series between 6/23 and 6/24 of 2013 due to the lack of other data sources.

Sao Luis

Population (census 2010) = 1,014,837

Year	Cases	Incidence	Temperature
2013	1,165	114.7968	From 5/24 to 12/31
2014	1,018	100.3117	From 1/1 to 11/13
2015	2,919	287.6324	6/5
2016	5,312	523.4338	-
2017	1,231	121.3003	-

Official climate date from INMET was only made available until 6/5 of 2015. For this reason, we could not perform the prediction method for the years 2016 and 2017.
