# Supplementary material for: Forecasting dengue fever in Brazil: An assessment of climate conditions
Source: PLoS One. 2019 Aug 8;14(8):e0220106. doi: 10.1371/journal.pone.0220106 (PMC6687106; doi:10.1371/journal.pone.0220106)
Supplement: S1 File — (ZIP) [file pone.0220106.s003.zip › data and codes_submit/training dataset analysis/data/Data IBGE/pop_municipios_2007.pdf]

CONTAGEM DA POPULAÇÃO

2007

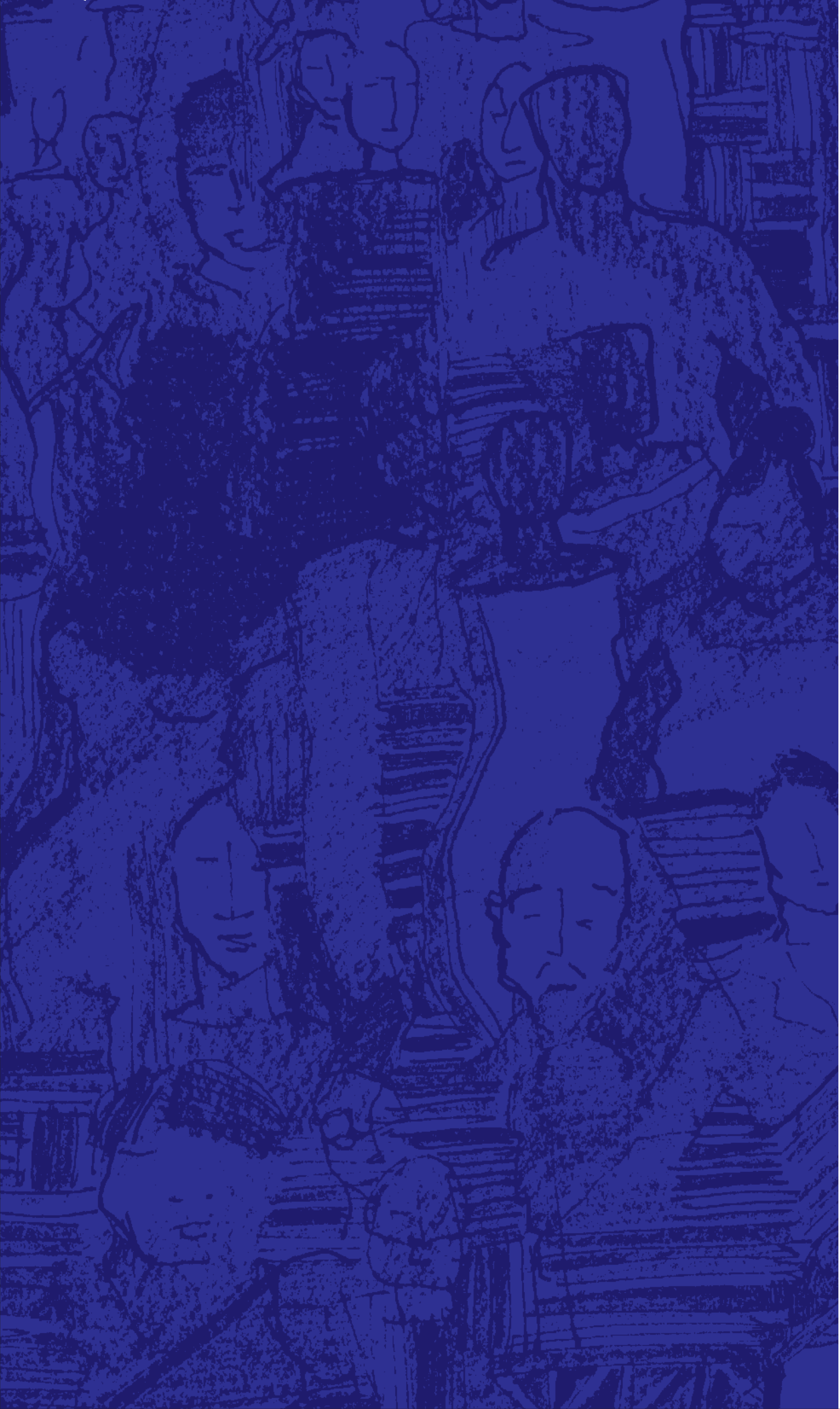

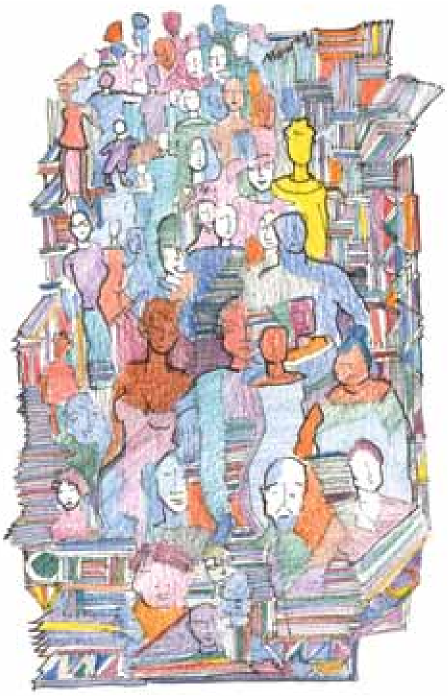

IBGE
Instituto Brasileiro de Geografia e Estatística

Presidente da República
Luiz Inácio Lula da Silva

Ministro do Planejamento, Orçamento e Gestão
Paulo Bernardo Silva

INSTITUTO BRASILEIRO DE GEOGRAFIA E ESTATÍSTICA - IBGE

Presidente
Eduardo Pereira Nunes

Diretor-Executivo
Sérgio da Costa Côrtes

ÓRGÃOS ESPECÍFICOS SINGULARES

Diretoria de Pesquisas
Wasmália Socorro Barata Bivar

Diretoria de Geociências
Luiz Paulo Souto Fortes

Diretoria de Informática
Luiz Fernando Pinto Mariano

Centro de Documentação e Disseminação de Informações
David Wu Tai

Escola Nacional de Ciências Estatísticas
Sérgio da Costa Côrtes (interino)

Ministério do Planejamento, Orçamento e Gestão
Instituto Brasileiro de Geografia e Estatística - IBGE

CONTAGEM DA POPULAÇÃO 2007

Rio de Janeiro
2007

Instituto Brasileiro de Geografia e Estatística - IBGE
Av. Franklin Roosevelt, 166 - Centro - 20021-120 - Rio de Janeiro, RJ - Brasil

ISBN 978-85-240-3995-9 (CD-ROM)

ISBN 978-85-240-3994-2 (meio impresso)

© IBGE. 2007

Elaboração o arquivo PDF
Roberto Cavararo

Produção da multimídia
Marisa Sigolo Mendonça
Márcia do Rosário Brauns

Capa
Renato J. Aguiar e Eduardo Sidney - Coordenação de *Marketing*/
Centro de Documentação e Disseminação de Informações - CDDI

Ilustração da capa e miolo
Aldo Victorio Filho

Sumário

Apresentação

Introdução

Notas técnicas

Fundamento legal

Obrigatoriedade e sigilo das informações

Data de referência

Âmbito

Base territorial

A coleta das informações

Tratamento das informações

Conceituação das características investigadas

Tabelas de resultados

População

População recenseada e estimada

1.1 - População recenseada e estimada, segundo as Grandes Regiões e as Unidades da Federação - 2007

1.1.1 - População recenseada e estimada, segundo os Municípios - Rondônia - 2007

1.1.2 - Acre
1.1.3 - Amazonas
1.1.4 - Roraima
1.1.5 - Pará
1.1.6 - Amapá
1.1.7 - Tocantins
1.1.8 - Maranhão
1.1.9 - Piauí
1.1.10 - Ceará
1.1.11 - Rio Grande do Norte
1.1.12 - Paraíba
1.1.13 - Pernambuco
1.1.14 - Alagoas
1.1.15 - Sergipe
1.1.16 - Bahia
1.1.17 - Minas Gerais
1.1.18 - Espírito Santo
1.1.19 - Rio de Janeiro
1.1.20 - São Paulo
1.1.21 - Paraná
1.1.22 - Santa Catarina
1.1.23 - Rio Grande do Sul
1.1.24 - Mato Grosso do Sul
1.1.25 - Mato Grosso
1.1.26 - Goiás

População recenseada

1.2.1 - População recenseada, por sexo, segundo a idade - Rondônia - 2007

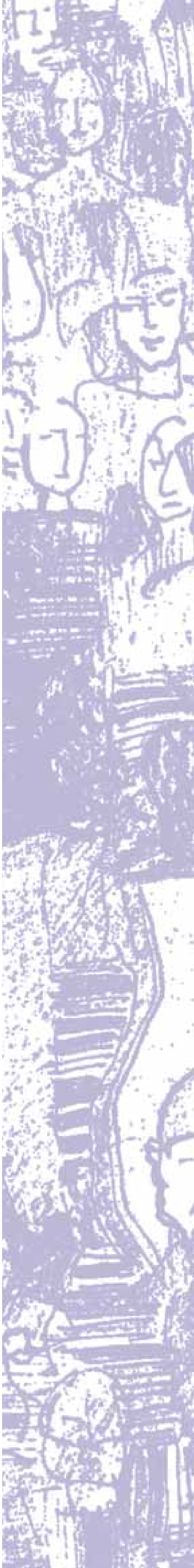

1.2.2 - Acre
1.2.3 - Amazonas
1.2.4 - Roraima
1.2.5 - Pará
1.2.6 - Amapá
1.2.7 - Tocantins
1.2.8 - Maranhão
1.2.9 - Piauí
1.2.10 - Ceará
1.2.11 - Rio Grande do Norte
1.2.12 - Paraíba
1.2.13 - Pernambuco
1.2.14 - Alagoas
1.2.15 - Sergipe
1.2.16 - Bahia
1.2.17 - Minas Gerais
1.2.18 - Espírito Santo
1.2.19 - Rio de Janeiro
1.2.20 - São Paulo
1.2.21 - Paraná
1.2.22 - Santa Catarina
1.2.23 - Rio Grande do Sul
1.2.24 - Mato Grosso do Sul
1.2.25 - Mato Grosso
1.2.26 - Goiás

Domicílios

Domicílios recenseados

2.1.1 - Domicílios recenseados, por espécie, segundo os Municípios - Rondônia - 2007
2.1.2 - Acre
2.1.3 - Amazonas
2.1.4 - Roraima
2.1.5 - Pará
2.1.6 - Amapá
2.1.7 - Tocantins
2.1.8 - Maranhão
2.1.9 - Piauí
2.1.10 - Ceará
2.1.11 - Rio Grande do Norte
2.1.12 - Paraíba
2.1.13 - Pernambuco
2.1.14 - Alagoas
2.1.15 - Sergipe
2.1.16 - Bahia
2.1.17 - Minas Gerais
2.1.18 - Espírito Santo
2.1.19 - Rio de Janeiro
2.1.20 - São Paulo
2.1.21 - Paraná
2.1.22 - Santa Catarina
2.1.23 - Rio Grande do Sul
2.1.24 - Mato Grosso do Sul
2.1.25 - Mato Grosso
2.1.26 - Goiás

Referências

Anexos

1 - Relação dos 128 municípios e do Distrito Federal, por Unidades da Federação, cujas populações foram estimadas para a data de referência de 1º de abril de 2007

2 - Metodologia de estimação do número de moradores em domicílios fechados

Convenções

-	Dado numérico igual a zero não resultante de arredondamento;
..	Não se aplica dado numérico;
...	Dado numérico não disponível;
x	Dado numérico omitido a fim de evitar a individualização da informação;
0; 0,0; 0,00	Dado numérico igual a zero resultante de arredondamento de um dado numérico originalmente positivo; e
-0; -0,0; -0,00	Dado numérico igual a zero resultante de arredondamento de um dado numérico originalmente negativo.

Apresentação

A história dos recenseamentos no Brasil teve seu início no Século XIX, mais precisamente no ano de 1872, quando foi realizado o primeiro censo. Sucederam os levantamentos de 1890, 1900 e 1920. Com a criação do IBGE em 1936, o instituto passou a realizar, sistematicamente, em todo fim de década a partir de 1940, o Censo Demográfico, sendo o último em 2000.

Em 1996, foi realizada a primeira Contagem da População em todo o Brasil, tendo em vista que a partir de 1989, o IBGE passou a ter a obrigação legal de fornecer anualmente estimativas municipais de população residente.

A Contagem da População é planejada para ser realizada no meio da década, de modo a atualizar as informações sobre o número de habitantes e outras características da população dos municípios brasileiros, se constituindo em importante subsídio para as estimativas anuais subsequentes.

A Contagem da População em 2007, segunda operação censitária com essas características, levantou os efetivos de população dos municípios, dando maior visibilidade às transformações demográficas ocorridas no país desde o Censo Demográfico de 2000.

Seus resultados estão apresentados em um volume impresso que contém os totais populacionais para Grandes Regiões, Unidades da Federação e Municípios.

Para as Unidades da Federação, foi produzida uma tabela por sexo e idade para população recenseada. E para os municípios contados, há uma tabela com resultados para a espécie do domicílio.

O volume impresso acompanha um CD ROM que contém, além dessas informações, resultados por sexo, idade, relação de parentesco com a pessoa responsável pelo domicílio e totais de domicílios ocupados ou não, particulares e coletivos, para cada município contado.

Eduardo Pereira Nunes
Presidente do IBGE

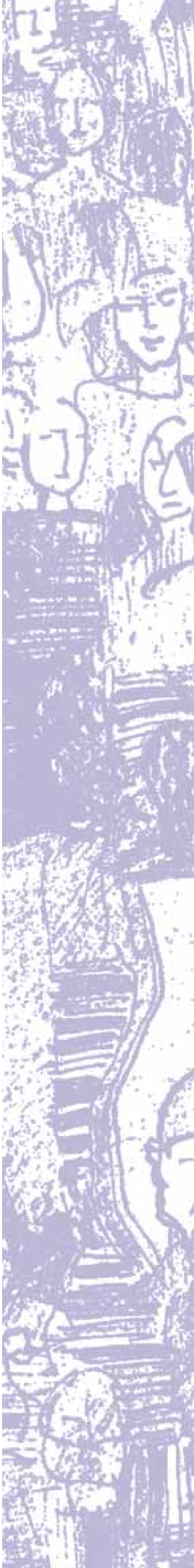

Introdução

O IBGE realizou, no ano de 2007, uma operação censitária que abrangeu o Censo Agropecuário 2006, a Contagem da População 2007 e o Cadastro Nacional de Endereços para Fins Estatísticos - CNEFE. Esta operação conjunta teve por objetivo atualizar as estimativas populacionais e as informações sobre as atividades econômicas realizadas, no País, pelos indivíduos e empresas agropecuárias. A integração dessas três pesquisas foi facilitada em função da utilização do computador de mão, o *Personal Digital Assistant - PDA*, na etapa da operação de campo.

Os Censos são operações de levantamento de dados fundamentais para a formulação de políticas públicas e para tomada de decisões acerca de investimentos privados e governamentais. A cada dez anos, o IBGE realiza o Censo Demográfico no País. Seus resultados são utilizados para analisar a tendência anual de crescimento da população verificada entre um censo e outro, e também para avaliar os fatores que compõem sua dinâmica demográfica, tais como: natalidade, mortalidade e migração.

A Contagem da População, assim denominada por ser uma operação muito mais simples do que um Censo Demográfico, é planejada para ser realizada no meio da década, com o objetivo de atualizar as estimativas de população, incorporando as mudanças demográficas ocorridas no Território Nacional, desde o último Censo Demográfico. Sua importância está naquilo que as estimativas de população representam para a sociedade, pois se trata de um exemplo expressivo da exigência de informações estatísticas que orientam diretamente a ação pública local.

Ao longo de sua história, o Brasil tem passado por transformações tanto no que diz respeito ao padrão de distribuição da população em seu território, como em relação ao movimento das fronteiras de ocupação. A partir de 1989, o IBGE passou a ter a obrigação legal de fornecer anualmente estimativas municipais de população residente, em cumprimento a dispositivo constitucional, regulamentado pela Lei Complementar nº 59, de 22 de dezembro de 1988. Além disso, de acordo com o que estabelece o Artigo 102, da Lei nº 8.443, de 16 de julho de 1992, o IBGE passou a publicar no Diário Oficial da União, até 31 de agosto de cada ano, a relação das estimativas populacionais anuais para estados e municí-

pios, e, até 31 de outubro, a encaminhar ao Tribunal de Contas da União essa relação, que aquele Tribunal utiliza como um dos critérios para a repartição do Fundo de Participação dos Municípios - FPM.

Com isso, a partir dos anos 1990, a contagem populacional passou a ser um instrumento essencial para que o IBGE pudesse atender às demandas de informações demográficas por parte de vários setores da sociedade, mantendo seu padrão habitual de eficiência. Por razões de natureza orçamentária, a realização da Contagem da População, prevista para 2005, só ocorreu em 2007. Pelas mesmas razões não foi possível realizar a contagem em todos os 5 564 municípios do País e, após estudos, o IBGE concluiu que os recursos disponíveis permitiriam a realização da Contagem da População nos municípios com até 170 mil habitantes, ou seja, pouco acima do limite em que o efetivo populacional causa impacto direto nos valores repassados pelo FPM.

Como em algumas Unidades da Federação apenas um ou dois municípios ficariam fora desta faixa por terem mais de 170 mil habitantes, o IBGE optou por incluí-los na pesquisa. Sendo assim, a Contagem abrangeu 5 435 municípios, os 5 414 com até 170 mil habitantes e mais 21 municípios incluídos pelo critério acima mencionado, perfazendo 97% do total de municípios do País. Para os 129 municípios restantes a população de 2007 foi estimada.

Notas técnicas

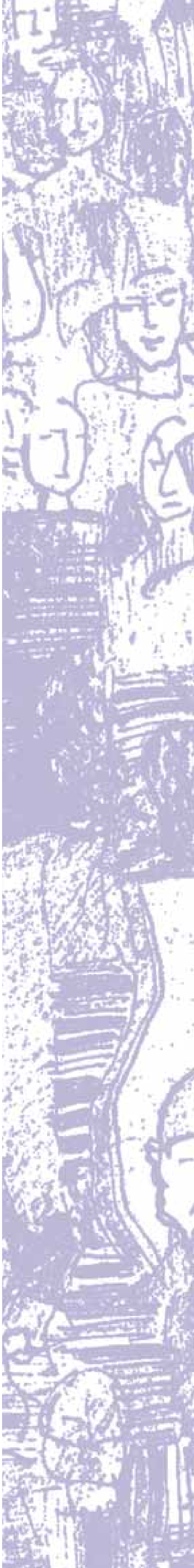

Fundamento legal

A realização da Contagem da População 2007 obedeceu às mesmas determinações da Lei nº 8.184, de 10 de maio de 1991.

Obrigatoriedade e sigilo das informações

A Contagem da População 2007 manteve o caráter obrigatório e confidencial atribuído às informações censitárias, que se destinam exclusivamente a fins estatísticos e não poderão ser objeto de certidão e nem terão eficácia jurídica como meio de prova.

Data de referência

A investigação das características das pessoas, residentes nos domicílios particulares e coletivos, tem como data de referência a noite de 31 de março para 01 de abril de 2007. Desse modo, as pessoas nascidas após 31 de março não foram incluídas na pesquisa, sendo no entanto, incluídas as pessoas falecidas, após aquela data, que residiam no domicílio na data de referência.

Âmbito

A limitação de recursos orçamentários para os Censos 2007 implicou na necessidade de se fazer um corte na abrangência da Contagem da População. Após alguns estudos, decidiu-se fazer a Contagem nos municípios com até 170 mil habitantes, faixa onde os efetivos de população causam impacto direto nos valores repassados pelo Fundo de Participação dos Municípios – FPM.

Entretanto, como em algumas Unidades da Federação apenas um ou dois municípios ficariam fora da cobertura da Contagem por terem mais de 170 mil habitantes, o IBGE decidiu incluí-los, também, na pesquisa. Dessa forma, além dos municípios com até 170 mil habitantes, a Contagem da População 2007 abrangeu, também, um conjunto de 21 municípios com população acima dessa faixa. Com isso, 14 estados foram integralmente abrangidos pela Contagem da

População 2007, a saber: Acre, Alagoas, Amapá, Amazonas, Maranhão, Mato Grosso, Mato Grosso do Sul, Paraíba, Piauí, Rio Grande do Norte, Rondônia, Roraima, Sergipe e Tocantins.

Para entender melhor a abrangência geográfica da Contagem da População 2007, é preciso levar em conta que o Brasil possui 5 564 municípios[1] e que o corte em 170 mil habitantes baseou-se na estimativa de população dos municípios em 2005. Deste total de municípios, 5 414 possuem menos de 170 mil habitantes e somados aos 21 citados perfazem 5 435, representando 97% do total de municípios brasileiros. Apenas 129 municípios[2], ou seja, 3% do total no País, não foram pesquisados.

Esses 5 435 municípios representam cerca de 108,7 milhões de pessoas, ou seja, em torno de 60% da população estimada do País. O universo pesquisado pela Contagem da População 2007 está distribuído em cerca de 30 milhões de domicílios, alcançando 57% do total de domicílios existentes no Brasil.

Base territorial

A Base Territorial, constituída por um conjunto de mapas (componente gráfica) e cadastros (componente alfanumérica), apontou para a coleta dos Censos 2007 - Contagem da População 2007 e Censo Agropecuário 2006 - 158 955 setores censitários, nos municípios onde a Contagem foi realizada, que revelam a atual distribuição da população brasileira.

Por Setor Censitário, entende-se a unidade territorial estabelecida para fins de controle cadastral, formado por área contínua, situada em um único quadro urbano ou rural com dimensão e número de domicílios ou de estabelecimentos que permitam, levantamento das informações por um único Agente Credenciado, segundo cronograma estabelecido. Na sua delimitação são respeitadas as diferentes estruturas territoriais para as quais são apuradas e divulgadas as informações.

A preparação dessa base leva em conta, além da organização da operação de coleta dos censos, a necessidade de atender às demandas por informações cada vez mais detalhadas, que subsidiem a tomada de decisão em investimentos públicos e privados por prefeituras, órgãos de planejamento e iniciativa privada.

Os trabalhos de revisão da Base Territorial para 2007 incluíram uma atualização cartográfica em gabinete e em campo, com a finalidade de enriquecer com informações atuais os mapas e cadastros da Base Territorial 2000, oferecendo, assim, ao recenseador o fácil reconhecimento e a orientação adequada para o perfeito recobrimento de sua área de trabalho.

---

[1] De fato, são 5 562 municípios. As outras duas unidades referem-se: uma, ao distrito de Fernando de Noronha, subordinado ao Estado de Pernambuco; e outra a Brasília, Distrito Federal, as quais são consideradas unidades de planejamento de atividades de pesquisa em nível municipal.

[2] Ver Anexo 1.

Foram atualizadas informações sobre: terras indígenas, áreas de proteção ambiental, redes viária e hidrográfica, povoados, aglomerados subnormais, perímetro urbano, divisão intraurbana, logradouros. Para os Censos 2007, foi incluída uma nova área de apuração: projetos de assentamento rurais do INCRA.

Todo esse trabalho se desenvolveu de forma descentralizada, nas equipes de Base Territorial das Unidades Estaduais do IBGE que contaram também com a participação de prefeituras e órgãos de planejamento para o êxito da operação.

Os arquivos de Setores Censitários e de descrições dos perímetros dos setores, bem como o Banco de Estruturas Territoriais foram atualizados para retratar a situação territorial de 2007.

A coleta das informações

A coleta de dados dos Censos 2007 foi iniciada em 16 de abril de 2007, através de entrevista direta com perguntas listadas sob a forma de questionário a ser preenchido no computador de mão.

Uma grande novidade trazida pelos Censos 2007 é a tecnológica, com a substituição do tradicional questionário em papel pelo computador de mão ou *Personal Digital Assistant* - PDA. Esse equipamento digital já era utilizado em outras pesquisas do IBGE, entretanto foi a primeira vez que a Instituição fez uso desse recurso numa operação censitária. O PDA, intensivamente empregado na coleta de dados, trouxe inúmeras vantagens, dentre as quais estão a crítica imediata no momento em que os dados eram coletados, possibilitando a correção da informação no ato da entrevista; o preenchimento de todos os quesitos obrigatórios, evitando a não resposta por esquecimento ou erro do recenseador; a otimização do preenchimento dos dados a partir de saltos automáticos no formulário, dispensando a passagem por quesitos para os quais, eventualmente, não havia informações e otimizando o tempo do recenseador e do entrevistado; e a dispensa do transporte de grandes volumes de questionários em papel e o manuseio dos mesmos nos centros de captura de dados, proporcionando ganhos na precisão da informação e agilidade no seu processamento.

Uma diferença substancial permitiu uma maior eficiência no acompanhamento e controle de coleta: o uso dos computadores de mão na coleta das informações pelos recenseadores. Após as entrevistas, o recenseador realizava a transmissão dos dados coletados para o computador central do IBGE, a partir de qualquer ponto de linha telefônica, sem a necessidade de passar por um microcomputador.

Tratamento das informações

Estimação de pessoas em domicílios fechados

Até hoje, em todos os Censos Demográficos, o IBGE sempre divulgou os totais da população levando em conta as pessoas residentes, na data de referência, em domicí-

lios particulares ocupados e nos domicílios coletivos. Os demais tipos de domicílios eram quantificados e divulgados sob a forma de Sinopse Preliminar.

Uma vez que a Contagem da População 2007 possui como principal objetivo a atualização das estimativas populacionais de cada município, foi decidido então estimar as pessoas residentes em domicílios fechados que por motivos vários não foi possível entrevistar seus moradores. Para tanto utilizou-se uma metodologia, denominada – *Metodologia de Estimação de Domicílios Fechados*, apresentada no Anexo 2. Isso significa dizer que os correspondentes populacionais estimados para esses domicílios encontram-se consolidados nos totais das tabelas aqui apresentadas, sendo que as características de cada pessoa não foram imputadas, mas sim atribuído código ignorado.

O procedimento de estimação foi aplicado aos domicílios efetivamente fechados, após todas as tentativas de obtenção da entrevista, que correspondem a 0,9% do total de domicílios ocupados nos municípios abrangidos pela Contagem.

Estimação de pessoas em setores com arquivos perdidos

Pela primeira vez em uma Contagem de População o IBGE utilizou um equipamento eletrônico, denominado *Personal Digital Assistant* – PDA, para a coleta dos dados no campo. Ao final da coleta dos dados de cada setor, era gerado um arquivo contendo todas as informações pesquisadas para cada um dos moradores. Ocorre que por algum problema durante a transmissão de apenas 11 setores, em nível Brasil, para o servidor central no Rio de Janeiro os mesmos foram perdidos ou danificados, restando, porém, os totais de domicílios particulares ocupados e das pessoas residentes.

Seguindo a mesma linha de raciocínio no que diz respeito ao aperfeiçoamento das estimativas populacionais em nível municipal, considerou-se relevante o aproveitamento desse contingente populacional já coletado, com 5 800 pessoas e 1 734 domicílios. Isso posto, foram criados os registros de domicílios e pessoas com código ignorado nas características de pessoas.

Imputação de características de pessoas recenseadas

Considerando-se que todas as críticas necessárias à correção de possíveis inconsistências estavam inseridas, testadas e aprovadas no aplicativo de coleta do PDA, para correção pelo Recenseador no ato da entrevista ou no encerramento da coleta no setor e antes da transmissão final do arquivo para a base de dados, não foi desenvolvido nenhum sistema probabilístico de detecção de erros e imputação. No entanto, após a etapa de consolidação do arquivo de dados (parte do sistema de processamento e apuração, que libera a base de dados para divulgação) foram detectadas algumas inconsistências que deveriam ter sido corrigidas através do aplicativo instalado no PDA. Do total de pessoas recenseadas, cerca de 0,2% estava com alguma inconsistência ou erro nos registros a ela associados, sendo os mesmos detectados a partir de um programa sob medida, atribuindo-se para correção destes casos o código ignorado.

Conceituação das características investigadas

População

A população foi constituída pelas pessoas residentes no domicílio, ou seja, aquelas que tinham o domicílio como local de residência habitual, quer estivessem presentes ou ausentes, temporariamente, na data de referência. Os moradores habituais do domicílio que estavam ausentes na data de referência foram recenseados, desde que sua ausência não tenha sido superior a 12 meses em relação àquela data.

Os dados apresentados para o total da população dizem respeito às pessoas residentes nos domicílios particulares – permanentes e improvisados - e domicílios coletivos.

Situação do domicílio

Segundo a localização do domicílio, a situação pode ser urbana ou rural, definida por Lei Municipal em vigor em 01 de abril de 2007. Na situação urbana, consideram-se as pessoas e os domicílios recenseados nas áreas urbanizadas ou não, correspondentes às cidades (sedes municipais), às vilas (sedes distritais) ou às áreas urbanas isoladas. A situação rural abrange a população e os domicílios recenseados em toda a área situada fora dos limites urbanos, inclusive os aglomerados rurais de extensão urbana, os povoados e os núcleos.

Unidades domiciliares

Considerou-se como Domicílio, o local estruturalmente separado e independente que se destina a servir de habitação a uma ou mais pessoas, ou que esteja sendo utilizado como tal. Por extensão, edifícios em construção, embarcações, veículos, tendas, barracas, grutas e outros locais que estavam, na data de referência da Contagem, servindo de moradia, também foram considerados como domicílios. As unidades domiciliares são compostas pelos domicílios particulares e unidades de habitação em domicílio coletivo.

Domicílio particular permanente

Considerou-se como Particular Permanente, aquele domicílio que foi construído para servir exclusivamente à habitação e, na data de referência, tinha a finalidade de servir de moradia a uma ou mais pessoas.

Domicílio particular permanente fechado

Considerou-se como Fechado, o domicílio particular permanente que, na data de referência, estava ocupado, porém seus moradores, durante todo o período da coleta, estiveram temporariamente ausentes.

Domicílio particular permanente vago

Considerou-se como Vago, o domicílio particular permanente que não tinha morador na data de referência.

Domicílio particular permanente de uso ocasional

Considerou-se como de Uso Ocasional, o domicílio particular permanente que, na data de referência, servia ocasionalmente de moradia.

Domicílio particular improvisado

Considerou-se como Improvisado, o domicílio localizado em uma edificação que não tinha dependências destinadas exclusivamente à moradia, assim como locais inadequados para habitação e que, na data de referência, estavam ocupados por morador(es).

Domicílio coletivo

Considerou-se como Coletivo, aquela instituição onde a relação entre as pessoas que nela habitavam na data de referência era restrita a normas de subordinação administrativa.

Pessoa responsável pelo domicílio

Considerou-se como Pessoa responsável pelo domicílio, a pessoa, homem ou mulher, responsável pelo domicílio ou que assim fosse considerada pelos demais moradores, com no mínimo 10 anos de idade.

Cônjuge, companheiro(a) de sexo diferente

Considerou-se como Cônjuge, companheiro(a) de sexo diferente, a pessoa (homem ou mulher) que vivia conjugalmente com a pessoa responsável pelo domicílio, sendo de sexo diferente e com no mínimo 10 anos de idade.

Cônjuge, companheiro(a) de mesmo sexo

Considerou-se como Cônjuge, companheiro(a) de mesmo sexo, a pessoa (homem ou mulher) que vivia conjugalmente com a pessoa responsável pelo domicílio, sendo ambas de mesmo sexo e com no mínimo 10 (dez) anos de idade.

Filho(a), enteado(a)

Considerou-se também o filho adotivo ou de criação e o filho somente do cônjuge, mesmo que o cônjuge já tivesse falecido ou não morasse mais no domicílio.

Pai, mãe, sogro(a)

Considerou-se também o(s) padrasto(s) e madrasta(s).

Neto(a), bisneto(a)

Considerou-se também o(s) que fosse(m) somente do cônjuge.

Irmão, irmã

Considerou-se também aqueles que não tinham laços consangüíneos (adotivos ou de criação).

Outro parente

Considerou-se como Outro parente, o avô(ó), bisavô(ó), genro ou nora, cunhado(a), tio(a), sobrinho(a), primo(a), sendo inclusive somente do cônjuge.

Agregado(a)

Considerou-se como Agregado, a pessoa que, sem ser parente, empregado doméstico ou parente do empregado doméstico, não pagava hospedagem nem contribuía para as despesas de alimentação e/ou moradia do domicílio.

Pensionista

Considerou-se como Pensionista, a pessoa residente em domicílio particular que, sem ser parente, pagava hospedagem.

Convivente

Considerou-se como Convivente, a pessoa residente em domicílio particular que, sem ser parente, dividia as despesas de alimentação e/ou moradia.

Empregado(a) doméstico(a)

Considerou-se como empregado doméstico, a pessoa residente no domicílio que prestava serviços domésticos remunerados a um ou mais moradores do domicílio;

Parente do empregado(a) doméstico(a)

Considerou-se como Parente do empregado doméstico a pessoa residente no domicílio que era parente do(a) empregado(a) doméstico(a) e que não prestava serviços domésticos remunerados a qualquer dos moradores do domicílio.

Individual em domicílio coletivo

Considerou-se como Individual, em domicílio coletivo a pessoa só que residia em domicílio coletivo, ainda que compartilhando a unidade com outra(s) pessoa(s) com a(s) qual(is) não tinha laços de parentesco ou dependência doméstica.

Idade

Os resultados obtidos referem-se às idades calculadas em anos ou em meses completos, na data de referência da Contagem da População. A idade foi formulada através de três quesitos: o mês e o ano de nascimento; a idade declarada; e a idade presumida para os que não sabiam informar o mês ou o ano de nascimento.

É apresentada, também, uma tabela com os dados relativos à forma de declaração da idade, ou seja, proveniente do mês e ano de nascimento ou da idade presumida.

Tabelas de resultados

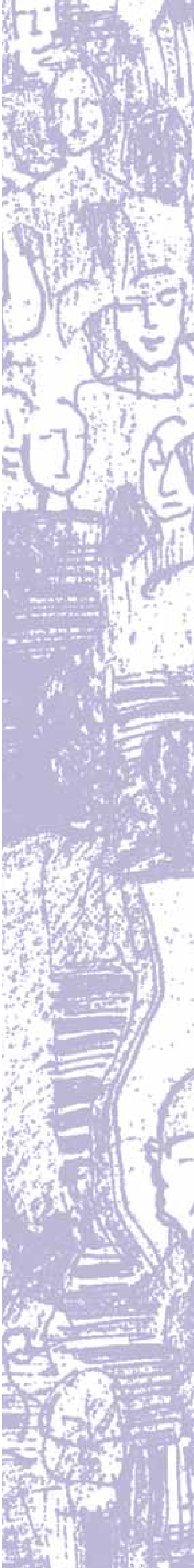

Tabela 1.1 - População recenseada e estimada, segundo as Grandes Regiões e as Unidades da Federação - 2007

Grandes Regiões e Unidades da Federação	População recenseada e estimada (1)
**Brasil**	**183 987 291**
**Norte**	**14 623 316**
Rondônia	1 453 756
Acre	655 385
Amazonas	3 221 939
Roraima	395 725
Pará	7 065 573
Amapá	587 311
Tocantins (2)	1 243 627
**Nordeste**	**51 534 406**
Maranhão	6 118 995
Piauí	3 032 421
Ceará	8 185 286
Rio Grande do Norte	3 013 740
Paraíba	3 641 395
Pernambuco	8 485 386
Alagoas (2)	3 037 103
Sergipe	1 939 426
Bahia (2)	14 080 654
**Sudeste**	**77 873 120**
Minas Gerais	19 273 506
Espírito Santo	3 351 669
Rio de Janeiro	15 420 375
São Paulo (2)	39 827 570
**Sul**	**26 733 595**
Paraná (2)	10 284 503
Santa Catarina	5 866 252
Rio Grande do Sul	10 582 840
**Centro-Oeste**	**13 222 854**
Mato Grosso do Sul	2 265 274
Mato Grosso	2 854 642
Goiás	5 647 035
Distrito Federal (3)	2 455 903

Fonte: IBGE, Contagem da População 2007.

(1) Inclusive a população estimada nos domicílios fechados. (2) Inclusive a população estimada nos domicílios provenientes de setores censitários cujos arquivos foram danificados. (3) População estimada.

Tabela 1.1.1 - População recenseada e estimada, segundo os municípios - Rondônia - 2007

Municípios	População recenseada e estimada (1)
**Rondônia**	**1 453 756**
Alta Floresta D'Oeste	23 857
Alto Alegre dos Parecis	11 615
Alto Paraíso	16 758
Alvorada D'Oeste	16 485
Ariquemes	82 388
Buritis	33 072
Cabixi	6 575
Cacaulândia	5 553
Cacoal	76 155
Campo Novo de Rondônia	12 455
Candeias do Jamari	16 736
Castanheiras	3 624
Cerejeiras	16 290
Chupinguaia	7 456
Colorado do Oeste	17 644
Corumbiara	9 476
Costa Marques	13 664
Cujubim	13 857
Espigão D'Oeste	27 867
Governador Jorge Teixeira	11 432
Guajará-Mirim	39 451
Itapuã do Oeste	7 905
Jaru	52 453
Ji-Paraná	107 679
Machadinho D'Oeste	31 475
Ministro Andreazza	10 343
Mirante da Serra	12 086
Monte Negro	12 357
Nova Brasilândia D'Oeste	17 170
Nova Mamoré	21 162
Nova União	7 750
Novo Horizonte do Oeste	9 648
Ouro Preto do Oeste	36 040
Parecis	4 583
Pimenta Bueno	32 893
Pimenteiras do Oeste	2 358
Porto Velho	369 345
Presidente Médici	22 197
Primavera de Rondônia	3 704
Rio Crespo	3 174
Rolim de Moura	48 894
Santa Luzia D'Oeste	9 264
São Felipe D'Oeste	6 286
São Francisco do Guaporé	15 710
São Miguel do Guaporé	22 622
Seringueiras	11 757
Teixeirópolis	4 919
Theobroma	9 952
Urupá	13 381
Vale do Anari	8 751
Vale do Paraíso	8 742
Vilhena	66 746

Fonte: IBGE, Contagem da População 2007.

(1) Inclusive a população estimada nos domicílios fechados.

Tabela 1.1.2 - População recenseada e estimada, segundo os municípios - Acre - 2007

Municípios	População recenseada e estimada (1)
**Acre**	**655 385**
Acrelândia	11 520
Assis Brasil	5 351
Brasiléia	19 065
Bujari	6 543
Capixaba	8 446
Cruzeiro do Sul	73 948
Epitaciolândia	13 434
Feijó	31 288
Jordão	6 059
Mâncio Lima	13 785
Manoel Urbano	7 148
Marechal Thaumaturgo	13 061
Plácido de Castro	17 258
Porto Acre	13 716
Porto Walter	8 170
Rio Branco	290 639
Rodrigues Alves	12 428
Santa Rosa do Purus	3 948
Sena Madureira	34 230
Senador Guiomard	18 863
Tarauacá	32 171
Xapuri	14 314

Fonte: IBGE, Contagem da População 2007.
(1) Inclusive a população estimada nos domicílios fechados.

CENSOS2007

Tabela 1.1.3 - População recenseada e estimada, segundo os municípios - Amazonas - 2007

Municípios	População recenseada e estimada (1)
**Amazonas**	**3 221 939**
Alvarães	13 010
Amaturá	8 384
Anamã	8 152
Anori	13 834
Apuí	17 451
Atalaia do Norte	13 682
Autazes	29 907
Barcelos	24 567
Barreirinha	26 645
Benjamin Constant	29 268
Beruri	13 809
Boa Vista do Ramos	13 138
Boca do Acre	29 818
Borba	31 098
Caapiranga	10 547
Canutama	11 463
Carauari	25 110
Careiro	31 070
Careiro da Várzea	23 023
Coari	65 222
Codajás	16 025
Eirunepé	29 411
Envira	16 438
Fonte Boa	19 726
Guajará	14 102
Humaitá	38 559
Ipixuna	17 177
Iranduba	32 869
Itacoatiara	84 676
Itamarati	8 078
Itapiranga	9 141
Japurá	5 281
Juruá	8 658
Jutaí	17 129
Lábrea	36 909
Manacapuru	82 309
Manaquiri	19 164
Manaus	1 646 602
Manicoré	44 327
Maraã	17 507
Maués	47 020
Nhamundá	17 553
Nova Olinda do Norte	29 184
Novo Airão	14 630
Novo Aripuanã	18 196
Parintins	102 044
Pauini	18 325
Presidente Figueiredo	24 360
Rio Preto da Eva	24 858
Santa Isabel do Rio Negro	16 921
Santo Antônio do Içá	29 249
São Gabriel da Cachoeira	39 129
São Paulo de Olivença	30 727
São Sebastião do Uatumã	8 731
Silves	8 211
Tabatinga	45 293
Tapauá	19 453
Tefé	62 920
Tonantins	19 090
Uarini	9 859
Urucará	15 716
Urucurituba	17 184

Fonte: IBGE, Contagem da População 2007.

(1) Inclusive a população estimada nos domicílios fechados.

Tabela 1.1.4 - População recenseada e estimada, segundo os municípios - Roraima - 2007

Municípios	População recenseada e estimada (1)
**Roraima**	**395 725**
Alto Alegre	14 386
Amajari	7 586
Boa Vista	249 853
Bonfim	10 231
Cantá	11 119
Caracaraí	17 981
Caroebe	7 086
Iracema	5 863
Mucajaí	12 546
Normandia	7 118
Pacaraima	8 640
Rorainópolis	24 466
São João da Baliza	5 727
São Luiz	5 720
Uiramutã	7 403

Fonte: IBGE, Contagem da População 2007.
(1) Inclusive a população estimada nos domicílios fechados.

Tabela 1.1.5 - População recenseada e estimada, segundo os municípios - Pará - 2007

(continua)

Municípios	População recenseada e estimada (1)
**Pará**	**7 065 573**
Abaetetuba	132 222
Abel Figueiredo	6 592
Acará	47 923
Afuá	31 183
Água Azul do Norte	28 658
Alenquer	52 661
Almeirim	30 903
Altamira	92 105
Anajás	24 942
Ananindeua (2)	484 278
Anapu	17 787
Augusto Corrêa	37 086
Aurora do Pará	21 239
Aveiro	18 830
Bagre	18 580
Baião	26 190
Bannach	3 812
Barcarena	84 560
Belém (2)	1 408 847
Belterra	12 707
Benevides	43 282
Bom Jesus do Tocantins	13 145
Bonito	11 279
Bragança	101 728
Brasil Novo	18 749
Brejo Grande do Araguaia	7 444
Breu Branco	47 069
Breves	94 458
Bujaru	22 535
Cachoeira do Arari	18 995
Cachoeira do Piriá	17 649
Cametá	110 323
Canaã dos Carajás	23 757
Capanema	61 350
Capitão Poço	50 839
Castanhal	152 126
Chaves	19 368
Colares	10 981
Conceição do Araguaia	45 267
Concórdia do Pará	21 422
Cumaru do Norte	10 452
Curionópolis	17 769
Curralinho	25 388
Curuá	11 928
Curuçá	33 768
Dom Eliseu	38 150
Eldorado dos Carajás	28 554
Faro	17 253
Floresta do Araguaia	14 964
Garrafão do Norte	24 619
Goianésia do Pará	27 166
Gurupá	24 384

Tabela 1.1.5 - População recenseada e estimada, segundo os municípios - Pará - 2007

(continuação)

Municípios	População recenseada e estimada (1)
Igarapé-Açu	33 778
Igarapé-Miri	54 673
Inhangapi	9 592
Ipixuna do Pará	39 563
Irituia	29 746
Itaituba	118 194
Itupiranga	42 002
Jacareacanga	37 073
Jacundá	51 511
Juruti	33 775
Limoeiro do Ajuru	23 284
Mãe do Rio	27 614
Magalhães Barata	7 650
Marabá (2)	196 468
Maracanã	28 296
Marapanim	26 651
Marituba	93 416
Medicilândia	22 624
Melgaço	17 845
Mocajuba	23 258
Moju	63 821
Monte Alegre	61 350
Muaná	28 796
Nova Esperança do Piriá	22 447
Nova Ipixuna	14 086
Nova Timboteua	12 103
Novo Progresso	21 598
Novo Repartimento	51 645
Óbidos	46 793
Oeiras do Pará	25 420
Oriximiná	55 175
Ourém	15 152
Ourilândia do Norte	20 415
Pacajá	38 365
Palestina do Pará	7 156
Paragominas	90 819
Parauapebas	133 298
Pau D'Arco	6 466
Peixe-Boi	7 679
Piçarra	12 707
Placas	17 898
Ponta de Pedras	24 276
Portel	45 586
Porto de Moz	26 489
Prainha	26 436
Primavera	10 463
Quatipuru	12 620
Redenção	64 583
Rio Maria	16 993
Rondon do Pará	45 016
Rurópolis	32 950
Salinópolis	37 066

Tabela 1.1.5 - População recenseada e estimada, segundo os municípios - Pará - 2007

(conclusão)

Municípios	População recenseada e estimada (1)
Salvaterra	17 077
Santa Bárbara do Pará	13 714
Santa Cruz do Arari	5 921
Santa Isabel do Pará	51 763
Santa Luzia do Pará	18 123
Santa Maria das Barreiras	16 012
Santa Maria do Pará	22 147
Santana do Araguaia	49 053
Santarém (2)	274 285
Santarém Novo	6 007
Santo Antônio do Tauá	24 814
São Caetano de Odivelas	16 179
São Domingos do Araguaia	21 094
São Domingos do Capim	27 094
São Félix do Xingu	59 238
São Francisco do Pará	11 913
São Geraldo do Araguaia	24 872
São João da Ponta	4 715
São João de Pirabas	18 919
São João do Araguaia	11 673
São Miguel do Guamá	42 987
São Sebastião da Boa Vista	20 500
Sapucaia	5 117
Senador José Porfírio	14 302
Soure	21 395
Tailândia	64 281
Terra Alta	9 861
Terra Santa	15 316
Tomé-Açu	47 081
Tracuateua	26 129
Trairão	16 097
Tucumã	26 513
Tucuruí	89 264
Ulianópolis	31 881
Uruará	35 076
Vigia	43 847
Viseu	53 217
Vitória do Xingu	9 693
Xinguara	38 457

Fonte: IBGE, Contagem da População 2007.

(1) Inclusive a população estimada nos domicílios fechados. (2) População estimada.

Tabela 1.1.6 - População recenseada e estimada, segundo os municípios - Amapá - 2007

Municípios	População recenseada e estimada (1)
**Amapá**	**587 311**
Amapá	7 492
Calçoene	8 656
Cutias	4 320
Ferreira Gomes	5 040
Itaubal	3 439
Laranjal do Jari	37 491
Macapá	344 153
Mazagão	13 862
Oiapoque	19 181
Pedra Branca do Amapari	7 332
Porto Grande	13 962
Pracuúba	3 353
Santana	92 098
Serra do Navio	3 772
Tartarugalzinho	12 395
Vitória do Jari	10 765

Fonte: IBGE, Contagem da População 2007.
(1) Inclusive a população estimada nos domicílios fechados.

Tabela 1.1.7 - População recenseada e estimada, segundo os municípios - Tocantins - 2007

(continua)

Municípios	População recenseada e estimada (1)
**Tocantins**	**1 243 627**
Abreulândia	2 245
Aguiarnópolis	3 995
Aliança do Tocantins	5 701
Almas	7 488
Alvorada	7 976
Ananás	9 358
Angico	3 169
Aparecida do Rio Negro	4 018
Aragominas	5 469
Araguacema	5 423
Araguaçu	8 989
Araguaína	115 759
Araguanã	5 000
Araguatins	25 973
Arapoema	6 839
Arraias	10 626
Augustinópolis	14 800
Aurora do Tocantins	3 385
Axixá do Tocantins	8 917
Babaçulândia	10 372
Bandeirantes do Tocantins	2 711
Barra do Ouro	3 581
Barrolândia	5 155
Bernardo Sayão	4 518
Bom Jesus do Tocantins	2 710
Brasilândia do Tocantins	2 119
Brejinho de Nazaré	5 295
Buriti do Tocantins	8 164
Cachoeirinha	2 171
Campos Lindos	7 615
Cariri do Tocantins	3 562
Carmolândia	2 313
Carrasco Bonito	3 314
Caseara	4 667
Centenário	2 386
Chapada da Natividade	3 680
Chapada de Areia	1 239
Colinas do Tocantins	29 298
Colméia	8 759
Combinado	4 878
Conceição do Tocantins	4 402
Couto de Magalhães	4 887
Cristalândia	6 520
Crixás do Tocantins	1 264
Darcinópolis	5 130
Dianópolis	18 584
Divinópolis do Tocantins	6 359
Dois Irmãos do Tocantins	7 060
Dueré	4 489
Esperantina	8 134
Fátima	3 984

Tabela 1.1.7 - População recenseada e estimada, segundo os municípios - Tocantins - 2007

(continuação)

Municípios	População recenseada e estimada (1)
Figueirópolis	4 820
Filadélfia	7 787
Formoso do Araguaia	18 225
Fortaleza do Tabocão	2 101
Goianorte	5 221
Goiatins	11 639
Guaraí	21 669
Gurupi	71 413
Ipueiras	1 698
Itacajá	6 386
Itaguatins	6 074
Itapiratins	3 421
Itaporã do Tocantins	2 989
Jaú do Tocantins	3 789
Juarina	2 141
Lagoa da Confusão	8 220
Lagoa do Tocantins	3 179
Lajeado	2 159
Lavandeira	1 590
Lizarda	3 634
Luzinópolis	2 784
Marianópolis do Tocantins	4 473
Mateiros	1 737
Maurilândia do Tocantins	3 185
Miracema do Tocantins	19 683
Miranorte	11 858
Monte do Carmo	6 387
Monte Santo do Tocantins	1 858
Muricilândia	2 850
Natividade	9 090
Nazaré	4 528
Nova Olinda	10 518
Nova Rosalândia	3 772
Novo Acordo	3 754
Novo Alegre	1 801
Novo Jardim	2 419
Oliveira de Fátima	1 081
Palmas	178 386
Palmeirante	4 689
Palmeiras do Tocantins	4 542
Palmeirópolis	8 120
Paraíso do Tocantins	40 290
Paranã	10 491
Pau D'Arco	4 767
Pedro Afonso	10 294
Peixe	8 750
Pequizeiro	4 799
Pindorama do Tocantins	4 397
Piraquê	3 014
Pium	6 403
Ponte Alta do Bom Jesus	4 529
Ponte Alta do Tocantins	6 569

Tabela 1.1.7 - População recenseada e estimada, segundo os municípios - Tocantins - 2007

(conclusão)

Municípios	População recenseada e estimada (1)
Porto Alegre do Tocantins	2 830
Porto Nacional	45 289
Praia Norte	7 060
Presidente Kennedy	3 680
Pugmil	2 165
Recursolândia	3 665
Riachinho	3 691
Rio da Conceição	1 454
Rio dos Bois	2 092
Rio Sono	6 167
Sampaio	3 672
Sandolândia	3 443
Santa Fé do Araguaia	5 610
Santa Maria do Tocantins	2 673
Santa Rita do Tocantins	2 260
Santa Rosa do Tocantins	4 417
Santa Tereza do Tocantins	2 297
Santa Terezinha do Tocantins	2 291
São Bento do Tocantins	4 447
São Félix do Tocantins	1 409
São Miguel do Tocantins	10 221
São Salvador do Tocantins	3 012
São Sebastião do Tocantins	4 244
São Valério da Natividade	4 885
Silvanópolis	5 098
Sítio Novo do Tocantins	9 302
Sucupira	1 667
Taguatinga	14 110
Taipas do Tocantins	1 916
Talismã	2 555
Tocantínia	6 663
Tocantinópolis	21 334
Tupirama	1 405
Tupiratins	2 007
Wanderlândia	9 317
Xambioá	10 856

Fonte: IBGE, Contagem da População 2007.

(1) Inclusive a população estimada nos domicílios fechados e nos domicílios provenientes de setor censitário cujo arquivo foi danificado.

Tabela 1.1.8 - População recenseada e estimada, segundo os municípios - Maranhão - 2007

(continua)

Municípios	População recenseada e estimada (1)
**Maranhão**	**6 118 995**
Açailândia	97 034
Afonso Cunha	5 651
Água Doce do Maranhão	11 829
Alcântara	21 349
Aldeias Altas	21 645
Altamira do Maranhão	7 365
Alto Alegre do Maranhão	22 002
Alto Alegre do Pindaré	31 992
Alto Parnaíba	10 304
Amapá do Maranhão	6 168
Amarante do Maranhão	35 727
Anajatuba	23 941
Anapurus	12 580
Apicum-Açu	13 216
Araguanã	9 918
Araioses	37 655
Arame	27 229
Arari	27 753
Axixá	14 249
Bacabal	95 124
Bacabeira	14 611
Bacuri	16 026
Bacurituba	5 425
Balsas	78 845
Barão de Grajaú	16 565
Barra do Corda	78 718
Barreirinhas	47 850
Bela Vista do Maranhão	8 603
Belágua	5 717
Benedito Leite	5 387
Bequimão	20 735
Bernardo do Mearim	5 972
Boa Vista do Gurupi	7 385
Bom Jardim	37 659
Bom Jesus das Selvas	23 827
Bom Lugar	12 825
Brejo	31 049
Brejo de Areia	5 803
Buriti	25 274
Buriti Bravo	22 279
Buriticupu	61 480
Buritirana	12 596
Cachoeira Grande	8 831
Cajapió	9 982
Cajari	12 842
Campestre do Maranhão	12 246
Cândido Mendes	18 820
Cantanhede	18 827
Capinzal do Norte	10 385
Carolina	24 442
Carutapera	20 285
Caxias	143 197

Tabela 1.1.8 - População recenseada e estimada, segundo os municípios - Maranhão - 2007

(continuação)

Municípios	População recenseada e estimada (1)
Cedral	9 841
Central do Maranhão	8 776
Centro do Guilherme	7 094
Centro Novo do Maranhão	15 127
Chapadinha	67 649
Cidelândia	12 407
Codó	110 574
Coelho Neto	44 031
Colinas	35 692
Conceição do Lago-Açu	14 063
Coroatá	60 589
Cururupu	34 018
Davinópolis	11 786
Dom Pedro	21 479
Duque Bacelar	10 384
Esperantinópolis	18 569
Estreito	26 490
Feira Nova do Maranhão	7 648
Fernando Falcão	8 105
Formosa da Serra Negra	16 882
Fortaleza dos Nogueiras	11 578
Fortuna	14 486
Godofredo Viana	10 452
Gonçalves Dias	16 444
Governador Archer	9 920
Governador Edison Lobão	14 086
Governador Eugênio Barros	15 857
Governador Luiz Rocha	6 884
Governador Newton Bello	11 346
Governador Nunes Freire	24 012
Graça Aranha	6 254
Grajaú	54 135
Guimarães	12 387
Humberto de Campos	24 275
Icatu	24 432
Igarapé do Meio	11 697
Igarapé Grande	10 676
Imperatriz	229 671
Itaipava do Grajaú	13 197
Itapecuru Mirim	54 573
Itinga do Maranhão	25 100
Jatobá	8 255
Jenipapo dos Vieiras	14 815
João Lisboa	19 928
Joselândia	15 583
Junco do Maranhão	4 014
Lago da Pedra	42 666
Lago do Junco	9 616
Lago dos Rodrigues	7 780
Lago Verde	14 580
Lagoa do Mato	10 225
Lagoa Grande do Maranhão	9 015
Lajeado Novo	6 620

Tabela 1.1.8 - População recenseada e estimada, segundo os municípios - Maranhão - 2007

(continuação)

Municípios	População recenseada e estimada (1)
Lima Campos	11 365
Loreto	10 340
Luís Domingues	6 672
Magalhães de Almeida	14 217
Maracaçumé	17 537
Marajá do Sena	6 790
Maranhãozinho	11 887
Mata Roma	13 812
Matinha	20 422
Matões	28 278
Matões do Norte	10 576
Milagres do Maranhão	7 617
Mirador	19 445
Miranda do Norte	17 742
Mirinzal	13 786
Monção	27 558
Montes Altos	8 828
Morros	17 077
Nina Rodrigues	10 003
Nova Colinas	4 829
Nova Iorque	4 892
Nova Olinda do Maranhão	17 121
Olho d'Água das Cunhãs	17 361
Olinda Nova do Maranhão	12 068
Paço do Lumiar	98 175
Palmeirândia	18 105
Paraibano	19 453
Parnarama	34 912
Passagem Franca	17 085
Pastos Bons	17 507
Paulino Neves	12 799
Paulo Ramos	16 129
Pedreiras	37 984
Pedro do Rosário	21 714
Penalva	33 473
Peri Mirim	12 219
Peritoró	19 017
Pindaré-Mirim	30 927
Pinheiro	74 123
Pio XII	21 821
Pirapemas	15 043
Poção de Pedras	15 853
Porto Franco	18 692
Porto Rico do Maranhão	6 900
Presidente Dutra	40 004
Presidente Juscelino	11 705
Presidente Médici	6 058
Presidente Sarney	15 606
Presidente Vargas	9 798
Primeira Cruz	11 999
Raposa	24 201
Riachão	21 016
Ribamar Fiquene	7 170
Rosário	37 920
Sambaíba	5 792
Santa Filomena do Maranhão	5 526

Tabela 1.1.8 - População recenseada e estimada, segundo os municípios - Maranhão - 2007

(conclusão)

Municípios	População recenseada e estimada (1)
Santa Helena	34 022
Santa Inês	82 026
Santa Luzia	69 306
Santa Luzia do Paruá	19 633
Santa Quitéria do Maranhão	28 341
Santa Rita	30 882
Santana do Maranhão	10 537
Santo Amaro do Maranhão	11 155
Santo Antônio dos Lopes	14 225
São Benedito do Rio Preto	17 191
São Bento	37 449
São Bernardo	25 480
São Domingos do Azeitão	6 953
São Domingos do Maranhão	32 557
São Félix de Balsas	4 398
São Francisco do Brejão	8 431
São Francisco do Maranhão	14 194
São João Batista	18 108
São João do Carú	12 281
São João do Paraíso	11 267
São João do Soter	16 592
São João dos Patos	23 576
São José de Ribamar	131 379
São José dos Basílios	7 282
São Luís	957 515
São Luís Gonzaga do Maranhão	19 655
São Mateus do Maranhão	38 045
São Pedro da Água Branca	11 113
São Pedro dos Crentes	4 020
São Raimundo das Mangabeiras	15 962
São Raimundo do Doca Bezerra	4 502
São Roberto	4 989
São Vicente Ferrer	19 692
Satubinha	8 399
Senador Alexandre Costa	9 071
Senador La Rocque	20 793
Serrano do Maranhão	10 576
Sítio Novo	15 549
Sucupira do Norte	10 235
Sucupira do Riachão	4 675
Tasso Fragoso	6 652
Timbiras	26 132
Timon	144 333
Trizidela do Vale	18 300
Tufilândia	5 517
Tuntum	37 894
Turiaçu	32 491
Turilândia	20 119
Tutóia	46 280
Urbano Santos	21 747
Vargem Grande	43 228
Viana	47 466
Vila Nova dos Martírios	8 671
Vitória do Mearim	30 935
Vitorino Freire	30 235
Zé Doca	45 008

Fonte: IBGE, Contagem da População 2007.
(1) Inclusive a população estimada nos domicílios fechados.

Tabela 1.1.9 - População recenseada e estimada, segundo os municípios - Piauí - 2007

(continua)

Municípios	População recenseada e estimada (1)
**Piauí**	**3 032 421**
Acauã	6 300
Agricolândia	4 980
Água Branca	15 887
Alagoinha do Piauí	7 447
Alegrete do Piauí	4 482
Alto Longá	13 612
Altos	38 328
Alvorada do Gurguéia	4 976
Amarante	17 316
Angical do Piauí	6 609
Anísio de Abreu	8 197
Antônio Almeida	3 140
Aroazes	5 857
Aroeiras do Itaim	2 654
Arraial	5 002
Assunção do Piauí	8 011
Avelino Lopes	11 507
Baixa Grande do Ribeiro	10 232
Barra D'Alcântara	3 774
Barras	43 328
Barreiras do Piauí	3 355
Barro Duro	6 661
Batalha	25 724
Bela Vista do Piauí	3 344
Belém do Piauí	2 788
Beneditinos	9 560
Bertolínia	5 302
Betânia do Piauí	9 312
Boa Hora	6 086
Bocaina	4 039
Bom Jesus	19 532
Bom Princípio do Piauí	5 276
Bonfim do Piauí	5 185
Boqueirão do Piauí	6 325
Brasileira	7 699
Brejo do Piauí	3 181
Buriti dos Lopes	19 150
Buriti dos Montes	7 820
Cabeceiras do Piauí	9 438
Cajazeiras do Piauí	3 193
Cajueiro da Praia	6 981
Caldeirão Grande do Piauí	5 622
Campinas do Piauí	5 598
Campo Alegre do Fidalgo	4 451
Campo Grande do Piauí	5 645
Campo Largo do Piauí	6 726
Campo Maior	44 548

Tabela 1.1.9 - População recenseada e estimada, segundo os municípios - Piauí - 2007

(continuação)

Municípios	População recenseada e estimada (1)
Canavieira	3 984
Canto do Buriti	20 673
Capitão de Campos	10 830
Capitão Gervásio Oliveira	3 865
Caracol	10 343
Caraúbas do Piauí	5 371
Caridade do Piauí	4 583
Castelo do Piauí	18 550
Caxingó	5 023
Cocal	26 201
Cocal de Telha	4 522
Cocal dos Alves	5 341
Coivaras	3 797
Colônia do Gurguéia	5 700
Colônia do Piauí	7 662
Conceição do Canindé	4 923
Coronel José Dias	4 356
Corrente	24 518
Cristalândia do Piauí	7 800
Cristino Castro	9 518
Curimatá	10 360
Currais	4 538
Curral Novo do Piauí	4 898
Curralinhos	4 072
Demerval Lobão	12 806
Dirceu Arcoverde	6 721
Dom Expedito Lopes	6 532
Dom Inocêncio	10 329
Domingos Mourão	4 312
Elesbão Veloso	14 174
Eliseu Martins	4 722
Esperantina	36 190
Fartura do Piauí	5 190
Flores do Piauí	4 468
Floresta do Piauí	2 551
Floriano	56 090
Francinópolis	5 292
Francisco Ayres	5 017
Francisco Macedo	2 255
Francisco Santos	8 293
Fronteiras	11 054
Geminiano	5 255
Gilbués	10 351
Guadalupe	9 587
Guaribas	4 343
Hugo Napoleão	3 674
Ilha Grande	8 420
Inhuma	14 973

Tabela 1.1.9 - População recenseada e estimada, segundo os municípios - Piauí - 2007

(continuação)

Municípios	População recenseada e estimada (1)
Ipiranga do Piauí	9 354
Isaías Coelho	7 790
Itainópolis	11 084
Itaueira	10 558
Jacobina do Piauí	5 597
Jaicós	16 826
Jardim do Mulato	4 171
Jatobá do Piauí	4 523
Jerumenha	4 371
João Costa	3 199
Joaquim Pires	13 779
Joca Marques	5 345
José de Freitas	35 164
Juazeiro do Piauí	4 769
Júlio Borges	5 250
Jurema	4 388
Lagoa Alegre	7 862
Lagoa de São Francisco	6 520
Lagoa do Barro do Piauí	4 543
Lagoa do Piauí	3 684
Lagoa do Sítio	5 042
Lagoinha do Piauí	2 563
Landri Sales	5 586
Luís Correia	26 147
Luzilândia	24 323
Madeiro	7 680
Manoel Emídio	5 357
Marcolândia	7 154
Marcos Parente	4 198
Massapê do Piauí	6 428
Matias Olímpio	10 465
Miguel Alves	32 178
Miguel Leão	1 194
Milton Brandão	7 018
Monsenhor Gil	10 321
Monsenhor Hipólito	7 163
Monte Alegre do Piauí	10 335
Morro Cabeça no Tempo	4 241
Morro do Chapéu do Piauí	6 377
Murici dos Portelas	7 655
Nazaré do Piauí	6 947
Nossa Senhora de Nazaré	4 048
Nossa Senhora dos Remédios	8 044
Nova Santa Rita	4 284
Novo Oriente do Piauí	6 191
Novo Santo Antônio	3 383
Oeiras	35 075
Olho D'Água do Piauí	2 637

Tabela 1.1.9 - População recenseada e estimada, segundo os municípios - Piauí - 2007

(continuação)

Municípios	População recenseada e estimada (1)
Padre Marcos	7 340
Paes Landim	4 462
Pajeú do Piauí	3 616
Palmeira do Piauí	4 985
Palmeirais	13 721
Paquetá	4 531
Parnaguá	10 313
Parnaíba	140 839
Passagem Franca do Piauí	4 127
Patos do Piauí	6 171
Pau D'Arco do Piauí	3 713
Paulistana	16 930
Pavussu	4 291
Pedro II	36 675
Pedro Laurentino	2 105
Picos	70 450
Pimenteiras	11 722
Pio IX	17 123
Piracuruca	25 625
Piripiri	60 249
Porto	11 492
Porto Alegre do Piauí	2 468
Prata do Piauí	3 159
Queimada Nova	9 146
Redenção do Gurguéia	8 263
Regeneração	17 633
Riacho Frio	4 884
Ribeira do Piauí	4 103
Ribeiro Gonçalves	6 577
Rio Grande do Piauí	6 430
Santa Cruz do Piauí	5 790
Santa Cruz dos Milagres	3 352
Santa Filomena	5 999
Santa Luz	5 280
Santa Rosa do Piauí	5 149
Santana do Piauí	4 841
Santo Antônio de Lisboa	5 729
Santo Antônio dos Milagres	1 963
Santo Inácio do Piauí	3 626
São Braz do Piauí	4 311
São Félix do Piauí	3 132
São Francisco de Assis do Piauí	5 015
São Francisco do Piauí	6 276

Tabela 1.1.9 - População recenseada e estimada, segundo os municípios - Piauí - 2007

(conclusão)

Municípios	População recenseada e estimada (1)
São Gonçalo do Gurguéia	2 455
São Gonçalo do Piauí	4 342
São João da Canabrava	4 364
São João da Fronteira	5 008
São João da Serra	6 672
São João da Varjota	4 776
São João do Arraial	7 081
São João do Piauí	18 689
São José do Divino	5 045
São José do Peixe	3 730
São José do Piauí	6 808
São Julião	5 908
São Lourenço do Piauí	4 899
São Luis do Piauí	2 596
São Miguel da Baixa Grande	2 083
São Miguel do Fidalgo	3 078
São Miguel do Tapuio	19 201
São Pedro do Piauí	13 083
São Raimundo Nonato	30 852
Sebastião Barros	4 178
Sebastião Leal	4 080
Sigefredo Pacheco	9 506
Simões	13 734
Simplício Mendes	11 472
Socorro do Piauí	4 599
Sussuapara	5 537
Tamboril do Piauí	2 897
Tanque do Piauí	2 605
Teresina	779 939
União	41 661
Uruçuí	19 017
Valença do Piauí	19 716
Várzea Branca	5 178
Várzea Grande	4 429
Vera Mendes	3 097
Vila Nova do Piauí	3 030
Wall Ferraz	4 452

Fonte: IBGE, Contagem da População 2007.

(1) Inclusive a população estimada nos domicílios fechados.

Tabela 1.1.10 - População recenseada e estimada, segundo os municípios - Ceará - 2007

(continua)

Municípios	População recenseada e estimada (1)
**Ceará**	**8 185 286**
Abaiara	10 227
Acarape	14 658
Acaraú	52 123
Acopiara	48 703
Aiuaba	15 585
Alcântaras	10 270
Altaneira	6 417
Alto Santo	19 154
Amontada	37 513
Antonina do Norte	6 761
Apuiarés	13 649
Aquiraz	67 265
Aracati	66 049
Aracoiaba	24 423
Ararendá	10 649
Araripe	21 214
Aratuba	12 129
Arneiroz	7 302
Assaré	21 616
Aurora	24 480
Baixio	5 780
Banabuiú	17 448
Barbalha	50 386
Barreira	18 453
Barro	20 673
Barroquinha	14 812
Baturité	31 669
Beberibe	46 155
Bela Cruz	29 566
Boa Viagem	53 538
Brejo Santo	39 613
Camocim	58 470
Campos Sales	25 553
Canindé	73 878
Capistrano	16 403
Caridade	17 977
Cariré	18 527
Caririaçu	26 325
Cariús	18 649
Carnaubal	16 001
Cascavel	63 932
Catarina	17 028
Catunda	10 508
Caucaia (2)	316 906
Cedro	24 607
Chaval	12 215
Choró	12 790
Chorozinho	18 261
Coreaú	21 171
Crateús	72 386
Crato	111 198
Croatá	17 317
Cruz	22 144
Deputado Irapuan Pinheiro	9 108
Ererê	6 927
Eusébio	38 189
Farias Brito	19 244
Forquilha	20 181
Fortaleza (2)	2 431 415

Tabela 1.1.10 - População recenseada e estimada, segundo os municípios - Ceará - 2007

(continuação)

Municípios	População recenseada e estimada (1)
Fortim	14 072
Frecheirinha	12 830
General Sampaio	6 540
Graça	15 297
Granja	51 410
Granjeiro	4 931
Groaíras	9 456
Guaiúba	22 405
Guaraciaba do Norte	36 705
Guaramiranga	4 307
Hidrolândia	18 534
Horizonte	48 660
Ibaretama	12 728
Ibiapina	23 088
Ibicuitinga	11 000
Icapuí	18 186
Icó	63 262
Iguatu	92 260
Independência	25 413
Ipaporanga	11 353
Ipaumirim	11 591
Ipu	39 438
Ipueiras	38 044
Iracema	14 313
Irauçuba	21 921
Itaiçaba	7 462
Itaitinga	31 107
Itapagé	45 426
Itapipoca	107 281
Itapiúna	17 602
Itarema	34 296
Itatira	17 689
Jaguaretama	17 851
Jaguaribara	9 780
Jaguaribe	35 237
Jaguaruana	30 965
Jardim	25 853
Jati	7 270
Jijoca de Jericoacoara	15 442
Juazeiro do Norte (2)	242 139
Jucás	22 890
Lavras da Mangabeira	29 872
Limoeiro do Norte	53 289
Madalena	17 051
Maracanaú (2)	197 301
Maranguape	102 982
Marco	23 107
Martinópole	10 304
Massapê	33 256
Mauriti	41 679
Meruoca	12 148
Milagres	27 355
Milhã	14 111
Miraíma	12 131
Missão Velha	33 690
Mombaça	44 364
Monsenhor Tabosa	16 557
Morada Nova	61 751
Moraújo	8 005
Morrinhos	21 111
Mucambo	14 007
Mulungu	10 975
Nova Olinda	12 974
Nova Russas	30 615

Tabela 1.1.10 - População recenseada e estimada, segundo os municípios - Ceará - 2007

(conclusão)

Municípios	População recenseada e estimada (1)
Novo Oriente	27 418
Ocara	23 359
Orós	21 268
Pacajus	54 881
Pacatuba	65 772
Pacoti	11 097
Pacujá	5 950
Palhano	8 797
Palmácia	10 352
Paracuru	30 665
Paraipaba	28 192
Parambu	30 596
Paramoti	11 573
Pedra Branca	40 762
Penaforte	7 715
Pentecoste	33 717
Pereiro	15 291
Pindoretama	17 143
Piquet Carneiro	14 736
Pires Ferreira	9 483
Poranga	11 905
Porteiras	14 792
Potengi	9 670
Potiretama	6 478
Quiterianópolis	20 079
Quixadá	76 105
Quixelô	15 708
Quixeramobim	68 966
Quixeré	18 652
Redenção	25 702
Reriutaba	19 310
Russas	63 975
Saboeiro	16 282
Salitre	15 798
Santa Quitéria	43 344
Santana do Acaraú	28 741
Santana do Cariri	17 574
São Benedito	43 077
São Gonçalo do Amarante	40 312
São João do Jaguaribe	8 310
São Luís do Curu	12 052
Senador Pompeu	25 290
Senador Sá	6 274
Sobral (2)	176 895
Solonópole	17 340
Tabuleiro do Norte	28 291
Tamboril	25 459
Tarrafas	8 734
Tauá	54 273
Tejuçuoca	15 062
Tianguá	64 612
Trairi	48 620
Tururu	13 350
Ubajara	29 569
Umari	7 591
Umirim	18 195
Uruburetama	19 218
Uruoca	12 973
Varjota	17 087
Várzea Alegre	37 740
Viçosa do Ceará	52 855

Fonte: IBGE, Contagem da População 2007.
(1) Inclusive a população estimada nos domicílios fechados. (2) População estimada

Tabela 1.1.11 - População recenseada e estimada, segundo os municípios - Rio Grande do Norte - 2007

(continua)

Municípios	População recenseada e estimada (1)
**Rio Grande do Norte**	**3 013 740**
Acari	10 911
Açu	51 262
Afonso Bezerra	10 339
Água Nova	2 843
Alexandria	13 729
Almino Afonso	4 948
Alto do Rodrigues	11 443
Angicos	11 227
Antônio Martins	6 997
Apodi	34 632
Areia Branca	24 398
Arês	12 236
Augusto Severo	8 936
Baía Formosa	8 466
Baraúna	23 098
Barcelona	3 928
Bento Fernandes	5 006
Bodó	2 542
Bom Jesus	8 478
Brejinho	11 135
Caiçara do Norte	6 384
Caiçara do Rio do Vento	3 064
Caicó	60 656
Campo Redondo	10 462
Canguaretama	29 334
Caraúbas	19 739
Carnaúba dos Dantas	6 836
Carnaubais	9 284
Ceará-Mirim	65 450
Cerro Corá	10 890
Coronel Ezequiel	5 255
Coronel João Pessoa	4 827
Cruzeta	7 825
Currais Novos	42 066
Doutor Severiano	6 431
Encanto	5 158
Equador	5 875
Espírito Santo	10 132
Extremoz	21 792
Felipe Guerra	5 680
Fernando Pedroza	2 876
Florânia	8 313
Francisco Dantas	2 928

Tabela 1.1.11 - População recenseada e estimada, segundo os municípios - Rio Grande do Norte - 2007

(continuação)

Municípios	População recenseada e estimada (1)
Frutuoso Gomes	4 360
Galinhos	2 149
Goianinha	20 347
Governador Dix-Sept Rosado	12 374
Grossos	9 441
Guamaré	11 737
Ielmo Marinho	11 649
Ipanguaçu	13 444
Ipueira	2 035
Itajá	6 410
Itaú	5 758
Jaçanã	7 788
Jandaíra	6 447
Janduís	5 416
Januário Cicco	8 294
Japi	5 610
Jardim de Angicos	2 536
Jardim de Piranhas	13 704
Jardim do Seridó	12 013
João Câmara	30 423
João Dias	2 723
José da Penha	5 982
Jucurutu	17 501
Jundiá	3 517
Lagoa d'Anta	5 940
Lagoa de Pedras	6 989
Lagoa de Velhos	2 699
Lagoa Nova	13 167
Lagoa Salgada	7 179
Lajes	10 412
Lajes Pintadas	4 217
Lucrécia	3 418
Luís Gomes	9 763
Macaíba	63 337
Macau	27 132
Major Sales	3 459
Marcelino Vieira	8 112
Martins	8 089
Maxaranguape	8 969
Messias Targino	3 795
Montanhas	12 393
Monte Alegre	20 590
Monte das Gameleiras	2 394
Mossoró	234 390

Tabela 1.1.11 - População recenseada e estimada, segundo os municípios - Rio Grande do Norte - 2007

(continuação)

Municípios	População recenseada e estimada (1)
Natal	774 230
Nísia Floresta	22 906
Nova Cruz	35 280
Olho-d'Água do Borges	4 442
Ouro Branco	4 973
Paraná	3 886
Paraú	3 880
Parazinho	4 772
Parelhas	19 972
Parnamirim	172 751
Passa e Fica	10 372
Passagem	2 629
Patu	11 303
Pau dos Ferros	26 728
Pedra Grande	3 918
Pedra Preta	2 659
Pedro Avelino	7 405
Pedro Velho	13 673
Pendências	12 505
Pilões	3 381
Poço Branco	12 288
Portalegre	6 855
Porto do Mangue	4 792
Presidente Juscelino	8 283
Pureza	8 030
Rafael Fernandes	4 608
Rafael Godeiro	3 131
Riacho da Cruz	3 025
Riacho de Santana	4 292
Riachuelo	6 824
Rio do Fogo	9 753
Rodolfo Fernandes	4 569
Ruy Barbosa	3 625
Santa Cruz	33 736
Santa Maria	4 659
Santana do Matos	14 312
Santana do Seridó	2 729
Santo Antônio	21 263
São Bento do Norte	3 529
São Bento do Trairí	3 702
São Fernando	3 381
São Francisco do Oeste	3 669
São Gonçalo do Amarante	77 363
São João do Sabugi	5 765

Tabela 1.1.11 - População recenseada e estimada, segundo os municípios - Rio Grande do Norte - 2007

(conclusão)

Municípios	População recenseada e estimada (1)
São José de Mipibu	36 990
São José do Campestre	11 744
São José do Seridó	3 925
São Miguel	22 579
São Miguel do Gostoso	8 810
São Paulo do Potengi	14 483
São Pedro	6 433
São Rafael	8 116
São Tomé	11 115
São Vicente	5 819
Senador Elói de Souza	5 906
Senador Georgino Avelino	3 690
Serra de São Bento	5 801
Serra do Mel	9 216
Serra Negra do Norte	7 241
Serrinha	6 740
Serrinha dos Pintos	4 360
Severiano Melo	5 671
Sítio Novo	5 212
Taboleiro Grande	2 278
Taipu	11 768
Tangará	13 081
Tenente Ananias	9 311
Tenente Laurentino Cruz	5 120
Tibau	3 750
Tibau do Sul	10 959
Timbaúba dos Batistas	2 295
Touros	29 436
Triunfo Potiguar	3 272
Umarizal	10 640
Upanema	12 719
Várzea	5 276
Venha-Ver	3 494
Vera Cruz	10 313
Viçosa	1 769
Vila Flor	2 647

Fonte: IBGE, Contagem da População 2007.
(1) Inclusive a população estimada nos domicílios fechados.

Tabela 1.1.12 - População recenseada e estimada, segundo os municípios - Paraíba - 2007

(continua)

Municípios	População recenseada e estimada (1)
**Paraíba**	**3 641 395**
Água Branca	9 224
Aguiar	5 629
Alagoa Grande	27 448
Alagoa Nova	19 163
Alagoinha	13 025
Alcantil	5 068
Algodão de Jandaíra	2 342
Alhandra	18 183
Amparo	2 007
Aparecida	7 323
Araçagi	17 376
Arara	12 356
Araruna	19 191
Areia	24 992
Areia de Baraúnas	2 096
Areial	6 234
Aroeiras	19 174
Assunção	3 336
Baía da Traição	7 630
Bananeiras	21 670
Baraúna	3 864
Barra de Santa Rosa	12 848
Barra de Santana	8 619
Barra de São Miguel	5 435
Bayeux	92 891
Belém	17 173
Belém do Brejo do Cruz	7 040
Bernardino Batista	3 164
Boa Ventura	5 800
Boa Vista	5 673
Bom Jesus	2 233
Bom Sucesso	5 152
Bonito de Santa Fé	10 253
Boqueirão	15 877
Borborema	5 009
Brejo do Cruz	12 424
Brejo dos Santos	5 743
Caaporã	19 388
Cabaceiras	4 907
Cabedelo	49 728
Cachoeira dos Índios	8 388
Cacimba de Areia	3 485
Cacimba de Dentro	17 108
Cacimbas	6 787
Caiçara	7 314
Cajazeiras	56 051
Cajazeirinhas	3 057
Caldas Brandão	5 363
Camalaú	5 761
Campina Grande	371 060
Campo de Santana	9 549
Capim	5 207
Caraúbas	3 824
Carrapateira	2 389
Casserengue	6 762
Catingueira	4 849

Tabela 1.1.12 - População recenseada e estimada, segundo os municípios - Paraíba - 2007

(continuação)

Municípios	População recenseda e estimada (1)
Catolé do Rocha	27 548
Caturité	4 467
Conceição	17 496
Condado	6 702
Conde	19 925
Congo	4 770
Coremas	15 236
Coxixola	1 705
Cruz do Espírito Santo	15 281
Cubati	6 356
Cuité	20 197
Cuité de Mamanguape	6 505
Cuitegi	7 047
Curral de Cima	5 469
Curral Velho	2 781
Damião	4 807
Desterro	7 929
Diamante	6 582
Dona Inês	10 832
Duas Estradas	3 748
Emas	3 257
Esperança	29 801
Fagundes	11 830
Frei Martinho	2 935
Gado Bravo	8 236
Guarabira	54 200
Gurinhém	13 209
Gurjão	2 985
Ibiara	6 139
Igaracy	6 334
Imaculada	11 451
Ingá	18 168
Itabaiana	24 752
Itaporanga	22 425
Itapororoca	15 966
Itatuba	9 841
Jacaraú	13 708
Jericó	7 825
João Pessoa	674 762
Juarez Távora	7 618
Juazeirinho	15 899
Junco do Seridó	6 486
Juripiranga	10 240
Juru	10 222
Lagoa	4 807
Lagoa de Dentro	7 258
Lagoa Seca	24 937
Lastro	2 834
Livramento	7 105
Logradouro	3 816
Lucena	10 943
Mãe d'Água	4 017
Malta	5 634
Mamanguape	40 283
Manaíra	10 986
Marcação	7 287
Mari	20 526
Marizópolis	6 214

Tabela 1.1.12 - População recenseada e estimada, segundo os municípios - Paraíba - 2007

(continuação)

Municípios	População recenseda e estimada (1)
Massaranduba	12 494
Mataraca	6 984
Matinhas	4 178
Mato Grosso	2 601
Maturéia	5 785
Mogeiro	12 305
Montadas	4 558
Monte Horebe	4 345
Monteiro	29 980
Mulungu	9 317
Natuba	10 216
Nazarezinho	6 933
Nova Floresta	10 032
Nova Olinda	6 280
Nova Palmeira	3 934
Olho d'Água	7 450
Olivedos	3 489
Ouro Velho	2 974
Parari	1 245
Passagem	2 124
Patos	97 276
Paulista	11 619
Pedra Branca	3 743
Pedra Lavrada	6 810
Pedras de Fogo	26 279
Pedro Régis	5 588
Piancó	15 881
Picuí	18 716
Pilar	11 301
Pilões	6 936
Pilõezinhos	5 319
Pirpirituba	10 232
Pitimbu	16 140
Pocinhos	15 956
Poço Dantas	3 919
Poço de José de Moura	3 959
Pombal	31 524
Prata	3 896
Princesa Isabel	19 330
Puxinanã	12 881
Queimadas	38 883
Quixabá	1 433
Remígio	16 748
Riachão	3 405
Riachão do Bacamarte	4 172
Riachão do Poço	4 239
Riacho de Santo Antônio	1 524
Riacho dos Cavalos	8 057
Rio Tinto	23 023
Salgadinho	3 367
Salgado de São Félix	12 526
Santa Cecília	7 016
Santa Cruz	6 480
Santa Helena	6 028
Santa Inês	3 707
Santa Luzia	14 292
Santa Rita	122 454
Santa Teresinha	4 644

Tabela 1.1.12 - População recenseada e estimada, segundo os municípios - Paraíba - 2007

(conclusão)

Municípios	População recenseada e estimada (1)
Santana de Mangueira	5 609
Santana dos Garrotes	7 610
Santarém	2 660
Santo André	2 641
São Bentinho	4 049
São Bento	29 196
São Domingos de Pombal	2 719
São Domingos do Cariri	2 265
São Francisco	3 442
São João do Cariri	4 438
São João do Rio do Peixe	17 773
São João do Tigre	4 578
São José da Lagoa Tapada	7 810
São José de Caiana	5 942
São José de Espinharas	4 913
São José de Piranhas	18 898
São José de Princesa	4 644
São José do Bonfim	3 180
São José do Brejo do Cruz	1 647
São José do Sabugi	3 986
São José dos Cordeiros	3 973
São José dos Ramos	5 482
São Mamede	7 782
São Miguel de Taipu	6 568
São Sebastião de Lagoa de Roça	10 908
São Sebastião do Umbuzeiro	3 061
Sapé	46 363
Seridó	9 737
Serra Branca	12 413
Serra da Raiz	3 130
Serra Grande	3 038
Serra Redonda	7 651
Serraria	6 602
Sertãozinho	4 213
Sobrado	7 340
Solânea	27 346
Soledade	13 128
Sossêgo	2 965
Sousa	63 783
Sumé	16 456
Taperoá	14 715
Tavares	14 021
Teixeira	13 685
Tenório	2 806
Triunfo	9 624
Uiraúna	14 454
Umbuzeiro	9 173
Várzea	2 457
Vieirópolis	4 756
Vista Serrana	3 253
Zabelê	2 024

Fonte: IBGE, Contagem da População 2007.

(1) Inclusive a população estimada nos domicílios fechados.

Tabela 1.1.13 - População recenseada e estimada, segundo os municípios - Pernambuco - 2007

(continua)

Municípios	População recenseada e estimada (1)
**Pernambuco**	**8 485 386**
Abreu e Lima	92 217
Afogados da Ingazeira	34 047
Afrânio	16 471
Agrestina	21 456
Água Preta	29 508
Águas Belas	37 992
Alagoinha	14 016
Aliança	34 740
Altinho	21 782
Amaraji	20 149
Angelim	9 836
Araçoiaba	16 520
Araripina	75 878
Arcoverde	64 863
Barra de Guabiraba	12 694
Barreiros	41 748
Belém de Maria	9 649
Belém de São Francisco	20 545
Belo Jardim	70 963
Betânia	11 548
Bezerros	56 629
Bodocó	33 381
Bom Conselho	43 397
Bom Jardim	39 023
Bonito	39 111
Brejão	9 341
Brejinho	7 158
Brejo da Madre de Deus	40 265
Buenos Aires	12 962
Buíque	49 937
Cabo de Santo Agostinho	163 139
Cabrobó	28 851
Cachoeirinha	17 421
Caetés	25 219
Calçado	11 337
Calumbi	7 577
Camaragibe	136 381
Camocim de São Félix	15 831
Camutanga	7 921
Canhotinho	24 218
Capoeiras	19 337
Carnaíba	18 345
Carnaubeira da Penha	11 689
Carpina	65 390
Caruaru (2)	289 086
Casinhas	14 103
Catende	33 479
Cedro	10 240
Chã de Alegria	11 636
Chã Grande	17 563
Condado	23 211
Correntes	16 330
Cortês	11 616
Cumaru	16 388
Cupira	22 106
Custódia	32 157
Dormentes	15 595
Escada	59 850
Exu	30 567
Feira Nova	19 276
Fernando de Noronha	2 801
Ferreiros	11 002

Tabela 1.1.13 - População recenseada e estimada, segundo os municípios - Pernambuco - 2007

(continuação)

Municípios	População recenseada e estimada (1)
Flores	21 962
Floresta	26 648
Frei Miguelinho	14 067
Gameleira	26 281
Garanhuns	124 996
Glória do Goitá	27 397
Goiana	71 796
Granito	6 593
Gravatá	71 570
Iati	17 733
Ibimirim	27 261
Ibirajuba	7 545
Igarassu	93 748
Iguaraci	11 880
Ilha de Itamaracá	17 573
Inajá	14 036
Ingazeira	4 444
Ipojuca	70 070
Ipubi	25 718
Itacuruba	4 097
Itaíba	26 735
Itambé	34 944
Itapetim	13 849
Itapissuma	22 852
Itaquitinga	14 985
Jaboatão dos Guararapes (2)	665 387
Jaqueira	12 102
Jataúba	14 813
Jatobá	13 797
João Alfredo	28 488
Joaquim Nabuco	15 947
Jucati	10 500
Jupi	13 628
Jurema	14 760
Lagoa do Carro	14 380
Lagoa do Itaenga	19 987
Lagoa do Ouro	11 655
Lagoa dos Gatos	15 967
Lagoa Grande	21 125
Lajedo	33 348
Limoeiro	55 560
Macaparana	23 078
Machados	11 152
Manari	16 540
Maraial	12 352
Mirandiba	13 304
Moreilândia	10 424
Moreno	52 830
Nazaré da Mata	29 202
Olinda (2)	391 433
Orobó	21 632
Orocó	13 167
Ouricuri	63 042
Palmares	56 643
Palmeirina	8 487
Panelas	24 918
Paranatama	11 669
Parnamirim	19 214
Passira	27 910
Paudalho	45 777
Paulista (2)	307 284
Pedra	20 132

Tabela 1.1.13 - População recenseada e estimada, segundo os municípios - Pernambuco - 2007

(conclusão)

Municípios	População recenseada e estimada (1)
Pesqueira	61 337
Petrolândia	30 597
Petrolina (2)	268 339
Poção	11 135
Pombos	21 810
Primavera	11 853
Quipapá	24 197
Quixaba	6 875
Recife (2)	1 533 580
Riacho das Almas	18 269
Ribeirão	38 755
Rio Formoso	21 024
Sairé	13 709
Salgadinho	7 770
Salgueiro	53 167
Saloá	15 027
Sanharó	17 627
Santa Cruz	13 644
Santa Cruz da Baixa Verde	11 610
Santa Cruz do Capibaribe	73 680
Santa Filomena	13 759
Santa Maria da Boa Vista	39 626
Santa Maria do Cambucá	12 348
Santa Terezinha	9 934
São Benedito do Sul	9 790
São Bento do Una	47 230
São Caitano	34 769
São João	21 011
São Joaquim do Monte	20 869
São José da Coroa Grande	17 090
São José do Belmonte	32 704
São José do Egito	30 469
São Lourenço da Mata	95 304
São Vicente Ferrer	16 598
Serra Talhada	76 198
Serrita	18 228
Sertânia	34 069
Sirinhaém	36 414
Solidão	5 837
Surubim	53 934
Tabira	25 836
Tacaimbó	12 095
Tacaratu	20 552
Tamandaré	18 137
Taquaritinga do Norte	21 447
Terezinha	6 496
Terra Nova	9 057
Timbaúba	51 529
Toritama	29 897
Tracunhaém	12 734
Trindade	24 642
Triunfo	15 225
Tupanatinga	18 913
Tuparetama	8 257
Venturosa	15 576
Verdejante	9 567
Vertente do Lério	7 500
Vertentes	17 021
Vicência	27 360
Vitória de Santo Antão	121 233
Xexéu	14 224

Fonte: IBGE, Contagem da População 2007.

(1) Inclusive a população estimada nos domicílios fechados. (2) População estimada.

Tabela 1.1.14 - População recenseada e estimada, segundo os municípios - Alagoas - 2007

(continua)

Municípios	População recenseada e estimada (1)
**Alagoas**	**3 037 103**
Água Branca	19 316
Anadia	17 274
Arapiraca	202 398
Atalaia	50 323
Barra de Santo Antônio	13 731
Barra de São Miguel	7 247
Batalha	16 247
Belém	5 031
Belo Monte	7 238
Boca da Mata	25 145
Branquinha	11 796
Cacimbinhas	9 843
Cajueiro	20 076
Campestre	6 016
Campo Alegre	45 307
Campo Grande	9 563
Canapi	17 793
Capela	17 034
Carneiros	8 044
Chã Preta	6 953
Coité do Nóia	10 916
Colônia Leopoldina	19 363
Coqueiro Seco	5 336
Coruripe	51 027
Craíbas	22 411
Delmiro Gouveia	46 599
Dois Riachos	10 885
Estrela de Alagoas	16 694
Feira Grande	21 180
Feliz Deserto	4 370
Flexeiras	11 928
Girau do Ponciano	35 162
Ibateguara	15 359
Igaci	25 140
Igreja Nova	22 925
Inhapi	17 637
Jacaré dos Homens	5 724
Jacuípe	6 883
Japaratinga	7 463
Jaramataia	5 939
Jequiá da Praia	11 430
Joaquim Gomes	21 735
Jundiá	4 569
Junqueiro	24 460
Lagoa da Canoa	17 889
Limoeiro de Anadia	25 484
Maceió	896 965
Major Isidoro	18 811
Mar Vermelho	4 014
Maragogi	25 726
Maravilha	10 203

Tabela 1.1.14 - População recenseada e estimada, segundo os municípios - Alagoas - 2007

(conclusão)

Municípios	População recenseada e estimada (1)
Marechal Deodoro	45 141
Maribondo	13 865
Mata Grande	24 599
Matriz de Camaragibe	24 656
Messias	15 072
Minador do Negrão	5 160
Monteirópolis	7 090
Murici	25 964
Novo Lino	11 903
Olho d'Água das Flores	19 885
Olho d'Água do Casado	8 139
Olho d'Água Grande	4 817
Olivença	10 522
Ouro Branco	11 049
Palestina	4 878
Palmeira dos Índios	70 151
Pão de Açúcar	23 855
Pariconha	10 209
Paripueira	9 725
Passo de Camaragibe	13 826
Paulo Jacinto	7 534
Penedo	59 020
Piaçabuçu	17 466
Pilar	31 627
Pindoba	3 126
Piranhas	23 910
Poço das Trincheiras	12 205
Porto Calvo	25 129
Porto de Pedras	10 321
Porto Real do Colégio	17 947
Quebrangulo	11 289
Rio Largo	65 432
Roteiro	6 692
Santa Luzia do Norte	7 248
Santana do Ipanema	42 296
Santana do Mundaú	11 663
São Brás	6 820
São José da Laje	22 282
São José da Tapera	30 129
São Luís do Quitunde	31 647
São Miguel dos Campos	51 473
São Miguel dos Milagres	7 219
São Sebastião	31 002
Satuba	14 154
Senador Rui Palmeira	12 641
Tanque d'Arca	5 695
Taquarana	18 157
Teotônio Vilela	40 291
Traipu	24 911
União dos Palmares	60 619
Viçosa	26 050

Fonte: IBGE, Contagem da População 2007.

(1) Inclusive a população estimada nos domicílios fechados e nos domicílios provenientes de setor censitário cujo arquivo foi danificado.

Tabela 1.1.15 - População recenseada e estimada, segundo os municípios - Sergipe - 2007

(continua)

Municípios	População recenseada e estimada (1)
**Sergipe**	**1 939 426**
Amparo de São Francisco	2 197
Aquidabã	19 185
Aracaju	520 303
Arauá	11 633
Areia Branca	16 072
Barra dos Coqueiros	19 218
Boquim	24 472
Brejo Grande	7 760
Campo do Brito	16 122
Canhoba	3 910
Canindé de São Francisco	21 806
Capela	27 913
Carira	18 965
Carmópolis	11 911
Cedro de São João	5 358
Cristinápolis	15 867
Cumbe	3 741
Divina Pastora	4 198
Estância	61 368
Feira Nova	5 628
Frei Paulo	12 589
Gararu	11 606
General Maynard	2 773
Gracho Cardoso	5 554
Ilha das Flores	8 598
Indiaroba	17 089
Itabaiana	83 161
Itabaianinha	37 431
Itabi	4 736
Itaporanga d'Ajuda	28 131
Japaratuba	15 450
Japoatã	13 539
Lagarto	88 980
Laranjeiras	23 923
Macambira	6 295
Malhada dos Bois	3 658
Malhador	11 728
Maruim	15 150

Tabela 1.1.15 - População recenseada e estimada, segundo os municípios - Sergipe - 2007

(conclusão)

Municípios	População recenseada e estimada (1)
Moita Bonita	10 910
Monte Alegre de Sergipe	13 199
Muribeca	7 225
Neópolis	18 909
Nossa Senhora Aparecida	8 517
Nossa Senhora da Glória	29 546
Nossa Senhora das Dores	23 800
Nossa Senhora de Lourdes	6 280
Nossa Senhora do Socorro	148 546
Pacatuba	12 377
Pedra Mole	2 774
Pedrinhas	8 389
Pinhão	5 590
Pirambu	8 227
Poço Redondo	28 969
Poço Verde	21 083
Porto da Folha	26 520
Propriá	27 629
Riachão do Dantas	19 019
Riachuelo	9 087
Ribeirópolis	15 676
Rosário do Catete	8 518
Salgado	18 563
Santa Luzia do Itanhy	13 041
Santa Rosa de Lima	3 844
Santana do São Francisco	6 596
Santo Amaro das Brotas	11 652
São Cristóvão	71 931
São Domingos	10 128
São Francisco	2 874
São Miguel do Aleixo	3 670
Simão Dias	37 145
Siriri	7 618
Telha	2 852
Tobias Barreto	47 265
Tomar do Geru	12 877
Umbaúba	20 562

Fonte: IBGE, Contagem da População 2007.
(1) Inclusive a população estimada nos domicílios fechados.

Tabela 1.1.16 - População recenseada e estimada, segundo os municípios - Bahia - 2007

(continua)

Municípios	População recenseada e estimada (1)
**Bahia**	**14 080 654**
Abaíra	8 638
Abaré	17 342
Acajutiba	14 628
Adustina	14 824
Água Fria	14 810
Aiquara	5 212
Alagoinhas	132 725
Alcobaça	19 840
Almadina	6 687
Amargosa	33 554
Amélia Rodrigues	22 096
América Dourada	16 189
Anagé	25 716
Andaraí	14 088
Andorinha	14 132
Angical	14 700
Anguera	9 523
Antas	16 446
Antônio Cardoso	12 074
Antônio Gonçalves	10 653
Aporá	17 870
Apuarema	7 379
Araças	11 662
Aracatu	14 168
Araci	51 912
Aramari	9 527
Arataca	10 717
Aratuípe	8 507
Aurelino Leal	14 458
Baianópolis	13 437
Baixa Grande	20 980
Banzaê	10 865
Barra	47 755
Barra da Estiva	20 750
Barra do Choça	32 489
Barra do Mendes	14 054
Barra do Rocha	6 133
Barreiras	129 501
Barro Alto	13 403
Barro Preto	6 669
Barrocas	13 182
Belmonte	21 479
Belo Campo	15 262
Biritinga	13 961
Boa Nova	16 007
Boa Vista do Tupim	17 841
Bom Jesus da Lapa	62 199
Bom Jesus da Serra	10 295
Boninal	13 227
Bonito	13 678
Boquira	21 856

Tabela 1.1.16 - População recenseada e estimada, segundo os municípios - Bahia - 2007

(continuação)

Municípios	População recenseada e estimada (1)
Botuporã	11 026
Brejões	12 677
Brejolândia	9 337
Brotas de Macaúbas	10 922
Brumado	62 381
Buerarema	19 956
Buritirama	18 656
Caatiba	10 367
Cabaceiras do Paraguaçu	17 502
Cachoeira	32 252
Caculé	21 847
Caém	10 377
Caetanos	12 133
Caetité	46 192
Cafarnaum	17 402
Cairu	13 712
Caldeirão Grande	13 072
Camacan	30 289
Camaçari (2)	220 495
Camamu	32 172
Campo Alegre de Lourdes	26 935
Campo Formoso	65 137
Canápolis	10 577
Canarana	24 436
Canavieiras	35 743
Candeal	9 019
Candeias	78 618
Candiba	12 352
Cândido Sales	26 727
Cansanção	32 789
Canudos	14 656
Capela do Alto Alegre	12 311
Capim Grosso	25 823
Caraíbas	10 541
Caravelas	21 150
Cardeal da Silva	8 280
Carinhanha	28 879
Casa Nova	62 862
Castro Alves	24 437
Catolândia	3 767
Catu	48 697
Caturama	8 545
Central	17 320
Chorrochó	10 571
Cícero Dantas	30 827
Cipó	15 063
Coaraci	22 764
Cocos	17 394
Conceição da Feira	19 091
Conceição do Almeida	17 684
Conceição do Coité	60 835
Conceição do Jacuípe	27 522

Tabela 1.1.16 - População recenseada e estimada, segundo os municípios - Bahia - 2007

(continuação)

Municípios	População recenseada e estimada (1)
Conde	22 034
Condeúba	17 004
Contendas do Sincorá	3 857
Coração de Maria	23 161
Cordeiros	8 518
Coribe	14 555
Coronel João Sá	18 168
Correntina	31 658
Cotegipe	13 663
Cravolândia	5 427
Crisópolis	19 510
Cristópolis	13 595
Cruz das Almas	54 827
Curaçá	32 449
Dário Meira	12 565
Dias d'Ávila	53 821
Dom Basílio	11 087
Dom Macedo Costa	3 809
Elísio Medrado	7 907
Encruzilhada	22 525
Entre Rios	38 886
Érico Cardoso	10 609
Esplanada	31 118
Euclides da Cunha	56 625
Eunápolis	93 984
Fátima	18 918
Feira da Mata	6 328
Feira de Santana (2)	571 997
Filadélfia	15 979
Firmino Alves	5 577
Floresta Azul	10 333
Formosa do Rio Preto	20 845
Gandu	30 091
Gavião	4 444
Gentio do Ouro	11 326
Glória	13 879
Gongogi	6 845
Governador Mangabeira	19 828
Guajeru	7 062
Guanambi	76 230
Guaratinga	22 621
Heliópolis	14 020
Iaçu	27 835
Ibiassucê	9 507
Ibicaraí	24 752
Ibicoara	15 856
Ibicuí	15 777
Ibipeba	16 762
Ibipitanga	13 841
Ibiquera	5 007
Ibirapitanga	23 197
Ibirapuã	7 534

Tabela 1.1.16 - População recenseada e estimada, segundo os municípios - Bahia - 2007

(continuação)

Municípios	População recenseada e estimada (1)
Ibirataia	23 940
Ibitiara	15 802
Ibititá	18 614
Ibotirama	25 292
Ichu	5 881
Igaporã	14 499
Igrapiúna	13 246
Iguaí	27 849
Ilhéus (2)	220 144
Inhambupe	34 388
Ipecaetá	16 055
Ipiaú	42 561
Ipirá	60 043
Ipupiara	8 931
Irajuba	7 215
Iramaia	15 134
Iraquara	22 607
Irará	25 012
Irecê	62 676
Itabela	25 821
Itaberaba	59 393
Itabuna (2)	210 604
Itacaré	24 720
Itaeté	14 154
Itagi	13 882
Itagibá	16 272
Itagimirim	7 049
Itaguaçu da Bahia	12 545
Itaju do Colônia	7 673
Itajuípe	20 343
Itamaraju	65 327
Itamari	8 468
Itambé	33 687
Itanagra	6 605
Itanhém	20 636
Itaparica	19 897
Itapé	11 123
Itapebi	11 520
Itapetinga	63 243
Itapicuru	30 703
Itapitanga	10 106
Itaquara	7 564
Itarantim	17 615
Itatim	14 569
Itiruçu	15 764
Itiúba	35 749
Itororó	20 165
Ituaçu	17 939
Ituberá	23 530
Iuiú	11 469
Jaborandi	8 931
Jacaraci	14 346

Tabela 1.1.16 - População recenseada e estimada, segundo os municípios - Bahia - 2007

(continuação)

Municípios	População recenseada e estimada (1)
Jacobina	76 463
Jaguaquara	46 641
Jaguarari	29 097
Jaguaripe	16 207
Jandaíra	9 758
Jequié	145 964
Jeremoabo	37 431
Jiquiriçá	13 386
Jitaúna	16 839
João Dourado	20 834
Juazeiro (2)	230 538
Jucuruçu	10 599
Jussara	14 833
Jussari	6 857
Jussiape	8 465
Lafaiete Coutinho	3 526
Lagoa Real	13 795
Laje	21 104
Lajedão	3 469
Lajedinho	4 329
Lajedo do Tabocal	8 591
Lamarão	11 988
Lapão	25 557
Lauro de Freitas	144 492
Lençóis	9 617
Licínio de Almeida	12 695
Livramento de Nossa Senhora	42 146
Luís Eduardo Magalhães	44 265
Macajuba	11 207
Macarani	16 046
Macaúbas	45 958
Macururé	7 779
Madre de Deus	15 432
Maetinga	8 793
Maiquinique	8 324
Mairi	19 259
Malhada	16 085
Malhada de Pedras	7 620
Manoel Vitorino	14 262
Mansidão	11 694
Maracás	34 221
Maragogipe	42 079
Maraú	17 029
Marcionílio Souza	10 716
Mascote	16 061
Mata de São João	37 201
Matina	12 322
Medeiros Neto	21 866
Miguel Calmon	27 213
Milagres	11 771
Mirangaba	17 474
Mirante	9 174

Tabela 1.1.16 - População recenseada e estimada, segundo os municípios - Bahia - 2007

(continuação)

Municípios	População recenseada e estimada (1)
Monte Santo	52 249
Morpará	8 586
Morro do Chapéu	34 012
Mortugaba	13 905
Mucugê	14 131
Mucuri	33 143
Mulungu do Morro	13 755
Mundo Novo	23 822
Muniz Ferreira	6 990
Muquém de São Francisco	10 096
Muritiba	27 212
Mutuípe	21 181
Nazaré	26 506
Nilo Peçanha	12 531
Nordestina	12 172
Nova Canaã	18 829
Nova Fátima	7 674
Nova Ibiá	6 871
Nova Itarana	7 423
Nova Redenção	8 943
Nova Soure	25 697
Nova Viçosa	34 623
Novo Horizonte	10 304
Novo Triunfo	14 153
Olindina	23 791
Oliveira dos Brejinhos	22 609
Ouriçangas	7 802
Ourolândia	16 302
Palmas de Monte Alto	21 107
Palmeiras	8 040
Paramirim	20 055
Paratinga	28 671
Paripiranga	28 347
Pau Brasil	12 155
Paulo Afonso	101 952
Pé de Serra	14 160
Pedrão	7 238
Pedro Alexandre	17 092
Piatã	18 085
Pilão Arcado	32 844
Pindaí	15 306
Pindobaçu	20 312
Pintadas	10 551
Piraí do Norte	8 897
Piripá	13 515
Piritiba	24 189
Planaltino	8 472
Planalto	21 486
Poções	44 759
Pojuca	30 221
Ponto Novo	14 820
Porto Seguro	114 459

Tabela 1.1.16 - População recenseada e estimada, segundo os municípios - Bahia - 2007

(continuação)

Municípios	População recenseada e estimada (1)
Potiraguá	10 350
Prado	25 429
Presidente Dutra	13 822
Presidente Jânio Quadros	14 803
Presidente Tancredo Neves	22 684
Queimadas	27 186
Quijingue	27 068
Quixabeira	9 348
Rafael Jambeiro	23 107
Remanso	38 004
Retirolândia	11 938
Riachão das Neves	22 528
Riachão do Jacuípe	32 522
Riacho de Santana	29 425
Ribeira do Amparo	14 101
Ribeira do Pombal	47 400
Ribeirão do Largo	14 297
Rio de Contas	13 447
Rio do Antônio	15 096
Rio do Pires	11 385
Rio Real	36 169
Rodelas	7 023
Ruy Barbosa	29 358
Salinas da Margarida	13 090
Salvador (2)	2 892 625
Santa Bárbara	19 440
Santa Brígida	15 483
Santa Cruz Cabrália	25 110
Santa Cruz da Vitória	6 386
Santa Inês	10 564
Santa Luzia	15 184
Santa Maria da Vitória	40 571
Santa Rita de Cássia	26 135
Santa Teresinha	9 914
Santaluz	33 633
Santana	25 947
Santanópolis	9 199
Santo Amaro	58 028
Santo Antônio de Jesus	84 256
Santo Estêvão	44 532
São Desidério	25 158
São Domingos	8 818
São Felipe	20 265
São Félix	15 302
São Félix do Coribe	12 815
São Francisco do Conde	29 829
São Gabriel	18 468
São Gonçalo dos Campos	29 205
São José da Vitória	6 095
São José do Jacuípe	10 477
São Miguel das Matas	10 270
São Sebastião do Passé	40 321
Sapeaçu	16 518

Tabela 1.1.16 - População recenseada e estimada, segundo os municípios - Bahia - 2007

(conclusão)

Municípios	População recenseada e estimada (1)
Sátiro Dias	18 104
Saubara	11 051
Saúde	11 866
Seabra	40 543
Sebastião Laranjeiras	10 712
Senhor do Bonfim	72 511
Sento Sé	36 517
Serra do Ramalho	31 130
Serra Dourada	17 422
Serra Preta	15 149
Serrinha	71 383
Serrolândia	12 120
Simões Filho	109 269
Sítio do Mato	12 567
Sítio do Quinto	14 069
Sobradinho	21 315
Souto Soares	18 013
Tabocas do Brejo Velho	12 281
Tanhaçu	19 591
Tanque Novo	15 745
Tanquinho	7 589
Taperoá	18 217
Tapiramutá	17 254
Teixeira de Freitas	118 702
Teodoro Sampaio	8 306
Teofilândia	20 702
Teolândia	12 429
Terra Nova	12 310
Tremedal	18 483
Tucano	48 740
Uauá	24 662
Ubaíra	20 708
Ubaitaba	20 478
Ubatã	24 791
Uibaí	13 719
Umburanas	16 081
Una	25 277
Urandi	15 822
Uruçuca	22 070
Utinga	19 229
Valença	84 931
Valente	21 512
Várzea da Roça	14 479
Várzea do Poço	8 569
Várzea Nova	13 949
Varzedo	9 054
Vera Cruz	35 060
Vereda	7 174
Vitória da Conquista (2)	308 204
Wagner	8 610
Wanderley	12 982
Wenceslau Guimarães	23 985
Xique-Xique	45 700

Fonte: IBGE, Contagem da População 2007.

(1) Inclusive a população estimada nos domicílios fechados e nos domicílios provenientes de setor censitário cujo arquivo foi danificado.

Tabela 1.1.17 - População recenseada e estimada, segundo os municípios - Minas Gerais - 2007

(continua)

Municípios	População recenseada e estimada (1)
**Minas Gerais**	**19 273 506**
Abadia dos Dourados	6 556
Abaeté	22 474
Abre Campo	12 867
Acaiaca	4 056
Açucena	11 127
Água Boa	16 435
Água Comprida	2 093
Aguanil	4 054
Águas Formosas	18 518
Águas Vermelhas	12 674
Aimorés	24 232
Aiuruoca	6 099
Alagoa	2 825
Albertina	2 872
Além Paraíba	33 495
Alfenas	71 628
Alfredo Vasconcelos	5 900
Almenara	36 907
Alpercata	7 007
Alpinópolis	17 821
Alterosa	13 286
Alto Caparaó	5 048
Alto Jequitibá	7 976
Alto Rio Doce	12 657
Alvarenga	4 558
Alvinópolis	15 251
Alvorada de Minas	3 482
Amparo do Serra	5 245
Andradas	34 956
Andrelândia	12 035
Angelândia	8 130
Antônio Carlos	11 172
Antônio Dias	9 435
Antônio Prado de Minas	1 962
Araçaí	2 384
Aracitaba	1 875
Araçuaí	36 083
Araguari	106 403
Arantina	2 544
Araponga	8 029
Araporã	6 113
Arapuá	2 699
Araújos	7 201
Araxá	87 764
Arceburgo	7 994
Arcos	34 763
Areado	13 181
Argirita	2 995
Aricanduva	4 832
Arinos	17 592
Astolfo Dutra	12 510
Ataléia	15 078
Augusto de Lima	4 589
Baependi	18 016
Baldim	8 274
Bambuí	21 850
Bandeira	5 337

Tabela 1.1.17 - População recenseada e estimada, segundo os municípios - Minas Gerais - 2007

(continuação)

Municípios	População recenseada e estimada (1)
Bandeira do Sul	5 106
Barão de Cocais	26 421
Barão de Monte Alto	5 656
Barbacena	122 377
Barra Longa	6 965
Barroso	19 352
Bela Vista de Minas	9 968
Belmiro Braga	3 067
Belo Horizonte (2)	2 412 937
Belo Oriente	21 369
Belo Vale	7 267
Berilo	13 214
Berizal	4 399
Bertópolis	4 588
Betim (2)	415 098
Bias Fortes	3 880
Bicas	13 638
Biquinhas	2 592
Boa Esperança	37 801
Bocaina de Minas	5 034
Bocaiúva	44 657
Bom Despacho	42 260
Bom Jardim de Minas	6 481
Bom Jesus da Penha	3 787
Bom Jesus do Amparo	5 412
Bom Jesus do Galho	15 198
Bom Repouso	10 482
Bom Sucesso	17 194
Bonfim	6 715
Bonfinópolis de Minas	5 828
Bonito de Minas	8 787
Borda da Mata	14 892
Botelhos	14 853
Botumirim	6 435
Brás Pires	4 592
Brasilândia de Minas	12 821
Brasília de Minas	31 165
Brasópolis	14 452
Braúnas	5 208
Brumadinho	31 965
Bueno Brandão	10 864
Buenópolis	9 522
Bugre	3 960
Buritis	21 472
Buritizeiro	26 133
Cabeceira Grande	6 294
Cabo Verde	13 614
Cachoeira da Prata	3 802
Cachoeira de Minas	10 820
Cachoeira de Pajeú	9 089
Cachoeira Dourada	2 470
Caetanópolis	9 490
Caeté	39 039
Caiana	4 537
Cajuri	4 015
Caldas	13 901
Camacho	3 204
Camanducaia	19 708

Tabela 1.1.17 - População recenseada e estimada, segundo os municípios - Minas Gerais - 2007

(continuação)

Municípios	População recenseada e estimada (1)
Cambuí	25 010
Cambuquira	12 520
Campanário	3 592
Campanha	15 169
Campestre	20 251
Campina Verde	18 680
Campo Azul	3 828
Campo Belo	51 375
Campo do Meio	11 476
Campo Florido	6 570
Campos Altos	13 184
Campos Gerais	26 954
Cana Verde	5 712
Canaã	4 668
Canápolis	11 313
Candeias	15 499
Cantagalo	3 967
Caparaó	4 863
Capela Nova	4 598
Capelinha	33 061
Capetinga	7 154
Capim Branco	8 763
Capinópolis	15 302
Capitão Andrade	4 801
Capitão Enéas	14 106
Capitólio	7 634
Caputira	8 855
Caraí	21 530
Caranaíba	3 445
Carandaí	22 240
Carangola	32 068
Caratinga	81 731
Carbonita	10 145
Careaçu	6 029
Carlos Chagas	20 812
Carmésia	2 550
Carmo da Cachoeira	11 656
Carmo da Mata	10 942
Carmo de Minas	13 657
Carmo do Cajuru	18 943
Carmo do Paranaíba	30 712
Carmo do Rio Claro	19 480
Carmópolis de Minas	15 743
Carneirinho	8 859
Carrancas	4 015
Carvalhópolis	3 234
Carvalhos	4 611
Casa Grande	2 100
Cascalho Rico	2 799
Cássia	17 067
Cataguases	67 384
Catas Altas	4 561
Catas Altas da Noruega	3 424
Catuji	6 597
Catuti	5 303
Caxambu	21 009
Cedro do Abaeté	1 203
Central de Minas	6 590

Tabela 1.1.17 - População recenseada e estimada, segundo os municípios - Minas Gerais - 2007

(continuação)

Municípios	População recenseada e estimada (1)
Centralina	10 219
Chácara	2 613
Chalé	5 465
Chapada do Norte	15 449
Chapada Gaúcha	10 266
Chiador	2 893
Cipotânea	6 539
Claraval	4 295
Claro dos Poções	8 131
Cláudio	24 590
Coimbra	6 886
Coluna	9 281
Comendador Gomes	3 087
Comercinho	8 720
Conceição da Aparecida	10 215
Conceição da Barra de Minas	3 960
Conceição das Alagoas	20 426
Conceição das Pedras	2 726
Conceição de Ipanema	4 396
Conceição do Mato Dentro	18 070
Conceição do Pará	4 725
Conceição do Rio Verde	12 708
Conceição dos Ouros	10 204
Cônego Marinho	6 279
Confins	5 680
Congonhal	9 692
Congonhas	45 984
Congonhas do Norte	5 110
Conquista	6 580
Conselheiro Lafaiete	109 280
Conselheiro Pena	21 793
Consolação	1 695
Contagem (2)	608 650
Coqueiral	9 466
Coração de Jesus	26 131
Cordisburgo	9 033
Cordislândia	3 570
Corinto	22 741
Coroaci	10 776
Coromandel	27 392
Coronel Fabriciano	100 805
Coronel Murta	9 120
Coronel Pacheco	2 457
Coronel Xavier Chaves	3 194
Córrego Danta	3 423
Córrego do Bom Jesus	3 724
Córrego Fundo	5 635
Córrego Novo	3 155
Couto de Magalhães de Minas	4 332
Crisólita	5 659
Cristais	10 631
Cristália	5 731
Cristiano Otoni	4 881
Cristina	10 955
Crucilândia	4 593
Cruzeiro da Fortaleza	3 760
Cruzília	14 656
Cuparaque	4 404

Tabela 1.1.17 - População recenseada e estimada, segundo os municípios - Minas Gerais - 2007

(continuação)

Municípios	População recenseada e estimada (1)
Curral de Dentro	6 907
Curvelo	71 611
Datas	5 418
Delfim Moreira	7 834
Delfinópolis	6 698
Delta	6 600
Descoberto	4 876
Desterro de Entre Rios	6 914
Desterro do Melo	3 198
Diamantina	44 746
Diogo de Vasconcelos	3 941
Dionísio	10 234
Divinésia	3 276
Divino	19 245
Divino das Laranjeiras	4 934
Divinolândia de Minas	6 724
Divinópolis (2)	209 921
Divisa Alegre	5 793
Divisa Nova	5 619
Divisópolis	7 852
Dom Bosco	3 781
Dom Cavati	5 593
Dom Joaquim	4 530
Dom Silvério	5 284
Dom Viçoso	3 020
Dona Eusébia	5 569
Dores de Campos	9 276
Dores de Guanhães	5 528
Dores do Indaiá	13 996
Dores do Turvo	4 572
Doresópolis	1 492
Douradoquara	1 846
Durandé	6 932
Elói Mendes	24 161
Engenheiro Caldas	10 317
Engenheiro Navarro	7 079
Entre Folhas	4 931
Entre Rios de Minas	13 887
Ervália	18 002
Esmeraldas	55 436
Espera Feliz	20 835
Espinosa	31 322
Espírito Santo do Dourado	4 293
Estiva	10 920
Estrela Dalva	2 497
Estrela do Indaiá	3 651
Estrela do Sul	7 136
Eugenópolis	10 291
Ewbank da Câmara	3 567
Extrema	24 886
Fama	2 219
Faria Lemos	3 612
Felício dos Santos	5 685
Felisburgo	6 687
Felixlândia	13 618
Fernandes Tourinho	2 612
Ferros	11 387
Fervedouro	10 261

Tabela 1.1.17 - População recenseada e estimada, segundo os municípios - Minas Gerais - 2007

(continuação)

Municípios	População recenseada e estimada (1)
Florestal	5 928
Formiga	64 585
Formoso	6 612
Fortaleza de Minas	3 837
Fortuna de Minas	2 454
Francisco Badaró	10 269
Francisco Dumont	4 759
Francisco Sá	24 838
Franciscópolis	5 664
Frei Gaspar	6 343
Frei Inocêncio	8 873
Frei Lagonegro	3 342
Fronteira	13 983
Fronteira dos Vales	4 835
Fruta de Leite	6 327
Frutal	51 766
Funilândia	3 639
Galiléia	7 302
Gameleiras	5 226
Glaucilândia	2 932
Goiabeira	3 052
Goianá	3 643
Gonçalves	4 270
Gonzaga	5 620
Gouveia	11 569
Governador Valadares (2)	260 396
Grão Mogol	14 594
Grupiara	1 412
Guanhães	29 286
Guapé	13 152
Guaraciaba	10 428
Guaraciama	4 554
Guaranésia	18 147
Guarani	9 487
Guarará	4 017
Guarda-Mor	6 577
Guaxupé	47 894
Guidoval	7 321
Guimarânia	6 946
Guiricema	8 906
Gurinhatã	6 194
Heliodora	6 005
Iapu	10 851
Ibertioga	5 057
Ibiá	22 069
Ibiaí	7 571
Ibiracatu	5 898
Ibiraci	11 023
Ibirité	148 535
Ibitiúra de Minas	3 382
Ibituruna	2 825
Icaraí de Minas	10 331
Igarapé	31 135
Igaratinga	8 477
Iguatama	7 632
Ijaci	5 687
Ilicínea	11 265
Imbé de Minas	6 324

Tabela 1.1.17 - População recenseada e estimada, segundo os municípios - Minas Gerais - 2007

(continuação)

Municípios	População recenseada e estimada (1)
Inconfidentes	7 253
Indaiabira	7 482
Indianópolis	6 244
Ingaí	2 496
Inhapim	24 289
Inhaúma	5 347
Inimutaba	6 420
Ipaba	14 844
Ipanema	17 128
Ipatinga (2)	238 397
Ipiaçu	4 191
Ipuiúna	9 183
Iraí de Minas	6 295
Itabira	105 159
Itabirinha	10 338
Itabirito	41 522
Itacambira	5 018
Itacarambi	17 626
Itaguara	12 292
Itaipé	11 497
Itajubá	86 673
Itamarandiba	31 883
Itamarati de Minas	4 035
Itambacuri	22 635
Itambé do Mato Dentro	2 434
Itamogi	10 828
Itamonte	13 756
Itanhandu	14 395
Itanhomi	11 880
Itaobim	20 986
Itapagipe	14 019
Itapecerica	20 653
Itapeva	7 722
Itatiaiuçu	8 953
Itaú de Minas	14 551
Itaúna	81 833
Itaverava	5 724
Itinga	14 587
Itueta	5 830
Ituiutaba	92 727
Itumirim	6 439
Iturama	31 495
Itutinga	4 051
Jaboticatubas	15 496
Jacinto	12 422
Jacuí	7 225
Jacutinga	20 389
Jaguaraçu	2 782
Jaíba	30 386
Jampruca	4 926
Janaúba	65 387
Januária	64 985
Japaraíba	3 688
Japonvar	8 232
Jeceaba	5 892
Jenipapo de Minas	6 905
Jequeri	12 965
Jequitaí	8 029

Tabela 1.1.17 - População recenseada e estimada, segundo os municípios - Minas Gerais - 2007

(continuação)

Municípios	População recenseada e estimada (1)
Jequitibá	5 491
Jequitinhonha	23 982
Jesuânia	4 821
Joaíma	14 881
Joanésia	5 628
João Monlevade	71 658
João Pinheiro	43 229
Joaquim Felício	3 937
Jordânia	10 751
José Gonçalves de Minas	4 547
José Raydan	4 146
Josenópolis	4 440
Juatuba	19 528
Juiz de Fora (2)	513 348
Juramento	3 960
Juruaia	8 260
Juvenília	6 050
Ladainha	16 479
Lagamar	7 636
Lagoa da Prata	44 159
Lagoa dos Patos	4 448
Lagoa Dourada	11 792
Lagoa Formosa	16 521
Lagoa Grande	8 660
Lagoa Santa	44 922
Lajinha	17 580
Lambari	18 547
Lamim	3 546
Laranjal	6 310
Lassance	6 458
Lavras	87 421
Leandro Ferreira	2 955
Leme do Prado	4 930
Leopoldina	49 915
Liberdade	5 333
Lima Duarte	15 909
Limeira do Oeste	6 492
Lontra	7 979
Luisburgo	6 276
Luislândia	6 432
Luminárias	5 374
Luz	17 173
Machacalis	6 855
Machado	37 567
Madre de Deus de Minas	4 951
Malacacheta	17 917
Mamonas	6 247
Manga	20 903
Manhuaçu	74 297
Manhumirim	20 209
Mantena	26 721
Mar de Espanha	11 139
Maravilhas	6 840
Maria da Fé	14 249
Mariana	51 693
Marilac	4 285
Mário Campos	11 421
Maripá de Minas	2 827

Tabela 1.1.17 - População recenseada e estimada, segundo os municípios - Minas Gerais - 2007

(continuação)

Municípios	População recenseada e estimada (1)
Marliéria	3 743
Marmelópolis	3 100
Martinho Campos	12 165
Martins Soares	6 338
Mata Verde	7 458
Materlândia	4 662
Mateus Leme	25 627
Mathias Lobato	3 457
Matias Barbosa	13 205
Matias Cardoso	10 270
Matipó	16 430
Mato Verde	12 664
Matozinhos	33 317
Matutina	3 700
Medeiros	3 238
Medina	20 667
Mendes Pimentel	6 431
Mercês	10 452
Mesquita	6 493
Minas Novas	30 578
Minduri	3 603
Mirabela	12 769
Miradouro	10 197
Miraí	12 949
Miravânia	4 708
Moeda	4 506
Moema	6 754
Monjolos	2 303
Monsenhor Paulo	7 391
Montalvânia	15 961
Monte Alegre de Minas	18 348
Monte Azul	22 437
Monte Belo	12 573
Monte Carmelo	44 367
Monte Formoso	4 709
Monte Santo de Minas	20 133
Monte Sião	19 228
Montes Claros (2)	352 384
Montezuma	7 259
Morada Nova de Minas	8 297
Morro da Garça	2 887
Morro do Pilar	3 474
Munhoz	6 298
Muriaé	95 548
Mutum	26 331
Muzambinho	19 925
Nacip Raydan	2 957
Nanuque	40 307
Naque	5 885
Natalândia	3 271
Natércia	4 623
Nazareno	7 716
Nepomuceno	24 430
Ninheira	10 414
Nova Belém	3 629
Nova Era	17 932
Nova Lima	72 207
Nova Módica	3 878

Tabela 1.1.17 - População recenseada e estimada, segundo os municípios - Minas Gerais - 2007

(continuação)

Municípios	População recenseada e estimada (1)
Nova Ponte	11 586
Nova Porteirinha	7 358
Nova Resende	14 145
Nova Serrana	60 195
Nova União	5 461
Novo Cruzeiro	30 331
Novo Oriente de Minas	10 327
Novorizonte	4 899
Olaria	2 380
Olhos-d'Água	4 991
Olímpio Noronha	2 505
Oliveira	37 805
Oliveira Fortes	1 939
Onça de Pitangui	3 019
Oratórios	4 385
Orizânia	6 771
Ouro Branco	33 548
Ouro Fino	31 154
Ouro Preto	67 048
Ouro Verde de Minas	6 832
Padre Carvalho	5 828
Padre Paraíso	18 120
Pai Pedro	5 979
Paineiras	4 594
Pains	8 122
Paiva	1 630
Palma	6 118
Palmópolis	7 041
Papagaios	14 410
Pará de Minas	79 852
Paracatu	79 739
Paraguaçu	19 603
Paraisópolis	18 088
Paraopeba	22 204
Passa Quatro	15 285
Passa Tempo	8 494
Passabém	1 801
Passa-Vinte	2 082
Passos	102 765
Patis	5 346
Patos de Minas	133 054
Patrocínio	81 589
Patrocínio do Muriaé	5 319
Paula Cândido	9 086
Paulistas	4 893
Pavão	8 868
Peçanha	17 157
Pedra Azul	24 851
Pedra Bonita	6 474
Pedra do Anta	3 672
Pedra do Indaiá	3 921
Pedra Dourada	2 100
Pedralva	11 184
Pedras de Maria da Cruz	10 976
Pedrinópolis	3 448
Pedro Leopoldo	56 518
Pedro Teixeira	1 658
Pequeri	2 997

Tabela 1.1.17 - População recenseada e estimada, segundo os municípios - Minas Gerais - 2007

(continuação)

Municípios	População recenseada e estimada (1)
Pequi	4 232
Perdigão	7 310
Perdizes	13 924
Perdões	19 407
Periquito	7 030
Pescador	4 056
Piau	2 973
Piedade de Caratinga	6 444
Piedade de Ponte Nova	4 113
Piedade do Rio Grande	4 781
Piedade dos Gerais	4 542
Pimenta	8 164
Pingo-d'Água	4 016
Pintópolis	7 727
Piracema	6 554
Pirajuba	3 694
Piranga	17 208
Piranguçu	5 113
Piranguinho	7 849
Pirapetinga	10 240
Pirapora	51 636
Piraúba	10 686
Pitangui	24 618
Piumhi	30 984
Planura	10 289
Poço Fundo	15 350
Poços de Caldas	144 386
Pocrane	8 769
Pompéu	28 393
Ponte Nova	55 687
Ponto Chique	4 046
Ponto dos Volantes	10 976
Porteirinha	36 864
Porto Firme	10 404
Poté	14 749
Pouso Alegre	120 467
Pouso Alto	6 359
Prados	8 168
Prata	25 511
Pratápolis	8 653
Pratinha	3 236
Presidente Bernardes	5 699
Presidente Juscelino	4 257
Presidente Kubitschek	2 978
Presidente Olegário	18 256
Prudente de Morais	8 874
Quartel Geral	3 200
Queluzito	1 826
Raposos	14 874
Raul Soares	23 901
Recreio	10 194
Reduto	6 344
Resende Costa	10 537
Resplendor	17 024
Ressaquinha	4 574
Riachinho	8 126
Riacho dos Machados	9 392
Ribeirão das Neves (2)	329 112

Tabela 1.1.17 - População recenseada e estimada, segundo os municípios - Minas Gerais - 2007

(continuação)

Municípios	População recenseada e estimada (1)
Ribeirão Vermelho	3 773
Rio Acima	8 257
Rio Casca	14 496
Rio do Prado	4 489
Rio Doce	2 520
Rio Espera	6 594
Rio Manso	5 007
Rio Novo	8 910
Rio Paranaíba	10 809
Rio Pardo de Minas	28 633
Rio Piracicaba	14 319
Rio Pomba	16 709
Rio Preto	5 388
Rio Vermelho	14 856
Ritápolis	5 068
Rochedo de Minas	2 036
Rodeiro	6 178
Romaria	3 561
Rosário da Limeira	4 151
Rubelita	8 299
Rubim	9 561
Sabará	120 770
Sabinópolis	15 889
Sacramento	22 159
Salinas	37 370
Salto da Divisa	6 896
Santa Bárbara	26 185
Santa Bárbara do Leste	7 451
Santa Bárbara do Monte Verde	2 796
Santa Bárbara do Tugúrio	4 504
Santa Cruz de Minas	7 347
Santa Cruz de Salinas	5 192
Santa Cruz do Escalvado	5 193
Santa Efigênia de Minas	4 519
Santa Fé de Minas	4 034
Santa Helena de Minas	5 892
Santa Juliana	10 582
Santa Luzia (2)	222 507
Santa Margarida	14 205
Santa Maria de Itabira	10 445
Santa Maria do Salto	5 724
Santa Maria do Suaçuí	14 427
Santa Rita de Caldas	9 078
Santa Rita de Ibitipoca	3 747
Santa Rita de Jacutinga	5 588
Santa Rita de Minas	5 787
Santa Rita do Itueto	5 652
Santa Rita do Sapucaí	34 246
Santa Rosa da Serra	3 261
Santa Vitória	15 492
Santana da Vargem	7 092
Santana de Cataguases	3 603
Santana de Pirapama	8 549
Santana do Deserto	3 833
Santana do Garambéu	2 104
Santana do Jacaré	4 532
Santana do Manhuaçu	8 185
Santana do Paraíso	22 765

Tabela 1.1.17 - População recenseada e estimada, segundo os municípios - Minas Gerais - 2007

(continuação)

Municípios	População recenseada e estimada (1)
Santana do Riacho	4 159
Santana dos Montes	3 989
Santo Antônio do Amparo	17 255
Santo Antônio do Aventureiro	3 489
Santo Antônio do Grama	4 241
Santo Antônio do Itambé	4 517
Santo Antônio do Jacinto	11 294
Santo Antônio do Monte	24 746
Santo Antônio do Retiro	6 817
Santo Antônio do Rio Abaixo	1 753
Santo Hipólito	3 541
Santos Dumont	45 922
São Bento Abade	4 400
São Brás do Suaçuí	3 488
São Domingos das Dores	5 232
São Domingos do Prata	17 349
São Félix de Minas	3 387
São Francisco	52 985
São Francisco de Paula	6 246
São Francisco de Sales	5 167
São Francisco do Glória	5 539
São Geraldo	9 171
São Geraldo da Piedade	4 768
São Geraldo do Baixio	3 253
São Gonçalo do Abaeté	6 156
São Gonçalo do Pará	10 308
São Gonçalo do Rio Abaixo	9 233
São Gonçalo do Rio Preto	3 124
São Gonçalo do Sapucaí	22 751
São Gotardo	30 757
São João Batista do Glória	6 828
São João da Lagoa	4 729
São João da Mata	2 858
São João da Ponte	26 091
São João das Missões	10 769
São João del Rei	81 918
São João do Manhuaçu	9 394
São João do Manteninha	4 855
São João do Oriente	7 988
São João do Pacuí	4 003
São João do Paraíso	21 839
São João Evangelista	15 686
São João Nepomuceno	25 011
São Joaquim de Bicas	22 214
São José da Barra	6 701
São José da Lapa	17 900
São José da Safira	3 929
São José da Varginha	3 797
São José do Alegre	3 908
São José do Divino	3 776
São José do Goiabal	5 646
São José do Jacuri	6 958
São José do Mantimento	2 472
São Lourenço	40 441
São Miguel do Anta	6 820
São Pedro da União	5 291
São Pedro do Suaçuí	5 801
São Pedro dos Ferros	8 880

Tabela 1.1.17 - População recenseada e estimada, segundo os municípios - Minas Gerais - 2007

(continuação)

Municípios	População recenseada e estimada (1)
São Romão	9 080
São Roque de Minas	6 141
São Sebastião da Bela Vista	4 884
São Sebastião da Vargem Alegre	2 743
São Sebastião do Anta	5 388
São Sebastião do Maranhão	11 686
São Sebastião do Oeste	5 336
São Sebastião do Paraíso	61 838
São Sebastião do Rio Preto	1 700
São Sebastião do Rio Verde	2 170
São Thomé das Letras	6 617
São Tiago	10 289
São Tomás de Aquino	6 934
São Vicente de Minas	6 283
Sapucaí-Mirim	5 772
Sardoá	5 196
Sarzedo	23 282
Sem-Peixe	2 950
Senador Amaral	5 051
Senador Cortes	2 011
Senador Firmino	7 019
Senador José Bento	1 908
Senador Modestino Gonçalves	4 988
Senhora de Oliveira	5 675
Senhora do Porto	3 517
Senhora dos Remédios	10 201
Sericita	7 083
Seritinga	1 755
Serra Azul de Minas	4 307
Serra da Saudade	863
Serra do Salitre	10 224
Serra dos Aimorés	8 345
Serrania	7 370
Serranópolis de Minas	4 515
Serranos	2 063
Serro	20 862
Sete Lagoas (2)	217 506
Setubinha	10 834
Silveirânia	2 125
Silvianópolis	6 018
Simão Pereira	2 503
Simonésia	17 257
Sobrália	5 990
Soledade de Minas	5 518
Tabuleiro	4 061
Taiobeiras	29 732
Taparuba	3 243
Tapira	3 575
Tapiraí	1 841

Tabela 1.1.17 - População recenseada e estimada, segundo os municípios - Minas Gerais - 2007

(conclusão)

Municípios	População recenseada e estimada (1)
Taquaraçu de Minas	3 757
Tarumirim	14 185
Teixeiras	11 665
Teófilo Otoni	126 895
Timóteo	76 092
Tiradentes	6 547
Tiros	7 416
Tocantins	15 704
Tocos do Moji	3 926
Toledo	5 720
Tombos	9 194
Três Corações	71 737
Três Marias	26 431
Três Pontas	52 121
Tumiritinga	5 964
Tupaciguara	23 076
Turmalina	17 219
Turvolândia	4 737
Ubá	94 228
Ubaí	11 834
Ubaporanga	12 060
Uberaba (2)	287 760
Uberlândia (2)	608 369
Umburatiba	2 776
Unaí	74 495
União de Minas	4 593
Uruana de Minas	2 777
Urucânia	10 203
Urucuia	11 376
Vargem Alegre	6 594
Vargem Bonita	2 098
Vargem Grande do Rio Pardo	4 703
Varginha	116 093
Varjão de Minas	5 993
Várzea da Palma	34 448
Varzelândia	19 137
Vazante	19 300
Verdelândia	8 029
Veredinha	5 732
Veríssimo	3 667
Vermelho Novo	4 551
Vespasiano	94 191
Viçosa	70 404
Vieiras	3 808
Virgem da Lapa	14 103
Virgínia	8 351
Virginópolis	10 891
Virgolândia	5 724
Visconde do Rio Branco	35 346
Volta Grande	5 166
Wenceslau Braz	2 509

Fonte: IBGE, Contagem da População 2007.

(1) Inclusive a população estimada nos domicílios fechados. (2) População estimada.

Tabela 1.1.18 - População recenseada e estimada, segundo os municípios - Espírito Santo - 2007

(continua)

Municípios	População recenseada e estimada (1)
**Espírito Santo**	**3 351 669**
Afonso Cláudio	30 773
Água Doce do Norte	11 934
Águia Branca	9 281
Alegre	30 473
Alfredo Chaves	13 983
Alto Rio Novo	6 198
Anchieta	19 459
Apiacá	7 617
Aracruz	73 358
Atilio Vivacqua	8 878
Baixo Guandu	28 637
Barra de São Francisco	39 627
Boa Esperança	12 912
Bom Jesus do Norte	9 318
Brejetuba	10 949
Cachoeiro de Itapemirim (2)	195 288
Cariacica (2)	356 536
Castelo	32 250
Colatina	106 637
Conceição da Barra	26 230
Conceição do Castelo	11 326
Divino de São Lourenço	4 837
Domingos Martins	31 175
Dores do Rio Preto	6 106
Ecoporanga	23 296
Fundão	15 209
Governador Lindenberg	9 890
Guaçuí	25 761
Guarapari	98 073
Ibatiba	19 649
Ibiraçu	10 312
Ibitirama	8 994
Iconha	11 496
Irupi	10 369
Itaguaçu	13 881
Itapemirim	30 833
Itarana	10 569
Iúna	25 533

Tabela 1.1.18 - População recenseada e estimada, segundo os municípios - Espírito Santo - 2007

(conclusão)

Municípios	População recenseada e estimada (1)
Jaguaré	21 949
Jerônimo Monteiro	10 701
João Neiva	14 403
Laranja da Terra	10 802
Linhares	124 564
Mantenópolis	11 463
Marataízes	31 221
Marechal Floriano	12 699
Marilândia	10 226
Mimoso do Sul	26 208
Montanha	17 998
Mucurici	5 755
Muniz Freire	18 196
Muqui	13 841
Nova Venécia	44 380
Pancas	18 465
Pedro Canário	23 204
Pinheiros	22 663
Piúma	16 249
Ponto Belo	6 831
Presidente Kennedy	10 307
Rio Bananal	16 587
Rio Novo do Sul	11 111
Santa Leopoldina	12 349
Santa Maria de Jetibá	31 845
Santa Teresa	20 179
São Domingos do Norte	7 840
São Gabriel da Palha	28 878
São José do Calçado	10 570
São Mateus	96 390
São Roque do Canaã	10 439
Serra (2)	385 370
Sooretama	21 867
Vargem Alta	17 862
Venda Nova do Imigrante	18 610
Viana	57 539
Vila Pavão	8 705
Vila Valério	13 646
Vila Velha (2)	398 068
Vitória (2)	314 042

Fonte: IBGE, Contagem da População 2007.
(1) Inclusive a população estimada nos domicílios fechados. (2) População estimada.

Tabela 1.1.19 - População recenseada e estimada, segundo os municípios - Rio de Janeiro - 2007

(continua)

Municípios	População recenseada e estimada (1)
**Rio de Janeiro**	**15 420 375**
Angra dos Reis	148 476
Aperibé	8 820
Araruama	98 268
Areal	11 009
Armação dos Búzios	24 560
Arraial do Cabo	25 248
Barra do Piraí	96 282
Barra Mansa (2)	175 315
Belford Roxo (2)	480 555
Bom Jardim	24 626
Bom Jesus do Itabapoana	33 888
Cabo Frio	162 229
Cachoeiras de Macacu	53 037
Cambuci	14 368
Campos dos Goytacazes (2)	426 154
Cantagalo	19 799
Carapebus	10 677
Cardoso Moreira	12 206
Carmo	16 690
Casimiro de Abreu	27 086
Comendador Levy Gasparian	8 322
Conceição de Macabu	19 479
Cordeiro	18 984
Duas Barras	10 438
Duque de Caxias (2)	842 686
Engenheiro Paulo de Frontin	12 544
Guapimirim	44 692
Iguaba Grande	19 716
Itaboraí (2)	215 792
Itaguaí	95 356
Italva	13 645
Itaocara	22 069
Itaperuna	92 852
Itatiaia	31 185
Japeri	93 197
Laje do Muriaé	7 769
Macaé	169 513
Macuco	5 246
Magé (2)	232 171
Mangaratiba	29 253
Maricá	105 294
Mendes	17 242
Mesquita (2)	182 495
Miguel Pereira	24 585
Miracema	26 231
Natividade	14 930
Nilópolis	153 581

Tabela 1.1.19 - População recenseada e estimada, segundo os municípios - Rio de Janeiro - 2007

(conclusão)

Municípios	População recenseada e estimada (1)
Niterói (2)	474 002
Nova Friburgo (2)	177 376
Nova Iguaçu (2)	830 672
Paracambi	42 423
Paraíba do Sul	39 257
Parati	32 838
Paty do Alferes	25 132
Petrópolis (2)	306 645
Pinheiral	20 885
Piraí	24 170
Porciúncula	17 178
Porto Real	14 503
Quatis	12 031
Queimados	130 275
Quissamã	17 376
Resende	118 547
Rio Bonito	51 942
Rio Claro	17 216
Rio das Flores	8 192
Rio das Ostras	74 750
Rio de Janeiro (2)	6 093 472
Santa Maria Madalena	10 409
Santo Antônio de Pádua	40 145
São Fidélis	37 477
São Francisco de Itabapoana	44 475
São Gonçalo (2)	960 631
São João da Barra	28 889
São João de Meriti (2)	464 282
São José de Ubá	6 829
São José do Vale do Rio Preto	19 439
São Pedro da Aldeia	75 869
São Sebastião do Alto	8 616
Sapucaia	16 858
Saquarema	62 174
Seropédica	72 466
Silva Jardim	21 362
Sumidouro	14 562
Tanguá	28 322
Teresópolis	150 268
Trajano de Morais	9 706
Três Rios	72 848
Valença	70 850
Varre-Sai	8 308
Vassouras	32 495
Volta Redonda (2)	255 653

Fonte: IBGE, Contagem da População 2007.

(1) Inclusive a população estimada nos domicílios fechados. (2) População estimada.

Tabela 1.1.20 - População recenseada e estimada, segundo os municípios - São Paulo - 2007

(continua)

Municípios	População recenseada e estimada (1)
**São Paulo**	**39 827 570**
Adamantina	33 289
Adolfo	3 609
Aguaí	30 181
Águas da Prata	7 367
Águas de Lindóia	15 867
Águas de Santa Bárbara	5 360
Águas de São Pedro	2 340
Agudos	34 221
Alambari	4 070
Alfredo Marcondes	3 851
Altair	3 272
Altinópolis	15 139
Alto Alegre	4 157
Alumínio	15 678
Álvares Florence	3 902
Álvares Machado	22 859
Álvaro de Carvalho	4 610
Alvinlândia	2 787
Americana (2)	199 094
Américo Brasiliense	31 005
Américo de Campos	5 379
Amparo	62 692
Analândia	4 166
Andradina	54 753
Angatuba	21 523
Anhembi	5 271
Anhumas	3 695
Aparecida	35 903
Aparecida d'Oeste	4 577
Apiaí	25 463
Araçariguama	12 291
Araçatuba (2)	178 839
Araçoiaba da Serra	24 022
Aramina	5 019
Arandu	6 013
Arapeí	2 527
Araraquara (2)	195 815
Araras	108 689
Arco-Íris	2 003
Arealva	7 504
Areias	3 571
Areiópolis	10 630
Ariranha	8 255
Artur Nogueira	39 457
Arujá	72 713
Aspásia	1 790
Assis	92 965
Atibaia	119 166
Auriflama	13 760

Tabela 1.1.20 - População recenseada e estimada, segundo os municípios - São Paulo - 2007

(continuação)

Municípios	População recenseada e estimada (1)
Avaí	4 877
Avanhandava	10 875
Avaré	80 026
Bady Bassitt	13 039
Balbinos	3 619
Bálsamo	7 767
Bananal	10 233
Barão de Antonina	2 751
Barbosa	6 454
Bariri	30 995
Barra Bonita	35 090
Barra do Chapéu	5 077
Barra do Turvo	7 620
Barretos	107 988
Barrinha	25 642
Barueri (2)	252 748
Bastos	20 613
Batatais	53 525
Bauru (2)	347 601
Bebedouro	74 865
Bento de Abreu	2 740
Bernardino de Campos	10 487
Bertioga	39 091
Bilac	6 905
Birigui	103 394
Biritiba-Mirim	27 483
Boa Esperança do Sul	13 208
Bocaina	10 299
Bofete	8 565
Boituva	40 783
Bom Jesus dos Perdões	16 211
Bom Sucesso de Itararé	3 658
Borá	804
Boracéia	4 128
Borborema	13 752
Borebi	2 172
Botucatu	120 800
Bragança Paulista	136 286
Braúna	4 728
Brejo Alegre	2 432
Brodowski	19 018
Brotas	20 996
Buri	17 539
Buritama	14 735
Buritizal	3 872
Cabrália Paulista	4 340
Cabreúva	38 898
Caçapava	80 458
Cachoeira Paulista	31 674
Caconde	18 552

Tabela 1.1.20 - População recenseada e estimada, segundo os municípios - São Paulo - 2007

(continuação)

Municípios	População recenseada e estimada (1)
Cafelândia	16 073
Caiabu	4 011
Caieiras	81 163
Caiuá	4 953
Cajamar	58 403
Cajati	28 285
Cajobi	9 519
Cajuru	22 695
Campina do Monte Alegre	5 320
Campinas (2)	1 039 297
Campo Limpo Paulista	69 810
Campos do Jordão	44 688
Campos Novos Paulista	4 651
Cananéia	12 039
Canas	4 318
Cândido Mota	29 572
Cândido Rodrigues	2 655
Canitar	4 265
Capão Bonito	45 275
Capela do Alto	15 950
Capivari	43 779
Caraguatatuba	88 815
Carapicuíba (2)	379 566
Cardoso	11 324
Casa Branca	27 081
Cássia dos Coqueiros	2 706
Castilho	15 410
Catanduva	109 362
Catiguá	6 870
Cedral	7 607
Cerqueira César	16 276
Cerquilho	34 769
Cesário Lange	14 005
Charqueada	14 356
Chavantes	12 226
Clementina	6 081
Colina	16 989
Colômbia	6 073
Conchal	23 352
Conchas	15 473
Cordeirópolis	19 309
Coroados	4 881
Coronel Macedo	5 277
Corumbataí	3 935
Cosmópolis	53 561
Cosmorama	6 951
Cotia (2)	172 823
Cravinhos	29 377
Cristais Paulista	7 005
Cruzália	2 368

Tabela 1.1.20 - População recenseada e estimada, segundo os municípios - São Paulo - 2007

(continuação)

Municípios	População recenseada e estimada (1)
Cruzeiro	76 098
Cubatão	120 271
Cunha	22 951
Descalvado	29 533
Diadema (2)	386 779
Dirce Reis	1 582
Divinolândia	11 246
Dobrada	7 664
Dois Córregos	24 384
Dolcinópolis	2 181
Dourado	8 751
Dracena	42 107
Duartina	12 381
Dumont	7 557
Echaporã	6 247
Eldorado	14 038
Elias Fausto	14 521
Elisiário	3 024
Embaúba	2 391
Embu (2)	237 318
Embu-Guaçu	59 083
Emilianópolis	3 021
Engenheiro Coelho	12 729
Espírito Santo do Pinhal	40 684
Espírito Santo do Turvo	4 103
Estiva Gerbi	9 185
Estrela do Norte	2 454
Estrela d'Oeste	8 590
Euclides da Cunha Paulista	9 923
Fartura	14 601
Fernando Prestes	5 212
Fernandópolis	61 392
Fernão	1 457
Ferraz de Vasconcelos (2)	168 897
Flora Rica	2 019
Floreal	2 907
Flórida Paulista	12 660
Florínia	2 860
Franca (2)	319 094
Francisco Morato	146 634
Franco da Rocha	121 451
Gabriel Monteiro	2 638
Gália	6 812
Garça	42 218
Gastão Vidigal	3 863
Gavião Peixoto	4 103
General Salgado	10 626
Getulina	10 515
Glicério	4 398
Guaiçara	10 357

Tabela 1.1.20 - População recenseada e estimada, segundo os municípios - São Paulo - 2007

(continuação)

Municípios	População recenseada e estimada (1)
Guaimbê	4 752
Guaíra	36 544
Guapiaçu	16 392
Guapiara	20 056
Guará	18 611
Guaraçaí	8 505
Guaraci	9 045
Guarani d'Oeste	1 963
Guarantã	6 410
Guararapes	28 662
Guararema	24 854
Guaratinguetá	107 895
Guareí	13 202
Guariba	32 664
Guarujá (2)	296 150
Guarulhos (2)	1 236 192
Guatapará	6 217
Guzolândia	4 616
Herculândia	8 573
Holambra	9 111
Hortolândia (2)	190 781
Iacanga	9 074
Iacri	6 677
Iaras	4 777
Ibaté	28 040
Ibirá	10 379
Ibirarema	6 617
Ibitinga	49 951
Ibiúna	64 832
Icém	6 429
Iepê	7 487
Igaraçu do Tietê	23 085
Igarapava	26 862
Igaratá	8 537
Iguape	28 977
Ilha Comprida	8 875
Ilha Solteira	24 181
Ilhabela	23 886
Indaiatuba (2)	173 508
Indiana	4 682
Indiaporã	3 880
Inúbia Paulista	3 595
Ipaussu	12 964
Iperó	24 239
Ipeúna	5 164
Ipiguá	3 925
Iporanga	4 603
Ipuã	14 344
Iracemápolis	18 026
Irapuã	6 710

Tabela 1.1.20 - População recenseada e estimada, segundo os municípios - São Paulo - 2007

(continuação)

Municípios	População recenseada e estimada (1)
Irapuru	7 556
Itaberá	17 576
Itaí	22 617
Itajobi	14 182
Itaju	2 624
Itanhaém	80 778
Itaóca	3 087
Itapecerica da Serra	148 728
Itapetininga	138 450
Itapeva	85 537
Itapevi (2)	193 686
Itapira	68 187
Itapirapuã Paulista	3 723
Itápolis	38 633
Itaporanga	14 284
Itapuí	11 605
Itapura	3 812
Itaquaquecetuba (2)	334 914
Itararé	48 732
Itariri	15 115
Itatiba	91 479
Itatinga	17 570
Itirapina	13 889
Itirapuã	5 614
Itobi	7 444
Itu	147 157
Itupeva	36 766
Ituverava	38 539
Jaborandi	6 462
Jaboticabal	69 624
Jacareí (2)	207 028
Jaci	5 008
Jacupiranga	16 112
Jaguariúna	36 804
Jales	47 649
Jambeiro	4 968
Jandira	103 531
Jardinópolis	34 611
Jarinu	20 606
Jaú	125 469
Jeriquara	3 153
Joanópolis	10 671
João Ramalho	4 092
José Bonifácio	30 639
Júlio Mesquita	4 318
Jumirim	2 205
Jundiaí (2)	342 983
Junqueirópolis	18 628
Juquiá	19 352
Juquitiba	27 777

Tabela 1.1.20 - População recenseada e estimada, segundo os municípios - São Paulo - 2007

(continuação)

Municípios	População recenseada e estimada (1)
Lagoinha	4 798
Laranjal Paulista	24 454
Lavínia	7 984
Lavrinhas	6 543
Leme	84 406
Lençóis Paulista	59 366
Limeira (2)	272 734
Lindóia	5 657
Lins	69 279
Lorena	79 317
Lourdes	1 974
Louveira	29 760
Lucélia	19 212
Lucianópolis	2 299
Luís Antônio	10 272
Luiziânia	4 763
Lupércio	4 238
Lutécia	2 794
Macatuba	16 173
Macaubal	7 396
Macedônia	3 411
Magda	3 154
Mairinque	41 508
Mairiporã	71 754
Manduri	8 651
Marabá Paulista	5 193
Maracaí	13 163
Marapoama	2 556
Mariápolis	3 786
Marília (2)	218 113
Marinópolis	2 114
Martinópolis	23 983
Matão	74 407
Mauá (2)	402 643
Mendonça	3 980
Meridiano	3 857
Mesópolis	1 768
Miguelópolis	19 972
Mineiros do Tietê	11 760
Mira Estrela	2 576
Miracatu	22 796
Mirandópolis	25 849
Mirante do Paranapanema	17 128
Mirassol	51 660
Mirassolândia	4 099
Mococa	66 086
Mogi das Cruzes (2)	362 991
Mogi Guaçu	131 870
Moji Mirim	84 176
Mombuca	3 280

Tabela 1.1.20 - População recenseada e estimada, segundo os municípios - São Paulo - 2007

(continuação)

Municípios	População recenseada e estimada (1)
Monções	2 054
Mongaguá	40 423
Monte Alegre do Sul	6 957
Monte Alto	44 085
Monte Aprazível	19 745
Monte Azul Paulista	19 187
Monte Castelo	4 014
Monte Mor	42 824
Monteiro Lobato	3 994
Morro Agudo	25 390
Morungaba	12 007
Motuca	4 340
Murutinga do Sul	4 091
Nantes	2 483
Narandiba	3 941
Natividade da Serra	7 275
Nazaré Paulista	14 613
Neves Paulista	8 825
Nhandeara	10 334
Nipoã	3 762
Nova Aliança	4 905
Nova Campina	8 474
Nova Canaã Paulista	2 205
Nova Castilho	1 057
Nova Europa	9 047
Nova Granada	17 739
Nova Guataporanga	2 101
Nova Independência	2 480
Nova Luzitânia	3 403
Nova Odessa	45 625
Novais	3 661
Novo Horizonte	34 264
Nuporanga	6 629
Ocauçu	4 180
Óleo	2 714
Olímpia	48 020
Onda Verde	3 736
Oriente	6 079
Orindiúva	4 916
Orlândia	36 149
Osasco (2)	701 012
Oscar Bressane	2 476
Osvaldo Cruz	30 150
Ourinhos	98 868
Ouro Verde	7 668
Ouroeste	7 035
Pacaembu	13 072
Palestina	10 428
Palmares Paulista	10 508
Palmeira d'Oeste	9 634

Tabela 1.1.20 - População recenseada e estimada, segundo os municípios - São Paulo - 2007

(continuação)

Municípios	População recenseada e estimada (1)
Palmital	21 298
Panorama	13 944
Paraguaçu Paulista	42 117
Paraibuna	16 456
Paraíso	5 559
Paranapanema	16 667
Paranapuã	3 614
Parapuã	11 029
Pardinho	5 030
Pariquera-Açu	18 079
Parisi	2 038
Patrocínio Paulista	12 183
Paulicéia	5 506
Paulínia	73 014
Paulistânia	1 824
Paulo de Faria	8 942
Pederneiras	40 270
Pedra Bela	5 835
Pedranópolis	2 734
Pedregulho	15 156
Pedreira	38 152
Pedrinhas Paulista	2 834
Pedro de Toledo	9 692
Penápolis	56 681
Pereira Barreto	24 220
Pereiras	7 347
Peruíbe	54 457
Piacatu	5 093
Piedade	48 430
Pilar do Sul	26 457
Pindamonhangaba	135 682
Pindorama	14 345
Pinhalzinho	11 817
Piquerobi	3 599
Piquete	14 475
Piracaia	22 335
Piracicaba (2)	358 108
Piraju	28 228
Pirajuí	21 035
Pirangi	10 315
Pirapora do Bom Jesus	14 370
Pirapozinho	23 709
Pirassununga	67 787
Piratininga	11 287
Pitangueiras	33 329
Planalto	4 014
Platina	3 167
Poá	104 904
Poloni	4 878
Pompéia	19 091

Tabela 1.1.20 - População recenseada e estimada, segundo os municípios - São Paulo - 2007

(continuação)

Municípios	População recenseada e estimada (1)
Pongaí	3 473
Pontal	35 560
Pontalinda	3 906
Pontes Gestal	2 487
Populina	4 201
Porangaba	8 069
Porto Feliz	46 054
Porto Ferreira	48 760
Potim	18 143
Potirendaba	14 327
Pracinha	2 667
Pradópolis	15 148
Praia Grande (2)	233 806
Pratânia	4 309
Presidente Alves	4 346
Presidente Bernardes	14 788
Presidente Epitácio	39 403
Presidente Prudente (2)	202 789
Presidente Venceslau	37 155
Promissão	34 786
Quadra	2 679
Quatá	11 971
Queiroz	2 603
Queluz	10 323
Quintana	5 654
Rafard	8 151
Rancharia	28 303
Redenção da Serra	4 081
Regente Feijó	17 070
Reginópolis	6 993
Registro	53 369
Restinga	6 350
Ribeira	3 444
Ribeirão Bonito	11 383
Ribeirão Branco	18 879
Ribeirão Corrente	4 014
Ribeirão do Sul	4 497
Ribeirão dos Índios	2 231
Ribeirão Grande	6 928
Ribeirão Pires	107 046
Ribeirão Preto (2)	547 417
Rifaina	3 587
Rincão	10 425
Rinópolis	9 393
Rio Claro (2)	185 421
Rio das Pedras	26 344
Rio Grande da Serra	39 270
Riolândia	9 713
Riversul	6 545
Rosana	19 943

Tabela 1.1.20 - População recenseada e estimada, segundo os municípios - São Paulo - 2007

(continuação)

Municípios	População recenseada e estimada (1)
Roseira	9 016
Rubiácea	2 501
Rubinéia	2 546
Sabino	5 150
Sagres	2 307
Sales	5 025
Sales Oliveira	8 187
Salesópolis	15 157
Salmourão	4 551
Saltinho	6 586
Salto	102 405
Salto de Pirapora	37 324
Salto Grande	8 592
Sandovalina	3 217
Santa Adélia	13 861
Santa Albertina	5 034
Santa Bárbara d'Oeste (2)	184 318
Santa Branca	13 282
Santa Clara d'Oeste	2 081
Santa Cruz da Conceição	3 872
Santa Cruz da Esperança	1 707
Santa Cruz das Palmeiras	30 458
Santa Cruz do Rio Pardo	41 655
Santa Ernestina	5 510
Santa Fé do Sul	27 693
Santa Gertrudes	19 044
Santa Isabel	44 817
Santa Lúcia	7 862
Santa Maria da Serra	5 417
Santa Mercedes	2 589
Santa Rita do Passa Quatro	26 456
Santa Rita d'Oeste	2 493
Santa Rosa de Viterbo	22 699
Santa Salete	1 390
Santana da Ponte Pensa	1 654
Santana de Parnaíba	100 236
Santo Anastácio	20 550
Santo André (2)	667 891
Santo Antônio da Alegria	6 020
Santo Antônio de Posse	19 824
Santo Antônio do Aracanguá	7 034
Santo Antônio do Jardim	5 743
Santo Antônio do Pinhal	6 560
Santo Expedito	2 759
Santópolis do Aguapeí	4 008
Santos (2)	418 288
São Bento do Sapucaí	10 515
São Bernardo do Campo (2)	781 390
São Caetano do Sul	144 857
São Carlos (2)	212 956

Tabela 1.1.20 - População recenseada e estimada, segundo os municípios - São Paulo - 2007

(continuação)

Municípios	População recenseada e estimada (1)
São Francisco	2 812
São João da Boa Vista	79 935
São João das Duas Pontes	2 571
São João de Iracema	1 725
São João do Pau d'Alho	2 132
São Joaquim da Barra	43 703
São José da Bela Vista	8 173
São José do Barreiro	4 278
São José do Rio Pardo	51 023
São José do Rio Preto (2)	402 770
São José dos Campos (2)	594 948
São Lourenço da Serra	16 121
São Luís do Paraitinga	10 496
São Manuel	37 797
São Miguel Arcanjo	30 384
São Paulo (2)	10 886 518
São Pedro	29 733
São Pedro do Turvo	7 094
São Roque	65 693
São Sebastião	67 348
São Sebastião da Grama	12 509
São Simão	13 781
São Vicente (2)	323 599
Sarapuí	8 158
Sarutaiá	3 680
Sebastianópolis do Sul	2 871
Serra Azul	9 107
Serra Negra	24 671
Serrana	36 596
Sertãozinho	103 558
Sete Barras	12 975
Severínia	14 713
Silveiras	5 562
Socorro	33 080
Sorocaba (2)	559 157
Sud Mennucci	7 714
Sumaré (2)	228 696
Suzanápolis	3 421
Suzano (2)	268 777
Tabapuã	11 255
Tabatinga	13 965
Taboão da Serra (2)	219 200
Taciba	5 402
Taguaí	9 616
Taiaçu	5 804
Taiúva	5 366
Tambaú	21 913
Tanabi	23 400
Tapiraí	7 953
Tapiratiba	12 246

Tabela 1.1.20 - População recenseada e estimada, segundo os municípios - São Paulo - 2007

(conclusão)

Municípios	População recenseada e estimada (1)
Taquaral	2 827
Taquaritinga	53 232
Taquarituba	22 170
Taquarivaí	5 098
Tarabai	6 108
Tarumã	12 298
Tatuí	101 838
Taubaté (2)	265 514
Tejupá	5 015
Teodoro Sampaio	20 325
Terra Roxa	8 155
Tietê	34 018
Timburi	2 535
Torre de Pedra	2 784
Torrinha	8 918
Trabiju	1 441
Tremembé	38 321
Três Fronteiras	5 031
Tuiuti	5 749
Tupã	62 256
Tupi Paulista	13 712
Turiúba	1 947
Turmalina	2 024
Ubarana	4 552
Ubatuba	75 008
Ubirajara	4 269
Uchoa	9 348
União Paulista	1 436
Urânia	8 727
Uru	1 362
Urupês	11 917
Valentim Gentil	9 408
Valinhos	97 814
Valparaíso	20 827
Vargem	6 883
Vargem Grande do Sul	37 357
Vargem Grande Paulista	40 200
Várzea Paulista	100 411
Vera Cruz	10 020
Vinhedo	57 435
Viradouro	17 043
Vista Alegre do Alto	6 100
Vitória Brasil	1 624
Votorantim	99 901
Votuporanga	77 622
Zacarias	2 229

Fonte: IBGE, Contagem da População 2007.

(1) Inclusive a população estimada nos domicílios fechados e nos domicílios provenientes de setores censitários cujos arquivos foram danificados.

(2) População estimada.

Tabela 1.1.21 - População recenseada e estimada, segundo os municípios - Paraná - 2007

(continua)

Municípios	População recenseada e estimada (1)
**Paraná**	**10 284 503**
Abatiá	7 791
Adrianópolis	6 709
Agudos do Sul	8 192
Almirante Tamandaré	93 055
Altamira do Paraná	4 369
Alto Paraíso	3 252
Alto Paraná	12 936
Alto Piquiri	10 210
Altônia	19 904
Alvorada do Sul	9 014
Amaporã	5 140
Ampére	17 067
Anahy	2 868
Andirá	21 330
Ângulo	2 807
Antonina	17 581
Antônio Olinto	7 477
Apucarana	115 323
Arapongas	96 669
Arapoti	25 645
Arapuã	3 945
Araruna	12 650
Araucária	109 943
Ariranha do Ivaí	2 540
Assaí	16 098
Assis Chateaubriand	32 226
Astorga	24 191
Atalaia	3 627
Balsa Nova	10 639
Bandeirantes	32 290
Barbosa Ferraz	13 655
Barra do Jacaré	2 757
Barracão	9 027
Bela Vista da Caroba	4 136
Bela Vista do Paraíso	14 996
Bituruna	16 142
Boa Esperança	4 706
Boa Esperança do Iguaçu	2 866
Boa Ventura de São Roque	6 744
Boa Vista da Aparecida	7 818
Bocaiúva do Sul	9 533
Bom Jesus do Sul	3 835
Bom Sucesso	6 397
Bom Sucesso do Sul	3 061
Borrazópolis	8 275
Braganey	6 044
Brasilândia do Sul	3 306
Cafeara	2 702
Cafelândia	13 065
Cafezal do Sul	4 271
Califórnia	7 546
Cambará	23 956
Cambé	92 888
Cambira	6 862
Campina da Lagoa	15 983

Tabela 1.1.21 - População recenseada e estimada, segundo os municípios - Paraná - 2007

(continuação)

Municípios	População recenseada e estimada (1)
Campina do Simão	4 180
Campina Grande do Sul	35 396
Campo Bonito	4 426
Campo do Tenente	6 461
Campo Largo	105 492
Campo Magro	22 325
Campo Mourão	82 530
Cândido de Abreu	17 775
Candói	15 412
Cantagalo	12 418
Capanema	18 103
Capitão Leônidas Marques	13 616
Carambeí	16 521
Carlópolis	13 170
Cascavel (2)	285 784
Castro	65 363
Catanduvas	9 578
Centenário do Sul	11 247
Cerro Azul	17 693
Céu Azul	10 914
Chopinzinho	19 224
Cianorte	64 498
Cidade Gaúcha	10 468
Clevelândia	17 599
Colombo (2)	233 916
Colorado	21 049
Congonhinhas	8 552
Conselheiro Mairinck	3 554
Contenda	14 800
Corbélia	15 428
Cornélio Procópio	46 931
Coronel Domingos Soares	7 480
Coronel Vivida	21 571
Corumbataí do Sul	4 262
Cruz Machado	18 329
Cruzeiro do Iguaçu	4 150
Cruzeiro do Oeste	20 182
Cruzeiro do Sul	4 493
Cruzmaltina	3 116
Curitiba (2)	1 797 408
Curiúva	14 338
Diamante do Norte	5 611
Diamante do Sul	3 665
Diamante D'Oeste	4 944
Dois Vizinhos	34 001
Douradina	6 530
Doutor Camargo	5 609
Doutor Ulysses	5 956
Enéas Marques	5 974
Engenheiro Beltrão	13 867
Entre Rios do Oeste	3 842
Esperança Nova	1 887
Espigão Alto do Iguaçu	5 104
Farol	3 394
Faxinal	15 527
Fazenda Rio Grande	75 006
Fênix	4 871

Tabela 1.1.21 - População recenseada e estimada, segundo os municípios - Paraná - 2007

(continuação)

Municípios	População recenseada e estimada (1)
Fernandes Pinheiro	5 688
Figueira	8 380
Flor da Serra do Sul	4 685
Floraí	5 051
Floresta	5 215
Florestópolis	11 571
Flórida	2 448
Formosa do Oeste	7 532
Foz do Iguaçu (2)	311 336
Foz do Jordão	5 832
Francisco Alves	6 342
Francisco Beltrão	72 409
General Carneiro	14 591
Godoy Moreira	3 568
Goioerê	28 941
Goioxim	7 993
Grandes Rios	7 641
Guaíra	28 683
Guairaçá	5 721
Guamiranga	7 548
Guapirama	3 639
Guaporema	2 190
Guaraci	4 993
Guaraniaçu	15 959
Guarapuava	164 567
Guaraqueçaba	7 732
Guaratuba	30 793
Honório Serpa	6 169
Ibaiti	28 050
Ibema	5 927
Ibiporã	45 158
Icaraíma	9 212
Iguaraçu	3 741
Iguatu	2 286
Imbaú	11 191
Imbituva	27 044
Inácio Martins	11 036
Inajá	2 810
Indianópolis	4 138
Ipiranga	13 993
Iporã	15 086
Iracema do Oeste	2 580
Irati	54 151
Iretama	11 174
Itaguajé	4 508
Itaipulândia	8 581
Itambaracá	6 935
Itambé	5 897
Itapejara d'Oeste	10 537
Itaperuçu	22 021
Itaúna do Sul	3 699
Ivaí	12 840
Ivaiporã	31 344
Ivaté	7 792
Ivatuba	2 715
Jaboti	5 019
Jacarezinho	39 327

Tabela 1.1.21 - População recenseada e estimada, segundo os municípios - Paraná - 2007

(continuação)

Municípios	População recenseada e estimada (1)
Jaguapitã	11 782
Jaguariaíva	31 865
Jandaia do Sul	19 534
Janiópolis	7 032
Japira	4 694
Japurá	8 248
Jardim Alegre	14 315
Jardim Olinda	1 461
Jataizinho	11 244
Jesuítas	8 825
Joaquim Távora	10 247
Jundiaí do Sul	3 654
Juranda	7 684
Jussara	6 090
Kaloré	4 664
Lapa	41 679
Laranjal	6 322
Laranjeiras do Sul	30 481
Leópolis	4 230
Lidianópolis	4 123
Lindoeste	5 446
Loanda	19 464
Lobato	4 219
Londrina (2)	497 833
Luiziana	7 204
Lunardelli	5 082
Lupionópolis	4 375
Mallet	12 414
Mamborê	14 132
Mandaguaçu	18 259
Mandaguari	31 890
Mandirituba	20 408
Manfrinópolis	3 306
Mangueirinha	17 119
Manoel Ribas	12 762
Marechal Cândido Rondon	44 562
Maria Helena	6 012
Marialva	30 017
Marilândia do Sul	8 932
Marilena	6 541
Mariluz	10 461
Maringá (2)	325 968
Mariópolis	5 805
Maripá	5 571
Marmeleiro	13 156
Marquinho	5 205
Marumbi	4 142
Matelândia	15 404
Matinhos	23 357
Mato Rico	4 156
Mauá da Serra	7 814
Medianeira	38 397
Mercedes	4 713
Mirador	2 336
Miraselva	1 899
Missal	10 412
Moreira Sales	12 926

Tabela 1.1.21 - População recenseada e estimada, segundo os municípios - Paraná - 2007

(continuação)

Municípios	População recenseada e estimada (1)
Morretes	16 198
Munhoz de Melo	3 552
Nossa Senhora das Graças	3 903
Nova Aliança do Ivaí	1 377
Nova América da Colina	3 298
Nova Aurora	11 753
Nova Cantu	7 795
Nova Esperança	25 719
Nova Esperança do Sudoeste	5 182
Nova Fátima	8 054
Nova Laranjeiras	11 302
Nova Londrina	12 619
Nova Olímpia	5 227
Nova Prata do Iguaçu	10 452
Nova Santa Bárbara	3 802
Nova Santa Rosa	7 582
Nova Tebas	8 317
Novo Itacolomi	2 747
Ortigueira	24 397
Ourizona	3 296
Ouro Verde do Oeste	5 465
Paiçandu	34 640
Palmas	40 485
Palmeira	31 234
Palmital	15 454
Palotina	27 545
Paraíso do Norte	11 169
Paranacity	9 513
Paranaguá	133 559
Paranapoema	2 656
Paranavaí	79 110
Pato Bragado	4 631
Pato Branco	66 680
Paula Freitas	5 457
Paulo Frontin	7 032
Peabiru	13 029
Perobal	5 055
Pérola	9 334
Pérola d'Oeste	7 046
Piên	11 083
Pinhais	112 038
Pinhal de São Bento	2 524
Pinhalão	5 893
Pinhão	29 113
Piraí do Sul	23 170
Piraquara	82 006
Pitanga	34 310
Pitangueiras	2 664
Planaltina do Paraná	3 791
Planalto	13 649
Ponta Grossa (2)	306 351
Pontal do Paraná	16 625
Porecatu	14 174
Porto Amazonas	4 212
Porto Barreiro	3 761
Porto Rico	2 462
Porto Vitória	3 779

Tabela 1.1.21 - População recenseada e estimada, segundo os municípios - Paraná - 2007

(continuação)

Municípios	População recenseada e estimada (1)
Prado Ferreira	3 344
Pranchita	5 811
Presidente Castelo Branco	4 674
Primeiro de Maio	10 753
Prudentópolis	48 708
Quarto Centenário	4 848
Quatiguá	6 877
Quatro Barras	18 125
Quatro Pontes	3 669
Quedas do Iguaçu	30 181
Querência do Norte	11 804
Quinta do Sol	5 173
Quitandinha	15 897
Ramilândia	4 147
Rancho Alegre	4 001
Rancho Alegre D'Oeste	2 928
Realeza	15 809
Rebouças	14 053
Renascença	6 762
Reserva	24 249
Reserva do Iguaçu	7 094
Ribeirão Claro	10 882
Ribeirão do Pinhal	13 389
Rio Azul	13 248
Rio Bom	3 260
Rio Bonito do Iguaçu	14 450
Rio Branco do Ivaí	3 850
Rio Branco do Sul	31 486
Rio Negro	29 862
Rolândia	53 437
Roncador	12 265
Rondon	9 023
Rosário do Ivaí	5 823
Sabáudia	5 447
Salgado Filho	4 666
Salto do Itararé	5 087
Salto do Lontra	12 480
Santa Amélia	4 062
Santa Cecília do Pavão	3 676
Santa Cruz de Monte Castelo	7 924
Santa Fé	9 774
Santa Helena	22 794
Santa Inês	1 878
Santa Isabel do Ivaí	8 509
Santa Izabel do Oeste	11 434
Santa Lúcia	3 725
Santa Maria do Oeste	11 548
Santa Mariana	11 992
Santa Mônica	3 453
Santa Tereza do Oeste	9 378
Santa Terezinha de Itaipu	19 552
Santana do Itararé	5 537
Santo Antônio da Platina	40 480
Santo Antônio do Caiuá	2 692
Santo Antônio do Paraíso	2 354
Santo Antônio do Sudoeste	18 565
Santo Inácio	4 876

Tabela 1.1.21 - População recenseada e estimada, segundo os municípios - Paraná - 2007

(conclusão)

Municípios	População recenseada e estimada (1)
São Carlos do Ivaí	5 817
São Jerônimo da Serra	11 563
São João	10 900
São João do Caiuá	5 979
São João do Ivaí	11 854
São João do Triunfo	13 611
São Jorge do Ivaí	5 286
São Jorge do Patrocínio	6 031
São Jorge d'Oeste	8 979
São José da Boa Vista	6 293
São José das Palmeiras	3 873
São José dos Pinhais (2)	263 622
São Manoel do Paraná	2 093
São Mateus do Sul	39 152
São Miguel do Iguaçu	25 341
São Pedro do Iguaçu	6 540
São Pedro do Ivaí	9 569
São Pedro do Paraná	2 532
São Sebastião da Amoreira	8 681
São Tomé	5 279
Sapopema	6 638
Sarandi	79 686
Saudade do Iguaçu	4 931
Sengés	19 356
Serranópolis do Iguaçu	4 327
Sertaneja	5 894
Sertanópolis	15 485
Siqueira Campos	16 663
Sulina	3 445
Tamarana	10 887
Tamboara	4 564
Tapejara	14 498
Tapira	5 829
Teixeira Soares	9 781
Telêmaco Borba	65 797
Terra Boa	14 588
Terra Rica	14 405
Terra Roxa	16 208
Tibagi	18 710
Tijucas do Sul	13 091
Toledo	109 857
Tomazina	8 814
Três Barras do Paraná	11 718
Tunas do Paraná	5 921
Tuneiras do Oeste	8 598
Tupãssi	7 755
Turvo	14 025
Ubiratã	21 214
Umuarama	95 153
União da Vitória	51 043
Uniflor	2 402
Uraí	11 489
Ventania	10 275
Vera Cruz do Oeste	9 099
Verê	8 002
Virmond	4 024
Vitorino	6 310
Wenceslau Braz	18 691
Xambrê	5 818

Fonte: IBGE, Contagem da População 2007.

(1) Inclusive a população estimada nos domicílios fechados e nos domicílios provenientes de setores censitários cujos arquivos foram danificados. (2) População estimada.

Tabela 1.1.22 - População recenseada e estimada, segundo os municípios - Santa Catarina - 2007

(continua)

Municípios	População recenseada e estimada (1)
**Santa Catarina**	**5 866 252**
Abdon Batista	2 726
Abelardo Luz	16 374
Agrolândia	9 080
Agronômica	4 677
Água Doce	6 756
Águas de Chapecó	6 086
Águas Frias	2 551
Águas Mornas	4 410
Alfredo Wagner	9 754
Alto Bela Vista	2 021
Anchieta	6 587
Angelina	5 322
Anita Garibaldi	9 141
Anitápolis	3 175
Antônio Carlos	7 087
Apiúna	10 270
Arabutã	3 962
Araquari	21 278
Araranguá	57 119
Armazém	7 312
Arroio Trinta	3 516
Arvoredo	2 193
Ascurra	6 761
Atalanta	3 317
Aurora	5 399
Balneário Arroio do Silva	8 089
Balneário Barra do Sul	7 278
Balneário Camboriú	94 344
Balneário Gaivota	7 307
Balneário Piçarras	13 760
Bandeirante	3 028
Barra Bonita	2 064
Barra Velha	18 575
Bela Vista do Toldo	5 909
Belmonte	2 681
Benedito Novo	9 841
Biguaçu	53 444
Blumenau (2)	292 972
Bocaina do Sul	3 047
Bom Jardim da Serra	4 214
Bom Jesus	2 296
Bom Jesus do Oeste	2 026
Bom Retiro	8 258
Bombinhas	12 456
Botuverá	4 127
Braço do Norte	27 730
Braço do Trombudo	3 288

Tabela 1.1.22 - População recenseada e estimada, segundo os municípios - Santa Catarina - 2007

(continuação)

Municípios	População recenseada e estimada (1)
Brunópolis	2 934
Brusque	94 962
Caçador	67 556
Caibi	6 217
Calmon	4 012
Camboriú	53 388
Campo Alegre	11 391
Campo Belo do Sul	7 968
Campo Erê	9 590
Campos Novos	28 447
Canelinha	9 696
Canoinhas	52 677
Capão Alto	3 210
Capinzal	18 465
Capivari de Baixo	20 064
Catanduvas	8 733
Caxambu do Sul	4 885
Celso Ramos	2 671
Cerro Negro	3 948
Chapadão do Lageado	2 749
Chapecó	164 803
Cocal do Sul	14 563
Concórdia	67 249
Cordilheira Alta	3 361
Coronel Freitas	10 246
Coronel Martins	2 481
Correia Pinto	14 838
Corupá	12 758
Criciúma (2)	185 506
Cunha Porã	10 638
Cunhataí	1 874
Curitibanos	37 493
Descanso	8 705
Dionísio Cerqueira	14 792
Dona Emma	3 441
Doutor Pedrinho	3 280
Entre Rios	2 979
Ermo	1 843
Erval Velho	4 098
Faxinal dos Guedes	10 339
Flor do Sertão	1 640
Florianópolis (2)	396 723
Formosa do Sul	2 620
Forquilhinha	20 719
Fraiburgo	34 889
Frei Rogério	2 655
Galvão	3 493
Garopaba	16 399
Garuva	13 393
Gaspar	52 428

Tabela 1.1.22 - População recenseada e estimada, segundo os municípios - Santa Catarina - 2007

(continuação)

Municípios	População recenseada e estimada (1)
Governador Celso Ramos	12 175
Grão Pará	6 051
Gravatal	10 510
Guabiruba	16 095
Guaraciaba	10 604
Guaramirim	29 932
Guarujá do Sul	4 711
Guatambú	4 505
Herval d'Oeste	18 942
Ibiam	1 987
Ibicaré	3 390
Ibirama	16 716
Içara	54 107
Ilhota	11 552
Imaruí	11 675
Imbituba	36 231
Imbuia	5 501
Indaial	47 686
Iomerê	2 558
Ipira	4 705
Iporã do Oeste	8 091
Ipuaçu	6 566
Ipumirim	7 118
Iraceminha	4 261
Irani	9 313
Irati	2 025
Irineópolis	10 287
Itá	6 417
Itaiópolis	19 752
Itajaí	163 218
Itapema	33 766
Itapiranga	15 238
Itapoá	10 719
Ituporanga	20 577
Jaborá	4 032
Jacinto Machado	10 738
Jaguaruna	15 668
Jaraguá do Sul	129 973
Jardinópolis	1 851
Joaçaba	24 435
Joinville (2)	487 003
José Boiteux	4 840
Jupiá	2 134
Lacerdópolis	2 190
Lages	161 583
Laguna	50 179
Lajeado Grande	1 461
Laurentino	5 483
Lauro Muller	13 700
Lebon Régis	11 735

Tabela 1.1.22 - População recenseada e estimada, segundo os municípios - Santa Catarina - 2007

(continuação)

Municípios	População recenseada e estimada (1)
Leoberto Leal	3 589
Lindóia do Sul	4 560
Lontras	9 180
Luiz Alves	8 986
Luzerna	5 391
Macieira	1 760
Mafra	51 014
Major Gercino	2 842
Major Vieira	7 337
Maracajá	5 909
Maravilha	21 684
Marema	2 282
Massaranduba	13 777
Matos Costa	2 818
Meleiro	6 880
Mirim Doce	2 545
Modelo	3 772
Mondaí	9 126
Monte Carlo	8 854
Monte Castelo	8 113
Morro da Fumaça	15 426
Morro Grande	2 727
Navegantes	52 638
Nova Erechim	4 118
Nova Itaberaba	4 117
Nova Trento	11 325
Nova Veneza	12 536
Novo Horizonte	2 902
Orleans	20 859
Otacílio Costa	15 693
Ouro	7 095
Ouro Verde	2 152
Paial	1 821
Painel	2 297
Palhoça	122 471
Palma Sola	7 942
Palmeira	2 334
Palmitos	16 061
Papanduva	17 056
Paraíso	4 195
Passo de Torres	5 313
Passos Maia	4 472
Paulo Lopes	6 830
Pedras Grandes	4 817
Penha	20 868
Peritiba	2 944
Petrolândia	6 064
Pinhalzinho	14 691
Pinheiro Preto	2 912
Piratuba	4 570

Tabela 1.1.22 - População recenseada e estimada, segundo os municípios - Santa Catarina - 2007

(continuação)

Municípios	População recenseada e estimada (1)
Planalto Alegre	2 639
Pomerode	25 261
Ponte Alta	5 080
Ponte Alta do Norte	3 500
Ponte Serrada	11 210
Porto Belo	13 232
Porto União	32 256
Pouso Redondo	13 722
Praia Grande	7 120
Presidente Castello Branco	1 757
Presidente Getúlio	13 651
Presidente Nereu	2 259
Princesa	2 604
Quilombo	10 871
Rancho Queimado	2 772
Rio das Antas	6 054
Rio do Campo	6 042
Rio do Oeste	6 795
Rio do Sul	56 919
Rio dos Cedros	9 685
Rio Fortuna	4 468
Rio Negrinho	42 237
Rio Rufino	2 433
Riqueza	4 998
Rodeio	10 773
Romelândia	5 738
Salete	7 432
Saltinho	4 072
Salto Veloso	4 172
Sangão	10 300
Santa Cecília	15 311
Santa Helena	2 437
Santa Rosa de Lima	2 031
Santa Rosa do Sul	7 949
Santa Terezinha	9 025
Santa Terezinha do Progresso	3 044
Santiago do Sul	1 450
Santo Amaro da Imperatriz	17 602
São Bento do Sul	72 548
São Bernardino	2 653
São Bonifácio	3 178
São Carlos	10 372
São Cristovão do Sul	4 850
São Domingos	9 346
São Francisco do Sul	37 613
São João Batista	22 089
São João do Itaperiú	3 289
São João do Oeste	6 020
São João do Sul	6 916
São Joaquim	24 058

Tabela 1.1.22 - População recenseada e estimada, segundo os municípios - Santa Catarina - 2007

(conclusão)

Municípios	População recenseada e estimada (1)
São José (2)	196 887
São José do Cedro	13 699
São José do Cerrito	10 304
São Lourenço do Oeste	21 799
São Ludgero	10 246
São Martinho	3 194
São Miguel da Boa Vista	1 972
São Miguel do Oeste	33 806
São Pedro de Alcântara	4 765
Saudades	8 587
Schroeder	12 776
Seara	17 121
Serra Alta	3 200
Siderópolis	12 470
Sombrio	24 424
Sul Brasil	3 061
Taió	16 838
Tangará	8 410
Tigrinhos	1 741
Tijucas	27 804
Timbé do Sul	5 133
Timbó	33 326
Timbó Grande	6 979
Três Barras	17 937
Treviso	3 498
Treze de Maio	6 599
Treze Tílias	5 641
Trombudo Central	6 221
Tubarão	92 569
Tunápolis	4 650
Turvo	11 031
União do Oeste	3 058
Urubici	10 439
Urupema	2 501
Urussanga	18 588
Vargeão	3 560
Vargem	3 110
Vargem Bonita	4 321
Vidal Ramos	5 981
Videira	44 479
Vitor Meireles	5 563
Witmarsum	3 431
Xanxerê	40 228
Xavantina	4 218
Xaxim	24 318
Zortéa	2 868

Fonte: IBGE, Contagem da População 2007.

(1) Inclusive a população estimada nos domicílios fechados. (2) População estimada.

Tabela 1.1.23 - População recenseada e estimada, segundo os municípios - Rio Grande do Sul - 2007

(continua)

Municípios	População recenseada e estimada (1)
**Rio Grande do Sul**	**10 582 840**
Aceguá	4 138
Água Santa	3 565
Agudo	16 714
Ajuricaba	7 261
Alecrim	7 357
Alegrete	78 188
Alegria	4 789
Almirante Tamandaré do Sul	2 158
Alpestre	8 972
Alto Alegre	1 940
Alto Feliz	2 934
Alvorada (2)	207 142
Amaral Ferrador	6 232
Ametista do Sul	8 058
André da Rocha	1 206
Anta Gorda	6 163
Antônio Prado	13 591
Arambaré	3 825
Araricá	4 781
Aratiba	6 616
Arroio do Meio	18 079
Arroio do Padre	2 734
Arroio do Sal	6 635
Arroio do Tigre	12 638
Arroio dos Ratos	13 619
Arroio Grande	18 358
Arvorezinha	10 210
Augusto Pestana	7 273
Áurea	3 715
Bagé	112 550
Balneário Pinhal	10 517
Barão	5 293
Barão de Cotegipe	6 519
Barão do Triunfo	6 924
Barra do Guarita	2 969
Barra do Quaraí	3 776
Barra do Ribeiro	11 478
Barra do Rio Azul	2 028
Barra Funda	2 338
Barracão	5 306
Barros Cassal	11 450
Benjamin Constant do Sul	2 256
Bento Gonçalves	100 643
Boa Vista das Missões	2 066
Boa Vista do Buricá	6 468
Boa Vista do Cadeado	2 447
Boa Vista do Incra	2 447
Boa Vista do Sul	2 663
Bom Jesus	11 843
Bom Princípio	10 910
Bom Progresso	2 441
Bom Retiro do Sul	11 130
Boqueirão do Leão	7 825
Bossoroca	7 652
Bozano	2 296

Tabela 1.1.23 - População recenseada e estimada, segundo os municípios - Rio Grande do Sul - 2007

(continuação)

Municípios	População recenseada e estimada (1)
Braga	3 826
Brochier	4 701
Butiá	19 717
Caçapava do Sul	32 574
Cacequi	13 629
Cachoeira do Sul	84 629
Cachoeirinha	112 603
Cacique Doble	4 824
Caibaté	5 080
Caiçara	5 184
Camaquã	60 563
Camargo	2 471
Cambará do Sul	6 959
Campestre da Serra	3 205
Campina das Missões	6 342
Campinas do Sul	5 588
Campo Bom	56 595
Campo Novo	5 581
Campos Borges	3 564
Candelária	29 444
Cândido Godói	6 634
Candiota	8 236
Canela	38 318
Canguçu	53 547
Canoas (2)	326 458
Canudos do Vale	1 941
Capão Bonito do Sul	1 837
Capão da Canoa	37 405
Capão do Cipó	3 180
Capão do Leão	23 655
Capela de Santana	10 950
Capitão	2 595
Capivari do Sul	3 339
Caraá	7 131
Carazinho	58 196
Carlos Barbosa	23 960
Carlos Gomes	1 719
Casca	8 381
Caseiros	2 989
Catuípe	9 499
Caxias do Sul (2)	399 038
Centenário	3 026
Cerrito	6 629
Cerro Branco	4 465
Cerro Grande	2 577
Cerro Grande do Sul	9 233
Cerro Largo	12 484
Chapada	9 440
Charqueadas	33 705
Charrua	3 581
Chiapetta	4 078
Chuí	5 278
Chuvisca	4 874
Cidreira	10 883
Ciríaco	4 945
Colinas	2 404
Colorado	3 744

Tabela 1.1.23 - População recenseada e estimada, segundo os municípios - Rio Grande do Sul - 2007

(continuação)

Municípios	População recenseada e estimada (1)
Condor	6 607
Constantina	9 842
Coqueiro Baixo	1 566
Coqueiros do Sul	3 106
Coronel Barros	2 441
Coronel Bicaco	7 873
Coronel Pilar	1 658
Cotiporã	4 577
Coxilha	2 916
Crissiumal	14 726
Cristal	7 026
Cristal do Sul	2 967
Cruz Alta	63 450
Cruzaltense	2 273
Cruzeiro do Sul	12 171
David Canabarro	4 704
Derrubadas	3 378
Dezesseis de Novembro	2 968
Dilermando de Aguiar	3 129
Dois Irmãos	24 815
Dois Irmãos das Missões	2 362
Dois Lajeados	3 334
Dom Feliciano	14 504
Dom Pedrito	38 148
Dom Pedro de Alcântara	2 728
Dona Francisca	3 572
Doutor Maurício Cardoso	5 494
Doutor Ricardo	2 053
Eldorado do Sul	31 316
Encantado	19 536
Encruzilhada do Sul	24 152
Engenho Velho	1 584
Entre Rios do Sul	3 101
Entre-Ijuís	9 126
Erebango	2 881
Erechim	92 945
Ernestina	3 010
Erval Grande	5 294
Erval Seco	8 212
Esmeralda	3 234
Esperança do Sul	3 445
Espumoso	14 991
Estação	6 086
Estância Velha	40 740
Esteio	78 816
Estrela	29 071
Estrela Velha	3 659
Eugênio de Castro	3 057
Fagundes Varela	2 508
Farroupilha	59 871
Faxinal do Soturno	6 343
Faxinalzinho	2 613
Fazenda Vilanova	3 068
Feliz	11 679
Flores da Cunha	25 307
Floriano Peixoto	2 148
Fontoura Xavier	11 074

Tabela 1.1.23 - População recenseada e estimada, segundo os municípios - Rio Grande do Sul - 2007

(continuação)

Municípios	População recenseada e estimada (1)
Formigueiro	7 116
Forquetinha	2 548
Fortaleza dos Valos	4 597
Frederico Westphalen	27 308
Garibaldi	28 791
Garruchos	3 457
Gaurama	6 108
General Câmara	8 782
Gentil	1 579
Getúlio Vargas	15 961
Giruá	17 070
Glorinha	6 908
Gramado	31 652
Gramado dos Loureiros	2 370
Gramado Xavier	3 911
Gravataí (2)	261 150
Guabiju	1 669
Guaíba	93 578
Guaporé	21 421
Guarani das Missões	8 331
Harmonia	3 658
Herval	6 873
Herveiras	2 825
Horizontina	18 305
Hulha Negra	6 030
Humaitá	4 923
Ibarama	4 331
Ibiaçá	4 681
Ibiraiaras	7 094
Ibirapuitã	4 182
Ibirubá	18 690
Igrejinha	31 113
Ijuí	76 739
Ilópolis	4 202
Imbé	14 940
Imigrante	3 013
Independência	6 679
Inhacorá	2 290
Ipê	5 875
Ipiranga do Sul	1 983
Iraí	8 468
Itaara	4 633
Itacurubi	3 568
Itapuca	2 454
Itaqui	36 361
Itati	2 677
Itatiba do Sul	4 574
Ivorá	2 378
Ivoti	18 517
Jaboticaba	4 173
Jacuizinho	2 619
Jacutinga	3 567
Jaguarão	27 944
Jaguari	11 626
Jaquirana	4 404
Jari	3 692
Jóia	8 279

Tabela 1.1.23 - População recenseada e estimada, segundo os municípios - Rio Grande do Sul - 2007

(continuação)

Municípios	População recenseada e estimada (1)
Júlio de Castilhos	19 541
Lagoa Bonita do Sul	2 617
Lagoa dos Três Cantos	1 590
Lagoa Vermelha	27 434
Lagoão	6 389
Lajeado	67 474
Lajeado do Bugre	2 536
Lavras do Sul	8 115
Liberato Salzano	6 102
Lindolfo Collor	5 279
Linha Nova	1 488
Maçambara	4 415
Machadinho	5 503
Mampituba	2 926
Manoel Viana	6 784
Maquiné	7 374
Maratá	2 444
Marau	33 778
Marcelino Ramos	5 372
Mariana Pimentel	3 964
Mariano Moro	2 284
Marques de Souza	4 043
Mata	5 291
Mato Castelhano	2 608
Mato Leitão	3 585
Mato Queimado	1 865
Maximiliano de Almeida	5 059
Minas do Leão	7 728
Miraguaí	4 869
Montauri	1 583
Monte Alegre dos Campos	3 122
Monte Belo do Sul	2 766
Montenegro	56 790
Mormaço	2 578
Morrinhos do Sul	3 241
Morro Redondo	6 199
Morro Reuter	5 599
Mostardas	11 903
Muçum	4 574
Muitos Capões	2 969
Muliterno	1 800
Não-Me-Toque	15 228
Nicolau Vergueiro	1 759
Nonoai	12 327
Nova Alvorada	3 058
Nova Araçá	3 775
Nova Bassano	8 683
Nova Boa Vista	2 083
Nova Bréscia	3 162
Nova Candelária	2 739
Nova Esperança do Sul	4 775
Nova Hartz	16 688
Nova Pádua	2 484
Nova Palma	6 432
Nova Petrópolis	17 747
Nova Prata	22 257
Nova Ramada	2 461

Tabela 1.1.23 - População recenseada e estimada, segundo os municípios - Rio Grande do Sul - 2007

(continuação)

Municípios	População recenseada e estimada (1)
Nova Roma do Sul	3 490
Nova Santa Rita	20 591
Novo Barreiro	3 858
Novo Cabrais	3 766
Novo Hamburgo (2)	253 067
Novo Machado	4 246
Novo Tiradentes	2 331
Novo Xingu	1 858
Osório	39 290
Paim Filho	4 480
Palmares do Sul	11 423
Palmeira das Missões	33 846
Palmitinho	6 905
Panambi	36 360
Pantano Grande	9 816
Paraí	6 577
Paraíso do Sul	7 346
Pareci Novo	3 151
Parobé	48 713
Passa Sete	4 996
Passo do Sobrado	5 967
Passo Fundo (2)	183 300
Paulo Bento	2 090
Paverama	7 616
Pedras Altas	2 546
Pedro Osório	8 039
Pejuçara	3 900
Pelotas (2)	339 934
Picada Café	4 824
Pinhal	2 362
Pinhal da Serra	2 058
Pinhal Grande	4 496
Pinheirinho do Vale	4 411
Pinheiro Machado	12 939
Pirapó	2 988
Piratini	20 225
Planalto	10 589
Poço das Antas	1 976
Pontão	3 904
Ponte Preta	1 840
Portão	28 583
Porto Alegre (2)	1 420 667
Porto Lucena	5 631
Porto Mauá	2 565
Porto Vera Cruz	2 084
Porto Xavier	10 857
Pouso Novo	1 992
Presidente Lucena	2 355
Progresso	6 210
Protásio Alves	2 114
Putinga	4 192
Quaraí	22 552
Quatro Irmãos	1 735
Quevedos	2 732
Quinze de Novembro	3 561
Redentora	9 600
Relvado	2 196

Tabela 1.1.23 - População recenseada e estimada, segundo os municípios - Rio Grande do Sul - 2007

(continuação)

Municípios	População recenseada e estimada (1)
Restinga Seca	15 595
Rio dos Índios	4 202
Rio Grande (2)	194 351
Rio Pardo	37 704
Riozinho	4 406
Roca Sales	9 922
Rodeio Bonito	5 698
Rolador	2 795
Rolante	19 213
Ronda Alta	9 654
Rondinha	5 674
Roque Gonzales	7 297
Rosário do Sul	40 509
Sagrada Família	2 595
Saldanha Marinho	2 982
Salto do Jacuí	12 154
Salvador das Missões	2 601
Salvador do Sul	6 644
Sananduva	14 714
Santa Bárbara do Sul	9 122
Santa Cecília do Sul	1 716
Santa Clara do Sul	5 471
Santa Cruz do Sul	115 857
Santa Margarida do Sul	2 163
Santa Maria (2)	263 403
Santa Maria do Herval	6 173
Santa Rosa	64 113
Santa Tereza	1 815
Santa Vitória do Palmar	31 183
Santana da Boa Vista	8 599
Santana do Livramento	83 479
Santiago	49 558
Santo Ângelo	73 800
Santo Antônio da Patrulha	37 910
Santo Antônio das Missões	11 863
Santo Antônio do Palma	2 216
Santo Antônio do Planalto	2 029
Santo Augusto	13 622
Santo Cristo	14 280
Santo Expedito do Sul	2 614
São Borja	61 834
São Domingos do Sul	2 854
São Francisco de Assis	19 523
São Francisco de Paula	21 278
São Gabriel	57 978
São Jerônimo	20 506
São João da Urtiga	4 946
São João do Polêsine	2 702
São Jorge	2 764
São José das Missões	2 952
São José do Herval	2 479
São José do Hortêncio	3 883
São José do Inhacorá	2 132
São José do Norte	24 905
São José do Ouro	6 973
São José do Sul	1 899
São José dos Ausentes	3 180

Tabela 1.1.23 - População recenseada e estimada, segundo os municípios - Rio Grande do Sul - 2007

(continuação)

Municípios	População recenseada e estimada (1)
São Leopoldo (2)	207 721
São Lourenço do Sul	42 339
São Luiz Gonzaga	34 487
São Marcos	19 641
São Martinho	5 853
São Martinho da Serra	3 409
São Miguel das Missões	7 382
São Nicolau	5 909
São Paulo das Missões	6 690
São Pedro da Serra	3 117
São Pedro das Missões	1 939
São Pedro do Butiá	2 744
São Pedro do Sul	16 613
São Sebastião do Caí	20 359
São Sepé	23 787
São Valentim	3 919
São Valentim do Sul	2 230
São Valério do Sul	2 635
São Vendelino	1 794
São Vicente do Sul	8 361
Sapiranga	73 979
Sapucaia do Sul	122 231
Sarandi	20 415
Seberi	10 870
Sede Nova	2 968
Segredo	7 022
Selbach	4 773
Senador Salgado Filho	2 861
Sentinela do Sul	5 290
Serafina Corrêa	13 463
Sério	2 399
Sertão	6 663
Sertão Santana	5 791
Sete de Setembro	2 131
Severiano de Almeida	3 907
Silveira Martins	2 479
Sinimbu	10 315
Sobradinho	14 162
Soledade	29 926
Tabaí	4 046
Tapejara	17 500
Tapera	10 457
Tapes	16 557
Taquara	53 428
Taquari	25 768
Taquaruçu do Sul	2 849
Tavares	5 160
Tenente Portela	13 906
Terra de Areia	9 709
Teutônia	25 105

Tabela 1.1.23 - População recenseada e estimada, segundo os municípios - Rio Grande do Sul - 2007

(conclusão)

Municípios	População recenseada e estimada (1)
Tio Hugo	2 593
Tiradentes do Sul	6 928
Toropi	3 070
Torres	32 358
Tramandaí	39 891
Travesseiro	2 379
Três Arroios	3 015
Três Cachoeiras	10 390
Três Coroas	22 905
Três de Maio	23 333
Três Forquilhas	3 073
Três Palmeiras	4 388
Três Passos	23 467
Trindade do Sul	5 905
Triunfo	23 976
Tucunduva	5 907
Tunas	4 378
Tupanci do Sul	1 723
Tupanciretã	22 556
Tupandi	3 604
Tuparendi	8 793
Turuçu	3 829
Ubiretama	2 440
União da Serra	1 666
Unistalda	2 392
Uruguaiana	123 743
Vacaria	59 938
Vale do Sol	10 817
Vale Real	4 741
Vale Verde	3 227
Vanini	1 956
Venâncio Aires	64 442
Vera Cruz	22 702
Veranópolis	23 904
Vespasiano Correa	1 973
Viadutos	5 663
Viamão (2)	253 264
Vicente Dutra	5 569
Victor Graeff	3 080
Vila Flores	3 169
Vila Lângaro	2 230
Vila Maria	4 159
Vila Nova do Sul	4 255
Vista Alegre	2 871
Vista Alegre do Prata	1 492
Vista Gaúcha	2 713
Vitória das Missões	3 652
Westfalia	2 716
Xangri-lá	10 602

Fonte: IBGE, Contagem da População 2007.

(1) Inclusive a população estimada nos domicílios fechados. (2) População estimada.

Tabela 1.1.24 - População recenseada e estimada, segundo os municípios - Mato Grosso do Sul - 2007

(continua)

Municípios	População recenseada e estimada (1)
**Mato Grosso do Sul**	**2 265 274**
Água Clara	13 183
Alcinópolis	4 299
Amambaí	33 426
Anastácio	22 364
Anaurilândia	8 380
Angélica	7 253
Antônio João	8 350
Aparecida do Taboado	19 819
Aquidauana	44 920
Aral Moreira	9 236
Bandeirantes	5 888
Bataguassu	18 687
Batayporã	10 564
Bela Vista	22 868
Bodoquena	8 168
Bonito	17 275
Brasilândia	12 136
Caarapó	22 723
Camapuã	13 192
Campo Grande	724 524
Caracol	5 095
Cassilândia	20 916
Chapadão do Sul	16 193
Corguinho	4 165
Coronel Sapucaia	13 979
Corumbá	96 373
Costa Rica	18 277
Coxim	31 816
Deodápolis	11 261
Dois Irmãos do Buriti	9 350
Douradina	4 900
Dourados	181 869
Eldorado	11 934
Fátima do Sul	18 789
Figueirão	3 280
Glória de Dourados	9 644
Guia Lopes da Laguna	10 208
Iguatemi	14 632

Tabela 1.1.24 - População recenseada e estimada, segundo os municípios - Mato Grosso do Sul - 2007

(conclusão)

Municípios	População recenseada e estimada (1)
Inocência	7 342
Itaporã	18 605
Itaquiraí	16 924
Ivinhema	20 567
Japorã	7 362
Jaraguari	5 577
Jardim	23 341
Jateí	3 808
Juti	5 353
Ladário	17 906
Laguna Carapã	5 813
Maracaju	30 912
Miranda	23 965
Mundo Novo	15 968
Naviraí	43 391
Nioaque	15 203
Nova Alvorada do Sul	12 026
Nova Andradina	43 495
Novo Horizonte do Sul	4 967
Paranaíba	38 969
Paranhos	11 092
Pedro Gomes	8 307
Ponta Porã	72 207
Porto Murtinho	14 861
Ribas do Rio Pardo	19 159
Rio Brilhante	26 560
Rio Negro	4 961
Rio Verde de Mato Grosso	18 579
Rochedo	4 346
Santa Rita do Pardo	7 162
São Gabriel do Oeste	20 524
Selvíria	6 413
Sete Quedas	10 659
Sidrolândia	38 147
Sonora	12 548
Tacuru	9 203
Taquarussu	3 117
Terenos	14 458
Três Lagoas	85 914
Vicentina	5 627

Fonte: IBGE, Contagem da População 2007.

(1) Inclusive a população estimada nos domicílios fechados. (2) População estimada.

Tabela 1.1.25 - População recenseada e estimada, segundo os municípios - Mato Grosso - 2007

(continua)

Municípios	População recenseada e estimada (1)
**Mato Grosso**	**2 854 642**
Acorizal	5 530
Água Boa	18 991
Alta Floresta	49 140
Alto Araguaia	13 790
Alto Boa Vista	5 025
Alto Garças	9 132
Alto Paraguai	8 144
Alto Taquari	6 058
Apiacás	7 926
Araguaiana	2 974
Araguainha	1 117
Araputanga	15 335
Arenápolis	9 869
Aripuanã	19 100
Barão de Melgaço	7 619
Barra do Bugres	32 490
Barra do Garças	53 243
Bom Jesus do Araguaia	4 520
Brasnorte	13 975
Cáceres	84 175
Campinápolis	13 666
Campo Novo do Parecis	22 322
Campo Verde	25 924
Campos de Júlio	4 770
Canabrava do Norte	5 337
Canarana	17 183
Carlinda	12 108
Castanheira	7 808
Chapada dos Guimarães	17 377
Cláudia	10 670
Cocalinho	5 841
Colíder	30 695
Colniza	27 882
Comodoro	17 939
Confresa	21 361
Conquista D'Oeste	3 106
Cotriguaçu	13 740
Cuiabá	526 830
Curvelândia	4 835
Denise	10 349
Diamantino	18 428
Dom Aquino	8 261
Feliz Natal	10 279
Figueirópolis D'Oeste	3 648
Gaúcha do Norte	5 816
General Carneiro	4 803
Glória D'Oeste	3 124
Guarantã do Norte	30 754

Tabela 1.1.25 - População recenseada e estimada, segundo os municípios - Mato Grosso - 2007

(continuação)

Municípios	População recenseada e estimada (1)
Guiratinga	13 883
Indiavaí	2 524
Ipiranga do Norte	4 129
Itanhangá	4 703
Itaúba	4 625
Itiquira	12 159
Jaciara	24 945
Jangada	8 058
Jauru	10 774
Juara	32 023
Juína	38 422
Juruena	8 731
Juscimeira	11 830
Lambari D'Oeste	4 870
Lucas do Rio Verde	30 741
Luciára	2 405
Marcelândia	14 084
Matupá	14 243
Mirassol d'Oeste	24 538
Nobres	14 862
Nortelândia	6 237
Nossa Senhora do Livramento	12 386
Nova Bandeirantes	12 742
Nova Brasilândia	4 891
Nova Canaã do Norte	12 652
Nova Guarita	4 877
Nova Lacerda	4 949
Nova Marilândia	2 315
Nova Maringá	5 554
Nova Monte Verde	8 133
Nova Mutum	24 368
Nova Nazaré	2 745
Nova Olímpia	19 474
Nova Santa Helena	3 347
Nova Ubiratã	7 782
Nova Xavantina	18 670
Novo Horizonte do Norte	3 802
Novo Mundo	6 725
Novo Santo Antônio	2 110
Novo São Joaquim	6 880
Paranaíta	11 540
Paranatinga	20 033
Pedra Preta	15 638
Peixoto de Azevedo	28 987
Planalto da Serra	2 734
Poconé	31 118

Tabela 1.1.25 - População recenseada e estimada, segundo os municípios - Mato Grosso - 2007

(conclusão)

Municípios	População recenseada e estimada (1)
Pontal do Araguaia	4 966
Ponte Branca	1 794
Pontes e Lacerda	37 910
Porto Alegre do Norte	9 639
Porto dos Gaúchos	6 116
Porto Esperidião	9 607
Porto Estrela	4 011
Poxoréo	17 592
Primavera do Leste	44 729
Querência	10 682
Reserva do Cabaçal	2 505
Ribeirão Cascalheira	8 677
Ribeirãozinho	2 107
Rio Branco	5 053
Rondolândia	3 348
Rondonópolis	172 783
Rosário Oeste	18 031
Salto do Céu	3 650
Santa Carmem	4 319
Santa Cruz do Xingu	2 116
Santa Rita do Trivelato	2 478
Santa Terezinha	7 293
Santo Afonso	2 855
Santo Antônio do Leste	3 249
Santo Antônio do Leverger	19 197
São Félix do Araguaia	10 713
São José do Povo	3 305
São José do Rio Claro	17 345
São José do Xingu	4 198
São José dos Quatro Marcos	19 001
São Pedro da Cipa	4 028
Sapezal	14 254
Serra Nova Dourada	1 349
Sinop	105 762
Sorriso	55 134
Tabaporã	10 484
Tangará da Serra	76 657
Tapurah	10 478
Terra Nova do Norte	14 584
Tesouro	3 109
Torixoréu	4 101
União do Sul	3 998
Vale de São Domingos	2 873
Várzea Grande	230 307
Vera	9 188
Vila Bela da Santíssima Trindade	13 886
Vila Rica	18 934

Fonte: IBGE, Contagem da População 2007.

(1) Inclusive a população estimada nos domicílios fechados. (2) População estimada.

Tabela 1.1.26 - População recenseada e estimada, segundo os municípios - Goiás - 2007

(continua)

Municípios	População recenseada e estimada (1)
**Goiás**	**5 647 035**
Abadia de Goiás	5 868
Abadiânia	12 640
Acreúna	18 553
Adelândia	2 510
Água Fria de Goiás	5 008
Água Limpa	2 074
Águas Lindas de Goiás	131 884
Alexânia	20 033
Aloândia	2 065
Alto Horizonte	3 136
Alto Paraíso de Goiás	6 638
Alvorada do Norte	8 217
Amaralina	3 492
Americano do Brasil	4 698
Amorinópolis	3 527
Anápolis (2)	325 544
Anhanguera	966
Anicuns	17 705
Aparecida de Goiânia (2)	475 303
Aparecida do Rio Doce	2 702
Aporé	3 554
Araçu	3 880
Aragarças	17 156
Aragoiânia	7 243
Araguapaz	7 482
Arenópolis	3 495
Aruanã	6 476
Aurilândia	3 719
Avelinópolis	2 375
Baliza	3 299
Barro Alto	6 446
Bela Vista de Goiás	20 615
Bom Jardim de Goiás	8 372
Bom Jesus de Goiás	19 574
Bonfinópolis	6 744
Bonópolis	3 160
Brazabrantes	3 142
Britânia	5 073
Buriti Alegre	8 287
Buriti de Goiás	2 238
Buritinópolis	3 442
Cabeceiras	6 610
Cachoeira Alta	8 103
Cachoeira de Goiás	1 410
Cachoeira Dourada	7 567
Caçu	10 892
Caiapônia	15 747
Caldas Novas	62 204
Caldazinha	3 157
Campestre de Goiás	3 411
Campinaçu	3 765
Campinorte	9 697
Campo Alegre de Goiás	5 767
Campo Limpo de Goiás	5 596
Campos Belos	18 238
Campos Verdes	6 331
Carmo do Rio Verde	8 897
Castelândia	3 530
Catalão	75 623
Caturaí	4 477

Tabela 1.1.26 - População recenseada e estimada, segundo os municípios - Goiás - 2007

(continuação)

Municípios	População recenseada e estimada (1)
Cavalcante	9 875
Ceres	18 637
Cezarina	7 361
Chapadão do Céu	5 289
Cidade Ocidental	48 589
Cocalzinho de Goiás	14 762
Colinas do Sul	3 856
Córrego do Ouro	2 633
Corumbá de Goiás	9 190
Corumbaíba	8 001
Cristalina	36 614
Cristianópolis	3 041
Crixás	14 547
Cromínia	3 618
Cumari	3 055
Damianópolis	3 507
Damolândia	2 688
Davinópolis	2 013
Diorama	2 236
Divinópolis de Goiás	5 344
Doverlândia	8 344
Edealina	3 718
Edéia	10 251
Estrela do Norte	3 174
Faina	6 918
Fazenda Nova	6 373
Firminópolis	10 286
Flores de Goiás	10 382
Formosa	90 212
Formoso	5 241
Gameleira de Goiás	3 289
Goianápolis	11 159
Goiandira	4 925
Goianésia	53 806
Goiânia (2)	1 244 645
Goianira	24 110
Goiás	24 472
Goiatuba	31 225
Gouvelândia	4 507
Guapó	13 586
Guaraíta	2 394
Guarani de Goiás	4 105
Guarinos	2 411
Heitoraí	3 556
Hidrolândia	14 004
Hidrolina	4 157
Iaciara	12 674
Inaciolândia	5 650
Indiara	12 703
Inhumas	44 983
Ipameri	23 114
Ipiranga de Goiás	2 813
Iporá	31 060
Israelândia	2 827
Itaberaí	30 609
Itaguari	4 254
Itaguaru	5 467
Itajá	5 409
Itapaci	16 003
Itapirapuã	8 208
Itapuranga	24 832

Tabela 1.1.26 - População recenseada e estimada, segundo os municípios - Goiás - 2007

(continuação)

Municípios	População recenseada e estimada (1)
Itarumã	5 338
Itauçu	8 710
Itumbiara	88 109
Ivolândia	2 718
Jandaia	6 373
Jaraguá	38 968
Jataí	81 972
Jaupaci	2 998
Jesúpolis	2 201
Joviânia	6 731
Jussara	18 814
Lagoa Santa	1 225
Leopoldo de Bulhões	8 916
Luziânia (2)	196 046
Mairipotaba	2 655
Mambaí	6 454
Mara Rosa	10 409
Marzagão	2 053
Matrinchã	4 325
Maurilândia	10 769
Mimoso de Goiás	2 836
Minaçu	31 041
Mineiros	45 189
Moiporá	1 848
Monte Alegre de Goiás	7 155
Montes Claros de Goiás	7 844
Montividiu	9 255
Montividiu do Norte	4 395
Morrinhos	38 997
Morro Agudo de Goiás	2 339
Mossâmedes	4 954
Mozarlândia	13 123
Mundo Novo	6 877
Mutunópolis	3 954
Nazário	7 223
Nerópolis	19 392
Niquelândia	38 517
Nova América	2 200
Nova Aurora	2 094
Nova Crixás	12 603
Nova Glória	8 470
Nova Iguaçu de Goiás	2 622
Nova Roma	3 555
Nova Veneza	6 884
Novo Brasil	3 451
Novo Gama	83 599
Novo Planalto	3 986
Orizona	14 374
Ouro Verde de Goiás	4 430
Ouvidor	4 736
Padre Bernardo	25 969
Palestina de Goiás	3 229
Palmeiras de Goiás	21 199
Palmelo	2 260
Palminópolis	3 622
Panamá	2 618
Paranaiguara	7 724
Paraúna	10 926
Perolândia	2 748
Petrolina de Goiás	9 864
Pilar de Goiás	2 852
Piracanjuba	23 310
Piranhas	11 139
Pirenópolis	20 460

Tabela 1.1.26 - População recenseada e estimada, segundo os municípios - Goiás - 2007

(conclusão)

Municípios	População recenseada e estimada (1)
Pires do Rio	26 857
Planaltina	76 376
Pontalina	16 226
Porangatu	39 238
Porteirão	3 008
Portelândia	3 310
Posse	28 850
Professor Jamil	3 298
Quirinópolis	38 064
Rialma	10 485
Rianápolis	4 167
Rio Quente	2 959
Rio Verde	149 382
Rubiataba	18 025
Sanclerlândia	7 647
Santa Bárbara de Goiás	5 658
Santa Cruz de Goiás	3 542
Santa Fé de Goiás	4 594
Santa Helena de Goiás	35 027
Santa Isabel	3 485
Santa Rita do Araguaia	5 873
Santa Rita do Novo Destino	3 372
Santa Rosa de Goiás	2 851
Santa Tereza de Goiás	4 145
Santa Terezinha de Goiás	11 558
Santo Antônio da Barra	4 134
Santo Antônio de Goiás	3 893
Santo Antônio do Descoberto	55 621
São Domingos	9 786
São Francisco de Goiás	5 713
São João da Paraúna	1 699
São João d'Aliança	8 177
São Luís de Montes Belos	26 784
São Luíz do Norte	4 266
São Miguel do Araguaia	22 468
São Miguel do Passa Quatro	3 680
São Patrício	2 051
São Simão	13 832
Senador Canedo	70 559
Serranópolis	7 333
Silvânia	18 370
Simolândia	6 915
Sítio d'Abadia	3 251
Taquaral de Goiás	3 404
Teresina de Goiás	2 773
Terezópolis de Goiás	5 620
Três Ranchos	2 862
Trindade	97 491
Trombas	3 537
Turvânia	4 962
Turvelândia	3 852
Uirapuru	3 021
Uruaçu	33 382
Uruana	13 712
Urutaí	2 727
Valparaíso de Goiás	114 450
Varjão	3 649
Vianópolis	12 187
Vicentinópolis	5 919
Vila Boa	4 198
Vila Propício	5 044

Fonte: IBGE, Contagem da População 2007.
(1) Inclusive a população estimada nos domicílios fechados. (2) População estimada

Tabela 1.2.1 - População recenseada, por sexo, segundo a idade - Rondônia - 2007

(continua)

Idade	População recenseada, por sexo		
	Total	Homens	Mulheres
**Total**	**(1) 1 453 756**	**733 811**	**709 923**
Menos de 1 ano	23 991	12 107	11 884
1 ano	25 313	12 853	12 460
2 anos	25 174	12 892	12 282
3 anos	26 035	13 363	12 672
4 anos	26 081	13 246	12 835
5 anos	27 309	13 893	13 416
6 anos	29 331	14 928	14 403
7 anos	30 165	15 393	14 772
8 anos	28 936	14 883	14 053
9 anos	29 998	15 381	14 617
10 anos	31 154	15 931	15 223
11 anos	31 063	15 691	15 372
12 anos	32 542	16 473	16 069
13 anos	31 272	15 693	15 579
14 anos	30 236	15 420	14 816
15 anos	29 909	15 043	14 866
16 anos	29 505	14 860	14 645
17 anos	30 075	15 208	14 867
18 anos	30 266	15 378	14 888
19 anos	28 936	14 491	14 445
20 anos	28 846	14 392	14 454
21 anos	27 765	13 895	13 870
22 anos	28 337	14 269	14 068
23 anos	27 579	13 943	13 636
24 anos	28 869	14 538	14 331
25 anos	27 864	13 776	14 088
26 anos	27 013	13 248	13 765
27 anos	27 263	13 502	13 761
28 anos	25 275	12 497	12 778
29 anos	24 482	11 992	12 490
30 anos	25 231	12 547	12 684
31 anos	22 871	11 005	11 866
32 anos	23 749	11 530	12 219
33 anos	22 244	10 959	11 285
34 anos	22 594	11 082	11 512
35 anos	21 757	10 653	11 104
36 anos	21 654	10 887	10 767
37 anos	21 760	10 824	10 936
38 anos	21 267	10 855	10 412

Tabela 1.2.1 - População recenseada, por sexo, segundo a idade - Rondônia - 2007

(conclusão)

Idade	População recenseada, por sexo		
	Total	Homens	Mulheres
39 anos	20 063	10 148	9 915
40 anos	21 626	11 154	10 472
41 anos	18 646	9 604	9 042
42 anos	20 033	10 527	9 506
43 anos	18 585	9 794	8 791
44 anos	17 621	9 219	8 402
45 anos	16 456	8 720	7 736
46 anos	15 984	8 409	7 575
47 anos	15 052	7 789	7 263
48 anos	14 408	7 383	7 025
49 anos	13 970	7 214	6 756
50 anos	13 470	7 095	6 375
51 anos	12 096	6 167	5 929
52 anos	12 128	6 388	5 740
53 anos	11 247	5 854	5 393
54 anos	10 632	5 505	5 127
55 anos	9 828	4 869	4 959
56 anos	9 820	5 132	4 688
57 anos	8 630	4 537	4 093
58 anos	7 885	4 161	3 724
59 anos	7 787	4 186	3 601
60 anos	7 496	3 982	3 514
61 anos	6 330	3 451	2 879
62 anos	6 399	3 386	3 013
63 anos	6 107	3 193	2 914
64 anos	6 178	3 276	2 902
65 anos	5 496	2 923	2 573
66 anos	5 584	2 947	2 637
67 anos	5 293	2 836	2 457
68 anos	4 437	2 344	2 093
69 anos	3 861	2 099	1 762
70 anos	3 962	2 196	1 766
71 anos	3 515	1 928	1 587
72 anos	3 404	1 834	1 570
73 anos	2 996	1 647	1 349
74 anos	2 979	1 590	1 389
75 anos	2 433	1 334	1 099
76 anos	2 707	1 467	1 240
77 anos	2 201	1 179	1 022
78 anos	1 913	1 050	863
79 anos	1 578	886	692
80 anos ou mais	8 870	4 734	4 136
Idade ignorada	317	153	164

Fonte: IBGE, Contagem da População 2007.
(1) Inclusive a população estimada nos domicílios fechados.

Tabela 1.2.2 - População recenseada, por sexo, segundo a idade - Acre - 2007

(continua)

Idade	População recenseada, por sexo		
	Total	Homens	Mulheres
**Total**	**(1) 655 385**	**329 001**	**323 752**
Menos de 1 ano	13 966	7 127	6 839
1 ano	15 044	7 652	7 392
2 anos	14 639	7 465	7 174
3 anos	15 086	7 783	7 303
4 anos	16 002	8 279	7 723
5 anos	15 389	7 870	7 519
6 anos	16 287	8 278	8 009
7 anos	17 225	8 833	8 392
8 anos	16 110	8 336	7 774
9 anos	16 275	8 234	8 041
10 anos	16 211	8 262	7 949
11 anos	15 738	7 898	7 840
12 anos	15 761	7 998	7 763
13 anos	14 720	7 398	7 322
14 anos	14 244	7 062	7 182
15 anos	13 959	6 924	7 035
16 anos	14 046	6 919	7 127
17 anos	13 958	7 085	6 873
18 anos	14 075	7 101	6 974
19 anos	12 756	6 360	6 396
20 anos	13 164	6 583	6 581
21 anos	12 912	6 414	6 498
22 anos	13 331	6 608	6 723
23 anos	12 923	6 565	6 358
24 anos	13 276	6 618	6 658
25 anos	12 723	6 344	6 379
26 anos	12 963	6 492	6 471
27 anos	12 533	6 260	6 273
28 anos	11 074	5 447	5 627
29 anos	10 849	5 281	5 568
30 anos	10 608	5 230	5 378
31 anos	9 509	4 710	4 799
32 anos	9 633	4 788	4 845
33 anos	9 046	4 489	4 557
34 anos	8 809	4 348	4 461
35 anos	8 300	4 150	4 150
36 anos	8 940	4 452	4 488
37 anos	8 275	4 219	4 056
38 anos	7 667	3 784	3 883

Tabela 1.2.2 - População recenseada, por sexo, segundo a idade - Acre - 2007

(conclusão)

Idade	População recenseada, por sexo		
	Total	Homens	Mulheres
39 anos	7 353	3 629	3 724
40 anos	7 927	3 990	3 937
41 anos	6 733	3 428	3 305
42 anos	6 805	3 448	3 357
43 anos	5 968	2 982	2 986
44 anos	6 072	3 057	3 015
45 anos	5 667	2 858	2 809
46 anos	5 645	2 814	2 831
47 anos	5 313	2 693	2 620
48 anos	5 047	2 538	2 509
49 anos	4 895	2 519	2 376
50 anos	4 659	2 386	2 273
51 anos	4 433	2 261	2 172
52 anos	4 419	2 294	2 125
53 anos	4 061	2 058	2 003
54 anos	4 043	2 010	2 033
55 anos	3 638	1 766	1 872
56 anos	3 821	1 913	1 908
57 anos	3 348	1 695	1 653
58 anos	2 913	1 498	1 415
59 anos	3 034	1 552	1 482
60 anos	2 822	1 452	1 370
61 anos	2 494	1 268	1 226
62 anos	2 739	1 344	1 395
63 anos	2 315	1 201	1 114
64 anos	2 341	1 188	1 153
65 anos	2 050	1 017	1 033
66 anos	2 340	1 172	1 168
67 anos	2 091	1 074	1 017
68 anos	1 680	871	809
69 anos	1 464	739	725
70 anos	1 481	733	748
71 anos	1 348	714	634
72 anos	1 284	673	611
73 anos	1 188	637	551
74 anos	1 201	629	572
75 anos	945	489	456
76 anos	1 225	643	582
77 anos	1 075	572	503
78 anos	880	465	415
79 anos	721	387	334
80 anos ou mais	4 980	2 566	2 414
Idade ignorada	269	132	137

Fonte: IBGE, Contagem da População 2007.

(1) Inclusive a população estimada nos domicílios fechados.

Tabela 1.2.3 - População recenseada, por sexo, segundo a idade - Amazonas - 2007

(continua)

Idade	População recenseada, por sexo		
	Total	Homens	Mulheres
**Total**	**(1) 3 221 939**	**1 592 067**	**1 565 850**
Menos de 1 ano	65 844	33 419	32 425
1 ano	73 402	37 238	36 164
2 anos	71 980	36 584	35 396
3 anos	74 269	37 925	36 344
4 anos	74 791	38 238	36 553
5 anos	74 287	37 933	36 354
6 anos	79 326	40 634	38 692
7 anos	80 570	40 889	39 681
8 anos	75 699	38 363	37 336
9 anos	74 618	37 809	36 809
10 anos	77 528	39 673	37 855
11 anos	73 551	37 279	36 272
12 anos	74 881	38 011	36 870
13 anos	72 117	36 418	35 699
14 anos	69 308	34 941	34 367
15 anos	68 981	34 430	34 551
16 anos	64 990	32 760	32 230
17 anos	66 397	33 320	33 077
18 anos	66 148	33 680	32 468
19 anos	64 368	31 886	32 482
20 anos	66 030	32 971	33 059
21 anos	62 723	31 504	31 219
22 anos	66 218	33 221	32 997
23 anos	62 010	31 230	30 780
24 anos	63 745	31 712	32 033
25 anos	62 740	31 300	31 440
26 anos	61 013	30 384	30 629
27 anos	61 035	30 140	30 895
28 anos	55 373	27 511	27 862
29 anos	54 520	27 255	27 265
30 anos	54 966	27 723	27 243
31 anos	47 145	23 255	23 890
32 anos	48 168	23 874	24 294
33 anos	44 405	22 120	22 285
34 anos	43 193	21 454	21 739
35 anos	42 378	21 203	21 175
36 anos	42 260	21 444	20 816
37 anos	40 776	20 603	20 173
38 anos	38 276	19 424	18 852

Tabela 1.2.3 - População recenseada, por sexo, segundo a idade - Amazonas - 2007

(conclusão)

Idade	População recenseada, por sexo		
	Total	Homens	Mulheres
39 anos	37 020	18 782	18 238
40 anos	39 300	20 270	19 030
41 anos	31 851	16 268	15 583
42 anos	34 951	17 909	17 042
43 anos	31 272	15 969	15 303
44 anos	30 848	15 770	15 078
45 anos	29 819	15 309	14 510
46 anos	27 832	14 252	13 580
47 anos	26 437	13 374	13 063
48 anos	25 030	12 921	12 109
49 anos	24 129	12 263	11 866
50 anos	24 435	12 579	11 856
51 anos	20 583	10 479	10 104
52 anos	20 570	10 526	10 044
53 anos	20 730	10 525	10 205
54 anos	19 147	9 597	9 550
55 anos	17 296	8 652	8 644
56 anos	17 597	8 961	8 636
57 anos	15 985	8 126	7 859
58 anos	14 269	7 238	7 031
59 anos	13 070	6 604	6 466
60 anos	13 345	6 814	6 531
61 anos	10 688	5 321	5 367
62 anos	11 971	5 975	5 996
63 anos	10 667	5 256	5 411
64 anos	10 441	5 181	5 260
65 anos	9 684	4 941	4 743
66 anos	9 871	4 985	4 886
67 anos	9 634	4 834	4 800
68 anos	7 784	3 678	4 106
69 anos	7 277	3 548	3 729
70 anos	7 436	3 673	3 763
71 anos	5 963	2 891	3 072
72 anos	5 919	2 860	3 059
73 anos	5 728	2 799	2 929
74 anos	5 281	2 636	2 645
75 anos	4 627	2 307	2 320
76 anos	5 187	2 594	2 593
77 anos	4 383	2 146	2 237
78 anos	3 731	1 774	1 957
79 anos	3 260	1 584	1 676
80 anos ou mais	21 989	9 720	12 269
Idade ignorada	851	418	433

Fonte: IBGE, Contagem da População 2007.

(1) Inclusive a população estimada nos domicílios fechados.

Tabela 1.2.4 - População recenseada, por sexo, segundo a idade - Roraima - 2007

(continua)

Idade	População recenseada, por sexo		
	Total	Homens	Mulheres
**Total**	**(1) 395 725**	**195 275**	**189 046**
Menos de 1 ano	7 241	3 713	3 528
1 ano	8 373	4 248	4 125
2 anos	8 480	4 352	4 128
3 anos	8 847	4 492	4 355
4 anos	8 877	4 578	4 299
5 anos	8 810	4 480	4 330
6 anos	9 453	4 833	4 620
7 anos	9 758	4 855	4 903
8 anos	9 212	4 752	4 460
9 anos	9 271	4 675	4 596
10 anos	9 438	4 810	4 628
11 anos	9 054	4 584	4 470
12 anos	9 377	4 793	4 584
13 anos	8 602	4 364	4 238
14 anos	8 355	4 163	4 192
15 anos	8 379	4 210	4 169
16 anos	8 024	4 118	3 906
17 anos	7 778	3 921	3 857
18 anos	8 033	4 153	3 880
19 anos	7 791	3 914	3 877
20 anos	7 940	4 003	3 937
21 anos	7 678	3 747	3 931
22 anos	7 928	4 036	3 892
23 anos	7 873	3 901	3 972
24 anos	7 896	3 956	3 940
25 anos	7 656	3 686	3 970
26 anos	7 639	3 763	3 876
27 anos	7 321	3 599	3 722
28 anos	6 677	3 311	3 366
29 anos	6 519	3 186	3 333
30 anos	6 638	3 284	3 354
31 anos	5 932	2 863	3 069
32 anos	5 875	2 887	2 988
33 anos	5 395	2 646	2 749
34 anos	5 445	2 727	2 718
35 anos	5 114	2 561	2 553
36 anos	5 309	2 706	2 603
37 anos	5 077	2 520	2 557
38 anos	4 857	2 452	2 405

Tabela 1.2.4 - População recenseada, por sexo, segundo a idade - Roraima - 2007

(conclusão)

Idade	População recenseada, por sexo		
	Total	Homens	Mulheres
39 anos	4 645	2 336	2 309
40 anos	5 153	2 691	2 462
41 anos	4 134	2 119	2 015
42 anos	4 563	2 392	2 171
43 anos	4 300	2 208	2 092
44 anos	3 972	2 084	1 888
45 anos	3 734	1 983	1 751
46 anos	3 821	1 989	1 832
47 anos	3 418	1 782	1 636
48 anos	3 269	1 676	1 593
49 anos	3 213	1 652	1 561
50 anos	3 109	1 653	1 456
51 anos	2 622	1 387	1 235
52 anos	2 672	1 445	1 227
53 anos	2 549	1 326	1 223
54 anos	2 459	1 333	1 126
55 anos	2 224	1 206	1 018
56 anos	2 248	1 242	1 006
57 anos	1 975	1 067	908
58 anos	1 674	898	776
59 anos	1 675	910	765
60 anos	1 656	936	720
61 anos	1 278	708	570
62 anos	1 420	729	691
63 anos	1 088	569	519
64 anos	1 149	649	500
65 anos	1 013	551	462
66 anos	1 096	597	499
67 anos	1 023	549	474
68 anos	787	417	370
69 anos	792	432	360
70 anos	735	393	342
71 anos	665	354	311
72 anos	598	323	275
73 anos	553	282	271
74 anos	546	292	254
75 anos	460	237	223
76 anos	557	311	246
77 anos	425	211	214
78 anos	379	199	180
79 anos	286	142	144
80 anos ou mais	2 141	1 009	1 132
Idade ignorada	323	164	159

Fonte: IBGE, Contagem da População 2007.
(1) Inclusive a população estimada nos domicílios fechados.

Tabela 1.2.5 - População recenseada, por sexo, segundo a idade - Pará - 2007

(continua)

Idade	População recenseada, por sexo		
	Total	Homens	Mulheres
**Total**	**(1) 4 701 695**	**2 405 662**	**2 241 306**
Menos de 1 ano	95 305	48 458	46 847
1 ano	106 420	54 327	52 093
2 anos	103 244	52 463	50 781
3 anos	108 464	54 956	53 508
4 anos	109 222	55 858	53 364
5 anos	110 189	56 136	54 053
6 anos	114 461	58 497	55 964
7 anos	118 687	60 432	58 255
8 anos	110 784	56 799	53 985
9 anos	112 957	57 238	55 719
10 anos	118 223	60 821	57 402
11 anos	112 734	57 686	55 048
12 anos	113 523	58 088	55 435
13 anos	110 185	56 231	53 954
14 anos	108 175	54 634	53 541
15 anos	107 271	54 382	52 889
16 anos	103 818	53 047	50 771
17 anos	103 149	52 852	50 297
18 anos	104 057	54 155	49 902
19 anos	98 430	50 309	48 121
20 anos	97 882	50 322	47 560
21 anos	93 005	48 002	45 003
22 anos	95 213	49 395	45 818
23 anos	91 650	47 317	44 333
24 anos	92 635	48 229	44 406
25 anos	88 738	45 590	43 148
26 anos	86 452	44 864	41 588
27 anos	85 361	44 206	41 155
28 anos	75 157	38 647	36 510
29 anos	75 061	38 597	36 464
30 anos	73 537	38 364	35 173
31 anos	65 210	33 495	31 715
32 anos	67 386	34 839	32 547
33 anos	62 002	32 185	29 817
34 anos	61 518	31 951	29 567
35 anos	57 913	30 191	27 722
36 anos	61 265	32 029	29 236
37 anos	57 965	30 265	27 700
38 anos	54 416	28 395	26 021

Tabela 1.2.5 - População recenseada, por sexo, segundo a idade - Pará - 2007

(conclusão)

Idade	População recenseada, por sexo		
	Total	Homens	Mulheres
39 anos	51 451	27 097	24 354
40 anos	55 237	29 720	25 517
41 anos	43 958	23 070	20 888
42 anos	47 389	25 410	21 979
43 anos	44 973	23 677	21 296
44 anos	42 402	22 626	19 776
45 anos	41 027	22 001	19 026
46 anos	40 011	21 376	18 635
47 anos	38 281	20 398	17 883
48 anos	35 745	18 955	16 790
49 anos	35 211	18 761	16 450
50 anos	34 648	18 619	16 029
51 anos	30 444	16 249	14 195
52 anos	30 641	16 613	14 028
53 anos	29 950	16 096	13 854
54 anos	29 006	15 247	13 759
55 anos	27 314	14 254	13 060
56 anos	27 096	14 575	12 521
57 anos	24 289	13 154	11 135
58 anos	21 205	11 503	9 702
59 anos	21 169	11 508	9 661
60 anos	21 278	11 670	9 608
61 anos	17 511	9 424	8 087
62 anos	18 836	10 157	8 679
63 anos	17 945	9 632	8 313
64 anos	17 726	9 678	8 048
65 anos	16 425	8 787	7 638
66 anos	17 045	9 186	7 859
67 anos	16 030	8 648	7 382
68 anos	12 394	6 581	5 813
69 anos	11 268	5 856	5 412
70 anos	11 633	6 185	5 448
71 anos	9 934	5 411	4 523
72 anos	9 601	5 136	4 465
73 anos	8 928	4 820	4 108
74 anos	8 902	4 750	4 152
75 anos	7 818	4 230	3 588
76 anos	8 481	4 479	4 002
77 anos	6 895	3 686	3 209
78 anos	5 706	3 021	2 685
79 anos	4 733	2 461	2 272
80 anos ou mais	33 924	16 292	17 632
Idade ignorada	844	411	433

Fonte: IBGE, Contagem da População 2007.

(1) Inclusive a população estimada nos domicílios fechados.

Tabela 1.2.6 - População recenseada, por sexo, segundo a idade - Amapá - 2007

(continua)

Idade	População recenseada, por sexo		
	Total	Homens	Mulheres
**Total**	**(1) 587 311**	**292 024**	**290 337**
Menos de 1 ano	12 205	6 184	6 021
1 ano	12 973	6 708	6 265
2 anos	12 433	6 374	6 059
3 anos	13 422	6 732	6 690
4 anos	13 659	6 968	6 691
5 anos	13 793	7 095	6 698
6 anos	14 675	7 570	7 105
7 anos	15 284	7 781	7 503
8 anos	14 345	7 300	7 045
9 anos	14 331	7 281	7 050
10 anos	15 142	7 648	7 494
11 anos	14 048	7 011	7 037
12 anos	14 000	7 083	6 917
13 anos	13 797	6 942	6 855
14 anos	12 955	6 594	6 361
15 anos	13 028	6 427	6 601
16 anos	12 297	6 109	6 188
17 anos	12 880	6 396	6 484
18 anos	13 196	6 696	6 500
19 anos	12 184	5 992	6 192
20 anos	12 294	5 955	6 339
21 anos	12 040	5 824	6 216
22 anos	12 498	6 233	6 265
23 anos	12 059	5 995	6 064
24 anos	12 260	5 970	6 290
25 anos	11 761	5 812	5 949
26 anos	11 141	5 462	5 679
27 anos	11 134	5 467	5 667
28 anos	10 303	4 950	5 353
29 anos	10 073	4 869	5 204
30 anos	10 051	4 881	5 170
31 anos	9 214	4 419	4 795
32 anos	9 416	4 654	4 762
33 anos	8 983	4 432	4 551
34 anos	8 358	4 224	4 134
35 anos	7 965	4 014	3 951
36 anos	8 290	4 110	4 180
37 anos	7 664	3 899	3 765
38 anos	7 178	3 482	3 696

Tabela 1.2.6 - População recenseada, por sexo, segundo a idade - Amapá - 2007

(conclusão)

Idade	População recenseada, por sexo		
	Total	Homens	Mulheres
39 anos	6 816	3 541	3 275
40 anos	7 252	3 677	3 575
41 anos	6 025	3 060	2 965
42 anos	6 492	3 341	3 151
43 anos	5 712	2 937	2 775
44 anos	5 207	2 694	2 513
45 anos	5 225	2 669	2 556
46 anos	5 031	2 558	2 473
47 anos	4 589	2 413	2 176
48 anos	4 420	2 312	2 108
49 anos	4 113	2 042	2 071
50 anos	4 151	2 096	2 055
51 anos	3 479	1 794	1 685
52 anos	3 579	1 892	1 687
53 anos	3 364	1 732	1 632
54 anos	3 200	1 600	1 600
55 anos	2 974	1 522	1 452
56 anos	2 804	1 450	1 354
57 anos	2 413	1 256	1 157
58 anos	2 140	1 092	1 048
59 anos	2 113	1 059	1 054
60 anos	2 037	1 012	1 025
61 anos	1 718	871	847
62 anos	1 827	918	909
63 anos	1 826	924	902
64 anos	1 795	927	868
65 anos	1 639	833	806
66 anos	1 575	798	777
67 anos	1 554	772	782
68 anos	1 214	631	583
69 anos	1 087	524	563
70 anos	1 090	553	537
71 anos	921	479	442
72 anos	913	440	473
73 anos	851	433	418
74 anos	830	382	448
75 anos	748	356	392
76 anos	778	371	407
77 anos	643	295	348
78 anos	547	260	287
79 anos	446	211	235
80 anos ou mais	3 266	1 415	1 851
Idade ignorada	628	339	289

Fonte: IBGE, Contagem da População 2007.

(1) Inclusive a população estimada nos domicílios fechados.

Tabela 1.2.7 - População recenseada, por sexo, segundo a idade - Tocantins - 2007

(continua)

Idade	População recenseada, por sexo		
	Total	Homens	Mulheres
**Total**	**(1) 1 243 627**	**626 434**	**602 339**
Menos de 1 ano	22 199	11 255	10 944
1 ano	23 476	11 902	11 574
2 anos	22 383	11 529	10 854
3 anos	23 797	12 207	11 590
4 anos	23 867	12 186	11 681
5 anos	24 371	12 428	11 943
6 anos	25 736	13 162	12 574
7 anos	26 592	13 558	13 034
8 anos	25 615	13 323	12 292
9 anos	26 860	13 753	13 107
10 anos	27 298	14 059	13 239
11 anos	27 225	13 871	13 354
12 anos	26 952	13 692	13 260
13 anos	26 474	13 348	13 126
14 anos	26 039	13 259	12 780
15 anos	25 908	13 108	12 800
16 anos	25 417	12 928	12 489
17 anos	25 799	13 096	12 703
18 anos	25 989	13 305	12 684
19 anos	25 041	12 726	12 315
20 anos	24 933	12 764	12 169
21 anos	24 549	12 351	12 198
22 anos	24 183	12 287	11 896
23 anos	23 364	11 749	11 615
24 anos	23 826	12 097	11 729
25 anos	23 044	11 611	11 433
26 anos	22 394	11 121	11 273
27 anos	22 202	10 925	11 277
28 anos	20 383	10 219	10 164
29 anos	20 775	10 240	10 535
30 anos	20 649	10 272	10 377
31 anos	19 028	9 385	9 643
32 anos	18 913	9 388	9 525
33 anos	17 650	8 909	8 741
34 anos	17 540	8 948	8 592
35 anos	16 287	8 223	8 064
36 anos	16 666	8 364	8 302
37 anos	16 422	8 155	8 267
38 anos	16 382	8 253	8 129

Tabela 1.2.7 - População recenseada, por sexo, segundo a idade - Tocantins - 2007

(conclusão)

Idade	População recenseada, por sexo		
	Total	Homens	Mulheres
39 anos	15 331	7 671	7 660
40 anos	16 300	8 526	7 774
41 anos	14 212	7 167	7 045
42 anos	15 080	7 790	7 290
43 anos	14 257	7 297	6 960
44 anos	13 484	6 930	6 554
45 anos	12 079	6 155	5 924
46 anos	12 256	6 244	6 012
47 anos	11 483	5 963	5 520
48 anos	11 107	5 677	5 430
49 anos	10 873	5 508	5 365
50 anos	10 767	5 546	5 221
51 anos	9 697	4 975	4 722
52 anos	9 764	5 052	4 712
53 anos	9 253	4 787	4 466
54 anos	9 287	4 761	4 526
55 anos	8 638	4 482	4 156
56 anos	8 516	4 453	4 063
57 anos	7 615	3 974	3 641
58 anos	7 130	3 770	3 360
59 anos	7 052	3 696	3 356
60 anos	6 987	3 697	3 290
61 anos	6 102	3 277	2 825
62 anos	6 160	3 220	2 940
63 anos	5 876	3 093	2 783
64 anos	6 077	3 169	2 908
65 anos	4 895	2 614	2 281
66 anos	5 486	2 900	2 586
67 anos	5 601	2 901	2 700
68 anos	4 556	2 455	2 101
69 anos	3 899	2 129	1 770
70 anos	4 249	2 238	2 011
71 anos	3 773	2 000	1 773
72 anos	3 544	1 895	1 649
73 anos	3 151	1 700	1 451
74 anos	3 336	1 809	1 527
75 anos	2 722	1 453	1 269
76 anos	2 821	1 507	1 314
77 anos	2 418	1 291	1 127
78 anos	2 160	1 181	979
79 anos	1 797	943	854
80 anos ou mais	12 482	6 442	6 040
Idade ignorada	272	140	132

Fonte: IBGE, Contagem da População 2007.

(1) Inclusive a população estimada nos domicílios fechados e nos domicílios provenientes de setor censitário cujo arquivo foi danificado.

Tabela 1.2.8 - População recenseada, por sexo, segundo a idade - Maranhão - 2007

(continua)

Idade	População recenseada, por sexo		
	Total	Homens	Mulheres
**Total**	**(1) 6 118 995**	**3 021 226**	**3 052 375**
Menos de 1 ano	118 604	60 000	58 604
1 ano	125 473	63 989	61 484
2 anos	122 788	62 340	60 448
3 anos	128 322	65 377	62 945
4 anos	128 559	65 687	62 872
5 anos	128 774	65 693	63 081
6 anos	138 659	70 465	68 194
7 anos	143 233	73 059	70 174
8 anos	131 966	67 176	64 790
9 anos	136 718	69 298	67 420
10 anos	139 093	70 701	68 392
11 anos	135 931	68 627	67 304
12 anos	135 177	68 123	67 054
13 anos	133 820	66 816	67 004
14 anos	130 404	65 542	64 862
15 anos	135 206	67 440	67 766
16 anos	132 459	66 839	65 620
17 anos	137 270	69 694	67 576
18 anos	139 330	71 197	68 133
19 anos	134 014	67 151	66 863
20 anos	133 180	66 801	66 379
21 anos	126 447	63 537	62 910
22 anos	126 931	64 332	62 599
23 anos	124 220	62 548	61 672
24 anos	121 230	60 654	60 576
25 anos	115 160	57 275	57 885
26 anos	108 524	53 796	54 728
27 anos	109 729	54 298	55 431
28 anos	96 036	47 330	48 706
29 anos	94 152	46 049	48 103
30 anos	93 924	46 898	47 026
31 anos	81 812	39 815	41 997
32 anos	83 103	40 708	42 395
33 anos	75 786	37 448	38 338
34 anos	76 443	37 295	39 148
35 anos	74 320	36 108	38 212
36 anos	76 371	37 220	39 151
37 anos	73 106	35 506	37 600
38 anos	69 474	33 828	35 646

Tabela 1.2.8 - População recenseada, por sexo, segundo a idade - Maranhão - 2007

(conclusão)

Idade	População recenseada, por sexo		
	Total	Homens	Mulheres
39 anos	66 278	31 808	34 470
40 anos	73 130	36 035	37 095
41 anos	58 741	28 303	30 438
42 anos	65 263	31 779	33 484
43 anos	64 627	31 054	33 573
44 anos	60 073	29 219	30 854
45 anos	56 308	27 771	28 537
46 anos	55 175	26 854	28 321
47 anos	51 295	24 790	26 505
48 anos	49 584	23 821	25 763
49 anos	51 776	24 646	27 130
50 anos	49 636	24 305	25 331
51 anos	44 186	21 426	22 760
52 anos	44 295	21 656	22 639
53 anos	43 684	21 223	22 461
54 anos	43 646	20 475	23 171
55 anos	42 097	19 899	22 198
56 anos	41 679	19 790	21 889
57 anos	36 952	17 911	19 041
58 anos	32 473	15 903	16 570
59 anos	33 889	16 913	16 976
60 anos	35 368	17 467	17 901
61 anos	27 358	13 374	13 984
62 anos	29 776	14 591	15 185
63 anos	27 462	13 528	13 934
64 anos	29 157	14 444	14 713
65 anos	26 497	13 125	13 372
66 anos	28 727	14 021	14 706
67 anos	26 769	13 067	13 702
68 anos	21 418	10 331	11 087
69 anos	19 671	9 326	10 345
70 anos	22 380	10 808	11 572
71 anos	18 188	8 909	9 279
72 anos	17 055	8 595	8 460
73 anos	15 578	7 601	7 977
74 anos	17 390	8 725	8 665
75 anos	14 336	7 016	7 320
76 anos	14 708	7 264	7 444
77 anos	11 919	5 795	6 124
78 anos	10 196	5 101	5 095
79 anos	8 608	4 047	4 561
80 anos ou mais	68 711	30 953	37 758
Idade ignorada	1 794	897	897

Fonte: IBGE, Contagem da População 2007.
(1) Inclusive a população estimada nos domicílios fechados.

Tabela 1.2.9 - População recenseada, por sexo, segundo a idade - Piauí - 2007

(continua)

Idade	População recenseada, por sexo		
	Total	Homens	Mulheres
**Total**	**(1) 3 032 421**	**1 481 576**	**1 529 053**
Menos de 1 ano	48 364	24 594	23 770
1 ano	51 912	26 404	25 508
2 anos	49 849	25 656	24 193
3 anos	51 622	26 365	25 257
4 anos	54 129	27 520	26 609
5 anos	55 329	28 187	27 142
6 anos	58 773	29 887	28 886
7 anos	59 159	30 201	28 958
8 anos	58 263	29 918	28 345
9 anos	61 318	31 199	30 119
10 anos	62 688	31 828	30 860
11 anos	62 711	31 946	30 765
12 anos	61 913	31 197	30 716
13 anos	58 768	29 808	28 960
14 anos	60 070	30 075	29 995
15 anos	62 291	31 272	31 019
16 anos	61 125	30 783	30 342
17 anos	67 117	34 091	33 026
18 anos	67 876	34 371	33 505
19 anos	64 564	32 095	32 469
20 anos	65 776	32 769	33 007
21 anos	63 356	31 632	31 724
22 anos	61 774	30 920	30 854
23 anos	58 929	29 382	29 547
24 anos	59 502	29 594	29 908
25 anos	55 179	27 303	27 876
26 anos	52 297	25 812	26 485
27 anos	53 691	26 488	27 203
28 anos	49 470	24 071	25 399
29 anos	48 366	23 382	24 984
30 anos	48 126	23 676	24 450
31 anos	43 625	21 103	22 522
32 anos	43 724	21 319	22 405
33 anos	40 625	19 714	20 911
34 anos	40 714	19 685	21 029
35 anos	38 544	18 630	19 914
36 anos	38 873	18 708	20 165
37 anos	38 772	18 625	20 147
38 anos	38 490	18 452	20 038

Tabela 1.2.9 - População recenseada, por sexo, segundo a idade - Piauí - 2007

(conclusão)

Idade	População recenseada, por sexo		
	Total	Homens	Mulheres
39 anos	35 961	17 314	18 647
40 anos	39 702	19 144	20 558
41 anos	34 423	16 401	18 022
42 anos	37 273	18 161	19 112
43 anos	36 461	17 411	19 050
44 anos	34 150	16 416	17 734
45 anos	31 073	14 978	16 095
46 anos	30 714	14 576	16 138
47 anos	28 906	13 735	15 171
48 anos	29 057	13 708	15 349
49 anos	28 691	13 607	15 084
50 anos	27 516	13 137	14 379
51 anos	24 793	11 726	13 067
52 anos	24 794	11 780	13 014
53 anos	24 572	11 675	12 897
54 anos	24 433	11 368	13 065
55 anos	24 663	11 407	13 256
56 anos	23 430	10 921	12 509
57 anos	21 391	10 103	11 288
58 anos	19 621	9 339	10 282
59 anos	20 321	9 728	10 593
60 anos	19 575	9 348	10 227
61 anos	16 726	8 069	8 657
62 anos	17 658	8 467	9 191
63 anos	16 592	7 833	8 759
64 anos	16 695	8 023	8 672
65 anos	14 348	7 026	7 322
66 anos	15 382	7 303	8 079
67 anos	15 655	7 432	8 223
68 anos	12 492	5 868	6 624
69 anos	11 755	5 447	6 308
70 anos	12 791	5 859	6 932
71 anos	11 164	5 127	6 037
72 anos	9 888	4 680	5 208
73 anos	7 767	3 601	4 166
74 anos	10 805	4 881	5 924
75 anos	9 018	4 183	4 835
76 anos	8 488	3 971	4 517
77 anos	7 561	3 527	4 034
78 anos	6 813	3 148	3 665
79 anos	6 198	2 838	3 360
80 anos ou mais	43 039	19 331	23 708
Idade ignorada	600	317	283

Fonte: IBGE, Contagem da População 2007.

(1) Inclusive a população estimada nos domicílios fechados.

Tabela 1.2.10 - População recenseada, por sexo, segundo a idade - Ceará - 2007

(continua)

Idade	População recenseada, por sexo		
	Total	Homens	Mulheres
**Total**	**(1) 4 820 630**	**2 398 046**	**2 404 540**
Menos de 1 ano	75 657	38 803	36 854
1 ano	80 777	41 123	39 654
2 anos	77 513	39 474	38 039
3 anos	80 496	40 732	39 764
4 anos	85 389	43 503	41 886
5 anos	90 080	45 942	44 138
6 anos	96 116	48 970	47 146
7 anos	100 428	51 196	49 232
8 anos	97 510	50 131	47 379
9 anos	103 743	52 695	51 048
10 anos	109 404	55 835	53 569
11 anos	113 661	57 972	55 689
12 anos	111 264	56 692	54 572
13 anos	104 704	52 926	51 778
14 anos	107 373	54 717	52 656
15 anos	104 680	52 985	51 695
16 anos	99 989	50 896	49 093
17 anos	105 250	53 865	51 385
18 anos	106 516	54 716	51 800
19 anos	98 497	49 762	48 735
20 anos	97 450	49 133	48 317
21 anos	95 025	48 299	46 726
22 anos	87 349	44 930	42 419
23 anos	82 998	42 436	40 562
24 anos	86 534	43 840	42 694
25 anos	82 364	41 774	40 590
26 anos	78 111	39 429	38 682
27 anos	76 693	38 724	37 969
28 anos	72 001	35 984	36 017
29 anos	71 602	35 587	36 015
30 anos	71 318	35 897	35 421
31 anos	64 587	31 998	32 589
32 anos	68 138	34 194	33 944
33 anos	64 907	32 492	32 415
34 anos	62 877	31 146	31 731
35 anos	58 069	28 770	29 299
36 anos	60 752	29 888	30 864
37 anos	61 478	30 267	31 211
38 anos	59 799	29 555	30 244

Tabela 1.2.10 - População recenseada, por sexo, segundo a idade - Ceará - 2007

(conclusão)

Idade	População recenseada, por sexo		
	Total	Homens	Mulheres
39 anos	57 322	28 126	29 196
40 anos	62 359	30 919	31 440
41 anos	53 142	26 147	26 995
42 anos	58 487	29 098	29 389
43 anos	57 237	28 065	29 172
44 anos	53 164	26 239	26 925
45 anos	50 005	25 144	24 861
46 anos	46 585	22 832	23 753
47 anos	41 677	20 384	21 293
48 anos	40 668	19 624	21 044
49 anos	41 810	19 897	21 913
50 anos	41 553	20 054	21 499
51 anos	38 398	18 473	19 925
52 anos	36 455	17 561	18 894
53 anos	36 358	17 602	18 756
54 anos	36 463	17 284	19 179
55 anos	38 086	17 514	20 572
56 anos	37 690	17 762	19 928
57 anos	35 639	16 783	18 856
58 anos	32 597	15 728	16 869
59 anos	33 434	16 323	17 111
60 anos	32 961	16 102	16 859
61 anos	27 343	13 247	14 096
62 anos	28 193	13 527	14 666
63 anos	26 354	12 644	13 710
64 anos	28 105	13 597	14 508
65 anos	26 943	13 317	13 626
66 anos	27 379	13 130	14 249
67 anos	27 422	12 992	14 430
68 anos	22 949	10 892	12 057
69 anos	20 697	9 724	10 973
70 anos	23 016	10 863	12 153
71 anos	20 259	9 456	10 803
72 anos	18 860	8 900	9 960
73 anos	14 483	6 919	7 564
74 anos	16 024	7 430	8 594
75 anos	15 782	7 430	8 352
76 anos	15 149	7 103	8 046
77 anos	14 586	6 968	7 618
78 anos	13 453	6 444	7 009
79 anos	12 281	5 765	6 516
80 anos ou mais	89 133	42 245	46 888
Idade ignorada	986	514	472

Fonte: IBGE, Contagem da População 2007.

(1) Inclusive a população estimada nos domicílios fechados.

Tabela 1.2.11 - População recenseada, por sexo, segundo a idade - Rio Grande do Norte - 2007

(continua)

Idade	População recenseada, por sexo		
	Total	Homens	Mulheres
**Total**	**(1) 3 013 740**	**1 457 143**	**1 517 826**
Menos de 1 ano	44 368	22 645	21 723
1 ano	47 741	24 314	23 427
2 anos	46 821	23 748	23 073
3 anos	48 048	24 637	23 411
4 anos	48 927	25 002	23 925
5 anos	50 689	25 797	24 892
6 anos	53 863	27 388	26 475
7 anos	56 012	28 802	27 210
8 anos	54 170	27 616	26 554
9 anos	55 982	28 355	27 627
10 anos	58 128	29 704	28 424
11 anos	58 840	29 866	28 974
12 anos	60 941	31 010	29 931
13 anos	57 942	29 019	28 923
14 anos	57 267	28 973	28 294
15 anos	55 578	27 908	27 670
16 anos	55 411	27 874	27 537
17 anos	59 610	29 899	29 711
18 anos	63 988	32 394	31 594
19 anos	61 756	30 736	31 020
20 anos	63 044	31 647	31 397
21 anos	60 719	30 375	30 344
22 anos	57 783	29 079	28 704
23 anos	57 212	28 686	28 526
24 anos	58 466	29 253	29 213
25 anos	58 021	28 755	29 266
26 anos	54 105	26 954	27 151
27 anos	52 361	26 048	26 313
28 anos	49 769	24 324	25 445
29 anos	47 269	23 307	23 962
30 anos	48 607	24 229	24 378
31 anos	42 420	20 348	22 072
32 anos	46 887	22 812	24 075
33 anos	44 963	22 001	22 962
34 anos	42 857	20 698	22 159
35 anos	40 620	19 801	20 819
36 anos	41 827	20 216	21 611
37 anos	42 307	20 524	21 783
38 anos	42 525	20 688	21 837

Tabela 1.2.11 - População recenseada, por sexo, segundo a idade - Rio Grande do Norte - 2007

(conclusão)

Idade	População recenseada, por sexo		
	Total	Homens	Mulheres
39 anos	41 604	19 998	21 606
40 anos	44 085	21 384	22 701
41 anos	37 387	17 993	19 394
42 anos	42 997	20 861	22 136
43 anos	38 888	18 532	20 356
44 anos	36 953	17 677	19 276
45 anos	34 984	16 880	18 104
46 anos	33 665	16 210	17 455
47 anos	29 832	14 193	15 639
48 anos	27 720	13 303	14 417
49 anos	28 916	13 503	15 413
50 anos	29 238	13 709	15 529
51 anos	24 738	11 505	13 233
52 anos	24 729	11 633	13 096
53 anos	22 911	10 737	12 174
54 anos	22 597	10 375	12 222
55 anos	21 642	9 953	11 689
56 anos	23 649	10 809	12 840
57 anos	22 709	10 477	12 232
58 anos	20 244	9 297	10 947
59 anos	20 673	9 578	11 095
60 anos	19 880	9 304	10 576
61 anos	17 143	7 919	9 224
62 anos	16 721	7 651	9 070
63 anos	14 337	6 568	7 769
64 anos	15 404	6 921	8 483
65 anos	16 192	7 414	8 778
66 anos	16 016	7 234	8 782
67 anos	15 117	6 796	8 321
68 anos	12 390	5 566	6 824
69 anos	11 664	5 173	6 491
70 anos	12 224	5 405	6 819
71 anos	10 797	4 792	6 005
72 anos	10 592	4 720	5 872
73 anos	8 713	3 851	4 862
74 anos	7 920	3 524	4 396
75 anos	7 865	3 367	4 498
76 anos	8 659	3 720	4 939
77 anos	8 237	3 704	4 533
78 anos	7 149	3 157	3 992
79 anos	6 775	3 005	3 770
80 anos ou mais	52 722	23 104	29 618
Idade ignorada	447	209	238

Fonte: IBGE, Contagem da População 2007.
(1) Inclusive a população estimada nos domicílios fechados.

Tabela 1.2.12 - População recenseada, por sexo, segundo a idade - Paraíba - 2007

(continua)

Idade	População recenseada, por sexo		
	Total	Homens	Mulheres
**Total**	**(1) 3 641 395**	**1 754 525**	**1 855 119**
Menos de 1 ano	55 344	28 222	27 122
1 ano	58 393	29 564	28 829
2 anos	57 806	29 510	28 296
3 anos	59 601	30 447	29 154
4 anos	61 928	31 491	30 437
5 anos	64 091	32 712	31 379
6 anos	65 529	33 456	32 073
7 anos	66 614	34 009	32 605
8 anos	64 372	32 660	31 712
9 anos	68 013	34 527	33 486
10 anos	70 589	36 147	34 442
11 anos	72 889	37 095	35 794
12 anos	72 673	36 748	35 925
13 anos	69 209	35 091	34 118
14 anos	70 388	35 427	34 961
15 anos	71 754	36 116	35 638
16 anos	70 292	35 579	34 713
17 anos	72 093	36 585	35 508
18 anos	74 994	38 079	36 915
19 anos	72 266	35 901	36 365
20 anos	73 534	36 342	37 192
21 anos	71 451	35 813	35 638
22 anos	68 639	34 325	34 314
23 anos	67 189	33 677	33 512
24 anos	68 043	33 843	34 200
25 anos	67 674	33 488	34 186
26 anos	63 296	31 298	31 998
27 anos	62 101	30 506	31 595
28 anos	59 839	29 065	30 774
29 anos	55 570	26 869	28 701
30 anos	58 113	28 300	29 813
31 anos	52 413	25 108	27 305
32 anos	55 204	26 540	28 664
33 anos	52 229	25 268	26 961
34 anos	50 901	24 653	26 248
35 anos	47 651	22 910	24 741
36 anos	48 635	23 330	25 305
37 anos	48 905	23 385	25 520
38 anos	48 100	22 966	25 134

Tabela 1.2.12 - População recenseada, por sexo, segundo a idade - Paraíba - 2007

(conclusão)

Idade	População recenseada, por sexo		
	Total	Homens	Mulheres
39 anos	47 063	22 348	24 715
40 anos	50 132	23 929	26 203
41 anos	43 242	20 449	22 793
42 anos	48 329	23 588	24 741
43 anos	45 092	21 507	23 585
44 anos	41 849	19 971	21 878
45 anos	39 645	19 082	20 563
46 anos	38 230	18 101	20 129
47 anos	35 181	16 544	18 637
48 anos	33 312	15 512	17 800
49 anos	33 754	15 763	17 991
50 anos	33 741	16 041	17 700
51 anos	30 223	14 051	16 172
52 anos	30 806	14 187	16 619
53 anos	30 308	13 963	16 345
54 anos	29 944	13 547	16 397
55 anos	28 036	12 651	15 385
56 anos	29 516	13 176	16 340
57 anos	27 980	12 599	15 381
58 anos	26 056	11 889	14 167
59 anos	25 973	11 789	14 184
60 anos	25 001	11 332	13 669
61 anos	21 897	10 051	11 846
62 anos	22 474	10 061	12 413
63 anos	20 091	8 969	11 122
64 anos	20 929	9 412	11 517
65 anos	20 753	9 417	11 336
66 anos	22 259	9 731	12 528
67 anos	20 520	8 997	11 523
68 anos	17 255	7 533	9 722
69 anos	16 456	7 242	9 214
70 anos	17 514	7 399	10 115
71 anos	15 282	6 672	8 610
72 anos	13 939	6 068	7 871
73 anos	11 748	5 170	6 578
74 anos	11 404	4 833	6 571
75 anos	11 504	4 984	6 520
76 anos	12 595	5 204	7 391
77 anos	11 572	5 036	6 536
78 anos	9 524	4 057	5 467
79 anos	8 899	3 849	5 050
80 anos ou mais	70 527	30 367	40 160
Idade ignorada	764	402	362

Fonte: IBGE, Contagem da População 2007.
(1) Inclusive a população estimada nos domicílios fechados.

Tabela 1.2.13 - População recenseada, por sexo, segundo a idade - Pernambuco - 2007

(continua)

Idade	População recenseada, por sexo		
	Total	Homens	Mulheres
**Total**	**(1) 5 030 277**	**2 452 542**	**2 546 413**
Menos de 1 ano	79 270	40 414	38 856
1 ano	86 939	44 412	42 527
2 anos	86 133	43 378	42 755
3 anos	90 220	45 675	44 545
4 anos	92 867	47 387	45 480
5 anos	97 297	49 537	47 760
6 anos	101 126	51 792	49 334
7 anos	104 120	52 940	51 180
8 anos	98 783	50 306	48 477
9 anos	102 525	51 586	50 939
10 anos	105 891	53 715	52 176
11 anos	106 799	54 039	52 760
12 anos	104 100	52 734	51 366
13 anos	97 792	49 064	48 728
14 anos	102 273	51 405	50 868
15 anos	103 974	51 945	52 029
16 anos	102 014	51 623	50 391
17 anos	104 248	52 834	51 414
18 anos	107 066	54 257	52 809
19 anos	100 233	50 008	50 225
20 anos	101 960	50 955	51 005
21 anos	97 679	48 856	48 823
22 anos	95 220	48 230	46 990
23 anos	94 832	47 580	47 252
24 anos	94 076	47 139	46 937
25 anos	93 759	46 827	46 932
26 anos	87 270	43 405	43 865
27 anos	84 904	42 266	42 638
28 anos	81 564	40 033	41 531
29 anos	78 447	38 334	40 113
30 anos	82 395	40 643	41 752
31 anos	71 050	34 437	36 613
32 anos	76 634	37 430	39 204
33 anos	70 843	34 303	36 540
34 anos	69 305	33 472	35 833
35 anos	65 162	31 380	33 782
36 anos	65 820	31 499	34 321
37 anos	66 111	31 502	34 609
38 anos	65 080	31 278	33 802

Tabela 1.2.13 - População recenseada, por sexo, segundo a idade - Pernambuco - 2007

(conclusão)

Idade	População recenseada, por sexo		
	Total	Homens	Mulheres
39 anos	60 781	29 160	31 621
40 anos	67 160	32 313	34 847
41 anos	54 879	26 193	28 686
42 anos	63 664	30 480	33 184
43 anos	57 845	27 713	30 132
44 anos	52 677	25 393	27 284
45 anos	52 191	25 348	26 843
46 anos	49 559	23 366	26 193
47 anos	48 050	22 966	25 084
48 anos	44 778	21 230	23 548
49 anos	43 427	20 475	22 952
50 anos	42 543	20 249	22 294
51 anos	39 236	18 268	20 968
52 anos	41 403	19 492	21 911
53 anos	39 076	18 188	20 888
54 anos	38 709	17 623	21 086
55 anos	36 570	16 450	20 120
56 anos	38 842	17 616	21 226
57 anos	35 889	16 502	19 387
58 anos	33 951	15 768	18 183
59 anos	33 889	16 246	17 643
60 anos	34 367	16 249	18 118
61 anos	27 929	13 144	14 785
62 anos	30 089	13 914	16 175
63 anos	27 802	12 732	15 070
64 anos	27 196	12 523	14 673
65 anos	27 461	12 758	14 703
66 anos	29 178	13 255	15 923
67 anos	26 983	12 345	14 638
68 anos	21 642	9 854	11 788
69 anos	21 424	9 712	11 712
70 anos	22 388	9 951	12 437
71 anos	17 961	8 198	9 763
72 anos	17 991	8 284	9 707
73 anos	15 026	6 799	8 227
74 anos	15 229	6 884	8 345
75 anos	14 686	6 674	8 012
76 anos	15 861	7 181	8 680
77 anos	13 727	6 209	7 518
78 anos	11 769	5 458	6 311
79 anos	10 115	4 783	5 332
80 anos ou mais	78 018	35 370	42 648
Idade ignorada	1 213	606	607

Fonte: IBGE, Contagem da População 2007.

(1) Inclusive a população estimada nos domicílios fechados.

Tabela 1.2.14 - População recenseada, por sexo, segundo a idade - Alagoas - 2007

(continua)

Idade	População recenseada, por sexo		
	Total	Homens	Mulheres
**Total**	**(1) 3 037 103**	**1 472 429**	**1 545 366**
Menos de 1 ano	51 579	26 117	25 462
1 ano	55 574	28 401	27 173
2 anos	54 855	28 051	26 804
3 anos	57 892	29 323	28 569
4 anos	60 680	30 962	29 718
5 anos	63 375	32 410	30 965
6 anos	65 279	33 295	31 984
7 anos	66 627	33 729	32 898
8 anos	64 037	32 546	31 491
9 anos	66 124	33 520	32 604
10 anos	69 231	35 097	34 134
11 anos	68 874	34 873	34 001
12 anos	67 803	34 220	33 583
13 anos	62 992	31 624	31 368
14 anos	62 895	31 628	31 267
15 anos	62 605	31 147	31 458
16 anos	60 239	30 295	29 944
17 anos	62 508	31 513	30 995
18 anos	64 890	32 634	32 256
19 anos	60 650	30 073	30 577
20 anos	62 377	30 773	31 604
21 anos	60 370	29 484	30 886
22 anos	58 819	29 031	29 788
23 anos	58 353	28 771	29 582
24 anos	58 320	28 649	29 671
25 anos	57 148	27 949	29 199
26 anos	53 381	26 097	27 284
27 anos	51 528	24 995	26 533
28 anos	50 315	24 012	26 303
29 anos	47 889	22 921	24 968
30 anos	51 706	24 679	27 027
31 anos	43 445	20 655	22 790
32 anos	47 246	22 362	24 884
33 anos	42 700	20 501	22 199
34 anos	41 702	19 722	21 980
35 anos	39 232	18 814	20 418
36 anos	40 367	18 977	21 390
37 anos	40 555	19 163	21 392
38 anos	40 667	19 106	21 561

Tabela 1.2.14 - População recenseada, por sexo, segundo a idade - Alagoas - 2007

(conclusão)

Idade	População recenseada, por sexo		
	Total	Homens	Mulheres
39 anos	37 549	17 817	19 732
40 anos	41 378	19 851	21 527
41 anos	31 817	15 051	16 766
42 anos	37 702	18 065	19 637
43 anos	34 929	16 638	18 291
44 anos	31 881	15 232	16 649
45 anos	30 948	15 088	15 860
46 anos	29 633	14 057	15 576
47 anos	28 010	13 083	14 927
48 anos	27 667	12 764	14 903
49 anos	26 022	12 273	13 749
50 anos	26 654	12 672	13 982
51 anos	22 810	10 630	12 180
52 anos	24 844	11 744	13 100
53 anos	23 618	11 159	12 459
54 anos	22 754	10 545	12 209
55 anos	21 527	10 034	11 493
56 anos	21 272	9 887	11 385
57 anos	19 226	9 080	10 146
58 anos	18 168	8 606	9 562
59 anos	18 383	8 773	9 610
60 anos	18 678	8 689	9 989
61 anos	14 097	6 659	7 438
62 anos	15 988	7 467	8 521
63 anos	14 764	6 888	7 876
64 anos	14 134	6 569	7 565
65 anos	14 489	6 729	7 760
66 anos	14 146	6 473	7 673
67 anos	12 645	5 855	6 790
68 anos	10 312	4 602	5 710
69 anos	9 688	4 386	5 302
70 anos	10 351	4 527	5 824
71 anos	8 237	3 671	4 566
72 anos	8 630	3 811	4 819
73 anos	7 563	3 287	4 276
74 anos	7 705	3 415	4 290
75 anos	6 781	2 940	3 841
76 anos	7 277	3 151	4 126
77 anos	5 956	2 653	3 303
78 anos	5 128	2 242	2 886
79 anos	4 319	1 874	2 445
80 anos ou mais	33 812	14 569	19 243
Idade ignorada	1 474	804	670

Fonte: IBGE, Contagem da População 2007.

(1) Inclusive a população estimada nos domicílios fechados e nos domicílios provenientes de setor censitário cujo arquivo foi danificado.

Tabela 1.2.15 - População recenseada, por sexo, segundo a idade - Sergipe - 2007

(continua)

Idade	População recenseada, por sexo		
	Total	Homens	Mulheres
**Total**	**(1) 1 939 426**	**936 306**	**981 208**
Menos de 1 ano	30 913	15 676	15 237
1 ano	33 229	16 771	16 458
2 anos	31 959	16 175	15 784
3 anos	33 166	16 846	16 320
4 anos	33 637	17 150	16 487
5 anos	35 501	18 235	17 266
6 anos	39 010	19 872	19 138
7 anos	40 084	20 518	19 566
8 anos	38 109	19 432	18 677
9 anos	39 199	19 809	19 390
10 anos	40 469	20 704	19 765
11 anos	40 531	20 647	19 884
12 anos	41 343	21 080	20 263
13 anos	39 852	19 908	19 944
14 anos	37 752	18 956	18 796
15 anos	37 454	18 798	18 656
16 anos	36 904	18 430	18 474
17 anos	39 067	19 874	19 193
18 anos	41 440	20 807	20 633
19 anos	39 571	19 718	19 853
20 anos	39 999	19 605	20 394
21 anos	39 380	19 604	19 776
22 anos	37 912	18 865	19 047
23 anos	37 660	18 576	19 084
24 anos	37 942	18 840	19 102
25 anos	37 921	18 691	19 230
26 anos	36 604	17 676	18 928
27 anos	35 233	16 937	18 296
28 anos	32 911	15 879	17 032
29 anos	31 447	15 242	16 205
30 anos	32 454	15 655	16 799
31 anos	28 443	13 503	14 940
32 anos	30 636	14 653	15 983
33 anos	28 970	13 949	15 021
34 anos	28 861	13 837	15 024
35 anos	26 776	12 945	13 831
36 anos	28 191	13 451	14 740
37 anos	27 491	13 133	14 358
38 anos	27 039	12 834	14 205

Tabela 1.2.15 - População recenseada, por sexo, segundo a idade - Sergipe - 2007

(conclusão)

Idade	População recenseada, por sexo		
	Total	Homens	Mulheres
39 anos	25 783	12 421	13 362
40 anos	27 621	13 382	14 239
41 anos	22 803	10 791	12 012
42 anos	26 085	12 506	13 579
43 anos	23 887	11 221	12 666
44 anos	21 864	10 672	11 192
45 anos	20 359	9 935	10 424
46 anos	20 431	9 742	10 689
47 anos	19 048	8 971	10 077
48 anos	17 857	8 493	9 364
49 anos	17 062	8 114	8 948
50 anos	16 781	8 053	8 728
51 anos	14 880	6 955	7 925
52 anos	15 866	7 642	8 224
53 anos	15 369	7 275	8 094
54 anos	14 693	6 818	7 875
55 anos	13 652	6 255	7 397
56 anos	13 703	6 368	7 335
57 anos	12 316	5 817	6 499
58 anos	11 384	5 460	5 924
59 anos	11 831	5 658	6 173
60 anos	11 549	5 401	6 148
61 anos	9 466	4 466	5 000
62 anos	10 043	4 666	5 377
63 anos	9 618	4 410	5 208
64 anos	9 083	4 215	4 868
65 anos	8 687	3 955	4 732
66 anos	8 509	3 848	4 661
67 anos	7 930	3 539	4 391
68 anos	6 701	3 060	3 641
69 anos	5 818	2 504	3 314
70 anos	6 594	2 889	3 705
71 anos	5 586	2 382	3 204
72 anos	5 327	2 389	2 938
73 anos	4 811	2 032	2 779
74 anos	5 289	2 213	3 076
75 anos	4 678	1 971	2 707
76 anos	4 530	1 903	2 627
77 anos	3 759	1 572	2 187
78 anos	3 312	1 411	1 901
79 anos	3 299	1 414	1 885
80 anos ou mais	24 236	10 070	14 166
Idade ignorada	354	166	188

Fonte: IBGE, Contagem da População 2007.

(1) Inclusive a população estimada nos domicílios fechados.

Tabela 1.2.16 - População recenseada, por sexo, segundo a idade - Bahia - 2007

(continua)

Idade	População recenseada, por sexo		
	Total	Homens	Mulheres
**Total**	**(1) 9 426 047**	**4 708 275**	**4 671 364**
Menos de 1 ano	143 264	72 851	70 413
1 ano	155 785	79 450	76 335
2 anos	160 535	81 663	78 872
3 anos	167 846	85 506	82 340
4 anos	174 392	88 498	85 894
5 anos	175 392	89 484	85 908
6 anos	187 717	95 978	91 739
7 anos	196 659	100 394	96 265
8 anos	185 659	95 005	90 654
9 anos	189 344	96 153	93 191
10 anos	194 585	99 470	95 115
11 anos	195 175	99 216	95 959
12 anos	203 296	103 761	99 535
13 anos	197 593	100 215	97 378
14 anos	194 576	98 744	95 832
15 anos	194 360	97 782	96 578
16 anos	190 926	97 569	93 357
17 anos	195 781	100 089	95 692
18 anos	200 592	103 568	97 024
19 anos	196 262	99 154	97 108
20 anos	198 505	100 836	97 669
21 anos	190 117	97 232	92 885
22 anos	190 435	98 223	92 212
23 anos	184 546	95 003	89 543
24 anos	183 100	93 075	90 025
25 anos	180 830	92 471	88 359
26 anos	169 768	86 277	83 491
27 anos	166 250	84 433	81 817
28 anos	153 994	78 000	75 994
29 anos	147 220	73 609	73 611
30 anos	147 220	74 804	72 416
31 anos	128 837	63 562	65 275
32 anos	138 221	69 198	69 023
33 anos	127 085	63 657	63 428
34 anos	122 660	61 068	61 592
35 anos	120 323	60 081	60 242
36 anos	122 377	60 627	61 750
37 anos	120 744	59 596	61 148
38 anos	120 825	60 148	60 677

Tabela 1.2.16 - População recenseada, por sexo, segundo a idade - Bahia - 2007

(conclusão)

Idade	População recenseada, por sexo		
	Total	Homens	Mulheres
39 anos	114 249	56 584	57 665
40 anos	126 260	63 976	62 284
41 anos	102 141	50 669	51 472
42 anos	114 704	58 257	56 447
43 anos	106 464	53 314	53 150
44 anos	98 028	49 189	48 839
45 anos	95 581	48 654	46 927
46 anos	94 298	46 990	47 308
47 anos	90 137	44 547	45 590
48 anos	87 806	43 621	44 185
49 anos	85 549	42 147	43 402
50 anos	83 393	41 933	41 460
51 anos	72 642	35 686	36 956
52 anos	75 086	37 306	37 780
53 anos	71 222	35 279	35 943
54 anos	72 081	34 966	37 115
55 anos	73 218	34 730	38 488
56 anos	70 953	34 503	36 450
57 anos	64 273	31 208	33 065
58 anos	61 977	30 590	31 387
59 anos	62 142	30 710	31 432
60 anos	64 328	31 338	32 990
61 anos	50 560	24 774	25 786
62 anos	56 042	27 196	28 846
63 anos	54 008	26 089	27 919
64 anos	52 973	25 904	27 069
65 anos	49 911	24 340	25 571
66 anos	49 737	23 640	26 097
67 anos	49 817	23 950	25 867
68 anos	41 133	19 485	21 648
69 anos	35 310	16 757	18 553
70 anos	40 653	18 764	21 889
71 anos	31 820	15 078	16 742
72 anos	31 742	15 211	16 531
73 anos	27 266	12 873	14 393
74 anos	31 374	14 485	16 889
75 anos	28 703	13 382	15 321
76 anos	28 465	13 074	15 391
77 anos	23 888	11 374	12 514
78 anos	22 268	10 483	11 785
79 anos	18 834	8 821	10 013
80 anos ou mais	152 917	68 454	84 463
Idade ignorada	2 890	1 494	1 396

Fonte: IBGE, Contagem da População 2007.

(1) Inclusive a população estimada nos domicílios fechados e nos domicílios provenientes de setor censitário cujo arquivo foi danificado.

Tabela 1.2.17 - População recenseada, por sexo, segundo a idade - Minas Gerais - 2007

(continua)

Idade	População recenseada, por sexo		
	Total	Homens	Mulheres
**Total**	**(1) 12 597 121**	**6 265 664**	**6 269 402**
Menos de 1 ano	166 696	84 735	81 961
1 ano	178 236	90 950	87 286
2 anos	175 658	89 199	86 459
3 anos	185 282	93 897	91 385
4 anos	190 457	97 097	93 360
5 anos	197 064	100 217	96 847
6 anos	216 166	109 693	106 473
7 anos	224 501	114 546	109 955
8 anos	217 192	111 112	106 080
9 anos	227 161	115 138	112 023
10 anos	232 669	118 300	114 369
11 anos	236 742	120 586	116 156
12 anos	241 493	122 749	118 744
13 anos	232 696	117 983	114 713
14 anos	231 933	117 769	114 164
15 anos	236 382	119 389	116 993
16 anos	236 794	119 874	116 920
17 anos	237 346	120 834	116 512
18 anos	242 487	124 240	118 247
19 anos	231 032	117 476	113 556
20 anos	228 816	116 736	112 080
21 anos	220 111	112 492	107 619
22 anos	221 673	114 224	107 449
23 anos	221 549	113 682	107 867
24 anos	228 682	117 037	111 645
25 anos	224 320	113 811	110 509
26 anos	212 442	108 361	104 081
27 anos	210 782	106 837	103 945
28 anos	198 629	99 923	98 706
29 anos	195 584	97 927	97 657
30 anos	196 683	99 327	97 356
31 anos	177 315	87 323	89 992
32 anos	186 450	92 461	93 989
33 anos	177 493	88 544	88 949
34 anos	179 392	89 101	90 291
35 anos	179 709	89 101	90 608
36 anos	179 999	89 136	90 863
37 anos	179 013	88 166	90 847
38 anos	180 417	89 482	90 935

Tabela 1.2.17 - População recenseada, por sexo, segundo a idade - Minas Gerais - 2007

(conclusão)

Idade	População recenseada, por sexo		
	Total	Homens	Mulheres
39 anos	175 477	86 540	88 937
40 anos	188 495	94 243	94 252
41 anos	168 095	83 405	84 690
42 anos	184 332	92 645	91 687
43 anos	174 237	86 989	87 248
44 anos	166 138	82 757	83 381
45 anos	162 026	81 814	80 212
46 anos	156 191	78 021	78 170
47 anos	151 886	75 608	76 278
48 anos	148 975	73 978	74 997
49 anos	145 450	72 247	73 203
50 anos	145 779	73 744	72 035
51 anos	128 863	63 998	64 865
52 anos	133 095	66 806	66 289
53 anos	125 830	62 763	63 067
54 anos	120 318	59 152	61 166
55 anos	116 149	56 794	59 355
56 anos	113 318	56 036	57 282
57 anos	100 970	50 362	50 608
58 anos	98 019	48 559	49 460
59 anos	96 208	47 583	48 625
60 anos	96 378	47 081	49 297
61 anos	80 196	39 212	40 984
62 anos	83 184	40 550	42 634
63 anos	80 376	38 926	41 450
64 anos	78 977	38 133	40 844
65 anos	74 954	36 207	38 747
66 anos	73 295	34 876	38 419
67 anos	71 271	33 877	37 394
68 anos	63 721	30 408	33 313
69 anos	57 104	27 087	30 017
70 anos	60 936	28 577	32 359
71 anos	53 435	25 263	28 172
72 anos	51 967	24 478	27 489
73 anos	47 813	22 143	25 670
74 anos	46 922	21 624	25 298
75 anos	42 040	19 439	22 601
76 anos	44 469	20 023	24 446
77 anos	38 144	17 350	20 794
78 anos	33 158	14 875	18 283
79 anos	28 991	12 881	16 110
80 anos ou mais	188 130	77 782	110 348
Idade ignorada	2 708	1 373	1 335

Fonte: IBGE, Contagem da População 2007.
(1) Inclusive a população estimada nos domicílios fechados.

Tabela 1.2.18 - População recenseada, por sexo, segundo a idade - Espírito Santo - 2007

(continua)

Idade	População recenseada, por sexo		
	Total	Homens	Mulheres
**Total**	**(1) 1 702 365**	**847 307**	**840 237**
Menos de 1 ano	23 306	11 836	11 470
1 ano	25 112	12 708	12 404
2 anos	24 894	12 666	12 228
3 anos	26 274	13 396	12 878
4 anos	26 643	13 649	12 994
5 anos	27 352	13 800	13 552
6 anos	29 201	14 740	14 461
7 anos	30 198	15 388	14 810
8 anos	29 553	15 093	14 460
9 anos	30 746	15 807	14 939
10 anos	31 049	15 745	15 304
11 anos	31 796	16 079	15 717
12 anos	32 030	16 257	15 773
13 anos	31 272	15 642	15 630
14 anos	30 379	15 426	14 953
15 anos	30 986	15 566	15 420
16 anos	31 409	16 026	15 383
17 anos	31 480	15 975	15 505
18 anos	32 443	16 539	15 904
19 anos	32 026	16 056	15 970
20 anos	32 323	16 300	16 023
21 anos	30 987	15 924	15 063
22 anos	30 684	15 798	14 886
23 anos	30 530	15 582	14 948
24 anos	31 926	16 396	15 530
25 anos	31 679	16 018	15 661
26 anos	30 123	15 406	14 717
27 anos	29 799	15 088	14 711
28 anos	28 447	14 208	14 239
29 anos	28 103	13 940	14 163
30 anos	28 213	14 203	14 010
31 anos	25 301	12 497	12 804
32 anos	26 207	13 088	13 119
33 anos	25 064	12 505	12 559
34 anos	25 246	12 600	12 646
35 anos	25 072	12 362	12 710
36 anos	24 958	12 471	12 487
37 anos	25 058	12 361	12 697
38 anos	24 623	12 221	12 402

Tabela 1.2.18 - População recenseada, por sexo, segundo a idade - Espírito Santo - 2007

(conclusão)

Idade	População recenseada, por sexo		
	Total	Homens	Mulheres
39 anos	24 115	12 145	11 970
40 anos	25 444	12 936	12 508
41 anos	22 745	11 442	11 303
42 anos	24 249	12 200	12 049
43 anos	23 109	11 659	11 450
44 anos	22 258	11 389	10 869
45 anos	20 933	10 679	10 254
46 anos	20 779	10 510	10 269
47 anos	19 670	9 800	9 870
48 anos	19 479	9 834	9 645
49 anos	19 263	9 538	9 725
50 anos	18 991	9 672	9 319
51 anos	17 078	8 605	8 473
52 anos	17 439	8 859	8 580
53 anos	16 447	8 236	8 211
54 anos	16 084	7 950	8 134
55 anos	14 924	7 403	7 521
56 anos	14 408	7 138	7 270
57 anos	12 739	6 244	6 495
58 anos	11 886	6 006	5 880
59 anos	11 957	6 061	5 896
60 anos	11 397	5 641	5 756
61 anos	9 660	4 772	4 888
62 anos	9 956	4 885	5 071
63 anos	9 494	4 658	4 836
64 anos	9 691	4 705	4 986
65 anos	9 044	4 430	4 614
66 anos	9 050	4 310	4 740
67 anos	8 531	4 162	4 369
68 anos	7 557	3 563	3 994
69 anos	7 049	3 391	3 658
70 anos	7 410	3 550	3 860
71 anos	6 362	3 131	3 231
72 anos	6 417	3 065	3 352
73 anos	6 076	2 859	3 217
74 anos	5 905	2 840	3 065
75 anos	5 114	2 403	2 711
76 anos	5 664	2 705	2 959
77 anos	5 065	2 393	2 672
78 anos	4 277	2 015	2 262
79 anos	3 771	1 763	2 008
80 anos ou mais	23 272	10 253	13 019
Idade ignorada	293	145	148

Fonte: IBGE, Contagem da População 2007.
(1) Inclusive a população estimada nos domicílios fechados.

Tabela 1.2.19 - População recenseada, por sexo, segundo a idade - Rio de Janeiro - 2007

(continua)

Idade	População recenseada, por sexo		
	Total	Homens	Mulheres
**Total**	**(1) 3 302 474**	**1 610 466**	**1 666 529**
Menos de 1 ano	41 716	21 145	20 571
1 ano	45 472	23 077	22 395
2 anos	45 550	23 183	22 367
3 anos	47 419	24 001	23 418
4 anos	48 080	24 411	23 669
5 anos	49 881	25 416	24 465
6 anos	55 687	28 412	27 275
7 anos	57 520	29 179	28 341
8 anos	55 988	28 648	27 340
9 anos	57 821	29 475	28 346
10 anos	58 627	29 939	28 688
11 anos	57 916	29 323	28 593
12 anos	59 128	29 934	29 194
13 anos	56 034	28 361	27 673
14 anos	55 381	27 984	27 397
15 anos	54 658	27 342	27 316
16 anos	54 539	27 561	26 978
17 anos	55 313	27 865	27 448
18 anos	57 697	29 103	28 594
19 anos	54 403	27 443	26 960
20 anos	55 865	27 935	27 930
21 anos	53 692	26 860	26 832
22 anos	55 405	27 780	27 625
23 anos	55 788	27 558	28 230
24 anos	58 523	28 946	29 577
25 anos	60 193	29 503	30 690
26 anos	57 970	28 708	29 262
27 anos	57 121	28 200	28 921
28 anos	54 919	26 785	28 134
29 anos	54 069	26 284	27 785
30 anos	56 655	27 856	28 799
31 anos	48 427	23 439	24 988
32 anos	51 662	25 162	26 500
33 anos	48 291	23 523	24 768
34 anos	49 644	24 093	25 551
35 anos	49 979	24 184	25 795
36 anos	51 506	24 871	26 635
37 anos	49 659	23 996	25 663
38 anos	49 302	23 921	25 381

Tabela 1.2.19 - População recenseada, por sexo, segundo a idade - Rio de Janeiro - 2007

(conclusão)

Idade	População recenseada, por sexo		
	Total	Homens	Mulheres
39 anos	48 263	23 272	24 991
40 anos	54 363	26 578	27 785
41 anos	46 872	22 772	24 100
42 anos	52 260	25 336	26 924
43 anos	47 578	23 071	24 507
44 anos	45 637	22 150	23 487
45 anos	45 495	22 262	23 233
46 anos	43 952	21 250	22 702
47 anos	42 041	20 373	21 668
48 anos	41 203	20 190	21 013
49 anos	40 980	19 724	21 256
50 anos	42 539	20 970	21 569
51 anos	35 722	17 158	18 564
52 anos	37 278	18 274	19 004
53 anos	35 041	17 143	17 898
54 anos	33 644	16 313	17 331
55 anos	31 504	15 515	15 989
56 anos	31 916	15 696	16 220
57 anos	28 417	13 907	14 510
58 anos	26 577	12 852	13 725
59 anos	25 766	12 466	13 300
60 anos	26 670	12 910	13 760
61 anos	21 420	10 288	11 132
62 anos	21 222	10 186	11 036
63 anos	20 423	9 782	10 641
64 anos	20 812	9 946	10 866
65 anos	19 856	9 506	10 350
66 anos	18 333	8 741	9 592
67 anos	17 288	8 289	8 999
68 anos	15 861	7 560	8 301
69 anos	14 591	6 919	7 672
70 anos	15 595	7 234	8 361
71 anos	13 010	6 133	6 877
72 anos	12 860	5 869	6 991
73 anos	11 578	5 225	6 353
74 anos	11 341	5 085	6 256
75 anos	10 199	4 608	5 591
76 anos	10 924	4 792	6 132
77 anos	9 354	4 147	5 207
78 anos	8 196	3 511	4 685
79 anos	7 145	3 067	4 078
80 anos ou mais	44 543	17 391	27 152
Idade ignorada	1 226	599	627

Fonte: IBGE, Contagem da População 2007.
(1) Inclusive a população estimada nos domicílios fechados.

Tabela 1.2.20 - População recenseada, por sexo, segundo a idade - São Paulo - 2007

(continua)

Idade	População recenseada, por sexo		
	Total	Homens	Mulheres
**Total**	**(1) 13 475 401**	**6 681 557**	**6 693 364**
Menos de 1 ano	170 379	86 880	83 499
1 ano	179 496	91 439	88 057
2 anos	181 050	92 153	88 897
3 anos	184 371	93 762	90 609
4 anos	187 894	96 309	91 585
5 anos	190 889	97 955	92 934
6 anos	213 168	108 915	104 253
7 anos	221 395	112 907	108 488
8 anos	219 393	112 586	106 807
9 anos	224 635	114 216	110 419
10 anos	229 282	116 987	112 295
11 anos	229 753	117 219	112 534
12 anos	237 277	120 821	116 456
13 anos	226 144	114 929	111 215
14 anos	224 706	114 712	109 994
15 anos	229 748	115 900	113 848
16 anos	228 868	116 020	112 848
17 anos	234 262	118 767	115 495
18 anos	244 303	124 509	119 794
19 anos	237 739	121 215	116 524
20 anos	246 104	126 016	120 088
21 anos	239 427	122 944	116 483
22 anos	237 017	121 966	115 051
23 anos	241 989	124 492	117 497
24 anos	256 935	132 099	124 836
25 anos	255 217	130 983	124 234
26 anos	236 270	120 858	115 412
27 anos	235 139	119 949	115 190
28 anos	225 770	114 569	111 201
29 anos	218 930	110 320	108 610
30 anos	229 669	116 170	113 499
31 anos	200 353	99 973	100 380
32 anos	214 845	107 441	107 404
33 anos	201 194	101 013	100 181
34 anos	203 255	101 882	101 373
35 anos	205 921	102 494	103 427
36 anos	200 866	99 505	101 361
37 anos	195 662	96 912	98 750
38 anos	201 855	100 217	101 638

Tabela 1.2.20 - População recenseada, por sexo, segundo a idade - São Paulo - 2007

(conclusão)

Idade	População recenseada, por sexo		
	Total	Homens	Mulheres
39 anos	191 819	94 818	97 001
40 anos	215 559	107 572	107 987
41 anos	188 365	92 094	96 271
42 anos	204 129	101 617	102 512
43 anos	190 757	94 123	96 634
44 anos	178 816	88 115	90 701
45 anos	181 347	90 432	90 915
46 anos	173 014	85 248	87 766
47 anos	167 993	82 632	85 361
48 anos	165 324	81 403	83 921
49 anos	161 799	79 699	82 100
50 anos	166 167	82 646	83 521
51 anos	145 889	71 578	74 311
52 anos	150 387	74 523	75 864
53 anos	140 855	69 913	70 942
54 anos	134 657	66 353	68 304
55 anos	129 628	63 524	66 104
56 anos	124 727	61 694	63 033
57 anos	114 207	56 377	57 830
58 anos	109 458	53 493	55 965
59 anos	106 055	51 827	54 228
60 anos	109 911	53 759	56 152
61 anos	89 559	43 483	46 076
62 anos	91 860	44 635	47 225
63 anos	85 781	41 611	44 170
64 anos	85 446	40 970	44 476
65 anos	80 646	38 759	41 887
66 anos	75 460	36 061	39 399
67 anos	72 732	34 715	38 017
68 anos	67 955	32 314	35 641
69 anos	62 401	29 558	32 843
70 anos	66 446	31 184	35 262
71 anos	56 032	26 435	29 597
72 anos	55 902	26 023	29 879
73 anos	50 129	23 178	26 951
74 anos	50 510	23 079	27 431
75 anos	45 653	21 054	24 599
76 anos	45 012	20 193	24 819
77 anos	40 720	18 264	22 456
78 anos	36 356	16 271	20 085
79 anos	31 691	14 013	17 678
80 anos ou mais	190 150	77 074	113 076
Idade ignorada	2 447	1 239	1 208

Fonte: IBGE, Contagem da População 2007.

(1) Inclusive a população estimada nos domicílios fechados e nos domicílios provenientes de setor censitário cujo arquivo foi danificado.

Tabela 1.2.21 - População recenseada, por sexo, segundo a idade - Paraná - 2007

(continua)

Idade	População recenseada, por sexo		
	Total	Homens	Mulheres
**Total**	**(1) 6 262 285**	**3 107 256**	**3 122 367**
Menos de 1 ano	86 138	43 770	42 368
1 ano	93 279	47 426	45 853
2 anos	90 947	46 342	44 605
3 anos	93 000	47 094	45 906
4 anos	96 296	49 000	47 296
5 anos	97 064	49 444	47 620
6 anos	107 952	54 715	53 237
7 anos	115 029	58 643	56 386
8 anos	108 689	55 738	52 951
9 anos	115 487	58 825	56 662
10 anos	119 491	60 987	58 504
11 anos	121 334	62 029	59 305
12 anos	126 968	65 017	61 951
13 anos	119 294	60 511	58 783
14 anos	118 271	60 070	58 201
15 anos	118 109	59 894	58 215
16 anos	117 083	59 511	57 572
17 anos	114 429	58 241	56 188
18 anos	116 089	59 418	56 671
19 anos	109 046	54 685	54 361
20 anos	107 936	54 386	53 550
21 anos	105 635	53 500	52 135
22 anos	102 376	52 238	50 138
23 anos	104 151	52 688	51 463
24 anos	109 602	55 453	54 149
25 anos	106 264	53 477	52 787
26 anos	98 530	49 644	48 886
27 anos	99 470	49 482	49 988
28 anos	95 954	47 782	48 172
29 anos	95 174	46 838	48 336
30 anos	98 494	48 769	49 725
31 anos	90 679	44 160	46 519
32 anos	96 128	47 366	48 762
33 anos	91 318	45 246	46 072
34 anos	94 436	46 318	48 118
35 anos	94 363	46 529	47 834
36 anos	95 255	46 750	48 505
37 anos	94 347	46 081	48 266
38 anos	95 802	47 020	48 782

Tabela 1.2.21 - População recenseada, por sexo, segundo a idade - Paraná - 2007

(conclusão)

Idade	População recenseada, por sexo		
	Total	Homens	Mulheres
39 anos	91 205	44 637	46 568
40 anos	98 118	48 708	49 410
41 anos	88 953	43 869	45 084
42 anos	94 527	47 202	47 325
43 anos	87 734	43 449	44 285
44 anos	84 398	41 477	42 921
45 anos	81 543	40 493	41 050
46 anos	78 453	38 513	39 940
47 anos	74 604	36 627	37 977
48 anos	73 131	35 909	37 222
49 anos	71 924	35 467	36 457
50 anos	70 613	35 017	35 596
51 anos	64 095	31 208	32 887
52 anos	65 769	32 502	33 267
53 anos	62 578	30 885	31 693
54 anos	59 824	29 156	30 668
55 anos	57 013	27 535	29 478
56 anos	56 321	27 461	28 860
57 anos	52 061	25 514	26 547
58 anos	48 726	24 032	24 694
59 anos	47 935	23 753	24 182
60 anos	48 280	23 802	24 478
61 anos	41 175	20 182	20 993
62 anos	42 372	20 749	21 623
63 anos	40 775	19 979	20 796
64 anos	39 604	19 256	20 348
65 anos	36 037	17 710	18 327
66 anos	35 792	17 228	18 564
67 anos	33 651	16 721	16 930
68 anos	31 380	15 562	15 818
69 anos	28 453	14 048	14 405
70 anos	28 720	14 104	14 616
71 anos	25 346	12 511	12 835
72 anos	24 086	11 829	12 257
73 anos	22 203	10 680	11 523
74 anos	22 181	10 712	11 469
75 anos	19 018	9 163	9 855
76 anos	19 792	9 211	10 581
77 anos	16 837	7 908	8 929
78 anos	14 738	7 099	7 639
79 anos	12 785	5 966	6 819
80 anos ou mais	74 639	33 135	41 504
Idade ignorada	2 325	1 200	1 125

Fonte: IBGE, Contagem da População 2007.

(1) Inclusive a população estimada nos domicílios fechados e nos domicílios provenientes de setor censitário cujo arquivo foi danificado.

Tabela 1.2.22 - População recenseada, por sexo, segundo a idade - Santa Catarina - 2007

(continua)

Idade	População recenseada, por sexo		
	Total	Homens	Mulheres
**Total**	**(1) 4 307 161**	**2 142 129**	**2 143 822**
Menos de 1 ano	56 259	28 559	27 700
1 ano	60 186	30 849	29 337
2 anos	59 726	30 646	29 080
3 anos	60 721	30 898	29 823
4 anos	61 945	31 833	30 112
5 anos	63 456	32 568	30 888
6 anos	70 946	36 340	34 606
7 anos	75 000	38 360	36 640
8 anos	72 578	37 098	35 480
9 anos	74 762	37 991	36 771
10 anos	76 568	38 955	37 613
11 anos	77 848	39 546	38 302
12 anos	82 542	41 898	40 644
13 anos	77 292	39 276	38 016
14 anos	76 963	39 114	37 849
15 anos	79 199	39 651	39 548
16 anos	80 754	41 150	39 604
17 anos	78 868	39 935	38 933
18 anos	81 330	41 592	39 738
19 anos	77 103	39 128	37 975
20 anos	76 821	38 666	38 155
21 anos	77 337	39 358	37 979
22 anos	76 023	38 759	37 264
23 anos	77 264	39 129	38 135
24 anos	80 692	41 169	39 523
25 anos	78 786	39 823	38 963
26 anos	72 868	36 944	35 924
27 anos	73 146	36 823	36 323
28 anos	69 176	34 952	34 224
29 anos	67 133	33 566	33 567
30 anos	67 995	34 408	33 587
31 anos	62 728	30 967	31 761
32 anos	66 798	33 253	33 545
33 anos	64 328	32 301	32 027
34 anos	66 016	32 736	33 280
35 anos	65 665	33 044	32 621
36 anos	65 933	32 970	32 963
37 anos	65 345	32 497	32 848
38 anos	65 437	32 704	32 733

Tabela 1.2.22 - População recenseada, por sexo, segundo a idade - Santa Catarina - 2007

(conclusão)

Idade	População recenseada, por sexo		
	Total	Homens	Mulheres
39 anos	64 738	32 148	32 590
40 anos	69 776	35 104	34 672
41 anos	64 425	32 097	32 328
42 anos	68 338	34 260	34 078
43 anos	63 885	31 944	31 941
44 anos	62 031	30 973	31 058
45 anos	60 281	30 248	30 033
46 anos	58 831	29 144	29 687
47 anos	54 859	27 507	27 352
48 anos	52 712	26 426	26 286
49 anos	51 907	25 719	26 188
50 anos	50 653	25 391	25 262
51 anos	45 274	22 420	22 854
52 anos	46 260	23 217	23 043
53 anos	44 127	22 137	21 990
54 anos	42 301	20 742	21 559
55 anos	40 202	19 837	20 365
56 anos	39 829	19 984	19 845
57 anos	36 694	18 182	18 512
58 anos	33 896	16 748	17 148
59 anos	32 170	15 882	16 288
60 anos	31 803	15 464	16 339
61 anos	27 495	13 353	14 142
62 anos	27 466	13 424	14 042
63 anos	25 795	12 632	13 163
64 anos	25 097	11 946	13 151
65 anos	22 508	10 807	11 701
66 anos	22 006	10 387	11 619
67 anos	20 957	9 952	11 005
68 anos	19 534	9 108	10 426
69 anos	17 825	8 337	9 488
70 anos	18 168	8 464	9 704
71 anos	15 841	7 283	8 558
72 anos	15 342	6 981	8 361
73 anos	13 990	6 268	7 722
74 anos	13 338	5 984	7 354
75 anos	11 831	5 226	6 605
76 anos	12 356	5 330	7 026
77 anos	10 625	4 639	5 986
78 anos	9 337	4 048	5 289
79 anos	8 373	3 601	4 772
80 anos ou mais	48 517	18 788	29 729
Idade ignorada	1 021	541	480

Fonte: IBGE, Contagem da População 2007.

(1) Inclusive a população estimada nos domicílios fechados.

Tabela 1.2.23 - População recenseada, por sexo, segundo a idade - Rio Grande do Sul - 2007

(continua)

Idade	População recenseada, por sexo		
	Total	Homens	Mulheres
**Total**	**(1) 6 273 345**	**3 095 615**	**3 150 909**
Menos de 1 ano	74 537	38 081	36 456
1 ano	79 538	40 734	38 804
2 anos	80 490	40 860	39 630
3 anos	83 070	42 624	40 446
4 anos	83 654	42 647	41 007
5 anos	86 330	44 115	42 215
6 anos	97 099	49 589	47 510
7 anos	104 421	53 248	51 173
8 anos	98 770	50 680	48 090
9 anos	100 743	51 174	49 569
10 anos	102 972	52 293	50 679
11 anos	106 420	54 389	52 031
12 anos	113 539	58 041	55 498
13 anos	106 273	54 084	52 189
14 anos	104 555	53 173	51 382
15 anos	106 653	53 886	52 767
16 anos	108 996	55 561	53 435
17 anos	107 058	54 579	52 479
18 anos	107 299	54 791	52 508
19 anos	100 590	51 034	49 556
20 anos	101 535	51 594	49 941
21 anos	102 454	52 084	50 370
22 anos	100 377	51 161	49 216
23 anos	102 850	52 359	50 491
24 anos	107 056	54 274	52 782
25 anos	103 446	52 272	51 174
26 anos	96 117	48 352	47 765
27 anos	95 190	47 912	47 278
28 anos	90 967	45 258	45 709
29 anos	89 510	44 805	44 705
30 anos	89 817	44 582	45 235
31 anos	83 209	41 074	42 135
32 anos	88 785	44 253	44 532
33 anos	86 012	43 059	42 953
34 anos	88 943	44 054	44 889
35 anos	88 118	43 769	44 349
36 anos	91 023	45 144	45 879
37 anos	91 146	44 706	46 440
38 anos	91 573	45 171	46 402

Tabela 1.2.23 - População recenseada, por sexo, segundo a idade - Rio Grande do Sul - 2007

(conclusão)

Idade	População recenseada, por sexo		
	Total	Homens	Mulheres
39 anos	91 453	45 070	46 383
40 anos	98 796	48 939	49 857
41 anos	94 468	46 878	47 590
42 anos	100 321	50 102	50 219
43 anos	95 163	47 072	48 091
44 anos	94 185	46 690	47 495
45 anos	90 763	45 361	45 402
46 anos	90 018	44 853	45 165
47 anos	85 802	42 425	43 377
48 anos	83 985	41 697	42 288
49 anos	83 318	41 449	41 869
50 anos	82 547	41 179	41 368
51 anos	76 463	37 834	38 629
52 anos	77 314	38 508	38 806
53 anos	73 682	36 856	36 826
54 anos	70 088	34 627	35 461
55 anos	68 365	33 685	34 680
56 anos	67 448	33 246	34 202
57 anos	63 267	31 049	32 218
58 anos	59 294	29 058	30 236
59 anos	57 606	28 429	29 177
60 anos	55 361	27 145	28 216
61 anos	50 027	24 203	25 824
62 anos	49 655	24 029	25 626
63 anos	47 199	22 704	24 495
64 anos	45 299	21 555	23 744
65 anos	41 511	19 836	21 675
66 anos	42 008	19 854	22 154
67 anos	38 860	18 220	20 640
68 anos	37 432	17 648	19 784
69 anos	34 275	16 027	18 248
70 anos	33 850	15 329	18 521
71 anos	31 167	14 115	17 052
72 anos	29 576	13 222	16 354
73 anos	28 023	12 220	15 803
74 anos	26 357	11 513	14 844
75 anos	23 130	9 865	13 265
76 anos	25 115	10 461	14 654
77 anos	21 613	8 874	12 739
78 anos	19 304	7 970	11 334
79 anos	17 294	7 074	10 220
80 anos ou mais	102 669	36 651	66 018
Idade ignorada	1 318	627	691

Fonte: IBGE, Contagem da População 2007.

(1) Inclusive a população estimada nos domicílios fechados.

Tabela 1.2.24 - População recenseada, por sexo, segundo a idade - Mato Grosso do Sul - 2007

(continua)

Idade	População recenseada, por sexo		
	Total	Homens	Mulheres
**Total**	**(1) 2 265 274**	**1 122 705**	**1 129 179**
Menos de 1 ano	34 956	17 824	17 132
1 ano	36 822	18 860	17 962
2 anos	36 384	18 616	17 768
3 anos	36 773	18 761	18 012
4 anos	36 958	19 089	17 869
5 anos	36 798	18 851	17 947
6 anos	40 255	20 326	19 929
7 anos	41 524	21 344	20 180
8 anos	40 241	20 698	19 543
9 anos	42 178	21 433	20 745
10 anos	43 119	21 771	21 348
11 anos	44 286	22 186	22 100
12 anos	45 652	23 246	22 406
13 anos	43 882	22 191	21 691
14 anos	43 293	21 948	21 345
15 anos	42 683	21 493	21 190
16 anos	42 125	21 394	20 731
17 anos	42 745	21 639	21 106
18 anos	43 250	21 817	21 433
19 anos	42 163	21 193	20 970
20 anos	41 515	20 862	20 653
21 anos	41 811	21 278	20 533
22 anos	40 999	20 572	20 427
23 anos	41 086	20 482	20 604
24 anos	43 533	21 859	21 674
25 anos	42 242	21 126	21 116
26 anos	40 118	19 949	20 169
27 anos	40 252	20 012	20 240
28 anos	37 464	18 508	18 956
29 anos	37 281	18 255	19 026
30 anos	37 136	18 237	18 899
31 anos	34 667	16 868	17 799
32 anos	36 020	17 580	18 440
33 anos	34 219	16 868	17 351
34 anos	34 972	17 232	17 740
35 anos	33 118	16 167	16 951
36 anos	34 358	16 866	17 492
37 anos	33 261	16 073	17 188
38 anos	33 277	16 532	16 745

Tabela 1.2.24 - População recenseada, por sexo, segundo a idade - Mato Grosso do Sul - 2007

(conclusão)

Idade	População recenseada, por sexo		
	Total	Homens	Mulheres
39 anos	31 970	15 472	16 498
40 anos	33 406	16 523	16 883
41 anos	30 959	15 023	15 936
42 anos	33 634	16 548	17 086
43 anos	31 262	15 273	15 989
44 anos	29 874	14 609	15 265
45 anos	28 024	13 922	14 102
46 anos	27 454	13 392	14 062
47 anos	26 076	12 645	13 431
48 anos	24 948	12 254	12 694
49 anos	24 916	12 169	12 747
50 anos	24 258	11 897	12 361
51 anos	22 112	10 740	11 372
52 anos	22 553	11 277	11 276
53 anos	20 935	10 396	10 539
54 anos	19 519	9 593	9 926
55 anos	18 427	9 127	9 300
56 anos	18 181	8 948	9 233
57 anos	16 156	8 079	8 077
58 anos	15 368	7 617	7 751
59 anos	15 044	7 453	7 591
60 anos	14 764	7 375	7 389
61 anos	12 897	6 338	6 559
62 anos	12 816	6 309	6 507
63 anos	12 312	5 996	6 316
64 anos	12 450	6 138	6 312
65 anos	11 077	5 443	5 634
66 anos	10 973	5 368	5 605
67 anos	10 570	5 341	5 229
68 anos	9 395	4 759	4 636
69 anos	8 399	4 238	4 161
70 anos	8 658	4 411	4 247
71 anos	7 849	3 806	4 043
72 anos	7 180	3 595	3 585
73 anos	6 401	3 116	3 285
74 anos	6 637	3 341	3 296
75 anos	5 726	2 827	2 899
76 anos	5 911	2 839	3 072
77 anos	5 119	2 527	2 592
78 anos	4 454	2 183	2 271
79 anos	3 851	1 891	1 960
80 anos ou mais	25 140	11 495	13 645
Idade ignorada	813	406	407

Fonte: IBGE, Contagem da População 2007.
(1) Inclusive a população estimada nos domicílios fechados.

Tabela 1.2.25 - População recenseada, por sexo, segundo a idade - Mato Grosso - 2007

(continua)

Idade	População recenseada, por sexo		
	Total	Homens	Mulheres
**Total**	**(1) 2 854 642**	**1 452 153**	**1 377 327**
Menos de 1 ano	44 706	22 795	21 911
1 ano	48 169	24 443	23 726
2 anos	47 396	24 057	23 339
3 anos	47 931	24 237	23 694
4 anos	47 841	24 179	23 662
5 anos	48 447	24 812	23 635
6 anos	51 932	26 830	25 102
7 anos	53 145	27 105	26 040
8 anos	51 482	26 472	25 010
9 anos	53 754	27 342	26 412
10 anos	56 216	28 768	27 448
11 anos	56 935	28 949	27 986
12 anos	58 394	29 725	28 669
13 anos	56 354	28 624	27 730
14 anos	54 141	27 401	26 740
15 anos	54 500	27 440	27 060
16 anos	54 378	27 634	26 744
17 anos	55 845	28 280	27 565
18 anos	57 100	29 385	27 715
19 anos	54 942	27 723	27 219
20 anos	55 897	28 429	27 468
21 anos	54 942	28 074	26 868
22 anos	55 562	28 320	27 242
23 anos	55 494	28 527	26 967
24 anos	56 842	29 110	27 732
25 anos	56 202	28 549	27 653
26 anos	54 105	27 545	26 560
27 anos	54 583	27 705	26 878
28 anos	51 176	26 091	25 085
29 anos	50 376	25 189	25 187
30 anos	52 003	26 251	25 752
31 anos	45 417	22 794	22 623
32 anos	48 751	24 470	24 281
33 anos	45 499	23 293	22 206
34 anos	46 102	23 340	22 762
35 anos	44 641	22 699	21 942
36 anos	44 813	22 787	22 026
37 anos	44 020	22 291	21 729
38 anos	43 679	22 349	21 330

Tabela 1.2.25 - População recenseada, por sexo, segundo a idade - Mato Grosso - 2007

(conclusão)

Idade	População recenseada, por sexo		
	Total	Homens	Mulheres
39 anos	41 013	20 989	20 024
40 anos	44 767	23 420	21 347
41 anos	37 953	19 544	18 409
42 anos	41 338	21 755	19 583
43 anos	38 273	19 956	18 317
44 anos	35 660	18 420	17 240
45 anos	35 142	18 560	16 582
46 anos	33 110	17 358	15 752
47 anos	31 179	16 270	14 909
48 anos	30 125	15 825	14 300
49 anos	28 950	14 976	13 974
50 anos	29 031	15 507	13 524
51 anos	24 960	13 061	11 899
52 anos	25 362	13 548	11 814
53 anos	23 571	12 359	11 212
54 anos	22 662	11 930	10 732
55 anos	21 161	11 048	10 113
56 anos	20 659	11 029	9 630
57 anos	18 062	9 587	8 475
58 anos	17 085	8 950	8 135
59 anos	16 298	8 575	7 723
60 anos	16 606	8 831	7 775
61 anos	13 255	6 975	6 280
62 anos	13 934	7 366	6 568
63 anos	12 892	6 765	6 127
64 anos	12 986	6 917	6 069
65 anos	11 856	6 354	5 502
66 anos	11 682	6 245	5 437
67 anos	11 094	5 996	5 098
68 anos	9 285	4 877	4 408
69 anos	8 397	4 491	3 906
70 anos	8 658	4 634	4 024
71 anos	7 270	3 946	3 324
72 anos	6 699	3 620	3 079
73 anos	6 047	3 192	2 855
74 anos	6 097	3 219	2 878
75 anos	5 303	2 808	2 495
76 anos	5 410	2 800	2 610
77 anos	4 300	2 264	2 036
78 anos	3 923	2 145	1 778
79 anos	3 205	1 702	1 503
80 anos ou mais	19 604	9 849	9 755
Idade ignorada	904	476	428

Fonte: IBGE, Contagem da População 2007.

(1) Inclusive a população estimada nos domicílios fechados.

Tabela 1.2.26 - População recenseada, por sexo, segundo a idade - Goiás - 2007

(continua)

Idade	População recenseada, por sexo		
	Total	Homens	Mulheres
**Total**	**(1) 3 405 497**	**1 702 655**	**1 667 722**
Menos de 1 ano	49 112	24 957	24 155
1 ano	53 047	27 103	25 944
2 anos	52 585	26 897	25 688
3 anos	55 307	28 142	27 165
4 anos	56 219	28 704	27 515
5 anos	57 102	29 193	27 909
6 anos	59 838	30 728	29 110
7 anos	62 692	32 102	30 590
8 anos	61 624	31 626	29 998
9 anos	63 477	32 425	31 052
10 anos	64 732	32 905	31 827
11 anos	64 855	33 093	31 762
12 anos	66 009	33 557	32 452
13 anos	63 167	32 234	30 933
14 anos	62 517	31 908	30 609
15 anos	61 967	31 279	30 688
16 anos	61 010	30 906	30 104
17 anos	60 779	30 669	30 110
18 anos	63 618	32 551	31 067
19 anos	60 795	31 036	29 759
20 anos	61 541	31 258	30 283
21 anos	59 928	30 413	29 515
22 anos	60 394	30 989	29 405
23 anos	59 968	30 659	29 309
24 anos	62 742	31 715	31 027
25 anos	63 319	31 905	31 414
26 anos	60 686	30 677	30 009
27 anos	61 061	30 350	30 711
28 anos	58 012	28 816	29 196
29 anos	57 941	28 460	29 481
30 anos	60 522	30 208	30 314
31 anos	53 927	26 209	27 718
32 anos	58 022	28 598	29 424
33 anos	53 609	26 676	26 933
34 anos	54 249	26 729	27 520
35 anos	53 269	26 118	27 151
36 anos	53 564	26 482	27 082
37 anos	53 273	26 475	26 798
38 anos	53 137	26 394	26 743

Tabela 1.2.26 - População recenseada, por sexo, segundo a idade - Goiás - 2007

(conclusão)

Idade	População recenseada, por sexo		
	Total	Homens	Mulheres
39 anos	49 153	24 267	24 886
40 anos	53 416	27 043	26 373
41 anos	45 328	22 771	22 557
42 anos	49 349	25 018	24 331
43 anos	46 022	23 133	22 889
44 anos	43 350	21 823	21 527
45 anos	41 337	21 054	20 283
46 anos	40 114	20 271	19 843
47 anos	37 508	18 645	18 863
48 anos	36 490	18 255	18 235
49 anos	35 603	17 966	17 637
50 anos	35 301	17 913	17 388
51 anos	31 524	15 972	15 552
52 anos	31 923	16 289	15 634
53 anos	29 990	15 286	14 704
54 anos	29 307	14 720	14 587
55 anos	28 512	14 190	14 322
56 anos	27 473	14 106	13 367
57 anos	24 151	12 364	11 787
58 anos	22 987	11 670	11 317
59 anos	23 493	11 929	11 564
60 anos	23 868	12 177	11 691
61 anos	19 050	9 844	9 206
62 anos	20 020	10 119	9 901
63 anos	19 007	9 685	9 322
64 anos	18 874	9 659	9 215
65 anos	18 231	9 387	8 844
66 anos	17 550	8 834	8 716
67 anos	17 108	8 765	8 343
68 anos	14 392	7 408	6 984
69 anos	12 549	6 403	6 146
70 anos	13 551	6 949	6 602
71 anos	11 842	6 032	5 810
72 anos	11 135	5 779	5 356
73 anos	9 791	5 040	4 751
74 anos	9 943	5 072	4 871
75 anos	8 477	4 293	4 184
76 anos	8 783	4 367	4 416
77 anos	7 158	3 612	3 546
78 anos	6 463	3 385	3 078
79 anos	5 593	2 862	2 731
80 anos ou mais	34 183	16 714	17 469
Idade ignorada	862	438	424

Fonte: IBGE, Contagem da População 2007.
(1) Inclusive a população estimada nos domicílios fechados.

Tabela 2.1.1 - Domicílios recenseados, por espécie, segundo os municípios - Rondônia - 2007

Municípios	Domicílios recenseados, por espécie				
	Total	Particulares			Coletivos
		Total	Ocupados (1)	Não-ocupados	
**Rondônia**	**459 731**	**458 749**	**409 536**	**49 213**	**982**
Alta Floresta D'Oeste	7 674	7 663	6 796	867	11
Alto Alegre dos Parecis	3 420	3 416	3 151	265	4
Alto Paraíso	5 036	5 029	4 704	325	7
Alvorada D'Oeste	5 546	5 537	4 733	804	9
Ariquemes	26 581	26 534	23 408	3 126	47
Buritis	10 480	10 462	9 110	1 352	18
Cabixi	2 224	2 216	1 946	270	8
Cacaulândia	1 848	1 847	1 579	268	1
Cacoal	24 770	24 739	22 391	2 348	31
Campo Novo de Rondônia	3 757	3 746	3 398	348	11
Candeias do Jamari	5 757	5 711	4 445	1 266	46
Castanheiras	1 222	1 219	1 018	201	3
Cerejeiras	5 399	5 390	5 045	345	9
Chupinguaia	2 260	2 237	2 052	185	23
Colorado do Oeste	6 004	5 999	5 374	625	5
Corumbiara	3 154	3 152	2 783	369	2
Costa Marques	4 522	4 504	3 571	933	18
Cujubim	4 463	4 455	3 909	546	8
Espigão D'Oeste	8 838	8 831	8 053	778	7
Governador Jorge Teixeira	3 346	3 340	3 046	294	6
Guajará-Mirim	10 847	10 799	9 498	1 301	48
Itapuã do Oeste	2 695	2 686	2 178	508	9
Jaru	17 281	17 248	15 166	2 082	33
Ji-Paraná	35 300	35 264	31 163	4 101	36
Machadinho D'Oeste	10 372	10 329	8 591	1 738	43
Ministro Andreazza	3 064	3 063	2 851	212	1
Mirante da Serra	3 801	3 795	3 393	402	6
Monte Negro	3 587	3 582	3 339	243	5
Nova Brasilândia D'Oeste	5 486	5 479	4 935	544	7
Nova Mamoré	6 717	6 703	5 857	846	14
Nova União	2 303	2 299	2 071	228	4
Novo Horizonte do Oeste	3 139	3 139	2 704	435	-
Ouro Preto do Oeste	11 598	11 571	10 427	1 144	27
Parecis	1 538	1 537	1 289	248	1
Pimenta Bueno	10 553	10 532	9 519	1 013	21
Pimenteiras do Oeste	906	897	667	230	9
Porto Velho	111 612	111 289	101 388	9 901	323
Presidente Médici	7 263	7 252	6 522	730	11
Primavera de Rondônia	1 252	1 252	1 097	155	-
Rio Crespo	1 078	1 075	920	155	3
Rolim de Moura	15 756	15 713	14 429	1 284	43
Santa Luzia D'Oeste	3 123	3 117	2 728	389	6
São Felipe D'Oeste	1 904	1 899	1 786	113	5
São Francisco do Guaporé	5 431	5 423	4 465	958	8
São Miguel do Guaporé	7 053	7 047	6 296	751	6
Seringueiras	3 562	3 551	3 207	344	11
Teixeirópolis	1 433	1 432	1 375	57	1
Theobroma	3 321	3 316	2 774	542	5
Urupá	4 357	4 353	3 718	635	4
Vale do Anari	2 848	2 845	2 479	366	3
Vale do Paraíso	2 924	2 923	2 448	475	1
Vilhena	21 326	21 312	19 744	1 568	14

Fonte: IBGE, Contagem da População 2007.

(1) Inclusive os domicílios fechados com população estimada.

Tabela 2.1.2 - Domicílios recenseados, por espécie, segundo os municípios - Acre - 2007

Municípios	Domicílios recenseados, por espécie				
	Total	Particulares			Coletivos
		Total	Ocupados (1)	Não-ocupados	
**Acre**	**181 599**	**181 183**	**165 037**	**16 146**	**416**
Acrelândia	3 508	3 507	2 914	593	1
Assis Brasil	1 564	1 543	1 295	248	21
Brasiléia	5 702	5 679	5 048	631	23
Bujari	2 008	2 006	1 776	230	2
Capixaba	2 581	2 576	2 213	363	5
Cruzeiro do Sul	18 109	18 049	16 898	1 151	60
Epitaciolândia	4 098	4 091	3 634	457	7
Feijó	6 726	6 713	6 466	247	13
Jordão	1 110	1 108	1 058	50	2
Mâncio Lima	3 484	3 477	3 128	349	7
Manoel Urbano	1 851	1 848	1 592	256	3
Marechal Thaumaturgo	2 701	2 694	2 531	163	7
Plácido de Castro	5 075	5 048	4 380	668	27
Porto Acre	4 590	4 576	3 584	992	14
Porto Walter	1 681	1 672	1 581	91	9
Rio Branco	86 434	86 294	79 563	6 731	140
Rodrigues Alves	3 044	3 042	2 698	344	2
Santa Rosa do Purus	823	820	702	118	3
Sena Madureira	8 917	8 898	8 034	864	19
Senador Guiomard	5 569	5 562	5 314	248	7
Tarauacá	7 499	7 471	6 718	753	28
Xapuri	4 525	4 509	3 910	599	16

Fonte: IBGE, Contagem da População 2007.
(1) Inclusive os domicílios fechados com população estimada.

Tabela 2.1.3 - Domicílios recenseados, por espécie, segundo os municípios - Amazonas - 2007

(continua)

Municípios	Domicílios recenseados, por espécie				
	Total	Particulares			Coletivos
		Total	Ocupados (1)	Não-ocupados	
**Amazonas**	**783 736**	**781 483**	**717 536**	**63 947**	**2 253**
Alvarães	2 522	2 513	2 299	214	9
Amaturá	1 629	1 615	1 500	115	14
Anamã	1 727	1 720	1 471	249	7
Anori	2 894	2 885	2 692	193	9
Apuí	4 211	4 206	3 882	324	5
Atalaia do Norte	2 424	2 411	2 267	144	13
Autazes	8 021	7 968	6 200	1 768	53
Barcelos	4 327	4 318	4 234	84	9
Barreirinha	5 092	5 080	4 826	254	12
Benjamin Constant	5 358	5 331	5 077	254	27
Beruri	2 706	2 700	2 513	187	6
Boa Vista do Ramos	2 777	2 764	2 377	387	13
Boca do Acre	7 070	7 031	6 324	707	39
Borba	6 735	6 706	6 079	627	29
Caapiranga	2 238	2 238	2 200	38	-
Canutama	2 439	2 434	2 225	209	5
Carauari	5 372	5 355	4 926	429	17
Careiro	7 989	7 951	7 012	939	38
Careiro da Várzea	5 675	5 667	5 337	330	8
Coari	13 020	12 963	12 230	733	57
Codajás	3 719	3 713	3 034	679	6
Eirunepé	6 419	6 369	5 831	538	50
Envira	3 462	3 450	3 257	193	12
Fonte Boa	3 912	3 891	3 575	316	21
Guajará	2 636	2 634	2 517	117	2
Humaitá	8 737	8 695	7 744	951	42
Ipixuna	2 924	2 916	2 841	75	8
Iranduba	9 328	9 274	7 584	1 690	54
Itacoatiara	21 397	21 269	18 417	2 852	128
Itamarati	1 597	1 597	1 510	87	-

Tabela 2.1.3 - Domicílios recenseados, por espécie, segundo os municípios - Amazonas - 2007

(conclusão)

Municípios	Domicílios recenseados, por espécie				
	Total	Particulares			Coletivos
		Total	Ocupados (1)	Não-ocupados	
Itapiranga	1 792	1 772	1 638	134	20
Japurá	1 131	1 105	998	107	26
Juruá	1 662	1 653	1 590	63	9
Jutaí	3 271	3 266	2 979	287	5
Lábrea	8 015	7 973	7 156	817	42
Manacapuru	19 019	18 950	16 893	2 057	69
Manaquiri	4 169	4 150	3 972	178	19
Manaus	448 967	448 384	414 446	33 938	583
Manicoré	9 730	9 680	8 652	1 028	50
Maraã	2 961	2 939	2 765	174	22
Maués	9 843	9 809	8 898	911	34
Nhamundá	3 384	3 376	3 223	153	8
Nova Olinda do Norte	5 316	5 299	5 024	275	17
Novo Airão	2 881	2 861	2 542	319	20
Novo Aripuanã	4 062	4 047	3 489	558	15
Parintins	19 754	19 645	18 930	715	109
Pauini	3 497	3 477	3 242	235	20
Presidente Figueiredo	7 205	7 002	5 876	1 126	203
Rio Preto da Eva	5 717	5 688	5 146	542	29
Santa Isabel do Rio Negro	2 741	2 724	2 648	76	17
Santo Antônio do Içá	4 976	4 951	4 895	56	25
São Gabriel da Cachoeira	8 738	8 672	7 263	1 409	66
São Paulo de Olivença	5 518	5 495	5 206	289	23
São Sebastião do Uatumã	1 894	1 887	1 605	282	7
Silves	2 004	1 993	1 665	328	11
Tabatinga	8 404	8 363	8 004	359	41
Tapauá	4 088	4 080	3 704	376	8
Tefé	12 704	12 675	11 986	689	29
Tonantins	2 971	2 967	2 897	70	4
Uarini	2 014	2 007	1 802	205	7
Urucará	3 481	3 474	3 329	145	7
Urucurituba	3 470	3 455	3 092	363	15

Fonte: IBGE, Contagem da População 2007.
(1) Inclusive os domicílios fechados com população estimada.

Tabela 2.1.4 - Domicílios recenseados, por espécie, segundo os municípios - Roraima - 2007

Municípios	Domicílios recenseados, por espécie				
	Total	Particulares			Coletivos
		Total	Ocupados (1)	Não-ocupados	
**Roraima**	**113 386**	**113 035**	**98 882**	**14 153**	**351**
Alto Alegre	3 262	3 195	3 003	192	67
Amajari	1 628	1 612	1 528	84	16
Boa Vista	74 243	74 133	65 487	8 646	110
Bonfim	2 639	2 634	2 373	261	5
Cantá	3 548	3 539	2 914	625	9
Caracaraí	4 670	4 645	4 019	626	25
Caroebe	2 181	2 157	1 698	459	24
Iracema	1 850	1 850	1 521	329	-
Mucajaí	3 661	3 646	3 124	522	15
Normandia	1 635	1 629	1 423	206	6
Pacaraima	2 168	2 146	1 869	277	22
Rorainópolis	6 614	6 577	5 607	970	37
São João da Baliza	1 886	1 878	1 467	411	8
São Luiz	1 943	1 939	1 535	404	4
Uiramutã	1 458	1 455	1 314	141	3

Fonte: IBGE, Contagem da População 2007.
(1) Inclusive os domicílios fechados com população estimada.

Tabela 2.1.5 - Domicílios recenseados, por espécie, segundo os municípios - Pará - 2007

(continua)

Municípios	Domicílios recenseados, por espécie				
	Total	Particulares			Coletivos
		Total	Ocupados (1)	Não-ocupados	
**Pará**	**1 216 902**	**1 213 760**	**1 068 091**	**145 669**	**3 142**
Abaetetuba	30 560	30 522	27 403	3 119	38
Abel Figueiredo	1 945	1 939	1 721	218	6
Acará	11 028	11 010	10 138	872	18
Afuá	6 545	6 527	5 823	704	18
Água Azul do Norte	6 320	6 309	6 131	178	11
Alenquer	12 025	12 005	10 828	1 177	20
Almeirim	9 109	8 920	6 922	1 998	189
Altamira	27 437	27 390	23 374	4 016	47
Anajás	4 814	4 811	4 387	424	3
Anapu	4 601	4 596	4 043	553	5
Augusto Corrêa	8 576	8 571	7 643	928	5
Aurora do Pará	5 608	5 607	4 740	867	1
Aveiro	4 134	4 128	3 724	404	6
Bagre	3 414	3 402	2 925	477	12
Baião	6 584	6 566	5 499	1 067	18
Bannach	1 050	1 040	994	46	10
Barcarena	24 659	24 616	20 131	4 485	43
Belterra	3 341	3 337	3 006	331	4
Benevides	13 159	13 147	10 871	2 276	12
Bom Jesus do Tocantins	3 585	3 577	3 326	251	8
Bonito	2 961	2 960	2 654	306	1
Bragança	25 205	25 179	22 532	2 647	26
Brasil Novo	5 027	5 022	4 740	282	5
Brejo Grande do Araguaia	2 195	2 192	1 851	341	3
Breu Branco	12 127	12 102	10 880	1 222	25
Breves	18 623	18 576	17 167	1 409	47
Bujaru	5 303	5 297	4 870	427	6
Cachoeira do Arari	4 202	4 195	3 927	268	7
Cachoeira do Piriá	4 422	4 416	3 800	616	6
Cametá	22 351	22 320	20 127	2 193	31
Canaã dos Carajás	7 664	7 630	6 559	1 071	34
Capanema	16 242	16 211	15 203	1 008	31
Capitão Poço	12 630	12 616	11 797	819	14
Castanhal	43 474	43 414	38 239	5 175	60
Chaves	4 122	4 105	3 735	370	17
Colares	3 207	3 203	2 451	752	4
Conceição do Araguaia	13 696	13 666	12 173	1 493	30
Concórdia do Pará	5 225	5 218	4 722	496	7
Cumaru do Norte	2 609	2 596	2 404	192	13
Curionópolis	6 133	6 105	4 753	1 352	28
Curralinho	5 219	5 209	4 636	573	10
Curuá	2 798	2 793	2 435	358	5
Curuçá	9 242	9 235	7 925	1 310	7
Dom Eliseu	9 818	9 793	9 196	597	25

Tabela 2.1.5 - Domicílios recenseados, por espécie, segundo os municípios - Pará - 2007

(continuação)

Municípios	Domicílios recenseados, por espécie				
	Total	Particulares			Coletivos
		Total	Ocupados (1)	Não-ocupados	
Eldorado dos Carajás	8 541	8 526	7 162	1 364	15
Faro	3 552	3 550	3 469	81	2
Floresta do Araguaia	4 371	4 353	3 803	550	18
Garrafão do Norte	5 894	5 893	5 405	488	1
Goianésia do Pará	7 389	7 388	6 576	812	1
Gurupá	5 086	5 081	4 487	594	5
Igarapé-Açu	9 257	9 244	8 038	1 206	13
Igarapé-Miri	12 300	12 286	10 776	1 510	14
Inhangapi	2 482	2 482	2 183	299	-
Ipixuna do Pará	8 028	8 018	7 623	395	10
Irituia	7 530	7 525	6 669	856	5
Itaituba	27 135	27 076	24 898	2 178	59
Itupiranga	12 416	12 399	10 104	2 295	17
Jacareacanga	5 333	5 289	5 178	111	44
Jacundá	12 010	12 003	11 858	145	7
Juruti	7 392	7 356	6 187	1 169	36
Limoeiro do Ajuru	4 357	4 354	4 117	237	3
Mãe do Rio	7 197	7 183	6 498	685	14
Magalhães Barata	2 019	2 018	1 732	286	1
Maracanã	7 867	7 846	6 446	1 400	21
Marapanim	9 393	9 362	6 354	3 008	31
Marituba	26 131	26 110	24 944	1 166	21
Medicilândia	6 961	6 941	6 036	905	20
Melgaço	3 400	3 393	3 187	206	7
Mocajuba	5 213	5 206	4 507	699	7
Moju	15 067	15 001	13 502	1 499	66
Monte Alegre	17 904	17 884	14 371	3 513	20
Muaná	6 617	6 592	5 776	816	25
Nova Esperança do Piriá	5 191	5 181	4 692	489	10
Nova Ipixuna	3 954	3 946	3 447	499	8
Nova Timboteua	3 540	3 538	3 163	375	2
Novo Progresso	6 823	6 805	6 270	535	18
Novo Repartimento	15 271	15 231	12 284	2 947	40
Óbidos	11 980	11 949	10 347	1 602	31
Oeiras do Pará	5 538	5 526	4 729	797	12
Oriximiná	13 447	13 307	11 706	1 601	140
Ourém	4 047	4 040	3 606	434	7
Ourilândia do Norte	5 314	5 252	4 790	462	62
Pacajá	9 980	9 970	8 822	1 148	10
Palestina do Pará	2 015	2 008	1 827	181	7
Paragominas	24 210	24 160	21 820	2 340	50
Parauapebas	40 493	40 355	35 914	4 441	138
Pau D'Arco	2 015	1 996	1 719	277	19
Peixe-Boi	2 453	2 453	2 034	419	-
Piçarra	3 802	3 786	3 390	396	16

Tabela 2.1.5 - Domicílios recenseados, por espécie, segundo os municípios - Pará - 2007

(conclusão)

Municípios	Domicílios recenseados, por espécie				
	Total	Particulares			Coletivos
		Total	Ocupados (1)	Não-ocupados	
Placas	4 191	4 177	3 888	289	14
Ponta de Pedras	5 727	5 714	5 431	283	13
Portel	9 206	9 174	8 428	746	32
Porto de Moz	5 682	5 665	5 203	462	17
Prainha	6 522	6 496	5 507	989	26
Primavera	2 928	2 924	2 540	384	4
Quatipuru	3 195	3 187	2 899	288	8
Redenção	18 853	18 769	16 863	1 906	84
Rio Maria	5 243	5 224	4 668	556	19
Rondon do Pará	11 114	11 101	10 650	451	13
Rurópolis	8 049	8 027	7 299	728	22
Salinópolis	16 320	16 220	8 421	7 799	100
Salvaterra	5 863	5 834	4 003	1 831	29
Santa Bárbara do Pará	4 136	4 129	3 272	857	7
Santa Cruz do Arari	1 319	1 315	1 198	117	4
Santa Isabel do Pará	14 691	14 675	12 573	2 102	16
Santa Luzia do Pará	4 667	4 652	4 123	529	15
Santa Maria das Barreiras	4 921	4 867	4 463	404	54
Santa Maria do Pará	6 649	6 637	5 808	829	12
Santana do Araguaia	12 768	12 716	11 906	810	52
Santarém Novo	1 613	1 610	1 410	200	3
Santo Antônio do Tauá	7 701	7 698	6 116	1 582	3
São Caetano de Odivelas	4 483	4 476	3 721	755	7
São Domingos do Araguaia	6 150	6 145	5 225	920	5
São Domingos do Capim	6 044	6 036	5 451	585	8
São Félix do Xingu	15 520	15 475	14 223	1 252	45
São Francisco do Pará	3 624	3 622	3 039	583	2
São Geraldo do Araguaia	7 672	7 659	6 359	1 300	13
São João da Ponta	1 498	1 498	1 169	329	-
São João de Pirabas	4 867	4 859	4 383	476	8
São João do Araguaia	3 293	3 292	2 818	474	1
São Miguel do Guamá	11 779	11 768	10 534	1 234	11
São Sebastião da Boa Vista	4 630	4 621	4 135	486	9
Sapucaia	1 644	1 628	1 359	269	16
Senador José Porfírio	3 543	3 522	3 232	290	21
Soure	6 049	6 026	4 855	1 171	23
Tailândia	16 443	16 392	14 451	1 941	51
Terra Alta	2 805	2 804	2 345	459	1
Terra Santa	3 522	3 512	3 134	378	10
Tomé-Açu	11 717	11 695	10 642	1 053	22
Tracuateua	6 273	6 263	5 777	486	10
Trairão	4 128	4 105	3 442	663	23
Tucumã	7 526	7 502	6 823	679	24
Tucuruí	22 980	22 929	20 494	2 435	51
Ulianópolis	7 229	7 113	6 656	457	116
Uruará	9 860	9 848	8 670	1 178	12
Vigia	12 270	12 256	10 145	2 111	14
Viseu	11 876	11 868	11 040	828	8
Vitória do Xingu	2 730	2 729	2 310	419	1
Xinguara	11 485	11 371	10 139	1 232	114

Fonte: IBGE, Contagem da População 2007.

(1) Inclusive os domicílios fechados com população estimada.

Tabela 2.1.6 - Domicílios recenseados, por espécie, segundo os municípios - Amapá - 2007

Municípios	Domicílios recenseados, por espécie				
	Total	Particulares			Coletivos
		Total	Ocupados (1)	Não-ocupados	
**Amapá**	**145 947**	**145 410**	**132 016**	**13 394**	**537**
Amapá	1 885	1 879	1 601	278	6
Calçoene	2 304	2 271	1 999	272	33
Cutias	984	980	856	124	4
Ferreira Gomes	1 551	1 540	1 132	408	11
Itaubal	880	870	696	174	10
Laranjal do Jari	9 976	9 966	8 921	1 045	10
Macapá	86 989	86 744	79 184	7 560	245
Mazagão	3 226	3 214	2 811	403	12
Oiapoque	5 381	5 262	4 167	1 095	119
Pedra Branca do Amapari	1 733	1 730	1 621	109	3
Porto Grande	3 688	3 678	3 273	405	10
Pracuúba	749	745	686	59	4
Santana	19 934	19 924	19 511	413	10
Serra do Navio	1 030	1 022	803	219	8
Tartarugalzinho	2 827	2 794	2 512	282	33
Vitória do Jari	2 810	2 791	2 243	548	19

Fonte: IBGE, Contagem da População 2007.
(1) Inclusive os domicílios fechados com população estimada.

Tabela 2.1.7 - Domicílios recenseados, por espécie, segundo os municípios - Tocantins - 2007

(continua)

Municípios	Domicílios recenseados, por espécie				
	Total	Particulares			Coletivos
		Total	Ocupados (1)	Não-ocupados	
**Tocantins**	**394 440**	**393 556**	**338 986**	**54 570**	**884**
Abreulândia	856	853	644	209	3
Aguiarnópolis	1 112	1 110	1 009	101	2
Aliança do Tocantins	1 999	1 996	1 668	328	3
Almas	2 241	2 240	1 857	383	1
Alvorada	2 808	2 803	2 446	357	5
Ananás	2 706	2 700	2 385	315	6
Angico	1 056	1 053	792	261	3
Aparecida do Rio Negro	1 230	1 228	1 069	159	2
Aragominas	1 647	1 647	1 446	201	-
Araguacema	1 793	1 785	1 476	309	8
Araguaçu	3 701	3 686	2 764	922	15
Araguaína	34 261	34 134	30 362	3 772	127
Araguanã	1 430	1 427	1 217	210	3
Araguatins	7 251	7 203	6 322	881	48
Arapoema	2 175	2 169	1 794	375	6
Arraias	3 623	3 616	2 941	675	7
Augustinópolis	4 441	4 438	3 807	631	3
Aurora do Tocantins	1 145	1 141	1 019	122	4
Axixá do Tocantins	2 647	2 641	2 236	405	6
Babaçulândia	2 541	2 538	2 379	159	3
Bandeirantes do Tocantins	878	875	737	138	3
Barra do Ouro	1 096	1 092	865	227	4
Barrolândia	1 781	1 774	1 528	246	7
Bernardo Sayão	1 683	1 679	1 359	320	4
Bom Jesus do Tocantins	784	784	703	81	-
Brasilândia do Tocantins	713	711	613	98	2
Brejinho de Nazaré	1 734	1 731	1 456	275	3
Buriti do Tocantins	2 247	2 242	1 920	322	5
Cachoeirinha	581	580	523	57	1
Campos Lindos	1 955	1 944	1 623	321	11
Cariri do Tocantins	1 068	1 067	975	92	1
Carmolândia	715	712	662	50	3
Carrasco Bonito	949	946	786	160	3
Caseara	1 497	1 491	1 289	202	6
Centenário	821	819	663	156	2
Chapada da Natividade	1 194	1 193	980	213	1
Chapada de Areia	393	393	355	38	-
Colinas do Tocantins	9 383	9 369	8 328	1 041	14
Colméia	3 101	3 095	2 656	439	6
Combinado	1 583	1 583	1 393	190	-
Conceição do Tocantins	1 308	1 303	1 089	214	5
Couto de Magalhães	1 650	1 647	1 376	271	3
Cristalândia	2 458	2 452	1 969	483	6
Crixás do Tocantins	430	430	366	64	-
Darcinópolis	1 610	1 605	1 321	284	5
Dianópolis	4 981	4 961	4 341	620	20
Divinópolis do Tocantins	2 142	2 141	1 834	307	1
Dois Irmãos do Tocantins	2 087	2 082	1 941	141	5

Tabela 2.1.7 - Domicílios recenseados, por espécie, segundo os municípios - Tocantins - 2007

(continuação)

Municípios	Domicílios recenseados, por espécie				
	Total	Particulares			Coletivos
		Total	Ocupados (1)	Não-ocupados	
Dueré	1 626	1 626	1 361	265	-
Esperantina	2 536	2 534	1 876	658	2
Fátima	1 323	1 322	1 111	211	1
Figueirópolis	1 896	1 892	1 506	386	4
Filadélfia	2 171	2 167	1 921	246	4
Formoso do Araguaia	5 974	5 952	5 109	843	22
Fortaleza do Tabocão	621	621	573	48	-
Goianorte	1 730	1 728	1 481	247	2
Goiatins	3 374	3 360	2 821	539	14
Guaraí	7 110	7 093	6 288	805	17
Gurupi	23 639	23 607	20 666	2 941	32
Ipueiras	596	594	483	111	2
Itacajá	1 722	1 716	1 656	60	6
Itaguatins	2 035	2 030	1 584	446	5
Itapiratins	1 090	1 088	920	168	2
Itaporã do Tocantins	962	959	847	112	3
Jaú do Tocantins	1 444	1 432	1 176	256	12
Juarina	706	704	583	121	2
Lagoa da Confusão	2 280	2 257	2 103	154	23
Lagoa do Tocantins	978	978	765	213	-
Lajeado	690	686	577	109	4
Lavandeira	504	504	430	74	-
Lizarda	1 163	1 163	914	249	-
Luzinópolis	839	839	736	103	-
Marianópolis do Tocantins	1 735	1 733	1 278	455	2
Mateiros	507	502	403	99	5
Maurilândia do Tocantins	1 091	1 089	842	247	2
Miracema do Tocantins	7 064	7 051	5 581	1 470	13
Miranorte	3 720	3 707	3 364	343	13
Monte do Carmo	2 059	2 054	1 747	307	5
Monte Santo do Tocantins	642	634	549	85	8
Muricilândia	1 007	1 006	835	171	1
Natividade	2 826	2 819	2 423	396	7
Nazaré	1 360	1 358	1 194	164	2
Nova Olinda	3 356	3 352	2 876	476	4
Nova Rosalândia	1 322	1 321	1 102	219	1
Novo Acordo	1 065	1 061	915	146	4
Novo Alegre	580	579	514	65	1
Novo Jardim	794	794	613	181	-
Oliveira de Fátima	385	385	310	75	-
Palmas	57 981	57 929	51 933	5 996	52
Palmeirante	1 408	1 404	1 208	196	4
Palmeiras do Tocantins	1 487	1 487	1 147	340	-
Palmeirópolis	2 663	2 653	2 313	340	10
Paraíso do Tocantins	13 429	13 395	11 613	1 782	34
Paranã	3 336	3 332	2 899	433	4
Pau D'Arco	1 425	1 418	1 254	164	7

Tabela 2.1.7 - Domicílios recenseados, por espécie, segundo os municípios - Tocantins - 2007

(conclusão)

Municípios	Domicílios recenseados, por espécie				
	Total	Particulares			Coletivos
		Total	Ocupados (1)	Não-ocupados	
Pedro Afonso	3 106	3 090	2 743	347	16
Peixe	3 529	3 515	2 625	890	14
Pequizeiro	1 616	1 615	1 355	260	1
Pindorama do Tocantins	1 593	1 590	1 125	465	3
Piraquê	965	965	868	97	-
Pium	2 354	2 345	1 834	511	9
Ponte Alta do Bom Jesus	1 444	1 444	1 225	219	-
Ponte Alta do Tocantins	2 108	2 100	1 731	369	8
Porto Alegre do Tocantins	786	784	702	82	2
Porto Nacional	14 013	13 989	12 065	1 924	24
Praia Norte	2 013	2 011	1 628	383	2
Presidente Kennedy	1 115	1 111	996	115	4
Pugmil	758	758	628	130	-
Recursolândia	1 004	1 001	873	128	3
Riachinho	1 155	1 155	916	239	-
Rio da Conceição	476	474	354	120	2
Rio dos Bois	578	578	559	19	-
Rio Sono	1 788	1 781	1 596	185	7
Sampaio	1 082	1 082	904	178	-
Sandolândia	1 401	1 396	1 106	290	5
Santa Fé do Araguaia	1 641	1 629	1 401	228	12
Santa Maria do Tocantins	807	807	694	113	-
Santa Rita do Tocantins	804	803	707	96	1
Santa Rosa do Tocantins	1 541	1 541	1 155	386	-
Santa Tereza do Tocantins	766	766	590	176	-
Santa Terezinha do Tocantins	693	693	585	108	-
São Bento do Tocantins	1 073	1 072	1 014	58	1
São Félix do Tocantins	380	379	337	42	1
São Miguel do Tocantins	2 894	2 891	2 513	378	3
São Salvador do Tocantins	1 071	1 061	813	248	10
São Sebastião do Tocantins	1 219	1 218	1 010	208	1
São Valério da Natividade	1 912	1 909	1 427	482	3
Silvanópolis	1 824	1 820	1 444	376	4
Sítio Novo do Tocantins	2 625	2 625	2 322	303	-
Sucupira	595	594	485	109	1
Taguatinga	4 820	4 816	3 937	879	4
Taipas do Tocantins	532	532	441	91	-
Talismã	912	912	799	113	-
Tocantínia	1 886	1 884	1 672	212	2
Tocantinópolis	6 342	6 328	5 651	677	14
Tupirama	441	441	414	27	-
Tupiratins	733	732	606	126	1
Wanderlândia	3 067	3 059	2 597	462	8
Xambioá	3 242	3 236	2 855	381	6

Fonte: IBGE, Contagem da População 2007.

(1) Inclusive os domicílios fechados e os domicílios provenientes de setor censitário cujo arquivo foi danificado, com população estimada.

Tabela 2.1.8 - Domicílios recenseados, por espécie, segundo os municípios - Maranhão - 2007

(continua)

Municípios	Domicílios recenseados, por espécie				
	Total	Particulares			Coletivos
		Total	Ocupados (1)	Não-ocupados	
**Maranhão**	**1 671 932**	**1 669 738**	**1 483 838**	**185 900**	**2 194**
Açailândia	26 393	26 347	24 011	2 336	46
Afonso Cunha	1 400	1 399	1 192	207	1
Água Doce do Maranhão	2 810	2 807	2 631	176	3
Alcântara	6 766	6 760	5 703	1 057	6
Aldeias Altas	5 901	5 893	5 074	819	8
Altamira do Maranhão	1 926	1 924	1 775	149	2
Alto Alegre do Maranhão	6 049	6 044	5 305	739	5
Alto Alegre do Pindaré	7 418	7 410	6 953	457	8
Alto Parnaíba	2 948	2 941	2 430	511	7
Amapá do Maranhão	1 635	1 630	1 341	289	5
Amarante do Maranhão	9 671	9 651	8 593	1 058	20
Anajatuba	6 262	6 256	5 983	273	6
Anapurus	3 241	3 239	2 795	444	2
Apicum-Açu	3 311	3 310	2 956	354	1
Araguanã	2 667	2 667	2 237	430	-
Araioses	9 885	9 872	8 740	1 132	13
Arame	7 213	7 206	6 129	1 077	7
Arari	7 314	7 309	6 523	786	5
Axixá	2 806	2 804	2 592	212	2
Bacabal	26 640	26 608	24 150	2 458	32
Bacabeira	3 580	3 578	3 328	250	2
Bacuri	4 583	4 573	3 837	736	10
Bacurituba	1 420	1 420	1 290	130	-
Balsas	22 105	22 068	19 343	2 725	37
Barão de Grajaú	5 151	5 149	4 304	845	2
Barra do Corda	22 624	22 610	19 325	3 285	14
Barreirinhas	12 233	12 154	10 164	1 990	79
Bela Vista do Maranhão	2 295	2 295	2 052	243	-
Belágua	1 404	1 403	1 120	283	1
Benedito Leite	1 806	1 805	1 390	415	1
Bequimão	5 895	5 891	5 329	562	4
Bernardo do Mearim	1 716	1 715	1 494	221	1
Boa Vista do Gurupi	1 878	1 871	1 615	256	7
Bom Jardim	9 616	9 605	8 716	889	11
Bom Jesus das Selvas	6 281	6 274	5 505	769	7
Bom Lugar	3 283	3 282	3 024	258	1
Brejo	8 207	8 200	7 236	964	7
Brejo de Areia	1 438	1 438	1 259	179	-
Buriti	6 489	6 484	5 587	897	5
Buriti Bravo	6 727	6 717	5 451	1 266	10
Buriticupu	15 138	15 110	13 401	1 709	28
Buritirana	3 625	3 625	3 015	610	-
Cachoeira Grande	2 088	2 088	1 864	224	-
Cajapió	2 422	2 418	2 264	154	4
Cajari	3 316	3 315	2 999	316	1
Campestre do Maranhão	3 627	3 625	3 063	562	2
Cândido Mendes	4 756	4 738	4 171	567	18
Cantanhede	5 158	5 153	4 506	647	5
Capinzal do Norte	3 019	3 019	2 722	297	-

Tabela 2.1.8 - Domicílios recenseados, por espécie, segundo os municípios - Maranhão - 2007

(continuação)

Municípios	Domicílios recenseados, por espécie				
	Total	Particulares			Coletivos
		Total	Ocupados (1)	Não-ocupados	
Carolina	6 681	6 657	6 120	537	24
Carutapera	4 472	4 465	4 281	184	7
Caxias	39 899	39 871	35 663	4 208	28
Cedral	2 715	2 710	2 393	317	5
Central do Maranhão	2 612	2 610	2 179	431	2
Centro do Guilherme	1 811	1 811	1 537	274	-
Centro Novo do Maranhão	3 849	3 843	3 289	554	6
Chapadinha	18 547	18 530	16 090	2 440	17
Cidelândia	3 371	3 371	3 086	285	-
Codó	29 669	29 640	26 990	2 650	29
Coelho Neto	11 596	11 580	10 106	1 474	16
Colinas	10 584	10 573	8 750	1 823	11
Conceição do Lago-Açu	3 498	3 498	3 092	406	-
Coroatá	16 263	16 243	15 405	838	20
Cururupu	9 235	9 221	8 362	859	14
Davinópolis	3 574	3 571	2 945	626	3
Dom Pedro	6 281	6 275	5 639	636	6
Duque Bacelar	2 609	2 604	2 241	363	5
Esperantinópolis	5 110	5 109	4 638	471	1
Estreito	7 978	7 969	6 633	1 336	9
Feira Nova do Maranhão	1 848	1 841	1 752	89	7
Fernando Falcão	1 782	1 782	1 647	135	-
Formosa da Serra Negra	3 666	3 660	3 516	144	6
Fortaleza dos Nogueiras	2 915	2 913	2 687	226	2
Fortuna	4 395	4 390	3 650	740	5
Godofredo Viana	2 545	2 536	2 454	82	9
Gonçalves Dias	5 058	5 055	4 261	794	3
Governador Archer	2 941	2 939	2 441	498	2
Governador Edison Lobão	4 227	4 224	3 490	734	3
Governador Eugênio Barros	4 709	4 708	3 999	709	1
Governador Luiz Rocha	2 052	2 051	1 689	362	1
Governador Newton Bello	2 853	2 851	2 609	242	2
Governador Nunes Freire	6 132	6 125	5 366	759	7
Graça Aranha	1 895	1 894	1 671	223	1
Grajaú	14 308	14 289	12 379	1 910	19
Guimarães	3 734	3 731	3 207	524	3
Humberto de Campos	5 789	5 788	4 940	848	1
Icatu	5 956	5 951	5 259	692	5
Igarapé do Meio	3 112	3 110	2 730	380	2
Igarapé Grande	2 912	2 912	2 678	234	-
Imperatriz	68 881	68 843	60 648	8 195	38
Itaipava do Grajaú	3 126	3 121	2 907	214	5
Itapecuru Mirim	14 933	14 908	13 203	1 705	25
Itinga do Maranhão	7 194	7 170	6 445	725	24
Jatobá	2 337	2 336	1 968	368	1
Jenipapo dos Vieiras	3 790	3 785	3 366	419	5
João Lisboa	5 702	5 699	5 008	691	3
Joselândia	4 229	4 225	3 756	469	4
Junco do Maranhão	1 083	1 080	943	137	3
Lago da Pedra	11 674	11 662	10 293	1 369	12
Lago do Junco	2 660	2 659	2 288	371	1
Lago dos Rodrigues	2 337	2 337	1 988	349	-

Tabela 2.1.8 - Domicílios recenseados, por espécie, segundo os municípios - Maranhão - 2007

(continuação)

Municípios	Domicílios recenseados, por espécie				
	Total	Particulares			Coletivos
		Total	Ocupados (1)	Não-ocupados	
Lago Verde	3 621	3 620	3 200	420	1
Lagoa do Mato	2 929	2 927	2 434	493	2
Lagoa Grande do Maranhão	2 347	2 345	2 034	311	2
Lajeado Novo	1 841	1 840	1 620	220	1
Lima Campos	3 567	3 566	3 036	530	1
Loreto	2 922	2 917	2 390	527	5
Luís Domingues	1 697	1 695	1 645	50	2
Magalhães de Almeida	3 829	3 820	3 261	559	9
Maracaçumé	4 692	4 688	4 004	684	4
Marajá do Sena	1 708	1 706	1 555	151	2
Maranhãozinho	2 658	2 657	2 436	221	1
Mata Roma	3 839	3 838	3 126	712	1
Matinha	5 414	5 411	4 948	463	3
Matões	7 667	7 657	6 663	994	10
Matões do Norte	2 259	2 259	2 123	136	-
Milagres do Maranhão	1 882	1 879	1 637	242	3
Mirador	5 509	5 505	4 612	893	4
Miranda do Norte	4 576	4 570	4 239	331	6
Mirinzal	3 666	3 661	3 237	424	5
Monção	7 328	7 326	6 276	1 050	2
Montes Altos	2 561	2 558	2 209	349	3
Morros	4 014	4 002	3 430	572	12
Nina Rodrigues	2 582	2 579	2 156	423	3
Nova Colinas	1 224	1 224	1 113	111	-
Nova Iorque	1 354	1 354	1 212	142	-
Nova Olinda do Maranhão	4 187	4 186	3 767	419	1
Olho d'Água das Cunhãs	4 919	4 916	4 386	530	3
Olinda Nova do Maranhão	3 050	3 050	2 816	234	-
Paço do Lumiar	27 441	27 415	25 053	2 362	26
Palmeirândia	4 870	4 867	4 416	451	3
Paraibano	5 569	5 564	4 929	635	5
Parnarama	9 714	9 708	8 341	1 367	6
Passagem Franca	5 512	5 512	4 333	1 179	-
Pastos Bons	5 231	5 230	4 264	966	1
Paulino Neves	2 893	2 892	2 484	408	1
Paulo Ramos	4 282	4 276	3 756	520	6
Pedreiras	10 984	10 969	9 917	1 052	15
Pedro do Rosário	5 665	5 661	5 066	595	4
Penalva	8 478	8 472	7 718	754	6
Peri Mirim	3 650	3 650	3 247	403	-
Peritoró	5 502	5 498	4 851	647	4
Pindaré-Mirim	7 844	7 833	7 286	547	11
Pinheiro	19 320	19 265	17 601	1 664	55
Pio XII	5 695	5 685	5 060	625	10
Pirapemas	4 484	4 481	3 626	855	3
Poção de Pedras	4 623	4 620	4 167	453	3
Porto Franco	5 159	5 133	4 628	505	26
Porto Rico do Maranhão	1 785	1 781	1 583	198	4
Presidente Dutra	11 541	11 524	10 441	1 083	17
Presidente Juscelino	2 786	2 786	2 389	397	-
Presidente Médici	1 401	1 398	1 301	97	3
Presidente Sarney	4 153	4 141	3 603	538	12
Presidente Vargas	2 483	2 483	2 225	258	-
Primeira Cruz	2 793	2 791	2 477	314	2
Raposa	6 907	6 894	5 862	1 032	13
Riachão	5 603	5 602	4 987	615	1
Ribamar Fiquene	2 184	2 179	1 801	378	5
Rosário	9 491	9 480	8 622	858	11

Tabela 2.1.8 - Domicílios recenseados, por espécie, segundo os municípios - Maranhão - 2007

(conclusão)

Municípios	Domicílios recenseados, por espécie				
	Total	Particulares			Coletivos
		Total	Ocupados (1)	Não-ocupados	
Sambaíba	1 444	1 442	1 292	150	2
Santa Filomena do Maranhão	1 583	1 582	1 319	263	1
Santa Helena	8 410	8 404	7 796	608	6
Santa Inês	21 634	21 588	19 981	1 607	46
Santa Luzia	17 828	17 809	15 696	2 113	19
Santa Luzia do Paruá	5 042	5 034	4 480	554	8
Santa Quitéria do Maranhão	6 871	6 868	5 943	925	3
Santa Rita	7 642	7 630	7 187	443	12
Santana do Maranhão	2 349	2 349	2 143	206	-
Santo Amaro do Maranhão	2 421	2 417	2 201	216	4
Santo Antônio dos Lopes	4 181	4 180	3 638	542	1
São Benedito do Rio Preto	4 265	4 264	3 648	616	1
São Bento	9 280	9 278	8 481	797	2
São Bernardo	6 435	6 429	5 700	729	6
São Domingos do Azeitão	2 022	2 019	1 611	408	3
São Domingos do Maranhão	9 403	9 399	8 237	1 162	4
São Félix de Balsas	1 303	1 303	1 115	188	-
São Francisco do Brejão	2 492	2 491	2 174	317	1
São Francisco do Maranhão	3 932	3 931	3 415	516	1
São João Batista	4 841	4 835	4 529	306	6
São João do Carú	2 940	2 927	2 482	445	13
São João do Paraíso	3 068	3 067	2 680	387	1
São João do Soter	5 231	5 230	4 023	1 207	1
São João dos Patos	7 649	7 640	6 373	1 267	9
São José de Ribamar	37 167	37 143	32 437	4 706	24
São José dos Basílios	1 962	1 961	1 717	244	1
São Luís	275 380	274 819	249 445	25 374	561
São Luís Gonzaga do Maranhão	5 772	5 768	4 934	834	4
São Mateus do Maranhão	10 284	10 273	9 066	1 207	11
São Pedro da Água Branca	3 020	3 005	2 662	343	15
São Pedro dos Crentes	1 069	1 068	951	117	1
São Raimundo das Mangabeiras	4 704	4 685	3 839	846	19
São Raimundo do Doca Bezerra	1 225	1 222	1 016	206	3
São Roberto	1 306	1 306	1 120	186	-
São Vicente Ferrer	5 184	5 181	4 742	439	3
Satubinha	2 231	2 231	1 909	322	-
Senador Alexandre Costa	2 797	2 792	2 232	560	5
Senador La Rocque	5 815	5 811	5 096	715	4
Serrano do Maranhão	3 138	3 126	2 620	506	12
Sítio Novo	4 236	4 203	3 581	622	33
Sucupira do Norte	3 036	3 032	2 554	478	4
Sucupira do Riachão	1 388	1 387	1 156	231	1
Tasso Fragoso	1 779	1 773	1 560	213	6
Timbiras	6 528	6 525	6 153	372	3
Timon	39 097	39 062	35 853	3 209	35
Trizidela do Vale	5 216	5 212	4 737	475	4
Tufilândia	1 514	1 506	1 277	229	8
Tuntum	11 535	11 530	9 746	1 784	5
Turiaçu	8 298	8 288	7 376	912	10
Turilândia	4 974	4 972	4 478	494	2
Tutóia	10 721	10 711	9 627	1 084	10
Urbano Santos	5 346	5 333	4 560	773	13
Vargem Grande	10 511	10 501	9 642	859	10
Viana	12 128	12 102	11 127	975	26
Vila Nova dos Martírios	2 667	2 663	2 237	426	4
Vitória do Mearim	7 837	7 827	7 132	695	10
Vitorino Freire	8 786	8 780	7 661	1 119	6
Zé Doca	11 865	11 852	10 688	1 164	13

Fonte: IBGE, Contagem da População 2007.

(1) Inclusive os domicílios fechados com população estimada.

Tabela 2.1.9 - Domicílios recenseados, por espécie, segundo os municípios - Piauí - 2007

(continua)

Municípios	Domicílios recenseados, por espécie				
	Total	Particulares			Coletivos
		Total	Ocupados (1)	Não-ocupados	
**Piauí**	**941 147**	**939 964**	**788 521**	**151 443**	**1 183**
Acauã	1 843	1 843	1 728	115	-
Agricolândia	1 876	1 876	1 377	499	-
Água Branca	4 898	4 894	4 327	567	4
Alagoinha do Piauí	3 201	3 196	2 216	980	5
Alegrete do Piauí	1 453	1 451	1 190	261	2
Alto Longá	4 349	4 348	3 691	657	1
Altos	11 536	11 530	9 835	1 695	6
Alvorada do Gurguéia	1 609	1 605	1 197	408	4
Amarante	5 899	5 897	4 819	1 078	2
Angical do Piauí	2 431	2 429	1 977	452	2
Anísio de Abreu	2 845	2 843	2 224	619	2
Antônio Almeida	1 098	1 096	857	239	2
Aroazes	1 805	1 803	1 518	285	2
Aroeiras do Itaim	835	835	709	126	-
Arraial	1 616	1 615	1 288	327	1
Assunção do Piauí	3 083	3 083	1 872	1 211	-
Avelino Lopes	3 463	3 462	3 053	409	1
Baixa Grande do Ribeiro	2 714	2 700	2 281	419	14
Barra D'Alcântara	1 348	1 348	1 101	247	-
Barras	11 878	11 865	10 468	1 397	13
Barreiras do Piauí	912	912	753	159	-
Barro Duro	2 132	2 131	1 787	344	1
Batalha	7 701	7 694	6 462	1 232	7
Bela Vista do Piauí	909	909	782	127	-
Belém do Piauí	947	946	780	166	1
Beneditinos	3 208	3 198	2 582	616	10
Bertolínia	1 744	1 742	1 410	332	2
Betânia do Piauí	3 272	3 271	2 297	974	1
Boa Hora	1 597	1 597	1 430	167	-
Bocaina	1 878	1 877	1 254	623	1
Bom Jesus	5 736	5 729	4 763	966	7
Bom Princípio do Piauí	1 462	1 461	1 276	185	1
Bonfim do Piauí	1 781	1 780	1 384	396	1
Boqueirão do Piauí	1 761	1 761	1 626	135	-
Brasileira	2 426	2 425	1 964	461	1
Brejo do Piauí	1 247	1 247	913	334	-
Buriti dos Lopes	5 467	5 466	4 650	816	1
Buriti dos Montes	2 444	2 442	1 954	488	2
Cabeceiras do Piauí	2 499	2 498	2 289	209	1
Cajazeiras do Piauí	1 100	1 096	869	227	4
Cajueiro da Praia	2 061	2 049	1 686	363	12
Caldeirão Grande do Piauí	1 923	1 923	1 450	473	-
Campinas do Piauí	1 984	1 984	1 580	404	-
Campo Alegre do Fidalgo	1 534	1 534	1 164	370	-
Campo Grande do Piauí	1 835	1 833	1 573	260	2
Campo Largo do Piauí	1 842	1 842	1 592	250	-
Campo Maior	13 613	13 598	12 176	1 422	15
Canavieira	1 445	1 444	1 071	373	1
Canto do Buriti	7 254	7 237	5 756	1 481	17
Capitão de Campos	3 301	3 298	2 777	521	3
Capitão Gervásio Oliveira	1 424	1 423	1 092	331	1
Caracol	3 092	3 090	2 551	539	2
Caraúbas do Piauí	1 545	1 544	1 341	203	1
Caridade do Piauí	1 414	1 414	1 106	308	-
Castelo do Piauí	5 644	5 637	4 916	721	7
Caxingó	1 357	1 355	1 176	179	2
Cocal	7 344	7 337	6 313	1 024	7
Cocal de Telha	1 430	1 430	1 241	189	-

Tabela 2.1.9 - Domicílios recenseados, por espécie, segundo os municípios - Piauí - 2007

(continuação)

Municípios	Domicílios recenseados, por espécie				
	Total	Particulares			Coletivos
		Total	Ocupados (1)	Não-ocupados	
Cocal dos Alves	1 628	1 626	1 395	231	2
Coivaras	1 192	1 192	967	225	-
Colônia do Gurguéia	1 931	1 931	1 496	435	-
Colônia do Piauí	2 352	2 352	1 947	405	-
Conceição do Canindé	1 644	1 641	1 339	302	3
Coronel José Dias	1 490	1 487	1 218	269	3
Corrente	7 255	7 241	6 070	1 171	14
Cristalândia do Piauí	2 142	2 140	1 857	283	2
Cristino Castro	2 982	2 971	2 396	575	11
Curimatá	3 831	3 827	2 629	1 198	4
Currais	1 512	1 512	1 081	431	-
Curral Novo do Piauí	1 351	1 350	1 132	218	1
Curralinhos	1 256	1 256	1 052	204	-
Demerval Lobão	4 238	4 236	3 488	748	2
Dirceu Arcoverde	2 190	2 189	1 780	409	1
Dom Expedito Lopes	2 110	2 108	1 840	268	2
Dom Inocêncio	3 313	3 313	2 550	763	-
Domingos Mourão	1 357	1 357	1 048	309	-
Elesbão Veloso	5 232	5 231	4 019	1 212	1
Eliseu Martins	1 512	1 507	1 305	202	5
Esperantina	10 709	10 700	9 222	1 478	9
Fartura do Piauí	1 643	1 642	1 307	335	1
Flores do Piauí	1 675	1 675	1 281	394	-
Floresta do Piauí	971	971	736	235	-
Floriano	17 051	17 018	14 888	2 130	33
Francinópolis	2 007	2 006	1 516	490	1
Francisco Ayres	1 607	1 605	1 410	195	2
Francisco Macedo	800	800	611	189	-
Francisco Santos	2 912	2 910	2 331	579	2
Fronteiras	3 769	3 769	2 972	797	-
Geminiano	1 953	1 953	1 451	502	-
Gilbués	2 886	2 880	2 386	494	6
Guadalupe	2 961	2 944	2 536	408	17
Guaribas	1 213	1 211	1 019	192	2
Hugo Napoleão	1 266	1 263	1 011	252	3
Ilha Grande	2 203	2 203	1 990	213	-
Inhuma	5 105	5 102	4 047	1 055	3
Ipiranga do Piauí	2 984	2 980	2 448	532	4
Isaías Coelho	2 654	2 654	1 993	661	-
Itainópolis	4 256	4 252	3 225	1 027	4
Itaueira	3 735	3 731	2 913	818	4
Jacobina do Piauí	2 097	2 096	1 507	589	1
Jaicós	6 311	6 310	4 521	1 789	1
Jardim do Mulato	1 396	1 395	1 141	254	1
Jatobá do Piauí	1 405	1 404	1 213	191	1
Jerumenha	1 669	1 669	1 188	481	-
João Costa	1 040	1 039	818	221	1
Joaquim Pires	3 645	3 644	3 243	401	1
Joca Marques	1 582	1 582	1 260	322	-
José de Freitas	9 623	9 617	8 350	1 267	6
Juazeiro do Piauí	1 405	1 403	1 138	265	2
Júlio Borges	1 599	1 599	1 285	314	-
Jurema	1 468	1 468	1 155	313	-
Lagoa Alegre	2 193	2 187	1 885	302	6
Lagoa de São Francisco	1 887	1 886	1 557	329	1
Lagoa do Barro do Piauí	1 575	1 575	1 139	436	-

Tabela 2.1.9 - Domicílios recenseados, por espécie, segundo os municípios - Piauí - 2007

(continuação)

Municípios	Domicílios recenseados, por espécie				
	Total	Particulares			Coletivos
		Total	Ocupados (1)	Não-ocupados	
Lagoa do Piauí	1 193	1 193	946	247	-
Lagoa do Sítio	2 068	2 067	1 446	621	1
Lagoinha do Piauí	929	929	750	179	-
Landri Sales	2 303	2 301	1 632	669	2
Luís Correia	9 862	9 840	6 158	3 682	22
Luzilândia	6 614	6 607	5 992	615	7
Madeiro	1 867	1 865	1 682	183	2
Manoel Emídio	1 738	1 735	1 392	343	3
Marcolândia	2 203	2 201	1 896	305	2
Marcos Parente	1 541	1 536	1 156	380	5
Massapê do Piauí	1 869	1 868	1 641	227	1
Matias Olímpio	2 963	2 962	2 517	445	1
Miguel Alves	7 947	7 944	7 354	590	3
Miguel Leão	359	359	309	50	-
Milton Brandão	2 298	2 297	1 687	610	1
Monsenhor Gil	3 303	3 299	2 704	595	4
Monsenhor Hipólito	2 781	2 780	2 042	738	1
Monte Alegre do Piauí	2 756	2 753	2 376	377	3
Morro Cabeça no Tempo	1 152	1 151	980	171	1
Morro do Chapéu do Piauí	1 970	1 970	1 520	450	-
Murici dos Portelas	1 975	1 975	1 752	223	-
Nazaré do Piauí	2 287	2 286	1 837	449	1
Nossa Senhora de Nazaré	1 254	1 254	1 092	162	-
Nossa Senhora dos Remédios	2 211	2 208	1 942	266	3
Nova Santa Rita	1 316	1 314	1 087	227	2
Novo Oriente do Piauí	2 223	2 222	1 765	457	1
Novo Santo Antônio	1 117	1 117	881	236	-
Oeiras	11 951	11 939	9 786	2 153	12
Olho D'Água do Piauí	907	907	726	181	-
Padre Marcos	2 628	2 627	2 137	490	1
Paes Landim	1 456	1 454	1 080	374	2
Pajeú do Piauí	1 472	1 470	1 047	423	2
Palmeira do Piauí	1 636	1 635	1 226	409	1
Palmeirais	4 473	4 469	3 704	765	4
Paquetá	1 423	1 423	1 164	259	-
Parnaguá	2 663	2 658	2 282	376	5
Parnaíba	39 276	39 190	35 246	3 944	86
Passagem Franca do Piauí	1 275	1 275	1 015	260	-
Patos do Piauí	2 438	2 432	1 752	680	6
Pau D'Arco do Piauí	1 125	1 122	936	186	3
Paulistana	6 165	6 160	4 637	1 523	5
Pavussu	1 723	1 719	1 106	613	4
Pedro II	11 199	11 192	9 034	2 158	7
Pedro Laurentino	793	793	566	227	-
Picos	22 433	22 397	19 544	2 853	36
Pimenteiras	4 269	4 266	3 033	1 233	3
Pio IX	6 057	6 053	4 532	1 521	4
Piracuruca	8 435	8 424	6 912	1 512	11
Piripiri	17 864	17 847	16 067	1 780	17
Porto	2 896	2 895	2 624	271	1
Porto Alegre do Piauí	864	862	634	228	2
Prata do Piauí	1 095	1 094	828	266	1
Queimada Nova	2 928	2 918	2 284	634	10
Redenção do Gurguéia	2 239	2 236	1 895	341	3
Regeneração	5 830	5 822	4 730	1 092	8
Riacho Frio	1 521	1 520	1 214	306	1

Tabela 2.1.9 - Domicílios recenseados, por espécie, segundo os municípios - Piauí - 2007

(conclusão)

Municípios	Domicílios recenseados, por espécie				
	Total	Particulares			Coletivos
		Total	Ocupados (1)	Não-ocupados	
Ribeira do Piauí	1 478	1 478	1 069	409	-
Ribeiro Gonçalves	1 882	1 870	1 524	346	12
Rio Grande do Piauí	2 873	2 871	1 836	1 035	2
Santa Cruz do Piauí	1 897	1 897	1 583	314	-
Santa Cruz dos Milagres	1 267	1 264	881	383	3
Santa Filomena	1 656	1 655	1 355	300	1
Santa Luz	1 690	1 688	1 332	356	2
Santa Rosa do Piauí	1 817	1 816	1 449	367	1
Santana do Piauí	1 598	1 597	1 307	290	1
Santo Antônio de Lisboa	2 205	2 204	1 648	556	1
Santo Antônio dos Milagres	571	570	489	81	1
Santo Inácio do Piauí	1 385	1 384	1 034	350	1
São Braz do Piauí	1 544	1 544	1 146	398	-
São Félix do Piauí	1 132	1 130	917	213	2
São Francisco de Assis do Piauí	1 588	1 586	1 291	295	2
São Francisco do Piauí	2 279	2 275	1 742	533	4
São Gonçalo do Gurguéia	658	658	583	75	-
São Gonçalo do Piauí	1 449	1 449	1 199	250	-
São João da Canabrava	1 638	1 638	1 256	382	-
São João da Fronteira	1 505	1 501	1 186	315	4
São João da Serra	2 198	2 196	1 806	390	2
São João da Varjota	1 471	1 470	1 228	242	1
São João do Arraial	2 360	2 359	1 825	534	1
São João do Piauí	6 323	6 318	4 953	1 365	5
São José do Divino	1 606	1 604	1 322	282	2
São José do Peixe	1 400	1 397	1 025	372	3
São José do Piauí	2 663	2 660	1 861	799	3
São Julião	2 283	2 280	1 739	541	3
São Lourenço do Piauí	1 562	1 562	1 393	169	-
São Luis do Piauí	987	987	730	257	-
São Miguel da Baixa Grande	748	748	579	169	-
São Miguel do Fidalgo	1 023	1 023	771	252	-
São Miguel do Tapuio	6 083	6 079	4 996	1 083	4
São Pedro do Piauí	4 197	4 196	3 530	666	1
São Raimundo Nonato	10 542	10 522	8 593	1 929	20
Sebastião Barros	1 142	1 139	1 014	125	3
Sebastião Leal	1 211	1 209	1 081	128	2
Sigefredo Pacheco	3 185	3 185	2 609	576	-
Simões	4 333	4 326	3 327	999	7
Simplício Mendes	3 658	3 644	3 118	526	14
Socorro do Piauí	1 574	1 572	1 224	348	2
Sussuapara	1 913	1 913	1 560	353	-
Tamboril do Piauí	1 039	1 039	768	271	-
Tanque do Piauí	880	877	745	132	3
Teresina	229 889	229 487	204 791	24 696	402
União	11 194	11 179	9 982	1 197	15
Uruçuí	6 135	6 127	4 855	1 272	8
Valença do Piauí	7 084	7 080	5 637	1 443	4
Várzea Branca	1 534	1 533	1 273	260	1
Várzea Grande	1 772	1 770	1 355	415	2
Vera Mendes	1 337	1 337	939	398	-
Vila Nova do Piauí	1 326	1 326	869	457	-
Wall Ferraz	1 432	1 430	1 103	327	2

Fonte: IBGE, Contagem da População 2007.
(1) Inclusive os domicílios fechados com população estimada.

Tabela 2.1.10 - Domicílios recenseados, por espécie, segundo os municípios - Ceará - 2007

(continua)

Municípios	Domicílios recenseados, por espécie				
	Total	Particulares			Coletivos
		Total	Ocupados (1)	Não-ocupados	
**Ceará**	**1 538 211**	**1 536 744**	**1 262 883**	**273 861**	**1 467**
Abaiara	3 037	3 035	2 541	494	2
Acarape	4 439	4 439	3 739	700	-
Acaraú	14 944	14 922	12 605	2 317	22
Acopiara	16 954	16 943	13 790	3 153	11
Aiuaba	5 477	5 475	4 139	1 336	2
Alcântaras	3 298	3 297	2 654	643	1
Altaneira	2 331	2 331	1 877	454	-
Alto Santo	5 326	5 326	4 464	862	-
Amontada	10 312	10 301	8 863	1 438	11
Antonina do Norte	2 456	2 455	1 880	575	1
Apuiarés	4 738	4 736	3 550	1 186	2
Aquiraz	23 762	23 713	17 922	5 791	49
Aracati	21 490	21 410	17 713	3 697	80
Aracoiaba	8 691	8 688	6 596	2 092	3
Ararendá	3 660	3 656	2 928	728	4
Araripe	6 276	6 272	5 431	841	4
Aratuba	3 431	3 428	2 912	516	3
Arneiroz	2 564	2 561	1 936	625	3
Assaré	7 601	7 601	5 750	1 851	-
Aurora	8 432	8 427	6 707	1 720	5
Baixio	1 953	1 952	1 585	367	1
Banabuiú	5 079	5 073	4 389	684	6
Barbalha	14 563	14 556	12 603	1 953	7
Barreira	6 080	6 080	4 980	1 100	-
Barro	7 121	7 118	5 573	1 545	3
Barroquinha	4 364	4 361	3 507	854	3
Baturité	9 900	9 881	8 286	1 595	19
Beberibe	17 123	17 093	12 316	4 777	30
Bela Cruz	8 783	8 778	7 231	1 547	5
Boa Viagem	17 868	17 855	14 490	3 365	13
Brejo Santo	12 207	12 195	10 478	1 717	12
Camocim	16 775	16 736	14 425	2 311	39
Campos Sales	8 742	8 731	6 937	1 794	11
Canindé	22 895	22 860	18 763	4 097	35
Capistrano	4 998	4 993	4 144	849	5
Caridade	5 689	5 688	4 439	1 249	1
Cariré	6 329	6 327	4 901	1 426	2
Caririaçu	8 668	8 665	6 594	2 071	3
Cariús	6 749	6 748	5 260	1 488	1
Carnaubal	4 926	4 920	4 092	828	6
Cascavel	21 309	21 284	17 301	3 983	25
Catarina	4 651	4 651	4 363	288	-
Catunda	3 284	3 283	2 683	600	1
Cedro	8 779	8 778	6 894	1 884	1
Chaval	3 607	3 605	2 962	643	2
Choró	3 691	3 688	3 052	636	3
Chorozinho	6 403	6 401	5 059	1 342	2
Coreaú	7 294	7 293	5 419	1 874	1

Tabela 2.1.10 - Domicílios recenseados, por espécie, segundo os municípios - Ceará - 2007

(continuação)

Municípios	Domicílios recenseados, por espécie				
	Total	Particulares			Coletivos
		Total	Ocupados (1)	Não-ocupados	
Crateús	24 652	24 632	20 488	4 144	20
Crato	34 000	33 971	29 919	4 052	29
Croatá	5 956	5 952	4 559	1 393	4
Cruz	6 841	6 830	5 660	1 170	11
Deputado Irapuan Pinheiro	3 303	3 303	2 658	645	-
Ererê	2 260	2 260	1 763	497	-
Eusébio	11 216	11 209	10 066	1 143	7
Farias Brito	6 657	6 656	5 211	1 445	1
Forquilha	6 544	6 542	5 452	1 090	2
Fortim	4 781	4 773	3 792	981	8
Frecheirinha	4 283	4 280	3 493	787	3
General Sampaio	1 791	1 791	1 455	336	-
Graça	5 173	5 170	4 001	1 169	3
Granja	15 153	15 143	12 297	2 846	10
Granjeiro	1 544	1 544	1 190	354	-
Groaíras	3 314	3 310	2 696	614	4
Guaiúba	6 750	6 748	5 715	1 033	2
Guaraciaba do Norte	11 236	11 226	9 360	1 866	10
Guaramiranga	1 588	1 575	1 029	546	13
Hidrolândia	6 390	6 387	4 999	1 388	3
Horizonte	15 875	15 872	13 172	2 700	3
Ibaretama	3 994	3 993	3 204	789	1
Ibiapina	6 740	6 735	5 837	898	5
Ibicuitinga	3 662	3 661	2 997	664	1
Icapuí	6 422	6 398	4 901	1 497	24
Icó	20 308	20 297	16 774	3 523	11
Iguatu	30 318	30 292	26 357	3 935	26
Independência	9 005	8 999	7 029	1 970	6
Ipaporanga	4 172	4 170	3 249	921	2
Ipaumirim	3 984	3 981	3 244	737	3
Ipu	12 829	12 813	10 475	2 338	16
Ipueiras	12 855	12 845	10 198	2 647	10
Iracema	4 642	4 636	3 843	793	6
Irauçuba	6 432	6 431	5 184	1 247	1
Itaiçaba	2 525	2 525	2 196	329	-
Itaitinga	9 553	9 546	7 955	1 591	7
Itapagé	13 460	13 449	11 412	2 037	11
Itapipoca	31 375	31 346	26 251	5 095	29
Itapiúna	5 419	5 415	4 485	930	4
Itarema	8 975	8 970	7 580	1 390	5
Itatira	5 453	5 448	4 216	1 232	5
Jaguaretama	5 884	5 878	4 854	1 024	6
Jaguaribara	2 966	2 962	2 527	435	4
Jaguaribe	11 764	11 753	9 724	2 029	11
Jaguaruana	10 539	10 533	8 713	1 820	6
Jardim	7 474	7 472	6 198	1 274	2
Jati	2 377	2 377	1 950	427	-
Jijoca de Jericoacoara	5 126	5 016	3 992	1 024	110
Jucás	7 731	7 728	6 172	1 556	3

Tabela 2.1.10 - Domicílios recenseados, por espécie, segundo os municípios - Ceará - 2007

(continuação)

Municípios	Domicílios recenseados, por espécie				
	Total	Particulares			Coletivos
		Total	Ocupados (1)	Não-ocupados	
Lavras da Mangabeira	10 352	10 347	8 039	2 308	5
Limoeiro do Norte	17 648	17 636	15 324	2 312	12
Madalena	5 263	5 258	4 343	915	5
Maranguape	25 935	25 918	25 108	810	17
Marco	6 390	6 376	5 383	993	14
Martinópole	3 091	3 088	2 562	526	3
Massapê	9 562	9 557	8 226	1 331	5
Mauriti	12 773	12 767	10 416	2 351	6
Meruoca	4 064	4 053	3 023	1 030	11
Milagres	8 977	8 970	7 111	1 859	7
Milhã	4 475	4 474	3 794	680	1
Miraíma	3 724	3 722	2 914	808	2
Missão Velha	9 797	9 794	8 493	1 301	3
Mombaça	14 755	14 741	11 579	3 162	14
Monsenhor Tabosa	5 522	5 518	4 478	1 040	4
Morada Nova	20 441	20 432	17 057	3 375	9
Moraújo	2 677	2 676	1 996	680	1
Morrinhos	6 496	6 492	5 171	1 321	4
Mucambo	4 780	4 776	3 973	803	4
Mulungu	3 691	3 682	2 704	978	9
Nova Olinda	4 325	4 319	3 472	847	6
Nova Russas	10 457	10 448	8 589	1 859	9
Novo Oriente	9 274	9 271	7 477	1 794	3
Ocara	7 984	7 981	6 151	1 830	3
Orós	7 458	7 455	6 199	1 256	3
Pacajus	17 178	17 170	14 598	2 572	8
Pacatuba	16 637	16 628	16 213	415	9
Pacoti	3 505	3 493	2 721	772	12
Pacujá	1 926	1 923	1 657	266	3
Palhano	2 903	2 902	2 365	537	1
Palmácia	3 228	3 222	2 585	637	6
Paracuru	10 726	10 705	8 019	2 686	21
Paraipaba	8 704	8 678	7 235	1 443	26
Parambu	11 027	11 023	8 168	2 855	4
Paramoti	3 624	3 622	2 846	776	2
Pedra Branca	14 416	14 406	10 880	3 526	10
Penaforte	2 694	2 693	2 082	611	1
Pentecoste	10 870	10 863	8 780	2 083	7
Pereiro	5 077	5 075	3 950	1 125	2
Pindoretama	5 330	5 324	4 608	716	6
Piquet Carneiro	5 353	5 351	4 249	1 102	2
Pires Ferreira	3 131	3 131	2 494	637	-

Tabela 2.1.10 - Domicílios recenseados, por espécie, segundo os municípios - Ceará - 2007

(conclusão)

Municípios	Domicílios recenseados, por espécie				
	Total	Particulares			Coletivos
		Total	Ocupados (1)	Não-ocupados	
Poranga	4 266	4 262	3 174	1 088	4
Porteiras	4 787	4 784	3 851	933	3
Potengi	3 330	3 328	2 646	682	2
Potiretama	2 219	2 219	1 689	530	-
Quiterianópolis	6 803	6 799	5 462	1 337	4
Quixadá	23 205	23 180	20 031	3 149	25
Quixelô	5 329	5 325	4 507	818	4
Quixeramobim	22 343	22 331	18 656	3 675	12
Quixeré	6 670	6 667	5 340	1 327	3
Redenção	8 110	8 107	6 842	1 265	3
Reriutaba	6 696	6 691	5 309	1 382	5
Russas	20 474	20 463	17 671	2 792	11
Saboeiro	5 793	5 791	4 373	1 418	2
Salitre	4 877	4 875	3 931	944	2
Santa Quitéria	13 673	13 669	11 260	2 409	4
Santana do Acaraú	8 152	8 149	6 864	1 285	3
Santana do Cariri	5 047	5 042	4 320	722	5
São Benedito	12 826	12 814	11 145	1 669	12
São Gonçalo do Amarante	15 063	15 030	10 655	4 375	33
São João do Jaguaribe	3 212	3 211	2 544	667	1
São Luís do Curu	4 318	4 317	3 352	965	1
Senador Pompeu	9 366	9 360	7 249	2 111	6
Senador Sá	2 059	2 057	1 606	451	2
Solonópole	5 908	5 903	4 937	966	5
Tabuleiro do Norte	10 439	10 430	8 445	1 985	9
Tamboril	7 969	7 964	6 463	1 501	5
Tarrafas	3 493	3 492	2 368	1 124	1
Tauá	19 066	19 058	14 851	4 207	8
Tejuçuoca	4 690	4 686	3 733	953	4
Tianguá	18 892	18 876	16 218	2 658	16
Trairi	14 622	14 574	11 986	2 588	48
Tururu	4 422	4 420	3 401	1 019	2
Ubajara	8 899	8 890	7 804	1 086	9
Umari	2 559	2 556	2 028	528	3
Umirim	5 361	5 359	4 389	970	2
Uruburetama	5 607	5 604	4 768	836	3
Uruoca	4 143	4 140	3 296	844	3
Varjota	5 308	5 302	4 453	849	6
Várzea Alegre	13 006	12 997	10 692	2 305	9
Viçosa do Ceará	14 629	14 610	12 327	2 283	19

Fonte: IBGE, Contagem da População 2007.
(1) Inclusive os domicílios fechados com população estimada.

Tabela 2.1.11 - Domicílios recenseados, por espécie, segundo os municípios - Rio Grande do Norte - 2007

(continua)

Municípios	Domicílios recenseados, por espécie				
	Total	Particulares			Coletivos
		Total	Ocupados (1)	Não-ocupados	
**Rio Grande do Norte**	**973 184**	**971 783**	**817 608**	**154 175**	**1 401**
Acari	3 611	3 602	3 042	560	9
Açu	15 412	15 382	13 479	1 903	30
Afonso Bezerra	3 284	3 281	2 761	520	3
Água Nova	873	872	737	135	1
Alexandria	4 470	4 464	3 823	641	6
Almino Afonso	1 674	1 673	1 361	312	1
Alto do Rodrigues	3 881	3 853	3 161	692	28
Angicos	3 712	3 709	3 024	685	3
Antônio Martins	2 482	2 482	1 989	493	-
Apodi	11 561	11 545	9 555	1 990	16
Areia Branca	8 002	7 982	6 720	1 262	20
Arês	3 527	3 526	3 083	443	1
Augusto Severo	3 060	3 057	2 420	637	3
Baía Formosa	2 622	2 601	2 095	506	21
Baraúna	7 692	7 689	6 186	1 503	3
Barcelona	1 276	1 273	1 048	225	3
Bento Fernandes	1 514	1 513	1 262	251	1
Bodó	900	899	686	213	1
Bom Jesus	2 751	2 750	2 268	482	1
Brejinho	3 450	3 445	2 973	472	5
Caiçara do Norte	1 874	1 870	1 625	245	4
Caiçara do Rio do Vento	1 020	1 020	835	185	-
Caicó	19 348	19 296	17 066	2 230	52
Campo Redondo	3 353	3 352	2 749	603	1
Canguaretama	8 321	8 237	6 912	1 325	84
Caraúbas	7 036	7 024	5 565	1 459	12
Carnaúba dos Dantas	2 544	2 544	2 021	523	-
Carnaubais	3 029	3 023	2 526	497	6
Ceará-Mirim	19 745	19 727	16 088	3 639	18
Cerro Corá	3 527	3 527	2 917	610	-
Coronel Ezequiel	2 017	2 015	1 468	547	2
Coronel João Pessoa	1 558	1 558	1 238	320	-
Cruzeta	2 707	2 702	2 292	410	5
Currais Novos	13 050	13 035	11 488	1 547	15
Doutor Severiano	1 872	1 872	1 620	252	-
Encanto	1 579	1 579	1 391	188	-
Equador	2 090	2 086	1 690	396	4
Espírito Santo	3 134	3 134	2 653	481	-
Extremoz	9 916	9 902	5 612	4 290	14
Felipe Guerra	1 877	1 875	1 607	268	2
Fernando Pedroza	979	977	799	178	2
Florânia	2 863	2 862	2 329	533	1
Francisco Dantas	1 075	1 075	826	249	-
Frutuoso Gomes	1 489	1 489	1 300	189	-
Galinhos	754	743	570	173	11
Goianinha	5 273	5 266	4 752	514	7
Governador Dix-Sept Rosado	4 307	4 302	3 391	911	5
Grossos	3 098	3 095	2 666	429	3
Guamaré	3 946	3 934	3 189	745	12
Ielmo Marinho	3 665	3 665	3 053	612	-
Ipanguaçu	4 205	4 203	3 519	684	2
Ipueira	669	669	590	79	-
Itajá	2 036	2 033	1 669	364	3

Tabela 2.1.11 - Domicílios recenseados, por espécie, segundo os municípios - Rio Grande do Norte - 2007

(continuação)

Municípios	Domicílios recenseados, por espécie				
	Total	Particulares			Coletivos
		Total	Ocupados (1)	Não-ocupados	
Itaú	1 918	1 913	1 623	290	5
Jaçanã	2 676	2 676	2 164	512	-
Jandaíra	2 052	2 052	1 675	377	-
Janduís	1 809	1 806	1 458	348	3
Januário Cicco	2 709	2 707	2 191	516	2
Japi	1 881	1 881	1 474	407	-
Jardim de Angicos	900	900	673	227	-
Jardim de Piranhas	4 137	4 133	3 636	497	4
Jardim do Seridó	4 380	4 377	3 597	780	3
João Câmara	9 017	9 015	7 721	1 294	2
João Dias	983	982	708	274	1
José da Penha	2 068	2 066	1 754	312	2
Jucurutu	5 896	5 891	4 996	895	5
Jundiá	1 069	1 068	924	144	1
Lagoa d'Anta	1 793	1 793	1 511	282	-
Lagoa de Pedras	2 236	2 236	1 842	394	-
Lagoa de Velhos	870	870	701	169	-
Lagoa Nova	4 125	4 119	3 314	805	6
Lagoa Salgada	2 343	2 342	1 872	470	1
Lajes	3 341	3 333	2 777	556	8
Lajes Pintadas	1 404	1 404	1 130	274	-
Lucrécia	1 109	1 109	938	171	-
Luís Gomes	2 990	2 989	2 480	509	1
Macaíba	20 145	20 130	17 105	3 025	15
Macau	9 125	9 097	7 745	1 352	28
Major Sales	970	970	874	96	-
Marcelino Vieira	2 804	2 804	2 208	596	-
Martins	2 726	2 717	2 267	450	9
Maxaranguape	3 631	3 614	2 119	1 495	17
Messias Targino	1 165	1 163	994	169	2
Montanhas	3 409	3 407	3 028	379	2
Monte Alegre	6 291	6 287	5 277	1 010	4
Monte das Gameleiras	919	919	645	274	-
Mossoró	74 761	74 648	63 655	10 993	113
Natal	244 481	244 106	216 258	27 848	375
Nísia Floresta	12 518	12 507	6 038	6 469	11
Nova Cruz	10 523	10 518	9 188	1 330	5
Olho-d'Água do Borges	1 461	1 459	1 250	209	2
Ouro Branco	1 625	1 623	1 370	253	2
Paraná	1 126	1 126	1 003	123	-
Paraú	1 401	1 400	1 082	318	1
Parazinho	1 343	1 342	1 140	202	1
Parelhas	6 646	6 640	5 648	992	6
Parnamirim	58 979	58 939	50 305	8 634	40
Passa e Fica	3 040	3 038	2 742	296	2
Passagem	835	835	732	103	-
Patu	4 328	4 324	3 354	970	4
Pau dos Ferros	8 400	8 388	7 331	1 057	12
Pedra Grande	1 314	1 312	984	328	2
Pedra Preta	769	769	667	102	-
Pedro Avelino	2 731	2 731	2 023	708	-
Pedro Velho	3 986	3 982	3 448	534	4
Pendências	4 351	4 344	3 471	873	7
Pilões	1 057	1 056	905	151	1
Poço Branco	3 263	3 262	2 849	413	1
Portalegre	2 362	2 359	1 944	415	3
Porto do Mangue	1 634	1 631	1 325	306	3
Presidente Juscelino	2 438	2 435	2 083	352	3

Tabela 2.1.11 - Domicílios recenseados, por espécie, segundo os municípios - Rio Grande do Norte - 2007

(conclusão)

Municípios	Domicílios recenseados, por espécie				
	Total	Particulares			Coletivos
		Total	Ocupados (1)	Não-ocupados	
Pureza	2 445	2 444	2 051	393	1
Rafael Fernandes	1 546	1 545	1 283	262	1
Rafael Godeiro	1 126	1 123	930	193	3
Riacho da Cruz	1 093	1 093	867	226	-
Riacho de Santana	1 462	1 462	1 198	264	-
Riachuelo	1 975	1 974	1 711	263	1
Rio do Fogo	3 540	3 528	2 363	1 165	12
Rodolfo Fernandes	1 364	1 364	1 233	131	-
Ruy Barbosa	1 147	1 147	929	218	-
Santa Cruz	10 342	10 332	8 921	1 411	10
Santa Maria	1 520	1 520	1 256	264	-
Santana do Matos	4 982	4 978	3 897	1 081	4
Santana do Seridó	844	844	740	104	-
Santo Antônio	6 900	6 893	5 821	1 072	7
São Bento do Norte	1 046	1 044	933	111	2
São Bento do Trairí	964	964	926	38	-
São Fernando	1 103	1 103	942	161	-
São Francisco do Oeste	1 256	1 256	1 081	175	-
São Gonçalo do Amarante	24 502	24 482	20 364	4 118	20
São João do Sabugi	1 906	1 906	1 649	257	-
São José de Mipibu	11 153	11 143	9 470	1 673	10
São José do Campestre	3 823	3 821	3 124	697	2
São José do Seridó	1 295	1 294	1 078	216	1
São Miguel	7 305	7 303	6 034	1 269	2
São Miguel do Gostoso	2 853	2 853	2 155	698	-
São Paulo do Potengi	4 621	4 614	3 962	652	7
São Pedro	1 970	1 970	1 682	288	-
São Rafael	2 859	2 856	2 316	540	3
São Tomé	3 965	3 961	3 067	894	4
São Vicente	2 050	2 050	1 718	332	-
Senador Elói de Souza	1 744	1 743	1 580	163	1
Senador Georgino Avelino	1 010	1 010	920	90	-
Serra de São Bento	1 921	1 921	1 481	440	-
Serra do Mel	2 976	2 969	2 438	531	7
Serra Negra do Norte	2 474	2 466	1 929	537	8
Serrinha	2 043	2 042	1 734	308	1
Serrinha dos Pintos	1 484	1 483	1 278	205	1
Severiano Melo	1 850	1 848	1 537	311	2
Sítio Novo	1 477	1 477	1 402	75	-
Taboleiro Grande	746	746	639	107	-
Taipu	3 373	3 371	2 866	505	2
Tangará	3 994	3 992	3 531	461	2
Tenente Ananias	2 783	2 780	2 397	383	3
Tenente Laurentino Cruz	1 662	1 660	1 400	260	2
Tibau	3 120	3 116	1 014	2 102	4
Tibau do Sul	4 005	3 896	2 915	981	109
Timbaúba dos Batistas	727	725	605	120	2
Touros	9 009	8 998	7 084	1 914	11
Triunfo Potiguar	1 044	1 037	856	181	7
Umarizal	3 666	3 662	2 970	692	4
Upanema	4 189	4 186	3 429	757	3
Várzea	1 664	1 664	1 491	173	-
Venha-Ver	1 134	1 133	853	280	1
Vera Cruz	2 975	2 974	2 571	403	1
Viçosa	527	527	469	58	-
Vila Flor	657	657	613	44	-

Fonte: IBGE, Contagem da População 2007.

(1) Inclusive os domicílios fechados com população estimada.

Tabela 2.1.12 - Domicílios recenseados, por espécie, segundo os municípios - Paraíba - 2007

(continua)

Municípios	Domicílios recenseados, por espécie				
	Total	Particulares			Coletivos
		Total	Ocupados (1)	Não-ocupados	
**Paraíba**	**1 185 908**	**1 184 929**	**996 575**	**188 354**	**979**
Água Branca	3 037	3 035	2 397	638	2
Aguiar	1 925	1 925	1 596	329	-
Alagoa Grande	9 044	9 037	7 458	1 579	7
Alagoa Nova	5 819	5 818	5 001	817	1
Alagoinha	4 078	4 073	3 407	666	5
Alcantil	1 797	1 796	1 417	379	1
Algodão de Jandaíra	936	935	621	314	1
Alhandra	5 535	5 531	4 822	709	4
Amparo	812	812	580	232	-
Aparecida	2 338	2 338	1 985	353	-
Araçagi	5 752	5 752	4 757	995	-
Arara	4 605	4 604	3 616	988	1
Araruna	6 233	6 228	5 006	1 222	5
Areia	7 582	7 551	6 316	1 235	31
Areia de Baraúnas	626	626	511	115	-
Areial	2 002	2 002	1 734	268	-
Aroeiras	6 792	6 789	5 155	1 634	3
Assunção	1 157	1 155	900	255	2
Baía da Traição	3 816	3 793	1 888	1 905	23
Bananeiras	6 819	6 801	5 559	1 242	18
Baraúna	1 517	1 517	1 161	356	-
Barra de Santa Rosa	5 409	5 407	3 682	1 725	2
Barra de Santana	3 253	3 253	2 393	860	-
Barra de São Miguel	2 064	2 064	1 559	505	-
Bayeux	26 623	26 617	25 005	1 612	6
Belém	5 538	5 532	4 887	645	6
Belém do Brejo do Cruz	2 270	2 269	1 819	450	1
Bernardino Batista	908	908	764	144	-
Boa Ventura	1 841	1 840	1 454	386	1
Boa Vista	1 809	1 809	1 511	298	-
Bom Jesus	808	808	611	197	-
Bom Sucesso	1 904	1 904	1 520	384	-
Bonito de Santa Fé	3 135	3 132	2 605	527	3
Boqueirão	5 238	5 232	4 376	856	6
Borborema	1 564	1 564	1 322	242	-
Brejo do Cruz	3 893	3 890	3 310	580	3
Brejo dos Santos	1 993	1 993	1 631	362	-
Caaporã	5 518	5 496	4 909	587	22
Cabaceiras	1 811	1 807	1 403	404	4
Cabedelo	19 400	19 394	14 090	5 304	6
Cachoeira dos Índios	2 802	2 802	2 191	611	-
Cacimba de Areia	1 196	1 196	958	238	-
Cacimba de Dentro	5 878	5 874	4 560	1 314	4
Cacimbas	2 496	2 496	1 712	784	-
Caiçara	2 399	2 397	2 003	394	2
Cajazeiras	18 630	18 604	15 991	2 613	26
Cajazeirinhas	1 028	1 028	760	268	-
Caldas Brandão	1 792	1 792	1 539	253	-
Camalaú	2 384	2 384	1 779	605	-
Campina Grande	114 762	114 668	103 458	11 210	94
Campo de Santana	3 142	3 142	2 541	601	-
Capim	1 513	1 513	1 323	190	-
Caraúbas	1 574	1 574	1 131	443	-

Tabela 2.1.12 - Domicílios recenseados, por espécie, segundo os municípios - Paraíba - 2007

(continuação)

Municípios	Domicílios recenseados, por espécie				
	Total	Particulares			Coletivos
		Total	Ocupados (1)	Não-ocupados	
Carrapateira	702	702	622	80	-
Casserengue	2 588	2 588	1 783	805	-
Catingueira	1 544	1 544	1 194	350	-
Catolé do Rocha	8 776	8 769	7 555	1 214	7
Caturité	1 491	1 491	1 234	257	-
Conceição	5 737	5 730	4 647	1 083	7
Condado	2 378	2 378	1 830	548	-
Conde	8 758	8 727	5 208	3 519	31
Congo	2 022	2 019	1 428	591	3
Coremas	5 016	5 006	4 184	822	10
Coxixola	694	694	539	155	-
Cruz do Espírito Santo	3 998	3 996	3 750	246	2
Cubati	2 269	2 268	1 807	461	1
Cuité	7 410	7 405	5 692	1 713	5
Cuité de Mamanguape	2 245	2 245	1 745	500	-
Cuitegi	2 140	2 140	1 841	299	-
Curral de Cima	1 848	1 848	1 498	350	-
Curral Velho	802	802	625	177	-
Damião	1 781	1 781	1 275	506	-
Desterro	2 728	2 725	2 176	549	3
Diamante	2 162	2 161	1 661	500	1
Dona Inês	3 652	3 649	2 843	806	3
Duas Estradas	1 262	1 262	1 057	205	-
Emas	921	921	795	126	-
Esperança	9 836	9 824	8 444	1 380	12
Fagundes	4 036	4 034	3 162	872	2
Frei Martinho	1 124	1 124	892	232	-
Gado Bravo	2 717	2 717	2 022	695	-
Guarabira	16 754	16 741	15 020	1 721	13
Gurinhém	4 125	4 122	3 448	674	3
Gurjão	1 085	1 084	843	241	1
Ibiara	2 350	2 350	1 701	649	-
Igaracy	2 129	2 127	1 684	443	2
Imaculada	3 500	3 500	2 815	685	-
Ingá	6 279	6 277	4 965	1 312	2
Itabaiana	8 103	8 098	7 135	963	5
Itaporanga	7 217	7 208	5 992	1 216	9
Itapororoca	5 046	5 043	4 256	787	3
Itatuba	3 278	3 278	2 581	697	-
Jacaraú	4 518	4 516	3 815	701	2
Jericó	2 517	2 517	2 040	477	-
João Pessoa	213 488	213 261	189 517	23 744	227
Juarez Távora	2 621	2 621	2 146	475	-
Juazeirinho	5 155	5 152	4 014	1 138	3
Junco do Seridó	2 061	2 061	1 757	304	-
Juripiranga	3 101	3 100	2 767	333	1
Juru	3 520	3 520	2 617	903	-
Lagoa	1 667	1 667	1 304	363	-
Lagoa de Dentro	2 509	2 508	2 065	443	1
Lagoa Seca	7 629	7 622	6 582	1 040	7
Lastro	802	802	692	110	-
Livramento	2 567	2 566	1 931	635	1
Logradouro	1 192	1 192	1 006	186	-
Lucena	5 617	5 602	2 830	2 772	15

Tabela 2.1.12 - Domicílios recenseados, por espécie, segundo os municípios - Paraíba - 2007

(continuação)

Municípios	Domicílios recenseados, por espécie				
	Total	Particulares			Coletivos
		Total	Ocupados (1)	Não-ocupados	
Mãe d'Água	1 556	1 556	1 096	460	-
Malta	1 841	1 839	1 581	258	2
Mamanguape	12 098	12 087	10 574	1 513	11
Manaíra	3 806	3 803	2 791	1 012	3
Marcação	2 226	2 226	1 871	355	-
Mari	7 014	7 013	5 862	1 151	1
Marizópolis	1 895	1 895	1 683	212	-
Massaranduba	4 363	4 360	3 507	853	3
Mataraca	2 014	2 008	1 728	280	6
Matinhas	1 246	1 246	1 054	192	-
Mato Grosso	872	872	672	200	-
Maturéia	1 874	1 872	1 516	356	2
Mogeiro	4 384	4 384	3 361	1 023	-
Montadas	1 483	1 482	1 269	213	1
Monte Horebe	1 484	1 484	1 209	275	-
Monteiro	11 192	11 181	9 077	2 104	11
Mulungu	3 353	3 353	2 644	709	-
Natuba	3 375	3 375	2 589	786	-
Nazarezinho	2 326	2 326	1 921	405	-
Nova Floresta	3 399	3 399	2 871	528	-
Nova Olinda	2 046	2 045	1 633	412	1
Nova Palmeira	1 409	1 408	1 149	259	1
Olho d'Água	2 581	2 581	1 839	742	-
Olivedos	1 284	1 284	990	294	-
Ouro Velho	1 185	1 184	929	255	1
Parari	502	502	379	123	-
Passagem	857	857	644	213	-
Patos	29 634	29 602	26 777	2 825	32
Paulista	3 595	3 595	3 007	588	-
Pedra Branca	1 111	1 111	925	186	-
Pedra Lavrada	2 355	2 353	1 935	418	2
Pedras de Fogo	7 384	7 355	6 748	607	29
Pedro Régis	2 112	2 111	1 579	532	1
Piancó	5 213	5 210	4 414	796	3
Picuí	7 250	7 241	5 265	1 976	9
Pilar	3 505	3 504	3 116	388	1
Pilões	1 918	1 915	1 627	288	3
Pilõezinhos	1 679	1 679	1 369	310	-
Pirpirituba	3 186	3 186	2 808	378	-
Pitimbu	7 654	7 643	4 243	3 400	11
Pocinhos	5 161	5 161	4 352	809	-
Poço Dantas	1 198	1 198	998	200	-
Poço de José de Moura	1 375	1 374	1 110	264	1
Pombal	10 747	10 738	8 812	1 926	9
Prata	1 414	1 414	1 182	232	-
Princesa Isabel	6 196	6 186	5 178	1 008	10
Puxinanã	4 364	4 363	3 549	814	1
Queimadas	13 053	13 049	10 803	2 246	4
Quixabá	451	451	361	90	-
Remígio	5 811	5 809	4 654	1 155	2
Riachão	1 062	1 062	873	189	-
Riachão do Bacamarte	1 434	1 434	1 178	256	-
Riachão do Poço	1 376	1 376	1 109	267	-
Riacho de Santo Antônio	484	484	402	82	-
Riacho dos Cavalos	2 687	2 686	2 071	615	1
Rio Tinto	7 085	7 079	6 298	781	6
Salgadinho	1 123	1 122	854	268	1
Salgado de São Félix	4 363	4 362	3 540	822	1
Santa Cecília	2 333	2 333	1 755	578	-

Tabela 2.1.12 - Domicílios recenseados, por espécie, segundo os municípios - Paraíba - 2007

(conclusão)

Municípios	Domicílios recenseados, por espécie				
	Total	Particulares			Coletivos
		Total	Ocupados (1)	Não-ocupados	
Santa Cruz	2 133	2 133	1 789	344	-
Santa Helena	2 314	2 313	1 730	583	1
Santa Inês	1 197	1 197	933	264	-
Santa Luzia	4 754	4 749	4 063	686	5
Santa Rita	36 002	35 990	32 718	3 272	12
Santa Teresinha	1 566	1 566	1 191	375	-
Santana de Mangueira	2 054	2 054	1 401	653	-
Santana dos Garrotes	2 518	2 516	1 970	546	2
Santarém	882	882	717	165	-
Santo André	944	944	751	193	-
São Bentinho	1 349	1 348	1 119	229	1
São Bento	8 277	8 270	7 526	744	7
São Domingos de Pombal	902	902	745	157	-
São Domingos do Cariri	937	937	659	278	-
São Francisco	1 132	1 132	981	151	-
São João do Cariri	1 793	1 789	1 288	501	4
São João do Rio do Peixe	5 911	5 908	4 986	922	3
São João do Tigre	1 954	1 954	1 415	539	-
São José da Lagoa Tapada	2 724	2 724	2 182	542	-
São José de Caiana	1 898	1 898	1 381	517	-
São José de Espinharas	1 641	1 640	1 266	374	1
São José de Piranhas	6 193	6 190	5 092	1 098	3
São José de Princesa	1 642	1 642	1 140	502	-
São José do Bonfim	1 102	1 102	845	257	-
São José do Brejo do Cruz	478	478	401	77	-
São José do Sabugi	1 348	1 348	1 085	263	-
São José dos Cordeiros	1 665	1 665	1 196	469	-
São José dos Ramos	1 549	1 549	1 412	137	-
São Mamede	2 847	2 847	2 194	653	-
São Miguel de Taipu	1 865	1 865	1 687	178	-
São Sebastião de Lagoa de Roça	3 411	3 409	2 870	539	2
São Sebastião do Umbuzeiro	1 338	1 338	967	371	-
Sapé	14 386	14 378	12 347	2 031	8
Seridó	2 958	2 958	2 387	571	-
Serra Branca	4 817	4 814	3 859	955	3
Serra da Raiz	1 064	1 060	850	210	4
Serra Grande	1 059	1 059	821	238	-
Serra Redonda	2 789	2 787	2 242	545	2
Serraria	2 215	2 212	1 672	540	3
Sertãozinho	1 284	1 284	1 177	107	-
Sobrado	2 351	2 351	1 921	430	-
Solânea	9 134	9 114	7 470	1 644	20
Soledade	4 328	4 323	3 587	736	5
Sossêgo	1 099	1 098	822	276	1
Sousa	19 517	19 494	17 426	2 068	23
Sumé	6 312	6 304	5 054	1 250	8
Taperoá	5 213	5 205	3 966	1 239	8
Tavares	4 307	4 306	3 537	769	1
Teixeira	4 523	4 520	3 666	854	3
Tenório	852	852	697	155	-
Triunfo	2 893	2 893	2 424	469	-
Uiraúna	4 831	4 827	4 045	782	4
Umbuzeiro	2 985	2 985	2 396	589	-
Várzea	954	952	728	224	2
Vieirópolis	1 669	1 668	1 279	389	1
Vista Serrana	948	948	822	126	-
Zabelê	858	858	633	225	-

Fonte: IBGE, Contagem da População 2007.

(1) Inclusive os domicílios fechados com população estimada.

Tabela 2.1.13 - Domicílios recenseados, por espécie, segundo os municípios - Pernambuco - 2007

(continua)

Municípios	Domicílios recenseados, por espécie				
	Total	Particulares			Coletivos
		Total	Ocupados (1)	Não-ocupados	
**Pernambuco**	**1 635 375**	**1 633 933**	**1 358 116**	**275 817**	**1 442**
Abreu e Lima	28 085	28 079	26 007	2 072	6
Afogados da Ingazeira	12 655	12 643	10 072	2 571	12
Afrânio	5 684	5 682	4 162	1 520	2
Agrestina	7 500	7 497	6 205	1 292	3
Água Preta	8 404	8 402	7 295	1 107	2
Águas Belas	12 816	12 808	10 013	2 795	8
Alagoinha	4 610	4 610	3 781	829	-
Aliança	10 902	10 898	8 939	1 959	4
Altinho	8 478	8 475	6 657	1 818	3
Amaraji	6 008	6 004	5 411	593	4
Angelim	3 164	3 162	2 713	449	2
Araçoiaba	4 581	4 581	4 291	290	-
Araripina	22 917	22 887	19 410	3 477	30
Arcoverde	21 541	21 518	18 249	3 269	23
Barra de Guabiraba	3 463	3 463	3 099	364	-
Barreiros	12 441	12 425	10 553	1 872	16
Belém de Maria	3 255	3 250	2 727	523	5
Belém de São Francisco	5 903	5 891	4 901	990	12
Belo Jardim	23 972	23 961	20 966	2 995	11
Betânia	3 829	3 827	3 089	738	2
Bezerros	20 939	20 925	17 909	3 016	14
Bodocó	10 306	10 306	8 056	2 250	-
Bom Conselho	13 817	13 806	11 817	1 989	11
Bom Jardim	12 944	12 938	10 592	2 346	6
Bonito	12 239	12 232	10 850	1 382	7
Brejão	2 867	2 865	2 503	362	2
Brejinho	2 635	2 635	1 994	641	-
Brejo da Madre de Deus	14 979	14 971	11 379	3 592	8
Buenos Aires	4 205	4 204	3 393	811	1
Buíque	14 632	14 629	11 903	2 726	3
Cabo de Santo Agostinho	53 974	53 927	44 581	9 346	47
Cabrobó	8 763	8 756	7 403	1 353	7
Cachoeirinha	6 380	6 379	5 276	1 103	1
Caetés	7 342	7 342	6 290	1 052	-
Calçado	3 725	3 722	3 079	643	3
Calumbi	2 525	2 523	2 088	435	2
Camaragibe	41 982	41 963	38 265	3 698	19
Camocim de São Félix	5 248	5 244	4 606	638	4
Camutanga	2 344	2 343	2 085	258	1
Canhotinho	7 574	7 568	6 444	1 124	6
Capoeiras	5 754	5 751	4 900	851	3
Carnaíba	6 866	6 863	5 050	1 813	3
Carnaubeira da Penha	3 173	3 172	2 477	695	1
Carpina	20 457	20 439	17 823	2 616	18
Casinhas	4 468	4 468	3 592	876	-
Catende	10 086	10 080	8 856	1 224	6
Cedro	3 114	3 113	2 430	683	1
Chã de Alegria	3 361	3 361	2 995	366	-
Chã Grande	5 881	5 867	5 010	857	14
Condado	6 925	6 923	6 056	867	2
Correntes	5 272	5 271	4 378	893	1
Cortês	3 384	3 382	3 043	339	2
Cumaru	6 124	6 122	4 697	1 425	2
Cupira	7 975	7 971	6 606	1 365	4
Custódia	11 363	11 355	9 144	2 211	8
Dormentes	5 430	5 428	4 031	1 397	2
Escada	18 491	18 473	16 119	2 354	18

Tabela 2.1.13 - Domicílios recenseados, por espécie, segundo os municípios - Pernambuco - 2007

(continuação)

Municípios	Domicílios recenseados, por espécie				
	Total	Particulares			Coletivos
		Total	Ocupados (1)	Não-ocupados	
Exu	9 944	9 941	7 520	2 421	3
Feira Nova	6 305	6 303	5 401	902	2
Fernando de Noronha	809	679	610	69	130
Ferreiros	3 270	3 268	2 864	404	2
Flores	8 227	8 221	6 362	1 859	6
Floresta	8 166	8 158	6 337	1 821	8
Frei Miguelinho	5 788	5 786	4 430	1 356	2
Gameleira	6 740	6 740	6 243	497	-
Garanhuns	39 280	39 221	34 565	4 656	59
Glória do Goitá	8 960	8 954	7 384	1 570	6
Goiana	27 147	27 104	19 084	8 020	43
Granito	2 220	2 218	1 598	620	2
Gravatá	29 478	29 451	20 971	8 480	27
Iati	5 448	5 447	4 385	1 062	1
Ibimirim	8 367	8 353	6 797	1 556	14
Ibirajuba	2 771	2 770	2 152	618	1
Igarassu	28 228	28 221	25 933	2 288	7
Iguaraci	4 561	4 560	3 444	1 116	1
Ilha de Itamaracá	14 388	14 375	4 260	10 115	13
Inajá	4 223	4 219	3 342	877	4
Ingazeira	1 609	1 609	1 229	380	-
Ipojuca	22 786	22 651	17 858	4 793	135
Ipubi	7 580	7 576	6 341	1 235	4
Itacuruba	1 106	1 105	1 017	88	1
Itaíba	8 611	8 600	6 865	1 735	11
Itambé	10 416	10 401	8 875	1 526	15
Itapetim	5 834	5 827	4 286	1 541	7
Itapissuma	6 371	6 370	5 996	374	1
Itaquitinga	4 386	4 386	3 808	578	-
Jaqueira	3 159	3 159	2 762	397	-
Jataúba	5 638	5 635	4 121	1 514	3
Jatobá	4 356	4 354	3 599	755	2
João Alfredo	10 213	10 211	8 092	2 119	2
Joaquim Nabuco	4 569	4 569	4 034	535	-
Jucati	3 252	3 252	2 834	418	-
Jupi	4 353	4 350	3 730	620	3
Jurema	4 630	4 630	4 073	557	-
Lagoa do Carro	4 482	4 481	3 878	603	1
Lagoa do Itaenga	6 016	6 014	5 252	762	2
Lagoa do Ouro	3 687	3 686	3 138	548	1
Lagoa dos Gatos	6 007	6 003	4 559	1 444	4
Lagoa Grande	6 388	6 383	5 344	1 039	5
Lajedo	11 402	11 392	9 590	1 802	10
Limoeiro	18 189	18 168	15 714	2 454	21
Macaparana	7 032	7 028	6 042	986	4
Machados	3 408	3 407	2 943	464	1
Manari	4 904	4 904	3 875	1 029	-
Maraial	3 571	3 569	3 092	477	2
Mirandiba	4 160	4 155	3 215	940	5
Moreilândia	3 501	3 498	2 690	808	3
Moreno	16 198	16 187	14 538	1 649	11
Nazaré da Mata	9 203	9 195	7 851	1 344	8
Orobó	7 156	7 154	5 715	1 439	2
Orocó	3 846	3 842	3 239	603	4
Ouricuri	19 523	19 506	15 507	3 999	17
Palmares	16 725	16 712	14 831	1 881	13
Palmeirina	2 716	2 714	2 313	401	2
Panelas	8 832	8 829	6 679	2 150	3
Paranatama	3 662	3 661	3 227	434	1
Parnamirim	5 969	5 968	4 727	1 241	1
Passira	9 638	9 633	7 895	1 738	5
Paudalho	14 304	14 298	12 053	2 245	6

Tabela 2.1.13 - Domicílios recenseados, por espécie, segundo os municípios - Pernambuco - 2007

(conclusão)

Municípios	Domicílios recenseados, por espécie				
	Total	Particulares			Coletivos
		Total	Ocupados (1)	Não-ocupados	
Pedra	6 509	6 501	5 326	1 175	8
Pesqueira	20 335	20 319	17 486	2 833	16
Petrolândia	8 698	8 692	7 770	922	6
Poção	3 962	3 959	3 263	696	3
Pombos	7 236	7 234	6 308	926	2
Primavera	3 570	3 566	3 120	446	4
Quipapá	6 876	6 875	6 029	846	1
Quixaba	2 322	2 322	1 751	571	-
Riacho das Almas	7 310	7 308	5 557	1 751	2
Ribeirão	11 349	11 336	10 284	1 052	13
Rio Formoso	5 773	5 764	5 043	721	9
Sairé	4 439	4 434	3 843	591	5
Salgadinho	2 359	2 358	2 059	299	1
Salgueiro	15 681	15 653	13 601	2 052	28
Saloá	5 224	5 221	4 188	1 033	3
Sanharó	6 134	6 133	5 127	1 006	1
Santa Cruz	4 500	4 498	3 355	1 143	2
Santa Cruz da Baixa Verde	4 219	4 217	3 089	1 128	2
Santa Cruz do Capibaribe	24 481	24 451	21 361	3 090	30
Santa Filomena	4 555	4 554	3 478	1 076	1
Santa Maria da Boa Vista	11 080	11 066	9 341	1 725	14
Santa Maria do Cambucá	4 206	4 204	3 454	750	2
Santa Terezinha	3 456	3 453	2 771	682	3
São Benedito do Sul	3 171	3 167	2 510	657	4
São Bento do Una	14 572	14 567	12 363	2 204	5
São Caitano	12 276	12 272	10 009	2 263	4
São João	6 322	6 322	5 500	822	-
São Joaquim do Monte	7 018	7 015	5 989	1 026	3
São José da Coroa Grande	6 965	6 951	4 341	2 610	14
São José do Belmonte	10 310	10 302	8 348	1 954	8
São José do Egito	11 623	11 614	9 159	2 455	9
São Lourenço da Mata	29 812	29 806	26 354	3 452	6
São Vicente Ferrer	4 829	4 829	4 243	586	-
Serra Talhada	26 069	26 049	21 269	4 780	20
Serrita	5 366	5 363	4 181	1 182	3
Sertânia	12 195	12 184	10 605	1 579	11
Sirinhaém	10 030	10 008	8 594	1 414	22
Solidão	2 061	2 059	1 456	603	2
Surubim	18 269	18 251	15 579	2 672	18
Tabira	9 148	9 143	7 428	1 715	5
Tacaimbó	4 383	4 383	3 532	851	-
Tacaratu	6 314	6 307	5 056	1 251	7
Tamandaré	9 511	9 470	4 495	4 975	41
Taquaritinga do Norte	7 688	7 682	6 384	1 298	6
Terezinha	1 982	1 982	1 644	338	-
Terra Nova	2 550	2 550	2 209	341	-
Timbaúba	15 768	15 750	13 745	2 005	18
Toritama	8 633	8 631	7 991	640	2
Tracunhaém	3 738	3 737	3 318	419	1
Trindade	7 194	7 187	6 287	900	7
Triunfo	5 390	5 374	4 148	1 226	16
Tupanatinga	6 148	6 146	4 664	1 482	2
Tuparetama	2 869	2 866	2 481	385	3
Venturosa	4 725	4 722	4 247	475	3
Verdejante	2 938	2 937	2 459	478	1
Vertente do Lério	2 649	2 649	2 110	539	-
Vertentes	5 844	5 843	4 870	973	1
Vicência	7 963	7 960	6 994	966	3
Vitória de Santo Antão	39 008	38 986	35 012	3 974	22
Xexéu	4 310	4 310	3 569	741	-

Fonte: IBGE, Contagem da População 2007.
(1) Inclusive os domicílios fechados com população estimada.

Tabela 2.1.14 - Domicílios recenseados, por espécie, segundo os municípios - Alagoas - 2007

(continua)

Municípios	Domicílios recenseados, por espécie				
	Total	Particulares			Coletivos
		Total	Ocupados (1)	Não-ocupados	
**Alagoas**	**901 727**	**900 966**	**785 233**	**115 733**	**761**
Água Branca	5 272	5 270	4 389	881	2
Anadia	5 141	5 141	4 502	639	-
Arapiraca	58 362	58 313	52 743	5 570	49
Atalaia	11 741	11 739	10 833	906	2
Barra de Santo Antônio	4 609	4 601	3 401	1 200	8
Barra de São Miguel	4 011	3 992	1 854	2 138	19
Batalha	4 697	4 694	3 994	700	3
Belém	1 687	1 687	1 345	342	-
Belo Monte	2 185	2 185	1 753	432	-
Boca da Mata	6 803	6 800	6 007	793	3
Branquinha	2 937	2 934	2 643	291	3
Cacimbinhas	3 089	3 087	2 678	409	2
Cajueiro	4 953	4 952	4 567	385	1
Campestre	1 764	1 763	1 480	283	1
Campo Alegre	10 007	9 994	9 359	635	13
Campo Grande	2 612	2 611	2 327	284	1
Canapi	5 727	5 725	4 352	1 373	2
Capela	4 418	4 416	3 970	446	2
Carneiros	2 207	2 207	1 905	302	-
Chã Preta	1 901	1 901	1 713	188	-
Coité do Nóia	3 111	3 111	2 755	356	-
Colônia Leopoldina	5 647	5 644	4 847	797	3
Coqueiro Seco	1 590	1 590	1 398	192	-
Coruripe	14 749	14 735	12 401	2 334	14
Craíbas	6 523	6 520	5 575	945	3
Delmiro Gouveia	14 166	14 159	12 282	1 877	7
Dois Riachos	3 173	3 173	2 796	377	-
Estrela de Alagoas	5 366	5 366	4 534	832	-
Feira Grande	6 109	6 106	5 358	748	3
Feliz Deserto	1 354	1 352	1 165	187	2
Flexeiras	3 377	3 370	2 990	380	7
Girau do Ponciano	9 347	9 345	8 249	1 096	2
Ibateguara	4 681	4 679	3 999	680	2
Igaci	7 807	7 806	6 689	1 117	1
Igreja Nova	6 459	6 457	5 704	753	2
Inhapi	4 960	4 960	4 190	770	-
Jacaré dos Homens	1 596	1 596	1 374	222	-
Jacuípe	1 950	1 950	1 700	250	-
Japaratinga	2 608	2 586	1 840	746	22
Jaramataia	1 564	1 563	1 359	204	1
Jequiá da Praia	3 073	3 072	2 649	423	1
Joaquim Gomes	5 972	5 965	4 982	983	7
Jundiá	1 628	1 628	1 145	483	-
Junqueiro	7 043	7 042	6 155	887	1
Lagoa da Canoa	5 041	5 040	4 490	550	1
Limoeiro de Anadia	6 503	6 503	6 168	335	-
Maceió	283 159	282 883	253 492	29 391	276
Major Isidoro	5 774	5 771	4 892	879	3
Mar Vermelho	1 141	1 140	1 051	89	1
Maragogi	9 848	9 818	6 478	3 340	30
Maravilha	3 200	3 199	2 571	628	1
Marechal Deodoro	14 329	14 290	11 457	2 833	39
Maribondo	4 201	4 199	3 712	487	2

Tabela 2.1.14 - Domicílios recenseados, por espécie, segundo os municípios - Alagoas - 2007

(conclusão)

Municípios	Domicílios recenseados, por espécie				
	Total	Particulares			Coletivos
		Total	Ocupados (1)	Não-ocupados	
Mata Grande	7 631	7 630	5 909	1 721	1
Matriz de Camaragibe	6 653	6 644	5 741	903	9
Messias	3 806	3 804	3 573	231	2
Minador do Negrão	1 609	1 609	1 364	245	-
Monteirópolis	1 957	1 957	1 651	306	-
Murici	7 157	7 155	6 175	980	2
Novo Lino	3 406	3 406	2 903	503	-
Olho d'Água das Flores	6 018	6 013	5 058	955	5
Olho d'Água do Casado	2 392	2 392	1 988	404	-
Olho d'Água Grande	1 436	1 436	1 213	223	-
Olivença	3 219	3 218	2 634	584	1
Ouro Branco	3 193	3 192	2 756	436	1
Palestina	1 270	1 270	1 142	128	-
Palmeira dos Índios	22 277	22 260	19 486	2 774	17
Pão de Açúcar	7 077	7 072	5 776	1 296	5
Pariconha	3 076	3 075	2 438	637	1
Paripueira	4 632	4 622	2 479	2 143	10
Passo de Camaragibe	3 872	3 871	3 339	532	1
Paulo Jacinto	2 237	2 232	1 999	233	5
Penedo	17 025	17 008	15 177	1 831	17
Piaçabuçu	5 243	5 234	4 511	723	9
Pilar	8 587	8 582	8 019	563	5
Pindoba	961	961	792	169	-
Piranhas	6 613	6 603	5 581	1 022	10
Poço das Trincheiras	3 290	3 290	2 876	414	-
Porto Calvo	7 050	7 042	5 958	1 084	8
Porto de Pedras	3 214	3 204	2 517	687	10
Porto Real do Colégio	5 405	5 403	4 571	832	2
Quebrangulo	3 417	3 416	2 938	478	1
Rio Largo	18 861	18 852	17 207	1 645	9
Roteiro	1 839	1 838	1 518	320	1
Santa Luzia do Norte	1 967	1 967	1 694	273	-
Santana do Ipanema	12 553	12 538	10 671	1 867	15
Santana do Mundaú	3 581	3 580	2 975	605	1
São Brás	2 012	2 012	1 753	259	-
São José da Laje	6 556	6 553	5 689	864	3
São José da Tapera	7 998	7 993	6 870	1 123	5
São Luís do Quitunde	8 273	8 271	7 367	904	2
São Miguel dos Campos	13 774	13 762	12 540	1 222	12
São Miguel dos Milagres	2 490	2 479	1 820	659	11
São Sebastião	9 414	9 408	7 851	1 557	6
Satuba	3 912	3 912	3 673	239	-
Senador Rui Palmeira	3 290	3 286	2 854	432	4
Tanque d'Arca	1 948	1 948	1 561	387	-
Taquarana	5 521	5 520	4 676	844	1
Teotônio Vilela	10 766	10 757	9 400	1 357	9
Traipu	7 174	7 173	5 994	1 179	1
União dos Palmares	17 572	17 561	15 689	1 872	11
Viçosa	7 331	7 325	6 605	720	6

Fonte: IBGE, Contagem da População 2007.

(1) Inclusive os domicílios fechados e os domicílios provenientes de setor censitário cujo arquivo foi danificado, com população estimada.

Tabela 2.1.15 - Domicílios recenseados, por espécie, segundo os municípios - Sergipe - 2007

(continua)

Municípios	Domicílios recenseados, por espécie				
	Total	Particulares			Coletivos
		Total	Ocupados (1)	Não-ocupados	
**Sergipe**	**626 396**	**625 844**	**534 075**	**91 769**	**552**
Amparo de São Francisco	726	726	625	101	-
Aquidabã	6 777	6 773	5 444	1 329	4
Aracaju	172 826	172 704	149 175	23 529	122
Arauá	3 455	3 455	2 976	479	-
Areia Branca	4 850	4 846	4 118	728	4
Barra dos Coqueiros	6 924	6 919	5 112	1 807	5
Boquim	7 954	7 949	6 547	1 402	5
Brejo Grande	2 322	2 317	1 913	404	5
Campo do Brito	5 554	5 554	4 845	709	-
Canhoba	1 254	1 254	994	260	-
Canindé de São Francisco	6 253	6 242	5 338	904	11
Capela	8 957	8 957	7 552	1 405	-
Carira	6 587	6 581	5 482	1 099	6
Carmópolis	3 595	3 587	3 178	409	8
Cedro de São João	1 886	1 886	1 561	325	-
Cristinápolis	4 633	4 628	3 908	720	5
Cumbe	1 402	1 402	1 069	333	-
Divina Pastora	1 350	1 350	1 100	250	-
Estância	20 745	20 721	16 921	3 800	24
Feira Nova	1 858	1 858	1 483	375	-
Frei Paulo	4 053	4 052	3 483	569	1
Gararu	3 914	3 912	3 141	771	2
General Maynard	949	949	807	142	-
Gracho Cardoso	1 864	1 861	1 597	264	3
Ilha das Flores	2 381	2 377	2 158	219	4
Indiaroba	4 832	4 827	4 176	651	5
Itabaiana	26 941	26 916	23 804	3 112	25
Itabaianinha	10 961	10 952	9 566	1 386	9
Itabi	1 966	1 965	1 420	545	1
Itaporanga d'Ajuda	9 365	9 357	7 227	2 130	8
Japaratuba	4 965	4 965	4 116	849	-
Japoatã	4 186	4 180	3 620	560	6
Lagarto	29 310	29 293	24 822	4 471	17
Laranjeiras	7 036	7 031	6 133	898	5
Macambira	2 093	2 093	1 854	239	-
Malhada dos Bois	1 052	1 052	974	78	-

Tabela 2.1.15 - Domicílios recenseados, por espécie, segundo os municípios - Sergipe - 2007

(conclusão)

Municípios	Domicílios recenseados, por espécie				
	Total	Particulares			Coletivos
		Total	Ocupados (1)	Não-ocupados	
Malhador	4 055	4 055	3 378	677	-
Maruim	4 549	4 547	3 898	649	2
Moita Bonita	3 655	3 650	3 262	388	5
Monte Alegre de Sergipe	4 171	4 169	3 459	710	2
Muribeca	2 247	2 247	1 950	297	-
Neópolis	5 632	5 626	5 012	614	6
Nossa Senhora Aparecida	2 838	2 837	2 419	418	1
Nossa Senhora da Glória	9 513	9 505	7 956	1 549	8
Nossa Senhora das Dores	8 031	8 027	6 813	1 214	4
Nossa Senhora de Lourdes	1 988	1 988	1 734	254	-
Nossa Senhora do Socorro	44 154	44 139	40 867	3 272	15
Pacatuba	3 967	3 967	3 216	751	-
Pedra Mole	997	997	808	189	-
Pedrinhas	2 601	2 600	2 184	416	1
Pinhão	1 972	1 972	1 557	415	-
Pirambu	2 932	2 925	2 090	835	7
Poço Redondo	8 566	8 562	6 963	1 599	4
Poço Verde	7 325	7 322	5 976	1 346	3
Porto da Folha	8 590	8 583	6 913	1 670	7
Propriá	8 511	8 497	7 565	932	14
Riachão do Dantas	5 801	5 801	4 754	1 047	-
Riachuelo	2 504	2 504	2 288	216	-
Ribeirópolis	5 425	5 422	4 674	748	3
Rosário do Catete	2 616	2 616	2 263	353	-
Salgado	6 176	6 175	4 967	1 208	1
Santa Luzia do Itanhy	3 877	3 860	3 291	569	17
Santa Rosa de Lima	1 269	1 269	1 030	239	-
Santana do São Francisco	1 924	1 921	1 643	278	3
Santo Amaro das Brotas	3 565	3 562	3 156	406	3
São Cristóvão	23 386	23 254	19 628	3 626	132
São Domingos	3 268	3 267	2 923	344	1
São Francisco	993	992	829	163	1
São Miguel do Aleixo	1 319	1 318	1 041	277	1
Simão Dias	12 730	12 718	10 545	2 173	12
Siriri	2 523	2 521	2 084	437	2
Telha	911	911	780	131	-
Tobias Barreto	15 781	15 773	13 180	2 593	8
Tomar do Geru	3 949	3 949	3 328	621	-
Umbaúba	6 309	6 305	5 412	893	4

Fonte: IBGE, Contagem da População 2007.

(1) Inclusive os domicílios fechados com população estimada.

Tabela 2.1.16 - Domicílios recenseados, por espécie, segundo os municípios - Bahia - 2007

(continua)

Municípios	Domicílios recenseados, por espécie				
	Total	Particulares			Coletivos
		Total	Ocupados (1)	Não-ocupados	
**Bahia**	**3 096 310**	**3 090 713**	**2 527 902**	**562 811**	**5 597**
Abaíra	3 794	3 789	2 598	1 191	5
Abaré	5 257	5 249	4 148	1 101	8
Acajutiba	4 885	4 883	3 861	1 022	2
Adustina	4 889	4 886	3 993	893	3
Água Fria	5 262	5 261	4 050	1 211	1
Aiquara	1 872	1 871	1 364	507	1
Alagoinhas	41 116	41 074	37 211	3 863	42
Alcobaça	7 192	7 158	5 188	1 970	34
Almadina	2 551	2 549	1 980	569	2
Amargosa	11 667	11 656	9 719	1 937	11
Amélia Rodrigues	7 093	7 089	5 804	1 285	4
América Dourada	5 283	5 282	4 116	1 166	1
Anagé	8 230	8 228	6 797	1 431	2
Andaraí	4 776	4 753	3 633	1 120	23
Andorinha	5 503	5 499	4 258	1 241	4
Angical	4 458	4 449	3 719	730	9
Anguera	2 845	2 844	2 264	580	1
Antas	4 900	4 896	3 997	899	4
Antônio Cardoso	3 278	3 278	2 718	560	-
Antônio Gonçalves	3 501	3 498	2 861	637	3
Aporá	6 143	6 134	4 945	1 189	9
Apuarema	2 472	2 469	1 957	512	3
Araças	3 502	3 497	2 887	610	5
Aracatu	4 466	4 464	3 530	934	2
Araci	15 977	15 967	12 770	3 197	10
Aramari	3 056	3 053	2 476	577	3
Arataca	5 139	5 137	3 073	2 064	2
Aratuípe	2 556	2 554	2 206	348	2
Aurelino Leal	4 911	4 909	3 940	969	2
Baianópolis	4 229	4 227	3 451	776	2
Baixa Grande	6 981	6 974	5 559	1 415	7
Banzaê	3 434	3 434	2 863	571	-
Barra	13 094	13 082	10 558	2 524	12
Barra da Estiva	6 138	6 131	5 202	929	7
Barra do Choça	9 350	9 306	8 080	1 226	44
Barra do Mendes	5 128	5 125	3 871	1 254	3
Barra do Rocha	1 833	1 833	1 632	201	-
Barreiras	39 812	39 703	35 187	4 516	109
Barro Alto	4 580	4 579	3 710	869	1
Barro Preto	2 877	2 876	1 886	990	1
Barrocas	4 060	4 057	3 407	650	3
Belmonte	8 212	8 196	5 987	2 209	16
Belo Campo	4 593	4 592	3 943	649	1
Biritinga	4 559	4 554	3 624	930	5
Boa Nova	5 255	5 253	4 057	1 196	2
Boa Vista do Tupim	6 054	6 050	4 579	1 471	4
Bom Jesus da Lapa	17 643	17 541	14 992	2 549	102
Bom Jesus da Serra	2 987	2 985	2 466	519	2

Tabela 2.1.16 - Domicílios recenseados, por espécie, segundo os municípios - Bahia - 2007

(continuação)

Municípios	Domicílios recenseados, por espécie				
	Total	Particulares			Coletivos
		Total	Ocupados (1)	Não-ocupados	
Boninal	4 175	4 169	3 253	916	6
Bonito	4 395	4 384	3 422	962	11
Boquira	7 155	7 151	5 483	1 668	4
Botuporã	3 568	3 566	2 758	808	2
Brejões	4 511	4 500	3 460	1 040	11
Brejolândia	3 058	3 056	2 535	521	2
Brotas de Macaúbas	4 126	4 125	3 058	1 067	1
Brumado	19 515	19 498	16 883	2 615	17
Buerarema	6 001	5 998	5 318	680	3
Buritirama	5 100	5 097	4 125	972	3
Caatiba	2 866	2 864	2 527	337	2
Cabaceiras do Paraguaçu	4 902	4 900	4 089	811	2
Cachoeira	10 711	10 613	8 758	1 855	98
Caculé	6 878	6 873	5 824	1 049	5
Caém	3 460	3 457	2 783	674	3
Caetanos	3 622	3 622	2 887	735	-
Caetité	14 485	14 468	11 868	2 600	17
Cafarnaum	5 824	5 819	4 519	1 300	5
Cairu	4 921	4 763	3 830	933	158
Caldeirão Grande	4 363	4 360	3 400	960	3
Camacan	11 110	11 096	8 305	2 791	14
Camamu	10 481	10 458	8 166	2 292	23
Campo Alegre de Lourdes	9 175	9 167	6 899	2 268	8
Campo Formoso	20 415	20 392	16 641	3 751	23
Canápolis	3 452	3 450	2 695	755	2
Canarana	8 040	8 035	6 670	1 365	5
Canavieiras	12 460	12 435	10 115	2 320	25
Candeal	3 043	3 043	2 438	605	-
Candeias	27 355	27 336	22 507	4 829	19
Candiba	4 227	4 227	3 341	886	-
Cândido Sales	8 296	8 286	7 050	1 236	10
Cansanção	10 706	10 695	8 404	2 291	11
Canudos	4 691	4 686	3 831	855	5
Capela do Alto Alegre	4 541	4 541	3 815	726	-
Capim Grosso	9 603	9 584	7 657	1 927	19
Caraíbas	3 141	3 129	2 585	544	12
Caravelas	7 172	7 160	5 730	1 430	12
Cardeal da Silva	2 673	2 670	2 130	540	3
Carinhanha	8 983	8 971	6 915	2 056	12
Casa Nova	18 314	18 307	15 750	2 557	7
Castro Alves	7 470	7 466	6 486	980	4
Catolândia	1 190	1 187	998	189	3
Catu	15 677	15 662	13 901	1 761	15
Caturama	2 862	2 861	2 231	630	1
Central	5 770	5 766	4 725	1 041	4
Chorrochó	3 682	3 675	2 573	1 102	7
Cícero Dantas	11 498	11 484	9 295	2 189	14
Cipó	4 697	4 688	3 942	746	9
Coaraci	7 962	7 958	6 636	1 322	4

Tabela 2.1.16 - Domicílios recenseados, por espécie, segundo os municípios - Bahia - 2007

(continuação)

Municípios	Domicílios recenseados, por espécie				
	Total	Particulares			Coletivos
		Total	Ocupados (1)	Não-ocupados	
Cocos	5 694	5 687	4 527	1 160	7
Conceição da Feira	5 738	5 735	4 997	738	3
Conceição do Almeida	5 651	5 644	4 877	767	7
Conceição do Coité	20 860	20 851	17 205	3 646	9
Conceição do Jacuípe	9 496	9 482	7 835	1 647	14
Conde	7 092	7 084	5 331	1 753	8
Condeúba	4 960	4 959	4 271	688	1
Contendas do Sincorá	1 538	1 534	1 095	439	4
Coração de Maria	7 315	7 310	5 959	1 351	5
Cordeiros	2 569	2 567	2 140	427	2
Coribe	5 016	5 011	4 002	1 009	5
Coronel João Sá	5 724	5 723	4 984	739	1
Correntina	9 925	9 880	8 280	1 600	45
Cotegipe	4 572	4 568	3 524	1 044	4
Cravolândia	1 984	1 983	1 491	492	1
Crisópolis	6 830	6 825	5 518	1 307	5
Cristópolis	4 269	4 264	3 511	753	5
Cruz das Almas	16 911	16 895	15 368	1 527	16
Curaçá	9 322	9 318	8 204	1 114	4
Dário Meira	4 056	4 052	3 200	852	4
Dias d'Ávila	19 386	19 367	15 271	4 096	19
Dom Basílio	3 188	3 185	2 741	444	3
Dom Macedo Costa	1 351	1 350	1 095	255	1
Elísio Medrado	2 979	2 976	2 353	623	3
Encruzilhada	7 054	7 048	5 993	1 055	6
Entre Rios	13 408	13 348	10 116	3 232	60
Érico Cardoso	3 196	3 185	2 550	635	11
Esplanada	9 317	9 295	7 714	1 581	22
Euclides da Cunha	18 035	18 013	15 084	2 929	22
Eunápolis	30 787	30 748	26 257	4 491	39
Fátima	6 324	6 322	5 278	1 044	2
Feira da Mata	2 197	2 196	1 676	520	1
Filadélfia	5 345	5 342	4 367	975	3
Firmino Alves	1 903	1 902	1 565	337	1
Floresta Azul	3 515	3 513	2 884	629	2
Formosa do Rio Preto	6 257	6 250	4 914	1 336	7
Gandu	10 192	10 165	8 572	1 593	27
Gavião	1 799	1 799	1 294	505	-
Gentio do Ouro	3 716	3 712	2 888	824	4
Glória	4 926	4 923	3 789	1 134	3
Gongogi	2 492	2 492	2 000	492	-
Governador Mangabeira	6 066	6 060	5 178	882	6
Guajeru	2 180	2 180	1 815	365	-
Guanambi	24 063	24 039	20 714	3 325	24
Guaratinga	8 450	8 443	6 399	2 044	7
Heliópolis	4 605	4 600	3 899	701	5
Iaçu	9 642	9 639	7 436	2 203	3
Ibiassucê	2 978	2 976	2 515	461	2
Ibicaraí	8 580	8 575	7 224	1 351	5

Tabela 2.1.16 - Domicílios recenseados, por espécie, segundo os municípios - Bahia - 2007

(continuação)

Municípios	Domicílios recenseados, por espécie				
	Total	Particulares			Coletivos
		Total	Ocupados (1)	Não-ocupados	
Ibicoara	4 968	4 950	4 081	869	18
Ibicuí	5 484	5 477	4 469	1 008	7
Ibipeba	5 825	5 818	4 647	1 171	7
Ibipitanga	4 698	4 697	3 605	1 092	1
Ibiquera	1 904	1 901	1 361	540	3
Ibirapitanga	7 529	7 519	6 115	1 404	10
Ibirapuã	2 331	2 328	2 201	127	3
Ibirataia	6 638	6 627	6 073	554	11
Ibitiara	5 238	5 236	4 063	1 173	2
Ibititá	6 505	6 500	5 148	1 352	5
Ibotirama	8 035	8 014	6 676	1 338	21
Ichu	2 027	2 026	1 542	484	1
Igaporã	4 569	4 566	3 707	859	3
Igrapiúna	4 182	4 179	3 341	838	3
Iguaí	8 313	8 308	7 016	1 292	5
Inhambupe	10 835	10 825	9 034	1 791	10
Ipecaetá	4 895	4 892	3 944	948	3
Ipiaú	14 312	14 290	12 131	2 159	22
Ipirá	19 387	19 364	16 064	3 300	23
Ipupiara	3 263	3 259	2 583	676	4
Irajuba	2 511	2 506	1 954	552	5
Iramaia	4 396	4 388	3 597	791	8
Iraquara	7 173	7 162	5 610	1 552	11
Irará	7 655	7 651	6 569	1 082	4
Irecê	20 545	20 520	17 481	3 039	25
Itabela	8 022	8 012	6 828	1 184	10
Itaberaba	19 703	19 675	16 278	3 397	28
Itacaré	7 846	7 739	6 293	1 446	107
Itaeté	4 889	4 886	3 694	1 192	3
Itagi	4 081	4 080	3 433	647	1
Itagibá	5 633	5 630	4 324	1 306	3
Itagimirim	2 441	2 435	2 000	435	6
Itaguaçu da Bahia	4 069	4 068	3 092	976	1
Itaju do Colônia	3 428	3 426	2 226	1 200	2
Itajuípe	7 527	7 525	5 733	1 792	2
Itamaraju	22 527	22 476	18 162	4 314	51
Itamari	3 163	3 158	2 383	775	5
Itambé	8 681	8 676	8 131	545	5
Itanagra	2 308	2 308	1 691	617	-
Itanhém	7 050	7 046	5 953	1 093	4
Itaparica	10 284	10 257	5 836	4 421	27
Itapé	3 734	3 732	3 076	656	2
Itapebi	4 220	4 208	2 988	1 220	12
Itapetinga	19 190	19 143	17 190	1 953	47
Itapicuru	10 349	10 345	8 198	2 147	4
Itapitanga	3 113	3 108	2 629	479	5
Itaquara	2 591	2 590	2 158	432	1
Itarantim	5 562	5 555	4 699	856	7
Itatim	4 603	4 595	3 838	757	8

Tabela 2.1.16 - Domicílios recenseados, por espécie, segundo os municípios - Bahia - 2007

(continuação)

Municípios	Domicílios recenseados, por espécie				
	Total	Particulares			Coletivos
		Total	Ocupados (1)	Não-ocupados	
Itiruçu	4 775	4 772	3 922	850	3
Itiúba	11 754	11 750	9 297	2 453	4
Itororó	6 937	6 930	5 657	1 273	7
Ituaçu	5 952	5 948	4 700	1 248	4
Ituberá	7 222	7 212	6 112	1 100	10
Iuiú	3 414	3 412	2 704	708	2
Jaborandi	3 023	3 015	2 434	581	8
Jacaraci	4 300	4 294	3 515	779	6
Jacobina	27 141	27 124	22 778	4 346	17
Jaguaquara	15 073	15 057	12 610	2 447	16
Jaguarari	10 506	10 498	8 332	2 166	8
Jaguaripe	5 770	5 762	4 229	1 533	8
Jandaíra	2 909	2 901	2 563	338	8
Jequié	46 664	46 631	41 130	5 501	33
Jeremoabo	12 253	12 243	10 075	2 168	10
Jiquiriçá	4 239	4 236	3 619	617	3
Jitaúna	5 565	5 562	4 577	985	3
João Dourado	6 138	6 132	5 407	725	6
Jucuruçu	3 386	3 382	2 933	449	4
Jussara	4 887	4 886	3 959	927	1
Jussari	2 719	2 718	1 949	769	1
Jussiape	2 928	2 924	2 331	593	4
Lafaiete Coutinho	1 305	1 305	1 092	213	-
Lagoa Real	3 599	3 597	3 076	521	2
Laje	6 589	6 585	5 667	918	4
Lajedão	1 298	1 297	1 074	223	1
Lajedinho	1 389	1 385	1 134	251	4
Lajedo do Tabocal	2 749	2 749	2 357	392	-
Lamarão	3 525	3 525	2 862	663	-
Lapão	7 792	7 791	6 741	1 050	1
Lauro de Freitas	47 597	47 556	42 082	5 474	41
Lençóis	3 691	3 639	2 615	1 024	52
Licínio de Almeida	4 163	4 160	3 400	760	3
Livramento de Nossa Senhora	12 303	12 283	10 625	1 658	20
Luís Eduardo Magalhães	14 604	14 563	12 371	2 192	41
Macajuba	3 809	3 804	3 077	727	5
Macarani	5 320	5 316	4 489	827	4
Macaúbas	14 138	14 127	11 113	3 014	11
Macururé	2 836	2 834	1 968	866	2
Madre de Deus	5 642	5 632	4 242	1 390	10
Maetinga	2 598	2 595	2 126	469	3
Maiquinique	2 866	2 866	2 403	463	-
Mairi	6 682	6 678	5 406	1 272	4
Malhada	4 378	4 375	3 589	786	3
Malhada de Pedras	2 482	2 482	2 026	456	-
Manoel Vitorino	5 101	5 100	3 708	1 392	1
Mansidão	3 142	3 136	2 580	556	6
Maracás	9 567	9 558	8 339	1 219	9
Maragogipe	13 411	13 395	11 436	1 959	16

Tabela 2.1.16 - Domicílios recenseados, por espécie, segundo os municípios - Bahia - 2007

(continuação)

Municípios	Domicílios recenseados, por espécie				
	Total	Particulares			Coletivos
		Total	Ocupados (1)	Não-ocupados	
Maraú	6 319	6 256	4 629	1 627	63
Marcionílio Souza	3 680	3 676	2 788	888	4
Mascote	5 677	5 672	4 330	1 342	5
Mata de São João	13 574	12 819	10 255	2 564	755
Matina	3 482	3 479	2 786	693	3
Medeiros Neto	7 585	7 578	6 449	1 129	7
Miguel Calmon	9 956	9 946	7 976	1 970	10
Milagres	4 006	4 005	3 448	557	1
Mirangaba	5 440	5 435	4 578	857	5
Mirante	2 867	2 866	2 260	606	1
Monte Santo	17 466	17 459	13 753	3 706	7
Morpará	3 027	3 020	2 319	701	7
Morro do Chapéu	11 733	11 713	9 173	2 540	20
Mortugaba	4 127	4 120	3 402	718	7
Mucugê	4 349	4 335	3 698	637	14
Mucuri	12 250	12 177	9 227	2 950	73
Mulungu do Morro	4 033	4 030	3 406	624	3
Mundo Novo	7 784	7 777	6 425	1 352	7
Muniz Ferreira	2 328	2 328	1 989	339	-
Muquém de São Francisco	3 005	2 997	2 426	571	8
Muritiba	8 859	8 854	7 530	1 324	5
Mutuípe	7 411	7 404	6 101	1 303	7
Nazaré	8 823	8 814	7 481	1 333	9
Nilo Peçanha	4 201	4 198	3 336	862	3
Nordestina	3 822	3 821	3 046	775	1
Nova Canaã	5 630	5 629	4 809	820	1
Nova Fátima	2 876	2 870	2 245	625	6
Nova Ibiá	2 394	2 394	1 950	444	-
Nova Itarana	2 574	2 571	1 993	578	3
Nova Redenção	2 871	2 865	2 255	610	6
Nova Soure	8 489	8 485	6 932	1 553	4
Nova Viçosa	12 912	12 865	9 037	3 828	47
Novo Horizonte	3 610	3 606	2 851	755	4
Novo Triunfo	4 055	4 055	3 304	751	-
Olindina	7 671	7 665	6 575	1 090	6
Oliveira dos Brejinhos	7 679	7 671	5 989	1 682	8
Ouriçangas	2 619	2 619	2 098	521	-
Ourolândia	5 169	5 167	4 263	904	2
Palmas de Monte Alto	6 737	6 730	5 183	1 547	7
Palmeiras	3 088	3 075	2 179	896	13
Paramirim	5 934	5 927	5 120	807	7
Paratinga	8 105	8 102	6 726	1 376	3
Paripiranga	9 751	9 747	7 906	1 841	4
Pau Brasil	4 333	4 332	3 272	1 060	1
Paulo Afonso	31 625	31 577	28 207	3 370	48

Tabela 2.1.16 - Domicílios recenseados, por espécie, segundo os municípios - Bahia - 2007

(continuação)

Municípios	Domicílios recenseados, por espécie				
	Total	Particulares			Coletivos
		Total	Ocupados (1)	Não-ocupados	
Pé de Serra	4 791	4 788	3 793	995	3
Pedrão	2 258	2 258	1 885	373	-
Pedro Alexandre	4 425	4 421	4 033	388	4
Piatã	6 069	6 058	4 588	1 470	11
Pilão Arcado	9 520	9 508	7 755	1 753	12
Pindaí	4 913	4 912	3 864	1 048	1
Pindobaçu	7 436	7 428	5 856	1 572	8
Pintadas	3 602	3 601	2 757	844	1
Piraí do Norte	2 584	2 583	2 322	261	1
Piripá	3 670	3 668	3 243	425	2
Piritiba	7 569	7 559	6 472	1 087	10
Planaltino	2 931	2 929	2 396	533	2
Planalto	7 129	7 066	5 776	1 290	63
Poções	15 466	15 456	12 201	3 255	10
Pojuca	9 773	9 765	8 636	1 129	8
Ponto Novo	5 185	5 182	3 967	1 215	3
Porto Seguro	44 697	44 246	32 720	11 526	451
Potiraguá	3 148	3 141	2 650	491	7
Prado	10 012	9 873	6 623	3 250	139
Presidente Dutra	4 727	4 717	3 934	783	10
Presidente Jânio Quadros	4 955	4 952	3 892	1 060	3
Presidente Tancredo Neves	6 949	6 943	5 906	1 037	6
Queimadas	9 091	9 088	7 308	1 780	3
Quijingue	8 497	8 490	6 804	1 686	7
Quixabeira	3 724	3 723	2 790	933	1
Rafael Jambeiro	6 842	6 830	5 893	937	12
Remanso	11 874	11 859	9 979	1 880	15
Retirolândia	4 523	4 522	3 476	1 046	1
Riachão das Neves	7 034	7 026	5 801	1 225	8
Riachão do Jacuípe	11 480	11 471	9 345	2 126	9
Riacho de Santana	9 171	9 164	7 411	1 753	7
Ribeira do Amparo	4 519	4 516	3 712	804	3
Ribeira do Pombal	15 168	15 158	13 022	2 136	10
Ribeirão do Largo	3 512	3 507	3 217	290	5
Rio de Contas	4 380	4 369	3 636	733	11
Rio do Antônio	4 887	4 882	3 959	923	5
Rio do Pires	4 023	4 022	3 061	961	1
Rio Real	11 448	11 435	9 861	1 574	13
Rodelas	2 045	2 043	1 850	193	2
Ruy Barbosa	9 808	9 799	8 154	1 645	9
Salinas da Margarida	5 039	5 030	3 426	1 604	9
Santa Bárbara	6 462	6 457	5 248	1 209	5
Santa Brígida	5 010	5 007	3 983	1 024	3
Santa Cruz Cabrália	9 433	9 373	7 058	2 315	60

Tabela 2.1.16 - Domicílios recenseados, por espécie, segundo os municípios - Bahia - 2007

(continuação)

Municípios	Domicílios recenseados, por espécie				
	Total	Particulares			Coletivos
		Total	Ocupados (1)	Não-ocupados	
Santa Cruz da Vitória	1 888	1 886	1 637	249	2
Santa Inês	4 094	4 090	3 150	940	4
Santa Luzia	5 084	5 079	4 067	1 012	5
Santa Maria da Vitória	12 559	12 544	10 566	1 978	15
Santa Rita de Cássia	9 101	9 090	6 776	2 314	11
Santa Teresinha	3 430	3 422	2 767	655	8
Santaluz	10 691	10 684	8 989	1 695	7
Santana	8 686	8 676	6 948	1 728	10
Santanópolis	2 962	2 962	2 412	550	-
Santo Amaro	20 781	20 769	16 344	4 425	12
Santo Antônio de Jesus	26 095	26 041	24 257	1 784	54
Santo Estêvão	13 860	13 853	11 634	2 219	7
São Desidério	7 775	7 636	6 246	1 390	139
São Domingos	2 998	2 996	2 592	404	2
São Felipe	6 354	6 346	5 565	781	8
São Félix	3 912	3 908	3 667	241	4
São Félix do Coribe	4 070	4 065	3 436	629	5
São Francisco do Conde	10 092	10 082	8 146	1 936	10
São Gabriel	5 724	5 720	4 892	828	4
São Gonçalo dos Campos	8 740	8 732	7 392	1 340	8
São José da Vitória	2 044	2 044	1 674	370	-
São José do Jacuípe	3 768	3 767	2 975	792	1
São Miguel das Matas	3 335	3 332	2 925	407	3
São Sebastião do Passé	13 604	13 593	11 032	2 561	11
Sapeaçu	5 202	5 196	4 568	628	6
Sátiro Dias	5 698	5 695	4 714	981	3
Saubara	7 565	7 549	3 270	4 279	16
Saúde	4 428	4 425	3 556	869	3
Seabra	13 253	13 226	10 530	2 696	27
Sebastião Laranjeiras	3 130	3 125	2 628	497	5
Senhor do Bonfim	23 595	23 573	20 103	3 470	22
Sento Sé	10 065	10 048	8 480	1 568	17
Serra do Ramalho	9 483	9 472	7 514	1 958	11
Serra Dourada	5 598	5 593	4 532	1 061	5
Serra Preta	5 351	5 349	4 060	1 289	2
Serrinha	22 955	22 933	19 401	3 532	22
Serrolândia	4 773	4 768	3 909	859	5
Simões Filho	35 814	35 794	31 563	4 231	20
Sítio do Mato	3 629	3 625	2 920	705	4

Tabela 2.1.16 - Domicílios recenseados, por espécie, segundo os municípios - Bahia - 2007

(conclusão)

Municípios	Domicílios recenseados, por espécie				
	Total	Particulares			Coletivos
		Total	Ocupados (1)	Não-ocupados	
Sítio do Quinto	5 090	5 088	4 005	1 083	2
Sobradinho	6 811	6 808	5 568	1 240	3
Souto Soares	5 015	5 014	4 252	762	1
Tabocas do Brejo Velho	3 806	3 804	3 167	637	2
Tanhaçu	6 557	6 550	5 188	1 362	7
Tanque Novo	4 951	4 950	4 043	907	1
Tanquinho	2 402	2 402	1 973	429	-
Taperoá	5 416	5 413	4 624	789	3
Tapiramutá	4 713	4 709	4 099	610	4
Teixeira de Freitas	39 245	39 198	33 707	5 491	47
Teodoro Sampaio	3 133	3 129	2 396	733	4
Teofilândia	6 384	6 377	5 076	1 301	7
Teolândia	3 855	3 853	3 276	577	2
Terra Nova	4 007	4 004	3 268	736	3
Tremedal	6 107	6 103	4 901	1 202	4
Tucano	16 423	16 396	13 172	3 224	27
Uauá	8 058	8 051	6 628	1 423	7
Ubaíra	7 105	7 099	5 702	1 397	6
Ubaitaba	6 940	6 926	5 569	1 357	14
Ubatã	8 062	8 057	6 645	1 412	5
Uibaí	4 690	4 689	3 983	706	1
Umburanas	4 342	4 337	3 870	467	5
Una	8 472	8 459	7 100	1 359	13
Urandi	5 044	5 040	3 991	1 049	4
Uruçuca	7 661	7 644	5 931	1 713	17
Utinga	6 195	6 191	4 918	1 273	4
Valença	28 454	28 355	23 651	4 704	99
Valente	7 909	7 906	6 404	1 502	3
Várzea da Roça	5 034	5 028	4 190	838	6
Várzea do Poço	3 386	3 384	2 654	730	2
Várzea Nova	4 616	4 614	3 801	813	2
Varzedo	2 999	2 997	2 569	428	2
Vera Cruz	26 602	26 540	10 438	16 102	62
Vereda	2 351	2 348	1 972	376	3
Wagner	2 845	2 844	2 305	539	1
Wanderley	4 168	4 161	3 405	756	7
Wenceslau Guimarães	7 006	7 000	6 146	854	6
Xique-Xique	13 034	13 016	11 006	2 010	18

Fonte: IBGE, Contagem da População 2007.

(1) Inclusive os domicílios fechados e os domicílios provenientes de setor censitário cujo arquivo foi danificado, com população estimada.

Tabela 2.1.17 - Domicílios recenseados, por espécie, segundo os municípios - Minas Gerais - 2007

(continua)

Municípios	Domicílios recenseados, por espécie				
	Total	Particulares			Coletivos
		Total	Ocupados (1)	Não-ocupados	
**Minas Gerais**	**4 356 906**	**4 349 260**	**3 671 809**	**677 451**	**7 646**
Abadia dos Dourados	2 784	2 773	2 273	500	11
Abaeté	8 894	8 882	7 320	1 562	12
Abre Campo	4 191	4 185	3 631	554	6
Acaiaca	1 402	1 401	1 194	207	1
Açucena	3 479	3 474	3 023	451	5
Água Boa	5 245	5 241	4 136	1 105	4
Água Comprida	805	805	692	113	-
Aguanil	1 931	1 929	1 328	601	2
Águas Formosas	5 854	5 845	5 066	779	9
Águas Vermelhas	3 741	3 738	3 245	493	3
Aimorés	9 148	9 130	7 669	1 461	18
Aiuruoca	2 687	2 667	1 916	751	20
Alagoa	1 174	1 169	862	307	5
Albertina	887	887	867	20	-
Além Paraíba	12 050	12 039	10 405	1 634	11
Alfenas	26 866	26 812	21 848	4 964	54
Alfredo Vasconcelos	2 044	2 044	1 672	372	-
Almenara	11 874	11 853	10 409	1 444	21
Alpercata	2 360	2 358	2 008	350	2
Alpinópolis	6 381	6 372	5 299	1 073	9
Alterosa	5 089	5 083	4 265	818	6
Alto Caparaó	1 875	1 865	1 576	289	10
Alto Jequitibá	3 014	3 010	2 525	485	4
Alto Rio Doce	4 636	4 629	3 649	980	7
Alvarenga	1 465	1 462	1 374	88	3
Alvinópolis	4 987	4 981	4 380	601	6
Alvorada de Minas	1 087	1 085	890	195	2
Amparo do Serra	1 784	1 783	1 433	350	1
Andradas	13 034	13 023	11 225	1 798	11
Andrelândia	5 085	5 068	3 703	1 365	17
Angelândia	2 540	2 537	2 034	503	3
Antônio Carlos	3 921	3 917	3 314	603	4
Antônio Dias	3 406	3 399	2 616	783	7
Antônio Prado de Minas	684	684	585	99	-
Araçaí	838	835	712	123	3
Aracitaba	852	839	594	245	13
Araçuaí	11 206	11 182	9 311	1 871	24
Araguari	36 519	36 474	33 262	3 212	45
Arantina	1 183	1 183	814	369	-
Araponga	2 504	2 502	2 166	336	2
Araporã	1 796	1 790	1 729	61	6
Arapuá	972	971	904	67	1
Araújos	2 739	2 734	2 193	541	5
Araxá	29 545	29 509	26 690	2 819	36
Arceburgo	3 010	3 007	2 415	592	3
Arcos	12 423	12 418	10 610	1 808	5
Areado	4 872	4 858	4 112	746	14
Argirita	1 184	1 181	939	242	3
Aricanduva	1 443	1 438	1 226	212	5
Arinos	5 495	5 481	4 751	730	14
Astolfo Dutra	4 272	4 268	3 789	479	4
Ataléia	5 486	5 484	4 303	1 181	2
Augusto de Lima	1 708	1 702	1 325	377	6

Tabela 2.1.17 - Domicílios recenseados, por espécie, segundo os municípios - Minas Gerais - 2007

(continuação)

Municípios	Domicílios recenseados, por espécie				
	Total	Particulares			Coletivos
		Total	Ocupados (1)	Não-ocupados	
Baependi	6 648	6 635	5 258	1 377	13
Baldim	3 392	3 383	2 531	852	9
Bambuí	9 217	9 189	7 516	1 673	28
Bandeira	1 701	1 699	1 554	145	2
Bandeira do Sul	1 761	1 759	1 558	201	2
Barão de Cocais	8 474	8 324	7 312	1 012	150
Barão de Monte Alto	2 064	2 064	1 653	411	-
Barbacena	42 650	42 584	36 908	5 676	66
Barra Longa	2 506	2 505	2 070	435	1
Barroso	6 540	6 533	5 716	817	7
Bela Vista de Minas	3 103	3 101	2 719	382	2
Belmiro Braga	1 303	1 299	932	367	4
Belo Oriente	6 757	6 735	5 894	841	22
Belo Vale	2 761	2 759	2 242	517	2
Berilo	4 437	4 418	3 309	1 109	19
Berizal	1 422	1 418	1 188	230	4
Bertópolis	1 389	1 388	1 216	172	1
Bias Fortes	1 566	1 566	1 170	396	-
Bicas	5 205	5 201	4 393	808	4
Biquinhas	1 170	1 169	937	232	1
Boa Esperança	12 505	12 484	10 720	1 764	21
Bocaina de Minas	2 779	2 723	1 709	1 014	56
Bocaiúva	14 891	14 874	12 274	2 600	17
Bom Despacho	14 676	14 639	12 752	1 887	37
Bom Jardim de Minas	2 841	2 834	2 123	711	7
Bom Jesus da Penha	1 528	1 527	1 255	272	1
Bom Jesus do Amparo	1 880	1 878	1 471	407	2
Bom Jesus do Galho	5 377	5 371	4 557	814	6
Bom Repouso	3 550	3 545	3 146	399	5
Bom Sucesso	5 799	5 793	5 014	779	6
Bonfim	3 496	3 494	2 284	1 210	2
Bonfinópolis de Minas	2 104	2 098	1 760	338	6
Bonito de Minas	2 413	2 411	1 965	446	2
Borda da Mata	5 691	5 684	4 705	979	7
Botelhos	5 739	5 733	4 718	1 015	6
Botumirim	2 156	2 141	1 625	516	15
Brás Pires	1 734	1 732	1 380	352	2
Brasilândia de Minas	4 337	4 328	3 441	887	9
Brasília de Minas	9 546	9 535	7 962	1 573	11
Brasópolis	5 699	5 688	4 402	1 286	11
Braúnas	1 704	1 701	1 483	218	3
Brumadinho	13 577	13 550	9 650	3 900	27
Bueno Brandão	3 888	3 865	3 339	526	23
Buenópolis	3 574	3 572	2 788	784	2
Bugre	1 504	1 504	1 213	291	-
Buritis	7 467	7 453	6 142	1 311	14
Buritizeiro	8 083	8 077	6 941	1 136	6
Cabeceira Grande	2 551	2 551	1 903	648	-
Cabo Verde	4 937	4 925	4 076	849	12
Cachoeira da Prata	1 290	1 289	1 095	194	1
Cachoeira de Minas	4 096	4 090	3 241	849	6
Cachoeira de Pajeú	2 980	2 974	2 368	606	6
Cachoeira Dourada	902	899	741	158	3
Caetanópolis	3 308	3 303	2 849	454	5

Tabela 2.1.17 - Domicílios recenseados, por espécie, segundo os municípios - Minas Gerais - 2007

(continuação)

Municípios	Domicílios recenseados, por espécie				
	Total	Particulares			Coletivos
		Total	Ocupados (1)	Não-ocupados	
Caeté	13 289	13 267	10 905	2 362	22
Caiana	1 691	1 690	1 383	307	1
Cajuri	1 327	1 327	1 136	191	-
Caldas	5 650	5 634	4 554	1 080	16
Camacho	1 145	1 145	960	185	-
Camanducaia	8 642	8 531	6 210	2 321	111
Cambuí	9 839	9 832	8 220	1 612	7
Cambuquira	4 748	4 731	3 766	965	17
Campanário	1 157	1 153	961	192	4
Campanha	5 373	5 365	4 496	869	8
Campestre	7 412	7 402	6 315	1 087	10
Campina Verde	6 979	6 971	6 220	751	8
Campo Azul	1 108	1 104	981	123	4
Campo Belo	18 827	18 815	16 456	2 359	12
Campo do Meio	4 002	4 000	3 346	654	2
Campo Florido	2 140	2 140	1 852	288	-
Campos Altos	4 214	4 209	3 842	367	5
Campos Gerais	9 738	9 729	7 979	1 750	9
Cana Verde	2 179	2 178	1 728	450	1
Canaã	1 560	1 559	1 342	217	1
Canápolis	3 597	3 579	3 417	162	18
Candeias	6 118	6 111	5 033	1 078	7
Cantagalo	1 258	1 257	1 042	215	1
Caparaó	1 775	1 774	1 417	357	1
Capela Nova	1 770	1 767	1 370	397	3
Capelinha	10 432	10 418	8 684	1 734	14
Capetinga	2 542	2 535	2 231	304	7
Capim Branco	3 282	3 279	2 529	750	3
Capinópolis	5 288	5 278	4 833	445	10
Capitão Andrade	1 750	1 749	1 503	246	1
Capitão Enéas	4 130	4 123	3 566	557	7
Capitólio	3 613	3 602	2 486	1 116	11
Caputira	2 606	2 604	2 420	184	2
Caraí	6 576	6 573	5 427	1 146	3
Caranaíba	1 068	1 068	909	159	-
Carandaí	7 080	7 074	6 202	872	6
Carangola	12 655	12 635	10 491	2 144	20
Caratinga	28 264	28 210	24 375	3 835	54
Carbonita	3 679	3 657	2 929	728	22
Careaçu	2 059	2 054	1 798	256	5
Carlos Chagas	7 281	7 274	6 307	967	7
Carmésia	905	902	722	180	3
Carmo da Cachoeira	3 842	3 826	3 188	638	16
Carmo da Mata	4 299	4 281	3 382	899	18
Carmo de Minas	4 605	4 599	3 724	875	6
Carmo do Cajuru	7 062	7 055	5 665	1 390	7
Carmo do Paranaíba	10 715	10 706	9 689	1 017	9
Carmo do Rio Claro	7 434	7 425	5 956	1 469	9
Carmópolis de Minas	5 746	5 739	4 467	1 272	7
Carneirinho	3 315	3 312	2 904	408	3
Carrancas	1 526	1 519	1 182	337	7
Carvalhópolis	1 111	1 110	981	129	1
Carvalhos	1 960	1 956	1 462	494	4
Casa Grande	726	726	573	153	-

Tabela 2.1.17 - Domicílios recenseados, por espécie, segundo os municípios - Minas Gerais - 2007

(continuação)

Municípios	Domicílios recenseados, por espécie				
	Total	Particulares			Coletivos
		Total	Ocupados (1)	Não-ocupados	
Cascalho Rico	1 258	1 254	953	301	4
Cássia	6 322	6 315	5 457	858	7
Cataguases	24 680	24 656	21 286	3 370	24
Catas Altas	1 463	1 447	1 284	163	16
Catas Altas da Noruega	1 127	1 126	885	241	1
Catuji	2 242	2 241	1 686	555	1
Catuti	1 807	1 807	1 411	396	-
Caxambu	8 779	8 759	6 434	2 325	20
Cedro do Abaeté	541	541	414	127	-
Central de Minas	2 419	2 419	2 186	233	-
Centralina	3 467	3 444	3 168	276	23
Chácara	1 276	1 274	854	420	2
Chalé	2 054	2 051	1 660	391	3
Chapada do Norte	4 875	4 870	3 579	1 291	5
Chapada Gaúcha	2 732	2 726	2 308	418	6
Chiador	1 181	1 181	909	272	-
Cipotânea	2 495	2 490	1 845	645	5
Claraval	1 738	1 734	1 275	459	4
Claro dos Poções	2 951	2 947	2 350	597	4
Cláudio	8 860	8 848	7 143	1 705	12
Coimbra	2 656	2 652	2 164	488	4
Coluna	2 774	2 772	2 397	375	2
Comendador Gomes	1 036	1 033	965	68	3
Comercinho	2 848	2 847	2 332	515	1
Conceição da Aparecida	3 587	3 581	3 140	441	6
Conceição da Barra de Minas	1 495	1 494	1 151	343	1
Conceição das Alagoas	6 853	6 843	5 966	877	10
Conceição das Pedras	885	885	790	95	-
Conceição de Ipanema	1 739	1 739	1 402	337	-
Conceição do Mato Dentro	6 652	6 621	4 967	1 654	31
Conceição do Pará	2 037	2 036	1 406	630	1
Conceição do Rio Verde	4 610	4 602	3 670	932	8
Conceição dos Ouros	3 485	3 481	2 973	508	4
Cônego Marinho	1 937	1 933	1 541	392	4
Confins	2 004	2 000	1 581	419	4
Congonhal	3 665	3 663	3 075	588	2
Congonhas	13 849	13 792	12 204	1 588	57
Congonhas do Norte	2 041	2 038	1 368	670	3
Conquista	2 323	2 318	1 979	339	5
Conselheiro Lafaiete	35 765	35 727	31 790	3 937	38
Conselheiro Pena	8 677	8 669	6 917	1 752	8
Consolação	740	738	544	194	2
Coqueiral	3 328	3 325	2 770	555	3
Coração de Jesus	8 819	8 807	7 197	1 610	12
Cordisburgo	3 151	3 149	2 530	619	2
Cordislândia	1 270	1 269	1 019	250	1
Corinto	7 902	7 889	6 736	1 153	13
Coroaci	3 329	3 326	2 870	456	3
Coromandel	11 281	11 237	9 126	2 111	44
Coronel Fabriciano	32 284	32 256	29 359	2 897	28
Coronel Murta	2 886	2 883	2 380	503	3
Coronel Pacheco	1 004	1 004	702	302	-
Coronel Xavier Chaves	1 139	1 138	874	264	1
Córrego Danta	1 528	1 526	1 137	389	2
Córrego do Bom Jesus	1 731	1 727	1 250	477	4
Córrego Fundo	2 015	2 014	1 685	329	1
Córrego Novo	1 163	1 162	982	180	1

Tabela 2.1.17 - Domicílios recenseados, por espécie, segundo os municípios - Minas Gerais - 2007

(continuação)

Municípios	Domicílios recenseados, por espécie				
	Total	Particulares			Coletivos
		Total	Ocupados (1)	Não-ocupados	
Couto de Magalhães de Minas	1 467	1 463	1 157	306	4
Crisólita	1 748	1 746	1 555	191	2
Cristais	3 947	3 942	3 388	554	5
Cristália	1 810	1 809	1 470	339	1
Cristiano Otoni	1 612	1 610	1 349	261	2
Cristina	3 542	3 537	3 069	468	5
Crucilândia	2 049	2 044	1 478	566	5
Cruzeiro da Fortaleza	1 328	1 326	1 156	170	2
Cruzília	5 483	5 476	4 333	1 143	7
Cuparaque	1 823	1 822	1 417	405	1
Curral de Dentro	2 353	2 345	1 824	521	8
Curvelo	23 855	23 821	21 336	2 485	34
Datas	1 577	1 576	1 291	285	1
Delfim Moreira	2 987	2 984	2 242	742	3
Delfinópolis	3 016	2 994	2 142	852	22
Delta	2 215	2 213	1 852	361	2
Descoberto	2 022	2 019	1 586	433	3
Desterro de Entre Rios	3 030	3 025	2 217	808	5
Desterro do Melo	1 144	1 140	953	187	4
Diamantina	14 866	14 809	11 876	2 933	57
Diogo de Vasconcelos	1 357	1 355	1 107	248	2
Dionísio	3 006	3 003	2 773	230	3
Divinésia	1 213	1 207	991	216	6
Divino	6 930	6 923	5 779	1 144	7
Divino das Laranjeiras	1 936	1 935	1 586	349	1
Divinolândia de Minas	2 106	2 105	1 778	327	1
Divisa Alegre	1 778	1 776	1 555	221	2
Divisa Nova	1 979	1 977	1 655	322	2
Divisópolis	2 619	2 618	2 171	447	1
Dom Bosco	1 559	1 558	1 176	382	1
Dom Cavati	2 032	2 027	1 739	288	5
Dom Joaquim	1 701	1 699	1 294	405	2
Dom Silvério	1 994	1 989	1 628	361	5
Dom Viçoso	1 087	1 087	936	151	-
Dona Eusébia	2 055	2 053	1 685	368	2
Dores de Campos	3 282	3 276	2 843	433	6
Dores de Guanhães	1 612	1 606	1 420	186	6
Dores do Indaiá	5 896	5 891	4 785	1 106	5
Dores do Turvo	1 711	1 709	1 289	420	2
Doresópolis	602	602	484	118	-
Douradoquara	845	844	657	187	1
Durandé	2 307	2 307	1 947	360	-
Elói Mendes	8 234	8 229	7 090	1 139	5
Engenheiro Caldas	3 525	3 518	2 999	519	7
Engenheiro Navarro	2 281	2 280	1 931	349	1
Entre Folhas	1 791	1 790	1 476	314	1
Entre Rios de Minas	4 817	4 808	4 047	761	9
Ervália	6 188	6 182	5 369	813	6
Esmeraldas	21 831	21 801	15 626	6 175	30
Espera Feliz	7 592	7 585	6 526	1 059	7
Espinosa	9 907	9 896	8 086	1 810	11
Espírito Santo do Dourado	1 650	1 650	1 329	321	-
Estiva	3 709	3 700	3 248	452	9
Estrela Dalva	1 002	1 001	808	193	1
Estrela do Indaiá	1 795	1 794	1 214	580	1
Estrela do Sul	2 851	2 846	2 282	564	5
Eugenópolis	3 913	3 908	3 351	557	5

Tabela 2.1.17 - Domicílios recenseados, por espécie, segundo os municípios - Minas Gerais - 2007

(continuação)

Municípios	Domicílios recenseados, por espécie				
	Total	Particulares			Coletivos
		Total	Ocupados (1)	Não-ocupados	
Ewbank da Câmara	1 254	1 253	1 011	242	1
Extrema	10 511	10 500	7 765	2 735	11
Fama	1 213	1 204	762	442	9
Faria Lemos	1 416	1 415	1 165	250	1
Felício dos Santos	1 816	1 815	1 486	329	1
Felisburgo	2 237	2 236	1 845	391	1
Felixlândia	5 695	5 684	4 121	1 563	11
Fernandes Tourinho	945	942	822	120	3
Ferros	4 113	4 108	3 283	825	5
Fervedouro	3 530	3 526	2 974	552	4
Florestal	2 353	2 349	1 733	616	4
Formiga	24 776	24 753	20 439	4 314	23
Formoso	2 369	2 369	1 932	437	-
Fortaleza de Minas	1 595	1 593	1 251	342	2
Fortuna de Minas	924	924	701	223	-
Francisco Badaró	3 370	3 366	2 729	637	4
Francisco Dumont	1 668	1 664	1 345	319	4
Francisco Sá	7 827	7 815	6 450	1 365	12
Franciscópolis	1 917	1 917	1 529	388	-
Frei Gaspar	2 107	2 106	1 714	392	1
Frei Inocêncio	2 765	2 762	2 449	313	3
Frei Lagonegro	953	952	846	106	1
Fronteira	4 897	4 891	4 115	776	6
Fronteira dos Vales	1 560	1 559	1 338	221	1
Fruta de Leite	2 043	2 043	1 565	478	-
Frutal	17 257	17 234	16 236	998	23
Funilândia	1 669	1 666	1 090	576	3
Galiléia	2 571	2 564	2 222	342	7
Gameleiras	2 034	2 032	1 395	637	2
Glaucilândia	986	986	812	174	-
Goiabeira	1 137	1 135	969	166	2
Goianá	1 585	1 584	1 173	411	1
Gonçalves	2 021	2 002	1 313	689	19
Gonzaga	1 607	1 607	1 406	201	-
Gouveia	3 686	3 678	3 069	609	8
Grão Mogol	4 619	4 607	3 658	949	12
Grupiara	605	604	487	117	1
Guanhães	9 293	9 280	8 161	1 119	13
Guapé	5 076	5 063	4 022	1 041	13
Guaraciaba	3 335	3 333	2 866	467	2
Guaraciama	1 690	1 689	1 231	458	1
Guaranésia	6 414	6 406	5 499	907	8
Guarani	3 208	3 196	2 662	534	12
Guarará	1 625	1 624	1 222	402	1
Guarda-Mor	2 434	2 425	2 033	392	9
Guaxupé	16 570	16 548	14 607	1 941	22
Guidoval	2 650	2 649	2 265	384	1
Guimarânia	2 378	2 374	2 127	247	4
Guiricema	3 365	3 360	2 797	563	5
Gurinhatã	2 777	2 774	2 190	584	3
Heliodora	2 201	2 196	1 812	384	5
Iapu	3 551	3 540	3 192	348	11
Ibertioga	1 879	1 876	1 454	422	3
Ibiá	7 544	7 533	6 804	729	11
Ibiaí	2 237	2 233	1 981	252	4
Ibiracatu	2 043	2 043	1 498	545	-
Ibiraci	4 270	4 252	3 090	1 162	18

Tabela 2.1.17 - Domicílios recenseados, por espécie, segundo os municípios - Minas Gerais - 2007

(continuação)

Municípios	Domicílios recenseados, por espécie				
	Total	Particulares			Coletivos
		Total	Ocupados (1)	Não-ocupados	
Ibirité	44 639	44 633	41 448	3 185	6
Ibitiúra de Minas	1 399	1 396	1 138	258	3
Ibituruna	1 074	1 071	817	254	3
Icaraí de Minas	2 826	2 822	2 315	507	4
Igarapé	10 719	10 714	8 836	1 878	5
Igaratinga	2 950	2 947	2 517	430	3
Iguatama	3 199	3 194	2 478	716	5
Ijaci	1 895	1 891	1 573	318	4
Ilicínea	4 142	4 141	3 367	774	1
Imbé de Minas	2 165	2 164	1 799	365	1
Inconfidentes	2 624	2 600	2 238	362	24
Indaiabira	2 300	2 295	1 827	468	5
Indianópolis	2 281	2 256	1 839	417	25
Ingaí	818	816	728	88	2
Inhapim	8 719	8 711	7 259	1 452	8
Inhaúma	1 853	1 844	1 486	358	9
Inimutaba	2 398	2 397	1 922	475	1
Ipaba	4 396	4 394	3 918	476	2
Ipanema	6 269	6 257	5 457	800	12
Ipiaçu	1 664	1 662	1 399	263	2
Ipuiúna	3 144	3 141	2 803	338	3
Iraí de Minas	2 221	2 220	1 890	330	1
Itabira	32 758	32 709	28 995	3 714	49
Itabirinha	3 322	3 315	3 021	294	7
Itabirito	13 507	13 485	11 690	1 795	22
Itacambira	1 324	1 311	1 152	159	13
Itacarambi	5 020	5 007	4 219	788	13
Itaguara	4 789	4 784	3 778	1 006	5
Itaipé	3 627	3 620	2 844	776	7
Itajubá	29 805	29 777	25 301	4 476	28
Itamarandiba	9 750	9 739	8 268	1 471	11
Itamarati de Minas	1 516	1 514	1 260	254	2
Itambacuri	7 059	7 043	6 074	969	16
Itambé do Mato Dentro	856	846	694	152	10
Itamogi	4 099	4 094	3 314	780	5
Itamonte	4 740	4 719	3 987	732	21
Itanhandu	4 681	4 671	4 171	500	10
Itanhomi	4 101	4 098	3 601	497	3
Itaobim	6 783	6 763	5 818	945	20
Itapagipe	4 637	4 628	4 134	494	9
Itapecerica	8 306	8 292	6 396	1 896	14
Itapeva	3 278	3 274	2 414	860	4
Itatiaiuçu	3 982	3 981	2 619	1 362	1
Itaú de Minas	5 165	5 161	4 476	685	4
Itaúna	27 932	27 906	24 764	3 142	26
Itaverava	2 143	2 143	1 602	541	-
Itinga	4 454	4 447	3 697	750	7
Itueta	2 010	2 009	1 751	258	1
Ituiutaba	33 185	33 144	30 124	3 020	41
Itumirim	2 306	2 306	1 837	469	-
Iturama	11 029	11 013	9 791	1 222	16
Itutinga	1 586	1 580	1 256	324	6
Jaboticatubas	8 271	8 234	4 563	3 671	37
Jacinto	4 581	4 577	3 729	848	4
Jacuí	2 741	2 739	2 273	466	2
Jacutinga	8 067	8 061	6 152	1 909	6
Jaguaraçu	1 001	1 000	807	193	1

Tabela 2.1.17 - Domicílios recenseados, por espécie, segundo os municípios - Minas Gerais - 2007

(continuação)

Municípios	Domicílios recenseados, por espécie				
	Total	Particulares			Coletivos
		Total	Ocupados (1)	Não-ocupados	
Jaíba	9 278	9 255	7 714	1 541	23
Jampruca	1 749	1 746	1 371	375	3
Janaúba	19 342	19 304	16 979	2 325	38
Januária	19 414	19 390	16 084	3 306	24
Japaraíba	1 224	1 223	1 064	159	1
Japonvar	2 645	2 641	2 108	533	4
Jeceaba	2 204	2 202	1 734	468	2
Jenipapo de Minas	2 135	2 134	1 576	558	1
Jequeri	4 890	4 885	3 798	1 087	5
Jequitaí	2 791	2 788	2 241	547	3
Jequitibá	2 345	2 340	1 588	752	5
Jequitinhonha	7 879	7 870	6 719	1 151	9
Jesuânia	1 867	1 865	1 472	393	2
Joaíma	4 783	4 774	3 950	824	9
Joanésia	2 002	2 000	1 631	369	2
João Monlevade	22 394	22 342	20 734	1 608	52
João Pinheiro	15 278	15 245	12 576	2 669	33
Joaquim Felício	1 403	1 397	1 164	233	6
Jordânia	3 326	3 320	2 888	432	6
José Gonçalves de Minas	1 452	1 443	1 120	323	9
José Raydan	1 271	1 271	1 136	135	-
Josenópolis	1 699	1 698	1 123	575	1
Juatuba	7 481	7 476	5 643	1 833	5
Juramento	1 570	1 569	1 105	464	1
Juruaia	2 652	2 650	2 403	247	2
Juvenília	1 869	1 866	1 493	373	3
Ladainha	4 575	4 574	3 942	632	1
Lagamar	3 187	3 182	2 608	574	5
Lagoa da Prata	14 426	14 409	12 970	1 439	17
Lagoa dos Patos	1 327	1 326	1 153	173	1
Lagoa Dourada	3 733	3 728	3 053	675	5
Lagoa Formosa	6 182	6 178	5 239	939	4
Lagoa Grande	2 709	2 704	2 553	151	5
Lagoa Santa	17 227	17 215	12 841	4 374	12
Lajinha	6 254	6 248	5 240	1 008	6
Lambari	7 703	7 686	5 825	1 861	17
Lamim	1 315	1 313	984	329	2
Laranjal	2 473	2 470	1 997	473	3
Lassance	2 601	2 562	1 878	684	39
Lavras	31 159	31 020	26 321	4 699	139
Leandro Ferreira	1 147	1 144	901	243	3
Leme do Prado	1 633	1 630	1 271	359	3
Leopoldina	18 975	18 954	15 592	3 362	21
Liberdade	2 184	2 180	1 623	557	4
Lima Duarte	7 106	7 080	5 045	2 035	26
Limeira do Oeste	2 481	2 471	2 080	391	10
Lontra	2 725	2 722	2 067	655	3
Luisburgo	2 142	2 142	1 786	356	-
Luislândia	1 820	1 820	1 531	289	-
Luminárias	1 878	1 872	1 667	205	6
Luz	6 339	6 318	5 435	883	21
Machacalis	2 370	2 366	1 969	397	4
Machado	12 896	12 868	10 806	2 062	28
Madre de Deus de Minas	2 042	2 040	1 412	628	2
Malacacheta	5 808	5 798	4 824	974	10
Mamonas	2 383	2 380	1 835	545	3
Manga	5 940	5 929	5 075	854	11

Tabela 2.1.17 - Domicílios recenseados, por espécie, segundo os municípios - Minas Gerais - 2007

(continuação)

Municípios	Domicílios recenseados, por espécie				
	Total	Particulares			Coletivos
		Total	Ocupados (1)	Não-ocupados	
Manhuaçu	24 628	24 603	21 613	2 990	25
Manhumirim	7 016	7 009	6 156	853	7
Mantena	10 033	10 027	8 379	1 648	6
Mar de Espanha	4 415	4 407	3 553	854	8
Maravilhas	2 130	2 127	1 860	267	3
Maria da Fé	4 403	4 402	3 796	606	1
Mariana	16 704	16 488	14 476	2 012	216
Marilac	1 495	1 493	1 226	267	2
Mário Campos	3 728	3 724	3 120	604	4
Maripá de Minas	1 075	1 072	878	194	3
Marliéria	1 424	1 419	1 096	323	5
Marmelópolis	1 122	1 119	887	232	3
Martinho Campos	4 726	4 719	3 745	974	7
Martins Soares	2 073	2 071	1 848	223	2
Mata Verde	2 189	2 183	1 999	184	6
Materlândia	1 367	1 367	1 201	166	-
Mateus Leme	10 045	10 033	7 703	2 330	12
Mathias Lobato	1 074	1 073	957	116	1
Matias Barbosa	4 731	4 727	3 949	778	4
Matias Cardoso	3 162	3 148	2 376	772	14
Matipó	5 170	5 162	4 405	757	8
Mato Verde	4 736	4 730	3 769	961	6
Matozinhos	10 564	10 550	9 262	1 288	14
Matutina	1 702	1 702	1 330	372	-
Medeiros	1 274	1 272	1 049	223	2
Medina	6 856	6 843	5 683	1 160	13
Mendes Pimentel	2 198	2 198	1 962	236	-
Mercês	4 072	4 067	3 150	917	5
Mesquita	2 315	2 315	1 863	452	-
Minas Novas	8 873	8 860	7 076	1 784	13
Minduri	1 327	1 323	1 021	302	4
Mirabela	4 197	4 195	3 323	872	2
Miradouro	3 769	3 764	3 081	683	5
Miraí	4 724	4 719	3 889	830	5
Miravânia	1 332	1 329	1 160	169	3
Moeda	1 993	1 983	1 379	604	10
Moema	2 427	2 425	2 035	390	2
Monjolos	811	809	649	160	2
Monsenhor Paulo	2 645	2 644	2 211	433	1
Montalvânia	4 901	4 896	4 142	754	5
Monte Alegre de Minas	7 474	7 457	6 143	1 314	17
Monte Azul	8 237	8 226	6 621	1 605	11
Monte Belo	4 546	4 541	3 909	632	5
Monte Carmelo	16 401	16 356	14 151	2 205	45
Monte Formoso	1 395	1 393	1 106	287	2
Monte Santo de Minas	7 892	7 885	6 476	1 409	7
Monte Sião	7 630	7 617	5 859	1 758	13
Montezuma	2 275	2 272	1 731	541	3
Morada Nova de Minas	3 343	3 333	2 653	680	10
Morro da Garça	954	952	802	150	2
Morro do Pilar	1 324	1 324	1 008	316	-
Munhoz	2 441	2 438	1 836	602	3
Muriaé	34 835	34 802	29 760	5 042	33
Mutum	9 478	9 468	8 030	1 438	10
Muzambinho	7 492	7 482	6 262	1 220	10
Nacip Raydan	994	991	851	140	3
Nanuque	13 634	13 617	12 200	1 417	17

Tabela 2.1.17 - Domicílios recenseados, por espécie, segundo os municípios - Minas Gerais - 2007

(continuação)

Municípios	Domicílios recenseados, por espécie				
	Total	Particulares			Coletivos
		Total	Ocupados (1)	Não-ocupados	
Naque	1 838	1 835	1 619	216	3
Natalândia	1 112	1 110	985	125	2
Natércia	1 834	1 833	1 495	338	1
Nazareno	3 172	3 168	2 268	900	4
Nepomuceno	8 601	8 593	7 134	1 459	8
Ninheira	3 051	3 048	2 654	394	3
Nova Belém	1 347	1 346	1 077	269	1
Nova Era	5 537	5 521	4 984	537	16
Nova Lima	24 059	23 999	20 836	3 163	60
Nova Módica	1 373	1 370	1 122	248	3
Nova Ponte	4 055	4 026	3 429	597	29
Nova Porteirinha	2 272	2 272	1 927	345	-
Nova Resende	5 098	5 091	4 439	652	7
Nova Serrana	18 842	18 829	16 852	1 977	13
Nova União	2 319	2 314	1 608	706	5
Novo Cruzeiro	8 845	8 836	7 352	1 484	9
Novo Oriente de Minas	3 503	3 501	2 829	672	2
Novorizonte	1 801	1 801	1 335	466	-
Olaria	860	859	699	160	1
Olhos-d'Água	1 634	1 628	1 305	323	6
Olímpio Noronha	875	875	733	142	-
Oliveira	12 935	12 920	11 056	1 864	15
Oliveira Fortes	753	752	599	153	1
Onça de Pitangui	1 304	1 304	873	431	-
Oratórios	1 350	1 350	1 171	179	-
Orizânia	2 150	2 149	1 938	211	1
Ouro Branco	10 803	10 755	9 422	1 333	48
Ouro Fino	11 531	11 523	9 696	1 827	8
Ouro Preto	23 616	23 441	18 695	4 746	175
Ouro Verde de Minas	1 994	1 992	1 752	240	2
Padre Carvalho	1 744	1 742	1 316	426	2
Padre Paraíso	5 429	5 427	4 679	748	2
Pai Pedro	2 053	2 052	1 575	477	1
Paineiras	1 990	1 985	1 595	390	5
Pains	3 218	3 217	2 572	645	1
Paiva	610	608	508	100	2
Palma	2 418	2 412	1 965	447	6
Palmópolis	2 288	2 285	1 961	324	3
Papagaios	4 521	4 506	3 896	610	15
Pará de Minas	26 470	26 449	23 198	3 251	21
Paracatu	24 682	24 615	21 685	2 930	67
Paraguaçu	6 402	6 397	5 647	750	5
Paraisópolis	7 077	7 064	5 595	1 469	13
Paraopeba	7 298	7 288	6 362	926	10
Passa Quatro	5 244	5 228	4 421	807	16
Passa Tempo	3 420	3 414	2 588	826	6
Passabém	646	643	525	118	3
Passa-Vinte	1 042	1 039	678	361	3
Passos	34 746	34 722	31 223	3 499	24
Patis	1 562	1 560	1 288	272	2
Patos de Minas	46 688	46 644	42 047	4 597	44
Patrocínio	26 460	26 392	24 094	2 298	68
Patrocínio do Muriaé	2 181	2 177	1 737	440	4
Paula Cândido	3 214	3 212	2 558	654	2
Paulistas	1 533	1 530	1 255	275	3
Pavão	2 819	2 800	2 393	407	19
Peçanha	5 480	5 474	4 546	928	6

Tabela 2.1.17 - Domicílios recenseados, por espécie, segundo os municípios - Minas Gerais - 2007

(continuação)

Municípios	Domicílios recenseados, por espécie				
	Total	Particulares			Coletivos
		Total	Ocupados (1)	Não-ocupados	
Pedra Azul	7 549	7 538	6 295	1 243	11
Pedra Bonita	1 930	1 928	1 656	272	2
Pedra do Anta	1 422	1 421	1 123	298	1
Pedra do Indaiá	1 732	1 732	1 296	436	-
Pedra Dourada	791	789	640	149	2
Pedralva	3 914	3 909	3 170	739	5
Pedras de Maria da Cruz	3 365	3 361	2 681	680	4
Pedrinópolis	1 309	1 306	1 079	227	3
Pedro Leopoldo	18 369	18 354	16 114	2 240	15
Pedro Teixeira	691	690	520	170	1
Pequeri	1 218	1 210	935	275	8
Pequi	1 696	1 684	1 297	387	12
Perdigão	2 775	2 773	2 184	589	2
Perdizes	5 021	5 011	4 075	936	10
Perdões	6 568	6 563	5 895	668	5
Periquito	2 240	2 240	1 915	325	-
Pescador	1 345	1 343	1 095	248	2
Piau	1 217	1 216	972	244	1
Piedade de Caratinga	2 055	2 055	1 831	224	-
Piedade de Ponte Nova	1 192	1 190	1 100	90	2
Piedade do Rio Grande	2 086	2 085	1 507	578	1
Piedade dos Gerais	2 011	2 009	1 488	521	2
Pimenta	3 372	3 364	2 591	773	8
Pingo-d'Água	1 135	1 135	1 090	45	-
Pintópolis	2 196	2 191	1 863	328	5
Piracema	2 850	2 849	2 030	819	1
Pirajuba	1 223	1 223	1 118	105	-
Piranga	5 536	5 529	4 417	1 112	7
Piranguçu	2 030	2 025	1 523	502	5
Piranguinho	2 934	2 932	2 317	615	2
Pirapetinga	3 801	3 797	3 306	491	4
Pirapora	15 428	15 394	14 162	1 232	34
Piraúba	3 774	3 768	3 225	543	6
Pitangui	8 487	8 475	7 080	1 395	12
Piumhi	11 327	11 292	10 015	1 277	35
Planura	3 274	3 272	2 960	312	2
Poço Fundo	5 569	5 563	4 786	777	6
Poços de Caldas	53 859	53 773	46 317	7 456	86
Pocrane	3 517	3 513	2 833	680	4
Pompéu	9 366	9 348	7 997	1 351	18
Ponte Nova	18 676	18 658	16 426	2 232	18
Ponto Chique	1 318	1 313	1 076	237	5
Ponto dos Volantes	3 387	3 375	2 811	564	12
Porteirinha	11 917	11 908	10 101	1 807	9
Porto Firme	3 334	3 327	2 667	660	7
Poté	4 907	4 905	3 988	917	2
Pouso Alegre	40 780	40 697	36 235	4 462	83
Pouso Alto	2 570	2 561	1 953	608	9
Prados	3 114	3 101	2 415	686	13
Prata	8 896	8 883	7 809	1 074	13
Pratápolis	3 670	3 667	2 884	783	3
Pratinha	1 487	1 479	1 092	387	8
Presidente Bernardes	1 891	1 889	1 544	345	2
Presidente Juscelino	1 472	1 468	1 204	264	4
Presidente Kubitschek	833	830	704	126	3
Presidente Olegário	7 353	7 344	5 834	1 510	9
Prudente de Morais	2 899	2 895	2 401	494	4

Tabela 2.1.17 - Domicílios recenseados, por espécie, segundo os municípios - Minas Gerais - 2007

(continuação)

Municípios	Domicílios recenseados, por espécie				
	Total	Particulares			Coletivos
		Total	Ocupados (1)	Não-ocupados	
Quartel Geral	1 401	1 399	1 093	306	2
Queluzito	749	749	563	186	-
Raposos	4 475	4 469	4 084	385	6
Raul Soares	8 650	8 642	7 256	1 386	8
Recreio	4 100	4 095	3 243	852	5
Reduto	2 171	2 167	1 822	345	4
Resende Costa	4 352	4 349	3 262	1 087	3
Resplendor	6 689	6 682	5 565	1 117	7
Ressaquinha	1 684	1 680	1 362	318	4
Riachinho	2 584	2 578	2 191	387	6
Riacho dos Machados	2 565	2 556	2 228	328	9
Ribeirão Vermelho	1 308	1 305	1 134	171	3
Rio Acima	3 192	3 189	2 282	907	3
Rio Casca	4 814	4 804	4 110	694	10
Rio do Prado	1 535	1 534	1 266	268	1
Rio Doce	916	914	736	178	2
Rio Espera	2 640	2 634	1 884	750	6
Rio Manso	2 122	2 120	1 561	559	2
Rio Novo	3 233	3 228	2 694	534	5
Rio Paranaíba	3 857	3 847	3 517	330	10
Rio Pardo de Minas	8 236	8 232	7 144	1 088	4
Rio Piracicaba	4 733	4 725	4 098	627	8
Rio Pomba	6 191	6 183	5 221	962	8
Rio Preto	2 427	2 417	1 703	714	10
Rio Vermelho	4 519	4 512	3 782	730	7
Ritápolis	2 171	2 171	1 649	522	-
Rochedo de Minas	810	809	627	182	1
Rodeiro	2 119	2 116	1 841	275	3
Romaria	1 478	1 469	1 105	364	9
Rosário da Limeira	1 508	1 504	1 286	218	4
Rubelita	3 019	3 017	2 216	801	2
Rubim	3 424	3 419	2 887	532	5
Sabará	36 997	36 980	33 780	3 200	17
Sabinópolis	5 049	5 043	4 468	575	6
Sacramento	8 386	8 373	6 833	1 540	13
Salinas	13 012	12 995	10 805	2 190	17
Salto da Divisa	2 149	2 142	1 820	322	7
Santa Bárbara	8 132	8 067	6 994	1 073	65
Santa Bárbara do Leste	2 609	2 607	2 219	388	2
Santa Bárbara do Monte Verde	1 362	1 360	914	446	2
Santa Bárbara do Tugúrio	1 684	1 684	1 311	373	-
Santa Cruz de Minas	2 616	2 614	2 246	368	2
Santa Cruz de Salinas	1 652	1 650	1 444	206	2
Santa Cruz do Escalvado	1 961	1 960	1 509	451	1
Santa Efigênia de Minas	1 388	1 388	1 224	164	-
Santa Fé de Minas	1 287	1 283	1 086	197	4
Santa Helena de Minas	1 751	1 748	1 492	256	3
Santa Juliana	3 459	3 450	3 124	326	9
Santa Margarida	4 492	4 488	3 916	572	4
Santa Maria de Itabira	3 213	3 210	2 663	547	3
Santa Maria do Salto	1 892	1 891	1 548	343	1
Santa Maria do Suaçuí	4 635	4 626	3 909	717	9
Santa Rita de Caldas	3 435	3 430	2 956	474	5
Santa Rita de Ibitipoca	1 362	1 358	1 054	304	4
Santa Rita de Jacutinga	2 344	2 339	1 837	502	5
Santa Rita de Minas	1 969	1 966	1 685	281	3
Santa Rita do Itueto	2 327	2 321	1 739	582	6
Santa Rita do Sapucaí	11 466	11 448	9 803	1 645	18

Tabela 2.1.17 - Domicílios recenseados, por espécie, segundo os municípios - Minas Gerais - 2007

(continuação)

Municípios	Domicílios recenseados, por espécie				
	Total	Particulares			Coletivos
		Total	Ocupados (1)	Não-ocupados	
Santa Rosa da Serra	1 231	1 230	1 036	194	1
Santa Vitória	6 297	6 288	5 305	983	9
Santana da Vargem	2 451	2 449	2 053	396	2
Santana de Cataguases	1 450	1 448	1 175	273	2
Santana de Pirapama	3 181	3 176	2 504	672	5
Santana do Deserto	1 406	1 403	1 083	320	3
Santana do Garambéu	820	816	581	235	4
Santana do Jacaré	1 714	1 712	1 442	270	2
Santana do Manhuaçu	2 767	2 762	2 330	432	5
Santana do Paraíso	7 167	7 162	6 303	859	5
Santana do Riacho	2 034	1 990	1 248	742	44
Santana dos Montes	1 576	1 570	1 153	417	6
Santo Antônio do Amparo	5 275	5 260	4 647	613	15
Santo Antônio do Aventureiro	1 360	1 358	1 162	196	2
Santo Antônio do Grama	1 368	1 367	1 200	167	1
Santo Antônio do Itambé	1 292	1 290	1 091	199	2
Santo Antônio do Jacinto	3 712	3 709	3 236	473	3
Santo Antônio do Monte	9 346	9 333	7 884	1 449	13
Santo Antônio do Retiro	2 144	2 137	1 666	471	7
Santo Antônio do Rio Abaixo	545	544	489	55	1
Santo Hipólito	1 485	1 482	1 051	431	3
Santos Dumont	16 081	16 072	13 883	2 189	9
São Bento Abade	1 243	1 242	1 165	77	1
São Brás do Suaçuí	1 351	1 351	1 065	286	-
São Domingos das Dores	1 596	1 596	1 402	194	-
São Domingos do Prata	6 111	6 103	5 108	995	8
São Félix de Minas	1 061	1 059	958	101	2
São Francisco	14 512	14 495	12 369	2 126	17
São Francisco de Paula	2 273	2 270	1 767	503	3
São Francisco de Sales	1 963	1 963	1 736	227	-
São Francisco do Glória	1 890	1 889	1 623	266	1
São Geraldo	3 323	3 320	2 904	416	3
São Geraldo da Piedade	1 455	1 454	1 268	186	1
São Geraldo do Baixio	1 374	1 372	1 045	327	2
São Gonçalo do Abaeté	2 515	2 506	1 949	557	9
São Gonçalo do Pará	3 740	3 738	3 099	639	2
São Gonçalo do Rio Abaixo	2 817	2 813	2 378	435	4
São Gonçalo do Rio Preto	1 181	1 172	892	280	9
São Gonçalo do Sapucaí	7 839	7 828	6 541	1 287	11
São Gotardo	10 445	10 436	9 523	913	9
São João Batista do Glória	2 486	2 479	2 170	309	7
São João da Lagoa	1 838	1 837	1 373	464	1
São João da Mata	953	951	901	50	2
São João da Ponte	7 803	7 792	6 257	1 535	11
São João das Missões	2 572	2 570	2 235	335	2
São João del Rei	30 730	30 679	25 633	5 046	51
São João do Manhuaçu	3 045	3 044	2 652	392	1
São João do Manteninha	1 877	1 874	1 583	291	3
São João do Oriente	2 690	2 689	2 379	310	1
São João do Pacuí	1 154	1 154	1 048	106	-
São João do Paraíso	7 045	7 037	5 627	1 410	8
São João Evangelista	4 969	4 953	4 228	725	16
São João Nepomuceno	9 695	9 689	7 801	1 888	6
São Joaquim de Bicas	7 581	7 574	5 828	1 746	7
São José da Barra	2 421	2 411	1 957	454	10
São José da Lapa	5 854	5 852	4 934	918	2
São José da Safira	1 398	1 396	1 025	371	2
São José da Varginha	1 488	1 488	1 126	362	-

Tabela 2.1.17 - Domicílios recenseados, por espécie, segundo os municípios - Minas Gerais - 2007

(continuação)

Municípios	Domicílios recenseados, por espécie				
	Total	Particulares			Coletivos
		Total	Ocupados (1)	Não-ocupados	
São José do Alegre	1 437	1 437	1 171	266	-
São José do Divino	1 254	1 253	1 051	202	1
São José do Goiabal	1 935	1 933	1 610	323	2
São José do Jacuri	1 992	1 990	1 789	201	2
São José do Mantimento	835	835	729	106	-
São Lourenço	17 016	16 945	12 687	4 258	71
São Miguel do Anta	2 314	2 313	1 968	345	1
São Pedro da União	2 284	2 283	1 767	516	1
São Pedro do Suaçuí	1 815	1 814	1 594	220	1
São Pedro dos Ferros	2 966	2 959	2 532	427	7
São Romão	2 365	2 365	2 082	283	-
São Roque de Minas	2 436	2 411	2 000	411	25
São Sebastião da Bela Vista	2 069	2 068	1 471	597	1
São Sebastião da Vargem Alegre	935	925	854	71	10
São Sebastião do Anta	1 790	1 789	1 481	308	1
São Sebastião do Maranhão	3 370	3 366	2 943	423	4
São Sebastião do Oeste	2 213	2 213	1 660	553	-
São Sebastião do Paraíso	22 153	22 130	19 200	2 930	23
São Sebastião do Rio Preto	597	596	532	64	1
São Sebastião do Rio Verde	830	830	686	144	-
São Thomé das Letras	2 781	2 712	1 998	714	69
São Tiago	3 773	3 767	3 222	545	6
São Tomás de Aquino	2 587	2 581	2 120	461	6
São Vicente de Minas	2 353	2 328	1 752	576	25
Sapucaí-Mirim	2 249	2 243	1 711	532	6
Sardoá	1 429	1 427	1 325	102	2
Sarzedo	7 475	7 474	6 488	986	1
Sem-Peixe	1 065	1 063	909	154	2
Senador Amaral	1 676	1 676	1 465	211	-
Senador Cortes	646	645	581	64	1
Senador Firmino	2 617	2 610	2 122	488	7
Senador José Bento	787	787	632	155	-
Senador Modestino Gonçalves	1 567	1 565	1 263	302	2
Senhora de Oliveira	1 985	1 981	1 549	432	4
Senhora do Porto	1 230	1 228	986	242	2
Senhora dos Remédios	3 450	3 446	2 890	556	4
Sericita	2 249	2 247	1 926	321	2
Seritinga	643	643	511	132	-
Serra Azul de Minas	1 319	1 318	1 066	252	1
Serra da Saudade	367	367	290	77	-
Serra do Salitre	3 546	3 519	3 038	481	27
Serra dos Aimorés	2 876	2 872	2 521	351	4
Serrania	2 548	2 547	2 113	434	1
Serranópolis de Minas	1 550	1 550	1 233	317	-
Serranos	809	807	641	166	2
Serro	6 510	6 488	5 195	1 293	22
Setubinha	2 972	2 969	2 430	539	3
Silveirânia	802	801	667	134	1
Silvianópolis	2 476	2 473	1 921	552	3
Simão Pereira	1 169	1 167	751	416	2
Simonésia	5 552	5 542	4 860	682	10
Sobrália	2 078	2 077	1 787	290	1
Soledade de Minas	2 322	2 318	1 787	531	4
Tabuleiro	1 556	1 554	1 240	314	2
Taiobeiras	9 694	9 669	8 146	1 523	25
Taparuba	1 251	1 251	1 091	160	-
Tapira	1 297	1 297	1 078	219	-
Tapiraí	754	752	613	139	2

Tabela 2.1.17 - Domicílios recenseados, por espécie, segundo os municípios - Minas Gerais - 2007

(conclusão)

Municípios	Domicílios recenseados, por espécie				
	Total	Particulares			Coletivos
		Total	Ocupados (1)	Não-ocupados	
Taquaraçu de Minas	1 891	1 889	1 091	798	2
Tarumirim	5 490	5 485	4 382	1 103	5
Teixeiras	4 169	4 163	3 506	657	6
Teófilo Otoni	41 435	41 384	36 797	4 587	51
Timóteo	23 767	23 742	22 181	1 561	25
Tiradentes	2 953	2 857	2 094	763	96
Tiros	3 140	3 135	2 658	477	5
Tocantins	5 485	5 479	4 795	684	6
Tocos do Moji	1 379	1 378	1 177	201	1
Toledo	1 986	1 985	1 698	287	1
Tombos	3 557	3 552	2 962	590	5
Três Corações	22 363	22 331	20 194	2 137	32
Três Marias	9 214	9 167	7 661	1 506	47
Três Pontas	17 524	17 512	15 068	2 444	12
Tumiritinga	2 524	2 522	1 863	659	2
Tupaciguara	8 909	8 897	7 590	1 307	12
Turmalina	5 864	5 855	4 674	1 181	9
Turvolândia	1 654	1 653	1 446	207	1
Ubá	32 230	32 204	28 727	3 477	26
Ubaí	3 444	3 439	2 816	623	5
Ubaporanga	4 047	4 040	3 466	574	7
Umburatiba	851	851	766	85	-
Unaí	25 097	25 048	22 170	2 878	49
União de Minas	1 593	1 593	1 450	143	-
Uruana de Minas	1 130	1 130	847	283	-
Urucânia	3 038	3 034	2 664	370	4
Urucuia	3 022	3 017	2 660	357	5
Vargem Alegre	2 347	2 344	1 994	350	3
Vargem Bonita	950	948	777	171	2
Vargem Grande do Rio Pardo	1 471	1 468	1 090	378	3
Varginha	38 777	38 733	34 353	4 380	44
Varjão de Minas	2 053	2 050	1 758	292	3
Várzea da Palma	10 713	10 694	9 724	970	19
Varzelândia	5 479	5 476	4 513	963	3
Vazante	6 629	6 606	5 974	632	23
Verdelândia	2 433	2 433	1 981	452	-
Veredinha	2 254	2 249	1 518	731	5
Veríssimo	1 470	1 468	1 236	232	2
Vermelho Novo	1 463	1 457	1 340	117	6
Vespasiano	28 379	28 357	26 068	2 289	22
Viçosa	25 886	25 858	20 875	4 983	28
Vieiras	1 298	1 297	1 135	162	1
Virgem da Lapa	4 960	4 954	3 877	1 077	6
Virgínia	3 021	3 015	2 454	561	6
Virginópolis	3 244	3 239	2 823	416	5
Virgolândia	1 950	1 949	1 656	293	1
Visconde do Rio Branco	12 156	12 148	10 743	1 405	8
Volta Grande	1 783	1 779	1 480	299	4
Wenceslau Braz	889	887	748	139	2

Fonte: IBGE, Contagem da População 2007.

(1) Inclusive os domicílios fechados com população estimada.

Tabela 2.1.18 - Domicílios recenseados, por espécie, segundo os municípios - Espírito Santo - 2007

(continua)

Municípios	Domicílios recenseados, por espécie				
	Total	Particulares			Coletivos
		Total	Ocupados (1)	Não-ocupados	
**Espírito Santo**	**635 241**	**634 019**	**505 427**	**128 592**	**1 222**
Afonso Cláudio	10 952	10 933	9 314	1 619	19
Água Doce do Norte	4 230	4 230	3 680	550	-
Águia Branca	3 106	3 104	2 735	369	2
Alegre	11 720	11 705	9 444	2 261	15
Alfredo Chaves	5 394	5 387	4 354	1 033	7
Alto Rio Novo	2 106	2 104	1 839	265	2
Anchieta	9 575	9 503	5 530	3 973	72
Apiacá	2 932	2 930	2 370	560	2
Aracruz	24 410	24 359	20 422	3 937	51
Atilio Vivacqua	2 903	2 902	2 582	320	1
Baixo Guandu	10 412	10 406	8 786	1 620	6
Barra de São Francisco	13 927	13 913	12 224	1 689	14
Boa Esperança	4 265	4 257	3 762	495	8
Bom Jesus do Norte	3 609	3 606	3 018	588	3
Brejetuba	3 643	3 639	3 060	579	4
Castelo	11 321	11 314	9 882	1 432	7
Colatina	36 927	36 907	33 158	3 749	20
Conceição da Barra	10 921	10 845	7 380	3 465	76
Conceição do Castelo	4 078	4 072	3 345	727	6
Divino de São Lourenço	1 709	1 701	1 430	271	8
Domingos Martins	10 906	10 885	9 086	1 799	21
Dores do Rio Preto	2 236	2 233	1 830	403	3
Ecoporanga	8 479	8 472	7 382	1 090	7
Fundão	6 627	6 617	4 574	2 043	10
Governador Lindenberg	3 148	3 146	2 825	321	2
Guaçuí	9 477	9 470	7 731	1 739	7
Guarapari	58 586	58 403	30 408	27 995	183
Ibatiba	6 691	6 685	5 968	717	6
Ibiraçu	3 378	3 376	3 041	335	2
Ibitirama	3 054	3 053	2 553	500	1
Iconha	4 004	3 999	3 519	480	5
Irupi	3 441	3 436	3 115	321	5
Itaguaçu	5 074	5 069	4 266	803	5
Itapemirim	12 439	12 406	8 832	3 574	33
Itarana	3 561	3 558	3 104	454	3

Tabela 2.1.18 - Domicílios recenseados, por espécie, segundo os municípios - Espírito Santo - 2007

(conclusão)

Municípios	Domicílios recenseados, por espécie				
	Total	Particulares			Coletivos
		Total	Ocupados (1)	Não-ocupados	
Iúna	9 197	9 186	7 824	1 362	11
Jaguaré	6 836	6 826	6 264	562	10
Jerônimo Monteiro	3 713	3 707	3 264	443	6
João Neiva	4 663	4 663	4 319	344	-
Laranja da Terra	3 888	3 886	3 328	558	2
Linhares	41 488	41 459	35 531	5 928	29
Mantenópolis	4 510	4 505	3 562	943	5
Marataízes	17 761	17 562	9 370	8 192	199
Marechal Floriano	4 719	4 716	3 842	874	3
Marilândia	3 555	3 551	3 086	465	4
Mimoso do Sul	9 807	9 794	8 038	1 756	13
Montanha	6 037	6 026	5 463	563	11
Mucurici	2 208	2 206	1 762	444	2
Muniz Freire	6 478	6 477	5 308	1 169	1
Muqui	4 510	4 509	3 927	582	1
Nova Venécia	15 271	15 247	13 271	1 976	24
Pancas	6 910	6 901	5 553	1 348	9
Pedro Canário	8 038	8 021	6 675	1 346	17
Pinheiros	7 355	7 348	6 798	550	7
Piúma	12 499	12 458	4 943	7 515	41
Ponto Belo	2 260	2 260	2 088	172	-
Presidente Kennedy	3 603	3 598	3 021	577	5
Rio Bananal	5 283	5 276	4 817	459	7
Rio Novo do Sul	3 742	3 728	3 345	383	14
Santa Leopoldina	4 661	4 650	3 694	956	11
Santa Maria de Jetibá	10 057	10 049	8 834	1 215	8
Santa Teresa	7 441	7 424	6 145	1 279	17
São Domingos do Norte	3 020	3 013	2 342	671	7
São Gabriel da Palha	9 989	9 978	8 759	1 219	11
São José do Calçado	4 375	4 371	3 427	944	4
São Mateus	34 615	34 550	27 715	6 835	65
São Roque do Canaã	3 275	3 274	3 081	193	1
Sooretama	6 242	6 235	6 032	203	7
Vargem Alta	5 786	5 783	4 984	799	3
Venda Nova do Imigrante	6 140	6 135	5 401	734	5
Viana	18 550	18 534	16 420	2 114	16
Vila Pavão	3 092	3 082	2 674	408	10
Vila Valério	4 426	4 406	3 971	435	20

Fonte: IBGE, Contagem da População 2007.

(1) Inclusive os domicílios fechados com população estimada.

Tabela 2.1.19 - Domicílios recenseados, por espécie, segundo os municípios - Rio de Janeiro - 2007

(continua)

Municípios	Domicílios recenseados, por espécie				
	Total	Particulares			Coletivos
		Total	Ocupados (1)	Não-ocupados	
**Rio de Janeiro**	**1 406 578**	**1 403 838**	**1 038 282**	**365 556**	**2 740**
Angra dos Reis	63 970	63 798	45 822	17 976	172
Aperibé	3 490	3 487	2 892	595	3
Araruama	53 002	52 968	31 201	21 767	34
Areal	4 133	4 131	3 221	910	2
Armação dos Búzios	16 135	15 927	8 043	7 884	208
Arraial do Cabo	16 941	16 906	7 900	9 006	35
Barra do Piraí	32 850	32 819	30 393	2 426	31
Bom Jardim	8 841	8 828	7 738	1 090	13
Bom Jesus do Itabapoana	12 584	12 566	10 578	1 988	18
Cabo Frio	94 038	93 895	51 447	42 448	143
Cachoeiras de Macacu	20 048	20 031	16 645	3 386	17
Cambuci	5 738	5 735	4 739	996	3
Cantagalo	7 543	7 533	6 101	1 432	10
Carapebus	4 217	4 198	3 355	843	19
Cardoso Moreira	5 099	5 096	4 051	1 045	3
Carmo	6 216	6 183	5 201	982	33
Casimiro de Abreu	11 066	11 059	8 548	2 511	7
Comendador Levy Gasparian	2 841	2 841	2 463	378	-
Conceição de Macabu	7 108	7 097	5 929	1 168	11
Cordeiro	6 957	6 949	6 004	945	8
Duas Barras	4 095	4 089	3 265	824	6
Engenheiro Paulo de Frontin	5 271	5 255	4 158	1 097	16
Guapimirim	19 337	19 311	13 636	5 675	26
Iguaba Grande	14 791	14 784	6 385	8 399	7
Itaguaí	36 230	36 199	29 475	6 724	31
Italva	5 733	5 727	4 575	1 152	6
Itaocara	8 923	8 912	7 431	1 481	11
Itaperuna	35 646	35 608	29 853	5 755	38
Itatiaia	11 400	11 241	10 044	1 197	159
Japeri	30 598	30 589	27 914	2 675	9
Laje do Muriaé	2 897	2 896	2 409	487	1
Macaé	66 313	66 141	54 596	11 545	172
Macuco	1 797	1 795	1 617	178	2
Mangaratiba	27 949	27 905	9 205	18 700	44
Maricá	57 804	57 779	34 341	23 438	25
Mendes	6 930	6 927	5 647	1 280	3
Miguel Pereira	11 465	11 448	8 117	3 331	17
Miracema	9 271	9 263	7 780	1 483	8
Natividade	5 926	5 920	4 705	1 215	6
Nilópolis	54 572	54 552	48 892	5 660	20

Tabela 2.1.19 - Domicílios recenseados, por espécie, segundo os municípios - Rio de Janeiro - 2007

(conclusão)

Municípios	Domicílios recenseados, por espécie				
	Total	Particulares			Coletivos
		Total	Ocupados (1)	Não-ocupados	
Paracambi	15 140	15 080	13 406	1 674	60
Paraíba do Sul	14 169	14 151	11 940	2 211	18
Parati	13 285	13 026	9 789	3 237	259
Paty do Alferes	10 238	10 227	7 506	2 721	11
Pinheiral	7 170	7 164	6 449	715	6
Piraí	8 811	8 802	7 577	1 225	9
Porciúncula	6 255	6 252	5 243	1 009	3
Porto Real	4 553	4 550	4 148	402	3
Quatis	4 375	4 349	3 704	645	26
Queimados	43 340	43 328	39 024	4 304	12
Quissamã	6 510	6 478	5 105	1 373	32
Resende	41 033	40 964	37 302	3 662	69
Rio Bonito	17 700	17 684	16 153	1 531	16
Rio Claro	7 217	7 200	5 246	1 954	17
Rio das Flores	2 851	2 850	2 374	476	1
Rio das Ostras	41 102	41 040	24 133	16 907	62
Santa Maria Madalena	4 725	4 715	3 336	1 379	10
Santo Antônio de Pádua	14 913	14 897	12 863	2 034	16
São Fidélis	15 321	15 312	12 405	2 907	9
São Francisco de Itabapoana	23 135	23 097	14 229	8 868	38
São João da Barra	20 506	20 481	9 259	11 222	25
São José de Ubá	2 488	2 480	2 114	366	8
São José do Vale do Rio Preto	7 131	7 127	5 914	1 213	4
São Pedro da Aldeia	36 053	36 039	23 275	12 764	14
São Sebastião do Alto	3 315	3 312	2 667	645	3
Sapucaia	6 582	6 573	5 258	1 315	9
Saquarema	40 914	40 730	20 597	20 133	184
Seropédica	25 644	25 620	21 750	3 870	24
Silva Jardim	8 204	8 187	6 611	1 576	17
Sumidouro	5 735	5 729	4 767	962	6
Tanguá	9 980	9 971	8 875	1 096	9
Teresópolis	68 158	67 888	49 193	18 695	270
Trajano de Morais	3 920	3 907	2 994	913	13
Três Rios	25 053	25 036	22 041	2 995	17
Valença	25 178	25 124	22 263	2 861	54
Varre-Sai	2 956	2 950	2 439	511	6
Vassouras	13 153	13 130	10 017	3 113	23

Fonte: IBGE, Contagem da População 2007.

(1) Inclusive os domicílios fechados com população estimada.

Tabela 2.1.20 - Domicílios recenseados, por espécie, segundo os municípios - São Paulo - 2007

(continua)

Municípios	Domicílios recenseados, por espécie				
	Total	Particulares			Coletivos
		Total	Ocupados (1)	Não-ocupados	
**São Paulo**	**4 915 329**	**4 907 929**	**4 054 597**	**853 332**	**7 400**
Adamantina	11 929	11 918	10 766	1 152	11
Adolfo	1 380	1 379	1 153	226	1
Aguaí	10 120	10 111	9 068	1 043	9
Águas da Prata	3 196	3 182	2 334	848	14
Águas de Lindóia	7 331	7 301	4 994	2 307	30
Águas de Santa Bárbara	2 722	2 709	1 737	972	13
Águas de São Pedro	1 737	1 716	852	864	21
Agudos	10 784	10 774	9 979	795	10
Alambari	1 674	1 674	1 240	434	-
Alfredo Marcondes	1 410	1 410	1 250	160	-
Altair	1 141	1 139	992	147	2
Altinópolis	5 000	4 989	4 534	455	11
Alto Alegre	1 608	1 608	1 349	259	-
Alumínio	5 116	5 106	4 494	612	10
Álvares Florence	1 652	1 652	1 366	286	-
Álvares Machado	7 812	7 803	7 009	794	9
Álvaro de Carvalho	1 027	1 026	949	77	1
Alvinlândia	960	959	834	125	1
Américo Brasiliense	9 444	9 400	8 820	580	44
Américo de Campos	2 234	2 234	1 868	366	-
Amparo	21 925	21 906	19 204	2 702	19
Analândia	1 863	1 862	1 291	571	1
Andradina	19 316	19 215	17 454	1 761	101
Angatuba	8 096	8 091	6 679	1 412	5
Anhembi	2 294	2 294	1 625	669	-
Anhumas	1 292	1 291	1 119	172	1
Aparecida	11 520	11 360	10 330	1 030	160
Aparecida d'Oeste	1 827	1 823	1 546	277	4
Apiaí	8 700	8 686	7 258	1 428	14
Araçariguama	4 250	4 247	3 338	909	3
Araçoiaba da Serra	12 054	12 040	7 253	4 787	14
Aramina	1 661	1 659	1 548	111	2
Arandu	2 446	2 442	1 778	664	4
Arapeí	924	921	768	153	3
Araras	37 547	37 511	31 792	5 719	36
Arco-Íris	830	829	628	201	1
Arealva	3 517	3 516	2 555	961	1
Areias	1 260	1 255	972	283	5
Areiópolis	3 413	3 413	2 941	472	-
Ariranha	2 855	2 854	2 574	280	1
Artur Nogueira	12 929	12 926	11 739	1 187	3
Arujá	23 414	23 400	20 681	2 719	14
Aspásia	664	664	570	94	-
Assis	31 973	31 937	29 650	2 287	36
Atibaia	44 899	44 852	35 877	8 975	47
Auriflama	4 735	4 726	4 407	319	9
Avaí	1 943	1 940	1 467	473	3
Avanhandava	3 310	3 303	2 878	425	7
Avaré	28 469	28 414	24 794	3 620	55
Bady Bassitt	4 489	4 489	4 056	433	-
Balbinos	581	577	460	117	4
Bálsamo	2 866	2 866	2 573	293	-
Bananal	3 908	3 901	3 131	770	7
Barão de Antonina	1 219	1 219	926	293	-
Barbosa	2 348	2 347	1 973	374	1

Tabela 2.1.20 - Domicílios recenseados, por espécie, segundo os municípios - São Paulo - 2007

(continuação)

Municípios	Domicílios recenseados, por espécie				
	Total	Particulares			Coletivos
		Total	Ocupados (1)	Não-ocupados	
Bariri	10 356	10 346	9 247	1 099	10
Barra Bonita	11 858	11 846	10 784	1 062	12
Barra do Chapéu	1 653	1 653	1 441	212	-
Barra do Turvo	3 191	3 181	2 284	897	10
Barretos	38 178	38 115	34 110	4 005	63
Barrinha	8 056	8 052	7 189	863	4
Bastos	6 964	6 960	6 199	761	4
Batatais	18 436	18 413	16 531	1 882	23
Bebedouro	25 263	25 240	22 844	2 396	23
Bento de Abreu	964	964	827	137	-
Bernardino de Campos	3 613	3 609	3 262	347	4
Bertioga	35 103	35 024	11 792	23 232	79
Bilac	2 476	2 472	2 207	265	4
Birigui	36 284	36 264	32 792	3 472	20
Biritiba-Mirim	10 040	10 038	7 837	2 201	2
Boa Esperança do Sul	4 437	4 435	3 913	522	2
Bocaina	3 520	3 515	3 079	436	5
Bofete	3 516	3 513	2 659	854	3
Boituva	15 529	15 514	12 010	3 504	15
Bom Jesus dos Perdões	6 394	6 391	4 836	1 555	3
Bom Sucesso de Itararé	1 161	1 160	940	220	1
Borá	288	287	245	42	1
Boracéia	1 373	1 373	1 174	199	-
Borborema	5 332	5 326	4 385	941	6
Borebi	683	682	629	53	1
Botucatu	47 606	47 516	38 673	8 843	90
Bragança Paulista	47 497	47 451	41 000	6 451	46
Braúna	1 692	1 692	1 464	228	-
Brejo Alegre	871	871	755	116	-
Brodowski	6 349	6 344	5 648	696	5
Brotas	8 468	8 446	6 520	1 926	22
Buri	5 951	5 945	4 981	964	6
Buritama	5 910	5 904	4 811	1 093	6
Buritizal	1 464	1 463	1 256	207	1
Cabrália Paulista	1 551	1 548	1 317	231	3
Cabreúva	14 027	13 997	11 048	2 949	30
Caçapava	25 965	25 932	22 960	2 972	33
Cachoeira Paulista	10 188	10 160	8 883	1 277	28
Caconde	6 966	6 951	5 526	1 425	15
Cafelândia	6 105	6 100	5 072	1 028	5
Caiabu	1 591	1 591	1 309	282	-
Caieiras	27 074	27 070	24 005	3 065	4
Caiuá	1 803	1 802	1 592	210	1
Cajamar	18 869	18 862	17 110	1 752	7
Cajati	9 975	9 969	7 982	1 987	6
Cajobi	3 315	3 313	2 916	397	2
Cajuru	7 559	7 551	6 746	805	8
Campina do Monte Alegre	2 383	2 379	1 652	727	4
Campo Limpo Paulista	23 933	23 918	20 480	3 438	15
Campos do Jordão	20 576	20 432	12 860	7 572	144
Campos Novos Paulista	1 686	1 684	1 473	211	2
Cananéia	5 167	5 123	3 399	1 724	44
Canas	1 299	1 299	1 194	105	-
Cândido Mota	9 909	9 905	9 073	832	4
Cândido Rodrigues	949	949	861	88	-
Canitar	1 340	1 340	1 148	192	-

Tabela 2.1.20 - Domicílios recenseados, por espécie, segundo os municípios - São Paulo - 2007

(continuação)

Municípios	Domicílios recenseados, por espécie				
	Total	Particulares			Coletivos
		Total	Ocupados (1)	Não-ocupados	
Capão Bonito	14 387	14 362	12 680	1 682	25
Capela do Alto	6 141	6 138	4 487	1 651	3
Capivari	14 032	14 021	12 718	1 303	11
Caraguatatuba	59 007	58 904	27 772	31 132	103
Cardoso	5 041	5 032	3 897	1 135	9
Casa Branca	9 052	8 971	7 646	1 325	81
Cássia dos Coqueiros	1 049	1 047	848	199	2
Castilho	5 588	5 585	4 738	847	3
Catanduva	39 643	39 608	35 437	4 171	35
Catiguá	2 207	2 199	2 036	163	8
Cedral	3 063	3 055	2 477	578	8
Cerqueira César	6 176	6 169	5 213	956	7
Cerquilho	11 774	11 763	10 393	1 370	11
Cesário Lange	5 312	5 310	4 407	903	2
Charqueada	4 939	4 935	4 117	818	4
Chavantes	4 179	4 175	3 628	547	4
Clementina	2 069	2 068	1 819	249	1
Colina	5 692	5 684	5 064	620	8
Colômbia	2 083	2 080	1 733	347	3
Conchal	7 372	7 368	6 538	830	4
Conchas	5 654	5 646	4 624	1 022	8
Cordeirópolis	6 314	6 309	5 686	623	5
Coroados	1 920	1 918	1 573	345	2
Coronel Macedo	1 917	1 917	1 669	248	-
Corumbataí	1 639	1 636	1 178	458	3
Cosmópolis	17 810	17 795	16 011	1 784	15
Cosmorama	2 957	2 955	2 337	618	2
Cravinhos	9 668	9 657	8 434	1 223	11
Cristais Paulista	2 528	2 522	2 071	451	6
Cruzália	863	863	777	86	-
Cruzeiro	24 080	24 053	21 854	2 199	27
Cubatão	39 954	39 927	36 587	3 340	27
Cunha	8 946	8 929	7 101	1 828	17
Descalvado	10 034	10 023	8 792	1 231	11
Dirce Reis	627	627	552	75	-
Divinolândia	4 116	4 112	3 496	616	4
Dobrada	2 515	2 515	2 229	286	-
Dois Córregos	8 639	8 629	7 377	1 252	10
Dolcinópolis	839	839	749	90	-
Dourado	3 298	3 295	2 758	537	3
Dracena	15 302	15 273	13 588	1 685	29
Duartina	4 734	4 727	3 967	760	7
Dumont	2 298	2 295	2 136	159	3
Echaporã	2 477	2 476	1 932	544	1
Eldorado	5 101	5 085	3 896	1 189	16
Elias Fausto	4 770	4 770	4 169	601	-
Elisiário	974	974	934	40	-
Embaúba	843	843	752	91	-
Embu-Guaçu	20 832	20 814	16 867	3 947	18
Emilianópolis	1 139	1 139	995	144	-
Engenheiro Coelho	4 111	4 106	3 532	574	5
Espírito Santo do Pinhal	13 923	13 906	11 996	1 910	17
Espírito Santo do Turvo	1 336	1 335	1 189	146	1
Estiva Gerbi	2 992	2 986	2 591	395	6
Estrela do Norte	910	910	722	188	-
Estrela d'Oeste	2 865	2 836	2 661	175	29

Tabela 2.1.20 - Domicílios recenseados, por espécie, segundo os municípios - São Paulo - 2007

(continuação)

Municípios	Domicílios recenseados, por espécie				
	Total	Particulares			Coletivos
		Total	Ocupados (1)	Não-ocupados	
Euclides da Cunha Paulista	3 284	3 283	2 986	297	1
Fartura	5 328	5 322	4 393	929	6
Fernando Prestes	2 046	2 044	1 699	345	2
Fernandópolis	22 865	22 843	20 000	2 843	22
Fernão	582	582	454	128	-
Flora Rica	735	735	666	69	-
Floreal	1 179	1 178	1 012	166	1
Flórida Paulista	4 233	4 228	3 672	556	5
Florínia	1 257	1 257	931	326	-
Francisco Morato	43 642	43 632	40 636	2 996	10
Franco da Rocha	38 756	38 716	33 620	5 096	40
Gabriel Monteiro	948	942	858	84	6
Gália	2 682	2 681	2 200	481	1
Garça	15 191	15 165	12 969	2 196	26
Gastão Vidigal	1 458	1 456	1 121	335	2
Gavião Peixoto	1 152	1 152	1 106	46	-
General Salgado	3 991	3 981	3 446	535	10
Getulina	3 312	3 303	2 841	462	9
Glicério	1 777	1 776	1 335	441	1
Guaiçara	3 617	3 613	3 033	580	4
Guaimbê	1 635	1 635	1 455	180	-
Guaíra	12 706	12 697	11 443	1 254	9
Guapiaçu	5 932	5 931	5 155	776	1
Guapiara	6 388	6 383	5 689	694	5
Guará	5 930	5 920	5 477	443	10
Guaraçaí	3 431	3 428	2 808	620	3
Guaraci	3 734	3 732	2 783	949	2
Guarani d'Oeste	744	743	680	63	1
Guarantã	2 247	2 246	1 869	377	1
Guararapes	10 191	10 150	8 970	1 180	41
Guararema	10 075	10 060	7 382	2 678	15
Guaratinguetá	36 697	36 598	32 067	4 531	99
Guareí	4 338	4 333	3 498	835	5
Guariba	10 828	10 823	9 578	1 245	5
Guatapará	2 323	2 322	1 760	562	1
Guzolândia	1 610	1 607	1 402	205	3
Herculândia	3 068	3 065	2 715	350	3
Holambra	2 983	2 978	2 615	363	5
Iacanga	3 390	3 354	2 820	534	36
Iacri	2 515	2 515	2 049	466	-
Iaras	1 381	1 370	1 119	251	11
Ibaté	8 805	8 803	7 823	980	2
Ibirá	4 093	4 077	3 354	723	16
Ibirarema	2 448	2 446	2 093	353	2
Ibitinga	18 271	18 255	15 620	2 635	16
Ibiúna	32 870	32 849	19 208	13 641	21
Icém	2 456	2 453	1 822	631	3
Iepê	2 633	2 629	2 384	245	4
Igaraçu do Tietê	7 442	7 441	6 759	682	1
Igarapava	9 410	9 404	8 315	1 089	6
Igaratá	4 212	4 210	2 617	1 593	2
Iguape	12 926	12 894	8 647	4 247	32
Ilha Comprida	8 588	8 429	2 812	5 617	159
Ilha Solteira	8 845	8 836	7 320	1 516	9
Ilhabela	11 864	11 710	7 272	4 438	154
Indiana	1 718	1 717	1 496	221	1

Tabela 2.1.20 - Domicílios recenseados, por espécie, segundo os municípios - São Paulo - 2007

(continuação)

Municípios	Domicílios recenseados, por espécie				
	Total	Particulares			Coletivos
		Total	Ocupados (1)	Não-ocupados	
Indiaporã	1 804	1 802	1 403	399	2
Inúbia Paulista	1 271	1 266	1 118	148	5
Ipaussu	4 414	4 410	3 900	510	4
Iperó	9 218	9 214	6 857	2 357	4
Ipeúna	2 293	2 293	1 544	749	-
Ipiguá	1 488	1 488	1 297	191	-
Iporanga	1 775	1 769	1 263	506	6
Ipuã	4 297	4 240	4 017	223	57
Iracemápolis	5 782	5 778	5 286	492	4
Irapuã	2 451	2 450	2 069	381	1
Irapuru	2 503	2 500	2 253	247	3
Itaberá	5 963	5 956	5 295	661	7
Itaí	8 198	8 184	6 575	1 609	14
Itajobi	4 841	4 837	4 514	323	4
Itaju	1 304	1 304	806	498	-
Itanhaém	63 084	63 001	25 736	37 265	83
Itaóca	1 043	1 042	906	136	1
Itapecerica da Serra	48 541	48 494	42 812	5 682	47
Itapetininga	45 893	45 854	40 193	5 661	39
Itapeva	27 362	27 339	24 789	2 550	23
Itapira	24 126	24 082	20 689	3 393	44
Itapirapuã Paulista	1 329	1 328	1 013	315	1
Itápolis	14 130	14 121	12 377	1 744	9
Itaporanga	5 187	5 174	4 440	734	13
Itapuí	4 034	4 028	3 435	593	6
Itapura	1 573	1 573	1 176	397	-
Itararé	16 276	16 255	14 659	1 596	21
Itariri	6 124	6 119	4 394	1 725	5
Itatiba	33 725	33 710	27 555	6 155	15
Itatinga	5 855	5 843	4 944	899	12
Itirapina	5 831	5 815	3 510	2 305	16
Itirapuã	1 951	1 945	1 615	330	6
Itobi	2 605	2 595	2 174	421	10
Itu	52 063	51 991	44 009	7 982	72
Itupeva	14 027	14 012	10 792	3 220	15
Ituverava	13 638	13 628	12 097	1 531	10
Jaborandi	2 293	2 290	1 962	328	3
Jaboticabal	24 656	24 618	21 460	3 158	38
Jaci	1 662	1 658	1 503	155	4
Jacupiranga	6 144	6 132	4 724	1 408	12
Jaguariúna	12 796	12 789	10 685	2 104	7
Jales	17 718	17 694	15 815	1 879	24
Jambeiro	2 197	2 194	1 518	676	3
Jandira	32 635	32 628	30 402	2 226	7
Jardinópolis	12 104	12 093	10 085	2 008	11
Jarinu	9 135	9 127	6 133	2 994	8
Jaú	43 642	43 620	38 258	5 362	22
Jeriquara	1 008	1 008	927	81	-
Joanópolis	4 133	4 131	3 467	664	2
João Ramalho	1 216	1 216	1 185	31	-
José Bonifácio	10 349	10 342	9 442	900	7
Júlio Mesquita	1 416	1 416	1 265	151	-
Jumirim	765	763	635	128	2
Junqueirópolis	6 398	6 393	5 530	863	5
Juquiá	7 090	7 078	5 667	1 411	12
Juquitiba	12 170	12 154	8 272	3 882	16

Tabela 2.1.20 - Domicílios recenseados, por espécie, segundo os municípios - São Paulo - 2007

(continuação)

Municípios	Domicílios recenseados, por espécie				
	Total	Particulares			Coletivos
		Total	Ocupados (1)	Não-ocupados	
Lagoinha	2 170	2 168	1 577	591	2
Laranjal Paulista	8 455	8 445	7 399	1 046	10
Lavínia	2 282	2 267	1 597	670	15
Lavrinhas	2 228	2 224	1 808	416	4
Leme	27 994	27 976	24 665	3 311	18
Lençóis Paulista	19 203	19 193	17 231	1 962	10
Lindóia	2 508	2 508	1 807	701	-
Lins	24 300	24 270	21 818	2 452	30
Lorena	26 390	26 371	23 294	3 077	19
Lourdes	681	680	626	54	1
Louveira	9 926	9 918	8 554	1 364	8
Lucélia	6 480	6 475	5 721	754	5
Lucianópolis	854	853	731	122	1
Luís Antônio	3 021	2 960	2 671	289	61
Luiziânia	1 645	1 643	1 452	191	2
Lupércio	1 361	1 360	1 263	97	1
Lutécia	1 078	1 077	938	139	1
Macatuba	4 844	4 842	4 501	341	2
Macaubal	2 822	2 817	2 505	312	5
Macedônia	1 336	1 336	1 107	229	-
Magda	1 311	1 310	1 100	210	1
Mairinque	16 649	16 644	12 029	4 615	5
Mairiporã	26 747	26 711	20 813	5 898	36
Manduri	3 217	3 209	2 681	528	8
Marabá Paulista	1 560	1 559	1 382	177	1
Maracaí	4 504	4 501	4 137	364	3
Marapoama	847	847	816	31	-
Mariápolis	1 592	1 592	1 236	356	-
Marinópolis	765	765	669	96	-
Martinópolis	8 868	8 864	7 323	1 541	4
Matão	23 701	23 681	22 247	1 434	20
Mendonça	1 529	1 529	1 308	221	-
Meridiano	1 457	1 455	1 240	215	2
Mesópolis	812	812	616	196	-
Miguelópolis	7 426	7 415	6 131	1 284	11
Mineiros do Tietê	4 372	4 369	3 486	883	3
Mira Estrela	1 215	1 215	919	296	-
Miracatu	9 267	9 256	6 650	2 606	11
Mirandópolis	9 821	9 807	7 849	1 958	14
Mirante do Paranapanema	6 114	6 111	5 451	660	3
Mirassol	18 040	18 027	16 879	1 148	13
Mirassolândia	1 565	1 565	1 338	227	-
Mococa	22 895	22 869	19 559	3 310	26
Mogi Guaçu	43 708	43 670	39 322	4 348	38
Moji Mirim	29 212	29 182	26 012	3 170	30
Mombuca	1 067	1 051	917	134	16
Monções	820	820	742	78	-
Mongaguá	38 856	38 789	12 199	26 590	67
Monte Alegre do Sul	3 275	3 260	2 216	1 044	15
Monte Alto	15 120	15 111	13 726	1 385	9
Monte Aprazível	7 629	7 620	6 509	1 111	9
Monte Azul Paulista	6 539	6 534	5 804	730	5
Monte Castelo	1 580	1 580	1 344	236	-
Monte Mor	13 949	13 941	12 504	1 437	8
Monteiro Lobato	1 818	1 817	1 224	593	1
Morro Agudo	8 648	8 646	7 202	1 444	2

Tabela 2.1.20 - Domicílios recenseados, por espécie, segundo os municípios - São Paulo - 2007

(continuação)

Municípios	Domicílios recenseados, por espécie				
	Total	Particulares			Coletivos
		Total	Ocupados (1)	Não-ocupados	
Morungaba	4 086	4 083	3 500	583	3
Motuca	1 239	1 235	1 192	43	4
Murutinga do Sul	1 477	1 476	1 380	96	1
Nantes	849	849	715	134	-
Narandiba	1 261	1 261	1 168	93	-
Natividade da Serra	3 586	3 574	2 398	1 176	12
Nazaré Paulista	7 081	7 060	4 549	2 511	21
Neves Paulista	3 423	3 419	2 912	507	4
Nhandeara	3 835	3 828	3 506	322	7
Nipoã	1 390	1 381	1 199	182	9
Nova Aliança	1 837	1 837	1 615	222	-
Nova Campina	2 496	2 496	2 258	238	-
Nova Canaã Paulista	965	965	746	219	-
Nova Castilho	446	446	347	99	-
Nova Europa	2 877	2 862	2 673	189	15
Nova Granada	6 608	6 596	5 523	1 073	12
Nova Guataporanga	844	844	725	119	-
Nova Independência	915	915	773	142	-
Nova Luzitânia	1 190	1 181	1 056	125	9
Nova Odessa	15 257	15 239	13 919	1 320	18
Novais	1 330	1 330	1 069	261	-
Novo Horizonte	13 188	13 180	11 106	2 074	8
Nuporanga	2 208	2 206	1 987	219	2
Ocauçu	1 721	1 720	1 312	408	1
Óleo	1 331	1 331	885	446	-
Olímpia	16 654	16 631	15 085	1 546	23
Onda Verde	1 272	1 267	1 112	155	5
Oriente	2 188	2 186	1 816	370	2
Orindiúva	1 555	1 553	1 471	82	2
Orlândia	11 658	11 649	10 553	1 096	9
Oscar Bressane	929	927	833	94	2
Osvaldo Cruz	10 771	10 761	9 545	1 216	10
Ourinhos	33 675	33 651	30 709	2 942	24
Ouro Verde	2 676	2 675	2 461	214	1
Ouroeste	2 505	2 505	2 280	225	-
Pacaembu	3 822	3 817	3 560	257	5
Palestina	3 846	3 829	3 352	477	17
Palmares Paulista	3 209	3 207	2 904	303	2
Palmeira d'Oeste	3 830	3 828	3 268	560	2
Palmital	7 723	7 719	6 940	779	4
Panorama	5 132	5 120	4 299	821	12
Paraguaçu Paulista	13 592	13 569	12 311	1 258	23
Paraibuna	6 698	6 685	4 871	1 814	13
Paraíso	1 853	1 853	1 702	151	-
Paranapanema	5 946	5 935	4 969	966	11
Paranapuã	1 364	1 363	1 237	126	1
Parapuã	4 269	4 266	3 604	662	3
Pardinho	1 806	1 804	1 475	329	2
Pariquera-Açu	6 210	6 200	5 197	1 003	10
Parisi	815	815	692	123	-
Patrocínio Paulista	4 422	4 417	3 572	845	5
Paulicéia	2 380	2 364	1 712	652	16
Paulínia	23 116	23 090	21 112	1 978	26
Paulistânia	705	705	572	133	-
Paulo de Faria	3 206	3 199	2 824	375	7
Pederneiras	13 090	13 085	11 869	1 216	5

Tabela 2.1.20 - Domicílios recenseados, por espécie, segundo os municípios - São Paulo - 2007

(continuação)

Municípios	Domicílios recenseados, por espécie				
	Total	Particulares			Coletivos
		Total	Ocupados (1)	Não-ocupados	
Pedra Bela	2 413	2 410	1 762	648	3
Pedranópolis	1 111	1 110	913	197	1
Pedregulho	4 866	4 862	4 423	439	4
Pedreira	12 546	12 535	11 437	1 098	11
Pedrinhas Paulista	1 030	1 029	869	160	1
Pedro de Toledo	4 524	4 522	2 945	1 577	2
Penápolis	20 128	20 111	18 033	2 078	17
Pereira Barreto	8 808	8 792	7 528	1 264	16
Pereiras	2 888	2 884	2 262	622	4
Peruíbe	37 766	37 685	17 132	20 553	81
Piacatu	1 733	1 730	1 540	190	3
Piedade	18 057	18 051	14 000	4 051	6
Pilar do Sul	9 281	9 275	7 458	1 817	6
Pindamonhangaba	43 702	43 657	38 780	4 877	45
Pindorama	5 059	5 053	4 461	592	6
Pinhalzinho	6 059	6 049	3 833	2 216	10
Piquerobi	1 355	1 354	1 169	185	1
Piquete	4 973	4 960	4 382	578	13
Piracaia	8 531	8 523	6 732	1 791	8
Piraju	10 508	10 496	8 757	1 739	12
Pirajuí	7 006	6 993	5 962	1 031	13
Pirangi	3 351	3 347	3 146	201	4
Pirapora do Bom Jesus	4 760	4 749	3 975	774	11
Pirapozinho	8 137	8 130	7 230	900	7
Pirassununga	24 548	24 511	21 097	3 414	37
Piratininga	4 263	4 255	3 405	850	8
Pitangueiras	10 991	10 917	9 714	1 203	74
Planalto	1 489	1 488	1 196	292	1
Platina	1 174	1 173	1 007	166	1
Poá	32 298	32 284	29 954	2 330	14
Poloni	1 832	1 830	1 677	153	2
Pompéia	6 349	6 343	5 869	474	6
Pongaí	1 355	1 355	1 150	205	-
Pontal	11 297	11 291	10 123	1 168	6
Pontalinda	1 332	1 331	1 223	108	1
Pontes Gestal	1 012	1 012	813	199	-
Populina	1 768	1 765	1 447	318	3
Porangaba	4 214	4 214	2 644	1 570	-
Porto Feliz	15 862	15 844	13 688	2 156	18
Porto Ferreira	16 293	16 277	14 679	1 598	16
Potim	4 498	4 481	4 040	441	17
Potirendaba	5 076	5 070	4 572	498	6
Pracinha	585	583	485	98	2
Pradópolis	4 897	4 895	4 236	659	2
Pratânia	1 493	1 493	1 255	238	-
Presidente Alves	1 629	1 628	1 355	273	1
Presidente Bernardes	5 196	5 191	4 422	769	5
Presidente Epitácio	14 145	14 111	12 139	1 972	34
Presidente Venceslau	13 159	13 144	11 807	1 337	15
Promissão	11 562	11 554	10 423	1 131	8
Quadra	1 205	1 203	858	345	2
Quatá	3 982	3 977	3 531	446	5
Queiroz	909	909	790	119	-
Queluz	3 200	3 195	2 901	294	5
Quintana	2 036	2 031	1 791	240	5
Rafard	2 545	2 540	2 298	242	5

Tabela 2.1.20 - Domicílios recenseados, por espécie, segundo os municípios - São Paulo - 2007

(continuação)

Municípios	Domicílios recenseados, por espécie				
	Total	Particulares			Coletivos
		Total	Ocupados (1)	Não-ocupados	
Rancharia	10 763	10 753	9 060	1 693	10
Redenção da Serra	2 075	2 069	1 296	773	6
Regente Feijó	5 789	5 782	5 275	507	7
Reginópolis	1 839	1 836	1 578	258	3
Registro	17 803	17 773	15 320	2 453	30
Restinga	2 019	2 018	1 770	248	1
Ribeira	1 287	1 286	1 044	242	1
Ribeirão Bonito	3 888	3 884	3 421	463	4
Ribeirão Branco	6 376	6 368	5 164	1 204	8
Ribeirão Corrente	1 476	1 476	1 185	291	-
Ribeirão do Sul	1 573	1 573	1 380	193	-
Ribeirão dos Índios	785	785	728	57	-
Ribeirão Grande	2 463	2 461	1 951	510	2
Ribeirão Pires	34 946	34 929	31 732	3 197	17
Rifaina	1 756	1 748	1 116	632	8
Rincão	3 435	3 433	2 858	575	2
Rinópolis	3 426	3 423	2 948	475	3
Rio das Pedras	8 285	8 278	7 575	703	7
Rio Grande da Serra	12 694	12 682	11 412	1 270	12
Riolândia	2 912	2 907	2 599	308	5
Riversul	2 388	2 387	2 055	332	1
Rosana	7 855	7 827	6 082	1 745	28
Roseira	2 919	2 915	2 619	296	4
Rubiácea	952	938	771	167	14
Rubinéia	1 280	1 276	876	400	4
Sabino	2 402	2 402	1 708	694	-
Sagres	906	902	728	174	4
Sales	2 583	2 579	1 606	973	4
Sales Oliveira	2 680	2 677	2 435	242	3
Salesópolis	5 866	5 856	4 485	1 371	10
Salmourão	1 629	1 622	1 425	197	7
Saltinho	2 278	2 278	1 959	319	-
Salto	35 678	35 652	31 019	4 633	26
Salto de Pirapora	13 295	13 291	10 435	2 856	4
Salto Grande	3 487	3 484	2 745	739	3
Sandovalina	1 127	1 127	934	193	-
Santa Adélia	4 706	4 702	4 274	428	4
Santa Albertina	2 256	2 254	1 775	479	2
Santa Branca	5 254	5 249	3 969	1 280	5
Santa Clara d'Oeste	855	854	683	171	1
Santa Cruz da Conceição	1 591	1 591	1 191	400	-
Santa Cruz da Esperança	671	671	528	143	-
Santa Cruz das Palmeiras	9 462	9 410	8 732	678	52
Santa Cruz do Rio Pardo	13 988	13 972	12 740	1 232	16
Santa Ernestina	1 694	1 689	1 579	110	5
Santa Fé do Sul	10 765	10 745	9 224	1 521	20
Santa Gertrudes	6 010	6 009	5 500	509	1
Santa Isabel	16 772	16 759	12 902	3 857	13
Santa Lúcia	2 386	2 386	2 140	246	-
Santa Maria da Serra	1 990	1 990	1 589	401	-
Santa Mercedes	1 007	1 007	853	154	-
Santa Rita do Passa Quatro	9 769	9 754	8 450	1 304	15
Santa Rita d'Oeste	1 047	1 047	866	181	-
Santa Rosa de Viterbo	7 436	7 431	6 826	605	5
Santa Salete	622	621	475	146	1
Santana da Ponte Pensa	747	747	581	166	-

Tabela 2.1.20 - Domicílios recenseados, por espécie, segundo os municípios - São Paulo - 2007

(continuação)

Municípios	Domicílios recenseados, por espécie				
	Total	Particulares			Coletivos
		Total	Ocupados (1)	Não-ocupados	
Santana de Parnaíba	32 290	32 281	28 232	4 049	9
Santo Anastácio	7 457	7 450	6 580	870	7
Santo Antônio da Alegria	2 176	2 169	1 876	293	7
Santo Antônio de Posse	7 073	7 069	5 930	1 139	4
Santo Antônio do Aracanguá	3 312	3 310	2 187	1 123	2
Santo Antônio do Jardim	1 962	1 961	1 691	270	1
Santo Antônio do Pinhal	2 920	2 895	1 958	937	25
Santo Expedito	1 121	1 121	908	213	-
Santópolis do Aguapeí	1 378	1 378	1 208	170	-
São Bento do Sapucaí	4 512	4 482	3 283	1 199	30
São Caetano do Sul	53 633	53 570	48 412	5 158	63
São Francisco	1 077	1 077	927	150	-
São João da Boa Vista	30 520	30 500	25 495	5 005	20
São João das Duas Pontes	918	918	816	102	-
São João de Iracema	701	692	558	134	9
São João do Pau d'Alho	780	779	721	58	1
São Joaquim da Barra	14 341	14 329	13 112	1 217	12
São José da Bela Vista	2 847	2 845	2 320	525	2
São José do Barreiro	1 837	1 809	1 290	519	28
São José do Rio Pardo	17 757	17 745	15 124	2 621	12
São Lourenço da Serra	6 230	6 225	4 811	1 414	5
São Luís do Paraitinga	4 523	4 510	3 327	1 183	13
São Manuel	13 019	13 010	11 258	1 752	9
São Miguel Arcanjo	10 640	10 633	8 731	1 902	7
São Pedro	13 090	13 074	9 337	3 737	16
São Pedro do Turvo	2 642	2 639	2 242	397	3
São Roque	25 350	25 325	19 752	5 573	25
São Sebastião	42 946	42 754	20 654	22 100	192
São Sebastião da Grama	4 546	4 544	3 663	881	2
São Simão	4 736	4 684	3 928	756	52
Sarapuí	3 464	3 459	2 367	1 092	5
Sarutaiá	1 433	1 431	1 096	335	2
Sebastianópolis do Sul	1 093	1 092	968	124	1
Serra Azul	2 835	2 829	2 195	634	6
Serra Negra	12 358	12 326	8 124	4 202	32
Serrana	11 421	11 419	10 422	997	2
Sertãozinho	33 579	33 560	30 389	3 171	19
Sete Barras	4 930	4 927	3 735	1 192	3
Severínia	4 757	4 751	4 233	518	6
Silveiras	2 422	2 418	1 630	788	4
Socorro	13 501	13 477	10 536	2 941	24
Sud Mennucci	2 600	2 587	2 319	268	13
Suzanápolis	1 170	1 169	1 060	109	1
Tabapuã	3 981	3 976	3 499	477	5
Tabatinga	4 759	4 755	4 323	432	4
Taciba	2 023	2 022	1 720	302	1
Taguaí	2 867	2 865	2 696	169	2
Taiaçu	1 966	1 965	1 746	219	1
Taiúva	1 987	1 987	1 679	308	-
Tambaú	7 360	7 351	6 454	897	9
Tanabi	9 083	9 077	7 680	1 397	6

Tabela 2.1.20 - Domicílios recenseados, por espécie, segundo os municípios - São Paulo - 2007

(conclusão)

Municípios	Domicílios recenseados, por espécie				
	Total	Particulares			Coletivos
		Total	Ocupados (1)	Não-ocupados	
Tapiraí	3 417	3 408	2 294	1 114	9
Tapiratiba	4 172	4 170	3 489	681	2
Taquaral	883	883	827	56	-
Taquaritinga	18 298	18 271	16 489	1 782	27
Taquarituba	7 428	7 421	6 803	618	7
Taquarivaí	1 655	1 653	1 402	251	2
Tarabai	2 079	2 069	1 850	219	10
Tarumã	3 766	3 764	3 649	115	2
Tatuí	37 853	37 800	31 116	6 684	53
Tejupá	1 782	1 780	1 390	390	2
Teodoro Sampaio	6 962	6 957	6 141	816	5
Terra Roxa	2 905	2 903	2 428	475	2
Tietê	12 114	12 102	10 338	1 764	12
Timburi	1 065	1 063	826	237	2
Torre de Pedra	1 218	1 218	919	299	-
Torrinha	3 247	3 240	2 693	547	7
Trabiju	491	491	395	96	-
Tremembé	11 016	11 002	9 926	1 076	14
Três Fronteiras	2 223	2 220	1 721	499	3
Tuiuti	2 808	2 795	1 851	944	13
Tupã	22 401	22 366	19 975	2 391	35
Tupi Paulista	4 847	4 835	4 320	515	12
Turiúba	970	970	687	283	-
Turmalina	834	833	720	113	1
Ubarana	1 694	1 694	1 366	328	-
Ubatuba	54 443	53 815	22 966	30 849	628
Ubirajara	1 526	1 525	1 333	192	1
Uchoa	3 462	3 459	3 053	406	3
União Paulista	522	522	452	70	-
Urânia	3 346	3 339	2 869	470	7
Uru	697	697	456	241	-
Urupês	4 158	4 151	3 771	380	7
Valentim Gentil	3 412	3 409	3 007	402	3
Valinhos	34 209	34 181	30 274	3 907	28
Valparaíso	6 128	6 076	5 521	555	52
Vargem	3 144	3 142	2 109	1 033	2
Vargem Grande do Sul	12 656	12 644	11 437	1 207	12
Vargem Grande Paulista	14 202	14 193	11 793	2 400	9
Várzea Paulista	31 752	31 743	29 491	2 252	9
Vera Cruz	3 602	3 593	3 035	558	9
Vinhedo	20 146	20 125	17 213	2 912	21
Viradouro	5 684	5 680	5 076	604	4
Vista Alegre do Alto	1 932	1 931	1 833	98	1
Vitória Brasil	622	622	518	104	-
Votorantim	30 785	30 778	28 273	2 505	7
Votuporanga	28 319	28 293	25 736	2 557	26
Zacarias	1 033	1 031	726	305	2

Fonte: IBGE, Contagem da População 2007.

(1) Inclusive os domicílios fechados e os domicílios provenientes de setor censitário cujo arquivo foi danificado, com população estimada.

Tabela 2.1.21 - Domicílios recenseados, por espécie, segundo os municípios - Paraná - 2007

(continua)

Municípios	Domicílios recenseados, por espécie				
	Total	Particulares			Coletivos
		Total	Ocupados (1)	Não-ocupados	
**Paraná**	**2 370 849**	**2 365 057**	**1 890 309**	**474 748**	**5 792**
Abatiá	3 500	3 498	2 438	1 060	2
Adrianópolis	3 140	3 036	1 948	1 088	104
Agudos do Sul	3 477	3 471	2 457	1 014	6
Almirante Tamandaré	31 216	31 182	26 724	4 458	34
Altamira do Paraná	1 661	1 661	1 337	324	-
Alto Paraíso	1 618	1 616	1 038	578	2
Alto Paraná	4 888	4 878	4 030	848	10
Alto Piquiri	3 988	3 974	3 062	912	14
Altônia	7 535	7 515	6 241	1 274	20
Alvorada do Sul	4 425	4 411	2 715	1 696	14
Amaporã	1 719	1 717	1 489	228	2
Ampére	5 653	5 643	5 129	514	10
Anahy	1 035	1 033	905	128	2
Andirá	7 715	7 699	6 631	1 068	16
Ângulo	1 052	1 048	888	160	4
Antonina	8 790	8 780	5 184	3 596	10
Antônio Olinto	2 976	2 966	2 328	638	10
Apucarana	41 562	41 486	35 686	5 800	76
Arapongas	34 550	34 524	29 866	4 658	26
Arapoti	9 215	9 173	7 507	1 666	42
Arapuã	1 539	1 539	1 217	322	-
Araruna	4 447	4 443	3 921	522	4
Araucária	37 138	37 102	32 270	4 832	36
Ariranha do Ivaí	1 093	1 093	805	288	-
Assaí	6 680	6 676	5 006	1 670	4
Assis Chateaubriand	12 088	12 060	10 400	1 660	28
Astorga	9 193	9 177	7 637	1 540	16
Atalaia	1 508	1 504	1 162	342	4
Balsa Nova	4 448	4 446	3 210	1 236	2
Bandeirantes	13 043	13 019	9 789	3 230	24
Barbosa Ferraz	5 071	5 065	4 255	810	6
Barra do Jacaré	1 098	1 096	842	254	2
Barracão	2 895	2 889	2 709	180	6
Bela Vista da Caroba	1 482	1 482	1 274	208	-
Bela Vista do Paraíso	5 909	5 895	4 621	1 274	14
Bituruna	5 388	5 376	4 336	1 040	12
Boa Esperança	1 723	1 719	1 465	254	4
Boa Esperança do Iguaçu	951	951	837	114	-
Boa Ventura de São Roque	2 193	2 193	1 883	310	-
Boa Vista da Aparecida	3 429	3 421	2 369	1 052	8
Bocaiúva do Sul	4 468	4 454	2 756	1 698	14
Bom Jesus do Sul	1 158	1 158	1 128	30	-
Bom Sucesso	2 472	2 468	1 942	526	4
Bom Sucesso do Sul	959	957	895	62	2
Borrazópolis	3 228	3 224	2 776	448	4
Braganey	1 989	1 987	1 803	184	2
Brasilândia do Sul	1 323	1 323	1 053	270	-
Cafeara	1 089	1 089	843	246	-

Tabela 2.1.21 - Domicílios recenseados, por espécie, segundo os municípios - Paraná - 2007

(continuação)

Municípios	Domicílios recenseados, por espécie				
	Total	Particulares			Coletivos
		Total	Ocupados (1)	Não-ocupados	
Cafelândia	4 332	4 328	3 988	340	4
Cafezal do Sul	1 763	1 759	1 367	392	4
Califórnia	2 777	2 773	2 329	444	4
Cambará	8 724	8 710	7 460	1 250	14
Cambé	33 377	33 357	28 423	4 934	20
Cambira	2 304	2 302	2 102	200	2
Campina da Lagoa	6 226	6 206	4 962	1 244	20
Campina do Simão	1 605	1 601	1 167	434	4
Campina Grande do Sul	12 720	12 684	10 314	2 370	36
Campo Bonito	1 576	1 576	1 302	274	-
Campo do Tenente	2 247	2 229	1 791	438	18
Campo Largo	37 718	37 668	31 628	6 040	50
Campo Magro	7 293	7 285	6 401	884	8
Campo Mourão	31 017	30 945	25 765	5 180	72
Cândido de Abreu	6 677	6 653	5 191	1 462	24
Candói	5 679	5 673	4 371	1 302	6
Cantagalo	4 100	4 086	3 484	602	14
Capanema	6 546	6 524	5 836	688	22
Capitão Leônidas Marques	4 703	4 683	4 153	530	20
Carambeí	5 241	5 213	4 737	476	28
Carlópolis	6 044	6 028	4 070	1 958	16
Castro	22 177	22 115	19 093	3 022	62
Catanduvas	3 031	3 021	2 683	338	10
Centenário do Sul	4 306	4 296	3 572	724	10
Cerro Azul	7 186	7 172	5 178	1 994	14
Céu Azul	3 755	3 745	3 355	390	10
Chopinzinho	6 775	6 753	5 689	1 064	22
Cianorte	22 817	22 747	20 161	2 586	70
Cidade Gaúcha	3 578	3 570	3 110	460	8
Clevelândia	6 267	6 257	5 153	1 104	10
Colorado	8 140	8 116	6 778	1 338	24
Congonhinhas	3 089	3 083	2 671	412	6
Conselheiro Mairinck	1 340	1 338	1 086	252	2
Contenda	5 126	5 122	4 146	976	4
Corbélia	5 280	5 266	4 760	506	14
Cornélio Procópio	17 776	17 742	14 676	3 066	34
Coronel Domingos Soares	2 723	2 685	2 081	604	38
Coronel Vivida	7 783	7 767	6 591	1 176	16
Corumbataí do Sul	1 706	1 704	1 290	414	2
Cruz Machado	5 962	5 946	4 916	1 030	16
Cruzeiro do Iguaçu	1 407	1 407	1 203	204	-
Cruzeiro do Oeste	8 204	8 176	6 542	1 634	28
Cruzeiro do Sul	1 631	1 625	1 365	260	6
Cruzmaltina	1 238	1 236	950	286	2
Curiúva	5 128	5 114	4 300	814	14
Diamante do Norte	2 289	2 287	1 725	562	2
Diamante do Sul	1 208	1 202	1 028	174	6
Diamante D'Oeste	1 704	1 694	1 452	242	10
Dois Vizinhos	11 672	11 660	10 402	1 258	12
Douradina	2 564	2 564	2 038	526	-

Tabela 2.1.21 - Domicílios recenseados, por espécie, segundo os municípios - Paraná - 2007

(continuação)

Municípios	Domicílios recenseados, por espécie				
	Total	Particulares			Coletivos
		Total	Ocupados (1)	Não-ocupados	
Doutor Camargo	2 162	2 156	1 870	286	6
Doutor Ulysses	2 097	2 085	1 685	400	12
Enéas Marques	1 929	1 927	1 795	132	2
Engenheiro Beltrão	5 345	5 335	4 361	974	10
Entre Rios do Oeste	1 389	1 383	1 201	182	6
Esperança Nova	695	695	595	100	-
Espigão Alto do Iguaçu	1 662	1 654	1 434	220	8
Farol	1 190	1 186	1 034	152	4
Faxinal	5 891	5 873	4 817	1 056	18
Fazenda Rio Grande	23 557	23 543	21 553	1 990	14
Fênix	1 870	1 862	1 564	298	8
Fernandes Pinheiro	1 788	1 784	1 570	214	4
Figueira	3 300	3 288	2 650	638	12
Flor da Serra do Sul	1 524	1 524	1 376	148	-
Floraí	1 950	1 938	1 676	262	12
Floresta	1 748	1 738	1 614	124	10
Florestópolis	3 946	3 936	3 470	466	10
Flórida	1 008	1 004	770	234	4
Formosa do Oeste	2 607	2 597	2 399	198	10
Foz do Jordão	2 158	2 156	1 640	516	2
Francisco Alves	2 258	2 256	2 002	254	2
Francisco Beltrão	25 682	25 620	22 626	2 994	62
General Carneiro	4 940	4 928	3 990	938	12
Godoy Moreira	1 397	1 393	1 183	210	4
Goioerê	10 523	10 503	9 115	1 388	20
Goioxim	2 584	2 582	2 108	474	2
Grandes Rios	2 866	2 856	2 408	448	10
Guaíra	10 446	10 428	8 796	1 632	18
Guairaçá	2 123	2 123	1 771	352	-
Guamiranga	2 430	2 416	2 120	296	14
Guapirama	1 501	1 499	1 143	356	2
Guaporema	751	743	637	106	8
Guaraci	1 738	1 734	1 572	162	4
Guaraniaçu	6 122	6 106	4 892	1 214	16
Guarapuava	56 872	56 774	48 312	8 462	98
Guaraqueçaba	4 122	4 084	2 210	1 874	38
Guaratuba	36 481	36 405	9 307	27 098	76
Honório Serpa	1 861	1 859	1 705	154	2
Ibaiti	10 483	10 467	8 659	1 808	16
Ibema	1 966	1 958	1 722	236	8
Ibiporã	17 141	17 121	13 885	3 236	20
Icaraíma	3 908	3 894	2 948	946	14
Iguaraçu	1 324	1 322	1 166	156	2
Iguatu	845	845	713	132	-
Imbaú	4 386	4 374	3 310	1 064	12
Imbituva	8 776	8 768	7 826	942	8
Inácio Martins	3 916	3 900	2 992	908	16
Inajá	988	986	888	98	2
Indianópolis	1 469	1 465	1 257	208	4
Ipiranga	4 425	4 419	3 909	510	6

Tabela 2.1.21 - Domicílios recenseados, por espécie, segundo os municípios - Paraná - 2007

(continuação)

Municípios	Domicílios recenseados, por espécie				
	Total	Particulares			Coletivos
		Total	Ocupados (1)	Não-ocupados	
Iporã	5 774	5 762	4 808	954	12
Iracema do Oeste	939	939	815	124	-
Irati	19 719	19 669	16 385	3 284	50
Iretama	4 324	4 312	3 418	894	12
Itaguajé	1 861	1 859	1 413	446	2
Itaipulândia	3 062	3 058	2 586	472	4
Itambaracá	2 750	2 746	2 136	610	4
Itambé	2 290	2 288	1 860	428	2
Itapejara d'Oeste	3 589	3 583	3 203	380	6
Itaperuçu	7 271	7 263	6 279	984	8
Itaúna do Sul	1 525	1 525	1 133	392	-
Ivaí	4 127	4 061	3 561	500	66
Ivaiporã	12 203	12 165	10 113	2 052	38
Ivaté	2 904	2 904	2 424	480	-
Ivatuba	1 398	1 390	828	562	8
Jaboti	1 802	1 802	1 596	206	-
Jacarezinho	15 122	15 046	11 802	3 244	76
Jaguapitã	4 470	4 458	3 626	832	12
Jaguariaíva	11 755	11 717	9 675	2 042	38
Jandaia do Sul	7 464	7 452	6 166	1 286	12
Janiópolis	2 741	2 735	2 233	502	6
Japira	1 851	1 851	1 431	420	-
Japurá	3 065	3 063	2 595	468	2
Jardim Alegre	4 644	4 636	4 234	402	8
Jardim Olinda	577	573	437	136	4
Jataizinho	3 774	3 768	3 250	518	6
Jesuítas	3 173	3 169	2 857	312	4
Joaquim Távora	4 222	4 210	3 336	874	12
Jundiaí do Sul	1 666	1 664	1 162	502	2
Juranda	2 754	2 750	2 376	374	4
Jussara	2 085	2 083	1 879	204	2
Kaloré	1 967	1 965	1 479	486	2
Lapa	16 024	16 008	12 680	3 328	16
Laranjal	1 920	1 918	1 636	282	2
Laranjeiras do Sul	10 461	10 431	9 107	1 324	30
Leópolis	1 757	1 757	1 281	476	-
Lidianópolis	1 457	1 457	1 315	142	-
Lindoeste	1 894	1 892	1 636	256	2
Loanda	6 921	6 895	6 055	840	26
Lobato	1 526	1 522	1 338	184	4
Luiziana	2 782	2 780	2 148	632	2
Lunardelli	1 736	1 732	1 540	192	4
Lupionópolis	1 617	1 613	1 393	220	4
Mallet	4 639	4 623	3 647	976	16
Mamborê	5 491	5 479	4 525	954	12
Mandaguaçu	6 543	6 541	5 507	1 034	2
Mandaguari	11 667	11 649	10 187	1 462	18
Mandirituba	8 397	8 371	5 955	2 416	26
Manfrinópolis	1 046	1 046	968	78	-

Tabela 2.1.21 - Domicílios recenseados, por espécie, segundo os municípios - Paraná - 2007

(continuação)

Municípios	Domicílios recenseados, por espécie				
	Total	Particulares			Coletivos
		Total	Ocupados (1)	Não-ocupados	
Mangueirinha	5 975	5 965	4 781	1 184	10
Manoel Ribas	4 436	4 420	3 810	610	16
Marechal Cândido Rondon	16 887	16 833	14 325	2 508	54
Maria Helena	2 544	2 544	1 888	656	-
Marialva	10 109	10 101	9 303	798	8
Marilândia do Sul	3 380	3 374	2 702	672	6
Marilena	2 722	2 718	1 950	768	4
Mariluz	3 628	3 618	3 160	458	10
Mariópolis	1 943	1 941	1 825	116	2
Maripá	2 008	2 006	1 770	236	2
Marmeleiro	4 442	4 434	3 902	532	8
Marquinho	1 563	1 561	1 389	172	2
Marumbi	1 746	1 746	1 298	448	-
Matelândia	5 135	5 103	4 575	528	32
Matinhos	52 499	52 381	7 293	45 088	118
Mato Rico	1 363	1 359	1 229	130	4
Mauá da Serra	2 667	2 659	2 263	396	8
Medianeira	13 297	13 245	11 863	1 382	52
Mercedes	1 528	1 520	1 452	68	8
Mirador	960	958	720	238	2
Miraselva	729	729	597	132	-
Missal	3 792	3 792	3 236	556	-
Moreira Sales	4 671	4 661	4 127	534	10
Morretes	8 343	8 271	4 855	3 416	72
Munhoz de Melo	1 392	1 392	1 106	286	-
Nossa Senhora das Graças	1 581	1 579	1 233	346	2
Nova Aliança do Ivaí	528	526	404	122	2
Nova América da Colina	1 257	1 257	979	278	-
Nova Aurora	4 013	4 001	3 675	326	12
Nova Cantu	2 522	2 520	2 260	260	2
Nova Esperança	9 214	9 202	7 994	1 208	12
Nova Esperança do Sudoeste	1 610	1 606	1 490	116	4
Nova Fátima	3 147	3 143	2 521	622	4
Nova Laranjeiras	3 351	3 349	3 079	270	2
Nova Londrina	4 618	4 606	3 918	688	12
Nova Olímpia	1 959	1 949	1 695	254	10
Nova Prata do Iguaçu	3 558	3 552	3 062	490	6
Nova Santa Bárbara	1 445	1 441	1 215	226	4
Nova Santa Rosa	2 653	2 643	2 395	248	10
Nova Tebas	3 283	3 277	2 469	808	6
Novo Itacolomi	1 032	1 032	836	196	-
Ortigueira	9 126	9 096	7 348	1 748	30
Ourizona	1 289	1 281	1 047	234	8
Ouro Verde do Oeste	1 815	1 813	1 651	162	2
Paiçandu	11 896	11 890	10 590	1 300	6
Palmas	12 707	12 653	10 871	1 782	54
Palmeira	10 978	10 954	9 176	1 778	24
Palmital	5 061	5 049	4 405	644	12
Palotina	9 916	9 894	8 362	1 532	22
Paraíso do Norte	3 949	3 943	3 427	516	6

Tabela 2.1.21 - Domicílios recenseados, por espécie, segundo os municípios - Paraná - 2007

(continuação)

Municípios	Domicílios recenseados, por espécie				
	Total	Particulares			Coletivos
		Total	Ocupados (1)	Não-ocupados	
Paranacity	3 442	3 440	2 872	568	2
Paranaguá	48 370	48 050	37 738	10 312	320
Paranapoema	962	960	824	136	2
Paranavaí	29 814	29 754	24 950	4 804	60
Pato Bragado	1 639	1 635	1 415	220	4
Pato Branco	24 248	24 216	20 904	3 312	32
Paula Freitas	2 075	2 071	1 617	454	4
Paulo Frontin	2 517	2 513	2 071	442	4
Peabiru	4 740	4 726	4 048	678	14
Perobal	1 869	1 869	1 549	320	-
Pérola	3 810	3 798	3 056	742	12
Pérola d'Oeste	2 503	2 493	2 203	290	10
Piên	4 114	4 106	3 158	948	8
Pinhais	36 174	36 136	33 516	2 620	38
Pinhal de São Bento	761	761	735	26	-
Pinhalão	2 128	2 126	1 730	396	2
Pinhão	10 364	10 350	7 906	2 444	14
Piraí do Sul	8 212	8 188	6 904	1 284	24
Piraquara	25 989	25 935	22 409	3 526	54
Pitanga	11 834	11 794	9 978	1 816	40
Pitangueiras	972	968	802	166	4
Planaltina do Paraná	1 416	1 414	1 168	246	2
Planalto	4 715	4 705	4 337	368	10
Pontal do Paraná	44 238	44 118	5 320	38 798	120
Porecatu	5 313	5 293	4 461	832	20
Porto Amazonas	1 668	1 666	1 240	426	2
Porto Barreiro	1 196	1 192	1 078	114	4
Porto Rico	1 355	1 349	789	560	6
Porto Vitória	1 407	1 403	1 073	330	4
Prado Ferreira	1 287	1 283	999	284	4
Pranchita	1 979	1 975	1 811	164	4
Presidente Castelo Branco	1 548	1 546	1 400	146	2
Primeiro de Maio	4 601	4 589	3 297	1 292	12
Prudentópolis	16 639	16 589	14 029	2 560	50
Quarto Centenário	1 661	1 659	1 447	212	2
Quatiguá	2 639	2 637	2 251	386	2
Quatro Barras	6 927	6 913	5 421	1 492	14
Quatro Pontes	1 260	1 250	1 138	112	10
Quedas do Iguaçu	9 757	9 749	8 565	1 184	8
Querência do Norte	4 681	4 679	3 617	1 062	2
Quinta do Sol	2 152	2 150	1 612	538	2
Quitandinha	6 467	6 459	4 785	1 674	8
Ramilândia	1 325	1 301	1 193	108	24
Rancho Alegre	1 505	1 501	1 275	226	4
Rancho Alegre D'Oeste	1 154	1 152	894	258	2
Realeza	5 671	5 651	4 903	748	20
Rebouças	4 808	4 796	4 242	554	12
Renascença	2 267	2 265	2 003	262	2

Tabela 2.1.21 - Domicílios recenseados, por espécie, segundo os municípios - Paraná - 2007

(continuação)

Municípios	Domicílios recenseados, por espécie				
	Total	Particulares			Coletivos
		Total	Ocupados (1)	Não-ocupados	
Reserva	8 659	8 643	7 109	1 534	16
Reserva do Iguaçu	2 428	2 420	1 858	562	8
Ribeirão Claro	4 726	4 710	3 364	1 346	16
Ribeirão do Pinhal	5 416	5 402	4 180	1 222	14
Rio Azul	4 290	4 282	3 698	584	8
Rio Bom	1 323	1 311	1 047	264	12
Rio Bonito do Iguaçu	4 356	4 352	3 778	574	4
Rio Branco do Ivaí	1 589	1 587	1 103	484	2
Rio Branco do Sul	10 280	10 266	8 902	1 364	14
Rio Negro	10 814	10 790	8 824	1 966	24
Rolândia	18 143	18 115	16 359	1 756	28
Roncador	4 493	4 469	3 771	698	24
Rondon	3 124	3 114	2 676	438	10
Rosário do Ivaí	2 252	2 248	1 772	476	4
Sabáudia	1 803	1 799	1 665	134	4
Salgado Filho	1 608	1 608	1 376	232	-
Salto do Itararé	2 281	2 275	1 645	630	6
Salto do Lontra	4 121	4 105	3 715	390	16
Santa Amélia	1 838	1 836	1 256	580	2
Santa Cecília do Pavão	1 765	1 763	1 173	590	2
Santa Cruz de Monte Castelo	3 014	3 008	2 466	542	6
Santa Fé	3 600	3 588	3 040	548	12
Santa Helena	8 565	8 523	7 139	1 384	42
Santa Inês	921	921	581	340	-
Santa Isabel do Ivaí	3 206	3 198	2 724	474	8
Santa Izabel do Oeste	3 825	3 817	3 485	332	8
Santa Lúcia	1 348	1 348	1 226	122	-
Santa Maria do Oeste	3 609	3 603	3 145	458	6
Santa Mariana	4 596	4 586	3 828	758	10
Santa Mônica	1 214	1 214	1 022	192	-
Santa Tereza do Oeste	3 181	3 181	2 785	396	-
Santa Terezinha de Itaipu	6 618	6 608	5 692	916	10
Santana do Itararé	2 315	2 313	1 727	586	2
Santo Antônio da Platina	15 770	15 722	12 460	3 262	48
Santo Antônio do Caiuá	1 068	1 066	848	218	2
Santo Antônio do Paraíso	879	879	757	122	-
Santo Antônio do Sudoeste	6 184	6 170	5 514	656	14
Santo Inácio	2 316	2 310	1 532	778	6
São Carlos do Ivaí	1 907	1 903	1 709	194	4
São Jerônimo da Serra	4 229	4 215	3 477	738	14
São João	3 682	3 674	3 294	380	8
São João do Caiuá	2 112	2 108	1 854	254	4
São João do Ivaí	4 636	4 620	3 768	852	16
São João do Triunfo	4 680	4 672	3 820	852	8
São Jorge do Ivaí	2 229	2 225	1 723	502	4
São Jorge do Patrocínio	2 211	2 209	1 857	352	2
São Jorge d'Oeste	3 562	3 558	2 780	778	4
São José da Boa Vista	2 581	2 575	1 975	600	6
São José das Palmeiras	1 331	1 329	1 181	148	2
São Manoel do Paraná	703	703	645	58	-

Tabela 2.1.21 - Domicílios recenseados, por espécie, segundo os municípios - Paraná - 2007

(conclusão)

Municípios	Domicílios recenseados, por espécie				
	Total	Particulares			Coletivos
		Total	Ocupados (1)	Não-ocupados	
São Mateus do Sul	14 380	14 342	11 790	2 552	38
São Miguel do Iguaçu	8 875	8 857	7 465	1 392	18
São Pedro do Iguaçu	2 314	2 312	2 042	270	2
São Pedro do Ivaí	3 551	3 547	2 947	600	4
São Pedro do Paraná	1 887	1 887	795	1 092	-
São Sebastião da Amoreira	2 842	2 834	2 472	362	8
São Tomé	1 852	1 848	1 612	236	4
Sapopema	2 289	2 283	1 923	360	6
Sarandi	26 943	26 927	24 551	2 376	16
Saudade do Iguaçu	1 779	1 779	1 375	404	-
Sengés	7 228	7 216	5 544	1 672	12
Serranópolis do Iguaçu	1 477	1 475	1 345	130	2
Sertaneja	2 342	2 338	1 912	426	4
Sertanópolis	6 110	6 094	4 736	1 358	16
Siqueira Campos	6 496	6 460	5 368	1 092	36
Sulina	1 271	1 271	1 043	228	-
Tamarana	4 406	4 402	3 194	1 208	4
Tamboara	1 703	1 697	1 439	258	6
Tapejara	5 083	5 083	4 423	660	-
Tapira	2 288	2 282	1 892	390	6
Teixeira Soares	3 112	3 098	2 694	404	14
Telêmaco Borba	23 639	23 299	19 821	3 478	340
Terra Boa	5 078	5 074	4 532	542	4
Terra Rica	5 439	5 425	4 399	1 026	14
Terra Roxa	5 870	5 854	5 124	730	16
Tibagi	7 282	7 236	5 286	1 950	46
Tijucas do Sul	6 376	6 374	3 880	2 494	2
Toledo	39 413	39 327	34 057	5 270	86
Tomazina	3 939	3 931	2 815	1 116	8
Três Barras do Paraná	3 856	3 850	3 438	412	6
Tunas do Paraná	2 339	2 329	1 607	722	10
Tuneiras do Oeste	2 926	2 922	2 738	184	4
Tupãssi	2 737	2 737	2 493	244	-
Turvo	4 440	4 436	3 932	504	4
Ubiratã	7 796	7 780	6 648	1 132	16
Umuarama	36 193	36 085	30 865	5 220	108
União da Vitória	18 367	18 321	15 357	2 964	46
Uniflor	883	881	737	144	2
Uraí	4 172	4 164	3 590	574	8
Ventania	4 029	3 997	3 035	962	32
Vera Cruz do Oeste	2 897	2 887	2 687	200	10
Verê	2 659	2 659	2 387	272	-
Virmond	1 292	1 292	1 164	128	-
Vitorino	2 300	2 288	1 934	354	12
Wenceslau Braz	6 786	6 766	5 836	930	20
Xambrê	2 938	2 928	1 904	1 024	10

Fonte: IBGE, Contagem da População 2007.

(1) Inclusive os domicílios fechados e os domicílios provenientes de setor censitário cujo arquivo foi danificado, com população estimada.

Tabela 2.1.22 - Domicílios recenseados, por espécie, segundo os municípios - Santa Catarina - 2007

(continua)

Municípios	Domicílios recenseados, por espécie				
	Total	Particulares			Coletivos
		Total	Ocupados (1)	Não-ocupados	
**Santa Catarina**	**1 575 938**	**1 573 112**	**1 306 056**	**267 056**	**2 826**
Abdon Batista	962	962	859	103	-
Abelardo Luz	4 899	4 890	4 643	247	9
Agrolândia	2 841	2 839	2 726	113	2
Agronômica	1 524	1 524	1 398	126	-
Água Doce	2 240	2 235	1 985	250	5
Águas de Chapecó	2 005	1 987	1 841	146	18
Águas Frias	753	753	729	24	-
Águas Mornas	1 618	1 611	1 272	339	7
Alfredo Wagner	3 425	3 421	2 942	479	4
Alto Bela Vista	699	697	627	70	2
Anchieta	2 043	2 041	1 926	115	2
Angelina	1 941	1 934	1 561	373	7
Anita Garibaldi	2 929	2 925	2 735	190	4
Anitápolis	1 293	1 292	988	304	1
Antônio Carlos	2 355	2 354	2 067	287	1
Apiúna	3 248	3 243	2 955	288	5
Arabutã	1 316	1 315	1 238	77	1
Araquari	6 931	6 922	5 885	1 037	9
Araranguá	20 885	20 857	17 943	2 914	28
Armazém	2 424	2 408	2 211	197	16
Arroio Trinta	1 206	1 204	1 095	109	2
Arvoredo	609	607	588	19	2
Ascurra	2 291	2 288	2 063	225	3
Atalanta	1 068	1 068	997	71	-
Aurora	1 726	1 726	1 572	154	-
Balneário Arroio do Silva	9 500	9 492	2 778	6 714	8
Balneário Barra do Sul	6 937	6 928	2 408	4 520	9
Balneário Camboriú	57 165	57 045	33 591	23 454	120
Balneário Gaivota	5 644	5 642	2 446	3 196	2
Balneário Piçarras	8 630	8 624	4 210	4 414	6
Bandeirante	950	950	872	78	-
Barra Bonita	637	636	588	48	1
Barra Velha	13 317	13 304	5 738	7 566	13
Bela Vista do Toldo	1 808	1 808	1 666	142	-
Belmonte	749	749	727	22	-
Benedito Novo	3 277	3 275	2 858	417	2
Biguaçu	18 022	18 008	16 161	1 847	14
Bocaina do Sul	1 050	1 049	903	146	1
Bom Jardim da Serra	1 522	1 517	1 246	271	5
Bom Jesus	674	673	659	14	1
Bom Jesus do Oeste	650	650	617	33	-
Bom Retiro	2 793	2 790	2 429	361	3
Bombinhas	13 307	13 142	3 854	9 288	165
Botuverá	1 538	1 537	1 306	231	1
Braço do Norte	8 886	8 879	8 178	701	7
Braço do Trombudo	1 130	1 126	1 047	79	4
Brunópolis	1 136	1 136	913	223	-
Brusque	31 390	31 366	29 242	2 124	24

Tabela 2.1.22 - Domicílios recenseados, por espécie, segundo os municípios - Santa Catarina - 2007

(continuação)

Municípios	Domicílios recenseados, por espécie				
	Total	Particulares			Coletivos
		Total	Ocupados (1)	Não-ocupados	
Caçador	21 772	21 757	20 154	1 603	15
Caibi	1 905	1 894	1 813	81	11
Calmon	1 149	1 148	1 023	125	1
Camboriú	16 679	16 650	14 922	1 728	29
Campo Alegre	4 149	4 143	3 388	755	6
Campo Belo do Sul	2 549	2 548	2 348	200	1
Campo Erê	2 982	2 978	2 740	238	4
Campos Novos	9 958	9 943	8 759	1 184	15
Canelinha	3 189	3 187	2 884	303	2
Canoinhas	16 955	16 936	15 431	1 505	19
Capão Alto	1 199	1 199	970	229	-
Capinzal	6 487	6 486	5 962	524	1
Capivari de Baixo	6 577	6 575	6 239	336	2
Catanduvas	2 735	2 730	2 579	151	5
Caxambu do Sul	1 579	1 578	1 501	77	1
Celso Ramos	914	914	810	104	-
Cerro Negro	1 232	1 231	1 123	108	1
Chapadão do Lageado	824	824	794	30	-
Chapecó	54 844	54 759	51 259	3 500	85
Cocal do Sul	4 704	4 702	4 375	327	2
Concórdia	23 544	23 500	21 614	1 886	44
Cordilheira Alta	1 021	1 021	941	80	-
Coronel Freitas	3 148	3 146	2 982	164	2
Coronel Martins	777	777	718	59	-
Correia Pinto	4 934	4 931	4 416	515	3
Corupá	4 145	4 138	3 810	328	7
Cunha Porã	3 569	3 566	3 351	215	3
Cunhataí	527	526	507	19	1
Curitibanos	12 298	12 280	11 245	1 035	18
Descanso	2 796	2 796	2 550	246	-
Dionísio Cerqueira	4 603	4 596	4 426	170	7
Dona Emma	1 053	1 051	980	71	2
Doutor Pedrinho	1 181	1 181	1 031	150	-
Entre Rios	832	830	804	26	2
Ermo	600	600	548	52	-
Erval Velho	1 530	1 529	1 300	229	1
Faxinal dos Guedes	3 014	3 013	2 951	62	1
Flor do Sertão	524	524	475	49	-
Formosa do Sul	770	768	743	25	2
Forquilhinha	6 519	6 512	6 115	397	7
Fraiburgo	10 886	10 871	9 748	1 123	15
Frei Rogério	828	828	748	80	-
Galvão	1 021	1 021	985	36	-
Garopaba	9 666	9 512	5 192	4 320	154
Garuva	4 355	4 349	3 798	551	6
Gaspar	16 429	16 406	15 726	680	23
Governador Celso Ramos	6 656	6 651	3 810	2 841	5
Grão Pará	1 902	1 900	1 748	152	2
Gravatal	3 933	3 928	3 387	541	5

Tabela 2.1.22 - Domicílios recenseados, por espécie, segundo os municípios - Santa Catarina - 2007

(continuação)

Municípios	Domicílios recenseados, por espécie				
	Total	Particulares			Coletivos
		Total	Ocupados (1)	Não-ocupados	
Guabiruba	5 320	5 320	4 776	544	-
Guaraciaba	3 282	3 279	3 079	200	3
Guaramirim	9 266	9 245	8 789	456	21
Guarujá do Sul	1 595	1 594	1 454	140	1
Guatambú	1 361	1 360	1 277	83	1
Herval d'Oeste	6 477	6 463	5 954	509	14
Ibiam	611	611	582	29	-
Ibicaré	1 142	1 139	1 068	71	3
Ibirama	5 514	5 509	5 105	404	5
Içara	26 466	26 456	16 576	9 880	10
Ilhota	3 707	3 695	3 387	308	12
Imaruí	4 763	4 754	3 712	1 042	9
Imbituba	17 410	17 334	11 634	5 700	76
Imbuia	1 767	1 765	1 654	111	2
Indaial	16 138	16 128	14 649	1 479	10
Iomerê	827	809	748	61	18
Ipira	1 713	1 711	1 542	169	2
Iporã do Oeste	2 538	2 538	2 368	170	-
Ipuaçu	1 869	1 859	1 712	147	10
Ipumirim	2 279	2 275	2 101	174	4
Iraceminha	1 243	1 241	1 211	30	2
Irani	2 834	2 832	2 685	147	2
Irati	605	605	587	18	-
Irineópolis	3 220	3 219	2 954	265	1
Itá	2 183	2 175	1 984	191	8
Itaiópolis	6 314	6 305	5 593	712	9
Itajaí	53 737	53 661	49 788	3 873	76
Itapema	25 681	25 651	10 590	15 061	30
Itapiranga	4 856	4 847	4 455	392	9
Itapoá	13 126	13 101	3 483	9 618	25
Ituporanga	6 705	6 697	6 106	591	8
Jaborá	1 315	1 313	1 224	89	2
Jacinto Machado	3 596	3 595	3 305	290	1
Jaguaruna	14 234	14 226	4 973	9 253	8
Jaraguá do Sul	42 466	42 380	39 735	2 645	86
Jardinópolis	562	562	532	30	-
Joaçaba	9 000	8 981	7 899	1 082	19
José Boiteux	1 405	1 404	1 230	174	1
Jupiá	640	640	617	23	-
Lacerdópolis	665	664	654	10	1
Lages	50 869	50 770	48 127	2 643	99
Laguna	23 221	23 191	16 153	7 038	30
Lajeado Grande	450	449	408	41	1
Laurentino	1 840	1 837	1 683	154	3
Lauro Muller	4 666	4 664	4 249	415	2
Lebon Régis	3 753	3 748	3 288	460	5
Leoberto Leal	1 221	1 221	1 077	144	-
Lindóia do Sul	1 423	1 422	1 292	130	1
Lontras	3 130	3 129	2 846	283	1

Tabela 2.1.22 - Domicílios recenseados, por espécie, segundo os municípios - Santa Catarina - 2007

(continuação)

Municípios	Domicílios recenseados, por espécie				
	Total	Particulares			Coletivos
		Total	Ocupados (1)	Não-ocupados	
Luiz Alves	2 827	2 825	2 568	257	2
Luzerna	1 900	1 900	1 762	138	-
Macieira	576	575	532	43	1
Mafra	16 871	16 863	15 342	1 521	8
Major Gercino	1 099	1 098	902	196	1
Major Vieira	2 333	2 331	2 195	136	2
Maracajá	1 929	1 925	1 785	140	4
Maravilha	7 013	7 010	6 699	311	3
Marema	686	686	643	43	-
Massaranduba	4 309	4 300	4 071	229	9
Matos Costa	924	924	776	148	-
Meleiro	2 285	2 284	2 087	197	1
Mirim Doce	885	885	771	114	-
Modelo	1 193	1 192	1 142	50	1
Mondaí	2 926	2 918	2 743	175	8
Monte Carlo	2 680	2 678	2 508	170	2
Monte Castelo	2 686	2 682	2 327	355	4
Morro da Fumaça	4 812	4 811	4 512	299	1
Morro Grande	928	928	807	121	-
Navegantes	22 854	22 838	15 403	7 435	16
Nova Erechim	1 316	1 315	1 232	83	1
Nova Itaberaba	1 231	1 231	1 184	47	-
Nova Trento	3 865	3 863	3 441	422	2
Nova Veneza	4 121	4 116	3 692	424	5
Novo Horizonte	868	868	812	56	-
Orleans	6 748	6 745	6 238	507	3
Otacílio Costa	5 217	5 217	4 790	427	-
Ouro	2 373	2 369	2 161	208	4
Ouro Verde	642	641	626	15	1
Paial	591	591	526	65	-
Painel	835	834	705	129	1
Palhoça	48 965	48 586	36 618	11 968	379
Palma Sola	2 350	2 343	2 174	169	7
Palmeira	901	900	738	162	1
Palmitos	5 584	5 574	5 022	552	10
Papanduva	5 406	5 395	4 919	476	11
Paraíso	1 315	1 313	1 225	88	2
Passo de Torres	3 689	3 688	1 655	2 033	1
Passos Maia	1 282	1 281	1 180	101	1
Paulo Lopes	2 413	2 405	1 967	438	8
Pedras Grandes	1 661	1 661	1 516	145	-
Penha	12 891	12 876	6 533	6 343	15
Peritiba	1 009	1 006	928	78	3
Petrolândia	2 053	2 051	1 837	214	2
Pinhalzinho	4 661	4 658	4 441	217	3
Pinheiro Preto	872	872	817	55	-
Piratuba	2 118	2 102	1 524	578	16
Planalto Alegre	789	789	774	15	-
Pomerode	8 120	8 106	7 630	476	14

Tabela 2.1.22 - Domicílios recenseados, por espécie, segundo os municípios - Santa Catarina - 2007

(continuação)

Municípios	Domicílios recenseados, por espécie				
	Total	Particulares			Coletivos
		Total	Ocupados (1)	Não-ocupados	
Ponte Alta	1 793	1 789	1 564	225	4
Ponte Alta do Norte	1 164	1 163	1 009	154	1
Ponte Serrada	3 419	3 414	3 203	211	5
Porto Belo	8 214	8 192	4 176	4 016	22
Porto União	10 998	10 984	9 745	1 239	14
Pouso Redondo	4 389	4 384	4 076	308	5
Praia Grande	2 614	2 609	2 303	306	5
Presidente Castello Branco	561	560	520	40	1
Presidente Getúlio	4 471	4 462	4 082	380	9
Presidente Nereu	805	805	697	108	-
Princesa	774	774	741	33	-
Quilombo	3 454	3 447	3 109	338	7
Rancho Queimado	1 249	1 247	911	336	2
Rio das Antas	2 002	2 002	1 809	193	-
Rio do Campo	2 072	2 072	1 788	284	-
Rio do Oeste	2 192	2 185	2 028	157	7
Rio do Sul	19 227	19 188	18 035	1 153	39
Rio dos Cedros	3 909	3 900	2 959	941	9
Rio Fortuna	1 290	1 289	1 246	43	1
Rio Negrinho	12 933	12 911	11 972	939	22
Rio Rufino	784	782	716	66	2
Riqueza	1 502	1 499	1 461	38	3
Rodeio	3 678	3 667	3 364	303	11
Romelândia	1 938	1 937	1 780	157	1
Salete	2 350	2 349	2 186	163	1
Saltinho	1 179	1 178	1 113	65	1
Salto Veloso	1 293	1 290	1 222	68	3
Sangão	3 107	3 103	2 949	154	4
Santa Cecília	4 861	4 856	4 287	569	5
Santa Helena	706	706	682	24	-
Santa Rosa de Lima	600	599	544	55	1
Santa Rosa do Sul	2 814	2 812	2 502	310	2
Santa Terezinha	2 527	2 526	2 394	132	1
Santa Terezinha do Progresso	964	964	854	110	-
Santiago do Sul	481	481	420	61	-
Santo Amaro da Imperatriz	6 064	6 053	5 262	791	11
São Bento do Sul	23 782	23 751	21 592	2 159	31
São Bernardino	842	842	730	112	-
São Bonifácio	1 148	1 146	974	172	2
São Carlos	3 376	3 371	3 092	279	5
São Cristovão do Sul	1 431	1 426	1 269	157	5
São Domingos	3 008	2 998	2 804	194	10
São Francisco do Sul	23 394	23 338	11 536	11 802	56
São João Batista	7 393	7 384	6 689	695	9
São João do Itaperiú	1 128	1 127	967	160	1
São João do Oeste	1 775	1 774	1 731	43	1
São João do Sul	2 487	2 487	2 207	280	-
São Joaquim	8 469	8 457	7 516	941	12
São José do Cedro	4 254	4 248	4 078	170	6

Tabela 2.1.22 - Domicílios recenseados, por espécie, segundo os municípios - Santa Catarina - 2007

(conclusão)

Municípios	Domicílios recenseados, por espécie				
	Total	Particulares			Coletivos
		Total	Ocupados (1)	Não-ocupados	
São José do Cerrito	3 516	3 514	3 132	382	2
São Lourenço do Oeste	6 987	6 962	6 532	430	25
São Ludgero	3 343	3 338	2 981	357	5
São Martinho	1 056	1 055	939	116	1
São Miguel da Boa Vista	579	579	561	18	-
São Miguel do Oeste	11 779	11 752	10 954	798	27
São Pedro de Alcântara	1 502	1 497	1 118	379	5
Saudades	2 617	2 611	2 452	159	6
Schroeder	3 945	3 941	3 727	214	4
Seara	5 445	5 444	5 152	292	1
Serra Alta	932	931	914	17	1
Siderópolis	4 103	4 099	3 709	390	4
Sombrio	8 586	8 582	7 704	878	4
Sul Brasil	869	869	846	23	-
Taió	5 713	5 698	5 144	554	15
Tangará	2 839	2 833	2 636	197	6
Tigrinhos	538	538	530	8	-
Tijucas	9 223	9 216	8 364	852	7
Timbé do Sul	1 783	1 782	1 592	190	1
Timbó	11 140	11 130	10 297	833	10
Timbó Grande	2 200	2 198	1 920	278	2
Três Barras	5 344	5 332	4 944	388	12
Treviso	1 182	1 182	1 031	151	-
Treze de Maio	2 068	2 068	1 964	104	-
Treze Tílias	2 010	2 010	1 751	259	-
Trombudo Central	2 057	2 056	1 905	151	1
Tubarão	33 202	33 160	29 851	3 309	42
Tunápolis	1 426	1 426	1 331	95	-
Turvo	3 635	3 629	3 404	225	6
União do Oeste	900	897	877	20	3
Urubici	3 381	3 377	3 141	236	4
Urupema	832	830	744	86	2
Urussanga	6 215	6 210	5 684	526	5
Vargeão	1 066	1 061	1 023	38	5
Vargem	1 046	1 044	948	96	2
Vargem Bonita	1 289	1 284	1 216	68	5
Vidal Ramos	2 102	2 102	1 790	312	-
Videira	15 045	15 020	13 848	1 172	25
Vitor Meireles	1 562	1 556	1 490	66	6
Witmarsum	1 004	1 000	947	53	4
Xanxerê	13 090	13 070	12 322	748	20
Xavantina	1 162	1 155	1 149	6	7
Xaxim	7 820	7 815	7 305	510	5
Zortéa	947	946	912	34	1

Fonte: IBGE, Contagem da População 2007.

(1) Inclusive os domicílios fechados com população estimada.

Tabela 2.1.23 - Domicílios recenseados, por espécie, segundo os municípios - Rio Grande do Sul - 2007

(continua)

Municípios	Domicílios recenseados, por espécie				
	Total	Particulares			Coletivos
		Total	Ocupados (1)	Não-ocupados	
**Rio Grande do Sul**	**2 391 918**	**2 388 663**	**2 018 847**	**369 816**	**3 255**
Aceguá	1 550	1 549	1 328	221	1
Água Santa	1 119	1 119	1 074	45	-
Agudo	5 779	5 772	5 166	606	7
Ajuricaba	2 623	2 620	2 405	215	3
Alecrim	2 743	2 741	2 434	307	2
Alegrete	28 197	28 157	25 134	3 023	40
Alegria	1 708	1 707	1 551	156	1
Almirante Tamandaré do Sul	792	791	734	57	1
Alpestre	2 968	2 966	2 819	147	2
Alto Alegre	654	654	615	39	-
Alto Feliz	1 183	1 183	973	210	-
Amaral Ferrador	2 315	2 314	1 968	346	1
Ametista do Sul	2 756	2 756	2 431	325	-
André da Rocha	489	487	388	99	2
Anta Gorda	1 969	1 964	1 863	101	5
Antônio Prado	4 758	4 753	4 236	517	5
Arambaré	3 327	3 308	1 291	2 017	19
Araricá	1 792	1 785	1 504	281	7
Aratiba	2 236	2 234	2 050	184	2
Arroio do Meio	6 250	6 236	5 725	511	14
Arroio do Padre	744	744	673	71	-
Arroio do Sal	10 828	10 775	2 325	8 450	53
Arroio do Tigre	3 917	3 913	3 737	176	4
Arroio dos Ratos	5 164	5 161	4 395	766	3
Arroio Grande	7 428	7 424	6 277	1 147	4
Arvorezinha	3 384	3 374	3 090	284	10
Augusto Pestana	2 626	2 625	2 464	161	1
Áurea	1 244	1 244	1 172	72	-
Bagé	40 755	40 709	36 299	4 410	46
Balneário Pinhal	14 816	14 782	3 421	11 361	34
Barão	1 856	1 855	1 657	198	1
Barão de Cotegipe	2 117	2 113	1 982	131	4
Barão do Triunfo	2 597	2 596	2 217	379	1
Barra do Guarita	967	967	919	48	-
Barra do Quaraí	1 306	1 301	1 165	136	5
Barra do Ribeiro	4 667	4 665	3 771	894	2
Barra do Rio Azul	630	630	592	38	-
Barra Funda	701	699	665	34	2
Barracão	1 933	1 930	1 721	209	3
Barros Cassal	3 918	3 913	3 529	384	5
Benjamin Constant do Sul	673	672	611	61	1
Bento Gonçalves	35 596	35 555	32 835	2 720	41
Boa Vista das Missões	673	673	646	27	-
Boa Vista do Buricá	2 251	2 243	2 085	158	8
Boa Vista do Cadeado	832	832	782	50	-
Boa Vista do Incra	828	828	769	59	-
Boa Vista do Sul	929	929	840	89	-
Bom Jesus	4 559	4 548	3 808	740	11
Bom Princípio	3 829	3 829	3 386	443	-
Bom Progresso	825	825	751	74	-
Bom Retiro do Sul	4 318	4 313	3 769	544	5
Boqueirão do Leão	2 660	2 654	2 411	243	6
Bossoroca	3 031	3 026	2 502	524	5

Tabela 2.1.23 - Domicílios recenseados, por espécie, segundo os municípios - Rio Grande do Sul - 2007

(continuação)

Municípios	Domicílios recenseados, por espécie				
	Total	Particulares			Coletivos
		Total	Ocupados (1)	Não-ocupados	
Bozano	763	763	690	73	-
Braga	1 345	1 344	1 226	118	1
Brochier	1 801	1 797	1 613	184	4
Butiá	7 123	7 115	6 276	839	8
Caçapava do Sul	13 772	13 762	11 386	2 376	10
Cacequi	4 889	4 883	4 322	561	6
Cachoeira do Sul	33 308	33 263	28 701	4 562	45
Cachoeirinha	39 013	38 992	35 901	3 091	21
Cacique Doble	1 439	1 436	1 366	70	3
Caibaté	1 835	1 834	1 696	138	1
Caiçara	1 704	1 701	1 569	132	3
Camaquã	21 691	21 677	19 344	2 333	14
Camargo	842	841	768	73	1
Cambará do Sul	2 600	2 585	2 175	410	15
Campestre da Serra	1 330	1 330	1 048	282	-
Campina das Missões	2 179	2 176	2 021	155	3
Campinas do Sul	2 081	2 080	1 818	262	1
Campo Bom	20 200	20 195	18 252	1 943	5
Campo Novo	1 916	1 916	1 773	143	-
Campos Borges	1 405	1 405	1 187	218	-
Candelária	10 903	10 894	9 886	1 008	9
Cândido Godói	2 229	2 228	2 080	148	1
Candiota	2 913	2 906	2 588	318	7
Canela	15 757	15 697	12 253	3 444	60
Canguçu	19 789	19 778	17 031	2 747	11
Canudos do Vale	648	648	607	41	-
Capão Bonito do Sul	649	649	586	63	-
Capão da Canoa	35 330	35 265	11 800	23 465	65
Capão do Cipó	1 135	1 135	1 028	107	-
Capão do Leão	8 066	8 062	7 357	705	4
Capela de Santana	4 024	4 024	3 531	493	-
Capitão	853	853	798	55	-
Capivari do Sul	1 222	1 220	1 053	167	2
Caraá	3 301	3 300	2 439	861	1
Carazinho	20 953	20 930	19 226	1 704	23
Carlos Barbosa	8 453	8 449	7 793	656	4
Carlos Gomes	543	543	495	48	-
Casca	2 828	2 819	2 549	270	9
Caseiros	1 118	1 118	954	164	-
Catuípe	3 356	3 355	3 134	221	1
Centenário	956	956	905	51	-
Cerrito	2 854	2 853	2 344	509	1
Cerro Branco	1 717	1 715	1 561	154	2
Cerro Grande	812	812	794	18	-
Cerro Grande do Sul	3 290	3 290	2 882	408	-
Cerro Largo	4 342	4 336	4 037	299	6
Chapada	3 494	3 489	3 160	329	5
Charqueadas	10 155	10 142	9 578	564	13
Charrua	959	959	920	39	-
Chiapetta	1 442	1 438	1 320	118	4
Chuí	2 305	2 299	1 699	600	6
Chuvisca	1 560	1 560	1 412	148	-
Cidreira	17 682	17 657	3 399	14 258	25
Ciríaco	1 706	1 705	1 584	121	1

Tabela 2.1.23 - Domicílios recenseados, por espécie, segundo os municípios - Rio Grande do Sul - 2007

(continuação)

Municípios	Domicílios recenseados, por espécie				
	Total	Particulares			Coletivos
		Total	Ocupados (1)	Não-ocupados	
Colinas	906	906	809	97	-
Colorado	1 323	1 321	1 258	63	2
Condor	2 276	2 274	2 075	199	2
Constantina	3 186	3 181	2 941	240	5
Coqueiro Baixo	581	581	519	62	-
Coqueiros do Sul	1 141	1 141	1 065	76	-
Coronel Barros	924	924	817	107	-
Coronel Bicaco	2 736	2 735	2 545	190	1
Coronel Pilar	559	559	482	77	-
Cotiporã	1 378	1 375	1 225	150	3
Coxilha	966	965	920	45	1
Crissiumal	5 174	5 168	4 832	336	6
Cristal	2 636	2 636	2 174	462	-
Cristal do Sul	1 022	1 022	940	82	-
Cruz Alta	23 578	23 555	20 576	2 979	23
Cruzaltense	754	753	692	61	1
Cruzeiro do Sul	4 518	4 515	3 945	570	3
David Canabarro	1 499	1 496	1 430	66	3
Derrubadas	1 119	1 119	1 057	62	-
Dezesseis de Novembro	1 058	1 058	960	98	-
Dilermando de Aguiar	1 232	1 232	1 066	166	-
Dois Irmãos	9 857	9 848	8 045	1 803	9
Dois Irmãos das Missões	798	798	729	69	-
Dois Lajeados	1 101	1 098	1 003	95	3
Dom Feliciano	5 114	5 111	4 453	658	3
Dom Pedrito	14 402	14 371	12 558	1 813	31
Dom Pedro de Alcântara	1 071	1 071	973	98	-
Dona Francisca	1 235	1 232	1 107	125	3
Doutor Maurício Cardoso	2 248	2 246	1 841	405	2
Doutor Ricardo	672	672	624	48	-
Eldorado do Sul	11 665	11 625	9 693	1 932	40
Encantado	7 128	7 119	6 441	678	9
Encruzilhada do Sul	9 623	9 608	8 034	1 574	15
Engenho Velho	451	451	424	27	-
Entre Rios do Sul	1 170	1 169	997	172	1
Entre-Ijuís	3 361	3 361	2 997	364	-
Erebango	944	944	883	61	-
Erechim	33 578	33 540	30 941	2 599	38
Ernestina	1 388	1 388	1 009	379	-
Erval Grande	1 708	1 704	1 651	53	4
Erval Seco	2 792	2 786	2 569	217	6
Esmeralda	1 341	1 337	1 107	230	4
Esperança do Sul	1 223	1 222	1 130	92	1
Espumoso	5 156	5 149	4 842	307	7
Estação	2 068	2 068	1 940	128	-
Estância Velha	13 881	13 876	12 884	992	5
Esteio	26 442	26 420	24 870	1 550	22
Estrela	10 488	10 480	9 529	951	8
Estrela Velha	1 136	1 135	1 091	44	1
Eugênio de Castro	1 167	1 166	1 052	114	1
Fagundes Varela	812	808	711	97	4
Farroupilha	19 937	19 921	18 462	1 459	16
Faxinal do Soturno	2 111	2 106	1 931	175	5
Faxinalzinho	895	894	823	71	1

Tabela 2.1.23 - Domicílios recenseados, por espécie, segundo os municípios - Rio Grande do Sul - 2007

(continuação)

Municípios	Domicílios recenseados, por espécie				
	Total	Particulares			Coletivos
		Total	Ocupados (1)	Não-ocupados	
Fazenda Vilanova	1 140	1 140	1 050	90	-
Feliz	4 319	4 314	3 797	517	5
Flores da Cunha	9 186	9 180	7 923	1 257	6
Floriano Peixoto	684	683	665	18	1
Fontoura Xavier	3 797	3 794	3 373	421	3
Formigueiro	2 668	2 666	2 284	382	2
Forquetinha	1 030	1 029	877	152	1
Fortaleza dos Valos	1 769	1 767	1 478	289	2
Frederico Westphalen	9 527	9 506	8 677	829	21
Garibaldi	10 061	10 052	9 187	865	9
Garruchos	1 303	1 301	1 121	180	2
Gaurama	2 099	2 096	1 936	160	3
General Câmara	3 799	3 796	3 063	733	3
Gentil	523	523	493	30	-
Getúlio Vargas	5 822	5 815	5 296	519	7
Giruá	6 025	6 015	5 655	360	10
Glorinha	3 063	3 060	2 320	740	3
Gramado	15 336	15 207	10 585	4 622	129
Gramado dos Loureiros	730	729	715	14	1
Gramado Xavier	1 223	1 223	1 142	81	-
Guabiju	564	563	515	48	1
Guaíba	32 344	32 317	29 808	2 509	27
Guaporé	7 409	7 401	6 989	412	8
Guarani das Missões	2 815	2 812	2 638	174	3
Harmonia	1 331	1 331	1 181	150	-
Herval	3 124	3 122	2 598	524	2
Herveiras	992	991	843	148	1
Horizontina	6 880	6 874	6 336	538	6
Hulha Negra	2 064	2 058	1 859	199	6
Humaitá	1 762	1 760	1 652	108	2
Ibarama	1 445	1 445	1 292	153	-
Ibiaçá	1 674	1 672	1 587	85	2
Ibiraiaras	2 364	2 363	2 105	258	1
Ibirapuitã	1 482	1 480	1 369	111	2
Ibirubá	6 840	6 830	6 348	482	10
Igrejinha	11 062	11 052	10 017	1 035	10
Ijuí	27 436	27 380	25 154	2 226	56
Ilópolis	1 363	1 359	1 233	126	4
Imbé	23 251	23 219	4 890	18 329	32
Imigrante	1 098	1 096	1 003	93	2
Independência	2 386	2 386	2 229	157	-
Inhacorá	845	843	734	109	2
Ipê	2 240	2 237	1 861	376	3
Ipiranga do Sul	670	670	636	34	-
Iraí	3 049	3 044	2 724	320	5
Itaara	2 673	2 671	1 483	1 188	2
Itacurubi	1 397	1 395	1 189	206	2
Itapuca	735	735	695	40	-
Itaqui	12 064	12 048	10 837	1 211	16
Itati	1 128	1 127	904	223	1
Itatiba do Sul	1 469	1 467	1 381	86	2
Ivorá	775	773	715	58	2
Ivoti	6 433	6 427	5 881	546	6
Jaboticaba	1 395	1 390	1 300	90	5

Tabela 2.1.23 - Domicílios recenseados, por espécie, segundo os municípios - Rio Grande do Sul - 2007

(continuação)

Municípios	Domicílios recenseados, por espécie				
	Total	Particulares			Coletivos
		Total	Ocupados (1)	Não-ocupados	
Jacuizinho	892	892	820	72	-
Jacutinga	1 295	1 295	1 153	142	-
Jaguarão	11 620	11 601	9 835	1 766	19
Jaguari	4 290	4 283	3 858	425	7
Jaquirana	1 769	1 763	1 403	360	6
Jari	1 344	1 342	1 208	134	2
Jóia	2 722	2 717	2 541	176	5
Júlio de Castilhos	6 706	6 698	6 078	620	8
Lagoa Bonita do Sul	834	833	806	27	1
Lagoa dos Três Cantos	563	563	523	40	-
Lagoa Vermelha	10 096	10 087	9 105	982	9
Lagoão	2 085	2 081	1 905	176	4
Lajeado	24 691	24 657	22 709	1 948	34
Lajeado do Bugre	832	832	779	53	-
Lavras do Sul	3 298	3 294	2 703	591	4
Liberato Salzano	1 987	1 985	1 842	143	2
Lindolfo Collor	1 845	1 845	1 662	183	-
Linha Nova	632	632	507	125	-
Maçambara	1 598	1 593	1 361	232	5
Machadinho	1 961	1 958	1 762	196	3
Mampituba	1 087	1 087	955	132	-
Manoel Viana	2 758	2 754	2 281	473	4
Maquiné	2 996	2 994	2 396	598	2
Maratá	855	853	799	54	2
Marau	11 522	11 509	10 798	711	13
Marcelino Ramos	2 048	2 033	1 730	303	15
Mariana Pimentel	1 654	1 651	1 372	279	3
Mariano Moro	807	807	739	68	-
Marques de Souza	1 831	1 830	1 373	457	1
Mata	1 969	1 967	1 792	175	2
Mato Castelhano	1 020	1 019	830	189	1
Mato Leitão	1 282	1 282	1 170	112	-
Mato Queimado	667	666	623	43	1
Maximiliano de Almeida	1 716	1 716	1 598	118	-
Minas do Leão	2 557	2 554	2 405	149	3
Miraguaí	1 672	1 672	1 560	112	-
Montauri	514	514	453	61	-
Monte Alegre dos Campos	1 157	1 157	961	196	-
Monte Belo do Sul	950	947	823	124	3
Montenegro	20 523	20 504	18 574	1 930	19
Mormaço	854	853	803	50	1
Morrinhos do Sul	1 234	1 234	1 109	125	-
Morro Redondo	2 588	2 585	2 158	427	3
Morro Reuter	2 243	2 242	1 801	441	1
Mostardas	6 704	6 694	4 029	2 665	10
Muçum	1 747	1 745	1 561	184	2
Muitos Capões	1 116	1 116	973	143	-
Muliterno	542	542	518	24	-
Não-Me-Toque	5 324	5 315	5 072	243	9
Nicolau Vergueiro	744	744	618	126	-
Nonoai	4 122	4 117	3 876	241	5
Nova Alvorada	962	959	889	70	3
Nova Araçá	1 263	1 262	1 168	94	1
Nova Bassano	2 727	2 721	2 520	201	6

Tabela 2.1.23 - Domicílios recenseados, por espécie, segundo os municípios - Rio Grande do Sul - 2007

(continuação)

Municípios	Domicílios recenseados, por espécie				
	Total	Particulares			Coletivos
		Total	Ocupados (1)	Não-ocupados	
Nova Boa Vista	709	708	661	47	1
Nova Bréscia	1 090	1 089	989	100	1
Nova Candelária	883	883	837	46	-
Nova Esperança do Sul	1 705	1 703	1 565	138	2
Nova Hartz	5 866	5 864	5 422	442	2
Nova Pádua	767	764	682	82	3
Nova Palma	1 943	1 941	1 810	131	2
Nova Petrópolis	7 411	7 389	5 948	1 441	22
Nova Prata	7 828	7 819	7 079	740	9
Nova Ramada	876	876	813	63	-
Nova Roma do Sul	1 163	1 159	1 005	154	4
Nova Santa Rita	7 213	7 211	6 318	893	2
Novo Barreiro	1 279	1 275	1 193	82	4
Novo Cabrais	1 474	1 473	1 271	202	1
Novo Machado	1 598	1 597	1 490	107	1
Novo Tiradentes	723	723	703	20	-
Novo Xingu	609	609	596	13	-
Osório	17 561	17 547	12 529	5 018	14
Paim Filho	1 473	1 470	1 349	121	3
Palmares do Sul	12 216	12 209	3 859	8 350	7
Palmeira das Missões	11 826	11 811	10 603	1 208	15
Palmitinho	2 306	2 304	2 158	146	2
Panambi	12 981	12 972	11 931	1 041	9
Pantano Grande	3 786	3 783	3 179	604	3
Paraí	2 195	2 194	1 987	207	1
Paraíso do Sul	2 613	2 612	2 389	223	1
Pareci Novo	1 180	1 180	1 026	154	-
Parobé	16 780	16 769	15 481	1 288	11
Passa Sete	1 623	1 621	1 488	133	2
Passo do Sobrado	2 217	2 216	1 981	235	1
Paulo Bento	693	692	631	61	1
Paverama	2 928	2 924	2 629	295	4
Pedras Altas	1 003	1 001	856	145	2
Pedro Osório	3 353	3 351	2 836	515	2
Pejuçara	1 254	1 252	1 173	79	2
Picada Café	1 821	1 795	1 558	237	26
Pinhal	789	789	733	56	-
Pinhal da Serra	822	822	700	122	-
Pinhal Grande	1 383	1 383	1 308	75	-
Pinheirinho do Vale	1 435	1 435	1 338	97	-
Pinheiro Machado	5 428	5 427	4 716	711	1
Pirapó	1 106	1 104	990	114	2
Piratini	8 481	8 474	7 081	1 393	7
Planalto	3 524	3 521	3 285	236	3
Poço das Antas	718	718	647	71	-
Pontão	1 425	1 422	1 250	172	3
Ponte Preta	569	568	551	17	1
Portão	9 987	9 981	9 072	909	6
Porto Lucena	2 104	2 102	1 933	169	2
Porto Mauá	1 036	1 034	810	224	2
Porto Vera Cruz	888	887	737	150	1
Porto Xavier	3 780	3 775	3 538	237	5
Pouso Novo	742	741	636	105	1
Presidente Lucena	889	889	732	157	-

Tabela 2.1.23 - Domicílios recenseados, por espécie, segundo os municípios - Rio Grande do Sul - 2007

(continuação)

Municípios	Domicílios recenseados, por espécie				
	Total	Particulares			Coletivos
		Total	Ocupados (1)	Não-ocupados	
Progresso	2 185	2 182	1 922	260	3
Protásio Alves	759	759	634	125	-
Putinga	1 390	1 389	1 289	100	1
Quaraí	8 233	8 225	7 182	1 043	8
Quatro Irmãos	631	630	530	100	1
Quevedos	987	986	884	102	1
Quinze de Novembro	1 527	1 526	1 186	340	1
Redentora	2 737	2 733	2 586	147	4
Relvado	832	832	727	105	-
Restinga Seca	6 081	6 080	5 002	1 078	1
Rio dos Índios	1 347	1 347	1 283	64	-
Rio Pardo	15 029	15 007	12 465	2 542	22
Riozinho	1 811	1 808	1 425	383	3
Roca Sales	3 687	3 683	3 303	380	4
Rodeio Bonito	1 995	1 993	1 865	128	2
Rolador	1 005	1 005	908	97	-
Rolante	7 360	7 356	6 400	956	4
Ronda Alta	3 642	3 624	2 908	716	18
Rondinha	1 765	1 764	1 643	121	1
Roque Gonzales	2 466	2 464	2 310	154	2
Rosário do Sul	13 755	13 733	12 789	944	22
Sagrada Família	925	925	855	70	-
Saldanha Marinho	1 126	1 125	1 008	117	1
Salto do Jacuí	4 299	4 294	3 828	466	5
Salvador das Missões	896	896	824	72	-
Salvador do Sul	2 271	2 265	2 062	203	6
Sananduva	5 364	5 351	4 860	491	13
Santa Bárbara do Sul	3 359	3 356	3 004	352	3
Santa Cecília do Sul	590	590	525	65	-
Santa Clara do Sul	1 854	1 849	1 726	123	5
Santa Cruz do Sul	42 346	42 252	38 462	3 790	94
Santa Margarida do Sul	909	908	748	160	1
Santa Maria do Herval	2 227	2 209	1 928	281	18
Santa Rosa	22 891	22 860	21 152	1 708	31
Santa Tereza	706	705	590	115	1
Santa Vitória do Palmar	16 194	16 136	10 875	5 261	58
Santana da Boa Vista	3 596	3 596	2 949	647	-
Santana do Livramento	31 542	31 506	27 013	4 493	36
Santiago	18 326	18 301	16 556	1 745	25
Santo Ângelo	26 701	26 668	24 193	2 475	33
Santo Antônio da Patrulha	15 439	15 418	12 724	2 694	21
Santo Antônio das Missões	4 297	4 293	3 817	476	4
Santo Antônio do Palma	661	658	628	30	3
Santo Antônio do Planalto	772	771	681	90	1
Santo Augusto	4 866	4 861	4 483	378	5
Santo Cristo	4 878	4 870	4 572	298	8
Santo Expedito do Sul	883	882	830	52	1
São Borja	20 498	20 483	18 894	1 589	15
São Domingos do Sul	946	946	879	67	-
São Francisco de Assis	7 583	7 576	6 647	929	7
São Francisco de Paula	9 422	9 396	6 880	2 516	26
São Gabriel	21 774	21 735	18 895	2 840	39
São Jerônimo	7 439	7 428	6 476	952	11
São João da Urtiga	1 654	1 653	1 553	100	1

Tabela 2.1.23 - Domicílios recenseados, por espécie, segundo os municípios - Rio Grande do Sul - 2007

(continuação)

Municípios	Domicílios recenseados, por espécie				
	Total	Particulares			Coletivos
		Total	Ocupados (1)	Não-ocupados	
São João do Polêsine	958	954	808	146	4
São Jorge	934	934	855	79	-
São José das Missões	973	972	921	51	1
São José do Herval	852	849	754	95	3
São José do Hortêncio	1 363	1 363	1 215	148	-
São José do Inhacorá	781	781	700	81	-
São José do Norte	10 406	10 391	8 352	2 039	15
São José do Ouro	2 435	2 432	2 252	180	3
São José do Sul	693	690	609	81	3
São José dos Ausentes	1 146	1 145	977	168	1
São Lourenço do Sul	14 628	14 610	12 531	2 079	18
São Luiz Gonzaga	12 648	12 633	11 406	1 227	15
São Marcos	7 134	7 126	6 333	793	8
São Martinho	2 125	2 123	1 984	139	2
São Martinho da Serra	1 331	1 331	1 139	192	-
São Miguel das Missões	2 624	2 622	2 339	283	2
São Nicolau	2 262	2 260	1 968	292	2
São Paulo das Missões	2 225	2 222	2 036	186	3
São Pedro da Serra	1 071	1 071	943	128	-
São Pedro das Missões	633	633	596	37	-
São Pedro do Butiá	919	919	864	55	-
São Pedro do Sul	6 435	6 429	5 691	738	6
São Sebastião do Caí	7 566	7 562	6 701	861	4
São Sepé	9 411	9 400	8 082	1 318	11
São Valentim	1 269	1 269	1 194	75	-
São Valentim do Sul	894	894	710	184	-
São Valério do Sul	765	765	736	29	-
São Vendelino	668	667	543	124	1
São Vicente do Sul	3 196	3 190	2 656	534	6
Sapiranga	25 317	25 308	23 316	1 992	9
Sapucaia do Sul	41 862	41 835	39 231	2 604	27
Sarandi	6 939	6 933	6 384	549	6
Seberi	3 894	3 886	3 571	315	8
Sede Nova	1 038	1 038	959	79	-
Segredo	2 193	2 193	2 069	124	-
Selbach	1 679	1 676	1 533	143	3
Senador Salgado Filho	979	979	926	53	-
Sentinela do Sul	2 127	2 127	1 771	356	-
Serafina Corrêa	4 322	4 317	4 077	240	5
Sério	852	851	785	66	1
Sertão	2 347	2 342	2 167	175	5
Sertão Santana	2 214	2 213	1 913	300	1
Sete de Setembro	731	730	687	43	1
Severiano de Almeida	1 395	1 393	1 229	164	2
Silveira Martins	934	932	798	134	2
Sinimbu	3 426	3 418	3 086	332	8
Sobradinho	4 965	4 961	4 600	361	4
Soledade	10 610	10 595	9 522	1 073	15
Tabaí	1 515	1 515	1 406	109	-
Tapejara	6 241	6 237	5 623	614	4
Tapera	3 642	3 639	3 359	280	3
Tapes	7 090	7 085	5 648	1 437	5
Taquara	20 343	20 309	17 310	2 999	34
Taquari	9 981	9 970	8 915	1 055	11

Tabela 2.1.23 - Domicílios recenseados, por espécie, segundo os municípios - Rio Grande do Sul - 2007

(conclusão)

Municípios	Domicílios recenseados, por espécie				
	Total	Particulares			Coletivos
		Total	Ocupados (1)	Não-ocupados	
Taquaruçu do Sul	923	922	837	85	1
Tavares	2 594	2 589	1 844	745	5
Tenente Portela	4 552	4 546	4 173	373	6
Terra de Areia	4 121	4 112	3 152	960	9
Teutônia	8 896	8 884	8 179	705	12
Tio Hugo	941	939	832	107	2
Tiradentes do Sul	2 444	2 443	2 251	192	1
Toropi	1 307	1 306	1 055	251	1
Torres	20 153	20 095	10 900	9 195	58
Tramandaí	31 925	31 794	12 761	19 033	131
Travesseiro	818	813	763	50	5
Três Arroios	1 038	1 036	925	111	2
Três Cachoeiras	3 531	3 528	3 310	218	3
Três Coroas	8 085	8 059	7 308	751	26
Três de Maio	8 361	8 353	7 800	553	8
Três Forquilhas	1 257	1 257	1 019	238	-
Três Palmeiras	1 501	1 500	1 376	124	1
Três Passos	8 641	8 632	8 084	548	9
Trindade do Sul	2 015	2 012	1 854	158	3
Triunfo	9 087	9 082	7 651	1 431	5
Tucunduva	2 110	2 109	1 945	164	1
Tunas	1 435	1 433	1 332	101	2
Tupanci do Sul	584	583	540	43	1
Tupanciretã	7 904	7 896	7 320	576	8
Tupandi	1 167	1 165	1 079	86	2
Tuparendi	3 170	3 169	2 966	203	1
Turuçu	1 170	1 168	1 063	105	2
Ubiretama	793	792	748	44	1
União da Serra	538	538	484	54	-
Unistalda	902	902	797	105	-
Uruguaiana	39 617	39 546	35 671	3 875	71
Vacaria	20 941	20 899	18 973	1 926	42
Vale do Sol	3 669	3 665	3 320	345	4
Vale Real	1 719	1 712	1 487	225	7
Vale Verde	1 389	1 389	1 108	281	-
Vanini	670	659	611	48	11
Venâncio Aires	23 859	23 836	21 527	2 309	23
Vera Cruz	8 123	8 118	7 316	802	5
Veranópolis	8 355	8 328	7 418	910	27
Vespasiano Correa	666	665	600	65	1
Viadutos	1 853	1 848	1 735	113	5
Vicente Dutra	2 067	2 066	1 720	346	1
Victor Graeff	1 114	1 114	1 035	79	-
Vila Flores	1 049	1 044	920	124	5
Vila Lângaro	731	731	684	47	-
Vila Maria	1 345	1 344	1 278	66	1
Vila Nova do Sul	1 661	1 657	1 467	190	4
Vista Alegre	921	921	858	63	-
Vista Alegre do Prata	518	517	447	70	1
Vista Gaúcha	873	873	858	15	-
Vitória das Missões	1 293	1 293	1 223	70	-
Westfalia	868	868	781	87	-
Xangri-lá	13 766	13 756	3 190	10 566	10

Fonte: IBGE, Contagem da População 2007.

(1) Inclusive os domicílios fechados com população estimada.

Tabela 2.1.24 - Domicílios recenseados, por espécie, segundo os municípios - Mato Grosso do Sul - 2007

(continua)

Municípios	Domicílios recenseados, por espécie				
	Total	Particulares			Coletivos
		Total	Ocupados (1)	Não-ocupados	
**Mato Grosso do Sul**	**783 947**	**782 494**	**680 431**	**102 063**	**1 453**
Água Clara	4 736	4 722	3 826	896	14
Alcinópolis	1 812	1 805	1 381	424	7
Amambaí	11 017	10 984	9 683	1 301	33
Anastácio	7 556	7 546	6 625	921	10
Anaurilândia	3 233	3 227	2 549	678	6
Angélica	2 818	2 810	2 303	507	8
Antônio João	2 782	2 775	2 417	358	7
Aparecida do Taboado	7 387	7 374	6 468	906	13
Aquidauana	15 913	15 877	13 105	2 772	36
Aral Moreira	2 509	2 505	2 327	178	4
Bandeirantes	2 373	2 371	1 900	471	2
Bataguassu	6 407	6 393	5 641	752	14
Batayporã	3 518	3 511	3 167	344	7
Bela Vista	7 320	7 298	6 394	904	22
Bodoquena	3 054	3 049	2 410	639	5
Bonito	6 111	6 059	5 119	940	52
Brasilândia	4 575	4 561	3 828	733	14
Caarapó	7 392	7 389	6 621	768	3
Camapuã	5 527	5 519	4 333	1 186	8
Campo Grande	255 846	255 623	224 374	31 249	223
Caracol	1 605	1 601	1 399	202	4
Cassilândia	8 300	8 283	7 033	1 250	17
Chapadão do Sul	5 844	5 804	4 965	839	40
Corguinho	2 016	2 003	1 349	654	13
Coronel Sapucaia	3 798	3 790	3 585	205	8
Corumbá	27 636	27 553	24 781	2 772	83
Costa Rica	6 896	6 859	5 779	1 080	37
Coxim	11 869	11 830	9 838	1 992	39
Deodápolis	4 137	4 132	3 490	642	5
Dois Irmãos do Buriti	3 400	3 394	2 682	712	6
Douradina	1 653	1 653	1 456	197	-
Dourados	62 208	62 151	54 472	7 679	57
Eldorado	4 036	4 030	3 646	384	6
Fátima do Sul	6 908	6 895	6 074	821	13
Figueirão	1 284	1 284	1 039	245	-
Glória de Dourados	3 568	3 562	3 108	454	6
Guia Lopes da Laguna	3 704	3 702	3 192	510	2
Iguatemi	5 239	5 224	4 383	841	15

Tabela 2.1.24 - Domicílios recenseados, por espécie, segundo os municípios - Mato Grosso do Sul - 2007

(conclusão)

Municípios	Domicílios recenseados, por espécie				
	Total	Particulares			Coletivos
		Total	Ocupados (1)	Não-ocupados	
Inocência	3 238	3 228	2 414	814	10
Itaporã	6 000	5 996	5 383	613	4
Itaquiraí	5 568	5 559	5 057	502	9
Ivinhema	7 431	7 416	6 494	922	15
Japorã	2 073	2 071	1 894	177	2
Jaraguari	2 573	2 568	1 851	717	5
Jardim	7 751	7 731	6 912	819	20
Jateí	1 475	1 471	1 169	302	4
Juti	2 007	2 005	1 638	367	2
Ladário	5 200	5 192	4 586	606	8
Laguna Carapã	2 043	2 043	1 669	374	-
Maracaju	9 783	9 771	8 757	1 014	12
Miranda	7 113	7 081	6 388	693	32
Mundo Novo	5 443	5 426	4 887	539	17
Naviraí	14 566	14 543	13 156	1 387	23
Nioaque	4 969	4 949	4 395	554	20
Nova Alvorada do Sul	4 181	4 165	3 603	562	16
Nova Andradina	15 173	15 147	13 351	1 796	26
Novo Horizonte do Sul	1 841	1 838	1 535	303	3
Paranaíba	14 928	14 912	12 655	2 257	16
Paranhos	3 039	3 038	2 694	344	1
Pedro Gomes	3 215	3 203	2 542	661	12
Ponta Porã	22 398	22 350	20 286	2 064	48
Porto Murtinho	4 194	4 161	3 812	349	33
Ribas do Rio Pardo	7 298	7 265	5 644	1 621	33
Rio Brilhante	8 296	8 285	7 675	610	11
Rio Negro	2 071	2 067	1 588	479	4
Rio Verde de Mato Grosso	7 292	7 273	5 816	1 457	19
Rochedo	1 946	1 945	1 418	527	1
Santa Rita do Pardo	3 175	3 167	2 404	763	8
São Gabriel do Oeste	7 347	7 289	6 299	990	58
Selvíria	2 433	2 428	1 941	487	5
Sete Quedas	3 354	3 349	2 995	354	5
Sidrolândia	12 635	12 624	11 368	1 256	11
Sonora	4 463	4 417	3 603	814	46
Tacuru	2 976	2 971	2 509	462	5
Taquarussu	1 087	1 086	999	87	1
Terenos	5 398	5 389	4 493	896	9
Três Lagoas	29 877	29 818	26 017	3 801	59
Vicentina	2 110	2 109	1 792	317	1

Fonte: IBGE, Contagem da População 2007.

(1) Inclusive os domicílios fechados com população estimada.

Tabela 2.1.25 - Domicílios recenseados, por espécie, segundo os municípios - Mato Grosso - 2007

(continua)

Municípios	Domicílios recenseados, por espécie				
	Total	Particulares			Coletivos
		Total	Ocupados (1)	Não-ocupados	
**Mato Grosso**	**941 157**	**937 987**	**820 047**	**117 940**	**3 170**
Acorizal	2 025	2 024	1 657	367	1
Água Boa	6 959	6 928	5 706	1 222	31
Alta Floresta	16 452	16 416	14 430	1 986	36
Alto Araguaia	5 078	5 042	4 353	689	36
Alto Boa Vista	1 774	1 760	1 344	416	14
Alto Garças	3 473	3 462	2 857	605	11
Alto Paraguai	2 960	2 959	2 440	519	1
Alto Taquari	2 401	2 339	1 802	537	62
Apiacás	2 577	2 566	2 309	257	11
Araguaiana	1 171	1 170	943	227	1
Araguainha	449	449	363	86	-
Araputanga	5 262	5 235	4 604	631	27
Arenápolis	3 574	3 566	2 977	589	8
Aripuanã	6 387	6 353	5 387	966	34
Barão de Melgaço	2 753	2 743	2 053	690	10
Barra do Bugres	9 566	9 493	8 699	794	73
Barra do Garças	19 655	19 557	16 309	3 248	98
Bom Jesus do Araguaia	1 893	1 881	1 428	453	12
Brasnorte	3 979	3 961	3 567	394	18
Cáceres	26 700	26 676	23 127	3 549	24
Campinápolis	3 437	3 413	2 882	531	24
Campo Novo do Parecis	7 141	7 005	6 236	769	136
Campo Verde	8 499	8 485	7 642	843	14
Campos de Júlio	1 547	1 515	1 397	118	32
Canabrava do Norte	1 735	1 697	1 546	151	38
Canarana	6 386	6 354	5 033	1 321	32
Carlinda	3 853	3 848	3 497	351	5
Castanheira	2 814	2 812	2 325	487	2
Chapada dos Guimarães	7 434	7 422	5 065	2 357	12
Cláudia	3 526	3 517	3 094	423	9
Cocalinho	2 615	2 586	1 817	769	29
Colíder	10 049	10 025	9 372	653	24
Colniza	9 106	9 086	7 533	1 553	20
Comodoro	6 064	6 039	4 768	1 271	25
Confresa	6 693	6 661	5 952	709	32
Conquista D'Oeste	1 025	1 023	909	114	2
Cotriguaçu	4 533	4 525	3 843	682	8
Cuiabá	166 122	165 795	150 577	15 218	327
Curvelândia	1 561	1 558	1 441	117	3
Denise	2 733	2 703	2 610	93	30
Diamantino	6 319	6 292	5 020	1 272	27
Dom Aquino	2 954	2 947	2 664	283	7
Feliz Natal	2 919	2 913	2 611	302	6
Figueirópolis D'Oeste	1 262	1 261	1 020	241	1
Gaúcha do Norte	1 932	1 922	1 434	488	10
General Carneiro	1 579	1 572	1 302	270	7
Glória D'Oeste	1 144	1 142	997	145	2
Guarantã do Norte	8 941	8 929	8 044	885	12
Guiratinga	5 036	5 023	4 314	709	13
Indiavaí	1 023	1 016	798	218	7
Ipiranga do Norte	1 778	1 773	1 248	525	5
Itanhangá	1 989	1 981	1 391	590	8
Itaúba	1 892	1 887	1 365	522	5

CENSOS2007

Tabela 2.1.25 - Domicílios recenseados, por espécie, segundo os municípios - Mato Grosso - 2007

(continuação)

Municípios	Domicílios recenseados, por espécie				
	Total	Particulares			Coletivos
		Total	Ocupados (1)	Não-ocupados	
Itiquira	4 626	4 550	3 778	772	76
Jaciara	8 202	8 162	7 428	734	40
Jangada	2 499	2 490	2 128	362	9
Jauru	3 424	3 412	3 056	356	12
Juara	10 664	10 610	9 296	1 314	54
Juína	12 916	12 888	11 153	1 735	28
Juruena	2 733	2 725	2 453	272	8
Juscimeira	4 036	4 034	3 645	389	2
Lambari D'Oeste	1 609	1 598	1 456	142	11
Lucas do Rio Verde	9 136	9 057	8 545	512	79
Luciára	784	784	677	107	-
Marcelândia	4 764	4 745	4 265	480	19
Matupá	4 310	4 288	3 923	365	22
Mirassol d'Oeste	8 179	8 172	7 505	667	7
Nobres	4 805	4 795	4 180	615	10
Nortelândia	2 274	2 268	1 855	413	6
Nossa Senhora do Livramento	4 101	4 098	3 524	574	3
Nova Bandeirantes	3 857	3 831	3 623	208	26
Nova Brasilândia	1 684	1 681	1 399	282	3
Nova Canaã do Norte	4 433	4 424	3 762	662	9
Nova Guarita	1 585	1 579	1 433	146	6
Nova Lacerda	1 780	1 760	1 445	315	20
Nova Marilândia	902	900	730	170	2
Nova Maringá	1 842	1 835	1 579	256	7
Nova Monte Verde	2 725	2 717	2 369	348	8
Nova Mutum	7 529	7 500	7 090	410	29
Nova Nazaré	788	784	630	154	4
Nova Olímpia	5 577	5 564	5 206	358	13
Nova Santa Helena	1 200	1 197	1 009	188	3
Nova Ubiratã	2 472	2 453	2 254	199	19
Nova Xavantina	7 342	7 317	5 999	1 318	25
Novo Horizonte do Norte	1 097	1 093	1 059	34	4
Novo Mundo	2 110	2 104	1 897	207	6
Novo Santo Antônio	759	754	621	133	5
Novo São Joaquim	2 789	2 772	2 217	555	17
Paranaíta	4 015	4 013	3 264	749	2
Paranatinga	6 681	6 672	5 632	1 040	9
Pedra Preta	5 437	5 425	4 764	661	12
Peixoto de Azevedo	9 618	9 594	7 806	1 788	24
Planalto da Serra	1 020	1 017	845	172	3
Poconé	9 990	9 932	8 201	1 731	58
Pontal do Araguaia	1 751	1 738	1 594	144	13
Ponte Branca	741	735	620	115	6
Pontes e Lacerda	12 688	12 678	11 161	1 517	10

Tabela 2.1.25 - Domicílios recenseados, por espécie, segundo os municípios - Mato Grosso - 2007

(conclusão)

Municípios	Domicílios recenseados, por espécie				
	Total	Particulares			Coletivos
		Total	Ocupados (1)	Não-ocupados	
Porto Alegre do Norte	3 286	3 269	2 620	649	17
Porto dos Gaúchos	2 047	2 042	1 770	272	5
Porto Esperidião	3 050	3 043	2 670	373	7
Porto Estrela	1 426	1 425	1 175	250	1
Poxoréo	6 459	6 443	5 487	956	16
Primavera do Leste	14 468	14 403	12 964	1 439	65
Querência	3 657	3 644	2 990	654	13
Reserva do Cabaçal	844	844	757	87	-
Ribeirão Cascalheira	3 138	3 116	2 460	656	22
Ribeirãozinho	874	869	681	188	5
Rio Branco	1 850	1 846	1 619	227	4
Rondolândia	1 090	1 088	917	171	2
Rondonópolis	60 339	60 224	51 843	8 381	115
Rosário Oeste	6 003	5 989	4 975	1 014	14
Salto do Céu	1 360	1 355	1 202	153	5
Santa Carmem	1 459	1 457	1 257	200	2
Santa Cruz do Xingu	882	882	635	247	-
Santa Rita do Trivelato	813	788	644	144	25
Santa Terezinha	2 238	2 236	1 884	352	2
Santo Afonso	1 056	1 055	941	114	1
Santo Antônio do Leste	1 164	1 048	930	118	116
Santo Antônio do Leverger	6 881	6 877	5 592	1 285	4
São Félix do Araguaia	3 639	3 612	3 026	586	27
São José do Povo	1 188	1 188	1 087	101	-
São José do Rio Claro	5 184	5 164	4 668	496	20
São José do Xingu	1 680	1 670	1 191	479	10
São José dos Quatro Marcos	6 879	6 870	5 919	951	9
São Pedro da Cipa	1 300	1 293	1 215	78	7
Sapezal	4 735	4 632	3 944	688	103
Serra Nova Dourada	488	487	425	62	1
Sinop	33 950	33 901	30 676	3 225	49
Sorriso	18 125	18 030	15 991	2 039	95
Tabaporã	3 624	3 604	2 915	689	20
Tangará da Serra	24 052	24 026	22 148	1 878	26
Tapurah	2 808	2 789	2 431	358	19
Terra Nova do Norte	4 207	4 204	3 798	406	3
Tesouro	1 437	1 426	1 007	419	11
Torixoréu	1 733	1 728	1 380	348	5
União do Sul	1 551	1 540	1 182	358	11
Vale de São Domingos	1 032	1 029	838	191	3
Várzea Grande	73 454	73 361	65 307	8 054	93
Vera	2 795	2 793	2 617	176	2
Vila Bela da Santíssima Trindade	4 603	4 572	3 673	899	31
Vila Rica	6 106	6 073	5 418	655	33

Fonte: IBGE, Contagem da População 2007.

(1) Inclusive os domicílios fechados com população estimada.

Tabela 2.1.26 - Domicílios recenseados, por espécie, segundo os municípios - Goiás - 2007

(continua)

Municípios	Domicílios recenseados, por espécie				
	Total	Particulares			Coletivos
		Total	Ocupados (1)	Não-ocupados	
**Goiás**	**1 213 067**	**1 210 609**	**1 036 501**	**174 108**	**2 458**
Abadia de Goiás	2 033	2 032	1 704	328	1
Abadiânia	4 851	4 818	3 845	973	33
Acreúna	6 839	6 832	5 555	1 277	7
Adelândia	927	923	813	110	4
Água Fria de Goiás	2 012	2 010	1 517	493	2
Água Limpa	992	992	734	258	-
Águas Lindas de Goiás	40 658	40 637	36 228	4 409	21
Alexânia	7 225	7 210	5 921	1 289	15
Aloândia	900	899	760	139	1
Alto Horizonte	1 123	1 122	972	150	1
Alto Paraíso de Goiás	2 648	2 583	1 971	612	65
Alvorada do Norte	2 780	2 774	2 323	451	6
Amaralina	1 251	1 251	1 119	132	-
Americano do Brasil	1 895	1 892	1 548	344	3
Amorinópolis	1 549	1 545	1 243	302	4
Anhanguera	421	420	337	83	1
Anicuns	6 134	6 129	5 622	507	5
Aparecida do Rio Doce	1 072	1 069	871	198	3
Aporé	1 519	1 515	1 159	356	4
Araçu	1 691	1 690	1 336	354	1
Aragarças	5 802	5 761	5 148	613	41
Aragoiânia	2 921	2 920	2 230	690	1
Araguapaz	2 926	2 922	2 407	515	4
Arenópolis	1 393	1 393	1 228	165	-
Aruanã	3 129	3 077	1 934	1 143	52
Aurilândia	1 652	1 647	1 363	284	5
Avelinópolis	938	938	801	137	-
Baliza	1 301	1 300	1 151	149	1
Barro Alto	2 216	2 208	1 912	296	8
Bela Vista de Goiás	8 417	8 407	6 664	1 743	10
Bom Jardim de Goiás	3 131	3 119	2 788	331	12
Bom Jesus de Goiás	6 781	6 748	6 148	600	33
Bonfinópolis	2 628	2 626	2 094	532	2
Bonópolis	1 167	1 165	981	184	2
Brazabrantes	1 285	1 285	1 016	269	-
Britânia	2 243	2 240	1 632	608	3
Buriti Alegre	4 039	4 034	2 905	1 129	5
Buriti de Goiás	1 052	1 051	789	262	1
Buritinópolis	1 049	1 049	919	130	-
Cabeceiras	2 317	2 313	1 924	389	4
Cachoeira Alta	3 072	3 067	2 748	319	5
Cachoeira de Goiás	557	557	492	65	-
Cachoeira Dourada	2 701	2 698	2 405	293	3
Caçu	4 402	4 376	3 767	609	26
Caiapônia	6 229	6 222	5 130	1 092	7
Caldas Novas	35 971	35 866	19 604	16 262	105
Caldazinha	1 169	1 169	1 018	151	-
Campestre de Goiás	1 138	1 136	1 041	95	2

Tabela 2.1.26 - Domicílios recenseados, por espécie, segundo os municípios - Goiás - 2007

(continuação)

Municípios	Domicílios recenseados, por espécie				
	Total	Particulares			Coletivos
		Total	Ocupados (1)	Não-ocupados	
Campinaçu	1 441	1 437	1 171	266	4
Campinorte	3 100	3 099	2 891	208	1
Campo Alegre de Goiás	2 250	2 238	1 802	436	12
Campo Limpo de Goiás	2 043	2 035	1 674	361	8
Campos Belos	5 896	5 880	5 028	852	16
Campos Verdes	2 421	2 412	1 966	446	9
Carmo do Rio Verde	3 469	3 465	2 934	531	4
Castelândia	1 394	1 389	1 078	311	5
Catalão	26 470	26 421	23 723	2 698	49
Caturaí	1 886	1 886	1 470	416	-
Cavalcante	3 531	3 527	2 668	859	4
Ceres	6 920	6 907	5 867	1 040	13
Cezarina	2 743	2 732	2 296	436	11
Chapadão do Céu	1 834	1 822	1 590	232	12
Cidade Ocidental	15 083	15 069	13 526	1 543	14
Cocalzinho de Goiás	5 004	5 002	4 252	750	2
Colinas do Sul	1 471	1 467	1 113	354	4
Córrego do Ouro	1 036	1 034	918	116	2
Corumbá de Goiás	3 260	3 250	2 729	521	10
Corumbaíba	2 969	2 959	2 650	309	10
Cristalina	11 576	11 543	10 249	1 294	33
Cristianópolis	1 342	1 336	1 018	318	6
Crixás	5 098	5 081	4 425	656	17
Cromínia	1 498	1 497	1 224	273	1
Cumari	1 390	1 384	1 030	354	6
Damianópolis	1 324	1 313	1 014	299	11
Damolândia	1 074	1 071	911	160	3
Davinópolis	920	916	718	198	4
Diorama	944	943	765	178	1
Divinópolis de Goiás	2 036	2 032	1 649	383	4
Doverlândia	3 426	3 416	2 668	748	10
Edealina	1 704	1 700	1 306	394	4
Edéia	3 560	3 546	3 255	291	14
Estrela do Norte	1 303	1 300	1 022	278	3
Faina	2 537	2 535	2 265	270	2
Fazenda Nova	2 640	2 638	2 141	497	2
Firminópolis	3 708	3 699	3 341	358	9
Flores de Goiás	3 431	3 418	2 891	527	13
Formosa	28 606	28 563	25 529	3 034	43
Formoso	2 065	2 060	1 702	358	5
Gameleira de Goiás	1 241	1 241	1 060	181	-
Goianápolis	3 853	3 851	3 308	543	2
Goiandira	2 029	2 023	1 651	372	6
Goianésia	17 857	17 836	16 412	1 424	21
Goianira	8 084	8 077	7 330	747	7
Goiás	10 405	10 374	7 944	2 430	31
Goiatuba	11 903	11 883	10 265	1 618	20

Tabela 2.1.26 - Domicílios recenseados, por espécie, segundo os municípios - Goiás - 2007

(continuação)

Municípios	Domicílios recenseados, por espécie				
	Total	Particulares			Coletivos
		Total	Ocupados (1)	Não-ocupados	
Gouvelândia	1 649	1 648	1 410	238	1
Guapó	4 903	4 895	4 293	602	8
Guaraíta	861	860	807	53	1
Guarani de Goiás	1 451	1 450	1 145	305	1
Guarinos	928	927	827	100	1
Heitoraí	1 382	1 382	1 141	241	-
Hidrolândia	5 113	5 104	4 229	875	9
Hidrolina	1 601	1 599	1 349	250	2
Iaciara	3 809	3 803	3 386	417	6
Inaciolândia	2 100	2 097	1 801	296	3
Indiara	4 291	4 284	3 943	341	7
Inhumas	14 610	14 597	13 602	995	13
Ipameri	9 069	9 015	7 389	1 626	54
Ipiranga de Goiás	1 176	1 175	966	209	1
Iporá	11 925	11 897	10 480	1 417	28
Israelândia	1 178	1 175	965	210	3
Itaberaí	10 029	10 015	9 409	606	14
Itaguari	1 648	1 646	1 476	170	2
Itaguaru	2 047	2 046	1 852	194	1
Itajá	2 134	2 128	1 776	352	6
Itapaci	5 692	5 678	4 847	831	14
Itapirapuã	3 586	3 583	2 789	794	3
Itapuranga	9 296	9 281	8 211	1 070	15
Itarumã	2 239	2 225	1 722	503	14
Itauçu	2 984	2 981	2 751	230	3
Itumbiara	31 516	31 484	28 311	3 173	32
Ivolândia	1 141	1 138	941	197	3
Jandaia	2 671	2 667	2 217	450	4
Jaraguá	13 326	13 307	11 700	1 607	19
Jataí	28 450	28 400	25 039	3 361	50
Jaupaci	1 220	1 219	1 000	219	1
Jesúpolis	766	766	683	83	-
Joviânia	2 485	2 463	2 225	238	22
Jussara	7 384	7 369	6 157	1 212	15
Lagoa Santa	574	563	392	171	11
Leopoldo de Bulhões	3 431	3 431	2 686	745	-
Mairipotaba	1 076	1 074	937	137	2
Mambaí	2 116	2 115	1 724	391	1
Mara Rosa	3 810	3 803	3 315	488	7
Marzagão	825	824	681	143	1
Matrinchã	1 702	1 697	1 390	307	5
Maurilândia	3 278	3 234	3 000	234	44
Mimoso de Goiás	1 110	1 108	870	238	2
Minaçu	10 543	10 527	9 313	1 214	16
Mineiros	15 406	15 367	13 275	2 092	39
Moiporá	764	763	659	104	1
Monte Alegre de Goiás	2 745	2 736	2 030	706	9

Tabela 2.1.26 - Domicílios recenseados, por espécie, segundo os municípios - Goiás - 2007

(continuação)

Municípios	Domicílios recenseados, por espécie				
	Total	Particulares			Coletivos
		Total	Ocupados (1)	Não-ocupados	
Montes Claros de Goiás	3 043	3 040	2 604	436	3
Montividiu	3 320	3 302	2 820	482	18
Montividiu do Norte	1 571	1 567	1 379	188	4
Morrinhos	14 879	14 859	13 072	1 787	20
Morro Agudo de Goiás	923	922	815	107	1
Mossâmedes	2 095	2 091	1 645	446	4
Mozarlândia	4 137	4 129	3 750	379	8
Mundo Novo	2 657	2 649	2 137	512	8
Mutunópolis	1 352	1 351	1 222	129	1
Nazário	2 831	2 827	2 452	375	4
Nerópolis	6 101	6 095	5 612	483	6
Niquelândia	13 530	13 482	11 408	2 074	48
Nova América	889	887	736	151	2
Nova Aurora	886	884	701	183	2
Nova Crixás	5 866	5 840	3 803	2 037	26
Nova Glória	3 387	3 384	2 847	537	3
Nova Iguaçu de Goiás	1 000	994	838	156	6
Nova Roma	1 467	1 465	1 076	389	2
Nova Veneza	2 425	2 424	2 075	349	1
Novo Brasil	1 558	1 553	1 266	287	5
Novo Gama	24 931	24 921	22 673	2 248	10
Novo Planalto	1 460	1 460	1 307	153	-
Orizona	5 535	5 527	4 613	914	8
Ouro Verde de Goiás	1 591	1 590	1 385	205	1
Ouvidor	1 969	1 968	1 569	399	1
Padre Bernardo	8 895	8 877	7 372	1 505	18
Palestina de Goiás	1 210	1 207	1 034	173	3
Palmeiras de Goiás	7 420	7 403	6 758	645	17
Palmelo	948	934	761	173	14
Palminópolis	1 577	1 573	1 250	323	4
Panamá	1 020	1 018	896	122	2
Paranaiguara	3 232	3 222	2 643	579	10
Paraúna	4 181	4 158	3 458	700	23
Perolândia	915	915	796	119	-
Petrolina de Goiás	3 797	3 793	3 169	624	4
Pilar de Goiás	1 104	1 100	853	247	4
Piracanjuba	9 613	9 592	7 889	1 703	21
Piranhas	4 386	4 379	3 793	586	7
Pirenópolis	7 441	7 391	6 188	1 203	50
Pires do Rio	9 683	9 667	8 527	1 140	16
Planaltina	22 876	22 860	20 772	2 088	16
Pontalina	6 171	6 160	5 503	657	11
Porangatu	13 998	13 967	12 104	1 863	31
Porteirão	1 016	1 006	920	86	10
Portelândia	1 255	1 254	1 017	237	1
Posse	8 894	8 861	7 753	1 108	33
Professor Jamil	1 320	1 314	1 043	271	6
Quirinópolis	13 195	13 178	11 995	1 183	17

Tabela 2.1.26 - Domicílios recenseados, por espécie, segundo os municípios - Goiás - 2007

(conclusão)

Municípios	Domicílios recenseados, por espécie				
	Total	Particulares			Coletivos
		Total	Ocupados (1)	Não-ocupados	
Rialma	3 600	3 594	3 257	337	6
Rianápolis	1 471	1 469	1 289	180	2
Rio Quente	2 853	2 829	919	1 910	24
Rio Verde	51 079	51 001	45 060	5 941	78
Rubiataba	6 711	6 692	5 805	887	19
Sanclerlândia	2 987	2 983	2 594	389	4
Santa Bárbara de Goiás	1 790	1 789	1 702	87	1
Santa Cruz de Goiás	1 631	1 628	1 176	452	3
Santa Fé de Goiás	1 535	1 534	1 383	151	1
Santa Helena de Goiás	11 668	11 648	10 796	852	20
Santa Isabel	1 446	1 446	1 195	251	-
Santa Rita do Araguaia	2 291	2 290	1 866	424	1
Santa Rita do Novo Destino	1 115	1 115	987	128	-
Santa Rosa de Goiás	1 209	1 206	969	237	3
Santa Tereza de Goiás	1 647	1 643	1 385	258	4
Santa Terezinha de Goiás	4 081	4 070	3 396	674	11
Santo Antônio da Barra	1 453	1 452	1 285	167	1
Santo Antônio de Goiás	1 224	1 224	1 111	113	-
Santo Antônio do Descoberto	16 952	16 933	14 989	1 944	19
São Domingos	3 384	3 375	2 798	577	9
São Francisco de Goiás	2 252	2 245	1 854	391	7
São João da Paraúna	716	714	605	109	2
São João d'Aliança	3 062	3 051	2 431	620	11
São Luís de Montes Belos	10 447	10 430	8 830	1 600	17
São Luíz do Norte	1 541	1 538	1 301	237	3
São Miguel do Araguaia	8 461	8 426	7 074	1 352	35
São Miguel do Passa Quatro	1 399	1 395	1 193	202	4
São Patrício	869	868	671	197	1
São Simão	5 122	5 108	4 521	587	14
Senador Canedo	21 256	21 250	19 537	1 713	6
Serranópolis	2 676	2 658	2 245	413	18
Silvânia	7 335	7 319	5 699	1 620	16
Simolândia	2 253	2 244	1 903	341	9
Sítio d'Abadia	1 077	1 076	903	173	1
Taquaral de Goiás	1 360	1 359	1 225	134	1
Teresina de Goiás	949	945	809	136	4
Terezópolis de Goiás	1 982	1 981	1 616	365	1
Três Ranchos	1 506	1 502	910	592	4
Trindade	31 510	31 472	28 527	2 945	38
Trombas	1 306	1 305	1 134	171	1
Turvânia	1 995	1 990	1 688	302	5
Turvelândia	1 256	1 254	1 101	153	2
Uirapuru	1 085	1 084	946	138	1
Uruaçu	12 464	12 445	10 546	1 899	19
Uruana	4 951	4 944	4 474	470	7
Urutaí	1 309	1 303	932	371	6
Valparaíso de Goiás	36 014	36 004	32 657	3 347	10
Varjão	1 364	1 363	1 249	114	1
Vianópolis	4 351	4 339	3 781	558	12
Vicentinópolis	2 060	2 053	1 859	194	7
Vila Boa	1 619	1 615	1 136	479	4
Vila Propício	1 882	1 878	1 516	362	4

Fonte: IBGE, Contagem da População 2007.

(1) Inclusive os domicílios fechados com população estimada.

Referências

II CONTEO de población y vivenda 2005. Aspectos metodológicos. Así hicimos el Conteo. Aguascalientes, Mx: INEGI, [2007]. Disponível em: <http://www.inegi.gob.mx/est/contenidos/espanol/proyectos/conteos/conteo2005>. Acesso em: nov. 2007.

ALBIERI, S. *A ausência de resposta em pesquisas*: uma aplicação de métodos de imputação. 1989. 138 p. Dissertação (Mestrado)-Instituto de Matemática Pura e Aplicada, Rio de Janeiro, 1989.

GARCIA-RUBIO, E.; VILLÁN-CRIADO, I. S*istema DIA: sistema de detección e imputación automática de errores para datos cualitativos*. Madrid: INE, 1988. v. 1: DIA: Descripción del sistema.

PESSOA, D. G. C.; MOREIRA, G. G.; SANTOS, A. R. *Imputação de rendimentos no questionário da amostra do censo demográfico 2000*. Rio de Janeiro: IBGE, Diretoria de Pesquisas, 2003. 17 p. Relatório Técnico.

PESSOA, D. G. C.; SANTOS, A. R. *Imputação de rendimento dos responsáveis por domicílios – conjunto universo do censo demográfico 2000*. Rio de Janeiro: IBGE, Diretoria de Pesquisas, 2003. 15 p. Relatório Técnico.

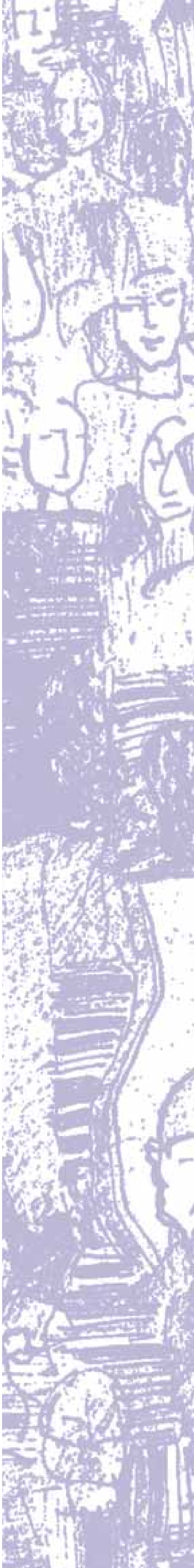

Anexos

Anexo 1 - Relação dos 128 municípios e do Distrito Federal, por Unidade da Federação, cujas populações foram estimadas para a data de referência de 1º de abril de 2007

Anexo 2 - Metodologia de estimação do número de moradores em domicílios fechados

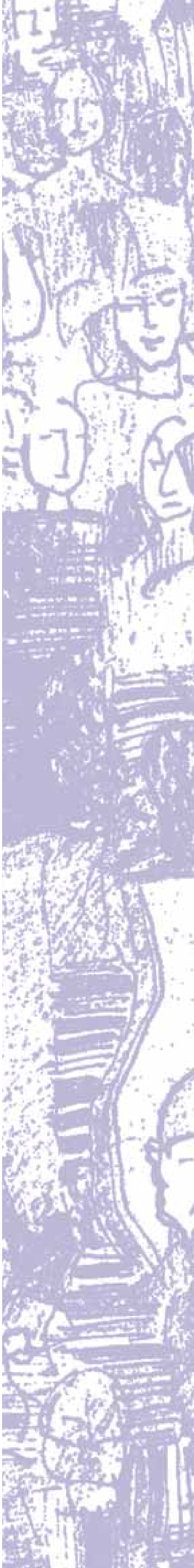

Anexo 1 – Relação dos 128 municípios e do Distrito Federal, por Unidades da Federação, cujas populações foram estimadas para a data de referência de 1º de abril de 2007

Pará: Ananindeua, Belém, Marabá e Santarém.

Ceará: Caucaia, Fortaleza, Juazeiro do Norte, Maracanaú e Sobral.

Pernambuco: Caruaru, Jaboatão dos Guararapes, Olinda, Paulista, Petrolina e Recife.

Bahia: Camaçari, Feira de Santana, Ilhéus, Itabuna, Juazeiro, Salvador e Vitória da Conquista.

Minas Gerais: Belo Horizonte, Betim, Contagem, Divinópolis, Governador Valadares, Ipatinga, Juiz de Fora, Montes Claros, Ribeirão das Neves, Santa Luzia, Sete Lagoas, Uberaba e Uberlândia.

Espírito Santo: Cachoeiro de Itapemirim, Cariacica, Serra, Vila Velha e Vitória.

Rio de Janeiro: Barra Mansa, Belford Roxo, Campos dos Goytacazes, Duque de Caxias, Itaboraí, Magé, Mesquita, Niterói, Nova Friburgo, Nova Iguaçu, Petrópolis, Rio de Janeiro, São Gonçalo, São João de Meriti e Volta Redonda.

São Paulo: Americana, Araçatuba, Araraquara, Barueri, Bauru, Campinas, Carapicuíba, Cotia, Diadema, Embu, Ferraz de Vasconcelos, Franca, Guarujá, Guarulhos, Hortolândia, Indaiatuba, Itapevi, Itaquaquecetuba, Jacareí, Jundiaí, Limeira, Marília, Mauá, Mogi das Cruzes, Osasco, Piracicaba, Praia Grande, Presidente Prudente, Ribeirão Preto, Rio Claro, Santa Bárbara d'Oeste, Santo André, Santos, São Bernardo do Campo, São Carlos, São José do Rio Preto, São José dos Campos, São Paulo, São Vicente, Sorocaba, Sumaré, Suzano, Taboão da Serra e Taubaté.

Paraná: Cascavel, Colombo, Curitiba, Foz do Iguaçu, Londrina, Maringá, Ponta Grossa e São José dos Pinhais.

Santa Catarina: Blumenau, Criciúma, Florianópolis, Joinville e São José.

Rio Grande do Sul: Alvorada, Canoas, Caxias do Sul, Gravataí, Novo Hamburgo, Passo Fundo, Pelotas, Porto Alegre, Rio Grande, Santa Maria, São Leopoldo e Viamão.

Goiás: Anápolis, Aparecida de Goiânia, Goiânia e Luziânia.

Distrito Federal: Brasília

Anexo 2 - Metodologia de estimação do número de moradores em domicílios fechados

Motivação

As unidades domiciliares pesquisadas nos Censos Demográficos e na Contagem da População são classificadas em categorias de acordo com a situação de seus moradores na data de referência da coleta, a saber: domicílios ocupados (particulares ou coletivos); domicílios fechados; domicílios vagos; e domicílios de uso ocasional. A operação censitária visa a obter informações das pessoas moradoras nos domicílios classificados nas duas primeiras categorias (domicílios ocupados e domicílios fechados), uma vez que os possíveis moradores em domicílios de uso ocasional possuem outro domicílio de moradia permanente objeto da investigação.

Os domicílios classificados como fechados são aqueles que sabidamente possuíam moradores na data de referência, mas que não tiveram entrevista realizada para o preenchimento das informações do questionário, independentemente do motivo da não realização da entrevista, que pode ser tanto uma recusa do morador em prestar informações como uma dificuldade do entrevistador em estabelecer contato com o informante (ou seja, a ausência de pessoas no domicílio nos momentos das visitas do entrevistador).

Nas divulgações de resultados de Censos Demográficos, os totais da população para cada um dos municípios brasileiros foram sempre divulgados considerando os domicílios ocupados (particulares e coletivos) na data de referência da operação censitária. As quantidades de domicílios de cada uma das categorias foram divulgadas sob a forma de Sinopse Preliminar juntamente com resultados da população recenseada.

As informações sobre o número de domicílios fechados, vagos e de uso ocasional são usadas, juntamente com outras informações disponíveis, para a avaliação da qualidade da cobertura das operações censitárias e, neste sentido, elas contribuem indiretamente para os procedimentos de avaliação das estimativas municipais de população.

Diferentemente de anos anteriores, a Contagem da População 2007 não foi realizada em todos os municípios brasileiros e um de seus objetivos foi subsidiar a elaboração das estimativas municipais de população acima citadas. Por esse motivo, com o objetivo de aperfeiçoar as estimativas municipais para o ano 2007, o IBGE julgou conveniente estimar a parcela da população moradora nos domicílios fechados em cada um dos municípios abrangidos pela operação da Contagem da População 2007.

Metodologia

No caso da estimação do número de moradores nos domicílios fechados da Contagem de População 2007, admitiu-se que o padrão dos domicílios fechados é diferente do padrão dos domicílios ocupados, que foram efetivamente investigados, no que se refere ao tamanho do domicílio. Ou seja, admitiu-se que os domicílios fechados possuem uma característica em sua composição, principalmente no número de moradores, que impli-

cou na dificuldade do entrevistador para realizar a entrevista ou na recusa do informante, e na sua classificação como fechado, após o término do período de coleta.

Para avaliar essa hipótese, para cada Unidade da Federação, foram obtidas as distribuições do número de domicílios particulares ocupados por tamanho de domicílio em dois conjuntos de entrevistas realizadas, a saber: (1) domicílios particulares ocupados que tiveram entrevista realizada durante o período inicialmente definido para a operação de coleta de dados; (2) domicílios classificados como fechados nesse mesmo período e que tiveram entrevista realizada após o encerramento desse período, ou seja, domicílios abertos e entrevistados após a prorrogação do período de coleta inicialmente definido. Em cada Unidade da Federação, a análise das duas distribuições confirmou a hipótese, apontando uma maior quantidade de domicílios pequenos, com um ou dois moradores, dentre os domicílios considerados fechados do que naqueles entrevistados durante o período normal.

A classificação de um domicílio na categoria de fechado é equivalente a considerá-lo como uma não-resposta, que é um dos erros não-amostrais mais comuns na realização de uma pesquisa, seja ela censitária ou por amostragem. Há muitas formas diferentes de se lidar com a não-resposta. Uma delas é a que utiliza procedimentos de imputação. Procedimento de imputação é aquele que atribui informações individuais às unidades sem informação. O pressuposto básico do procedimento de imputação é que a perda de dados seja aleatória, e se não for, que o padrão de não-resposta seja conhecido ou pelo menos estimado, para ser considerado durante o tratamento da não-resposta por imputação.

Para estimar o número de moradores em domicílios fechados para cada município abrangido pela Contagem da População 2007, definiu-se cada domicílio fechado como uma não-resposta cujo atributo necessário é o número de moradores. O tratamento adotado para essa não-resposta foi um procedimento de imputação por meio de seleção aleatória de um domicílio doador entre um conjunto de possíveis doadores, considerando a distribuição do número de domicílios particulares ocupados por tamanho em número de moradores definida no conjunto de fechados tal como explicado no conjunto (2).

Em termos operacionais, o procedimento consistiu em imputar, para cada município, tantos domicílios quantos os classificados como fechados, com número de moradores de acordo com a distribuição obtida pelo conjunto de domicílios fechados da Unidade da Federação correspondente.

Assim, o total de moradores estimados no conjunto de domicílios fechados de cada município foi obtido pela soma dos moradores nos domicílios imputados.

O procedimento de estimação foi aplicado aos domicílios efetivamente fechados, após todas as tentativas de obtenção da entrevista, que correspondem a 0,9% do total de domicílios ocupados nos municípios abrangidos pela Contagem da População 2007.

Equipe técnica

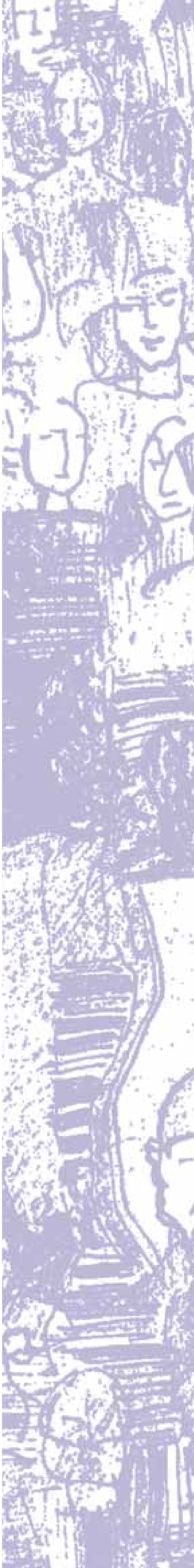

Comissão de Planejamento e Organização Geral

Presidente: Eduardo Pereira Nunes

Secretaria-executiva: Maria Vilma Salles Garcia

Membros
Alicia Bercovich
Antonio Carlos Simões Florido
Ataíde José de Oliveira Venâncio
David Wu Tai
Franklin Moreira de Almeida
Guido Gelli
Heleno Ferreira Mansoldo
Luiz Fernando Pinto Mariano
Luiz Paulo Souto Fortes
Marco Antonio dos Santos Alexandre
Moema José de Carvalho Augusto
Paulo César Martins
Pedro Luis do Nascimento Silva
Rodolpho Alves Simas
Rose Maria Barros de Almeida
Sandra Furtado de Oliveira
Sérgio da Costa Côrtes
Wasmália Socorro Barata Bivar
Wolney Cogoy de Menezes

Coordenação de Acompanhamento e Controle Operacional dos Censos

Coordenadora: Maria Vilma Salles Garcia

Gerentes e Consultor
Elson dos Santos Mattos – Consultor
Germano Augusto Zulchner Gonçalves de Andrade
Laura Baridó Indá
Marfisa Maria Teixeira Guimarães
Maria Angélica Vasconcelos de Araújo
Wolney Cogoy de Menezes

Técnicos
Ana Maria de Oliveira Silva
Artur de Freitas Pires Neto
Edmundo Maldes Contar
Fábio Sciberras de Carvalho
Gilberto Macedo Pina
Márcia Regina Alonso de Oliveira
Maria Áurea Carmo Canedo Medeiros
Maria Salete da Silva se Souza
Nádia Regina Paiva de Souza
Nélio Ferreira Machado
Sandra Fidalgo Zettel
Wilson da Costa Leite

Apoio Administrativo
Célia de Lacerda Gil
Patrícia Lobo Figueiredo
Rosani Vicente da Silva

Diretoria de Pesquisas

Assessora: Zélia Magalhães Bianchini

Assistente: Terezinha Batista Coutinho

Gerência Técnica do Censo Demográfico

Coordenador: Marco Antonio dos Santos Alexandre

Gerentes
Cleber Felix
Eneiza de Andrade Silva
Geraldo José Polidoro
Luís Carlos de Souza Oliveira
Maria de Fátima Lobo Augusto
Mauro Sorge
Ricardo Luiz Cardoso

Técnicos
Albina Ferreira da Silva
Aureir Faria José de Oliveira
Carlos José da Fonseca Caride
Claudia Maria Ferreira Nascimento

Diuzamar Francisca dos Santos
Edie da Silva de Mattos
Francisco Nelson Pereira do Prado
Isis Gertrudes dos Santos
João José Amado Ramalho Júnior
Joceilma Oliveira Fernandes
José Ademir Campos de Carvalho
José Ângelo Goulart Gil
Juarez Vicente Vieira
Lilian Rose Rabello Ribas
Luciano Tavares Duarte
Luis Carlos Rodrigues
Márcia Luzia Coenca Maia
Márcia Regina Martins Lima Dias
Maria Aparecida Juliano de Aguiar
Mário Luiz Carelli
Nelson Cardoso Osório Neto
Oswaldo Francisco de Luca
Roberto Miranda Nogueira
Rogerio Araujo da Silva
Romeu Ferreira Emygdio
Sidney da Silva Alves
Wanderson Suzart da Costa

Apoio administrativo
Lenilda Lima de Castro
Marco Antônio dos Santos Xavier
Paulo César Ferreira Brasil
Paulo Roberto da Silva
Paulo Roberto Gomes dos Santos
Walter Fonseca Filho

Gerência Técnica do Censo Agropecuário

Coordenador: Antônio Carlos Simões Florido

Gerente
Luiz Fernando Pereira Rodrigues

Técnicos
Aída Maria Pinto de Sá Barreto
Ana Lúcia da Silva de Almeida
Carlos Alberto Barreto Rodrigues
Fernanda Cíntia Pires e Teixeira
Flávio Barreto de Abreu
Jorge Antônio Rodrigues
José Carlos Jesus de Oliveira
Luiz Scherer de Paula Xavier Junior
Marcos Thanus Nunes Andrade
Marcos Zurita Fernandes
Maria Anita Evangelista de Oliveira
Paulo Ricardo de Brito Soares
Rafael Kessler Fernandez
Regina Célia Alves de Araújo
Roberto Ricardo Gomes de Carvalho
Roberto Silva Ramos
Rosamaria Silva Moreira
Sandra Passos Chrisóstomo
Sérgio Deleage Ferreira
Sônia Regina Madeira
Vilma de Oliveira Campos Peixoto
Wanderci Lopes da Silva

Comitê do Censo Demográfico

Coordenadora: Alicia Bercovich

Gerentes
Cezar Cioffi Camardella
Eliane Aparecida de Araújo Xavier
Jacqueline dos Santos Manhães

Técnicos
Andréa da Silva Borges
Rodrigo Aires Lemes
Rosangela Filhote Ferreira

Coordenação de Métodos e Qualidade

Coordenadora: Sonia Albieri

Gerentes e Consultores
Antônio José Ribeiro Dias
Ari do Nascimento Silva - Consultor
Djalma Galvão Carneiro Pessoa - Consultor
Luiz Alberto Matzenbacher - Consultor

Técnicos
Alexandre dos Reis Santos
Mauricio Franca Lila

Coordenação de População e Indicadores Sociais

Coordenador: Luiz Antonio Pinto de Oliveira

Gerentes
Fernando Roberto Pires de Albuquerque
Ivan Braga Lins
Juarez de Castro Oliveira
Leila Regina Ervatti
Maria Isabel Fernandes Mendes
Nadja Loureiro Pernes da Silva

Técnicos
Antonio Roberto Pereira Garcez
Carlos Alberto Maia
Fátima Honorata Brandão Prates
Rosane Teixeira de Siqueira e Oliveira

Apoio administrativo
Jerinha Mariano Souza
Fátima Aude Fernandes Fortes
Sheila Gil dos Santos Magno

Diretoria de Geociências

Assessor: Luiz Paulo Souto Fortes

Coordenação do Censo

Coordenador: Rodolpho Alves Simas

Técnicos
Adalberto Dutra Cardoso
Afonso Carlos Paixão
Alcir da Costa Ribeiro
Amauri da Silva
Dario Bazilio Theodoro Filho
Denise Santos Rodrigues
Elben Pinto dos Santos
Milton Bastos Andrade Junior
Monica Malaquias de Campos
Nelson Rodrigues Vasques
Vilma Vicente de Paula Souza

Coordenação de Estruturas Territoriais

Coordenador: Paulo Cesar Martins

Gerentes
Angela Maria de Souza Ferreira
Carmen Zagari Machado
Cláudio Maia Peres
José Henrique da Silva

Técnicos
Carlos Alberto Elbert Queiroz
Carlos Augusto dos Santos
Cláudio Cabral da Silva
Ricardo Carneiro Teixeira

Coordenação de Cartografia

Coordenadora: Moema José de Carvalho Augusto

Gerentes
Dulce Santoro Mendes
Edison Pereira Ribeiro
Miriam Mattos da Silva Barbuda

Técnicos
Alexandre José Almeida Teixeira
Aline Santos Paes
Francisca Eugenia Soares Dias
Irenil Leocádio da Conceição
Júlio Cesar Feliciano Vieira
Levy Lopes Furtado
Monica Fontes
Paulo Roberto de Oliveira
Silvio de Paula e Silva Filho
Solange Soares de Mello
Sonia Maria Ribeiro da Silva
Tiago Ferreira de Queroz
Thiago Medina da Silva
Viviane Barbosa Diniz
Wesley Silva Fernandes

Coordenação de Geografia

Coordenadora: Maria Luisa Gomes Castello Branco

Gerentes
Adma Hamam de Figueiredo
Claudio Stenner

Técnicos
Cleber de Azevedo Fernandes
Denise Maria Penna Kronemberger Dantas
Evangelina Xavier Gouveia de Oliveira
Ivete Oliveira Rodrigues
José Carlos Louzada Morelli
Luís Sérgio Pires Guimarães
Rogério Botelho de Mattos
Vera Maria d'Ávila Cavalcanti

Coordenação de Recursos Naturais e Estudos Ambientais

Coordenador: Celso José Monteiro Filho

Técnicos
Eloisa Domingues
Elpídio Antonio Venturini de Freitas
Ione Vieira Rabelo da Cunha
José Aldo Gonçalves Coutinho
Judicael Clevelario Junior
Paula Terezina Tudesco Macedo de Oliveira
Rosa Luzia Saísse Brum
Valdir Neves
Valéria Grace Costa

Diretoria de Informática

Assessor: Paulo Cesar Moraes Simões

Coordenação Operacional de Informática

Coordenador: Heleno Ferreira Mansoldo

Gerência de Censo

Gerente: Ataide José de Oliveira Venâncio

Técnicos
Antônio José de Oliveira
Cássia Rezende de Pinho
Davi Faria Rocha
Edson Orofino de Souza
Isa Maria Mendonça Bastos
Marcos Barros Leite
Norberto Contardo Silvino Pereira
Rames Chhangalal

Coordenação de Serviços de Informática

Coordenador: Sérgio Baía Ferreira

Gerentes
Ecio Tadeu Moraes Pedro
Marcus Vinicius Morgado Nogueira
Administrativo
Líria Cristina de Souza Machado
Madeleine Louise Menezes Ferreira
Maria Regina Pinto Mariano
Mônica Maria Rocha Vieira

Centro de Captura de Dados

Coordenador Geral: Celso Sampaio da Silva

Gerentes
Eduardo da Costa Romero
Enio Schiavo
Maria da Penha Ferreira da Silva
Ricardo Luiz Silva Maciel
Sérgio Botelho Ferreira
Sergio Luiz de Pinho Barbosa
Valci Furtado da Silva

Técnicos
Diógenes Vieira Lima
Jorge Fernando de Oliveira

Gerência de Administração dos Serviços de Produção

Gerente: Marcio Tavares Fernandes

Gerentes
Fernando Espirito Santo Cataldo
Jorge Nélson Lopes da Cunha
Roberto de Andrade França Junior
Ronaldo Mereson Wittitz

Técnicos
Andréa Moreira Torres
Bruno Gonçalves Santos
Carlos Brandão Fernandes da Silva
Flávio Marcellus Massiotti
Geórgia de Souza Assumpção
Osmar Alves Araújo
Osni Alves Barroso
Paulo Lincoln Ribeiro de Oliveira

Gerência de Tecnologia e Suporte em Redes de Comunicação de Dados e Sistemas Abertos

Gerente: Robson Rodrigues Vaz

Gerentes
Alberto Luiz Gonçalves Perez
Ângela Patrício de Lima
Cláudia de Almeida Nogueira Gonçalves
Elias Pereira Lima
Leila de Assis Barbosa Costa
Mário Luiz Nunes Souto
Martha de Mattos Seixas
Nílson Carlos de Magalhães Pontes
Sylvio Romero Bicalho Barbosa

Técnicos
Angélica da Costa Pedreira
Daniela de Sousa Sant´ana
Lúcia de Fátima Santos Castro
Márcio Pereira Cardoso
Marlúcia Moraes Moreira
Paulo Jorge Maia Prata
Rodney Loyola Monte da Silva
Sônia Vasques Nogueira

Gerência de Administração do Sistema Central de Processamento

Gerente: Maria Luiza Duarte Pinto Henning

Gerentes
Joaquim Romualdo Teixeira
Luiz Carlos de Castro Neves
Luiz Fernando de França Carvalho
Maria Helena Stefano Ferreira
Ronaldo Pinheiro Ferrari

Técnicos
André Luis da Mata
Carlos Eduardo Manhães Martins
Celso Barbosa dos Santos
Ezer Bianchi
Hélio Pinto de Miranda Filho
Hércules Bruno Moreira de Almeida
Ílton José dos Santos
José Carlos Gouvea de Oliveira
Júlio Cesar Segal
Luiz Alberto Ferreira dos Santos
Robson Jorge Rocha

Gerência de Administração e Manutenção de Equipamentos de Informática

Gerente: Silvino Cavalcanti de Albuquerque Junior

Gerentes
Carlos Alberto Dias de Souza
Romualdo Carneiro da Cunha

Técnicos
Antônio Carlos Vieira dos Santos
Maria de Fátima Branco Fonseca
Oto Luiz Villa Real Americano
Sérgio Rubens Sarlo Ribeiro

Coordenação de Metodologia e Banco de Dados

Coordenadora: Maria Célia Pelisson Jacon

Gerentes
Dulce Maria Rocha Barbosa
José Masello
Luiz Antônio Gauziski de Araújo Figueredo
Paulo Bahia Araújo
Reina Marta Hanono

Técnicos
Bianca Gonçalves Fernandes
Carla Maria Silveira de Sá
Cecília Maria Alves de Andrade
Magali Ribeiro Chaves
Normando Duarte de Oliveira

Diretoria Executiva

Assessora: Virgínia Pegado Gonçalves

Coordenação das Atividades de Apoio Administrativos

Coordenador: Franklin Moreira de Almeida

Coordenadores e Gerentes
Andréa Tommasi Oliveira
Ângela Cristina Bartelega Areias
Ângela Maria Francisco de Paula
Antonio Carlos Mantuano
Antonio Fernando de Andrade Alves
Aurelino Domingues Souto Filho
Carlos Augusto Martins Gomes
Castoel Monteiro Wanzeller
Célia Regina Fonseca Grangeiro
Edson Wanderlei Fontana
Eduardo Alfredo Passos Rodrigues
Eneida Marisa Carvalho de Mattos
Fernando César Almeida Rosado
Georgete da Cruz Gomes
Gylcilene Ribeiro Storino
Josiane Heil Figueira
Leila Ribeiro Galart
Maria de Lourdes Adorno Alves
Maria Eliseta da Cruz Sor
Mário José Silva de Andrade
Roberto dos Passos Guimarães
Rose Mary Rodrigues
Rossana Patitucci Franco
Sergio Ribamar Horta Pimentel
Valmir Ferreira da Silva Júnior
Waldir Fortunato Junior
Wander Frauches Andrade

Técnicos
Adilson de Almeida
Alaíde Maria Barcelos Santos
Alexandre Chaves Farias Leite
Alexandre Loures Leite
Ana Cristina Rodrigues Pereira
Artur Amorim Americano
Bruno Gabriel de Castro
Celso Ferreira da Cunha Ribeiro

Escola Nacional de Ciências Estatísticas

Assessora: Sandra Furtado de Oliveira

Técnicos
Adilson Ribeiro da Silva
Ana Paula Donizetti Lins de Albuquerque
Rosana Pimentel de Almeida Gama

Unidades Estaduais

Chefes das Unidades Estaduais
AC: Adão Delfino dos Santos
AL: André Luís Figueredo da Silva
AP: Haroldo Canto Ferreira
BA: Artur Ferreira da Silva Filho
CE: Francisco José Moreira Lopes
DF: Walker Roberto Moura
ES: Max Athayde Fraga
GO: Daniel Ribeiro de Oliveira
MA: Pedro James de Souza Guedelha
MG: Maria Antonia Esteves da Silva
MS: Carlita Estevam de Souza
MT: Delvaldo Benedito Souza
PA: Antonio José de Souza Biffi
PB: Aniberto Mendonça de Melo
PE: Nilton Luiz de Nadai
PI: Raimundo Nonato da Silva Filho
PR: Sinval Dias dos Santos
RJ: Romualdo Pereira Rezende
RN: Elder de Oliveira Costa
RO: Argemiro Carvalho de Oliveira
RR: Vicente de Paulo Joaquim
RS: José Renato Braga de Almeida
SC: Mauricio Batista
SE: Antonio Pereira da Silva Marinho
SP: Francisco Garrido Barcia
TO: Ari Azevedo Soares

Gerências de Geodésia e Cartografia
BA: Hildeberto Biserra Lins
CE: Marcelo Campos Maia
DF: Silvio Rogerio Potier dos Santos
GO: Carlos Alberto Corrêa e Castro Junior
PA: Ariowaldo Banhos Cabral
SC: Paulo Roberto Guimarães Leal

Coordenadores Técnicos
AC: Célia Brandão de Souza

AL: Ramiro Parente de Oliveira
AM: Maria de Fatima Santos Silva
AP: Adrimauro da Silva Gemaque
BA: Dominique Marie Meduline Dupuit
CE: Francisco Otávio Cunha Pires
DF: Maria dos Reis Rodrigues Pinheiro
ES: Silvana Maria Paes Cangiani Pigato
GO: Onesio Francisco Dutra
MA: Demiurgo Lopes Trinta
MG: Maria Virgínia Fonseca Rocha
MS: Loide Bueno de Souza
MT: Valdemir José de Miranda
PA: Paulo Sérgio de Moraes Borges
PB: José Pereira de Araújo
PE: Otacílio Gonçalves Pereira
PI: Pedro Andrade de Oliveira
PR: Edemilson Mainardes Gonçalves
RJ: Alberto Azemiro Martins de Carvalho
RN: Maria Alzenira da Silva
RO: Devalcir Moreira dos Santos
RR: Murilo Cidade Junior
RS: Vanderlan Alves de Souza
SC: Mario Roberto Schmidt
SE: Alberto Ruan Correia
SP: Mitsuo Ito
TO: Raimundo da Costa Barbosa

Assistentes dos Coordenadores Técnicos
AL: Hélio Augusto Fonseca Pereira e Selma Regina dos Santos
BA: Hellie de Cássia Nunes Mansur e Fernando José da Silva Braga
CE: Ana Eugenia Ribeiro de Almeida e Josemar Tiné de Oliveira
DF: Gisela Rodrigues Vaz de Mello
ES: Maria de Lourdes Nunes Piontkovsky e Regina Celia Sunderhus Lube
GO: Ângela Maria Pereira Gandolfi e Eleci Raimunda Xavier de Oliveira
MA; Francisco Alberto Bastos Oliveira e Zilmar Alves Ferreira
MG: Abieser Knaip Horst e Vilma de Jesus Santos Cruz
MS: José Aparecido de Lima Albuquerque e Sely Batista Cavalcante
MT: Deajan David Montanha e Fernando Marques de Figueiredo
PA: José Nazareno de Azevedo
PB: Gelisa Fonseca Ribeiro e José Rinaldo de Souza
PE: José Homero Leite Vieira e Márcio Alekssander Granzotto Kuntze
PI: Jesus Ribeiro Soares e Pedro Soares da Silva
PR: Arnoldo Picelli e Jorge Mryczka
RJ: Eliana Maria Lisboa Garrão e José Cândido de Almeida Rodrigues
RN: José Aldemir Freire e Tarcísio Alberto Lopes Soares
RR: Murilo Cidade Junior
RS: Angelino Gomes Soares Neto e Claudio Franco Sant'Anna
SC: Carlos Roberto Roncatto Filho
SE: José Ailton Santos
SP: Paulo César Bertoli e Ricardo Yoshiyuki Hirata

Coordenadores Administrativos
AC: Jose Pedro Rea Ortiz
AL: Joel Gomes Vieira
AM: Sandra Cristina Vercosa Gomes
AP: Ariete Maria Sa de Souza
BA: Vilma da Rocha Quintanilha
CE: Rozimar Braga de Sousa
DF: Maria Helena Alves Santos
ES: Ana de Fatima Guaitolini
GO: Marina Luzia Rosa Ludegero
MA: Cynara Castro Barbosa do Carmo
MG: Jonas Pio da Veiga e Lúcio Flávio Couto Moreira
MS: Ceila Maria da Silva Veras dos Santos
MT: Ana Ortencia Teixeira Pinto
PA: Rony Helder Nogueira Cordeiro
PE: Ivanaldo Alves Nogueira
PB: Djaci Cavalcanti de Queiroz
PI: Elicio Rodrigues de Abreu
PR: Olindo Frazeto Filho
RJ: Henrique Vinicius Coelho de Souza
RN: Jose Erimar de Azevedo
RO: Maria Etelvina Cavalcanti Lacerda
RR: Silvania da Rocha Vila Nova Bertholini
RS: Flavia Marisa Klein
SC: Jose Paulo Simas
SE: Terezinha de Santana Almeida
SP: Aparecido Soares da Cunha
TO: Maria Aparecida de Almeida Valadares

Coordenadores de Informática
AC: Evandro Cavalcanti Araújo
AL: Mílton José do Nascimento
AM: Darlan Viana Cavalcante
AP: Ronaldo Barroso Sinimbu
BA: André Luiz Ferreira Urpia
CE: Júlio Marcus Vinícius Coelho
DF: José Magno de Ávila Junior
ES: Eric Alves Buhr
GO: Sebastião Gonçalves Matos
MA: Wellington Luís Mineiro França
MG: Carlos Cardoso Silva
MS: Emílio Flávio Vieira

MT : Camilo Gonçalo Stabilito
PA: Sílvio Costa de Sousa
PB: Haroldo Paulino de Medeiros
PE: Edílson Bronzeado Quirino
PI: Pedro Ribeiro Soares da Silva
PR: Márcio Rogério Kurz
RN: Edson Moreira Aguiar
RO: Antônio Carlos Lopes
RR: Marcelo Luiz Babick
RS: Sérgio Murilo Pereira Gil
SC: Luís Augusto de Souza Bevacqua
SE: Muciano Menezes Junqueira
SP: Wlamir Almeida Pinheiro
TO: Valmir Laurentino Gouveia

Coordenadores da Base Territorial
AC: Agmar Lopes de Souza
AL: Sérgio de Souza Alves
AM: Fernando de Souza Lima
AP: Marconi Edson Silva Uchôa
BA: Izail Arnaldo de Castro
CE: José Jerônimo Ribeiro Dias
DF: Sileimann de Carvalho Lemos
ES: Eugênio Ferreira da Silva Junior
GO: Colemar José de Moura e Lúcia Helena Resende Freitas Souza
MA: Francisco Alberto Bastos Oliveira
MG: Rodolfo Ricardo Ferreira
MS: Jovelino Alves de Souza
MT José Eduardo Araújo
PA: Edison Carvalho Nogueira
PB: João Batista de Melo Filho
PE: Marco Queiroz
PI: Bartolomeu da Silva Melo Filho
PR: Luiz Augusto Loyola Macedo
RJ: Antônio Jorge da Rocha Teixeira
RN: Orlando Batista de Vasconcelos
RO: Raimundo Ferreira Nobre Filho e Antonio Carlos Lopes
RR: Murilo Cidade Júnior
RS: Fernando Antonio Ballester Câmara
SC: Roque Bohnenberger e Antonio Guarda
SE: Alberto Loyola Monte Silva
SP: José Carlos dos Santos Oliveira
TO: Donizete Marques Galvão

Coordenadores das Comissões Censitárias Municipais
AC: Célia Brandão de Souza
AL: Ana Lucia Almeida da Silva
AM: Paulo da Silva Rodrigues de Almeida Filho
AP: Jeffenice Mirtes Picanço Costa
BA: Ana Lucia Valadares Rodrigues
CE: Luciana Martins Prazeres
DF: Maria Aparecida Gomes da Silva
ES: Claudio José Maltinti
GO: Sandra Maria Leandra Machado
MA: Antonio Henrique Silva Franco
MG: Narciza Sara Amador Santiago
MS: Rosangela Cypriano
MT: Micael Etiene de Souza
PA: Maria Ivone Costa e Silva Maciel
PB: Lamartine Candeia de Andrade
PE: Jáiniton Pereira da Costa Filho
PI: Solange de Souza Lopes Araújo
PR: Luis Fernando Rocha
RJ: Celso Mendes Targueta
RN: Zuleide Arcoverde de Melo
RO: Edinilce da Silva de Oliveira
RR: Murilo Cidade Junior
RS: Ernani Claire Valente Rodrigues
SC: Rogerio de Oliveira Rosa
SE: Eliana Lisboa Porto
SP: Reinaldo Apolinario dos Santos
TO: Francisco Soares Ferreira

Coordenadores de Treinamento
RO: Angela Ilcelina Holanda Nery
AC: Marcel Silva de Melo
AM: Lucia Tereza Porto Rego
RR: Murilo Cidade Junior
PA: Maria Angela Gemaque Alvaro
AP: Mário Picanço Flexa
TO: Geraldo Noronha Junqueira Filho
MA: Davi Souza da Costa
PI: Wilma Barbosa de Sosa Leite
CE: José Vanglésio Aguiar
RN: Débora Barbosa da Silva França
PB: Ramon Limeira Cavalcanti de Arruda
PE: Margareth Carneiro de Lima
AL: Jacy Pereira de Andrade Silva
SE: Adriane Almeida do Sacramento
BA: Rosa Amália Meireles Quadros
MG: Cláudia Tito Guimarães
ES: Carlos Alberto D' Almeida
RJ: Sergio Rosa Farias
SP: Nadir Alves Barbosa Ribeiro
PR: Erlete Luiza Schecheli
SC: Sonia de Fatima Sagaz Livramento
RS: Luercio Dantas Rego
MS: Albertino Lima Ribeiro
MT: Millane Chaves da Silva
GO: Alessandro de Siqueira Arantes
DF: Verônica Teixeira Magalhães dos Santos

Coordenadores de Área
AL
George Marcos de Oliveira Barbosa
Haroldo Alves de Farias
Hermano Ferreira da Silva Filho
Neilson Negrão Antelo Romar

AM
Carlos Alberto Lélis de Oliveira
Virginia Antas Cezario

BA
Aildete Nascimento Santana
Ana Cristina Almeida Serravalle
Artur Constantino Figueiredo Machado
Carlos Rui Costa Miranda
Enéas Gois da Fonseca
Graciete Silva de Souza
Irineu Santos dos Reis
José Antônio Araújo
José Carvalho Costa
Luiz Mafra de Santana
Manuel Lamartin Montes
Maria Thereza Cerqueira Silva
Paulo Macelo Gonzales Raña
Ronaldo Nascimento Gonçalves

CE
Antônio José Onofre Sampaio
Antonio Nogueira Amora
Jerônimo Candéa do Nascimento
José Jerônimo Ribeiro Dias
Luiz Facundo de Almeida
Maria Marlene Dantas de Vasconcelos
Paulo Cordeiro Duarte
Raimundo José Aguiar Ribeiro
Thony Batista Silva

DF
Paulo Roberto Lembi Alves

ES
Dermeval Mariani
Eugênio Ferreira da Silva Junior
Fernando Francisco de Paula
Sonia Cristina Machado Barbosa

GO
Carlos Augusto Canedo
Emival Ludovino de Santana
João Carlos de Oliveira
José Nilton de Brito
Valdino Esteves Rodrigues

MG
Adelimárcio Leonidas Viana Gonçalves
Anselmo de Moura
Antonio Augusto Correa
Benedito Augusto Barros Liarth
Dionel Novaes Miranda
Enide de Almeida Souza
Ernane de Campos Pereira
Eugenio Pacelli Morais Rennó
Evandro Mendes
Gilmar Moraes de Freitas
Helio Soares Pereira
João dos Santos Braga
José Antonio Felipe
José Cirilo Magalhães
José Marcílio Matos Costa
Josias Nogueira Giffoni
Marcia Maria Pinto de Moura Barros
Margareth Barros Santos
Maria Cristina de Almeida
Maria Das Graças Oliveira Souza
Maria Lúcia Laender Pita
Marlice de Matos da Silva
Oscar Tona Júnior
Raimundo Otávio Bicalho
Rosangela Lago de Souza Barbosa
Sérgio Antônio do Amaral Resende

MS
Espedito Soares de Souza
Everaldo Assad Arguello
Helio dos Santos Oliveira
Mauro Jordão da Silva
Wilson Douglas de Queiroz Bini

MT
Mateus João Weber
Remildo Rodrigues de Souza
Ricardo Litran
Sebastião de Assunção
Wandir da Costa Ribeiro

PB
Auseni Augusto de Araújo
Deodato Fortunato de Sousa
Gilberto Cavalcante de Medeiros
João Coelho de Lemos
Osvaldo de Sousa
Paulo Roberto da Silva
Rinaldo Toscano de Sousa

PE
Agnaldo Israel Mascena Pires
Isailda Maria Barros Pereira
José Francisco Olinda de Souza
Karla Valéria Annes de Sá Leitão da Costa
Normélia Carneiro de Lira
Pedro Salvador da Rocha
Remonde de Lourdes Godim Oliveira
Rosângela Barros Veras
Sérgio Caldeira Bueno

PI
Alberto Batista da Silva

Bartolomeu da Silva Melo Filho
Carlos Benevides Amorin
Euripedes Ferreira Sobrinho
José Dirso Alves de Meneses
Pedro Ribeiro Soares

PR
Albertino Franzoni
Angela Maria Barbosa
Antonio Zuber Neto
Arnaldo de Oliveira
Devair Jesus de Souza
João Batista Tacon
José de Nez
José dos Santos Miranda
Luiz Carlos Garcia
Uzias Marcelino da Silva

RJ
Antônio Jorge da Rocha Teixeira
Carlos Otto Espindola
Celso da Cunha Ferreira
Edson Henrique Teixeira de Moura
José Armando de Oliveira
José Marcos de Albuquerque
José Vitor Neves Guimar
Júlio Cesar dos Santos Freiras

RN
Antonio Esildo Costa
Jailson Filgueira Peregrino da Silva
Jose Nunes de Araújo
Manoel Gomes de Medeiros Neto

RO
Edinilce da Silva de Oliveira

RS
Alceu José Vanzella
André Pacheco Rocha
Clayton Costa da Silva
Elis Regina Manhabosco Alegranzi
Getúlio Moacir Ramos Durgante
Jones Domingo Bianchetti
José Roberto Alves
Mário de Ávila
Renato Barbieri Lima
Rogério Michelon Krause

SC
Darcio Francisco Borges
Gilmar Orsi
Gomercindo de Deus E Silva
Jair Aguilar Quaresma
Mariangela Ribeiro Brelinger
Soldemir Antonio Zanella
Tania Maria Bortoluzzi
Valmir Jose Leal

SE
Alberto Loyola Monte da Silva
João José de Santana
Leonardo Souza Leão Leite de Sá
Marcos Antonio Borges Correia

SP
Aparecido Donizetti Guirao
Carlos Alberto da Silva
Dagnaldo de Alcantara Rios
Dimas Carvalho Marques
Enive Violin
Eva Neide Ragozoni
Fernando Carraro
Hildebrando Neves Publio
Idilia Marques Pereira de Oliveira
Iedo Vecchi Machado
Ivan Donizetti Marafon
Jefferson Dias da Rocha
José Aparecido Mocheti
José Edimilson dos Santos
José Luiz Cardoso
Juarez Oliveira
Julio Cesar Mora
Luiz Carlos Estevam Foglia
Manoel Mauricio Santana Lins
Marcilio Pascoal Felippe
Marco Antonio Ornelas
Octavio Carrilo Junior
Paula Marques Meyer
Roberto Mitsuo Yamamoto
Sonia Antonia Soares

TO
Dilmas Pereira Santana
José Ribamar de Oliveira
Maximo Levi Leite Gomes

Coordenadores de Subárea
AC
Alcides Gadelha da Silva
Alcimar Borges de Freitas
Edson Prudencio da Silva
Olavo Ximendes Gonçalves

AL
Abelardo Leite de Gusmão
Addson da Silva Lima
Aldo Jorge Alves da Silva
Carlos Roberto Cavalcante Tenório

Cremilson Campos Viana
Edivaldo Barbosa Calheiros
José Carlos Ferreira da Silva
José Lucindo da Silva
José Luiz de Oliveira Lima
Maria de Fátima dos Santos Silva
Neuton Tavares Barbosa
Pedro Pinto da Silva
Robson Cavalcante Lopes
Silvio Bezerra Sobral
Washington Barreto Oliveira

AM
Antonio Alfredo da Costa Rezende
Antonio Tavares de Souza
Argemiro Nogueira do Nascimento Neto
Carlos Eleoterio de Moraes
Edinéia Macedo do Nascimento
Ednéia Roque Cortezão
Eliezer Lopes Moreira
Fernanda Rodrigues Gomes
Haroldo Bruno Campos dos Santos
Jessika Paloma Vieira Lima
João Monteiro de Souza Junior
Jorge Wilson de Andrade
Jose Carlos Santiago Magalhães
Jose Ilcleson Mendes Coelho
José Roberval Calheiros Gonzaga
Luiz Stanislau Vital
Manoel Teixeira de Melo
Marilúcia Silva de Moraes
Míriam Motta Correa Pinto
Norma Maria Bentes de Sousa
Orjanildo Nunes Carvalho
Ronaldo dos Santos Dezincourt
Rosinda dos Santos Guimarães
Sandra Maria Torres de Brito
Simplicio Oliveira de Almeida
Washington Reginaldo de Oliveira Maciel

AP
Adelson Silva Uchôa
Eduardo Fisbhen
Jose Maria Oliveira Monteiro
Marconi Edson Silva Uchoa

BA
Abelardo da Silva Normanha
Ademir Silva Sousa
Aderivaldo Lima da Silva
Alexandre Souza Silva Filho
Ana Maria Lobo de Melo
Antonio Carlos da Silva Pereira
Arivaldo Lima da Cruz
Carlos Fernando Lisboa Lobo
Claudia Gonçalves Xavier
Cleide Lopes Viana
Cristoval Pádua de França
Dacilio Chaves Campos
Dagoberto de Almeida e Marinho
Delma Almeida Sampaio
Dionir Pereira
Edigar Alves Gouveia
Edil da Silva Dourado
Edverges Ramos de Albuquerque
Eliete Oliveira Almeida
Elizabete Conceição Leal de Oliveira
Erica dos Santos Brites Guimarães
Esmeraldo de Araujo Santos
Evaristo Roque Lopes das Virgens
Felisberto Jose de Santana
Gilda Valverde Lima de Oliveira
Giltonei Everton Santos Barros
Graciete Silva de Souza
Gustavo Lefundes Blumetti
Hermes Araújo Barros
Humberto Soares Prazeres
Israel Vieira de Castro
Ivete Maria Dias Lago
Jeronymo Carneiro da Silva Filho
João Caldas Neto
Joel de Souza Vasconcelos
Jorge Luiz Prates Dill
José Arnaldo Oliveira Morais
José Esteves Ribeiro Neto
José Raimundo Lima de Cerqueira
José Santana de Oliveira
Joselino Rodrigues de Souza
Kátia Regina Lopes Dourado
Marcos Rogério Paixão Pestana
Maria Ana Souza Rego
Maria Angelica Borges Vieira
Maria Aparecida Pereira Mantovani
Maria Aparecida Souza Feitosa Brandão
Maria de Fátima de Moraes
Maria José Costa Leite dos Santos
Maria Ruth Moreira Cerqueira
Marília Oliveira Gama Brito Mendonça
Marleide Campos Santos Costa
Murilo Romão Gama
Nelson Lara da Costa
Nilo Antonio Monteiro de Mendonça
Olga Maria Freire Santos Falcão
Osvaldo Batista de Oliveira Filho
Paulo Ives Silva Trindade
Paulo Moreira Junior
Paulo Roberto Ribeiro Lopez
Railda Souza Ribeiro Melo
Raimundo Gomes da Silva
Ricardo Tavares Santana
Rita de Cassia Campos Nunes da Cruz

Robson Souza Ribeiro
Sandoval Martins Manciola Filho
Sônia Fuezi de Moura Barbosa
Tania Nogueira do Amaral
Teotonio Durval de Castro Dourado
Valdolino Mineiro Cunha
Virginia Maria Amancio da Silva Pereira
Wilma Souto Cardoso
Yola Ester de Queiroz Ferreira
Zeny Pereira Azevedo

CE
Antônio Carlos Cavalcante Dias Filho
Antônio Cesar Ferreira Lima
Benedito Luís de Lima
Cícero Pereira de Oliveira
Edilson Paulo da Silva
Francisco Conrado Chaves
Francisco Baltazar de Sousa Neto
Francisco Erivaldo Costa
Francisco Jairo Rocha Macedo
Francisco Luiz da Silva
Joana D'Arc Balbino da Silva
João Luis dos Santos Melo
José Alberto Façanha
José Arodo Nobre
José da Silva Nascimento
José Firmino da Silva
José Hermínio Botelho da Silveira
José Orion de Vasconcelos
José Roberto Bezerra Tavares
Júlio César de Brito Pinheiro
Lúcia de Fátima Mapurunga Batista
Marlene Teixeira Bessa
Miguel Fontes Ferreira
Ney Facundo Onofre
Osvaldo de Araújo Filho
Raimundo Rogaciano Montenegro

DF
Erenides Nunes de Souza
Felipe Fernandes Queiroz
Isac Gomes de Oliveira

ES
Eleni Dionizio de Oliveira
Eliomar Ferreira Pimentel
Gilson Vignatti
Ilmar Vicente Moreira
João Odílio Guedes Faria
Luiz Carlos Moraes
Marcia Vargas Santos Meneguelli
Neidimar Teixeira Narcizo
Sérgio Gago Amaro
Sidney Henrique Dalmaso

Tércio Engelhardt
Valter Nery Oliveira

GO
Adilson Lelis Nunes
Alberto Leite de Almeida
Alda Monteiro de Abreu Coutinho
Carlos Roberto Vieira Silva
Cícero Montesquieu Silva
Clovis Cruvinel da Silva
Colemar José de Freitas
Denilio Mendes Porto
Dilmar de Jesus Cavalcante
Ecinval Nunes da Fonseca
Ezimar Pereira da Costa
Goias do Couto Sobrinho
Helio Aires Alves Cabral
Iolando de Oliveira Cabral
Itamar Rosa Parreira
João Batista Eduardo de Souza
João Francisco de Sousa
João Tiburtino Alves
José Belisário Monteiro
Jovercino Alexandre dos Santos
Luiz Fernando da Silva
Olivio Romano Mereti
Ovidio Joaquim dos Santos

MA
Alenice Austríaco Lima
Antônio Lima Tavares
Audízio Araújo dos Santos
Elizaldo Pereira Quixaba
Fernando Morais da Costa
Francisco Aguiar dos Santos
Francisco Bastos
Francisco de Souza Lima
João Francisco da Luz Costa
João Márcio Fernandes Mendonça
José Carlos de Carvalho Pinto
José Natan Andrade
José Nunes Soares
José Ribamar Matos Borges
José Ribamar Pereira
Josué Nunes Brito
Lilio Remi Lago
Luana Santana dos Santos
Maria Luzenir Uchoa Diniz
Melquisedek Rodrigues de Oliveira
Robespierre Rocha Fontes
Saulo Carvalho Pires
Sóstono Alves da Silva
Washington Monteiro Santos
Wellington Georges Costa da Silva
Zacarias Meneses Carvalho

MG
Adilio Alves Leal
Alberto Martins Pereira
Alessandro Jose Silva Sant'Anna
Alexandre Luiz Gonçalves
Anderson Oliveira de Carvalho
Angelina Orlando Borges
Anna Elisa Godinho da Silva
Antônio de Pádua Alves Perdigão
Antônio João de Souza
Antonio Sergio de Melo Queiroz
Belmiro Dias Gomes
Bernadete Fagundes Lage
Bruno Fernandes Mourão
Carla Maria Rocha Alves
Carlos Alberto Vieira
Carlos Augusto Pereira Barros de Souza
Carlos Eduardo da Costa Araújo
Carlos Henrique Meireles Ávila
Cássio Rogério Ramos
Cátia Aparecida Rodrigues
Christiane Ávila de Lima Mascarenhas
Clevérson Ferreira da Silva
Daniel Aniceto de Souza da Silva
Darci Nunes Ferreira Freitas
Deise Cristina Barreto
Deise Maria Guerreiro Ribeiro
Delmir Silva Reis
Denise Araújo Perim Negri
Donizetti Domingos
Douglas Garcia de Menezes Silva
Ederson Tadeu Mourão
Edson Mendes Nascimento
Eduardo Antônio Mendes Oliveira
Eduardo Caldeira de Carvalho
Elane Cristina Lopes da Costa
Elizabeth Rosangela de Figueiredo Murta
Emilia Isolina Motta Coutinho
Ernei Barbosa Silva
Eunice Nery da Silva
Fabiana Fabrega de Oliveira
Flávio Ribeiro de Melo
Francisca Dulce Bauer de Assis
Geraldo das Graças Vasconcelos
Geraldo Gomes de Oliveira
Geraldo Mariano da Silva
Geraldo Mendes Santiago
Gilberto Arantes Junqueira
Gislene Maria Ferreira Ramos
Guilherme de Oliveira Assis
Gutemberg Jose de Freitas Filho
Hélcio Resende Júnior
Heliana Maria Ribeiro da Silva Araújo
Helton Pignolati
Hermano Souza Figueiredo
Hugo Rocha Carvalho Moraes
Humberto Marcus Leão Sette
Irapuan Vidal Junqueira da Silva
Iron Fernandes Pereira
Jioji Nishida
João Almeida Filho
João Batista Ferreira
João Bosco dos Reis
João Gonçalves Filho
João Rodrigues Pinto
Joaquim Cláudio Vieira de Rezende
Joel Rodrigues Reis
Joelson de Oliveira Carvalho
Jorge Nunes Medeiros
José Eduardo Franco
José Fernando Vasconcelos
José Geraldo de Souza
José Geraldo Freitas Queiróz
José Oraldo Meireles Rocha
José Teófilo Filho
Julio Cesar Tercetti Belli
Júlio Antônio Moreira Gomes
Júlio Cesar de Oliveira Moreira
Leonardo Fonseca Reis
Leonardo Frossard Alves
Leonardo Luiz Cabral
Lourdes Maria Gori Braga
Lúcio Rubens de Barros
Luis Eduardo da Silva Moreira
Luis Pedro Guimarães
Luiz Carlos Nunes Coelho
Luiz Fernando Santos Andrade
Luiz Flávio Lemos
Luiz Otávio Sábato
Manoel da Trindade Gaia da Silva
Marcelo Aguiar
Marcelo Gomes Martins
Márcio do Nascimento Pinto
Márcio José Guimarães
Márcio Silva Balão
Marco Aurélio dos Santos
Marcos André Costa Rodrigues
Maria Cecília de Santana Parreiras
Maria de Fátima Camargos Guimarães
Maria Goreth Perpétuo Alves
Maria Helena Morato Álvares da Silva
Maria Lúcia de Andrade Nogueira
Maria Rita Siqueira Moreira
Maria Sélia Coelho Souza Oliveira
Marilene Silva Gurgel Sampaio
Mariza Domingues Braga
Moacir Teixeira Leão
Nelson Hissao Nishicava
Orsini Lopes Vieira Sobrinho
Otacilio Rodrigues Alves
Otamir José de Andrade
Paulo Araújo Queiroz

Paulo Cesar Cerqueira Mendes
Paulo Ildecio Gonçalves
Rafael Moreira de Oliveira e Souza
Raimundo Nonato de Andrade Alves
Regina das Graças Costa Gonçalves
Roberto Lemes
Rogério Nolasco do Nascimento
Rogério Rodrigues da Silva
Ronaldo Contão Brauer
Rosamira Magalhães Costa
Rosangela Macedo de Sousa
Sara de Alvarenga Andrade
Sávio Rogério Beraldo Trombini
Sebastião Campos de Oliveira
Sebastião Cesar Almeida Machado
Selma Evangelista Jerônimo
Sérgio Abritta
Sérgio de Oliveira Sofiati
Sérgio Luiz Caixeta
Sérgio Mourão Rodrigues
Silvia Regina da Silva Queiróz
Sonia Marisa Jorge Marinho
Terezinha Alves Meira
Terezinha Stela Lambert Rosa
Uiara Terezinha Araújo Prado
Waldor Andrade Neto
Wodson Souto Lepesqueur

MS
Alcyone de Lamare
Aparecido Freitas Britto
Aureliano da Rosa Dutra
Bruno Billerbeck Carrapateira Junior
Carlos Roberto Rodrigues de Rodrigues
Claudionor Brunetto
Eloizio Correa da Costa
Ernesto Klais
Fernando César Fruguli Moreira
Honório Marcos Machado
Jair Alves dos Santos
Jairo Antonio de Queiroz
João Paulo dos Santos Vanin
Jorge Miranda Quevedo
José Tiago Leal
Lourival Oliveira Azambuja Neto
Paulo Cezar Rodrigues Martins
Samuel Claudio Aló de Alvarenga
Tadeu José Denardi

MT
Alcy Silva
Antônio Rubens Rodrigues dos Santos
Ariston Jerônimo dos Santos
Atahide Carvalhães de Oliveira
Aurelino Levy Dias de Campos
Carlos Antonio Neto Santos
Evande Praxedes da Silva
Evelino Martins da Cruz
Helito Serra
Ivan da Silva Maia
José Eduardo de Araújo
Lauro Antônio de Oliveira Auad
Nilson Santana Filho
Paulo Afonso Lírio
Valter Benedicto Ribeiro Pires
Wanderley Sebastião da Silva Fraga
William Foschiera

PA
Almir de Vasconcelos Uchôa
Antonio Araujo Gomes
Arlindo Pedroso Ferreira Filho
Djair Cardoso de Almeida
Douglas Gomes de Oliveira
Edilberto Figueira de Castro
Edvaldo Barbosa da Silva
Elson Queiroz Correa
Etelvina do Socorro Paranhos da Silva
Ezequiel Pereira de Araujo
Fernando Ferreira Gomes
Ivonilson Brito Rolim
Jeferson Antonio da Silva Paiva
José Danuzio Pinto Pompeu
Luiz Pedro Felgueiras Neto
Marco Aurelio Feliciano Andrade
Maria Gorete Monteiro Pompeu
Maria Tereza da Silva Penha
Nilton da Cruz Rocha
Osvaldo Camara da Silva
Osvaldo Nascimento
Otavio Almeida de Souza
Raymundo Nonato Ferreira Viana
Renaldo Ferreira do Carmo
Valdir Borges de Oliveira

PB
Antonio Félix dos Santos
Antonio Gabriel Ramos
Antonio Valdecy Martins
Davila Maria Andrade Figueiredo
Francisco de Assis Araújo
Francisco de Assis Nóbrega
Germano Bezerra da Nóbrega Júnior
João Alfredo Netto de Oliveira
João Araújo de Medeiros
José Alves Santil
José Antonio de Almeida Castro
José Félix de Lima
Livaldo Albino de Araújo
Luiz Carlos de Medeiros
Mauro Dias Velozo

Rafael Marconi dos Santos
Valdir Cayres Lacerda
Vicente Barbosa dos Santos

PE
Adelmo Costa Estima
Asarias Freitas de Lima
Edna Alzira Carvalho Pena da Rocha
Francisco Eudes Pereira
Francisco José de Carvalho
Givaldo Ferreira de Lima
Isaac Alves da Silva
Ivo de Sousa
João Hélio Beserra Guerra
José Amaury de Araújo
José Baltazar Soares da Silva
José Carlos Soares de Castro
José Fernando Fernandes de Carvalho
José Maria Maia Júnior
Josenilson Cavalcanti Catolé
Lourinaldo Bezerra dos Santos
Luzinelma Velozo Gonçalves
Marcelo Virginio de Melo
Marcos Augusto Monteiro Pontes
Marcos José de Lima Carvalho
Mônica Albuquerque Angelino Vilela Freitas
Otoniel Alves Alcantara
Pedro Ferreira Filho
Pedro Jorge Leitão de Melo
Sérgio Murilo Fagundes de França
William Roberto Paterson

PI
Antônio Gonçalves dos Santos
Antônio Raimundo Gonzaga Martins
Benedito Rodrigues da Silva
Carlos Benevides Amorim
Carlos Eugênio Lages Veras
Eurico Ângelo Bezerra
Filomeno Richard Portela Neto
Francisco das Chagas Sotero
Francisco das Chagas Rodrigues Miranda
Francisco Lima Neto
Geraldo Martins Filho
Hermes Rodrigues de Araújo
Horácio Fernandes Lima Neto
João da Cruz Sousa Araújo
Nilo Cunha E Silva Filho
Pedro Barros Feitoza
Raimundo Nonato Mendes de Sousa
Sergio Rubens Sarlo Ribeiro

PR
Adison Tulio Ayres do Nascimento
Admocir de Sant´ana Silva
Alfeu Celso Campiolo
Antonio Carlos de Almeida Costa
Antonio Maioli
Antonio Mozair de Souza
Antonio Norberto Schneider
Belmiro Bachett
Berenildo Fernandes Chagas
Carlos Alberto de Sá
Carlos Henrique Petroski
Clóvis dos Santos
Darlene Dona
Delmo de Carvalho
Edson dos Santos Ferreira
Francisco Rodrigues Neves
Glaucio Cicero da Silva
Guilherme Ernesto Tonin
Helena José da Silva de Oliveira
Hilário Bedendo Pricinato
João da Silva
Jorge Luiz Strapasson
José Carlos Koeche
José Leocádio Pedroso
José Nicoletti
José Tadeu do Amaral
Laercio Arali
Luiz Carlos Lubczyk
Luiz Henrique Pedrozo de Morais
Maria Divina Rodrigues
Maria Neuza Janeiro
Max Nuni Cesca Battisti
Nilza Terezinha Toczek
Orlando Sérgio Rizental da Luz
Paulo Camargo França
Paulo Roberto de Freitas
Rudimar Antonio Stefanello
Sebastião Aparecido de Azevedo
Simão Pedro de Brito
Solange Mary da Silva Soares
Valdir Matuchake Ascari
Vera Lúcia Borges de Carvalho Toneti
Wanderley José dos Santos
Wilson Barbosa
Zélia de Lucca Debiasi
Zido Raddatz

RJ
Aldir da Silva Ferreira
Alan Aziz de Morais
Carlos Augusto Bernardo da Conceição
Carlos Luis Nogueira Gentil
Carlos Roberto Gomes Viggiano
Cid Oliveira de Macedo
Clóvis Bergamini de Souza Filho
Déa Dolores Amaral Alves
Denise Guimarães Vieira
Dilma Rios Cordeiro

Domingos Fernando Andreoni
Fátima Petra da Silva
Gerson da Costa Gonçalves
Gerson Ferraz Filho
Jairo Cortes Vilella
Jorge Medeiros Lima do Nascimento
José Roberto Bittencourt Boia
Luiz Carlos Facchinetti Chrispino
Luiz Cláudio da Silva Malvino
Marcos Vinícius da Silva Pacheco
Miraldo Fernandes Ribeiro
Paulo Sérgio Reis de Siqueira
Roberto Brito Velasco
Rogério de Souza Machado Costa
Rony Andrade Vieira
Rosangela Silva Galiaço
Tania Mara de Augustinis Gama
Valéria de Barros Ribas
Wagner Scheid da Fonseca
Walter Tadeu Garcia Menezes

RN
Dulce Leda Pinheiro Guanabara
Erasmo Freire Bezerra
Eriosvaldo Duarte Celestino
João Batista da Farias Filho
José de Souza Torres
José Euzébio dos Santos Filho
José Pinheiro Nunes
José Wanderley dos Santos
Luís Fernando Ventura da Conceição
Luiz Carlos Dias Lopes
Moysés Mário de Paiva Júnior
Rogério Henrique da Costa Campelo
Severino Batista de Araújo
Temistocles Barros da Rocha
Vitor George Costa Gameleira
Yale Clecino Martins

RO
Ademilson Uchoa Matos
Airton José Dalpias
Antonio Nirvando Maciel Rocha
Fábio José Alves de Souza
Fernando Augusto Nery Lima
Gerino Alves da Silva Filho
Joaquim Lopes Lamego
Mirocem da Rocha Macieira
Valter Nichio Bertoni

RR
Amancio Guerra Raposo Junior
Francisco Carlos Alberto da Silva
Jose Carlos Ramires

RS
Ademar Camargo dos Reis
Admar Helinton Dornfeld
Ana Lucia dos Santos Silva Boni
Arthur Krzyzaniak
Carlos Albano Thomas
Carlos Augusto Costa Nunes
Carlos Rosano Schmidt
Cirio Ernesto Sabin
Claudio Roberto da Rosa Santos
Daniel de Pietro da Rocha
Dirceu Alves de Moraes
Domelviro Moraes Trindade
Elinton da Silva Vasconcelos
Elza Zardo
Enio Luiz Perrando
Erci da Silva Fraga
Fabio Einsfeld
Flavio Antonio Freire da Rosa
Gustavo Reginatto
Homero da Silva Pompeo
Ivanete Baroni
Ivon Adolfo Schaedler
João Alberto Bernardi
João Mario Mattozo Zipperer
João Pedro Perufo
Jorge Benhur Bilhar
Jorge Luis Feiten
José Antonio Insabralde dos Santos
José Inácio Finatto
Júlio Francisco dos Reis
Lauro Lindolfo Steffan
Luiz Alfredo Gallas
Luiz Carlos Fava
Luiz Eduardo Braga
Luiz Flávio de Lima Dias
Luiz Sérgio Mello Perin
Manoel Lentz de Oliveira
Marcos Laureano Santos Guerra
Maria Cristhina Webster
Maria do Carmo Rodrigues Trugillo
Milton Paulo Justen Boelke
Nei Oliveira Pereira
Neivo Spessatto
Nilton Bresolin
Osmar de Martini
Paulo Muszinski
Paulo Renato Pinto Lemos
Paulo Ricardo Hamester
Paulo Roberto Hartmann
Paulo Roberto Rodrigues da Silva
Silvio Feroleto
Solon Wagner dos Santos
Valdir José Froner
Valério Neumann
Vera Lucia Freitag

SC
Acelino Ortiz dos Santos
Ademir Karsten
Adriana Bandeira Seibert
Airton Ribeiro dos Santos
Alexandre Magno Camargo
Alvaro Antonio Watzko
Alvimir Boos Gomes
Amilton Marinho Machado
Bernardete Maria Krindges
Clacir Virmes
Gilberto Joel Segundo Postalli Lanzarin
Isaac Tegnalt Carrer
Ivo Pereira
Ivon Silvestre Sedlacek
João Paulo Filho
Joao Volni Madruga da Silva
Juarez Amaral
Luana Vicente dos Santos
Maria Salete de Souza Pereira
Maristela Zanini Pompermayer
Nair Henning
Olavo Machado da Silva
Omar Ivan Diesel
Oneide Margarete Lazzarin
Pedro Joel Alves Figueira
Ricardo de Queiroz Ribeiro
Sady Roque Silvestrin
Sirley Muller Lencina
Valdir Spadotto

SE
Andir do Carmo Wanderley
Cézar Oliveira Freitas
Ewerton Fernando Santana Coelho
José Almeida
Manoel Messias Alves
Mário Jorge Andrade Oliveira
Marise Lima e Silva
Moisés Araújo Guimarães
Roberto Menezes do Amor Divino

SP
Abdias Silveira Alves Ribeiro
Acyr Moraes Filho
Afonso Maria Nogueira
Aguinaldo Silva
Alaor Jakson Gardenal
Amadeu Bispo da Silva
Ana Maria Pedro Soriano
Andrea Cristina Berti
Antonio Aderci Moitinho
Antonio Carlos Amaro da Silva
Antonio dos Santos Junior
Antonio Eliseu Marques Lontra
Antonio Inacio da Silva Neto
Antonio Izidio de Souza Filho
Antonio Tadeu Furlani
Auad Atala Junior
Ayrton Prado Santos
Benedito Roberto da Silva
Benevaldo Julio Cardoso
Carlos Alberto Ianguas
Carlos Roberto da Silva
Carolina da Costa Ziviani
Claudio Dias Ferreira da Cunha
Deise Salles Garcia
Dejair Jose Delalibera
Edison Augusto Machado
Edison Pereira
Edson da Silva
Elson Maciel Coutinho
Eugenio Gehring Filho
Fernando Martinelli
Flavio Bonamini
Florindo Jacinto da Silva
Francisco Ferreira Martins
Gilberto Fontolan Costa
Guilherme Dias Batista
Helio Rodrigues
Ione de Alencar Silva
Ivan Nogueira do Amaral
Jackson da Silva
Jair Ananias Soldera
Jair da Silva Filho
Jefferson Joel de Carvalho Junior
Joao Batista Bertoli
Joao Carlos Rodrigues
Joao Roberto Rocha de Moraes
Jose Aldo Barreto
Jose Antonio Gomes Fontes
Jose Aparecido da Silveira
Jose Carlos Cogo
Jose Claudio Rotta
Jose Jorge Pereira Filho
Jose Maria Gurgel Fernandes
Jose Moreira Gomes
Jose Roberto Oseas da Silva
Jose Teixeira Guimarães
Julio Cesar Biaggio
Laerte Silva Junior
Lenira Cristina Oriente Blanco
Luiz Carlos Xavier Filho
Luiz Felix Bertoli
Luiz Fernando Nicolini Lemos
Marcela Fantucci de Castro
Marcia Maria Ribeiro de Paula Spano
Marcilei Correa Ventris
Marcos Antonio Cecato
Marcos Antonio Melo
Marcus Vinicius Farbelow
Maria de Lourdes Monteiro dos Santos

Maria Jose Alves Felippe
Maria Jose Alves Martins
Maria Jose Menk
Maria Odete Dias Viana
Marileia Pultz
Mario Sergio de Alencar
Mario Sergio Matheus dos Santos
Matilde Tabanez dos Santos Pereira
Mauricio Pilar da Silva
Mauricio Sinezio Sarquis Pinto
Mauricio Zacharias Moreira
Milto Tavares da Silva
Miriam Aparecida Espagnolo
Neide Davi Olivo
Nelson Belentani
Nelson Hissao Komiyama
Nildo Eduardo Martins
Nilton Aparecido Moraes de Oliveira
Osvaldo Cesar Ferraro
Patricia Regina Nunes Marques
Paula Marques Meyer
Paulo Freitas Brito
Paulo Roberto Devides
Paulo Roberto Segato
Renato Bueno de Camargo
Roberto Hauck Reichert Filho
Romessi Ferraz dos Santos
Romulo Gasparini da Cunha
Rubens Paulo de Lazari Pastana
Selma Sapone
Sergio Majewski
Sueli Tereza Rodrigues Scarso
Valdemar da Cruz Rodrigues
Vanessa Soares de Abreu
Vlademir de Azevedo
Yoshimi Takahashi

TO
Aldercio Diniz Gusmão
Antonio José Pereira Mota
Celio Costa Lacerda
Desirée Thommen Dias
Donizete Marques Galvão
Erildo Vicente de Oliveira
Fábio Arnaldo Ozório dos Santos
Fernando Cezar Rodrigues Póvoa
Gerisvaldo Pereira da Silva
Manoel Oliveira Castro Junior
Maria Francisca Pereira dos Santos Paolini
Olívio Gomes Ferreira
Silveirinha Guimarães Lima

Centro de Documentação e Disseminação de Informações – CDDI

Marise Maria Ferreira

Coordenação dos Censos

Coordenadora: Rose Maria Barros de Almeida

Coordenadores e Gerentes
Ana Raquel Gomes da Silva
Carlos José Lessa de Vasconcellos
Carmen Danielle Lins Mendes Macedo
Delfim Teixeira
Ednalva Maia do Monte
Edna Campello
Evilmerodac Domingos da Silva
Jorge Calian
Jorge Luis Loureiro de Araújo
Katia Vaz Cavalcanti
Luiz Sérgio Cardoso de Sá
Maria Alice da Silva Neves Nabuco
Maria Teresa Passos Bastos
Mário Henrique Monteiro Mattos
Paulo Cesar de Sousa Quintslr
Taíssa Abdalla Filgueiras de Souza

Técnicos
Agláia Pereira Tavares de Almeida
Camila Ermida Pinto
Eduardo Sidney Rodrigues de Araújo
Elizabeth Santos da Fontoura
Giovanna Altomare Catão
Helga Spiz
Helena Kiyoe Ito
Herben Kally de Almeida
Ivan Pereira Jordão Júnior
Izabelle de Oliveira
Leandro Albertini
Luiz Carlos Chagas Teixeira
Luiz Paulo do Nascimento
Marcelo Thadeu Rodrigues
Márcia Silva Passos
Maria do Carmo Dias Bueno
Paulo Fernandes
Raphael Soares de Moraes
Reynaldo José Monteiro
Ricardo da Silva Lopes
Rita de Cássia Ataualpa da Silva
Roberto Stoeterau

Projeto Editorial

Centro de Documentação e Disseminação de Informações

Coordenação de Produção

Marise Maria Ferreira

Gerência de Editoração

Estruturação textual, tabular e de gráficos
Beth Fontoura
