# Supplementary material for: Forecasting dengue fever in Brazil: An assessment of climate conditions
Source: PLoS One. 2019 Aug 8;14(8):e0220106. doi: 10.1371/journal.pone.0220106 (PMC6687106; doi:10.1371/journal.pone.0220106)
Supplement: S1 File — (ZIP) [file pone.0220106.s003.zip › data and codes_submit/training dataset analysis/data/Data IBGE/pop_municipios_2011.pdf]

ESTIMATIVAS DA POPULAÇÃO RESIDENTE NOS MUNICÍPIOS BRASILEIROS COM DATA DE REFERÊNCIA EM 1º DE JULHO DE 2011

NOTAS EXPLICATIVAS

A população do Brasil difere em 2.791 pessoas (192.379.287 pessoas) em relação àquela divulgada no D.O.U em 31/08/2011 (192.376.496 pessoas) em decorrência do critério de arredondamento para números inteiros, a ser adotado a partir deste ano.
Para maiores detalhes ver: www.ibge.gov.br/home/estatistica/estimativa2011/default.shtm

* o Município de Santarém-PB, código 13653, passou a ser denominado oficialmente de Joca Claudino-PB a partir de 23/11/2010, por força de Lei Municipal nº 049/2010.

* o Município de Campo de Santana-PB, código 16409, passou a ser denominado oficialmente de Tacima-PB a partir de 30/12/2009, por força de Lei Complementar nº 016/2009.

UF	COD. UF	COD. MUNIC	NOME DO MUNICÍPIO	POPULAÇÃO ESTIMADA
RO	11	00015	Alta Floresta D'Oeste	24.228
RO	11	00379	Alto Alegre dos Parecis	12.825
RO	11	00403	Alto Paraíso	17.444
RO	11	00346	Alvorada D'Oeste	16.625
RO	11	00023	Ariquemes	91.570
RO	11	00452	Buritis	32.899
RO	11	00031	Cabixi	6.221
RO	11	00601	Cacaulândia	5.764
RO	11	00049	Cacoal	78.959
RO	11	00700	Campo Novo de Rondônia	12.758
RO	11	00809	Candeias do Jamari	20.292
RO	11	00908	Castanheiras	3.527
RO	11	00056	Cerejeiras	16.939
RO	11	00924	Chupinguaia	8.515
RO	11	00064	Colorado do Oeste	18.338
RO	11	00072	Corumbiara	8.655
RO	11	00080	Costa Marques	14.023
RO	11	00940	Cujubim	16.570
RO	11	00098	Espigão D'Oeste	28.963
RO	11	01005	Governador Jorge Teixeira	10.272
RO	11	00106	Guajará-Mirim	41.934
RO	11	01104	Itapuã do Oeste	8.700
RO	11	00114	Jaru	51.883
RO	11	00122	Ji-Paraná	117.363
RO	11	00130	Machadinho D'Oeste	31.780
RO	11	01203	Ministro Andreazza	10.277
RO	11	01302	Mirante da Serra	11.781
RO	11	01401	Monte Negro	14.204
RO	11	00148	Nova Brasilândia D'Oeste	19.883
RO	11	00338	Nova Mamoré	23.143
RO	11	01435	Nova União	7.437
RO	11	00502	Novo Horizonte do Oeste	10.084
RO	11	00155	Ouro Preto do Oeste	37.702
RO	11	01450	Parecis	4.902
RO	11	00189	Pimenta Bueno	33.981
RO	11	01468	Pimenteiras do Oeste	2.299
RO	11	00205	Porto Velho	435.732
RO	11	00254	Presidente Médici	22.009
RO	11	01476	Primavera de Rondônia	3.464
RO	11	00262	Rio Crespo	3.346

RO	11	00288	Rolim de Moura	50.899
RO	11	00296	Santa Luzia D'Oeste	8.678
RO	11	01484	São Felipe D'Oeste	5.939
RO	11	01492	São Francisco do Guaporé	16.341
RO	11	00320	São Miguel do Guaporé	21.879
RO	11	01500	Seringueiras	11.624
RO	11	01559	Teixeirópolis	4.832
RO	11	01609	Theobroma	10.612
RO	11	01708	Urupá	12.828
RO	11	01757	Vale do Anari	9.511
RO	11	01807	Vale do Paraíso	8.084
RO	11	00304	Vilhena	77.937
AC	12	00013	Acrelândia	12.779
AC	12	00054	Assis Brasil	6.192
AC	12	00104	Brasiléia	21.837
AC	12	00138	Bujari	8.629
AC	12	00179	Capixaba	9.088
AC	12	00203	Cruzeiro do Sul	79.174
AC	12	00252	Epitaciolândia	15.394
AC	12	00302	Feijó	32.487
AC	12	00328	Jordão	6.740
AC	12	00336	Mâncio Lima	15.554
AC	12	00344	Manoel Urbano	8.105
AC	12	00351	Marechal Thaumaturgo	14.683
AC	12	00385	Plácido de Castro	17.401
AC	12	00807	Porto Acre	15.213
AC	12	00393	Porto Walter	9.448
AC	12	00401	Rio Branco	342.299
AC	12	00427	Rodrigues Alves	14.832
AC	12	00435	Santa Rosa do Purus	4.879
AC	12	00500	Sena Madureira	38.709
AC	12	00450	Senador Guiomard	20.387
AC	12	00609	Tarauacá	36.186
AC	12	00708	Xapuri	16.370
AM	13	00029	Alvarães	14.237
AM	13	00060	Amaturá	9.633
AM	13	00086	Anamã	10.495
AM	13	00102	Anori	16.701
AM	13	00144	Apuí	18.326
AM	13	00201	Atalaia do Norte	15.545
AM	13	00300	Autazes	32.733
AM	13	00409	Barcelos	25.835
AM	13	00508	Barreirinha	27.722
AM	13	00607	Benjamin Constant	34.194
AM	13	00631	Beruri	15.828
AM	13	00680	Boa Vista do Ramos	15.325
AM	13	00706	Boca do Acre	30.906
AM	13	00805	Borba	35.448
AM	13	00839	Caapiranga	11.142
AM	13	00904	Canutama	13.843
AM	13	01001	Carauari	25.955
AM	13	01100	Careiro	33.132
AM	13	01159	Careiro da Várzea	24.442
AM	13	01209	Coari	76.646
AM	13	01308	Codajás	23.644
AM	13	01407	Eirunepé	31.020
AM	13	01506	Envira	16.635

AM	13	01605	Fonte Boa	23.011
AM	13	01654	Guajará	14.189
AM	13	01704	Humaitá	45.105
AM	13	01803	Ipixuna	22.867
AM	13	01852	Iranduba	41.374
AM	13	01902	Itacoatiara	87.970
AM	13	01951	Itamarati	8.010
AM	13	02009	Itapiranga	8.281
AM	13	02108	Japurá	7.388
AM	13	02207	Juruá	11.126
AM	13	02306	Jutaí	18.145
AM	13	02405	Lábrea	38.373
AM	13	02504	Manacapuru	86.078
AM	13	02553	Manaquiri	23.576
AM	13	02603	Manaus	1.832.424
AM	13	02702	Manicoré	47.707
AM	13	02801	Maraã	17.563
AM	13	02900	Maués	53.173
AM	13	03007	Nhamundá	18.503
AM	13	03106	Nova Olinda do Norte	31.232
AM	13	03205	Novo Airão	15.113
AM	13	03304	Novo Aripuanã	21.784
AM	13	03403	Parintins	102.946
AM	13	03502	Pauini	18.249
AM	13	03536	Presidente Figueiredo	27.926
AM	13	03569	Rio Preto da Eva	26.344
AM	13	03601	Santa Isabel do Rio Negro	18.729
AM	13	03700	Santo Antônio do Içá	24.689
AM	13	03809	São Gabriel da Cachoeira	38.507
AM	13	03908	São Paulo de Olivença	32.060
AM	13	03957	São Sebastião do Uatumã	10.978
AM	13	04005	Silves	8.495
AM	13	04062	Tabatinga	53.374
AM	13	04104	Tapauá	18.010
AM	13	04203	Tefé	61.223
AM	13	04237	Tonantins	17.200
AM	13	04260	Uarini	12.017
AM	13	04302	Urucará	16.996
AM	13	04401	Urucurituba	18.265
RR	14	00050	Alto Alegre	16.337
RR	14	00027	Amajari	9.637
RR	14	00100	Boa Vista	290.741
RR	14	00159	Bonfim	11.068
RR	14	00175	Cantá	14.312
RR	14	00209	Caracaraí	18.714
RR	14	00233	Caroebe	8.300
RR	14	00282	Iracema	8.997
RR	14	00308	Mucajaí	15.065
RR	14	00407	Normandia	9.156
RR	14	00456	Pacaraima	10.698
RR	14	00472	Rorainópolis	24.808
RR	14	00506	São João da Baliza	6.898
RR	14	00605	São Luiz	6.861
RR	14	00704	Uiramutã	8.573
PA	15	00107	Abaetetuba	142.785
PA	15	00131	Abel Figueiredo	6.844
PA	15	00206	Acará	53.680

PA	15	00305	Afuá	35.467
PA	15	00347	Água Azul do Norte	25.286
PA	15	00404	Alenquer	53.004
PA	15	00503	Almeirim	33.588
PA	15	00602	Altamira	100.736
PA	15	00701	Anajás	25.254
PA	15	00800	Ananindeua	477.999
PA	15	00859	Anapu	21.398
PA	15	00909	Augusto Corrêa	41.072
PA	15	00958	Aurora do Pará	27.070
PA	15	01006	Aveiro	15.875
PA	15	01105	Bagre	24.644
PA	15	01204	Baião	38.092
PA	15	01253	Bannach	3.405
PA	15	01303	Barcarena	102.668
PA	15	01402	Belém	1.402.056
PA	15	01451	Belterra	16.451
PA	15	01501	Benevides	52.888
PA	15	01576	Bom Jesus do Tocantins	15.467
PA	15	01600	Bonito	13.923
PA	15	01709	Bragança	114.720
PA	15	01725	Brasil Novo	15.575
PA	15	01758	Brejo Grande do Araguaia	7.306
PA	15	01782	Breu Branco	54.032
PA	15	01808	Breves	93.835
PA	15	01907	Bujaru	26.053
PA	15	02004	Cachoeira do Arari	20.801
PA	15	01956	Cachoeira do Piriá	27.332
PA	15	02103	Cametá	122.683
PA	15	02152	Canaã dos Carajás	27.929
PA	15	02202	Capanema	64.140
PA	15	02301	Capitão Poço	52.057
PA	15	02400	Castanhal	176.116
PA	15	02509	Chaves	21.286
PA	15	02608	Colares	11.439
PA	15	02707	Conceição do Araguaia	45.724
PA	15	02756	Concórdia do Pará	28.774
PA	15	02764	Cumaru do Norte	10.811
PA	15	02772	Curionópolis	18.197
PA	15	02806	Curralinho	29.204
PA	15	02855	Curuá	12.487
PA	15	02905	Curuçá	34.919
PA	15	02939	Dom Eliseu	52.224
PA	15	02954	Eldorado dos Carajás	31.954
PA	15	03002	Faro	8.035
PA	15	03044	Floresta do Araguaia	18.036
PA	15	03077	Garrafão do Norte	25.097
PA	15	03093	Goianésia do Pará	31.031
PA	15	03101	Gurupá	29.520
PA	15	03200	Igarapé-Açu	36.155
PA	15	03309	Igarapé-Miri	58.498
PA	15	03408	Inhangapi	10.218
PA	15	03457	Ipixuna do Pará	53.318
PA	15	03507	Irituia	31.429
PA	15	03606	Itaituba	97.704
PA	15	03705	Itupiranga	51.341
PA	15	03754	Jacareacanga	14.223

PA	15	03804	Jacundá	52.191
PA	15	03903	Juruti	48.306
PA	15	04000	Limoeiro do Ajuru	25.440
PA	15	04059	Mãe do Rio	28.100
PA	15	04109	Magalhães Barata	8.148
PA	15	04208	Marabá	238.708
PA	15	04307	Maracanã	28.438
PA	15	04406	Marapanim	26.750
PA	15	04422	Marituba	110.842
PA	15	04455	Medicilândia	27.785
PA	15	04505	Melgaço	25.096
PA	15	04604	Mocajuba	27.207
PA	15	04703	Moju	71.329
PA	15	04802	Monte Alegre	55.636
PA	15	04901	Muaná	34.875
PA	15	04950	Nova Esperança do Piriá	20.256
PA	15	04976	Nova Ipixuna	14.859
PA	15	05007	Nova Timboteua	13.844
PA	15	05031	Novo Progresso	25.138
PA	15	05064	Novo Repartimento	63.604
PA	15	05106	Óbidos	49.552
PA	15	05205	Oeiras do Pará	29.005
PA	15	05304	Oriximiná	63.905
PA	15	05403	Ourém	16.458
PA	15	05437	Ourilândia do Norte	27.965
PA	15	05486	Pacajá	40.831
PA	15	05494	Palestina do Pará	7.470
PA	15	05502	Paragominas	99.460
PA	15	05536	Parauapebas	160.229
PA	15	05551	Pau D'Arco	5.950
PA	15	05601	Peixe-Boi	7.862
PA	15	05635	Piçarra	12.699
PA	15	05650	Placas	24.744
PA	15	05700	Ponta de Pedras	26.560
PA	15	05809	Portel	53.257
PA	15	05908	Porto de Moz	34.756
PA	15	06005	Prainha	29.337
PA	15	06104	Primavera	10.311
PA	15	06112	Quatipuru	12.527
PA	15	06138	Redenção	76.501
PA	15	06161	Rio Maria	17.713
PA	15	06187	Rondon do Pará	47.509
PA	15	06195	Rurópolis	41.272
PA	15	06203	Salinópolis	37.726
PA	15	06302	Salvaterra	20.572
PA	15	06351	Santa Bárbara do Pará	17.584
PA	15	06401	Santa Cruz do Arari	8.378
PA	15	06500	Santa Isabel do Pará	60.713
PA	15	06559	Santa Luzia do Pará	19.426
PA	15	06583	Santa Maria das Barreiras	17.686
PA	15	06609	Santa Maria do Pará	23.194
PA	15	06708	Santana do Araguaia	58.067
PA	15	06807	Santarém	297.040
PA	15	06906	Santarém Novo	6.196
PA	15	07003	Santo Antônio do Tauá	27.199
PA	15	07102	São Caetano de Odivelas	16.991
PA	15	07151	São Domingos do Araguaia	23.370

PA	15	07201	São Domingos do Capim	30.034
PA	15	07300	São Félix do Xingu	95.694
PA	15	07409	São Francisco do Pará	15.123
PA	15	07458	São Geraldo do Araguaia	25.429
PA	15	07466	São João da Ponta	5.360
PA	15	07474	São João de Pirabas	20.890
PA	15	07508	São João do Araguaia	13.225
PA	15	07607	São Miguel do Guamá	52.350
PA	15	07706	São Sebastião da Boa Vista	23.307
PA	15	07755	Sapucaia	5.144
PA	15	07805	Senador José Porfírio	12.840
PA	15	07904	Soure	23.235
PA	15	07953	Tailândia	82.434
PA	15	07961	Terra Alta	10.416
PA	15	07979	Terra Santa	17.130
PA	15	08001	Tomé-Açu	57.228
PA	15	08035	Tracuateua	27.817
PA	15	08050	Trairão	17.093
PA	15	08084	Tucumã	34.334
PA	15	08100	Tucuruí	98.919
PA	15	08126	Ulianópolis	45.190
PA	15	08159	Uruará	44.758
PA	15	08209	Vigia	48.482
PA	15	08308	Viseu	57.148
PA	15	08357	Vitória do Xingu	13.607
PA	15	08407	Xinguara	40.984
AP	16	00105	Amapá	8.142
AP	16	00204	Calçoene	9.175
AP	16	00212	Cutias	4.805
AP	16	00238	Ferreira Gomes	5.974
AP	16	00253	Itaubal	4.371
AP	16	00279	Laranjal do Jari	40.820
AP	16	00303	Macapá	407.023
AP	16	00402	Mazagão	17.420
AP	16	00501	Oiapoque	21.095
AP	16	00154	Pedra Branca do Amapari	11.292
AP	16	00535	Porto Grande	17.252
AP	16	00550	Pracuúba	3.909
AP	16	00600	Santana	102.861
AP	16	00055	Serra do Navio	4.464
AP	16	00709	Tartarugalzinho	12.981
AP	16	00808	Vitória do Jari	12.725
TO	17	00251	Abreulândia	2.407
TO	17	00301	Aguiarnópolis	5.317
TO	17	00350	Aliança do Tocantins	5.633
TO	17	00400	Almas	7.518
TO	17	00707	Alvorada	8.364
TO	17	01002	Ananás	9.816
TO	17	01051	Angico	3.197
TO	17	01101	Aparecida do Rio Negro	4.267
TO	17	01309	Aragominas	5.860
TO	17	01903	Araguacema	6.387
TO	17	02000	Araguaçu	8.744
TO	17	02109	Araguaína	153.351
TO	17	02158	Araguanã	5.095
TO	17	02208	Araguatins	31.738
TO	17	02307	Arapoema	6.721

TO	17	02406	Arraias	10.619
TO	17	02554	Augustinópolis	16.180
TO	17	02703	Aurora do Tocantins	3.473
TO	17	02901	Axixá do Tocantins	9.310
TO	17	03008	Babaçulândia	10.432
TO	17	03057	Bandeirantes do Tocantins	3.162
TO	17	03073	Barra do Ouro	4.165
TO	17	03107	Barrolândia	5.370
TO	17	03206	Bernardo Sayão	4.449
TO	17	03305	Bom Jesus do Tocantins	3.879
TO	17	03602	Brasilândia do Tocantins	2.075
TO	17	03701	Brejinho de Nazaré	5.209
TO	17	03800	Buriti do Tocantins	9.916
TO	17	03826	Cachoeirinha	2.158
TO	17	03842	Campos Lindos	8.331
TO	17	03867	Cariri do Tocantins	3.815
TO	17	03883	Carmolândia	2.340
TO	17	03891	Carrasco Bonito	3.725
TO	17	03909	Caseara	4.674
TO	17	04105	Centenário	2.597
TO	17	05102	Chapada da Natividade	3.278
TO	17	04600	Chapada de Areia	1.340
TO	17	05508	Colinas do Tocantins	31.263
TO	17	16703	Colméia	8.555
TO	17	05557	Combinado	4.681
TO	17	05607	Conceição do Tocantins	4.168
TO	17	06001	Couto de Magalhães	5.061
TO	17	06100	Cristalândia	7.228
TO	17	06258	Crixás do Tocantins	1.578
TO	17	06506	Darcinópolis	5.350
TO	17	07009	Dianópolis	19.395
TO	17	07108	Divinópolis do Tocantins	6.409
TO	17	07207	Dois Irmãos do Tocantins	7.153
TO	17	07306	Dueré	4.595
TO	17	07405	Esperantina	9.619
TO	17	07553	Fátima	3.802
TO	17	07652	Figueirópolis	5.323
TO	17	07702	Filadélfia	8.528
TO	17	08205	Formoso do Araguaia	18.398
TO	17	08254	Fortaleza do Tabocão	2.433
TO	17	08304	Goianorte	4.965
TO	17	09005	Goiatins	12.143
TO	17	09302	Guaraí	23.445
TO	17	09500	Gurupi	77.655
TO	17	09807	Ipueiras	1.676
TO	17	10508	Itacajá	7.127
TO	17	10706	Itaguatins	6.002
TO	17	10904	Itapiratins	3.552
TO	17	11100	Itaporã do Tocantins	2.440
TO	17	11506	Jaú do Tocantins	3.537
TO	17	11803	Juarina	2.224
TO	17	11902	Lagoa da Confusão	10.521
TO	17	11951	Lagoa do Tocantins	3.602
TO	17	12009	Lajeado	2.806
TO	17	12157	Lavandeira	1.636
TO	17	12405	Lizarda	3.721
TO	17	12454	Luzinópolis	2.669

TO	17	12504	Marianópolis do Tocantins	4.431
TO	17	12702	Mateiros	2.268
TO	17	12801	Maurilândia do Tocantins	3.178
TO	17	13205	Miracema do Tocantins	20.396
TO	17	13304	Miranorte	12.687
TO	17	13601	Monte do Carmo	6.833
TO	17	13700	Monte Santo do Tocantins	2.102
TO	17	13957	Muricilândia	3.189
TO	17	14203	Natividade	9.011
TO	17	14302	Nazaré	4.328
TO	17	14880	Nova Olinda	10.786
TO	17	15002	Nova Rosalândia	3.815
TO	17	15101	Novo Acordo	3.817
TO	17	15150	Novo Alegre	2.287
TO	17	15259	Novo Jardim	2.481
TO	17	15507	Oliveira de Fátima	1.044
TO	17	21000	Palmas	235.316
TO	17	15705	Palmeirante	5.058
TO	17	13809	Palmeiras do Tocantins	5.826
TO	17	15754	Palmeirópolis	7.360
TO	17	16109	Paraíso do Tocantins	45.054
TO	17	16208	Paranã	10.333
TO	17	16307	Pau D'Arco	4.608
TO	17	16505	Pedro Afonso	11.732
TO	17	16604	Peixe	10.509
TO	17	16653	Pequizeiro	5.090
TO	17	17008	Pindorama do Tocantins	4.493
TO	17	17206	Piraquê	2.927
TO	17	17503	Pium	6.783
TO	17	17800	Ponte Alta do Bom Jesus	4.542
TO	17	17909	Ponte Alta do Tocantins	7.258
TO	17	18006	Porto Alegre do Tocantins	2.827
TO	17	18204	Porto Nacional	49.465
TO	17	18303	Praia Norte	7.727
TO	17	18402	Presidente Kennedy	3.676
TO	17	18451	Pugmil	2.399
TO	17	18501	Recursolândia	3.817
TO	17	18550	Riachinho	4.231
TO	17	18659	Rio da Conceição	1.755
TO	17	18709	Rio dos Bois	2.594
TO	17	18758	Rio Sono	6.267
TO	17	18808	Sampaio	3.946
TO	17	18840	Sandolândia	3.326
TO	17	18865	Santa Fé do Araguaia	6.683
TO	17	18881	Santa Maria do Tocantins	2.946
TO	17	18899	Santa Rita do Tocantins	2.150
TO	17	18907	Santa Rosa do Tocantins	4.588
TO	17	19004	Santa Tereza do Tocantins	2.555
TO	17	20002	Santa Terezinha do Tocantins	2.476
TO	17	20101	São Bento do Tocantins	4.675
TO	17	20150	São Félix do Tocantins	1.450
TO	17	20200	São Miguel do Tocantins	10.635
TO	17	20259	São Salvador do Tocantins	2.924
TO	17	20309	São Sebastião do Tocantins	4.331
TO	17	20499	São Valério da Natividade	4.332
TO	17	20655	Silvanópolis	5.095
TO	17	20804	Sítio Novo do Tocantins	9.122

TO	17	20853	Sucupira	1.763
TO	17	20903	Taguatinga	15.196
TO	17	20937	Taipas do Tocantins	1.963
TO	17	20978	Talismã	2.582
TO	17	21109	Tocantínia	6.809
TO	17	21208	Tocantinópolis	22.607
TO	17	21257	Tupirama	1.605
TO	17	21307	Tupiratins	2.154
TO	17	22081	Wanderlândia	11.036
TO	17	22107	Xambioá	11.471
MA	21	00055	Açailândia	105.255
MA	21	00105	Afonso Cunha	6.000
MA	21	00154	Água Doce do Maranhão	11.726
MA	21	00204	Alcântara	21.564
MA	21	00303	Aldeias Altas	24.346
MA	21	00402	Altamira do Maranhão	11.225
MA	21	00436	Alto Alegre do Maranhão	24.969
MA	21	00477	Alto Alegre do Pindaré	31.125
MA	21	00501	Alto Parnaíba	10.812
MA	21	00550	Amapá do Maranhão	6.508
MA	21	00600	Amarante do Maranhão	38.451
MA	21	00709	Anajatuba	25.629
MA	21	00808	Anapurus	14.220
MA	21	00832	Apicum-Açu	15.256
MA	21	00873	Araguanã	11.508
MA	21	00907	Araioses	43.089
MA	21	00956	Arame	31.835
MA	21	01004	Arari	28.651
MA	21	01103	Axixá	11.505
MA	21	01202	Bacabal	100.615
MA	21	01251	Bacabeira	15.264
MA	21	01301	Bacuri	16.687
MA	21	01350	Bacurituba	5.341
MA	21	01400	Balsas	85.322
MA	21	01509	Barão de Grajaú	17.681
MA	21	01608	Barra do Corda	83.454
MA	21	01707	Barreirinhas	56.123
MA	21	01772	Bela Vista do Maranhão	12.194
MA	21	01731	Belágua	6.622
MA	21	01806	Benedito Leite	5.483
MA	21	01905	Bequimão	20.725
MA	21	01939	Bernardo do Mearim	6.055
MA	21	01970	Boa Vista do Gurupi	8.166
MA	21	02002	Bom Jardim	39.401
MA	21	02036	Bom Jesus das Selvas	29.374
MA	21	02077	Bom Lugar	15.071
MA	21	02101	Brejo	33.808
MA	21	02150	Brejo de Areia	5.265
MA	21	02200	Buriti	27.235
MA	21	02309	Buriti Bravo	23.011
MA	21	02325	Buriticupu	66.326
MA	21	02358	Buritirana	14.858
MA	21	02374	Cachoeira Grande	8.528
MA	21	02408	Cajapió	10.668
MA	21	02507	Cajari	18.473
MA	21	02556	Campestre do Maranhão	13.511
MA	21	02606	Cândido Mendes	19.046

MA	21	02705	Cantanhede	20.667
MA	21	02754	Capinzal do Norte	10.710
MA	21	02804	Carolina	23.957
MA	21	02903	Carutapera	22.266
MA	21	03000	Caxias	156.327
MA	21	03109	Cedral	10.336
MA	21	03125	Central do Maranhão	8.006
MA	21	03158	Centro do Guilherme	11.632
MA	21	03174	Centro Novo do Maranhão	19.581
MA	21	03208	Chapadinha	74.274
MA	21	03257	Cidelândia	13.825
MA	21	03307	Codó	118.568
MA	21	03406	Coelho Neto	47.099
MA	21	03505	Colinas	39.388
MA	21	03554	Conceição do Lago-Açu	14.718
MA	21	03604	Coroatá	62.190
MA	21	03703	Cururupu	32.568
MA	21	03752	Davinópolis	12.603
MA	21	03802	Dom Pedro	22.737
MA	21	03901	Duque Bacelar	10.744
MA	21	04008	Esperantinópolis	17.917
MA	21	04057	Estreito	36.826
MA	21	04073	Feira Nova do Maranhão	8.171
MA	21	04081	Fernando Falcão	9.415
MA	21	04099	Formosa da Serra Negra	17.464
MA	21	04107	Fortaleza dos Nogueiras	12.271
MA	21	04206	Fortuna	15.137
MA	21	04305	Godofredo Viana	10.523
MA	21	04404	Gonçalves Dias	17.514
MA	21	04503	Governador Archer	10.290
MA	21	04552	Governador Edison Lobão	16.280
MA	21	04602	Governador Eugênio Barros	16.096
MA	21	04628	Governador Luiz Rocha	7.401
MA	21	04651	Governador Newton Bello	10.205
MA	21	04677	Governador Nunes Freire	25.362
MA	21	04701	Graça Aranha	6.145
MA	21	04800	Grajaú	63.203
MA	21	04909	Guimarães	12.039
MA	21	05005	Humberto de Campos	26.567
MA	21	05104	Icatu	25.426
MA	21	05153	Igarapé do Meio	12.806
MA	21	05203	Igarapé Grande	11.167
MA	21	05302	Imperatriz	248.806
MA	21	05351	Itaipava do Grajaú	12.337
MA	21	05401	Itapecuru Mirim	63.024
MA	21	05427	Itinga do Maranhão	24.997
MA	21	05450	Jatobá	8.793
MA	21	05476	Jenipapo dos Vieiras	15.589
MA	21	05500	João Lisboa	23.641
MA	21	05609	Joselândia	15.626
MA	21	05658	Junco do Maranhão	3.904
MA	21	05708	Lago da Pedra	46.701
MA	21	05807	Lago do Junco	10.798
MA	21	05948	Lago dos Rodrigues	7.769
MA	21	05906	Lago Verde	15.520
MA	21	05922	Lagoa do Mato	10.922
MA	21	05963	Lagoa Grande do Maranhão	12.879

MA	21	05989	Lajeado Novo	7.016
MA	21	06003	Lima Campos	11.475
MA	21	06102	Loreto	11.495
MA	21	06201	Luís Domingues	6.571
MA	21	06300	Magalhães de Almeida	17.938
MA	21	06326	Maracaçumé	19.566
MA	21	06359	Marajá do Sena	7.772
MA	21	06375	Maranhãozinho	14.118
MA	21	06409	Mata Roma	15.408
MA	21	06508	Matinha	22.089
MA	21	06607	Matões	31.307
MA	21	06631	Matões do Norte	14.283
MA	21	06672	Milagres do Maranhão	8.157
MA	21	06706	Mirador	20.495
MA	21	06755	Miranda do Norte	25.065
MA	21	06805	Mirinzal	14.312
MA	21	06904	Monção	32.157
MA	21	07001	Montes Altos	9.342
MA	21	07100	Morros	18.028
MA	21	07209	Nina Rodrigues	12.785
MA	21	07258	Nova Colinas	4.961
MA	21	07308	Nova Iorque	4.594
MA	21	07357	Nova Olinda do Maranhão	19.401
MA	21	07407	Olho d'Água das Cunhãs	18.711
MA	21	07456	Olinda Nova do Maranhão	13.416
MA	21	07506	Paço do Lumiar	107.764
MA	21	07605	Palmeirândia	18.896
MA	21	07704	Paraibano	20.276
MA	21	07803	Parnarama	34.791
MA	21	07902	Passagem Franca	17.773
MA	21	08009	Pastos Bons	18.268
MA	21	08058	Paulino Neves	14.749
MA	21	08108	Paulo Ramos	20.396
MA	21	08207	Pedreiras	39.419
MA	21	08256	Pedro do Rosário	23.099
MA	21	08306	Penalva	34.717
MA	21	08405	Peri Mirim	13.844
MA	21	08454	Peritoró	21.498
MA	21	08504	Pindaré-Mirim	31.384
MA	21	08603	Pinheiro	78.876
MA	21	08702	Pio XII	21.860
MA	21	08801	Pirapemas	17.555
MA	21	08900	Poção de Pedras	19.592
MA	21	09007	Porto Franco	21.890
MA	21	09056	Porto Rico do Maranhão	6.004
MA	21	09106	Presidente Dutra	45.155
MA	21	09205	Presidente Juscelino	11.722
MA	21	09239	Presidente Médici	6.471
MA	21	09270	Presidente Sarney	17.430
MA	21	09304	Presidente Vargas	10.843
MA	21	09403	Primeira Cruz	14.158
MA	21	09452	Raposa	27.037
MA	21	09502	Riachão	20.150
MA	21	09551	Ribamar Fiquene	7.382
MA	21	09601	Rosário	40.030
MA	21	09700	Sambaíba	5.505
MA	21	09759	Santa Filomena do Maranhão	7.155

MA	21	09809	Santa Helena	39.744
MA	21	09908	Santa Inês	78.020
MA	21	10005	Santa Luzia	74.501
MA	21	10039	Santa Luzia do Paruá	22.843
MA	21	10104	Santa Quitéria do Maranhão	29.460
MA	21	10203	Santa Rita	33.117
MA	21	10237	Santana do Maranhão	11.937
MA	21	10278	Santo Amaro do Maranhão	14.143
MA	21	10302	Santo Antônio dos Lopes	14.291
MA	21	10401	São Benedito do Rio Preto	17.904
MA	21	10500	São Bento	41.421
MA	21	10609	São Bernardo	26.765
MA	21	10658	São Domingos do Azeitão	7.037
MA	21	10708	São Domingos do Maranhão	33.650
MA	21	10807	São Félix de Balsas	4.669
MA	21	10856	São Francisco do Brejão	10.507
MA	21	10906	São Francisco do Maranhão	11.908
MA	21	11003	São João Batista	19.997
MA	21	11029	São João do Carú	15.649
MA	21	11052	São João do Paraíso	10.849
MA	21	11078	São João do Soter	17.423
MA	21	11102	São João dos Patos	24.927
MA	21	11201	São José de Ribamar	165.418
MA	21	11250	São José dos Basílios	7.501
MA	21	11300	São Luís	1.027.430
MA	21	11409	São Luís Gonzaga do Maranhão	19.952
MA	21	11508	São Mateus do Maranhão	39.418
MA	21	11532	São Pedro da Água Branca	12.113
MA	21	11573	São Pedro dos Crentes	4.456
MA	21	11607	São Raimundo das Mangabeiras	17.674
MA	21	11631	São Raimundo do Doca Bezerra	5.921
MA	21	11672	São Roberto	6.077
MA	21	11706	São Vicente Ferrer	21.052
MA	21	11722	Satubinha	12.301
MA	21	11748	Senador Alexandre Costa	10.386
MA	21	11763	Senador La Rocque	14.550
MA	21	11789	Serrano do Maranhão	11.080
MA	21	11805	Sítio Novo	17.147
MA	21	11904	Sucupira do Norte	10.450
MA	21	11953	Sucupira do Riachão	5.437
MA	21	12001	Tasso Fragoso	7.904
MA	21	12100	Timbiras	28.120
MA	21	12209	Timon	157.438
MA	21	12233	Trizidela do Vale	19.149
MA	21	12274	Tufilândia	5.624
MA	21	12308	Tuntum	39.560
MA	21	12407	Turiaçu	34.136
MA	21	12456	Turilândia	23.277
MA	21	12506	Tutóia	53.724
MA	21	12605	Urbano Santos	25.108
MA	21	12704	Vargem Grande	50.541
MA	21	12803	Viana	49.883
MA	21	12852	Vila Nova dos Martírios	11.608
MA	21	12902	Vitória do Mearim	31.406
MA	21	13009	Vitorino Freire	31.684
MA	21	14007	Zé Doca	51.615
PI	22	00053	Acauã	6.795

PI	22	00103	Agricolândia	5.080
PI	22	00202	Água Branca	16.600
PI	22	00251	Alagoinha do Piauí	7.378
PI	22	00277	Alegrete do Piauí	5.163
PI	22	00301	Alto Longá	13.735
PI	22	00400	Altos	39.031
PI	22	00459	Alvorada do Gurguéia	5.115
PI	22	00509	Amarante	17.155
PI	22	00608	Angical do Piauí	6.664
PI	22	00707	Anísio de Abreu	9.244
PI	22	00806	Antônio Almeida	3.054
PI	22	00905	Aroazes	5.761
PI	22	00954	Aroeiras do Itaim	2.441
PI	22	01002	Arraial	4.672
PI	22	01051	Assunção do Piauí	7.547
PI	22	01101	Avelino Lopes	11.164
PI	22	01150	Baixa Grande do Ribeiro	10.727
PI	22	01176	Barra D'Alcântara	3.855
PI	22	01200	Barras	45.154
PI	22	01309	Barreiras do Piauí	3.245
PI	22	01408	Barro Duro	6.594
PI	22	01507	Batalha	25.901
PI	22	01556	Bela Vista do Piauí	3.817
PI	22	01572	Belém do Piauí	3.337
PI	22	01606	Beneditinos	9.927
PI	22	01705	Bertolínia	5.335
PI	22	01739	Betânia do Piauí	6.029
PI	22	01770	Boa Hora	6.383
PI	22	01804	Bocaina	4.382
PI	22	01903	Bom Jesus	23.144
PI	22	01919	Bom Princípio do Piauí	5.357
PI	22	01929	Bonfim do Piauí	5.433
PI	22	01945	Boqueirão do Piauí	6.242
PI	22	01960	Brasileira	8.013
PI	22	01988	Brejo do Piauí	3.786
PI	22	02000	Buriti dos Lopes	19.144
PI	22	02026	Buriti dos Montes	8.027
PI	22	02059	Cabeceiras do Piauí	10.038
PI	22	02075	Cajazeiras do Piauí	3.379
PI	22	02083	Cajueiro da Praia	7.243
PI	22	02091	Caldeirão Grande do Piauí	5.686
PI	22	02109	Campinas do Piauí	5.429
PI	22	02117	Campo Alegre do Fidalgo	4.755
PI	22	02133	Campo Grande do Piauí	5.649
PI	22	02174	Campo Largo do Piauí	6.885
PI	22	02208	Campo Maior	45.338
PI	22	02251	Canavieira	3.907
PI	22	02307	Canto do Buriti	20.201
PI	22	02406	Capitão de Campos	11.024
PI	22	02455	Capitão Gervásio Oliveira	3.913
PI	22	02505	Caracol	10.332
PI	22	02539	Caraúbas do Piauí	5.580
PI	22	02554	Caridade do Piauí	4.871
PI	22	02604	Castelo do Piauí	18.336
PI	22	02653	Caxingó	5.108
PI	22	02703	Cocal	26.923
PI	22	02711	Cocal de Telha	4.547

PI	22	02729	Cocal dos Alves	5.605
PI	22	02737	Coivaras	3.842
PI	22	02752	Colônia do Gurguéia	6.115
PI	22	02778	Colônia do Piauí	7.447
PI	22	02802	Conceição do Canindé	4.486
PI	22	02851	Coronel José Dias	4.551
PI	22	02901	Corrente	25.575
PI	22	03008	Cristalândia do Piauí	7.904
PI	22	03107	Cristino Castro	10.036
PI	22	03206	Curimatá	10.857
PI	22	03230	Currais	4.741
PI	22	03271	Curral Novo do Piauí	4.931
PI	22	03255	Curralinhos	4.225
PI	22	03305	Demerval Lobão	13.339
PI	22	03354	Dirceu Arcoverde	6.722
PI	22	03404	Dom Expedito Lopes	6.617
PI	22	03453	Dom Inocêncio	9.271
PI	22	03420	Domingos Mourão	4.263
PI	22	03503	Elesbão Veloso	14.452
PI	22	03602	Eliseu Martins	4.702
PI	22	03701	Esperantina	38.049
PI	22	03750	Fartura do Piauí	5.104
PI	22	03800	Flores do Piauí	4.366
PI	22	03859	Floresta do Piauí	2.488
PI	22	03909	Floriano	57.928
PI	22	04006	Francinópolis	5.234
PI	22	04105	Francisco Ayres	4.419
PI	22	04154	Francisco Macedo	2.921
PI	22	04204	Francisco Santos	8.727
PI	22	04303	Fronteiras	11.202
PI	22	04352	Geminiano	5.194
PI	22	04402	Gilbués	10.416
PI	22	04501	Guadalupe	10.268
PI	22	04550	Guaribas	4.417
PI	22	04600	Hugo Napoleão	3.777
PI	22	04659	Ilha Grande	8.993
PI	22	04709	Inhuma	14.878
PI	22	04808	Ipiranga do Piauí	9.397
PI	22	04907	Isaías Coelho	8.265
PI	22	05003	Itainópolis	11.165
PI	22	05102	Itaueira	10.704
PI	22	05151	Jacobina do Piauí	5.696
PI	22	05201	Jaicós	18.202
PI	22	05250	Jardim do Mulato	4.334
PI	22	05276	Jatobá do Piauí	4.683
PI	22	05300	Jerumenha	4.381
PI	22	05359	João Costa	2.956
PI	22	05409	Joaquim Pires	13.874
PI	22	05458	Joca Marques	5.158
PI	22	05508	José de Freitas	37.410
PI	22	05516	Juazeiro do Piauí	4.775
PI	22	05524	Júlio Borges	5.407
PI	22	05532	Jurema	4.554
PI	22	05557	Lagoa Alegre	8.097
PI	22	05573	Lagoa de São Francisco	6.471
PI	22	05565	Lagoa do Barro do Piauí	4.529
PI	22	05581	Lagoa do Piauí	3.892

PI	22	05599	Lagoa do Sítio	4.905
PI	22	05540	Lagoinha do Piauí	2.689
PI	22	05607	Landri Sales	5.255
PI	22	05706	Luís Correia	28.725
PI	22	05805	Luzilândia	24.774
PI	22	05854	Madeiro	7.897
PI	22	05904	Manoel Emídio	5.218
PI	22	05953	Marcolândia	7.938
PI	22	06001	Marcos Parente	4.455
PI	22	06050	Massapê do Piauí	6.240
PI	22	06100	Matias Olímpio	10.531
PI	22	06209	Miguel Alves	32.477
PI	22	06308	Miguel Leão	1.245
PI	22	06357	Milton Brandão	6.759
PI	22	06407	Monsenhor Gil	10.335
PI	22	06506	Monsenhor Hipólito	7.440
PI	22	06605	Monte Alegre do Piauí	10.354
PI	22	06654	Morro Cabeça no Tempo	4.061
PI	22	06670	Morro do Chapéu do Piauí	6.537
PI	22	06696	Murici dos Portelas	8.591
PI	22	06704	Nazaré do Piauí	7.284
PI	22	06720	Nazária	8.149
PI	22	06753	Nossa Senhora de Nazaré	4.610
PI	22	06803	Nossa Senhora dos Remédios	8.283
PI	22	07959	Nova Santa Rita	4.211
PI	22	06902	Novo Oriente do Piauí	6.478
PI	22	06951	Novo Santo Antônio	3.295
PI	22	07009	Oeiras	35.788
PI	22	07108	Olho D'Água do Piauí	2.653
PI	22	07207	Padre Marcos	6.673
PI	22	07306	Paes Landim	4.054
PI	22	07355	Pajeú do Piauí	3.399
PI	22	07405	Palmeira do Piauí	4.978
PI	22	07504	Palmeirais	13.868
PI	22	07553	Paquetá	3.908
PI	22	07603	Parnaguá	10.348
PI	22	07702	Parnaíba	146.736
PI	22	07751	Passagem Franca do Piauí	4.573
PI	22	07777	Patos do Piauí	6.142
PI	22	07793	Pau D'Arco do Piauí	3.809
PI	22	07801	Paulistana	19.867
PI	22	07850	Pavussu	3.646
PI	22	07900	Pedro II	37.596
PI	22	07934	Pedro Laurentino	2.427
PI	22	08007	Picos	74.967
PI	22	08106	Pimenteiras	11.766
PI	22	08205	Pio IX	17.761
PI	22	08304	Piracuruca	27.766
PI	22	08403	Piripiri	61.963
PI	22	08502	Porto	11.999
PI	22	08551	Porto Alegre do Piauí	2.583
PI	22	08601	Prata do Piauí	3.083
PI	22	08650	Queimada Nova	8.617
PI	22	08700	Redenção do Gurguéia	8.448
PI	22	08809	Regeneração	17.563
PI	22	08858	Riacho Frio	4.235
PI	22	08874	Ribeira do Piauí	4.293

PI	22	08908	Ribeiro Gonçalves	6.932
PI	22	09005	Rio Grande do Piauí	6.278
PI	22	09104	Santa Cruz do Piauí	6.047
PI	22	09153	Santa Cruz dos Milagres	3.830
PI	22	09203	Santa Filomena	6.101
PI	22	09302	Santa Luz	5.570
PI	22	09377	Santa Rosa do Piauí	5.147
PI	22	09351	Santana do Piauí	4.468
PI	22	09401	Santo Antônio de Lisboa	6.073
PI	22	09450	Santo Antônio dos Milagres	2.074
PI	22	09500	Santo Inácio do Piauí	3.664
PI	22	09559	São Braz do Piauí	4.323
PI	22	09609	São Félix do Piauí	3.044
PI	22	09658	São Francisco de Assis do Piauí	5.628
PI	22	09708	São Francisco do Piauí	6.294
PI	22	09757	São Gonçalo do Gurguéia	2.864
PI	22	09807	São Gonçalo do Piauí	4.793
PI	22	09856	São João da Canabrava	4.461
PI	22	09872	São João da Fronteira	5.664
PI	22	09906	São João da Serra	6.118
PI	22	09955	São João da Varjota	4.673
PI	22	09971	São João do Arraial	7.459
PI	22	10003	São João do Piauí	19.703
PI	22	10052	São José do Divino	5.169
PI	22	10102	São José do Peixe	3.691
PI	22	10201	São José do Piauí	6.583
PI	22	10300	São Julião	5.697
PI	22	10359	São Lourenço do Piauí	4.439
PI	22	10375	São Luis do Piauí	2.567
PI	22	10383	São Miguel da Baixa Grande	2.117
PI	22	10391	São Miguel do Fidalgo	2.975
PI	22	10409	São Miguel do Tapuio	18.083
PI	22	10508	São Pedro do Piauí	13.726
PI	22	10607	São Raimundo Nonato	32.745
PI	22	10623	Sebastião Barros	3.517
PI	22	10631	Sebastião Leal	4.138
PI	22	10656	Sigefredo Pacheco	9.663
PI	22	10706	Simões	14.225
PI	22	10805	Simplício Mendes	12.166
PI	22	10904	Socorro do Piauí	4.509
PI	22	10938	Sussuapara	6.321
PI	22	10953	Tamboril do Piauí	2.780
PI	22	10979	Tanque do Piauí	2.642
PI	22	11001	Teresina	822.364
PI	22	11100	União	42.873
PI	22	11209	Uruçuí	20.390
PI	22	11308	Valença do Piauí	20.360
PI	22	11357	Várzea Branca	4.894
PI	22	11407	Várzea Grande	4.326
PI	22	11506	Vera Mendes	2.993
PI	22	11605	Vila Nova do Piauí	3.033
PI	22	11704	Wall Ferraz	4.302
CE	23	00101	Abaiara	10.659
CE	23	00150	Acarape	15.509
CE	23	00200	Acaraú	58.210
CE	23	00309	Acopiara	51.469
CE	23	00408	Aiuaba	16.338

CE	23	00507	Alcântaras	10.865
CE	23	00606	Altaneira	6.946
CE	23	00705	Alto Santo	16.434
CE	23	00754	Amontada	39.762
CE	23	00804	Antonina do Norte	7.021
CE	23	00903	Apuiarés	14.032
CE	23	01000	Aquiraz	73.562
CE	23	01109	Aracati	69.771
CE	23	01208	Aracoiaba	25.493
CE	23	01257	Ararendá	10.529
CE	23	01307	Araripe	20.768
CE	23	01406	Aratuba	11.466
CE	23	01505	Arneiroz	7.659
CE	23	01604	Assaré	22.541
CE	23	01703	Aurora	24.517
CE	23	01802	Baixio	6.050
CE	23	01851	Banabuiú	17.403
CE	23	01901	Barbalha	55.960
CE	23	01950	Barreira	19.769
CE	23	02008	Barro	21.630
CE	23	02057	Barroquinha	14.519
CE	23	02107	Baturité	33.597
CE	23	02206	Beberibe	49.846
CE	23	02305	Bela Cruz	31.072
CE	23	02404	Boa Viagem	52.667
CE	23	02503	Brejo Santo	45.708
CE	23	02602	Camocim	60.520
CE	23	02701	Campos Sales	26.579
CE	23	02800	Canindé	74.847
CE	23	02909	Capistrano	17.134
CE	23	03006	Caridade	20.359
CE	23	03105	Cariré	18.369
CE	23	03204	Caririaçu	26.433
CE	23	03303	Cariús	18.577
CE	23	03402	Carnaubal	16.863
CE	23	03501	Cascavel	66.834
CE	23	03600	Catarina	18.991
CE	23	03659	Catunda	10.004
CE	23	03709	Caucaia	330.855
CE	23	03808	Cedro	24.576
CE	23	03907	Chaval	12.650
CE	23	03931	Choró	12.919
CE	23	03956	Chorozinho	18.931
CE	23	04004	Coreaú	22.106
CE	23	04103	Crateús	72.959
CE	23	04202	Crato	122.717
CE	23	04236	Croatá	17.173
CE	23	04251	Cruz	22.687
CE	23	04269	Deputado Irapuan Pinheiro	9.150
CE	23	04277	Ererê	6.882
CE	23	04285	Eusébio	47.030
CE	23	04301	Farias Brito	18.932
CE	23	04350	Forquilha	22.116
CE	23	04400	Fortaleza	2.476.589
CE	23	04459	Fortim	15.029
CE	23	04509	Frecheirinha	13.080
CE	23	04608	General Sampaio	6.322

CE	23	04657	Graça	15.068
CE	23	04707	Granja	52.223
CE	23	04806	Granjeiro	4.590
CE	23	04905	Groaíras	10.339
CE	23	04954	Guaiúba	24.414
CE	23	05001	Guaraciaba do Norte	37.986
CE	23	05100	Guaramiranga	4.059
CE	23	05209	Hidrolândia	19.439
CE	23	05233	Horizonte	56.830
CE	23	05266	Ibaretama	12.950
CE	23	05308	Ibiapina	23.935
CE	23	05332	Ibicuitinga	11.481
CE	23	05357	Icapuí	18.572
CE	23	05407	Icó	65.682
CE	23	05506	Iguatu	97.331
CE	23	05605	Independência	25.597
CE	23	05654	Ipaporanga	11.351
CE	23	05704	Ipaumirim	12.046
CE	23	05803	Ipu	40.440
CE	23	05902	Ipueiras	37.809
CE	23	06009	Iracema	13.766
CE	23	06108	Irauçuba	22.537
CE	23	06207	Itaiçaba	7.373
CE	23	06256	Itaitinga	36.324
CE	23	06306	Itapagé	48.908
CE	23	06405	Itapipoca	117.720
CE	23	06504	Itapiúna	18.821
CE	23	06553	Itarema	38.018
CE	23	06603	Itatira	19.152
CE	23	06702	Jaguaretama	17.851
CE	23	06801	Jaguaribara	10.528
CE	23	06900	Jaguaribe	34.362
CE	23	07007	Jaguaruana	32.428
CE	23	07106	Jardim	26.710
CE	23	07205	Jati	7.691
CE	23	07254	Jijoca de Jericoacoara	17.380
CE	23	07304	Juazeiro do Norte	252.841
CE	23	07403	Jucás	23.898
CE	23	07502	Lavras da Mangabeira	31.082
CE	23	07601	Limoeiro do Norte	56.774
CE	23	07635	Madalena	18.336
CE	23	07650	Maracanaú	211.267
CE	23	07700	Maranguape	115.465
CE	23	07809	Marco	25.032
CE	23	07908	Martinópole	10.338
CE	23	08005	Massapê	35.623
CE	23	08104	Mauriti	44.543
CE	23	08203	Meruoca	13.874
CE	23	08302	Milagres	28.259
CE	23	08351	Milhã	13.074
CE	23	08377	Miraíma	12.907
CE	23	08401	Missão Velha	34.404
CE	23	08500	Mombaça	42.793
CE	23	08609	Monsenhor Tabosa	16.733
CE	23	08708	Morada Nova	61.886
CE	23	08807	Moraújo	8.149
CE	23	08906	Morrinhos	20.913

CE	23	09003	Mucambo	14.125
CE	23	09102	Mulungu	11.684
CE	23	09201	Nova Olinda	14.424
CE	23	09300	Nova Russas	31.090
CE	23	09409	Novo Oriente	27.556
CE	23	09458	Ocara	24.193
CE	23	09508	Orós	21.341
CE	23	09607	Pacajus	63.202
CE	23	09706	Pacatuba	73.881
CE	23	09805	Pacoti	11.646
CE	23	09904	Pacujá	6.012
CE	23	10001	Palhano	8.920
CE	23	10100	Palmácia	12.170
CE	23	10209	Paracuru	31.951
CE	23	10258	Paraipaba	30.393
CE	23	10308	Parambu	31.233
CE	23	10407	Paramoti	11.334
CE	23	10506	Pedra Branca	41.979
CE	23	10605	Penaforte	8.319
CE	23	10704	Pentecoste	35.615
CE	23	10803	Pereiro	15.798
CE	23	10852	Pindoretama	18.970
CE	23	10902	Piquet Carneiro	15.647
CE	23	10951	Pires Ferreira	10.292
CE	23	11009	Poranga	12.022
CE	23	11108	Porteiras	15.016
CE	23	11207	Potengi	10.364
CE	23	11231	Potiretama	6.154
CE	23	11264	Quiterianópolis	20.042
CE	23	11306	Quixadá	81.445
CE	23	11355	Quixelô	14.955
CE	23	11405	Quixeramobim	72.866
CE	23	11504	Quixeré	19.608
CE	23	11603	Redenção	26.540
CE	23	11702	Reriutaba	19.315
CE	23	11801	Russas	70.794
CE	23	11900	Saboeiro	15.716
CE	23	11959	Salitre	15.571
CE	23	12205	Santa Quitéria	42.793
CE	23	12007	Santana do Acaraú	30.234
CE	23	12106	Santana do Cariri	17.195
CE	23	12304	São Benedito	44.507
CE	23	12403	São Gonçalo do Amarante	44.526
CE	23	12502	São João do Jaguaribe	7.843
CE	23	12601	São Luís do Curu	12.397
CE	23	12700	Senador Pompeu	26.425
CE	23	12809	Senador Sá	6.948
CE	23	12908	Sobral	190.724
CE	23	13005	Solonópole	17.718
CE	23	13104	Tabuleiro do Norte	29.366
CE	23	13203	Tamboril	25.424
CE	23	13252	Tarrafas	8.887
CE	23	13302	Tauá	56.017
CE	23	13351	Tejuçuoca	17.081
CE	23	13401	Tianguá	69.723
CE	23	13500	Trairi	51.952
CE	23	13559	Tururu	14.632

CE	23	13609	Ubajara	32.148
CE	23	13708	Umari	7.554
CE	23	13757	Umirim	18.914
CE	23	13807	Uruburetama	20.031
CE	23	13906	Uruoca	12.991
CE	23	13955	Varjota	17.670
CE	23	14003	Várzea Alegre	38.698
CE	23	14102	Viçosa do Ceará	55.687
RN	24	00109	Acari	11.024
RN	24	00208	Açu	53.636
RN	24	00307	Afonso Bezerra	10.843
RN	24	00406	Água Nova	3.004
RN	24	00505	Alexandria	13.487
RN	24	00604	Almino Afonso	4.847
RN	24	00703	Alto do Rodrigues	12.521
RN	24	00802	Angicos	11.544
RN	24	00901	Antônio Martins	6.919
RN	24	01008	Apodi	34.809
RN	24	01107	Areia Branca	25.529
RN	24	01206	Arês	13.047
RN	24	01305	Augusto Severo	9.310
RN	24	01404	Baía Formosa	8.631
RN	24	01453	Baraúna	24.586
RN	24	01503	Barcelona	3.947
RN	24	01602	Bento Fernandes	5.145
RN	24	01651	Bodó	2.399
RN	24	01701	Bom Jesus	9.504
RN	24	01800	Brejinho	11.675
RN	24	01859	Caiçara do Norte	6.030
RN	24	01909	Caiçara do Rio do Vento	3.342
RN	24	02006	Caicó	63.148
RN	24	02105	Campo Redondo	10.348
RN	24	02204	Canguaretama	31.216
RN	24	02303	Caraúbas	19.635
RN	24	02402	Carnaúba dos Dantas	7.495
RN	24	02501	Carnaubais	9.883
RN	24	02600	Ceará-Mirim	68.580
RN	24	02709	Cerro Corá	10.922
RN	24	02808	Coronel Ezequiel	5.405
RN	24	02907	Coronel João Pessoa	4.778
RN	24	03004	Cruzeta	7.954
RN	24	03103	Currais Novos	42.795
RN	24	03202	Doutor Severiano	6.488
RN	24	03301	Encanto	5.265
RN	24	03400	Equador	5.835
RN	24	03509	Espírito Santo	10.457
RN	24	03608	Extremoz	24.953
RN	24	03707	Felipe Guerra	5.750
RN	24	03756	Fernando Pedroza	2.870
RN	24	03806	Florânia	8.958
RN	24	03905	Francisco Dantas	2.863
RN	24	04002	Frutuoso Gomes	4.207
RN	24	04101	Galinhos	2.223
RN	24	04200	Goianinha	22.851
RN	24	04309	Governador Dix-Sept Rosado	12.421
RN	24	04408	Grossos	9.481
RN	24	04507	Guamaré	12.731

RN	24	04606	Ielmo Marinho	12.319
RN	24	04705	Ipanguaçu	14.005
RN	24	04804	Ipueira	2.091
RN	24	04853	Itajá	6.985
RN	24	04903	Itaú	5.587
RN	24	05009	Jaçanã	8.040
RN	24	05108	Jandaíra	6.820
RN	24	05207	Janduís	5.326
RN	24	05306	Januário Cicco	9.113
RN	24	05405	Japi	5.461
RN	24	05504	Jardim de Angicos	2.603
RN	24	05603	Jardim de Piranhas	13.623
RN	24	05702	Jardim do Seridó	12.119
RN	24	05801	João Câmara	32.456
RN	24	05900	João Dias	2.602
RN	24	06007	José da Penha	5.865
RN	24	06106	Jucurutu	17.721
RN	24	06155	Jundiá	3.609
RN	24	06205	Lagoa d'Anta	6.273
RN	24	06304	Lagoa de Pedras	7.035
RN	24	06403	Lagoa de Velhos	2.670
RN	24	06502	Lagoa Nova	14.131
RN	24	06601	Lagoa Salgada	7.623
RN	24	06700	Lajes	10.457
RN	24	06809	Lajes Pintadas	4.619
RN	24	06908	Lucrécia	3.665
RN	24	07005	Luís Gomes	9.646
RN	24	07104	Macaíba	70.587
RN	24	07203	Macau	29.204
RN	24	07252	Major Sales	3.582
RN	24	07302	Marcelino Vieira	8.257
RN	24	07401	Martins	8.256
RN	24	07500	Maxaranguape	10.629
RN	24	07609	Messias Targino	4.225
RN	24	07708	Montanhas	11.372
RN	24	07807	Monte Alegre	20.825
RN	24	07906	Monte das Gameleiras	2.240
RN	24	08003	Mossoró	263.344
RN	24	08102	Natal	810.780
RN	24	08201	Nísia Floresta	24.149
RN	24	08300	Nova Cruz	35.618
RN	24	08409	Olho-d'Água do Borges	4.283
RN	24	08508	Ouro Branco	4.702
RN	24	08607	Paraná	3.977
RN	24	08706	Paraú	3.842
RN	24	08805	Parazinho	4.885
RN	24	08904	Parelhas	20.434
RN	24	03251	Parnamirim	208.426
RN	24	09100	Passa e Fica	11.313
RN	24	09209	Passagem	2.910
RN	24	09308	Patu	12.025
RN	24	09407	Pau dos Ferros	27.975
RN	24	09506	Pedra Grande	3.483
RN	24	09605	Pedra Preta	2.571
RN	24	09704	Pedro Avelino	7.107
RN	24	09803	Pedro Velho	14.160
RN	24	09902	Pendências	13.588

RN	24	10009	Pilões	3.488
RN	24	10108	Poço Branco	14.079
RN	24	10207	Portalegre	7.365
RN	24	10256	Porto do Mangue	5.306
RN	24	10306	Presidente Juscelino	8.904
RN	24	10405	Pureza	8.537
RN	24	10504	Rafael Fernandes	4.727
RN	24	10603	Rafael Godeiro	3.072
RN	24	10702	Riacho da Cruz	3.204
RN	24	10801	Riacho de Santana	4.153
RN	24	10900	Riachuelo	7.168
RN	24	08953	Rio do Fogo	10.124
RN	24	11007	Rodolfo Fernandes	4.415
RN	24	11106	Ruy Barbosa	3.589
RN	24	11205	Santa Cruz	36.143
RN	24	09332	Santa Maria	4.838
RN	24	11403	Santana do Matos	13.642
RN	24	11429	Santana do Seridó	2.538
RN	24	11502	Santo Antônio	22.378
RN	24	11601	São Bento do Norte	2.945
RN	24	11700	São Bento do Trairí	3.956
RN	24	11809	São Fernando	3.414
RN	24	11908	São Francisco do Oeste	3.905
RN	24	12005	São Gonçalo do Amarante	89.045
RN	24	12104	São João do Sabugi	5.940
RN	24	12203	São José de Mipibu	40.150
RN	24	12302	São José do Campestre	12.385
RN	24	12401	São José do Seridó	4.266
RN	24	12500	São Miguel	22.314
RN	24	12559	São Miguel do Gostoso	8.754
RN	24	12609	São Paulo do Potengi	15.999
RN	24	12708	São Pedro	6.194
RN	24	12807	São Rafael	8.105
RN	24	12906	São Tomé	10.830
RN	24	13003	São Vicente	6.059
RN	24	13102	Senador Elói de Souza	5.684
RN	24	13201	Senador Georgino Avelino	3.972
RN	24	13300	Serra de São Bento	5.734
RN	24	13359	Serra do Mel	10.445
RN	24	13409	Serra Negra do Norte	7.788
RN	24	13508	Serrinha	6.530
RN	24	13557	Serrinha dos Pintos	4.559
RN	24	13607	Severiano Melo	5.801
RN	24	13706	Sítio Novo	5.064
RN	24	13805	Taboleiro Grande	2.340
RN	24	13904	Taipu	11.860
RN	24	14001	Tangará	14.333
RN	24	14100	Tenente Ananias	9.961
RN	24	14159	Tenente Laurentino Cruz	5.483
RN	24	11056	Tibau	3.725
RN	24	14209	Tibau do Sul	11.665
RN	24	14308	Timbaúba dos Batistas	2.304
RN	24	14407	Touros	31.336
RN	24	14456	Triunfo Potiguar	3.347
RN	24	14506	Umarizal	10.626
RN	24	14605	Upanema	13.146
RN	24	14704	Várzea	5.254

RN	24	14753	Venha-Ver	3.852
RN	24	14803	Vera Cruz	10.888
RN	24	14902	Viçosa	1.626
RN	24	15008	Vila Flor	2.899
PB	25	00106	Água Branca	9.532
PB	25	00205	Aguiar	5.522
PB	25	00304	Alagoa Grande	28.427
PB	25	00403	Alagoa Nova	19.766
PB	25	00502	Alagoinha	13.660
PB	25	00536	Alcantil	5.261
PB	25	00577	Algodão de Jandaíra	2.379
PB	25	00601	Alhandra	18.168
PB	25	00734	Amparo	2.104
PB	25	00775	Aparecida	7.756
PB	25	00809	Araçagi	17.158
PB	25	00908	Arara	12.738
PB	25	01005	Araruna	19.054
PB	25	01104	Areia	23.653
PB	25	01153	Areia de Baraúnas	1.914
PB	25	01203	Areial	6.504
PB	25	01302	Aroeiras	19.049
PB	25	01351	Assunção	3.566
PB	25	01401	Baía da Traição	8.130
PB	25	01500	Bananeiras	21.801
PB	25	01534	Baraúna	4.301
PB	25	01609	Barra de Santa Rosa	14.287
PB	25	01575	Barra de Santana	8.198
PB	25	01708	Barra de São Miguel	5.646
PB	25	01807	Bayeux	100.137
PB	25	01906	Belém	17.131
PB	25	02003	Belém do Brejo do Cruz	7.154
PB	25	02052	Bernardino Batista	3.115
PB	25	02102	Boa Ventura	5.687
PB	25	02151	Boa Vista	6.323
PB	25	02201	Bom Jesus	2.416
PB	25	02300	Bom Sucesso	5.016
PB	25	02409	Bonito de Santa Fé	10.925
PB	25	02508	Boqueirão	16.967
PB	25	02706	Borborema	5.141
PB	25	02805	Brejo do Cruz	13.220
PB	25	02904	Brejo dos Santos	6.218
PB	25	03001	Caaporã	20.510
PB	25	03100	Cabaceiras	5.093
PB	25	03209	Cabedelo	59.104
PB	25	03308	Cachoeira dos Índios	9.617
PB	25	03407	Cacimba de Areia	3.574
PB	25	03506	Cacimba de Dentro	16.743
PB	25	03555	Cacimbas	6.847
PB	25	03605	Caiçara	7.212
PB	25	03704	Cajazeiras	58.794
PB	25	03753	Cajazeirinhas	3.048
PB	25	03803	Caldas Brandão	5.674
PB	25	03902	Camalaú	5.771
PB	25	04009	Campina Grande	387.644
PB	25	04033	Capim	5.711
PB	25	04074	Caraúbas	3.925
PB	25	04108	Carrapateira	2.410

PB	25	04157	Casserengue	7.096
PB	25	04207	Catingueira	4.817
PB	25	04306	Catolé do Rocha	28.922
PB	25	04355	Caturité	4.571
PB	25	04405	Conceição	18.397
PB	25	04504	Condado	6.591
PB	25	04603	Conde	21.783
PB	25	04702	Congo	4.690
PB	25	04801	Coremas	15.151
PB	25	04850	Coxixola	1.787
PB	25	04900	Cruz do Espírito Santo	16.158
PB	25	05006	Cubati	6.903
PB	25	05105	Cuité	19.981
PB	25	05238	Cuité de Mamanguape	6.208
PB	25	05204	Cuitegi	6.861
PB	25	05279	Curral de Cima	5.201
PB	25	05303	Curral Velho	2.501
PB	25	05352	Damião	4.946
PB	25	05402	Desterro	8.014
PB	25	05600	Diamante	6.593
PB	25	05709	Dona Inês	10.477
PB	25	05808	Duas Estradas	3.625
PB	25	05907	Emas	3.337
PB	25	06004	Esperança	31.320
PB	25	06103	Fagundes	11.368
PB	25	06202	Frei Martinho	2.934
PB	25	06251	Gado Bravo	8.365
PB	25	06301	Guarabira	55.657
PB	25	06400	Gurinhém	13.875
PB	25	06509	Gurjão	3.188
PB	25	06608	Ibiara	6.004
PB	25	02607	Igaracy	6.145
PB	25	06707	Imaculada	11.388
PB	25	06806	Ingá	18.235
PB	25	06905	Itabaiana	24.426
PB	25	07002	Itaporanga	23.351
PB	25	07101	Itapororoca	17.179
PB	25	07200	Itatuba	10.265
PB	25	07309	Jacaraú	13.967
PB	25	07408	Jericó	7.548
PB	25	07507	João Pessoa	733.155
PB	25	13653	Joca Claudino*	2.619
PB	25	07606	Juarez Távora	7.506
PB	25	07705	Juazeirinho	16.923
PB	25	07804	Junco do Seridó	6.695
PB	25	07903	Juripiranga	10.283
PB	25	08000	Juru	9.810
PB	25	08109	Lagoa	4.669
PB	25	08208	Lagoa de Dentro	7.392
PB	25	08307	Lagoa Seca	26.035
PB	25	08406	Lastro	2.820
PB	25	08505	Livramento	7.177
PB	25	08554	Logradouro	3.985
PB	25	08604	Lucena	11.882
PB	25	08703	Mãe d'Água	4.009
PB	25	08802	Malta	5.607
PB	25	08901	Mamanguape	42.602

PB	25	09008	Manaíra	10.781
PB	25	09057	Marcação	7.717
PB	25	09107	Mari	21.216
PB	25	09156	Marizópolis	6.216
PB	25	09206	Massaranduba	12.995
PB	25	09305	Mataraca	7.526
PB	25	09339	Matinhas	4.340
PB	25	09370	Mato Grosso	2.724
PB	25	09396	Maturéia	6.009
PB	25	09404	Mogeiro	12.468
PB	25	09503	Montadas	5.069
PB	25	09602	Monte Horebe	4.539
PB	25	09701	Monteiro	31.095
PB	25	09800	Mulungu	9.506
PB	25	09909	Natuba	10.567
PB	25	10006	Nazarezinho	7.266
PB	25	10105	Nova Floresta	10.524
PB	25	10204	Nova Olinda	6.041
PB	25	10303	Nova Palmeira	4.422
PB	25	10402	Olho d'Água	6.862
PB	25	10501	Olivedos	3.661
PB	25	10600	Ouro Velho	2.937
PB	25	10659	Parari	1.243
PB	25	10709	Passagem	2.253
PB	25	10808	Patos	101.359
PB	25	10907	Paulista	11.829
PB	25	11004	Pedra Branca	3.724
PB	25	11103	Pedra Lavrada	7.541
PB	25	11202	Pedras de Fogo	27.389
PB	25	12721	Pedro Régis	5.795
PB	25	11301	Piancó	15.511
PB	25	11400	Picuí	18.248
PB	25	11509	Pilar	11.262
PB	25	11608	Pilões	6.915
PB	25	11707	Pilõezinhos	5.134
PB	25	11806	Pirpirituba	10.336
PB	25	11905	Pitimbu	17.262
PB	25	12002	Pocinhos	17.198
PB	25	12036	Poço Dantas	3.746
PB	25	12077	Poço de José de Moura	4.013
PB	25	12101	Pombal	32.122
PB	25	12200	Prata	3.887
PB	25	12309	Princesa Isabel	21.518
PB	25	12408	Puxinanã	12.996
PB	25	12507	Queimadas	41.298
PB	25	12606	Quixabá	1.730
PB	25	12705	Remígio	17.786
PB	25	12747	Riachão	3.303
PB	25	12754	Riachão do Bacamarte	4.289
PB	25	12762	Riachão do Poço	4.201
PB	25	12788	Riacho de Santo Antônio	1.752
PB	25	12804	Riacho dos Cavalos	8.334
PB	25	12903	Rio Tinto	23.028
PB	25	13000	Salgadinho	3.561
PB	25	13109	Salgado de São Félix	11.971
PB	25	13158	Santa Cecília	6.643
PB	25	13208	Santa Cruz	6.471

PB	25	13307	Santa Helena	5.352
PB	25	13356	Santa Inês	3.539
PB	25	13406	Santa Luzia	14.774
PB	25	13703	Santa Rita	121.166
PB	25	13802	Santa Teresinha	4.570
PB	25	13505	Santana de Mangueira	5.298
PB	25	13604	Santana dos Garrotes	7.219
PB	25	13851	Santo André	2.626
PB	25	13927	São Bentinho	4.181
PB	25	13901	São Bento	31.237
PB	25	13968	São Domingos de Pombal	2.883
PB	25	13943	São Domingos do Cariri	2.438
PB	25	13984	São Francisco	3.357
PB	25	14008	São João do Cariri	4.329
PB	25	00700	São João do Rio do Peixe	18.199
PB	25	14107	São João do Tigre	4.390
PB	25	14206	São José da Lagoa Tapada	7.562
PB	25	14305	São José de Caiana	6.031
PB	25	14404	São José de Espinharas	4.734
PB	25	14503	São José de Piranhas	19.190
PB	25	14552	São José de Princesa	4.162
PB	25	14602	São José do Bonfim	3.269
PB	25	14651	São José do Brejo do Cruz	1.696
PB	25	14701	São José do Sabugi	4.019
PB	25	14800	São José dos Cordeiros	3.974
PB	25	14453	São José dos Ramos	5.555
PB	25	14909	São Mamede	7.728
PB	25	15005	São Miguel de Taipu	6.743
PB	25	15104	São Sebastião de Lagoa de Roça	11.119
PB	25	15203	São Sebastião do Umbuzeiro	3.262
PB	25	15302	Sapé	50.358
PB	25	15401	Seridó	10.317
PB	25	15500	Serra Branca	13.038
PB	25	15609	Serra da Raiz	3.187
PB	25	15708	Serra Grande	2.985
PB	25	15807	Serra Redonda	7.031
PB	25	15906	Serraria	6.206
PB	25	15930	Sertãozinho	4.468
PB	25	15971	Sobrado	7.411
PB	25	16003	Solânea	26.505
PB	25	16102	Soledade	13.868
PB	25	16151	Sossêgo	3.213
PB	25	16201	Sousa	66.136
PB	25	16300	Sumé	16.139
PB	25	16409	Tacima *	10.330
PB	25	16508	Taperoá	15.016
PB	25	16607	Tavares	14.143
PB	25	16706	Teixeira	14.255
PB	25	16755	Tenório	2.840
PB	25	16805	Triunfo	9.233
PB	25	16904	Uiraúna	14.654
PB	25	17001	Umbuzeiro	9.307
PB	25	17100	Várzea	2.539
PB	25	17209	Vieirópolis	5.074
PB	25	05501	Vista Serrana	3.543
PB	25	17407	Zabelê	2.093
PE	26	00054	Abreu e Lima	94.843

PE	26	00104	Afogados da Ingazeira	35.255
PE	26	00203	Afrânio	17.784
PE	26	00302	Agrestina	22.882
PE	26	00401	Água Preta	33.446
PE	26	00500	Águas Belas	40.511
PE	26	00609	Alagoinha	13.853
PE	26	00708	Aliança	37.433
PE	26	00807	Altinho	22.371
PE	26	00906	Amaraji	21.988
PE	26	01003	Angelim	10.288
PE	26	01052	Araçoiaba	18.390
PE	26	01102	Araripina	77.794
PE	26	01201	Arcoverde	69.346
PE	26	01300	Barra de Guabiraba	12.918
PE	26	01409	Barreiros	40.855
PE	26	01508	Belém de Maria	11.409
PE	26	01607	Belém de São Francisco	20.257
PE	26	01706	Belo Jardim	72.719
PE	26	01805	Betânia	12.057
PE	26	01904	Bezerros	58.768
PE	26	02001	Bodocó	35.422
PE	26	02100	Bom Conselho	45.747
PE	26	02209	Bom Jardim	37.889
PE	26	02308	Bonito	37.552
PE	26	02407	Brejão	8.839
PE	26	02506	Brejinho	7.310
PE	26	02605	Brejo da Madre de Deus	45.723
PE	26	02704	Buenos Aires	12.578
PE	26	02803	Buíque	52.715
PE	26	02902	Cabo de Santo Agostinho	187.159
PE	26	03009	Cabrobó	31.191
PE	26	03108	Cachoeirinha	18.956
PE	26	03207	Caetés	26.765
PE	26	03306	Calçado	11.088
PE	26	03405	Calumbi	5.646
PE	26	03454	Camaragibe	145.676
PE	26	03504	Camocim de São Félix	17.257
PE	26	03603	Camutanga	8.180
PE	26	03702	Canhotinho	24.491
PE	26	03801	Capoeiras	19.596
PE	26	03900	Carnaíba	18.642
PE	26	03926	Carnaubeira da Penha	11.888
PE	26	04007	Carpina	75.706
PE	26	04106	Caruaru	319.580
PE	26	04155	Casinhas	13.799
PE	26	04205	Catende	38.324
PE	26	04304	Cedro	10.873
PE	26	04403	Chã de Alegria	12.504
PE	26	04502	Chã Grande	20.270
PE	26	04601	Condado	24.473
PE	26	04700	Correntes	17.491
PE	26	04809	Cortês	12.435
PE	26	04908	Cumaru	17.329
PE	26	05004	Cupira	23.468
PE	26	05103	Custódia	34.154
PE	26	05152	Dormentes	17.110
PE	26	05202	Escada	63.963

PE	26	05301	Exu	31.576
PE	26	05400	Feira Nova	20.703
PE	26	05459	Fernando de Noronha	2.675
PE	26	05509	Ferreiros	11.484
PE	26	05608	Flores	22.166
PE	26	05707	Floresta	29.635
PE	26	05806	Frei Miguelinho	14.394
PE	26	05905	Gameleira	28.213
PE	26	06002	Garanhuns	130.303
PE	26	06101	Glória do Goitá	29.132
PE	26	06200	Goiana	75.987
PE	26	06309	Granito	6.913
PE	26	06408	Gravatá	77.164
PE	26	06507	Iati	18.412
PE	26	06606	Ibimirim	27.155
PE	26	06705	Ibirajuba	7.542
PE	26	06804	Igarassu	103.537
PE	26	06903	Iguaraci	11.802
PE	26	07604	Ilha de Itamaracá	22.347
PE	26	07000	Inajá	19.527
PE	26	07109	Ingazeira	4.491
PE	26	07208	Ipojuca	82.277
PE	26	07307	Ipubi	28.510
PE	26	07406	Itacuruba	4.423
PE	26	07505	Itaíba	26.215
PE	26	07653	Itambé	35.430
PE	26	07703	Itapetim	13.814
PE	26	07752	Itapissuma	24.050
PE	26	07802	Itaquitinga	15.749
PE	26	07901	Jaboatão dos Guararapes	649.788
PE	26	07950	Jaqueira	11.490
PE	26	08008	Jataúba	15.909
PE	26	08057	Jatobá	14.026
PE	26	08107	João Alfredo	31.029
PE	26	08206	Joaquim Nabuco	15.762
PE	26	08255	Jucati	10.674
PE	26	08305	Jupi	13.804
PE	26	08404	Jurema	14.603
PE	26	08453	Lagoa do Carro	16.211
PE	26	08503	Lagoa do Itaenga	20.697
PE	26	08602	Lagoa do Ouro	12.221
PE	26	08701	Lagoa dos Gatos	15.649
PE	26	08750	Lagoa Grande	23.039
PE	26	08800	Lajedo	36.968
PE	26	08909	Limoeiro	55.391
PE	26	09006	Macaparana	24.035
PE	26	09105	Machados	13.857
PE	26	09154	Manari	18.472
PE	26	09204	Maraial	12.093
PE	26	09303	Mirandiba	14.400
PE	26	14303	Moreilândia	11.134
PE	26	09402	Moreno	57.271
PE	26	09501	Nazaré da Mata	30.915
PE	26	09600	Olinda	378.538
PE	26	09709	Orobó	22.938
PE	26	09808	Orocó	13.361
PE	26	09907	Ouricuri	64.944

PE	26	10004	Palmares	59.813
PE	26	10103	Palmeirina	8.086
PE	26	10202	Panelas	25.628
PE	26	10301	Paranatama	11.052
PE	26	10400	Parnamirim	20.327
PE	26	10509	Passira	28.590
PE	26	10608	Paudalho	51.835
PE	26	10707	Paulista	303.401
PE	26	10806	Pedra	20.998
PE	26	10905	Pesqueira	63.320
PE	26	11002	Petrolândia	32.889
PE	26	11101	Petrolina	299.752
PE	26	11200	Poção	11.247
PE	26	11309	Pombos	24.100
PE	26	11408	Primavera	13.560
PE	26	11507	Quipapá	24.343
PE	26	11533	Quixaba	6.731
PE	26	11606	Recife	1.546.516
PE	26	11705	Riacho das Almas	19.277
PE	26	11804	Ribeirão	44.728
PE	26	11903	Rio Formoso	22.258
PE	26	12000	Sairé	11.056
PE	26	12109	Salgadinho	9.479
PE	26	12208	Salgueiro	56.992
PE	26	12307	Saloá	15.333
PE	26	12406	Sanharó	22.434
PE	26	12455	Santa Cruz	13.773
PE	26	12471	Santa Cruz da Baixa Verde	11.836
PE	26	12505	Santa Cruz do Capibaribe	89.773
PE	26	12554	Santa Filomena	13.468
PE	26	12604	Santa Maria da Boa Vista	39.629
PE	26	12703	Santa Maria do Cambucá	13.120
PE	26	12802	Santa Terezinha	11.048
PE	26	12901	São Benedito do Sul	14.137
PE	26	13008	São Bento do Una	53.847
PE	26	13107	São Caitano	35.416
PE	26	13206	São João	21.433
PE	26	13305	São Joaquim do Monte	20.538
PE	26	13404	São José da Coroa Grande	18.504
PE	26	13503	São José do Belmonte	32.692
PE	26	13602	São José do Egito	32.011
PE	26	13701	São Lourenço da Mata	103.854
PE	26	13800	São Vicente Ferrer	17.077
PE	26	13909	Serra Talhada	79.871
PE	26	14006	Serrita	18.369
PE	26	14105	Sertânia	33.951
PE	26	14204	Sirinhaém	40.853
PE	26	14402	Solidão	5.761
PE	26	14501	Surubim	59.144
PE	26	14600	Tabira	26.609
PE	26	14709	Tacaimbó	12.710
PE	26	14808	Tacaratu	22.450
PE	26	14857	Tamandaré	20.979
PE	26	15003	Taquaritinga do Norte	25.298
PE	26	15102	Terezinha	6.771
PE	26	15201	Terra Nova	9.409
PE	26	15300	Timbaúba	53.589

PE	26	15409	Toritama	36.610
PE	26	15508	Tracunhaém	13.106
PE	26	15607	Trindade	26.438
PE	26	15706	Triunfo	14.997
PE	26	15805	Tupanatinga	24.704
PE	26	15904	Tuparetama	7.938
PE	26	16001	Venturosa	16.251
PE	26	16100	Verdejante	9.165
PE	26	16183	Vertente do Lério	7.823
PE	26	16209	Vertentes	18.473
PE	26	16308	Vicência	30.879
PE	26	16407	Vitória de Santo Antão	130.924
PE	26	16506	Xexéu	14.132
AL	27	00102	Água Branca	19.433
AL	27	00201	Anadia	17.392
AL	27	00300	Arapiraca	216.108
AL	27	00409	Atalaia	44.612
AL	27	00508	Barra de Santo Antônio	14.451
AL	27	00607	Barra de São Miguel	7.666
AL	27	00706	Batalha	17.251
AL	27	00805	Belém	4.446
AL	27	00904	Belo Monte	7.043
AL	27	01001	Boca da Mata	25.895
AL	27	01100	Branquinha	10.527
AL	27	01209	Cacimbinhas	10.245
AL	27	01308	Cajueiro	20.520
AL	27	01357	Campestre	6.627
AL	27	01407	Campo Alegre	51.584
AL	27	01506	Campo Grande	9.046
AL	27	01605	Canapi	17.244
AL	27	01704	Capela	16.953
AL	27	01803	Carneiros	8.421
AL	27	01902	Chã Preta	7.146
AL	27	02009	Coité do Nóia	10.845
AL	27	02108	Colônia Leopoldina	20.213
AL	27	02207	Coqueiro Seco	5.557
AL	27	02306	Coruripe	52.716
AL	27	02355	Craíbas	22.784
AL	27	02405	Delmiro Gouveia	48.493
AL	27	02504	Dois Riachos	10.866
AL	27	02553	Estrela de Alagoas	17.332
AL	27	02603	Feira Grande	21.338
AL	27	02702	Feliz Deserto	4.385
AL	27	02801	Flexeiras	12.352
AL	27	02900	Girau do Ponciano	37.118
AL	27	03007	Ibateguara	15.165
AL	27	03106	Igaci	25.158
AL	27	03205	Igreja Nova	23.434
AL	27	03304	Inhapi	17.908
AL	27	03403	Jacaré dos Homens	5.357
AL	27	03502	Jacuípe	6.973
AL	27	03601	Japaratinga	7.823
AL	27	03700	Jaramataia	5.541
AL	27	03759	Jequiá da Praia	11.957
AL	27	03809	Joaquim Gomes	22.717
AL	27	03908	Jundiá	4.172
AL	27	04005	Junqueiro	23.824

AL	27	04104	Lagoa da Canoa	18.117
AL	27	04203	Limoeiro de Anadia	27.215
AL	27	04302	Maceió	943.110
AL	27	04401	Major Isidoro	18.994
AL	27	04906	Mar Vermelho	3.620
AL	27	04500	Maragogi	29.280
AL	27	04609	Maravilha	10.023
AL	27	04708	Marechal Deodoro	46.754
AL	27	04807	Maribondo	13.502
AL	27	05002	Mata Grande	24.673
AL	27	05101	Matriz de Camaragibe	23.768
AL	27	05200	Messias	15.966
AL	27	05309	Minador do Negrão	5.263
AL	27	05408	Monteirópolis	6.945
AL	27	05507	Murici	26.867
AL	27	05606	Novo Lino	12.184
AL	27	05705	Olho d'Água das Flores	20.437
AL	27	05804	Olho d'Água do Casado	8.601
AL	27	05903	Olho d'Água Grande	4.966
AL	27	06000	Olivença	11.100
AL	27	06109	Ouro Branco	10.977
AL	27	06208	Palestina	5.158
AL	27	06307	Palmeira dos Índios	70.556
AL	27	06406	Pão de Açúcar	23.730
AL	27	06422	Pariconha	10.274
AL	27	06448	Paripueira	11.601
AL	27	06505	Passo de Camaragibe	14.783
AL	27	06604	Paulo Jacinto	7.419
AL	27	06703	Penedo	60.638
AL	27	06802	Piaçabuçu	17.236
AL	27	06901	Pilar	33.467
AL	27	07008	Pindoba	2.862
AL	27	07107	Piranhas	23.279
AL	27	07206	Poço das Trincheiras	13.922
AL	27	07305	Porto Calvo	25.843
AL	27	07404	Porto de Pedras	8.291
AL	27	07503	Porto Real do Colégio	19.410
AL	27	07602	Quebrangulo	11.404
AL	27	07701	Rio Largo	68.885
AL	27	07800	Roteiro	6.631
AL	27	07909	Santa Luzia do Norte	6.930
AL	27	08006	Santana do Ipanema	45.197
AL	27	08105	Santana do Mundaú	10.875
AL	27	08204	São Brás	6.731
AL	27	08303	São José da Laje	22.798
AL	27	08402	São José da Tapera	30.323
AL	27	08501	São Luís do Quitunde	32.633
AL	27	08600	São Miguel dos Campos	55.463
AL	27	08709	São Miguel dos Milagres	7.264
AL	27	08808	São Sebastião	32.232
AL	27	08907	Satuba	14.815
AL	27	08956	Senador Rui Palmeira	13.129
AL	27	09004	Tanque d'Arca	6.086
AL	27	09103	Taquarana	19.172
AL	27	09152	Teotônio Vilela	41.480
AL	27	09202	Traipu	25.880
AL	27	09301	União dos Palmares	62.645

AL	27	09400	Viçosa	25.342
SE	28	00100	Amparo de São Francisco	2.283
SE	28	00209	Aquidabã	20.188
SE	28	00308	Aracaju	579.563
SE	28	00407	Arauá	10.772
SE	28	00506	Areia Branca	17.014
SE	28	00605	Barra dos Coqueiros	25.527
SE	28	00670	Boquim	25.632
SE	28	00704	Brejo Grande	7.792
SE	28	01009	Campo do Brito	16.870
SE	28	01108	Canhoba	3.956
SE	28	01207	Canindé de São Francisco	25.219
SE	28	01306	Capela	31.087
SE	28	01405	Carira	20.179
SE	28	01504	Carmópolis	13.822
SE	28	01603	Cedro de São João	5.653
SE	28	01702	Cristinápolis	16.692
SE	28	01900	Cumbe	3.826
SE	28	02007	Divina Pastora	4.408
SE	28	02106	Estância	64.825
SE	28	02205	Feira Nova	5.344
SE	28	02304	Frei Paulo	14.020
SE	28	02403	Gararu	11.409
SE	28	02502	General Maynard	2.970
SE	28	02601	Gracho Cardoso	5.655
SE	28	02700	Ilha das Flores	8.354
SE	28	02809	Indiaroba	16.037
SE	28	02908	Itabaiana	87.747
SE	28	03005	Itabaianinha	39.176
SE	28	03104	Itabi	4.957
SE	28	03203	Itaporanga d'Ajuda	30.798
SE	28	03302	Japaratuba	17.042
SE	28	03401	Japoatã	12.932
SE	28	03500	Lagarto	95.746
SE	28	03609	Laranjeiras	27.177
SE	28	03708	Macambira	6.447
SE	28	03807	Malhada dos Bois	3.476
SE	28	03906	Malhador	12.086
SE	28	04003	Maruim	16.412
SE	28	04102	Moita Bonita	11.020
SE	28	04201	Monte Alegre de Sergipe	13.784
SE	28	04300	Muribeca	7.363
SE	28	04409	Neópolis	18.500
SE	28	04458	Nossa Senhora Aparecida	8.526
SE	28	04508	Nossa Senhora da Glória	32.926
SE	28	04607	Nossa Senhora das Dores	24.764
SE	28	04706	Nossa Senhora de Lourdes	6.255
SE	28	04805	Nossa Senhora do Socorro	163.047
SE	28	04904	Pacatuba	13.260
SE	28	05000	Pedra Mole	3.001
SE	28	05109	Pedrinhas	8.903
SE	28	05208	Pinhão	6.029
SE	28	05307	Pirambu	8.455
SE	28	05406	Poço Redondo	31.253
SE	28	05505	Poço Verde	22.138
SE	28	05604	Porto da Folha	27.260
SE	28	05703	Propriá	28.533

SE	28	05802	Riachão do Dantas	19.401
SE	28	05901	Riachuelo	9.434
SE	28	06008	Ribeirópolis	17.307
SE	28	06107	Rosário do Catete	9.384
SE	28	06206	Salgado	19.403
SE	28	06305	Santa Luzia do Itanhy	13.064
SE	28	06503	Santa Rosa de Lima	3.761
SE	28	06404	Santana do São Francisco	7.108
SE	28	06602	Santo Amaro das Brotas	11.467
SE	28	06701	São Cristóvão	79.956
SE	28	06800	São Domingos	10.349
SE	28	06909	São Francisco	3.460
SE	28	07006	São Miguel do Aleixo	3.718
SE	28	07105	Simão Dias	38.847
SE	28	07204	Siriri	8.088
SE	28	07303	Telha	2.982
SE	28	07402	Tobias Barreto	48.414
SE	28	07501	Tomar do Geru	12.857
SE	28	07600	Umbaúba	22.709
BA	29	00108	Abaíra	8.687
BA	29	00207	Abaré	17.380
BA	29	00306	Acajutiba	14.692
BA	29	00355	Adustina	15.810
BA	29	00405	Água Fria	15.809
BA	29	00603	Aiquara	4.569
BA	29	00702	Alagoinhas	142.870
BA	29	00801	Alcobaça	21.300
BA	29	00900	Almadina	6.242
BA	29	01007	Amargosa	34.602
BA	29	01106	Amélia Rodrigues	25.134
BA	29	01155	América Dourada	15.962
BA	29	01205	Anagé	25.049
BA	29	01304	Andaraí	13.951
BA	29	01353	Andorinha	14.310
BA	29	01403	Angical	14.032
BA	29	01502	Anguera	10.336
BA	29	01601	Antas	17.303
BA	29	01700	Antônio Cardoso	11.549
BA	29	01809	Antônio Gonçalves	11.124
BA	29	01908	Aporá	17.805
BA	29	01957	Apuarema	7.428
BA	29	02054	Araças	11.602
BA	29	02005	Aracatu	13.641
BA	29	02104	Araci	51.994
BA	29	02203	Aramari	10.111
BA	29	02252	Arataca	10.349
BA	29	02302	Aratuípe	8.616
BA	29	02401	Aurelino Leal	13.323
BA	29	02500	Baianópolis	13.919
BA	29	02609	Baixa Grande	20.046
BA	29	02658	Banzaê	11.827
BA	29	02708	Barra	49.736
BA	29	02807	Barra da Estiva	20.974
BA	29	02906	Barra do Choça	35.084
BA	29	03003	Barra do Mendes	13.950
BA	29	03102	Barra do Rocha	6.174
BA	29	03201	Barreiras	139.285

BA	29	03235	Barro Alto	13.766
BA	29	03300	Barro Preto	6.285
BA	29	03276	Barrocas	14.346
BA	29	03409	Belmonte	21.935
BA	29	03508	Belo Campo	15.915
BA	29	03607	Biritinga	14.851
BA	29	03706	Boa Nova	15.048
BA	29	03805	Boa Vista do Tupim	17.944
BA	29	03904	Bom Jesus da Lapa	64.121
BA	29	03953	Bom Jesus da Serra	10.054
BA	29	04001	Boninal	13.796
BA	29	04050	Bonito	14.983
BA	29	04100	Boquira	22.031
BA	29	04209	Botuporã	11.050
BA	29	04308	Brejões	14.201
BA	29	04407	Brejolândia	11.164
BA	29	04506	Brotas de Macaúbas	10.596
BA	29	04605	Brumado	64.790
BA	29	04704	Buerarema	18.566
BA	29	04753	Buritirama	19.729
BA	29	04803	Caatiba	11.016
BA	29	04852	Cabaceiras do Paraguaçu	17.457
BA	29	04902	Cachoeira	32.150
BA	29	05008	Caculé	22.396
BA	29	05107	Caém	10.188
BA	29	05156	Caetanos	13.727
BA	29	05206	Caetité	47.647
BA	29	05305	Cafarnaum	17.305
BA	29	05404	Cairu	15.679
BA	29	05503	Caldeirão Grande	12.576
BA	29	05602	Camacan	31.505
BA	29	05701	Camaçari	249.206
BA	29	05800	Camamu	35.275
BA	29	05909	Campo Alegre de Lourdes	28.124
BA	29	06006	Campo Formoso	66.967
BA	29	06105	Canápolis	9.403
BA	29	06204	Canarana	24.252
BA	29	06303	Canavieiras	32.116
BA	29	06402	Candeal	8.806
BA	29	06501	Candeias	83.648
BA	29	06600	Candiba	13.271
BA	29	06709	Cândido Sales	27.747
BA	29	06808	Cansanção	32.982
BA	29	06824	Canudos	15.839
BA	29	06857	Capela do Alto Alegre	11.506
BA	29	06873	Capim Grosso	26.826
BA	29	06899	Caraíbas	10.048
BA	29	06907	Caravelas	21.515
BA	29	07004	Cardeal da Silva	8.966
BA	29	07103	Carinhanha	28.451
BA	29	07202	Casa Nova	65.647
BA	29	07301	Castro Alves	25.483
BA	29	07400	Catolândia	2.632
BA	29	07509	Catu	51.411
BA	29	07558	Caturama	8.830
BA	29	07608	Central	17.035
BA	29	07707	Chorrochó	10.765

BA	29	07806	Cícero Dantas	32.387
BA	29	07905	Cipó	15.821
BA	29	08002	Coaraci	20.442
BA	29	08101	Cocos	18.195
BA	29	08200	Conceição da Feira	20.612
BA	29	08309	Conceição do Almeida	17.796
BA	29	08408	Conceição do Coité	62.545
BA	29	08507	Conceição do Jacuípe	30.425
BA	29	08606	Conde	23.866
BA	29	08705	Condeúba	16.842
BA	29	08804	Contendas do Sincorá	4.638
BA	29	08903	Coração de Maria	22.273
BA	29	09000	Cordeiros	8.205
BA	29	09109	Coribe	14.258
BA	29	09208	Coronel João Sá	16.855
BA	29	09307	Correntina	31.324
BA	29	09406	Cotegipe	13.625
BA	29	09505	Cravolândia	5.045
BA	29	09604	Crisópolis	20.124
BA	29	09703	Cristópolis	13.328
BA	29	09802	Cruz das Almas	59.045
BA	29	09901	Curaçá	32.403
BA	29	10008	Dário Meira	12.609
BA	29	10057	Dias d'Ávila	68.061
BA	29	10107	Dom Basílio	11.405
BA	29	10206	Dom Macedo Costa	3.884
BA	29	10305	Elísio Medrado	7.954
BA	29	10404	Encruzilhada	23.968
BA	29	10503	Entre Rios	40.029
BA	29	00504	Érico Cardoso	10.802
BA	29	10602	Esplanada	33.217
BA	29	10701	Euclides da Cunha	56.631
BA	29	10727	Eunápolis	101.432
BA	29	10750	Fátima	17.603
BA	29	10776	Feira da Mata	6.181
BA	29	10800	Feira de Santana	562.466
BA	29	10859	Filadélfia	16.706
BA	29	10909	Firmino Alves	5.401
BA	29	11006	Floresta Azul	10.605
BA	29	11105	Formosa do Rio Preto	22.854
BA	29	11204	Gandu	30.580
BA	29	11253	Gavião	4.535
BA	29	11303	Gentio do Ouro	10.657
BA	29	11402	Glória	15.096
BA	29	11501	Gongogi	8.191
BA	29	11600	Governador Mangabeira	19.873
BA	29	11659	Guajeru	10.039
BA	29	11709	Guanambi	79.394
BA	29	11808	Guaratinga	22.000
BA	29	11857	Heliópolis	13.145
BA	29	11907	Iaçu	25.524
BA	29	12004	Ibiassucê	9.831
BA	29	12103	Ibicaraí	23.910
BA	29	12202	Ibicoara	17.548
BA	29	12301	Ibicuí	15.830
BA	29	12400	Ibipeba	17.145
BA	29	12509	Ibipitanga	14.229

BA	29	12608	Ibiquera	4.870
BA	29	12707	Ibirapitanga	22.641
BA	29	12806	Ibirapuã	8.023
BA	29	12905	Ibirataia	18.443
BA	29	13002	Ibitiara	15.590
BA	29	13101	Ibititá	17.801
BA	29	13200	Ibotirama	25.522
BA	29	13309	Ichu	5.269
BA	29	13408	Igaporã	15.222
BA	29	13457	Igrapiúna	13.183
BA	29	13507	Iguaí	25.768
BA	29	13606	Ilhéus	185.801
BA	29	13705	Inhambupe	36.822
BA	29	13804	Ipecaetá	15.176
BA	29	13903	Ipiaú	44.465
BA	29	14000	Ipirá	59.169
BA	29	14109	Ipupiara	9.343
BA	29	14208	Irajuba	7.024
BA	29	14307	Iramaia	11.564
BA	29	14406	Iraquara	22.929
BA	29	14505	Irará	27.643
BA	29	14604	Irecê	66.865
BA	29	14653	Itabela	28.593
BA	29	14703	Itaberaba	61.838
BA	29	14802	Itabuna	205.286
BA	29	14901	Itacaré	24.794
BA	29	15007	Itaeté	14.995
BA	29	15106	Itagi	12.926
BA	29	15205	Itagibá	15.053
BA	29	15304	Itagimirim	7.061
BA	29	15353	Itaguaçu da Bahia	13.350
BA	29	15403	Itaju do Colônia	7.212
BA	29	15502	Itajuípe	20.978
BA	29	15601	Itamaraju	63.053
BA	29	15700	Itamari	7.869
BA	29	15809	Itambé	22.828
BA	29	15908	Itanagra	7.594
BA	29	16005	Itanhém	20.114
BA	29	16104	Itaparica	20.862
BA	29	16203	Itapé	10.711
BA	29	16302	Itapebi	10.446
BA	29	16401	Itapetinga	69.067
BA	29	16500	Itapicuru	32.641
BA	29	16609	Itapitanga	10.194
BA	29	16708	Itaquara	7.715
BA	29	16807	Itarantim	18.664
BA	29	16856	Itatim	13.775
BA	29	16906	Itiruçu	12.640
BA	29	17003	Itiúba	36.157
BA	29	17102	Itororó	19.928
BA	29	17201	Ituaçu	18.216
BA	29	17300	Ituberá	26.764
BA	29	17334	Iuiú	10.932
BA	29	17359	Jaborandi	8.849
BA	29	17409	Jacaraci	13.664
BA	29	17508	Jacobina	79.417
BA	29	17607	Jaguaquara	51.328

BA	29	17706	Jaguarari	30.560
BA	29	17805	Jaguaripe	16.701
BA	29	17904	Jandaíra	10.355
BA	29	18001	Jequié	152.138
BA	29	18100	Jeremoabo	37.926
BA	29	18209	Jiquiriçá	14.147
BA	29	18308	Jitaúna	13.691
BA	29	18357	João Dourado	22.812
BA	29	18407	Juazeiro	199.761
BA	29	18456	Jucuruçu	10.129
BA	29	18506	Jussara	15.028
BA	29	18555	Jussari	6.397
BA	29	18605	Jussiape	7.778
BA	29	18704	Lafaiete Coutinho	3.865
BA	29	18753	Lagoa Real	14.063
BA	29	18803	Laje	22.444
BA	29	18902	Lajedão	3.758
BA	29	19009	Lajedinho	3.908
BA	29	19058	Lajedo do Tabocal	8.326
BA	29	19108	Lamarão	9.413
BA	29	19157	Lapão	25.717
BA	29	19207	Lauro de Freitas	167.309
BA	29	19306	Lençóis	10.480
BA	29	19405	Licínio de Almeida	12.295
BA	29	19504	Livramento de Nossa Senhora	43.110
BA	29	19553	Luís Eduardo Magalhães	63.290
BA	29	19603	Macajuba	11.215
BA	29	19702	Macarani	17.285
BA	29	19801	Macaúbas	47.490
BA	29	19900	Macururé	8.032
BA	29	19926	Madre de Deus	17.786
BA	29	19959	Maetinga	6.554
BA	29	20007	Maiquinique	8.894
BA	29	20106	Mairi	19.243
BA	29	20205	Malhada	16.037
BA	29	20304	Malhada de Pedras	8.428
BA	29	20403	Manoel Vitorino	14.221
BA	29	20452	Mansidão	12.707
BA	29	20502	Maracás	24.822
BA	29	20601	Maragogipe	42.967
BA	29	20700	Maraú	19.158
BA	29	20809	Marcionílio Souza	10.473
BA	29	20908	Mascote	14.520
BA	29	21005	Mata de São João	40.866
BA	29	21054	Matina	11.246
BA	29	21104	Medeiros Neto	21.602
BA	29	21203	Miguel Calmon	26.329
BA	29	21302	Milagres	11.057
BA	29	21401	Mirangaba	16.445
BA	29	21450	Mirante	10.265
BA	29	21500	Monte Santo	52.178
BA	29	21609	Morpará	8.256
BA	29	21708	Morro do Chapéu	35.208
BA	29	21807	Mortugaba	12.496
BA	29	21906	Mucugê	10.342
BA	29	22003	Mucuri	36.638
BA	29	22052	Mulungu do Morro	11.992

BA	29	22102	Mundo Novo	24.635
BA	29	22201	Muniz Ferreira	7.346
BA	29	22250	Muquém de São Francisco	10.354
BA	29	22300	Muritiba	28.922
BA	29	22409	Mutuípe	21.530
BA	29	22508	Nazaré	27.366
BA	29	22607	Nilo Peçanha	12.632
BA	29	22656	Nordestina	12.415
BA	29	22706	Nova Canaã	16.731
BA	29	22730	Nova Fátima	7.616
BA	29	22755	Nova Ibiá	6.609
BA	29	22805	Nova Itarana	7.500
BA	29	22854	Nova Redenção	8.044
BA	29	22904	Nova Soure	24.202
BA	29	23001	Nova Viçosa	39.054
BA	29	23035	Novo Horizonte	10.840
BA	29	23050	Novo Triunfo	15.059
BA	29	23100	Olindina	25.023
BA	29	23209	Oliveira dos Brejinhos	21.822
BA	29	23308	Ouriçangas	8.307
BA	29	23357	Ourolândia	16.503
BA	29	23407	Palmas de Monte Alto	20.836
BA	29	23506	Palmeiras	8.479
BA	29	23605	Paramirim	21.115
BA	29	23704	Paratinga	29.682
BA	29	23803	Paripiranga	27.870
BA	29	23902	Pau Brasil	10.684
BA	29	24009	Paulo Afonso	109.310
BA	29	24058	Pé de Serra	13.729
BA	29	24108	Pedrão	6.936
BA	29	24207	Pedro Alexandre	17.020
BA	29	24306	Piatã	17.417
BA	29	24405	Pilão Arcado	33.021
BA	29	24504	Pindaí	15.662
BA	29	24603	Pindobaçu	20.064
BA	29	24652	Pintadas	10.295
BA	29	24678	Piraí do Norte	9.816
BA	29	24702	Piripá	12.506
BA	29	24801	Piritiba	22.658
BA	29	24900	Planaltino	8.884
BA	29	25006	Planalto	24.694
BA	29	25105	Poções	44.723
BA	29	25204	Pojuca	33.595
BA	29	25253	Ponto Novo	15.632
BA	29	25303	Porto Seguro	129.325
BA	29	25402	Potiraguá	9.444
BA	29	25501	Prado	27.661
BA	29	25600	Presidente Dutra	13.779
BA	29	25709	Presidente Jânio Quadros	13.484
BA	29	25758	Presidente Tancredo Neves	24.187
BA	29	25808	Queimadas	24.602
BA	29	25907	Quijingue	27.294
BA	29	25931	Quixabeira	9.534
BA	29	25956	Rafael Jambeiro	22.896
BA	29	26004	Remanso	39.165
BA	29	26103	Retirolândia	12.170
BA	29	26202	Riachão das Neves	21.939

BA	29	26301	Riachão do Jacuípe	33.222
BA	29	26400	Riacho de Santana	30.840
BA	29	26509	Ribeira do Amparo	14.305
BA	29	26608	Ribeira do Pombal	47.701
BA	29	26657	Ribeirão do Largo	8.675
BA	29	26707	Rio de Contas	12.948
BA	29	26806	Rio do Antônio	14.917
BA	29	26905	Rio do Pires	11.933
BA	29	27002	Rio Real	37.464
BA	29	27101	Rodelas	7.912
BA	29	27200	Ruy Barbosa	29.950
BA	29	27309	Salinas da Margarida	13.693
BA	29	27408	Salvador	2.693.606
BA	29	27507	Santa Bárbara	19.180
BA	29	27606	Santa Brígida	14.876
BA	29	27705	Santa Cruz Cabrália	26.447
BA	29	27804	Santa Cruz da Vitória	6.630
BA	29	27903	Santa Inês	10.337
BA	29	28059	Santa Luzia	13.182
BA	29	28109	Santa Maria da Vitória	40.236
BA	29	28406	Santa Rita de Cássia	26.425
BA	29	28505	Santa Teresinha	9.722
BA	29	28000	Santaluz	34.060
BA	29	28208	Santana	24.871
BA	29	28307	Santanópolis	8.806
BA	29	28604	Santo Amaro	57.891
BA	29	28703	Santo Antônio de Jesus	92.049
BA	29	28802	Santo Estêvão	48.397
BA	29	28901	São Desidério	28.301
BA	29	28950	São Domingos	9.247
BA	29	29107	São Felipe	20.317
BA	29	29008	São Félix	14.129
BA	29	29057	São Félix do Coribe	13.147
BA	29	29206	São Francisco do Conde	33.713
BA	29	29255	São Gabriel	18.429
BA	29	29305	São Gonçalo dos Campos	33.766
BA	29	29354	São José da Vitória	5.662
BA	29	29370	São José do Jacuípe	10.238
BA	29	29404	São Miguel das Matas	10.445
BA	29	29503	São Sebastião do Passé	42.322
BA	29	29602	Sapeaçu	16.603
BA	29	29701	Sátiro Dias	19.010
BA	29	29750	Saubara	11.279
BA	29	29800	Saúde	11.884
BA	29	29909	Seabra	41.984
BA	29	30006	Sebastião Laranjeiras	10.469
BA	29	30105	Senhor do Bonfim	74.937
BA	29	30204	Sento Sé	37.806
BA	29	30154	Serra do Ramalho	31.581
BA	29	30303	Serra Dourada	18.036
BA	29	30402	Serra Preta	15.194
BA	29	30501	Serrinha	77.309
BA	29	30600	Serrolândia	12.405
BA	29	30709	Simões Filho	119.760
BA	29	30758	Sítio do Mato	12.107
BA	29	30766	Sítio do Quinto	12.256
BA	29	30774	Sobradinho	22.056

BA	29	30808	Souto Soares	15.986
BA	29	30907	Tabocas do Brejo Velho	11.432
BA	29	31004	Tanhaçu	20.007
BA	29	31053	Tanque Novo	16.228
BA	29	31103	Tanquinho	8.022
BA	29	31202	Taperoá	18.965
BA	29	31301	Tapiramutá	16.475
BA	29	31350	Teixeira de Freitas	140.710
BA	29	31400	Teodoro Sampaio	7.820
BA	29	31509	Teofilândia	21.533
BA	29	31608	Teolândia	14.941
BA	29	31707	Terra Nova	12.798
BA	29	31806	Tremedal	16.796
BA	29	31905	Tucano	52.579
BA	29	32002	Uauá	24.152
BA	29	32101	Ubaíra	19.728
BA	29	32200	Ubaitaba	20.449
BA	29	32309	Ubatã	25.295
BA	29	32408	Uibaí	13.634
BA	29	32457	Umburanas	17.220
BA	29	32507	Una	23.542
BA	29	32606	Urandi	16.480
BA	29	32705	Uruçuca	19.738
BA	29	32804	Utinga	18.272
BA	29	32903	Valença	89.510
BA	29	33000	Valente	24.958
BA	29	33059	Várzea da Roça	13.811
BA	29	33109	Várzea do Poço	8.711
BA	29	33158	Várzea Nova	12.991
BA	29	33174	Varzedo	9.047
BA	29	33208	Vera Cruz	38.168
BA	29	33257	Vereda	6.740
BA	29	33307	Vitória da Conquista	310.129
BA	29	33406	Wagner	8.984
BA	29	33455	Wanderley	12.420
BA	29	33505	Wenceslau Guimarães	22.047
BA	29	33604	Xique-Xique	45.599
MG	31	00104	Abadia dos Dourados	6.724
MG	31	00203	Abaeté	22.716
MG	31	00302	Abre Campo	13.309
MG	31	00401	Acaiaca	3.923
MG	31	00500	Açucena	10.183
MG	31	00609	Água Boa	14.996
MG	31	00708	Água Comprida	2.020
MG	31	00807	Aguanil	4.092
MG	31	00906	Águas Formosas	18.528
MG	31	01003	Águas Vermelhas	12.787
MG	31	01102	Aimorés	24.948
MG	31	01201	Aiuruoca	6.139
MG	31	01300	Alagoa	2.703
MG	31	01409	Albertina	2.919
MG	31	01508	Além Paraíba	34.406
MG	31	01607	Alfenas	74.298
MG	31	01631	Alfredo Vasconcelos	6.150
MG	31	01706	Almenara	39.036
MG	31	01805	Alpercata	7.188
MG	31	01904	Alpinópolis	18.600

MG	31	02001	Alterosa	13.774
MG	31	02050	Alto Caparaó	5.345
MG	31	53509	Alto Jequitibá	8.308
MG	31	02100	Alto Rio Doce	12.029
MG	31	02209	Alvarenga	4.386
MG	31	02308	Alvinópolis	15.236
MG	31	02407	Alvorada de Minas	3.548
MG	31	02506	Amparo do Serra	5.021
MG	31	02605	Andradas	37.601
MG	31	02803	Andrelândia	12.163
MG	31	02852	Angelândia	8.045
MG	31	02902	Antônio Carlos	11.133
MG	31	03009	Antônio Dias	9.529
MG	31	03108	Antônio Prado de Minas	1.662
MG	31	03207	Araçaí	2.251
MG	31	03306	Aracitaba	2.056
MG	31	03405	Araçuaí	36.037
MG	31	03504	Araguari	110.402
MG	31	03603	Arantina	2.817
MG	31	03702	Araponga	8.171
MG	31	03751	Araporã	6.209
MG	31	03801	Arapuá	2.778
MG	31	03900	Araújos	8.011
MG	31	04007	Araxá	94.799
MG	31	04106	Arceburgo	9.623
MG	31	04205	Arcos	36.898
MG	31	04304	Areado	13.847
MG	31	04403	Argirita	2.881
MG	31	04452	Aricanduva	4.810
MG	31	04502	Arinos	17.672
MG	31	04601	Astolfo Dutra	13.145
MG	31	04700	Ataléia	14.280
MG	31	04809	Augusto de Lima	4.945
MG	31	04908	Baependi	18.368
MG	31	05004	Baldim	7.895
MG	31	05103	Bambuí	22.814
MG	31	05202	Bandeira	4.962
MG	31	05301	Bandeira do Sul	5.372
MG	31	05400	Barão de Cocais	28.830
MG	31	05509	Barão de Monte Alto	5.681
MG	31	05608	Barbacena	127.218
MG	31	05707	Barra Longa	6.035
MG	31	05905	Barroso	19.695
MG	31	06002	Bela Vista de Minas	10.017
MG	31	06101	Belmiro Braga	3.402
MG	31	06200	Belo Horizonte	2.385.640
MG	31	06309	Belo Oriente	23.695
MG	31	06408	Belo Vale	7.545
MG	31	06507	Berilo	12.248
MG	31	06655	Berizal	4.401
MG	31	06606	Bertópolis	4.503
MG	31	06705	Betim	383.571
MG	31	06804	Bias Fortes	3.748
MG	31	06903	Bicas	13.720
MG	31	07000	Biquinhas	2.616
MG	31	07109	Boa Esperança	38.627
MG	31	07208	Bocaina de Minas	5.009

MG	31	07307	Bocaiúva	46.950
MG	31	07406	Bom Despacho	46.061
MG	31	07505	Bom Jardim de Minas	6.491
MG	31	07604	Bom Jesus da Penha	3.915
MG	31	07703	Bom Jesus do Amparo	5.543
MG	31	07802	Bom Jesus do Galho	15.302
MG	31	07901	Bom Repouso	10.453
MG	31	08008	Bom Sucesso	17.257
MG	31	08107	Bonfim	6.815
MG	31	08206	Bonfinópolis de Minas	5.821
MG	31	08255	Bonito de Minas	9.812
MG	31	08305	Borda da Mata	17.324
MG	31	08404	Botelhos	14.907
MG	31	08503	Botumirim	6.472
MG	31	08701	Brás Pires	4.601
MG	31	08552	Brasilândia de Minas	14.438
MG	31	08602	Brasília de Minas	31.286
MG	31	08909	Brasópolis	14.623
MG	31	08800	Braúnas	5.001
MG	31	09006	Brumadinho	34.538
MG	31	09105	Bueno Brandão	10.889
MG	31	09204	Buenópolis	10.287
MG	31	09253	Bugre	3.996
MG	31	09303	Buritis	22.917
MG	31	09402	Buritizeiro	27.001
MG	31	09451	Cabeceira Grande	6.494
MG	31	09501	Cabo Verde	13.831
MG	31	09600	Cachoeira da Prata	3.645
MG	31	09709	Cachoeira de Minas	11.071
MG	31	02704	Cachoeira de Pajeú	8.993
MG	31	09808	Cachoeira Dourada	2.521
MG	31	09907	Caetanópolis	10.345
MG	31	10004	Caeté	41.092
MG	31	10103	Caiana	5.015
MG	31	10202	Cajuri	4.037
MG	31	10301	Caldas	13.700
MG	31	10400	Camacho	3.125
MG	31	10509	Camanducaia	21.122
MG	31	10608	Cambuí	26.759
MG	31	10707	Cambuquira	12.607
MG	31	10806	Campanário	3.576
MG	31	10905	Campanha	15.536
MG	31	11002	Campestre	20.697
MG	31	11101	Campina Verde	19.342
MG	31	11150	Campo Azul	3.693
MG	31	11200	Campo Belo	51.725
MG	31	11309	Campo do Meio	11.480
MG	31	11408	Campo Florido	6.989
MG	31	11507	Campos Altos	14.313
MG	31	11606	Campos Gerais	27.682
MG	31	11903	Cana Verde	5.584
MG	31	11705	Canaã	4.616
MG	31	11804	Canápolis	11.422
MG	31	12000	Candeias	14.606
MG	31	12059	Cantagalo	4.223
MG	31	12109	Caparaó	5.226
MG	31	12208	Capela Nova	4.739

MG	31	12307	Capelinha	35.090
MG	31	12406	Capetinga	7.064
MG	31	12505	Capim Branco	8.957
MG	31	12604	Capinópolis	15.359
MG	31	12653	Capitão Andrade	4.973
MG	31	12703	Capitão Enéas	14.290
MG	31	12802	Capitólio	8.218
MG	31	12901	Caputira	9.046
MG	31	13008	Caraí	22.448
MG	31	13107	Caranaíba	3.274
MG	31	13206	Carandaí	23.522
MG	31	13305	Carangola	32.325
MG	31	13404	Caratinga	85.811
MG	31	13503	Carbonita	9.162
MG	31	13602	Careaçu	6.336
MG	31	13701	Carlos Chagas	19.922
MG	31	13800	Carmésia	2.462
MG	31	13909	Carmo da Cachoeira	11.855
MG	31	14006	Carmo da Mata	10.968
MG	31	14105	Carmo de Minas	13.843
MG	31	14204	Carmo do Cajuru	20.232
MG	31	14303	Carmo do Paranaíba	29.757
MG	31	14402	Carmo do Rio Claro	20.480
MG	31	14501	Carmópolis de Minas	17.256
MG	31	14550	Carneirinho	9.515
MG	31	14600	Carrancas	3.953
MG	31	14709	Carvalhópolis	3.361
MG	31	14808	Carvalhos	4.543
MG	31	14907	Casa Grande	2.243
MG	31	15003	Cascalho Rico	2.876
MG	31	15102	Cássia	17.423
MG	31	15300	Cataguases	70.201
MG	31	15359	Catas Altas	4.893
MG	31	15409	Catas Altas da Noruega	3.476
MG	31	15458	Catuji	6.661
MG	31	15474	Catuti	5.084
MG	31	15508	Caxambu	21.673
MG	31	15607	Cedro do Abaeté	1.204
MG	31	15706	Central de Minas	6.790
MG	31	15805	Centralina	10.269
MG	31	15904	Chácara	2.825
MG	31	16001	Chalé	5.644
MG	31	16100	Chapada do Norte	15.187
MG	31	16159	Chapada Gaúcha	11.077
MG	31	16209	Chiador	2.772
MG	31	16308	Cipotânea	6.563
MG	31	16407	Claraval	4.566
MG	31	16506	Claro dos Poções	7.743
MG	31	16605	Cláudio	26.021
MG	31	16704	Coimbra	7.095
MG	31	16803	Coluna	8.998
MG	31	16902	Comendador Gomes	2.982
MG	31	17009	Comercinho	8.152
MG	31	17108	Conceição da Aparecida	9.855
MG	31	15201	Conceição da Barra de Minas	3.949
MG	31	17306	Conceição das Alagoas	23.495
MG	31	17207	Conceição das Pedras	2.752

MG	31	17405	Conceição de Ipanema	4.463
MG	31	17504	Conceição do Mato Dentro	17.853
MG	31	17603	Conceição do Pará	5.187
MG	31	17702	Conceição do Rio Verde	13.001
MG	31	17801	Conceição dos Ouros	10.500
MG	31	17836	Cônego Marinho	7.149
MG	31	17876	Confins	6.008
MG	31	17900	Congonhal	10.602
MG	31	18007	Congonhas	49.077
MG	31	18106	Congonhas do Norte	4.947
MG	31	18205	Conquista	6.559
MG	31	18304	Conselheiro Lafaiete	117.562
MG	31	18403	Conselheiro Pena	22.281
MG	31	18502	Consolação	1.730
MG	31	18601	Contagem	608.715
MG	31	18700	Coqueiral	9.265
MG	31	18809	Coração de Jesus	26.057
MG	31	18908	Cordisburgo	8.679
MG	31	19005	Cordislândia	3.441
MG	31	19104	Corinto	23.866
MG	31	19203	Coroaci	10.230
MG	31	19302	Coromandel	27.555
MG	31	19401	Coronel Fabriciano	104.174
MG	31	19500	Coronel Murta	9.116
MG	31	19609	Coronel Pacheco	2.990
MG	31	19708	Coronel Xavier Chaves	3.310
MG	31	19807	Córrego Danta	3.370
MG	31	19906	Córrego do Bom Jesus	3.723
MG	31	19955	Córrego Fundo	5.837
MG	31	20003	Córrego Novo	3.088
MG	31	20102	Couto de Magalhães de Minas	4.220
MG	31	20151	Crisólita	6.105
MG	31	20201	Cristais	11.422
MG	31	20300	Cristália	5.774
MG	31	20409	Cristiano Otoni	5.015
MG	31	20508	Cristina	10.201
MG	31	20607	Crucilândia	4.779
MG	31	20706	Cruzeiro da Fortaleza	3.951
MG	31	20805	Cruzília	14.655
MG	31	20839	Cuparaque	4.705
MG	31	20870	Curral de Dentro	6.986
MG	31	20904	Curvelo	74.734
MG	31	21001	Datas	5.225
MG	31	21100	Delfim Moreira	7.967
MG	31	21209	Delfinópolis	6.850
MG	31	21258	Delta	8.322
MG	31	21308	Descoberto	4.787
MG	31	21407	Desterro de Entre Rios	7.017
MG	31	21506	Desterro do Melo	3.000
MG	31	21605	Diamantina	46.005
MG	31	21704	Diogo de Vasconcelos	3.839
MG	31	21803	Dionísio	8.628
MG	31	21902	Divinésia	3.302
MG	31	22009	Divino	19.188
MG	31	22108	Divino das Laranjeiras	4.935
MG	31	22207	Divinolândia de Minas	7.070
MG	31	22306	Divinópolis	215.247

MG	31	22355	Divisa Alegre	5.967
MG	31	22405	Divisa Nova	5.781
MG	31	22454	Divisópolis	9.166
MG	31	22470	Dom Bosco	3.796
MG	31	22504	Dom Cavati	5.189
MG	31	22603	Dom Joaquim	4.523
MG	31	22702	Dom Silvério	5.194
MG	31	22801	Dom Viçoso	2.991
MG	31	22900	Dona Eusébia	6.051
MG	31	23007	Dores de Campos	9.372
MG	31	23106	Dores de Guanhães	5.211
MG	31	23205	Dores do Indaiá	13.732
MG	31	23304	Dores do Turvo	4.437
MG	31	23403	Doresópolis	1.447
MG	31	23502	Douradoquara	1.846
MG	31	23528	Durandé	7.456
MG	31	23601	Elói Mendes	25.472
MG	31	23700	Engenheiro Caldas	10.352
MG	31	23809	Engenheiro Navarro	7.125
MG	31	23858	Entre Folhas	5.185
MG	31	23908	Entre Rios de Minas	14.329
MG	31	24005	Ervália	18.018
MG	31	24104	Esmeraldas	61.283
MG	31	24203	Espera Feliz	23.035
MG	31	24302	Espinosa	31.124
MG	31	24401	Espírito Santo do Dourado	4.450
MG	31	24500	Estiva	10.882
MG	31	24609	Estrela Dalva	2.455
MG	31	24708	Estrela do Indaiá	3.510
MG	31	24807	Estrela do Sul	7.490
MG	31	24906	Eugenópolis	10.600
MG	31	25002	Ewbank da Câmara	3.765
MG	31	25101	Extrema	29.319
MG	31	25200	Fama	2.350
MG	31	25309	Faria Lemos	3.359
MG	31	25408	Felício dos Santos	5.097
MG	31	25606	Felisburgo	6.926
MG	31	25705	Felixlândia	14.224
MG	31	25804	Fernandes Tourinho	3.066
MG	31	25903	Ferros	10.723
MG	31	25952	Fervedouro	10.402
MG	31	26000	Florestal	6.674
MG	31	26109	Formiga	65.299
MG	31	26208	Formoso	8.305
MG	31	26307	Fortaleza de Minas	4.125
MG	31	26406	Fortuna de Minas	2.726
MG	31	26505	Francisco Badaró	10.244
MG	31	26604	Francisco Dumont	4.892
MG	31	26703	Francisco Sá	25.016
MG	31	26752	Franciscópolis	5.752
MG	31	26802	Frei Gaspar	5.872
MG	31	26901	Frei Inocêncio	8.978
MG	31	26950	Frei Lagonegro	3.340
MG	31	27008	Fronteira	14.427
MG	31	27057	Fronteira dos Vales	4.671
MG	31	27073	Fruta de Leite	5.876
MG	31	27107	Frutal	53.998

MG	31	27206	Funilândia	3.900
MG	31	27305	Galiléia	6.929
MG	31	27339	Gameleiras	5.130
MG	31	27354	Glaucilândia	2.977
MG	31	27370	Goiabeira	3.079
MG	31	27388	Goianá	3.685
MG	31	27404	Gonçalves	4.228
MG	31	27503	Gonzaga	5.937
MG	31	27602	Gouveia	11.681
MG	31	27701	Governador Valadares	264.960
MG	31	27800	Grão Mogol	15.086
MG	31	27909	Grupiara	1.373
MG	31	28006	Guanhães	31.526
MG	31	28105	Guapé	13.892
MG	31	28204	Guaraciaba	10.221
MG	31	28253	Guaraciama	4.738
MG	31	28303	Guaranésia	18.721
MG	31	28402	Guarani	8.691
MG	31	28501	Guarará	3.911
MG	31	28600	Guarda-Mor	6.559
MG	31	28709	Guaxupé	49.614
MG	31	28808	Guidoval	7.185
MG	31	28907	Guimarânia	7.333
MG	31	29004	Guiricema	8.665
MG	31	29103	Gurinhatã	6.080
MG	31	29202	Heliodora	6.157
MG	31	29301	Iapu	10.361
MG	31	29400	Ibertioga	5.029
MG	31	29509	Ibiá	23.385
MG	31	29608	Ibiaí	7.885
MG	31	29657	Ibiracatu	6.126
MG	31	29707	Ibiraci	12.326
MG	31	29806	Ibirité	160.943
MG	31	29905	Ibitiúra de Minas	3.389
MG	31	30002	Ibituruna	2.875
MG	31	30051	Icaraí de Minas	10.856
MG	31	30101	Igarapé	35.620
MG	31	30200	Igaratinga	9.411
MG	31	30309	Iguatama	8.011
MG	31	30408	Ijaci	5.921
MG	31	30507	Ilicínea	11.562
MG	31	30556	Imbé de Minas	6.464
MG	31	30606	Inconfidentes	6.941
MG	31	30655	Indaiabira	7.323
MG	31	30705	Indianópolis	6.252
MG	31	30804	Ingaí	2.640
MG	31	30903	Inhapim	24.248
MG	31	31000	Inhaúma	5.804
MG	31	31109	Inimutaba	6.771
MG	31	31158	Ipaba	16.876
MG	31	31208	Ipanema	18.315
MG	31	31307	Ipatinga	241.539
MG	31	31406	Ipiaçu	4.114
MG	31	31505	Ipuiúna	9.565
MG	31	31604	Iraí de Minas	6.511
MG	31	31703	Itabira	110.663
MG	31	31802	Itabirinha	10.760

MG	31	31901	Itabirito	46.029
MG	31	32008	Itacambira	5.022
MG	31	32107	Itacarambi	17.741
MG	31	32206	Itaguara	12.455
MG	31	32305	Itaipé	11.879
MG	31	32404	Itajubá	91.159
MG	31	32503	Itamarandiba	32.388
MG	31	32602	Itamarati de Minas	4.102
MG	31	32701	Itambacuri	22.820
MG	31	32800	Itambé do Mato Dentro	2.261
MG	31	32909	Itamogi	10.321
MG	31	33006	Itamonte	14.142
MG	31	33105	Itanhandu	14.272
MG	31	33204	Itanhomi	11.878
MG	31	33303	Itaobim	20.981
MG	31	33402	Itapagipe	13.797
MG	31	33501	Itapecerica	21.388
MG	31	33600	Itapeva	8.765
MG	31	33709	Itatiaiuçu	10.037
MG	31	33758	Itaú de Minas	15.042
MG	31	33808	Itaúna	86.124
MG	31	33907	Itaverava	5.754
MG	31	34004	Itinga	14.447
MG	31	34103	Itueta	5.845
MG	31	34202	Ituiutaba	97.792
MG	31	34301	Itumirim	6.120
MG	31	34400	Iturama	34.890
MG	31	34509	Itutinga	3.896
MG	31	34608	Jaboticatubas	17.411
MG	31	34707	Jacinto	12.138
MG	31	34806	Jacuí	7.511
MG	31	34905	Jacutinga	23.062
MG	31	35001	Jaguaraçu	3.001
MG	31	35050	Jaíba	34.071
MG	31	35076	Jampruca	5.094
MG	31	35100	Janaúba	67.199
MG	31	35209	Januária	65.606
MG	31	35308	Japaraíba	3.975
MG	31	35357	Japonvar	8.315
MG	31	35407	Jeceaba	5.341
MG	31	35456	Jenipapo de Minas	7.165
MG	31	35506	Jequeri	12.786
MG	31	35605	Jequitaí	7.948
MG	31	35704	Jequitibá	5.155
MG	31	35803	Jequitinhonha	24.226
MG	31	35902	Jesuânia	4.764
MG	31	36009	Joaíma	14.971
MG	31	36108	Joanésia	5.334
MG	31	36207	João Monlevade	74.142
MG	31	36306	João Pinheiro	45.559
MG	31	36405	Joaquim Felício	4.339
MG	31	36504	Jordânia	10.360
MG	31	36520	José Gonçalves de Minas	4.543
MG	31	36553	José Raydan	4.432
MG	31	36579	Josenópolis	4.591
MG	31	36652	Juatuba	22.649
MG	31	36702	Juiz de Fora	520.811

MG	31	36801	Juramento	4.130
MG	31	36900	Juruaia	9.358
MG	31	36959	Juvenília	5.703
MG	31	37007	Ladainha	17.084
MG	31	37106	Lagamar	7.592
MG	31	37205	Lagoa da Prata	46.539
MG	31	37304	Lagoa dos Patos	4.208
MG	31	37403	Lagoa Dourada	12.316
MG	31	37502	Lagoa Formosa	17.228
MG	31	37536	Lagoa Grande	8.710
MG	31	37601	Lagoa Santa	53.645
MG	31	37700	Lajinha	19.616
MG	31	37809	Lambari	19.655
MG	31	37908	Lamim	3.442
MG	31	38005	Laranjal	6.492
MG	31	38104	Lassance	6.479
MG	31	38203	Lavras	93.231
MG	31	38302	Leandro Ferreira	3.204
MG	31	38351	Leme do Prado	4.810
MG	31	38401	Leopoldina	51.210
MG	31	38500	Liberdade	5.312
MG	31	38609	Lima Duarte	16.183
MG	31	38625	Limeira do Oeste	6.946
MG	31	38658	Lontra	8.453
MG	31	38674	Luisburgo	6.230
MG	31	38682	Luislândia	6.422
MG	31	38708	Luminárias	5.418
MG	31	38807	Luz	17.537
MG	31	38906	Machacalis	6.981
MG	31	39003	Machado	38.981
MG	31	39102	Madre de Deus de Minas	4.918
MG	31	39201	Malacacheta	18.740
MG	31	39250	Mamonas	6.336
MG	31	39300	Manga	19.649
MG	31	39409	Manhuaçu	80.530
MG	31	39508	Manhumirim	21.487
MG	31	39607	Mantena	27.130
MG	31	39805	Mar de Espanha	11.840
MG	31	39706	Maravilhas	7.235
MG	31	39904	Maria da Fé	14.186
MG	31	40001	Mariana	54.796
MG	31	40100	Marilac	4.204
MG	31	40159	Mário Campos	13.396
MG	31	40209	Maripá de Minas	2.803
MG	31	40308	Marliéria	4.010
MG	31	40407	Marmelópolis	2.944
MG	31	40506	Martinho Campos	12.672
MG	31	40530	Martins Soares	7.288
MG	31	40555	Mata Verde	7.935
MG	31	40605	Materlândia	4.576
MG	31	40704	Mateus Leme	28.141
MG	31	71501	Mathias Lobato	3.350
MG	31	40803	Matias Barbosa	13.521
MG	31	40852	Matias Cardoso	10.085
MG	31	40902	Matipó	17.743
MG	31	41009	Mato Verde	12.646
MG	31	41108	Matozinhos	34.295

MG	31	41207	Matutina	3.756
MG	31	41306	Medeiros	3.476
MG	31	41405	Medina	20.979
MG	31	41504	Mendes Pimentel	6.335
MG	31	41603	Mercês	10.392
MG	31	41702	Mesquita	6.016
MG	31	41801	Minas Novas	30.824
MG	31	41900	Minduri	3.841
MG	31	42007	Mirabela	13.080
MG	31	42106	Miradouro	10.288
MG	31	42205	Miraí	13.911
MG	31	42254	Miravânia	4.577
MG	31	42304	Moeda	4.706
MG	31	42403	Moema	7.068
MG	31	42502	Monjolos	2.344
MG	31	42601	Monsenhor Paulo	8.203
MG	31	42700	Montalvânia	15.745
MG	31	42809	Monte Alegre de Minas	19.743
MG	31	42908	Monte Azul	21.853
MG	31	43005	Monte Belo	13.055
MG	31	43104	Monte Carmelo	45.916
MG	31	43153	Monte Formoso	4.675
MG	31	43203	Monte Santo de Minas	21.236
MG	31	43401	Monte Sião	21.434
MG	31	43302	Montes Claros	366.135
MG	31	43450	Montezuma	7.533
MG	31	43500	Morada Nova de Minas	8.305
MG	31	43609	Morro da Garça	2.637
MG	31	43708	Morro do Pilar	3.374
MG	31	43807	Munhoz	6.227
MG	31	43906	Muriaé	101.431
MG	31	44003	Mutum	26.659
MG	31	44102	Muzambinho	20.418
MG	31	44201	Nacip Raydan	3.157
MG	31	44300	Nanuque	40.774
MG	31	44359	Naque	6.398
MG	31	44375	Natalândia	3.280
MG	31	44409	Natércia	4.660
MG	31	44508	Nazareno	8.009
MG	31	44607	Nepomuceno	25.803
MG	31	44656	Ninheira	9.851
MG	31	44672	Nova Belém	3.674
MG	31	44706	Nova Era	17.511
MG	31	44805	Nova Lima	82.273
MG	31	44904	Nova Módica	3.767
MG	31	45000	Nova Ponte	13.067
MG	31	45059	Nova Porteirinha	7.399
MG	31	45109	Nova Resende	15.489
MG	31	45208	Nova Serrana	76.482
MG	31	36603	Nova União	5.565
MG	31	45307	Novo Cruzeiro	30.746
MG	31	45356	Novo Oriente de Minas	10.368
MG	31	45372	Novorizonte	4.991
MG	31	45406	Olaria	1.951
MG	31	45455	Olhos-d'Água	5.343
MG	31	45505	Olímpio Noronha	2.555
MG	31	45604	Oliveira	39.637

MG	31	45703	Oliveira Fortes	2.122
MG	31	45802	Onça de Pitangui	3.061
MG	31	45851	Oratórios	4.504
MG	31	45877	Orizânia	7.348
MG	31	45901	Ouro Branco	35.643
MG	31	46008	Ouro Fino	31.734
MG	31	46107	Ouro Preto	70.589
MG	31	46206	Ouro Verde de Minas	6.001
MG	31	46255	Padre Carvalho	5.881
MG	31	46305	Padre Paraíso	18.955
MG	31	46552	Pai Pedro	5.942
MG	31	46404	Paineiras	4.611
MG	31	46503	Pains	8.031
MG	31	46602	Paiva	1.554
MG	31	46701	Palma	6.544
MG	31	46750	Palmópolis	6.781
MG	31	46909	Papagaios	14.306
MG	31	47105	Pará de Minas	85.076
MG	31	47006	Paracatu	85.448
MG	31	47204	Paraguaçu	20.346
MG	31	47303	Paraisópolis	19.524
MG	31	47402	Paraopeba	22.731
MG	31	47600	Passa Quatro	15.638
MG	31	47709	Passa Tempo	8.176
MG	31	47501	Passabém	1.753
MG	31	47808	Passa-Vinte	2.073
MG	31	47907	Passos	106.987
MG	31	47956	Patis	5.611
MG	31	48004	Patos de Minas	139.849
MG	31	48103	Patrocínio	83.188
MG	31	48202	Patrocínio do Muriaé	5.320
MG	31	48301	Paula Cândido	9.289
MG	31	48400	Paulistas	4.904
MG	31	48509	Pavão	8.565
MG	31	48608	Peçanha	17.266
MG	31	48707	Pedra Azul	23.857
MG	31	48756	Pedra Bonita	6.707
MG	31	48806	Pedra do Anta	3.323
MG	31	48905	Pedra do Indaiá	3.880
MG	31	49002	Pedra Dourada	2.220
MG	31	49101	Pedralva	11.426
MG	31	49150	Pedras de Maria da Cruz	10.426
MG	31	49200	Pedrinópolis	3.500
MG	31	49309	Pedro Leopoldo	59.213
MG	31	49408	Pedro Teixeira	1.785
MG	31	49507	Pequeri	3.177
MG	31	49606	Pequi	4.104
MG	31	49705	Perdigão	9.159
MG	31	49804	Perdizes	14.561
MG	31	49903	Perdões	20.191
MG	31	49952	Periquito	7.005
MG	31	50000	Pescador	4.135
MG	31	50109	Piau	2.829
MG	31	50158	Piedade de Caratinga	7.246
MG	31	50208	Piedade de Ponte Nova	4.065
MG	31	50307	Piedade do Rio Grande	4.682
MG	31	50406	Piedade dos Gerais	4.669

MG	31	50505	Pimenta	8.268
MG	31	50539	Pingo-d'Água	4.467
MG	31	50570	Pintópolis	7.232
MG	31	50604	Piracema	6.399
MG	31	50703	Pirajuba	4.803
MG	31	50802	Piranga	17.250
MG	31	50901	Piranguçu	5.236
MG	31	51008	Piranguinho	8.064
MG	31	51107	Pirapetinga	10.390
MG	31	51206	Pirapora	53.604
MG	31	51305	Piraúba	10.841
MG	31	51404	Pitangui	25.545
MG	31	51503	Piumhi	32.121
MG	31	51602	Planura	10.545
MG	31	51701	Poço Fundo	16.022
MG	31	51800	Poços de Caldas	153.726
MG	31	51909	Pocrane	8.920
MG	31	52006	Pompéu	29.337
MG	31	52105	Ponte Nova	57.551
MG	31	52131	Ponto Chique	3.991
MG	31	52170	Ponto dos Volantes	11.408
MG	31	52204	Porteirinha	37.607
MG	31	52303	Porto Firme	10.490
MG	31	52402	Poté	15.736
MG	31	52501	Pouso Alegre	132.445
MG	31	52600	Pouso Alto	6.178
MG	31	52709	Prados	8.444
MG	31	52808	Prata	25.973
MG	31	52907	Pratápolis	8.776
MG	31	53004	Pratinha	3.295
MG	31	53103	Presidente Bernardes	5.514
MG	31	53202	Presidente Juscelino	3.877
MG	31	53301	Presidente Kubitschek	2.960
MG	31	53400	Presidente Olegário	18.639
MG	31	53608	Prudente de Morais	9.676
MG	31	53707	Quartel Geral	3.325
MG	31	53806	Queluzito	1.867
MG	31	53905	Raposos	15.423
MG	31	54002	Raul Soares	23.783
MG	31	54101	Recreio	10.308
MG	31	54150	Reduto	6.619
MG	31	54200	Resende Costa	10.958
MG	31	54309	Resplendor	17.098
MG	31	54408	Ressaquinha	4.723
MG	31	54457	Riachinho	8.010
MG	31	54507	Riacho dos Machados	9.361
MG	31	54606	Ribeirão das Neves	299.729
MG	31	54705	Ribeirão Vermelho	3.842
MG	31	54804	Rio Acima	9.200
MG	31	54903	Rio Casca	14.120
MG	31	55108	Rio do Prado	5.204
MG	31	55009	Rio Doce	2.477
MG	31	55207	Rio Espera	6.004
MG	31	55306	Rio Manso	5.325
MG	31	55405	Rio Novo	8.725
MG	31	55504	Rio Paranaíba	11.913
MG	31	55603	Rio Pardo de Minas	29.242

MG	31	55702	Rio Piracicaba	14.150
MG	31	55801	Rio Pomba	17.168
MG	31	55900	Rio Preto	5.304
MG	31	56007	Rio Vermelho	13.549
MG	31	56106	Ritápolis	4.887
MG	31	56205	Rochedo de Minas	2.133
MG	31	56304	Rodeiro	6.982
MG	31	56403	Romaria	3.586
MG	31	56452	Rosário da Limeira	4.277
MG	31	56502	Rubelita	7.586
MG	31	56601	Rubim	9.939
MG	31	56700	Sabará	127.097
MG	31	56809	Sabinópolis	15.661
MG	31	56908	Sacramento	24.093
MG	31	57005	Salinas	39.367
MG	31	57104	Salto da Divisa	6.866
MG	31	57203	Santa Bárbara	28.160
MG	31	57252	Santa Bárbara do Leste	7.719
MG	31	57278	Santa Bárbara do Monte Verde	2.821
MG	31	57302	Santa Bárbara do Tugúrio	4.551
MG	31	57336	Santa Cruz de Minas	7.929
MG	31	57377	Santa Cruz de Salinas	4.366
MG	31	57401	Santa Cruz do Escalvado	4.963
MG	31	57500	Santa Efigênia de Minas	4.576
MG	31	57609	Santa Fé de Minas	3.951
MG	31	57658	Santa Helena de Minas	6.079
MG	31	57708	Santa Juliana	11.588
MG	31	57807	Santa Luzia	204.327
MG	31	57906	Santa Margarida	15.111
MG	31	58003	Santa Maria de Itabira	10.568
MG	31	58102	Santa Maria do Salto	5.273
MG	31	58201	Santa Maria do Suaçuí	14.399
MG	31	59209	Santa Rita de Caldas	9.008
MG	31	59407	Santa Rita de Ibitipoca	3.563
MG	31	59308	Santa Rita de Jacutinga	4.976
MG	31	59357	Santa Rita de Minas	6.605
MG	31	59506	Santa Rita do Itueto	5.670
MG	31	59605	Santa Rita do Sapucaí	38.253
MG	31	59704	Santa Rosa da Serra	3.233
MG	31	59803	Santa Vitória	18.275
MG	31	58300	Santana da Vargem	7.209
MG	31	58409	Santana de Cataguases	3.643
MG	31	58508	Santana de Pirapama	7.963
MG	31	58607	Santana do Deserto	3.867
MG	31	58706	Santana do Garambéu	2.254
MG	31	58805	Santana do Jacaré	4.623
MG	31	58904	Santana do Manhuaçu	8.581
MG	31	58953	Santana do Paraíso	27.965
MG	31	59001	Santana do Riacho	4.045
MG	31	59100	Santana dos Montes	3.813
MG	31	59902	Santo Antônio do Amparo	17.440
MG	31	60009	Santo Antônio do Aventureiro	3.540
MG	31	60108	Santo Antônio do Grama	4.063
MG	31	60207	Santo Antônio do Itambé	4.101
MG	31	60306	Santo Antônio do Jacinto	11.747
MG	31	60405	Santo Antônio do Monte	26.168
MG	31	60454	Santo Antônio do Retiro	6.979

MG	31	60504	Santo Antônio do Rio Abaixo	1.774
MG	31	60603	Santo Hipólito	3.219
MG	31	60702	Santos Dumont	46.246
MG	31	60801	São Bento Abade	4.642
MG	31	60900	São Brás do Suaçuí	3.531
MG	31	60959	São Domingos das Dores	5.425
MG	31	61007	São Domingos do Prata	17.336
MG	31	61056	São Félix de Minas	3.377
MG	31	61106	São Francisco	54.007
MG	31	61205	São Francisco de Paula	6.480
MG	31	61304	São Francisco de Sales	5.815
MG	31	61403	São Francisco do Glória	5.139
MG	31	61502	São Geraldo	10.459
MG	31	61601	São Geraldo da Piedade	4.341
MG	31	61650	São Geraldo do Baixio	3.534
MG	31	61700	São Gonçalo do Abaeté	6.328
MG	31	61809	São Gonçalo do Pará	10.585
MG	31	61908	São Gonçalo do Rio Abaixo	9.878
MG	31	25507	São Gonçalo do Rio Preto	3.064
MG	31	62005	São Gonçalo do Sapucaí	24.029
MG	31	62104	São Gotardo	32.141
MG	31	62203	São João Batista do Glória	6.935
MG	31	62252	São João da Lagoa	4.676
MG	31	62302	São João da Mata	2.730
MG	31	62401	São João da Ponte	25.307
MG	31	62450	São João das Missões	11.829
MG	31	62500	São João del Rei	84.919
MG	31	62559	São João do Manhuaçu	10.363
MG	31	62575	São João do Manteninha	5.249
MG	31	62609	São João do Oriente	7.827
MG	31	62658	São João do Pacuí	4.091
MG	31	62708	São João do Paraíso	22.420
MG	31	62807	São João Evangelista	15.556
MG	31	62906	São João Nepomuceno	25.155
MG	31	62922	São Joaquim de Bicas	26.104
MG	31	62948	São José da Barra	6.834
MG	31	62955	São José da Lapa	20.168
MG	31	63003	São José da Safira	4.089
MG	31	63102	São José da Varginha	4.273
MG	31	63201	São José do Alegre	4.011
MG	31	63300	São José do Divino	3.832
MG	31	63409	São José do Goiabal	5.608
MG	31	63508	São José do Jacuri	6.535
MG	31	63607	São José do Mantimento	2.609
MG	31	63706	São Lourenço	42.021
MG	31	63805	São Miguel do Anta	6.770
MG	31	63904	São Pedro da União	4.996
MG	31	64100	São Pedro do Suaçuí	5.531
MG	31	64001	São Pedro dos Ferros	8.289
MG	31	64209	São Romão	10.468
MG	31	64308	São Roque de Minas	6.714
MG	31	64407	São Sebastião da Bela Vista	4.997
MG	31	64431	São Sebastião da Vargem Alegre	2.816
MG	31	64472	São Sebastião do Anta	5.813
MG	31	64506	São Sebastião do Maranhão	10.574
MG	31	64605	São Sebastião do Oeste	5.894
MG	31	64704	São Sebastião do Paraíso	65.491

MG	31	64803	São Sebastião do Rio Preto	1.601
MG	31	64902	São Sebastião do Rio Verde	2.121
MG	31	65206	São Thomé das Letras	6.690
MG	31	65008	São Tiago	10.586
MG	31	65107	São Tomás de Aquino	7.077
MG	31	65305	São Vicente de Minas	7.073
MG	31	65404	Sapucaí-Mirim	6.302
MG	31	65503	Sardoá	5.657
MG	31	65537	Sarzedo	26.470
MG	31	65560	Sem-Peixe	2.823
MG	31	65578	Senador Amaral	5.226
MG	31	65602	Senador Cortes	1.988
MG	31	65701	Senador Firmino	7.279
MG	31	65800	Senador José Bento	1.830
MG	31	65909	Senador Modestino Gonçalves	4.527
MG	31	66006	Senhora de Oliveira	5.687
MG	31	66105	Senhora do Porto	3.496
MG	31	66204	Senhora dos Remédios	10.210
MG	31	66303	Sericita	7.139
MG	31	66402	Seritinga	1.793
MG	31	66501	Serra Azul de Minas	4.222
MG	31	66600	Serra da Saudade	811
MG	31	66808	Serra do Salitre	10.638
MG	31	66709	Serra dos Aimorés	8.430
MG	31	66907	Serrania	7.545
MG	31	66956	Serranópolis de Minas	4.455
MG	31	67004	Serranos	1.990
MG	31	67103	Serro	20.822
MG	31	67202	Sete Lagoas	216.400
MG	31	65552	Setubinha	11.008
MG	31	67301	Silveirânia	2.197
MG	31	67400	Silvianópolis	6.041
MG	31	67509	Simão Pereira	2.542
MG	31	67608	Simonésia	18.408
MG	31	67707	Sobrália	5.796
MG	31	67806	Soledade de Minas	5.716
MG	31	67905	Tabuleiro	4.042
MG	31	68002	Taiobeiras	31.192
MG	31	68051	Taparuba	3.131
MG	31	68101	Tapira	4.173
MG	31	68200	Tapiraí	1.871
MG	31	68309	Taquaraçu de Minas	3.818
MG	31	68408	Tarumirim	14.279
MG	31	68507	Teixeiras	11.371
MG	31	68606	Teófilo Otoni	135.154
MG	31	68705	Timóteo	81.993
MG	31	68804	Tiradentes	7.054
MG	31	68903	Tiros	6.855
MG	31	69000	Tocantins	15.886
MG	31	69059	Tocos do Moji	3.960
MG	31	69109	Toledo	5.806
MG	31	69208	Tombos	9.375
MG	31	69307	Três Corações	73.339
MG	31	69356	Três Marias	28.683
MG	31	69406	Três Pontas	54.078
MG	31	69505	Tumiritinga	6.329
MG	31	69604	Tupaciguara	24.271

MG	31	69703	Turmalina	18.222
MG	31	69802	Turvolândia	4.690
MG	31	69901	Ubá	102.782
MG	31	70008	Ubaí	11.751
MG	31	70057	Ubaporanga	12.068
MG	31	70107	Uberaba	299.361
MG	31	70206	Uberlândia	611.904
MG	31	70305	Umburatiba	2.693
MG	31	70404	Unaí	78.144
MG	31	70438	União de Minas	4.402
MG	31	70479	Uruana de Minas	3.233
MG	31	70503	Urucânia	10.285
MG	31	70529	Urucuia	13.911
MG	31	70578	Vargem Alegre	6.455
MG	31	70602	Vargem Bonita	2.160
MG	31	70651	Vargem Grande do Rio Pardo	4.755
MG	31	70701	Varginha	124.162
MG	31	70750	Varjão de Minas	6.158
MG	31	70800	Várzea da Palma	36.129
MG	31	70909	Varzelândia	19.112
MG	31	71006	Vazante	19.785
MG	31	71030	Verdelândia	8.436
MG	31	71071	Veredinha	5.559
MG	31	71105	Veríssimo	3.530
MG	31	71154	Vermelho Novo	4.698
MG	31	71204	Vespasiano	106.685
MG	31	71303	Viçosa	72.786
MG	31	71402	Vieiras	3.715
MG	31	71600	Virgem da Lapa	13.615
MG	31	71709	Virgínia	8.618
MG	31	71808	Virginópolis	10.553
MG	31	71907	Virgolândia	5.624
MG	31	72004	Visconde do Rio Branco	38.353
MG	31	72103	Volta Grande	5.082
MG	31	72202	Wenceslau Braz	2.550
ES	32	00102	Afonso Cláudio	31.004
ES	32	00169	Água Doce do Norte	11.696
ES	32	00136	Águia Branca	9.513
ES	32	00201	Alegre	30.696
ES	32	00300	Alfredo Chaves	13.982
ES	32	00359	Alto Rio Novo	7.345
ES	32	00409	Anchieta	24.265
ES	32	00508	Apiacá	7.505
ES	32	00607	Aracruz	83.152
ES	32	00706	Atilio Vivacqua	9.967
ES	32	00805	Baixo Guandu	29.178
ES	32	00904	Barra de São Francisco	40.884
ES	32	01001	Boa Esperança	14.239
ES	32	01100	Bom Jesus do Norte	9.496
ES	32	01159	Brejetuba	11.933
ES	32	01209	Cachoeiro de Itapemirim	191.042
ES	32	01308	Cariacica	350.615
ES	32	01407	Castelo	34.900
ES	32	01506	Colatina	112.432
ES	32	01605	Conceição da Barra	28.600
ES	32	01704	Conceição do Castelo	11.741
ES	32	01803	Divino de São Lourenço	4.493

ES	32	01902	Domingos Martins	31.946
ES	32	02009	Dores do Rio Preto	6.414
ES	32	02108	Ecoporanga	23.154
ES	32	02207	Fundão	17.334
ES	32	02256	Governador Lindenberg	10.990
ES	32	02306	Guaçuí	28.033
ES	32	02405	Guarapari	106.583
ES	32	02454	Ibatiba	22.609
ES	32	02504	Ibiraçu	11.258
ES	32	02553	Ibitirama	8.938
ES	32	02603	Iconha	12.603
ES	32	02652	Irupi	11.829
ES	32	02702	Itaguaçu	14.107
ES	32	02801	Itapemirim	31.209
ES	32	02900	Itarana	10.840
ES	32	03007	Iúna	27.422
ES	32	03056	Jaguaré	25.073
ES	32	03106	Jerônimo Monteiro	10.932
ES	32	03130	João Neiva	15.848
ES	32	03163	Laranja da Terra	10.818
ES	32	03205	Linhares	143.509
ES	32	03304	Mantenópolis	13.721
ES	32	03320	Marataízes	34.412
ES	32	03346	Marechal Floriano	14.422
ES	32	03353	Marilândia	11.198
ES	32	03403	Mimoso do Sul	25.880
ES	32	03502	Montanha	17.894
ES	32	03601	Mucurici	5.637
ES	32	03700	Muniz Freire	18.298
ES	32	03809	Muqui	14.452
ES	32	03908	Nova Venécia	46.263
ES	32	04005	Pancas	21.636
ES	32	04054	Pedro Canário	23.935
ES	32	04104	Pinheiros	24.093
ES	32	04203	Piúma	18.364
ES	32	04252	Ponto Belo	7.034
ES	32	04302	Presidente Kennedy	10.373
ES	32	04351	Rio Bananal	17.623
ES	32	04401	Rio Novo do Sul	11.330
ES	32	04500	Santa Leopoldina	12.223
ES	32	04559	Santa Maria de Jetibá	34.591
ES	32	04609	Santa Teresa	21.916
ES	32	04658	São Domingos do Norte	8.036
ES	32	04708	São Gabriel da Palha	32.264
ES	32	04807	São José do Calçado	10.403
ES	32	04906	São Mateus	110.454
ES	32	04955	São Roque do Canaã	11.341
ES	32	05002	Serra	416.029
ES	32	05010	Sooretama	24.271
ES	32	05036	Vargem Alta	19.265
ES	32	05069	Venda Nova do Imigrante	20.776
ES	32	05101	Viana	65.888
ES	32	05150	Vila Pavão	8.699
ES	32	05176	Vila Valério	13.827
ES	32	05200	Vila Velha	419.854
ES	32	05309	Vitória	330.526
RJ	33	00100	Angra dos Reis	173.370

RJ	33	00159	Aperibé	10.382
RJ	33	00209	Araruama	114.250
RJ	33	00225	Areal	11.540
RJ	33	00233	Armação dos Búzios	28.279
RJ	33	00258	Arraial do Cabo	28.010
RJ	33	00308	Barra do Piraí	95.260
RJ	33	00407	Barra Mansa	178.355
RJ	33	00456	Belford Roxo	472.008
RJ	33	00506	Bom Jardim	25.539
RJ	33	00605	Bom Jesus do Itabapoana	35.546
RJ	33	00704	Cabo Frio	190.787
RJ	33	00803	Cachoeiras de Macacu	54.713
RJ	33	00902	Cambuci	14.840
RJ	33	01009	Campos dos Goytacazes	468.087
RJ	33	01108	Cantagalo	19.830
RJ	33	00936	Carapebus	13.697
RJ	33	01157	Cardoso Moreira	12.601
RJ	33	01207	Carmo	17.599
RJ	33	01306	Casimiro de Abreu	36.360
RJ	33	00951	Comendador Levy Gasparian	8.200
RJ	33	01405	Conceição de Macabu	21.416
RJ	33	01504	Cordeiro	20.571
RJ	33	01603	Duas Barras	10.976
RJ	33	01702	Duque de Caxias	861.158
RJ	33	01801	Engenheiro Paulo de Frontin	13.324
RJ	33	01850	Guapimirim	52.522
RJ	33	01876	Iguaba Grande	23.475
RJ	33	01900	Itaboraí	220.352
RJ	33	02007	Itaguaí	111.171
RJ	33	02056	Italva	14.174
RJ	33	02106	Itaocara	22.892
RJ	33	02205	Itaperuna	96.542
RJ	33	02254	Itatiaia	29.094
RJ	33	02270	Japeri	96.430
RJ	33	02304	Laje do Muriaé	7.455
RJ	33	02403	Macaé	212.433
RJ	33	02452	Macuco	5.299
RJ	33	02502	Magé	228.972
RJ	33	02601	Mangaratiba	37.343
RJ	33	02700	Maricá	131.355
RJ	33	02809	Mendes	17.981
RJ	33	02858	Mesquita	168.966
RJ	33	02908	Miguel Pereira	24.699
RJ	33	03005	Miracema	26.827
RJ	33	03104	Natividade	15.079
RJ	33	03203	Nilópolis	157.710
RJ	33	03302	Niterói	489.720
RJ	33	03401	Nova Friburgo	182.748
RJ	33	03500	Nova Iguaçu	799.047
RJ	33	03609	Paracambi	47.635
RJ	33	03708	Paraíba do Sul	41.367
RJ	33	03807	Parati	38.147
RJ	33	03856	Paty do Alferes	26.469
RJ	33	03906	Petrópolis	296.565
RJ	33	03955	Pinheiral	22.968
RJ	33	04003	Piraí	26.637
RJ	33	04102	Porciúncula	17.899

RJ	33	04110	Porto Real	16.938
RJ	33	04128	Quatis	12.952
RJ	33	04144	Queimados	139.188
RJ	33	04151	Quissamã	20.747
RJ	33	04201	Resende	120.938
RJ	33	04300	Rio Bonito	56.001
RJ	33	04409	Rio Claro	17.517
RJ	33	04508	Rio das Flores	8.633
RJ	33	04524	Rio das Ostras	110.992
RJ	33	04557	Rio de Janeiro	6.355.949
RJ	33	04607	Santa Maria Madalena	10.310
RJ	33	04706	Santo Antônio de Pádua	40.735
RJ	33	04805	São Fidélis	37.601
RJ	33	04755	São Francisco de Itabapoana	41.371
RJ	33	04904	São Gonçalo	1.008.065
RJ	33	05000	São João da Barra	33.136
RJ	33	05109	São João de Meriti	459.379
RJ	33	05133	São José de Ubá	7.049
RJ	33	05158	São José do Vale do Rio Preto	20.398
RJ	33	05208	São Pedro da Aldeia	89.739
RJ	33	05307	São Sebastião do Alto	8.933
RJ	33	05406	Sapucaia	17.554
RJ	33	05505	Saquarema	75.906
RJ	33	05554	Seropédica	79.179
RJ	33	05604	Silva Jardim	21.356
RJ	33	05703	Sumidouro	14.956
RJ	33	05752	Tanguá	31.091
RJ	33	05802	Teresópolis	165.716
RJ	33	05901	Trajano de Morais	10.309
RJ	33	06008	Três Rios	77.851
RJ	33	06107	Valença	72.268
RJ	33	06156	Varre-Sai	9.600
RJ	33	06206	Vassouras	34.638
RJ	33	06305	Volta Redonda	259.012
SP	35	00105	Adamantina	33.821
SP	35	00204	Adolfo	3.548
SP	35	00303	Aguaí	32.452
SP	35	00402	Águas da Prata	7.619
SP	35	00501	Águas de Lindóia	17.354
SP	35	00550	Águas de Santa Bárbara	5.630
SP	35	00600	Águas de São Pedro	2.771
SP	35	00709	Agudos	34.681
SP	35	00758	Alambari	4.979
SP	35	00808	Alfredo Marcondes	3.906
SP	35	00907	Altair	3.837
SP	35	01004	Altinópolis	15.617
SP	35	01103	Alto Alegre	4.090
SP	35	01152	Alumínio	16.961
SP	35	01202	Álvares Florence	3.865
SP	35	01301	Álvares Machado	23.579
SP	35	01400	Álvaro de Carvalho	4.692
SP	35	01509	Alvinlândia	3.013
SP	35	01608	Americana	212.791
SP	35	01707	Américo Brasiliense	34.954
SP	35	01806	Américo de Campos	5.715
SP	35	01905	Amparo	66.246
SP	35	02002	Analândia	4.348

SP	35	02101	Andradina	55.348
SP	35	02200	Angatuba	22.434
SP	35	02309	Anhembi	5.739
SP	35	02408	Anhumas	3.764
SP	35	02507	Aparecida	35.015
SP	35	02606	Aparecida d'Oeste	4.413
SP	35	02705	Apiaí	25.040
SP	35	02754	Araçariguama	17.535
SP	35	02804	Araçatuba	182.526
SP	35	02903	Araçoiaba da Serra	27.874
SP	35	03000	Aramina	5.182
SP	35	03109	Arandu	6.128
SP	35	03158	Arapeí	2.484
SP	35	03208	Araraquara	210.673
SP	35	03307	Araras	119.968
SP	35	03356	Arco-Íris	1.907
SP	35	03406	Arealva	7.887
SP	35	03505	Areias	3.704
SP	35	03604	Areiópolis	10.601
SP	35	03703	Ariranha	8.630
SP	35	03802	Artur Nogueira	45.026
SP	35	03901	Arujá	76.112
SP	35	03950	Aspásia	1.806
SP	35	04008	Assis	95.750
SP	35	04107	Atibaia	127.778
SP	35	04206	Auriflama	14.255
SP	35	04305	Avaí	4.987
SP	35	04404	Avanhandava	11.501
SP	35	04503	Avaré	83.430
SP	35	04602	Bady Bassitt	14.838
SP	35	04701	Balbinos	3.886
SP	35	04800	Bálsamo	8.223
SP	35	04909	Bananal	10.263
SP	35	05005	Barão de Antonina	3.141
SP	35	05104	Barbosa	6.652
SP	35	05203	Bariri	31.852
SP	35	05302	Barra Bonita	35.228
SP	35	05351	Barra do Chapéu	5.275
SP	35	05401	Barra do Turvo	7.700
SP	35	05500	Barretos	112.730
SP	35	05609	Barrinha	28.826
SP	35	05708	Barueri	243.242
SP	35	05807	Bastos	20.435
SP	35	05906	Batatais	56.888
SP	35	06003	Bauru	346.077
SP	35	06102	Bebedouro	75.052
SP	35	06201	Bento de Abreu	2.696
SP	35	06300	Bernardino de Campos	10.780
SP	35	06359	Bertioga	48.997
SP	35	06409	Bilac	7.122
SP	35	06508	Birigui	109.836
SP	35	06607	Biritiba-Mirim	28.877
SP	35	06706	Boa Esperança do Sul	13.728
SP	35	06805	Bocaina	10.968
SP	35	06904	Bofete	9.792
SP	35	07001	Boituva	49.385
SP	35	07100	Bom Jesus dos Perdões	20.199

SP	35	07159	Bom Sucesso de Itararé	3.598
SP	35	07209	Borá	806
SP	35	07308	Boracéia	4.309
SP	35	07407	Borborema	14.632
SP	35	07456	Borebi	2.321
SP	35	07506	Botucatu	128.789
SP	35	07605	Bragança Paulista	148.411
SP	35	07704	Braúna	5.070
SP	35	07753	Brejo Alegre	2.594
SP	35	07803	Brodowski	21.412
SP	35	07902	Brotas	21.787
SP	35	08009	Buri	18.635
SP	35	08108	Buritama	15.539
SP	35	08207	Buritizal	4.083
SP	35	08306	Cabrália Paulista	4.343
SP	35	08405	Cabreúva	42.257
SP	35	08504	Caçapava	85.414
SP	35	08603	Cachoeira Paulista	30.313
SP	35	08702	Caconde	18.551
SP	35	08801	Cafelândia	16.670
SP	35	08900	Caiabu	4.072
SP	35	09007	Caieiras	87.704
SP	35	09106	Caiuá	5.105
SP	35	09205	Cajamar	65.139
SP	35	09254	Cajati	28.307
SP	35	09304	Cajobi	9.814
SP	35	09403	Cajuru	23.571
SP	35	09452	Campina do Monte Alegre	5.595
SP	35	09502	Campinas	1.090.386
SP	35	09601	Campo Limpo Paulista	74.869
SP	35	09700	Campos do Jordão	48.061
SP	35	09809	Campos Novos Paulista	4.567
SP	35	09908	Cananéia	12.221
SP	35	09957	Canas	4.445
SP	35	10005	Cândido Mota	29.931
SP	35	10104	Cândido Rodrigues	2.673
SP	35	10153	Canitar	4.438
SP	35	10203	Capão Bonito	46.136
SP	35	10302	Capela do Alto	17.785
SP	35	10401	Capivari	49.122
SP	35	10500	Caraguatatuba	102.523
SP	35	10609	Carapicuíba	371.502
SP	35	10708	Cardoso	11.821
SP	35	10807	Casa Branca	28.423
SP	35	10906	Cássia dos Coqueiros	2.616
SP	35	11003	Castilho	18.238
SP	35	11102	Catanduva	113.356
SP	35	11201	Catiguá	7.171
SP	35	11300	Cedral	8.070
SP	35	11409	Cerqueira César	17.716
SP	35	11508	Cerquilho	40.393
SP	35	11607	Cesário Lange	15.744
SP	35	11706	Charqueada	15.243
SP	35	57204	Chavantes	12.108
SP	35	11904	Clementina	7.193
SP	35	12001	Colina	17.426
SP	35	12100	Colômbia	5.998

SP	35	12209	Conchal	25.425
SP	35	12308	Conchas	16.395
SP	35	12407	Cordeirópolis	21.348
SP	35	12506	Coroados	5.302
SP	35	12605	Coronel Macedo	4.956
SP	35	12704	Corumbataí	3.881
SP	35	12803	Cosmópolis	59.938
SP	35	12902	Cosmorama	7.202
SP	35	13009	Cotia	205.154
SP	35	13108	Cravinhos	31.943
SP	35	13207	Cristais Paulista	7.666
SP	35	13306	Cruzália	2.249
SP	35	13405	Cruzeiro	77.312
SP	35	13504	Cubatão	119.520
SP	35	13603	Cunha	21.773
SP	35	13702	Descalvado	31.220
SP	35	13801	Diadema	388.576
SP	35	13850	Dirce Reis	1.695
SP	35	13900	Divinolândia	11.146
SP	35	14007	Dobrada	8.011
SP	35	14106	Dois Córregos	24.933
SP	35	14205	Dolcinópolis	2.092
SP	35	14304	Dourado	8.610
SP	35	14403	Dracena	43.470
SP	35	14502	Duartina	12.234
SP	35	14601	Dumont	8.284
SP	35	14700	Echaporã	6.279
SP	35	14809	Eldorado	14.680
SP	35	14908	Elias Fausto	15.920
SP	35	14924	Elisiário	3.162
SP	35	14957	Embaúba	2.419
SP	35	15004	Embu	242.730
SP	35	15103	Embu-Guaçu	63.219
SP	35	15129	Emilianópolis	3.030
SP	35	15152	Engenheiro Coelho	16.158
SP	35	15186	Espírito Santo do Pinhal	42.017
SP	35	15194	Espírito Santo do Turvo	4.288
SP	35	57303	Estiva Gerbi	10.136
SP	35	15301	Estrela do Norte	2.661
SP	35	15202	Estrela d'Oeste	8.205
SP	35	15350	Euclides da Cunha Paulista	9.537
SP	35	15400	Fartura	15.344
SP	35	15608	Fernando Prestes	5.542
SP	35	15509	Fernandópolis	64.931
SP	35	15657	Fernão	1.574
SP	35	15707	Ferraz de Vasconcelos	170.297
SP	35	15806	Flora Rica	1.720
SP	35	15905	Floreal	2.987
SP	35	16002	Flórida Paulista	12.982
SP	35	16101	Florínia	2.807
SP	35	16200	Franca	321.012
SP	35	16309	Francisco Morato	156.064
SP	35	16408	Franco da Rocha	133.407
SP	35	16507	Gabriel Monteiro	2.707
SP	35	16606	Gália	6.947
SP	35	16705	Garça	43.112
SP	35	16804	Gastão Vidigal	4.240

SP	35	16853	Gavião Peixoto	4.442
SP	35	16903	General Salgado	10.658
SP	35	17000	Getulina	10.796
SP	35	17109	Glicério	4.576
SP	35	17208	Guaiçara	10.782
SP	35	17307	Guaimbê	5.442
SP	35	17406	Guaíra	37.619
SP	35	17505	Guapiaçu	18.160
SP	35	17604	Guapiara	17.866
SP	35	17703	Guará	19.931
SP	35	17802	Guaraçaí	8.400
SP	35	17901	Guaraci	10.063
SP	35	18008	Guarani d'Oeste	1.968
SP	35	18107	Guarantã	6.411
SP	35	18206	Guararapes	30.732
SP	35	18305	Guararema	26.147
SP	35	18404	Guaratinguetá	112.675
SP	35	18503	Guareí	14.901
SP	35	18602	Guariba	35.824
SP	35	18701	Guarujá	292.744
SP	35	18800	Guarulhos	1.233.436
SP	35	18859	Guatapará	7.012
SP	35	18909	Guzolândia	4.790
SP	35	19006	Herculândia	8.751
SP	35	19055	Holambra	11.613
SP	35	19071	Hortolândia	195.776
SP	35	19105	Iacanga	10.146
SP	35	19204	Iacri	6.392
SP	35	19253	Iaras	6.631
SP	35	19303	Ibaté	31.062
SP	35	19402	Ibirá	11.008
SP	35	19501	Ibirarema	6.804
SP	35	19600	Ibitinga	53.660
SP	35	19709	Ibiúna	71.742
SP	35	19808	Icém	7.515
SP	35	19907	Iepê	7.657
SP	35	20004	Igaraçu do Tietê	23.420
SP	35	20103	Igarapava	28.108
SP	35	20202	Igaratá	8.873
SP	35	20301	Iguape	28.950
SP	35	20426	Ilha Comprida	9.204
SP	35	20442	Ilha Solteira	25.146
SP	35	20400	Ilhabela	28.761
SP	35	20509	Indaiatuba	205.808
SP	35	20608	Indiana	4.817
SP	35	20707	Indiaporã	3.892
SP	35	20806	Inúbia Paulista	3.654
SP	35	20905	Ipaussu	13.749
SP	35	21002	Iperó	29.062
SP	35	21101	Ipeúna	6.145
SP	35	21150	Ipiguá	4.539
SP	35	21200	Iporanga	4.279
SP	35	21309	Ipuã	14.323
SP	35	21408	Iracemápolis	20.373
SP	35	21507	Irapuã	7.323
SP	35	21606	Irapuru	7.815
SP	35	21705	Itaberá	17.778

SP	35	21804	Itaí	24.236
SP	35	21903	Itajobi	14.582
SP	35	22000	Itaju	3.293
SP	35	22109	Itanhaém	88.214
SP	35	22158	Itaóca	3.229
SP	35	22208	Itapecerica da Serra	154.374
SP	35	22307	Itapetininga	145.822
SP	35	22406	Itapeva	88.129
SP	35	22505	Itapevi	203.712
SP	35	22604	Itapira	68.934
SP	35	22653	Itapirapuã Paulista	3.904
SP	35	22703	Itápolis	40.228
SP	35	22802	Itaporanga	14.564
SP	35	22901	Itapuí	12.312
SP	35	23008	Itapura	4.397
SP	35	23107	Itaquaquecetuba	325.518
SP	35	23206	Itararé	48.040
SP	35	23305	Itariri	15.614
SP	35	23404	Itatiba	103.028
SP	35	23503	Itatinga	18.253
SP	35	23602	Itirapina	15.731
SP	35	23701	Itirapuã	5.953
SP	35	23800	Itobi	7.553
SP	35	23909	Itu	155.589
SP	35	24006	Itupeva	46.294
SP	35	24105	Ituverava	38.882
SP	35	24204	Jaborandi	6.605
SP	35	24303	Jaboticabal	71.989
SP	35	24402	Jacareí	212.744
SP	35	24501	Jaci	5.776
SP	35	24600	Jacupiranga	17.221
SP	35	24709	Jaguariúna	45.441
SP	35	24808	Jales	47.076
SP	35	24907	Jambeiro	5.454
SP	35	25003	Jandira	109.614
SP	35	25102	Jardinópolis	38.194
SP	35	25201	Jarinu	24.370
SP	35	25300	Jaú	132.494
SP	35	25409	Jeriquara	3.151
SP	35	25508	Joanópolis	11.873
SP	35	25607	João Ramalho	4.174
SP	35	25706	José Bonifácio	33.074
SP	35	25805	Júlio Mesquita	4.451
SP	35	25854	Jumirim	2.845
SP	35	25904	Jundiaí	373.713
SP	35	26001	Junqueirópolis	18.859
SP	35	26100	Juquiá	19.149
SP	35	26209	Juquitiba	28.912
SP	35	26308	Lagoinha	4.833
SP	35	26407	Laranjal Paulista	25.490
SP	35	26506	Lavínia	9.060
SP	35	26605	Lavrinhas	6.635
SP	35	26704	Leme	92.601
SP	35	26803	Lençóis Paulista	61.919
SP	35	26902	Limeira	278.093
SP	35	27009	Lindóia	6.814
SP	35	27108	Lins	71.853

SP	35	27207	Lorena	82.887
SP	35	27256	Lourdes	2.138
SP	35	27306	Louveira	38.140
SP	35	27405	Lucélia	20.003
SP	35	27504	Lucianópolis	2.257
SP	35	27603	Luís Antônio	11.603
SP	35	27702	Luiziânia	5.089
SP	35	27801	Lupércio	4.363
SP	35	27900	Lutécia	2.700
SP	35	28007	Macatuba	16.298
SP	35	28106	Macaubal	7.685
SP	35	28205	Macedônia	3.657
SP	35	28304	Magda	3.184
SP	35	28403	Mairinque	43.473
SP	35	28502	Mairiporã	82.556
SP	35	28601	Manduri	9.048
SP	35	28700	Marabá Paulista	4.898
SP	35	28809	Maracaí	13.358
SP	35	28858	Marapoama	2.664
SP	35	28908	Mariápolis	3.921
SP	35	29005	Marília	218.229
SP	35	29104	Marinópolis	2.107
SP	35	29203	Martinópolis	24.363
SP	35	29302	Matão	77.173
SP	35	29401	Mauá	421.184
SP	35	29500	Mendonça	4.708
SP	35	29609	Meridiano	3.842
SP	35	29658	Mesópolis	1.883
SP	35	29708	Miguelópolis	20.561
SP	35	29807	Mineiros do Tietê	12.087
SP	35	30003	Mira Estrela	2.838
SP	35	29906	Miracatu	20.455
SP	35	30102	Mirandópolis	27.602
SP	35	30201	Mirante do Paranapanema	17.124
SP	35	30300	Mirassol	54.212
SP	35	30409	Mirassolândia	4.338
SP	35	30508	Mococa	66.345
SP	35	30607	Mogi das Cruzes	392.196
SP	35	30706	Mogi Guaçu	138.245
SP	35	30805	Moji Mirim	86.892
SP	35	30904	Mombuca	3.279
SP	35	31001	Monções	2.138
SP	35	31100	Mongaguá	47.153
SP	35	31209	Monte Alegre do Sul	7.216
SP	35	31308	Monte Alto	46.875
SP	35	31407	Monte Aprazível	22.002
SP	35	31506	Monte Azul Paulista	18.884
SP	35	31605	Monte Castelo	4.062
SP	35	31803	Monte Mor	49.841
SP	35	31704	Monteiro Lobato	4.159
SP	35	31902	Morro Agudo	29.400
SP	35	32009	Morungaba	11.912
SP	35	32058	Motuca	4.323
SP	35	32108	Murutinga do Sul	4.203
SP	35	32157	Nantes	2.741
SP	35	32207	Narandiba	4.330
SP	35	32306	Natividade da Serra	6.657

SP	35	32405	Nazaré Paulista	16.568
SP	35	32504	Neves Paulista	8.762
SP	35	32603	Nhandeara	10.766
SP	35	32702	Nipoã	4.352
SP	35	32801	Nova Aliança	5.978
SP	35	32827	Nova Campina	8.609
SP	35	32843	Nova Canaã Paulista	2.086
SP	35	32868	Nova Castilho	1.136
SP	35	32900	Nova Europa	9.453
SP	35	33007	Nova Granada	19.346
SP	35	33106	Nova Guataporanga	2.184
SP	35	33205	Nova Independência	3.146
SP	35	33304	Nova Luzitânia	3.495
SP	35	33403	Nova Odessa	51.946
SP	35	33254	Novais	4.697
SP	35	33502	Novo Horizonte	36.913
SP	35	33601	Nuporanga	6.856
SP	35	33700	Ocauçu	4.163
SP	35	33809	Óleo	2.649
SP	35	33908	Olímpia	50.332
SP	35	34005	Onda Verde	3.921
SP	35	34104	Oriente	6.120
SP	35	34203	Orindiúva	5.792
SP	35	34302	Orlândia	40.071
SP	35	34401	Osasco	667.826
SP	35	34500	Oscar Bressane	2.536
SP	35	34609	Osvaldo Cruz	31.015
SP	35	34708	Ourinhos	103.739
SP	35	34807	Ouro Verde	7.851
SP	35	34757	Ouroeste	8.568
SP	35	34906	Pacaembu	13.281
SP	35	35002	Palestina	11.201
SP	35	35101	Palmares Paulista	11.126
SP	35	35200	Palmeira d'Oeste	9.528
SP	35	35309	Palmital	21.224
SP	35	35408	Panorama	14.655
SP	35	35507	Paraguaçu Paulista	42.483
SP	35	35606	Paraibuna	17.418
SP	35	35705	Paraíso	5.934
SP	35	35804	Paranapanema	17.985
SP	35	35903	Paranapuã	3.830
SP	35	36000	Parapuã	10.825
SP	35	36109	Pardinho	5.648
SP	35	36208	Pariquera-Açu	18.508
SP	35	36257	Parisi	2.039
SP	35	36307	Patrocínio Paulista	13.122
SP	35	36406	Paulicéia	6.419
SP	35	36505	Paulínia	84.512
SP	35	36570	Paulistânia	1.779
SP	35	36604	Paulo de Faria	8.598
SP	35	36703	Pederneiras	41.872
SP	35	36802	Pedra Bela	5.794
SP	35	36901	Pedranópolis	2.545
SP	35	37008	Pedregulho	15.755
SP	35	37107	Pedreira	42.045
SP	35	37156	Pedrinhas Paulista	2.947
SP	35	37206	Pedro de Toledo	10.283

SP	35	37305	Penápolis	58.808
SP	35	37404	Pereira Barreto	24.957
SP	35	37503	Pereiras	7.549
SP	35	37602	Peruíbe	60.412
SP	35	37701	Piacatu	5.338
SP	35	37800	Piedade	52.298
SP	35	37909	Pilar do Sul	26.595
SP	35	38006	Pindamonhangaba	148.605
SP	35	38105	Pindorama	15.188
SP	35	38204	Pinhalzinho	13.268
SP	35	38303	Piquerobi	3.542
SP	35	38501	Piquete	14.024
SP	35	38600	Piracaia	25.252
SP	35	38709	Piracicaba	367.290
SP	35	38808	Piraju	28.520
SP	35	38907	Pirajuí	22.905
SP	35	39004	Pirangi	10.668
SP	35	39103	Pirapora do Bom Jesus	15.990
SP	35	39202	Pirapozinho	24.893
SP	35	39301	Pirassununga	70.482
SP	35	39400	Piratininga	12.187
SP	35	39509	Pitangueiras	35.626
SP	35	39608	Planalto	4.524
SP	35	39707	Platina	3.217
SP	35	39806	Poá	106.797
SP	35	39905	Poloni	5.443
SP	35	40002	Pompéia	20.102
SP	35	40101	Pongaí	3.465
SP	35	40200	Pontal	41.055
SP	35	40259	Pontalinda	4.116
SP	35	40309	Pontes Gestal	2.517
SP	35	40408	Populina	4.206
SP	35	40507	Porangaba	8.455
SP	35	40606	Porto Feliz	49.153
SP	35	40705	Porto Ferreira	51.705
SP	35	40754	Potim	19.842
SP	35	40804	Potirendaba	15.587
SP	35	40853	Pracinha	2.968
SP	35	40903	Pradópolis	17.720
SP	35	41000	Praia Grande	267.307
SP	35	41059	Pratânia	4.649
SP	35	41109	Presidente Alves	4.109
SP	35	41208	Presidente Bernardes	13.487
SP	35	41307	Presidente Epitácio	41.474
SP	35	41406	Presidente Prudente	209.025
SP	35	41505	Presidente Venceslau	37.954
SP	35	41604	Promissão	36.025
SP	35	41653	Quadra	3.281
SP	35	41703	Quatá	12.887
SP	35	41802	Queiroz	2.857
SP	35	41901	Queluz	11.478
SP	35	42008	Quintana	6.048
SP	35	42107	Rafard	8.632
SP	35	42206	Rancharia	28.807
SP	35	42305	Redenção da Serra	3.860
SP	35	42404	Regente Feijó	18.609
SP	35	42503	Reginópolis	7.522

SP	35	42602	Registro	54.301
SP	35	42701	Restinga	6.664
SP	35	42800	Ribeira	3.347
SP	35	42909	Ribeirão Bonito	12.204
SP	35	43006	Ribeirão Branco	18.042
SP	35	43105	Ribeirão Corrente	4.304
SP	35	43204	Ribeirão do Sul	4.443
SP	35	43238	Ribeirão dos Índios	2.185
SP	35	43253	Ribeirão Grande	7.425
SP	35	43303	Ribeirão Pires	113.726
SP	35	43402	Ribeirão Preto	612.340
SP	35	43600	Rifaina	3.445
SP	35	43709	Rincão	10.421
SP	35	43808	Rinópolis	9.911
SP	35	43907	Rio Claro	187.638
SP	35	44004	Rio das Pedras	29.963
SP	35	44103	Rio Grande da Serra	44.503
SP	35	44202	Riolândia	10.730
SP	35	43501	Riversul	6.085
SP	35	44251	Rosana	19.343
SP	35	44301	Roseira	9.678
SP	35	44400	Rubiácea	2.760
SP	35	44509	Rubinéia	2.881
SP	35	44608	Sabino	5.238
SP	35	44707	Sagres	2.392
SP	35	44806	Sales	5.520
SP	35	44905	Sales Oliveira	10.664
SP	35	45001	Salesópolis	15.734
SP	35	45100	Salmourão	4.851
SP	35	45159	Saltinho	7.156
SP	35	45209	Salto	106.465
SP	35	45308	Salto de Pirapora	40.521
SP	35	45407	Salto Grande	8.814
SP	35	45506	Sandovalina	3.746
SP	35	45605	Santa Adélia	14.401
SP	35	45704	Santa Albertina	5.734
SP	35	45803	Santa Bárbara d'Oeste	180.772
SP	35	46009	Santa Branca	13.821
SP	35	46108	Santa Clara d'Oeste	2.082
SP	35	46207	Santa Cruz da Conceição	4.039
SP	35	46256	Santa Cruz da Esperança	1.966
SP	35	46306	Santa Cruz das Palmeiras	30.268
SP	35	46405	Santa Cruz do Rio Pardo	44.152
SP	35	46504	Santa Ernestina	5.555
SP	35	46603	Santa Fé do Sul	29.449
SP	35	46702	Santa Gertrudes	22.074
SP	35	46801	Santa Isabel	50.969
SP	35	46900	Santa Lúcia	8.279
SP	35	47007	Santa Maria da Serra	5.470
SP	35	47106	Santa Mercedes	2.834
SP	35	47502	Santa Rita do Passa Quatro	26.505
SP	35	47403	Santa Rita d'Oeste	2.532
SP	35	47601	Santa Rosa de Viterbo	24.049
SP	35	47650	Santa Salete	1.453
SP	35	47205	Santana da Ponte Pensa	1.622
SP	35	47304	Santana de Parnaíba	111.422
SP	35	47700	Santo Anastácio	20.454

SP	35	47809	Santo André	678.486
SP	35	47908	Santo Antônio da Alegria	6.346
SP	35	48005	Santo Antônio de Posse	20.844
SP	35	48054	Santo Antônio do Aracanguá	7.680
SP	35	48104	Santo Antônio do Jardim	5.927
SP	35	48203	Santo Antônio do Pinhal	6.499
SP	35	48302	Santo Expedito	2.825
SP	35	48401	Santópolis do Aguapeí	4.313
SP	35	48500	Santos	419.509
SP	35	48609	São Bento do Sapucaí	10.477
SP	35	48708	São Bernardo do Campo	770.253
SP	35	48807	São Caetano do Sul	149.962
SP	35	48906	São Carlos	224.173
SP	35	49003	São Francisco	2.788
SP	35	49102	São João da Boa Vista	84.119
SP	35	49201	São João das Duas Pontes	2.559
SP	35	49250	São João de Iracema	1.789
SP	35	49300	São João do Pau d'Alho	2.098
SP	35	49409	São Joaquim da Barra	46.891
SP	35	49508	São José da Bela Vista	8.432
SP	35	49607	São José do Barreiro	4.072
SP	35	49706	São José do Rio Pardo	52.040
SP	35	49805	São José do Rio Preto	412.076
SP	35	49904	São José dos Campos	636.876
SP	35	49953	São Lourenço da Serra	14.110
SP	35	50001	São Luís do Paraitinga	10.395
SP	35	50100	São Manuel	38.480
SP	35	50209	São Miguel Arcanjo	31.501
SP	35	50308	São Paulo	11.316.119
SP	35	50407	São Pedro	31.951
SP	35	50506	São Pedro do Turvo	7.222
SP	35	50605	São Roque	79.757
SP	35	50704	São Sebastião	75.163
SP	35	50803	São Sebastião da Grama	12.072
SP	35	50902	São Simão	14.398
SP	35	51009	São Vicente	334.663
SP	35	51108	Sarapuí	9.121
SP	35	51207	Sarutaiá	3.614
SP	35	51306	Sebastianópolis do Sul	3.069
SP	35	51405	Serra Azul	11.549
SP	35	51603	Serra Negra	26.582
SP	35	51504	Serrana	39.360
SP	35	51702	Sertãozinho	111.257
SP	35	51801	Sete Barras	12.951
SP	35	51900	Severínia	15.647
SP	35	52007	Silveiras	5.824
SP	35	52106	Socorro	36.992
SP	35	52205	Sorocaba	593.776
SP	35	52304	Sud Mennucci	7.441
SP	35	52403	Sumaré	242.960
SP	35	52551	Suzanápolis	3.429
SP	35	52502	Suzano	265.074
SP	35	52601	Tabapuã	11.430
SP	35	52700	Tabatinga	14.817
SP	35	52809	Taboão da Serra	248.127
SP	35	52908	Taciba	5.752
SP	35	53005	Taguaí	11.086

SP	35	53104	Taiaçu	5.916
SP	35	53203	Taiúva	5.443
SP	35	53302	Tambaú	22.418
SP	35	53401	Tanabi	24.168
SP	35	53500	Tapiraí	7.970
SP	35	53609	Tapiratiba	12.722
SP	35	53658	Taquaral	2.727
SP	35	53708	Taquaritinga	54.136
SP	35	53807	Taquarituba	22.315
SP	35	53856	Taquarivaí	5.204
SP	35	53906	Tarabai	6.671
SP	35	53955	Tarumã	13.050
SP	35	54003	Tatuí	108.393
SP	35	54102	Taubaté	281.336
SP	35	54201	Tejupá	4.769
SP	35	54300	Teodoro Sampaio	21.493
SP	35	54409	Terra Roxa	8.563
SP	35	54508	Tietê	37.229
SP	35	54607	Timburi	2.640
SP	35	54656	Torre de Pedra	2.263
SP	35	54706	Torrinha	9.368
SP	35	54755	Trabiju	1.557
SP	35	54805	Tremembé	41.457
SP	35	54904	Três Fronteiras	5.448
SP	35	54953	Tuiuti	6.005
SP	35	55000	Tupã	63.487
SP	35	55109	Tupi Paulista	14.345
SP	35	55208	Turiúba	1.933
SP	35	55307	Turmalina	1.949
SP	35	55356	Ubarana	5.372
SP	35	55406	Ubatuba	79.718
SP	35	55505	Ubirajara	4.448
SP	35	55604	Uchoa	9.505
SP	35	55703	União Paulista	1.618
SP	35	55802	Urânia	8.837
SP	35	55901	Uru	1.240
SP	35	56008	Urupês	12.782
SP	35	56107	Valentim Gentil	11.223
SP	35	56206	Valinhos	108.622
SP	35	56305	Valparaíso	22.884
SP	35	56354	Vargem	8.942
SP	35	56404	Vargem Grande do Sul	39.494
SP	35	56453	Vargem Grande Paulista	43.789
SP	35	56503	Várzea Paulista	108.186
SP	35	56602	Vera Cruz	10.745
SP	35	56701	Vinhedo	64.870
SP	35	56800	Viradouro	17.400
SP	35	56909	Vista Alegre do Alto	7.050
SP	35	56958	Vitória Brasil	1.742
SP	35	57006	Votorantim	109.798
SP	35	57105	Votuporanga	85.387
SP	35	57154	Zacarias	2.365
PR	41	00103	Abatiá	7.727
PR	41	00202	Adrianópolis	6.328
PR	41	00301	Agudos do Sul	8.351
PR	41	00400	Almirante Tamandaré	104.350
PR	41	00459	Altamira do Paraná	4.100

PR	41	28625	Alto Paraíso	3.162
PR	41	00608	Alto Paraná	13.736
PR	41	00707	Alto Piquiri	10.135
PR	41	00509	Altônia	20.615
PR	41	00806	Alvorada do Sul	10.363
PR	41	00905	Amaporã	5.504
PR	41	01002	Ampére	17.438
PR	41	01051	Anahy	2.864
PR	41	01101	Andirá	20.530
PR	41	01150	Ângulo	2.861
PR	41	01200	Antonina	18.870
PR	41	01309	Antônio Olinto	7.347
PR	41	01408	Apucarana	121.924
PR	41	01507	Arapongas	105.588
PR	41	01606	Arapoti	26.007
PR	41	01655	Arapuã	3.515
PR	41	01705	Araruna	13.445
PR	41	01804	Araucária	121.032
PR	41	01853	Ariranha do Ivaí	2.420
PR	41	01903	Assaí	16.225
PR	41	02000	Assis Chateaubriand	33.003
PR	41	02109	Astorga	24.780
PR	41	02208	Atalaia	3.906
PR	41	02307	Balsa Nova	11.422
PR	41	02406	Bandeirantes	32.066
PR	41	02505	Barbosa Ferraz	12.545
PR	41	02703	Barra do Jacaré	2.728
PR	41	02604	Barracão	9.766
PR	41	02752	Bela Vista da Caroba	3.903
PR	41	02802	Bela Vista do Paraíso	15.083
PR	41	02901	Bituruna	15.892
PR	41	03008	Boa Esperança	4.523
PR	41	03024	Boa Esperança do Iguaçu	2.738
PR	41	03040	Boa Ventura de São Roque	6.537
PR	41	03057	Boa Vista da Aparecida	7.872
PR	41	03107	Bocaiúva do Sul	11.136
PR	41	03156	Bom Jesus do Sul	3.769
PR	41	03206	Bom Sucesso	6.591
PR	41	03222	Bom Sucesso do Sul	3.286
PR	41	03305	Borrazópolis	7.758
PR	41	03354	Braganey	5.700
PR	41	03370	Brasilândia do Sul	3.157
PR	41	03404	Cafeara	2.712
PR	41	03453	Cafelândia	14.933
PR	41	03479	Cafezal do Sul	4.263
PR	41	03503	Califórnia	8.100
PR	41	03602	Cambará	23.974
PR	41	03701	Cambé	97.390
PR	41	03800	Cambira	7.279
PR	41	03909	Campina da Lagoa	15.270
PR	41	03958	Campina do Simão	4.054
PR	41	04006	Campina Grande do Sul	39.092
PR	41	04055	Campo Bonito	4.352
PR	41	04105	Campo do Tenente	7.186
PR	41	04204	Campo Largo	113.882
PR	41	04253	Campo Magro	25.184
PR	41	04303	Campo Mourão	87.710

PR	41	04402	Cândido de Abreu	16.491
PR	41	04428	Candói	15.045
PR	41	04451	Cantagalo	12.963
PR	41	04501	Capanema	18.549
PR	41	04600	Capitão Leônidas Marques	15.016
PR	41	04659	Carambeí	19.494
PR	41	04709	Carlópolis	13.737
PR	41	04808	Cascavel	289.340
PR	41	04907	Castro	67.353
PR	41	05003	Catanduvas	10.186
PR	41	05102	Centenário do Sul	11.142
PR	41	05201	Cerro Azul	16.983
PR	41	05300	Céu Azul	11.078
PR	41	05409	Chopinzinho	19.613
PR	41	05508	Cianorte	70.922
PR	41	05607	Cidade Gaúcha	11.180
PR	41	05706	Clevelândia	17.156
PR	41	05805	Colombo	215.242
PR	41	05904	Colorado	22.452
PR	41	06001	Congonhinhas	8.312
PR	41	06100	Conselheiro Mairinck	3.650
PR	41	06209	Contenda	16.095
PR	41	06308	Corbélia	16.352
PR	41	06407	Cornélio Procópio	46.934
PR	41	06456	Coronel Domingos Soares	7.256
PR	41	06506	Coronel Vivida	21.630
PR	41	06555	Corumbataí do Sul	3.930
PR	41	06803	Cruz Machado	18.069
PR	41	06571	Cruzeiro do Iguaçu	4.270
PR	41	06605	Cruzeiro do Oeste	20.431
PR	41	06704	Cruzeiro do Sul	4.548
PR	41	06852	Cruzmaltina	3.140
PR	41	06902	Curitiba	1.764.541
PR	41	07009	Curiúva	14.002
PR	41	07108	Diamante do Norte	5.472
PR	41	07124	Diamante do Sul	3.499
PR	41	07157	Diamante D'Oeste	5.039
PR	41	07207	Dois Vizinhos	36.501
PR	41	07256	Douradina	7.544
PR	41	07306	Doutor Camargo	5.832
PR	41	28633	Doutor Ulysses	5.706
PR	41	07405	Enéas Marques	6.082
PR	41	07504	Engenheiro Beltrão	13.893
PR	41	07538	Entre Rios do Oeste	3.972
PR	41	07520	Esperança Nova	1.945
PR	41	07546	Espigão Alto do Iguaçu	4.623
PR	41	07553	Farol	3.435
PR	41	07603	Faxinal	16.369
PR	41	07652	Fazenda Rio Grande	83.118
PR	41	07702	Fênix	4.792
PR	41	07736	Fernandes Pinheiro	5.899
PR	41	07751	Figueira	8.236
PR	41	07850	Flor da Serra do Sul	4.711
PR	41	07801	Floraí	5.032
PR	41	07900	Floresta	5.994
PR	41	08007	Florestópolis	11.148
PR	41	08106	Flórida	2.552

PR	41	08205	Formosa do Oeste	7.448
PR	41	08304	Foz do Iguaçu	255.900
PR	41	08452	Foz do Jordão	5.347
PR	41	08320	Francisco Alves	6.377
PR	41	08403	Francisco Beltrão	79.850
PR	41	08502	General Carneiro	13.652
PR	41	08551	Godoy Moreira	3.299
PR	41	08601	Goioerê	28.962
PR	41	08650	Goioxim	7.459
PR	41	08700	Grandes Rios	6.530
PR	41	08809	Guaíra	30.861
PR	41	08908	Guairaçá	6.220
PR	41	08957	Guamiranga	7.959
PR	41	09005	Guapirama	3.878
PR	41	09104	Guaporema	2.225
PR	41	09203	Guaraci	5.159
PR	41	09302	Guaraniaçu	14.381
PR	41	09401	Guarapuava	168.349
PR	41	09500	Guaraqueçaba	7.839
PR	41	09609	Guaratuba	32.467
PR	41	09658	Honório Serpa	5.883
PR	41	09708	Ibaiti	28.928
PR	41	09757	Ibema	6.081
PR	41	09807	Ibiporã	48.662
PR	41	09906	Icaraíma	8.747
PR	41	10003	Iguaraçu	4.012
PR	41	10052	Iguatu	2.233
PR	41	10078	Imbaú	11.413
PR	41	10102	Imbituva	28.759
PR	41	10201	Inácio Martins	10.942
PR	41	10300	Inajá	2.994
PR	41	10409	Indianópolis	4.306
PR	41	10508	Ipiranga	14.215
PR	41	10607	Iporã	14.869
PR	41	10656	Iracema do Oeste	2.550
PR	41	10706	Irati	56.503
PR	41	10805	Iretama	10.568
PR	41	10904	Itaguajé	4.553
PR	41	10953	Itaipulândia	9.195
PR	41	11001	Itambaracá	6.734
PR	41	11100	Itambé	5.981
PR	41	11209	Itapejara d'Oeste	10.637
PR	41	11258	Itaperuçu	24.236
PR	41	11308	Itaúna do Sul	3.517
PR	41	11407	Ivaí	12.886
PR	41	11506	Ivaiporã	31.782
PR	41	11555	Ivaté	7.560
PR	41	11605	Ivatuba	3.027
PR	41	11704	Jaboti	4.926
PR	41	11803	Jacarezinho	39.083
PR	41	11902	Jaguapitã	12.325
PR	41	12009	Jaguariaíva	32.747
PR	41	12108	Jandaia do Sul	20.315
PR	41	12207	Janiópolis	6.413
PR	41	12306	Japira	4.904
PR	41	12405	Japurá	8.610
PR	41	12504	Jardim Alegre	12.221

PR	41	12603	Jardim Olinda	1.401
PR	41	12702	Jataizinho	11.918
PR	41	12751	Jesuítas	8.938
PR	41	12801	Joaquim Távora	10.819
PR	41	12900	Jundiaí do Sul	3.416
PR	41	12959	Juranda	7.604
PR	41	13007	Jussara	6.634
PR	41	13106	Kaloré	4.465
PR	41	13205	Lapa	45.137
PR	41	13254	Laranjal	6.308
PR	41	13304	Laranjeiras do Sul	30.835
PR	41	13403	Leópolis	4.123
PR	41	13429	Lidianópolis	3.911
PR	41	13452	Lindoeste	5.295
PR	41	13502	Loanda	21.328
PR	41	13601	Lobato	4.427
PR	41	13700	Londrina	511.279
PR	41	13734	Luiziana	7.298
PR	41	13759	Lunardelli	5.122
PR	41	13809	Lupionópolis	4.613
PR	41	13908	Mallet	13.002
PR	41	14005	Mamborê	13.870
PR	41	14104	Mandaguaçu	20.008
PR	41	14203	Mandaguari	32.755
PR	41	14302	Mandirituba	22.580
PR	41	14351	Manfrinópolis	3.076
PR	41	14401	Mangueirinha	16.994
PR	41	14500	Manoel Ribas	13.177
PR	41	14609	Marechal Cândido Rondon	47.266
PR	41	14708	Maria Helena	5.924
PR	41	14807	Marialva	32.209
PR	41	14906	Marilândia do Sul	8.848
PR	41	15002	Marilena	6.866
PR	41	15101	Mariluz	10.219
PR	41	15200	Maringá	362.329
PR	41	15309	Mariópolis	6.288
PR	41	15358	Maripá	5.669
PR	41	15408	Marmeleiro	13.919
PR	41	15457	Marquinho	4.929
PR	41	15507	Marumbi	4.603
PR	41	15606	Matelândia	16.212
PR	41	15705	Matinhos	29.831
PR	41	15739	Mato Rico	3.766
PR	41	15754	Mauá da Serra	8.715
PR	41	15804	Medianeira	42.124
PR	41	15853	Mercedes	5.080
PR	41	15903	Mirador	2.314
PR	41	16000	Miraselva	1.855
PR	41	16059	Missal	10.478
PR	41	16109	Moreira Sales	12.546
PR	41	16208	Morretes	15.753
PR	41	16307	Munhoz de Melo	3.693
PR	41	16406	Nossa Senhora das Graças	3.929
PR	41	16505	Nova Aliança do Ivaí	1.439
PR	41	16604	Nova América da Colina	3.470
PR	41	16703	Nova Aurora	11.730
PR	41	16802	Nova Cantu	7.234

PR	41	16901	Nova Esperança	26.684
PR	41	16950	Nova Esperança do Sudoeste	5.086
PR	41	17008	Nova Fátima	8.135
PR	41	17057	Nova Laranjeiras	11.206
PR	41	17107	Nova Londrina	13.060
PR	41	17206	Nova Olímpia	5.521
PR	41	17255	Nova Prata do Iguaçu	10.376
PR	41	17214	Nova Santa Bárbara	3.931
PR	41	17222	Nova Santa Rosa	7.665
PR	41	17271	Nova Tebas	7.239
PR	41	17297	Novo Itacolomi	2.825
PR	41	17305	Ortigueira	23.240
PR	41	17404	Ourizona	3.379
PR	41	17453	Ouro Verde do Oeste	5.709
PR	41	17503	Paiçandu	36.333
PR	41	17602	Palmas	43.508
PR	41	17701	Palmeira	32.232
PR	41	17800	Palmital	14.699
PR	41	17909	Palotina	28.907
PR	41	18006	Paraíso do Norte	11.929
PR	41	18105	Paranacity	10.338
PR	41	18204	Paranaguá	141.477
PR	41	18303	Paranapoema	2.822
PR	41	18402	Paranavaí	82.039
PR	41	18451	Pato Bragado	4.882
PR	41	18501	Pato Branco	73.148
PR	41	18600	Paula Freitas	5.463
PR	41	18709	Paulo Frontin	6.940
PR	41	18808	Peabiru	13.635
PR	41	18857	Perobal	5.681
PR	41	18907	Pérola	10.280
PR	41	19004	Pérola d'Oeste	6.716
PR	41	19103	Piên	11.347
PR	41	19152	Pinhais	118.334
PR	41	19251	Pinhal de São Bento	2.630
PR	41	19202	Pinhalão	6.215
PR	41	19301	Pinhão	30.347
PR	41	19400	Piraí do Sul	23.561
PR	41	19509	Piraquara	94.518
PR	41	19608	Pitanga	32.391
PR	41	19657	Pitangueiras	2.845
PR	41	19707	Planaltina do Paraná	4.103
PR	41	19806	Planalto	13.619
PR	41	19905	Ponta Grossa	314.518
PR	41	19954	Pontal do Paraná	21.427
PR	41	20002	Porecatu	14.060
PR	41	20101	Porto Amazonas	4.536
PR	41	20150	Porto Barreiro	3.622
PR	41	20200	Porto Rico	2.529
PR	41	20309	Porto Vitória	4.018
PR	41	20333	Prado Ferreira	3.456
PR	41	20358	Pranchita	5.580
PR	41	20408	Presidente Castelo Branco	4.821
PR	41	20507	Primeiro de Maio	10.840
PR	41	20606	Prudentópolis	48.933
PR	41	20655	Quarto Centenário	4.820
PR	41	20705	Quatiguá	7.069

PR	41	20804	Quatro Barras	20.135
PR	41	20853	Quatro Pontes	3.816
PR	41	20903	Quedas do Iguaçu	30.854
PR	41	21000	Querência do Norte	11.752
PR	41	21109	Quinta do Sol	5.037
PR	41	21208	Quitandinha	17.229
PR	41	21257	Ramilândia	4.155
PR	41	21307	Rancho Alegre	3.937
PR	41	21356	Rancho Alegre D'Oeste	2.827
PR	41	21406	Realeza	16.363
PR	41	21505	Rebouças	14.216
PR	41	21604	Renascença	6.801
PR	41	21703	Reserva	25.264
PR	41	21752	Reserva do Iguaçu	7.356
PR	41	21802	Ribeirão Claro	10.661
PR	41	21901	Ribeirão do Pinhal	13.462
PR	41	22008	Rio Azul	14.176
PR	41	22107	Rio Bom	3.318
PR	41	22156	Rio Bonito do Iguaçu	13.652
PR	41	22172	Rio Branco do Ivaí	3.909
PR	41	22206	Rio Branco do Sul	30.751
PR	41	22305	Rio Negro	31.471
PR	41	22404	Rolândia	58.511
PR	41	22503	Roncador	11.377
PR	41	22602	Rondon	9.025
PR	41	22651	Rosário do Ivaí	5.512
PR	41	22701	Sabáudia	6.149
PR	41	22800	Salgado Filho	4.327
PR	41	22909	Salto do Itararé	5.150
PR	41	23006	Salto do Lontra	13.761
PR	41	23105	Santa Amélia	3.757
PR	41	23204	Santa Cecília do Pavão	3.614
PR	41	23303	Santa Cruz de Monte Castelo	8.055
PR	41	23402	Santa Fé	10.552
PR	41	23501	Santa Helena	23.638
PR	41	23600	Santa Inês	1.797
PR	41	23709	Santa Isabel do Ivaí	8.730
PR	41	23808	Santa Izabel do Oeste	13.242
PR	41	23824	Santa Lúcia	3.910
PR	41	23857	Santa Maria do Oeste	11.336
PR	41	23907	Santa Mariana	12.356
PR	41	23956	Santa Mônica	3.601
PR	41	24020	Santa Tereza do Oeste	10.300
PR	41	24053	Santa Terezinha de Itaipu	21.031
PR	41	24004	Santana do Itararé	5.220
PR	41	24103	Santo Antônio da Platina	42.920
PR	41	24202	Santo Antônio do Caiuá	2.716
PR	41	24301	Santo Antônio do Paraíso	2.379
PR	41	24400	Santo Antônio do Sudoeste	18.972
PR	41	24509	Santo Inácio	5.276
PR	41	24608	São Carlos do Ivaí	6.389
PR	41	24707	São Jerônimo da Serra	11.306
PR	41	24806	São João	10.553
PR	41	24905	São João do Caiuá	5.898
PR	41	25001	São João do Ivaí	11.397
PR	41	25100	São João do Triunfo	13.803
PR	41	25308	São Jorge do Ivaí	5.512

PR	41	25357	São Jorge do Patrocínio	5.998
PR	41	25209	São Jorge d'Oeste	9.068
PR	41	25407	São José da Boa Vista	6.476
PR	41	25456	São José das Palmeiras	3.810
PR	41	25506	São José dos Pinhais	268.808
PR	41	25555	São Manoel do Paraná	2.100
PR	41	25605	São Mateus do Sul	41.617
PR	41	25704	São Miguel do Iguaçu	25.872
PR	41	25753	São Pedro do Iguaçu	6.431
PR	41	25803	São Pedro do Ivaí	10.221
PR	41	25902	São Pedro do Paraná	2.473
PR	41	26009	São Sebastião da Amoreira	8.632
PR	41	26108	São Tomé	5.373
PR	41	26207	Sapopema	6.726
PR	41	26256	Sarandi	83.724
PR	41	26272	Saudade do Iguaçu	5.061
PR	41	26306	Sengés	18.463
PR	41	26355	Serranópolis do Iguaçu	4.555
PR	41	26405	Sertaneja	5.763
PR	41	26504	Sertanópolis	15.676
PR	41	26603	Siqueira Campos	18.643
PR	41	26652	Sulina	3.354
PR	41	26678	Tamarana	12.458
PR	41	26702	Tamboara	4.696
PR	41	26801	Tapejara	14.712
PR	41	26900	Tapira	5.802
PR	41	27007	Teixeira Soares	10.444
PR	41	27106	Telêmaco Borba	70.535
PR	41	27205	Terra Boa	15.864
PR	41	27304	Terra Rica	15.331
PR	41	27403	Terra Roxa	16.795
PR	41	27502	Tibagi	19.414
PR	41	27601	Tijucas do Sul	14.712
PR	41	27700	Toledo	120.934
PR	41	27809	Tomazina	8.704
PR	41	27858	Três Barras do Paraná	11.825
PR	41	27882	Tunas do Paraná	6.460
PR	41	27908	Tuneiras do Oeste	8.671
PR	41	27957	Tupãssi	7.996
PR	41	27965	Turvo	13.717
PR	41	28005	Ubiratã	21.479
PR	41	28104	Umuarama	101.443
PR	41	28203	União da Vitória	53.059
PR	41	28302	Uniflor	2.474
PR	41	28401	Uraí	11.441
PR	41	28534	Ventania	10.106
PR	41	28559	Vera Cruz do Oeste	8.921
PR	41	28609	Verê	7.814
PR	41	28658	Virmond	3.951
PR	41	28708	Vitorino	6.531
PR	41	28500	Wenceslau Braz	19.278
PR	41	28807	Xambrê	5.975
SC	42	00051	Abdon Batista	2.644
SC	42	00101	Abelardo Luz	17.151
SC	42	00200	Agrolândia	9.440
SC	42	00309	Agronômica	4.954
SC	42	00408	Água Doce	6.971

SC	42	00507	Águas de Chapecó	6.136
SC	42	00556	Águas Frias	2.417
SC	42	00606	Águas Mornas	5.618
SC	42	00705	Alfredo Wagner	9.453
SC	42	00754	Alto Bela Vista	1.998
SC	42	00804	Anchieta	6.323
SC	42	00903	Angelina	5.210
SC	42	01000	Anita Garibaldi	8.497
SC	42	01109	Anitápolis	3.213
SC	42	01208	Antônio Carlos	7.537
SC	42	01257	Apiúna	9.683
SC	42	01273	Arabutã	4.196
SC	42	01307	Araquari	25.860
SC	42	01406	Araranguá	61.817
SC	42	01505	Armazém	7.821
SC	42	01604	Arroio Trinta	3.503
SC	42	01653	Arvoredo	2.257
SC	42	01703	Ascurra	7.449
SC	42	01802	Atalanta	3.291
SC	42	01901	Aurora	5.555
SC	42	01950	Balneário Arroio do Silva	9.858
SC	42	02057	Balneário Barra do Sul	8.614
SC	42	02008	Balneário Camboriú	110.748
SC	42	02073	Balneário Gaivota	8.448
SC	42	12809	Balneário Piçarras	17.552
SC	42	02081	Bandeirante	2.886
SC	42	02099	Barra Bonita	1.860
SC	42	02107	Barra Velha	22.913
SC	42	02131	Bela Vista do Toldo	6.026
SC	42	02156	Belmonte	2.639
SC	42	02206	Benedito Novo	10.434
SC	42	02305	Biguaçu	58.984
SC	42	02404	Blumenau	312.635
SC	42	02438	Bocaina do Sul	3.303
SC	42	02503	Bom Jardim da Serra	4.420
SC	42	02537	Bom Jesus	2.563
SC	42	02578	Bom Jesus do Oeste	2.131
SC	42	02602	Bom Retiro	9.017
SC	42	02453	Bombinhas	14.722
SC	42	02701	Botuverá	4.527
SC	42	02800	Braço do Norte	29.351
SC	42	02859	Braço do Trombudo	3.478
SC	42	02875	Brunópolis	2.814
SC	42	02909	Brusque	107.764
SC	42	03006	Caçador	71.334
SC	42	03105	Caibi	6.209
SC	42	03154	Calmon	3.381
SC	42	03204	Camboriú	63.967
SC	42	03303	Campo Alegre	11.757
SC	42	03402	Campo Belo do Sul	7.440
SC	42	03501	Campo Erê	9.295
SC	42	03600	Campos Novos	33.073
SC	42	03709	Canelinha	10.726
SC	42	03808	Canoinhas	52.853
SC	42	03253	Capão Alto	2.733
SC	42	03907	Capinzal	20.919
SC	42	03956	Capivari de Baixo	21.913

SC	42	04004	Catanduvas	9.653
SC	42	04103	Caxambu do Sul	4.346
SC	42	04152	Celso Ramos	2.766
SC	42	04178	Cerro Negro	3.542
SC	42	04194	Chapadão do Lageado	2.778
SC	42	04202	Chapecó	186.337
SC	42	04251	Cocal do Sul	15.269
SC	42	04301	Concórdia	69.048
SC	42	04350	Cordilheira Alta	3.819
SC	42	04400	Coronel Freitas	10.189
SC	42	04459	Coronel Martins	2.464
SC	42	04558	Correia Pinto	14.613
SC	42	04509	Corupá	14.006
SC	42	04608	Criciúma	193.989
SC	42	04707	Cunha Porã	10.643
SC	42	04756	Cunhataí	1.887
SC	42	04806	Curitibanos	37.878
SC	42	04905	Descanso	8.597
SC	42	05001	Dionísio Cerqueira	14.855
SC	42	05100	Dona Emma	3.753
SC	42	05159	Doutor Pedrinho	3.645
SC	42	05175	Entre Rios	3.031
SC	42	05191	Ermo	2.050
SC	42	05209	Erval Velho	4.359
SC	42	05308	Faxinal dos Guedes	10.653
SC	42	05357	Flor do Sertão	1.587
SC	42	05407	Florianópolis	427.298
SC	42	05431	Formosa do Sul	2.592
SC	42	05456	Forquilhinha	22.871
SC	42	05506	Fraiburgo	34.677
SC	42	05555	Frei Rogério	2.436
SC	42	05605	Galvão	3.414
SC	42	05704	Garopaba	18.520
SC	42	05803	Garuva	15.021
SC	42	05902	Gaspar	58.869
SC	42	06009	Governador Celso Ramos	13.107
SC	42	06108	Grão Pará	6.246
SC	42	06207	Gravatal	10.698
SC	42	06306	Guabiruba	18.849
SC	42	06405	Guaraciaba	10.457
SC	42	06504	Guaramirim	35.918
SC	42	06603	Guarujá do Sul	4.925
SC	42	06652	Guatambú	4.678
SC	42	06702	Herval d'Oeste	21.331
SC	42	06751	Ibiam	1.945
SC	42	06801	Ibicaré	3.357
SC	42	06900	Ibirama	17.448
SC	42	07007	Içara	59.616
SC	42	07106	Ilhota	12.492
SC	42	07205	Imaruí	11.540
SC	42	07304	Imbituba	40.514
SC	42	07403	Imbuia	5.743
SC	42	07502	Indaial	55.980
SC	42	07577	Iomerê	2.754
SC	42	07601	Ipira	4.725
SC	42	07650	Iporã do Oeste	8.450
SC	42	07684	Ipuaçu	6.850

SC	42	07700	Ipumirim	7.245
SC	42	07759	Iraceminha	4.227
SC	42	07809	Irani	9.595
SC	42	07858	Irati	2.081
SC	42	07908	Irineópolis	10.503
SC	42	08005	Itá	6.401
SC	42	08104	Itaiópolis	20.395
SC	42	08203	Itajaí	186.127
SC	42	08302	Itapema	47.327
SC	42	08401	Itapiranga	15.518
SC	42	08450	Itapoá	15.218
SC	42	08500	Ituporanga	22.462
SC	42	08609	Jaborá	4.030
SC	42	08708	Jacinto Machado	10.585
SC	42	08807	Jaguaruna	17.496
SC	42	08906	Jaraguá do Sul	145.782
SC	42	08955	Jardinópolis	1.749
SC	42	09003	Joaçaba	27.247
SC	42	09102	Joinville	520.905
SC	42	09151	José Boiteux	4.731
SC	42	09177	Jupiá	2.143
SC	42	09201	Lacerdópolis	2.201
SC	42	09300	Lages	156.665
SC	42	09409	Laguna	51.869
SC	42	09458	Lajeado Grande	1.484
SC	42	09508	Laurentino	6.077
SC	42	09607	Lauro Muller	14.426
SC	42	09706	Lebon Régis	11.850
SC	42	09805	Leoberto Leal	3.337
SC	42	09854	Lindóia do Sul	4.632
SC	42	09904	Lontras	10.387
SC	42	10001	Luiz Alves	10.628
SC	42	10035	Luzerna	5.603
SC	42	10050	Macieira	1.821
SC	42	10100	Mafra	53.141
SC	42	10209	Major Gercino	3.290
SC	42	10308	Major Vieira	7.523
SC	42	10407	Maracajá	6.471
SC	42	10506	Maravilha	22.376
SC	42	10555	Marema	2.169
SC	42	10605	Massaranduba	14.837
SC	42	10704	Matos Costa	2.811
SC	42	10803	Meleiro	6.994
SC	42	10852	Mirim Doce	2.495
SC	42	10902	Modelo	4.054
SC	42	11009	Mondaí	10.347
SC	42	11058	Monte Carlo	9.347
SC	42	11108	Monte Castelo	8.346
SC	42	11207	Morro da Fumaça	16.247
SC	42	11256	Morro Grande	2.888
SC	42	11306	Navegantes	62.187
SC	42	11405	Nova Erechim	4.332
SC	42	11454	Nova Itaberaba	4.268
SC	42	11504	Nova Trento	12.370
SC	42	11603	Nova Veneza	13.448
SC	42	11652	Novo Horizonte	2.724
SC	42	11702	Orleans	21.498

SC	42	11751	Otacílio Costa	16.517
SC	42	11801	Ouro	7.360
SC	42	11850	Ouro Verde	2.265
SC	42	11876	Paial	1.741
SC	42	11892	Painel	2.352
SC	42	11900	Palhoça	139.990
SC	42	12007	Palma Sola	7.732
SC	42	12056	Palmeira	2.392
SC	42	12106	Palmitos	16.019
SC	42	12205	Papanduva	18.013
SC	42	12239	Paraíso	4.026
SC	42	12254	Passo de Torres	6.798
SC	42	12270	Passos Maia	4.400
SC	42	12304	Paulo Lopes	6.751
SC	42	12403	Pedras Grandes	4.093
SC	42	12502	Penha	25.714
SC	42	12601	Peritiba	2.970
SC	42	12700	Petrolândia	6.110
SC	42	12908	Pinhalzinho	16.638
SC	42	13005	Pinheiro Preto	3.180
SC	42	13104	Piratuba	4.708
SC	42	13153	Planalto Alegre	2.670
SC	42	13203	Pomerode	28.192
SC	42	13302	Ponte Alta	4.873
SC	42	13351	Ponte Alta do Norte	3.310
SC	42	13401	Ponte Serrada	11.068
SC	42	13500	Porto Belo	16.496
SC	42	13609	Porto União	33.619
SC	42	13708	Pouso Redondo	15.011
SC	42	13807	Praia Grande	7.266
SC	42	13906	Presidente Castello Branco	1.711
SC	42	14003	Presidente Getúlio	15.084
SC	42	14102	Presidente Nereu	2.283
SC	42	14151	Princesa	2.770
SC	42	14201	Quilombo	10.211
SC	42	14300	Rancho Queimado	2.757
SC	42	14409	Rio das Antas	6.145
SC	42	14508	Rio do Campo	6.167
SC	42	14607	Rio do Oeste	7.118
SC	42	14805	Rio do Sul	61.931
SC	42	14706	Rio dos Cedros	10.388
SC	42	14904	Rio Fortuna	4.456
SC	42	15000	Rio Negrinho	40.011
SC	42	15059	Rio Rufino	2.438
SC	42	15075	Riqueza	4.813
SC	42	15109	Rodeio	10.964
SC	42	15208	Romelândia	5.479
SC	42	15307	Salete	7.386
SC	42	15356	Saltinho	3.943
SC	42	15406	Salto Veloso	4.332
SC	42	15455	Sangão	10.575
SC	42	15505	Santa Cecília	15.831
SC	42	15554	Santa Helena	2.367
SC	42	15604	Santa Rosa de Lima	2.070
SC	42	15653	Santa Rosa do Sul	8.073
SC	42	15679	Santa Terezinha	8.762
SC	42	15687	Santa Terezinha do Progresso	2.857

SC	42	15695	Santiago do Sul	1.448
SC	42	15703	Santo Amaro da Imperatriz	20.082
SC	42	15802	São Bento do Sul	75.520
SC	42	15752	São Bernardino	2.642
SC	42	15901	São Bonifácio	2.992
SC	42	16008	São Carlos	10.363
SC	42	16057	São Cristovão do Sul	5.051
SC	42	16107	São Domingos	9.488
SC	42	16206	São Francisco do Sul	43.305
SC	42	16305	São João Batista	27.135
SC	42	16354	São João do Itaperiú	3.457
SC	42	16255	São João do Oeste	6.055
SC	42	16404	São João do Sul	7.019
SC	42	16503	São Joaquim	24.964
SC	42	16602	São José	212.587
SC	42	16701	São José do Cedro	13.685
SC	42	16800	São José do Cerrito	9.188
SC	42	16909	São Lourenço do Oeste	21.964
SC	42	17006	São Ludgero	11.178
SC	42	17105	São Martinho	3.205
SC	42	17154	São Miguel da Boa Vista	1.896
SC	42	17204	São Miguel do Oeste	36.612
SC	42	17253	São Pedro de Alcântara	4.790
SC	42	17303	Saudades	9.070
SC	42	17402	Schroeder	15.790
SC	42	17501	Seara	16.971
SC	42	17550	Serra Alta	3.282
SC	42	17600	Siderópolis	13.069
SC	42	17709	Sombrio	26.894
SC	42	17758	Sul Brasil	2.740
SC	42	17808	Taió	17.337
SC	42	17907	Tangará	8.668
SC	42	17956	Tigrinhos	1.748
SC	42	18004	Tijucas	31.533
SC	42	18103	Timbé do Sul	5.307
SC	42	18202	Timbó	37.344
SC	42	18251	Timbó Grande	7.219
SC	42	18301	Três Barras	18.207
SC	42	18350	Treviso	3.557
SC	42	18400	Treze de Maio	6.889
SC	42	18509	Treze Tílias	6.457
SC	42	18608	Trombudo Central	6.612
SC	42	18707	Tubarão	97.833
SC	42	18756	Tunápolis	4.622
SC	42	18806	Turvo	11.929
SC	42	18855	União do Oeste	2.874
SC	42	18905	Urubici	10.734
SC	42	18954	Urupema	2.479
SC	42	19002	Urussanga	20.291
SC	42	19101	Vargeão	3.533
SC	42	19150	Vargem	2.776
SC	42	19176	Vargem Bonita	4.765
SC	42	19200	Vidal Ramos	6.287
SC	42	19309	Videira	47.618
SC	42	19358	Vitor Meireles	5.184
SC	42	19408	Witmarsum	3.627
SC	42	19507	Xanxerê	44.643

SC	42	19606	Xavantina	4.122
SC	42	19705	Xaxim	25.933
SC	42	19853	Zortéa	3.019
RS	43	00034	Aceguá	4.430
RS	43	00059	Água Santa	3.717
RS	43	00109	Agudo	16.666
RS	43	00208	Ajuricaba	7.221
RS	43	00307	Alecrim	6.935
RS	43	00406	Alegrete	77.140
RS	43	00455	Alegria	4.220
RS	43	00471	Almirante Tamandaré do Sul	2.054
RS	43	00505	Alpestre	7.856
RS	43	00554	Alto Alegre	1.826
RS	43	00570	Alto Feliz	2.924
RS	43	00604	Alvorada	196.572
RS	43	00638	Amaral Ferrador	6.401
RS	43	00646	Ametista do Sul	7.317
RS	43	00661	André da Rocha	1.224
RS	43	00703	Anta Gorda	6.054
RS	43	00802	Antônio Prado	12.827
RS	43	00851	Arambaré	3.676
RS	43	00877	Araricá	4.928
RS	43	00901	Aratiba	6.523
RS	43	01008	Arroio do Meio	18.924
RS	43	01073	Arroio do Padre	2.743
RS	43	01057	Arroio do Sal	7.930
RS	43	01206	Arroio do Tigre	12.742
RS	43	01107	Arroio dos Ratos	13.627
RS	43	01305	Arroio Grande	18.418
RS	43	01404	Arvorezinha	10.223
RS	43	01503	Augusto Pestana	7.042
RS	43	01552	Áurea	3.648
RS	43	01602	Bagé	116.944
RS	43	01636	Balneário Pinhal	11.118
RS	43	01651	Barão	5.768
RS	43	01701	Barão de Cotegipe	6.525
RS	43	01750	Barão do Triunfo	7.046
RS	43	01859	Barra do Guarita	3.097
RS	43	01875	Barra do Quaraí	4.022
RS	43	01909	Barra do Ribeiro	12.628
RS	43	01925	Barra do Rio Azul	1.972
RS	43	01958	Barra Funda	2.378
RS	43	01800	Barracão	5.339
RS	43	02006	Barros Cassal	11.117
RS	43	02055	Benjamin Constant do Sul	2.275
RS	43	02105	Bento Gonçalves	108.481
RS	43	02154	Boa Vista das Missões	2.109
RS	43	02204	Boa Vista do Buricá	6.574
RS	43	02220	Boa Vista do Cadeado	2.439
RS	43	02238	Boa Vista do Incra	2.436
RS	43	02253	Boa Vista do Sul	2.772
RS	43	02303	Bom Jesus	11.482
RS	43	02352	Bom Princípio	11.966
RS	43	02378	Bom Progresso	2.290
RS	43	02402	Bom Retiro do Sul	11.525
RS	43	02451	Boqueirão do Leão	7.662
RS	43	02501	Bossoroca	6.817

RS	43	02584	Bozano	2.189
RS	43	02600	Braga	3.664
RS	43	02659	Brochier	4.701
RS	43	02709	Butiá	20.413
RS	43	02808	Caçapava do Sul	33.617
RS	43	02907	Cacequi	13.551
RS	43	03004	Cachoeira do Sul	83.517
RS	43	03103	Cachoeirinha	119.101
RS	43	03202	Cacique Doble	4.876
RS	43	03301	Caibaté	4.934
RS	43	03400	Caiçara	5.032
RS	43	03509	Camaquã	62.947
RS	43	03558	Camargo	2.600
RS	43	03608	Cambará do Sul	6.520
RS	43	03673	Campestre da Serra	3.253
RS	43	03707	Campina das Missões	6.049
RS	43	03806	Campinas do Sul	5.491
RS	43	03905	Campo Bom	60.539
RS	43	04002	Campo Novo	5.363
RS	43	04101	Campos Borges	3.472
RS	43	04200	Candelária	30.216
RS	43	04309	Cândido Godói	6.493
RS	43	04358	Candiota	8.826
RS	43	04408	Canela	39.660
RS	43	04507	Canguçu	53.399
RS	43	04606	Canoas	325.189
RS	43	04614	Canudos do Vale	1.796
RS	43	04622	Capão Bonito do Sul	1.742
RS	43	04630	Capão da Canoa	42.926
RS	43	04655	Capão do Cipó	3.147
RS	43	04663	Capão do Leão	24.343
RS	43	04689	Capela de Santana	11.734
RS	43	04697	Capitão	2.642
RS	43	04671	Capivari do Sul	3.951
RS	43	04713	Caraá	7.382
RS	43	04705	Carazinho	59.445
RS	43	04804	Carlos Barbosa	25.551
RS	43	04853	Carlos Gomes	1.584
RS	43	04903	Casca	8.668
RS	43	04952	Caseiros	3.019
RS	43	05009	Catuípe	9.256
RS	43	05108	Caxias do Sul	441.332
RS	43	05116	Centenário	2.953
RS	43	05124	Cerrito	6.362
RS	43	05132	Cerro Branco	4.467
RS	43	05157	Cerro Grande	2.403
RS	43	05173	Cerro Grande do Sul	10.422
RS	43	05207	Cerro Largo	13.337
RS	43	05306	Chapada	9.349
RS	43	05355	Charqueadas	35.732
RS	43	05371	Charrua	3.448
RS	43	05405	Chiapetta	4.011
RS	43	05439	Chuí	5.975
RS	43	05447	Chuvisca	4.978
RS	43	05454	Cidreira	12.959
RS	43	05504	Ciríaco	4.897
RS	43	05587	Colinas	2.417

RS	43	05603	Colorado	3.510
RS	43	05702	Condor	6.557
RS	43	05801	Constantina	9.747
RS	43	05835	Coqueiro Baixo	1.523
RS	43	05850	Coqueiros do Sul	2.439
RS	43	05871	Coronel Barros	2.460
RS	43	05900	Coronel Bicaco	7.696
RS	43	05934	Coronel Pilar	1.713
RS	43	05959	Cotiporã	3.904
RS	43	05975	Coxilha	2.815
RS	43	06007	Crissiumal	14.000
RS	43	06056	Cristal	7.330
RS	43	06072	Cristal do Sul	2.823
RS	43	06106	Cruz Alta	62.474
RS	43	06130	Cruzaltense	2.110
RS	43	06205	Cruzeiro do Sul	12.371
RS	43	06304	David Canabarro	4.679
RS	43	06320	Derrubadas	3.150
RS	43	06353	Dezesseis de Novembro	2.822
RS	43	06379	Dilermando de Aguiar	3.054
RS	43	06403	Dois Irmãos	27.967
RS	43	06429	Dois Irmãos das Missões	2.142
RS	43	06452	Dois Lajeados	3.283
RS	43	06502	Dom Feliciano	14.443
RS	43	06601	Dom Pedrito	38.782
RS	43	06551	Dom Pedro de Alcântara	2.544
RS	43	06700	Dona Francisca	3.363
RS	43	06734	Doutor Maurício Cardoso	5.236
RS	43	06759	Doutor Ricardo	2.023
RS	43	06767	Eldorado do Sul	34.887
RS	43	06809	Encantado	20.663
RS	43	06908	Encruzilhada do Sul	24.604
RS	43	06924	Engenho Velho	1.481
RS	43	06957	Entre Rios do Sul	3.049
RS	43	06932	Entre-Ijuís	8.880
RS	43	06973	Erebango	2.966
RS	43	07005	Erechim	96.757
RS	43	07054	Ernestina	3.089
RS	43	07203	Erval Grande	5.126
RS	43	07302	Erval Seco	7.779
RS	43	07401	Esmeralda	3.172
RS	43	07450	Esperança do Sul	3.235
RS	43	07500	Espumoso	15.241
RS	43	07559	Estação	5.995
RS	43	07609	Estância Velha	43.146
RS	43	07708	Esteio	80.810
RS	43	07807	Estrela	30.867
RS	43	07815	Estrela Velha	3.624
RS	43	07831	Eugênio de Castro	2.759
RS	43	07864	Fagundes Varela	2.588
RS	43	07906	Farroupilha	64.275
RS	43	08003	Faxinal do Soturno	6.660
RS	43	08052	Faxinalzinho	2.540
RS	43	08078	Fazenda Vilanova	3.764
RS	43	08102	Feliz	12.440
RS	43	08201	Flores da Cunha	27.391
RS	43	08250	Floriano Peixoto	1.992

RS	43	08300	Fontoura Xavier	10.662
RS	43	08409	Formigueiro	6.970
RS	43	08433	Forquetinha	2.469
RS	43	08458	Fortaleza dos Valos	4.544
RS	43	08508	Frederico Westphalen	29.003
RS	43	08607	Garibaldi	31.014
RS	43	08656	Garruchos	3.201
RS	43	08706	Gaurama	5.822
RS	43	08805	General Câmara	8.425
RS	43	08854	Gentil	1.670
RS	43	08904	Getúlio Vargas	16.127
RS	43	09001	Giruá	16.947
RS	43	09050	Glorinha	6.984
RS	43	09100	Gramado	32.556
RS	43	09126	Gramado dos Loureiros	2.248
RS	43	09159	Gramado Xavier	3.994
RS	43	09209	Gravataí	257.428
RS	43	09258	Guabiju	1.587
RS	43	09308	Guaíba	95.273
RS	43	09407	Guaporé	23.026
RS	43	09506	Guarani das Missões	8.048
RS	43	09555	Harmonia	4.300
RS	43	07104	Herval	6.746
RS	43	09571	Herveiras	2.954
RS	43	09605	Horizontina	18.398
RS	43	09654	Hulha Negra	6.096
RS	43	09704	Humaitá	4.896
RS	43	09753	Ibarama	4.374
RS	43	09803	Ibiaçá	4.701
RS	43	09902	Ibiraiaras	7.172
RS	43	09951	Ibirapuitã	4.049
RS	43	10009	Ibirubá	19.364
RS	43	10108	Igrejinha	32.036
RS	43	10207	Ijuí	79.160
RS	43	10306	Ilópolis	4.091
RS	43	10330	Imbé	18.087
RS	43	10363	Imigrante	3.026
RS	43	10405	Independência	6.566
RS	43	10413	Inhacorá	2.259
RS	43	10439	Ipê	6.059
RS	43	10462	Ipiranga do Sul	1.936
RS	43	10504	Iraí	7.989
RS	43	10538	Itaara	5.044
RS	43	10553	Itacurubi	3.437
RS	43	10579	Itapuca	2.318
RS	43	10603	Itaqui	38.036
RS	43	10652	Itati	2.565
RS	43	10702	Itatiba do Sul	4.089
RS	43	10751	Ivorá	2.130
RS	43	10801	Ivoti	20.224
RS	43	10850	Jaboticaba	4.065
RS	43	10876	Jacuizinho	2.519
RS	43	10900	Jacutinga	3.620
RS	43	11007	Jaguarão	27.766
RS	43	11106	Jaguari	11.396
RS	43	11122	Jaquirana	4.129
RS	43	11130	Jari	3.562

RS	43	11155	Jóia	8.335
RS	43	11205	Júlio de Castilhos	19.515
RS	43	11239	Lagoa Bonita do Sul	2.678
RS	43	11270	Lagoa dos Três Cantos	1.596
RS	43	11304	Lagoa Vermelha	27.495
RS	43	11254	Lagoão	6.240
RS	43	11403	Lajeado	72.338
RS	43	11429	Lajeado do Bugre	2.489
RS	43	11502	Lavras do Sul	7.646
RS	43	11601	Liberato Salzano	5.720
RS	43	11627	Lindolfo Collor	5.290
RS	43	11643	Linha Nova	1.629
RS	43	11718	Maçambara	4.716
RS	43	11700	Machadinho	5.494
RS	43	11734	Mampituba	2.996
RS	43	11759	Manoel Viana	7.078
RS	43	11775	Maquiné	6.875
RS	43	11791	Maratá	2.537
RS	43	11809	Marau	36.979
RS	43	11908	Marcelino Ramos	5.060
RS	43	11981	Mariana Pimentel	3.771
RS	43	12005	Mariano Moro	2.190
RS	43	12054	Marques de Souza	4.055
RS	43	12104	Mata	5.076
RS	43	12138	Mato Castelhano	2.472
RS	43	12153	Mato Leitão	3.916
RS	43	12179	Mato Queimado	1.782
RS	43	12203	Maximiliano de Almeida	4.855
RS	43	12252	Minas do Leão	7.655
RS	43	12302	Miraguaí	4.842
RS	43	12351	Montauri	1.532
RS	43	12377	Monte Alegre dos Campos	3.107
RS	43	12385	Monte Belo do Sul	2.653
RS	43	12401	Montenegro	59.812
RS	43	12427	Mormaço	2.774
RS	43	12443	Morrinhos do Sul	3.156
RS	43	12450	Morro Redondo	6.245
RS	43	12476	Morro Reuter	5.730
RS	43	12500	Mostardas	12.160
RS	43	12609	Muçum	4.796
RS	43	12617	Muitos Capões	2.998
RS	43	12625	Muliterno	1.817
RS	43	12658	Não-Me-Toque	16.053
RS	43	12674	Nicolau Vergueiro	1.715
RS	43	12708	Nonoai	12.017
RS	43	12757	Nova Alvorada	3.215
RS	43	12807	Nova Araçá	4.060
RS	43	12906	Nova Bassano	8.918
RS	43	12955	Nova Boa Vista	1.940
RS	43	13003	Nova Bréscia	3.191
RS	43	13011	Nova Candelária	2.741
RS	43	13037	Nova Esperança do Sul	4.722
RS	43	13060	Nova Hartz	18.598
RS	43	13086	Nova Pádua	2.455
RS	43	13102	Nova Palma	6.345
RS	43	13201	Nova Petrópolis	19.211
RS	43	13300	Nova Prata	23.175

RS	43	13334	Nova Ramada	2.416
RS	43	13359	Nova Roma do Sul	3.367
RS	43	13375	Nova Santa Rita	23.251
RS	43	13490	Novo Barreiro	3.987
RS	43	13391	Novo Cabrais	3.878
RS	43	13409	Novo Hamburgo	239.151
RS	43	13425	Novo Machado	3.865
RS	43	13441	Novo Tiradentes	2.267
RS	43	13466	Novo Xingu	1.751
RS	43	13508	Osório	41.273
RS	43	13607	Paim Filho	4.198
RS	43	13656	Palmares do Sul	10.978
RS	43	13706	Palmeira das Missões	34.170
RS	43	13805	Palmitinho	6.919
RS	43	13904	Panambi	38.477
RS	43	13953	Pantano Grande	9.812
RS	43	14001	Paraí	6.873
RS	43	14027	Paraíso do Sul	7.346
RS	43	14035	Pareci Novo	3.532
RS	43	14050	Parobé	52.019
RS	43	14068	Passa Sete	5.183
RS	43	14076	Passo do Sobrado	6.046
RS	43	14100	Passo Fundo	186.083
RS	43	14134	Paulo Bento	2.201
RS	43	14159	Paverama	8.068
RS	43	14175	Pedras Altas	2.188
RS	43	14209	Pedro Osório	7.789
RS	43	14308	Pejuçara	3.957
RS	43	14407	Pelotas	328.865
RS	43	14423	Picada Café	5.222
RS	43	14456	Pinhal	2.514
RS	43	14464	Pinhal da Serra	2.110
RS	43	14472	Pinhal Grande	4.452
RS	43	14498	Pinheirinho do Vale	4.522
RS	43	14506	Pinheiro Machado	12.710
RS	43	14555	Pirapó	2.712
RS	43	14605	Piratini	19.874
RS	43	14704	Planalto	10.465
RS	43	14753	Poço das Antas	2.020
RS	43	14779	Pontão	3.854
RS	43	14787	Ponte Preta	1.729
RS	43	14803	Portão	31.401
RS	43	14902	Porto Alegre	1.413.094
RS	43	15008	Porto Lucena	5.338
RS	43	15057	Porto Mauá	2.523
RS	43	15073	Porto Vera Cruz	1.806
RS	43	15107	Porto Xavier	10.510
RS	43	15131	Pouso Novo	1.851
RS	43	15149	Presidente Lucena	2.516
RS	43	15156	Progresso	6.158
RS	43	15172	Protásio Alves	1.992
RS	43	15206	Putinga	4.114
RS	43	15305	Quaraí	22.946
RS	43	15313	Quatro Irmãos	1.777
RS	43	15321	Quevedos	2.712
RS	43	15354	Quinze de Novembro	3.659
RS	43	15404	Redentora	10.328

RS	43	15453	Relvado	2.145
RS	43	15503	Restinga Seca	15.869
RS	43	15552	Rio dos Índios	3.533
RS	43	15602	Rio Grande	198.049
RS	43	15701	Rio Pardo	37.577
RS	43	15750	Riozinho	4.350
RS	43	15800	Roca Sales	10.362
RS	43	15909	Rodeio Bonito	5.743
RS	43	15958	Rolador	2.522
RS	43	16006	Rolante	19.611
RS	43	16105	Ronda Alta	10.235
RS	43	16204	Rondinha	5.473
RS	43	16303	Roque Gonzales	7.158
RS	43	16402	Rosário do Sul	39.604
RS	43	16428	Sagrada Família	2.591
RS	43	16436	Saldanha Marinho	2.844
RS	43	16451	Salto do Jacuí	11.907
RS	43	16477	Salvador das Missões	2.670
RS	43	16501	Salvador do Sul	6.827
RS	43	16600	Sananduva	15.422
RS	43	16709	Santa Bárbara do Sul	8.738
RS	43	16733	Santa Cecília do Sul	1.651
RS	43	16758	Santa Clara do Sul	5.766
RS	43	16808	Santa Cruz do Sul	119.199
RS	43	16972	Santa Margarida do Sul	2.366
RS	43	16907	Santa Maria	262.369
RS	43	16956	Santa Maria do Herval	6.066
RS	43	17202	Santa Rosa	68.862
RS	43	17251	Santa Tereza	1.729
RS	43	17301	Santa Vitória do Palmar	30.813
RS	43	17004	Santana da Boa Vista	8.213
RS	43	17103	Santana do Livramento	81.821
RS	43	17400	Santiago	49.005
RS	43	17509	Santo Ângelo	76.239
RS	43	17608	Santo Antônio da Patrulha	39.889
RS	43	17707	Santo Antônio das Missões	11.097
RS	43	17558	Santo Antônio do Palma	2.134
RS	43	17756	Santo Antônio do Planalto	1.986
RS	43	17806	Santo Augusto	13.933
RS	43	17905	Santo Cristo	14.339
RS	43	17954	Santo Expedito do Sul	2.444
RS	43	18002	São Borja	61.426
RS	43	18051	São Domingos do Sul	2.934
RS	43	18101	São Francisco de Assis	19.135
RS	43	18200	São Francisco de Paula	20.600
RS	43	18309	São Gabriel	60.452
RS	43	18408	São Jerônimo	22.277
RS	43	18424	São João da Urtiga	4.711
RS	43	18432	São João do Polêsine	2.580
RS	43	18440	São Jorge	2.767
RS	43	18457	São José das Missões	2.699
RS	43	18465	São José do Herval	2.179
RS	43	18481	São José do Hortêncio	4.149
RS	43	18499	São José do Inhacorá	2.185
RS	43	18507	São José do Norte	25.635
RS	43	18606	São José do Ouro	6.893
RS	43	18614	São José do Sul	2.108

RS	43	18622	São José dos Ausentes	3.305
RS	43	18705	São Leopoldo	215.664
RS	43	18804	São Lourenço do Sul	43.067
RS	43	18903	São Luiz Gonzaga	34.393
RS	43	19000	São Marcos	20.191
RS	43	19109	São Martinho	5.731
RS	43	19125	São Martinho da Serra	3.198
RS	43	19158	São Miguel das Missões	7.429
RS	43	19208	São Nicolau	5.675
RS	43	19307	São Paulo das Missões	6.301
RS	43	19356	São Pedro da Serra	3.352
RS	43	19364	São Pedro das Missões	1.894
RS	43	19372	São Pedro do Butiá	2.874
RS	43	19406	São Pedro do Sul	16.321
RS	43	19505	São Sebastião do Caí	22.104
RS	43	19604	São Sepé	23.735
RS	43	19703	São Valentim	3.596
RS	43	19711	São Valentim do Sul	2.171
RS	43	19737	São Valério do Sul	2.649
RS	43	19752	São Vendelino	1.965
RS	43	19802	São Vicente do Sul	8.448
RS	43	19901	Sapiranga	75.430
RS	43	20008	Sapucaia do Sul	131.587
RS	43	20107	Sarandi	21.525
RS	43	20206	Seberi	10.863
RS	43	20230	Sede Nova	2.996
RS	43	20263	Segredo	7.069
RS	43	20305	Selbach	4.935
RS	43	20321	Senador Salgado Filho	2.806
RS	43	20354	Sentinela do Sul	5.222
RS	43	20404	Serafina Corrêa	14.511
RS	43	20453	Sério	2.249
RS	43	20503	Sertão	6.205
RS	43	20552	Sertão Santana	5.895
RS	43	20578	Sete de Setembro	2.107
RS	43	20602	Severiano de Almeida	3.819
RS	43	20651	Silveira Martins	2.425
RS	43	20677	Sinimbu	10.058
RS	43	20701	Sobradinho	14.317
RS	43	20800	Soledade	30.069
RS	43	20859	Tabaí	4.175
RS	43	20909	Tapejara	19.640
RS	43	21006	Tapera	10.440
RS	43	21105	Tapes	16.655
RS	43	21204	Taquara	54.783
RS	43	21303	Taquari	26.108
RS	43	21329	Taquaruçu do Sul	2.970
RS	43	21352	Tavares	5.352
RS	43	21402	Tenente Portela	13.672
RS	43	21436	Terra de Areia	9.976
RS	43	21451	Teutônia	27.743
RS	43	21469	Tio Hugo	2.746
RS	43	21477	Tiradentes do Sul	6.382
RS	43	21493	Toropi	2.934
RS	43	21501	Torres	34.946
RS	43	21600	Tramandaí	42.395
RS	43	21626	Travesseiro	2.312

RS	43	21634	Três Arroios	2.833
RS	43	21667	Três Cachoeiras	10.271
RS	43	21709	Três Coroas	24.188
RS	43	21808	Três de Maio	23.695
RS	43	21832	Três Forquilhas	2.890
RS	43	21857	Três Palmeiras	4.363
RS	43	21907	Três Passos	23.912
RS	43	21956	Trindade do Sul	5.777
RS	43	22004	Triunfo	26.072
RS	43	22103	Tucunduva	5.867
RS	43	22152	Tunas	4.402
RS	43	22186	Tupanci do Sul	1.562
RS	43	22202	Tupanciretã	22.384
RS	43	22251	Tupandi	3.999
RS	43	22301	Tuparendi	8.482
RS	43	22327	Turuçu	3.508
RS	43	22343	Ubiretama	2.267
RS	43	22350	União da Serra	1.455
RS	43	22376	Unistalda	2.436
RS	43	22400	Uruguaiana	125.320
RS	43	22509	Vacaria	61.650
RS	43	22533	Vale do Sol	11.117
RS	43	22541	Vale Real	5.178
RS	43	22525	Vale Verde	3.269
RS	43	22558	Vanini	1.992
RS	43	22608	Venâncio Aires	66.308
RS	43	22707	Vera Cruz	24.189
RS	43	22806	Veranópolis	23.067
RS	43	22855	Vespasiano Correa	1.956
RS	43	22905	Viadutos	5.252
RS	43	23002	Viamão	240.302
RS	43	23101	Vicente Dutra	5.221
RS	43	23200	Victor Graeff	3.017
RS	43	23309	Vila Flores	3.217
RS	43	23358	Vila Lângaro	2.143
RS	43	23408	Vila Maria	4.225
RS	43	23457	Vila Nova do Sul	4.218
RS	43	23507	Vista Alegre	2.820
RS	43	23606	Vista Alegre do Prata	1.566
RS	43	23705	Vista Gaúcha	2.762
RS	43	23754	Vitória das Missões	3.448
RS	43	23770	Westfalia	2.807
RS	43	23804	Xangri-lá	12.760
MS	50	00203	Água Clara	14.686
MS	50	00252	Alcinópolis	4.638
MS	50	00609	Amambaí	35.133
MS	50	00708	Anastácio	23.940
MS	50	00807	Anaurilândia	8.535
MS	50	00856	Angélica	9.326
MS	50	00906	Antônio João	8.270
MS	50	01003	Aparecida do Taboado	22.621
MS	50	01102	Aquidauana	45.781
MS	50	01243	Aral Moreira	10.420
MS	50	01508	Bandeirantes	6.624
MS	50	01904	Bataguassu	20.119
MS	50	02001	Batayporã	10.960
MS	50	02100	Bela Vista	23.290

MS	50	02159	Bodoquena	7.956
MS	50	02209	Bonito	19.789
MS	50	02308	Brasilândia	11.817
MS	50	02407	Caarapó	26.156
MS	50	02605	Camapuã	13.617
MS	50	02704	Campo Grande	796.252
MS	50	02803	Caracol	5.460
MS	50	02902	Cassilândia	21.034
MS	50	02951	Chapadão do Sul	20.262
MS	50	03108	Corguinho	4.960
MS	50	03157	Coronel Sapucaia	14.161
MS	50	03207	Corumbá	104.318
MS	50	03256	Costa Rica	20.027
MS	50	03306	Coxim	32.259
MS	50	03454	Deodápolis	12.200
MS	50	03488	Dois Irmãos do Buriti	10.442
MS	50	03504	Douradina	5.413
MS	50	03702	Dourados	198.422
MS	50	03751	Eldorado	11.743
MS	50	03801	Fátima do Sul	19.030
MS	50	03900	Figueirão	2.937
MS	50	04007	Glória de Dourados	9.919
MS	50	04106	Guia Lopes da Laguna	10.309
MS	50	04304	Iguatemi	14.972
MS	50	04403	Inocência	7.654
MS	50	04502	Itaporã	21.159
MS	50	04601	Itaquiraí	18.833
MS	50	04700	Ivinhema	22.395
MS	50	04809	Japorã	7.854
MS	50	04908	Jaraguari	6.415
MS	50	05004	Jardim	24.485
MS	50	05103	Jateí	4.008
MS	50	05152	Juti	5.971
MS	50	05202	Ladário	19.948
MS	50	05251	Laguna Carapã	6.565
MS	50	05400	Maracaju	38.264
MS	50	05608	Miranda	25.794
MS	50	05681	Mundo Novo	17.149
MS	50	05707	Naviraí	47.174
MS	50	05806	Nioaque	14.338
MS	50	06002	Nova Alvorada do Sul	16.930
MS	50	06200	Nova Andradina	46.369
MS	50	06259	Novo Horizonte do Sul	4.827
MS	50	06309	Paranaíba	40.330
MS	50	06358	Paranhos	12.514
MS	50	06408	Pedro Gomes	7.924
MS	50	06606	Ponta Porã	79.174
MS	50	06903	Porto Murtinho	15.530
MS	50	07109	Ribas do Rio Pardo	21.271
MS	50	07208	Rio Brilhante	31.279
MS	50	07307	Rio Negro	5.006
MS	50	07406	Rio Verde de Mato Grosso	18.948
MS	50	07505	Rochedo	4.972
MS	50	07554	Santa Rita do Pardo	7.307
MS	50	07695	São Gabriel do Oeste	22.617
MS	50	07802	Selvíria	6.303
MS	50	07703	Sete Quedas	10.769

MS	50	07901	Sidrolândia	43.564
MS	50	07935	Sonora	15.240
MS	50	07950	Tacuru	10.330
MS	50	07976	Taquarussu	3.520
MS	50	08008	Terenos	17.567
MS	50	08305	Três Lagoas	103.536
MS	50	08404	Vicentina	5.911
MT	51	00102	Acorizal	5.493
MT	51	00201	Água Boa	21.325
MT	51	00250	Alta Floresta	49.332
MT	51	00300	Alto Araguaia	15.969
MT	51	00359	Alto Boa Vista	5.403
MT	51	00409	Alto Garças	10.505
MT	51	00508	Alto Paraguai	10.180
MT	51	00607	Alto Taquari	8.349
MT	51	00805	Apiacás	8.713
MT	51	01001	Araguaiana	3.180
MT	51	01209	Araguainha	1.077
MT	51	01258	Araputanga	15.470
MT	51	01308	Arenápolis	10.218
MT	51	01407	Aripuanã	19.006
MT	51	01605	Barão de Melgaço	7.585
MT	51	01704	Barra do Bugres	32.134
MT	51	01803	Barra do Garças	56.903
MT	51	01852	Bom Jesus do Araguaia	5.437
MT	51	01902	Brasnorte	15.783
MT	51	02504	Cáceres	88.428
MT	51	02603	Campinápolis	14.450
MT	51	02637	Campo Novo do Parecis	28.340
MT	51	02678	Campo Verde	32.692
MT	51	02686	Campos de Júlio	5.327
MT	51	02694	Canabrava do Norte	4.771
MT	51	02702	Canarana	19.011
MT	51	02793	Carlinda	10.890
MT	51	02850	Castanheira	8.265
MT	51	03007	Chapada dos Guimarães	17.980
MT	51	03056	Cláudia	11.122
MT	51	03106	Cocalinho	5.500
MT	51	03205	Colíder	30.975
MT	51	03254	Colniza	27.616
MT	51	03304	Comodoro	18.419
MT	51	03353	Confresa	25.684
MT	51	03361	Conquista D'Oeste	3.447
MT	51	03379	Cotriguaçu	15.455
MT	51	03403	Cuiabá	556.299
MT	51	03437	Curvelândia	4.893
MT	51	03452	Denise	8.605
MT	51	03502	Diamantino	20.475
MT	51	03601	Dom Aquino	8.153
MT	51	03700	Feliz Natal	11.253
MT	51	03809	Figueirópolis D'Oeste	3.757
MT	51	03858	Gaúcha do Norte	6.423
MT	51	03908	General Carneiro	5.080
MT	51	03957	Glória D'Oeste	3.118
MT	51	04104	Guarantã do Norte	32.525
MT	51	04203	Guiratinga	14.038
MT	51	04500	Indiavaí	2.424

MT	51	04526	Ipiranga do Norte	5.382
MT	51	04542	Itanhangá	5.420
MT	51	04559	Itaúba	4.483
MT	51	04609	Itiquira	11.653
MT	51	04807	Jaciara	25.790
MT	51	04906	Jangada	7.740
MT	51	05002	Jauru	10.255
MT	51	05101	Juara	32.948
MT	51	05150	Juína	39.351
MT	51	05176	Juruena	11.671
MT	51	05200	Juscimeira	11.382
MT	51	05234	Lambari D'Oeste	5.492
MT	51	05259	Lucas do Rio Verde	47.571
MT	51	05309	Luciára	2.204
MT	51	05580	Marcelândia	11.819
MT	51	05606	Matupá	14.396
MT	51	05622	Mirassol d'Oeste	25.495
MT	51	05903	Nobres	15.003
MT	51	06000	Nortelândia	6.374
MT	51	06109	Nossa Senhora do Livramento	11.579
MT	51	06158	Nova Bandeirantes	12.004
MT	51	06208	Nova Brasilândia	4.495
MT	51	06216	Nova Canaã do Norte	12.174
MT	51	08808	Nova Guarita	4.877
MT	51	06182	Nova Lacerda	5.544
MT	51	08857	Nova Marilândia	2.980
MT	51	08907	Nova Maringá	6.793
MT	51	08956	Nova Monte Verde	8.191
MT	51	06224	Nova Mutum	33.034
MT	51	06174	Nova Nazaré	3.110
MT	51	06232	Nova Olímpia	17.771
MT	51	06190	Nova Santa Helena	3.487
MT	51	06240	Nova Ubiratã	9.492
MT	51	06257	Nova Xavantina	19.783
MT	51	06273	Novo Horizonte do Norte	3.768
MT	51	06265	Novo Mundo	7.512
MT	51	06315	Novo Santo Antônio	2.068
MT	51	06281	Novo São Joaquim	5.924
MT	51	06299	Paranaíta	10.718
MT	51	06307	Paranatinga	19.594
MT	51	06372	Pedra Preta	15.920
MT	51	06422	Peixoto de Azevedo	31.170
MT	51	06455	Planalto da Serra	2.715
MT	51	06505	Poconé	31.857
MT	51	06653	Pontal do Araguaia	5.523
MT	51	06703	Ponte Branca	1.744
MT	51	06752	Pontes e Lacerda	41.741
MT	51	06778	Porto Alegre do Norte	10.912
MT	51	06802	Porto dos Gaúchos	5.433
MT	51	06828	Porto Esperidião	11.111
MT	51	06851	Porto Estrela	3.568
MT	51	07008	Poxoréo	17.413
MT	51	07040	Primavera do Leste	53.004
MT	51	07065	Querência	13.476
MT	51	07156	Reserva do Cabaçal	2.584
MT	51	07180	Ribeirão Cascalheira	9.002
MT	51	07198	Ribeirãozinho	2.216

MT	51	07206	Rio Branco	5.069
MT	51	07578	Rondolândia	3.638
MT	51	07602	Rondonópolis	198.950
MT	51	07701	Rosário Oeste	17.601
MT	51	07750	Salto do Céu	3.842
MT	51	07248	Santa Carmem	4.123
MT	51	07743	Santa Cruz do Xingu	1.967
MT	51	07768	Santa Rita do Trivelato	2.585
MT	51	07776	Santa Terezinha	7.484
MT	51	07263	Santo Afonso	3.001
MT	51	07792	Santo Antônio do Leste	3.898
MT	51	07800	Santo Antônio do Leverger	18.696
MT	51	07859	São Félix do Araguaia	10.716
MT	51	07297	São José do Povo	3.634
MT	51	07305	São José do Rio Claro	17.461
MT	51	07354	São José do Xingu	5.266
MT	51	07107	São José dos Quatro Marcos	18.945
MT	51	07404	São Pedro da Cipa	4.209
MT	51	07875	Sapezal	18.880
MT	51	07883	Serra Nova Dourada	1.393
MT	51	07909	Sinop	116.014
MT	51	07925	Sorriso	68.894
MT	51	07941	Tabaporã	9.863
MT	51	07958	Tangará da Serra	85.319
MT	51	08006	Tapurah	10.723
MT	51	08055	Terra Nova do Norte	11.107
MT	51	08105	Tesouro	3.436
MT	51	08204	Torixoréu	4.013
MT	51	08303	União do Sul	3.727
MT	51	08352	Vale de São Domingos	3.052
MT	51	08402	Várzea Grande	255.449
MT	51	08501	Vera	10.326
MT	51	05507	Vila Bela da Santíssima Trindade	14.634
MT	51	08600	Vila Rica	21.828
GO	52	00050	Abadia de Goiás	7.023
GO	52	00100	Abadiânia	16.088
GO	52	00134	Acreúna	20.431
GO	52	00159	Adelândia	2.479
GO	52	00175	Água Fria de Goiás	5.138
GO	52	00209	Água Limpa	1.999
GO	52	00258	Águas Lindas de Goiás	163.495
GO	52	00308	Alexânia	24.104
GO	52	00506	Aloândia	2.046
GO	52	00555	Alto Horizonte	4.654
GO	52	00605	Alto Paraíso de Goiás	6.939
GO	52	00803	Alvorada do Norte	8.125
GO	52	00829	Amaralina	3.462
GO	52	00852	Americano do Brasil	5.553
GO	52	00902	Amorinópolis	3.568
GO	52	01108	Anápolis	338.545
GO	52	01207	Anhanguera	1.030
GO	52	01306	Anicuns	20.353
GO	52	01405	Aparecida de Goiânia	465.093
GO	52	01454	Aparecida do Rio Doce	2.429
GO	52	01504	Aporé	3.832
GO	52	01603	Araçu	3.778
GO	52	01702	Aragarças	18.437

GO	52	01801	Aragoiânia	8.514
GO	52	02155	Araguapaz	7.526
GO	52	02353	Arenópolis	3.222
GO	52	02502	Aruanã	7.681
GO	52	02601	Aurilândia	3.606
GO	52	02809	Avelinópolis	2.446
GO	52	03104	Baliza	3.825
GO	52	03203	Barro Alto	8.906
GO	52	03302	Bela Vista de Goiás	24.965
GO	52	03401	Bom Jardim de Goiás	8.451
GO	52	03500	Bom Jesus de Goiás	21.071
GO	52	03559	Bonfinópolis	7.704
GO	52	03575	Bonópolis	3.573
GO	52	03609	Brazabrantes	3.268
GO	52	03807	Britânia	5.527
GO	52	03906	Buriti Alegre	9.080
GO	52	03939	Buriti de Goiás	2.553
GO	52	03962	Buritinópolis	3.317
GO	52	04003	Cabeceiras	7.400
GO	52	04102	Cachoeira Alta	10.700
GO	52	04201	Cachoeira de Goiás	1.411
GO	52	04250	Cachoeira Dourada	8.234
GO	52	04300	Caçu	13.491
GO	52	04409	Caiapônia	16.917
GO	52	04508	Caldas Novas	72.071
GO	52	04557	Caldazinha	3.361
GO	52	04607	Campestre de Goiás	3.404
GO	52	04656	Campinaçu	3.653
GO	52	04706	Campinorte	11.224
GO	52	04805	Campo Alegre de Goiás	6.178
GO	52	04854	Campo Limpo de Goiás	6.361
GO	52	04904	Campos Belos	18.515
GO	52	04953	Campos Verdes	4.787
GO	52	05000	Carmo do Rio Verde	9.014
GO	52	05059	Castelândia	3.620
GO	52	05109	Catalão	88.354
GO	52	05208	Caturaí	4.714
GO	52	05307	Cavalcante	9.411
GO	52	05406	Ceres	20.825
GO	52	05455	Cezarina	7.625
GO	52	05471	Chapadão do Céu	7.249
GO	52	05497	Cidade Ocidental	57.108
GO	52	05513	Cocalzinho de Goiás	17.621
GO	52	05521	Colinas do Sul	3.510
GO	52	05703	Córrego do Ouro	2.606
GO	52	05802	Corumbá de Goiás	10.414
GO	52	05901	Corumbaíba	8.299
GO	52	06206	Cristalina	47.537
GO	52	06305	Cristianópolis	2.933
GO	52	06404	Crixás	15.844
GO	52	06503	Cromínia	3.547
GO	52	06602	Cumari	2.954
GO	52	06701	Damianópolis	3.292
GO	52	06800	Damolândia	2.761
GO	52	06909	Davinópolis	2.058
GO	52	07105	Diorama	2.478
GO	52	08301	Divinópolis de Goiás	4.946

GO	52	07253	Doverlândia	7.841
GO	52	07352	Edealina	3.728
GO	52	07402	Edéia	11.347
GO	52	07501	Estrela do Norte	3.315
GO	52	07535	Faina	6.950
GO	52	07600	Fazenda Nova	6.263
GO	52	07808	Firminópolis	11.709
GO	52	07907	Flores de Goiás	12.416
GO	52	08004	Formosa	101.731
GO	52	08103	Formoso	4.829
GO	52	08152	Gameleira de Goiás	3.328
GO	52	08400	Goianápolis	10.697
GO	52	08509	Goiandira	5.288
GO	52	08608	Goianésia	60.347
GO	52	08707	Goiânia	1.318.149
GO	52	08806	Goianira	34.852
GO	52	08905	Goiás	24.544
GO	52	09101	Goiatuba	32.597
GO	52	09150	Gouvelândia	5.022
GO	52	09200	Guapó	13.985
GO	52	09291	Guaraíta	2.344
GO	52	09408	Guarani de Goiás	4.226
GO	52	09457	Guarinos	2.258
GO	52	09606	Heitoraí	3.581
GO	52	09705	Hidrolândia	17.729
GO	52	09804	Hidrolina	3.990
GO	52	09903	Iaciara	12.540
GO	52	09937	Inaciolândia	5.735
GO	52	09952	Indiara	13.831
GO	52	10000	Inhumas	48.580
GO	52	10109	Ipameri	24.897
GO	52	10158	Ipiranga de Goiás	2.846
GO	52	10208	Iporá	31.273
GO	52	10307	Israelândia	2.879
GO	52	10406	Itaberaí	35.947
GO	52	10562	Itaguari	4.523
GO	52	10604	Itaguaru	5.418
GO	52	10802	Itajá	5.017
GO	52	10901	Itapaci	18.806
GO	52	11008	Itapirapuã	7.604
GO	52	11206	Itapuranga	26.078
GO	52	11305	Itarumã	6.366
GO	52	11404	Itauçu	8.598
GO	52	11503	Itumbiara	93.763
GO	52	11602	Ivolândia	2.638
GO	52	11701	Jandaia	6.151
GO	52	11800	Jaraguá	42.530
GO	52	11909	Jataí	88.970
GO	52	12006	Jaupaci	2.989
GO	52	12055	Jesúpolis	2.314
GO	52	12105	Joviânia	7.135
GO	52	12204	Jussara	19.086
GO	52	12253	Lagoa Santa	1.280
GO	52	12303	Leopoldo de Bulhões	7.891
GO	52	12501	Luziânia	177.099
GO	52	12600	Mairipotaba	2.372
GO	52	12709	Mambaí	7.028

GO	52	12808	Mara Rosa	10.550
GO	52	12907	Marzagão	2.084
GO	52	12956	Matrinchã	4.406
GO	52	13004	Maurilândia	11.717
GO	52	13053	Mimoso de Goiás	2.677
GO	52	13087	Minaçu	30.966
GO	52	13103	Mineiros	54.003
GO	52	13400	Moiporá	1.744
GO	52	13509	Monte Alegre de Goiás	7.795
GO	52	13707	Montes Claros de Goiás	7.987
GO	52	13756	Montividiu	10.790
GO	52	13772	Montividiu do Norte	4.148
GO	52	13806	Morrinhos	41.804
GO	52	13855	Morro Agudo de Goiás	2.346
GO	52	13905	Mossâmedes	4.947
GO	52	14002	Mozarlândia	13.575
GO	52	14051	Mundo Novo	6.310
GO	52	14101	Mutunópolis	3.841
GO	52	14408	Nazário	7.970
GO	52	14507	Nerópolis	24.643
GO	52	14606	Niquelândia	42.652
GO	52	14705	Nova América	2.265
GO	52	14804	Nova Aurora	2.073
GO	52	14838	Nova Crixás	11.994
GO	52	14861	Nova Glória	8.475
GO	52	14879	Nova Iguaçu de Goiás	2.833
GO	52	14903	Nova Roma	3.453
GO	52	15009	Nova Veneza	8.261
GO	52	15207	Novo Brasil	3.469
GO	52	15231	Novo Gama	96.603
GO	52	15256	Novo Planalto	3.997
GO	52	15306	Orizona	14.395
GO	52	15405	Ouro Verde de Goiás	4.010
GO	52	15504	Ouvidor	5.559
GO	52	15603	Padre Bernardo	28.144
GO	52	15652	Palestina de Goiás	3.376
GO	52	15702	Palmeiras de Goiás	23.762
GO	52	15801	Palmelo	2.337
GO	52	15900	Palminópolis	3.557
GO	52	16007	Panamá	2.675
GO	52	16304	Paranaiguara	9.170
GO	52	16403	Paraúna	10.866
GO	52	16452	Perolândia	2.963
GO	52	16809	Petrolina de Goiás	10.276
GO	52	16908	Pilar de Goiás	2.730
GO	52	17104	Piracanjuba	24.062
GO	52	17203	Piranhas	11.188
GO	52	17302	Pirenópolis	23.142
GO	52	17401	Pires do Rio	28.957
GO	52	17609	Planaltina	82.258
GO	52	17708	Pontalina	17.165
GO	52	18003	Porangatu	42.568
GO	52	18052	Porteirão	3.388
GO	52	18102	Portelândia	3.850
GO	52	18300	Posse	31.833
GO	52	18391	Professor Jamil	3.227
GO	52	18508	Quirinópolis	43.735

GO	52	18607	Rialma	10.548
GO	52	18706	Rianápolis	4.582
GO	52	18789	Rio Quente	3.406
GO	52	18805	Rio Verde	181.020
GO	52	18904	Rubiataba	18.979
GO	52	19001	Sanclerlândia	7.552
GO	52	19100	Santa Bárbara de Goiás	5.812
GO	52	19209	Santa Cruz de Goiás	3.117
GO	52	19258	Santa Fé de Goiás	4.815
GO	52	19308	Santa Helena de Goiás	36.617
GO	52	19357	Santa Isabel	3.694
GO	52	19407	Santa Rita do Araguaia	7.066
GO	52	19456	Santa Rita do Novo Destino	3.185
GO	52	19506	Santa Rosa de Goiás	2.860
GO	52	19605	Santa Tereza de Goiás	3.942
GO	52	19704	Santa Terezinha de Goiás	10.171
GO	52	19712	Santo Antônio da Barra	4.452
GO	52	19738	Santo Antônio de Goiás	4.826
GO	52	19753	Santo Antônio do Descoberto	64.120
GO	52	19803	São Domingos	11.398
GO	52	19902	São Francisco de Goiás	6.128
GO	52	20058	São João da Paraúna	1.664
GO	52	20009	São João d'Aliança	10.528
GO	52	20108	São Luís de Montes Belos	30.315
GO	52	20157	São Luíz do Norte	4.658
GO	52	20207	São Miguel do Araguaia	22.244
GO	52	20264	São Miguel do Passa Quatro	3.779
GO	52	20280	São Patrício	1.994
GO	52	20405	São Simão	17.360
GO	52	20454	Senador Canedo	86.849
GO	52	20504	Serranópolis	7.561
GO	52	20603	Silvânia	19.193
GO	52	20686	Simolândia	6.537
GO	52	20702	Sítio d'Abadia	2.837
GO	52	21007	Taquaral de Goiás	3.538
GO	52	21080	Teresina de Goiás	3.050
GO	52	21197	Terezópolis de Goiás	6.675
GO	52	21304	Três Ranchos	2.819
GO	52	21403	Trindade	106.256
GO	52	21452	Trombas	3.454
GO	52	21502	Turvânia	4.817
GO	52	21551	Turvelândia	4.467
GO	52	21577	Uirapuru	2.925
GO	52	21601	Uruaçu	37.190
GO	52	21700	Uruana	13.818
GO	52	21809	Urutaí	3.072
GO	52	21858	Valparaíso de Goiás	135.909
GO	52	21908	Varjão	3.670
GO	52	22005	Vianópolis	12.644
GO	52	22054	Vicentinópolis	7.476
GO	52	22203	Vila Boa	4.847
GO	52	22302	Vila Propício	5.196
DF	53	00108	Brasília	2.609.998

Fonte: IBGE, Diretoria de Pesquisas - DPE. Coordenação de População e Indicadores Socias - COPIS.
