# Supplementary material for: Forecasting dengue fever in Brazil: An assessment of climate conditions
Source: PLoS One. 2019 Aug 8;14(8):e0220106. doi: 10.1371/journal.pone.0220106 (PMC6687106; doi:10.1371/journal.pone.0220106)
Supplement: S1 File — (ZIP) [file pone.0220106.s003.zip › data and codes_submit/training dataset analysis/data/Data IBGE/pop_municipios_2012.pdf]

ESTIMATIVAS DA POPULAÇÃO RESIDENTE NOS MUNICÍPIOS BRASILEIROS COM DATA DE REFERÊNCIA EM 1º DE JULHO DE 2012				
UF	COD. UF	COD. MUNIC	NOME DO MUNICÍPIO	POPULAÇÃO ESTIMADA
RO	11	00015	Alta Floresta D'Oeste	24.069
RO	11	00379	Alto Alegre dos Parecis	12.833
RO	11	00403	Alto Paraíso	17.742
RO	11	00346	Alvorada D'Oeste	16.404
RO	11	00023	Ariquemes	92.747
RO	11	00452	Buritis	33.397
RO	11	00031	Cabixi	6.132
RO	11	00601	Cacaulândia	5.791
RO	11	00049	Cacoal	79.330
RO	11	00700	Campo Novo de Rondônia	12.847
RO	11	00809	Candeias do Jamari	20.787
RO	11	00908	Castanheiras	3.479
RO	11	00056	Cerejeiras	16.852
RO	11	00924	Chupinguaia	8.721
RO	11	00064	Colorado do Oeste	18.093
RO	11	00072	Corumbiara	8.530
RO	11	00080	Costa Marques	14.355
RO	11	00940	Cujubim	17.262
RO	11	00098	Espigão D'Oeste	29.189
RO	11	01005	Governador Jorge Teixeira	10.040
RO	11	00106	Guajará-Mirim	42.202
RO	11	01104	Itapuã do Oeste	8.830
RO	11	00114	Jaru	51.765
RO	11	00122	Ji-Paraná	118.092
RO	11	00130	Machadinho D'Oeste	32.403
RO	11	01203	Ministro Andreazza	10.203
RO	11	01302	Mirante da Serra	11.686
RO	11	01401	Monte Negro	14.313
RO	11	00148	Nova Brasilândia D'Oeste	19.891
RO	11	00338	Nova Mamoré	23.719
RO	11	01435	Nova União	7.382
RO	11	00502	Novo Horizonte do Oeste	9.933
RO	11	00155	Ouro Preto do Oeste	37.482
RO	11	01450	Parecis	4.990
RO	11	00189	Pimenta Bueno	34.135
RO	11	01468	Pimenteiras do Oeste	2.283
RO	11	00205	Porto Velho	442.701
RO	11	00254	Presidente Médici	21.709
RO	11	01476	Primavera de Rondônia	3.406
RO	11	00262	Rio Crespo	3.374
RO	11	00288	Rolim de Moura	51.142

RO	11	00296	Santa Luzia D'Oeste	8.476
RO	11	01484	São Felipe D'Oeste	5.862
RO	11	01492	São Francisco do Guaporé	16.636
RO	11	00320	São Miguel do Guaporé	21.927
RO	11	01500	Seringueiras	11.619
RO	11	01559	Teixeirópolis	4.778
RO	11	01609	Theobroma	10.575
RO	11	01708	Urupá	12.687
RO	11	01757	Vale do Anari	9.633
RO	11	01807	Vale do Paraíso	7.961
RO	11	00304	Vilhena	79.616
AC	12	00013	Acrelândia	13.011
AC	12	00054	Assis Brasil	6.308
AC	12	00104	Brasiléia	22.261
AC	12	00138	Bujari	8.782
AC	12	00179	Capixaba	9.368
AC	12	00203	Cruzeiro do Sul	79.819
AC	12	00252	Epitaciolândia	15.679
AC	12	00302	Feijó	32.560
AC	12	00328	Jordão	6.898
AC	12	00336	Mâncio Lima	15.890
AC	12	00344	Manoel Urbano	8.224
AC	12	00351	Marechal Thaumaturgo	15.123
AC	12	00385	Plácido de Castro	17.587
AC	12	00807	Porto Acre	15.534
AC	12	00393	Porto Walter	9.711
AC	12	00401	Rio Branco	348.354
AC	12	00427	Rodrigues Alves	15.260
AC	12	00435	Santa Rosa do Purus	5.061
AC	12	00500	Sena Madureira	39.366
AC	12	00450	Senador Guiomard	20.588
AC	12	00609	Tarauacá	36.763
AC	12	00708	Xapuri	16.639
AM	13	00029	Alvarães	14.381
AM	13	00060	Amaturá	9.794
AM	13	00086	Anamã	10.766
AM	13	00102	Anori	17.072
AM	13	00144	Apuí	18.633
AM	13	00201	Atalaia do Norte	15.924
AM	13	00300	Autazes	33.312
AM	13	00409	Barcelos	25.948
AM	13	00508	Barreirinha	28.077
AM	13	00607	Benjamin Constant	34.950
AM	13	00631	Beruri	16.158
AM	13	00680	Boa Vista do Ramos	15.659
AM	13	00706	Boca do Acre	31.171

AM	13	00805	Borba	35.919
AM	13	00839	Caapiranga	11.303
AM	13	00904	Canutama	13.986
AM	13	01001	Carauari	26.130
AM	13	01100	Careiro	33.517
AM	13	01159	Careiro da Várzea	24.937
AM	13	01209	Coari	77.305
AM	13	01308	Codajás	24.067
AM	13	01407	Eirunepé	31.364
AM	13	01506	Envira	16.923
AM	13	01605	Fonte Boa	23.198
AM	13	01654	Guajará	14.396
AM	13	01704	Humaitá	45.954
AM	13	01803	Ipixuna	23.460
AM	13	01852	Iranduba	41.947
AM	13	01902	Itacoatiara	89.064
AM	13	01951	Itamarati	7.983
AM	13	02009	Itapiranga	8.348
AM	13	02108	Japurá	7.448
AM	13	02207	Juruá	11.439
AM	13	02306	Jutaí	18.293
AM	13	02405	Lábrea	39.022
AM	13	02504	Manacapuru	86.985
AM	13	02553	Manaquiri	24.325
AM	13	02603	Manaus	1.861.838
AM	13	02702	Manicoré	48.373
AM	13	02801	Maraã	17.596
AM	13	02900	Maués	54.079
AM	13	03007	Nhamundá	18.720
AM	13	03106	Nova Olinda do Norte	31.749
AM	13	03205	Novo Airão	15.489
AM	13	03304	Novo Aripuanã	22.106
AM	13	03403	Parintins	103.828
AM	13	03502	Pauini	18.329
AM	13	03536	Presidente Figueiredo	28.652
AM	13	03569	Rio Preto da Eva	26.948
AM	13	03601	Santa Isabel do Rio Negro	19.292
AM	13	03700	Santo Antônio do Içá	24.890
AM	13	03809	São Gabriel da Cachoeira	39.097
AM	13	03908	São Paulo de Olivença	32.677
AM	13	03957	São Sebastião do Uatumã	11.241
AM	13	04005	Silves	8.544
AM	13	04062	Tabatinga	54.440
AM	13	04104	Tapauá	17.903
AM	13	04203	Tefé	61.000
AM	13	04237	Tonantins	17.316

AM	13	04260	Uarini	12.139
AM	13	04302	Urucará	16.902
AM	13	04401	Urucurituba	18.679
RR	14	00050	Alto Alegre	16.228
RR	14	00027	Amajari	9.936
RR	14	00100	Boa Vista	296.959
RR	14	00159	Bonfim	11.188
RR	14	00175	Cantá	14.707
RR	14	00209	Caracaraí	19.019
RR	14	00233	Caroebe	8.480
RR	14	00282	Iracema	9.288
RR	14	00308	Mucajaí	15.328
RR	14	00407	Normandia	9.364
RR	14	00456	Pacaraima	10.953
RR	14	00472	Rorainópolis	25.319
RR	14	00506	São João da Baliza	7.023
RR	14	00605	São Luiz	6.968
RR	14	00704	Uiramutã	8.764
PA	15	00107	Abaetetuba	144.415
PA	15	00131	Abel Figueiredo	6.905
PA	15	00206	Acará	53.787
PA	15	00305	Afuá	35.879
PA	15	00347	Água Azul do Norte	25.506
PA	15	00404	Alenquer	53.369
PA	15	00503	Almeirim	33.563
PA	15	00602	Altamira	102.343
PA	15	00701	Anajás	25.731
PA	15	00800	Ananindeua	483.821
PA	15	00859	Anapu	22.225
PA	15	00909	Augusto Corrêa	41.628
PA	15	00958	Aurora do Pará	27.576
PA	15	01006	Aveiro	15.899
PA	15	01105	Bagre	25.398
PA	15	01204	Baião	39.263
PA	15	01253	Bannach	3.379
PA	15	01303	Barcarena	105.385
PA	15	01402	Belém	1.410.430
PA	15	01451	Belterra	16.579
PA	15	01501	Benevides	54.083
PA	15	01576	Bom Jesus do Tocantins	15.629
PA	15	01600	Bonito	14.207
PA	15	01709	Bragança	116.164
PA	15	01725	Brasil Novo	(*) 17.960
PA	15	01758	Brejo Grande do Araguaia	7.295
PA	15	01782	Breu Branco	55.521
PA	15	01808	Breves	94.779

PA	15	01907	Bujaru	26.400
PA	15	02004	Cachoeira do Arari	21.147
PA	15	01956	Cachoeira do Piriá	28.153
PA	15	02103	Cametá	124.411
PA	15	02152	Canaã dos Carajás	29.101
PA	15	02202	Capanema	64.624
PA	15	02301	Capitão Poço	52.214
PA	15	02400	Castanhal	178.986
PA	15	02509	Chaves	21.557
PA	15	02608	Colares	11.495
PA	15	02707	Conceição do Araguaia	45.885
PA	15	02756	Concórdia do Pará	29.313
PA	15	02764	Cumaru do Norte	11.144
PA	15	02772	Curionópolis	18.108
PA	15	02806	Curralinho	29.838
PA	15	02855	Curuá	12.712
PA	15	02905	Curuçá	35.523
PA	15	02939	Dom Eliseu	53.100
PA	15	02954	Eldorado dos Carajás	32.115
PA	15	03002	Faro	7.897
PA	15	03044	Floresta do Araguaia	18.295
PA	15	03077	Garrafão do Norte	25.157
PA	15	03093	Goianésia do Pará	35.299
PA	15	03101	Gurupá	29.963
PA	15	03200	Igarapé-Açu	36.414
PA	15	03309	Igarapé-Miri	58.904
PA	15	03408	Inhangapi	10.393
PA	15	03457	Ipixuna do Pará	51.569
PA	15	03507	Irituia	31.492
PA	15	03606	Itaituba	97.908
PA	15	03705	Itupiranga	51.457
PA	15	03754	Jacareacanga	(*) 41.487
PA	15	03804	Jacundá	52.993
PA	15	03903	Juruti	49.486
PA	15	04000	Limoeiro do Ajuru	25.846
PA	15	04059	Mãe do Rio	28.290
PA	15	04109	Magalhães Barata	8.179
PA	15	04208	Marabá	243.583
PA	15	04307	Maracanã	28.498
PA	15	04406	Marapanim	26.890
PA	15	04422	Marituba	113.353
PA	15	04455	Medicilândia	28.227
PA	15	04505	Melgaço	25.374
PA	15	04604	Mocajuba	27.666
PA	15	04703	Moju	72.597
PA	15	04752	Mojuí dos Campos (**)	15.018

PA	15	04802	Monte Alegre	55.804
PA	15	04901	Muaná	35.524
PA	15	04950	Nova Esperança do Piriá	20.350
PA	15	04976	Nova Ipixuna	15.065
PA	15	05007	Nova Timboteua	14.012
PA	15	05031	Novo Progresso	25.151
PA	15	05064	Novo Repartimento	65.106
PA	15	05106	Óbidos	49.763
PA	15	05205	Oeiras do Pará	29.402
PA	15	05304	Oriximiná	64.978
PA	15	05403	Ourém	16.601
PA	15	05437	Ourilândia do Norte	28.551
PA	15	05486	Pacajá	41.654
PA	15	05494	Palestina do Pará	7.465
PA	15	05502	Paragominas	101.046
PA	15	05536	Parauapebas	166.342
PA	15	05551	Pau D'Arco	5.869
PA	15	05601	Peixe-Boi	7.869
PA	15	05635	Piçarra	12.701
PA	15	05650	Placas	25.526
PA	15	05700	Ponta de Pedras	27.103
PA	15	05809	Portel	54.306
PA	15	05908	Porto de Moz	35.529
PA	15	06005	Prainha	29.325
PA	15	06104	Primavera	10.352
PA	15	06112	Quatipuru	12.639
PA	15	06138	Redenção	77.415
PA	15	06161	Rio Maria	17.728
PA	15	06187	Rondon do Pará	48.036
PA	15	06195	Rurópolis	42.417
PA	15	06203	Salinópolis	38.021
PA	15	06302	Salvaterra	20.948
PA	15	06351	Santa Bárbara do Pará	18.012
PA	15	06401	Santa Cruz do Arari	8.593
PA	15	06500	Santa Isabel do Pará	61.919
PA	15	06559	Santa Luzia do Pará	19.428
PA	15	06583	Santa Maria das Barreiras	18.150
PA	15	06609	Santa Maria do Pará	23.355
PA	15	06708	Santana do Araguaia	59.919
PA	15	06807	Santarém	284.401
PA	15	06906	Santarém Novo	6.248
PA	15	07003	Santo Antônio do Tauá	27.707
PA	15	07102	São Caetano de Odivelas	17.087
PA	15	07151	São Domingos do Araguaia	23.602
PA	15	07201	São Domingos do Capim	30.215
PA	15	07300	São Félix do Xingu	99.905

PA	15	07409	São Francisco do Pará	15.184
PA	15	07458	São Geraldo do Araguaia	25.277
PA	15	07466	São João da Ponta	5.451
PA	15	07474	São João de Pirabas	21.125
PA	15	07508	São João do Araguaia	13.293
PA	15	07607	São Miguel do Guamá	53.108
PA	15	07706	São Sebastião da Boa Vista	23.696
PA	15	07755	Sapucaia	5.236
PA	15	07805	Senador José Porfírio	12.641
PA	15	07904	Soure	23.461
PA	15	07953	Tailândia	85.468
PA	15	07961	Terra Alta	10.565
PA	15	07979	Terra Santa	17.305
PA	15	08001	Tomé-Açu	57.914
PA	15	08035	Tracuateua	28.167
PA	15	08050	Trairão	17.303
PA	15	08084	Tucumã	34.956
PA	15	08100	Tucuruí	100.651
PA	15	08126	Ulianópolis	46.979
PA	15	08159	Uruará	44.727
PA	15	08209	Vigia	49.054
PA	15	08308	Viseu	57.566
PA	15	08357	Vitória do Xingu	13.777
PA	15	08407	Xinguara	41.382
AP	16	00105	Amapá	8.213
AP	16	00204	Calçoene	9.343
AP	16	00212	Cutias	4.910
AP	16	00238	Ferreira Gomes	6.141
AP	16	00253	Itaubal	4.473
AP	16	00279	Laranjal do Jari	41.668
AP	16	00303	Macapá	415.554
AP	16	00402	Mazagão	17.794
AP	16	00501	Oiapoque	21.661
AP	16	00154	Pedra Branca do Amapari	11.794
AP	16	00535	Porto Grande	17.680
AP	16	00550	Pracuúba	4.021
AP	16	00600	Santana	104.407
AP	16	00055	Serra do Navio	4.545
AP	16	00709	Tartarugalzinho	13.385
AP	16	00808	Vitória do Jari	13.013
TO	17	00251	Abreulândia	2.422
TO	17	00301	Aguiarnópolis	5.467
TO	17	00350	Aliança do Tocantins	5.595
TO	17	00400	Almas	7.452
TO	17	00707	Alvorada	8.354
TO	17	01002	Ananás	9.768

TO	17	01051	Angico	3.219
TO	17	01101	Aparecida do Rio Negro	4.319
TO	17	01309	Aragominas	5.838
TO	17	01903	Araguacema	6.454
TO	17	02000	Araguaçu	8.702
TO	17	02109	Araguaína	156.123
TO	17	02158	Araguanã	5.157
TO	17	02208	Araguatins	32.133
TO	17	02307	Arapoema	6.700
TO	17	02406	Arraias	10.594
TO	17	02554	Augustinópolis	16.401
TO	17	02703	Aurora do Tocantins	3.499
TO	17	02901	Axixá do Tocantins	9.343
TO	17	03008	Babaçulândia	10.439
TO	17	03057	Bandeirantes do Tocantins	3.200
TO	17	03073	Barra do Ouro	4.206
TO	17	03107	Barrolândia	5.390
TO	17	03206	Bernardo Sayão	4.442
TO	17	03305	Bom Jesus do Tocantins	3.987
TO	17	03602	Brasilândia do Tocantins	2.086
TO	17	03701	Brejinho de Nazaré	5.232
TO	17	03800	Buriti do Tocantins	10.059
TO	17	03826	Cachoeirinha	2.167
TO	17	03842	Campos Lindos	8.517
TO	17	03867	Cariri do Tocantins	3.872
TO	17	03883	Carmolândia	2.363
TO	17	03891	Carrasco Bonito	3.759
TO	17	03909	Caseara	4.744
TO	17	04105	Centenário	2.627
TO	17	05102	Chapada da Natividade	3.278
TO	17	04600	Chapada de Areia	1.345
TO	17	05508	Colinas do Tocantins	31.675
TO	17	16703	Colméia	8.500
TO	17	05557	Combinado	4.691
TO	17	05607	Conceição do Tocantins	4.153
TO	17	06001	Couto de Magalhães	5.111
TO	17	06100	Cristalândia	7.222
TO	17	06258	Crixás do Tocantins	1.592
TO	17	06506	Darcinópolis	5.425
TO	17	07009	Dianópolis	19.669
TO	17	07108	Divinópolis do Tocantins	6.452
TO	17	07207	Dois Irmãos do Tocantins	7.145
TO	17	07306	Dueré	4.597
TO	17	07405	Esperantina	9.756
TO	17	07553	Fátima	3.799
TO	17	07652	Figueirópolis	5.307

TO	17	07702	Filadélfia	8.549
TO	17	08205	Formoso do Araguaia	18.369
TO	17	08254	Fortaleza do Tabocão	2.446
TO	17	08304	Goianorte	4.974
TO	17	09005	Goiatins	12.220
TO	17	09302	Guaraí	23.681
TO	17	09500	Gurupi	78.525
TO	17	09807	Ipueiras	1.711
TO	17	10508	Itacajá	7.148
TO	17	10706	Itaguatins	5.976
TO	17	10904	Itapiratins	3.571
TO	17	11100	Itaporã do Tocantins	2.434
TO	17	11506	Jaú do Tocantins	3.566
TO	17	11803	Juarina	2.216
TO	17	11902	Lagoa da Confusão	10.821
TO	17	11951	Lagoa do Tocantins	3.676
TO	17	12009	Lajeado	2.838
TO	17	12157	Lavandeira	1.665
TO	17	12405	Lizarda	3.716
TO	17	12454	Luzinópolis	2.713
TO	17	12504	Marianópolis do Tocantins	4.507
TO	17	12702	Mateiros	2.311
TO	17	12801	Maurilândia do Tocantins	3.200
TO	17	13205	Miracema do Tocantins	20.117
TO	17	13304	Miranorte	12.747
TO	17	13601	Monte do Carmo	6.946
TO	17	13700	Monte Santo do Tocantins	2.118
TO	17	13957	Muricilândia	3.224
TO	17	14203	Natividade	9.021
TO	17	14302	Nazaré	4.271
TO	17	14880	Nova Olinda	10.883
TO	17	15002	Nova Rosalândia	3.858
TO	17	15101	Novo Acordo	3.869
TO	17	15150	Novo Alegre	2.288
TO	17	15259	Novo Jardim	2.504
TO	17	15507	Oliveira de Fátima	1.049
TO	17	21000	Palmas	242.070
TO	17	15705	Palmeirante	5.157
TO	17	13809	Palmeiras do Tocantins	5.909
TO	17	15754	Palmeirópolis	7.380
TO	17	16109	Paraíso do Tocantins	45.669
TO	17	16208	Paranã	10.327
TO	17	16307	Pau D'Arco	4.627
TO	17	16505	Pedro Afonso	11.919
TO	17	16604	Peixe	10.629
TO	17	16653	Pequizeiro	5.124

TO	17	17008	Pindorama do Tocantins	4.479
TO	17	17206	Piraquê	2.933
TO	17	17503	Pium	6.869
TO	17	17800	Ponte Alta do Bom Jesus	4.540
TO	17	17909	Ponte Alta do Tocantins	7.333
TO	17	18006	Porto Alegre do Tocantins	2.857
TO	17	18204	Porto Nacional	49.774
TO	17	18303	Praia Norte	7.792
TO	17	18402	Presidente Kennedy	3.670
TO	17	18451	Pugmil	2.427
TO	17	18501	Recursolândia	3.864
TO	17	18550	Riachinho	4.270
TO	17	18659	Rio da Conceição	1.794
TO	17	18709	Rio dos Bois	2.616
TO	17	18758	Rio Sono	6.279
TO	17	18808	Sampaio	4.025
TO	17	18840	Sandolândia	3.326
TO	17	18865	Santa Fé do Araguaia	6.764
TO	17	18881	Santa Maria do Tocantins	2.995
TO	17	18899	Santa Rita do Tocantins	2.170
TO	17	18907	Santa Rosa do Tocantins	4.607
TO	17	19004	Santa Tereza do Tocantins	2.585
TO	17	20002	Santa Terezinha do Tocantins	2.477
TO	17	20101	São Bento do Tocantins	4.740
TO	17	20150	São Félix do Tocantins	1.463
TO	17	20200	São Miguel do Tocantins	10.783
TO	17	20259	São Salvador do Tocantins	2.936
TO	17	20309	São Sebastião do Tocantins	4.376
TO	17	20499	São Valério da Natividade	4.282
TO	17	20655	Silvanópolis	5.120
TO	17	20804	Sítio Novo do Tocantins	9.097
TO	17	20853	Sucupira	1.783
TO	17	20903	Taguatinga	15.336
TO	17	20937	Taipas do Tocantins	1.981
TO	17	20978	Talismã	2.601
TO	17	21109	Tocantínia	6.880
TO	17	21208	Tocantinópolis	22.596
TO	17	21257	Tupirama	1.634
TO	17	21307	Tupiratins	2.208
TO	17	22081	Wanderlândia	11.088
TO	17	22107	Xambioá	11.458
MA	21	00055	Açailândia	106.422
MA	21	00105	Afonso Cunha	6.090
MA	21	00154	Água Doce do Maranhão	11.865
MA	21	00204	Alcântara	21.605
MA	21	00303	Aldeias Altas	24.726

MA	21	00402	Altamira do Maranhão	11.381
MA	21	00436	Alto Alegre do Maranhão	25.326
MA	21	00477	Alto Alegre do Pindaré	31.190
MA	21	00501	Alto Parnaíba	10.856
MA	21	00550	Amapá do Maranhão	6.583
MA	21	00600	Amarante do Maranhão	38.953
MA	21	00709	Anajatuba	25.955
MA	21	00808	Anapurus	14.492
MA	21	00832	Apicum-Açu	15.542
MA	21	00873	Araguanã	14.407
MA	21	00907	Araioses	43.653
MA	21	00956	Arame	31.729
MA	21	01004	Arari	28.809
MA	21	01103	Axixá	11.599
MA	21	01202	Bacabal	101.195
MA	21	01251	Bacabeira	15.591
MA	21	01301	Bacuri	17.437
MA	21	01350	Bacurituba	5.387
MA	21	01400	Balsas	87.057
MA	21	01509	Barão de Grajaú	17.862
MA	21	01608	Barra do Corda	84.180
MA	21	01707	Barreirinhas	58.083
MA	21	01772	Bela Vista do Maranhão	12.335
MA	21	01731	Belágua	6.986
MA	21	01806	Benedito Leite	5.497
MA	21	01905	Bequimão	20.773
MA	21	01939	Bernardo do Mearim	6.111
MA	21	01970	Boa Vista do Gurupi	8.375
MA	21	02002	Bom Jardim	39.740
MA	21	02036	Bom Jesus das Selvas	30.259
MA	21	02077	Bom Lugar	15.314
MA	21	02101	Brejo	34.242
MA	21	02150	Brejo de Areia	4.962
MA	21	02200	Buriti	27.449
MA	21	02309	Buriti Bravo	23.119
MA	21	02325	Buriticupu	67.378
MA	21	02358	Buritirana	14.930
MA	21	02374	Cachoeira Grande	8.607
MA	21	02408	Cajapió	10.740
MA	21	02507	Cajari	18.603
MA	21	02556	Campestre do Maranhão	13.649
MA	21	02606	Cândido Mendes	19.222
MA	21	02705	Cantanhede	20.879
MA	21	02754	Capinzal do Norte	10.722
MA	21	02804	Carolina	23.955
MA	21	02903	Carutapera	22.517

MA	21	03000	Caxias	158.059
MA	21	03109	Cedral	10.374
MA	21	03125	Central do Maranhão	8.120
MA	21	03158	Centro do Guilherme	11.979
MA	21	03174	Centro Novo do Maranhão	19.947
MA	21	03208	Chapadinha	75.167
MA	21	03257	Cidelândia	13.963
MA	21	03307	Codó	119.079
MA	21	03406	Coelho Neto	47.435
MA	21	03505	Colinas	39.635
MA	21	03554	Conceição do Lago-Açu	14.989
MA	21	03604	Coroatá	62.639
MA	21	03703	Cururupu	32.487
MA	21	03752	Davinópolis	12.625
MA	21	03802	Dom Pedro	22.791
MA	21	03901	Duque Bacelar	10.836
MA	21	04008	Esperantinópolis	17.715
MA	21	04057	Estreito	37.784
MA	21	04073	Feira Nova do Maranhão	8.215
MA	21	04081	Fernando Falcão	9.584
MA	21	04099	Formosa da Serra Negra	17.749
MA	21	04107	Fortaleza dos Nogueiras	12.306
MA	21	04206	Fortuna	15.174
MA	21	04305	Godofredo Viana	10.762
MA	21	04404	Gonçalves Dias	17.545
MA	21	04503	Governador Archer	10.372
MA	21	04552	Governador Edison Lobão	16.651
MA	21	04602	Governador Eugênio Barros	16.197
MA	21	04628	Governador Luiz Rocha	7.462
MA	21	04651	Governador Newton Bello	10.166
MA	21	04677	Governador Nunes Freire	25.323
MA	21	04701	Graça Aranha	6.150
MA	21	04800	Grajaú	64.510
MA	21	04909	Guimarães	11.997
MA	21	05005	Humberto de Campos	26.933
MA	21	05104	Icatu	25.698
MA	21	05153	Igarapé do Meio	13.052
MA	21	05203	Igarapé Grande	11.289
MA	21	05302	Imperatriz	250.063
MA	21	05351	Itaipava do Grajaú	13.103
MA	21	05401	Itapecuru Mirim	63.907
MA	21	05427	Itinga do Maranhão	25.125
MA	21	05450	Jatobá	9.051
MA	21	05476	Jenipapo dos Vieiras	15.733
MA	21	05500	João Lisboa	23.561
MA	21	05609	Joselândia	15.688

MA	21	05658	Junco do Maranhão	3.792
MA	21	05708	Lago da Pedra	47.298
MA	21	05807	Lago do Junco	10.865
MA	21	05948	Lago dos Rodrigues	7.744
MA	21	05906	Lago Verde	15.624
MA	21	05922	Lagoa do Mato	10.955
MA	21	05963	Lagoa Grande do Maranhão	12.501
MA	21	05989	Lajeado Novo	7.106
MA	21	06003	Lima Campos	11.525
MA	21	06102	Loreto	11.597
MA	21	06201	Luís Domingues	6.629
MA	21	06300	Magalhães de Almeida	18.277
MA	21	06326	Maracaçumé	19.887
MA	21	06359	Marajá do Sena	7.751
MA	21	06375	Maranhãozinho	14.524
MA	21	06409	Mata Roma	15.657
MA	21	06508	Matinha	22.286
MA	21	06607	Matões	32.216
MA	21	06631	Matões do Norte	14.755
MA	21	06672	Milagres do Maranhão	8.195
MA	21	06706	Mirador	20.537
MA	21	06755	Miranda do Norte	25.681
MA	21	06805	Mirinzal	14.402
MA	21	06904	Monção	31.717
MA	21	07001	Montes Altos	9.272
MA	21	07100	Morros	18.265
MA	21	07209	Nina Rodrigues	13.095
MA	21	07258	Nova Colinas	5.034
MA	21	07308	Nova Iorque	4.598
MA	21	07357	Nova Olinda do Maranhão	19.659
MA	21	07407	Olho d'Água das Cunhãs	18.816
MA	21	07456	Olinda Nova do Maranhão	13.643
MA	21	07506	Paço do Lumiar	110.321
MA	21	07605	Palmeirândia	19.007
MA	21	07704	Paraibano	20.443
MA	21	07803	Parnarama	33.669
MA	21	07902	Passagem Franca	17.977
MA	21	08009	Pastos Bons	18.461
MA	21	08058	Paulino Neves	14.971
MA	21	08108	Paulo Ramos	20.454
MA	21	08207	Pedreiras	39.391
MA	21	08256	Pedro do Rosário	23.454
MA	21	08306	Penalva	35.996
MA	21	08405	Peri Mirim	13.898
MA	21	08454	Peritoró	21.785
MA	21	08504	Pindaré-Mirim	31.609

MA	21	08603	Pinheiro	79.566
MA	21	08702	Pio XII	21.708
MA	21	08801	Pirapemas	17.722
MA	21	08900	Poção de Pedras	19.165
MA	21	09007	Porto Franco	22.239
MA	21	09056	Porto Rico do Maranhão	5.978
MA	21	09106	Presidente Dutra	45.564
MA	21	09205	Presidente Juscelino	11.897
MA	21	09239	Presidente Médici	6.564
MA	21	09270	Presidente Sarney	17.686
MA	21	09304	Presidente Vargas	10.964
MA	21	09403	Primeira Cruz	14.355
MA	21	09452	Raposa	27.723
MA	21	09502	Riachão	20.093
MA	21	09551	Ribamar Fiquene	7.444
MA	21	09601	Rosário	40.469
MA	21	09700	Sambaíba	5.522
MA	21	09759	Santa Filomena do Maranhão	7.246
MA	21	09809	Santa Helena	40.356
MA	21	09908	Santa Inês	78.733
MA	21	10005	Santa Luzia	74.943
MA	21	10039	Santa Luzia do Paruá	23.035
MA	21	10104	Santa Quitéria do Maranhão	28.914
MA	21	10203	Santa Rita	33.843
MA	21	10237	Santana do Maranhão	12.203
MA	21	10278	Santo Amaro do Maranhão	14.456
MA	21	10302	Santo Antônio dos Lopes	14.294
MA	21	10401	São Benedito do Rio Preto	18.004
MA	21	10500	São Bento	42.083
MA	21	10609	São Bernardo	27.044
MA	21	10658	São Domingos do Azeitão	7.088
MA	21	10708	São Domingos do Maranhão	33.692
MA	21	10807	São Félix de Balsas	4.636
MA	21	10856	São Francisco do Brejão	10.745
MA	21	10906	São Francisco do Maranhão	11.932
MA	21	11003	São João Batista	20.072
MA	21	11029	São João do Carú	15.631
MA	21	11052	São João do Paraíso	10.882
MA	21	11078	São João do Soter	17.602
MA	21	11102	São João dos Patos	25.056
MA	21	11201	São José de Ribamar	167.714
MA	21	11250	São José dos Basílios	7.506
MA	21	11300	São Luís	1.039.610
MA	21	11409	São Luís Gonzaga do Maranhão	19.758
MA	21	11508	São Mateus do Maranhão	39.733
MA	21	11532	São Pedro da Água Branca	12.195

MA	21	11573	São Pedro dos Crentes	4.486
MA	21	11607	São Raimundo das Mangabeiras	17.868
MA	21	11631	São Raimundo do Doca Bezerra	5.757
MA	21	11672	São Roberto	6.193
MA	21	11706	São Vicente Ferrer	21.235
MA	21	11722	Satubinha	12.600
MA	21	11748	Senador Alexandre Costa	10.511
MA	21	11763	Senador La Rocque	14.447
MA	21	11789	Serrano do Maranhão	10.545
MA	21	11805	Sítio Novo	17.288
MA	21	11904	Sucupira do Norte	10.454
MA	21	11953	Sucupira do Riachão	5.466
MA	21	12001	Tasso Fragoso	8.008
MA	21	12100	Timbiras	28.238
MA	21	12209	Timon	159.471
MA	21	12233	Trizidela do Vale	19.339
MA	21	12274	Tufilândia	5.651
MA	21	12308	Tuntum	39.924
MA	21	12407	Turiaçu	34.333
MA	21	12456	Turilândia	23.694
MA	21	12506	Tutóia	54.629
MA	21	12605	Urbano Santos	25.356
MA	21	12704	Vargem Grande	51.633
MA	21	12803	Viana	50.257
MA	21	12852	Vila Nova dos Martírios	11.946
MA	21	12902	Vitória do Mearim	31.588
MA	21	13009	Vitorino Freire	31.709
MA	21	14007	Zé Doca	49.355
PI	22	00053	Acauã	6.840
PI	22	00103	Agricolândia	5.062
PI	22	00202	Água Branca	16.744
PI	22	00251	Alagoinha do Piauí	7.413
PI	22	00277	Alegrete do Piauí	5.173
PI	22	00301	Alto Longá	13.820
PI	22	00400	Altos	39.232
PI	22	00459	Alvorada do Gurguéia	5.177
PI	22	00509	Amarante	17.173
PI	22	00608	Angical do Piauí	6.655
PI	22	00707	Anísio de Abreu	9.385
PI	22	00806	Antônio Almeida	3.068
PI	22	00905	Aroazes	5.742
PI	22	00954	Aroeiras do Itaim	2.442
PI	22	01002	Arraial	4.655
PI	22	01051	Assunção do Piauí	7.590
PI	22	01101	Avelino Lopes	11.258
PI	22	01150	Baixa Grande do Ribeiro	10.930

PI	22	01176	Barra D'Alcântara	3.858
PI	22	01200	Barras	45.448
PI	22	01309	Barreiras do Piauí	3.255
PI	22	01408	Barro Duro	6.580
PI	22	01507	Batalha	26.023
PI	22	01556	Bela Vista do Piauí	3.854
PI	22	01572	Belém do Piauí	3.388
PI	22	01606	Beneditinos	9.943
PI	22	01705	Bertolínia	5.350
PI	22	01739	Betânia do Piauí	6.042
PI	22	01770	Boa Hora	6.467
PI	22	01804	Bocaina	4.394
PI	22	01903	Bom Jesus	23.642
PI	22	01919	Bom Princípio do Piauí	5.407
PI	22	01929	Bonfim do Piauí	5.471
PI	22	01945	Boqueirão do Piauí	6.288
PI	22	01960	Brasileira	8.057
PI	22	01988	Brejo do Piauí	3.724
PI	22	02000	Buriti dos Lopes	19.212
PI	22	02026	Buriti dos Montes	8.079
PI	22	02059	Cabeceiras do Piauí	10.144
PI	22	02075	Cajazeiras do Piauí	3.413
PI	22	02083	Cajueiro da Praia	7.321
PI	22	02091	Caldeirão Grande do Piauí	5.700
PI	22	02109	Campinas do Piauí	5.449
PI	22	02117	Campo Alegre do Fidalgo	4.815
PI	22	02133	Campo Grande do Piauí	5.704
PI	22	02174	Campo Largo do Piauí	6.964
PI	22	02208	Campo Maior	45.493
PI	22	02251	Canavieira	3.892
PI	22	02307	Canto do Buriti	20.375
PI	22	02406	Capitão de Campos	11.092
PI	22	02455	Capitão Gervásio Oliveira	3.946
PI	22	02505	Caracol	10.448
PI	22	02539	Caraúbas do Piauí	5.634
PI	22	02554	Caridade do Piauí	4.915
PI	22	02604	Castelo do Piauí	18.336
PI	22	02653	Caxingó	5.174
PI	22	02703	Cocal	27.067
PI	22	02711	Cocal de Telha	4.567
PI	22	02729	Cocal dos Alves	5.635
PI	22	02737	Coivaras	3.872
PI	22	02752	Colônia do Gurguéia	6.191
PI	22	02778	Colônia do Piauí	7.461
PI	22	02802	Conceição do Canindé	4.496
PI	22	02851	Coronel José Dias	4.561

PI	22	02901	Corrente	25.737
PI	22	03008	Cristalândia do Piauí	7.973
PI	22	03107	Cristino Castro	10.089
PI	22	03206	Curimatá	10.948
PI	22	03230	Currais	4.776
PI	22	03271	Curral Novo do Piauí	4.990
PI	22	03255	Curralinhos	4.265
PI	22	03305	Demerval Lobão	13.398
PI	22	03354	Dirceu Arcoverde	6.767
PI	22	03404	Dom Expedito Lopes	6.662
PI	22	03453	Dom Inocêncio	9.296
PI	22	03420	Domingos Mourão	4.261
PI	22	03503	Elesbão Veloso	14.394
PI	22	03602	Eliseu Martins	4.738
PI	22	03701	Esperantina	38.322
PI	22	03750	Fartura do Piauí	5.133
PI	22	03800	Flores do Piauí	4.366
PI	22	03859	Floresta do Piauí	2.492
PI	22	03909	Floriano	58.158
PI	22	04006	Francinópolis	5.233
PI	22	04105	Francisco Ayres	4.363
PI	22	04154	Francisco Macedo	2.961
PI	22	04204	Francisco Santos	8.857
PI	22	04303	Fronteiras	11.284
PI	22	04352	Geminiano	5.237
PI	22	04402	Gilbués	10.429
PI	22	04501	Guadalupe	10.268
PI	22	04550	Guaribas	4.432
PI	22	04600	Hugo Napoleão	3.782
PI	22	04659	Ilha Grande	9.069
PI	22	04709	Inhuma	14.909
PI	22	04808	Ipiranga do Piauí	9.463
PI	22	04907	Isaías Coelho	8.307
PI	22	05003	Itainópolis	11.219
PI	22	05102	Itaueira	10.728
PI	22	05151	Jacobina do Piauí	5.670
PI	22	05201	Jaicós	18.364
PI	22	05250	Jardim do Mulato	4.358
PI	22	05276	Jatobá do Piauí	4.708
PI	22	05300	Jerumenha	4.372
PI	22	05359	João Costa	2.951
PI	22	05409	Joaquim Pires	13.929
PI	22	05458	Joca Marques	5.214
PI	22	05508	José de Freitas	37.724
PI	22	05516	Juazeiro do Piauí	4.793
PI	22	05524	Júlio Borges	5.439

PI	22	05532	Jurema	4.588
PI	22	05557	Lagoa Alegre	8.184
PI	22	05573	Lagoa de São Francisco	6.517
PI	22	05565	Lagoa do Barro do Piauí	4.535
PI	22	05581	Lagoa do Piauí	3.920
PI	22	05599	Lagoa do Sítio	4.958
PI	22	05540	Lagoinha do Piauí	2.721
PI	22	05607	Landri Sales	5.229
PI	22	05706	Luís Correia	29.034
PI	22	05805	Luzilândia	24.824
PI	22	05854	Madeiro	7.974
PI	22	05904	Manoel Emídio	5.223
PI	22	05953	Marcolândia	8.059
PI	22	06001	Marcos Parente	4.453
PI	22	06050	Massapê do Piauí	6.260
PI	22	06100	Matias Olímpio	10.586
PI	22	06209	Miguel Alves	32.658
PI	22	06308	Miguel Leão	1.236
PI	22	06357	Milton Brandão	6.750
PI	22	06407	Monsenhor Gil	10.337
PI	22	06506	Monsenhor Hipólito	7.486
PI	22	06605	Monte Alegre do Piauí	10.363
PI	22	06654	Morro Cabeça no Tempo	4.053
PI	22	06670	Morro do Chapéu do Piauí	6.574
PI	22	06696	Murici dos Portelas	8.714
PI	22	06704	Nazaré do Piauí	7.248
PI	22	06720	Nazária	8.227
PI	22	06753	Nossa Senhora de Nazaré	4.661
PI	22	06803	Nossa Senhora dos Remédios	8.356
PI	22	07959	Nova Santa Rita	4.233
PI	22	06902	Novo Oriente do Piauí	6.459
PI	22	06951	Novo Santo Antônio	3.329
PI	22	07009	Oeiras	35.931
PI	22	07108	Olho D'Água do Piauí	2.678
PI	22	07207	Padre Marcos	6.687
PI	22	07306	Paes Landim	4.049
PI	22	07355	Pajeú do Piauí	3.434
PI	22	07405	Palmeira do Piauí	4.962
PI	22	07504	Palmeirais	13.986
PI	22	07553	Paquetá	3.891
PI	22	07603	Parnaguá	10.417
PI	22	07702	Parnaíba	147.732
PI	22	07751	Passagem Franca do Piauí	4.424
PI	22	07777	Patos do Piauí	6.178
PI	22	07793	Pau D'Arco do Piauí	3.858
PI	22	07801	Paulistana	19.947

PI	22	07850	Pavussu	3.629
PI	22	07900	Pedro II	37.692
PI	22	07934	Pedro Laurentino	2.445
PI	22	08007	Picos	75.481
PI	22	08106	Pimenteiras	11.798
PI	22	08205	Pio IX	17.848
PI	22	08304	Piracuruca	27.971
PI	22	08403	Piripiri	62.088
PI	22	08502	Porto	12.097
PI	22	08551	Porto Alegre do Piauí	2.606
PI	22	08601	Prata do Piauí	3.088
PI	22	08650	Queimada Nova	8.679
PI	22	08700	Redenção do Gurguéia	8.494
PI	22	08809	Regeneração	17.569
PI	22	08858	Riacho Frio	4.229
PI	22	08874	Ribeira do Piauí	4.321
PI	22	08908	Ribeiro Gonçalves	7.015
PI	22	09005	Rio Grande do Piauí	6.282
PI	22	09104	Santa Cruz do Piauí	6.065
PI	22	09153	Santa Cruz dos Milagres	3.864
PI	22	09203	Santa Filomena	6.106
PI	22	09302	Santa Luz	5.624
PI	22	09377	Santa Rosa do Piauí	5.145
PI	22	09351	Santana do Piauí	4.489
PI	22	09401	Santo Antônio de Lisboa	6.136
PI	22	09450	Santo Antônio dos Milagres	2.087
PI	22	09500	Santo Inácio do Piauí	3.679
PI	22	09559	São Braz do Piauí	4.332
PI	22	09609	São Félix do Piauí	2.925
PI	22	09658	São Francisco de Assis do Piauí	5.686
PI	22	09708	São Francisco do Piauí	6.290
PI	22	09757	São Gonçalo do Gurguéia	2.901
PI	22	09807	São Gonçalo do Piauí	4.831
PI	22	09856	São João da Canabrava	4.476
PI	22	09872	São João da Fronteira	5.718
PI	22	09906	São João da Serra	6.079
PI	22	09955	São João da Varjota	4.693
PI	22	09971	São João do Arraial	7.578
PI	22	10003	São João do Piauí	19.852
PI	22	10052	São José do Divino	5.189
PI	22	10102	São José do Peixe	3.682
PI	22	10201	São José do Piauí	6.574
PI	22	10300	São Julião	5.719
PI	22	10359	São Lourenço do Piauí	4.451
PI	22	10375	São Luis do Piauí	2.573
PI	22	10383	São Miguel da Baixa Grande	2.386

PI	22	10391	São Miguel do Fidalgo	2.974
PI	22	10409	São Miguel do Tapuio	18.033
PI	22	10508	São Pedro do Piauí	13.810
PI	22	10607	São Raimundo Nonato	33.148
PI	22	10623	Sebastião Barros	3.475
PI	22	10631	Sebastião Leal	4.159
PI	22	10656	Sigefredo Pacheco	9.706
PI	22	10706	Simões	14.267
PI	22	10805	Simplício Mendes	12.251
PI	22	10904	Socorro do Piauí	4.495
PI	22	10938	Sussuapara	6.409
PI	22	10953	Tamboril do Piauí	2.805
PI	22	10979	Tanque do Piauí	2.663
PI	22	11001	Teresina	830.231
PI	22	11100	União	43.085
PI	22	11209	Uruçuí	20.623
PI	22	11308	Valença do Piauí	20.393
PI	22	11357	Várzea Branca	4.875
PI	22	11407	Várzea Grande	4.316
PI	22	11506	Vera Mendes	2.998
PI	22	11605	Vila Nova do Piauí	2.990
PI	22	11704	Wall Ferraz	4.323
CE	23	00101	Abaiara	10.815
CE	23	00150	Acarape	15.673
CE	23	00200	Acaraú	58.848
CE	23	00309	Acopiara	51.768
CE	23	00408	Aiuaba	16.468
CE	23	00507	Alcântaras	10.956
CE	23	00606	Altaneira	7.033
CE	23	00705	Alto Santo	16.505
CE	23	00754	Amontada	40.274
CE	23	00804	Antonina do Norte	7.056
CE	23	00903	Apuiarés	14.135
CE	23	01000	Aquiraz	74.465
CE	23	01109	Aracati	70.363
CE	23	01208	Aracoiaba	25.592
CE	23	01257	Ararendá	10.564
CE	23	01307	Araripe	20.848
CE	23	01406	Aratuba	11.404
CE	23	01505	Arneiroz	7.667
CE	23	01604	Assaré	22.633
CE	23	01703	Aurora	24.470
CE	23	01802	Baixio	6.072
CE	23	01851	Banabuiú	17.488
CE	23	01901	Barbalha	56.576
CE	23	01950	Barreira	19.958

CE	23	02008	Barro	21.742
CE	23	02057	Barroquinha	14.560
CE	23	02107	Baturité	33.863
CE	23	02206	Beberibe	50.364
CE	23	02305	Bela Cruz	31.259
CE	23	02404	Boa Viagem	52.829
CE	23	02503	Brejo Santo	46.207
CE	23	02602	Camocim	60.870
CE	23	02701	Campos Sales	26.648
CE	23	02800	Canindé	75.209
CE	23	02909	Capistrano	17.202
CE	23	03006	Caridade	20.687
CE	23	03105	Cariré	18.391
CE	23	03204	Caririaçu	26.471
CE	23	03303	Cariús	18.586
CE	23	03402	Carnaubal	16.975
CE	23	03501	Cascavel	67.503
CE	23	03600	Catarina	19.228
CE	23	03659	Catunda	10.053
CE	23	03709	Caucaia	336.091
CE	23	03808	Cedro	24.622
CE	23	03907	Chaval	12.684
CE	23	03931	Choró	12.982
CE	23	03956	Chorozinho	18.947
CE	23	04004	Coreaú	22.252
CE	23	04103	Crateús	73.102
CE	23	04202	Crato	123.963
CE	23	04236	Croatá	17.272
CE	23	04251	Cruz	22.887
CE	23	04269	Deputado Irapuan Pinheiro	9.203
CE	23	04277	Ererê	6.922
CE	23	04285	Eusébio	47.993
CE	23	04301	Farias Brito	18.859
CE	23	04350	Forquilha	22.435
CE	23	04400	Fortaleza	2.500.194
CE	23	04459	Fortim	15.233
CE	23	04509	Frecheirinha	13.167
CE	23	04608	General Sampaio	6.423
CE	23	04657	Graça	15.085
CE	23	04707	Granja	52.528
CE	23	04806	Granjeiro	4.551
CE	23	04905	Groaíras	10.445
CE	23	04954	Guaiúba	24.727
CE	23	05001	Guaraciaba do Norte	38.189
CE	23	05100	Guaramiranga	3.956
CE	23	05209	Hidrolândia	19.548

CE	23	05233	Horizonte	58.418
CE	23	05266	Ibaretama	12.977
CE	23	05308	Ibiapina	24.058
CE	23	05332	Ibicuitinga	11.622
CE	23	05357	Icapuí	18.746
CE	23	05407	Icó	65.900
CE	23	05506	Iguatu	98.138
CE	23	05605	Independência	25.620
CE	23	05654	Ipaporanga	11.358
CE	23	05704	Ipaumirim	12.080
CE	23	05803	Ipu	40.579
CE	23	05902	Ipueiras	37.758
CE	23	06009	Iracema	13.808
CE	23	06108	Irauçuba	22.742
CE	23	06207	Itaiçaba	7.428
CE	23	06256	Itaitinga	36.814
CE	23	06306	Itapagé	49.130
CE	23	06405	Itapipoca	119.320
CE	23	06504	Itapiúna	19.009
CE	23	06553	Itarema	38.547
CE	23	06603	Itatira	19.401
CE	23	06702	Jaguaretama	17.839
CE	23	06801	Jaguaribara	10.652
CE	23	06900	Jaguaribe	34.317
CE	23	07007	Jaguaruana	32.614
CE	23	07106	Jardim	26.730
CE	23	07205	Jati	7.647
CE	23	07254	Jijoca de Jericoacoara	17.744
CE	23	07304	Juazeiro do Norte	255.648
CE	23	07403	Jucás	23.985
CE	23	07502	Lavras da Mangabeira	31.073
CE	23	07601	Limoeiro do Norte	56.255
CE	23	07635	Madalena	18.575
CE	23	07650	Maracanaú	213.404
CE	23	07700	Maranguape	117.306
CE	23	07809	Marco	25.349
CE	23	07908	Martinópole	10.458
CE	23	08005	Massapê	36.040
CE	23	08104	Mauriti	44.836
CE	23	08203	Meruoca	14.049
CE	23	08302	Milagres	28.204
CE	23	08351	Milhã	13.062
CE	23	08377	Miraíma	13.009
CE	23	08401	Missão Velha	34.529
CE	23	08500	Mombaça	42.891
CE	23	08609	Monsenhor Tabosa	16.760

CE	23	08708	Morada Nova	61.713
CE	23	08807	Moraújo	8.225
CE	23	08906	Morrinhos	21.119
CE	23	09003	Mucambo	14.146
CE	23	09102	Mulungu	11.876
CE	23	09201	Nova Olinda	14.586
CE	23	09300	Nova Russas	31.210
CE	23	09409	Novo Oriente	27.655
CE	23	09458	Ocara	24.373
CE	23	09508	Orós	21.294
CE	23	09607	Pacajus	64.521
CE	23	09706	Pacatuba	75.411
CE	23	09805	Pacoti	11.684
CE	23	09904	Pacujá	6.037
CE	23	10001	Palhano	8.972
CE	23	10100	Palmácia	12.330
CE	23	10209	Paracuru	32.255
CE	23	10258	Paraipaba	30.733
CE	23	10308	Parambu	31.160
CE	23	10407	Paramoti	11.360
CE	23	10506	Pedra Branca	42.064
CE	23	10605	Penaforte	8.483
CE	23	10704	Pentecoste	35.823
CE	23	10803	Pereiro	15.838
CE	23	10852	Pindoretama	19.247
CE	23	10902	Piquet Carneiro	15.820
CE	23	10951	Pires Ferreira	10.365
CE	23	11009	Poranga	12.041
CE	23	11108	Porteiras	14.971
CE	23	11207	Potengi	10.448
CE	23	11231	Potiretama	6.181
CE	23	11264	Quiterianópolis	20.158
CE	23	11306	Quixadá	82.258
CE	23	11355	Quixelô	14.911
CE	23	11405	Quixeramobim	73.812
CE	23	11504	Quixeré	20.810
CE	23	11603	Redenção	26.660
CE	23	11702	Reriutaba	19.179
CE	23	11801	Russas	71.723
CE	23	11900	Saboeiro	15.681
CE	23	11959	Salitre	15.684
CE	23	12205	Santa Quitéria	42.822
CE	23	12007	Santana do Acaraú	30.512
CE	23	12106	Santana do Cariri	17.219
CE	23	12304	São Benedito	44.825
CE	23	12403	São Gonçalo do Amarante	45.141

CE	23	12502	São João do Jaguaribe	7.788
CE	23	12601	São Luís do Curu	12.459
CE	23	12700	Senador Pompeu	26.382
CE	23	12809	Senador Sá	7.041
CE	23	12908	Sobral	193.134
CE	23	13005	Solonópole	17.768
CE	23	13104	Tabuleiro do Norte	29.522
CE	23	13203	Tamboril	25.397
CE	23	13252	Tarrafas	8.865
CE	23	13302	Tauá	56.307
CE	23	13351	Tejuçuoca	17.643
CE	23	13401	Tianguá	70.527
CE	23	13500	Trairi	52.464
CE	23	13559	Tururu	14.848
CE	23	13609	Ubajara	32.496
CE	23	13708	Umari	7.562
CE	23	13757	Umirim	19.023
CE	23	13807	Uruburetama	20.289
CE	23	13906	Uruoca	13.096
CE	23	13955	Varjota	17.745
CE	23	14003	Várzea Alegre	38.952
CE	23	14102	Viçosa do Ceará	56.394
RN	24	00109	Acari	11.012
RN	24	00208	Açu	54.031
RN	24	00307	Afonso Bezerra	10.841
RN	24	00406	Água Nova	3.026
RN	24	00505	Alexandria	13.467
RN	24	00604	Almino Afonso	4.823
RN	24	00703	Alto do Rodrigues	12.729
RN	24	00802	Angicos	11.538
RN	24	00901	Antônio Martins	6.930
RN	24	01008	Apodi	34.852
RN	24	01107	Areia Branca	25.736
RN	24	01206	Arês	13.166
RN	24	01305	Augusto Severo	9.330
RN	24	01404	Baía Formosa	8.687
RN	24	01453	Baraúna	24.977
RN	24	01503	Barcelona	3.944
RN	24	01602	Bento Fernandes	5.175
RN	24	01651	Bodó	2.373
RN	24	01701	Bom Jesus	9.566
RN	24	01800	Brejinho	11.769
RN	24	01859	Caiçara do Norte	6.043
RN	24	01909	Caiçara do Rio do Vento	3.375
RN	24	02006	Caicó	63.571
RN	24	02105	Campo Redondo	10.427

RN	24	02204	Canguaretama	31.506
RN	24	02303	Caraúbas	19.692
RN	24	02402	Carnaúba dos Dantas	7.559
RN	24	02501	Carnaubais	10.000
RN	24	02600	Ceará-Mirim	69.005
RN	24	02709	Cerro Corá	10.928
RN	24	02808	Coronel Ezequiel	5.405
RN	24	02907	Coronel João Pessoa	4.783
RN	24	03004	Cruzeta	7.942
RN	24	03103	Currais Novos	42.934
RN	24	03202	Doutor Severiano	6.954
RN	24	03301	Encanto	5.297
RN	24	03400	Equador	5.846
RN	24	03509	Espírito Santo	10.439
RN	24	03608	Extremoz	25.324
RN	24	03707	Felipe Guerra	5.765
RN	24	03756	Fernando Pedroza	2.885
RN	24	03806	Florânia	8.957
RN	24	03905	Francisco Dantas	2.852
RN	24	04002	Frutuoso Gomes	4.181
RN	24	04101	Galinhos	2.284
RN	24	04200	Goianinha	23.209
RN	24	04309	Governador Dix-Sept Rosado	12.465
RN	24	04408	Grossos	9.566
RN	24	04507	Guamaré	13.047
RN	24	04606	Ielmo Marinho	12.462
RN	24	04705	Ipanguaçu	14.148
RN	24	04804	Ipueira	2.104
RN	24	04853	Itajá	7.036
RN	24	04903	Itaú	5.609
RN	24	05009	Jaçanã	8.150
RN	24	05108	Jandaíra	6.838
RN	24	05207	Janduís	5.307
RN	24	05306	Januário Cicco	9.211
RN	24	05405	Japi	5.401
RN	24	05504	Jardim de Angicos	2.598
RN	24	05603	Jardim de Piranhas	13.735
RN	24	05702	Jardim do Seridó	12.124
RN	24	05801	João Câmara	32.677
RN	24	05900	João Dias	2.602
RN	24	06007	José da Penha	5.862
RN	24	06106	Jucurutu	17.749
RN	24	06155	Jundiá	3.635
RN	24	06205	Lagoa d'Anta	6.318
RN	24	06304	Lagoa de Pedras	7.079
RN	24	06403	Lagoa de Velhos	2.671

RN	24	06502	Lagoa Nova	14.274
RN	24	06601	Lagoa Salgada	7.679
RN	24	06700	Lajes	10.530
RN	24	06809	Lajes Pintadas	4.625
RN	24	06908	Lucrécia	3.696
RN	24	07005	Luís Gomes	9.679
RN	24	07104	Macaíba	71.670
RN	24	07203	Macau	29.446
RN	24	07252	Major Sales	3.625
RN	24	07302	Marcelino Vieira	8.249
RN	24	07401	Martins	8.293
RN	24	07500	Maxaranguape	10.810
RN	24	07609	Messias Targino	4.259
RN	24	07708	Montanhas	11.333
RN	24	07807	Monte Alegre	20.959
RN	24	07906	Monte das Gameleiras	2.219
RN	24	08003	Mossoró	266.758
RN	24	08102	Natal	817.590
RN	24	08201	Nísia Floresta	24.501
RN	24	08300	Nova Cruz	35.741
RN	24	08409	Olho-d'Água do Borges	4.270
RN	24	08508	Ouro Branco	4.704
RN	24	08607	Paraná	4.001
RN	24	08706	Paraú	3.824
RN	24	08805	Parazinho	4.924
RN	24	08904	Parelhas	20.511
RN	24	03251	Parnamirim	214.199
RN	24	09100	Passa e Fica	11.519
RN	24	09209	Passagem	2.925
RN	24	09308	Patu	12.084
RN	24	09407	Pau dos Ferros	28.197
RN	24	09506	Pedra Grande	3.447
RN	24	09605	Pedra Preta	2.552
RN	24	09704	Pedro Avelino	7.045
RN	24	09803	Pedro Velho	14.204
RN	24	09902	Pendências	13.739
RN	24	10009	Pilões	3.522
RN	24	10108	Poço Branco	14.204
RN	24	10207	Portalegre	7.407
RN	24	10256	Porto do Mangue	5.392
RN	24	10306	Presidente Juscelino	9.035
RN	24	10405	Pureza	8.645
RN	24	10504	Rafael Fernandes	4.760
RN	24	10603	Rafael Godeiro	3.080
RN	24	10702	Riacho da Cruz	3.241
RN	24	10801	Riacho de Santana	4.150

RN	24	10900	Riachuelo	7.265
RN	24	08953	Rio do Fogo	10.187
RN	24	11007	Rodolfo Fernandes	4.411
RN	24	11106	Ruy Barbosa	3.582
RN	24	11205	Santa Cruz	36.477
RN	24	09332	Santa Maria	4.911
RN	24	11403	Santana do Matos	13.481
RN	24	11429	Santana do Seridó	2.549
RN	24	11502	Santo Antônio	22.535
RN	24	11601	São Bento do Norte	2.915
RN	24	11700	São Bento do Trairí	4.005
RN	24	11809	São Fernando	3.427
RN	24	11908	São Francisco do Oeste	3.934
RN	24	12005	São Gonçalo do Amarante	90.376
RN	24	12104	São João do Sabugi	5.956
RN	24	12203	São José de Mipibu	40.511
RN	24	12302	São José do Campestre	12.413
RN	24	12401	São José do Seridó	4.300
RN	24	12500	São Miguel	21.994
RN	24	12559	São Miguel do Gostoso	8.835
RN	24	12609	São Paulo do Potengi	16.149
RN	24	12708	São Pedro	6.154
RN	24	12807	São Rafael	8.098
RN	24	12906	São Tomé	10.832
RN	24	13003	São Vicente	6.088
RN	24	13102	Senador Elói de Souza	5.729
RN	24	13201	Senador Georgino Avelino	4.018
RN	24	13300	Serra de São Bento	5.724
RN	24	13359	Serra do Mel	10.597
RN	24	13409	Serra Negra do Norte	7.805
RN	24	13508	Serrinha	6.480
RN	24	13557	Serrinha dos Pintos	4.577
RN	24	13607	Severiano Melo	5.848
RN	24	13706	Sítio Novo	5.107
RN	24	13805	Taboleiro Grande	2.361
RN	24	13904	Taipu	11.883
RN	24	14001	Tangará	14.486
RN	24	14100	Tenente Ananias	10.036
RN	24	14159	Tenente Laurentino Cruz	5.557
RN	24	11056	Tibau	3.761
RN	24	14209	Tibau do Sul	11.935
RN	24	14308	Timbaúba dos Batistas	2.312
RN	24	14407	Touros	31.574
RN	24	14456	Triunfo Potiguar	3.327
RN	24	14506	Umarizal	10.594
RN	24	14605	Upanema	13.295

RN	24	14704	Várzea	5.271
RN	24	14753	Venha-Ver	3.882
RN	24	14803	Vera Cruz	11.051
RN	24	14902	Viçosa	1.633
RN	24	15008	Vila Flor	2.924
PB	25	00106	Água Branca	9.611
PB	25	00205	Aguiar	5.514
PB	25	00304	Alagoa Grande	28.375
PB	25	00403	Alagoa Nova	19.849
PB	25	00502	Alagoinha	13.740
PB	25	00536	Alcantil	5.282
PB	25	00577	Algodão de Jandaíra	2.390
PB	25	00601	Alhandra	18.324
PB	25	00734	Amparo	2.119
PB	25	00775	Aparecida	7.832
PB	25	00809	Araçagi	17.093
PB	25	00908	Arara	12.820
PB	25	01005	Araruna	19.076
PB	25	01104	Areia	23.391
PB	25	01153	Areia de Baraúnas	1.901
PB	25	01203	Areial	6.536
PB	25	01302	Aroeiras	19.016
PB	25	01351	Assunção	3.607
PB	25	01401	Baía da Traição	8.243
PB	25	01500	Bananeiras	21.753
PB	25	01534	Baraúna	4.379
PB	25	01609	Barra de Santa Rosa	14.413
PB	25	01575	Barra de Santana	8.191
PB	25	01708	Barra de São Miguel	5.679
PB	25	01807	Bayeux	100.543
PB	25	01906	Belém	17.167
PB	25	02003	Belém do Brejo do Cruz	7.163
PB	25	02052	Bernardino Batista	3.153
PB	25	02102	Boa Ventura	5.625
PB	25	02151	Boa Vista	6.415
PB	25	02201	Bom Jesus	2.432
PB	25	02300	Bom Sucesso	4.998
PB	25	02409	Bonito de Santa Fé	11.042
PB	25	02508	Boqueirão	17.043
PB	25	02706	Borborema	5.169
PB	25	02805	Brejo do Cruz	13.313
PB	25	02904	Brejo dos Santos	6.236
PB	25	03001	Caaporã	20.653
PB	25	03100	Cabaceiras	5.148
PB	25	03209	Cabedelo	60.226
PB	25	03308	Cachoeira dos Índios	9.685

PB	25	03407	Cacimba de Areia	3.590
PB	25	03506	Cacimba de Dentro	16.885
PB	25	03555	Cacimbas	6.877
PB	25	03605	Caiçara	7.205
PB	25	03704	Cajazeiras	59.130
PB	25	03753	Cajazeirinhas	3.061
PB	25	03803	Caldas Brandão	5.710
PB	25	03902	Camalaú	5.793
PB	25	04009	Campina Grande	389.995
PB	25	04033	Capim	5.816
PB	25	04074	Caraúbas	3.951
PB	25	04108	Carrapateira	2.441
PB	25	04157	Casserengue	7.132
PB	25	04207	Catingueira	4.822
PB	25	04306	Catolé do Rocha	29.079
PB	25	04355	Caturité	4.598
PB	25	04405	Conceição	18.429
PB	25	04504	Condado	6.598
PB	25	04603	Conde	22.154
PB	25	04702	Congo	4.692
PB	25	04801	Coremas	15.152
PB	25	04850	Coxixola	1.802
PB	25	04900	Cruz do Espírito Santo	16.317
PB	25	05006	Cubati	6.939
PB	25	05105	Cuité	19.983
PB	25	05238	Cuité de Mamanguape	6.214
PB	25	05204	Cuitegi	6.834
PB	25	05279	Curral de Cima	5.192
PB	25	05303	Curral Velho	2.497
PB	25	05352	Damião	4.990
PB	25	05402	Desterro	8.035
PB	25	05600	Diamante	6.571
PB	25	05709	Dona Inês	10.438
PB	25	05808	Duas Estradas	3.611
PB	25	05907	Emas	3.356
PB	25	06004	Esperança	31.538
PB	25	06103	Fagundes	11.332
PB	25	06202	Frei Martinho	2.935
PB	25	06251	Gado Bravo	8.355
PB	25	06301	Guarabira	55.977
PB	25	06400	Gurinhém	13.877
PB	25	06509	Gurjão	3.215
PB	25	06608	Ibiara	5.978
PB	25	02607	Igaracy	6.134
PB	25	06707	Imaculada	11.423
PB	25	06806	Ingá	17.555

PB	25	06905	Itabaiana	24.372
PB	25	07002	Itaporanga	23.505
PB	25	07101	Itapororoca	17.354
PB	25	07200	Itatuba	10.326
PB	25	07309	Jacaraú	13.991
PB	25	07408	Jericó	7.557
PB	25	07507	João Pessoa	742.478
PB	25	13653	Joca Claudino	2.623
PB	25	07606	Juarez Távora	7.550
PB	25	07705	Juazeirinho	17.064
PB	25	07804	Junco do Seridó	6.745
PB	25	07903	Juripiranga	10.327
PB	25	08000	Juru	9.793
PB	25	08109	Lagoa	4.657
PB	25	08208	Lagoa de Dentro	7.413
PB	25	08307	Lagoa Seca	26.164
PB	25	08406	Lastro	2.800
PB	25	08505	Livramento	7.189
PB	25	08554	Logradouro	4.026
PB	25	08604	Lucena	12.029
PB	25	08703	Mãe d'Água	3.999
PB	25	08802	Malta	5.602
PB	25	08901	Mamanguape	42.537
PB	25	09008	Manaíra	10.803
PB	25	09057	Marcação	7.822
PB	25	09107	Mari	21.254
PB	25	09156	Marizópolis	6.257
PB	25	09206	Massaranduba	13.084
PB	25	09305	Mataraca	7.641
PB	25	09339	Matinhas	4.357
PB	25	09370	Mato Grosso	2.744
PB	25	09396	Maturéia	6.076
PB	25	09404	Mogeiro	13.178
PB	25	09503	Montadas	5.145
PB	25	09602	Monte Horebe	4.568
PB	25	09701	Monteiro	31.330
PB	25	09800	Mulungu	9.542
PB	25	09909	Natuba	10.278
PB	25	10006	Nazarezinho	7.252
PB	25	10105	Nova Floresta	10.514
PB	25	10204	Nova Olinda	6.012
PB	25	10303	Nova Palmeira	4.480
PB	25	10402	Olho d'Água	6.796
PB	25	10501	Olivedos	3.693
PB	25	10600	Ouro Velho	2.944
PB	25	10659	Parari	1.816

PB	25	10709	Passagem	2.272
PB	25	10808	Patos	102.020
PB	25	10907	Paulista	11.867
PB	25	11004	Pedra Branca	3.726
PB	25	11103	Pedra Lavrada	7.605
PB	25	11202	Pedras de Fogo	27.479
PB	25	12721	Pedro Régis	5.824
PB	25	11301	Piancó	15.555
PB	25	11400	Picuí	18.272
PB	25	11509	Pilar	11.330
PB	25	11608	Pilões	6.854
PB	25	11707	Pilõezinhos	5.114
PB	25	11806	Pirpirituba	10.346
PB	25	11905	Pitimbu	17.492
PB	25	12002	Pocinhos	17.357
PB	25	12036	Poço Dantas	3.740
PB	25	12077	Poço de José de Moura	4.046
PB	25	12101	Pombal	32.134
PB	25	12200	Prata	3.919
PB	25	12309	Princesa Isabel	21.744
PB	25	12408	Puxinanã	13.066
PB	25	12507	Queimadas	41.538
PB	25	12606	Quixabá	1.759
PB	25	12705	Remígio	18.075
PB	25	12747	Riachão	3.338
PB	25	12754	Riachão do Bacamarte	4.312
PB	25	12762	Riachão do Poço	4.235
PB	25	12788	Riacho de Santo Antônio	1.781
PB	25	12804	Riacho dos Cavalos	8.352
PB	25	12903	Rio Tinto	23.431
PB	25	13000	Salgadinho	3.612
PB	25	13109	Salgado de São Félix	11.966
PB	25	13158	Santa Cecília	6.533
PB	25	13208	Santa Cruz	6.471
PB	25	13307	Santa Helena	5.886
PB	25	13356	Santa Inês	3.538
PB	25	13406	Santa Luzia	14.826
PB	25	13703	Santa Rita	121.994
PB	25	13802	Santa Teresinha	4.559
PB	25	13505	Santana de Mangueira	5.265
PB	25	13604	Santana dos Garrotes	7.173
PB	25	13851	Santo André	2.545
PB	25	13927	São Bentinho	4.221
PB	25	13901	São Bento	31.582
PB	25	13968	São Domingos de Pombal	2.909
PB	25	13943	São Domingos do Cariri	2.455

PB	25	13984	São Francisco	3.349
PB	25	14008	São João do Cariri	4.309
PB	25	00700	São João do Rio do Peixe	17.646
PB	25	14107	São João do Tigre	4.384
PB	25	14206	São José da Lagoa Tapada	7.560
PB	25	14305	São José de Caiana	6.052
PB	25	14404	São José de Espinharas	4.708
PB	25	14503	São José de Piranhas	19.281
PB	25	14552	São José de Princesa	4.106
PB	25	14602	São José do Bonfim	3.303
PB	25	14651	São José do Brejo do Cruz	1.707
PB	25	14701	São José do Sabugi	4.027
PB	25	14800	São José dos Cordeiros	3.709
PB	25	14453	São José dos Ramos	5.600
PB	25	14909	São Mamede	7.708
PB	25	15005	São Miguel de Taipu	6.789
PB	25	15104	São Sebastião de Lagoa de Roça	11.195
PB	25	15203	São Sebastião do Umbuzeiro	3.287
PB	25	15302	Sapé	50.565
PB	25	15401	Seridó	10.400
PB	25	15500	Serra Branca	13.101
PB	25	15609	Serra da Raiz	3.169
PB	25	15708	Serra Grande	2.994
PB	25	15807	Serra Redonda	7.012
PB	25	15906	Serraria	6.175
PB	25	15930	Sertãozinho	4.539
PB	25	15971	Sobrado	7.447
PB	25	16003	Solânea	26.323
PB	25	16102	Soledade	13.993
PB	25	16151	Sossêgo	3.256
PB	25	16201	Sousa	66.457
PB	25	16300	Sumé	16.215
PB	25	16409	Tacima	10.394
PB	25	16508	Taperoá	14.833
PB	25	16607	Tavares	14.182
PB	25	16706	Teixeira	14.352
PB	25	16755	Tenório	2.865
PB	25	16805	Triunfo	9.246
PB	25	16904	Uiraúna	14.721
PB	25	17001	Umbuzeiro	9.698
PB	25	17100	Várzea	2.573
PB	25	17209	Vieirópolis	5.102
PB	25	05501	Vista Serrana	3.572
PB	25	17407	Zabelê	2.109
PE	26	00054	Abreu e Lima	95.243
PE	26	00104	Afogados da Ingazeira	35.416

PE	26	00203	Afrânio	17.975
PE	26	00302	Agrestina	23.079
PE	26	00401	Água Preta	33.785
PE	26	00500	Águas Belas	40.778
PE	26	00609	Alagoinha	13.741
PE	26	00708	Aliança	37.450
PE	26	00807	Altinho	22.371
PE	26	00906	Amaraji	22.035
PE	26	01003	Angelim	10.372
PE	26	01052	Araçoiaba	18.617
PE	26	01102	Araripina	78.270
PE	26	01201	Arcoverde	69.880
PE	26	01300	Barra de Guabiraba	13.054
PE	26	01409	Barreiros	40.973
PE	26	01508	Belém de Maria	11.463
PE	26	01607	Belém de São Francisco	20.260
PE	26	01706	Belo Jardim	72.996
PE	26	01805	Betânia	12.109
PE	26	01904	Bezerros	58.864
PE	26	02001	Bodocó	35.676
PE	26	02100	Bom Conselho	45.983
PE	26	02209	Bom Jardim	37.949
PE	26	02308	Bonito	37.539
PE	26	02407	Brejão	8.834
PE	26	02506	Brejinho	7.312
PE	26	02605	Brejo da Madre de Deus	46.248
PE	26	02704	Buenos Aires	12.618
PE	26	02803	Buíque	53.304
PE	26	02902	Cabo de Santo Agostinho	189.222
PE	26	03009	Cabrobó	31.497
PE	26	03108	Cachoeirinha	19.088
PE	26	03207	Caetés	26.946
PE	26	03306	Calçado	11.051
PE	26	03405	Calumbi	5.643
PE	26	03454	Camaragibe	146.847
PE	26	03504	Camocim de São Félix	17.405
PE	26	03603	Camutanga	8.204
PE	26	03702	Canhotinho	24.461
PE	26	03801	Capoeiras	19.599
PE	26	03900	Carnaíba	18.707
PE	26	03926	Carnaubeira da Penha	11.991
PE	26	04007	Carpina	76.527
PE	26	04106	Caruaru	324.095
PE	26	04155	Casinhas	13.830
PE	26	04205	Catende	38.812
PE	26	04304	Cedro	10.964

PE	26	04403	Chã de Alegria	12.601
PE	26	04502	Chã Grande	20.399
PE	26	04601	Condado	24.658
PE	26	04700	Correntes	17.374
PE	26	04809	Cortês	12.418
PE	26	04908	Cumaru	17.470
PE	26	05004	Cupira	23.114
PE	26	05103	Custódia	34.442
PE	26	05152	Dormentes	17.296
PE	26	05202	Escada	64.422
PE	26	05301	Exu	31.518
PE	26	05400	Feira Nova	20.830
PE	26	05459	Fernando de Noronha	2.718
PE	26	05509	Ferreiros	11.537
PE	26	05608	Flores	22.162
PE	26	05707	Floresta	29.973
PE	26	05806	Frei Miguelinho	14.492
PE	26	05905	Gameleira	28.503
PE	26	06002	Garanhuns	131.169
PE	26	06101	Glória do Goitá	29.241
PE	26	06200	Goiana	75.902
PE	26	06309	Granito	6.968
PE	26	06408	Gravatá	77.845
PE	26	06507	Iati	18.462
PE	26	06606	Ibimirim	27.349
PE	26	06705	Ibirajuba	7.549
PE	26	06804	Igarassu	105.003
PE	26	06903	Iguaraci	11.824
PE	26	07604	Ilha de Itamaracá	22.794
PE	26	07000	Inajá	19.957
PE	26	07109	Ingazeira	4.486
PE	26	07208	Ipojuca	83.862
PE	26	07307	Ipubi	28.887
PE	26	07406	Itacuruba	4.475
PE	26	07505	Itaíba	26.175
PE	26	07653	Itambé	35.461
PE	26	07703	Itapetim	13.748
PE	26	07752	Itapissuma	24.321
PE	26	07802	Itaquitinga	16.221
PE	26	07901	Jaboatão dos Guararapes	654.786
PE	26	07950	Jaqueira	11.479
PE	26	08008	Jataúba	16.219
PE	26	08057	Jatobá	14.087
PE	26	08107	João Alfredo	31.305
PE	26	08206	Joaquim Nabuco	15.751
PE	26	08255	Jucati	10.742

PE	26	08305	Jupi	13.899
PE	26	08404	Jurema	14.662
PE	26	08453	Lagoa do Carro	16.408
PE	26	08503	Lagoa do Itaenga	20.733
PE	26	08602	Lagoa do Ouro	12.307
PE	26	08701	Lagoa dos Gatos	15.731
PE	26	08750	Lagoa Grande	23.308
PE	26	08800	Lajedo	37.296
PE	26	08909	Limoeiro	55.343
PE	26	09006	Macaparana	24.142
PE	26	09105	Machados	14.109
PE	26	09154	Manari	18.847
PE	26	09204	Maraial	11.961
PE	26	09303	Mirandiba	14.488
PE	26	14303	Moreilândia	11.020
PE	26	09402	Moreno	57.828
PE	26	09501	Nazaré da Mata	31.029
PE	26	09600	Olinda	379.271
PE	26	09709	Orobó	22.996
PE	26	09808	Orocó	13.536
PE	26	09907	Ouricuri	65.510
PE	26	10004	Palmares	60.091
PE	26	10103	Palmeirina	8.172
PE	26	10202	Panelas	26.005
PE	26	10301	Paranatama	11.100
PE	26	10400	Parnamirim	20.425
PE	26	10509	Passira	28.552
PE	26	10608	Paudalho	52.297
PE	26	10707	Paulista	306.239
PE	26	10806	Pedra	21.050
PE	26	10905	Pesqueira	63.519
PE	26	11002	Petrolândia	33.273
PE	26	11101	Petrolina	305.352
PE	26	11200	Poção	11.029
PE	26	11309	Pombos	26.086
PE	26	11408	Primavera	13.705
PE	26	11507	Quipapá	24.495
PE	26	11533	Quixaba	6.722
PE	26	11606	Recife	1.555.039
PE	26	11705	Riacho das Almas	19.387
PE	26	11804	Ribeirão	44.950
PE	26	11903	Rio Formoso	22.361
PE	26	12000	Sairé	10.877
PE	26	12109	Salgadinho	9.641
PE	26	12208	Salgueiro	57.343
PE	26	12307	Saloá	15.355

PE	26	12406	Sanharó	22.896
PE	26	12455	Santa Cruz	13.946
PE	26	12471	Santa Cruz da Baixa Verde	11.901
PE	26	12505	Santa Cruz do Capibaribe	91.891
PE	26	12554	Santa Filomena	13.561
PE	26	12604	Santa Maria da Boa Vista	39.816
PE	26	12703	Santa Maria do Cambucá	13.215
PE	26	12802	Santa Terezinha	11.103
PE	26	12901	São Benedito do Sul	14.326
PE	26	13008	São Bento do Una	54.433
PE	26	13107	São Caitano	35.554
PE	26	13206	São João	21.549
PE	26	13305	São Joaquim do Monte	20.586
PE	26	13404	São José da Coroa Grande	18.816
PE	26	13503	São José do Belmonte	32.763
PE	26	13602	São José do Egito	32.186
PE	26	13701	São Lourenço da Mata	104.782
PE	26	13800	São Vicente Ferrer	17.151
PE	26	13909	Serra Talhada	80.489
PE	26	14006	Serrita	18.519
PE	26	14105	Sertânia	34.109
PE	26	14204	Sirinhaém	41.391
PE	26	14402	Solidão	5.777
PE	26	14501	Surubim	59.751
PE	26	14600	Tabira	26.784
PE	26	14709	Tacaimbó	12.695
PE	26	14808	Tacaratu	22.819
PE	26	14857	Tamandaré	21.234
PE	26	15003	Taquaritinga do Norte	25.681
PE	26	15102	Terezinha	6.803
PE	26	15201	Terra Nova	9.534
PE	26	15300	Timbaúba	53.360
PE	26	15409	Toritama	37.631
PE	26	15508	Tracunhaém	13.155
PE	26	15607	Trindade	26.749
PE	26	15706	Triunfo	14.987
PE	26	15805	Tupanatinga	24.973
PE	26	15904	Tuparetama	7.950
PE	26	16001	Venturosa	16.823
PE	26	16100	Verdejante	9.187
PE	26	16183	Vertente do Lério	7.773
PE	26	16209	Vertentes	18.716
PE	26	16308	Vicência	31.021
PE	26	16407	Vitória de Santo Antão	129.907
PE	26	16506	Xexéu	14.168
AL	27	00102	Água Branca	19.763

AL	27	00201	Anadia	17.360
AL	27	00300	Arapiraca	218.140
AL	27	00409	Atalaia	44.892
AL	27	00508	Barra de Santo Antônio	14.665
AL	27	00607	Barra de São Miguel	7.755
AL	27	00706	Batalha	17.420
AL	27	00805	Belém	4.635
AL	27	00904	Belo Monte	6.499
AL	27	01001	Boca da Mata	26.010
AL	27	01100	Branquinha	10.471
AL	27	01209	Cacimbinhas	10.307
AL	27	01308	Cajueiro	20.626
AL	27	01357	Campestre	6.655
AL	27	01407	Campo Alegre	52.327
AL	27	01506	Campo Grande	9.273
AL	27	01605	Canapi	17.238
AL	27	01704	Capela	16.728
AL	27	01803	Carneiros	8.548
AL	27	01902	Chã Preta	7.146
AL	27	02009	Coité do Nóia	10.765
AL	27	02108	Colônia Leopoldina	20.401
AL	27	02207	Coqueiro Seco	5.586
AL	27	02306	Coruripe	53.224
AL	27	02355	Craíbas	22.921
AL	27	02405	Delmiro Gouveia	48.876
AL	27	02504	Dois Riachos	10.838
AL	27	02553	Estrela de Alagoas	17.410
AL	27	02603	Feira Grande	21.342
AL	27	02702	Feliz Deserto	4.482
AL	27	02801	Flexeiras	12.378
AL	27	02900	Girau do Ponciano	37.858
AL	27	03007	Ibateguara	15.180
AL	27	03106	Igaci	25.129
AL	27	03205	Igreja Nova	23.570
AL	27	03304	Inhapi	17.839
AL	27	03403	Jacaré dos Homens	5.352
AL	27	03502	Jacuípe	6.950
AL	27	03601	Japaratinga	7.888
AL	27	03700	Jaramataia	5.524
AL	27	03759	Jequiá da Praia	11.887
AL	27	03809	Joaquim Gomes	22.853
AL	27	03908	Jundiá	4.142
AL	27	04005	Junqueiro	24.173
AL	27	04104	Lagoa da Canoa	17.988
AL	27	04203	Limoeiro de Anadia	27.069
AL	27	04302	Maceió	953.393

AL	27	04401	Major Isidoro	19.087
AL	27	04906	Mar Vermelho	3.588
AL	27	04500	Maragogi	29.794
AL	27	04609	Maravilha	9.981
AL	27	04708	Marechal Deodoro	47.504
AL	27	04807	Maribondo	13.389
AL	27	05002	Mata Grande	24.449
AL	27	05101	Matriz de Camaragibe	23.750
AL	27	05200	Messias	16.292
AL	27	05309	Minador do Negrão	5.251
AL	27	05408	Monteirópolis	6.952
AL	27	05507	Murici	27.030
AL	27	05606	Novo Lino	12.303
AL	27	05705	Olho d'Água das Flores	20.460
AL	27	05804	Olho d'Água do Casado	8.708
AL	27	05903	Olho d'Água Grande	4.967
AL	27	06000	Olivença	11.150
AL	27	06109	Ouro Branco	10.953
AL	27	06208	Palestina	5.201
AL	27	06307	Palmeira dos Índios	70.738
AL	27	06406	Pão de Açúcar	23.651
AL	27	06422	Pariconha	10.282
AL	27	06448	Paripueira	11.845
AL	27	06505	Passo de Camaragibe	14.802
AL	27	06604	Paulo Jacinto	7.412
AL	27	06703	Penedo	60.890
AL	27	06802	Piaçabuçu	17.268
AL	27	06901	Pilar	33.623
AL	27	07008	Pindoba	2.857
AL	27	07107	Piranhas	23.504
AL	27	07206	Poço das Trincheiras	13.845
AL	27	07305	Porto Calvo	25.974
AL	27	07404	Porto de Pedras	8.156
AL	27	07503	Porto Real do Colégio	19.288
AL	27	07602	Quebrangulo	11.330
AL	27	07701	Rio Largo	68.952
AL	27	07800	Roteiro	6.607
AL	27	07909	Santa Luzia do Norte	6.967
AL	27	08006	Santana do Ipanema	45.453
AL	27	08105	Santana do Mundaú	10.792
AL	27	08204	São Brás	6.744
AL	27	08303	São José da Laje	22.906
AL	27	08402	São José da Tapera	30.549
AL	27	08501	São Luís do Quitunde	32.846
AL	27	08600	São Miguel dos Campos	56.319
AL	27	08709	São Miguel dos Milagres	7.360

AL	27	08808	São Sebastião	32.446
AL	27	08907	Satuba	15.020
AL	27	08956	Senador Rui Palmeira	13.209
AL	27	09004	Tanque d'Arca	6.172
AL	27	09103	Taquarana	18.907
AL	27	09152	Teotônio Vilela	41.797
AL	27	09202	Traipu	26.369
AL	27	09301	União dos Palmares	62.923
AL	27	09400	Viçosa	25.384
SE	28	00100	Amparo de São Francisco	2.290
SE	28	00209	Aquidabã	20.315
SE	28	00308	Aracaju	587.701
SE	28	00407	Arauá	9.495
SE	28	00506	Areia Branca	17.164
SE	28	00605	Barra dos Coqueiros	26.059
SE	28	00670	Boquim	25.727
SE	28	00704	Brejo Grande	7.839
SE	28	01009	Campo do Brito	16.987
SE	28	01108	Canhoba	3.955
SE	28	01207	Canindé de São Francisco	25.733
SE	28	01306	Capela	31.402
SE	28	01405	Carira	20.345
SE	28	01504	Carmópolis	14.130
SE	28	01603	Cedro de São João	5.672
SE	28	01702	Cristinápolis	16.859
SE	28	01900	Cumbe	3.839
SE	28	02007	Divina Pastora	4.487
SE	28	02106	Estância	65.226
SE	28	02205	Feira Nova	5.363
SE	28	02304	Frei Paulo	14.162
SE	28	02403	Gararu	11.412
SE	28	02502	General Maynard	3.009
SE	28	02601	Gracho Cardoso	5.665
SE	28	02700	Ilha das Flores	8.359
SE	28	02809	Indiaroba	16.236
SE	28	02908	Itabaiana	88.501
SE	28	03005	Itabaianinha	39.432
SE	28	03104	Itabi	4.942
SE	28	03203	Itaporanga d'Ajuda	31.165
SE	28	03302	Japaratuba	17.213
SE	28	03401	Japoatã	12.926
SE	28	03500	Lagarto	96.602
SE	28	03609	Laranjeiras	27.442
SE	28	03708	Macambira	6.492
SE	28	03807	Malhada dos Bois	3.494
SE	28	03906	Malhador	12.127

SE	28	04003	Maruim	16.478
SE	28	04102	Moita Bonita	11.038
SE	28	04201	Monte Alegre de Sergipe	13.936
SE	28	04300	Muribeca	7.381
SE	28	04409	Neópolis	18.493
SE	28	04458	Nossa Senhora Aparecida	8.543
SE	28	04508	Nossa Senhora da Glória	33.341
SE	28	04607	Nossa Senhora das Dores	24.941
SE	28	04706	Nossa Senhora de Lourdes	6.271
SE	28	04805	Nossa Senhora do Socorro	165.194
SE	28	04904	Pacatuba	13.379
SE	28	05000	Pedra Mole	3.026
SE	28	05109	Pedrinhas	8.970
SE	28	05208	Pinhão	6.084
SE	28	05307	Pirambu	8.538
SE	28	05406	Poço Redondo	31.614
SE	28	05505	Poço Verde	22.287
SE	28	05604	Porto da Folha	27.370
SE	28	05703	Propriá	28.612
SE	28	05802	Riachão do Dantas	19.414
SE	28	05901	Riachuelo	9.509
SE	28	06008	Ribeirópolis	17.435
SE	28	06107	Rosário do Catete	9.541
SE	28	06206	Salgado	19.439
SE	28	06305	Santa Luzia do Itanhy	14.081
SE	28	06503	Santa Rosa de Lima	3.773
SE	28	06404	Santana do São Francisco	7.175
SE	28	06602	Santo Amaro das Brotas	11.522
SE	28	06701	São Cristóvão	81.011
SE	28	06800	São Domingos	10.424
SE	28	06909	São Francisco	3.524
SE	28	07006	São Miguel do Aleixo	3.736
SE	28	07105	Simão Dias	38.988
SE	28	07204	Siriri	8.169
SE	28	07303	Telha	3.006
SE	28	07402	Tobias Barreto	48.776
SE	28	07501	Tomar do Geru	12.858
SE	28	07600	Umbaúba	23.223
BA	29	00108	Abaíra	8.659
BA	29	00207	Abaré	17.685
BA	29	00306	Acajutiba	14.730
BA	29	00355	Adustina	15.914
BA	29	00405	Água Fria	15.884
BA	29	00603	Aiquara	4.536
BA	29	00702	Alagoinhas	143.460
BA	29	00801	Alcobaça	21.328

BA	29	00900	Almadina	6.130
BA	29	01007	Amargosa	34.845
BA	29	01106	Amélia Rodrigues	25.080
BA	29	01155	América Dourada	15.962
BA	29	01205	Anagé	19.889
BA	29	01304	Andaraí	13.942
BA	29	01353	Andorinha	14.209
BA	29	01403	Angical	13.992
BA	29	01502	Anguera	10.427
BA	29	01601	Antas	17.526
BA	29	01700	Antônio Cardoso	11.545
BA	29	01809	Antônio Gonçalves	11.229
BA	29	01908	Aporá	17.877
BA	29	01957	Apuarema	7.397
BA	29	02054	Araças	11.642
BA	29	02005	Aracatu	13.542
BA	29	02104	Araci	52.325
BA	29	02203	Aramari	10.483
BA	29	02252	Arataca	10.307
BA	29	02302	Aratuípe	8.632
BA	29	02401	Aurelino Leal	13.059
BA	29	02500	Baianópolis	13.420
BA	29	02609	Baixa Grande	20.031
BA	29	02658	Banzaê	11.840
BA	29	02708	Barra	50.134
BA	29	02807	Barra da Estiva	20.767
BA	29	02906	Barra do Choça	35.501
BA	29	03003	Barra do Mendes	13.914
BA	29	03102	Barra do Rocha	6.038
BA	29	03201	Barreiras	141.081
BA	29	03235	Barro Alto	13.914
BA	29	03300	Barro Preto	6.122
BA	29	03276	Barrocas	14.495
BA	29	03409	Belmonte	22.067
BA	29	03508	Belo Campo	17.625
BA	29	03607	Biritinga	14.866
BA	29	03706	Boa Nova	14.620
BA	29	03805	Boa Vista do Tupim	17.898
BA	29	03904	Bom Jesus da Lapa	64.740
BA	29	03953	Bom Jesus da Serra	10.120
BA	29	04001	Boninal	13.893
BA	29	04050	Bonito	15.126
BA	29	04100	Boquira	22.025
BA	29	04209	Botuporã	10.950
BA	29	04308	Brejões	14.123
BA	29	04407	Brejolândia	11.247

BA	29	04506	Brotas de Macaúbas	10.479
BA	29	04605	Brumado	64.972
BA	29	04704	Buerarema	18.528
BA	29	04753	Buritirama	19.853
BA	29	04803	Caatiba	10.576
BA	29	04852	Cabaceiras do Paraguaçu	17.582
BA	29	04902	Cachoeira	32.270
BA	29	05008	Caculé	22.577
BA	29	05107	Caém	10.013
BA	29	05156	Caetanos	14.926
BA	29	05206	Caetité	47.774
BA	29	05305	Cafarnaum	17.398
BA	29	05404	Cairu	15.973
BA	29	05503	Caldeirão Grande	12.658
BA	29	05602	Camacan	31.535
BA	29	05701	Camaçari	255.238
BA	29	05800	Camamu	35.366
BA	29	05909	Campo Alegre de Lourdes	28.156
BA	29	06006	Campo Formoso	67.305
BA	29	06105	Canápolis	9.395
BA	29	06204	Canarana	24.430
BA	29	06303	Canavieiras	31.902
BA	29	06402	Candeal	8.720
BA	29	06501	Candeias	84.121
BA	29	06600	Candiba	13.329
BA	29	06709	Cândido Sales	25.711
BA	29	06808	Cansanção	33.054
BA	29	06824	Canudos	15.941
BA	29	06857	Capela do Alto Alegre	11.485
BA	29	06873	Capim Grosso	27.067
BA	29	06899	Caraíbas	9.879
BA	29	06907	Caravelas	21.612
BA	29	07004	Cardeal da Silva	9.030
BA	29	07103	Carinhanha	28.519
BA	29	07202	Casa Nova	66.331
BA	29	07301	Castro Alves	25.555
BA	29	07400	Catolândia	3.215
BA	29	07509	Catu	51.734
BA	29	07558	Caturama	8.817
BA	29	07608	Central	17.057
BA	29	07707	Chorrochó	10.794
BA	29	07806	Cícero Dantas	32.470
BA	29	07905	Cipó	15.884
BA	29	08002	Coaraci	19.937
BA	29	08101	Cocos	18.235
BA	29	08200	Conceição da Feira	20.826

BA	29	08309	Conceição do Almeida	17.705
BA	29	08408	Conceição do Coité	63.033
BA	29	08507	Conceição do Jacuípe	30.717
BA	29	08606	Conde	24.103
BA	29	08705	Condeúba	17.421
BA	29	08804	Contendas do Sincorá	4.613
BA	29	08903	Coração de Maria	22.149
BA	29	09000	Cordeiros	8.245
BA	29	09109	Coribe	14.210
BA	29	09208	Coronel João Sá	16.650
BA	29	09307	Correntina	31.397
BA	29	09406	Cotegipe	13.614
BA	29	09505	Cravolândia	5.048
BA	29	09604	Crisópolis	20.199
BA	29	09703	Cristópolis	13.374
BA	29	09802	Cruz das Almas	59.470
BA	29	09901	Curaçá	32.631
BA	29	10008	Dário Meira	12.217
BA	29	10057	Dias d'Ávila	69.628
BA	29	10107	Dom Basílio	11.454
BA	29	10206	Dom Macedo Costa	3.894
BA	29	10305	Elísio Medrado	7.961
BA	29	10404	Encruzilhada	22.478
BA	29	10503	Entre Rios	40.180
BA	29	00504	Érico Cardoso	10.746
BA	29	10602	Esplanada	33.618
BA	29	10701	Euclides da Cunha	56.962
BA	29	10727	Eunápolis	102.628
BA	29	10750	Fátima	17.555
BA	29	10776	Feira da Mata	6.177
BA	29	10800	Feira de Santana	568.099
BA	29	10859	Filadélfia	16.672
BA	29	10909	Firmino Alves	5.417
BA	29	11006	Floresta Azul	10.657
BA	29	11105	Formosa do Rio Preto	23.169
BA	29	11204	Gandu	30.816
BA	29	11253	Gavião	4.510
BA	29	11303	Gentio do Ouro	10.690
BA	29	11402	Glória	15.114
BA	29	11501	Gongogi	8.031
BA	29	11600	Governador Mangabeira	19.926
BA	29	11659	Guajeru	9.182
BA	29	11709	Guanambi	79.936
BA	29	11808	Guaratinga	21.840
BA	29	11857	Heliópolis	13.099
BA	29	11907	Iaçu	25.319

BA	29	12004	Ibiassucê	9.607
BA	29	12103	Ibicaraí	23.560
BA	29	12202	Ibicoara	17.805
BA	29	12301	Ibicuí	15.650
BA	29	12400	Ibipeba	17.277
BA	29	12509	Ibipitanga	14.285
BA	29	12608	Ibiquera	4.874
BA	29	12707	Ibirapitanga	22.683
BA	29	12806	Ibirapuã	8.086
BA	29	12905	Ibirataia	17.959
BA	29	13002	Ibitiara	15.669
BA	29	13101	Ibititá	17.763
BA	29	13200	Ibotirama	25.617
BA	29	13309	Ichu	5.908
BA	29	13408	Igaporã	15.238
BA	29	13457	Igrapiúna	13.028
BA	29	13507	Iguaí	26.053
BA	29	13606	Ilhéus	187.315
BA	29	13705	Inhambupe	37.321
BA	29	13804	Ipecaetá	15.025
BA	29	13903	Ipiaú	44.538
BA	29	14000	Ipirá	59.001
BA	29	14109	Ipupiara	9.398
BA	29	14208	Irajuba	7.046
BA	29	14307	Iramaia	11.150
BA	29	14406	Iraquara	23.246
BA	29	14505	Irará	27.814
BA	29	14604	Irecê	67.527
BA	29	14653	Itabela	28.790
BA	29	14703	Itaberaba	62.037
BA	29	14802	Itabuna	205.885
BA	29	14901	Itacaré	25.254
BA	29	15007	Itaeté	15.063
BA	29	15106	Itagi	12.805
BA	29	15205	Itagibá	15.088
BA	29	15304	Itagimirim	7.013
BA	29	15353	Itaguaçu da Bahia	13.487
BA	29	15403	Itaju do Colônia	7.118
BA	29	15502	Itajuípe	20.878
BA	29	15601	Itamaraju	63.037
BA	29	15700	Itamari	7.836
BA	29	15809	Itambé	22.650
BA	29	15908	Itanagra	7.590
BA	29	16005	Itanhém	20.015
BA	29	16104	Itaparica	20.994
BA	29	16203	Itapé	10.436

BA	29	16302	Itapebi	10.398
BA	29	16401	Itapetinga	69.903
BA	29	16500	Itapicuru	33.008
BA	29	16609	Itapitanga	10.181
BA	29	16708	Itaquara	7.751
BA	29	16807	Itarantim	18.651
BA	29	16856	Itatim	13.841
BA	29	16906	Itiruçu	12.589
BA	29	17003	Itiúba	36.200
BA	29	17102	Itororó	19.942
BA	29	17201	Ituaçu	18.302
BA	29	17300	Ituberá	26.930
BA	29	17334	Iuiú	10.963
BA	29	17359	Jaborandi	8.728
BA	29	17409	Jacaraci	14.500
BA	29	17508	Jacobina	79.580
BA	29	17607	Jaguaquara	51.635
BA	29	17706	Jaguarari	30.769
BA	29	17805	Jaguaripe	16.927
BA	29	17904	Jandaíra	10.377
BA	29	18001	Jequié	152.372
BA	29	18100	Jeremoabo	38.163
BA	29	18209	Jiquiriçá	14.096
BA	29	18308	Jitaúna	13.280
BA	29	18357	João Dourado	23.066
BA	29	18407	Juazeiro	201.499
BA	29	18456	Jucuruçu	9.972
BA	29	18506	Jussara	15.004
BA	29	18555	Jussari	6.322
BA	29	18605	Jussiape	7.533
BA	29	18704	Lafaiete Coutinho	3.830
BA	29	18753	Lagoa Real	14.187
BA	29	18803	Laje	22.679
BA	29	18902	Lajedão	3.782
BA	29	19009	Lajedinho	3.881
BA	29	19058	Lajedo do Tabocal	8.346
BA	29	19108	Lamarão	9.271
BA	29	19157	Lapão	25.785
BA	29	19207	Lauro de Freitas	171.042
BA	29	19306	Lençóis	10.589
BA	29	19405	Licínio de Almeida	12.268
BA	29	19504	Livramento de Nossa Senhora	43.514
BA	29	19553	Luís Eduardo Magalhães	66.371
BA	29	19603	Macajuba	11.201
BA	29	19702	Macarani	17.253
BA	29	19801	Macaúbas	47.915

BA	29	19900	Macururé	7.992
BA	29	19926	Madre de Deus	18.183
BA	29	19959	Maetinga	6.048
BA	29	20007	Maiquinique	9.229
BA	29	20106	Mairi	19.163
BA	29	20205	Malhada	16.058
BA	29	20304	Malhada de Pedras	8.389
BA	29	20403	Manoel Vitorino	13.948
BA	29	20452	Mansidão	12.759
BA	29	20502	Maracás	25.024
BA	29	20601	Maragogipe	43.114
BA	29	20700	Maraú	19.212
BA	29	20809	Marcionílio Souza	10.447
BA	29	20908	Mascote	14.257
BA	29	21005	Mata de São João	41.527
BA	29	21054	Matina	11.342
BA	29	21104	Medeiros Neto	21.642
BA	29	21203	Miguel Calmon	26.188
BA	29	21302	Milagres	10.994
BA	29	21401	Mirangaba	16.606
BA	29	21450	Mirante	9.902
BA	29	21500	Monte Santo	52.023
BA	29	21609	Morpará	8.233
BA	29	21708	Morro do Chapéu	35.251
BA	29	21807	Mortugaba	11.729
BA	29	21906	Mucugê	10.145
BA	29	22003	Mucuri	37.229
BA	29	22052	Mulungu do Morro	11.743
BA	29	22102	Mundo Novo	24.867
BA	29	22201	Muniz Ferreira	7.374
BA	29	22250	Muquém de São Francisco	10.433
BA	29	22300	Muritiba	28.944
BA	29	22409	Mutuípe	21.608
BA	29	22508	Nazaré	27.454
BA	29	22607	Nilo Peçanha	12.729
BA	29	22656	Nordestina	12.458
BA	29	22706	Nova Canaã	16.070
BA	29	22730	Nova Fátima	7.630
BA	29	22755	Nova Ibiá	6.570
BA	29	22805	Nova Itarana	7.563
BA	29	22854	Nova Redenção	8.053
BA	29	22904	Nova Soure	24.265
BA	29	23001	Nova Viçosa	39.535
BA	29	23035	Novo Horizonte	11.001
BA	29	23050	Novo Triunfo	15.067
BA	29	23100	Olindina	25.100

BA	29	23209	Oliveira dos Brejinhos	21.813
BA	29	23308	Ouriçangas	8.316
BA	29	23357	Ourolândia	16.578
BA	29	23407	Palmas de Monte Alto	20.894
BA	29	23506	Palmeiras	8.545
BA	29	23605	Paramirim	21.226
BA	29	23704	Paratinga	29.853
BA	29	23803	Paripiranga	27.958
BA	29	23902	Pau Brasil	10.479
BA	29	24009	Paulo Afonso	110.193
BA	29	24058	Pé de Serra	13.707
BA	29	24108	Pedrão	6.993
BA	29	24207	Pedro Alexandre	17.045
BA	29	24306	Piatã	17.257
BA	29	24405	Pilão Arcado	33.176
BA	29	24504	Pindaí	15.695
BA	29	24603	Pindobaçu	20.009
BA	29	24652	Pintadas	10.250
BA	29	24678	Piraí do Norte	9.833
BA	29	24702	Piripá	12.219
BA	29	24801	Piritiba	22.907
BA	29	24900	Planaltino	8.944
BA	29	25006	Planalto	24.627
BA	29	25105	Poções	45.903
BA	29	25204	Pojuca	34.106
BA	29	25253	Ponto Novo	15.524
BA	29	25303	Porto Seguro	131.642
BA	29	25402	Potiraguá	9.360
BA	29	25501	Prado	27.693
BA	29	25600	Presidente Dutra	13.807
BA	29	25709	Presidente Jânio Quadros	12.854
BA	29	25758	Presidente Tancredo Neves	24.517
BA	29	25808	Queimadas	24.602
BA	29	25907	Quijingue	27.357
BA	29	25931	Quixabeira	9.514
BA	29	25956	Rafael Jambeiro	22.916
BA	29	26004	Remanso	39.365
BA	29	26103	Retirolândia	12.281
BA	29	26202	Riachão das Neves	21.941
BA	29	26301	Riachão do Jacuípe	33.271
BA	29	26400	Riacho de Santana	31.027
BA	29	26509	Ribeira do Amparo	14.333
BA	29	26608	Ribeira do Pombal	47.877
BA	29	26657	Ribeirão do Largo	10.432
BA	29	26707	Rio de Contas	12.891
BA	29	26806	Rio do Antônio	15.015

BA	29	26905	Rio do Pires	11.948
BA	29	27002	Rio Real	37.754
BA	29	27101	Rodelas	8.045
BA	29	27200	Ruy Barbosa	30.010
BA	29	27309	Salinas da Margarida	13.921
BA	29	27408	Salvador	2.710.968
BA	29	27507	Santa Bárbara	19.292
BA	29	27606	Santa Brígida	14.698
BA	29	27705	Santa Cruz Cabrália	26.623
BA	29	27804	Santa Cruz da Vitória	6.481
BA	29	27903	Santa Inês	10.312
BA	29	28059	Santa Luzia	13.025
BA	29	28109	Santa Maria da Vitória	40.165
BA	29	28406	Santa Rita de Cássia	26.653
BA	29	28505	Santa Teresinha	9.792
BA	29	28000	Santaluz	34.274
BA	29	28208	Santana	24.987
BA	29	28307	Santanópolis	8.835
BA	29	28604	Santo Amaro	57.978
BA	29	28703	Santo Antônio de Jesus	93.077
BA	29	28802	Santo Estêvão	48.897
BA	29	28901	São Desidério	28.921
BA	29	28950	São Domingos	9.266
BA	29	29107	São Felipe	20.329
BA	29	29008	São Félix	14.159
BA	29	29057	São Félix do Coribe	13.243
BA	29	29206	São Francisco do Conde	34.226
BA	29	29255	São Gabriel	18.430
BA	29	29305	São Gonçalo dos Campos	34.232
BA	29	29354	São José da Vitória	5.609
BA	29	29370	São José do Jacuípe	10.293
BA	29	29404	São Miguel das Matas	10.474
BA	29	29503	São Sebastião do Passé	42.485
BA	29	29602	Sapeaçu	16.619
BA	29	29701	Sátiro Dias	19.054
BA	29	29750	Saubara	11.354
BA	29	29800	Saúde	11.921
BA	29	29909	Seabra	42.163
BA	29	30006	Sebastião Laranjeiras	10.563
BA	29	30105	Senhor do Bonfim	75.437
BA	29	30204	Sento Sé	38.174
BA	29	30154	Serra do Ramalho	31.525
BA	29	30303	Serra Dourada	17.963
BA	29	30402	Serra Preta	14.993
BA	29	30501	Serrinha	77.211
BA	29	30600	Serrolândia	12.464

BA	29	30709	Simões Filho	121.416
BA	29	30758	Sítio do Mato	12.161
BA	29	30766	Sítio do Quinto	11.930
BA	29	30774	Sobradinho	22.109
BA	29	30808	Souto Soares	16.069
BA	29	30907	Tabocas do Brejo Velho	11.433
BA	29	31004	Tanhaçu	20.001
BA	29	31053	Tanque Novo	16.323
BA	29	31103	Tanquinho	8.035
BA	29	31202	Taperoá	19.174
BA	29	31301	Tapiramutá	16.434
BA	29	31350	Teixeira de Freitas	143.001
BA	29	31400	Teodoro Sampaio	7.746
BA	29	31509	Teofilândia	21.581
BA	29	31608	Teolândia	14.113
BA	29	31707	Terra Nova	12.793
BA	29	31806	Tremedal	17.750
BA	29	31905	Tucano	52.734
BA	29	32002	Uauá	24.015
BA	29	32101	Ubaíra	20.714
BA	29	32200	Ubaitaba	20.214
BA	29	32309	Ubatã	25.575
BA	29	32408	Uibaí	13.642
BA	29	32457	Umburanas	17.432
BA	29	32507	Una	22.992
BA	29	32606	Urandi	16.493
BA	29	32705	Uruçuca	19.642
BA	29	32804	Utinga	18.367
BA	29	32903	Valença	90.319
BA	29	33000	Valente	25.342
BA	29	33059	Várzea da Roça	13.834
BA	29	33109	Várzea do Poço	8.759
BA	29	33158	Várzea Nova	12.910
BA	29	33174	Varzedo	8.987
BA	29	33208	Vera Cruz	38.748
BA	29	33257	Vereda	6.681
BA	29	33307	Vitória da Conquista	315.884
BA	29	33406	Wagner	8.985
BA	29	33455	Wanderley	12.356
BA	29	33505	Wenceslau Guimarães	21.910
BA	29	33604	Xique-Xique	45.660
MG	31	00104	Abadia dos Dourados	6.743
MG	31	00203	Abaeté	22.740
MG	31	00302	Abre Campo	13.306
MG	31	00401	Acaiaca	3.925
MG	31	00500	Açucena	10.093

MG	31	00609	Água Boa	14.803
MG	31	00708	Água Comprida	2.015
MG	31	00807	Aguanil	4.129
MG	31	00906	Águas Formosas	18.575
MG	31	01003	Águas Vermelhas	12.850
MG	31	01102	Aimorés	24.937
MG	31	01201	Aiuruoca	6.116
MG	31	01300	Alagoa	2.696
MG	31	01409	Albertina	2.924
MG	31	01508	Além Paraíba	34.461
MG	31	01607	Alfenas	74.804
MG	31	01631	Alfredo Vasconcelos	6.223
MG	31	01706	Almenara	39.287
MG	31	01805	Alpercata	7.204
MG	31	01904	Alpinópolis	18.709
MG	31	02001	Alterosa	13.829
MG	31	02050	Alto Caparaó	5.392
MG	31	53509	Alto Jequitibá	8.297
MG	31	02100	Alto Rio Doce	11.903
MG	31	02209	Alvarenga	4.329
MG	31	02308	Alvinópolis	15.212
MG	31	02407	Alvorada de Minas	3.549
MG	31	02506	Amparo do Serra	4.910
MG	31	02605	Andradas	37.920
MG	31	02803	Andrelândia	12.153
MG	31	02852	Angelândia	8.084
MG	31	02902	Antônio Carlos	11.151
MG	31	03009	Antônio Dias	9.493
MG	31	03108	Antônio Prado de Minas	1.653
MG	31	03207	Araçaí	2.258
MG	31	03306	Aracitaba	2.054
MG	31	03405	Araçuaí	36.059
MG	31	03504	Araguari	110.983
MG	31	03603	Arantina	2.811
MG	31	03702	Araponga	8.188
MG	31	03751	Araporã	6.271
MG	31	03801	Arapuá	2.780
MG	31	03900	Araújos	8.135
MG	31	04007	Araxá	95.888
MG	31	04106	Arceburgo	9.732
MG	31	04205	Arcos	37.188
MG	31	04304	Areado	13.958
MG	31	04403	Argirita	2.860
MG	31	04452	Aricanduva	4.848
MG	31	04502	Arinos	17.669
MG	31	04601	Astolfo Dutra	13.237

MG	31	04700	Ataléia	14.109
MG	31	04809	Augusto de Lima	4.930
MG	31	04908	Baependi	18.426
MG	31	05004	Baldim	7.877
MG	31	05103	Bambuí	22.891
MG	31	05202	Bandeira	4.938
MG	31	05301	Bandeira do Sul	5.405
MG	31	05400	Barão de Cocais	29.205
MG	31	05509	Barão de Monte Alto	5.643
MG	31	05608	Barbacena	128.120
MG	31	05707	Barra Longa	5.930
MG	31	05905	Barroso	19.787
MG	31	06002	Bela Vista de Minas	10.028
MG	31	06101	Belmiro Braga	3.400
MG	31	06200	Belo Horizonte	2.395.785
MG	31	06309	Belo Oriente	23.984
MG	31	06408	Belo Vale	7.553
MG	31	06507	Berilo	12.198
MG	31	06655	Berizal	4.431
MG	31	06606	Bertópolis	4.508
MG	31	06705	Betim	388.873
MG	31	06804	Bias Fortes	3.703
MG	31	06903	Bicas	13.783
MG	31	07000	Biquinhas	2.602
MG	31	07109	Boa Esperança	38.734
MG	31	07208	Bocaina de Minas	5.011
MG	31	07307	Bocaiúva	47.236
MG	31	07406	Bom Despacho	46.482
MG	31	07505	Bom Jardim de Minas	6.480
MG	31	07604	Bom Jesus da Penha	3.942
MG	31	07703	Bom Jesus do Amparo	5.593
MG	31	07802	Bom Jesus do Galho	15.242
MG	31	07901	Bom Repouso	10.449
MG	31	08008	Bom Sucesso	17.271
MG	31	08107	Bonfim	6.811
MG	31	08206	Bonfinópolis de Minas	5.778
MG	31	08255	Bonito de Minas	9.947
MG	31	08305	Borda da Mata	17.523
MG	31	08404	Botelhos	14.893
MG	31	08503	Botumirim	6.447
MG	31	08701	Brás Pires	4.567
MG	31	08552	Brasilândia de Minas	14.642
MG	31	08602	Brasília de Minas	31.356
MG	31	08909	Brasópolis	14.585
MG	31	08800	Braúnas	4.973
MG	31	09006	Brumadinho	35.085

MG	31	09105	Bueno Brandão	10.886
MG	31	09204	Buenópolis	10.281
MG	31	09253	Bugre	3.999
MG	31	09303	Buritis	23.091
MG	31	09402	Buritizeiro	27.076
MG	31	09451	Cabeceira Grande	6.534
MG	31	09501	Cabo Verde	13.838
MG	31	09600	Cachoeira da Prata	3.635
MG	31	09709	Cachoeira de Minas	11.107
MG	31	02704	Cachoeira de Pajeú	9.025
MG	31	09808	Cachoeira Dourada	2.536
MG	31	09907	Caetanópolis	10.467
MG	31	10004	Caeté	41.423
MG	31	10103	Caiana	5.059
MG	31	10202	Cajuri	4.026
MG	31	10301	Caldas	13.764
MG	31	10400	Camacho	3.097
MG	31	10509	Camanducaia	21.162
MG	31	10608	Cambuí	27.020
MG	31	10707	Cambuquira	12.612
MG	31	10806	Campanário	3.586
MG	31	10905	Campanha	15.635
MG	31	11002	Campestre	20.707
MG	31	11101	Campina Verde	19.358
MG	31	11150	Campo Azul	3.701
MG	31	11200	Campo Belo	51.900
MG	31	11309	Campo do Meio	11.483
MG	31	11408	Campo Florido	7.103
MG	31	11507	Campos Altos	14.416
MG	31	11606	Campos Gerais	27.760
MG	31	11903	Cana Verde	5.578
MG	31	11705	Canaã	4.604
MG	31	11804	Canápolis	11.476
MG	31	12000	Candeias	14.616
MG	31	12059	Cantagalo	4.249
MG	31	12109	Caparaó	5.241
MG	31	12208	Capela Nova	4.724
MG	31	12307	Capelinha	35.368
MG	31	12406	Capetinga	7.039
MG	31	12505	Capim Branco	9.030
MG	31	12604	Capinópolis	15.424
MG	31	12653	Capitão Andrade	5.019
MG	31	12703	Capitão Enéas	14.372
MG	31	12802	Capitólio	8.251
MG	31	12901	Caputira	9.060
MG	31	13008	Caraí	22.549

MG	31	13107	Caranaíba	3.260
MG	31	13206	Carandaí	23.692
MG	31	13305	Carangola	32.353
MG	31	13404	Caratinga	86.364
MG	31	13503	Carbonita	9.176
MG	31	13602	Careaçu	6.372
MG	31	13701	Carlos Chagas	19.779
MG	31	13800	Carmésia	2.477
MG	31	13909	Carmo da Cachoeira	11.872
MG	31	14006	Carmo da Mata	11.007
MG	31	14105	Carmo de Minas	13.932
MG	31	14204	Carmo do Cajuru	20.444
MG	31	14303	Carmo do Paranaíba	29.777
MG	31	14402	Carmo do Rio Claro	20.531
MG	31	14501	Carmópolis de Minas	17.456
MG	31	14550	Carneirinho	9.556
MG	31	14600	Carrancas	3.958
MG	31	14709	Carvalhópolis	3.380
MG	31	14808	Carvalhos	4.530
MG	31	14907	Casa Grande	2.241
MG	31	15003	Cascalho Rico	2.893
MG	31	15102	Cássia	17.433
MG	31	15300	Cataguases	70.630
MG	31	15359	Catas Altas	4.938
MG	31	15409	Catas Altas da Noruega	3.489
MG	31	15458	Catuji	6.614
MG	31	15474	Catuti	5.067
MG	31	15508	Caxambu	21.641
MG	31	15607	Cedro do Abaeté	1.199
MG	31	15706	Central de Minas	6.806
MG	31	15805	Centralina	10.271
MG	31	15904	Chácara	2.856
MG	31	16001	Chalé	5.643
MG	31	16100	Chapada do Norte	15.184
MG	31	16159	Chapada Gaúcha	11.339
MG	31	16209	Chiador	2.759
MG	31	16308	Cipotânea	6.578
MG	31	16407	Claraval	4.588
MG	31	16506	Claro dos Poções	7.712
MG	31	16605	Cláudio	26.262
MG	31	16704	Coimbra	7.135
MG	31	16803	Coluna	8.972
MG	31	16902	Comendador Gomes	2.992
MG	31	17009	Comercinho	8.011
MG	31	17108	Conceição da Aparecida	9.888
MG	31	15201	Conceição da Barra de Minas	3.944

MG	31	17306	Conceição das Alagoas	23.932
MG	31	17207	Conceição das Pedras	2.755
MG	31	17405	Conceição de Ipanema	4.468
MG	31	17504	Conceição do Mato Dentro	17.798
MG	31	17603	Conceição do Pará	5.214
MG	31	17702	Conceição do Rio Verde	13.052
MG	31	17801	Conceição dos Ouros	10.609
MG	31	17836	Cônego Marinho	7.196
MG	31	17876	Confins	6.077
MG	31	17900	Congonhal	10.732
MG	31	18007	Congonhas	49.616
MG	31	18106	Congonhas do Norte	4.950
MG	31	18205	Conquista	6.591
MG	31	18304	Conselheiro Lafaiete	118.578
MG	31	18403	Conselheiro Pena	22.319
MG	31	18502	Consolação	1.732
MG	31	18601	Contagem	613.815
MG	31	18700	Coqueiral	9.241
MG	31	18809	Coração de Jesus	26.079
MG	31	18908	Cordisburgo	8.689
MG	31	19005	Cordislândia	3.447
MG	31	19104	Corinto	23.819
MG	31	19203	Coroaci	10.190
MG	31	19302	Coromandel	27.562
MG	31	19401	Coronel Fabriciano	104.637
MG	31	19500	Coronel Murta	9.115
MG	31	19609	Coronel Pacheco	2.996
MG	31	19708	Coronel Xavier Chaves	3.319
MG	31	19807	Córrego Danta	3.349
MG	31	19906	Córrego do Bom Jesus	3.716
MG	31	19955	Córrego Fundo	5.883
MG	31	20003	Córrego Novo	3.050
MG	31	20102	Couto de Magalhães de Minas	4.234
MG	31	20151	Crisólita	6.161
MG	31	20201	Cristais	11.553
MG	31	20300	Cristália	5.787
MG	31	20409	Cristiano Otoni	5.023
MG	31	20508	Cristina	10.191
MG	31	20607	Crucilândia	4.800
MG	31	20706	Cruzeiro da Fortaleza	3.967
MG	31	20805	Cruzília	14.716
MG	31	20839	Cuparaque	4.728
MG	31	20870	Curral de Dentro	7.055
MG	31	20904	Curvelo	75.014
MG	31	21001	Datas	5.237
MG	31	21100	Delfim Moreira	7.962

MG	31	21209	Delfinópolis	6.869
MG	31	21258	Delta	8.546
MG	31	21308	Descoberto	4.804
MG	31	21407	Desterro de Entre Rios	7.032
MG	31	21506	Desterro do Melo	2.986
MG	31	21605	Diamantina	46.125
MG	31	21704	Diogo de Vasconcelos	3.830
MG	31	21803	Dionísio	8.520
MG	31	21902	Divinésia	3.309
MG	31	22009	Divino	19.241
MG	31	22108	Divino das Laranjeiras	4.933
MG	31	22207	Divinolândia de Minas	7.114
MG	31	22306	Divinópolis	217.404
MG	31	22355	Divisa Alegre	6.046
MG	31	22405	Divisa Nova	5.797
MG	31	22454	Divisópolis	9.351
MG	31	22470	Dom Bosco	3.778
MG	31	22504	Dom Cavati	5.170
MG	31	22603	Dom Joaquim	4.511
MG	31	22702	Dom Silvério	5.192
MG	31	22801	Dom Viçoso	2.988
MG	31	22900	Dona Eusébia	6.098
MG	31	23007	Dores de Campos	9.443
MG	31	23106	Dores de Guanhães	5.200
MG	31	23205	Dores do Indaiá	13.686
MG	31	23304	Dores do Turvo	4.412
MG	31	23403	Doresópolis	1.454
MG	31	23502	Douradoquara	1.850
MG	31	23528	Durandé	7.487
MG	31	23601	Elói Mendes	25.715
MG	31	23700	Engenheiro Caldas	10.421
MG	31	23809	Engenheiro Navarro	7.128
MG	31	23858	Entre Folhas	5.194
MG	31	23908	Entre Rios de Minas	14.413
MG	31	24005	Ervália	18.087
MG	31	24104	Esmeraldas	62.262
MG	31	24203	Espera Feliz	23.208
MG	31	24302	Espinosa	31.134
MG	31	24401	Espírito Santo do Dourado	4.470
MG	31	24500	Estiva	10.918
MG	31	24609	Estrela Dalva	2.440
MG	31	24708	Estrela do Indaiá	3.504
MG	31	24807	Estrela do Sul	7.532
MG	31	24906	Eugenópolis	10.657
MG	31	25002	Ewbank da Câmara	3.775
MG	31	25101	Extrema	30.016

MG	31	25200	Fama	2.350
MG	31	25309	Faria Lemos	3.342
MG	31	25408	Felício dos Santos	5.054
MG	31	25606	Felisburgo	6.974
MG	31	25705	Felixlândia	14.323
MG	31	25804	Fernandes Tourinho	3.101
MG	31	25903	Ferros	10.612
MG	31	25952	Fervedouro	10.452
MG	31	26000	Florestal	6.744
MG	31	26109	Formiga	65.464
MG	31	26208	Formoso	8.427
MG	31	26307	Fortaleza de Minas	4.150
MG	31	26406	Fortuna de Minas	2.746
MG	31	26505	Francisco Badaró	10.239
MG	31	26604	Francisco Dumont	4.920
MG	31	26703	Francisco Sá	25.116
MG	31	26752	Franciscópolis	5.706
MG	31	26802	Frei Gaspar	5.865
MG	31	26901	Frei Inocêncio	9.033
MG	31	26950	Frei Lagonegro	3.350
MG	31	27008	Fronteira	14.799
MG	31	27057	Fronteira dos Vales	4.655
MG	31	27073	Fruta de Leite	5.814
MG	31	27107	Frutal	54.511
MG	31	27206	Funilândia	3.942
MG	31	27305	Galiléia	6.908
MG	31	27339	Gameleiras	5.121
MG	31	27354	Glaucilândia	2.992
MG	31	27370	Goiabeira	3.105
MG	31	27388	Goianá	3.710
MG	31	27404	Gonçalves	4.235
MG	31	27503	Gonzaga	5.953
MG	31	27602	Gouveia	11.680
MG	31	27701	Governador Valadares	266.190
MG	31	27800	Grão Mogol	15.145
MG	31	27909	Grupiara	1.373
MG	31	28006	Guanhães	31.781
MG	31	28105	Guapé	13.911
MG	31	28204	Guaraciaba	10.218
MG	31	28253	Guaraciama	4.756
MG	31	28303	Guaranésia	18.727
MG	31	28402	Guarani	8.702
MG	31	28501	Guarará	3.894
MG	31	28600	Guarda-Mor	6.552
MG	31	28709	Guaxupé	49.792
MG	31	28808	Guidoval	7.164

MG	31	28907	Guimarânia	7.399
MG	31	29004	Guiricema	8.624
MG	31	29103	Gurinhatã	6.025
MG	31	29202	Heliodora	6.192
MG	31	29301	Iapu	10.406
MG	31	29400	Ibertioga	5.021
MG	31	29509	Ibiá	23.547
MG	31	29608	Ibiaí	7.928
MG	31	29657	Ibiracatu	6.098
MG	31	29707	Ibiraci	12.470
MG	31	29806	Ibirité	162.867
MG	31	29905	Ibitiúra de Minas	3.395
MG	31	30002	Ibituruna	2.883
MG	31	30051	Icaraí de Minas	10.963
MG	31	30101	Igarapé	36.363
MG	31	30200	Igaratinga	9.553
MG	31	30309	Iguatama	7.993
MG	31	30408	Ijaci	5.980
MG	31	30507	Ilicínea	11.633
MG	31	30556	Imbé de Minas	6.502
MG	31	30606	Inconfidentes	6.973
MG	31	30655	Indaiabira	7.316
MG	31	30705	Indianópolis	6.312
MG	31	30804	Ingaí	2.650
MG	31	30903	Inhapim	24.204
MG	31	31000	Inhaúma	5.846
MG	31	31109	Inimutaba	7.034
MG	31	31158	Ipaba	17.037
MG	31	31208	Ipanema	18.455
MG	31	31307	Ipatinga	243.541
MG	31	31406	Ipiaçu	4.120
MG	31	31505	Ipuiúna	9.607
MG	31	31604	Iraí de Minas	6.553
MG	31	31703	Itabira	111.514
MG	31	31802	Itabirinha	10.826
MG	31	31901	Itabirito	46.589
MG	31	32008	Itacambira	5.053
MG	31	32107	Itacarambi	17.761
MG	31	32206	Itaguara	12.534
MG	31	32305	Itaipé	11.957
MG	31	32404	Itajubá	91.643
MG	31	32503	Itamarandiba	32.595
MG	31	32602	Itamarati de Minas	4.123
MG	31	32701	Itambacuri	22.831
MG	31	32800	Itambé do Mato Dentro	2.238
MG	31	32909	Itamogi	10.293

MG	31	33006	Itamonte	14.276
MG	31	33105	Itanhandu	14.366
MG	31	33204	Itanhomi	11.899
MG	31	33303	Itaobim	20.961
MG	31	33402	Itapagipe	13.932
MG	31	33501	Itapecerica	21.399
MG	31	33600	Itapeva	8.861
MG	31	33709	Itatiaiuçu	10.142
MG	31	33758	Itaú de Minas	15.135
MG	31	33808	Itaúna	86.762
MG	31	33907	Itaverava	5.711
MG	31	34004	Itinga	14.485
MG	31	34103	Itueta	5.859
MG	31	34202	Ituiutaba	98.392
MG	31	34301	Itumirim	6.101
MG	31	34400	Iturama	35.308
MG	31	34509	Itutinga	3.879
MG	31	34608	Jaboticatubas	17.679
MG	31	34707	Jacinto	12.142
MG	31	34806	Jacuí	7.520
MG	31	34905	Jacutinga	23.341
MG	31	35001	Jaguaraçu	3.011
MG	31	35050	Jaíba	34.539
MG	31	35076	Jampruca	5.121
MG	31	35100	Janaúba	67.581
MG	31	35209	Januária	65.744
MG	31	35308	Japaraíba	4.010
MG	31	35357	Japonvar	8.331
MG	31	35407	Jeceaba	5.288
MG	31	35456	Jenipapo de Minas	7.211
MG	31	35506	Jequeri	12.726
MG	31	35605	Jequitaí	7.893
MG	31	35704	Jequitibá	5.154
MG	31	35803	Jequitinhonha	24.317
MG	31	35902	Jesuânia	4.760
MG	31	36009	Joaíma	15.000
MG	31	36108	Joanésia	5.246
MG	31	36207	João Monlevade	74.655
MG	31	36306	João Pinheiro	45.848
MG	31	36405	Joaquim Felício	4.371
MG	31	36504	Jordânia	10.394
MG	31	36520	José Gonçalves de Minas	4.532
MG	31	36553	José Raydan	4.487
MG	31	36579	Josenópolis	4.614
MG	31	36652	Juatuba	23.080
MG	31	36702	Juiz de Fora	525.225

MG	31	36801	Juramento	4.146
MG	31	36900	Juruaia	9.474
MG	31	36959	Juvenília	5.697
MG	31	37007	Ladainha	17.170
MG	31	37106	Lagamar	7.584
MG	31	37205	Lagoa da Prata	47.076
MG	31	37304	Lagoa dos Patos	4.191
MG	31	37403	Lagoa Dourada	12.373
MG	31	37502	Lagoa Formosa	17.293
MG	31	37536	Lagoa Grande	8.786
MG	31	37601	Lagoa Santa	54.732
MG	31	37700	Lajinha	19.622
MG	31	37809	Lambari	19.752
MG	31	37908	Lamim	3.432
MG	31	38005	Laranjal	6.517
MG	31	38104	Lassance	6.474
MG	31	38203	Lavras	94.228
MG	31	38302	Leandro Ferreira	3.202
MG	31	38351	Leme do Prado	4.815
MG	31	38401	Leopoldina	51.286
MG	31	38500	Liberdade	5.279
MG	31	38609	Lima Duarte	16.216
MG	31	38625	Limeira do Oeste	6.999
MG	31	38658	Lontra	8.506
MG	31	38674	Luisburgo	6.225
MG	31	38682	Luislândia	6.443
MG	31	38708	Luminárias	5.413
MG	31	38807	Luz	17.585
MG	31	38906	Machacalis	6.985
MG	31	39003	Machado	39.264
MG	31	39102	Madre de Deus de Minas	4.930
MG	31	39201	Malacacheta	18.705
MG	31	39250	Mamonas	6.349
MG	31	39300	Manga	19.489
MG	31	39409	Manhuaçu	81.455
MG	31	39508	Manhumirim	21.587
MG	31	39607	Mantena	27.148
MG	31	39805	Mar de Espanha	11.928
MG	31	39706	Maravilhas	7.304
MG	31	39904	Maria da Fé	14.157
MG	31	40001	Mariana	55.353
MG	31	40100	Marilac	4.189
MG	31	40159	Mário Campos	13.594
MG	31	40209	Maripá de Minas	2.818
MG	31	40308	Marliéria	4.008
MG	31	40407	Marmelópolis	2.919

MG	31	40506	Martinho Campos	12.731
MG	31	40530	Martins Soares	7.398
MG	31	40555	Mata Verde	7.994
MG	31	40605	Materlândia	4.558
MG	31	40704	Mateus Leme	28.417
MG	31	71501	Mathias Lobato	3.329
MG	31	40803	Matias Barbosa	13.603
MG	31	40852	Matias Cardoso	10.188
MG	31	40902	Matipó	17.843
MG	31	41009	Mato Verde	12.609
MG	31	41108	Matozinhos	34.624
MG	31	41207	Matutina	3.750
MG	31	41306	Medeiros	3.506
MG	31	41405	Medina	20.934
MG	31	41504	Mendes Pimentel	6.338
MG	31	41603	Mercês	10.415
MG	31	41702	Mesquita	5.963
MG	31	41801	Minas Novas	30.852
MG	31	41900	Minduri	3.841
MG	31	42007	Mirabela	13.116
MG	31	42106	Miradouro	10.324
MG	31	42205	Miraí	14.009
MG	31	42254	Miravânia	4.604
MG	31	42304	Moeda	4.723
MG	31	42403	Moema	7.106
MG	31	42502	Monjolos	2.327
MG	31	42601	Monsenhor Paulo	8.244
MG	31	42700	Montalvânia	15.631
MG	31	42809	Monte Alegre de Minas	19.863
MG	31	42908	Monte Azul	21.717
MG	31	43005	Monte Belo	13.049
MG	31	43104	Monte Carmelo	46.055
MG	31	43153	Monte Formoso	4.693
MG	31	43203	Monte Santo de Minas	21.238
MG	31	43401	Monte Sião	21.658
MG	31	43302	Montes Claros	370.216
MG	31	43450	Montezuma	7.599
MG	31	43500	Morada Nova de Minas	8.353
MG	31	43609	Morro da Garça	2.615
MG	31	43708	Morro do Pilar	3.349
MG	31	43807	Munhoz	6.197
MG	31	43906	Muriaé	102.074
MG	31	44003	Mutum	26.657
MG	31	44102	Muzambinho	20.406
MG	31	44201	Nacip Raydan	3.159
MG	31	44300	Nanuque	40.716

MG	31	44359	Naque	6.453
MG	31	44375	Natalândia	3.279
MG	31	44409	Natércia	4.661
MG	31	44508	Nazareno	8.062
MG	31	44607	Nepomuceno	25.871
MG	31	44656	Ninheira	9.885
MG	31	44672	Nova Belém	3.617
MG	31	44706	Nova Era	17.494
MG	31	44805	Nova Lima	83.507
MG	31	44904	Nova Módica	3.744
MG	31	45000	Nova Ponte	13.314
MG	31	45059	Nova Porteirinha	7.400
MG	31	45109	Nova Resende	15.599
MG	31	45208	Nova Serrana	79.174
MG	31	36603	Nova União	5.575
MG	31	45307	Novo Cruzeiro	30.767
MG	31	45356	Novo Oriente de Minas	10.395
MG	31	45372	Novorizonte	5.017
MG	31	45406	Olaria	1.927
MG	31	45455	Olhos-d'Água	5.416
MG	31	45505	Olímpio Noronha	2.577
MG	31	45604	Oliveira	39.801
MG	31	45703	Oliveira Fortes	2.120
MG	31	45802	Onça de Pitangui	3.066
MG	31	45851	Oratórios	4.514
MG	31	45877	Orizânia	7.409
MG	31	45901	Ouro Branco	36.006
MG	31	46008	Ouro Fino	31.893
MG	31	46107	Ouro Preto	70.886
MG	31	46206	Ouro Verde de Minas	5.985
MG	31	46255	Padre Carvalho	5.926
MG	31	46305	Padre Paraíso	19.057
MG	31	46552	Pai Pedro	5.950
MG	31	46404	Paineiras	4.592
MG	31	46503	Pains	8.047
MG	31	46602	Paiva	1.549
MG	31	46701	Palma	6.543
MG	31	46750	Palmópolis	6.636
MG	31	46909	Papagaios	14.433
MG	31	47105	Pará de Minas	85.908
MG	31	47006	Paracatu	86.153
MG	31	47204	Paraguaçu	20.442
MG	31	47303	Paraisópolis	19.664
MG	31	47402	Paraopeba	22.893
MG	31	47600	Passa Quatro	15.692
MG	31	47709	Passa Tempo	8.155

MG	31	47501	Passabém	1.739
MG	31	47808	Passa-Vinte	2.067
MG	31	47907	Passos	107.661
MG	31	47956	Patis	5.642
MG	31	48004	Patos de Minas	140.950
MG	31	48103	Patrocínio	83.882
MG	31	48202	Patrocínio do Muriaé	5.352
MG	31	48301	Paula Cândido	9.307
MG	31	48400	Paulistas	4.889
MG	31	48509	Pavão	8.541
MG	31	48608	Peçanha	17.272
MG	31	48707	Pedra Azul	23.874
MG	31	48756	Pedra Bonita	6.739
MG	31	48806	Pedra do Anta	3.361
MG	31	48905	Pedra do Indaiá	3.885
MG	31	49002	Pedra Dourada	2.247
MG	31	49101	Pedralva	11.386
MG	31	49150	Pedras de Maria da Cruz	10.534
MG	31	49200	Pedrinópolis	3.510
MG	31	49309	Pedro Leopoldo	59.670
MG	31	49408	Pedro Teixeira	1.785
MG	31	49507	Pequeri	3.188
MG	31	49606	Pequi	4.131
MG	31	49705	Perdigão	9.396
MG	31	49804	Perdizes	14.713
MG	31	49903	Perdões	20.292
MG	31	49952	Periquito	6.975
MG	31	50000	Pescador	4.142
MG	31	50109	Piau	2.816
MG	31	50158	Piedade de Caratinga	7.377
MG	31	50208	Piedade de Ponte Nova	4.067
MG	31	50307	Piedade do Rio Grande	4.656
MG	31	50406	Piedade dos Gerais	4.696
MG	31	50505	Pimenta	8.299
MG	31	50539	Pingo-d'Água	4.511
MG	31	50570	Pintópolis	7.251
MG	31	50604	Piracema	6.391
MG	31	50703	Pirajuba	4.946
MG	31	50802	Piranga	17.266
MG	31	50901	Piranguçu	5.254
MG	31	51008	Piranguinho	8.110
MG	31	51107	Pirapetinga	10.414
MG	31	51206	Pirapora	53.832
MG	31	51305	Piraúba	10.821
MG	31	51404	Pitangui	25.771
MG	31	51503	Piumhi	32.352

MG	31	51602	Planura	10.700
MG	31	51701	Poço Fundo	16.082
MG	31	51800	Poços de Caldas	154.974
MG	31	51909	Pocrane	8.856
MG	31	52006	Pompéu	29.561
MG	31	52105	Ponte Nova	57.706
MG	31	52131	Ponto Chique	4.014
MG	31	52170	Ponto dos Volantes	11.469
MG	31	52204	Porteirinha	37.588
MG	31	52303	Porto Firme	10.560
MG	31	52402	Poté	15.801
MG	31	52501	Pouso Alegre	134.215
MG	31	52600	Pouso Alto	6.145
MG	31	52709	Prados	8.495
MG	31	52808	Prata	26.139
MG	31	52907	Pratápolis	8.746
MG	31	53004	Pratinha	3.323
MG	31	53103	Presidente Bernardes	5.491
MG	31	53202	Presidente Juscelino	3.846
MG	31	53301	Presidente Kubitschek	2.961
MG	31	53400	Presidente Olegário	18.698
MG	31	53608	Prudente de Morais	9.776
MG	31	53707	Quartel Geral	3.346
MG	31	53806	Queluzito	1.872
MG	31	53905	Raposos	15.502
MG	31	54002	Raul Soares	23.748
MG	31	54101	Recreio	10.316
MG	31	54150	Reduto	6.667
MG	31	54200	Resende Costa	11.001
MG	31	54309	Resplendor	17.107
MG	31	54408	Ressaquinha	4.735
MG	31	54457	Riachinho	8.013
MG	31	54507	Riacho dos Machados	9.361
MG	31	54606	Ribeirão das Neves	303.029
MG	31	54705	Ribeirão Vermelho	3.857
MG	31	54804	Rio Acima	9.307
MG	31	54903	Rio Casca	14.042
MG	31	55108	Rio do Prado	5.191
MG	31	55009	Rio Doce	2.488
MG	31	55207	Rio Espera	5.939
MG	31	55306	Rio Manso	5.372
MG	31	55405	Rio Novo	8.737
MG	31	55504	Rio Paranaíba	11.939
MG	31	55603	Rio Pardo de Minas	29.381
MG	31	55702	Rio Piracicaba	14.151
MG	31	55801	Rio Pomba	17.224

MG	31	55900	Rio Preto	5.315
MG	31	56007	Rio Vermelho	13.455
MG	31	56106	Ritápolis	4.850
MG	31	56205	Rochedo de Minas	2.148
MG	31	56304	Rodeiro	7.093
MG	31	56403	Romaria	3.575
MG	31	56452	Rosário da Limeira	4.305
MG	31	56502	Rubelita	7.406
MG	31	56601	Rubim	9.958
MG	31	56700	Sabará	127.897
MG	31	56809	Sabinópolis	15.619
MG	31	56908	Sacramento	24.283
MG	31	57005	Salinas	39.550
MG	31	57104	Salto da Divisa	6.872
MG	31	57203	Santa Bárbara	28.435
MG	31	57252	Santa Bárbara do Leste	7.754
MG	31	57278	Santa Bárbara do Monte Verde	2.852
MG	31	57302	Santa Bárbara do Tugúrio	4.532
MG	31	57336	Santa Cruz de Minas	7.990
MG	31	57377	Santa Cruz de Salinas	4.336
MG	31	57401	Santa Cruz do Escalvado	4.934
MG	31	57500	Santa Efigênia de Minas	4.552
MG	31	57609	Santa Fé de Minas	3.935
MG	31	57658	Santa Helena de Minas	6.101
MG	31	57708	Santa Juliana	11.830
MG	31	57807	Santa Luzia	205.666
MG	31	57906	Santa Margarida	15.207
MG	31	58003	Santa Maria de Itabira	10.584
MG	31	58102	Santa Maria do Salto	5.261
MG	31	58201	Santa Maria do Suaçuí	14.402
MG	31	59209	Santa Rita de Caldas	8.990
MG	31	59407	Santa Rita de Ibitipoca	3.544
MG	31	59308	Santa Rita de Jacutinga	4.960
MG	31	59357	Santa Rita de Minas	6.661
MG	31	59506	Santa Rita do Itueto	5.643
MG	31	59605	Santa Rita do Sapucaí	38.734
MG	31	59704	Santa Rosa da Serra	3.241
MG	31	59803	Santa Vitória	18.406
MG	31	58300	Santana da Vargem	7.188
MG	31	58409	Santana de Cataguases	3.662
MG	31	58508	Santana de Pirapama	7.918
MG	31	58607	Santana do Deserto	3.873
MG	31	58706	Santana do Garambéu	2.273
MG	31	58805	Santana do Jacaré	4.638
MG	31	58904	Santana do Manhuaçu	8.579
MG	31	58953	Santana do Paraíso	28.641

MG	31	59001	Santana do Riacho	4.066
MG	31	59100	Santana dos Montes	3.804
MG	31	59902	Santo Antônio do Amparo	17.532
MG	31	60009	Santo Antônio do Aventureiro	3.542
MG	31	60108	Santo Antônio do Grama	4.041
MG	31	60207	Santo Antônio do Itambé	4.067
MG	31	60306	Santo Antônio do Jacinto	11.720
MG	31	60405	Santo Antônio do Monte	26.353
MG	31	60454	Santo Antônio do Retiro	7.001
MG	31	60504	Santo Antônio do Rio Abaixo	1.771
MG	31	60603	Santo Hipólito	3.201
MG	31	60702	Santos Dumont	46.208
MG	31	60801	São Bento Abade	4.704
MG	31	60900	São Brás do Suaçuí	3.548
MG	31	60959	São Domingos das Dores	5.441
MG	31	61007	São Domingos do Prata	17.314
MG	31	61056	São Félix de Minas	3.372
MG	31	61106	São Francisco	54.180
MG	31	61205	São Francisco de Paula	6.476
MG	31	61304	São Francisco de Sales	5.852
MG	31	61403	São Francisco do Glória	5.100
MG	31	61502	São Geraldo	10.648
MG	31	61601	São Geraldo da Piedade	4.295
MG	31	61650	São Geraldo do Baixio	3.580
MG	31	61700	São Gonçalo do Abaeté	6.390
MG	31	61809	São Gonçalo do Pará	10.765
MG	31	61908	São Gonçalo do Rio Abaixo	9.976
MG	31	25507	São Gonçalo do Rio Preto	3.071
MG	31	62005	São Gonçalo do Sapucaí	24.148
MG	31	62104	São Gotardo	32.452
MG	31	62203	São João Batista do Glória	6.981
MG	31	62252	São João da Lagoa	4.695
MG	31	62302	São João da Mata	2.728
MG	31	62401	São João da Ponte	25.257
MG	31	62450	São João das Missões	11.940
MG	31	62500	São João del Rei	85.353
MG	31	62559	São João do Manhuaçu	10.476
MG	31	62575	São João do Manteninha	5.307
MG	31	62609	São João do Oriente	7.781
MG	31	62658	São João do Pacuí	4.120
MG	31	62708	São João do Paraíso	22.517
MG	31	62807	São João Evangelista	15.558
MG	31	62906	São João Nepomuceno	25.249
MG	31	62922	São Joaquim de Bicas	26.653
MG	31	62948	São José da Barra	6.888
MG	31	62955	São José da Lapa	20.524

MG	31	63003	São José da Safira	4.103
MG	31	63102	São José da Varginha	4.345
MG	31	63201	São José do Alegre	4.026
MG	31	63300	São José do Divino	3.830
MG	31	63409	São José do Goiabal	5.580
MG	31	63508	São José do Jacuri	6.518
MG	31	63607	São José do Mantimento	2.625
MG	31	63706	São Lourenço	42.372
MG	31	63805	São Miguel do Anta	6.778
MG	31	63904	São Pedro da União	4.953
MG	31	64100	São Pedro do Suaçuí	5.493
MG	31	64001	São Pedro dos Ferros	8.223
MG	31	64209	São Romão	10.653
MG	31	64308	São Roque de Minas	6.741
MG	31	64407	São Sebastião da Bela Vista	5.045
MG	31	64431	São Sebastião da Vargem Alegre	2.832
MG	31	64472	São Sebastião do Anta	5.884
MG	31	64506	São Sebastião do Maranhão	10.503
MG	31	64605	São Sebastião do Oeste	5.980
MG	31	64704	São Sebastião do Paraíso	65.984
MG	31	64803	São Sebastião do Rio Preto	1.588
MG	31	64902	São Sebastião do Rio Verde	2.131
MG	31	65206	São Thomé das Letras	6.724
MG	31	65008	São Tiago	10.609
MG	31	65107	São Tomás de Aquino	7.062
MG	31	65305	São Vicente de Minas	7.136
MG	31	65404	Sapucaí-Mirim	6.360
MG	31	65503	Sardoá	5.718
MG	31	65537	Sarzedo	27.104
MG	31	65560	Sem-Peixe	2.799
MG	31	65578	Senador Amaral	5.233
MG	31	65602	Senador Cortes	1.987
MG	31	65701	Senador Firmino	7.326
MG	31	65800	Senador José Bento	1.793
MG	31	65909	Senador Modestino Gonçalves	4.481
MG	31	66006	Senhora de Oliveira	5.690
MG	31	66105	Senhora do Porto	3.494
MG	31	66204	Senhora dos Remédios	10.222
MG	31	66303	Sericita	7.149
MG	31	66402	Seritinga	1.797
MG	31	66501	Serra Azul de Minas	4.224
MG	31	66600	Serra da Saudade	807
MG	31	66808	Serra do Salitre	10.725
MG	31	66709	Serra dos Aimorés	8.447
MG	31	66907	Serrania	7.548
MG	31	66956	Serranópolis de Minas	4.484

MG	31	67004	Serranos	1.984
MG	31	67103	Serro	20.809
MG	31	67202	Sete Lagoas	218.574
MG	31	65552	Setubinha	11.126
MG	31	67301	Silveirânia	2.201
MG	31	67400	Silvianópolis	6.053
MG	31	67509	Simão Pereira	2.546
MG	31	67608	Simonésia	18.513
MG	31	67707	Sobrália	5.762
MG	31	67806	Soledade de Minas	5.755
MG	31	67905	Tabuleiro	4.005
MG	31	68002	Taiobeiras	31.457
MG	31	68051	Taparuba	3.124
MG	31	68101	Tapira	4.231
MG	31	68200	Tapiraí	1.869
MG	31	68309	Taquaraçu de Minas	3.840
MG	31	68408	Tarumirim	14.264
MG	31	68507	Teixeiras	11.387
MG	31	68606	Teófilo Otoni	135.549
MG	31	68705	Timóteo	82.718
MG	31	68804	Tiradentes	7.143
MG	31	68903	Tiros	6.806
MG	31	69000	Tocantins	15.947
MG	31	69059	Tocos do Moji	3.970
MG	31	69109	Toledo	5.846
MG	31	69208	Tombos	9.218
MG	31	69307	Três Corações	73.894
MG	31	69356	Três Marias	29.036
MG	31	69406	Três Pontas	54.289
MG	31	69505	Tumiritinga	6.363
MG	31	69604	Tupaciguara	24.350
MG	31	69703	Turmalina	18.383
MG	31	69802	Turvolândia	4.721
MG	31	69901	Ubá	104.004
MG	31	70008	Ubaí	11.818
MG	31	70057	Ubaporanga	12.095
MG	31	70107	Uberaba	302.623
MG	31	70206	Uberlândia	619.536
MG	31	70305	Umburatiba	2.680
MG	31	70404	Unaí	78.703
MG	31	70438	União de Minas	4.385
MG	31	70479	Uruana de Minas	3.231
MG	31	70503	Urucânia	10.279
MG	31	70529	Urucuia	14.207
MG	31	70578	Vargem Alegre	6.449
MG	31	70602	Vargem Bonita	2.156

MG	31	70651	Vargem Grande do Rio Pardo	4.775
MG	31	70701	Varginha	125.208
MG	31	70750	Varjão de Minas	6.259
MG	31	70800	Várzea da Palma	36.439
MG	31	70909	Varzelândia	19.108
MG	31	71006	Vazante	19.844
MG	31	71030	Verdelândia	8.523
MG	31	71071	Veredinha	5.569
MG	31	71105	Veríssimo	3.575
MG	31	71154	Vermelho Novo	4.707
MG	31	71204	Vespasiano	108.771
MG	31	71303	Viçosa	73.333
MG	31	71402	Vieiras	3.698
MG	31	71600	Virgem da Lapa	13.611
MG	31	71709	Virgínia	8.612
MG	31	71808	Virginópolis	10.534
MG	31	71907	Virgolândia	5.590
MG	31	72004	Visconde do Rio Branco	38.749
MG	31	72103	Volta Grande	5.093
MG	31	72202	Wenceslau Braz	2.547
ES	32	00102	Afonso Cláudio	30.919
ES	32	00169	Água Doce do Norte	11.624
ES	32	00136	Águia Branca	9.507
ES	32	00201	Alegre	30.626
ES	32	00300	Alfredo Chaves	14.007
ES	32	00359	Alto Rio Novo	7.371
ES	32	00409	Anchieta	24.616
ES	32	00508	Apiacá	7.497
ES	32	00607	Aracruz	84.429
ES	32	00706	Atilio Vivacqua	10.080
ES	32	00805	Baixo Guandu	29.272
ES	32	00904	Barra de São Francisco	41.110
ES	32	01001	Boa Esperança	14.278
ES	32	01100	Bom Jesus do Norte	9.514
ES	32	01159	Brejetuba	11.950
ES	32	01209	Cachoeiro de Itapemirim	192.156
ES	32	01308	Cariacica	352.431
ES	32	01407	Castelo	35.048
ES	32	01506	Colatina	113.054
ES	32	01605	Conceição da Barra	28.745
ES	32	01704	Conceição do Castelo	11.798
ES	32	01803	Divino de São Lourenço	4.471
ES	32	01902	Domingos Martins	32.042
ES	32	02009	Dores do Rio Preto	6.429
ES	32	02108	Ecoporanga	23.097
ES	32	02207	Fundão	17.632

ES	32	02256	Governador Lindenberg	11.106
ES	32	02306	Guaçuí	28.208
ES	32	02405	Guarapari	107.836
ES	32	02454	Ibatiba	22.843
ES	32	02504	Ibiraçu	11.335
ES	32	02553	Ibitirama	8.919
ES	32	02603	Iconha	12.681
ES	32	02652	Irupi	11.930
ES	32	02702	Itaguaçu	14.080
ES	32	02801	Itapemirim	31.421
ES	32	02900	Itarana	10.799
ES	32	03007	Iúna	27.512
ES	32	03056	Jaguaré	25.454
ES	32	03106	Jerônimo Monteiro	10.984
ES	32	03130	João Neiva	15.886
ES	32	03163	Laranja da Terra	10.810
ES	32	03205	Linhares	145.639
ES	32	03304	Mantenópolis	13.826
ES	32	03320	Marataízes	34.675
ES	32	03346	Marechal Floriano	14.576
ES	32	03353	Marilândia	11.286
ES	32	03403	Mimoso do Sul	25.858
ES	32	03502	Montanha	17.938
ES	32	03601	Mucurici	5.619
ES	32	03700	Muniz Freire	18.202
ES	32	03809	Muqui	14.506
ES	32	03908	Nova Venécia	46.487
ES	32	04005	Pancas	21.722
ES	32	04054	Pedro Canário	24.071
ES	32	04104	Pinheiros	24.284
ES	32	04203	Piúma	18.597
ES	32	04252	Ponto Belo	7.088
ES	32	04302	Presidente Kennedy	10.429
ES	32	04351	Rio Bananal	17.713
ES	32	04401	Rio Novo do Sul	11.334
ES	32	04500	Santa Leopoldina	12.207
ES	32	04559	Santa Maria de Jetibá	34.992
ES	32	04609	Santa Teresa	22.005
ES	32	04658	São Domingos do Norte	8.070
ES	32	04708	São Gabriel da Palha	32.655
ES	32	04807	São José do Calçado	10.397
ES	32	04906	São Mateus	111.832
ES	32	04955	São Roque do Canaã	11.406
ES	32	05002	Serra	422.569
ES	32	05010	Sooretama	24.685
ES	32	05036	Vargem Alta	19.395

ES	32	05069	Venda Nova do Imigrante	21.094
ES	32	05101	Viana	66.745
ES	32	05150	Vila Pavão	8.724
ES	32	05176	Vila Valério	13.824
ES	32	05200	Vila Velha	424.948
ES	32	05309	Vitória	333.162
RJ	33	00100	Angra dos Reis	177.101
RJ	33	00159	Aperibé	10.545
RJ	33	00209	Araruama	116.418
RJ	33	00225	Areal	11.654
RJ	33	00233	Armação dos Búzios	28.973
RJ	33	00258	Arraial do Cabo	28.295
RJ	33	00308	Barra do Piraí	95.726
RJ	33	00407	Barra Mansa	178.880
RJ	33	00456	Belford Roxo	474.596
RJ	33	00506	Bom Jardim	25.738
RJ	33	00605	Bom Jesus do Itabapoana	35.677
RJ	33	00704	Cabo Frio	195.197
RJ	33	00803	Cachoeiras de Macacu	55.139
RJ	33	00902	Cambuci	14.851
RJ	33	01009	Campos dos Goytacazes	472.300
RJ	33	01108	Cantagalo	19.830
RJ	33	00936	Carapebus	14.024
RJ	33	01157	Cardoso Moreira	12.601
RJ	33	01207	Carmo	17.758
RJ	33	01306	Casimiro de Abreu	37.340
RJ	33	00951	Comendador Levy Gasparian	8.219
RJ	33	01405	Conceição de Macabu	21.613
RJ	33	01504	Cordeiro	20.707
RJ	33	01603	Duas Barras	11.020
RJ	33	01702	Duque de Caxias	867.067
RJ	33	01801	Engenheiro Paulo de Frontin	13.408
RJ	33	01850	Guapimirim	53.527
RJ	33	01876	Iguaba Grande	24.079
RJ	33	01900	Itaboraí	222.618
RJ	33	02007	Itaguaí	113.182
RJ	33	02056	Italva	14.281
RJ	33	02106	Itaocara	22.884
RJ	33	02205	Itaperuna	97.219
RJ	33	02254	Itatiaia	29.394
RJ	33	02270	Japeri	97.337
RJ	33	02304	Laje do Muriaé	7.424
RJ	33	02403	Macaé	217.951
RJ	33	02452	Macuco	5.327
RJ	33	02502	Magé	230.568
RJ	33	02601	Mangaratiba	38.201

RJ	33	02700	Maricá	135.121
RJ	33	02809	Mendes	18.024
RJ	33	02858	Mesquita	169.537
RJ	33	02908	Miguel Pereira	24.754
RJ	33	03005	Miracema	26.810
RJ	33	03104	Natividade	15.076
RJ	33	03203	Nilópolis	157.986
RJ	33	03302	Niterói	491.807
RJ	33	03401	Nova Friburgo	183.391
RJ	33	03500	Nova Iguaçu	801.746
RJ	33	03609	Paracambi	48.129
RJ	33	03708	Paraíba do Sul	41.639
RJ	33	03807	Parati	38.740
RJ	33	03856	Paty do Alferes	26.575
RJ	33	03906	Petrópolis	297.192
RJ	33	03955	Pinheiral	23.208
RJ	33	04003	Piraí	26.948
RJ	33	04102	Porciúncula	18.034
RJ	33	04110	Porto Real	17.272
RJ	33	04128	Quatis	13.105
RJ	33	04144	Queimados	140.374
RJ	33	04151	Quissamã	21.234
RJ	33	04201	Resende	122.068
RJ	33	04300	Rio Bonito	56.436
RJ	33	04409	Rio Claro	17.606
RJ	33	04508	Rio das Flores	8.703
RJ	33	04524	Rio das Ostras	116.134
RJ	33	04557	Rio de Janeiro	6.390.290
RJ	33	04607	Santa Maria Madalena	10.298
RJ	33	04706	Santo Antônio de Pádua	40.876
RJ	33	04805	São Fidélis	37.657
RJ	33	04755	São Francisco de Itabapoana	41.386
RJ	33	04904	São Gonçalo	1.016.128
RJ	33	05000	São João da Barra	33.512
RJ	33	05109	São João de Meriti	460.062
RJ	33	05133	São José de Ubá	7.093
RJ	33	05158	São José do Vale do Rio Preto	20.540
RJ	33	05208	São Pedro da Aldeia	91.542
RJ	33	05307	São Sebastião do Alto	8.970
RJ	33	05406	Sapucaia	17.581
RJ	33	05505	Saquarema	77.522
RJ	33	05554	Seropédica	80.138
RJ	33	05604	Silva Jardim	21.362
RJ	33	05703	Sumidouro	15.010
RJ	33	05752	Tanguá	31.438
RJ	33	05802	Teresópolis	167.622

RJ	33	05901	Trajano de Morais	10.327
RJ	33	06008	Três Rios	78.256
RJ	33	06107	Valença	72.679
RJ	33	06156	Varre-Sai	9.720
RJ	33	06206	Vassouras	34.858
RJ	33	06305	Volta Redonda	260.180
SP	35	00105	Adamantina	33.843
SP	35	00204	Adolfo	3.538
SP	35	00303	Aguaí	32.745
SP	35	00402	Águas da Prata	7.653
SP	35	00501	Águas de Lindóia	17.438
SP	35	00550	Águas de Santa Bárbara	5.658
SP	35	00600	Águas de São Pedro	2.832
SP	35	00709	Agudos	34.833
SP	35	00758	Alambari	5.071
SP	35	00808	Alfredo Marcondes	3.921
SP	35	00907	Altair	3.859
SP	35	01004	Altinópolis	15.627
SP	35	01103	Alto Alegre	4.078
SP	35	01152	Alumínio	17.079
SP	35	01202	Álvares Florence	3.834
SP	35	01301	Álvares Machado	23.642
SP	35	01400	Álvaro de Carvalho	4.732
SP	35	01509	Alvinlândia	3.025
SP	35	01608	Americana	214.873
SP	35	01707	Américo Brasiliense	35.413
SP	35	01806	Américo de Campos	5.723
SP	35	01905	Amparo	66.649
SP	35	02002	Analândia	4.401
SP	35	02101	Andradina	55.361
SP	35	02200	Angatuba	22.650
SP	35	02309	Anhembi	5.822
SP	35	02408	Anhumas	3.788
SP	35	02507	Aparecida	35.023
SP	35	02606	Aparecida d'Oeste	4.377
SP	35	02705	Apiaí	24.894
SP	35	02754	Araçariguama	17.975
SP	35	02804	Araçatuba	183.441
SP	35	02903	Araçoiaba da Serra	28.429
SP	35	03000	Aramina	5.211
SP	35	03109	Arandu	6.132
SP	35	03158	Arapeí	2.475
SP	35	03208	Araraquara	212.617
SP	35	03307	Araras	121.055
SP	35	03356	Arco-Íris	1.890
SP	35	03406	Arealva	7.932

SP	35	03505	Areias	3.711
SP	35	03604	Areiópolis	10.622
SP	35	03703	Ariranha	8.709
SP	35	03802	Artur Nogueira	45.847
SP	35	03901	Arujá	77.279
SP	35	03950	Aspásia	1.802
SP	35	04008	Assis	96.336
SP	35	04107	Atibaia	128.914
SP	35	04206	Auriflama	14.307
SP	35	04305	Avaí	5.014
SP	35	04404	Avanhandava	11.685
SP	35	04503	Avaré	83.910
SP	35	04602	Bady Bassitt	15.065
SP	35	04701	Balbinos	4.063
SP	35	04800	Bálsamo	8.284
SP	35	04909	Bananal	10.301
SP	35	05005	Barão de Antonina	3.165
SP	35	05104	Barbosa	6.708
SP	35	05203	Bariri	32.102
SP	35	05302	Barra Bonita	35.210
SP	35	05351	Barra do Chapéu	5.305
SP	35	05401	Barra do Turvo	7.672
SP	35	05500	Barretos	113.338
SP	35	05609	Barrinha	29.144
SP	35	05708	Barueri	245.652
SP	35	05807	Bastos	20.424
SP	35	05906	Batatais	57.286
SP	35	06003	Bauru	348.146
SP	35	06102	Bebedouro	75.069
SP	35	06201	Bento de Abreu	2.717
SP	35	06300	Bernardino de Campos	10.784
SP	35	06359	Bertioga	50.304
SP	35	06409	Bilac	7.193
SP	35	06508	Birigui	110.907
SP	35	06607	Biritiba-Mirim	29.168
SP	35	06706	Boa Esperança do Sul	13.807
SP	35	06805	Bocaina	11.073
SP	35	06904	Bofete	9.960
SP	35	07001	Boituva	50.420
SP	35	07100	Bom Jesus dos Perdões	20.674
SP	35	07159	Bom Sucesso de Itararé	3.623
SP	35	07209	Borá	807
SP	35	07308	Boracéia	4.348
SP	35	07407	Borborema	14.731
SP	35	07456	Borebi	2.348
SP	35	07506	Botucatu	130.201

SP	35	07605	Bragança Paulista	150.023
SP	35	07704	Braúna	5.118
SP	35	07753	Brejo Alegre	2.614
SP	35	07803	Brodowski	21.707
SP	35	07902	Brotas	21.987
SP	35	08009	Buri	18.705
SP	35	08108	Buritama	15.655
SP	35	08207	Buritizal	4.111
SP	35	08306	Cabrália Paulista	4.322
SP	35	08405	Cabreúva	42.889
SP	35	08504	Caçapava	86.054
SP	35	08603	Cachoeira Paulista	30.527
SP	35	08702	Caconde	18.563
SP	35	08801	Cafelândia	16.730
SP	35	08900	Caiabu	4.072
SP	35	09007	Caieiras	88.841
SP	35	09106	Caiuá	5.167
SP	35	09205	Cajamar	66.131
SP	35	09254	Cajati	28.243
SP	35	09304	Cajobi	9.858
SP	35	09403	Cajuru	23.763
SP	35	09452	Campina do Monte Alegre	5.622
SP	35	09502	Campinas	1.098.630
SP	35	09601	Campo Limpo Paulista	75.637
SP	35	09700	Campos do Jordão	48.324
SP	35	09809	Campos Novos Paulista	4.594
SP	35	09908	Cananéia	12.216
SP	35	09957	Canas	4.502
SP	35	10005	Cândido Mota	29.976
SP	35	10104	Cândido Rodrigues	2.677
SP	35	10153	Canitar	4.504
SP	35	10203	Capão Bonito	46.095
SP	35	10302	Capela do Alto	18.029
SP	35	10401	Capivari	49.650
SP	35	10500	Caraguatatuba	104.150
SP	35	10609	Carapicuíba	373.358
SP	35	10708	Cardoso	11.836
SP	35	10807	Casa Branca	28.535
SP	35	10906	Cássia dos Coqueiros	2.599
SP	35	11003	Castilho	18.465
SP	35	11102	Catanduva	113.873
SP	35	11201	Catiguá	7.214
SP	35	11300	Cedral	8.165
SP	35	11409	Cerqueira César	17.893
SP	35	11508	Cerquilho	41.144
SP	35	11607	Cesário Lange	15.942

SP	35	11706	Charqueada	15.395
SP	35	57204	Chavantes	12.102
SP	35	11904	Clementina	7.316
SP	35	12001	Colina	17.478
SP	35	12100	Colômbia	6.001
SP	35	12209	Conchal	25.615
SP	35	12308	Conchas	16.497
SP	35	12407	Cordeirópolis	21.607
SP	35	12506	Coroados	5.362
SP	35	12605	Coronel Macedo	4.913
SP	35	12704	Corumbataí	3.887
SP	35	12803	Cosmópolis	61.013
SP	35	12902	Cosmorama	7.191
SP	35	13009	Cotia	209.027
SP	35	13108	Cravinhos	32.187
SP	35	13207	Cristais Paulista	7.741
SP	35	13306	Cruzália	2.224
SP	35	13405	Cruzeiro	77.575
SP	35	13504	Cubatão	120.293
SP	35	13603	Cunha	21.682
SP	35	13702	Descalvado	31.379
SP	35	13801	Diadema	390.980
SP	35	13850	Dirce Reis	1.699
SP	35	13900	Divinolândia	11.086
SP	35	14007	Dobrada	8.080
SP	35	14106	Dois Córregos	25.100
SP	35	14205	Dolcinópolis	2.088
SP	35	14304	Dourado	8.610
SP	35	14403	Dracena	43.675
SP	35	14502	Duartina	12.218
SP	35	14601	Dumont	8.421
SP	35	14700	Echaporã	6.242
SP	35	14809	Eldorado	14.718
SP	35	14908	Elias Fausto	16.060
SP	35	14924	Elisiário	3.202
SP	35	14957	Embaúba	2.415
SP	35	15004	Embu	245.148
SP	35	15103	Embu-Guaçu	63.653
SP	35	15129	Emilianópolis	3.040
SP	35	15152	Engenheiro Coelho	16.580
SP	35	15186	Espírito Santo do Pinhal	42.123
SP	35	15194	Espírito Santo do Turvo	4.330
SP	35	57303	Estiva Gerbi	10.224
SP	35	15301	Estrela do Norte	2.663
SP	35	15202	Estrela d'Oeste	8.201
SP	35	15350	Euclides da Cunha Paulista	9.491

SP	35	15400	Fartura	15.367
SP	35	15608	Fernando Prestes	5.550
SP	35	15509	Fernandópolis	65.157
SP	35	15657	Fernão	1.583
SP	35	15707	Ferraz de Vasconcelos	172.222
SP	35	15806	Flora Rica	1.688
SP	35	15905	Floreal	2.970
SP	35	16002	Flórida Paulista	13.112
SP	35	16101	Florínia	2.785
SP	35	16200	Franca	323.307
SP	35	16309	Francisco Morato	157.603
SP	35	16408	Franco da Rocha	135.150
SP	35	16507	Gabriel Monteiro	2.706
SP	35	16606	Gália	6.884
SP	35	16705	Garça	43.108
SP	35	16804	Gastão Vidigal	4.285
SP	35	16853	Gavião Peixoto	4.464
SP	35	16903	General Salgado	10.646
SP	35	17000	Getulina	10.825
SP	35	17109	Glicério	4.586
SP	35	17208	Guaiçara	10.891
SP	35	17307	Guaimbê	5.458
SP	35	17406	Guaíra	37.826
SP	35	17505	Guapiaçu	18.441
SP	35	17604	Guapiara	17.738
SP	35	17703	Guará	20.001
SP	35	17802	Guaraçaí	8.366
SP	35	17901	Guaraci	10.147
SP	35	18008	Guarani d'Oeste	1.965
SP	35	18107	Guarantã	6.417
SP	35	18206	Guararapes	30.862
SP	35	18305	Guararema	26.439
SP	35	18404	Guaratinguetá	113.258
SP	35	18503	Guareí	15.225
SP	35	18602	Guariba	36.151
SP	35	18701	Guarujá	294.669
SP	35	18800	Guarulhos	1.244.518
SP	35	18859	Guatapará	7.056
SP	35	18909	Guzolândia	4.824
SP	35	19006	Herculândia	8.803
SP	35	19055	Holambra	11.917
SP	35	19071	Hortolândia	198.758
SP	35	19105	Iacanga	10.275
SP	35	19204	Iacri	6.365
SP	35	19253	Iaras	6.878
SP	35	19303	Ibaté	31.380

SP	35	19402	Ibirá	11.115
SP	35	19501	Ibirarema	6.880
SP	35	19600	Ibitinga	54.146
SP	35	19709	Ibiúna	72.249
SP	35	19808	Icém	7.567
SP	35	19907	Iepê	7.685
SP	35	20004	Igaraçu do Tietê	23.475
SP	35	20103	Igarapava	28.259
SP	35	20202	Igaratá	8.913
SP	35	20301	Iguape	29.055
SP	35	20426	Ilha Comprida	9.376
SP	35	20442	Ilha Solteira	25.226
SP	35	20400	Ilhabela	29.308
SP	35	20509	Indaiatuba	209.859
SP	35	20608	Indiana	4.809
SP	35	20707	Indiaporã	3.880
SP	35	20806	Inúbia Paulista	3.678
SP	35	20905	Ipaussu	13.831
SP	35	21002	Iperó	29.798
SP	35	21101	Ipeúna	6.270
SP	35	21150	Ipiguá	4.613
SP	35	21200	Iporanga	4.260
SP	35	21309	Ipuã	14.492
SP	35	21408	Iracemápolis	20.705
SP	35	21507	Irapuã	7.369
SP	35	21606	Irapuru	7.840
SP	35	21705	Itaberá	17.699
SP	35	21804	Itaí	24.457
SP	35	21903	Itajobi	14.606
SP	35	22000	Itaju	3.338
SP	35	22109	Itanhaém	89.332
SP	35	22158	Itaóca	3.229
SP	35	22208	Itapecerica da Serra	156.077
SP	35	22307	Itapetininga	147.219
SP	35	22406	Itapeva	88.491
SP	35	22505	Itapevi	206.558
SP	35	22604	Itapira	69.317
SP	35	22653	Itapirapuã Paulista	3.926
SP	35	22703	Itápolis	40.399
SP	35	22802	Itaporanga	14.579
SP	35	22901	Itapuí	12.446
SP	35	23008	Itapura	4.436
SP	35	23107	Itaquaquecetuba	329.144
SP	35	23206	Itararé	48.143
SP	35	23305	Itariri	15.752
SP	35	23404	Itatiba	104.533

SP	35	23503	Itatinga	18.446
SP	35	23602	Itirapina	15.930
SP	35	23701	Itirapuã	5.990
SP	35	23800	Itobi	7.559
SP	35	23909	Itu	156.983
SP	35	24006	Itupeva	47.682
SP	35	24105	Ituverava	39.062
SP	35	24204	Jaborandi	6.618
SP	35	24303	Jaboticabal	72.305
SP	35	24402	Jacareí	214.223
SP	35	24501	Jaci	5.890
SP	35	24600	Jacupiranga	17.234
SP	35	24709	Jaguariúna	46.533
SP	35	24808	Jales	47.137
SP	35	24907	Jambeiro	5.554
SP	35	25003	Jandira	110.842
SP	35	25102	Jardinópolis	38.708
SP	35	25201	Jarinu	24.875
SP	35	25300	Jaú	133.900
SP	35	25409	Jeriquara	3.142
SP	35	25508	Joanópolis	11.974
SP	35	25607	João Ramalho	4.197
SP	35	25706	José Bonifácio	33.375
SP	35	25805	Júlio Mesquita	4.470
SP	35	25854	Jumirim	2.889
SP	35	25904	Jundiaí	377.183
SP	35	26001	Junqueirópolis	18.986
SP	35	26100	Juquiá	19.055
SP	35	26209	Juquitiba	29.081
SP	35	26308	Lagoinha	4.824
SP	35	26407	Laranjal Paulista	25.721
SP	35	26506	Lavínia	9.330
SP	35	26605	Lavrinhas	6.678
SP	35	26704	Leme	93.417
SP	35	26803	Lençóis Paulista	62.393
SP	35	26902	Limeira	280.096
SP	35	27009	Lindóia	6.912
SP	35	27108	Lins	72.260
SP	35	27207	Lorena	83.224
SP	35	27256	Lourdes	2.147
SP	35	27306	Louveira	39.122
SP	35	27405	Lucélia	20.119
SP	35	27504	Lucianópolis	2.264
SP	35	27603	Luís Antônio	11.910
SP	35	27702	Luiziânia	5.145
SP	35	27801	Lupércio	4.372

SP	35	27900	Lutécia	2.687
SP	35	28007	Macatuba	16.336
SP	35	28106	Macaubal	7.705
SP	35	28205	Macedônia	3.650
SP	35	28304	Magda	3.167
SP	35	28403	Mairinque	43.714
SP	35	28502	Mairiporã	84.104
SP	35	28601	Manduri	9.101
SP	35	28700	Marabá Paulista	4.981
SP	35	28809	Maracaí	13.382
SP	35	28858	Marapoama	2.693
SP	35	28908	Mariápolis	3.926
SP	35	29005	Marília	219.664
SP	35	29104	Marinópolis	2.101
SP	35	29203	Martinópolis	24.502
SP	35	29302	Matão	77.546
SP	35	29401	Mauá	425.169
SP	35	29500	Mendonça	4.774
SP	35	29609	Meridiano	3.830
SP	35	29658	Mesópolis	1.880
SP	35	29708	Miguelópolis	20.668
SP	35	29807	Mineiros do Tietê	12.133
SP	35	30003	Mira Estrela	2.854
SP	35	29906	Miracatu	20.322
SP	35	30102	Mirandópolis	27.717
SP	35	30201	Mirante do Paranapanema	17.187
SP	35	30300	Mirassol	54.618
SP	35	30409	Mirassolândia	4.379
SP	35	30508	Mococa	66.399
SP	35	30607	Mogi das Cruzes	396.468
SP	35	30706	Mogi Guaçu	139.211
SP	35	30805	Moji Mirim	87.266
SP	35	30904	Mombuca	3.291
SP	35	31001	Monções	2.144
SP	35	31100	Mongaguá	47.984
SP	35	31209	Monte Alegre do Sul	7.278
SP	35	31308	Monte Alto	47.100
SP	35	31407	Monte Aprazível	22.250
SP	35	31506	Monte Azul Paulista	18.838
SP	35	31605	Monte Castelo	4.060
SP	35	31803	Monte Mor	50.702
SP	35	31704	Monteiro Lobato	4.197
SP	35	31902	Morro Agudo	29.673
SP	35	32009	Morungaba	12.050
SP	35	32058	Motuca	4.354
SP	35	32108	Murutinga do Sul	4.219

SP	35	32157	Nantes	2.774
SP	35	32207	Narandiba	4.371
SP	35	32306	Natividade da Serra	6.637
SP	35	32405	Nazaré Paulista	16.717
SP	35	32504	Neves Paulista	8.752
SP	35	32603	Nhandeara	10.806
SP	35	32702	Nipoã	4.427
SP	35	32801	Nova Aliança	6.061
SP	35	32827	Nova Campina	8.700
SP	35	32843	Nova Canaã Paulista	2.059
SP	35	32868	Nova Castilho	1.146
SP	35	32900	Nova Europa	9.601
SP	35	33007	Nova Granada	19.507
SP	35	33106	Nova Guataporanga	2.191
SP	35	33205	Nova Independência	3.220
SP	35	33304	Nova Luzitânia	3.546
SP	35	33403	Nova Odessa	52.627
SP	35	33254	Novais	4.799
SP	35	33502	Novo Horizonte	37.222
SP	35	33601	Nuporanga	6.894
SP	35	33700	Ocauçu	4.163
SP	35	33809	Óleo	2.625
SP	35	33908	Olímpia	50.630
SP	35	34005	Onda Verde	3.956
SP	35	34104	Oriente	6.141
SP	35	34203	Orindiúva	5.904
SP	35	34302	Orlândia	40.352
SP	35	34401	Osasco	668.877
SP	35	34500	Oscar Bressane	2.535
SP	35	34609	Osvaldo Cruz	31.109
SP	35	34708	Ourinhos	104.420
SP	35	34807	Ouro Verde	7.899
SP	35	34757	Ouroeste	8.725
SP	35	34906	Pacaembu	13.333
SP	35	35002	Palestina	11.346
SP	35	35101	Palmares Paulista	11.312
SP	35	35200	Palmeira d'Oeste	9.473
SP	35	35309	Palmital	21.260
SP	35	35408	Panorama	14.725
SP	35	35507	Paraguaçu Paulista	42.680
SP	35	35606	Paraibuna	17.446
SP	35	35705	Paraíso	5.969
SP	35	35804	Paranapanema	18.155
SP	35	35903	Paranapuã	3.843
SP	35	36000	Parapuã	10.805
SP	35	36109	Pardinho	5.711

SP	35	36208	Pariquera-Açu	18.567
SP	35	36257	Parisi	2.045
SP	35	36307	Patrocínio Paulista	13.240
SP	35	36406	Paulicéia	6.496
SP	35	36505	Paulínia	86.800
SP	35	36570	Paulistânia	1.779
SP	35	36604	Paulo de Faria	8.607
SP	35	36703	Pederneiras	42.235
SP	35	36802	Pedra Bela	5.806
SP	35	36901	Pedranópolis	2.532
SP	35	37008	Pedregulho	15.807
SP	35	37107	Pedreira	42.516
SP	35	37156	Pedrinhas Paulista	2.952
SP	35	37206	Pedro de Toledo	10.358
SP	35	37305	Penápolis	59.096
SP	35	37404	Pereira Barreto	24.953
SP	35	37503	Pereiras	7.640
SP	35	37602	Peruíbe	61.030
SP	35	37701	Piacatu	5.387
SP	35	37800	Piedade	52.447
SP	35	37909	Pilar do Sul	26.778
SP	35	38006	Pindamonhangaba	150.162
SP	35	38105	Pindorama	15.331
SP	35	38204	Pinhalzinho	13.425
SP	35	38303	Piquerobi	3.546
SP	35	38501	Piquete	13.942
SP	35	38600	Piracaia	25.384
SP	35	38709	Piracicaba	369.919
SP	35	38808	Piraju	28.563
SP	35	38907	Pirajuí	23.098
SP	35	39004	Pirangi	10.712
SP	35	39103	Pirapora do Bom Jesus	16.238
SP	35	39202	Pirapozinho	25.086
SP	35	39301	Pirassununga	70.869
SP	35	39400	Piratininga	12.297
SP	35	39509	Pitangueiras	35.934
SP	35	39608	Planalto	4.583
SP	35	39707	Platina	3.242
SP	35	39806	Poá	107.556
SP	35	39905	Poloni	5.489
SP	35	40002	Pompéia	20.235
SP	35	40101	Pongaí	3.449
SP	35	40200	Pontal	41.840
SP	35	40259	Pontalinda	4.155
SP	35	40309	Pontes Gestal	2.515
SP	35	40408	Populina	4.189

SP	35	40507	Porangaba	8.579
SP	35	40606	Porto Feliz	49.404
SP	35	40705	Porto Ferreira	51.999
SP	35	40754	Potim	20.272
SP	35	40804	Potirendaba	15.720
SP	35	40853	Pracinha	3.074
SP	35	40903	Pradópolis	18.052
SP	35	41000	Praia Grande	272.390
SP	35	41059	Pratânia	4.697
SP	35	41109	Presidente Alves	4.094
SP	35	41208	Presidente Bernardes	13.406
SP	35	41307	Presidente Epitácio	41.624
SP	35	41406	Presidente Prudente	210.393
SP	35	41505	Presidente Venceslau	37.996
SP	35	41604	Promissão	36.364
SP	35	41653	Quadra	3.325
SP	35	41703	Quatá	12.972
SP	35	41802	Queiroz	2.905
SP	35	41901	Queluz	11.641
SP	35	42008	Quintana	6.089
SP	35	42107	Rafard	8.651
SP	35	42206	Rancharia	28.809
SP	35	42305	Redenção da Serra	3.847
SP	35	42404	Regente Feijó	18.720
SP	35	42503	Reginópolis	7.713
SP	35	42602	Registro	54.338
SP	35	42701	Restinga	6.739
SP	35	42800	Ribeira	3.336
SP	35	42909	Ribeirão Bonito	12.270
SP	35	43006	Ribeirão Branco	17.822
SP	35	43105	Ribeirão Corrente	4.333
SP	35	43204	Ribeirão do Sul	4.439
SP	35	43238	Ribeirão dos Índios	2.182
SP	35	43253	Ribeirão Grande	7.427
SP	35	43303	Ribeirão Pires	114.361
SP	35	43402	Ribeirão Preto	619.746
SP	35	43600	Rifaina	3.453
SP	35	43709	Rincão	10.427
SP	35	43808	Rinópolis	9.887
SP	35	43907	Rio Claro	188.977
SP	35	44004	Rio das Pedras	30.409
SP	35	44103	Rio Grande da Serra	45.014
SP	35	44202	Riolândia	10.880
SP	35	43501	Riversul	6.008
SP	35	44251	Rosana	19.006
SP	35	44301	Roseira	9.754

SP	35	44400	Rubiácea	2.789
SP	35	44509	Rubinéia	2.900
SP	35	44608	Sabino	5.258
SP	35	44707	Sagres	2.389
SP	35	44806	Sales	5.586
SP	35	44905	Sales Oliveira	10.756
SP	35	45001	Salesópolis	15.828
SP	35	45100	Salmourão	4.881
SP	35	45159	Saltinho	7.250
SP	35	45209	Salto	107.382
SP	35	45308	Salto de Pirapora	40.897
SP	35	45407	Salto Grande	8.839
SP	35	45506	Sandovalina	3.792
SP	35	45605	Santa Adélia	14.467
SP	35	45704	Santa Albertina	5.744
SP	35	45803	Santa Bárbara d'Oeste	181.509
SP	35	46009	Santa Branca	13.877
SP	35	46108	Santa Clara d'Oeste	2.079
SP	35	46207	Santa Cruz da Conceição	4.074
SP	35	46256	Santa Cruz da Esperança	1.977
SP	35	46306	Santa Cruz das Palmeiras	30.593
SP	35	46405	Santa Cruz do Rio Pardo	44.375
SP	35	46504	Santa Ernestina	5.542
SP	35	46603	Santa Fé do Sul	29.651
SP	35	46702	Santa Gertrudes	22.499
SP	35	46801	Santa Isabel	51.467
SP	35	46900	Santa Lúcia	8.308
SP	35	47007	Santa Maria da Serra	5.525
SP	35	47106	Santa Mercedes	2.836
SP	35	47502	Santa Rita do Passa Quatro	26.530
SP	35	47403	Santa Rita d'Oeste	2.521
SP	35	47601	Santa Rosa de Viterbo	24.229
SP	35	47650	Santa Salete	1.458
SP	35	47205	Santana da Ponte Pensa	1.603
SP	35	47304	Santana de Parnaíba	113.945
SP	35	47700	Santo Anastácio	20.434
SP	35	47809	Santo André	680.496
SP	35	47908	Santo Antônio da Alegria	6.386
SP	35	48005	Santo Antônio de Posse	21.032
SP	35	48054	Santo Antônio do Aracanguá	7.732
SP	35	48104	Santo Antônio do Jardim	5.912
SP	35	48203	Santo Antônio do Pinhal	6.510
SP	35	48302	Santo Expedito	2.845
SP	35	48401	Santópolis do Aguapeí	4.347
SP	35	48500	Santos	419.614
SP	35	48609	São Bento do Sapucaí	10.486

SP	35	48708	São Bernardo do Campo	774.886
SP	35	48807	São Caetano do Sul	150.638
SP	35	48906	São Carlos	226.322
SP	35	49003	São Francisco	2.783
SP	35	49102	São João da Boa Vista	84.584
SP	35	49201	São João das Duas Pontes	2.552
SP	35	49250	São João de Iracema	1.797
SP	35	49300	São João do Pau d'Alho	2.092
SP	35	49409	São Joaquim da Barra	47.256
SP	35	49508	São José da Bela Vista	8.456
SP	35	49607	São José do Barreiro	4.068
SP	35	49706	São José do Rio Pardo	52.176
SP	35	49805	São José do Rio Preto	415.769
SP	35	49904	São José dos Campos	643.603
SP	35	49953	São Lourenço da Serra	14.241
SP	35	50001	São Luís do Paraitinga	10.393
SP	35	50100	São Manuel	38.614
SP	35	50209	São Miguel Arcanjo	31.549
SP	35	50308	São Paulo	11.376.685
SP	35	50407	São Pedro	32.231
SP	35	50506	São Pedro do Turvo	7.245
SP	35	50605	São Roque	80.661
SP	35	50704	São Sebastião	76.344
SP	35	50803	São Sebastião da Grama	12.046
SP	35	50902	São Simão	14.448
SP	35	51009	São Vicente	336.809
SP	35	51108	Sarapuí	9.212
SP	35	51207	Sarutaiá	3.605
SP	35	51306	Sebastianópolis do Sul	3.105
SP	35	51405	Serra Azul	11.832
SP	35	51603	Serra Negra	26.770
SP	35	51504	Serrana	39.826
SP	35	51702	Sertãozinho	112.401
SP	35	51801	Sete Barras	12.898
SP	35	51900	Severínia	15.788
SP	35	52007	Silveiras	5.855
SP	35	52106	Socorro	37.288
SP	35	52205	Sorocaba	600.692
SP	35	52304	Sud Mennucci	7.446
SP	35	52403	Sumaré	246.247
SP	35	52551	Suzanápolis	3.473
SP	35	52502	Suzano	267.583
SP	35	52601	Tabapuã	11.495
SP	35	52700	Tabatinga	14.943
SP	35	52809	Taboão da Serra	251.608
SP	35	52908	Taciba	5.789

SP	35	53005	Taguaí	11.336
SP	35	53104	Taiaçu	5.936
SP	35	53203	Taiúva	5.439
SP	35	53302	Tambaú	22.429
SP	35	53401	Tanabi	24.277
SP	35	53500	Tapiraí	7.928
SP	35	53609	Tapiratiba	12.707
SP	35	53658	Taquaral	2.727
SP	35	53708	Taquaritinga	54.279
SP	35	53807	Taquarituba	22.338
SP	35	53856	Taquarivaí	5.254
SP	35	53906	Tarabai	6.731
SP	35	53955	Tarumã	13.209
SP	35	54003	Tatuí	109.425
SP	35	54102	Taubaté	283.899
SP	35	54201	Tejupá	4.730
SP	35	54300	Teodoro Sampaio	21.595
SP	35	54409	Terra Roxa	8.619
SP	35	54508	Tietê	37.609
SP	35	54607	Timburi	2.634
SP	35	54656	Torre de Pedra	2.271
SP	35	54706	Torrinha	9.405
SP	35	54755	Trabiju	1.569
SP	35	54805	Tremembé	41.915
SP	35	54904	Três Fronteiras	5.468
SP	35	54953	Tuiuti	6.078
SP	35	55000	Tupã	63.498
SP	35	55109	Tupi Paulista	14.418
SP	35	55208	Turiúba	1.936
SP	35	55307	Turmalina	1.920
SP	35	55356	Ubarana	5.451
SP	35	55406	Ubatuba	80.604
SP	35	55505	Ubirajara	4.468
SP	35	55604	Uchoa	9.537
SP	35	55703	União Paulista	1.636
SP	35	55802	Urânia	8.838
SP	35	55901	Uru	1.228
SP	35	56008	Urupês	12.848
SP	35	56107	Valentim Gentil	11.404
SP	35	56206	Valinhos	110.390
SP	35	56305	Valparaíso	23.181
SP	35	56354	Vargem	9.077
SP	35	56404	Vargem Grande do Sul	39.714
SP	35	56453	Vargem Grande Paulista	44.555
SP	35	56503	Várzea Paulista	109.247
SP	35	56602	Vera Cruz	10.722

SP	35	56701	Vinhedo	66.087
SP	35	56800	Viradouro	17.499
SP	35	56909	Vista Alegre do Alto	7.208
SP	35	56958	Vitória Brasil	1.747
SP	35	57006	Votorantim	110.755
SP	35	57105	Votuporanga	86.059
SP	35	57154	Zacarias	2.394
PR	41	00103	Abatiá	7.690
PR	41	00202	Adrianópolis	6.281
PR	41	00301	Agudos do Sul	8.429
PR	41	00400	Almirante Tamandaré	105.458
PR	41	00459	Altamira do Paraná	3.900
PR	41	28625	Alto Paraíso	3.119
PR	41	00608	Alto Paraná	13.806
PR	41	00707	Alto Piquiri	10.092
PR	41	00509	Altônia	20.711
PR	41	00806	Alvorada do Sul	10.439
PR	41	00905	Amaporã	5.562
PR	41	01002	Ampére	17.563
PR	41	01051	Anahy	2.854
PR	41	01101	Andirá	20.451
PR	41	01150	Ângulo	2.862
PR	41	01200	Antonina	18.849
PR	41	01309	Antônio Olinto	7.343
PR	41	01408	Apucarana	122.896
PR	41	01507	Arapongas	106.978
PR	41	01606	Arapoti	26.153
PR	41	01655	Arapuã	3.469
PR	41	01705	Araruna	13.471
PR	41	01804	Araucária	122.878
PR	41	01853	Ariranha do Ivaí	2.389
PR	41	01903	Assaí	16.099
PR	41	02000	Assis Chateaubriand	32.981
PR	41	02109	Astorga	24.859
PR	41	02208	Atalaia	3.898
PR	41	02307	Balsa Nova	11.539
PR	41	02406	Bandeirantes	31.951
PR	41	02505	Barbosa Ferraz	12.437
PR	41	02703	Barra do Jacaré	2.728
PR	41	02604	Barracão	9.796
PR	41	02752	Bela Vista da Caroba	3.861
PR	41	02802	Bela Vista do Paraíso	15.087
PR	41	02901	Bituruna	15.903
PR	41	03008	Boa Esperança	4.479
PR	41	03024	Boa Esperança do Iguaçu	2.713
PR	41	03040	Boa Ventura de São Roque	6.520

PR	41	03057	Boa Vista da Aparecida	7.834
PR	41	03107	Bocaiúva do Sul	11.280
PR	41	03156	Bom Jesus do Sul	3.742
PR	41	03206	Bom Sucesso	6.620
PR	41	03222	Bom Sucesso do Sul	3.279
PR	41	03305	Borrazópolis	7.641
PR	41	03354	Braganey	5.667
PR	41	03370	Brasilândia do Sul	3.107
PR	41	03404	Cafeara	2.727
PR	41	03453	Cafelândia	15.194
PR	41	03479	Cafezal do Sul	4.236
PR	41	03503	Califórnia	8.129
PR	41	03602	Cambará	24.060
PR	41	03701	Cambé	98.024
PR	41	03800	Cambira	7.319
PR	41	03909	Campina da Lagoa	15.149
PR	41	03958	Campina do Simão	4.033
PR	41	04006	Campina Grande do Sul	39.404
PR	41	04055	Campo Bonito	4.299
PR	41	04105	Campo do Tenente	7.245
PR	41	04204	Campo Largo	115.336
PR	41	04253	Campo Magro	25.513
PR	41	04303	Campo Mourão	88.209
PR	41	04402	Cândido de Abreu	16.332
PR	41	04428	Candói	15.104
PR	41	04451	Cantagalo	12.974
PR	41	04501	Capanema	18.570
PR	41	04600	Capitão Leônidas Marques	15.060
PR	41	04659	Carambeí	19.813
PR	41	04709	Carlópolis	13.767
PR	41	04808	Cascavel	292.372
PR	41	04907	Castro	67.613
PR	41	05003	Catanduvas	10.169
PR	41	05102	Centenário do Sul	11.096
PR	41	05201	Cerro Azul	17.027
PR	41	05300	Céu Azul	11.121
PR	41	05409	Chopinzinho	19.549
PR	41	05508	Cianorte	71.855
PR	41	05607	Cidade Gaúcha	11.294
PR	41	05706	Clevelândia	17.075
PR	41	05805	Colombo	217.443
PR	41	05904	Colorado	22.555
PR	41	06001	Congonhinhas	8.344
PR	41	06100	Conselheiro Mairinck	3.663
PR	41	06209	Contenda	16.292
PR	41	06308	Corbélia	16.389

PR	41	06407	Cornélio Procópio	46.939
PR	41	06456	Coronel Domingos Soares	7.274
PR	41	06506	Coronel Vivida	21.514
PR	41	06555	Corumbataí do Sul	3.860
PR	41	06803	Cruz Machado	18.097
PR	41	06571	Cruzeiro do Iguaçu	4.261
PR	41	06605	Cruzeiro do Oeste	20.446
PR	41	06704	Cruzeiro do Sul	4.534
PR	41	06852	Cruzmaltina	3.118
PR	41	06902	Curitiba	1.776.761
PR	41	07009	Curiúva	14.077
PR	41	07108	Diamante do Norte	5.428
PR	41	07124	Diamante do Sul	3.488
PR	41	07157	Diamante D'Oeste	5.050
PR	41	07207	Dois Vizinhos	36.813
PR	41	07256	Douradina	7.640
PR	41	07306	Doutor Camargo	5.836
PR	41	28633	Doutor Ulysses	5.686
PR	41	07405	Enéas Marques	6.061
PR	41	07504	Engenheiro Beltrão	13.880
PR	41	07538	Entre Rios do Oeste	4.017
PR	41	07520	Esperança Nova	1.919
PR	41	07546	Espigão Alto do Iguaçu	4.570
PR	41	07553	Farol	3.398
PR	41	07603	Faxinal	16.421
PR	41	07652	Fazenda Rio Grande	84.514
PR	41	07702	Fênix	4.781
PR	41	07736	Fernandes Pinheiro	5.867
PR	41	07751	Figueira	8.181
PR	41	07850	Flor da Serra do Sul	4.695
PR	41	07801	Floraí	5.015
PR	41	07900	Floresta	6.054
PR	41	08007	Florestópolis	11.076
PR	41	08106	Flórida	2.560
PR	41	08205	Formosa do Oeste	7.358
PR	41	08304	Foz do Iguaçu	255.718
PR	41	08452	Foz do Jordão	5.276
PR	41	08320	Francisco Alves	6.337
PR	41	08403	Francisco Beltrão	80.727
PR	41	08502	General Carneiro	13.635
PR	41	08551	Godoy Moreira	3.262
PR	41	08601	Goioerê	28.908
PR	41	08650	Goioxim	7.415
PR	41	08700	Grandes Rios	6.438
PR	41	08809	Guaíra	31.013
PR	41	08908	Guairaçá	6.243

PR	41	08957	Guamiranga	8.016
PR	41	09005	Guapirama	3.865
PR	41	09104	Guaporema	2.223
PR	41	09203	Guaraci	5.181
PR	41	09302	Guaraniaçu	14.187
PR	41	09401	Guarapuava	169.252
PR	41	09500	Guaraqueçaba	7.809
PR	41	09609	Guaratuba	32.826
PR	41	09658	Honório Serpa	5.813
PR	41	09708	Ibaiti	29.099
PR	41	09757	Ibema	6.096
PR	41	09807	Ibiporã	49.111
PR	41	09906	Icaraíma	8.657
PR	41	10003	Iguaraçu	4.040
PR	41	10052	Iguatu	2.231
PR	41	10078	Imbaú	11.546
PR	41	10102	Imbituva	29.053
PR	41	10201	Inácio Martins	10.940
PR	41	10300	Inajá	3.000
PR	41	10409	Indianópolis	4.313
PR	41	10508	Ipiranga	14.278
PR	41	10607	Iporã	14.760
PR	41	10656	Iracema do Oeste	2.522
PR	41	10706	Irati	56.790
PR	41	10805	Iretama	10.515
PR	41	10904	Itaguajé	4.538
PR	41	10953	Itaipulândia	9.357
PR	41	11001	Itambaracá	6.710
PR	41	11100	Itambé	5.983
PR	41	11209	Itapejara d'Oeste	10.738
PR	41	11258	Itaperuçu	24.573
PR	41	11308	Itaúna do Sul	3.453
PR	41	11407	Ivaí	12.954
PR	41	11506	Ivaiporã	31.748
PR	41	11555	Ivaté	7.603
PR	41	11605	Ivatuba	3.043
PR	41	11704	Jaboti	4.950
PR	41	11803	Jacarezinho	39.045
PR	41	11902	Jaguapitã	12.421
PR	41	12009	Jaguariaíva	32.882
PR	41	12108	Jandaia do Sul	20.359
PR	41	12207	Janiópolis	6.298
PR	41	12306	Japira	4.904
PR	41	12405	Japurá	8.669
PR	41	12504	Jardim Alegre	12.121
PR	41	12603	Jardim Olinda	1.392

PR	41	12702	Jataizinho	11.958
PR	41	12751	Jesuítas	8.876
PR	41	12801	Joaquim Távora	10.899
PR	41	12900	Jundiaí do Sul	3.399
PR	41	12959	Juranda	7.567
PR	41	13007	Jussara	6.657
PR	41	13106	Kaloré	4.425
PR	41	13205	Lapa	45.334
PR	41	13254	Laranjal	6.257
PR	41	13304	Laranjeiras do Sul	30.891
PR	41	13403	Leópolis	4.101
PR	41	13429	Lidianópolis	3.851
PR	41	13452	Lindoeste	5.231
PR	41	13502	Loanda	21.451
PR	41	13601	Lobato	4.452
PR	41	13700	Londrina	515.707
PR	41	13734	Luiziana	7.282
PR	41	13759	Lunardelli	5.084
PR	41	13809	Lupionópolis	4.633
PR	41	13908	Mallet	13.030
PR	41	14005	Mamborê	13.781
PR	41	14104	Mandaguaçu	20.227
PR	41	14203	Mandaguari	32.849
PR	41	14302	Mandirituba	22.927
PR	41	14351	Manfrinópolis	3.026
PR	41	14401	Mangueirinha	16.941
PR	41	14500	Manoel Ribas	13.185
PR	41	14609	Marechal Cândido Rondon	47.697
PR	41	14708	Maria Helena	5.892
PR	41	14807	Marialva	32.451
PR	41	14906	Marilândia do Sul	8.832
PR	41	15002	Marilena	6.874
PR	41	15101	Mariluz	10.214
PR	41	15200	Maringá	367.410
PR	41	15309	Mariópolis	6.306
PR	41	15358	Maripá	5.654
PR	41	15408	Marmeleiro	13.936
PR	41	15457	Marquinho	4.879
PR	41	15507	Marumbi	4.602
PR	41	15606	Matelândia	16.340
PR	41	15705	Matinhos	30.220
PR	41	15739	Mato Rico	3.716
PR	41	15754	Mauá da Serra	8.870
PR	41	15804	Medianeira	42.420
PR	41	15853	Mercedes	5.113
PR	41	15903	Mirador	2.301

PR	41	16000	Miraselva	1.848
PR	41	16059	Missal	10.481
PR	41	16109	Moreira Sales	12.487
PR	41	16208	Morretes	15.785
PR	41	16307	Munhoz de Melo	3.713
PR	41	16406	Nossa Senhora das Graças	3.930
PR	41	16505	Nova Aliança do Ivaí	1.446
PR	41	16604	Nova América da Colina	3.462
PR	41	16703	Nova Aurora	11.598
PR	41	16802	Nova Cantu	7.050
PR	41	16901	Nova Esperança	26.749
PR	41	16950	Nova Esperança do Sudoeste	5.074
PR	41	17008	Nova Fátima	8.124
PR	41	17057	Nova Laranjeiras	11.690
PR	41	17107	Nova Londrina	13.052
PR	41	17206	Nova Olímpia	5.537
PR	41	17255	Nova Prata do Iguaçu	10.374
PR	41	17214	Nova Santa Bárbara	3.953
PR	41	17222	Nova Santa Rosa	7.702
PR	41	17271	Nova Tebas	7.085
PR	41	17297	Novo Itacolomi	2.822
PR	41	17305	Ortigueira	23.103
PR	41	17404	Ourizona	3.378
PR	41	17453	Ouro Verde do Oeste	5.726
PR	41	17503	Paiçandu	36.717
PR	41	17602	Palmas	44.107
PR	41	17701	Palmeira	32.326
PR	41	17800	Palmital	14.538
PR	41	17909	Palotina	29.123
PR	41	18006	Paraíso do Norte	12.079
PR	41	18105	Paranacity	10.423
PR	41	18204	Paranaguá	142.452
PR	41	18303	Paranapoema	2.852
PR	41	18402	Paranavaí	82.472
PR	41	18451	Pato Bragado	4.939
PR	41	18501	Pato Branco	73.901
PR	41	18600	Paula Freitas	5.491
PR	41	18709	Paulo Frontin	6.966
PR	41	18808	Peabiru	13.645
PR	41	18857	Perobal	5.708
PR	41	18907	Pérola	10.348
PR	41	19004	Pérola d'Oeste	6.672
PR	41	19103	Piên	11.454
PR	41	19152	Pinhais	119.379
PR	41	19251	Pinhal de São Bento	2.635
PR	41	19202	Pinhalão	6.215

PR	41	19301	Pinhão	30.480
PR	41	19400	Piraí do Sul	23.693
PR	41	19509	Piraquara	96.023
PR	41	19608	Pitanga	32.152
PR	41	19657	Pitangueiras	2.874
PR	41	19707	Planaltina do Paraná	4.111
PR	41	19806	Planalto	13.584
PR	41	19905	Ponta Grossa	317.339
PR	41	19954	Pontal do Paraná	21.917
PR	41	20002	Porecatu	13.934
PR	41	20101	Porto Amazonas	4.556
PR	41	20150	Porto Barreiro	3.582
PR	41	20200	Porto Rico	2.527
PR	41	20309	Porto Vitória	4.016
PR	41	20333	Prado Ferreira	3.477
PR	41	20358	Pranchita	5.533
PR	41	20408	Presidente Castelo Branco	4.857
PR	41	20507	Primeiro de Maio	10.848
PR	41	20606	Prudentópolis	49.150
PR	41	20655	Quarto Centenário	4.784
PR	41	20705	Quatiguá	7.091
PR	41	20804	Quatro Barras	20.409
PR	41	20853	Quatro Pontes	3.827
PR	41	20903	Quedas do Iguaçu	31.095
PR	41	21000	Querência do Norte	11.773
PR	41	21109	Quinta do Sol	4.987
PR	41	21208	Quitandinha	17.364
PR	41	21257	Ramilândia	4.175
PR	41	21307	Rancho Alegre	3.919
PR	41	21356	Rancho Alegre D'Oeste	2.807
PR	41	21406	Realeza	16.386
PR	41	21505	Rebouças	14.254
PR	41	21604	Renascença	6.790
PR	41	21703	Reserva	25.353
PR	41	21752	Reserva do Iguaçu	7.402
PR	41	21802	Ribeirão Claro	10.645
PR	41	21901	Ribeirão do Pinhal	13.401
PR	41	22008	Rio Azul	14.255
PR	41	22107	Rio Bom	3.302
PR	41	22156	Rio Bonito do Iguaçu	13.125
PR	41	22172	Rio Branco do Ivaí	3.920
PR	41	22206	Rio Branco do Sul	30.848
PR	41	22305	Rio Negro	31.662
PR	41	22404	Rolândia	59.139
PR	41	22503	Roncador	11.221
PR	41	22602	Rondon	9.060

PR	41	22651	Rosário do Ivaí	5.438
PR	41	22701	Sabáudia	6.200
PR	41	22800	Salgado Filho	4.253
PR	41	22909	Salto do Itararé	5.122
PR	41	23006	Salto do Lontra	13.830
PR	41	23105	Santa Amélia	3.712
PR	41	23204	Santa Cecília do Pavão	3.583
PR	41	23303	Santa Cruz de Monte Castelo	8.019
PR	41	23402	Santa Fé	10.668
PR	41	23501	Santa Helena	23.855
PR	41	23600	Santa Inês	1.776
PR	41	23709	Santa Isabel do Ivaí	8.701
PR	41	23808	Santa Izabel do Oeste	13.347
PR	41	23824	Santa Lúcia	3.895
PR	41	23857	Santa Maria do Oeste	11.178
PR	41	23907	Santa Mariana	12.279
PR	41	23956	Santa Mônica	3.629
PR	41	24020	Santa Tereza do Oeste	10.269
PR	41	24053	Santa Terezinha de Itaipu	21.215
PR	41	24004	Santana do Itararé	5.191
PR	41	24103	Santo Antônio da Platina	43.125
PR	41	24202	Santo Antônio do Caiuá	2.705
PR	41	24301	Santo Antônio do Paraíso	2.351
PR	41	24400	Santo Antônio do Sudoeste	19.048
PR	41	24509	Santo Inácio	5.282
PR	41	24608	São Carlos do Ivaí	6.422
PR	41	24707	São Jerônimo da Serra	11.275
PR	41	24806	São João	10.508
PR	41	24905	São João do Caiuá	5.884
PR	41	25001	São João do Ivaí	11.273
PR	41	25100	São João do Triunfo	13.899
PR	41	25308	São Jorge do Ivaí	5.506
PR	41	25357	São Jorge do Patrocínio	5.956
PR	41	25209	São Jorge d'Oeste	9.052
PR	41	25407	São José da Boa Vista	6.441
PR	41	25456	São José das Palmeiras	3.789
PR	41	25506	São José dos Pinhais	273.255
PR	41	25555	São Manoel do Paraná	2.102
PR	41	25605	São Mateus do Sul	41.965
PR	41	25704	São Miguel do Iguaçu	25.971
PR	41	25753	São Pedro do Iguaçu	6.373
PR	41	25803	São Pedro do Ivaí	10.272
PR	41	25902	São Pedro do Paraná	2.454
PR	41	26009	São Sebastião da Amoreira	8.638
PR	41	26108	São Tomé	5.395
PR	41	26207	Sapopema	6.716

PR	41	26256	Sarandi	84.573
PR	41	26272	Saudade do Iguaçu	5.092
PR	41	26306	Sengés	18.511
PR	41	26355	Serranópolis do Iguaçu	4.543
PR	41	26405	Sertaneja	5.711
PR	41	26504	Sertanópolis	15.713
PR	41	26603	Siqueira Campos	18.825
PR	41	26652	Sulina	3.315
PR	41	26678	Tamarana	12.647
PR	41	26702	Tamboara	4.726
PR	41	26801	Tapejara	14.822
PR	41	26900	Tapira	5.769
PR	41	27007	Teixeira Soares	10.599
PR	41	27106	Telêmaco Borba	71.176
PR	41	27205	Terra Boa	15.948
PR	41	27304	Terra Rica	15.437
PR	41	27403	Terra Roxa	16.829
PR	41	27502	Tibagi	19.482
PR	41	27601	Tijucas do Sul	14.881
PR	41	27700	Toledo	122.502
PR	41	27809	Tomazina	8.619
PR	41	27858	Três Barras do Paraná	11.825
PR	41	27882	Tunas do Paraná	6.656
PR	41	27908	Tuneiras do Oeste	8.647
PR	41	27957	Tupãssi	7.994
PR	41	27965	Turvo	13.628
PR	41	28005	Ubiratã	21.402
PR	41	28104	Umuarama	102.184
PR	41	28203	União da Vitória	53.372
PR	41	28302	Uniflor	2.482
PR	41	28401	Uraí	11.411
PR	41	28534	Ventania	10.249
PR	41	28559	Vera Cruz do Oeste	8.871
PR	41	28609	Verê	7.751
PR	41	28658	Virmond	3.951
PR	41	28708	Vitorino	6.548
PR	41	28500	Wenceslau Braz	19.259
PR	41	28807	Xambrê	5.939
SC	42	00051	Abdon Batista	2.635
SC	42	00101	Abelardo Luz	17.200
SC	42	00200	Agrolândia	9.552
SC	42	00309	Agronômica	4.985
SC	42	00408	Água Doce	6.979
SC	42	00507	Águas de Chapecó	6.160
SC	42	00556	Águas Frias	2.409
SC	42	00606	Águas Mornas	5.685

SC	42	00705	Alfredo Wagner	9.494
SC	42	00754	Alto Bela Vista	1.991
SC	42	00804	Anchieta	6.172
SC	42	00903	Angelina	5.171
SC	42	01000	Anita Garibaldi	8.374
SC	42	01109	Anitápolis	3.211
SC	42	01208	Antônio Carlos	7.613
SC	42	01257	Apiúna	9.764
SC	42	01273	Arabutã	4.198
SC	42	01307	Araquari	26.875
SC	42	01406	Araranguá	62.308
SC	42	01505	Armazém	7.886
SC	42	01604	Arroio Trinta	3.504
SC	42	01653	Arvoredo	2.254
SC	42	01703	Ascurra	7.485
SC	42	01802	Atalanta	3.281
SC	42	01901	Aurora	5.561
SC	42	01950	Balneário Arroio do Silva	10.121
SC	42	02057	Balneário Barra do Sul	8.791
SC	42	02008	Balneário Camboriú	113.319
SC	42	02073	Balneário Gaivota	8.655
SC	42	12809	Balneário Piçarras	18.010
SC	42	02081	Bandeirante	2.866
SC	42	02099	Barra Bonita	1.853
SC	42	02107	Barra Velha	23.422
SC	42	02131	Bela Vista do Toldo	6.047
SC	42	02156	Belmonte	2.643
SC	42	02206	Benedito Novo	10.528
SC	42	02305	Biguaçu	59.736
SC	42	02404	Blumenau	316.139
SC	42	02438	Bocaina do Sul	3.314
SC	42	02503	Bom Jardim da Serra	4.443
SC	42	02537	Bom Jesus	2.599
SC	42	02578	Bom Jesus do Oeste	2.130
SC	42	02602	Bom Retiro	9.090
SC	42	02453	Bombinhas	15.136
SC	42	02701	Botuverá	4.584
SC	42	02800	Braço do Norte	29.672
SC	42	02859	Braço do Trombudo	3.498
SC	42	02875	Brunópolis	2.778
SC	42	02909	Brusque	109.950
SC	42	03006	Caçador	71.886
SC	42	03105	Caibi	6.199
SC	42	03154	Calmon	3.375
SC	42	03204	Camboriú	65.520
SC	42	03303	Campo Alegre	11.766

SC	42	03402	Campo Belo do Sul	7.398
SC	42	03501	Campo Erê	9.222
SC	42	03600	Campos Novos	33.313
SC	42	03709	Canelinha	10.845
SC	42	03808	Canoinhas	52.937
SC	42	03253	Capão Alto	2.713
SC	42	03907	Capinzal	21.064
SC	42	03956	Capivari de Baixo	22.145
SC	42	04004	Catanduvas	9.746
SC	42	04103	Caxambu do Sul	4.283
SC	42	04152	Celso Ramos	2.760
SC	42	04178	Cerro Negro	3.503
SC	42	04194	Chapadão do Lageado	2.793
SC	42	04202	Chapecó	189.052
SC	42	04251	Cocal do Sul	15.376
SC	42	04301	Concórdia	69.462
SC	42	04350	Cordilheira Alta	3.869
SC	42	04400	Coronel Freitas	10.165
SC	42	04459	Coronel Martins	2.469
SC	42	04558	Correia Pinto	14.447
SC	42	04509	Corupá	14.155
SC	42	04608	Criciúma	195.614
SC	42	04707	Cunha Porã	10.671
SC	42	04756	Cunhataí	1.892
SC	42	04806	Curitibanos	38.003
SC	42	04905	Descanso	8.560
SC	42	05001	Dionísio Cerqueira	14.896
SC	42	05100	Dona Emma	3.784
SC	42	05159	Doutor Pedrinho	3.683
SC	42	05175	Entre Rios	3.043
SC	42	05191	Ermo	2.049
SC	42	05209	Erval Velho	4.365
SC	42	05308	Faxinal dos Guedes	10.645
SC	42	05357	Flor do Sertão	1.585
SC	42	05407	Florianópolis	433.158
SC	42	05431	Formosa do Sul	2.583
SC	42	05456	Forquilhinha	23.183
SC	42	05506	Fraiburgo	34.796
SC	42	05555	Frei Rogério	2.399
SC	42	05605	Galvão	3.452
SC	42	05704	Garopaba	18.890
SC	42	05803	Garuva	15.272
SC	42	05902	Gaspar	59.728
SC	42	06009	Governador Celso Ramos	13.211
SC	42	06108	Grão Pará	6.268
SC	42	06207	Gravatal	10.758

SC	42	06306	Guabiruba	19.254
SC	42	06405	Guaraciaba	10.417
SC	42	06504	Guaramirim	36.640
SC	42	06603	Guarujá do Sul	4.941
SC	42	06652	Guatambú	4.676
SC	42	06702	Herval d'Oeste	21.420
SC	42	06751	Ibiam	1.944
SC	42	06801	Ibicaré	3.341
SC	42	06900	Ibirama	17.561
SC	42	07007	Içara	49.238
SC	42	07106	Ilhota	12.624
SC	42	07205	Imaruí	11.411
SC	42	07304	Imbituba	40.845
SC	42	07403	Imbuia	5.777
SC	42	07502	Indaial	57.068
SC	42	07577	Iomerê	2.768
SC	42	07601	Ipira	4.699
SC	42	07650	Iporã do Oeste	8.490
SC	42	07684	Ipuaçu	6.901
SC	42	07700	Ipumirim	7.268
SC	42	07759	Iraceminha	4.202
SC	42	07809	Irani	9.656
SC	42	07858	Irati	2.067
SC	42	07908	Irineópolis	10.556
SC	42	08005	Itá	6.375
SC	42	08104	Itaiópolis	20.485
SC	42	08203	Itajaí	188.791
SC	42	08302	Itapema	48.807
SC	42	08401	Itapiranga	15.623
SC	42	08450	Itapoá	15.658
SC	42	08500	Ituporanga	22.667
SC	42	08609	Jaborá	4.018
SC	42	08708	Jacinto Machado	10.562
SC	42	08807	Jaguaruna	17.695
SC	42	08906	Jaraguá do Sul	148.353
SC	42	08955	Jardinópolis	1.732
SC	42	09003	Joaçaba	27.467
SC	42	09102	Joinville	526.338
SC	42	09151	José Boiteux	4.741
SC	42	09177	Jupiá	2.138
SC	42	09201	Lacerdópolis	2.203
SC	42	09300	Lages	156.604
SC	42	09409	Laguna	42.750
SC	42	09458	Lajeado Grande	1.478
SC	42	09508	Laurentino	6.147
SC	42	09607	Lauro Muller	14.483

SC	42	09706	Lebon Régis	11.862
SC	42	09805	Leoberto Leal	3.309
SC	42	09854	Lindóia do Sul	4.622
SC	42	09904	Lontras	10.526
SC	42	10001	Luiz Alves	10.811
SC	42	10035	Luzerna	5.605
SC	42	10050	Macieira	1.815
SC	42	10100	Mafra	53.361
SC	42	10209	Major Gercino	3.300
SC	42	10308	Major Vieira	7.566
SC	42	10407	Maracajá	6.535
SC	42	10506	Maravilha	22.642
SC	42	10555	Marema	2.136
SC	42	10605	Massaranduba	14.993
SC	42	10704	Matos Costa	2.784
SC	42	10803	Meleiro	6.988
SC	42	10852	Mirim Doce	2.477
SC	42	10902	Modelo	4.063
SC	42	11009	Mondaí	10.458
SC	42	11058	Monte Carlo	9.381
SC	42	11108	Monte Castelo	8.346
SC	42	11207	Morro da Fumaça	16.364
SC	42	11256	Morro Grande	2.886
SC	42	11306	Navegantes	63.764
SC	42	11405	Nova Erechim	4.386
SC	42	11454	Nova Itaberaba	4.269
SC	42	11504	Nova Trento	12.544
SC	42	11603	Nova Veneza	13.581
SC	42	11652	Novo Horizonte	2.697
SC	42	11702	Orleans	21.599
SC	42	11751	Otacílio Costa	16.691
SC	42	11801	Ouro	7.348
SC	42	11850	Ouro Verde	2.259
SC	42	11876	Paial	1.720
SC	42	11892	Painel	2.351
SC	42	11900	Palhoça	142.558
SC	42	12007	Palma Sola	7.699
SC	42	12056	Palmeira	2.410
SC	42	12106	Palmitos	16.018
SC	42	12205	Papanduva	18.096
SC	42	12239	Paraíso	3.972
SC	42	12254	Passo de Torres	6.964
SC	42	12270	Passos Maia	4.374
SC	42	12304	Paulo Lopes	6.808
SC	42	12403	Pedras Grandes	4.078
SC	42	12502	Penha	26.268

SC	42	12601	Peritiba	2.952
SC	42	12650	Pescaria Brava (**)	9.416
SC	42	12700	Petrolândia	6.090
SC	42	12908	Pinhalzinho	16.933
SC	42	13005	Pinheiro Preto	3.190
SC	42	13104	Piratuba	4.632
SC	42	13153	Planalto Alegre	2.685
SC	42	13203	Pomerode	28.610
SC	42	13302	Ponte Alta	4.853
SC	42	13351	Ponte Alta do Norte	3.316
SC	42	13401	Ponte Serrada	11.102
SC	42	13500	Porto Belo	16.896
SC	42	13609	Porto União	33.740
SC	42	13708	Pouso Redondo	15.204
SC	42	13807	Praia Grande	7.265
SC	42	13906	Presidente Castello Branco	1.697
SC	42	14003	Presidente Getúlio	15.273
SC	42	14102	Presidente Nereu	2.281
SC	42	14151	Princesa	2.780
SC	42	14201	Quilombo	10.175
SC	42	14300	Rancho Queimado	2.765
SC	42	14409	Rio das Antas	6.146
SC	42	14508	Rio do Campo	6.143
SC	42	14607	Rio do Oeste	7.145
SC	42	14805	Rio do Sul	62.658
SC	42	14706	Rio dos Cedros	10.488
SC	42	14904	Rio Fortuna	4.466
SC	42	15000	Rio Negrinho	40.169
SC	42	15059	Rio Rufino	2.440
SC	42	15075	Riqueza	4.789
SC	42	15109	Rodeio	11.004
SC	42	15208	Romelândia	5.494
SC	42	15307	Salete	7.402
SC	42	15356	Saltinho	3.926
SC	42	15406	Salto Veloso	4.361
SC	42	15455	Sangão	10.744
SC	42	15505	Santa Cecília	15.902
SC	42	15554	Santa Helena	2.351
SC	42	15604	Santa Rosa de Lima	2.074
SC	42	15653	Santa Rosa do Sul	8.091
SC	42	15679	Santa Terezinha	8.756
SC	42	15687	Santa Terezinha do Progresso	2.818
SC	42	15695	Santiago do Sul	1.431
SC	42	15703	Santo Amaro da Imperatriz	20.332
SC	42	15802	São Bento do Sul	76.215
SC	42	15752	São Bernardino	2.676

SC	42	15901	São Bonifácio	2.977
SC	42	16008	São Carlos	10.431
SC	42	16057	São Cristovão do Sul	5.089
SC	42	16107	São Domingos	9.389
SC	42	16206	São Francisco do Sul	44.064
SC	42	16305	São João Batista	27.982
SC	42	16354	São João do Itaperiú	3.477
SC	42	16255	São João do Oeste	6.074
SC	42	16404	São João do Sul	7.035
SC	42	16503	São Joaquim	25.111
SC	42	16602	São José	215.278
SC	42	16701	São José do Cedro	13.685
SC	42	16800	São José do Cerrito	9.104
SC	42	16909	São Lourenço do Oeste	22.062
SC	42	17006	São Ludgero	11.357
SC	42	17105	São Martinho	3.200
SC	42	17154	São Miguel da Boa Vista	1.887
SC	42	17204	São Miguel do Oeste	36.908
SC	42	17253	São Pedro de Alcântara	4.874
SC	42	17303	Saudades	9.121
SC	42	17402	Schroeder	16.248
SC	42	17501	Seara	17.005
SC	42	17550	Serra Alta	3.279
SC	42	17600	Siderópolis	13.137
SC	42	17709	Sombrio	27.165
SC	42	17758	Sul Brasil	2.714
SC	42	17808	Taió	17.412
SC	42	17907	Tangará	8.653
SC	42	17956	Tigrinhos	1.739
SC	42	18004	Tijucas	32.087
SC	42	18103	Timbé do Sul	5.306
SC	42	18202	Timbó	37.894
SC	42	18251	Timbó Grande	7.268
SC	42	18301	Três Barras	18.281
SC	42	18350	Treviso	3.585
SC	42	18400	Treze de Maio	6.901
SC	42	18509	Treze Tílias	6.568
SC	42	18608	Trombudo Central	6.668
SC	42	18707	Tubarão	98.412
SC	42	18756	Tunápolis	4.612
SC	42	18806	Turvo	12.001
SC	42	18855	União do Oeste	2.838
SC	42	18905	Urubici	10.767
SC	42	18954	Urupema	2.476
SC	42	19002	Urussanga	20.356
SC	42	19101	Vargeão	3.533

SC	42	19150	Vargem	2.746
SC	42	19176	Vargem Bonita	4.738
SC	42	19200	Vidal Ramos	6.284
SC	42	19309	Videira	48.064
SC	42	19358	Vitor Meireles	5.160
SC	42	19408	Witmarsum	3.653
SC	42	19507	Xanxerê	45.140
SC	42	19606	Xavantina	4.103
SC	42	19705	Xaxim	26.145
SC	42	19853	Zortéa	3.046
SC	42	20000	Balneário Rincão (**)	11.136
RS	43	00034	Aceguá	4.465
RS	43	00059	Água Santa	3.712
RS	43	00109	Agudo	16.612
RS	43	00208	Ajuricaba	7.187
RS	43	00307	Alecrim	6.828
RS	43	00406	Alegrete	76.644
RS	43	00455	Alegria	4.141
RS	43	00471	Almirante Tamandaré do Sul	2.041
RS	43	00505	Alpestre	7.689
RS	43	00554	Alto Alegre	1.805
RS	43	00570	Alto Feliz	2.930
RS	43	00604	Alvorada	197.441
RS	43	00638	Amaral Ferrador	6.446
RS	43	00646	Ametista do Sul	7.310
RS	43	00661	André da Rocha	1.232
RS	43	00703	Anta Gorda	6.035
RS	43	00802	Antônio Prado	12.821
RS	43	00851	Arambaré	3.660
RS	43	00877	Araricá	4.990
RS	43	00901	Aratiba	6.482
RS	43	01008	Arroio do Meio	19.060
RS	43	01073	Arroio do Padre	2.756
RS	43	01057	Arroio do Sal	8.113
RS	43	01206	Arroio do Tigre	12.774
RS	43	01107	Arroio dos Ratos	13.647
RS	43	01305	Arroio Grande	18.368
RS	43	01404	Arvorezinha	10.220
RS	43	14548	Pinto Bandeira (**)	2.578
RS	43	01503	Augusto Pestana	6.990
RS	43	01552	Áurea	3.632
RS	43	01602	Bagé	117.090
RS	43	01636	Balneário Pinhal	11.371
RS	43	01651	Barão	5.793
RS	43	01701	Barão de Cotegipe	6.521
RS	43	01750	Barão do Triunfo	7.072

RS	43	01859	Barra do Guarita	3.105
RS	43	01875	Barra do Quaraí	4.032
RS	43	01909	Barra do Ribeiro	12.682
RS	43	01925	Barra do Rio Azul	1.941
RS	43	01958	Barra Funda	2.388
RS	43	01800	Barracão	5.322
RS	43	02006	Barros Cassal	11.101
RS	43	02055	Benjamin Constant do Sul	2.244
RS	43	02105	Bento Gonçalves	107.075
RS	43	02154	Boa Vista das Missões	2.103
RS	43	02204	Boa Vista do Buricá	6.573
RS	43	02220	Boa Vista do Cadeado	2.437
RS	43	02238	Boa Vista do Incra	2.447
RS	43	02253	Boa Vista do Sul	2.767
RS	43	02303	Bom Jesus	11.445
RS	43	02352	Bom Princípio	12.136
RS	43	02378	Bom Progresso	2.253
RS	43	02402	Bom Retiro do Sul	11.576
RS	43	02451	Boqueirão do Leão	7.651
RS	43	02501	Bossoroca	6.753
RS	43	02584	Bozano	2.179
RS	43	02600	Braga	3.628
RS	43	02659	Brochier	4.726
RS	43	02709	Butiá	20.419
RS	43	02808	Caçapava do Sul	33.547
RS	43	02907	Cacequi	13.430
RS	43	03004	Cachoeira do Sul	83.217
RS	43	03103	Cachoeirinha	119.896
RS	43	03202	Cacique Doble	4.883
RS	43	03301	Caibaté	4.914
RS	43	03400	Caiçara	4.995
RS	43	03509	Camaquã	63.124
RS	43	03558	Camargo	2.607
RS	43	03608	Cambará do Sul	6.498
RS	43	03673	Campestre da Serra	3.259
RS	43	03707	Campina das Missões	5.982
RS	43	03806	Campinas do Sul	5.476
RS	43	03905	Campo Bom	60.989
RS	43	04002	Campo Novo	5.269
RS	43	04101	Campos Borges	3.451
RS	43	04200	Candelária	30.260
RS	43	04309	Cândido Godói	6.451
RS	43	04358	Candiota	8.878
RS	43	04408	Canela	40.076
RS	43	04507	Canguçu	53.533
RS	43	04606	Canoas	326.505

RS	43	04614	Canudos do Vale	1.785
RS	43	04622	Capão Bonito do Sul	1.730
RS	43	04630	Capão da Canoa	43.783
RS	43	04655	Capão do Cipó	3.187
RS	43	04663	Capão do Leão	24.386
RS	43	04689	Capela de Santana	11.851
RS	43	04697	Capitão	2.647
RS	43	04671	Capivari do Sul	4.009
RS	43	04713	Caraá	7.450
RS	43	04705	Carazinho	59.569
RS	43	04804	Carlos Barbosa	25.898
RS	43	04853	Carlos Gomes	1.561
RS	43	04903	Casca	8.683
RS	43	04952	Caseiros	3.030
RS	43	05009	Catuípe	9.191
RS	43	05108	Caxias do Sul	446.911
RS	43	05116	Centenário	2.941
RS	43	05124	Cerrito	6.324
RS	43	05132	Cerro Branco	4.478
RS	43	05157	Cerro Grande	2.390
RS	43	05173	Cerro Grande do Sul	10.570
RS	43	05207	Cerro Largo	13.384
RS	43	05306	Chapada	9.322
RS	43	05355	Charqueadas	36.130
RS	43	05371	Charrua	3.424
RS	43	05405	Chiapetta	3.979
RS	43	05439	Chuí	6.031
RS	43	05447	Chuvisca	5.011
RS	43	05454	Cidreira	13.240
RS	43	05504	Ciríaco	4.873
RS	43	05587	Colinas	2.414
RS	43	05603	Colorado	3.472
RS	43	05702	Condor	6.562
RS	43	05801	Constantina	9.742
RS	43	05835	Coqueiro Baixo	1.518
RS	43	05850	Coqueiros do Sul	2.422
RS	43	05871	Coronel Barros	2.460
RS	43	05900	Coronel Bicaco	7.645
RS	43	05934	Coronel Pilar	1.702
RS	43	05959	Cotiporã	3.891
RS	43	05975	Coxilha	2.803
RS	43	06007	Crissiumal	13.919
RS	43	06056	Cristal	7.378
RS	43	06072	Cristal do Sul	2.819
RS	43	06106	Cruz Alta	62.138
RS	43	06130	Cruzaltense	2.080

RS	43	06205	Cruzeiro do Sul	12.420
RS	43	06304	David Canabarro	4.675
RS	43	06320	Derrubadas	3.111
RS	43	06353	Dezesseis de Novembro	2.779
RS	43	06379	Dilermando de Aguiar	3.044
RS	43	06403	Dois Irmãos	28.348
RS	43	06429	Dois Irmãos das Missões	2.126
RS	43	06452	Dois Lajeados	3.287
RS	43	06502	Dom Feliciano	14.503
RS	43	06601	Dom Pedrito	38.670
RS	43	06551	Dom Pedro de Alcântara	2.538
RS	43	06700	Dona Francisca	3.326
RS	43	06734	Doutor Maurício Cardoso	5.160
RS	43	06759	Doutor Ricardo	2.016
RS	43	06767	Eldorado do Sul	35.412
RS	43	06809	Encantado	20.810
RS	43	06908	Encruzilhada do Sul	24.671
RS	43	06924	Engenho Velho	1.436
RS	43	06957	Entre Rios do Sul	3.018
RS	43	06932	Entre-Ijuís	8.823
RS	43	06973	Erebango	2.962
RS	43	07005	Erechim	97.404
RS	43	07054	Ernestina	3.090
RS	43	07203	Erval Grande	5.090
RS	43	07302	Erval Seco	7.682
RS	43	07401	Esmeralda	3.176
RS	43	07450	Esperança do Sul	3.200
RS	43	07500	Espumoso	15.241
RS	43	07559	Estação	5.979
RS	43	07609	Estância Velha	43.698
RS	43	07708	Esteio	80.862
RS	43	07807	Estrela	31.105
RS	43	07815	Estrela Velha	3.619
RS	43	07831	Eugênio de Castro	2.721
RS	43	07864	Fagundes Varela	2.596
RS	43	07906	Farroupilha	64.893
RS	43	08003	Faxinal do Soturno	6.647
RS	43	08052	Faxinalzinho	2.514
RS	43	08078	Fazenda Vilanova	3.828
RS	43	08102	Feliz	12.517
RS	43	08201	Flores da Cunha	27.647
RS	43	08250	Floriano Peixoto	1.967
RS	43	08300	Fontoura Xavier	10.606
RS	43	08409	Formigueiro	6.926
RS	43	08433	Forquetinha	2.458
RS	43	08458	Fortaleza dos Valos	4.513

RS	43	08508	Frederico Westphalen	29.158
RS	43	08607	Garibaldi	31.328
RS	43	08656	Garruchos	3.168
RS	43	08706	Gaurama	5.783
RS	43	08805	General Câmara	8.404
RS	43	08854	Gentil	1.663
RS	43	08904	Getúlio Vargas	16.101
RS	43	09001	Giruá	16.823
RS	43	09050	Glorinha	7.074
RS	43	09100	Gramado	32.829
RS	43	09126	Gramado dos Loureiros	2.228
RS	43	09159	Gramado Xavier	4.016
RS	43	09209	Gravataí	259.138
RS	43	09258	Guabiju	1.576
RS	43	09308	Guaíba	95.340
RS	43	09407	Guaporé	23.230
RS	43	09506	Guarani das Missões	7.983
RS	43	09555	Harmonia	4.344
RS	43	07104	Herval	6.739
RS	43	09571	Herveiras	2.954
RS	43	09605	Horizontina	18.446
RS	43	09654	Hulha Negra	6.147
RS	43	09704	Humaitá	4.873
RS	43	09753	Ibarama	4.368
RS	43	09803	Ibiaçá	4.692
RS	43	09902	Ibiraiaras	7.173
RS	43	09951	Ibirapuitã	4.037
RS	43	10009	Ibirubá	19.415
RS	43	10108	Igrejinha	32.399
RS	43	10207	Ijuí	79.396
RS	43	10306	Ilópolis	4.079
RS	43	10330	Imbé	18.490
RS	43	10363	Imigrante	3.029
RS	43	10405	Independência	6.514
RS	43	10413	Inhacorá	2.251
RS	43	10439	Ipê	6.101
RS	43	10462	Ipiranga do Sul	1.927
RS	43	10504	Iraí	7.902
RS	43	10538	Itaara	5.076
RS	43	10553	Itacurubi	3.432
RS	43	10579	Itapuca	2.292
RS	43	10603	Itaqui	37.916
RS	43	10652	Itati	2.546
RS	43	10702	Itatiba do Sul	4.008
RS	43	10751	Ivorá	2.105
RS	43	10801	Ivoti	20.562

RS	43	10850	Jaboticaba	4.032
RS	43	10876	Jacuizinho	2.530
RS	43	10900	Jacutinga	3.607
RS	43	11007	Jaguarão	27.605
RS	43	11106	Jaguari	11.320
RS	43	11122	Jaquirana	4.081
RS	43	11130	Jari	3.549
RS	43	11155	Jóia	8.339
RS	43	11205	Júlio de Castilhos	19.453
RS	43	11239	Lagoa Bonita do Sul	2.694
RS	43	11270	Lagoa dos Três Cantos	1.594
RS	43	11304	Lagoa Vermelha	27.466
RS	43	11254	Lagoão	6.247
RS	43	11403	Lajeado	73.201
RS	43	11429	Lajeado do Bugre	2.491
RS	43	11502	Lavras do Sul	7.615
RS	43	11601	Liberato Salzano	5.661
RS	43	11627	Lindolfo Collor	5.350
RS	43	11643	Linha Nova	1.634
RS	43	11718	Maçambara	4.694
RS	43	11700	Machadinho	5.478
RS	43	11734	Mampituba	2.988
RS	43	11759	Manoel Viana	7.084
RS	43	11775	Maquiné	6.845
RS	43	11791	Maratá	2.546
RS	43	11809	Marau	37.573
RS	43	11908	Marcelino Ramos	4.987
RS	43	11981	Mariana Pimentel	3.774
RS	43	12005	Mariano Moro	2.171
RS	43	12054	Marques de Souza	4.042
RS	43	12104	Mata	5.041
RS	43	12138	Mato Castelhano	2.473
RS	43	12153	Mato Leitão	3.964
RS	43	12179	Mato Queimado	1.766
RS	43	12203	Maximiliano de Almeida	4.800
RS	43	12252	Minas do Leão	7.678
RS	43	12302	Miraguaí	4.828
RS	43	12351	Montauri	1.521
RS	43	12377	Monte Alegre dos Campos	3.112
RS	43	12385	Monte Belo do Sul	2.637
RS	43	12401	Montenegro	60.196
RS	43	12427	Mormaço	2.797
RS	43	12443	Morrinhos do Sul	3.129
RS	43	12450	Morro Redondo	6.262
RS	43	12476	Morro Reuter	5.781
RS	43	12500	Mostardas	12.195

RS	43	12609	Muçum	4.801
RS	43	12617	Muitos Capões	3.007
RS	43	12625	Muliterno	1.820
RS	43	12658	Não-Me-Toque	16.166
RS	43	12674	Nicolau Vergueiro	1.708
RS	43	12708	Nonoai	11.962
RS	43	12757	Nova Alvorada	3.247
RS	43	12807	Nova Araçá	4.117
RS	43	12906	Nova Bassano	8.992
RS	43	12955	Nova Boa Vista	1.921
RS	43	13003	Nova Bréscia	3.197
RS	43	13011	Nova Candelária	2.732
RS	43	13037	Nova Esperança do Sul	4.771
RS	43	13060	Nova Hartz	18.841
RS	43	13086	Nova Pádua	2.459
RS	43	13102	Nova Palma	6.347
RS	43	13201	Nova Petrópolis	19.371
RS	43	13300	Nova Prata	23.508
RS	43	13334	Nova Ramada	2.394
RS	43	13359	Nova Roma do Sul	3.390
RS	43	13375	Nova Santa Rita	23.768
RS	43	13490	Novo Barreiro	3.995
RS	43	13391	Novo Cabrais	3.899
RS	43	13409	Novo Hamburgo	239.355
RS	43	13425	Novo Machado	3.806
RS	43	13441	Novo Tiradentes	2.257
RS	43	13466	Novo Xingu	1.744
RS	43	13508	Osório	41.628
RS	43	13607	Paim Filho	4.155
RS	43	13656	Palmares do Sul	10.987
RS	43	13706	Palmeira das Missões	34.016
RS	43	13805	Palmitinho	6.917
RS	43	13904	Panambi	38.881
RS	43	13953	Pantano Grande	9.732
RS	43	14001	Paraí	6.932
RS	43	14027	Paraíso do Sul	7.355
RS	43	14035	Pareci Novo	3.552
RS	43	14050	Parobé	52.518
RS	43	14068	Passa Sete	5.220
RS	43	14076	Passo do Sobrado	6.079
RS	43	14100	Passo Fundo	187.298
RS	43	14134	Paulo Bento	2.206
RS	43	14159	Paverama	8.090
RS	43	14175	Pedras Altas	2.164
RS	43	14209	Pedro Osório	7.767
RS	43	14308	Pejuçara	3.941

RS	43	14407	Pelotas	329.435
RS	43	14423	Picada Café	5.259
RS	43	14456	Pinhal	2.515
RS	43	14464	Pinhal da Serra	2.089
RS	43	14472	Pinhal Grande	4.433
RS	43	14498	Pinheirinho do Vale	4.545
RS	43	14506	Pinheiro Machado	12.642
RS	43	14555	Pirapó	2.668
RS	43	14605	Piratini	19.906
RS	43	14704	Planalto	10.407
RS	43	14753	Poço das Antas	2.023
RS	43	14779	Pontão	3.850
RS	43	14787	Ponte Preta	1.709
RS	43	14803	Portão	31.866
RS	43	14902	Porto Alegre	1.416.714
RS	43	15008	Porto Lucena	5.265
RS	43	15057	Porto Mauá	2.503
RS	43	15073	Porto Vera Cruz	1.760
RS	43	15107	Porto Xavier	10.463
RS	43	15131	Pouso Novo	1.827
RS	43	15149	Presidente Lucena	2.547
RS	43	15156	Progresso	6.153
RS	43	15172	Protásio Alves	1.984
RS	43	15206	Putinga	4.087
RS	43	15305	Quaraí	22.873
RS	43	15313	Quatro Irmãos	1.779
RS	43	15321	Quevedos	2.713
RS	43	15354	Quinze de Novembro	3.664
RS	43	15404	Redentora	10.430
RS	43	15453	Relvado	2.136
RS	43	15503	Restinga Seca	15.828
RS	43	15552	Rio dos Índios	3.453
RS	43	15602	Rio Grande	198.842
RS	43	15701	Rio Pardo	37.563
RS	43	15750	Riozinho	4.370
RS	43	15800	Roca Sales	10.436
RS	43	15909	Rodeio Bonito	5.742
RS	43	15958	Rolador	2.498
RS	43	16006	Rolante	19.732
RS	43	16105	Ronda Alta	10.247
RS	43	16204	Rondinha	5.430
RS	43	16303	Roque Gonzales	7.114
RS	43	16402	Rosário do Sul	39.503
RS	43	16428	Sagrada Família	2.587
RS	43	16436	Saldanha Marinho	2.820
RS	43	16451	Salto do Jacuí	11.933

RS	43	16477	Salvador das Missões	2.670
RS	43	16501	Salvador do Sul	6.903
RS	43	16600	Sananduva	15.468
RS	43	16709	Santa Bárbara do Sul	8.650
RS	43	16733	Santa Cecília do Sul	1.646
RS	43	16758	Santa Clara do Sul	5.832
RS	43	16808	Santa Cruz do Sul	119.997
RS	43	16972	Santa Margarida do Sul	2.380
RS	43	16907	Santa Maria	263.662
RS	43	16956	Santa Maria do Herval	6.078
RS	43	17202	Santa Rosa	69.127
RS	43	17251	Santa Tereza	1.725
RS	43	17301	Santa Vitória do Palmar	30.641
RS	43	17004	Santana da Boa Vista	8.185
RS	43	17103	Santana do Livramento	81.198
RS	43	17400	Santiago	48.940
RS	43	17509	Santo Ângelo	76.205
RS	43	17608	Santo Antônio da Patrulha	40.086
RS	43	17707	Santo Antônio das Missões	10.987
RS	43	17558	Santo Antônio do Palma	2.129
RS	43	17756	Santo Antônio do Planalto	1.985
RS	43	17806	Santo Augusto	13.899
RS	43	17905	Santo Cristo	14.301
RS	43	17954	Santo Expedito do Sul	2.428
RS	43	18002	São Borja	61.189
RS	43	18051	São Domingos do Sul	2.941
RS	43	18101	São Francisco de Assis	19.020
RS	43	18200	São Francisco de Paula	20.660
RS	43	18309	São Gabriel	60.478
RS	43	18408	São Jerônimo	22.414
RS	43	18424	São João da Urtiga	4.696
RS	43	18432	São João do Polêsine	2.572
RS	43	18440	São Jorge	2.759
RS	43	18457	São José das Missões	2.678
RS	43	18465	São José do Herval	2.155
RS	43	18481	São José do Hortêncio	4.201
RS	43	18499	São José do Inhacorá	2.170
RS	43	18507	São José do Norte	25.761
RS	43	18606	São José do Ouro	6.882
RS	43	18614	São José do Sul	2.132
RS	43	18622	São José dos Ausentes	3.319
RS	43	18705	São Leopoldo	217.189
RS	43	18804	São Lourenço do Sul	43.024
RS	43	18903	São Luiz Gonzaga	34.235
RS	43	19000	São Marcos	20.276
RS	43	19109	São Martinho	5.691

RS	43	19125	São Martinho da Serra	3.195
RS	43	19158	São Miguel das Missões	7.436
RS	43	19208	São Nicolau	5.625
RS	43	19307	São Paulo das Missões	6.240
RS	43	19356	São Pedro da Serra	3.387
RS	43	19364	São Pedro das Missões	1.900
RS	43	19372	São Pedro do Butiá	2.875
RS	43	19406	São Pedro do Sul	16.275
RS	43	19505	São Sebastião do Caí	22.270
RS	43	19604	São Sepé	23.674
RS	43	19703	São Valentim	3.560
RS	43	19711	São Valentim do Sul	2.173
RS	43	19737	São Valério do Sul	2.651
RS	43	19752	São Vendelino	1.984
RS	43	19802	São Vicente do Sul	8.456
RS	43	19901	Sapiranga	75.861
RS	43	20008	Sapucaia do Sul	132.197
RS	43	20107	Sarandi	21.757
RS	43	20206	Seberi	10.829
RS	43	20230	Sede Nova	2.982
RS	43	20263	Segredo	7.087
RS	43	20305	Selbach	4.940
RS	43	20321	Senador Salgado Filho	2.797
RS	43	20354	Sentinela do Sul	5.245
RS	43	20404	Serafina Corrêa	14.761
RS	43	20453	Sério	2.217
RS	43	20503	Sertão	6.118
RS	43	20552	Sertão Santana	5.938
RS	43	20578	Sete de Setembro	2.089
RS	43	20602	Severiano de Almeida	3.796
RS	43	20651	Silveira Martins	2.416
RS	43	20677	Sinimbu	10.047
RS	43	20701	Sobradinho	14.348
RS	43	20800	Soledade	30.092
RS	43	20859	Tabaí	4.217
RS	43	20909	Tapejara	20.017
RS	43	21006	Tapera	10.431
RS	43	21105	Tapes	16.681
RS	43	21204	Taquara	54.918
RS	43	21303	Taquari	26.123
RS	43	21329	Taquaruçu do Sul	2.973
RS	43	21352	Tavares	5.353
RS	43	21402	Tenente Portela	13.625
RS	43	21436	Terra de Areia	10.070
RS	43	21451	Teutônia	28.198
RS	43	21469	Tio Hugo	2.767

RS	43	21477	Tiradentes do Sul	6.305
RS	43	21493	Toropi	2.916
RS	43	21501	Torres	35.227
RS	43	21600	Tramandaí	43.178
RS	43	21626	Travesseiro	2.309
RS	43	21634	Três Arroios	2.812
RS	43	21667	Três Cachoeiras	10.322
RS	43	21709	Três Coroas	24.516
RS	43	21808	Três de Maio	23.665
RS	43	21832	Três Forquilhas	2.865
RS	43	21857	Três Palmeiras	4.345
RS	43	21907	Três Passos	23.861
RS	43	21956	Trindade do Sul	5.767
RS	43	22004	Triunfo	26.341
RS	43	22103	Tucunduva	5.837
RS	43	22152	Tunas	4.408
RS	43	22186	Tupanci do Sul	1.550
RS	43	22202	Tupanciretã	22.483
RS	43	22251	Tupandi	4.070
RS	43	22301	Tuparendi	8.409
RS	43	22327	Turuçu	3.494
RS	43	22343	Ubiretama	2.239
RS	43	22350	União da Serra	1.424
RS	43	22376	Unistalda	2.421
RS	43	22400	Uruguaiana	125.209
RS	43	22509	Vacaria	61.947
RS	43	22533	Vale do Sol	11.156
RS	43	22541	Vale Real	5.236
RS	43	22525	Vale Verde	3.283
RS	43	22558	Vanini	1.999
RS	43	22608	Venâncio Aires	66.658
RS	43	22707	Vera Cruz	24.389
RS	43	22806	Veranópolis	23.315
RS	43	22855	Vespasiano Correa	1.939
RS	43	22905	Viadutos	5.194
RS	43	23002	Viamão	241.190
RS	43	23101	Vicente Dutra	5.158
RS	43	23200	Victor Graeff	2.998
RS	43	23309	Vila Flores	3.226
RS	43	23358	Vila Lângaro	2.134
RS	43	23408	Vila Maria	4.229
RS	43	23457	Vila Nova do Sul	4.215
RS	43	23507	Vista Alegre	2.808
RS	43	23606	Vista Alegre do Prata	1.562
RS	43	23705	Vista Gaúcha	2.765
RS	43	23754	Vitória das Missões	3.411

RS	43	23770	Westfalia	2.821
RS	43	23804	Xangri-lá	13.074
MS	50	00203	Água Clara	13.358
MS	50	00252	Alcinópolis	4.704
MS	50	00609	Amambaí	35.523
MS	50	00708	Anastácio	24.041
MS	50	00807	Anaurilândia	8.575
MS	50	00856	Angélica	9.462
MS	50	00906	Antônio João	8.329
MS	50	01003	Aparecida do Taboado	22.912
MS	50	01102	Aquidauana	45.943
MS	50	01243	Aral Moreira	10.583
MS	50	01508	Bandeirantes	6.637
MS	50	01904	Bataguassu	20.389
MS	50	02001	Batayporã	10.983
MS	50	02100	Bela Vista	23.395
MS	50	02159	Bodoquena	7.928
MS	50	02209	Bonito	19.985
MS	50	02308	Brasilândia	11.807
MS	50	02407	Caarapó	26.532
MS	50	02605	Camapuã	13.609
MS	50	02704	Campo Grande	805.397
MS	50	02803	Caracol	5.520
MS	50	02902	Cassilândia	21.099
MS	50	02951	Chapadão do Sul	19.974
MS	50	03108	Corguinho	5.054
MS	50	03157	Coronel Sapucaia	14.254
MS	50	03207	Corumbá	104.912
MS	50	03256	Costa Rica	18.087
MS	50	03306	Coxim	32.355
MS	50	03454	Deodápolis	12.259
MS	50	03488	Dois Irmãos do Buriti	10.519
MS	50	03504	Douradina	5.460
MS	50	03702	Dourados	200.729
MS	50	03751	Eldorado	11.790
MS	50	03801	Fátima do Sul	19.024
MS	50	03900	Figueirão	2.945
MS	50	04007	Glória de Dourados	9.911
MS	50	04106	Guia Lopes da Laguna	10.253
MS	50	04304	Iguatemi	15.065
MS	50	04403	Inocência	7.639
MS	50	04502	Itaporã	21.442
MS	50	04601	Itaquiraí	19.044
MS	50	04700	Ivinhema	22.447
MS	50	04809	Japorã	7.972
MS	50	04908	Jaraguari	6.485

MS	50	05004	Jardim	24.619
MS	50	05103	Jateí	4.005
MS	50	05152	Juti	6.039
MS	50	05202	Ladário	20.267
MS	50	05251	Laguna Carapã	6.636
MS	50	05400	Maracaju	39.095
MS	50	05608	Miranda	25.986
MS	50	05681	Mundo Novo	17.251
MS	50	05707	Naviraí	47.899
MS	50	05806	Nioaque	14.287
MS	50	06002	Nova Alvorada do Sul	17.410
MS	50	06200	Nova Andradina	47.126
MS	50	06259	Novo Horizonte do Sul	4.718
MS	50	06275	Paraíso das Águas (**)	4.723
MS	50	06309	Paranaíba	40.462
MS	50	06358	Paranhos	12.673
MS	50	06408	Pedro Gomes	7.882
MS	50	06606	Ponta Porã	80.433
MS	50	06903	Porto Murtinho	15.683
MS	50	07109	Ribas do Rio Pardo	21.584
MS	50	07208	Rio Brilhante	31.875
MS	50	07307	Rio Negro	4.977
MS	50	07406	Rio Verde de Mato Grosso	19.004
MS	50	07505	Rochedo	5.015
MS	50	07554	Santa Rita do Pardo	7.353
MS	50	07695	São Gabriel do Oeste	23.016
MS	50	07802	Selvíria	6.318
MS	50	07703	Sete Quedas	10.757
MS	50	07901	Sidrolândia	44.949
MS	50	07935	Sonora	15.632
MS	50	07950	Tacuru	10.442
MS	50	07976	Taquarussu	3.522
MS	50	08008	Terenos	17.975
MS	50	08305	Três Lagoas	105.224
MS	50	08404	Vicentina	5.920
MT	51	00102	Acorizal	5.471
MT	51	00201	Água Boa	21.778
MT	51	00250	Alta Floresta	49.494
MT	51	00300	Alto Araguaia	16.284
MT	51	00359	Alto Boa Vista	5.553
MT	51	00409	Alto Garças	10.655
MT	51	00508	Alto Paraguai	10.290
MT	51	00607	Alto Taquari	8.615
MT	51	00805	Apiacás	8.855
MT	51	01001	Araguaiana	3.163
MT	51	01209	Araguainha	1.058

MT	51	01258	Araputanga	15.594
MT	51	01308	Arenápolis	10.122
MT	51	01407	Aripuanã	19.344
MT	51	01605	Barão de Melgaço	7.578
MT	51	01704	Barra do Bugres	32.464
MT	51	01803	Barra do Garças	57.235
MT	51	01852	Bom Jesus do Araguaia	5.555
MT	51	01902	Brasnorte	16.194
MT	51	02504	Cáceres	88.897
MT	51	02603	Campinápolis	14.590
MT	51	02637	Campo Novo do Parecis	29.078
MT	51	02678	Campo Verde	33.759
MT	51	02686	Campos de Júlio	5.494
MT	51	02694	Canabrava do Norte	4.756
MT	51	02702	Canarana	19.260
MT	51	02793	Carlinda	10.793
MT	51	02850	Castanheira	8.298
MT	51	03007	Chapada dos Guimarães	18.133
MT	51	03056	Cláudia	11.213
MT	51	03106	Cocalinho	5.510
MT	51	03205	Colíder	31.176
MT	51	03254	Colniza	28.810
MT	51	03304	Comodoro	18.651
MT	51	03353	Confresa	26.224
MT	51	03361	Conquista D'Oeste	3.506
MT	51	03379	Cotriguaçu	15.912
MT	51	03403	Cuiabá	561.329
MT	51	03437	Curvelândia	4.918
MT	51	03452	Denise	8.684
MT	51	03502	Diamantino	20.605
MT	51	03601	Dom Aquino	8.134
MT	51	03700	Feliz Natal	11.562
MT	51	03809	Figueirópolis D'Oeste	3.718
MT	51	03858	Gaúcha do Norte	6.548
MT	51	03908	General Carneiro	5.130
MT	51	03957	Glória D'Oeste	3.101
MT	51	04104	Guarantã do Norte	32.823
MT	51	04203	Guiratinga	14.137
MT	51	04500	Indiavaí	2.449
MT	51	04526	Ipiranga do Norte	5.631
MT	51	04542	Itanhangá	5.558
MT	51	04559	Itaúba	4.393
MT	51	04609	Itiquira	11.822
MT	51	04807	Jaciara	25.927
MT	51	04906	Jangada	7.781
MT	51	05002	Jauru	10.062

MT	51	05101	Juara	33.100
MT	51	05150	Juína	39.442
MT	51	05176	Juruena	12.125
MT	51	05200	Juscimeira	11.335
MT	51	05234	Lambari D'Oeste	5.550
MT	51	05259	Lucas do Rio Verde	49.519
MT	51	05309	Luciára	2.184
MT	51	05580	Marcelândia	11.638
MT	51	05606	Matupá	14.610
MT	51	05622	Mirassol d'Oeste	25.684
MT	51	05903	Nobres	15.004
MT	51	06000	Nortelândia	6.314
MT	51	06109	Nossa Senhora do Livramento	11.550
MT	51	06158	Nova Bandeirantes	12.352
MT	51	06208	Nova Brasilândia	4.406
MT	51	06216	Nova Canaã do Norte	12.220
MT	51	08808	Nova Guarita	4.824
MT	51	06182	Nova Lacerda	5.648
MT	51	08857	Nova Marilândia	3.007
MT	51	08907	Nova Maringá	6.989
MT	51	08956	Nova Monte Verde	8.285
MT	51	06224	Nova Mutum	34.374
MT	51	06174	Nova Nazaré	3.187
MT	51	06232	Nova Olímpia	18.018
MT	51	06190	Nova Santa Helena	3.505
MT	51	06240	Nova Ubiratã	9.757
MT	51	06257	Nova Xavantina	19.917
MT	51	06273	Novo Horizonte do Norte	3.785
MT	51	06265	Novo Mundo	7.685
MT	51	06315	Novo Santo Antônio	2.129
MT	51	06281	Novo São Joaquim	5.810
MT	51	06299	Paranaíta	10.749
MT	51	06307	Paranatinga	19.887
MT	51	06372	Pedra Preta	16.079
MT	51	06422	Peixoto de Azevedo	31.516
MT	51	06455	Planalto da Serra	2.703
MT	51	06505	Poconé	31.931
MT	51	06653	Pontal do Araguaia	5.646
MT	51	06703	Ponte Branca	1.720
MT	51	06752	Pontes e Lacerda	42.063
MT	51	06778	Porto Alegre do Norte	11.069
MT	51	06802	Porto dos Gaúchos	5.417
MT	51	06828	Porto Esperidião	11.188
MT	51	06851	Porto Estrela	3.490
MT	51	07008	Poxoréo	17.232
MT	51	07040	Primavera do Leste	53.910

MT	51	07065	Querência	13.903
MT	51	07156	Reserva do Cabaçal	2.595
MT	51	07180	Ribeirão Cascalheira	9.118
MT	51	07198	Ribeirãozinho	2.233
MT	51	07206	Rio Branco	5.067
MT	51	07578	Rondolândia	3.671
MT	51	07602	Rondonópolis	202.309
MT	51	07701	Rosário Oeste	17.526
MT	51	07750	Salto do Céu	3.777
MT	51	07248	Santa Carmem	4.159
MT	51	07743	Santa Cruz do Xingu	2.031
MT	51	07768	Santa Rita do Trivelato	2.676
MT	51	07776	Santa Terezinha	7.568
MT	51	07263	Santo Afonso	3.010
MT	51	07792	Santo Antônio do Leste	4.038
MT	51	07800	Santo Antônio do Leverger	18.921
MT	51	07859	São Félix do Araguaia	10.804
MT	51	07297	São José do Povo	3.673
MT	51	07305	São José do Rio Claro	17.786
MT	51	07354	São José do Xingu	5.291
MT	51	07107	São José dos Quatro Marcos	18.894
MT	51	07404	São Pedro da Cipa	4.259
MT	51	07875	Sapezal	19.639
MT	51	07883	Serra Nova Dourada	1.419
MT	51	07909	Sinop	118.833
MT	51	07925	Sorriso	71.190
MT	51	07941	Tabaporã	9.795
MT	51	07958	Tangará da Serra	87.145
MT	51	08006	Tapurah	11.042
MT	51	08055	Terra Nova do Norte	10.929
MT	51	08105	Tesouro	3.454
MT	51	08204	Torixoréu	3.957
MT	51	08303	União do Sul	3.695
MT	51	08352	Vale de São Domingos	3.052
MT	51	08402	Várzea Grande	258.208
MT	51	08501	Vera	10.414
MT	51	05507	Vila Bela da Santíssima Trindade	14.770
MT	51	08600	Vila Rica	22.258
GO	52	00050	Abadia de Goiás	7.164
GO	52	00100	Abadiânia	16.408
GO	52	00134	Acreúna	20.578
GO	52	00159	Adelândia	2.480
GO	52	00175	Água Fria de Goiás	5.184
GO	52	00209	Água Limpa	1.985
GO	52	00258	Águas Lindas de Goiás	167.477
GO	52	00308	Alexânia	24.383

GO	52	00506	Aloândia	2.040
GO	52	00555	Alto Horizonte	4.799
GO	52	00605	Alto Paraíso de Goiás	6.992
GO	52	00803	Alvorada do Norte	8.164
GO	52	00829	Amaralina	3.489
GO	52	00852	Americano do Brasil	5.595
GO	52	00902	Amorinópolis	3.529
GO	52	01108	Anápolis	342.347
GO	52	01207	Anhanguera	1.039
GO	52	01306	Anicuns	20.464
GO	52	01405	Aparecida de Goiânia	474.219
GO	52	01454	Aparecida do Rio Doce	2.431
GO	52	01504	Aporé	3.860
GO	52	01603	Araçu	3.753
GO	52	01702	Aragarças	18.564
GO	52	01801	Aragoiânia	8.659
GO	52	02155	Araguapaz	7.541
GO	52	02353	Arenópolis	3.168
GO	52	02502	Aruanã	7.859
GO	52	02601	Aurilândia	3.562
GO	52	02809	Avelinópolis	2.442
GO	52	03104	Baliza	3.933
GO	52	03203	Barro Alto	9.089
GO	52	03302	Bela Vista de Goiás	25.361
GO	52	03401	Bom Jardim de Goiás	8.477
GO	52	03500	Bom Jesus de Goiás	21.402
GO	52	03559	Bonfinópolis	7.866
GO	52	03575	Bonópolis	3.640
GO	52	03609	Brazabrantes	3.302
GO	52	03807	Britânia	5.544
GO	52	03906	Buriti Alegre	9.105
GO	52	03939	Buriti de Goiás	2.546
GO	52	03962	Buritinópolis	3.312
GO	52	04003	Cabeceiras	7.444
GO	52	04102	Cachoeira Alta	10.841
GO	52	04201	Cachoeira de Goiás	1.405
GO	52	04250	Cachoeira Dourada	8.214
GO	52	04300	Caçu	13.692
GO	52	04409	Caiapônia	17.072
GO	52	04508	Caldas Novas	73.616
GO	52	04557	Caldazinha	3.396
GO	52	04607	Campestre de Goiás	3.421
GO	52	04656	Campinaçu	3.649
GO	52	04706	Campinorte	11.333
GO	52	04805	Campo Alegre de Goiás	6.292
GO	52	04854	Campo Limpo de Goiás	6.476

GO	52	04904	Campos Belos	18.616
GO	52	04953	Campos Verdes	4.562
GO	52	05000	Carmo do Rio Verde	9.097
GO	52	05059	Castelândia	3.602
GO	52	05109	Catalão	90.004
GO	52	05208	Caturaí	4.740
GO	52	05307	Cavalcante	9.429
GO	52	05406	Ceres	20.924
GO	52	05455	Cezarina	7.701
GO	52	05471	Chapadão do Céu	7.488
GO	52	05497	Cidade Ocidental	58.262
GO	52	05513	Cocalzinho de Goiás	17.827
GO	52	05521	Colinas do Sul	3.496
GO	52	05703	Córrego do Ouro	2.581
GO	52	05802	Corumbá de Goiás	10.464
GO	52	05901	Corumbaíba	8.412
GO	52	06206	Cristalina	48.463
GO	52	06305	Cristianópolis	2.934
GO	52	06404	Crixás	15.925
GO	52	06503	Cromínia	3.540
GO	52	06602	Cumari	2.943
GO	52	06701	Damianópolis	3.291
GO	52	06800	Damolândia	2.774
GO	52	06909	Davinópolis	2.060
GO	52	07105	Diorama	2.477
GO	52	08301	Divinópolis de Goiás	4.931
GO	52	07253	Doverlândia	7.792
GO	52	07352	Edealina	3.723
GO	52	07402	Edéia	11.424
GO	52	07501	Estrela do Norte	3.309
GO	52	07535	Faina	6.918
GO	52	07600	Fazenda Nova	6.206
GO	52	07808	Firminópolis	11.833
GO	52	07907	Flores de Goiás	12.754
GO	52	08004	Formosa	103.322
GO	52	08103	Formoso	4.777
GO	52	08152	Gameleira de Goiás	3.378
GO	52	08400	Goianápolis	10.699
GO	52	08509	Goiandira	5.310
GO	52	08608	Goianésia	61.118
GO	52	08707	Goiânia	1.333.767
GO	52	08806	Goianira	35.617
GO	52	08905	Goiás	24.366
GO	52	09101	Goiatuba	32.698
GO	52	09150	Gouvelândia	5.091
GO	52	09200	Guapó	13.994

GO	52	09291	Guaraíta	2.313
GO	52	09408	Guarani de Goiás	4.195
GO	52	09457	Guarinos	2.217
GO	52	09606	Heitoraí	3.591
GO	52	09705	Hidrolândia	18.050
GO	52	09804	Hidrolina	3.951
GO	52	09903	Iaciara	12.648
GO	52	09937	Inaciolândia	5.769
GO	52	09952	Indiara	13.970
GO	52	10000	Inhumas	48.903
GO	52	10109	Ipameri	25.054
GO	52	10158	Ipiranga de Goiás	2.848
GO	52	10208	Iporá	31.271
GO	52	10307	Israelândia	2.870
GO	52	10406	Itaberaí	36.503
GO	52	10562	Itaguari	4.533
GO	52	10604	Itaguaru	5.398
GO	52	10802	Itajá	4.973
GO	52	10901	Itapaci	19.142
GO	52	11008	Itapirapuã	7.379
GO	52	11206	Itapuranga	26.033
GO	52	11305	Itarumã	6.429
GO	52	11404	Itauçu	8.620
GO	52	11503	Itumbiara	94.613
GO	52	11602	Ivolândia	2.614
GO	52	11701	Jandaia	6.138
GO	52	11800	Jaraguá	43.167
GO	52	11909	Jataí	89.902
GO	52	12006	Jaupaci	2.977
GO	52	12055	Jesúpolis	2.327
GO	52	12105	Joviânia	7.151
GO	52	12204	Jussara	19.020
GO	52	12253	Lagoa Santa	1.305
GO	52	12303	Leopoldo de Bulhões	7.900
GO	52	12501	Luziânia	179.582
GO	52	12600	Mairipotaba	2.370
GO	52	12709	Mambaí	7.178
GO	52	12808	Mara Rosa	10.455
GO	52	12907	Marzagão	2.095
GO	52	12956	Matrinchã	4.398
GO	52	13004	Maurilândia	11.907
GO	52	13053	Mimoso de Goiás	2.668
GO	52	13087	Minaçu	30.784
GO	52	13103	Mineiros	55.036
GO	52	13400	Moiporá	1.724
GO	52	13509	Monte Alegre de Goiás	7.857

GO	52	13707	Montes Claros de Goiás	7.987
GO	52	13756	Montividiu	11.001
GO	52	13772	Montividiu do Norte	4.173
GO	52	13806	Morrinhos	42.135
GO	52	13855	Morro Agudo de Goiás	2.336
GO	52	13905	Mossâmedes	4.888
GO	52	14002	Mozarlândia	13.739
GO	52	14051	Mundo Novo	6.186
GO	52	14101	Mutunópolis	3.833
GO	52	14408	Nazário	8.062
GO	52	14507	Nerópolis	25.061
GO	52	14606	Niquelândia	42.933
GO	52	14705	Nova América	2.271
GO	52	14804	Nova Aurora	2.083
GO	52	14838	Nova Crixás	12.058
GO	52	14861	Nova Glória	8.443
GO	52	14879	Nova Iguaçu de Goiás	2.839
GO	52	14903	Nova Roma	3.434
GO	52	15009	Nova Veneza	8.388
GO	52	15207	Novo Brasil	3.420
GO	52	15231	Novo Gama	98.135
GO	52	15256	Novo Planalto	4.036
GO	52	15306	Orizona	14.487
GO	52	15405	Ouro Verde de Goiás	3.986
GO	52	15504	Ouvidor	5.648
GO	52	15603	Padre Bernardo	28.601
GO	52	15652	Palestina de Goiás	3.381
GO	52	15702	Palmeiras de Goiás	24.171
GO	52	15801	Palmelo	2.339
GO	52	15900	Palminópolis	3.557
GO	52	16007	Panamá	2.668
GO	52	16304	Paranaiguara	9.238
GO	52	16403	Paraúna	10.868
GO	52	16452	Perolândia	2.975
GO	52	16809	Petrolina de Goiás	10.269
GO	52	16908	Pilar de Goiás	2.688
GO	52	17104	Piracanjuba	23.987
GO	52	17203	Piranhas	11.112
GO	52	17302	Pirenópolis	23.272
GO	52	17401	Pires do Rio	29.145
GO	52	17609	Planaltina	82.847
GO	52	17708	Pontalina	17.207
GO	52	18003	Porangatu	42.773
GO	52	18052	Porteirão	3.427
GO	52	18102	Portelândia	3.861
GO	52	18300	Posse	32.234

GO	52	18391	Professor Jamil	3.325
GO	52	18508	Quirinópolis	44.233
GO	52	18607	Rialma	10.571
GO	52	18706	Rianápolis	4.597
GO	52	18789	Rio Quente	3.496
GO	52	18805	Rio Verde	185.465
GO	52	18904	Rubiataba	19.041
GO	52	19001	Sanclerlândia	7.554
GO	52	19100	Santa Bárbara de Goiás	5.870
GO	52	19209	Santa Cruz de Goiás	3.093
GO	52	19258	Santa Fé de Goiás	4.865
GO	52	19308	Santa Helena de Goiás	36.760
GO	52	19357	Santa Isabel	3.701
GO	52	19407	Santa Rita do Araguaia	7.202
GO	52	19456	Santa Rita do Novo Destino	3.196
GO	52	19506	Santa Rosa de Goiás	2.813
GO	52	19605	Santa Tereza de Goiás	3.889
GO	52	19704	Santa Terezinha de Goiás	10.044
GO	52	19712	Santo Antônio da Barra	4.480
GO	52	19738	Santo Antônio de Goiás	4.945
GO	52	19753	Santo Antônio do Descoberto	64.963
GO	52	19803	São Domingos	11.520
GO	52	19902	São Francisco de Goiás	6.134
GO	52	20058	São João da Paraúna	1.639
GO	52	20009	São João d'Aliança	10.789
GO	52	20108	São Luís de Montes Belos	30.586
GO	52	20157	São Luíz do Norte	4.697
GO	52	20207	São Miguel do Araguaia	22.206
GO	52	20264	São Miguel do Passa Quatro	3.799
GO	52	20280	São Patrício	1.996
GO	52	20405	São Simão	17.622
GO	52	20454	Senador Canedo	89.176
GO	52	20504	Serranópolis	7.638
GO	52	20603	Silvânia	19.293
GO	52	20686	Simolândia	6.559
GO	52	20702	Sítio d'Abadia	2.847
GO	52	21007	Taquaral de Goiás	3.535
GO	52	21080	Teresina de Goiás	3.082
GO	52	21197	Terezópolis de Goiás	6.785
GO	52	21304	Três Ranchos	2.818
GO	52	21403	Trindade	107.966
GO	52	21452	Trombas	3.455
GO	52	21502	Turvânia	4.795
GO	52	21551	Turvelândia	4.532
GO	52	21577	Uirapuru	2.917
GO	52	21601	Uruaçu	37.443

GO	52	21700	Uruana	13.810
GO	52	21809	Urutaí	3.070
GO	52	21858	Valparaíso de Goiás	138.740
GO	52	21908	Varjão	3.681
GO	52	22005	Vianópolis	12.737
GO	52	22054	Vicentinópolis	7.576
GO	52	22203	Vila Boa	4.954
GO	52	22302	Vila Propício	5.244
DF	53	00108	Brasília	2.648.532

Fonte: IBGE. Diretoria de Pesquisas - DPE - Coordenação de População e Indicadores Sociais -

NOTA: (*) EM DECORRÊNCIA DE DECISÃO JUDICIAL

NOTA: (**) MUNICÍPIOS INSTALADOS EM 1º DE JANEIRO DE 2013
