# Supplementary material for: Forecasting dengue fever in Brazil: An assessment of climate conditions
Source: PLoS One. 2019 Aug 8;14(8):e0220106. doi: 10.1371/journal.pone.0220106 (PMC6687106; doi:10.1371/journal.pone.0220106)
Supplement: S1 File — (ZIP) [file pone.0220106.s003.zip › data and codes_submit/training dataset analysis/data/Data IBGE/pop_capitais_2010.pdf]

Brasil

- Escolha uma opção -

1.6 - População nos Censos Demográficos, segundo os municípios das capitais - 1872/2010

População nos Censos Demográficos, segundo os municípios das capitais - 2000/2010

*Em milhões de pessoas.

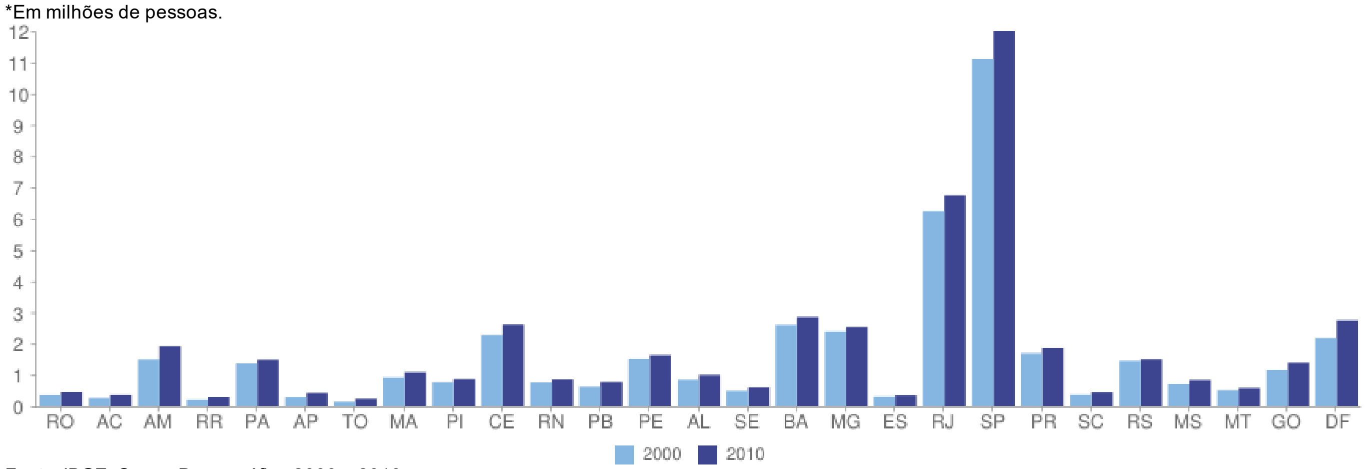

Fonte: IBGE, Censo Demográfico 2000 e 2010.

Tabela 1.6 - População nos Censos Demográficos, segundo os municípios das capitais - 1872/2010

Região	Capital	1872[1]	1890[1]	1900[1]	1920[1]	1940[1]	1950[1]	1960[2]	1970[2]	1980[2]	1991[3]	2000[3]	2010[3]
N	Porto Velho	...	...	...	...	...	27.244	51.049	88.856	138.289	286.471	334.585	428.527
N	Rio Branco	...	...	...	19.930	16.038	28.246	47.882	84.845	119.815	196.871	252.885	336.038
N	Manaus	29.334	38.720	50.300	75.704	106.399	139.620	175.343	314.197	642.492	1.010.544	1.403.796	1.802.014
N	Boa Vista	...	...	...	...	...	17.247	26.168	37.062	69.627	142.902	200.383	284.313
N	Belém	61.997	50.064	96.560	236.402	206.331	254.949	402.170	642.514	949.545	1.244.688	1.279.861	1.393.399
N	Macapá	...	...	...	...	...	20.594	46.905	87.755	140.624	179.252	282.745	398.204
N	Palmas	...	...	...	...	...	...	...	...	3.288	24.261	137.045	228.332
NE	São Luís	31.604	29.308	36.798	52.929	85.583	119.785	159.628	270.651	460.320	695.199	868.047	1.014.837
NE	Teresina	21.692	31.523	45.316	57.500	67.641	90.723	144.799	230.168	388.922	598.411	714.583	814.230
NE	Fortaleza	42.458	40.902	48.369	78.536	180.185	270.169	514.818	872.702	1.338.793	1.765.794	2.138.234	2.452.185
NE	Natal	20.392	13.725	16.056	30.696	54.836	103.215	162.537	270.127	428.721	606.681	709.536	803.739
NE	João Pessoa	24.714	18.645	28.793	52.990	94.333	119.326	155.117	228.418	338.629	497.306	595.429	723.515
NE	Recife	116.671	111.556	113.106	238.843	348.424	524.682	797.234	1.084.459	1.240.937	1.296.995	1.421.993	1.537.704
NE	Maceió	27.703	31.498	36.427	74.166	90.253	120.980	170.134	269.415	409.191	628.241	796.842	932.748
NE	Aracaju	9.559	16.336	21.132	37.440	59.031	78.364	115.713	186.838	299.422	401.676	461.083	571.149
NE	Salvador	129.109	174.412	205.813	283.422	290.443	417.235	655.735	1.027.142	1.531.242	2.072.058	2.440.828	2.675.656
SE	Belo Horizonte	...	...	13.472	55.563	211.377	352.724	693.328	1.255.415	1.822.221	2.017.127	2.232.747	2.375.151
SE	Vitória	16.157	16.887	11.850	21.866	45.212	50.922	85.242	136.391	215.073	258.243	291.941	327.801
SE	Rio de Janeiro	274.972	522.651	811.443	1.157.873	1.764.141	2.377.451	3.307.163	4.315.746	5.183.992	5.473.909	5.851.914	6.320.446
SE	São Paulo	31.385	64.934	239.820	579.033	1.326.261	2.198.096	3.825.351	5.978.977	8.587.665	9.626.894	10.405.867	11.253.503
S	Curitiba	12.651	24.553	49.755	78.986	140.656	180.575	361.309	624.362	1.052.147	1.313.094	1.586.848	1.751.907
S	Florianópolis	25.709	30.687	32.229	41.338	46.771	67.630	98.520	143.414	196.055	254.941	341.781	421.240
S	Porto Alegre	43.998	52.421	73.674	179.263	272.232	394.151	641.173	903.175	1.158.709	1.263.239	1.360.033	1.409.351
CO	Campo Grande	...	...	...	...	49.629	57.033	74.249	143.271	298.878	525.463	662.534	786.797
CO	Cuiabá	35.987	17.815	34.393	33.678	54.394	56.204	57.860	103.427	219.477	401.303	483.044	551.098
CO	Goiânia	...	...	...	...	48.166	53.389	153.505	389.784	738.117	920.840	1.090.737	1.302.001
CO	Brasília	...	...	...	...	...	...	141.742	546.015	1.203.333	1.598.415	2.043.169	2.570.160

Fonte: IBGE, Censo Demográfico 1872, 1890, 1900, 1920,1940, 1950, 1960,1970, 1980,1991, 2000 e 2010.
(1) População presente. (2) População recenseada. (3) População residente.
