# Supplementary figures and images for: Forecasting dengue fever in Brazil: An assessment of climate conditions
Source: PLoS One. 2019 Aug 8;14(8):e0220106. doi: 10.1371/journal.pone.0220106 (PMC6687106; doi:10.1371/journal.pone.0220106)

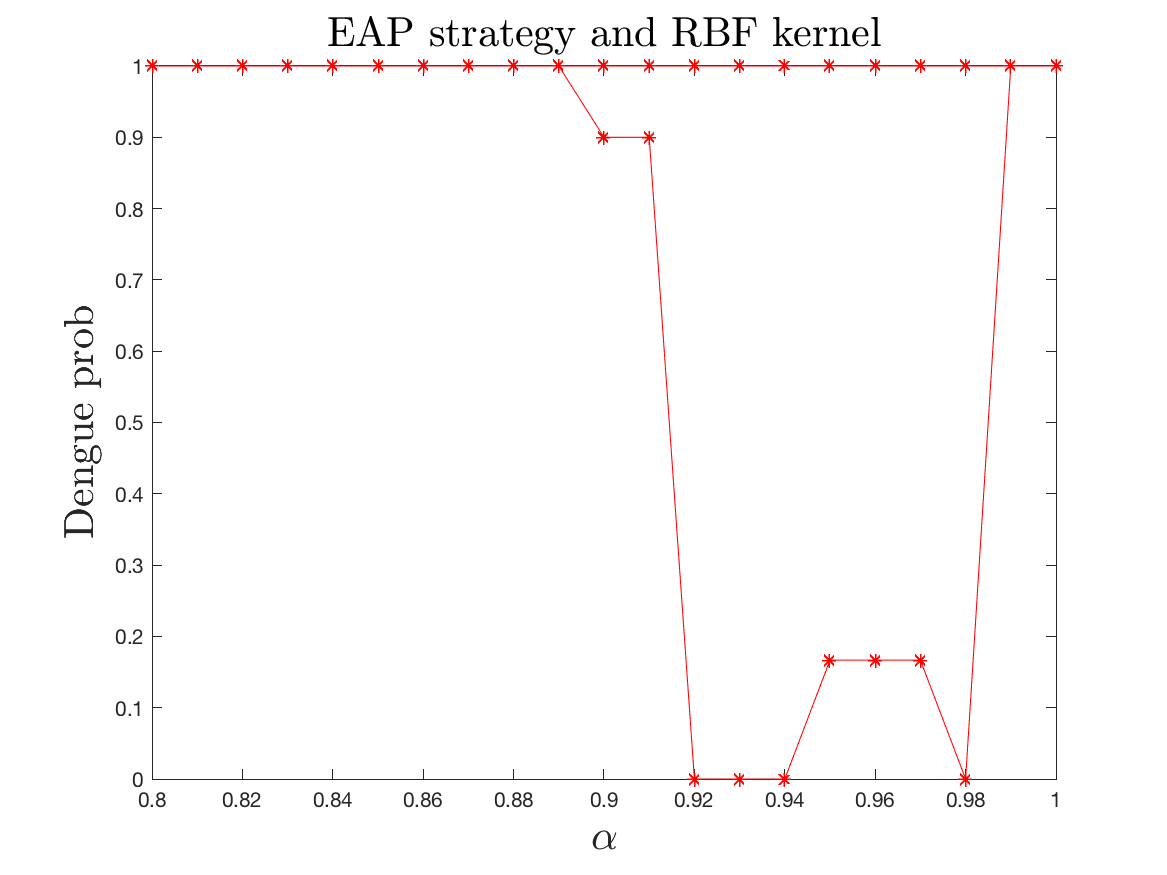

Supplement: S1 File — (ZIP) [file pone.0220106.s003.zip › data and codes_submit/holdout dataset analysis/prediction 2013-2017/capitals/results/Sao Luis/FigureEAP_Sao_Luis_RBF.png]

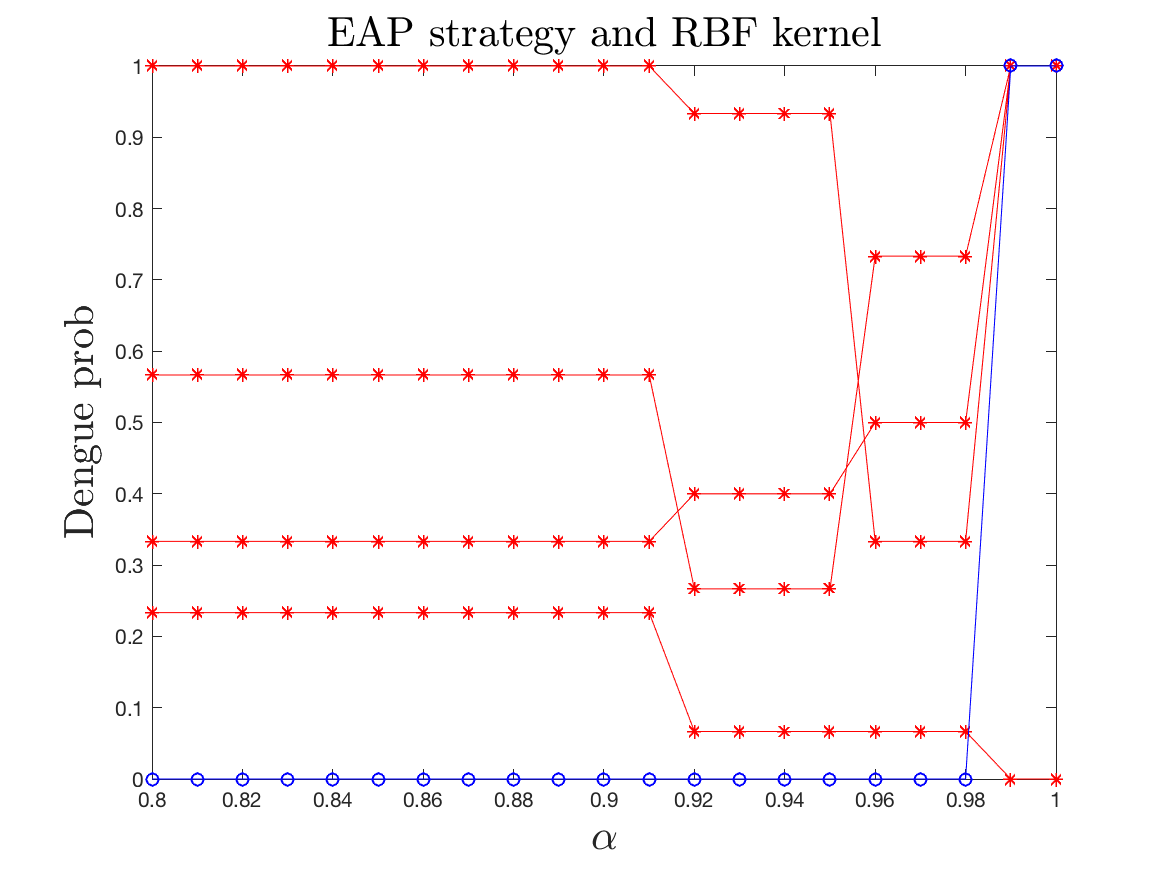

Supplement: S1 File — (ZIP) [file pone.0220106.s003.zip › data and codes_submit/holdout dataset analysis/prediction 2013-2017/capitals/results/Belo Horizonte/FigureEAP_Belo_Horizonte_RBF.png]

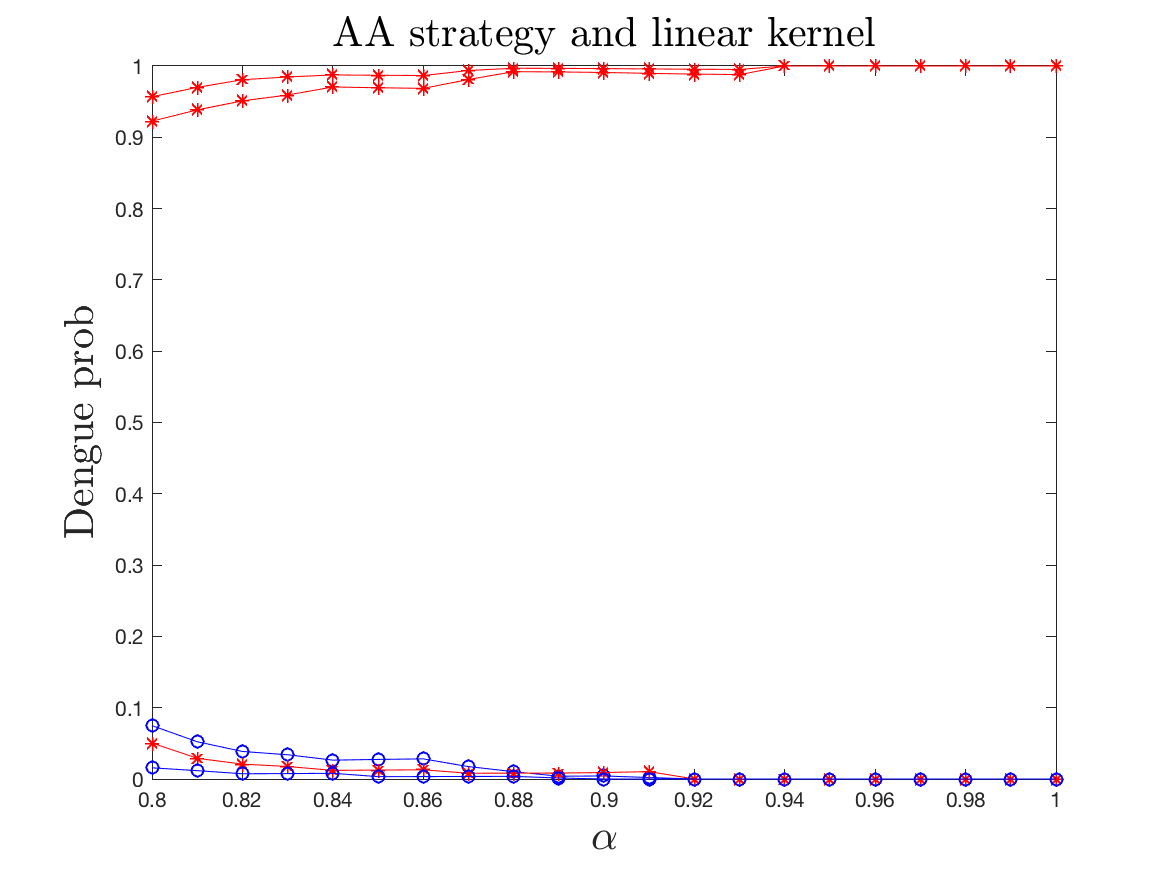

Supplement: S1 File — (ZIP) [file pone.0220106.s003.zip › data and codes_submit/holdout dataset analysis/prediction 2013-2017/capitals/results/Salvador/FigureAA_Salvador_linear.png]

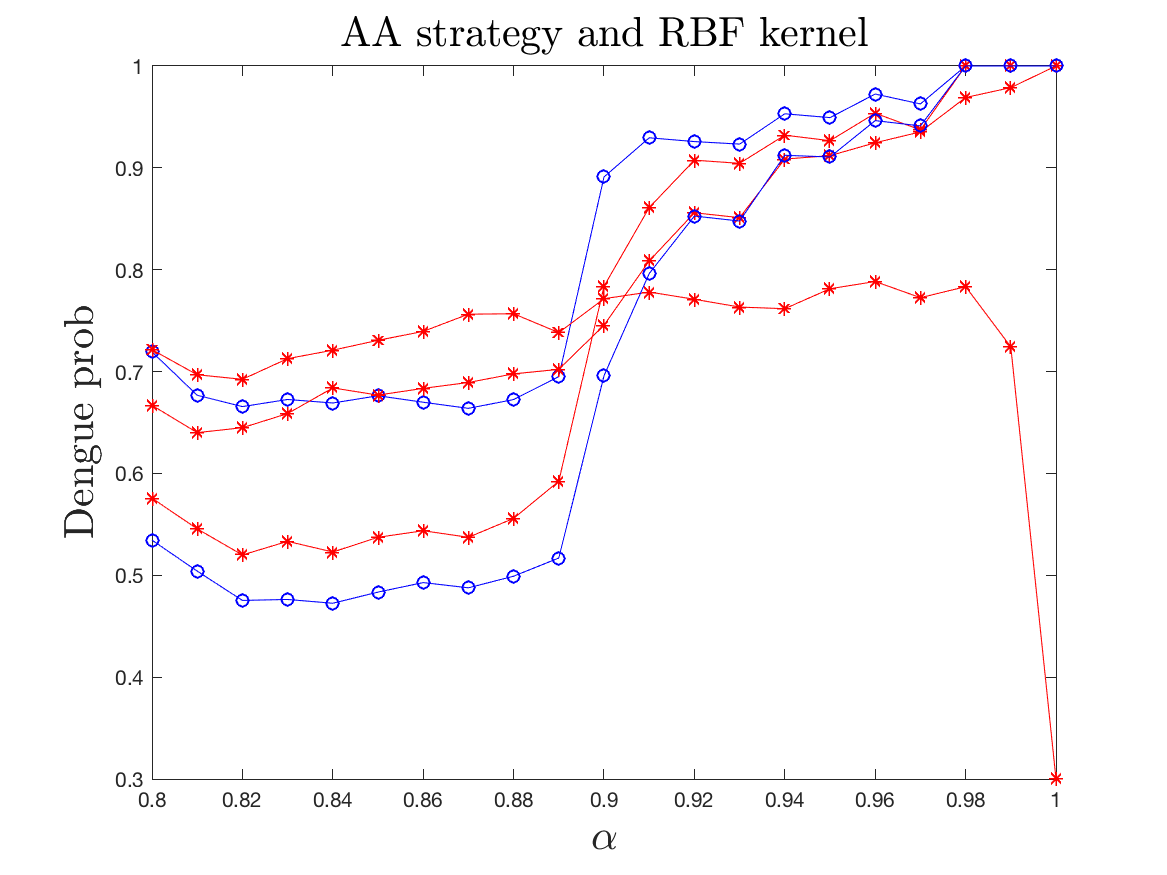

Supplement: S1 File — (ZIP) [file pone.0220106.s003.zip › data and codes_submit/holdout dataset analysis/prediction 2013-2017/capitals/results/Aracaju/FigureAA_Aracaju_RBF.png]

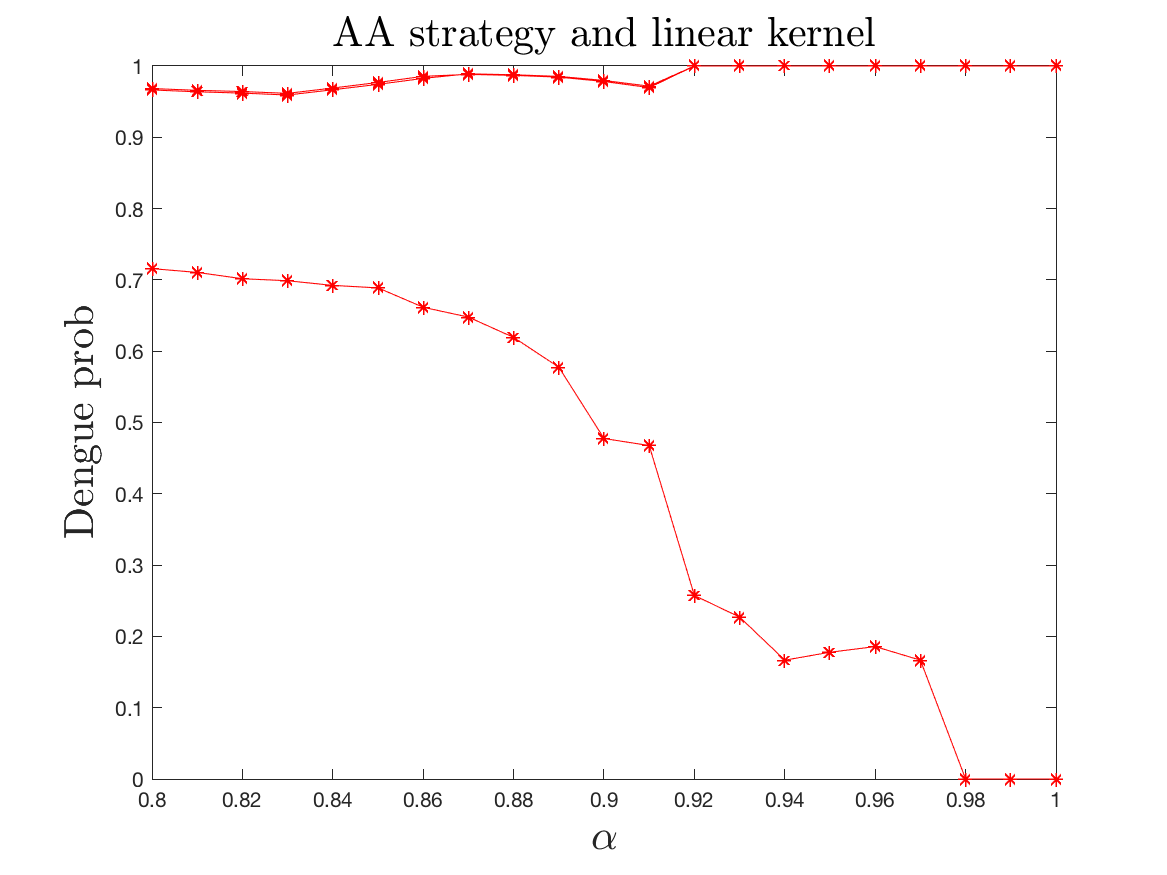

Supplement: S1 File — (ZIP) [file pone.0220106.s003.zip › data and codes_submit/holdout dataset analysis/prediction 2013-2017/capitals/results/Sao Luis/FigureAA_Sao_Luis_linear.png]

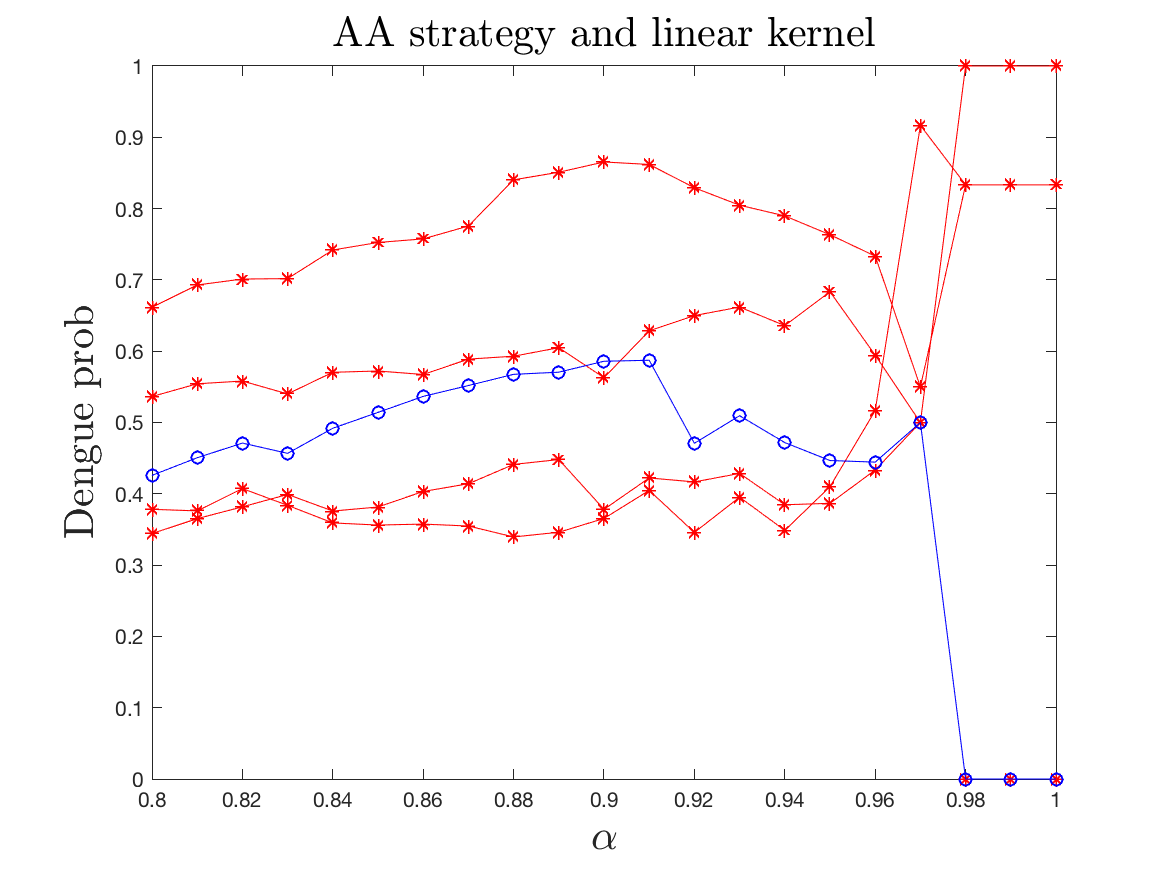

Supplement: S1 File — (ZIP) [file pone.0220106.s003.zip › data and codes_submit/holdout dataset analysis/prediction 2013-2017/capitals/results/Belo Horizonte/FigureAA_Belo_Horizonte_linear.png]

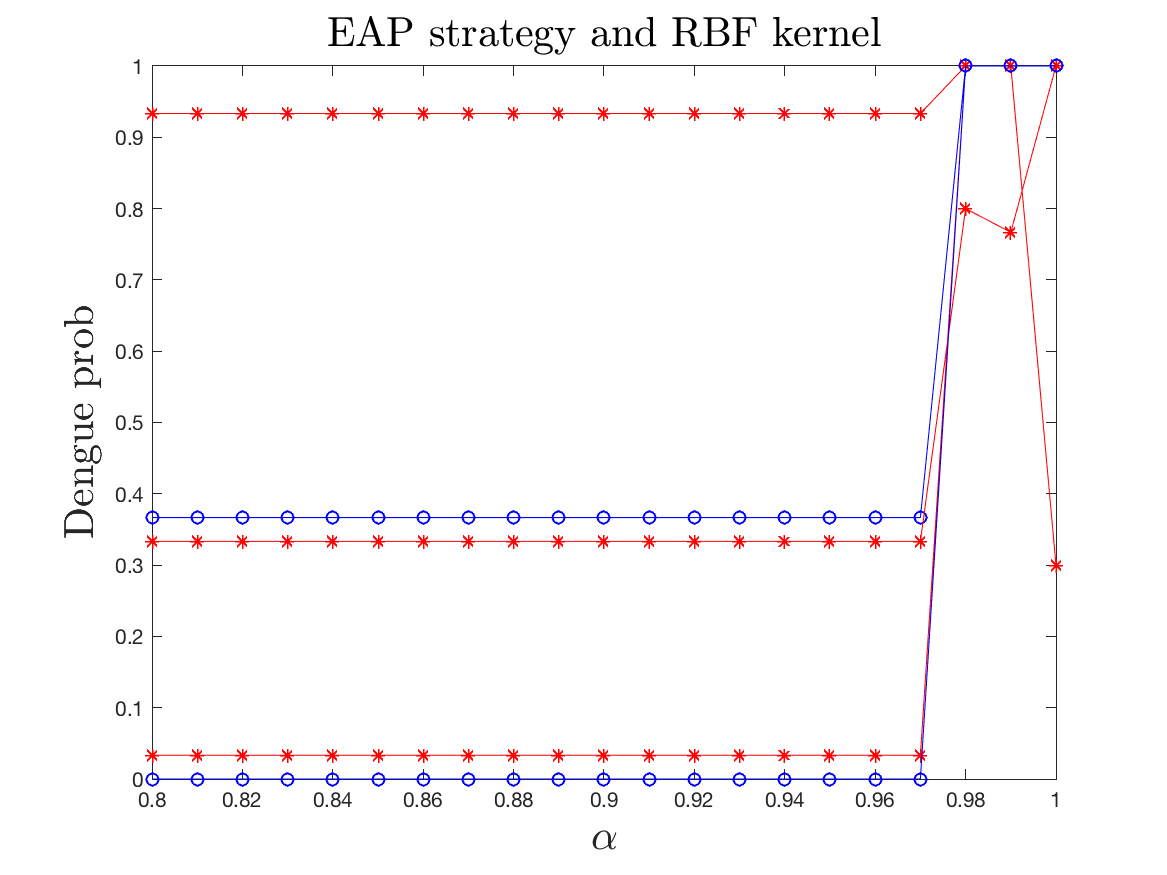

Supplement: S1 File — (ZIP) [file pone.0220106.s003.zip › data and codes_submit/holdout dataset analysis/prediction 2013-2017/capitals/results/Aracaju/FigureEAP_Aracaju_RBF.png]

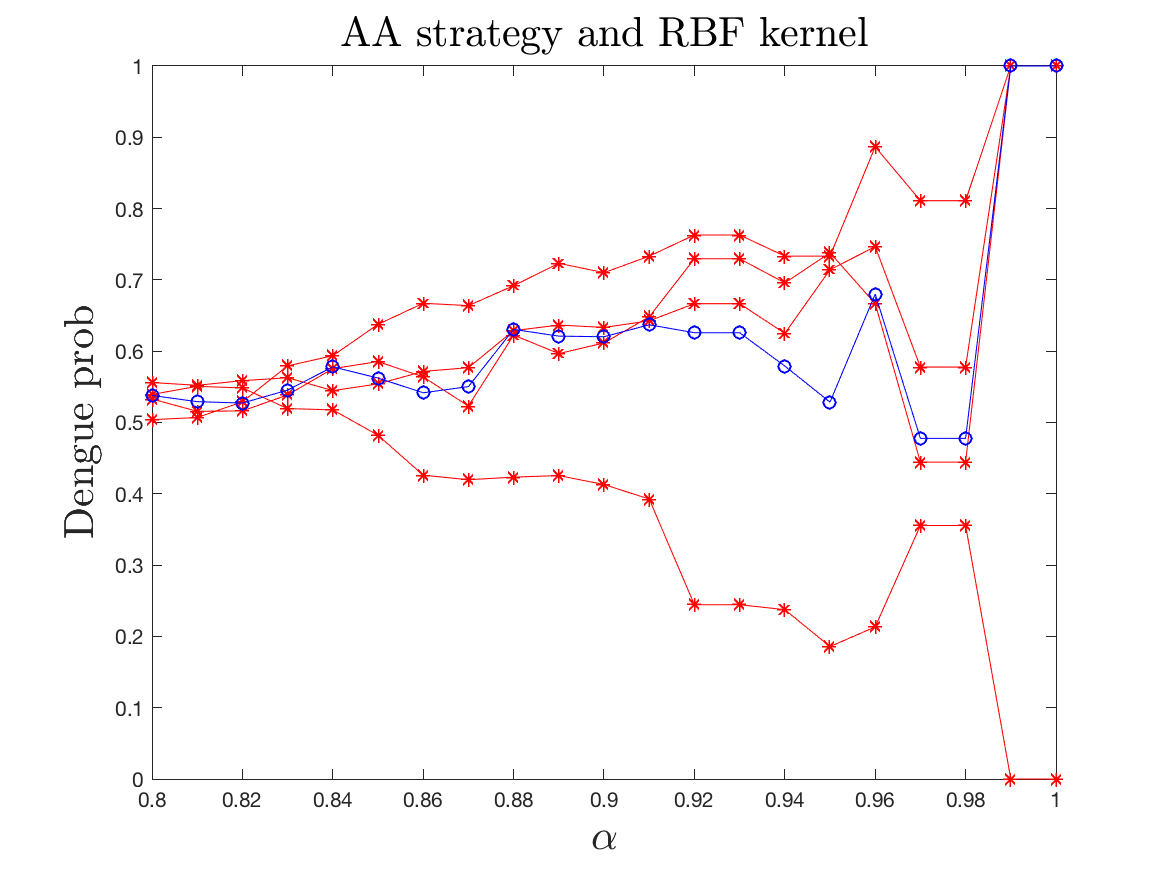

Supplement: S1 File — (ZIP) [file pone.0220106.s003.zip › data and codes_submit/holdout dataset analysis/prediction 2013-2017/capitals/results/Belo Horizonte/FigureAA_Belo_Horizonte_RBF.png]

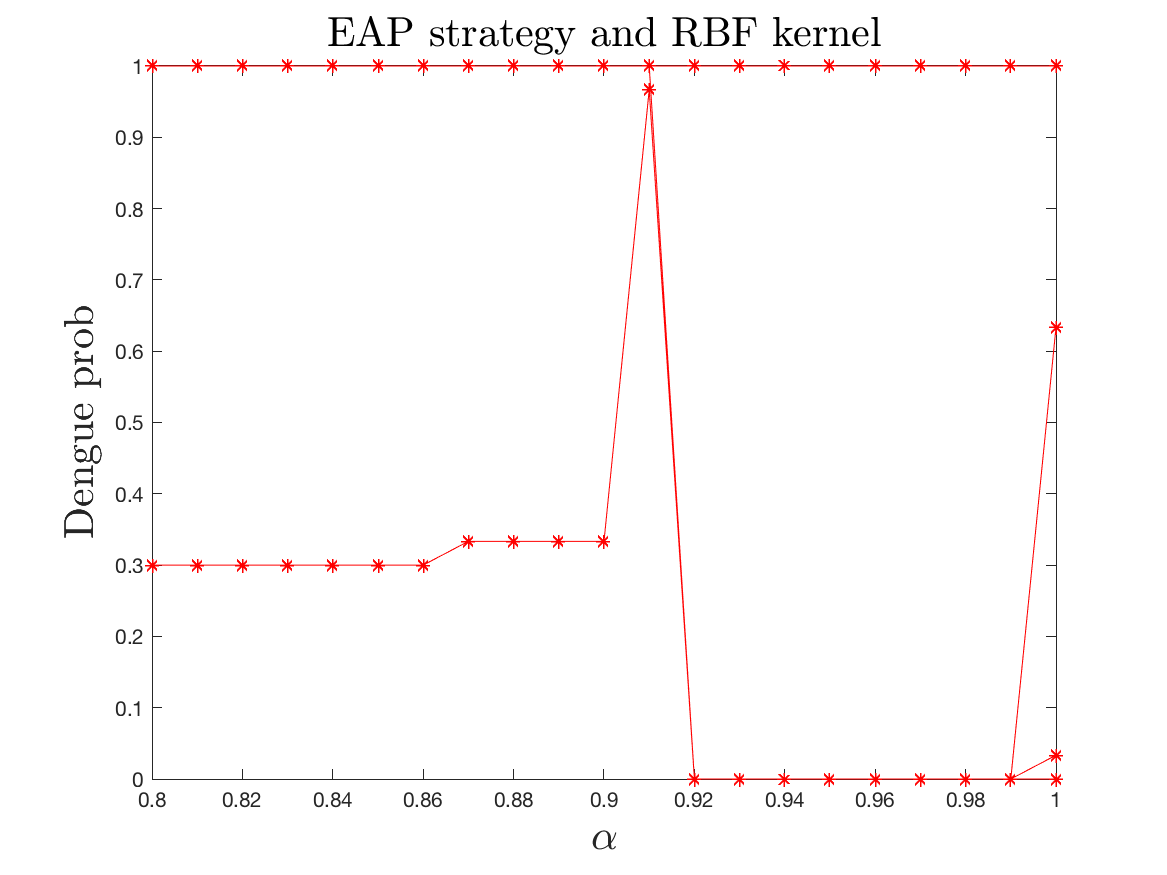

Supplement: S1 File — (ZIP) [file pone.0220106.s003.zip › data and codes_submit/holdout dataset analysis/prediction 2013-2017/capitals/results/Recife/FigureEAP_Recife_RBF.png]

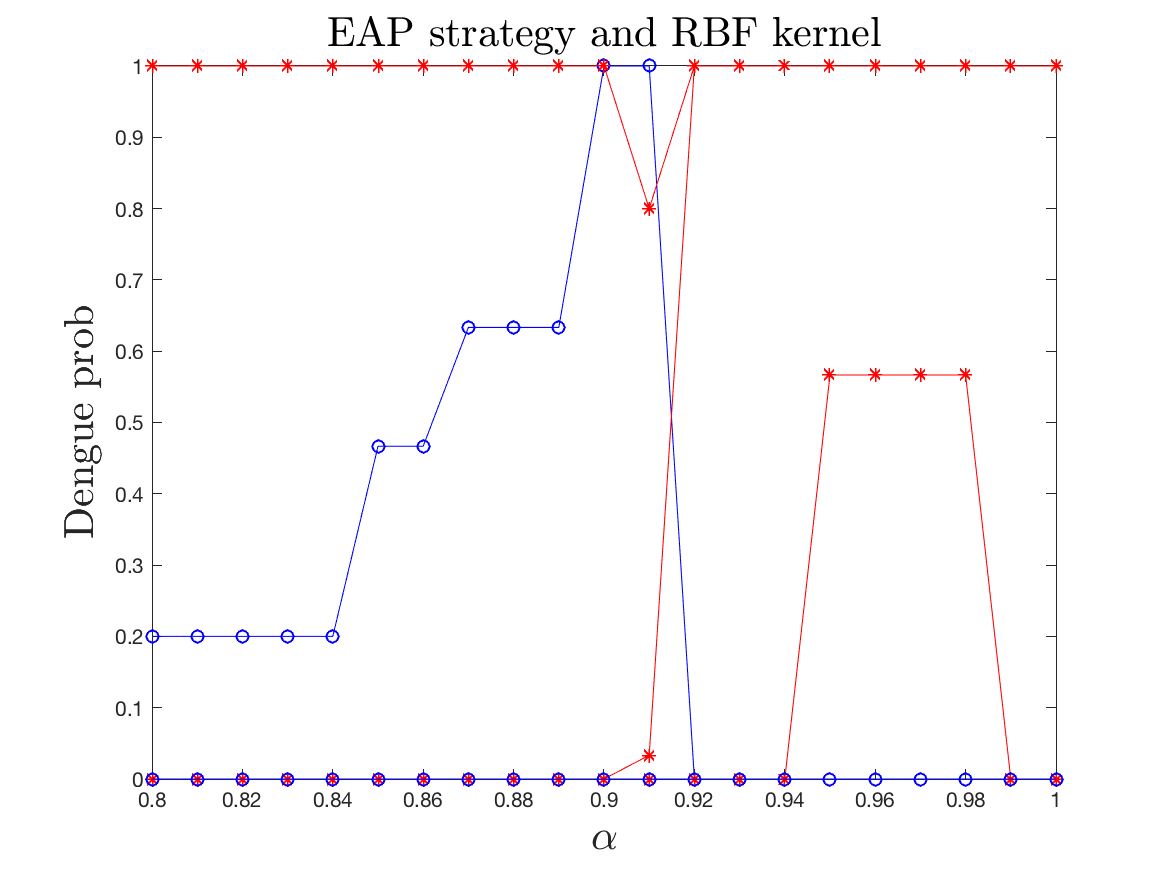

Supplement: S1 File — (ZIP) [file pone.0220106.s003.zip › data and codes_submit/holdout dataset analysis/prediction 2013-2017/capitals/results/Salvador/FigureEAP_Salvador_RBF.png]

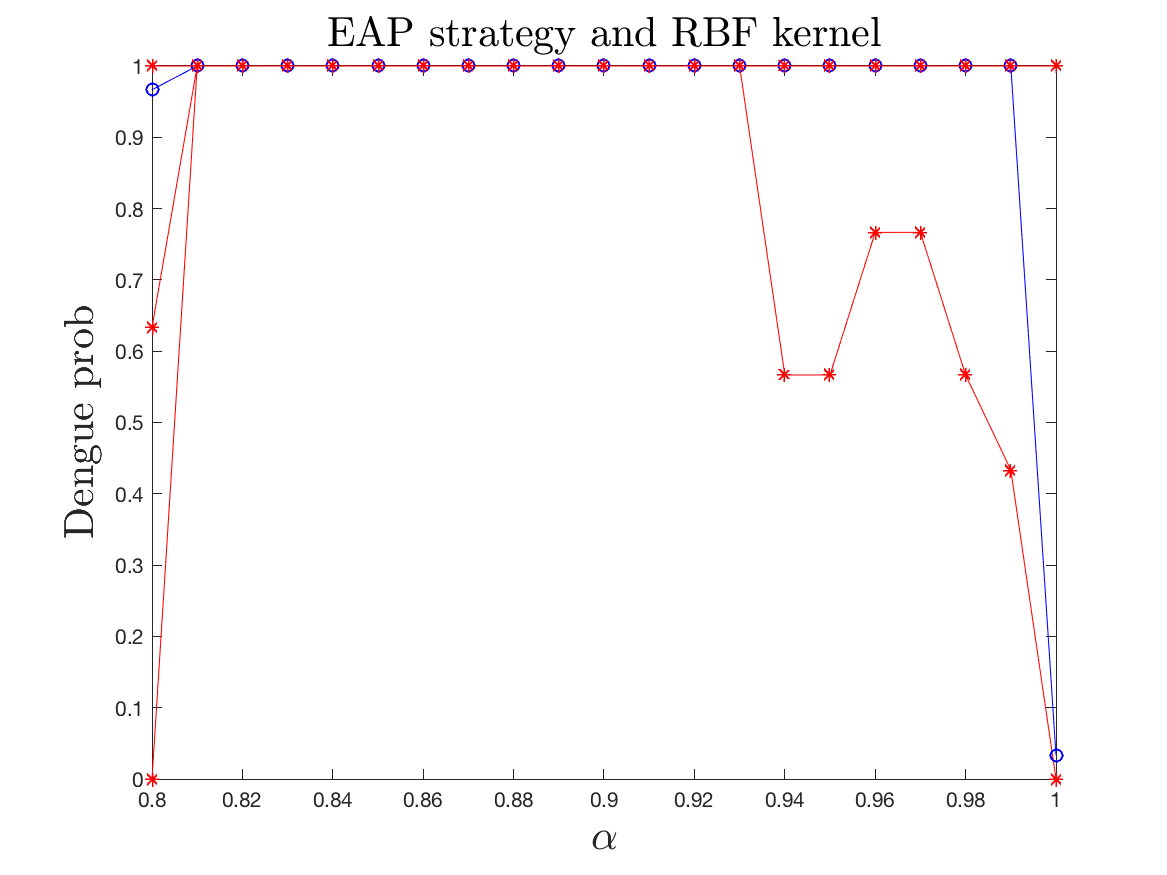

Supplement: S1 File — (ZIP) [file pone.0220106.s003.zip › data and codes_submit/holdout dataset analysis/prediction 2013-2017/capitals/results/Manaus/FigureEAP_Manaus_RBF.png]

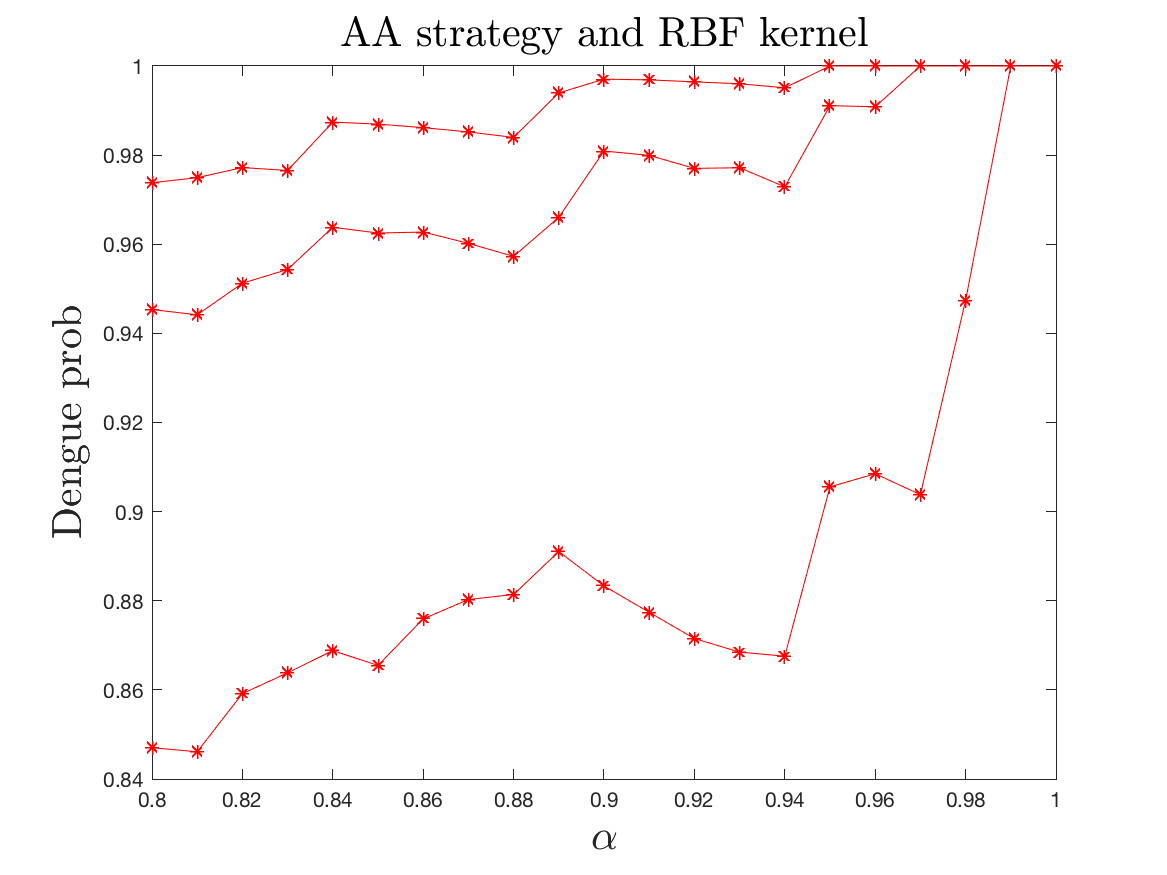

Supplement: S1 File — (ZIP) [file pone.0220106.s003.zip › data and codes_submit/holdout dataset analysis/prediction 2013-2017/capitals/results/Sao Luis/FigureAA_Sao_Luis_RBF.png]

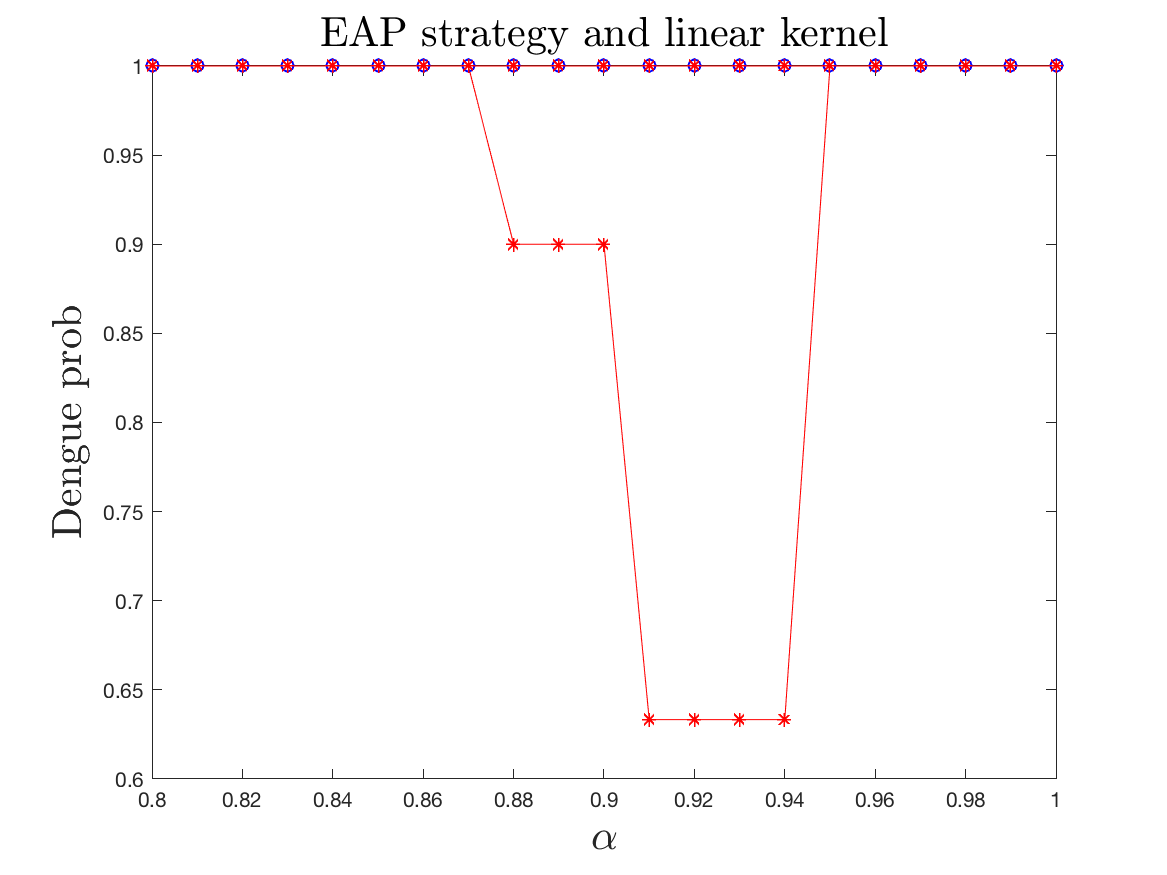

Supplement: S1 File — (ZIP) [file pone.0220106.s003.zip › data and codes_submit/holdout dataset analysis/prediction 2013-2017/capitals/results/Manaus/FigureEAP_Manaus_linear.png]

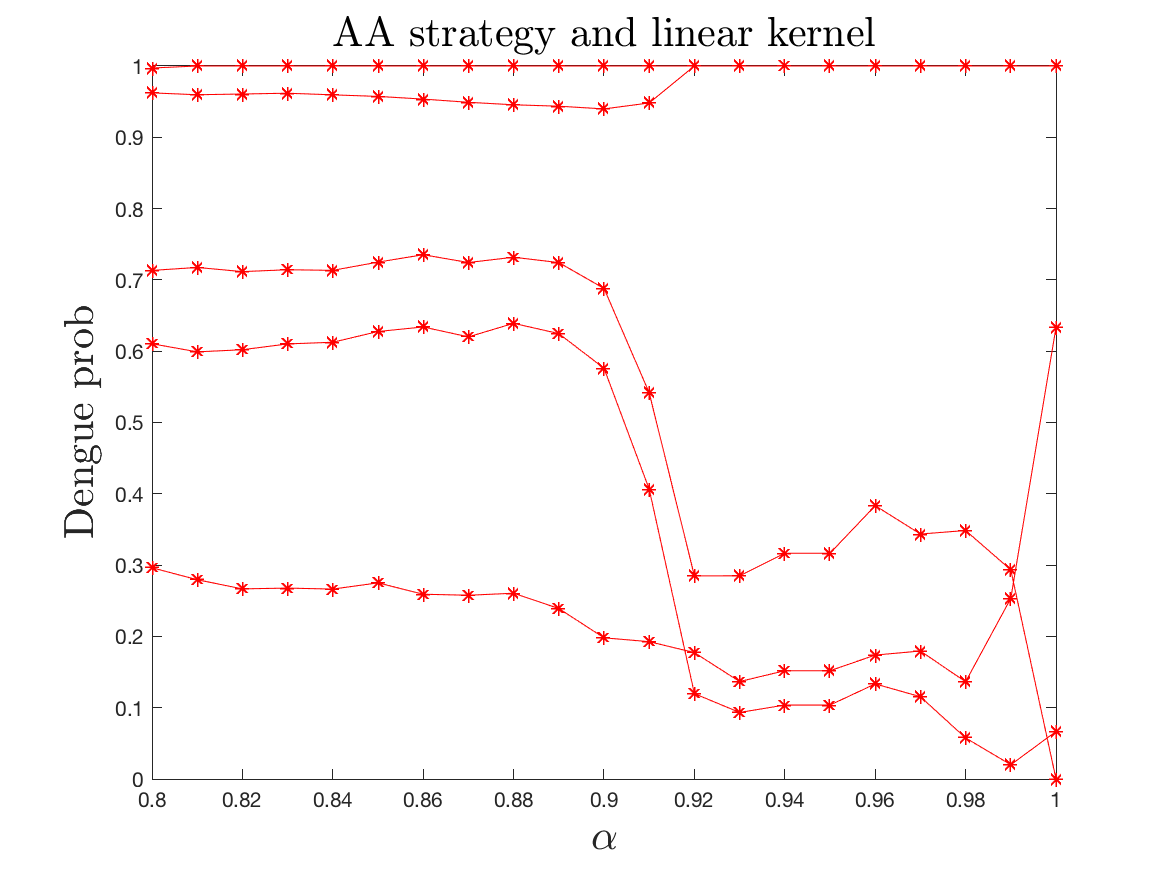

Supplement: S1 File — (ZIP) [file pone.0220106.s003.zip › data and codes_submit/holdout dataset analysis/prediction 2013-2017/capitals/results/Recife/FigureAA_Recife_linear.png]

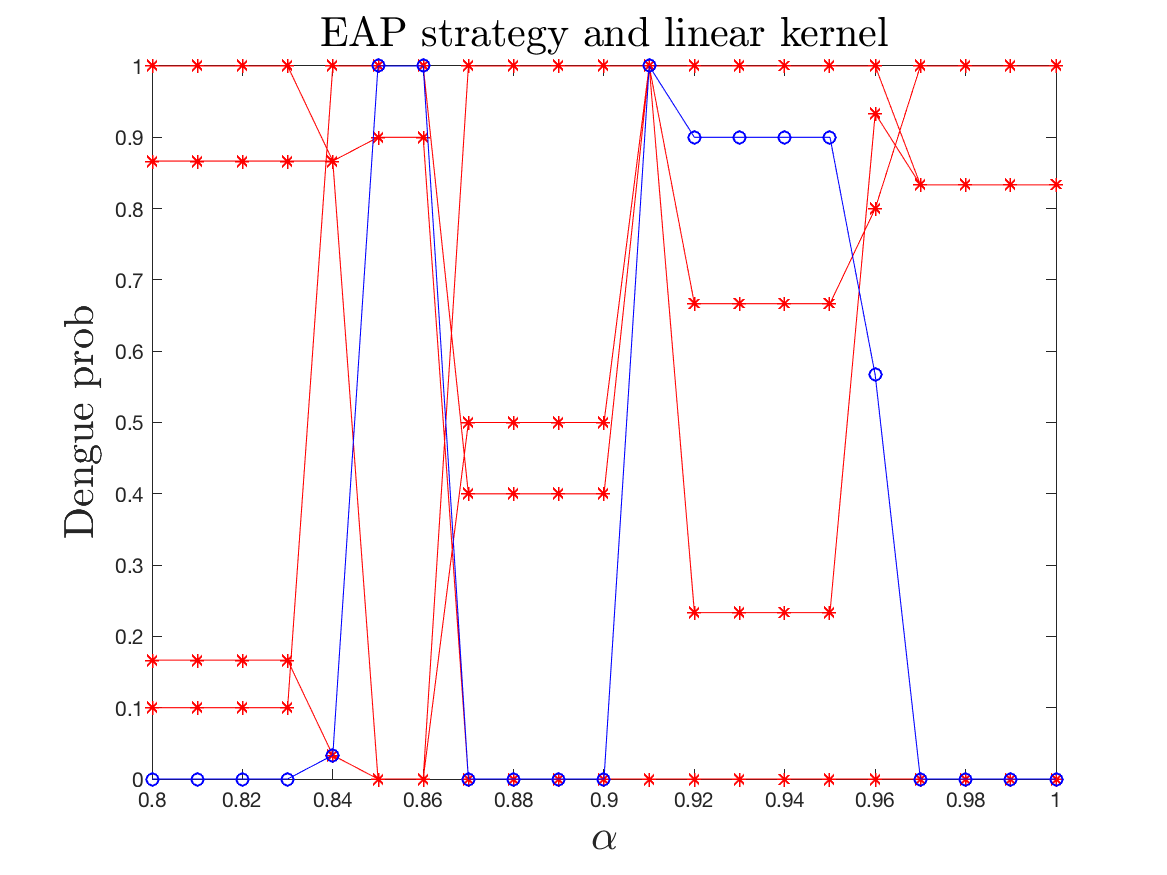

Supplement: S1 File — (ZIP) [file pone.0220106.s003.zip › data and codes_submit/holdout dataset analysis/prediction 2013-2017/capitals/results/Belo Horizonte/FigureEAP_Belo_Horizonte_linear.png]

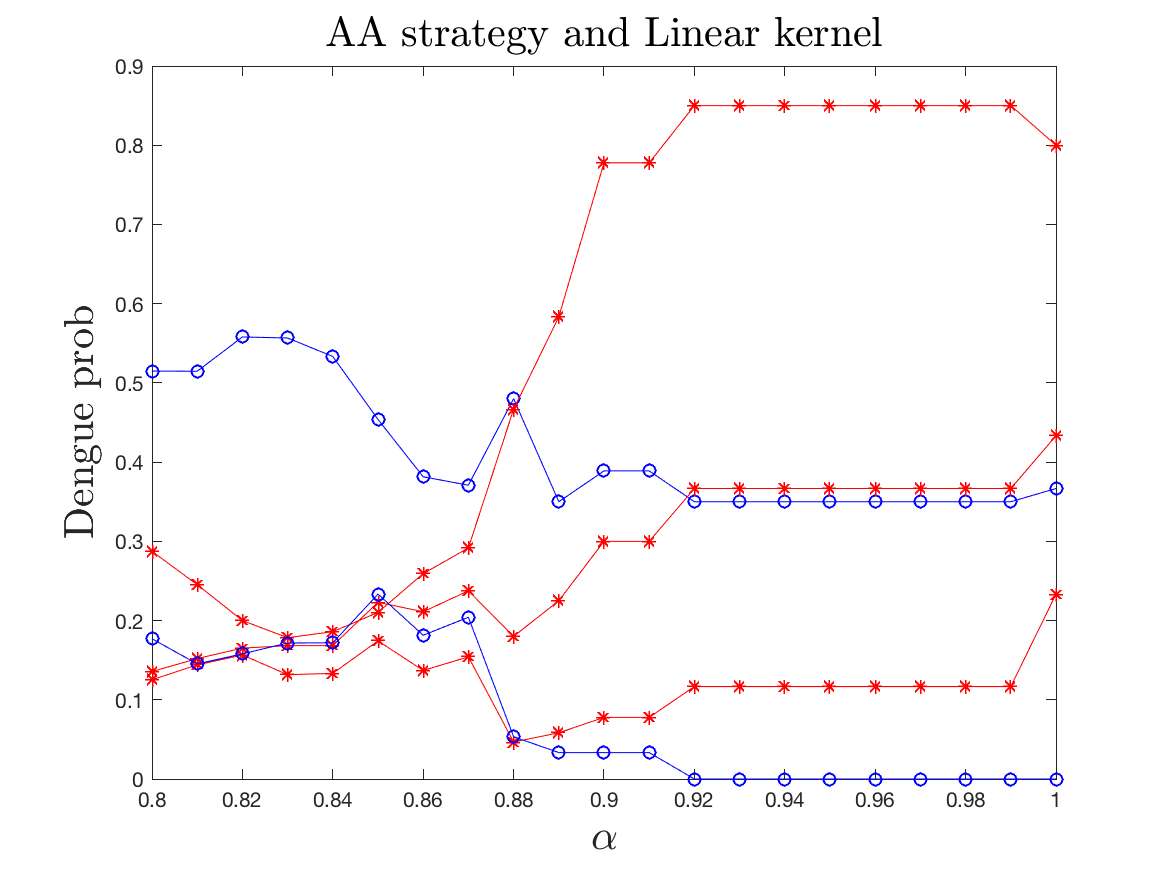

Supplement: S1 File — (ZIP) [file pone.0220106.s003.zip › data and codes_submit/holdout dataset analysis/prediction 2013-2017/capitals/results/Aracaju/FigureAA_Aracaju_Linear.png]

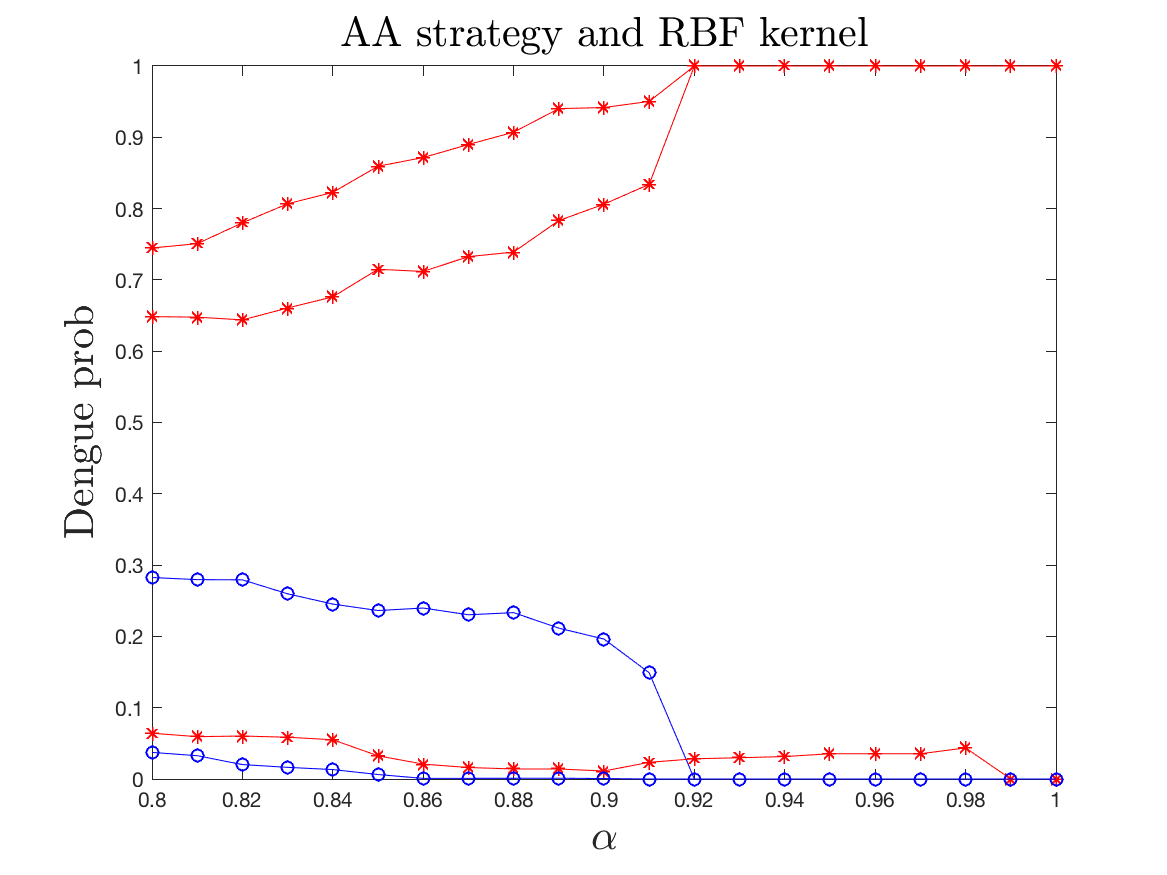

Supplement: S1 File — (ZIP) [file pone.0220106.s003.zip › data and codes_submit/holdout dataset analysis/prediction 2013-2017/capitals/results/Salvador/FigureAA_Salvador_RBF.png]

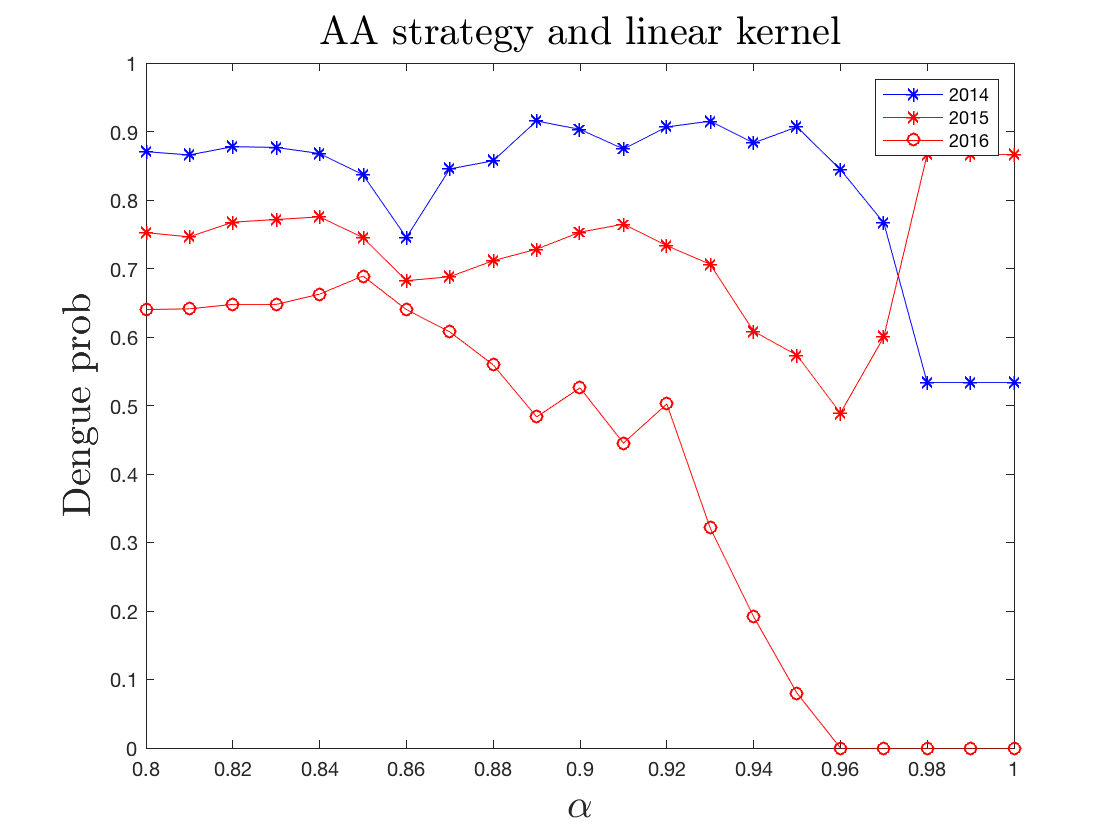

Supplement: S1 File — (ZIP) [file pone.0220106.s003.zip › data and codes_submit/holdout dataset analysis/prediction 2013-2017/rio de janeiro/Results/Results_AA_linear.png]

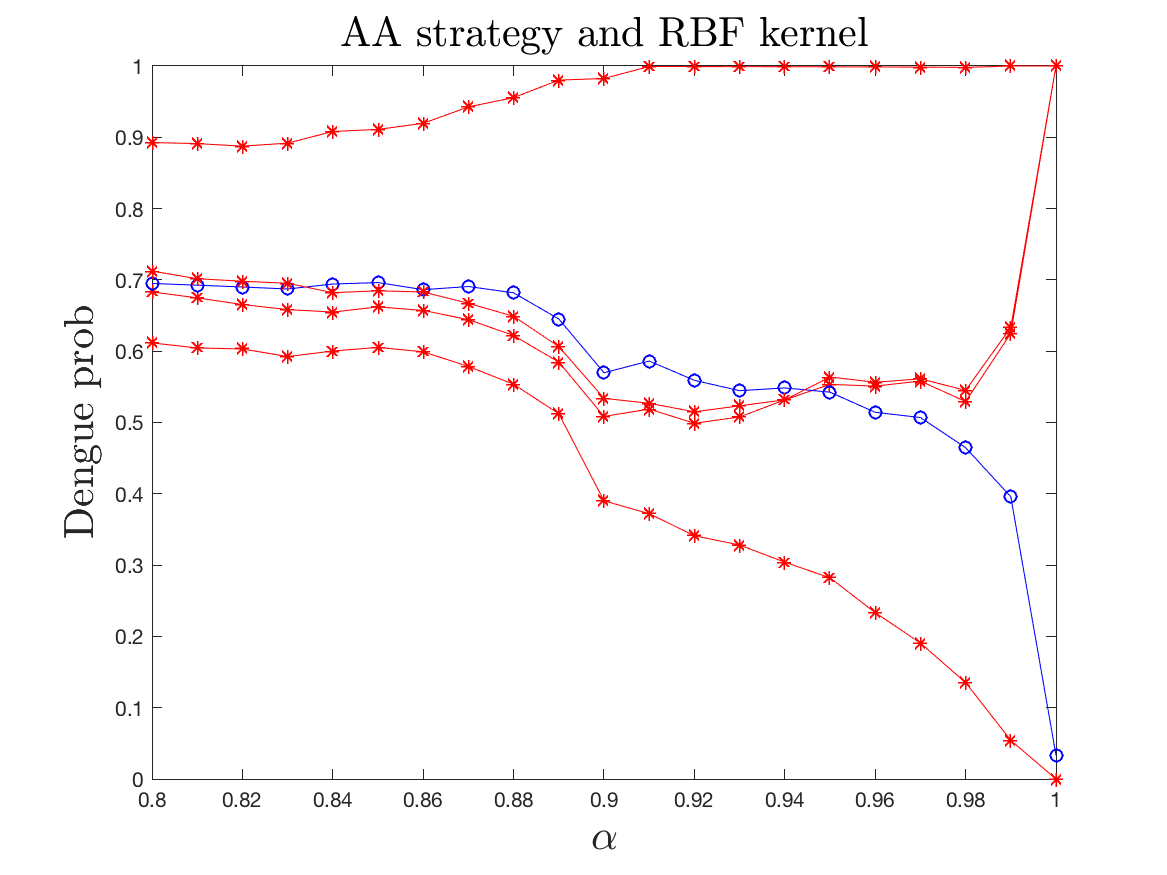

Supplement: S1 File — (ZIP) [file pone.0220106.s003.zip › data and codes_submit/holdout dataset analysis/prediction 2013-2017/capitals/results/Manaus/FigureAA_Manaus_RBF.png]

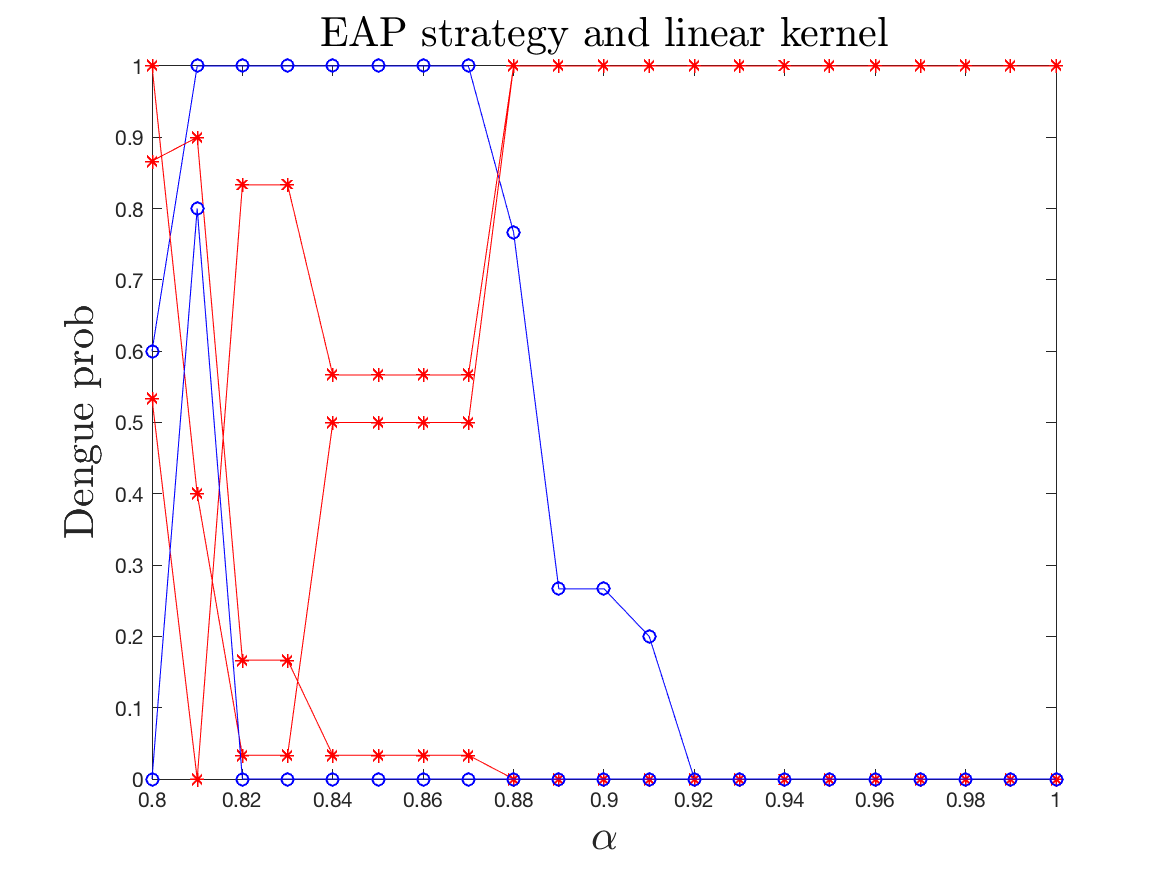

Supplement: S1 File — (ZIP) [file pone.0220106.s003.zip › data and codes_submit/holdout dataset analysis/prediction 2013-2017/capitals/results/Salvador/FigureEAP_Salvador_linear.png]

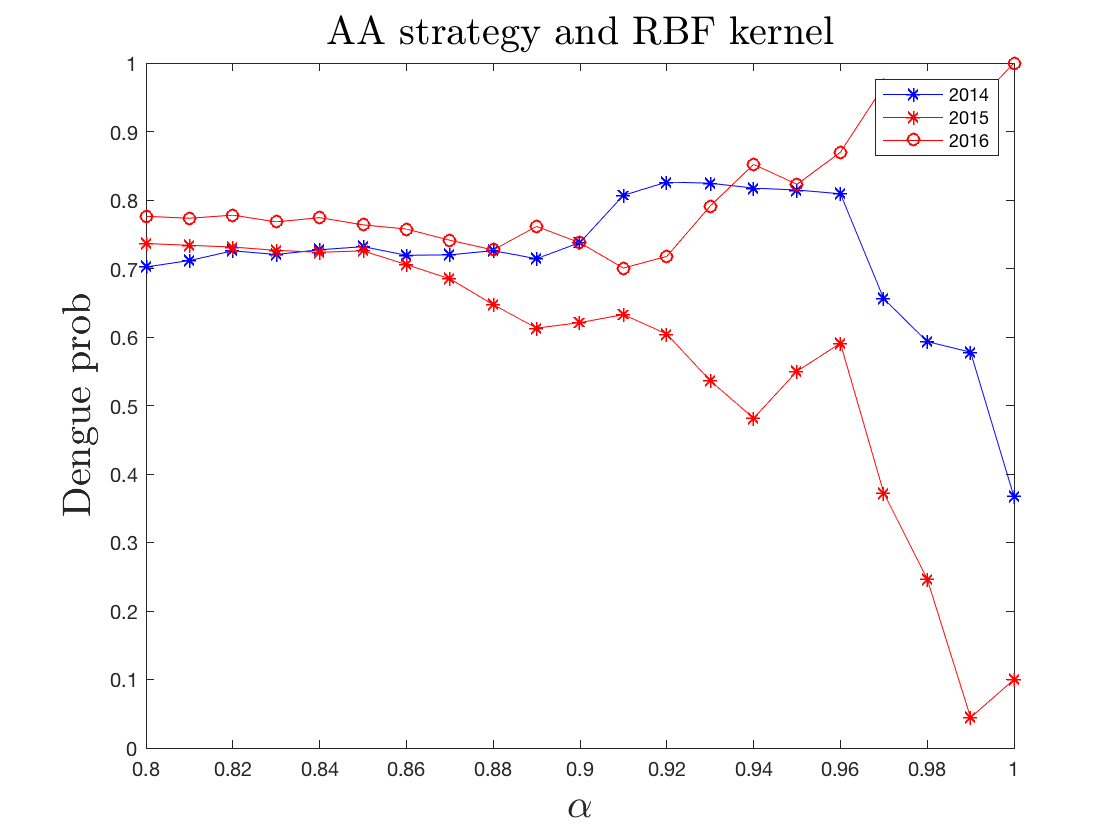

Supplement: S1 File — (ZIP) [file pone.0220106.s003.zip › data and codes_submit/holdout dataset analysis/prediction 2013-2017/rio de janeiro/Results/Results_AA_RBF.png]

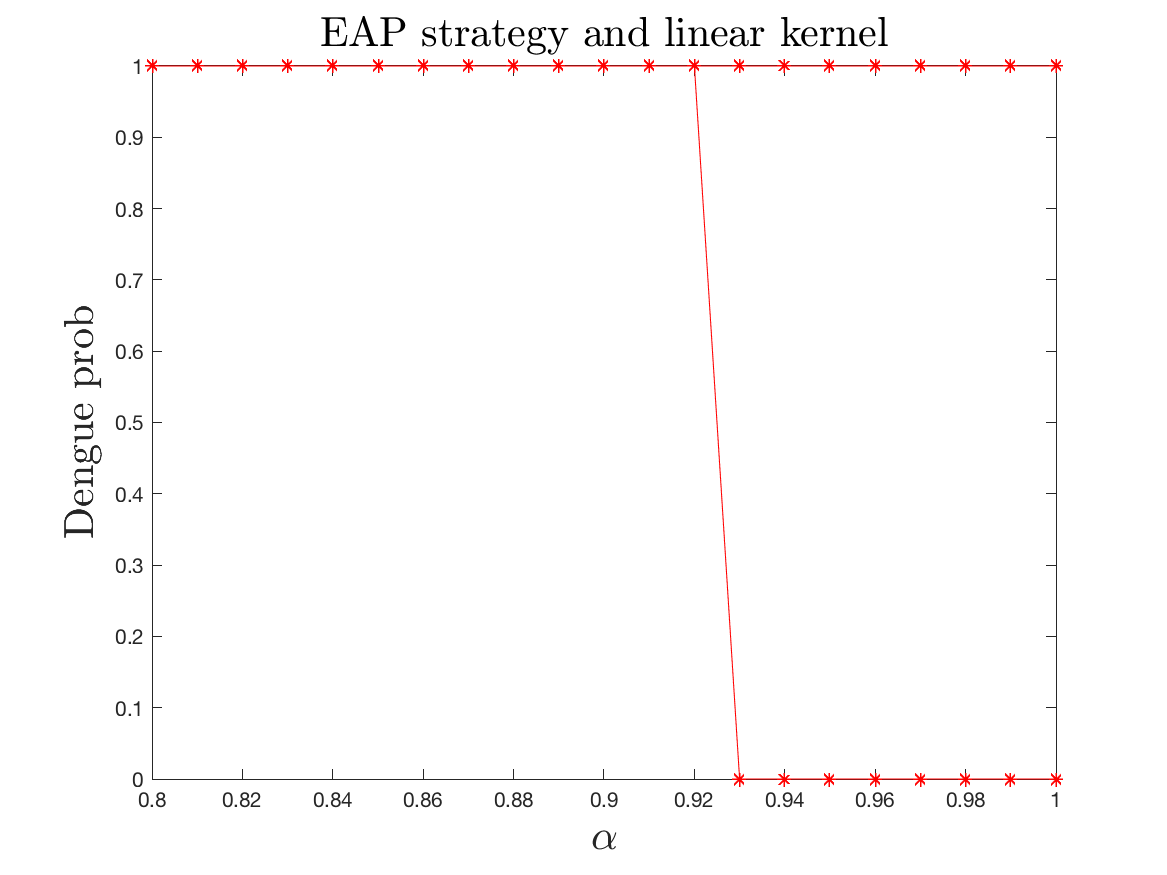

Supplement: S1 File — (ZIP) [file pone.0220106.s003.zip › data and codes_submit/holdout dataset analysis/prediction 2013-2017/capitals/results/Sao Luis/FigureEAP_Sao_Luis_linear.png]

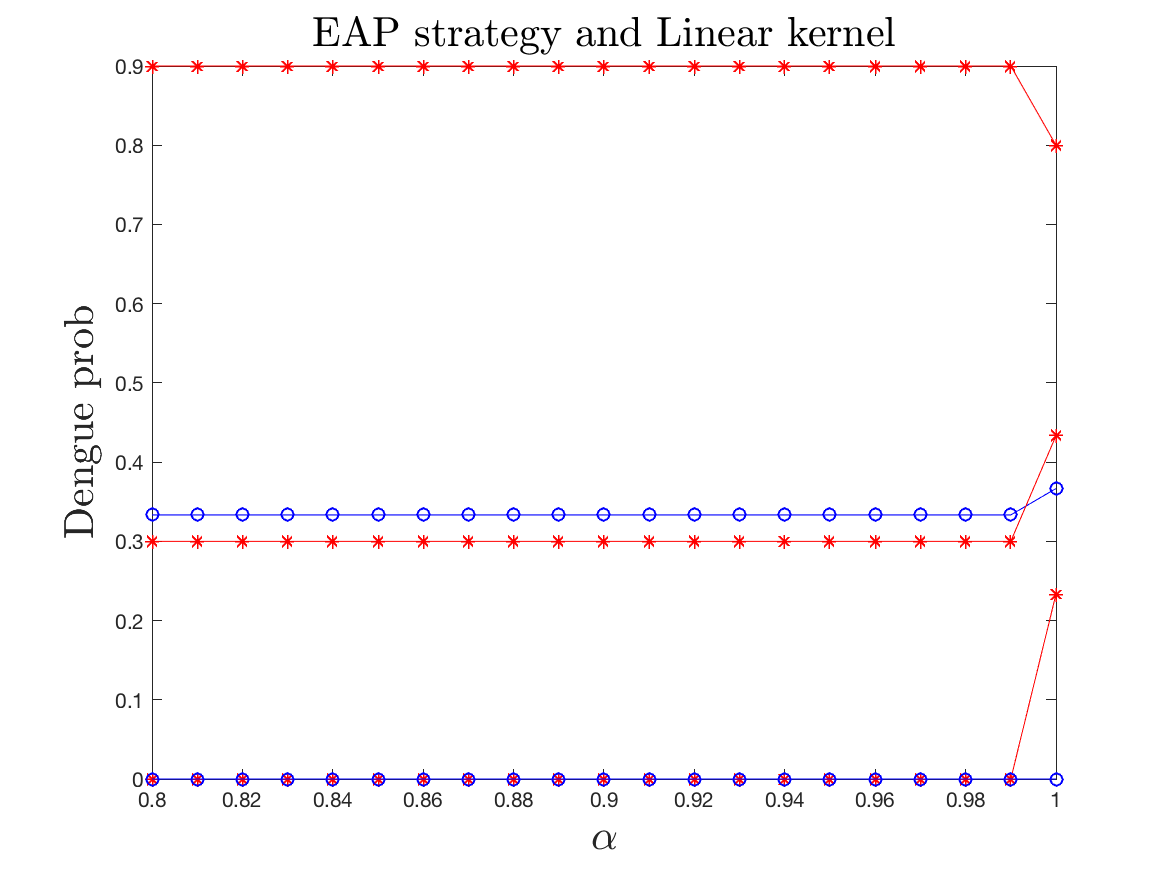

Supplement: S1 File — (ZIP) [file pone.0220106.s003.zip › data and codes_submit/holdout dataset analysis/prediction 2013-2017/capitals/results/Aracaju/FigureEAP_Aracaju_Linear.png]

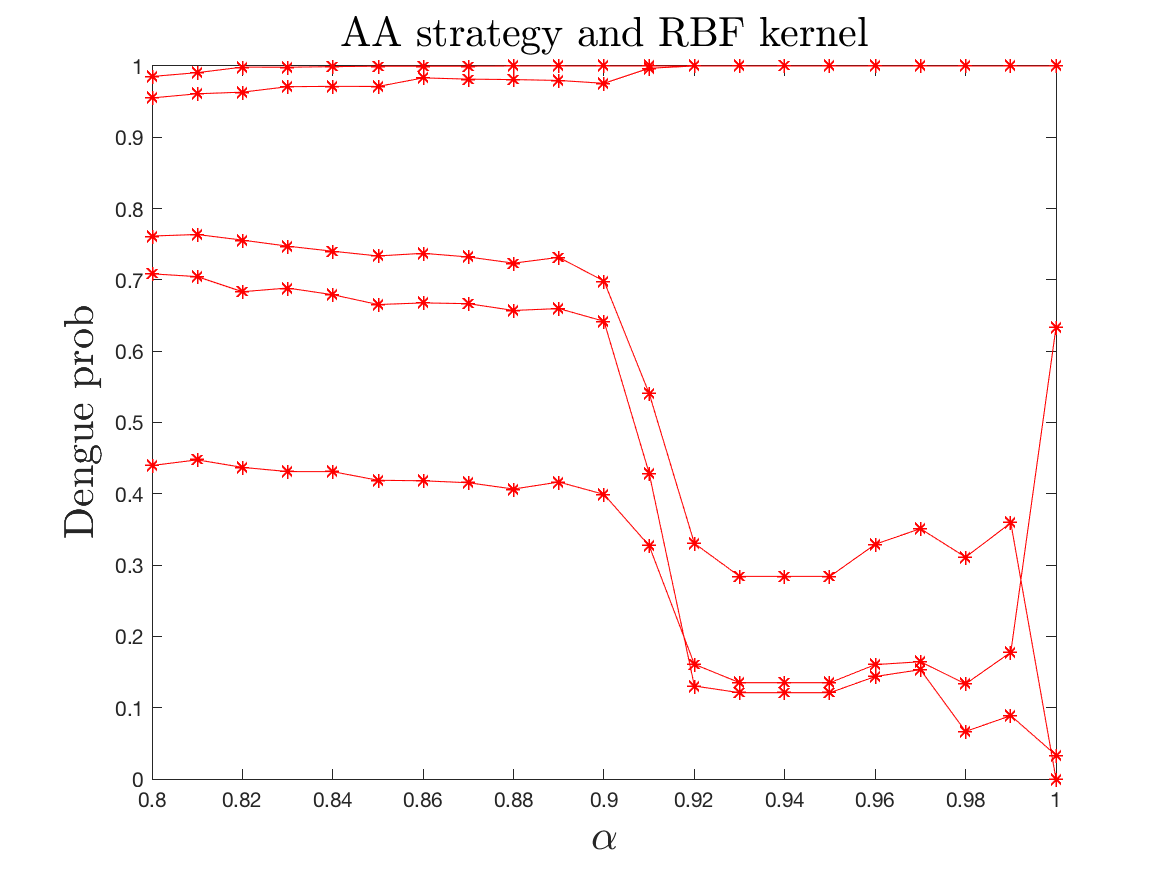

Supplement: S1 File — (ZIP) [file pone.0220106.s003.zip › data and codes_submit/holdout dataset analysis/prediction 2013-2017/capitals/results/Recife/FigureAA_Recife_RBF.png]

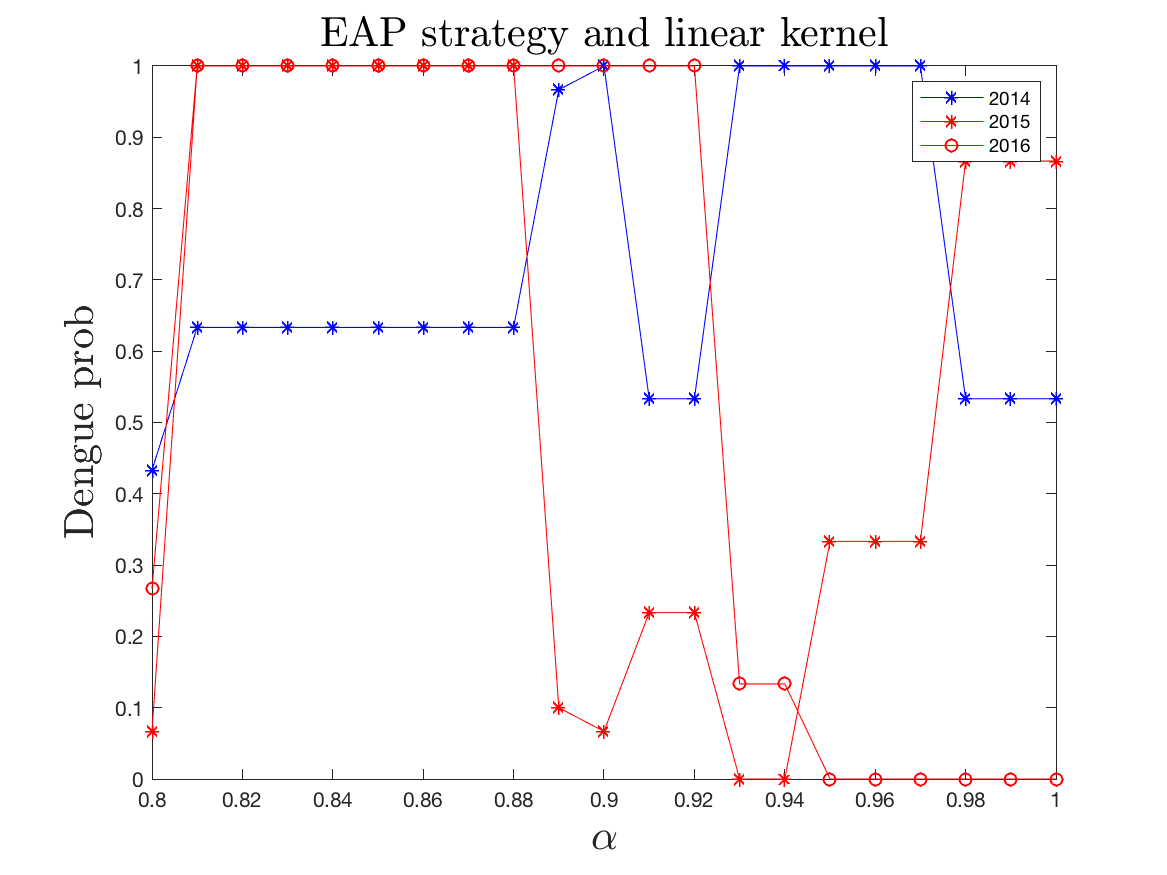

Supplement: S1 File — (ZIP) [file pone.0220106.s003.zip › data and codes_submit/holdout dataset analysis/prediction 2013-2017/Rio de Janeiro/FigureEAP_Rio_de_Janeiro_linear.png]

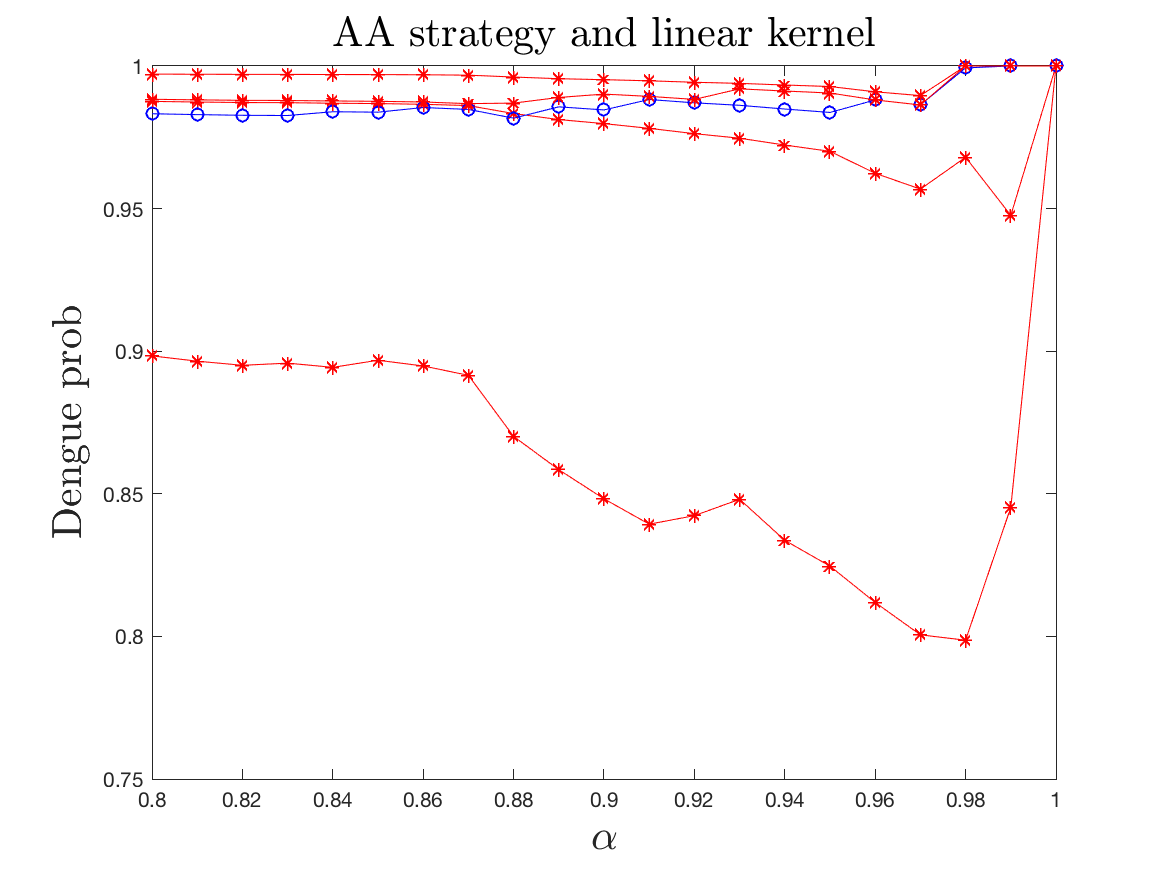

Supplement: S1 File — (ZIP) [file pone.0220106.s003.zip › data and codes_submit/holdout dataset analysis/prediction 2013-2017/capitals/results/Manaus/FigureAA_Manaus_linear.png]

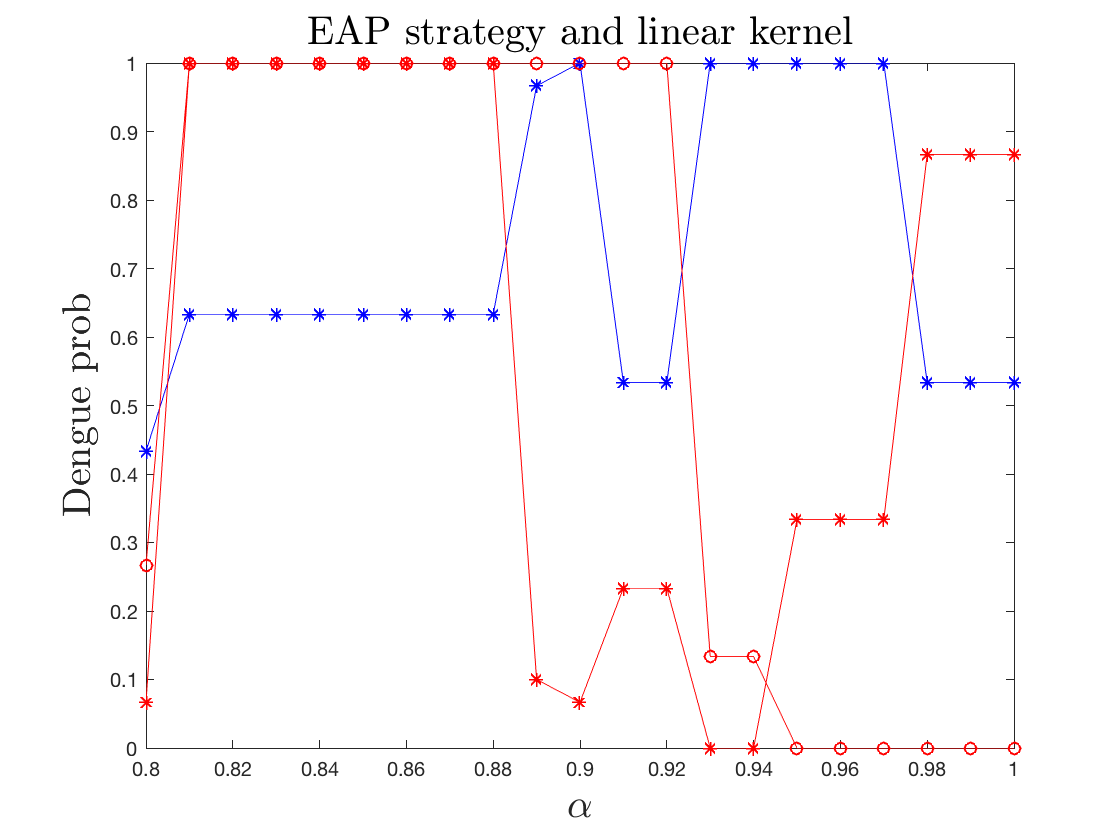

Supplement: S1 File — (ZIP) [file pone.0220106.s003.zip › data and codes_submit/holdout dataset analysis/prediction 2013-2017/rio de janeiro/Results/Results_EAP_linear.png]
