# Supplementary material for: Forecasting dengue fever in Brazil: An assessment of climate conditions
Source: PLoS One. 2019 Aug 8;14(8):e0220106. doi: 10.1371/journal.pone.0220106 (PMC6687106; doi:10.1371/journal.pone.0220106)
Supplement: S1 File — (ZIP) [file pone.0220106.s003.zip › data and codes_submit/training dataset analysis/data/Data IBGE/pop_municipios_2008.pdf]

ESTIMATIVAS DAS POPULAÇÕES RESIDENTES, EM 1º DE JULHO DE 2008, SEGUNDO OS MUNICÍPIOS				
U.F.		MUNICÍPIOS		POPULAÇÃO ESTIMADA
SIGLA	COD	COD	NOMES	
RO	11	00015	Alta Floresta D'Oeste	24.577
RO	11	00379	Alto Alegre dos Parecis	11.962
RO	11	00403	Alto Paraíso	17.169
RO	11	00346	Alvorada D'Oeste	17.009
RO	11	00023	Ariquemes	84.581
RO	11	00452	Buritis	33.879
RO	11	00031	Cabixi	6.777
RO	11	00601	Cacaulândia	5.707
RO	11	00049	Cacoal	78.263
RO	11	00700	Campo Novo de Rondônia	12.790
RO	11	00809	Candeias do Jamari	17.147
RO	11	00908	Castanheiras	3.737
RO	11	00056	Cerejeiras	16.784
RO	11	00924	Chupinguaia	7.633
RO	11	00064	Colorado do Oeste	18.216
RO	11	00072	Corumbiara	9.761
RO	11	00080	Costa Marques	13.973
RO	11	00940	Cujubim	14.122
RO	11	00098	Espigão D'Oeste	28.617
RO	11	01005	Governador Jorge Teixeira	11.793
RO	11	00106	Guajará-Mirim	40.541
RO	11	01104	Itapuã do Oeste	8.110
RO	11	00114	Jaru	53.955
RO	11	00122	Ji-Paraná	110.707
RO	11	00130	Machadinho D'Oeste	32.214
RO	11	01203	Ministro Andreazza	10.653
RO	11	01302	Mirante da Serra	12.446
RO	11	01401	Monte Negro	12.710
RO	11	00148	Nova Brasilândia D'Oeste	17.653
RO	11	00338	Nova Mamoré	21.650
RO	11	01435	Nova União	7.978
RO	11	00502	Novo Horizonte do Oeste	9.966
RO	11	00155	Ouro Preto do Oeste	37.142
RO	11	01450	Parecis	4.696
RO	11	00189	Pimenta Bueno	33.803
RO	11	01468	Pimenteiras do Oeste	2.427
RO	11	00205	Porto Velho	379.186
RO	11	00254	Presidente Médici	22.896
RO	11	01476	Primavera de Rondônia	3.819

U.F.		MUNICÍPIOS		POPULAÇÃO ESTIMADA
SIGLA	COD	COD	NOMES	
RO	11	00262	Rio Crespo	3.260
RO	11	00288	Rolim de Moura	50.249
RO	11	00296	Santa Luzia D'Oeste	9.566
RO	11	01484	São Felipe D'Oeste	6.477
RO	11	01492	São Francisco do Guaporé	16.091
RO	11	00320	São Miguel do Guaporé	23.283
RO	11	01500	Seringueiras	12.088
RO	11	01559	Teixeirópolis	5.070
RO	11	01609	Theobroma	10.254
RO	11	01708	Urupá	13.785
RO	11	01757	Vale do Anari	8.981
RO	11	01807	Vale do Paraíso	9.008
RO	11	00304	Vilhena	68.405
AC	12	00013	Acrelândia	11.987
AC	12	00054	Assis Brasil	5.559
AC	12	00104	Brasiléia	19.829
AC	12	00138	Bujari	6.745
AC	12	00179	Capixaba	8.897
AC	12	00203	Cruzeiro do Sul	76.392
AC	12	00252	Epitaciolândia	13.960
AC	12	00302	Feijó	32.210
AC	12	00328	Jordão	6.333
AC	12	00336	Mâncio Lima	14.387
AC	12	00344	Manoel Urbano	7.405
AC	12	00351	Marechal Thaumaturgo	13.728
AC	12	00385	Plácido de Castro	17.921
AC	12	00807	Porto Acre	14.309
AC	12	00393	Porto Walter	8.562
AC	12	00401	Rio Branco	301.398
AC	12	00427	Rodrigues Alves	13.020
AC	12	00435	Santa Rosa do Purus	4.165
AC	12	00500	Sena Madureira	35.544
AC	12	00450	Senador Guiomard	19.506
AC	12	00609	Tarauacá	33.368
AC	12	00708	Xapuri	14.848
AM	13	00029	Alvarães	13.445
AM	13	00060	Amaturá	8.694
AM	13	00086	Anamã	8.483
AM	13	00102	Anori	14.386
AM	13	00144	Apuí	18.170
AM	13	00201	Atalaia do Norte	14.292

U.F.		MUNICÍPIOS		POPULAÇÃO ESTIMADA
SIGLA	COD	COD	NOMES	
AM	13	00300	Autazes	31.107
AM	13	00409	Barcelos	25.318
AM	13	00508	Barreirinha	27.664
AM	13	00607	Benjamin Constant	30.476
AM	13	00631	Beruri	14.374
AM	13	00680	Boa Vista do Ramos	13.677
AM	13	00706	Boca do Acre	30.858
AM	13	00805	Borba	32.160
AM	13	00839	Caapiranga	10.958
AM	13	00904	Canutama	11.844
AM	13	01001	Carauari	25.950
AM	13	01100	Careiro	32.190
AM	13	01159	Careiro da Várzea	24.030
AM	13	01209	Coari	67.055
AM	13	01308	Codajás	16.418
AM	13	01407	Eirunepé	30.473
AM	13	01506	Envira	17.148
AM	13	01605	Fonte Boa	19.783
AM	13	01654	Guajará	14.684
AM	13	01704	Humaitá	40.027
AM	13	01803	Ipixuna	17.850
AM	13	01852	Iranduba	33.834
AM	13	01902	Itacoatiara	87.896
AM	13	01951	Itamarati	8.300
AM	13	02009	Itapiranga	9.515
AM	13	02108	Japurá	5.025
AM	13	02207	Juruá	9.032
AM	13	02306	Jutaí	17.337
AM	13	02405	Lábrea	38.451
AM	13	02504	Manacapuru	85.279
AM	13	02553	Manaquiri	20.095
AM	13	02603	Manaus	1.709.010
AM	13	02702	Manicoré	45.996
AM	13	02801	Maraã	18.051
AM	13	02900	Maués	48.808
AM	13	03007	Nhamundá	18.198
AM	13	03106	Nova Olinda do Norte	30.357
AM	13	03205	Novo Airão	15.343
AM	13	03304	Novo Aripuanã	18.796
AM	13	03403	Parintins	105.742
AM	13	03502	Pauini	18.938

U.F.		MUNICÍPIOS		POPULAÇÃO ESTIMADA
SIGLA	COD	COD	NOMES	
AM	13	03536	Presidente Figueiredo	25.474
AM	13	03569	Rio Preto da Eva	26.004
AM	13	03601	Santa Isabel do Rio Negro	17.780
AM	13	03700	Santo Antônio do Içá	30.176
AM	13	03809	São Gabriel da Cachoeira	40.806
AM	13	03908	São Paulo de Olivença	32.067
AM	13	03957	São Sebastião do Uatumã	9.079
AM	13	04005	Silves	8.479
AM	13	04062	Tabatinga	47.051
AM	13	04104	Tapauá	19.966
AM	13	04203	Tefé	64.703
AM	13	04237	Tonantins	19.858
AM	13	04260	Uarini	10.130
AM	13	04302	Urucará	16.035
AM	13	04401	Urucurituba	17.971
RR	14	00050	Alto Alegre	14.562
RR	14	00027	Amajari	7.980
RR	14	00100	Boa Vista	260.930
RR	14	00159	Bonfim	10.604
RR	14	00175	Cantá	11.638
RR	14	00209	Caracaraí	18.789
RR	14	00233	Caroebe	7.400
RR	14	00282	Iracema	6.118
RR	14	00308	Mucajaí	13.017
RR	14	00407	Normandia	7.403
RR	14	00456	Pacaraima	9.019
RR	14	00472	Rorainópolis	25.714
RR	14	00506	São João da Baliza	5.945
RR	14	00605	São Luiz	5.922
RR	14	00704	Uiramutã	7.742
PA	15	00107	Abaetetuba	138.005
PA	15	00131	Abel Figueiredo	6.879
PA	15	00206	Acará	48.878
PA	15	00305	Afuá	32.368
PA	15	00347	Água Azul do Norte	30.389
PA	15	00404	Alenquer	55.688
PA	15	00503	Almeirim	31.475
PA	15	00602	Altamira	96.842
PA	15	00701	Anajás	26.563
PA	15	00800	Ananindeua	495.480
PA	15	00859	Anapu	19.410

U.F.		MUNICÍPIOS		POPULAÇÃO ESTIMADA
SIGLA	COD	COD	NOMES	
PA	15	00909	Augusto Corrêa	38.760
PA	15	00958	Aurora do Pará	22.092
PA	15	01006	Aveiro	19.839
PA	15	01105	Bagre	19.780
PA	15	01204	Baião	27.652
PA	15	01253	Bannach	3.935
PA	15	01303	Barcarena	89.909
PA	15	01402	Belém	1.424.124
PA	15	01451	Belterra	12.861
PA	15	01501	Benevides	45.616
PA	15	01576	Bom Jesus do Tocantins	13.559
PA	15	01600	Bonito	11.817
PA	15	01709	Bragança	105.908
PA	15	01725	Brasil Novo	19.531
PA	15	01758	Brejo Grande do Araguaia	7.673
PA	15	01782	Breu Branco	50.400
PA	15	01808	Breves	99.223
PA	15	01907	Bujaru	23.429
PA	15	02004	Cachoeira do Arari	19.996
PA	15	01956	Cachoeira do Piriá	18.481
PA	15	02103	Cametá	115.377
PA	15	02152	Canaã dos Carajás	26.135
PA	15	02202	Capanema	63.799
PA	15	02301	Capitão Poço	52.557
PA	15	02400	Castanhal	159.110
PA	15	02509	Chaves	20.228
PA	15	02608	Colares	11.367
PA	15	02707	Conceição do Araguaia	46.916
PA	15	02756	Concórdia do Pará	22.148
PA	15	02764	Cumaru do Norte	11.348
PA	15	02772	Curionópolis	18.102
PA	15	02806	Curralinho	26.864
PA	15	02855	Curuá	12.644
PA	15	02905	Curuçá	35.790
PA	15	02939	Dom Eliseu	39.161
PA	15	02954	Eldorado dos Carajás	29.308
PA	15	03002	Faro	18.710
PA	15	03044	Floresta do Araguaia	15.516
PA	15	03077	Garrafão do Norte	25.436
PA	15	03093	Goianésia do Pará	28.583
PA	15	03101	Gurupá	25.306

U.F.		MUNICÍPIOS		POPULAÇÃO ESTIMADA
SIGLA	COD	COD	NOMES	
PA	15	03200	Igarapé-Açu	35.005
PA	15	03309	Igarapé-Miri	56.639
PA	15	03408	Inhangapi	10.134
PA	15	03457	Ipixuna do Pará	42.636
PA	15	03507	Irituia	30.573
PA	15	03606	Itaituba	124.865
PA	15	03705	Itupiranga	42.332
PA	15	03754	Jacareacanga	39.892
PA	15	03804	Jacundá	54.513
PA	15	03903	Juruti	35.155
PA	15	04000	Limoeiro do Ajuru	24.483
PA	15	04059	Mãe do Rio	28.762
PA	15	04109	Magalhães Barata	7.883
PA	15	04208	Marabá	199.946
PA	15	04307	Maracanã	29.269
PA	15	04406	Marapanim	27.727
PA	15	04422	Marituba	98.746
PA	15	04455	Medicilândia	23.487
PA	15	04505	Melgaço	17.989
PA	15	04604	Mocajuba	24.328
PA	15	04703	Moju	67.195
PA	15	04802	Monte Alegre	63.543
PA	15	04901	Muaná	30.117
PA	15	04950	Nova Esperança do Piriá	23.599
PA	15	04976	Nova Ipixuna	14.808
PA	15	05007	Nova Timboteua	12.568
PA	15	05031	Novo Progresso	21.843
PA	15	05064	Novo Repartimento	54.506
PA	15	05106	Óbidos	48.287
PA	15	05205	Oeiras do Pará	26.487
PA	15	05304	Oriximiná	57.765
PA	15	05403	Ourém	15.719
PA	15	05437	Ourilândia do Norte	21.171
PA	15	05486	Pacajá	40.768
PA	15	05494	Palestina do Pará	7.329
PA	15	05502	Paragominas	95.479
PA	15	05536	Parauapebas	145.326
PA	15	05551	Pau D'Arco	6.583
PA	15	05601	Peixe-Boi	7.908
PA	15	05635	Piçarra	13.106
PA	15	05650	Placas	19.030

U.F.		MUNICÍPIOS		POPULAÇÃO ESTIMADA
SIGLA	COD	COD	NOMES	
PA	15	05700	Ponta de Pedras	25.743
PA	15	05809	Portel	47.967
PA	15	05908	Porto de Moz	27.689
PA	15	06005	Prainha	26.866
PA	15	06104	Primavera	10.883
PA	15	06112	Quatipuru	13.232
PA	15	06138	Redenção	66.762
PA	15	06161	Rio Maria	17.457
PA	15	06187	Rondon do Pará	47.074
PA	15	06195	Rurópolis	35.033
PA	15	06203	Salinópolis	38.681
PA	15	06302	Salvaterra	17.858
PA	15	06351	Santa Bárbara do Pará	14.439
PA	15	06401	Santa Cruz do Arari	6.190
PA	15	06500	Santa Isabel do Pará	54.464
PA	15	06559	Santa Luzia do Pará	18.523
PA	15	06583	Santa Maria das Barreiras	17.156
PA	15	06609	Santa Maria do Pará	23.001
PA	15	06708	Santana do Araguaia	52.856
PA	15	06807	Santarém	275.571
PA	15	06906	Santarém Novo	6.267
PA	15	07003	Santo Antônio do Tauá	26.222
PA	15	07102	São Caetano de Odivelas	16.757
PA	15	07151	São Domingos do Araguaia	21.889
PA	15	07201	São Domingos do Capim	27.897
PA	15	07300	São Félix do Xingu	64.223
PA	15	07409	São Francisco do Pará	11.986
PA	15	07458	São Geraldo do Araguaia	25.291
PA	15	07466	São João da Ponta	4.949
PA	15	07474	São João de Pirabas	19.691
PA	15	07508	São João do Araguaia	11.963
PA	15	07607	São Miguel do Guamá	44.532
PA	15	07706	São Sebastião da Boa Vista	21.499
PA	15	07755	Sapucaia	5.444
PA	15	07805	Senador José Porfírio	14.566
PA	15	07904	Soure	22.244
PA	15	07953	Tailândia	69.581
PA	15	07961	Terra Alta	10.372
PA	15	07979	Terra Santa	15.885
PA	15	08001	Tomé-Açu	48.522
PA	15	08035	Tracuateua	27.374

U.F.		MUNICÍPIOS		POPULAÇÃO ESTIMADA
SIGLA	COD	COD	NOMES	
PA	15	08050	Trairão	16.860
PA	15	08084	Tucumã	27.491
PA	15	08100	Tucuruí	94.015
PA	15	08126	Ulianópolis	34.485
PA	15	08159	Uruará	34.875
PA	15	08209	Vigia	45.680
PA	15	08308	Viseu	55.144
PA	15	08357	Vitória do Xingu	9.809
PA	15	08407	Xinguara	40.067
AP	16	00105	Amapá	7.745
AP	16	00204	Calçoene	9.060
AP	16	00212	Cutias	4.528
AP	16	00238	Ferreira Gomes	5.301
AP	16	00253	Itaubal	3.583
AP	16	00279	Laranjal do Jari	39.285
AP	16	00303	Macapá	359.020
AP	16	00402	Mazagão	14.418
AP	16	00501	Oiapoque	20.226
AP	16	00154	Pedra Branca do Amapari	7.800
AP	16	00535	Porto Grande	14.598
AP	16	00550	Pracuúba	3.533
AP	16	00600	Santana	95.733
AP	16	00055	Serra do Navio	3.921
AP	16	00709	Tartarugalzinho	13.160
AP	16	00808	Vitória do Jari	11.253
TO	17	00251	Abreulândia	2.310
TO	17	00301	Aguiarnópolis	4.120
TO	17	00350	Aliança do Tocantins	5.860
TO	17	00400	Almas	7.692
TO	17	00707	Alvorada	8.200
TO	17	01002	Ananás	9.615
TO	17	01051	Angico	3.264
TO	17	01101	Aparecida do Rio Negro	4.140
TO	17	01309	Aragominas	5.618
TO	17	01903	Araguacema	5.579
TO	17	02000	Araguaçu	9.244
TO	17	02109	Araguaína	119.128
TO	17	02158	Araguanã	5.154
TO	17	02208	Araguatins	26.722
TO	17	02307	Arapoema	7.034
TO	17	02406	Arraias	10.928

U.F.		MUNICÍPIOS		POPULAÇÃO ESTIMADA
SIGLA	COD	COD	NOMES	
TO	17	02554	Augustinópolis	15.248
TO	17	02703	Aurora do Tocantins	3.486
TO	17	02901	Axixá do Tocantins	9.175
TO	17	03008	Babaçulândia	10.672
TO	17	03057	Bandeirantes do Tocantins	2.791
TO	17	03073	Barra do Ouro	3.684
TO	17	03107	Barrolândia	5.304
TO	17	03206	Bernardo Sayão	4.648
TO	17	03305	Bom Jesus do Tocantins	2.793
TO	17	03602	Brasilândia do Tocantins	2.183
TO	17	03701	Brejinho de Nazaré	5.452
TO	17	03800	Buriti do Tocantins	8.404
TO	17	03826	Cachoeirinha	2.235
TO	17	03842	Campos Lindos	7.858
TO	17	03867	Cariri do Tocantins	3.672
TO	17	03883	Carmolândia	2.383
TO	17	03891	Carrasco Bonito	3.411
TO	17	03909	Caseara	4.813
TO	17	04105	Centenário	2.457
TO	17	05102	Chapada da Natividade	3.791
TO	17	04600	Chapada de Areia	1.274
TO	17	05508	Colinas do Tocantins	30.190
TO	17	16703	Colméia	9.005
TO	17	05557	Combinado	5.023
TO	17	05607	Conceição do Tocantins	4.529
TO	17	06001	Couto de Magalhães	5.035
TO	17	06100	Cristalândia	6.698
TO	17	06258	Crixás do Tocantins	1.299
TO	17	06506	Darcinópolis	5.288
TO	17	07009	Dianópolis	19.158
TO	17	07108	Divinópolis do Tocantins	6.549
TO	17	07207	Dois Irmãos do Tocantins	7.261
TO	17	07306	Dueré	4.617
TO	17	07405	Esperantina	8.375
TO	17	07553	Fátima	4.101
TO	17	07652	Figueirópolis	4.950
TO	17	07702	Filadélfia	8.007
TO	17	08205	Formoso do Araguaia	18.743
TO	17	08254	Fortaleza do Tabocão	2.160
TO	17	08304	Goianorte	5.376
TO	17	09005	Goiatins	11.982

U.F.		MUNICÍPIOS		POPULAÇÃO ESTIMADA
SIGLA	COD	COD	NOMES	
TO	17	09302	Guaraí	22.314
TO	17	09500	Gurupi	73.548
TO	17	09807	Ipueiras	1.754
TO	17	10508	Itacajá	6.565
TO	17	10706	Itaguatins	6.246
TO	17	10904	Itapiratins	3.521
TO	17	11100	Itaporã do Tocantins	3.080
TO	17	11506	Jaú do Tocantins	3.906
TO	17	11803	Juarina	2.200
TO	17	11902	Lagoa da Confusão	8.481
TO	17	11951	Lagoa do Tocantins	3.278
TO	17	12009	Lajeado	2.219
TO	17	12157	Lavandeira	1.640
TO	17	12405	Lizarda	3.737
TO	17	12454	Luzinópolis	2.874
TO	17	12504	Marianópolis do Tocantins	4.616
TO	17	12702	Mateiros	1.788
TO	17	12801	Maurilândia do Tocantins	3.281
TO	17	13205	Miracema do Tocantins	20.194
TO	17	13304	Miranorte	12.200
TO	17	13601	Monte do Carmo	6.586
TO	17	13700	Monte Santo do Tocantins	1.911
TO	17	13957	Muricilândia	2.935
TO	17	14203	Natividade	9.355
TO	17	14302	Nazaré	4.652
TO	17	14880	Nova Olinda	10.835
TO	17	15002	Nova Rosalândia	3.888
TO	17	15101	Novo Acordo	3.870
TO	17	15150	Novo Alegre	1.848
TO	17	15259	Novo Jardim	2.492
TO	17	15507	Oliveira de Fátima	1.114
TO	17	21000	Palmas	184.010
TO	17	15705	Palmeirante	4.837
TO	17	13809	Palmeiras do Tocantins	4.672
TO	17	15754	Palmeirópolis	8.366
TO	17	16109	Paraíso do Tocantins	41.500
TO	17	16208	Paranã	10.794
TO	17	16307	Pau D'Arco	4.909
TO	17	16505	Pedro Afonso	10.605
TO	17	16604	Peixe	9.002
TO	17	16653	Pequizeiro	4.940

U.F.		MUNICÍPIOS		POPULAÇÃO ESTIMADA
SIGLA	COD	COD	NOMES	
TO	17	17008	Pindorama do Tocantins	4.521
TO	17	17206	Piraquê	3.103
TO	17	17503	Pium	6.598
TO	17	17800	Ponte Alta do Bom Jesus	4.660
TO	17	17909	Ponte Alta do Tocantins	6.763
TO	17	18006	Porto Alegre do Tocantins	2.917
TO	17	18204	Porto Nacional	46.598
TO	17	18303	Praia Norte	7.267
TO	17	18402	Presidente Kennedy	3.785
TO	17	18451	Pugmil	2.230
TO	17	18501	Recursolândia	3.777
TO	17	18550	Riachinho	3.798
TO	17	18659	Rio da Conceição	1.499
TO	17	18709	Rio dos Bois	2.150
TO	17	18758	Rio Sono	6.345
TO	17	18808	Sampaio	3.788
TO	17	18840	Sandolândia	3.543
TO	17	18865	Santa Fé do Araguaia	5.773
TO	17	18881	Santa Maria do Tocantins	2.755
TO	17	18899	Santa Rita do Tocantins	2.330
TO	17	18907	Santa Rosa do Tocantins	4.545
TO	17	19004	Santa Tereza do Tocantins	2.366
TO	17	20002	Santa Terezinha do Tocantins	2.356
TO	17	20101	São Bento do Tocantins	4.583
TO	17	20150	São Félix do Tocantins	1.451
TO	17	20200	São Miguel do Tocantins	10.536
TO	17	20259	São Salvador do Tocantins	3.102
TO	17	20309	São Sebastião do Tocantins	4.373
TO	17	20499	São Valério da Natividade	5.024
TO	17	20655	Silvanópolis	5.250
TO	17	20804	Sítio Novo do Tocantins	9.568
TO	17	20853	Sucupira	1.718
TO	17	20903	Taguatinga	14.528
TO	17	20937	Taipas do Tocantins	1.974
TO	17	20978	Talismã	2.632
TO	17	21109	Tocantínia	6.866
TO	17	21208	Tocantinópolis	21.932
TO	17	21257	Tupirama	1.448
TO	17	21307	Tupiratins	2.072
TO	17	22081	Wanderlândia	9.574
TO	17	22107	Xambioá	11.160

U.F.		MUNICÍPIOS		POPULAÇÃO ESTIMADA
SIGLA	COD	COD	NOMES	
MA	21	00055	Açailândia	100.017
MA	21	00105	Afonso Cunha	5.834
MA	21	00154	Água Doce do Maranhão	12.213
MA	21	00204	Alcântara	21.969
MA	21	00303	Aldeias Altas	22.328
MA	21	00402	Altamira do Maranhão	7.607
MA	21	00436	Alto Alegre do Maranhão	22.676
MA	21	00477	Alto Alegre do Pindaré	32.955
MA	21	00501	Alto Parnaíba	10.606
MA	21	00550	Amapá do Maranhão	6.361
MA	21	00600	Amarante do Maranhão	36.850
MA	21	00709	Anajatuba	24.695
MA	21	00808	Anapurus	12.990
MA	21	00832	Apicum-Açu	13.641
MA	21	00873	Araguanã	10.221
MA	21	00907	Araioses	38.800
MA	21	00956	Arame	27.967
MA	21	01004	Arari	28.585
MA	21	01103	Axixá	14.743
MA	21	01202	Bacabal	97.946
MA	21	01251	Bacabeira	15.115
MA	21	01301	Bacuri	16.500
MA	21	01350	Bacurituba	5.597
MA	21	01400	Balsas	81.497
MA	21	01509	Barão de Grajaú	17.069
MA	21	01608	Barra do Corda	81.027
MA	21	01707	Barreirinhas	49.398
MA	21	01772	Bela Vista do Maranhão	8.850
MA	21	01731	Belágua	5.892
MA	21	01806	Benedito Leite	5.545
MA	21	01905	Bequimão	21.356
MA	21	01939	Bernardo do Mearim	6.160
MA	21	01970	Boa Vista do Gurupi	7.643
MA	21	02002	Bom Jardim	38.813
MA	21	02036	Bom Jesus das Selvas	24.661
MA	21	02077	Bom Lugar	13.222
MA	21	02101	Brejo	32.018
MA	21	02150	Brejo de Areia	5.896
MA	21	02200	Buriti	26.029
MA	21	02309	Buriti Bravo	22.941
MA	21	02325	Buriticupu	63.466

U.F.		MUNICÍPIOS		POPULAÇÃO ESTIMADA
SIGLA	COD	COD	NOMES	
MA	21	02358	Buritirana	15.027
MA	21	02374	Cachoeira Grande	9.116
MA	21	02408	Cajapió	10.279
MA	21	02507	Cajari	13.203
MA	21	02556	Campestre do Maranhão	12.615
MA	21	02606	Cândido Mendes	19.409
MA	21	02705	Cantanhede	19.396
MA	21	02754	Capinzal do Norte	10.683
MA	21	02804	Carolina	25.159
MA	21	02903	Carutapera	20.905
MA	21	03000	Caxias	147.416
MA	21	03109	Cedral	10.127
MA	21	03125	Central do Maranhão	9.062
MA	21	03158	Centro do Guilherme	7.318
MA	21	03174	Centro Novo do Maranhão	15.577
MA	21	03208	Chapadinha	69.734
MA	21	03257	Cidelândia	12.778
MA	21	03307	Codó	113.768
MA	21	03406	Coelho Neto	45.343
MA	21	03505	Colinas	36.725
MA	21	03554	Conceição do Lago-Açu	14.535
MA	21	03604	Coroatá	62.442
MA	21	03703	Cururupu	35.009
MA	21	03752	Davinópolis	12.118
MA	21	03802	Dom Pedro	22.092
MA	21	03901	Duque Bacelar	10.704
MA	21	04008	Esperantinópolis	19.055
MA	21	04057	Estreito	27.328
MA	21	04073	Feira Nova do Maranhão	7.872
MA	21	04081	Fernando Falcão	8.404
MA	21	04099	Formosa da Serra Negra	17.432
MA	21	04107	Fortaleza dos Nogueiras	11.919
MA	21	04206	Fortuna	14.903
MA	21	04305	Godofredo Viana	10.823
MA	21	04404	Gonçalves Dias	16.914
MA	21	04503	Governador Archer	10.224
MA	21	04552	Governador Edison Lobão	14.557
MA	21	04602	Governador Eugênio Barros	16.341
MA	21	04628	Governador Luiz Rocha	7.091
MA	21	04651	Governador Newton Bello	11.666
MA	21	04677	Governador Nunes Freire	24.671

U.F.		MUNICÍPIOS		POPULAÇÃO ESTIMADA
SIGLA	COD	COD	NOMES	
MA	21	04701	Graça Aranha	6.439
MA	21	04800	Grajaú	55.833
MA	21	04909	Guimarães	12.741
MA	21	05005	Humberto de Campos	25.037
MA	21	05104	Icatu	25.198
MA	21	05153	Igarapé do Meio	12.073
MA	21	05203	Igarapé Grande	11.003
MA	21	05302	Imperatriz	236.311
MA	21	05351	Itaipava do Grajaú	13.636
MA	21	05401	Itapecuru Mirim	56.241
MA	21	05427	Itinga do Maranhão	25.866
MA	21	05450	Jatobá	8.557
MA	21	05476	Jenipapo dos Vieiras	15.270
MA	21	05500	João Lisboa	20.482
MA	21	05609	Joselândia	16.054
MA	21	05658	Junco do Maranhão	4.101
MA	21	05708	Lago da Pedra	43.947
MA	21	05807	Lago do Junco	9.890
MA	21	05948	Lago dos Rodrigues	7.993
MA	21	05906	Lago Verde	15.014
MA	21	05922	Lagoa do Mato	10.536
MA	21	05963	Lagoa Grande do Maranhão	9.287
MA	21	05989	Lajeado Novo	6.829
MA	21	06003	Lima Campos	11.706
MA	21	06102	Loreto	10.646
MA	21	06201	Luís Domingues	6.884
MA	21	06300	Magalhães de Almeida	14.653
MA	21	06326	Maracaçumé	18.098
MA	21	06359	Marajá do Sena	6.979
MA	21	06375	Maranhãozinho	12.299
MA	21	06409	Mata Roma	14.252
MA	21	06508	Matinha	21.037
MA	21	06607	Matões	29.134
MA	21	06631	Matões do Norte	10.944
MA	21	06672	Milagres do Maranhão	7.838
MA	21	06706	Mirador	19.999
MA	21	06755	Miranda do Norte	18.288
MA	21	06805	Mirinzal	14.201
MA	21	06904	Monção	28.386
MA	21	07001	Montes Altos	9.055
MA	21	07100	Morros	17.620

U.F.		MUNICÍPIOS		POPULAÇÃO ESTIMADA
SIGLA	COD	COD	NOMES	
MA	21	07209	Nina Rodrigues	10.326
MA	21	07258	Nova Colinas	4.987
MA	21	07308	Nova Iorque	5.041
MA	21	07357	Nova Olinda do Maranhão	17.646
MA	21	07407	Olho d'Água das Cunhãs	17.868
MA	21	07456	Olinda Nova do Maranhão	12.456
MA	21	07506	Paço do Lumiar	101.452
MA	21	07605	Palmeirândia	18.646
MA	21	07704	Paraibano	20.048
MA	21	07803	Parnarama	35.972
MA	21	07902	Passagem Franca	17.625
MA	21	08009	Pastos Bons	18.055
MA	21	08058	Paulino Neves	13.195
MA	21	08108	Paulo Ramos	16.534
MA	21	08207	Pedreiras	39.049
MA	21	08256	Pedro do Rosário	22.417
MA	21	08306	Penalva	34.505
MA	21	08405	Peri Mirim	12.556
MA	21	08454	Peritoró	19.601
MA	21	08504	Pindaré-Mirim	31.879
MA	21	08603	Pinheiro	76.391
MA	21	08702	Pio XII	22.410
MA	21	08801	Pirapemas	15.477
MA	21	08900	Poção de Pedras	16.184
MA	21	09007	Porto Franco	19.271
MA	21	09056	Porto Rico do Maranhão	7.110
MA	21	09106	Presidente Dutra	41.173
MA	21	09205	Presidente Juscelino	12.094
MA	21	09239	Presidente Médici	6.252
MA	21	09270	Presidente Sarney	16.095
MA	21	09304	Presidente Vargas	10.096
MA	21	09403	Primeira Cruz	12.366
MA	21	09452	Raposa	25.042
MA	21	09502	Riachão	21.626
MA	21	09551	Ribamar Fiquene	7.391
MA	21	09601	Rosário	39.103
MA	21	09700	Sambaíba	5.971
MA	21	09759	Santa Filomena do Maranhão	5.703
MA	21	09809	Santa Helena	35.070
MA	21	09908	Santa Inês	84.582
MA	21	10005	Santa Luzia	71.314

U.F.		MUNICÍPIOS		POPULAÇÃO ESTIMADA
SIGLA	COD	COD	NOMES	
MA	21	10039	Santa Luzia do Paruá	20.194
MA	21	10104	Santa Quitéria do Maranhão	29.215
MA	21	10203	Santa Rita	31.940
MA	21	10237	Santana do Maranhão	10.890
MA	21	10278	Santo Amaro do Maranhão	11.509
MA	21	10302	Santo Antônio dos Lopes	14.637
MA	21	10401	São Benedito do Rio Preto	17.704
MA	21	10500	São Bento	38.645
MA	21	10609	São Bernardo	26.273
MA	21	10658	São Domingos do Azeitão	7.168
MA	21	10708	São Domingos do Maranhão	33.491
MA	21	10807	São Félix de Balsas	4.511
MA	21	10856	São Francisco do Brejão	8.702
MA	21	10906	São Francisco do Maranhão	14.632
MA	21	11029	São João do Carú	12.613
MA	21	11052	São João do Paraíso	11.611
MA	21	11078	São João do Soter	17.108
MA	21	11102	São João dos Patos	24.267
MA	21	11003	São João Batista	18.617
MA	21	11201	São José de Ribamar	135.821
MA	21	11250	São José dos Basílios	7.490
MA	21	11300	São Luís	986.826
MA	21	11409	São Luís Gonzaga do Maranhão	20.163
MA	21	11508	São Mateus do Maranhão	39.210
MA	21	11532	São Pedro da Água Branca	11.439
MA	21	11573	São Pedro dos Crentes	4.136
MA	21	11607	São Raimundo das Mangabeiras	16.447
MA	21	11631	São Raimundo do Doca Bezerra	4.590
MA	21	11672	São Roberto	5.145
MA	21	11706	São Vicente Ferrer	20.288
MA	21	11722	Satubinha	8.651
MA	21	11748	Senador Alexandre Costa	9.343
MA	21	11763	Senador La Rocque	19.328
MA	21	11789	Serrano do Maranhão	10.911
MA	21	11805	Sítio Novo	16.009
MA	21	11904	Sucupira do Norte	10.529
MA	21	11953	Sucupira do Riachão	4.818
MA	21	12001	Tasso Fragoso	6.850
MA	21	12100	Timbiras	26.884
MA	21	12209	Timon	148.804
MA	21	12233	Trizidela do Vale	18.867

U.F.		MUNICÍPIOS		POPULAÇÃO ESTIMADA
SIGLA	COD	COD	NOMES	
MA	21	12274	Tufilândia	5.677
MA	21	12308	Tuntum	39.041
MA	21	12407	Turiaçu	33.456
MA	21	12456	Turilândia	20.758
MA	21	12506	Tutóia	47.733
MA	21	12605	Urbano Santos	22.459
MA	21	12704	Vargem Grande	44.648
MA	21	12803	Viana	48.907
MA	21	12852	Vila Nova dos Martírios	8.961
MA	21	12902	Vitória do Mearim	31.851
MA	21	13009	Vitorino Freire	31.107
MA	21	14007	Zé Doca	46.290
PI	22	00053	Acauã	6.437
PI	22	00103	Agricolândia	5.121
PI	22	00202	Água Branca	16.345
PI	22	00251	Alagoinha do Piauí	7.662
PI	22	00277	Alegrete do Piauí	4.610
PI	22	00301	Alto Longá	14.004
PI	22	00400	Altos	39.430
PI	22	00459	Alvorada do Gurguéia	5.120
PI	22	00509	Amarante	17.813
PI	22	00608	Angical do Piauí	6.798
PI	22	00707	Anísio de Abreu	8.434
PI	22	00806	Antônio Almeida	3.231
PI	22	00905	Aroazes	6.025
PI	22	00954	Aroeiras do Itaim	2.731
PI	22	01002	Arraial	5.145
PI	22	01051	Assunção do Piauí	8.243
PI	22	01101	Avelino Lopes	11.840
PI	22	01150	Baixa Grande do Ribeiro	10.531
PI	22	01176	Barra D'Alcântara	3.882
PI	22	01200	Barras	44.574
PI	22	01309	Barreiras do Piauí	3.452
PI	22	01408	Barro Duro	6.852
PI	22	01507	Batalha	26.465
PI	22	01556	Bela Vista do Piauí	3.441
PI	22	01572	Belém do Piauí	2.869
PI	22	01606	Beneditinos	9.833
PI	22	01705	Bertolínia	5.455
PI	22	01739	Betânia do Piauí	6.379
PI	22	01770	Boa Hora	6.262

U.F.		MUNICÍPIOS		POPULAÇÃO ESTIMADA
SIGLA	COD	COD	NOMES	
PI	22	01804	Bocaina	4.155
PI	22	01903	Bom Jesus	20.100
PI	22	01919	Bom Princípio do Piauí	5.429
PI	22	01929	Bonfim do Piauí	5.334
PI	22	01945	Boqueirão do Piauí	6.508
PI	22	01960	Brasileira	7.920
PI	22	01988	Brejo do Piauí	3.986
PI	22	02000	Buriti dos Lopes	19.700
PI	22	02026	Buriti dos Montes	8.046
PI	22	02059	Cabeceiras do Piauí	9.710
PI	22	02075	Cajazeiras do Piauí	3.285
PI	22	02083	Cajueiro da Praia	7.183
PI	22	02091	Caldeirão Grande do Piauí	5.784
PI	22	02109	Campinas do Piauí	5.759
PI	22	02117	Campo Alegre do Fidalgo	4.579
PI	22	02133	Campo Grande do Piauí	5.808
PI	22	02174	Campo Largo do Piauí	6.920
PI	22	02208	Campo Maior	45.827
PI	22	02251	Canavieira	4.098
PI	22	02307	Canto do Buriti	20.556
PI	22	02406	Capitão de Campos	11.142
PI	22	02455	Capitão Gervásio Oliveira	3.977
PI	22	02505	Caracol	10.643
PI	22	02539	Caraúbas do Piauí	5.526
PI	22	02554	Caridade do Piauí	4.715
PI	22	02604	Castelo do Piauí	19.082
PI	22	02653	Caxingó	5.169
PI	22	02703	Cocal	26.956
PI	22	02711	Cocal de Telha	4.652
PI	22	02729	Cocal dos Alves	5.495
PI	22	02737	Coivaras	3.907
PI	22	02752	Colônia do Gurguéia	5.865
PI	22	02778	Colônia do Piauí	7.882
PI	22	02802	Conceição do Canindé	5.064
PI	22	02851	Coronel José Dias	4.481
PI	22	02901	Corrente	25.223
PI	22	03008	Cristalândia do Piauí	8.026
PI	22	03107	Cristino Castro	9.791
PI	22	03206	Curimatá	10.658
PI	22	03230	Currais	4.669
PI	22	03271	Curral Novo do Piauí	5.040

U.F.		MUNICÍPIOS		POPULAÇÃO ESTIMADA
SIGLA	COD	COD	NOMES	
PI	22	03255	Curralinhos	4.189
PI	22	03305	Demerval Lobão	13.173
PI	22	03354	Dirceu Arcoverde	6.915
PI	22	03404	Dom Expedito Lopes	6.721
PI	22	03453	Dom Inocêncio	10.628
PI	22	03420	Domingos Mourão	4.435
PI	22	03503	Elesbão Veloso	14.577
PI	22	03602	Eliseu Martins	4.858
PI	22	03701	Esperantina	37.231
PI	22	03750	Fartura do Piauí	5.339
PI	22	03800	Flores do Piauí	4.596
PI	22	03859	Floresta do Piauí	2.624
PI	22	03909	Floriano	57.700
PI	22	04006	Francinópolis	5.443
PI	22	04105	Francisco Ayres	5.160
PI	22	04154	Francisco Macedo	2.319
PI	22	04204	Francisco Santos	8.533
PI	22	04303	Fronteiras	11.373
PI	22	04352	Geminiano	5.406
PI	22	04402	Gilbués	10.648
PI	22	04501	Guadalupe	9.860
PI	22	04550	Guaribas	4.468
PI	22	04600	Hugo Napoleão	3.779
PI	22	04659	Ilha Grande	8.663
PI	22	04709	Inhuma	15.403
PI	22	04808	Ipiranga do Piauí	9.624
PI	22	04907	Isaías Coelho	8.014
PI	22	05003	Itainópolis	11.403
PI	22	05102	Itaueira	10.861
PI	22	05151	Jacobina do Piauí	5.774
PI	22	05201	Jaicós	17.310
PI	22	05250	Jardim do Mulato	4.291
PI	22	05276	Jatobá do Piauí	4.653
PI	22	05300	Jerumenha	4.496
PI	22	05359	João Costa	3.291
PI	22	05409	Joaquim Pires	14.175
PI	22	05458	Joca Marques	5.500
PI	22	05508	José de Freitas	36.177
PI	22	05516	Juazeiro do Piauí	4.906
PI	22	05524	Júlio Borges	5.401
PI	22	05532	Jurema	4.515

U.F.		MUNICÍPIOS		POPULAÇÃO ESTIMADA
SIGLA	COD	COD	NOMES	
PI	22	05573	Lagoa de São Francisco	6.708
PI	22	05565	Lagoa do Barro do Piauí	4.673
PI	22	05581	Lagoa do Piauí	3.790
PI	22	05599	Lagoa do Sítio	5.188
PI	22	05557	Lagoa Alegre	8.090
PI	22	05540	Lagoinha do Piauí	2.637
PI	22	05607	Landri Sales	5.746
PI	22	05706	Luís Correia	26.900
PI	22	05805	Luzilândia	25.021
PI	22	05854	Madeiro	7.902
PI	22	05904	Manoel Emídio	5.511
PI	22	05953	Marcolândia	7.361
PI	22	06001	Marcos Parente	4.318
PI	22	06050	Massapê do Piauí	6.613
PI	22	06100	Matias Olímpio	10.766
PI	22	06209	Miguel Alves	33.104
PI	22	06308	Miguel Leão	1.228
PI	22	06357	Milton Brandão	7.220
PI	22	06407	Monsenhor Gil	10.616
PI	22	06506	Monsenhor Hipólito	7.369
PI	22	06605	Monte Alegre do Piauí	10.632
PI	22	06670	Morro do Chapéu do Piauí	6.560
PI	22	06654	Morro Cabeça no Tempo	4.362
PI	22	06696	Murici dos Portelas	7.877
PI	22	06704	Nazaré do Piauí	7.144
PI	22	06720	Nazária	7.817
PI	22	06753	Nossa Senhora de Nazaré	4.165
PI	22	06803	Nossa Senhora dos Remédios	8.276
PI	22	07959	Nova Santa Rita	4.407
PI	22	06902	Novo Oriente do Piauí	6.367
PI	22	06951	Novo Santo Antônio	3.481
PI	22	07009	Oeiras	36.082
PI	22	07108	Olho D'Água do Piauí	2.713
PI	22	07207	Padre Marcos	7.551
PI	22	07306	Paes Landim	4.517
PI	22	07355	Pajeú do Piauí	3.721
PI	22	07405	Palmeira do Piauí	5.128
PI	22	07504	Palmeirais	14.117
PI	22	07553	Paquetá	4.662
PI	22	07603	Parnaguá	10.611
PI	22	07702	Parnaíba	144.892

U.F.		MUNICÍPIOS		POPULAÇÃO ESTIMADA
SIGLA	COD	COD	NOMES	
PI	22	07751	Passagem Franca do Piauí	4.245
PI	22	07777	Patos do Piauí	6.349
PI	22	07793	Pau D'Arco do Piauí	3.821
PI	22	07801	Paulistana	20.416
PI	22	07850	Pavussu	4.415
PI	22	07900	Pedro II	37.727
PI	22	07934	Pedro Laurentino	2.394
PI	22	08007	Picos	72.477
PI	22	08106	Pimenteiras	12.058
PI	22	08205	Pio IX	17.615
PI	22	08304	Piracuruca	26.361
PI	22	08403	Piripiri	61.975
PI	22	08502	Porto	11.824
PI	22	08551	Porto Alegre do Piauí	2.540
PI	22	08601	Prata do Piauí	3.249
PI	22	08650	Queimada Nova	9.642
PI	22	08700	Redenção do Gurguéia	8.500
PI	22	08809	Regeneração	18.138
PI	22	08858	Riacho Frio	5.025
PI	22	08874	Ribeira do Piauí	4.221
PI	22	08908	Ribeiro Gonçalves	6.767
PI	22	09005	Rio Grande do Piauí	6.615
PI	22	09104	Santa Cruz do Piauí	5.956
PI	22	09153	Santa Cruz dos Milagres	3.448
PI	22	09203	Santa Filomena	6.171
PI	22	09302	Santa Luz	5.432
PI	22	09377	Santa Rosa do Piauí	5.296
PI	22	09351	Santana do Piauí	4.980
PI	22	09401	Santo Antônio de Lisboa	5.895
PI	22	09450	Santo Antônio dos Milagres	2.019
PI	22	09500	Santo Inácio do Piauí	3.731
PI	22	09559	São Braz do Piauí	4.434
PI	22	09609	São Félix do Piauí	3.222
PI	22	09658	São Francisco de Assis do Piauí	5.159
PI	22	09708	São Francisco do Piauí	6.456
PI	22	09757	São Gonçalo do Gurguéia	2.525
PI	22	09807	São Gonçalo do Piauí	4.466
PI	22	09856	São João da Canabrava	4.489
PI	22	09872	São João da Fronteira	5.151
PI	22	09906	São João da Serra	6.863
PI	22	09955	São João da Varjota	4.914

U.F.		MUNICÍPIOS		POPULAÇÃO ESTIMADA
SIGLA	COD	COD	NOMES	
PI	22	09971	São João do Arraial	7.287
PI	22	10003	São João do Piauí	19.073
PI	22	10052	São José do Divino	5.190
PI	22	10102	São José do Peixe	3.837
PI	22	10201	São José do Piauí	7.004
PI	22	10300	São Julião	6.078
PI	22	10359	São Lourenço do Piauí	5.041
PI	22	10375	São Luis do Piauí	2.670
PI	22	10383	São Miguel da Baixa Grande	2.143
PI	22	10391	São Miguel do Fidalgo	3.166
PI	22	10409	São Miguel do Tapuio	19.752
PI	22	10508	São Pedro do Piauí	13.459
PI	22	10607	São Raimundo Nonato	31.744
PI	22	10623	Sebastião Barros	4.297
PI	22	10631	Sebastião Leal	4.198
PI	22	10656	Sigefredo Pacheco	9.779
PI	22	10706	Simões	14.128
PI	22	10805	Simplício Mendes	11.802
PI	22	10904	Socorro do Piauí	4.731
PI	22	10938	Sussuapara	5.696
PI	22	10953	Tamboril do Piauí	2.981
PI	22	10979	Tanque do Piauí	2.681
PI	22	11001	Teresina	794.599
PI	22	11100	União	42.859
PI	22	11209	Uruçuí	19.566
PI	22	11308	Valença do Piauí	20.280
PI	22	11357	Várzea Branca	5.326
PI	22	11407	Várzea Grande	4.556
PI	22	11506	Vera Mendes	3.187
PI	22	11605	Vila Nova do Piauí	3.117
PI	22	11704	Wall Ferraz	4.580
CE	23	00101	Abaiara	10.835
CE	23	00150	Acarape	15.388
CE	23	00200	Acaraú	54.257
CE	23	00309	Acopiara	50.485
CE	23	00408	Aiuaba	16.253
CE	23	00507	Alcântaras	10.706
CE	23	00606	Altaneira	6.733
CE	23	00705	Alto Santo	20.339
CE	23	00754	Amontada	39.497
CE	23	00804	Antonina do Norte	7.013

U.F.		MUNICÍPIOS		POPULAÇÃO ESTIMADA
SIGLA	COD	COD	NOMES	
CE	23	00903	Apuiarés	14.251
CE	23	01000	Aquiraz	70.439
CE	23	01109	Aracati	68.886
CE	23	01208	Aracoiaba	25.251
CE	23	01257	Ararendá	11.085
CE	23	01307	Araripe	22.133
CE	23	01406	Aratuba	12.477
CE	23	01505	Arneiroz	7.497
CE	23	01604	Assaré	22.412
CE	23	01703	Aurora	25.142
CE	23	01802	Baixio	5.971
CE	23	01851	Banabuiú	18.196
CE	23	01901	Barbalha	52.496
CE	23	01950	Barreira	19.257
CE	23	02008	Barro	21.429
CE	23	02057	Barroquinha	15.418
CE	23	02107	Baturité	32.968
CE	23	02206	Beberibe	48.201
CE	23	02305	Bela Cruz	30.687
CE	23	02404	Boa Viagem	55.728
CE	23	02503	Brejo Santo	41.040
CE	23	02602	Camocim	60.784
CE	23	02701	Campos Sales	26.359
CE	23	02800	Canindé	76.873
CE	23	02909	Capistrano	16.963
CE	23	03006	Caridade	18.911
CE	23	03105	Cariré	19.099
CE	23	03204	Caririaçu	27.249
CE	23	03303	Cariús	19.270
CE	23	03402	Carnaubal	16.626
CE	23	03501	Cascavel	67.002
CE	23	03600	Catarina	17.794
CE	23	03659	Catunda	11.029
CE	23	03709	Caucaia	326.811
CE	23	03808	Cedro	25.469
CE	23	03907	Chaval	12.609
CE	23	03931	Choró	13.316
CE	23	03956	Chorozinho	18.770
CE	23	04004	Coreaú	22.024
CE	23	04103	Crateús	74.904
CE	23	04202	Crato	115.724

U.F.		MUNICÍPIOS		POPULAÇÃO ESTIMADA
SIGLA	COD	COD	NOMES	
CE	23	04236	Croatá	18.057
CE	23	04251	Cruz	23.208
CE	23	04269	Deputado Irapuan Pinheiro	9.508
CE	23	04277	Ererê	7.242
CE	23	04285	Eusébio	40.426
CE	23	04301	Farias Brito	19.687
CE	23	04350	Forquilha	21.233
CE	23	04400	Fortaleza	2.473.614
CE	23	04459	Fortim	14.825
CE	23	04509	Frecheirinha	13.389
CE	23	04608	General Sampaio	6.602
CE	23	04657	Graça	15.856
CE	23	04707	Granja	53.486
CE	23	04806	Granjeiro	5.031
CE	23	04905	Groaíras	9.865
CE	23	04954	Guaiúba	23.502
CE	23	05001	Guaraciaba do Norte	38.123
CE	23	05100	Guaramiranga	4.227
CE	23	05209	Hidrolândia	19.103
CE	23	05233	Horizonte	52.488
CE	23	05266	Ibaretama	13.156
CE	23	05308	Ibiapina	23.961
CE	23	05332	Ibicuitinga	11.589
CE	23	05357	Icapuí	19.089
CE	23	05407	Icó	65.377
CE	23	05506	Iguatu	96.201
CE	23	05605	Independência	26.240
CE	23	05654	Ipaporanga	11.729
CE	23	05704	Ipaumirim	11.966
CE	23	05803	Ipu	40.841
CE	23	05902	Ipueiras	39.220
CE	23	06009	Iracema	14.943
CE	23	06108	Irauçuba	22.978
CE	23	06207	Itaiçaba	7.833
CE	23	06256	Itaitinga	32.382
CE	23	06306	Itapagé	46.989
CE	23	06405	Itapipoca	112.662
CE	23	06504	Itapiúna	18.390
CE	23	06553	Itarema	35.988
CE	23	06603	Itatira	18.579
CE	23	06702	Jaguaretama	18.389

U.F.		MUNICÍPIOS		POPULAÇÃO ESTIMADA
SIGLA	COD	COD	NOMES	
CE	23	06801	Jaguaribara	10.251
CE	23	06900	Jaguaribe	36.385
CE	23	07007	Jaguaruana	32.134
CE	23	07106	Jardim	26.584
CE	23	07205	Jati	7.501
CE	23	07254	Jijoca de Jericoacoara	16.447
CE	23	07304	Juazeiro do Norte	246.515
CE	23	07403	Jucás	23.654
CE	23	07502	Lavras da Mangabeira	30.612
CE	23	07601	Limoeiro do Norte	55.539
CE	23	07635	Madalena	17.636
CE	23	07650	Maracanaú	199.808
CE	23	07700	Maranguape	108.525
CE	23	07809	Marco	24.250
CE	23	07908	Martinópole	10.892
CE	23	08005	Massapê	34.874
CE	23	08104	Mauriti	43.871
CE	23	08203	Meruoca	12.656
CE	23	08302	Milagres	27.861
CE	23	08351	Milhã	14.691
CE	23	08377	Miraíma	12.625
CE	23	08401	Missão Velha	34.926
CE	23	08500	Mombaça	46.252
CE	23	08609	Monsenhor Tabosa	17.114
CE	23	08708	Morada Nova	63.296
CE	23	08807	Moraújo	8.406
CE	23	08906	Morrinhos	22.269
CE	23	09003	Mucambo	14.481
CE	23	09102	Mulungu	11.642
CE	23	09201	Nova Olinda	13.522
CE	23	09300	Nova Russas	31.770
CE	23	09409	Novo Oriente	28.485
CE	23	09458	Ocara	24.371
CE	23	09508	Orós	21.824
CE	23	09607	Pacajus	58.281
CE	23	09706	Pacatuba	70.018
CE	23	09805	Pacoti	11.473
CE	23	09904	Pacujá	6.184
CE	23	10001	Palhano	9.173
CE	23	10100	Palmácia	10.755
CE	23	10209	Paracuru	32.116

U.F.		MUNICÍPIOS		POPULAÇÃO ESTIMADA
SIGLA	COD	COD	NOMES	
CE	23	10258	Paraipaba	29.503
CE	23	10308	Parambu	31.983
CE	23	10407	Paramoti	12.032
CE	23	10506	Pedra Branca	42.055
CE	23	10605	Penaforte	8.067
CE	23	10704	Pentecoste	34.955
CE	23	10803	Pereiro	15.784
CE	23	10852	Pindoretama	18.022
CE	23	10902	Piquet Carneiro	15.449
CE	23	10951	Pires Ferreira	9.812
CE	23	11009	Poranga	12.308
CE	23	11108	Porteiras	15.127
CE	23	11207	Potengi	10.058
CE	23	11231	Potiretama	6.792
CE	23	11264	Quiterianópolis	20.979
CE	23	11306	Quixadá	79.505
CE	23	11355	Quixelô	16.222
CE	23	11405	Quixeramobim	72.951
CE	23	11504	Quixeré	19.517
CE	23	11603	Redenção	26.624
CE	23	11702	Reriutaba	19.626
CE	23	11801	Russas	67.023
CE	23	11900	Saboeiro	16.806
CE	23	11959	Salitre	16.586
CE	23	12205	Santa Quitéria	44.864
CE	23	12007	Santana do Acaraú	30.041
CE	23	12106	Santana do Cariri	18.242
CE	23	12304	São Benedito	44.929
CE	23	12403	São Gonçalo do Amarante	42.311
CE	23	12502	São João do Jaguaribe	8.521
CE	23	12601	São Luís do Curu	12.519
CE	23	12700	Senador Pompeu	25.069
CE	23	12809	Senador Sá	6.575
CE	23	12908	Sobral	180.046
CE	23	13005	Solonópole	17.943
CE	23	13104	Tabuleiro do Norte	29.369
CE	23	13203	Tamboril	26.650
CE	23	13252	Tarrafas	8.937
CE	23	13302	Tauá	56.202
CE	23	13351	Tejuçuoca	16.056
CE	23	13401	Tianguá	67.663

U.F.		MUNICÍPIOS		POPULAÇÃO ESTIMADA
SIGLA	COD	COD	NOMES	
CE	23	13500	Trairi	50.788
CE	23	13559	Tururu	14.057
CE	23	13609	Ubajara	30.885
CE	23	13708	Umari	7.855
CE	23	13757	Umirim	18.901
CE	23	13807	Uruburetama	20.253
CE	23	13906	Uruoca	13.613
CE	23	13955	Varjota	17.704
CE	23	14003	Várzea Alegre	39.379
CE	23	14102	Viçosa do Ceará	55.670
RN	24	00109	Acari	11.222
RN	24	00208	Açu	52.824
RN	24	00307	Afonso Bezerra	10.628
RN	24	00406	Água Nova	2.929
RN	24	00505	Alexandria	14.127
RN	24	00604	Almino Afonso	5.087
RN	24	00703	Alto do Rodrigues	11.817
RN	24	00802	Angicos	11.544
RN	24	00901	Antônio Martins	7.205
RN	24	01008	Apodi	35.648
RN	24	01107	Areia Branca	25.147
RN	24	01206	Arês	12.611
RN	24	01305	Augusto Severo	9.194
RN	24	01404	Baía Formosa	8.726
RN	24	01453	Baraúna	23.860
RN	24	01503	Barcelona	4.040
RN	24	01602	Bento Fernandes	5.158
RN	24	01651	Bodó	2.611
RN	24	01701	Bom Jesus	8.721
RN	24	01800	Brejinho	11.476
RN	24	01859	Caiçara do Norte	6.582
RN	24	01909	Caiçara do Rio do Vento	3.158
RN	24	02006	Caicó	62.497
RN	24	02105	Campo Redondo	10.793
RN	24	02204	Canguaretama	30.237
RN	24	02303	Caraúbas	20.333
RN	24	02402	Carnaúba dos Dantas	7.041
RN	24	02501	Carnaubais	9.578
RN	24	02600	Ceará-Mirim	67.416
RN	24	02709	Cerro Corá	11.207
RN	24	02808	Coronel Ezequiel	5.404

U.F.		MUNICÍPIOS		POPULAÇÃO ESTIMADA
SIGLA	COD	COD	NOMES	
RN	24	02907	Coronel João Pessoa	4.970
RN	24	03004	Cruzeta	8.046
RN	24	03103	Currais Novos	43.315
RN	24	03202	Doutor Severiano	6.615
RN	24	03301	Encanto	5.316
RN	24	03400	Equador	6.050
RN	24	03509	Espírito Santo	10.414
RN	24	03608	Extremoz	22.473
RN	24	03707	Felipe Guerra	5.848
RN	24	03756	Fernando Pedroza	2.964
RN	24	03806	Florânia	8.540
RN	24	03905	Francisco Dantas	3.011
RN	24	04002	Frutuoso Gomes	4.482
RN	24	04101	Galinhos	2.219
RN	24	04200	Goianinha	20.997
RN	24	04309	Governador Dix-Sept Rosado	12.747
RN	24	04408	Grossos	9.741
RN	24	04507	Guamaré	12.157
RN	24	04606	Ielmo Marinho	12.018
RN	24	04705	Ipanguaçu	13.868
RN	24	04804	Ipueira	2.097
RN	24	04853	Itajá	6.599
RN	24	04903	Itaú	5.936
RN	24	05009	Jaçanã	8.017
RN	24	05108	Jandaíra	6.641
RN	24	05207	Janduís	5.569
RN	24	05306	Januário Cicco	8.548
RN	24	05405	Japi	5.757
RN	24	05504	Jardim de Angicos	2.607
RN	24	05603	Jardim de Piranhas	14.139
RN	24	05702	Jardim do Seridó	12.362
RN	24	05801	João Câmara	31.332
RN	24	05900	João Dias	2.805
RN	24	06007	José da Penha	6.157
RN	24	06106	Jucurutu	18.014
RN	24	06155	Jundiá	3.626
RN	24	06205	Lagoa d'Anta	6.119
RN	24	06304	Lagoa de Pedras	7.205
RN	24	06403	Lagoa de Velhos	2.778
RN	24	06502	Lagoa Nova	13.574
RN	24	06601	Lagoa Salgada	7.395

U.F.		MUNICÍPIOS		POPULAÇÃO ESTIMADA
SIGLA	COD	COD	NOMES	
RN	24	06700	Lajes	10.737
RN	24	06809	Lajes Pintadas	4.333
RN	24	06908	Lucrécia	3.522
RN	24	07005	Luís Gomes	10.060
RN	24	07104	Macaíba	65.361
RN	24	07203	Macau	27.951
RN	24	07252	Major Sales	3.571
RN	24	07302	Marcelino Vieira	8.342
RN	24	07401	Martins	8.331
RN	24	07500	Maxaranguape	9.251
RN	24	07609	Messias Targino	3.907
RN	24	07708	Montanhas	12.762
RN	24	07906	Monte das Gameleiras	2.460
RN	24	07807	Monte Alegre	21.225
RN	24	08003	Mossoró	241.645
RN	24	08102	Natal	798.065
RN	24	08201	Nísia Floresta	23.655
RN	24	08300	Nova Cruz	36.336
RN	24	08409	Olho-d'Água do Borges	4.570
RN	24	08508	Ouro Branco	5.124
RN	24	08607	Paraná	4.004
RN	24	08706	Paraú	3.988
RN	24	08805	Parazinho	4.920
RN	24	08904	Parelhas	20.566
RN	24	03251	Parnamirim	178.819
RN	24	09100	Passa e Fica	10.718
RN	24	09209	Passagem	2.704
RN	24	09308	Patu	11.634
RN	24	09407	Pau dos Ferros	27.547
RN	24	09506	Pedra Grande	4.030
RN	24	09605	Pedra Preta	2.732
RN	24	09704	Pedro Avelino	7.607
RN	24	09803	Pedro Velho	14.073
RN	24	09902	Pendências	12.893
RN	24	10009	Pilões	3.488
RN	24	10108	Poço Branco	12.645
RN	24	10207	Portalegre	7.056
RN	24	10256	Porto do Mangue	4.947
RN	24	10306	Presidente Juscelino	8.552
RN	24	10405	Pureza	8.287
RN	24	10504	Rafael Fernandes	4.750

U.F.		MUNICÍPIOS		POPULAÇÃO ESTIMADA
SIGLA	COD	COD	NOMES	
RN	24	10603	Rafael Godeiro	3.226
RN	24	10702	Riacho da Cruz	3.121
RN	24	10801	Riacho de Santana	4.419
RN	24	10900	Riachuelo	7.045
RN	24	08953	Rio do Fogo	10.048
RN	24	11007	Rodolfo Fernandes	4.704
RN	24	11106	Ruy Barbosa	3.729
RN	24	11205	Santa Cruz	34.769
RN	24	09332	Santa Maria	4.813
RN	24	11403	Santana do Matos	14.690
RN	24	11429	Santana do Seridó	2.816
RN	24	11502	Santo Antônio	21.905
RN	24	11601	São Bento do Norte	3.635
RN	24	11700	São Bento do Trairí	3.820
RN	24	11809	São Fernando	3.483
RN	24	11908	São Francisco do Oeste	3.780
RN	24	12005	São Gonçalo do Amarante	79.776
RN	24	12104	São João do Sabugi	5.934
RN	24	12203	São José de Mipibu	38.109
RN	24	12302	São José do Campestre	12.080
RN	24	12401	São José do Seridó	4.042
RN	24	12500	São Miguel	23.288
RN	24	12559	São Miguel do Gostoso	9.093
RN	24	12609	São Paulo do Potengi	14.918
RN	24	12708	São Pedro	6.612
RN	24	12807	São Rafael	8.350
RN	24	12906	São Tomé	11.445
RN	24	13003	São Vicente	5.992
RN	24	13102	Senador Elói de Souza	6.097
RN	24	13201	Senador Georgino Avelino	3.806
RN	24	13300	Serra de São Bento	5.968
RN	24	13359	Serra do Mel	9.505
RN	24	13409	Serra Negra do Norte	7.445
RN	24	13508	Serrinha	6.925
RN	24	13557	Serrinha dos Pintos	4.488
RN	24	13607	Severiano Melo	5.728
RN	24	13706	Sítio Novo	5.380
RN	24	13805	Taboleiro Grande	2.349
RN	24	13904	Taipu	12.115
RN	24	14001	Tangará	13.482
RN	24	14100	Tenente Ananias	9.591

U.F.		MUNICÍPIOS		POPULAÇÃO ESTIMADA
SIGLA	COD	COD	NOMES	
RN	24	14159	Tenente Laurentino Cruz	5.284
RN	24	11056	Tibau	3.871
RN	24	14209	Tibau do Sul	11.347
RN	24	14308	Timbaúba dos Batistas	2.364
RN	24	14407	Touros	30.325
RN	24	14456	Triunfo Potiguar	3.359
RN	24	14506	Umarizal	10.939
RN	24	14605	Upanema	13.126
RN	24	14704	Várzea	5.435
RN	24	14753	Venha-Ver	3.597
RN	24	14803	Vera Cruz	10.652
RN	24	14902	Viçosa	1.826
RN	24	15008	Vila Flor	2.727
PB	25	00106	Água Branca	9.477
PB	25	00205	Aguiar	5.789
PB	25	00304	Alagoa Grande	28.246
PB	25	00403	Alagoa Nova	19.700
PB	25	00502	Alagoinha	13.389
PB	25	00536	Alcantil	5.211
PB	25	00577	Algodão de Jandaíra	2.407
PB	25	00601	Alhandra	18.674
PB	25	00734	Amparo	2.062
PB	25	00775	Aparecida	7.523
PB	25	00809	Araçagi	17.877
PB	25	00908	Arara	12.698
PB	25	01005	Araruna	19.708
PB	25	01104	Areia	25.714
PB	25	01153	Areia de Baraúnas	2.156
PB	25	01203	Areial	6.409
PB	25	01302	Aroeiras	19.722
PB	25	01351	Assunção	3.426
PB	25	01401	Baía da Traição	7.834
PB	25	01500	Bananeiras	22.287
PB	25	01534	Baraúna	3.967
PB	25	01609	Barra de Santa Rosa	13.208
PB	25	01575	Barra de Santana	8.861
PB	25	01708	Barra de São Miguel	5.586
PB	25	01807	Bayeux	95.470
PB	25	01906	Belém	17.654
PB	25	02003	Belém do Brejo do Cruz	7.239
PB	25	02052	Bernardino Batista	3.247

U.F.		MUNICÍPIOS		POPULAÇÃO ESTIMADA
SIGLA	COD	COD	NOMES	
PB	25	02102	Boa Ventura	5.973
PB	25	02151	Boa Vista	5.826
PB	25	02201	Bom Jesus	2.296
PB	25	02300	Bom Sucesso	5.299
PB	25	02409	Bonito de Santa Fé	10.533
PB	25	02508	Boqueirão	16.327
PB	25	02706	Borborema	5.148
PB	25	02805	Brejo do Cruz	12.770
PB	25	02904	Brejo dos Santos	5.908
PB	25	03001	Caaporã	19.929
PB	25	03100	Cabaceiras	5.039
PB	25	03209	Cabedelo	51.064
PB	25	03308	Cachoeira dos Índios	8.620
PB	25	03407	Cacimba de Areia	3.583
PB	25	03506	Cacimba de Dentro	17.590
PB	25	03555	Cacimbas	6.975
PB	25	03605	Caiçara	7.522
PB	25	03704	Cajazeiras	57.627
PB	25	03753	Cajazeirinhas	3.141
PB	25	03803	Caldas Brandão	5.513
PB	25	03902	Camalaú	5.922
PB	25	04009	Campina Grande	381.422
PB	25	16409	Campo de Santana	9.818
PB	25	04033	Capim	5.344
PB	25	04074	Caraúbas	3.929
PB	25	04108	Carrapateira	2.345
PB	25	04157	Casserengue	6.951
PB	25	04207	Catingueira	4.986
PB	25	04306	Catolé do Rocha	28.320
PB	25	04355	Caturité	4.591
PB	25	04405	Conceição	17.997
PB	25	04504	Condado	6.890
PB	25	04603	Conde	20.452
PB	25	04702	Congo	4.903
PB	25	04801	Coremas	15.668
PB	25	04850	Coxixola	1.752
PB	25	04900	Cruz do Espírito Santo	15.702
PB	25	05006	Cubati	6.537
PB	25	05105	Cuité	20.768
PB	25	05238	Cuité de Mamanguape	6.685
PB	25	05204	Cuitegi	7.250

U.F.		MUNICÍPIOS		POPULAÇÃO ESTIMADA
SIGLA	COD	COD	NOMES	
PB	25	05279	Curral de Cima	5.622
PB	25	05303	Curral Velho	2.858
PB	25	05352	Damião	4.938
PB	25	05402	Desterro	8.152
PB	25	05600	Diamante	6.772
PB	25	05709	Dona Inês	11.142
PB	25	05808	Duas Estradas	3.855
PB	25	05907	Emas	3.347
PB	25	06004	Esperança	30.629
PB	25	06103	Fagundes	12.166
PB	25	06202	Frei Martinho	3.018
PB	25	06251	Gado Bravo	8.473
PB	25	06301	Guarabira	55.704
PB	25	06400	Gurinhém	13.987
PB	25	06509	Gurjão	3.067
PB	25	06608	Ibiara	6.316
PB	25	02607	Igaracy	6.513
PB	25	06707	Imaculada	11.770
PB	25	06806	Ingá	18.677
PB	25	06905	Itabaiana	25.460
PB	25	07002	Itaporanga	23.047
PB	25	07101	Itapororoca	16.405
PB	25	07200	Itatuba	10.115
PB	25	07309	Jacaraú	14.102
PB	25	07408	Jericó	8.043
PB	25	07507	João Pessoa	693.082
PB	25	07606	Juarez Távora	7.597
PB	25	07705	Juazeirinho	16.339
PB	25	07804	Junco do Seridó	6.665
PB	25	07903	Juripiranga	10.524
PB	25	08000	Juru	10.510
PB	25	08109	Lagoa	4.943
PB	25	08208	Lagoa de Dentro	7.463
PB	25	08307	Lagoa Seca	25.636
PB	25	08406	Lastro	2.917
PB	25	08505	Livramento	7.305
PB	25	08554	Logradouro	3.920
PB	25	08604	Lucena	11.241
PB	25	08703	Mãe d'Água	4.133
PB	25	08802	Malta	5.794
PB	25	08901	Mamanguape	41.406

U.F.		MUNICÍPIOS		POPULAÇÃO ESTIMADA
SIGLA	COD	COD	NOMES	
PB	25	09008	Manaíra	11.292
PB	25	09057	Marcação	7.482
PB	25	09107	Mari	21.110
PB	25	09156	Marizópolis	6.384
PB	25	09206	Massaranduba	12.840
PB	25	09305	Mataraca	7.170
PB	25	09339	Matinhas	4.296
PB	25	09370	Mato Grosso	2.673
PB	25	09396	Maturéia	5.941
PB	25	09404	Mogeiro	12.493
PB	25	09503	Montadas	4.681
PB	25	09602	Monte Horebe	4.466
PB	25	09701	Monteiro	30.807
PB	25	09800	Mulungu	9.577
PB	25	09909	Natuba	10.510
PB	25	10006	Nazarezinho	7.242
PB	25	10105	Nova Floresta	10.310
PB	25	10204	Nova Olinda	6.461
PB	25	10303	Nova Palmeira	4.042
PB	25	10402	Olho d'Água	7.666
PB	25	10501	Olivedos	3.585
PB	25	10600	Ouro Velho	3.057
PB	25	10659	Parari	1.283
PB	25	10709	Passagem	2.183
PB	25	10808	Patos	99.977
PB	25	10907	Paulista	11.945
PB	25	11004	Pedra Branca	3.849
PB	25	11103	Pedra Lavrada	7.001
PB	25	11202	Pedras de Fogo	27.021
PB	25	12721	Pedro Régis	5.739
PB	25	11301	Piancó	16.321
PB	25	11400	Picuí	19.238
PB	25	11509	Pilar	11.610
PB	25	11608	Pilões	7.142
PB	25	11707	Pilõezinhos	5.471
PB	25	11806	Pirpirituba	10.522
PB	25	11905	Pitimbu	16.574
PB	25	12002	Pocinhos	16.397
PB	25	12077	Poço de José de Moura	4.066
PB	25	12036	Poço Dantas	4.029
PB	25	12101	Pombal	32.424

U.F.		MUNICÍPIOS		POPULAÇÃO ESTIMADA
SIGLA	COD	COD	NOMES	
PB	25	12200	Prata	4.001
PB	25	12309	Princesa Isabel	19.867
PB	25	12408	Puxinanã	13.236
PB	25	12507	Queimadas	39.956
PB	25	12606	Quixabá	1.472
PB	25	12705	Remígio	17.203
PB	25	12747	Riachão	3.495
PB	25	12754	Riachão do Bacamarte	4.288
PB	25	12762	Riachão do Poço	4.353
PB	25	12788	Riacho de Santo Antônio	1.566
PB	25	12804	Riacho dos Cavalos	8.285
PB	25	12903	Rio Tinto	23.669
PB	25	13000	Salgadinho	3.456
PB	25	13109	Salgado de São Félix	12.876
PB	25	13158	Santa Cecília	7.214
PB	25	13208	Santa Cruz	6.664
PB	25	13307	Santa Helena	6.200
PB	25	13356	Santa Inês	3.810
PB	25	13406	Santa Luzia	14.695
PB	25	13703	Santa Rita	125.858
PB	25	13802	Santa Teresinha	4.777
PB	25	13505	Santana de Mangueira	5.770
PB	25	13604	Santana dos Garrotes	7.829
PB	25	13653	Santarém	2.734
PB	25	13851	Santo André	2.718
PB	25	13927	São Bentinho	4.159
PB	25	13901	São Bento	29.992
PB	25	13968	São Domingos de Pombal	2.794
PB	25	13943	São Domingos do Cariri	2.329
PB	25	13984	São Francisco	3.540
PB	25	14008	São João do Cariri	4.565
PB	25	00700	São João do Rio do Peixe	18.276
PB	25	14107	São João do Tigre	4.707
PB	25	14206	São José da Lagoa Tapada	8.029
PB	25	14305	São José de Caiana	6.108
PB	25	14404	São José de Espinharas	5.055
PB	25	14503	São José de Piranhas	19.424
PB	25	14552	São José de Princesa	4.780
PB	25	14602	São José do Bonfim	3.266
PB	25	14651	São José do Brejo do Cruz	1.692
PB	25	14701	São José do Sabugi	4.098

U.F.		MUNICÍPIOS		POPULAÇÃO ESTIMADA
SIGLA	COD	COD	NOMES	
PB	25	14800	São José dos Cordeiros	4.088
PB	25	14453	São José dos Ramos	5.631
PB	25	14909	São Mamede	8.006
PB	25	15005	São Miguel de Taipu	6.749
PB	25	15104	São Sebastião de Lagoa de Roça	11.208
PB	25	15203	São Sebastião do Umbuzeiro	3.147
PB	25	15302	Sapé	47.690
PB	25	15401	Seridó	10.007
PB	25	15609	Serra da Raiz	3.223
PB	25	15500	Serra Branca	12.762
PB	25	15708	Serra Grande	3.122
PB	25	15807	Serra Redonda	7.865
PB	25	15906	Serraria	6.790
PB	25	15930	Sertãozinho	4.325
PB	25	15971	Sobrado	7.543
PB	25	16003	Solânea	28.149
PB	25	16102	Soledade	13.489
PB	25	16151	Sossêgo	3.045
PB	25	16201	Sousa	65.568
PB	25	16300	Sumé	16.908
PB	25	16508	Taperoá	15.124
PB	25	16607	Tavares	14.414
PB	25	16706	Teixeira	14.064
PB	25	16755	Tenório	2.882
PB	25	16805	Triunfo	9.891
PB	25	16904	Uiraúna	14.855
PB	25	17001	Umbuzeiro	9.433
PB	25	17100	Várzea	2.522
PB	25	17209	Vieirópolis	4.890
PB	25	05501	Vista Serrana	3.344
PB	25	17407	Zabelê	2.080
PE	26	00054	Abreu e Lima	95.670
PE	26	00104	Afogados da Ingazeira	35.314
PE	26	00203	Afrânio	17.233
PE	26	00302	Agrestina	22.371
PE	26	00401	Água Preta	30.606
PE	26	00500	Águas Belas	39.421
PE	26	00609	Alagoinha	14.704
PE	26	00708	Aliança	35.446
PE	26	00807	Altinho	22.418
PE	26	00906	Amaraji	20.600

U.F.		MUNICÍPIOS		POPULAÇÃO ESTIMADA
SIGLA	COD	COD	NOMES	
PE	26	01003	Angelim	10.272
PE	26	01052	Araçoiaba	17.276
PE	26	01102	Araripina	79.104
PE	26	01201	Arcoverde	67.458
PE	26	01300	Barra de Guabiraba	13.384
PE	26	01409	Barreiros	43.502
PE	26	01508	Belém de Maria	9.797
PE	26	01607	Belém de São Francisco	21.254
PE	26	01706	Belo Jardim	73.590
PE	26	01805	Betânia	11.955
PE	26	01904	Bezerros	58.310
PE	26	02001	Bodocó	34.712
PE	26	02100	Bom Conselho	44.991
PE	26	02209	Bom Jardim	40.592
PE	26	02308	Bonito	40.578
PE	26	02407	Brejão	9.707
PE	26	02506	Brejinho	7.366
PE	26	02605	Brejo da Madre de Deus	41.898
PE	26	02704	Buenos Aires	13.530
PE	26	02803	Buíque	52.466
PE	26	02902	Cabo de Santo Agostinho	169.986
PE	26	03009	Cabrobó	30.113
PE	26	03108	Cachoeirinha	18.037
PE	26	03207	Caetés	26.197
PE	26	03306	Calçado	11.637
PE	26	03405	Calumbi	7.900
PE	26	03454	Camaragibe	141.973
PE	26	03504	Camocim de São Félix	16.452
PE	26	03603	Camutanga	8.186
PE	26	03702	Canhotinho	24.874
PE	26	03801	Capoeiras	19.916
PE	26	03900	Carnaíba	19.035
PE	26	03926	Carnaubeira da Penha	12.270
PE	26	04007	Carpina	67.727
PE	26	04106	Caruaru	294.558
PE	26	04155	Casinhas	14.675
PE	26	04205	Catende	34.907
PE	26	04304	Cedro	10.678
PE	26	04403	Chã de Alegria	12.094
PE	26	04502	Chã Grande	17.984
PE	26	04601	Condado	24.180

U.F.		MUNICÍPIOS		POPULAÇÃO ESTIMADA
SIGLA	COD	COD	NOMES	
PE	26	04700	Correntes	16.733
PE	26	04809	Cortês	11.812
PE	26	04908	Cumaru	15.099
PE	26	05004	Cupira	22.764
PE	26	05103	Custódia	33.537
PE	26	05152	Dormentes	16.284
PE	26	05202	Escada	62.163
PE	26	05301	Exu	31.236
PE	26	05400	Feira Nova	19.957
PE	26	05459	Fernando de Noronha	3.012
PE	26	05509	Ferreiros	11.397
PE	26	05608	Flores	22.846
PE	26	05707	Floresta	27.809
PE	26	05806	Frei Miguelinho	14.692
PE	26	05905	Gameleira	27.489
PE	26	06002	Garanhuns	130.154
PE	26	06101	Glória do Goitá	28.243
PE	26	06200	Goiana	74.182
PE	26	06309	Granito	6.882
PE	26	06408	Gravatá	74.548
PE	26	06507	Iati	18.304
PE	26	06606	Ibimirim	28.605
PE	26	06705	Ibirajuba	7.803
PE	26	06804	Igarassu	98.601
PE	26	06903	Iguaraci	12.322
PE	26	07604	Ilha de Itamaracá	18.412
PE	26	07000	Inajá	14.605
PE	26	07109	Ingazeira	4.565
PE	26	07208	Ipojuca	74.059
PE	26	07307	Ipubi	26.973
PE	26	07406	Itacuruba	4.297
PE	26	07505	Itaíba	27.576
PE	26	07653	Itambé	36.049
PE	26	07703	Itapetim	14.140
PE	26	07752	Itapissuma	24.026
PE	26	07802	Itaquitinga	15.468
PE	26	07901	Jaboatão dos Guararapes	678.346
PE	26	07950	Jaqueira	12.560
PE	26	08008	Jataúba	15.311
PE	26	08057	Jatobá	14.342
PE	26	08107	João Alfredo	29.634

U.F.		MUNICÍPIOS		POPULAÇÃO ESTIMADA
SIGLA	COD	COD	NOMES	
PE	26	08206	Joaquim Nabuco	16.458
PE	26	08255	Jucati	10.965
PE	26	08305	Jupi	14.274
PE	26	08404	Jurema	15.396
PE	26	08453	Lagoa do Carro	15.044
PE	26	08503	Lagoa do Itaenga	20.593
PE	26	08602	Lagoa do Ouro	12.136
PE	26	08701	Lagoa dos Gatos	15.589
PE	26	08750	Lagoa Grande	22.121
PE	26	08800	Lajedo	34.595
PE	26	08909	Limoeiro	57.203
PE	26	09006	Macaparana	23.907
PE	26	09105	Machados	11.662
PE	26	09154	Manari	17.639
PE	26	09204	Maraial	12.473
PE	26	09303	Mirandiba	13.757
PE	26	14303	Moreilândia	10.643
PE	26	09402	Moreno	55.102
PE	26	09501	Nazaré da Mata	30.122
PE	26	09600	Olinda	394.850
PE	26	09709	Orobó	22.244
PE	26	09808	Orocó	13.968
PE	26	09907	Ouricuri	66.077
PE	26	10004	Palmares	58.584
PE	26	10103	Palmeirina	8.586
PE	26	10202	Panelas	25.555
PE	26	10301	Paranatama	12.256
PE	26	10400	Parnamirim	19.813
PE	26	10509	Passira	28.598
PE	26	10608	Paudalho	47.337
PE	26	10707	Paulista	314.302
PE	26	10806	Pedra	20.754
PE	26	10905	Pesqueira	63.878
PE	26	11002	Petrolândia	32.105
PE	26	11101	Petrolina	276.174
PE	26	11200	Poção	11.482
PE	26	11309	Pombos	22.253
PE	26	11408	Primavera	12.291
PE	26	11507	Quipapá	25.301
PE	26	11533	Quixaba	7.097
PE	26	11606	Recife	1.549.980

U.F.		MUNICÍPIOS		POPULAÇÃO ESTIMADA
SIGLA	COD	COD	NOMES	
PE	26	11705	Riacho das Almas	18.871
PE	26	11804	Ribeirão	39.548
PE	26	11903	Rio Formoso	21.735
PE	26	12000	Sairé	14.155
PE	26	12109	Salgadinho	8.120
PE	26	12208	Salgueiro	55.119
PE	26	12307	Saloá	15.509
PE	26	12406	Sanharó	18.473
PE	26	12455	Santa Cruz	14.466
PE	26	12471	Santa Cruz da Baixa Verde	12.096
PE	26	12505	Santa Cruz do Capibaribe	78.410
PE	26	12554	Santa Filomena	14.465
PE	26	12604	Santa Maria da Boa Vista	41.329
PE	26	12703	Santa Maria do Cambucá	12.840
PE	26	12802	Santa Terezinha	10.198
PE	26	12901	São Benedito do Sul	10.854
PE	26	13008	São Bento do Una	49.038
PE	26	13107	São Caitano	36.094
PE	26	13206	São João	21.886
PE	26	13305	São Joaquim do Monte	21.700
PE	26	13404	São José da Coroa Grande	18.143
PE	26	13503	São José do Belmonte	33.916
PE	26	13602	São José do Egito	31.601
PE	26	13701	São Lourenço da Mata	99.136
PE	26	13800	São Vicente Ferrer	17.223
PE	26	13909	Serra Talhada	79.484
PE	26	14006	Serrita	18.869
PE	26	14105	Sertânia	35.546
PE	26	14204	Sirinhaém	38.122
PE	26	14402	Solidão	6.073
PE	26	14501	Surubim	56.238
PE	26	14600	Tabira	26.947
PE	26	14709	Tacaimbó	12.344
PE	26	14808	Tacaratu	21.770
PE	26	14857	Tamandaré	18.854
PE	26	15003	Taquaritinga do Norte	22.404
PE	26	15102	Terezinha	6.735
PE	26	15201	Terra Nova	9.596
PE	26	15300	Timbaúba	52.291
PE	26	15409	Toritama	32.169
PE	26	15508	Tracunhaém	13.194

U.F.		MUNICÍPIOS		POPULAÇÃO ESTIMADA
SIGLA	COD	COD	NOMES	
PE	26	15607	Trindade	25.869
PE	26	15706	Triunfo	15.724
PE	26	15805	Tupanatinga	19.207
PE	26	15904	Tuparetama	8.600
PE	26	16001	Venturosa	16.417
PE	26	16100	Verdejante	9.989
PE	26	16183	Vertente do Lério	7.570
PE	26	16209	Vertentes	17.899
PE	26	16308	Vicência	27.993
PE	26	16407	Vitória de Santo Antão	125.681
PE	26	16506	Xexéu	14.779
AL	27	00102	Água Branca	19.882
AL	27	00201	Anadia	17.765
AL	27	00300	Arapiraca	208.447
AL	27	00409	Atalaia	51.903
AL	27	00508	Barra de Santo Antônio	14.159
AL	27	00607	Barra de São Miguel	7.467
AL	27	00706	Batalha	16.735
AL	27	00805	Belém	5.165
AL	27	00904	Belo Monte	7.452
AL	27	01001	Boca da Mata	25.882
AL	27	01100	Branquinha	12.142
AL	27	01209	Cacimbinhas	10.131
AL	27	01308	Cajueiro	20.670
AL	27	01357	Campestre	6.187
AL	27	01407	Campo Alegre	46.671
AL	27	01506	Campo Grande	9.845
AL	27	01605	Canapi	18.313
AL	27	01704	Capela	17.504
AL	27	01803	Carneiros	8.295
AL	27	01902	Chã Preta	7.143
AL	27	02009	Coité do Nóia	11.217
AL	27	02108	Colônia Leopoldina	19.946
AL	27	02207	Coqueiro Seco	5.493
AL	27	02306	Coruripe	52.584
AL	27	02355	Craíbas	23.079
AL	27	02405	Delmiro Gouveia	47.991
AL	27	02504	Dois Riachos	11.196
AL	27	02553	Estrela de Alagoas	17.180
AL	27	02603	Feira Grande	21.790
AL	27	02702	Feliz Deserto	4.503

U.F.		MUNICÍPIOS		POPULAÇÃO ESTIMADA
SIGLA	COD	COD	NOMES	
AL	27	02801	Flexeiras	12.271
AL	27	02900	Girau do Ponciano	36.250
AL	27	03007	Ibateguara	15.805
AL	27	03106	Igaci	25.860
AL	27	03205	Igreja Nova	23.606
AL	27	03304	Inhapi	18.144
AL	27	03403	Jacaré dos Homens	5.890
AL	27	03502	Jacuípe	7.076
AL	27	03601	Japaratinga	7.686
AL	27	03700	Jaramataia	6.112
AL	27	03759	Jequiá da Praia	11.741
AL	27	03809	Joaquim Gomes	22.366
AL	27	03908	Jundiá	4.700
AL	27	04005	Junqueiro	25.175
AL	27	04104	Lagoa da Canoa	18.378
AL	27	04203	Limoeiro de Anadia	26.236
AL	27	04302	Maceió	924.143
AL	27	04401	Major Isidoro	19.369
AL	27	04906	Mar Vermelho	4.129
AL	27	04500	Maragogi	26.520
AL	27	04609	Maravilha	10.452
AL	27	04708	Marechal Deodoro	46.565
AL	27	04807	Maribondo	14.249
AL	27	05002	Mata Grande	25.304
AL	27	05101	Matriz de Camaragibe	25.376
AL	27	05200	Messias	15.547
AL	27	05309	Minador do Negrão	5.306
AL	27	05408	Monteirópolis	7.293
AL	27	05507	Murici	26.731
AL	27	05606	Novo Lino	12.266
AL	27	05705	Olho d'Água das Flores	20.465
AL	27	05804	Olho d'Água do Casado	8.388
AL	27	05903	Olho d'Água Grande	4.956
AL	27	06000	Olivença	10.828
AL	27	06109	Ouro Branco	11.381
AL	27	06208	Palestina	5.024
AL	27	06307	Palmeira dos Índios	72.202
AL	27	06406	Pão de Açúcar	24.537
AL	27	06422	Pariconha	10.505
AL	27	06448	Paripueira	10.027
AL	27	06505	Passo de Camaragibe	14.225

U.F.		MUNICÍPIOS		POPULAÇÃO ESTIMADA
SIGLA	COD	COD	NOMES	
AL	27	06604	Paulo Jacinto	7.752
AL	27	06703	Penedo	60.750
AL	27	06802	Piaçabuçu	17.980
AL	27	06901	Pilar	32.546
AL	27	07008	Pindoba	3.218
AL	27	07107	Piranhas	24.651
AL	27	07206	Poço das Trincheiras	12.544
AL	27	07404	Porto de Pedras	10.620
AL	27	07305	Porto Calvo	25.870
AL	27	07503	Porto Real do Colégio	18.459
AL	27	07602	Quebrangulo	11.607
AL	27	07701	Rio Largo	67.359
AL	27	07800	Roteiro	6.881
AL	27	07909	Santa Luzia do Norte	7.468
AL	27	08006	Santana do Ipanema	43.527
AL	27	08105	Santana do Mundaú	12.002
AL	27	08204	São Brás	7.020
AL	27	08303	São José da Laje	22.941
AL	27	08402	São José da Tapera	31.032
AL	27	08501	São Luís do Quitunde	32.588
AL	27	08600	São Miguel dos Campos	53.070
AL	27	08709	São Miguel dos Milagres	7.445
AL	27	08808	São Sebastião	31.922
AL	27	08907	Satuba	14.584
AL	27	08956	Senador Rui Palmeira	13.015
AL	27	09004	Tanque d'Arca	5.848
AL	27	09103	Taquarana	18.695
AL	27	09152	Teotônio Vilela	41.498
AL	27	09202	Traipu	25.650
AL	27	09301	União dos Palmares	62.395
AL	27	09400	Viçosa	26.799
SE	28	00100	Amparo de São Francisco	2.261
SE	28	00209	Aquidabã	19.762
SE	28	00308	Aracaju	536.785
SE	28	00407	Arauá	11.980
SE	28	00506	Areia Branca	16.568
SE	28	00605	Barra dos Coqueiros	19.809
SE	28	00670	Boquim	25.190
SE	28	00704	Brejo Grande	8.001
SE	28	01009	Campo do Brito	16.612
SE	28	01108	Canhoba	4.023

U.F.		MUNICÍPIOS		POPULAÇÃO ESTIMADA
SIGLA	COD	COD	NOMES	
SE	28	01207	Canindé de São Francisco	22.534
SE	28	01306	Capela	28.756
SE	28	01405	Carira	19.543
SE	28	01504	Carmópolis	12.317
SE	28	01603	Cedro de São João	5.513
SE	28	01702	Cristinápolis	16.006
SE	28	01900	Cumbe	3.852
SE	28	02007	Divina Pastora	4.341
SE	28	02106	Estância	63.206
SE	28	02205	Feira Nova	5.805
SE	28	02304	Frei Paulo	12.969
SE	28	02403	Gararu	11.949
SE	28	02502	General Maynard	2.862
SE	28	02601	Gracho Cardoso	5.716
SE	28	02700	Ilha das Flores	8.855
SE	28	02809	Indiaroba	17.677
SE	28	02908	Itabaiana	85.725
SE	28	03005	Itabaianinha	38.565
SE	28	03104	Itabi	4.863
SE	28	03203	Itaporanga d'Ajuda	29.010
SE	28	03302	Japaratuba	15.920
SE	28	03401	Japoatã	13.944
SE	28	03500	Lagarto	91.696
SE	28	03609	Laranjeiras	24.626
SE	28	03708	Macambira	6.489
SE	28	03807	Malhada dos Bois	3.775
SE	28	03906	Malhador	12.074
SE	28	04003	Maruim	15.583
SE	28	04102	Moita Bonita	11.230
SE	28	04201	Monte Alegre de Sergipe	13.620
SE	28	04300	Muribeca	7.438
SE	28	04409	Neópolis	19.466
SE	28	04508	Nossa Senhora da Glória	30.466
SE	28	04607	Nossa Senhora das Dores	24.529
SE	28	04706	Nossa Senhora de Lourdes	6.469
SE	28	04805	Nossa Senhora do Socorro	153.253
SE	28	04458	Nossa Senhora Aparecida	8.770
SE	28	04904	Pacatuba	12.756
SE	28	05000	Pedra Mole	2.858
SE	28	05109	Pedrinhas	8.643
SE	28	05208	Pinhão	5.761

U.F.		MUNICÍPIOS		POPULAÇÃO ESTIMADA
SIGLA	COD	COD	NOMES	
SE	28	05307	Pirambu	8.488
SE	28	05406	Poço Redondo	29.879
SE	28	05505	Poço Verde	21.721
SE	28	05604	Porto da Folha	27.311
SE	28	05703	Propriá	28.437
SE	28	05802	Riachão do Dantas	19.567
SE	28	05901	Riachuelo	9.369
SE	28	06008	Ribeirópolis	16.137
SE	28	06107	Rosário do Catete	8.799
SE	28	06206	Salgado	19.095
SE	28	06305	Santa Luzia do Itanhy	13.430
SE	28	06503	Santa Rosa de Lima	3.962
SE	28	06404	Santana do São Francisco	6.799
SE	28	06602	Santo Amaro das Brotas	12.013
SE	28	06701	São Cristóvão	74.189
SE	28	06800	São Domingos	10.443
SE	28	06909	São Francisco	2.965
SE	28	07006	São Miguel do Aleixo	3.782
SE	28	07105	Simão Dias	38.232
SE	28	07204	Siriri	7.856
SE	28	07303	Telha	2.940
SE	28	07402	Tobias Barreto	48.733
SE	28	07501	Tomar do Geru	13.611
SE	28	07600	Umbaúba	21.195
BA	29	00108	Abaíra	8.851
BA	29	00207	Abaré	18.362
BA	29	00306	Acajutiba	15.123
BA	29	00355	Adustina	15.354
BA	29	00405	Água Fria	15.284
BA	29	00603	Aiquara	5.361
BA	29	00702	Alagoinhas	137.202
BA	29	00801	Alcobaça	20.320
BA	29	00900	Almadina	6.742
BA	29	01007	Amargosa	34.918
BA	29	01106	Amélia Rodrigues	24.192
BA	29	01155	América Dourada	16.723
BA	29	01205	Anagé	25.823
BA	29	01304	Andaraí	14.554
BA	29	01353	Andorinha	14.359
BA	29	01403	Angical	15.157
BA	29	01502	Anguera	9.707

U.F.		MUNICÍPIOS		POPULAÇÃO ESTIMADA
SIGLA	COD	COD	NOMES	
BA	29	01601	Antas	17.268
BA	29	01700	Antônio Cardoso	12.509
BA	29	01809	Antônio Gonçalves	11.107
BA	29	01908	Aporá	18.570
BA	29	01957	Apuarema	7.601
BA	29	02054	Araças	12.108
BA	29	02005	Aracatu	14.437
BA	29	02104	Araci	54.092
BA	29	02203	Aramari	9.858
BA	29	02252	Arataca	10.986
BA	29	02302	Aratuípe	8.788
BA	29	02401	Aurelino Leal	14.559
BA	29	02500	Baianópolis	14.019
BA	29	02609	Baixa Grande	21.704
BA	29	02658	Banzaê	11.166
BA	29	02708	Barra	49.705
BA	29	02807	Barra da Estiva	20.918
BA	29	02906	Barra do Choça	32.419
BA	29	03003	Barra do Mendes	14.458
BA	29	03102	Barra do Rocha	6.071
BA	29	03201	Barreiras	135.650
BA	29	03235	Barro Alto	13.990
BA	29	03300	Barro Preto	6.626
BA	29	03276	Barrocas	13.722
BA	29	03409	Belmonte	22.336
BA	29	03508	Belo Campo	15.427
BA	29	03607	Biritinga	14.307
BA	29	03706	Boa Nova	15.916
BA	29	03805	Boa Vista do Tupim	18.323
BA	29	03904	Bom Jesus da Lapa	65.148
BA	29	03953	Bom Jesus da Serra	10.588
BA	29	04001	Boninal	13.738
BA	29	04050	Bonito	14.205
BA	29	04100	Boquira	22.502
BA	29	04209	Botuporã	11.186
BA	29	04308	Brejões	12.726
BA	29	04407	Brejolândia	9.703
BA	29	04506	Brotas de Macaúbas	11.083
BA	29	04605	Brumado	64.417
BA	29	04704	Buerarema	20.687
BA	29	04753	Buritirama	19.349

U.F.		MUNICÍPIOS		POPULAÇÃO ESTIMADA
SIGLA	COD	COD	NOMES	
BA	29	04803	Caatiba	10.023
BA	29	04852	Cabaceiras do Paraguaçu	18.302
BA	29	04902	Cachoeira	33.495
BA	29	05008	Caculé	22.282
BA	29	05107	Caém	10.417
BA	29	05156	Caetanos	12.389
BA	29	05206	Caetité	47.774
BA	29	05305	Cafarnaum	18.119
BA	29	05404	Cairu	14.438
BA	29	05503	Caldeirão Grande	13.697
BA	29	05602	Camacan	31.133
BA	29	05701	Camaçari	227.955
BA	29	05800	Camamu	32.981
BA	29	05909	Campo Alegre de Lourdes	27.686
BA	29	06006	Campo Formoso	67.582
BA	29	06105	Canápolis	11.016
BA	29	06204	Canarana	25.557
BA	29	06303	Canavieiras	36.911
BA	29	06402	Candeal	9.157
BA	29	06501	Candeias	81.306
BA	29	06600	Candiba	12.766
BA	29	06709	Cândido Sales	27.327
BA	29	06808	Cansanção	33.920
BA	29	06824	Canudos	15.229
BA	29	06857	Capela do Alto Alegre	12.748
BA	29	06873	Capim Grosso	26.877
BA	29	06899	Caraíbas	10.659
BA	29	06907	Caravelas	21.945
BA	29	07004	Cardeal da Silva	8.570
BA	29	07103	Carinhanha	29.988
BA	29	07202	Casa Nova	65.747
BA	29	07301	Castro Alves	25.052
BA	29	07400	Catolândia	3.972
BA	29	07509	Catu	50.470
BA	29	07558	Caturama	8.749
BA	29	07608	Central	17.928
BA	29	07707	Chorrochó	10.952
BA	29	07806	Cícero Dantas	31.774
BA	29	07905	Cipó	15.634
BA	29	08002	Coaraci	22.812
BA	29	08101	Cocos	17.908

U.F.		MUNICÍPIOS		POPULAÇÃO ESTIMADA
SIGLA	COD	COD	NOMES	
BA	29	08200	Conceição da Feira	19.891
BA	29	08309	Conceição do Almeida	18.076
BA	29	08408	Conceição do Coité	63.318
BA	29	08507	Conceição do Jacuípe	28.552
BA	29	08606	Conde	22.929
BA	29	08705	Condeúba	17.000
BA	29	08804	Contendas do Sincorá	3.924
BA	29	08903	Coração de Maria	23.797
BA	29	09000	Cordeiros	8.826
BA	29	09109	Coribe	14.931
BA	29	09208	Coronel João Sá	18.540
BA	29	09307	Correntina	32.784
BA	29	09406	Cotegipe	14.127
BA	29	09505	Cravolândia	5.651
BA	29	09604	Crisópolis	20.179
BA	29	09703	Cristópolis	14.140
BA	29	09802	Cruz das Almas	56.766
BA	29	09901	Curaçá	33.929
BA	29	10008	Dário Meira	12.611
BA	29	10057	Dias d'Ávila	56.600
BA	29	10107	Dom Basílio	11.518
BA	29	10206	Dom Macedo Costa	3.936
BA	29	10305	Elísio Medrado	8.159
BA	29	10404	Encruzilhada	21.875
BA	29	10503	Entre Rios	40.276
BA	29	00504	Érico Cardoso	10.809
BA	29	10602	Esplanada	32.593
BA	29	10701	Euclides da Cunha	58.746
BA	29	10727	Eunápolis	98.194
BA	29	10750	Fátima	19.588
BA	29	10776	Feira da Mata	6.537
BA	29	10800	Feira de Santana	584.497
BA	29	10859	Filadélfia	16.319
BA	29	10909	Firmino Alves	5.804
BA	29	11006	Floresta Azul	10.489
BA	29	11105	Formosa do Rio Preto	21.827
BA	29	11204	Gandu	31.410
BA	29	11253	Gavião	4.537
BA	29	11303	Gentio do Ouro	11.829
BA	29	11402	Glória	14.223
BA	29	11501	Gongogi	6.581

U.F.		MUNICÍPIOS		POPULAÇÃO ESTIMADA
SIGLA	COD	COD	NOMES	
BA	29	11600	Governador Mangabeira	20.539
BA	29	11659	Guajeru	9.216
BA	29	11709	Guanambi	79.190
BA	29	11808	Guaratinga	23.105
BA	29	11857	Heliópolis	14.575
BA	29	11907	Iaçu	28.616
BA	29	12004	Ibiassucê	9.372
BA	29	12103	Ibicaraí	24.989
BA	29	12202	Ibicoara	16.643
BA	29	12301	Ibicuí	16.353
BA	29	12400	Ibipeba	17.466
BA	29	12509	Ibipitanga	14.327
BA	29	12608	Ibiquera	5.229
BA	29	12707	Ibirapitanga	24.052
BA	29	12806	Ibirapuã	7.825
BA	29	12905	Ibirataia	24.582
BA	29	13002	Ibitiara	16.471
BA	29	13101	Ibititá	19.286
BA	29	13200	Ibotirama	26.228
BA	29	13309	Ichu	6.101
BA	29	13408	Igaporã	14.944
BA	29	13457	Igrapiúna	13.436
BA	29	13507	Iguaí	29.070
BA	29	13606	Ilhéus	219.710
BA	29	13705	Inhambupe	36.084
BA	29	13804	Ipecaetá	15.694
BA	29	13903	Ipiaú	43.749
BA	29	14000	Ipirá	61.439
BA	29	14109	Ipupiara	9.259
BA	29	14208	Irajuba	7.550
BA	29	14307	Iramaia	15.291
BA	29	14406	Iraquara	23.867
BA	29	14505	Irará	25.771
BA	29	14604	Irecê	65.310
BA	29	14653	Itabela	26.636
BA	29	14703	Itaberaba	61.302
BA	29	14802	Itabuna	212.245
BA	29	14901	Itacaré	26.348
BA	29	15007	Itaeté	14.614
BA	29	15106	Itagi	14.217
BA	29	15205	Itagibá	16.659

U.F.		MUNICÍPIOS		POPULAÇÃO ESTIMADA
SIGLA	COD	COD	NOMES	
BA	29	15304	Itagimirim	7.181
BA	29	15353	Itaguaçu da Bahia	13.097
BA	29	15403	Itaju do Colônia	7.794
BA	29	15502	Itajuípe	20.695
BA	29	15601	Itamaraju	67.517
BA	29	15700	Itamari	8.747
BA	29	15809	Itambé	35.106
BA	29	15908	Itanagra	6.841
BA	29	16005	Itanhém	21.188
BA	29	16104	Itaparica	20.641
BA	29	16203	Itapé	11.013
BA	29	16302	Itapebi	11.931
BA	29	16401	Itapetinga	65.904
BA	29	16500	Itapicuru	32.100
BA	29	16609	Itapitanga	10.385
BA	29	16708	Itaquara	7.761
BA	29	16807	Itarantim	18.254
BA	29	16856	Itatim	15.266
BA	29	16906	Itiruçu	16.538
BA	29	17003	Itiúba	36.890
BA	29	17102	Itororó	20.841
BA	29	17201	Ituaçu	18.621
BA	29	17300	Ituberá	24.185
BA	29	17334	Iuiú	11.954
BA	29	17359	Jaborandi	9.032
BA	29	17409	Jacaraci	14.656
BA	29	17508	Jacobina	78.842
BA	29	17607	Jaguaquara	48.097
BA	29	17706	Jaguarari	30.222
BA	29	17805	Jaguaripe	17.074
BA	29	17904	Jandaíra	10.027
BA	29	18001	Jequié	150.351
BA	29	18100	Jeremoabo	38.924
BA	29	18209	Jiquiriçá	13.771
BA	29	18308	Jitaúna	16.816
BA	29	18357	João Dourado	21.726
BA	29	18407	Juazeiro	237.627
BA	29	18456	Jucuruçu	10.698
BA	29	18506	Jussara	15.229
BA	29	18555	Jussari	6.980
BA	29	18605	Jussiape	8.356

U.F.		MUNICÍPIOS		POPULAÇÃO ESTIMADA
SIGLA	COD	COD	NOMES	
BA	29	18704	Lafaiete Coutinho	3.561
BA	29	18753	Lagoa Real	14.359
BA	29	18803	Laje	21.957
BA	29	18902	Lajedão	3.585
BA	29	19009	Lajedinho	4.461
BA	29	19058	Lajedo do Tabocal	8.923
BA	29	19108	Lamarão	12.682
BA	29	19157	Lapão	26.461
BA	29	19207	Lauro de Freitas	153.016
BA	29	19306	Lençóis	10.008
BA	29	19405	Licínio de Almeida	13.135
BA	29	19504	Livramento de Nossa Senhora	43.994
BA	29	19553	Luís Eduardo Magalhães	48.977
BA	29	19603	Macajuba	11.522
BA	29	19702	Macarani	16.735
BA	29	19801	Macaúbas	47.991
BA	29	19900	Macururé	7.913
BA	29	19926	Madre de Deus	16.354
BA	29	19959	Maetinga	8.432
BA	29	20007	Maiquinique	8.713
BA	29	20106	Mairi	19.752
BA	29	20205	Malhada	16.647
BA	29	20304	Malhada de Pedras	7.753
BA	29	20403	Manoel Vitorino	14.389
BA	29	20452	Mansidão	12.142
BA	29	20502	Maracás	35.617
BA	29	20601	Maragogipe	43.620
BA	29	20700	Maraú	17.386
BA	29	20809	Marcionílio Souza	11.043
BA	29	20908	Mascote	16.557
BA	29	21005	Mata de São João	38.962
BA	29	21054	Matina	12.976
BA	29	21104	Medeiros Neto	22.629
BA	29	21203	Miguel Calmon	27.850
BA	29	21302	Milagres	12.100
BA	29	21401	Mirangaba	18.436
BA	29	21450	Mirante	8.876
BA	29	21500	Monte Santo	53.577
BA	29	21609	Morpará	8.853
BA	29	21708	Morro do Chapéu	35.009
BA	29	21807	Mortugaba	14.508

U.F.		MUNICÍPIOS		POPULAÇÃO ESTIMADA
SIGLA	COD	COD	NOMES	
BA	29	21906	Mucugê	14.629
BA	29	22003	Mucuri	34.836
BA	29	22052	Mulungu do Morro	14.006
BA	29	22102	Mundo Novo	24.895
BA	29	22201	Muniz Ferreira	7.214
BA	29	22250	Muquém de São Francisco	10.547
BA	29	22300	Muritiba	27.866
BA	29	22409	Mutuípe	21.935
BA	29	22508	Nazaré	27.350
BA	29	22607	Nilo Peçanha	13.092
BA	29	22656	Nordestina	12.599
BA	29	22706	Nova Canaã	19.866
BA	29	22730	Nova Fátima	7.930
BA	29	22755	Nova Ibiá	7.047
BA	29	22805	Nova Itarana	7.762
BA	29	22854	Nova Redenção	9.262
BA	29	22904	Nova Soure	26.666
BA	29	23001	Nova Viçosa	36.032
BA	29	23035	Novo Horizonte	10.860
BA	29	23050	Novo Triunfo	14.666
BA	29	23100	Olindina	24.516
BA	29	23209	Oliveira dos Brejinhos	23.436
BA	29	23308	Ouriçangas	8.081
BA	29	23357	Ourolândia	16.933
BA	29	23407	Palmas de Monte Alto	21.896
BA	29	23506	Palmeiras	8.358
BA	29	23605	Paramirim	20.756
BA	29	23704	Paratinga	29.693
BA	29	23803	Paripiranga	29.459
BA	29	23902	Pau Brasil	12.418
BA	29	24009	Paulo Afonso	105.837
BA	29	24058	Pé de Serra	14.683
BA	29	24108	Pedrão	7.525
BA	29	24207	Pedro Alexandre	17.643
BA	29	24306	Piatã	18.532
BA	29	24405	Pilão Arcado	34.144
BA	29	24504	Pindaí	15.759
BA	29	24603	Pindobaçu	20.873
BA	29	24652	Pintadas	10.831
BA	29	24678	Piraí do Norte	8.975
BA	29	24702	Piripá	13.597

U.F.		MUNICÍPIOS		POPULAÇÃO ESTIMADA
SIGLA	COD	COD	NOMES	
BA	29	24801	Piritiba	25.612
BA	29	24900	Planaltino	8.802
BA	29	25006	Planalto	22.127
BA	29	25105	Poções	46.224
BA	29	25204	Pojuca	31.687
BA	29	25253	Ponto Novo	14.974
BA	29	25303	Porto Seguro	120.460
BA	29	25402	Potiraguá	10.123
BA	29	25501	Prado	26.082
BA	29	25600	Presidente Dutra	14.264
BA	29	25709	Presidente Jânio Quadros	14.039
BA	29	25758	Presidente Tancredo Neves	23.817
BA	29	25808	Queimadas	28.368
BA	29	25907	Quijingue	28.001
BA	29	25931	Quixabeira	9.624
BA	29	25956	Rafael Jambeiro	23.893
BA	29	26004	Remanso	39.415
BA	29	26103	Retirolândia	12.446
BA	29	26202	Riachão das Neves	23.309
BA	29	26301	Riachão do Jacuípe	33.326
BA	29	26400	Riacho de Santana	30.444
BA	29	26509	Ribeira do Amparo	14.566
BA	29	26608	Ribeira do Pombal	49.024
BA	29	26657	Ribeirão do Largo	14.612
BA	29	26707	Rio de Contas	13.823
BA	29	26806	Rio do Antônio	14.926
BA	29	26905	Rio do Pires	11.659
BA	29	27002	Rio Real	37.675
BA	29	27101	Rodelas	7.341
BA	29	27200	Ruy Barbosa	30.316
BA	29	27309	Salinas da Margarida	13.850
BA	29	27408	Salvador	2.948.733
BA	29	27507	Santa Bárbara	20.242
BA	29	27606	Santa Brígida	15.781
BA	29	27804	Santa Cruz da Vitória	6.502
BA	29	27705	Santa Cruz Cabrália	26.051
BA	29	27903	Santa Inês	10.833
BA	29	28059	Santa Luzia	15.615
BA	29	28109	Santa Maria da Vitória	41.745
BA	29	28406	Santa Rita de Cássia	27.224
BA	29	28505	Santa Teresinha	10.382

U.F.		MUNICÍPIOS		POPULAÇÃO ESTIMADA
SIGLA	COD	COD	NOMES	
BA	29	28000	Santaluz	35.029
BA	29	28208	Santana	26.990
BA	29	28307	Santanópolis	9.558
BA	29	28604	Santo Amaro	57.675
BA	29	28703	Santo Antônio de Jesus	87.776
BA	29	28802	Santo Estêvão	46.360
BA	29	28901	São Desidério	26.742
BA	29	28950	São Domingos	9.130
BA	29	29107	São Felipe	20.902
BA	29	29008	São Félix	15.987
BA	29	29057	São Félix do Coribe	13.352
BA	29	29206	São Francisco do Conde	31.219
BA	29	29255	São Gabriel	19.050
BA	29	29305	São Gonçalo dos Campos	30.401
BA	29	29354	São José da Vitória	6.270
BA	29	29370	São José do Jacuípe	10.965
BA	29	29404	São Miguel das Matas	10.623
BA	29	29503	São Sebastião do Passé	41.624
BA	29	29602	Sapeaçu	17.041
BA	29	29701	Sátiro Dias	18.779
BA	29	29750	Saubara	11.507
BA	29	29800	Saúde	12.284
BA	29	29909	Seabra	41.952
BA	29	30006	Sebastião Laranjeiras	11.231
BA	29	30105	Senhor do Bonfim	75.393
BA	29	30204	Sento Sé	38.181
BA	29	30154	Serra do Ramalho	31.909
BA	29	30303	Serra Dourada	17.887
BA	29	30402	Serra Preta	15.971
BA	29	30501	Serrinha	73.655
BA	29	30600	Serrolândia	12.572
BA	29	30709	Simões Filho	114.649
BA	29	30758	Sítio do Mato	13.064
BA	29	30766	Sítio do Quinto	14.122
BA	29	30774	Sobradinho	21.978
BA	29	30808	Souto Soares	18.992
BA	29	30907	Tabocas do Brejo Velho	12.620
BA	29	31004	Tanhaçu	20.142
BA	29	31053	Tanque Novo	16.338
BA	29	31103	Tanquinho	7.842
BA	29	31202	Taperoá	19.081

U.F.		MUNICÍPIOS		POPULAÇÃO ESTIMADA
SIGLA	COD	COD	NOMES	
BA	29	31301	Tapiramutá	17.817
BA	29	31350	Teixeira de Freitas	123.858
BA	29	31400	Teodoro Sampaio	8.490
BA	29	31509	Teofilândia	21.382
BA	29	31608	Teolândia	12.798
BA	29	31707	Terra Nova	12.620
BA	29	31806	Tremedal	18.706
BA	29	31905	Tucano	49.972
BA	29	32002	Uauá	25.257
BA	29	32101	Ubaíra	21.368
BA	29	32200	Ubaitaba	20.678
BA	29	32309	Ubatã	25.952
BA	29	32408	Uibaí	14.160
BA	29	32457	Umburanas	16.834
BA	29	32507	Una	25.287
BA	29	32606	Urandi	16.282
BA	29	32705	Uruçuca	22.984
BA	29	32804	Utinga	20.132
BA	29	32903	Valença	88.542
BA	29	33000	Valente	22.489
BA	29	33059	Várzea da Roça	15.040
BA	29	33109	Várzea do Poço	8.908
BA	29	33158	Várzea Nova	14.357
BA	29	33174	Varzedo	9.386
BA	29	33208	Vera Cruz	36.843
BA	29	33257	Vereda	7.361
BA	29	33307	Vitória da Conquista	313.898
BA	29	33406	Wagner	8.830
BA	29	33455	Wanderley	13.300
BA	29	33505	Wenceslau Guimarães	24.740
BA	29	33604	Xique-Xique	47.251
MG	31	00104	Abadia dos Dourados	6.777
MG	31	00203	Abaeté	23.193
MG	31	00302	Abre Campo	13.204
MG	31	00401	Acaiaca	4.206
MG	31	00500	Açucena	11.425
MG	31	00609	Água Boa	16.764
MG	31	00708	Água Comprida	2.159
MG	31	00807	Aguanil	4.248
MG	31	00906	Águas Formosas	19.189
MG	31	01003	Águas Vermelhas	13.179

U.F.		MUNICÍPIOS		POPULAÇÃO ESTIMADA
SIGLA	COD	COD	NOMES	
MG	31	01102	Aimorés	24.871
MG	31	01201	Aiuruoca	6.239
MG	31	01300	Alagoa	2.917
MG	31	01409	Albertina	2.966
MG	31	01508	Além Paraíba	34.527
MG	31	01607	Alfenas	74.505
MG	31	01631	Alfredo Vasconcelos	6.194
MG	31	01706	Almenara	38.269
MG	31	01805	Alpercata	7.231
MG	31	01904	Alpinópolis	18.486
MG	31	02001	Alterosa	13.744
MG	31	02050	Alto Caparaó	5.257
MG	31	53509	Alto Jequitibá	8.160
MG	31	02100	Alto Rio Doce	12.889
MG	31	02209	Alvarenga	4.611
MG	31	02308	Alvinópolis	15.682
MG	31	02407	Alvorada de Minas	3.585
MG	31	02506	Amparo do Serra	5.377
MG	31	02605	Andradas	36.320
MG	31	02803	Andrelândia	12.374
MG	31	02852	Angelândia	8.475
MG	31	02902	Antônio Carlos	11.563
MG	31	03009	Antônio Dias	9.647
MG	31	03108	Antônio Prado de Minas	2.046
MG	31	03207	Araçaí	2.491
MG	31	03306	Aracitaba	1.905
MG	31	03405	Araçuaí	37.262
MG	31	03504	Araguari	110.334
MG	31	03603	Arantina	2.575
MG	31	03702	Araponga	8.296
MG	31	03751	Araporã	6.414
MG	31	03801	Arapuá	2.777
MG	31	03900	Araújos	7.560
MG	31	04007	Araxá	91.703
MG	31	04106	Arceburgo	8.239
MG	31	04205	Arcos	36.133
MG	31	04304	Areado	13.723
MG	31	04403	Argirita	3.065
MG	31	04452	Aricanduva	5.062
MG	31	04502	Arinos	18.126
MG	31	04601	Astolfo Dutra	12.998

U.F.		MUNICÍPIOS		POPULAÇÃO ESTIMADA
SIGLA	COD	COD	NOMES	
MG	31	04700	Ataléia	15.322
MG	31	04809	Augusto de Lima	4.655
MG	31	04908	Baependi	18.646
MG	31	05004	Baldim	8.549
MG	31	05103	Bambuí	22.554
MG	31	05202	Bandeira	5.507
MG	31	05301	Bandeira do Sul	5.294
MG	31	05400	Barão de Cocais	27.660
MG	31	05509	Barão de Monte Alto	5.755
MG	31	05608	Barbacena	127.328
MG	31	05707	Barra Longa	7.102
MG	31	05905	Barroso	20.093
MG	31	06002	Bela Vista de Minas	10.296
MG	31	06101	Belmiro Braga	3.114
MG	31	06200	Belo Horizonte	2.434.642
MG	31	06309	Belo Oriente	22.289
MG	31	06408	Belo Vale	7.472
MG	31	06507	Berilo	13.659
MG	31	06655	Berizal	4.595
MG	31	06606	Bertópolis	4.752
MG	31	06705	Betim	429.507
MG	31	06804	Bias Fortes	3.932
MG	31	06903	Bicas	14.180
MG	31	07000	Biquinhas	2.642
MG	31	07109	Boa Esperança	39.082
MG	31	07208	Bocaina de Minas	5.198
MG	31	07307	Bocaiúva	46.306
MG	31	07406	Bom Despacho	43.898
MG	31	07505	Bom Jardim de Minas	6.661
MG	31	07604	Bom Jesus da Penha	3.941
MG	31	07703	Bom Jesus do Amparo	5.663
MG	31	07802	Bom Jesus do Galho	15.541
MG	31	07901	Bom Repouso	10.806
MG	31	08008	Bom Sucesso	17.750
MG	31	08107	Bonfim	6.904
MG	31	08206	Bonfinópolis de Minas	5.927
MG	31	08255	Bonito de Minas	9.187
MG	31	08305	Borda da Mata	15.420
MG	31	08404	Botelhos	15.284
MG	31	08503	Botumirim	6.582
MG	31	08701	Brás Pires	4.665

U.F.		MUNICÍPIOS		POPULAÇÃO ESTIMADA
SIGLA	COD	COD	NOMES	
MG	31	08552	Brasilândia de Minas	13.406
MG	31	08602	Brasília de Minas	32.262
MG	31	08909	Brasópolis	14.807
MG	31	08800	Braúnas	5.344
MG	31	09006	Brumadinho	33.693
MG	31	09105	Bueno Brandão	11.195
MG	31	09204	Buenópolis	9.704
MG	31	09253	Bugre	4.085
MG	31	09303	Buritis	22.290
MG	31	09402	Buritizeiro	26.981
MG	31	09451	Cabeceira Grande	6.542
MG	31	09501	Cabo Verde	14.024
MG	31	09600	Cachoeira da Prata	3.924
MG	31	09709	Cachoeira de Minas	11.194
MG	31	02704	Cachoeira de Pajeú	9.450
MG	31	09808	Cachoeira Dourada	2.570
MG	31	09907	Caetanópolis	9.911
MG	31	10004	Caeté	40.634
MG	31	10103	Caiana	4.702
MG	31	10202	Cajuri	4.117
MG	31	10301	Caldas	14.491
MG	31	10400	Camacho	3.260
MG	31	10509	Camanducaia	20.212
MG	31	10608	Cambuí	26.070
MG	31	10707	Cambuquira	12.909
MG	31	10806	Campanário	3.728
MG	31	10905	Campanha	15.790
MG	31	11002	Campestre	20.843
MG	31	11101	Campina Verde	19.207
MG	31	11309	Campo do Meio	11.840
MG	31	11150	Campo Azul	3.982
MG	31	11200	Campo Belo	53.280
MG	31	11408	Campo Florido	6.945
MG	31	11507	Campos Altos	13.646
MG	31	11606	Campos Gerais	27.853
MG	31	11903	Cana Verde	5.897
MG	31	11705	Canaã	4.798
MG	31	11804	Canápolis	11.760
MG	31	12000	Candeias	16.125
MG	31	12059	Cantagalo	4.108
MG	31	12109	Caparaó	4.996

U.F.		MUNICÍPIOS		POPULAÇÃO ESTIMADA
SIGLA	COD	COD	NOMES	
MG	31	12208	Capela Nova	4.693
MG	31	12307	Capelinha	34.345
MG	31	12406	Capetinga	7.341
MG	31	12505	Capim Branco	9.155
MG	31	12604	Capinópolis	15.903
MG	31	12653	Capitão Andrade	5.018
MG	31	12703	Capitão Enéas	14.682
MG	31	12802	Capitólio	7.858
MG	31	12901	Caputira	9.135
MG	31	13008	Caraí	22.278
MG	31	13107	Caranaíba	3.549
MG	31	13206	Carandaí	23.097
MG	31	13305	Carangola	33.091
MG	31	13404	Caratinga	84.825
MG	31	13503	Carbonita	10.623
MG	31	13602	Careaçu	6.247
MG	31	13701	Carlos Chagas	21.302
MG	31	13800	Carmésia	2.671
MG	31	13909	Carmo da Cachoeira	12.028
MG	31	14006	Carmo da Mata	11.358
MG	31	14105	Carmo de Minas	14.236
MG	31	14204	Carmo do Cajuru	19.779
MG	31	14303	Carmo do Paranaíba	31.843
MG	31	14402	Carmo do Rio Claro	20.055
MG	31	14501	Carmópolis de Minas	16.425
MG	31	14550	Carneirinho	9.129
MG	31	14600	Carrancas	4.158
MG	31	14709	Carvalhópolis	3.355
MG	31	14808	Carvalhos	4.739
MG	31	14907	Casa Grande	2.143
MG	31	15003	Cascalho Rico	2.911
MG	31	15102	Cássia	17.572
MG	31	15300	Cataguases	69.955
MG	31	15359	Catas Altas	4.747
MG	31	15409	Catas Altas da Noruega	3.550
MG	31	15458	Catuji	6.704
MG	31	15474	Catuti	5.465
MG	31	15508	Caxambu	21.514
MG	31	15607	Cedro do Abaeté	1.228
MG	31	15706	Central de Minas	6.801
MG	31	15805	Centralina	10.536

U.F.		MUNICÍPIOS		POPULAÇÃO ESTIMADA
SIGLA	COD	COD	NOMES	
MG	31	15904	Chácara	2.728
MG	31	16001	Chalé	5.609
MG	31	16100	Chapada do Norte	15.963
MG	31	16159	Chapada Gaúcha	10.995
MG	31	16209	Chiador	2.975
MG	31	16308	Cipotânea	6.770
MG	31	16407	Claraval	4.437
MG	31	16506	Claro dos Poções	8.377
MG	31	16605	Cláudio	25.640
MG	31	16704	Coimbra	7.151
MG	31	16803	Coluna	9.559
MG	31	16902	Comendador Gomes	3.217
MG	31	17009	Comercinho	8.790
MG	31	17108	Conceição da Aparecida	10.649
MG	31	15201	Conceição da Barra de Minas	4.075
MG	31	17306	Conceição das Alagoas	21.510
MG	31	17207	Conceição das Pedras	2.813
MG	31	17405	Conceição de Ipanema	4.536
MG	31	17504	Conceição do Mato Dentro	18.558
MG	31	17603	Conceição do Pará	4.863
MG	31	17702	Conceição do Rio Verde	13.164
MG	31	17801	Conceição dos Ouros	10.697
MG	31	17836	Cônego Marinho	6.449
MG	31	17876	Confins	5.966
MG	31	17900	Congonhal	10.127
MG	31	18007	Congonhas	48.066
MG	31	18106	Congonhas do Norte	5.299
MG	31	18205	Conquista	6.851
MG	31	18304	Conselheiro Lafaiete	113.576
MG	31	18403	Conselheiro Pena	22.482
MG	31	18502	Consolação	1.747
MG	31	18601	Contagem	617.749
MG	31	18700	Coqueiral	9.743
MG	31	18809	Coração de Jesus	27.003
MG	31	18908	Cordisburgo	9.385
MG	31	19005	Cordislândia	3.710
MG	31	19104	Corinto	23.206
MG	31	19203	Coroaci	11.109
MG	31	19302	Coromandel	28.240
MG	31	19401	Coronel Fabriciano	104.415
MG	31	19500	Coronel Murta	9.404

U.F.		MUNICÍPIOS		POPULAÇÃO ESTIMADA
SIGLA	COD	COD	NOMES	
MG	31	19609	Coronel Pacheco	2.473
MG	31	19708	Coronel Xavier Chaves	3.295
MG	31	19906	Córrego do Bom Jesus	3.827
MG	31	19807	Córrego Danta	3.496
MG	31	19955	Córrego Fundo	5.873
MG	31	20003	Córrego Novo	3.188
MG	31	20102	Couto de Magalhães de Minas	4.512
MG	31	20151	Crisólita	5.885
MG	31	20201	Cristais	11.115
MG	31	20300	Cristália	5.930
MG	31	20409	Cristiano Otoni	5.031
MG	31	20508	Cristina	11.381
MG	31	20607	Crucilândia	4.752
MG	31	20706	Cruzeiro da Fortaleza	3.884
MG	31	20805	Cruzília	15.236
MG	31	20839	Cuparaque	4.547
MG	31	20870	Curral de Dentro	7.251
MG	31	20904	Curvelo	74.409
MG	31	21001	Datas	5.639
MG	31	21100	Delfim Moreira	8.052
MG	31	21209	Delfinópolis	6.924
MG	31	21258	Delta	7.016
MG	31	21308	Descoberto	5.075
MG	31	21407	Desterro de Entre Rios	7.145
MG	31	21506	Desterro do Melo	3.296
MG	31	21605	Diamantina	46.212
MG	31	21704	Diogo de Vasconcelos	4.060
MG	31	21803	Dionísio	10.560
MG	31	21902	Divinésia	3.391
MG	31	22009	Divino	19.959
MG	31	22108	Divino das Laranjeiras	5.084
MG	31	22207	Divinolândia de Minas	6.973
MG	31	22306	Divinópolis	213.277
MG	31	22355	Divisa Alegre	6.108
MG	31	22405	Divisa Nova	5.806
MG	31	22454	Divisópolis	8.285
MG	31	22470	Dom Bosco	3.862
MG	31	22504	Dom Cavati	5.784
MG	31	22603	Dom Joaquim	4.649
MG	31	22702	Dom Silvério	5.457
MG	31	22801	Dom Viçoso	3.112

U.F.		MUNICÍPIOS		POPULAÇÃO ESTIMADA
SIGLA	COD	COD	NOMES	
MG	31	22900	Dona Eusébia	5.771
MG	31	23007	Dores de Campos	9.692
MG	31	23106	Dores de Guanhães	5.721
MG	31	23205	Dores do Indaiá	14.380
MG	31	23304	Dores do Turvo	4.684
MG	31	23403	Doresópolis	1.558
MG	31	23502	Douradoquara	1.912
MG	31	23528	Durandé	7.139
MG	31	23601	Elói Mendes	25.218
MG	31	23700	Engenheiro Caldas	10.772
MG	31	23809	Engenheiro Navarro	7.299
MG	31	23858	Entre Folhas	5.068
MG	31	23908	Entre Rios de Minas	14.426
MG	31	24005	Ervália	18.699
MG	31	24104	Esmeraldas	58.307
MG	31	24203	Espera Feliz	21.529
MG	31	24302	Espinosa	32.349
MG	31	24401	Espírito Santo do Dourado	4.445
MG	31	24500	Estiva	11.337
MG	31	24708	Estrela do Indaiá	3.772
MG	31	24807	Estrela do Sul	7.394
MG	31	24609	Estrela Dalva	2.551
MG	31	24906	Eugenópolis	10.684
MG	31	25002	Ewbank da Câmara	3.673
MG	31	25101	Extrema	26.436
MG	31	25200	Fama	2.270
MG	31	25309	Faria Lemos	3.725
MG	31	25408	Felício dos Santos	5.857
MG	31	25606	Felisburgo	6.957
MG	31	25705	Felixlândia	14.158
MG	31	25804	Fernandes Tourinho	2.701
MG	31	25903	Ferros	11.615
MG	31	25952	Fervedouro	10.662
MG	31	26000	Florestal	6.152
MG	31	26109	Formiga	66.834
MG	31	26208	Formoso	6.831
MG	31	26307	Fortaleza de Minas	3.968
MG	31	26406	Fortuna de Minas	2.533
MG	31	26505	Francisco Badaró	10.585
MG	31	26604	Francisco Dumont	4.945
MG	31	26703	Francisco Sá	25.788

U.F.		MUNICÍPIOS		POPULAÇÃO ESTIMADA
SIGLA	COD	COD	NOMES	
MG	31	26752	Franciscópolis	5.737
MG	31	26802	Frei Gaspar	6.591
MG	31	26901	Frei Inocêncio	9.246
MG	31	26950	Frei Lagonegro	3.468
MG	31	27008	Fronteira	15.096
MG	31	27057	Fronteira dos Vales	4.977
MG	31	27073	Fruta de Leite	6.464
MG	31	27107	Frutal	54.094
MG	31	27206	Funilândia	3.801
MG	31	27305	Galiléia	7.539
MG	31	27339	Gameleiras	5.384
MG	31	27354	Glaucilândia	3.046
MG	31	27370	Goiabeira	3.193
MG	31	27388	Goianá	3.800
MG	31	27404	Gonçalves	4.423
MG	31	27503	Gonzaga	5.783
MG	31	27602	Gouveia	11.915
MG	31	27701	Governador Valadares	261.981
MG	31	27800	Grão Mogol	15.100
MG	31	27909	Grupiara	1.461
MG	31	28006	Guanhães	30.401
MG	31	28105	Guapé	13.500
MG	31	28204	Guaraciaba	10.776
MG	31	28253	Guaraciama	4.708
MG	31	28303	Guaranésia	18.649
MG	31	28402	Guarani	9.915
MG	31	28501	Guarará	4.123
MG	31	28600	Guarda-Mor	6.772
MG	31	28709	Guaxupé	49.509
MG	31	28808	Guidoval	7.526
MG	31	28907	Guimarânia	7.240
MG	31	29004	Guiricema	9.137
MG	31	29103	Gurinhatã	6.294
MG	31	29202	Heliodora	6.241
MG	31	29301	Iapu	11.344
MG	31	29400	Ibertioga	5.204
MG	31	29509	Ibiá	22.899
MG	31	29608	Ibiaí	7.852
MG	31	29657	Ibiracatu	5.996
MG	31	29707	Ibiraci	11.476
MG	31	29806	Ibirité	155.290

U.F.		MUNICÍPIOS		POPULAÇÃO ESTIMADA
SIGLA	COD	COD	NOMES	
MG	31	29905	Ibitiúra de Minas	3.499
MG	31	30002	Ibituruna	2.923
MG	31	30051	Icaraí de Minas	10.792
MG	31	30101	Igarapé	32.967
MG	31	30200	Igaratinga	8.894
MG	31	30309	Iguatama	7.784
MG	31	30408	Ijaci	5.950
MG	31	30507	Ilicínea	11.717
MG	31	30556	Imbé de Minas	6.578
MG	31	30606	Inconfidentes	7.585
MG	31	30655	Indaiabira	7.724
MG	31	30705	Indianópolis	6.556
MG	31	30804	Ingaí	2.574
MG	31	30903	Inhapim	24.967
MG	31	31000	Inhaúma	5.535
MG	31	31109	Inimutaba	6.663
MG	31	31158	Ipaba	15.351
MG	31	31208	Ipanema	17.778
MG	31	31307	Ipatinga	241.720
MG	31	31406	Ipiaçu	4.345
MG	31	31505	Ipuiúna	9.501
MG	31	31604	Iraí de Minas	6.545
MG	31	31703	Itabira	109.380
MG	31	31802	Itabirinha	10.734
MG	31	31901	Itabirito	43.314
MG	31	32008	Itacambira	5.238
MG	31	32107	Itacarambi	18.200
MG	31	32206	Itaguara	12.812
MG	31	32305	Itaipé	11.958
MG	31	32404	Itajubá	89.730
MG	31	32503	Itamarandiba	33.219
MG	31	32602	Itamarati de Minas	4.194
MG	31	32701	Itambacuri	23.338
MG	31	32800	Itambé do Mato Dentro	2.490
MG	31	32909	Itamogi	11.181
MG	31	33006	Itamonte	14.398
MG	31	33105	Itanhandu	15.047
MG	31	33204	Itanhomi	12.294
MG	31	33303	Itaobim	21.603
MG	31	33402	Itapagipe	14.755
MG	31	33501	Itapecerica	21.220

U.F.		MUNICÍPIOS		POPULAÇÃO ESTIMADA
SIGLA	COD	COD	NOMES	
MG	31	33600	Itapeva	8.013
MG	31	33709	Itatiaiuçu	9.292
MG	31	33758	Itaú de Minas	15.123
MG	31	33808	Itaúna	85.070
MG	31	33907	Itaverava	5.813
MG	31	34004	Itinga	15.137
MG	31	34103	Itueta	6.038
MG	31	34202	Ituiutaba	96.122
MG	31	34301	Itumirim	6.647
MG	31	34400	Iturama	32.845
MG	31	34509	Itutinga	4.166
MG	31	34608	Jaboticatubas	16.248
MG	31	34707	Jacinto	12.856
MG	31	34806	Jacuí	7.429
MG	31	34905	Jacutinga	21.215
MG	31	35001	Jaguaraçu	2.860
MG	31	35050	Jaíba	31.758
MG	31	35076	Jampruca	5.109
MG	31	35100	Janaúba	67.941
MG	31	35209	Januária	67.206
MG	31	35308	Japaraíba	3.832
MG	31	35357	Japonvar	8.504
MG	31	35407	Jeceaba	6.047
MG	31	35456	Jenipapo de Minas	7.178
MG	31	35506	Jequeri	13.276
MG	31	35605	Jequitaí	8.182
MG	31	35704	Jequitibá	5.706
MG	31	35803	Jequitinhonha	24.879
MG	31	35902	Jesuânia	4.972
MG	31	36009	Joaíma	15.391
MG	31	36108	Joanésia	5.669
MG	31	36207	João Monlevade	74.576
MG	31	36306	João Pinheiro	44.834
MG	31	36405	Joaquim Felício	4.069
MG	31	36504	Jordânia	11.209
MG	31	36520	José Gonçalves de Minas	4.669
MG	31	36553	José Raydan	4.344
MG	31	36579	Josenópolis	4.604
MG	31	36652	Juatuba	20.567
MG	31	36702	Juiz de Fora	520.612
MG	31	36801	Juramento	4.092

U.F.		MUNICÍPIOS		POPULAÇÃO ESTIMADA
SIGLA	COD	COD	NOMES	
MG	31	36900	Juruaia	8.597
MG	31	36959	Juvenília	6.276
MG	31	37007	Ladainha	17.082
MG	31	37106	Lagamar	7.864
MG	31	37205	Lagoa da Prata	46.276
MG	31	37304	Lagoa dos Patos	4.586
MG	31	37403	Lagoa Dourada	12.202
MG	31	37502	Lagoa Formosa	17.069
MG	31	37536	Lagoa Grande	9.074
MG	31	37601	Lagoa Santa	47.287
MG	31	37700	Lajinha	17.865
MG	31	37809	Lambari	19.167
MG	31	37908	Lamim	3.651
MG	31	38005	Laranjal	6.532
MG	31	38104	Lassance	6.647
MG	31	38203	Lavras	91.333
MG	31	38302	Leandro Ferreira	3.011
MG	31	38351	Leme do Prado	5.111
MG	31	38401	Leopoldina	51.452
MG	31	38500	Liberdade	5.438
MG	31	38609	Lima Duarte	16.434
MG	31	38625	Limeira do Oeste	6.739
MG	31	38658	Lontra	8.274
MG	31	38674	Luisburgo	6.470
MG	31	38682	Luislândia	6.676
MG	31	38708	Luminárias	5.528
MG	31	38807	Luz	17.756
MG	31	38906	Machacalis	7.061
MG	31	39003	Machado	39.109
MG	31	39102	Madre de Deus de Minas	5.136
MG	31	39201	Malacacheta	18.296
MG	31	39250	Mamonas	6.458
MG	31	39300	Manga	21.413
MG	31	39409	Manhuaçu	77.598
MG	31	39508	Manhumirim	20.866
MG	31	39607	Mantena	27.536
MG	31	39805	Mar de Espanha	11.566
MG	31	39706	Maravilhas	7.137
MG	31	39904	Maria da Fé	14.646
MG	31	40001	Mariana	53.989
MG	31	40100	Marilac	4.401

U.F.		MUNICÍPIOS		POPULAÇÃO ESTIMADA
SIGLA	COD	COD	NOMES	
MG	31	40159	Mário Campos	11.899
MG	31	40209	Maripá de Minas	2.947
MG	31	40308	Marliéria	3.819
MG	31	40407	Marmelópolis	3.171
MG	31	40506	Martinho Campos	12.593
MG	31	40530	Martins Soares	6.625
MG	31	40555	Mata Verde	7.742
MG	31	40605	Materlândia	4.782
MG	31	40704	Mateus Leme	26.631
MG	31	71501	Mathias Lobato	3.540
MG	31	40803	Matias Barbosa	13.738
MG	31	40852	Matias Cardoso	10.819
MG	31	40902	Matipó	16.963
MG	31	41009	Mato Verde	12.989
MG	31	41108	Matozinhos	34.789
MG	31	41207	Matutina	3.797
MG	31	41306	Medeiros	3.367
MG	31	41405	Medina	21.181
MG	31	41504	Mendes Pimentel	6.652
MG	31	41603	Mercês	10.832
MG	31	41702	Mesquita	6.659
MG	31	41801	Minas Novas	31.557
MG	31	41900	Minduri	3.684
MG	31	42007	Mirabela	13.198
MG	31	42106	Miradouro	10.575
MG	31	42205	Miraí	13.418
MG	31	42254	Miravânia	4.927
MG	31	42304	Moeda	4.652
MG	31	42403	Moema	6.998
MG	31	42502	Monjolos	2.338
MG	31	42601	Monsenhor Paulo	7.591
MG	31	42700	Montalvânia	16.265
MG	31	42809	Monte Alegre de Minas	18.969
MG	31	42908	Monte Azul	22.949
MG	31	43005	Monte Belo	12.889
MG	31	43104	Monte Carmelo	45.819
MG	31	43153	Monte Formoso	4.897
MG	31	43203	Monte Santo de Minas	20.616
MG	31	43401	Monte Sião	19.970
MG	31	43302	Montes Claros	358.271
MG	31	43450	Montezuma	7.580

U.F.		MUNICÍPIOS		POPULAÇÃO ESTIMADA
SIGLA	COD	COD	NOMES	
MG	31	43500	Morada Nova de Minas	8.650
MG	31	43609	Morro da Garça	2.968
MG	31	43708	Morro do Pilar	3.547
MG	31	43807	Munhoz	6.446
MG	31	43906	Muriaé	99.006
MG	31	44003	Mutum	27.105
MG	31	44102	Muzambinho	20.458
MG	31	44201	Nacip Raydan	3.028
MG	31	44300	Nanuque	41.389
MG	31	44359	Naque	6.107
MG	31	44375	Natalândia	3.371
MG	31	44409	Natércia	4.764
MG	31	44508	Nazareno	8.023
MG	31	44607	Nepomuceno	25.141
MG	31	44656	Ninheira	10.884
MG	31	44672	Nova Belém	3.625
MG	31	44706	Nova Era	18.517
MG	31	44805	Nova Lima	75.530
MG	31	44904	Nova Módica	3.969
MG	31	45000	Nova Ponte	12.233
MG	31	45059	Nova Porteirinha	7.583
MG	31	45109	Nova Resende	14.623
MG	31	45208	Nova Serrana	65.176
MG	31	36603	Nova União	5.636
MG	31	45307	Novo Cruzeiro	31.264
MG	31	45356	Novo Oriente de Minas	10.698
MG	31	45372	Novorizonte	5.092
MG	31	45406	Olaria	2.465
MG	31	45455	Olhos-d'Água	5.244
MG	31	45505	Olímpio Noronha	2.619
MG	31	45604	Oliveira	39.063
MG	31	45703	Oliveira Fortes	1.971
MG	31	45802	Onça de Pitangui	3.118
MG	31	45851	Oratórios	4.525
MG	31	45877	Orizânia	7.026
MG	31	45901	Ouro Branco	35.029
MG	31	46008	Ouro Fino	32.365
MG	31	46107	Ouro Preto	69.251
MG	31	46206	Ouro Verde de Minas	7.128
MG	31	46255	Padre Carvalho	6.092
MG	31	46305	Padre Paraíso	18.774

U.F.		MUNICÍPIOS		POPULAÇÃO ESTIMADA
SIGLA	COD	COD	NOMES	
MG	31	46552	Pai Pedro	6.186
MG	31	46404	Paineiras	4.696
MG	31	46503	Pains	8.420
MG	31	46602	Paiva	1.683
MG	31	46701	Palma	6.249
MG	31	46750	Palmópolis	7.010
MG	31	46909	Papagaios	15.124
MG	31	47105	Pará de Minas	83.282
MG	31	47006	Paracatu	82.850
MG	31	47204	Paraguaçu	20.307
MG	31	47303	Paraisópolis	18.734
MG	31	47402	Paraopeba	23.146
MG	31	47600	Passa Quatro	15.821
MG	31	47709	Passa Tempo	8.762
MG	31	47808	Passa-Vinte	2.136
MG	31	47501	Passabém	1.838
MG	31	47907	Passos	106.735
MG	31	47956	Patis	5.538
MG	31	48004	Patos de Minas	138.466
MG	31	48103	Patrocínio	85.293
MG	31	48202	Patrocínio do Muriaé	5.548
MG	31	48301	Paula Cândido	9.377
MG	31	48400	Paulistas	5.016
MG	31	48509	Pavão	9.140
MG	31	48608	Peçanha	17.691
MG	31	48806	Pedra do Anta	3.752
MG	31	48905	Pedra do Indaiá	4.058
MG	31	48707	Pedra Azul	25.798
MG	31	48756	Pedra Bonita	6.709
MG	31	49002	Pedra Dourada	2.203
MG	31	49101	Pedralva	11.422
MG	31	49150	Pedras de Maria da Cruz	11.606
MG	31	49200	Pedrinópolis	3.568
MG	31	49309	Pedro Leopoldo	58.635
MG	31	49408	Pedro Teixeira	1.692
MG	31	49507	Pequeri	3.089
MG	31	49606	Pequi	4.434
MG	31	49705	Perdigão	7.757
MG	31	49804	Perdizes	14.572
MG	31	49903	Perdões	20.105
MG	31	49952	Periquito	7.193

U.F.		MUNICÍPIOS		POPULAÇÃO ESTIMADA
SIGLA	COD	COD	NOMES	
MG	31	50000	Pescador	4.185
MG	31	50109	Piau	3.061
MG	31	50158	Piedade de Caratinga	6.795
MG	31	50208	Piedade de Ponte Nova	4.254
MG	31	50307	Piedade do Rio Grande	4.892
MG	31	50406	Piedade dos Gerais	4.721
MG	31	50505	Pimenta	8.466
MG	31	50539	Pingo-d'Água	4.169
MG	31	50570	Pintópolis	8.075
MG	31	50604	Piracema	6.765
MG	31	50703	Pirajuba	3.940
MG	31	50802	Piranga	17.773
MG	31	50901	Piranguçu	5.292
MG	31	51008	Piranguinho	8.156
MG	31	51107	Pirapetinga	10.588
MG	31	51206	Pirapora	53.433
MG	31	51305	Piraúba	10.959
MG	31	51404	Pitangui	25.708
MG	31	51503	Piumhi	32.253
MG	31	51602	Planura	10.882
MG	31	51701	Poço Fundo	15.858
MG	31	51800	Poços de Caldas	150.095
MG	31	51909	Pocrane	8.896
MG	31	52006	Pompéu	29.595
MG	31	52105	Ponte Nova	57.482
MG	31	52170	Ponto dos Volantes	11.380
MG	31	52131	Ponto Chique	4.227
MG	31	52204	Porteirinha	37.877
MG	31	52303	Porto Firme	10.857
MG	31	52402	Poté	15.206
MG	31	52501	Pouso Alegre	126.100
MG	31	52600	Pouso Alto	6.516
MG	31	52709	Prados	8.487
MG	31	52808	Prata	26.573
MG	31	52907	Pratápolis	8.846
MG	31	53004	Pratinha	3.386
MG	31	53103	Presidente Bernardes	5.857
MG	31	53202	Presidente Juscelino	4.382
MG	31	53301	Presidente Kubitschek	3.075
MG	31	53400	Presidente Olegário	18.892
MG	31	53608	Prudente de Morais	9.239

U.F.		MUNICÍPIOS		POPULAÇÃO ESTIMADA
SIGLA	COD	COD	NOMES	
MG	31	53707	Quartel Geral	3.325
MG	31	53806	Queluzito	1.888
MG	31	53905	Raposos	15.418
MG	31	54002	Raul Soares	24.596
MG	31	54101	Recreio	10.514
MG	31	54150	Reduto	6.599
MG	31	54200	Resende Costa	10.894
MG	31	54309	Resplendor	17.563
MG	31	54408	Ressaquinha	4.720
MG	31	54457	Riachinho	8.401
MG	31	54507	Riacho dos Machados	9.691
MG	31	54606	Ribeirão das Neves	340.033
MG	31	54705	Ribeirão Vermelho	3.912
MG	31	55108	Rio do Prado	4.506
MG	31	54804	Rio Acima	8.597
MG	31	54903	Rio Casca	14.846
MG	31	55009	Rio Doce	2.626
MG	31	55207	Rio Espera	6.753
MG	31	55306	Rio Manso	5.212
MG	31	55405	Rio Novo	9.238
MG	31	55504	Rio Paranaíba	11.049
MG	31	55603	Rio Pardo de Minas	29.719
MG	31	55702	Rio Piracicaba	14.792
MG	31	55801	Rio Pomba	17.279
MG	31	55900	Rio Preto	5.590
MG	31	56007	Rio Vermelho	15.314
MG	31	56106	Ritápolis	5.178
MG	31	56205	Rochedo de Minas	2.117
MG	31	56304	Rodeiro	6.481
MG	31	56403	Romaria	3.649
MG	31	56452	Rosário da Limeira	4.320
MG	31	56502	Rubelita	8.300
MG	31	56601	Rubim	9.845
MG	31	56700	Sabará	125.285
MG	31	56809	Sabinópolis	16.334
MG	31	56908	Sacramento	22.965
MG	31	57005	Salinas	38.628
MG	31	57104	Salto da Divisa	7.127
MG	31	57203	Santa Bárbara	27.277
MG	31	57252	Santa Bárbara do Leste	7.717
MG	31	57278	Santa Bárbara do Monte Verde	2.942

U.F.		MUNICÍPIOS		POPULAÇÃO ESTIMADA
SIGLA	COD	COD	NOMES	
MG	31	57302	Santa Bárbara do Tugúrio	4.601
MG	31	57336	Santa Cruz de Minas	7.618
MG	31	57377	Santa Cruz de Salinas	5.408
MG	31	57401	Santa Cruz do Escalvado	5.331
MG	31	57500	Santa Efigênia de Minas	4.605
MG	31	57609	Santa Fé de Minas	4.138
MG	31	57658	Santa Helena de Minas	6.096
MG	31	57708	Santa Juliana	11.254
MG	31	57807	Santa Luzia	227.438
MG	31	57906	Santa Margarida	14.716
MG	31	58003	Santa Maria de Itabira	10.785
MG	31	58102	Santa Maria do Salto	5.942
MG	31	58201	Santa Maria do Suaçuí	14.889
MG	31	59209	Santa Rita de Caldas	9.335
MG	31	59407	Santa Rita de Ibitipoca	3.850
MG	31	59308	Santa Rita de Jacutinga	5.813
MG	31	59357	Santa Rita de Minas	5.967
MG	31	59506	Santa Rita do Itueto	5.774
MG	31	59605	Santa Rita do Sapucaí	35.724
MG	31	59704	Santa Rosa da Serra	3.383
MG	31	59803	Santa Vitória	15.858
MG	31	58300	Santana da Vargem	7.256
MG	31	58409	Santana de Cataguases	3.749
MG	31	58508	Santana de Pirapama	8.808
MG	31	58607	Santana do Deserto	3.961
MG	31	58706	Santana do Garambéu	2.186
MG	31	58805	Santana do Jacaré	4.691
MG	31	58904	Santana do Manhuaçu	8.384
MG	31	58953	Santana do Paraíso	24.105
MG	31	59001	Santana do Riacho	4.346
MG	31	59100	Santana dos Montes	4.120
MG	31	59902	Santo Antônio do Amparo	17.951
MG	31	60009	Santo Antônio do Aventureiro	3.595
MG	31	60108	Santo Antônio do Grama	4.355
MG	31	60207	Santo Antônio do Itambé	4.648
MG	31	60306	Santo Antônio do Jacinto	11.532
MG	31	60405	Santo Antônio do Monte	25.694
MG	31	60454	Santo Antônio do Retiro	7.052
MG	31	60504	Santo Antônio do Rio Abaixo	1.798
MG	31	60603	Santo Hipólito	3.659
MG	31	60702	Santos Dumont	47.240

U.F.		MUNICÍPIOS		POPULAÇÃO ESTIMADA
SIGLA	COD	COD	NOMES	
MG	31	60801	São Bento Abade	4.628
MG	31	60900	São Brás do Suaçuí	3.625
MG	31	60959	São Domingos das Dores	5.402
MG	31	61007	São Domingos do Prata	17.852
MG	31	61056	São Félix de Minas	3.484
MG	31	61106	São Francisco	54.846
MG	31	61205	São Francisco de Paula	6.403
MG	31	61304	São Francisco de Sales	5.314
MG	31	61403	São Francisco do Glória	5.690
MG	31	61502	São Geraldo	9.656
MG	31	61601	São Geraldo da Piedade	4.883
MG	31	61650	São Geraldo do Baixio	3.408
MG	31	61700	São Gonçalo do Abaeté	6.447
MG	31	61809	São Gonçalo do Pará	10.949
MG	31	61908	São Gonçalo do Rio Abaixo	9.627
MG	31	25507	São Gonçalo do Rio Preto	3.243
MG	31	62005	São Gonçalo do Sapucaí	23.523
MG	31	62104	São Gotardo	32.145
MG	31	62252	São João da Lagoa	4.921
MG	31	62302	São João da Mata	2.962
MG	31	62401	São João da Ponte	26.915
MG	31	62450	São João das Missões	11.179
MG	31	62500	São João del Rei	84.930
MG	31	62559	São João do Manhuaçu	9.781
MG	31	62575	São João do Manteninha	5.068
MG	31	62609	São João do Oriente	8.169
MG	31	62658	São João do Pacuí	4.174
MG	31	62708	São João do Paraíso	22.635
MG	31	62203	São João Batista do Glória	7.117
MG	31	62807	São João Evangelista	16.199
MG	31	62906	São João Nepomuceno	25.960
MG	31	62922	São Joaquim de Bicas	23.462
MG	31	62948	São José da Barra	6.999
MG	31	62955	São José da Lapa	18.855
MG	31	63003	São José da Safira	4.056
MG	31	63102	São José da Varginha	3.994
MG	31	63201	São José do Alegre	4.045
MG	31	63300	São José do Divino	3.882
MG	31	63409	São José do Goiabal	5.773
MG	31	63508	São José do Jacuri	7.199
MG	31	63607	São José do Mantimento	2.562

U.F.		MUNICÍPIOS		POPULAÇÃO ESTIMADA
SIGLA	COD	COD	NOMES	
MG	31	63706	São Lourenço	42.185
MG	31	63805	São Miguel do Anta	7.058
MG	31	63904	São Pedro da União	5.412
MG	31	64100	São Pedro do Suaçuí	5.944
MG	31	64001	São Pedro dos Ferros	9.109
MG	31	64209	São Romão	9.541
MG	31	64308	São Roque de Minas	6.308
MG	31	64407	São Sebastião da Bela Vista	5.115
MG	31	64431	São Sebastião da Vargem Alegre	2.851
MG	31	64472	São Sebastião do Anta	5.639
MG	31	64506	São Sebastião do Maranhão	12.062
MG	31	64605	São Sebastião do Oeste	5.597
MG	31	64704	São Sebastião do Paraíso	64.250
MG	31	64803	São Sebastião do Rio Preto	1.743
MG	31	64902	São Sebastião do Rio Verde	2.264
MG	31	65206	São Thomé das Letras	6.880
MG	31	65008	São Tiago	10.616
MG	31	65107	São Tomás de Aquino	7.100
MG	31	65305	São Vicente de Minas	6.496
MG	31	65404	Sapucaí-Mirim	5.996
MG	31	65503	Sardoá	5.416
MG	31	65537	Sarzedo	24.828
MG	31	65560	Sem-Peixe	3.012
MG	31	65578	Senador Amaral	5.198
MG	31	65602	Senador Cortes	2.076
MG	31	65701	Senador Firmino	7.296
MG	31	65800	Senador José Bento	1.905
MG	31	65909	Senador Modestino Gonçalves	5.117
MG	31	66006	Senhora de Oliveira	5.857
MG	31	66105	Senhora do Porto	3.627
MG	31	66204	Senhora dos Remédios	10.544
MG	31	66303	Sericita	7.318
MG	31	66402	Seritinga	1.812
MG	31	66600	Serra da Saudade	889
MG	31	66808	Serra do Salitre	10.657
MG	31	66709	Serra dos Aimorés	8.629
MG	31	66501	Serra Azul de Minas	4.456
MG	31	66907	Serrania	7.582
MG	31	66956	Serranópolis de Minas	4.721
MG	31	67004	Serranos	2.126
MG	31	67103	Serro	21.494

U.F.		MUNICÍPIOS		POPULAÇÃO ESTIMADA
SIGLA	COD	COD	NOMES	
MG	31	67202	Sete Lagoas	221.764
MG	31	65552	Setubinha	11.383
MG	31	67301	Silveirânia	2.190
MG	31	67400	Silvianópolis	6.228
MG	31	67509	Simão Pereira	2.585
MG	31	67608	Simonésia	17.849
MG	31	67707	Sobrália	6.137
MG	31	67806	Soledade de Minas	5.740
MG	31	67905	Tabuleiro	4.118
MG	31	68002	Taiobeiras	30.986
MG	31	68051	Taparuba	3.347
MG	31	68101	Tapira	3.720
MG	31	68200	Tapiraí	1.891
MG	31	68309	Taquaraçu de Minas	3.910
MG	31	68408	Tarumirim	14.588
MG	31	68507	Teixeiras	12.101
MG	31	68606	Teófilo Otoni	130.521
MG	31	68705	Timóteo	79.100
MG	31	68804	Tiradentes	6.859
MG	31	68903	Tiros	7.627
MG	31	69000	Tocantins	16.290
MG	31	69059	Tocos do Moji	4.063
MG	31	69109	Toledo	5.967
MG	31	69208	Tombos	9.147
MG	31	69307	Três Corações	74.859
MG	31	69356	Três Marias	27.648
MG	31	69406	Três Pontas	53.901
MG	31	69505	Tumiritinga	6.169
MG	31	69604	Tupaciguara	23.792
MG	31	69703	Turmalina	17.939
MG	31	69802	Turvolândia	4.952
MG	31	69901	Ubá	98.423
MG	31	70008	Ubaí	12.348
MG	31	70057	Ubaporanga	12.489
MG	31	70107	Uberaba	292.377
MG	31	70206	Uberlândia	622.441
MG	31	70305	Umburatiba	2.849
MG	31	70404	Unaí	77.433
MG	31	70438	União de Minas	4.730
MG	31	70479	Uruana de Minas	2.797
MG	31	70503	Urucânia	10.499

U.F.		MUNICÍPIOS		POPULAÇÃO ESTIMADA
SIGLA	COD	COD	NOMES	
MG	31	70529	Urucuia	11.971
MG	31	70578	Vargem Alegre	6.808
MG	31	70602	Vargem Bonita	2.148
MG	31	70651	Vargem Grande do Rio Pardo	4.884
MG	31	70701	Varginha	120.691
MG	31	70750	Varjão de Minas	6.356
MG	31	70800	Várzea da Palma	35.907
MG	31	70909	Varzelândia	19.731
MG	31	71006	Vazante	19.954
MG	31	71030	Verdelândia	8.396
MG	31	71071	Veredinha	5.976
MG	31	71105	Veríssimo	3.890
MG	31	71154	Vermelho Novo	4.690
MG	31	71204	Vespasiano	99.557
MG	31	71303	Viçosa	73.362
MG	31	71402	Vieiras	3.908
MG	31	71600	Virgem da Lapa	14.602
MG	31	71709	Virgínia	8.565
MG	31	71808	Virginópolis	11.241
MG	31	71907	Virgolândia	5.851
MG	31	72004	Visconde do Rio Branco	36.826
MG	31	72103	Volta Grande	5.362
MG	31	72202	Wenceslau Braz	2.576
ES	32	00102	Afonso Cláudio	31.489
ES	32	00169	Água Doce do Norte	12.163
ES	32	00136	Águia Branca	9.520
ES	32	00201	Alegre	31.222
ES	32	00300	Alfredo Chaves	14.507
ES	32	00359	Alto Rio Novo	6.251
ES	32	00409	Anchieta	20.144
ES	32	00508	Apiacá	7.864
ES	32	00607	Aracruz	77.414
ES	32	00706	Atilio Vivacqua	9.272
ES	32	00805	Baixo Guandu	29.722
ES	32	00904	Barra de São Francisco	41.301
ES	32	01001	Boa Esperança	13.182
ES	32	01100	Bom Jesus do Norte	9.638
ES	32	01159	Brejetuba	11.161
ES	32	01209	Cachoeiro de Itapemirim	198.962
ES	32	01308	Cariacica	362.277
ES	32	01407	Castelo	33.197

U.F.		MUNICÍPIOS		POPULAÇÃO ESTIMADA
SIGLA	COD	COD	NOMES	
ES	32	01506	Colatina	110.713
ES	32	01605	Conceição da Barra	27.029
ES	32	01704	Conceição do Castelo	11.773
ES	32	01803	Divino de São Lourenço	4.997
ES	32	01902	Domingos Martins	32.304
ES	32	02009	Dores do Rio Preto	6.288
ES	32	02108	Ecoporanga	23.919
ES	32	02207	Fundão	16.125
ES	32	02256	Governador Lindenberg	10.324
ES	32	02306	Guaçuí	26.648
ES	32	02405	Guarapari	103.113
ES	32	02454	Ibatiba	20.370
ES	32	02504	Ibiraçu	10.679
ES	32	02553	Ibitirama	9.243
ES	32	02603	Iconha	11.872
ES	32	02652	Irupi	10.708
ES	32	02702	Itaguaçu	14.212
ES	32	02801	Itapemirim	32.354
ES	32	02900	Itarana	10.746
ES	32	03007	Iúna	26.248
ES	32	03056	Jaguaré	23.125
ES	32	03106	Jerônimo Monteiro	11.146
ES	32	03130	João Neiva	14.697
ES	32	03163	Laranja da Terra	11.126
ES	32	03205	Linhares	130.901
ES	32	03304	Mantenópolis	11.692
ES	32	03320	Marataízes	32.351
ES	32	03346	Marechal Floriano	13.208
ES	32	03353	Marilândia	10.615
ES	32	03403	Mimoso do Sul	27.059
ES	32	03502	Montanha	18.723
ES	32	03601	Mucurici	5.914
ES	32	03700	Muniz Freire	18.497
ES	32	03809	Muqui	14.322
ES	32	03908	Nova Venécia	46.080
ES	32	04005	Pancas	18.690
ES	32	04054	Pedro Canário	24.196
ES	32	04104	Pinheiros	23.656
ES	32	04203	Piúma	17.019
ES	32	04252	Ponto Belo	7.161
ES	32	04302	Presidente Kennedy	10.786

U.F.		MUNICÍPIOS		POPULAÇÃO ESTIMADA
SIGLA	COD	COD	NOMES	
ES	32	04351	Rio Bananal	17.174
ES	32	04401	Rio Novo do Sul	11.440
ES	32	04500	Santa Leopoldina	12.727
ES	32	04559	Santa Maria de Jetibá	33.468
ES	32	04609	Santa Teresa	20.747
ES	32	04658	São Domingos do Norte	8.150
ES	32	04708	São Gabriel da Palha	30.255
ES	32	04807	São José do Calçado	10.929
ES	32	04906	São Mateus	100.655
ES	32	04955	São Roque do Canaã	10.786
ES	32	05002	Serra	397.226
ES	32	05010	Sooretama	23.268
ES	32	05036	Vargem Alta	18.534
ES	32	05069	Venda Nova do Imigrante	19.684
ES	32	05101	Viana	60.191
ES	32	05150	Vila Pavão	9.059
ES	32	05176	Vila Valério	14.044
ES	32	05200	Vila Velha	407.579
ES	32	05309	Vitória	317.817
RJ	33	00100	Angra dos Reis	164.191
RJ	33	00159	Aperibé	9.420
RJ	33	00209	Araruama	107.285
RJ	33	00225	Areal	11.797
RJ	33	00233	Armação dos Búzios	27.701
RJ	33	00258	Arraial do Cabo	26.636
RJ	33	00308	Barra do Piraí	102.487
RJ	33	00407	Barra Mansa	176.469
RJ	33	00456	Belford Roxo	495.694
RJ	33	00506	Bom Jardim	26.207
RJ	33	00605	Bom Jesus do Itabapoana	35.178
RJ	33	00704	Cabo Frio	180.635
RJ	33	00803	Cachoeiras de Macacu	56.529
RJ	33	00902	Cambuci	14.772
RJ	33	01009	Campos dos Goytacazes	431.839
RJ	33	01108	Cantagalo	20.491
RJ	33	00936	Carapebus	11.671
RJ	33	01157	Cardoso Moreira	12.502
RJ	33	01207	Carmo	17.784
RJ	33	01306	Casimiro de Abreu	29.811
RJ	33	00951	Comendador Levy Gasparian	8.760
RJ	33	01405	Conceição de Macabu	20.505

U.F.		MUNICÍPIOS		POPULAÇÃO ESTIMADA
SIGLA	COD	COD	NOMES	
RJ	33	01504	Cordeiro	19.797
RJ	33	01603	Duas Barras	10.848
RJ	33	01702	Duque de Caxias	864.392
RJ	33	01801	Engenheiro Paulo de Frontin	13.127
RJ	33	01850	Guapimirim	48.688
RJ	33	01876	Iguaba Grande	22.199
RJ	33	01900	Itaboraí	225.309
RJ	33	02007	Itaguaí	103.515
RJ	33	02056	Italva	14.496
RJ	33	02106	Itaocara	22.522
RJ	33	02205	Itaperuna	98.347
RJ	33	02254	Itatiaia	34.595
RJ	33	02270	Japeri	100.055
RJ	33	02304	Laje do Muriaé	7.996
RJ	33	02403	Macaé	188.787
RJ	33	02452	Macuco	5.561
RJ	33	02502	Magé	240.940
RJ	33	02601	Mangaratiba	31.848
RJ	33	02700	Maricá	119.231
RJ	33	02809	Mendes	17.839
RJ	33	02858	Mesquita	187.949
RJ	33	02908	Miguel Pereira	25.704
RJ	33	03005	Miracema	26.868
RJ	33	03104	Natividade	15.392
RJ	33	03203	Nilópolis	159.005
RJ	33	03302	Niterói	477.912
RJ	33	03401	Nova Friburgo	178.310
RJ	33	03500	Nova Iguaçu	855.500
RJ	33	03609	Paracambi	44.629
RJ	33	03708	Paraíba do Sul	41.315
RJ	33	03807	Parati	35.182
RJ	33	03856	Paty do Alferes	26.099
RJ	33	03906	Petrópolis	312.766
RJ	33	03955	Pinheiral	22.129
RJ	33	04003	Piraí	25.762
RJ	33	04102	Porciúncula	18.227
RJ	33	04110	Porto Real	15.879
RJ	33	04128	Quatis	12.923
RJ	33	04144	Queimados	137.870
RJ	33	04151	Quissamã	19.315
RJ	33	04201	Resende	127.763

U.F.		MUNICÍPIOS		POPULAÇÃO ESTIMADA
SIGLA	COD	COD	NOMES	
RJ	33	04508	Rio das Flores	8.686
RJ	33	04524	Rio das Ostras	91.085
RJ	33	04557	Rio de Janeiro	6.161.047
RJ	33	04300	Rio Bonito	54.596
RJ	33	04409	Rio Claro	18.181
RJ	33	04607	Santa Maria Madalena	10.756
RJ	33	04706	Santo Antônio de Pádua	42.093
RJ	33	04805	São Fidélis	39.057
RJ	33	04755	São Francisco de Itabapoana	47.247
RJ	33	04904	São Gonçalo	982.832
RJ	33	05000	São João da Barra	30.348
RJ	33	05109	São João de Meriti	468.309
RJ	33	05133	São José de Ubá	7.220
RJ	33	05158	São José do Vale do Rio Preto	20.412
RJ	33	05208	São Pedro da Aldeia	82.951
RJ	33	05307	São Sebastião do Alto	8.998
RJ	33	05406	Sapucaia	17.352
RJ	33	05505	Saquarema	67.853
RJ	33	05554	Seropédica	77.618
RJ	33	05604	Silva Jardim	22.158
RJ	33	05703	Sumidouro	15.219
RJ	33	05752	Tanguá	30.139
RJ	33	05802	Teresópolis	159.968
RJ	33	05901	Trajano de Morais	9.934
RJ	33	06008	Três Rios	75.754
RJ	33	06107	Valença	74.993
RJ	33	06156	Varre-Sai	8.766
RJ	33	06206	Vassouras	34.024
RJ	33	06305	Volta Redonda	259.811
SP	35	00105	Adamantina	34.366
SP	35	00204	Adolfo	3.711
SP	35	00303	Aguaí	31.763
SP	35	00402	Águas da Prata	7.684
SP	35	00501	Águas de Lindóia	16.335
SP	35	00550	Águas de Santa Bárbara	5.580
SP	35	00600	Águas de São Pedro	2.547
SP	35	00709	Agudos	35.872
SP	35	00758	Alambari	4.325
SP	35	00808	Alfredo Marcondes	4.025
SP	35	00907	Altair	3.312
SP	35	01004	Altinópolis	15.560

U.F.		MUNICÍPIOS		POPULAÇÃO ESTIMADA
SIGLA	COD	COD	NOMES	
SP	35	01103	Alto Alegre	4.270
SP	35	01152	Alumínio	16.331
SP	35	01202	Álvares Florence	3.920
SP	35	01301	Álvares Machado	23.694
SP	35	01400	Álvaro de Carvalho	4.907
SP	35	01509	Alvinlândia	2.868
SP	35	01608	Americana	203.283
SP	35	01806	Américo de Campos	5.503
SP	35	01707	Américo Brasiliense	32.819
SP	35	01905	Amparo	65.466
SP	35	02002	Analândia	4.471
SP	35	02101	Andradina	56.505
SP	35	02200	Angatuba	22.877
SP	35	02309	Anhembi	5.656
SP	35	02408	Anhumas	3.900
SP	35	02507	Aparecida	37.405
SP	35	02606	Aparecida d'Oeste	4.633
SP	35	02705	Apiaí	25.857
SP	35	02754	Araçariguama	13.027
SP	35	02804	Araçatuba	181.143
SP	35	02903	Araçoiaba da Serra	26.012
SP	35	03000	Aramina	5.262
SP	35	03109	Arandu	6.204
SP	35	03158	Arapeí	2.587
SP	35	03208	Araraquara	199.132
SP	35	03307	Araras	113.645
SP	35	03356	Arco-Íris	2.026
SP	35	03406	Arealva	7.833
SP	35	03505	Areias	3.684
SP	35	03604	Areiópolis	11.085
SP	35	03703	Ariranha	8.753
SP	35	03802	Artur Nogueira	42.567
SP	35	03901	Arujá	78.960
SP	35	03950	Aspásia	1.831
SP	35	04008	Assis	97.725
SP	35	04107	Atibaia	125.418
SP	35	04206	Auriflama	14.298
SP	35	04305	Avaí	5.121
SP	35	04404	Avanhandava	11.816
SP	35	04503	Avaré	83.744
SP	35	04602	Bady Bassitt	13.898

U.F.		MUNICÍPIOS		POPULAÇÃO ESTIMADA
SIGLA	COD	COD	NOMES	
SP	35	04701	Balbinos	4.385
SP	35	04800	Bálsamo	8.151
SP	35	04909	Bananal	10.727
SP	35	05005	Barão de Antonina	2.833
SP	35	05104	Barbosa	6.846
SP	35	05203	Bariri	32.824
SP	35	05351	Barra do Chapéu	5.314
SP	35	05401	Barra do Turvo	7.744
SP	35	05302	Barra Bonita	36.176
SP	35	05500	Barretos	112.804
SP	35	05609	Barrinha	26.915
SP	35	05708	Barueri	264.619
SP	35	05807	Bastos	21.323
SP	35	05906	Batatais	56.022
SP	35	06003	Bauru	355.675
SP	35	06102	Bebedouro	77.430
SP	35	06201	Bento de Abreu	2.929
SP	35	06300	Bernardino de Campos	10.780
SP	35	06359	Bertioga	42.945
SP	35	06409	Bilac	7.369
SP	35	06508	Birigui	109.451
SP	35	06607	Biritiba-Mirim	29.208
SP	35	06706	Boa Esperança do Sul	13.835
SP	35	06805	Bocaina	10.889
SP	35	06904	Bofete	9.194
SP	35	07001	Boituva	43.959
SP	35	07100	Bom Jesus dos Perdões	17.571
SP	35	07159	Bom Sucesso de Itararé	3.902
SP	35	07209	Borá	834
SP	35	07308	Boracéia	4.377
SP	35	07407	Borborema	14.376
SP	35	07456	Borebi	2.312
SP	35	07506	Botucatu	128.397
SP	35	07605	Bragança Paulista	144.066
SP	35	07704	Braúna	4.985
SP	35	07753	Brejo Alegre	2.550
SP	35	07803	Brodowski	20.190
SP	35	07902	Brotas	22.300
SP	35	08009	Buri	18.112
SP	35	08108	Buritama	15.482
SP	35	08207	Buritizal	4.059

U.F.		MUNICÍPIOS		POPULAÇÃO ESTIMADA
SIGLA	COD	COD	NOMES	
SP	35	08306	Cabrália Paulista	4.400
SP	35	08405	Cabreúva	41.839
SP	35	08504	Caçapava	84.406
SP	35	08603	Cachoeira Paulista	33.999
SP	35	08702	Caconde	19.233
SP	35	08801	Cafelândia	16.699
SP	35	08900	Caiabu	4.130
SP	35	09007	Caieiras	86.698
SP	35	09106	Caiuá	5.334
SP	35	09205	Cajamar	62.522
SP	35	09254	Cajati	28.987
SP	35	09304	Cajobi	9.939
SP	35	09403	Cajuru	24.003
SP	35	09452	Campina do Monte Alegre	5.532
SP	35	09502	Campinas	1.056.644
SP	35	09601	Campo Limpo Paulista	73.885
SP	35	09700	Campos do Jordão	46.332
SP	35	09809	Campos Novos Paulista	4.940
SP	35	09908	Cananéia	12.377
SP	35	09957	Canas	4.662
SP	35	10005	Cândido Mota	30.662
SP	35	10104	Cândido Rodrigues	2.757
SP	35	10153	Canitar	4.629
SP	35	10203	Capão Bonito	46.412
SP	35	10302	Capela do Alto	16.968
SP	35	10401	Capivari	45.915
SP	35	10500	Caraguatatuba	94.598
SP	35	10609	Carapicuíba	388.532
SP	35	10708	Cardoso	11.632
SP	35	10807	Casa Branca	28.083
SP	35	10906	Cássia dos Coqueiros	2.752
SP	35	11003	Castilho	16.064
SP	35	11102	Catanduva	114.069
SP	35	11201	Catiguá	7.191
SP	35	11300	Cedral	8.119
SP	35	11409	Cerqueira César	17.147
SP	35	11508	Cerquilho	37.419
SP	35	11607	Cesário Lange	14.795
SP	35	11706	Charqueada	15.213
SP	35	57204	Chavantes	12.652
SP	35	11904	Clementina	6.477

U.F.		MUNICÍPIOS		POPULAÇÃO ESTIMADA
SIGLA	COD	COD	NOMES	
SP	35	12001	Colina	17.659
SP	35	12100	Colômbia	6.314
SP	35	12209	Conchal	24.336
SP	35	12308	Conchas	16.160
SP	35	12407	Cordeirópolis	20.445
SP	35	12506	Coroados	5.177
SP	35	12605	Coronel Macedo	5.370
SP	35	12704	Corumbataí	4.109
SP	35	12803	Cosmópolis	57.951
SP	35	12902	Cosmorama	7.071
SP	35	13009	Cotia	179.109
SP	35	13108	Cravinhos	30.647
SP	35	13207	Cristais Paulista	7.362
SP	35	13306	Cruzália	2.382
SP	35	13405	Cruzeiro	79.418
SP	35	13504	Cubatão	127.702
SP	35	13603	Cunha	23.694
SP	35	13702	Descalvado	30.710
SP	35	13801	Diadema	394.266
SP	35	13850	Dirce Reis	1.624
SP	35	13900	Divinolândia	11.415
SP	35	14007	Dobrada	8.108
SP	35	14106	Dois Córregos	25.733
SP	35	14205	Dolcinópolis	2.263
SP	35	14304	Dourado	9.089
SP	35	14403	Dracena	43.989
SP	35	14502	Duartina	12.777
SP	35	14601	Dumont	8.163
SP	35	14700	Echaporã	6.299
SP	35	14809	Eldorado	14.490
SP	35	14908	Elias Fausto	15.192
SP	35	14924	Elisiário	3.251
SP	35	14957	Embaúba	2.448
SP	35	15004	Embu	245.093
SP	35	15103	Embu-Guaçu	61.701
SP	35	15129	Emilianópolis	3.159
SP	35	15152	Engenheiro Coelho	13.914
SP	35	15186	Espírito Santo do Pinhal	42.128
SP	35	15194	Espírito Santo do Turvo	4.361
SP	35	57303	Estiva Gerbi	9.590
SP	35	15202	Estrela d'Oeste	8.976

U.F.		MUNICÍPIOS		POPULAÇÃO ESTIMADA
SIGLA	COD	COD	NOMES	
SP	35	15301	Estrela do Norte	2.490
SP	35	15350	Euclides da Cunha Paulista	10.180
SP	35	15400	Fartura	14.985
SP	35	15608	Fernando Prestes	5.328
SP	35	15509	Fernandópolis	63.414
SP	35	15657	Fernão	1.514
SP	35	15707	Ferraz de Vasconcelos	175.939
SP	35	15806	Flora Rica	2.044
SP	35	15905	Floreal	2.918
SP	35	16002	Flórida Paulista	13.524
SP	35	16101	Florínia	2.883
SP	35	16200	Franca	327.176
SP	35	16309	Francisco Morato	155.224
SP	35	16408	Franco da Rocha	129.304
SP	35	16507	Gabriel Monteiro	2.703
SP	35	16606	Gália	6.754
SP	35	16705	Garça	43.395
SP	35	16804	Gastão Vidigal	4.071
SP	35	16853	Gavião Peixoto	4.236
SP	35	16903	General Salgado	10.933
SP	35	17000	Getulina	10.914
SP	35	17109	Glicério	4.540
SP	35	17208	Guaiçara	11.029
SP	35	17307	Guaimbê	4.787
SP	35	17406	Guaíra	38.328
SP	35	17505	Guapiaçu	17.593
SP	35	17604	Guapiara	20.832
SP	35	17703	Guará	19.160
SP	35	17802	Guaraçaí	8.686
SP	35	17901	Guaraci	9.409
SP	35	18008	Guarani d'Oeste	2.018
SP	35	18107	Guarantã	6.652
SP	35	18206	Guararapes	29.589
SP	35	18305	Guararema	26.523
SP	35	18404	Guaratinguetá	112.596
SP	35	18503	Guareí	14.490
SP	35	18602	Guariba	34.217
SP	35	18701	Guarujá	304.274
SP	35	18800	Guarulhos	1.279.202
SP	35	18859	Guatapará	6.386
SP	35	18909	Guzolândia	4.863

U.F.		MUNICÍPIOS		POPULAÇÃO ESTIMADA
SIGLA	COD	COD	NOMES	
SP	35	19006	Herculândia	9.027
SP	35	19055	Holambra	9.951
SP	35	19071	Hortolândia	201.049
SP	35	19105	Iacanga	9.605
SP	35	19204	Iacri	6.875
SP	35	19253	Iaras	5.420
SP	35	19303	Ibaté	29.435
SP	35	19402	Ibirá	10.992
SP	35	19501	Ibirarema	7.097
SP	35	19600	Ibitinga	52.582
SP	35	19709	Ibiúna	67.166
SP	35	19808	Icém	6.553
SP	35	19907	Iepê	7.806
SP	35	20004	Igaraçu do Tietê	24.003
SP	35	20103	Igarapava	28.038
SP	35	20202	Igaratá	8.896
SP	35	20301	Iguape	30.397
SP	35	20426	Ilha Comprida	9.782
SP	35	20442	Ilha Solteira	25.057
SP	35	20400	Ilhabela	25.550
SP	35	20509	Indaiatuba	180.524
SP	35	20608	Indiana	4.772
SP	35	20707	Indiaporã	3.963
SP	35	20806	Inúbia Paulista	3.795
SP	35	20905	Ipaussu	13.521
SP	35	21002	Iperó	26.696
SP	35	21101	Ipeúna	5.570
SP	35	21150	Ipiguá	4.184
SP	35	21200	Iporanga	4.772
SP	35	21309	Ipuã	15.522
SP	35	21408	Iracemápolis	19.329
SP	35	21507	Irapuã	6.953
SP	35	21606	Irapuru	7.841
SP	35	21705	Itaberá	17.804
SP	35	21804	Itaí	23.828
SP	35	21903	Itajobi	14.652
SP	35	22000	Itaju	2.709
SP	35	22109	Itanhaém	85.977
SP	35	22158	Itaóca	3.153
SP	35	22208	Itapecerica da Serra	159.102
SP	35	22307	Itapetininga	146.760

U.F.		MUNICÍPIOS		POPULAÇÃO ESTIMADA
SIGLA	COD	COD	NOMES	
SP	35	22406	Itapeva	89.197
SP	35	22505	Itapevi	201.995
SP	35	22604	Itapira	71.850
SP	35	22653	Itapirapuã Paulista	3.890
SP	35	22703	Itápolis	40.196
SP	35	22802	Itaporanga	14.752
SP	35	22901	Itapuí	12.344
SP	35	23008	Itapura	3.935
SP	35	23107	Itaquaquecetuba	351.493
SP	35	23206	Itararé	51.000
SP	35	23305	Itariri	16.048
SP	35	23404	Itatiba	97.462
SP	35	23503	Itatinga	18.761
SP	35	23602	Itirapina	14.655
SP	35	23701	Itirapuã	5.862
SP	35	23800	Itobi	7.692
SP	35	23909	Itu	155.457
SP	35	24006	Itupeva	40.972
SP	35	24105	Ituverava	40.485
SP	35	24204	Jaborandi	6.693
SP	35	24303	Jaboticabal	72.614
SP	35	24402	Jacareí	210.988
SP	35	24501	Jaci	5.427
SP	35	24600	Jacupiranga	16.403
SP	35	24709	Jaguariúna	40.066
SP	35	24808	Jales	49.681
SP	35	24907	Jambeiro	5.409
SP	35	25003	Jandira	110.325
SP	35	25102	Jardinópolis	36.872
SP	35	25201	Jarinu	22.301
SP	35	25300	Jaú	133.469
SP	35	25409	Jeriquara	3.225
SP	35	25508	Joanópolis	11.107
SP	35	25607	João Ramalho	4.301
SP	35	25706	José Bonifácio	32.219
SP	35	25805	Júlio Mesquita	4.508
SP	35	25854	Jumirim	2.283
SP	35	25904	Jundiaí	347.738
SP	35	26001	Junqueirópolis	19.715
SP	35	26100	Juquiá	19.688
SP	35	26209	Juquitiba	29.090

U.F.		MUNICÍPIOS		POPULAÇÃO ESTIMADA
SIGLA	COD	COD	NOMES	
SP	35	26308	Lagoinha	4.917
SP	35	26407	Laranjal Paulista	25.930
SP	35	26506	Lavínia	9.050
SP	35	26605	Lavrinhas	6.915
SP	35	26704	Leme	88.299
SP	35	26803	Lençóis Paulista	62.594
SP	35	26902	Limeira	278.776
SP	35	27009	Lindóia	5.924
SP	35	27108	Lins	72.568
SP	35	27207	Lorena	82.391
SP	35	27256	Lourdes	2.031
SP	35	27306	Louveira	32.406
SP	35	27405	Lucélia	20.117
SP	35	27504	Lucianópolis	2.418
SP	35	27603	Luís Antônio	11.489
SP	35	27702	Luiziânia	5.061
SP	35	27801	Lupércio	4.384
SP	35	27900	Lutécia	2.861
SP	35	28007	Macatuba	16.842
SP	35	28106	Macaubal	7.652
SP	35	28205	Macedônia	3.430
SP	35	28304	Magda	3.187
SP	35	28403	Mairinque	43.349
SP	35	28502	Mairiporã	77.443
SP	35	28601	Manduri	9.051
SP	35	28700	Marabá Paulista	5.787
SP	35	28809	Maracaí	13.655
SP	35	28858	Marapoama	2.731
SP	35	28908	Mariápolis	3.896
SP	35	29005	Marília	223.454
SP	35	29104	Marinópolis	2.163
SP	35	29203	Martinópolis	25.256
SP	35	29302	Matão	77.682
SP	35	29401	Mauá	412.753
SP	35	29500	Mendonça	4.177
SP	35	29609	Meridiano	3.941
SP	35	29658	Mesópolis	1.784
SP	35	29708	Miguelópolis	20.918
SP	35	29807	Mineiros do Tietê	12.258
SP	35	30003	Mira Estrela	2.659
SP	35	29906	Miracatu	23.688

U.F.		MUNICÍPIOS		POPULAÇÃO ESTIMADA
SIGLA	COD	COD	NOMES	
SP	35	30102	Mirandópolis	26.705
SP	35	30201	Mirante do Paranapanema	17.967
SP	35	30300	Mirassol	54.350
SP	35	30409	Mirassolândia	4.338
SP	35	30508	Mococa	68.481
SP	35	30607	Mogi das Cruzes	371.372
SP	35	30706	Mogi Guaçu	138.494
SP	35	30805	Moji Mirim	87.800
SP	35	30904	Mombuca	3.440
SP	35	31001	Monções	2.124
SP	35	31100	Mongaguá	43.284
SP	35	31209	Monte Alegre do Sul	7.371
SP	35	31308	Monte Alto	45.719
SP	35	31407	Monte Aprazível	20.789
SP	35	31506	Monte Azul Paulista	19.739
SP	35	31605	Monte Castelo	4.130
SP	35	31803	Monte Mor	45.811
SP	35	31704	Monteiro Lobato	4.235
SP	35	31902	Morro Agudo	26.245
SP	35	32009	Morungaba	12.999
SP	35	32058	Motuca	4.618
SP	35	32108	Murutinga do Sul	4.264
SP	35	32157	Nantes	2.627
SP	35	32207	Narandiba	4.131
SP	35	32306	Natividade da Serra	7.613
SP	35	32405	Nazaré Paulista	15.168
SP	35	32504	Neves Paulista	9.102
SP	35	32603	Nhandeara	10.726
SP	35	32702	Nipoã	4.029
SP	35	32801	Nova Aliança	5.110
SP	35	32827	Nova Campina	9.091
SP	35	32843	Nova Canaã Paulista	2.203
SP	35	32868	Nova Castilho	1.111
SP	35	32900	Nova Europa	9.840
SP	35	33007	Nova Granada	18.543
SP	35	33106	Nova Guataporanga	2.176
SP	35	33205	Nova Independência	2.681
SP	35	33304	Nova Luzitânia	3.701
SP	35	33403	Nova Odessa	48.170
SP	35	33254	Novais	3.908
SP	35	33502	Novo Horizonte	35.942

U.F.		MUNICÍPIOS		POPULAÇÃO ESTIMADA
SIGLA	COD	COD	NOMES	
SP	35	33601	Nuporanga	6.945
SP	35	33700	Ocauçu	4.328
SP	35	33809	Óleo	2.729
SP	35	33908	Olímpia	50.215
SP	35	34005	Onda Verde	3.953
SP	35	34104	Oriente	6.341
SP	35	34203	Orindiúva	5.294
SP	35	34302	Orlândia	37.422
SP	35	34401	Osasco	713.066
SP	35	34500	Oscar Bressane	2.540
SP	35	34609	Osvaldo Cruz	31.317
SP	35	34708	Ourinhos	103.631
SP	35	34807	Ouro Verde	8.074
SP	35	34757	Ouroeste	7.482
SP	35	34906	Pacaembu	13.672
SP	35	35002	Palestina	11.153
SP	35	35101	Palmares Paulista	11.444
SP	35	35200	Palmeira d'Oeste	9.771
SP	35	35309	Palmital	22.190
SP	35	35408	Panorama	14.502
SP	35	35507	Paraguaçu Paulista	44.249
SP	35	35606	Paraibuna	16.863
SP	35	35705	Paraíso	5.785
SP	35	35804	Paranapanema	17.558
SP	35	35903	Paranapuã	3.732
SP	35	36000	Parapuã	11.383
SP	35	36109	Pardinho	5.285
SP	35	36208	Pariquera-Açu	18.815
SP	35	36257	Parisi	2.132
SP	35	36307	Patrocínio Paulista	12.811
SP	35	36406	Paulicéia	5.750
SP	35	36505	Paulínia	81.544
SP	35	36570	Paulistânia	1.898
SP	35	36604	Paulo de Faria	9.378
SP	35	36703	Pederneiras	42.661
SP	35	36802	Pedra Bela	6.097
SP	35	36901	Pedranópolis	2.827
SP	35	37008	Pedregulho	15.717
SP	35	37107	Pedreira	40.269
SP	35	37156	Pedrinhas Paulista	2.923
SP	35	37206	Pedro de Toledo	10.163

U.F.		MUNICÍPIOS		POPULAÇÃO ESTIMADA
SIGLA	COD	COD	NOMES	
SP	35	37305	Penápolis	59.183
SP	35	37404	Pereira Barreto	24.821
SP	35	37503	Pereiras	7.910
SP	35	37602	Peruíbe	57.151
SP	35	37701	Piacatu	5.398
SP	35	37800	Piedade	49.607
SP	35	37909	Pilar do Sul	28.058
SP	35	38006	Pindamonhangaba	142.997
SP	35	38105	Pindorama	15.178
SP	35	38204	Pinhalzinho	12.451
SP	35	38303	Piquerobi	3.756
SP	35	38501	Piquete	14.766
SP	35	38600	Piracaia	22.815
SP	35	38709	Piracicaba	365.440
SP	35	38808	Piraju	29.282
SP	35	38907	Pirajuí	22.014
SP	35	39004	Pirangi	10.744
SP	35	39103	Pirapora do Bom Jesus	15.410
SP	35	39202	Pirapozinho	24.964
SP	35	39301	Pirassununga	70.912
SP	35	39400	Piratininga	11.868
SP	35	39509	Pitangueiras	35.070
SP	35	39608	Planalto	4.247
SP	35	39707	Platina	3.359
SP	35	39806	Poá	111.016
SP	35	39905	Poloni	5.074
SP	35	40002	Pompéia	19.998
SP	35	40101	Pongaí	3.530
SP	35	40200	Pontal	38.409
SP	35	40259	Pontalinda	4.142
SP	35	40309	Pontes Gestal	2.557
SP	35	40408	Populina	4.275
SP	35	40507	Porangaba	8.739
SP	35	40606	Porto Feliz	47.774
SP	35	40705	Porto Ferreira	50.791
SP	35	40754	Potim	20.026
SP	35	40804	Potirendaba	15.003
SP	35	40853	Pracinha	3.102
SP	35	40903	Pradópolis	16.287
SP	35	41000	Praia Grande	244.533
SP	35	41059	Pratânia	4.555

U.F.		MUNICÍPIOS		POPULAÇÃO ESTIMADA
SIGLA	COD	COD	NOMES	
SP	35	41109	Presidente Alves	4.502
SP	35	41208	Presidente Bernardes	15.327
SP	35	41307	Presidente Epitácio	40.775
SP	35	41406	Presidente Prudente	206.164
SP	35	41505	Presidente Venceslau	38.368
SP	35	41604	Promissão	36.997
SP	35	41653	Quadra	2.779
SP	35	41703	Quatá	12.467
SP	35	41802	Queiroz	2.811
SP	35	41901	Queluz	11.012
SP	35	42008	Quintana	5.906
SP	35	42107	Rafard	8.370
SP	35	42206	Rancharia	29.138
SP	35	42305	Redenção da Serra	4.230
SP	35	42404	Regente Feijó	17.671
SP	35	42503	Reginópolis	7.859
SP	35	42602	Registro	55.081
SP	35	42701	Restinga	6.780
SP	35	42800	Ribeira	3.544
SP	35	43204	Ribeirão do Sul	4.651
SP	35	43238	Ribeirão dos Índios	2.310
SP	35	42909	Ribeirão Bonito	11.810
SP	35	43006	Ribeirão Branco	18.867
SP	35	43105	Ribeirão Corrente	4.188
SP	35	43253	Ribeirão Grande	7.035
SP	35	43303	Ribeirão Pires	111.402
SP	35	43402	Ribeirão Preto	558.136
SP	35	43600	Rifaina	3.783
SP	35	43709	Rincão	10.807
SP	35	43808	Rinópolis	9.473
SP	35	44004	Rio das Pedras	28.036
SP	35	43907	Rio Claro	189.834
SP	35	44103	Rio Grande da Serra	41.215
SP	35	44202	Riolândia	10.365
SP	35	43501	Riversul	6.588
SP	35	44251	Rosana	19.428
SP	35	44301	Roseira	9.446
SP	35	44400	Rubiácea	2.633
SP	35	44509	Rubinéia	2.614
SP	35	44608	Sabino	5.381
SP	35	44707	Sagres	2.349

U.F.		MUNICÍPIOS		POPULAÇÃO ESTIMADA
SIGLA	COD	COD	NOMES	
SP	35	44806	Sales	5.325
SP	35	44905	Sales Oliveira	8.149
SP	35	45001	Salesópolis	15.897
SP	35	45100	Salmourão	4.748
SP	35	45159	Saltinho	7.029
SP	35	45209	Salto	108.471
SP	35	45308	Salto de Pirapora	39.224
SP	35	45407	Salto Grande	8.926
SP	35	45506	Sandovalina	3.362
SP	35	45605	Santa Adélia	14.448
SP	35	45704	Santa Albertina	5.052
SP	35	45803	Santa Bárbara d'Oeste	187.908
SP	35	46009	Santa Branca	13.811
SP	35	46108	Santa Clara d'Oeste	2.141
SP	35	46207	Santa Cruz da Conceição	4.099
SP	35	46256	Santa Cruz da Esperança	1.741
SP	35	46306	Santa Cruz das Palmeiras	32.861
SP	35	46405	Santa Cruz do Rio Pardo	43.279
SP	35	46504	Santa Ernestina	5.634
SP	35	46603	Santa Fé do Sul	28.966
SP	35	46702	Santa Gertrudes	20.568
SP	35	46801	Santa Isabel	46.645
SP	35	46900	Santa Lúcia	8.133
SP	35	47007	Santa Maria da Serra	5.809
SP	35	47106	Santa Mercedes	2.617
SP	35	47403	Santa Rita d'Oeste	2.521
SP	35	47502	Santa Rita do Passa Quatro	27.447
SP	35	47601	Santa Rosa de Viterbo	23.824
SP	35	47650	Santa Salete	1.441
SP	35	47205	Santana da Ponte Pensa	1.643
SP	35	47304	Santana de Parnaíba	110.730
SP	35	47700	Santo Anastácio	21.195
SP	35	47809	Santo André	671.696
SP	35	47908	Santo Antônio da Alegria	6.296
SP	35	48005	Santo Antônio de Posse	20.973
SP	35	48054	Santo Antônio do Aracanguá	7.303
SP	35	48104	Santo Antônio do Jardim	5.824
SP	35	48203	Santo Antônio do Pinhal	6.849
SP	35	48302	Santo Expedito	2.918
SP	35	48401	Santópolis do Aguapeí	4.199
SP	35	48500	Santos	417.518

U.F.		MUNICÍPIOS		POPULAÇÃO ESTIMADA
SIGLA	COD	COD	NOMES	
SP	35	48609	São Bento do Sapucaí	10.918
SP	35	48708	São Bernardo do Campo	801.580
SP	35	48807	São Caetano do Sul	151.103
SP	35	48906	São Carlos	218.080
SP	35	49003	São Francisco	2.893
SP	35	49102	São João da Boa Vista	83.369
SP	35	49201	São João das Duas Pontes	2.635
SP	35	49250	São João de Iracema	1.798
SP	35	49300	São João do Pau d'Alho	2.191
SP	35	49409	São Joaquim da Barra	45.782
SP	35	49508	São José da Bela Vista	8.479
SP	35	49607	São José do Barreiro	4.461
SP	35	49706	São José do Rio Pardo	53.025
SP	35	49805	São José do Rio Preto	414.272
SP	35	49904	São José dos Campos	609.229
SP	35	49953	São Lourenço da Serra	17.763
SP	35	50001	São Luís do Paraitinga	10.872
SP	35	50100	São Manuel	39.434
SP	35	50209	São Miguel Arcanjo	31.304
SP	35	50308	São Paulo	10.990.249
SP	35	50407	São Pedro	31.257
SP	35	50506	São Pedro do Turvo	7.393
SP	35	50605	São Roque	67.669
SP	35	50704	São Sebastião	72.236
SP	35	50803	São Sebastião da Grama	12.951
SP	35	50902	São Simão	14.280
SP	35	51009	São Vicente	328.522
SP	35	51108	Sarapuí	8.534
SP	35	51207	Sarutaiá	3.789
SP	35	51306	Sebastianópolis do Sul	3.059
SP	35	51405	Serra Azul	9.880
SP	35	51603	Serra Negra	25.741
SP	35	51504	Serrana	38.956
SP	35	51702	Sertãozinho	109.565
SP	35	51801	Sete Barras	13.211
SP	35	51900	Severínia	15.523
SP	35	52007	Silveiras	5.803
SP	35	52106	Socorro	34.312
SP	35	52205	Sorocaba	576.312
SP	35	52304	Sud Mennucci	8.075
SP	35	52403	Sumaré	237.135

U.F.		MUNICÍPIOS		POPULAÇÃO ESTIMADA
SIGLA	COD	COD	NOMES	
SP	35	52551	Suzanápolis	3.714
SP	35	52502	Suzano	279.394
SP	35	52601	Tabapuã	11.851
SP	35	52700	Tabatinga	14.712
SP	35	52809	Taboão da Serra	224.757
SP	35	52908	Taciba	5.637
SP	35	53005	Taguaí	10.542
SP	35	53104	Taiaçu	6.053
SP	35	53203	Taiúva	5.509
SP	35	53302	Tambaú	22.564
SP	35	53401	Tanabi	24.424
SP	35	53500	Tapiraí	8.052
SP	35	53609	Tapiratiba	12.469
SP	35	53658	Taquaral	2.953
SP	35	53708	Taquaritinga	55.372
SP	35	53807	Taquarituba	22.978
SP	35	53856	Taquarivaí	5.446
SP	35	53906	Tarabai	6.406
SP	35	53955	Tarumã	13.151
SP	35	54003	Tatuí	107.651
SP	35	54102	Taubaté	270.918
SP	35	54201	Tejupá	5.096
SP	35	54300	Teodoro Sampaio	21.107
SP	35	54409	Terra Roxa	8.545
SP	35	54508	Tietê	35.821
SP	35	54607	Timburi	2.567
SP	35	54656	Torre de Pedra	3.058
SP	35	54706	Torrinha	9.245
SP	35	54755	Trabiju	1.507
SP	35	54805	Tremembé	40.601
SP	35	54904	Três Fronteiras	5.167
SP	35	54953	Tuiuti	6.165
SP	35	55000	Tupã	64.078
SP	35	55109	Tupi Paulista	14.298
SP	35	55208	Turiúba	2.028
SP	35	55307	Turmalina	1.998
SP	35	55356	Ubarana	4.799
SP	35	55406	Ubatuba	79.834
SP	35	55505	Ubirajara	4.446
SP	35	55604	Uchoa	9.753
SP	35	55703	União Paulista	1.508

U.F.		MUNICÍPIOS		POPULAÇÃO ESTIMADA
SIGLA	COD	COD	NOMES	
SP	35	55802	Urânia	8.998
SP	35	55901	Uru	1.397
SP	35	56008	Urupês	12.347
SP	35	56107	Valentim Gentil	9.952
SP	35	56206	Valinhos	105.282
SP	35	56305	Valparaíso	22.165
SP	35	56354	Vargem	7.092
SP	35	56404	Vargem Grande do Sul	38.925
SP	35	56453	Vargem Grande Paulista	43.664
SP	35	56503	Várzea Paulista	105.954
SP	35	56602	Vera Cruz	10.065
SP	35	56701	Vinhedo	62.240
SP	35	56800	Viradouro	17.924
SP	35	56909	Vista Alegre do Alto	6.682
SP	35	56958	Vitória Brasil	1.664
SP	35	57006	Votorantim	104.413
SP	35	57105	Votuporanga	80.819
SP	35	57154	Zacarias	2.384
PR	41	00103	Abatiá	7.965
PR	41	00202	Adrianópolis	6.875
PR	41	00301	Agudos do Sul	8.601
PR	41	00400	Almirante Tamandaré	96.739
PR	41	00459	Altamira do Paraná	4.101
PR	41	28625	Alto Paraíso	3.273
PR	41	00608	Alto Paraná	13.379
PR	41	00707	Alto Piquiri	10.448
PR	41	00509	Altônia	20.638
PR	41	00806	Alvorada do Sul	9.262
PR	41	00905	Amaporã	5.378
PR	41	01002	Ampére	17.830
PR	41	01051	Anahy	2.937
PR	41	01101	Andirá	21.953
PR	41	01150	Ângulo	2.890
PR	41	01200	Antonina	17.891
PR	41	01309	Antônio Olinto	7.725
PR	41	01408	Apucarana	120.133
PR	41	01507	Arapongas	101.467
PR	41	01606	Arapoti	26.730
PR	41	01655	Arapuã	4.035
PR	41	01705	Araruna	12.984
PR	41	01804	Araucária	115.849

U.F.		MUNICÍPIOS		POPULAÇÃO ESTIMADA
SIGLA	COD	COD	NOMES	
PR	41	01853	Ariranha do Ivaí	2.568
PR	41	01903	Assaí	16.306
PR	41	02000	Assis Chateaubriand	33.077
PR	41	02109	Astorga	25.042
PR	41	02208	Atalaia	3.682
PR	41	02307	Balsa Nova	11.118
PR	41	02406	Bandeirantes	33.089
PR	41	02505	Barbosa Ferraz	14.017
PR	41	02703	Barra do Jacaré	2.850
PR	41	02604	Barracão	9.275
PR	41	02752	Bela Vista da Caroba	4.210
PR	41	02802	Bela Vista do Paraíso	15.465
PR	41	02901	Bituruna	16.716
PR	41	03008	Boa Esperança	4.785
PR	41	03024	Boa Esperança do Iguaçu	2.919
PR	41	03040	Boa Ventura de São Roque	6.952
PR	41	03057	Boa Vista da Aparecida	7.972
PR	41	03107	Bocaiúva do Sul	9.910
PR	41	03156	Bom Jesus do Sul	3.907
PR	41	03206	Bom Sucesso	6.634
PR	41	03222	Bom Sucesso do Sul	3.107
PR	41	03305	Borrazópolis	8.355
PR	41	03354	Braganey	6.213
PR	41	03370	Brasilândia do Sul	3.321
PR	41	03404	Cafeara	2.821
PR	41	03453	Cafelândia	13.776
PR	41	03479	Cafezal do Sul	4.348
PR	41	03503	Califórnia	7.765
PR	41	03602	Cambará	24.902
PR	41	03701	Cambé	96.555
PR	41	03800	Cambira	7.106
PR	41	03909	Campina da Lagoa	16.329
PR	41	03958	Campina do Simão	4.284
PR	41	04006	Campina Grande do Sul	36.644
PR	41	04105	Campo do Tenente	6.685
PR	41	04055	Campo Bonito	4.458
PR	41	04204	Campo Largo	110.796
PR	41	04253	Campo Magro	23.328
PR	41	04303	Campo Mourão	85.460
PR	41	04402	Cândido de Abreu	18.179
PR	41	04428	Candói	16.090

U.F.		MUNICÍPIOS		POPULAÇÃO ESTIMADA
SIGLA	COD	COD	NOMES	
PR	41	04451	Cantagalo	12.750
PR	41	04501	Capanema	18.655
PR	41	04600	Capitão Leônidas Marques	13.929
PR	41	04659	Carambeí	17.301
PR	41	04709	Carlópolis	13.566
PR	41	04808	Cascavel	291.747
PR	41	04907	Castro	67.708
PR	41	05003	Catanduvas	9.751
PR	41	05102	Centenário do Sul	11.515
PR	41	05201	Cerro Azul	18.460
PR	41	05300	Céu Azul	11.332
PR	41	05409	Chopinzinho	19.628
PR	41	05508	Cianorte	67.637
PR	41	05607	Cidade Gaúcha	10.944
PR	41	05706	Clevelândia	18.041
PR	41	05805	Colombo	241.505
PR	41	05904	Colorado	21.729
PR	41	06001	Congonhinhas	8.931
PR	41	06100	Conselheiro Mairinck	3.681
PR	41	06209	Contenda	15.509
PR	41	06308	Corbélia	15.858
PR	41	06407	Cornélio Procópio	48.427
PR	41	06456	Coronel Domingos Soares	7.790
PR	41	06506	Coronel Vivida	21.985
PR	41	06555	Corumbataí do Sul	4.291
PR	41	06803	Cruz Machado	19.011
PR	41	06571	Cruzeiro do Iguaçu	4.244
PR	41	06605	Cruzeiro do Oeste	20.814
PR	41	06704	Cruzeiro do Sul	4.595
PR	41	06852	Cruzmaltina	3.161
PR	41	06902	Curitiba	1.828.092
PR	41	07009	Curiúva	15.013
PR	41	07108	Diamante do Norte	5.713
PR	41	07124	Diamante do Sul	3.782
PR	41	07157	Diamante D'Oeste	5.110
PR	41	07207	Dois Vizinhos	35.389
PR	41	07256	Douradina	6.794
PR	41	07306	Doutor Camargo	5.760
PR	41	28633	Doutor Ulysses	6.137
PR	41	07405	Enéas Marques	6.100
PR	41	07504	Engenheiro Beltrão	14.273

U.F.		MUNICÍPIOS		POPULAÇÃO ESTIMADA
SIGLA	COD	COD	NOMES	
PR	41	07538	Entre Rios do Oeste	4.043
PR	41	07520	Esperança Nova	1.882
PR	41	07546	Espigão Alto do Iguaçu	5.222
PR	41	07553	Farol	3.413
PR	41	07603	Faxinal	16.006
PR	41	07652	Fazenda Rio Grande	79.255
PR	41	07702	Fênix	5.014
PR	41	07736	Fernandes Pinheiro	5.763
PR	41	07751	Figueira	8.544
PR	41	07850	Flor da Serra do Sul	4.775
PR	41	07801	Floraí	5.174
PR	41	07900	Floresta	5.394
PR	41	08007	Florestópolis	11.841
PR	41	08106	Flórida	2.528
PR	41	08205	Formosa do Oeste	7.581
PR	41	08304	Foz do Iguaçu	319.189
PR	41	08452	Foz do Jordão	5.932
PR	41	08320	Francisco Alves	6.447
PR	41	08403	Francisco Beltrão	75.517
PR	41	08502	General Carneiro	15.159
PR	41	08551	Godoy Moreira	3.639
PR	41	08601	Goioerê	29.732
PR	41	08650	Goioxim	8.232
PR	41	08700	Grandes Rios	7.848
PR	41	08809	Guaíra	29.594
PR	41	08908	Guairaçá	5.875
PR	41	08957	Guamiranga	7.851
PR	41	09005	Guapirama	3.688
PR	41	09104	Guaporema	2.251
PR	41	09203	Guaraci	5.163
PR	41	09302	Guaraniaçu	16.271
PR	41	09401	Guarapuava	171.230
PR	41	09500	Guaraqueçaba	7.890
PR	41	09609	Guaratuba	32.315
PR	41	09658	Honório Serpa	6.251
PR	41	09708	Ibaiti	29.185
PR	41	09757	Ibema	6.124
PR	41	09807	Ibiporã	47.052
PR	41	09906	Icaraíma	9.374
PR	41	10003	Iguaraçu	3.882
PR	41	10052	Iguatu	2.363

U.F.		MUNICÍPIOS		POPULAÇÃO ESTIMADA
SIGLA	COD	COD	NOMES	
PR	41	10078	Imbaú	11.811
PR	41	10102	Imbituva	28.295
PR	41	10201	Inácio Martins	11.397
PR	41	10300	Inajá	2.882
PR	41	10409	Indianópolis	4.257
PR	41	10508	Ipiranga	14.542
PR	41	10607	Iporã	15.353
PR	41	10656	Iracema do Oeste	2.604
PR	41	10706	Irati	56.143
PR	41	10805	Iretama	11.503
PR	41	10904	Itaguajé	4.610
PR	41	10953	Itaipulândia	9.122
PR	41	11001	Itambaracá	7.130
PR	41	11100	Itambé	6.075
PR	41	11209	Itapejara d'Oeste	11.083
PR	41	11258	Itaperuçu	23.132
PR	41	11308	Itaúna do Sul	3.701
PR	41	11407	Ivaí	13.392
PR	41	11506	Ivaiporã	32.193
PR	41	11555	Ivaté	8.173
PR	41	11605	Ivatuba	2.789
PR	41	11704	Jaboti	5.245
PR	41	11803	Jacarezinho	40.526
PR	41	11902	Jaguapitã	12.286
PR	41	12009	Jaguariaíva	33.041
PR	41	12108	Jandaia do Sul	20.131
PR	41	12207	Janiópolis	7.092
PR	41	12306	Japira	4.810
PR	41	12405	Japurá	8.585
PR	41	12504	Jardim Alegre	14.867
PR	41	12603	Jardim Olinda	1.498
PR	41	12702	Jataizinho	11.587
PR	41	12751	Jesuítas	8.948
PR	41	12801	Joaquim Távora	10.662
PR	41	12900	Jundiaí do Sul	3.769
PR	41	12959	Juranda	7.858
PR	41	13007	Jussara	6.250
PR	41	13106	Kaloré	4.752
PR	41	13205	Lapa	42.906
PR	41	13254	Laranjal	6.410
PR	41	13304	Laranjeiras do Sul	31.516

U.F.		MUNICÍPIOS		POPULAÇÃO ESTIMADA
SIGLA	COD	COD	NOMES	
PR	41	13403	Leópolis	4.331
PR	41	13429	Lidianópolis	4.151
PR	41	13452	Lindoeste	5.498
PR	41	13502	Loanda	20.067
PR	41	13601	Lobato	4.377
PR	41	13700	Londrina	505.184
PR	41	13734	Luiziana	7.380
PR	41	13759	Lunardelli	5.152
PR	41	13809	Lupionópolis	4.521
PR	41	13908	Mallet	12.778
PR	41	14005	Mamborê	14.421
PR	41	14104	Mandaguaçu	19.058
PR	41	14203	Mandaguari	32.976
PR	41	14302	Mandirituba	21.498
PR	41	14351	Manfrinópolis	3.334
PR	41	14401	Mangueirinha	17.562
PR	41	14500	Manoel Ribas	13.119
PR	41	14609	Marechal Cândido Rondon	46.523
PR	41	14708	Maria Helena	6.145
PR	41	14807	Marialva	31.171
PR	41	14906	Marilândia do Sul	9.193
PR	41	15002	Marilena	6.715
PR	41	15101	Mariluz	10.817
PR	41	15200	Maringá	331.412
PR	41	15309	Mariópolis	5.956
PR	41	15358	Maripá	5.698
PR	41	15408	Marmeleiro	13.493
PR	41	15457	Marquinho	5.300
PR	41	15507	Marumbi	4.200
PR	41	15606	Matelândia	16.056
PR	41	15705	Matinhos	23.968
PR	41	15739	Mato Rico	4.235
PR	41	15754	Mauá da Serra	8.268
PR	41	15804	Medianeira	39.700
PR	41	15853	Mercedes	4.879
PR	41	15903	Mirador	2.385
PR	41	16000	Miraselva	1.949
PR	41	16059	Missal	10.739
PR	41	16109	Moreira Sales	13.263
PR	41	16208	Morretes	16.853
PR	41	16307	Munhoz de Melo	3.688

U.F.		MUNICÍPIOS		POPULAÇÃO ESTIMADA
SIGLA	COD	COD	NOMES	
PR	41	16406	Nossa Senhora das Graças	4.038
PR	41	16505	Nova Aliança do Ivaí	1.426
PR	41	16604	Nova América da Colina	3.358
PR	41	16703	Nova Aurora	11.833
PR	41	16802	Nova Cantu	7.714
PR	41	16901	Nova Esperança	26.532
PR	41	16950	Nova Esperança do Sudoeste	5.334
PR	41	17008	Nova Fátima	8.270
PR	41	17057	Nova Laranjeiras	11.598
PR	41	17107	Nova Londrina	12.933
PR	41	17206	Nova Olímpia	5.385
PR	41	17255	Nova Prata do Iguaçu	10.791
PR	41	17214	Nova Santa Bárbara	3.952
PR	41	17222	Nova Santa Rosa	7.893
PR	41	17271	Nova Tebas	8.401
PR	41	17297	Novo Itacolomi	2.816
PR	41	17305	Ortigueira	25.043
PR	41	17404	Ourizona	3.384
PR	41	17453	Ouro Verde do Oeste	5.636
PR	41	17503	Paiçandu	36.336
PR	41	17602	Palmas	42.643
PR	41	17701	Palmeira	32.282
PR	41	17800	Palmital	15.699
PR	41	17909	Palotina	28.691
PR	41	18006	Paraíso do Norte	11.743
PR	41	18105	Paranacity	9.876
PR	41	18204	Paranaguá	138.748
PR	41	18303	Paranapoema	2.781
PR	41	18402	Paranavaí	82.133
PR	41	18451	Pato Bragado	4.867
PR	41	18501	Pato Branco	69.478
PR	41	18600	Paula Freitas	5.691
PR	41	18709	Paulo Frontin	7.327
PR	41	18808	Peabiru	13.371
PR	41	18857	Perobal	5.179
PR	41	18907	Pérola	9.637
PR	41	19004	Pérola d'Oeste	7.222
PR	41	19103	Piên	11.632
PR	41	19152	Pinhais	116.984
PR	41	19251	Pinhal de São Bento	2.598
PR	41	19202	Pinhalão	6.030

U.F.		MUNICÍPIOS		POPULAÇÃO ESTIMADA
SIGLA	COD	COD	NOMES	
PR	41	19301	Pinhão	30.144
PR	41	19400	Piraí do Sul	24.139
PR	41	19509	Piraquara	86.012
PR	41	19608	Pitanga	35.155
PR	41	19657	Pitangueiras	2.786
PR	41	19707	Planaltina do Paraná	3.879
PR	41	19806	Planalto	14.007
PR	41	19905	Ponta Grossa	311.106
PR	41	19954	Pontal do Paraná	17.508
PR	41	20002	Porecatu	14.358
PR	41	20101	Porto Amazonas	4.341
PR	41	20150	Porto Barreiro	3.812
PR	41	20200	Porto Rico	2.526
PR	41	20309	Porto Vitória	3.856
PR	41	20333	Prado Ferreira	3.480
PR	41	20358	Pranchita	5.925
PR	41	20408	Presidente Castelo Branco	4.879
PR	41	20507	Primeiro de Maio	11.098
PR	41	20606	Prudentópolis	50.614
PR	41	20655	Quarto Centenário	4.927
PR	41	20705	Quatiguá	7.116
PR	41	20804	Quatro Barras	19.002
PR	41	20853	Quatro Pontes	3.789
PR	41	20903	Quedas do Iguaçu	31.572
PR	41	21000	Querência do Norte	12.235
PR	41	21109	Quinta do Sol	5.246
PR	41	21208	Quitandinha	16.497
PR	41	21257	Ramilândia	4.322
PR	41	21307	Rancho Alegre	4.097
PR	41	21356	Rancho Alegre D'Oeste	2.992
PR	41	21406	Realeza	16.276
PR	41	21505	Rebouças	14.558
PR	41	21604	Renascença	6.945
PR	41	21703	Reserva	25.059
PR	41	21752	Reserva do Iguaçu	7.383
PR	41	21901	Ribeirão do Pinhal	13.666
PR	41	21802	Ribeirão Claro	11.223
PR	41	22008	Rio Azul	13.702
PR	41	22107	Rio Bom	3.319
PR	41	22156	Rio Bonito do Iguaçu	15.009
PR	41	22172	Rio Branco do Ivaí	3.986

U.F.		MUNICÍPIOS		POPULAÇÃO ESTIMADA
SIGLA	COD	COD	NOMES	
PR	41	22206	Rio Branco do Sul	32.815
PR	41	22305	Rio Negro	30.985
PR	41	22404	Rolândia	55.750
PR	41	22503	Roncador	12.442
PR	41	22602	Rondon	9.385
PR	41	22651	Rosário do Ivaí	5.890
PR	41	22701	Sabáudia	5.625
PR	41	22800	Salgado Filho	4.709
PR	41	22909	Salto do Itararé	5.177
PR	41	23006	Salto do Lontra	12.832
PR	41	23105	Santa Amélia	4.137
PR	41	23204	Santa Cecília do Pavão	3.732
PR	41	23303	Santa Cruz de Monte Castelo	8.074
PR	41	23402	Santa Fé	10.224
PR	41	23501	Santa Helena	23.872
PR	41	23600	Santa Inês	1.904
PR	41	23709	Santa Isabel do Ivaí	8.678
PR	41	23808	Santa Izabel do Oeste	11.753
PR	41	23824	Santa Lúcia	3.781
PR	41	23857	Santa Maria do Oeste	11.590
PR	41	23907	Santa Mariana	12.143
PR	41	23956	Santa Mônica	3.603
PR	41	24020	Santa Tereza do Oeste	9.462
PR	41	24053	Santa Terezinha de Itaipu	20.354
PR	41	24004	Santana do Itararé	5.696
PR	41	24103	Santo Antônio da Platina	41.844
PR	41	24202	Santo Antônio do Caiuá	2.748
PR	41	24301	Santo Antônio do Paraíso	2.361
PR	41	24400	Santo Antônio do Sudoeste	19.260
PR	41	24509	Santo Inácio	4.982
PR	41	24608	São Carlos do Ivaí	5.987
PR	41	24707	São Jerônimo da Serra	11.900
PR	41	24806	São João	11.198
PR	41	24905	São João do Caiuá	6.151
PR	41	25001	São João do Ivaí	12.022
PR	41	25100	São João do Triunfo	14.226
PR	41	25209	São Jorge d'Oeste	9.213
PR	41	25308	São Jorge do Ivaí	5.406
PR	41	25357	São Jorge do Patrocínio	6.134
PR	41	25407	São José da Boa Vista	6.386
PR	41	25456	São José das Palmeiras	3.960

U.F.		MUNICÍPIOS		POPULAÇÃO ESTIMADA
SIGLA	COD	COD	NOMES	
PR	41	25506	São José dos Pinhais	272.530
PR	41	25555	São Manoel do Paraná	2.163
PR	41	25605	São Mateus do Sul	40.791
PR	41	25704	São Miguel do Iguaçu	26.284
PR	41	25753	São Pedro do Iguaçu	6.633
PR	41	25803	São Pedro do Ivaí	9.887
PR	41	25902	São Pedro do Paraná	2.580
PR	41	26009	São Sebastião da Amoreira	8.976
PR	41	26108	São Tomé	5.482
PR	41	26207	Sapopema	6.812
PR	41	26256	Sarandi	83.486
PR	41	26272	Saudade do Iguaçu	5.137
PR	41	26306	Sengés	20.213
PR	41	26355	Serranópolis do Iguaçu	4.400
PR	41	26405	Sertaneja	5.984
PR	41	26504	Sertanópolis	16.027
PR	41	26603	Siqueira Campos	17.293
PR	41	26652	Sulina	3.481
PR	41	26678	Tamarana	11.413
PR	41	26702	Tamboara	4.757
PR	41	26801	Tapejara	15.170
PR	41	26900	Tapira	5.944
PR	41	27007	Teixeira Soares	10.337
PR	41	27106	Telêmaco Borba	68.584
PR	41	27205	Terra Boa	15.041
PR	41	27304	Terra Rica	14.955
PR	41	27403	Terra Roxa	16.707
PR	41	27502	Tibagi	19.345
PR	41	27601	Tijucas do Sul	13.633
PR	41	27700	Toledo	115.136
PR	41	27809	Tomazina	8.920
PR	41	27858	Três Barras do Paraná	12.073
PR	41	27882	Tunas do Paraná	6.465
PR	41	27908	Tuneiras do Oeste	8.806
PR	41	27957	Tupãssi	7.960
PR	41	27965	Turvo	14.391
PR	41	28005	Ubiratã	21.672
PR	41	28104	Umuarama	98.855
PR	41	28203	União da Vitória	53.048
PR	41	28302	Uniflor	2.484
PR	41	28401	Uraí	11.793

U.F.		MUNICÍPIOS		POPULAÇÃO ESTIMADA
SIGLA	COD	COD	NOMES	
PR	41	28534	Ventania	10.948
PR	41	28559	Vera Cruz do Oeste	9.301
PR	41	28609	Verê	8.144
PR	41	28658	Virmond	4.163
PR	41	28708	Vitorino	6.513
PR	41	28500	Wenceslau Braz	19.149
PR	41	28807	Xambrê	5.896
SC	42	00051	Abdon Batista	2.805
SC	42	00101	Abelardo Luz	16.870
SC	42	00200	Agrolândia	9.496
SC	42	00309	Agronômica	4.866
SC	42	00408	Água Doce	6.954
SC	42	00507	Águas de Chapecó	6.306
SC	42	00556	Águas Frias	2.632
SC	42	00606	Águas Mornas	4.520
SC	42	00705	Alfredo Wagner	10.150
SC	42	00754	Alto Bela Vista	2.075
SC	42	00804	Anchieta	6.730
SC	42	00903	Angelina	5.436
SC	42	01000	Anita Garibaldi	9.299
SC	42	01109	Anitápolis	3.266
SC	42	01208	Antônio Carlos	7.375
SC	42	01257	Apiúna	10.774
SC	42	01273	Arabutã	4.062
SC	42	01307	Araquari	22.467
SC	42	01406	Araranguá	59.134
SC	42	01505	Armazém	7.584
SC	42	01604	Arroio Trinta	3.627
SC	42	01653	Arvoredo	2.249
SC	42	01703	Ascurra	6.950
SC	42	01802	Atalanta	3.407
SC	42	01901	Aurora	5.556
SC	42	01950	Balneário Arroio do Silva	8.558
SC	42	02057	Balneário Barra do Sul	7.634
SC	42	02008	Balneário Camboriú	99.493
SC	42	02073	Balneário Gaivota	7.732
SC	42	12809	Balneário Piçarras	14.490
SC	42	02081	Bandeirante	3.105
SC	42	02099	Barra Bonita	2.122
SC	42	02107	Barra Velha	19.474
SC	42	02131	Bela Vista do Toldo	6.111

U.F.		MUNICÍPIOS		POPULAÇÃO ESTIMADA
SIGLA	COD	COD	NOMES	
SC	42	02156	Belmonte	2.774
SC	42	02206	Benedito Novo	10.226
SC	42	02305	Biguaçu	55.665
SC	42	02404	Blumenau	296.151
SC	42	02438	Bocaina do Sul	3.131
SC	42	02503	Bom Jardim da Serra	4.359
SC	42	02537	Bom Jesus	2.394
SC	42	02578	Bom Jesus do Oeste	2.075
SC	42	02602	Bom Retiro	8.543
SC	42	02453	Bombinhas	13.241
SC	42	02701	Botuverá	4.294
SC	42	02800	Braço do Norte	28.909
SC	42	02859	Braço do Trombudo	3.400
SC	42	02875	Brunópolis	2.981
SC	42	02909	Brusque	99.917
SC	42	03006	Caçador	70.088
SC	42	03105	Caibi	6.393
SC	42	03154	Calmon	4.194
SC	42	03204	Camboriú	56.315
SC	42	03303	Campo Alegre	11.715
SC	42	03402	Campo Belo do Sul	8.204
SC	42	03501	Campo Erê	9.802
SC	42	03600	Campos Novos	29.199
SC	42	03709	Canelinha	10.068
SC	42	03808	Canoinhas	54.408
SC	42	03253	Capão Alto	3.329
SC	42	03907	Capinzal	18.994
SC	42	03956	Capivari de Baixo	20.843
SC	42	04004	Catanduvas	9.049
SC	42	04103	Caxambu do Sul	4.995
SC	42	04152	Celso Ramos	2.734
SC	42	04178	Cerro Negro	4.053
SC	42	04194	Chapadão do Lageado	2.854
SC	42	04202	Chapecó	171.789
SC	42	04251	Cocal do Sul	15.101
SC	42	04301	Concórdia	69.766
SC	42	04350	Cordilheira Alta	3.493
SC	42	04400	Coronel Freitas	10.530
SC	42	04459	Coronel Martins	2.567
SC	42	04558	Correia Pinto	15.058
SC	42	04509	Corupá	13.248

U.F.		MUNICÍPIOS		POPULAÇÃO ESTIMADA
SIGLA	COD	COD	NOMES	
SC	42	04608	Criciúma	187.018
SC	42	04707	Cunha Porã	11.009
SC	42	04756	Cunhataí	1.938
SC	42	04806	Curitibanos	38.799
SC	42	04905	Descanso	8.927
SC	42	05001	Dionísio Cerqueira	15.305
SC	42	05100	Dona Emma	3.560
SC	42	05159	Doutor Pedrinho	3.402
SC	42	05175	Entre Rios	3.083
SC	42	05191	Ermo	1.877
SC	42	05209	Erval Velho	4.205
SC	42	05308	Faxinal dos Guedes	10.611
SC	42	05357	Flor do Sertão	1.694
SC	42	05407	Florianópolis	402.346
SC	42	05431	Formosa do Sul	2.689
SC	42	05456	Forquilhinha	21.611
SC	42	05506	Fraiburgo	36.170
SC	42	05555	Frei Rogério	2.703
SC	42	05605	Galvão	3.520
SC	42	05704	Garopaba	16.577
SC	42	05803	Garuva	14.022
SC	42	05902	Gaspar	54.687
SC	42	06009	Governador Celso Ramos	12.611
SC	42	06108	Grão Pará	6.250
SC	42	06207	Gravatal	10.802
SC	42	06306	Guabiruba	16.925
SC	42	06405	Guaraciaba	10.883
SC	42	06504	Guaramirim	31.334
SC	42	06603	Guarujá do Sul	4.858
SC	42	06652	Guatambú	4.622
SC	42	06702	Herval d'Oeste	19.405
SC	42	06751	Ibiam	2.052
SC	42	06801	Ibicaré	3.473
SC	42	06900	Ibirama	17.328
SC	42	07007	Içara	56.360
SC	42	07106	Ilhota	12.012
SC	42	07205	Imaruí	11.847
SC	42	07304	Imbituba	38.574
SC	42	07403	Imbuia	5.697
SC	42	07502	Indaial	49.959
SC	42	07577	Iomerê	2.637

U.F.		MUNICÍPIOS		POPULAÇÃO ESTIMADA
SIGLA	COD	COD	NOMES	
SC	42	07601	Ipira	4.807
SC	42	07650	Iporã do Oeste	8.362
SC	42	07684	Ipuaçu	6.816
SC	42	07700	Ipumirim	7.360
SC	42	07759	Iraceminha	4.356
SC	42	07809	Irani	9.664
SC	42	07858	Irati	2.059
SC	42	07908	Irineópolis	10.662
SC	42	08005	Itá	6.577
SC	42	08104	Itaiópolis	20.431
SC	42	08203	Itajaí	169.927
SC	42	08302	Itapema	35.655
SC	42	08401	Itapiranga	15.840
SC	42	08450	Itapoá	11.251
SC	42	08500	Ituporanga	21.327
SC	42	08609	Jaborá	4.138
SC	42	08708	Jacinto Machado	11.048
SC	42	08807	Jaguaruna	16.263
SC	42	08906	Jaraguá do Sul	136.282
SC	42	08955	Jardinópolis	1.893
SC	42	09003	Joaçaba	25.226
SC	42	09102	Joinville	492.101
SC	42	09151	José Boiteux	5.015
SC	42	09177	Jupiá	2.190
SC	42	09201	Lacerdópolis	2.260
SC	42	09300	Lages	166.983
SC	42	09409	Laguna	51.282
SC	42	09458	Lajeado Grande	1.494
SC	42	09508	Laurentino	5.697
SC	42	09607	Lauro Muller	14.131
SC	42	09706	Lebon Régis	12.102
SC	42	09805	Leoberto Leal	3.683
SC	42	09854	Lindóia do Sul	4.676
SC	42	09904	Lontras	9.549
SC	42	10001	Luiz Alves	9.371
SC	42	10035	Luzerna	5.537
SC	42	10050	Macieira	1.799
SC	42	10100	Mafra	52.697
SC	42	10209	Major Gercino	2.897
SC	42	10308	Major Vieira	7.609
SC	42	10407	Maracajá	6.130

U.F.		MUNICÍPIOS		POPULAÇÃO ESTIMADA
SIGLA	COD	COD	NOMES	
SC	42	10506	Maravilha	22.691
SC	42	10555	Marema	2.312
SC	42	10605	Massaranduba	14.331
SC	42	10704	Matos Costa	2.863
SC	42	10803	Meleiro	7.070
SC	42	10852	Mirim Doce	2.601
SC	42	10902	Modelo	3.871
SC	42	11009	Mondaí	9.449
SC	42	11058	Monte Carlo	9.125
SC	42	11108	Monte Castelo	8.337
SC	42	11207	Morro da Fumaça	15.994
SC	42	11256	Morro Grande	2.790
SC	42	11306	Navegantes	55.691
SC	42	11405	Nova Erechim	4.307
SC	42	11454	Nova Itaberaba	4.229
SC	42	11504	Nova Trento	11.832
SC	42	11603	Nova Veneza	13.032
SC	42	11652	Novo Horizonte	2.970
SC	42	11702	Orleans	21.590
SC	42	11751	Otacílio Costa	16.359
SC	42	11801	Ouro	7.266
SC	42	11850	Ouro Verde	2.197
SC	42	11876	Paial	1.852
SC	42	11892	Painel	2.360
SC	42	11900	Palhoça	128.360
SC	42	12007	Palma Sola	8.157
SC	42	12056	Palmeira	2.428
SC	42	12106	Palmitos	16.557
SC	42	12205	Papanduva	17.605
SC	42	12239	Paraíso	4.259
SC	42	12254	Passo de Torres	5.575
SC	42	12270	Passos Maia	4.578
SC	42	12304	Paulo Lopes	7.198
SC	42	12403	Pedras Grandes	4.479
SC	42	12502	Penha	21.853
SC	42	12601	Peritiba	3.003
SC	42	12700	Petrolândia	6.213
SC	42	12908	Pinhalzinho	15.394
SC	42	13005	Pinheiro Preto	3.021
SC	42	13104	Piratuba	4.577
SC	42	13153	Planalto Alegre	2.740

U.F.		MUNICÍPIOS		POPULAÇÃO ESTIMADA
SIGLA	COD	COD	NOMES	
SC	42	13203	Pomerode	26.375
SC	42	13302	Ponte Alta	5.226
SC	42	13351	Ponte Alta do Norte	3.637
SC	42	13401	Ponte Serrada	11.625
SC	42	13500	Porto Belo	13.910
SC	42	13609	Porto União	33.290
SC	42	13708	Pouso Redondo	14.307
SC	42	13807	Praia Grande	7.321
SC	42	13906	Presidente Castello Branco	1.794
SC	42	14003	Presidente Getúlio	14.212
SC	42	14102	Presidente Nereu	2.324
SC	42	14151	Princesa	2.682
SC	42	14201	Quilombo	11.220
SC	42	14300	Rancho Queimado	2.871
SC	42	14409	Rio das Antas	6.232
SC	42	14508	Rio do Campo	6.176
SC	42	14607	Rio do Oeste	7.010
SC	42	14805	Rio do Sul	59.236
SC	42	14706	Rio dos Cedros	10.063
SC	42	14904	Rio Fortuna	4.621
SC	42	15000	Rio Negrinho	44.023
SC	42	15059	Rio Rufino	2.510
SC	42	15075	Riqueza	5.134
SC	42	15109	Rodeio	11.146
SC	42	15208	Romelândia	5.833
SC	42	15307	Salete	7.690
SC	42	15356	Saltinho	4.183
SC	42	15406	Salto Veloso	4.328
SC	42	15455	Sangão	10.851
SC	42	15505	Santa Cecília	15.836
SC	42	15554	Santa Helena	2.496
SC	42	15604	Santa Rosa de Lima	2.096
SC	42	15653	Santa Rosa do Sul	8.208
SC	42	15679	Santa Terezinha	9.322
SC	42	15687	Santa Terezinha do Progresso	3.097
SC	42	15695	Santiago do Sul	1.467
SC	42	15703	Santo Amaro da Imperatriz	18.266
SC	42	15802	São Bento do Sul	75.543
SC	42	15752	São Bernardino	2.682
SC	42	15901	São Bonifácio	3.271
SC	42	16008	São Carlos	10.800

U.F.		MUNICÍPIOS		POPULAÇÃO ESTIMADA
SIGLA	COD	COD	NOMES	
SC	42	16057	São Cristovão do Sul	5.037
SC	42	16107	São Domingos	9.612
SC	42	16206	São Francisco do Sul	39.341
SC	42	16354	São João do Itaperiú	3.404
SC	42	16255	São João do Oeste	6.230
SC	42	16404	São João do Sul	7.143
SC	42	16305	São João Batista	23.547
SC	42	16503	São Joaquim	24.929
SC	42	16602	São José	199.280
SC	42	16701	São José do Cedro	14.122
SC	42	16800	São José do Cerrito	10.611
SC	42	16909	São Lourenço do Oeste	22.711
SC	42	17006	São Ludgero	10.740
SC	42	17105	São Martinho	3.283
SC	42	17154	São Miguel da Boa Vista	2.027
SC	42	17204	São Miguel do Oeste	35.004
SC	42	17253	São Pedro de Alcântara	5.038
SC	42	17303	Saudades	8.880
SC	42	17402	Schroeder	13.559
SC	42	17501	Seara	17.716
SC	42	17550	Serra Alta	3.285
SC	42	17600	Siderópolis	12.895
SC	42	17709	Sombrio	25.332
SC	42	17758	Sul Brasil	3.150
SC	42	17808	Taió	17.418
SC	42	17907	Tangará	8.632
SC	42	17956	Tigrinhos	1.780
SC	42	18004	Tijucas	29.122
SC	42	18103	Timbé do Sul	5.270
SC	42	18202	Timbó	34.777
SC	42	18251	Timbó Grande	7.245
SC	42	18301	Três Barras	18.576
SC	42	18350	Treviso	3.644
SC	42	18400	Treze de Maio	6.789
SC	42	18509	Treze Tílias	5.900
SC	42	18608	Trombudo Central	6.458
SC	42	18707	Tubarão	95.855
SC	42	18756	Tunápolis	4.779
SC	42	18806	Turvo	11.386
SC	42	18855	União do Oeste	3.116
SC	42	18905	Urubici	10.780

U.F.		MUNICÍPIOS		POPULAÇÃO ESTIMADA
SIGLA	COD	COD	NOMES	
SC	42	18954	Urupema	2.576
SC	42	19002	Urussanga	19.778
SC	42	19101	Vargeão	3.673
SC	42	19150	Vargem	3.193
SC	42	19176	Vargem Bonita	4.364
SC	42	19200	Vidal Ramos	6.133
SC	42	19309	Videira	46.157
SC	42	19358	Vitor Meireles	5.739
SC	42	19408	Witmarsum	3.556
SC	42	19507	Xanxerê	41.766
SC	42	19606	Xavantina	4.328
SC	42	19705	Xaxim	25.223
SC	42	19853	Zortéa	2.981
RS	43	00034	Aceguá	4.312
RS	43	00059	Água Santa	3.638
RS	43	00109	Agudo	17.114
RS	43	00208	Ajuricaba	7.411
RS	43	00307	Alecrim	7.380
RS	43	00406	Alegrete	79.548
RS	43	00455	Alegria	4.834
RS	43	00471	Almirante Tamandaré do Sul	2.212
RS	43	00505	Alpestre	9.017
RS	43	00554	Alto Alegre	1.965
RS	43	00570	Alto Feliz	3.048
RS	43	00604	Alvorada	211.276
RS	43	00638	Amaral Ferrador	6.527
RS	43	00646	Ametista do Sul	8.440
RS	43	00661	André da Rocha	1.262
RS	43	00703	Anta Gorda	6.331
RS	43	00802	Antônio Prado	14.159
RS	43	00851	Arambaré	3.931
RS	43	00877	Araricá	5.078
RS	43	00901	Aratiba	6.735
RS	43	01008	Arroio do Meio	18.878
RS	43	01073	Arroio do Padre	2.855
RS	43	01057	Arroio do Sal	7.109
RS	43	01206	Arroio do Tigre	13.126
RS	43	01107	Arroio dos Ratos	14.114
RS	43	01305	Arroio Grande	18.801
RS	43	01404	Arvorezinha	10.530
RS	43	01503	Augusto Pestana	7.408

U.F.		MUNICÍPIOS		POPULAÇÃO ESTIMADA
SIGLA	COD	COD	NOMES	
RS	43	01552	Áurea	3.802
RS	43	01602	Bagé	115.755
RS	43	01636	Balneário Pinhal	11.440
RS	43	01651	Barão	5.444
RS	43	01701	Barão de Cotegipe	6.716
RS	43	01750	Barão do Triunfo	7.198
RS	43	01859	Barra do Guarita	3.062
RS	43	01875	Barra do Quaraí	3.878
RS	43	01909	Barra do Ribeiro	11.779
RS	43	01925	Barra do Rio Azul	2.020
RS	43	01958	Barra Funda	2.434
RS	43	01800	Barracão	5.424
RS	43	02006	Barros Cassal	11.839
RS	43	02055	Benjamin Constant do Sul	2.240
RS	43	02105	Bento Gonçalves	105.639
RS	43	02154	Boa Vista das Missões	2.110
RS	43	02204	Boa Vista do Buricá	6.655
RS	43	02220	Boa Vista do Cadeado	2.522
RS	43	02238	Boa Vista do Incra	2.557
RS	43	02253	Boa Vista do Sul	2.715
RS	43	02303	Bom Jesus	12.194
RS	43	02352	Bom Princípio	11.532
RS	43	02378	Bom Progresso	2.446
RS	43	02402	Bom Retiro do Sul	11.555
RS	43	02451	Boqueirão do Leão	8.078
RS	43	02501	Bossoroca	7.880
RS	43	02584	Bozano	2.361
RS	43	02600	Braga	3.878
RS	43	02659	Brochier	4.846
RS	43	02709	Butiá	20.240
RS	43	02808	Caçapava do Sul	33.235
RS	43	02907	Cacequi	13.750
RS	43	03004	Cachoeira do Sul	86.750
RS	43	03103	Cachoeirinha	117.203
RS	43	03202	Cacique Doble	4.990
RS	43	03301	Caibaté	5.217
RS	43	03400	Caiçara	5.276
RS	43	03509	Camaquã	62.556
RS	43	03558	Camargo	2.546
RS	43	03608	Cambará do Sul	7.207
RS	43	03673	Campestre da Serra	3.316

U.F.		MUNICÍPIOS		POPULAÇÃO ESTIMADA
SIGLA	COD	COD	NOMES	
RS	43	03707	Campina das Missões	6.419
RS	43	03806	Campinas do Sul	5.746
RS	43	03905	Campo Bom	58.915
RS	43	04002	Campo Novo	5.545
RS	43	04101	Campos Borges	3.671
RS	43	04200	Candelária	30.369
RS	43	04309	Cândido Godói	6.762
RS	43	04358	Candiota	8.535
RS	43	04408	Canela	40.449
RS	43	04507	Canguçu	55.679
RS	43	04606	Canoas	329.903
RS	43	04614	Canudos do Vale	2.001
RS	43	04630	Capão da Canoa	39.928
RS	43	04655	Capão do Cipó	3.402
RS	43	04663	Capão do Leão	24.408
RS	43	04622	Capão Bonito do Sul	1.882
RS	43	04689	Capela de Santana	11.478
RS	43	04697	Capitão	2.684
RS	43	04671	Capivari do Sul	3.492
RS	43	04713	Caraá	7.500
RS	43	04705	Carazinho	60.182
RS	43	04804	Carlos Barbosa	25.388
RS	43	04853	Carlos Gomes	1.738
RS	43	04903	Casca	8.641
RS	43	04952	Caseiros	3.109
RS	43	05009	Catuípe	9.674
RS	43	05108	Caxias do Sul	405.858
RS	43	05116	Centenário	3.105
RS	43	05124	Cerrito	6.788
RS	43	05132	Cerro Branco	4.642
RS	43	05157	Cerro Grande	2.656
RS	43	05173	Cerro Grande do Sul	9.714
RS	43	05207	Cerro Largo	12.853
RS	43	05306	Chapada	9.687
RS	43	05355	Charqueadas	35.507
RS	43	05371	Charrua	3.658
RS	43	05405	Chiapetta	4.134
RS	43	05439	Chuí	5.470
RS	43	05447	Chuvisca	5.102
RS	43	05454	Cidreira	11.615
RS	43	05504	Ciríaco	5.046

U.F.		MUNICÍPIOS		POPULAÇÃO ESTIMADA
SIGLA	COD	COD	NOMES	
RS	43	05587	Colinas	2.471
RS	43	05603	Colorado	3.803
RS	43	05702	Condor	6.843
RS	43	05801	Constantina	10.165
RS	43	05835	Coqueiro Baixo	1.610
RS	43	05850	Coqueiros do Sul	3.285
RS	43	05871	Coronel Barros	2.518
RS	43	05900	Coronel Bicaco	8.021
RS	43	05934	Coronel Pilar	1.668
RS	43	05959	Cotiporã	4.817
RS	43	05975	Coxilha	2.998
RS	43	06007	Crissiumal	15.116
RS	43	06056	Cristal	7.328
RS	43	06072	Cristal do Sul	3.081
RS	43	06106	Cruz Alta	64.762
RS	43	06130	Cruzaltense	2.294
RS	43	06205	Cruzeiro do Sul	12.661
RS	43	06304	David Canabarro	4.850
RS	43	06320	Derrubadas	3.424
RS	43	06353	Dezesseis de Novembro	2.974
RS	43	06379	Dilermando de Aguiar	3.216
RS	43	06403	Dois Irmãos	26.070
RS	43	06429	Dois Irmãos das Missões	2.438
RS	43	06452	Dois Lajeados	3.462
RS	43	06502	Dom Feliciano	15.151
RS	43	06601	Dom Pedrito	38.952
RS	43	06551	Dom Pedro de Alcântara	2.834
RS	43	06700	Dona Francisca	3.625
RS	43	06734	Doutor Maurício Cardoso	5.513
RS	43	06759	Doutor Ricardo	2.105
RS	43	06767	Eldorado do Sul	33.098
RS	43	06809	Encantado	20.360
RS	43	06908	Encruzilhada do Sul	25.032
RS	43	06924	Engenho Velho	1.530
RS	43	06957	Entre Rios do Sul	3.127
RS	43	06932	Entre-Ijuís	9.312
RS	43	06973	Erebango	2.947
RS	43	07005	Erechim	97.011
RS	43	07054	Ernestina	3.094
RS	43	07203	Erval Grande	5.398
RS	43	07302	Erval Seco	8.294

U.F.		MUNICÍPIOS		POPULAÇÃO ESTIMADA
SIGLA	COD	COD	NOMES	
RS	43	07401	Esmeralda	3.361
RS	43	07450	Esperança do Sul	3.498
RS	43	07500	Espumoso	15.429
RS	43	07559	Estação	6.256
RS	43	07609	Estância Velha	43.123
RS	43	07708	Esteio	81.131
RS	43	07807	Estrela	30.329
RS	43	07815	Estrela Velha	3.772
RS	43	07831	Eugênio de Castro	3.107
RS	43	07864	Fagundes Varela	2.596
RS	43	07906	Farroupilha	62.674
RS	43	08003	Faxinal do Soturno	6.453
RS	43	08052	Faxinalzinho	2.639
RS	43	08078	Fazenda Vilanova	3.212
RS	43	08102	Feliz	12.126
RS	43	08201	Flores da Cunha	26.435
RS	43	08250	Floriano Peixoto	2.176
RS	43	08300	Fontoura Xavier	11.357
RS	43	08409	Formigueiro	7.255
RS	43	08433	Forquetinha	2.617
RS	43	08458	Fortaleza dos Valos	4.671
RS	43	08508	Frederico Westphalen	28.295
RS	43	08607	Garibaldi	30.165
RS	43	08656	Garruchos	3.528
RS	43	08706	Gaurama	6.252
RS	43	08805	General Câmara	9.074
RS	43	08854	Gentil	1.593
RS	43	08904	Getúlio Vargas	16.374
RS	43	09001	Giruá	17.303
RS	43	09050	Glorinha	7.364
RS	43	09100	Gramado	33.257
RS	43	09126	Gramado dos Loureiros	2.414
RS	43	09159	Gramado Xavier	4.084
RS	43	09209	Gravataí	266.230
RS	43	09258	Guabiju	1.708
RS	43	09308	Guaíba	96.467
RS	43	09407	Guaporé	22.372
RS	43	09506	Guarani das Missões	8.475
RS	43	09555	Harmonia	3.777
RS	43	07104	Herval	7.100
RS	43	09571	Herveiras	2.891

U.F.		MUNICÍPIOS		POPULAÇÃO ESTIMADA
SIGLA	COD	COD	NOMES	
RS	43	09605	Horizontina	19.013
RS	43	09654	Hulha Negra	6.352
RS	43	09704	Humaitá	5.024
RS	43	09753	Ibarama	4.448
RS	43	09803	Ibiaçá	4.804
RS	43	09902	Ibiraiaras	7.310
RS	43	09951	Ibirapuitã	4.310
RS	43	10009	Ibirubá	19.306
RS	43	10108	Igrejinha	32.945
RS	43	10207	Ijuí	79.412
RS	43	10306	Ilópolis	4.328
RS	43	10330	Imbé	15.936
RS	43	10363	Imigrante	3.115
RS	43	10405	Independência	6.775
RS	43	10413	Inhacorá	2.347
RS	43	10439	Ipê	6.145
RS	43	10462	Ipiranga do Sul	2.033
RS	43	10504	Iraí	8.593
RS	43	10538	Itaara	4.794
RS	43	10553	Itacurubi	3.696
RS	43	10579	Itapuca	2.488
RS	43	10603	Itaqui	36.889
RS	43	10652	Itati	2.733
RS	43	10702	Itatiba do Sul	4.593
RS	43	10751	Ivorá	2.433
RS	43	10801	Ivoti	19.724
RS	43	10850	Jaboticaba	4.238
RS	43	10876	Jacuizinho	2.748
RS	43	10900	Jacutinga	3.636
RS	43	11007	Jaguarão	28.439
RS	43	11106	Jaguari	11.839
RS	43	11122	Jaquirana	4.469
RS	43	11130	Jari	3.801
RS	43	11155	Jóia	8.546
RS	43	11205	Júlio de Castilhos	20.007
RS	43	11270	Lagoa dos Três Cantos	1.634
RS	43	11239	Lagoa Bonita do Sul	2.733
RS	43	11304	Lagoa Vermelha	28.230
RS	43	11254	Lagoão	6.651
RS	43	11403	Lajeado	71.111
RS	43	11429	Lajeado do Bugre	2.632

U.F.		MUNICÍPIOS		POPULAÇÃO ESTIMADA
SIGLA	COD	COD	NOMES	
RS	43	11502	Lavras do Sul	8.378
RS	43	11601	Liberato Salzano	6.210
RS	43	11627	Lindolfo Collor	5.614
RS	43	11643	Linha Nova	1.522
RS	43	11718	Maçambara	4.440
RS	43	11700	Machadinho	5.638
RS	43	11734	Mampituba	2.987
RS	43	11759	Manoel Viana	6.963
RS	43	11775	Maquiné	7.626
RS	43	11791	Maratá	2.531
RS	43	11809	Marau	35.900
RS	43	11908	Marcelino Ramos	5.406
RS	43	11981	Mariana Pimentel	4.136
RS	43	12005	Mariano Moro	2.321
RS	43	12054	Marques de Souza	4.137
RS	43	12104	Mata	5.408
RS	43	12138	Mato Castelhano	2.722
RS	43	12153	Mato Leitão	3.773
RS	43	12179	Mato Queimado	1.895
RS	43	12203	Maximiliano de Almeida	5.111
RS	43	12252	Minas do Leão	8.055
RS	43	12302	Miraguaí	4.995
RS	43	12351	Montauri	1.615
RS	43	12377	Monte Alegre dos Campos	3.238
RS	43	12385	Monte Belo do Sul	2.834
RS	43	12401	Montenegro	59.110
RS	43	12427	Mormaço	2.689
RS	43	12443	Morrinhos do Sul	3.291
RS	43	12450	Morro Redondo	6.437
RS	43	12476	Morro Reuter	5.897
RS	43	12500	Mostardas	12.334
RS	43	12609	Muçum	4.693
RS	43	12617	Muitos Capões	3.085
RS	43	12625	Muliterno	1.864
RS	43	12658	Não-Me-Toque	15.876
RS	43	12674	Nicolau Vergueiro	1.805
RS	43	12708	Nonoai	12.632
RS	43	12757	Nova Alvorada	3.214
RS	43	12807	Nova Araçá	4.000
RS	43	12906	Nova Bassano	9.124
RS	43	12955	Nova Boa Vista	2.125

U.F.		MUNICÍPIOS		POPULAÇÃO ESTIMADA
SIGLA	COD	COD	NOMES	
RS	43	13003	Nova Bréscia	3.276
RS	43	13011	Nova Candelária	2.800
RS	43	13037	Nova Esperança do Sul	5.075
RS	43	13060	Nova Hartz	17.535
RS	43	13086	Nova Pádua	2.581
RS	43	13102	Nova Palma	6.663
RS	43	13201	Nova Petrópolis	18.484
RS	43	13300	Nova Prata	23.721
RS	43	13334	Nova Ramada	2.490
RS	43	13359	Nova Roma do Sul	3.690
RS	43	13375	Nova Santa Rita	22.177
RS	43	13490	Novo Barreiro	3.981
RS	43	13391	Novo Cabrais	3.926
RS	43	13409	Novo Hamburgo	255.945
RS	43	13425	Novo Machado	4.293
RS	43	13441	Novo Tiradentes	2.391
RS	43	13466	Novo Xingu	1.920
RS	43	13508	Osório	41.161
RS	43	13607	Paim Filho	4.558
RS	43	13656	Palmares do Sul	11.901
RS	43	13706	Palmeira das Missões	34.456
RS	43	13805	Palmitinho	7.122
RS	43	13904	Panambi	38.249
RS	43	13953	Pantano Grande	9.913
RS	43	14001	Paraí	6.895
RS	43	14027	Paraíso do Sul	7.609
RS	43	14035	Pareci Novo	3.236
RS	43	14050	Parobé	51.036
RS	43	14068	Passa Sete	5.225
RS	43	14076	Passo do Sobrado	6.237
RS	43	14100	Passo Fundo	185.882
RS	43	14134	Paulo Bento	2.149
RS	43	14159	Paverama	7.838
RS	43	14175	Pedras Altas	2.631
RS	43	14209	Pedro Osório	8.286
RS	43	14308	Pejuçara	3.972
RS	43	14407	Pelotas	343.167
RS	43	14423	Picada Café	5.009
RS	43	14456	Pinhal	2.412
RS	43	14464	Pinhal da Serra	2.059
RS	43	14472	Pinhal Grande	4.598

U.F.		MUNICÍPIOS		POPULAÇÃO ESTIMADA
SIGLA	COD	COD	NOMES	
RS	43	14498	Pinheirinho do Vale	4.597
RS	43	14506	Pinheiro Machado	13.213
RS	43	14555	Pirapó	3.016
RS	43	14605	Piratini	21.033
RS	43	14704	Planalto	10.796
RS	43	14753	Poço das Antas	2.086
RS	43	14779	Pontão	4.031
RS	43	14787	Ponte Preta	1.864
RS	43	14803	Portão	30.254
RS	43	14902	Porto Alegre	1.430.220
RS	43	15008	Porto Lucena	5.667
RS	43	15057	Porto Mauá	2.602
RS	43	15073	Porto Vera Cruz	2.080
RS	43	15107	Porto Xavier	11.145
RS	43	15131	Pouso Novo	2.018
RS	43	15149	Presidente Lucena	2.485
RS	43	15156	Progresso	6.407
RS	43	15172	Protásio Alves	2.182
RS	43	15206	Putinga	4.269
RS	43	15305	Quaraí	23.006
RS	43	15313	Quatro Irmãos	1.789
RS	43	15321	Quevedos	2.828
RS	43	15354	Quinze de Novembro	3.672
RS	43	15404	Redentora	10.054
RS	43	15453	Relvado	2.250
RS	43	15503	Restinga Seca	15.946
RS	43	15552	Rio dos Índios	4.243
RS	43	15602	Rio Grande	195.582
RS	43	15701	Rio Pardo	38.908
RS	43	15750	Riozinho	4.612
RS	43	15800	Roca Sales	10.366
RS	43	15909	Rodeio Bonito	5.872
RS	43	15958	Rolador	2.872
RS	43	16006	Rolante	20.094
RS	43	16105	Ronda Alta	9.891
RS	43	16204	Rondinha	5.775
RS	43	16303	Roque Gonzales	7.438
RS	43	16402	Rosário do Sul	41.715
RS	43	16428	Sagrada Família	2.669
RS	43	16436	Saldanha Marinho	3.038
RS	43	16451	Salto do Jacuí	12.665

U.F.		MUNICÍPIOS		POPULAÇÃO ESTIMADA
SIGLA	COD	COD	NOMES	
RS	43	16477	Salvador das Missões	2.673
RS	43	16501	Salvador do Sul	7.036
RS	43	16600	Sananduva	15.184
RS	43	16709	Santa Bárbara do Sul	9.250
RS	43	16733	Santa Cecília do Sul	1.771
RS	43	16758	Santa Clara do Sul	5.774
RS	43	16808	Santa Cruz do Sul	121.168
RS	43	16972	Santa Margarida do Sul	2.231
RS	43	16907	Santa Maria	266.822
RS	43	16956	Santa Maria do Herval	6.427
RS	43	17202	Santa Rosa	66.015
RS	43	17251	Santa Tereza	1.883
RS	43	17301	Santa Vitória do Palmar	31.789
RS	43	17004	Santana da Boa Vista	8.873
RS	43	17103	Santana do Livramento	84.779
RS	43	17400	Santiago	51.088
RS	43	17509	Santo Ângelo	75.627
RS	43	17608	Santo Antônio da Patrulha	39.302
RS	43	17707	Santo Antônio das Missões	12.089
RS	43	17558	Santo Antônio do Palma	2.289
RS	43	17756	Santo Antônio do Planalto	2.100
RS	43	17806	Santo Augusto	13.910
RS	43	17905	Santo Cristo	14.625
RS	43	17954	Santo Expedito do Sul	2.686
RS	43	18002	São Borja	63.257
RS	43	18051	São Domingos do Sul	2.951
RS	43	18101	São Francisco de Assis	19.909
RS	43	18200	São Francisco de Paula	22.262
RS	43	18309	São Gabriel	59.454
RS	43	18408	São Jerônimo	21.212
RS	43	18424	São João da Urtiga	5.109
RS	43	18432	São João do Polêsine	2.781
RS	43	18440	São Jorge	2.832
RS	43	18457	São José das Missões	3.038
RS	43	18465	São José do Herval	2.550
RS	43	18481	São José do Hortêncio	4.103
RS	43	18499	São José do Inhacorá	2.150
RS	43	18507	São José do Norte	25.921
RS	43	18606	São José do Ouro	7.183
RS	43	18614	São José do Sul	1.988
RS	43	18622	São José dos Ausentes	3.297

U.F.		MUNICÍPIOS		POPULAÇÃO ESTIMADA
SIGLA	COD	COD	NOMES	
RS	43	18705	São Leopoldo	210.145
RS	43	18804	São Lourenço do Sul	43.451
RS	43	18903	São Luiz Gonzaga	35.184
RS	43	19000	São Marcos	20.406
RS	43	19109	São Martinho	5.953
RS	43	19125	São Martinho da Serra	3.551
RS	43	19158	São Miguel das Missões	7.632
RS	43	19208	São Nicolau	6.006
RS	43	19307	São Paulo das Missões	6.811
RS	43	19356	São Pedro da Serra	3.270
RS	43	19364	São Pedro das Missões	2.029
RS	43	19372	São Pedro do Butiá	2.810
RS	43	19406	São Pedro do Sul	17.079
RS	43	19505	São Sebastião do Caí	21.142
RS	43	19604	São Sepé	24.397
RS	43	19703	São Valentim	4.010
RS	43	19711	São Valentim do Sul	2.319
RS	43	19737	São Valério do Sul	2.722
RS	43	19752	São Vendelino	1.873
RS	43	19802	São Vicente do Sul	8.636
RS	43	19901	Sapiranga	77.282
RS	43	20008	Sapucaia do Sul	126.085
RS	43	20107	Sarandi	21.504
RS	43	20206	Seberi	11.131
RS	43	20230	Sede Nova	3.019
RS	43	20263	Segredo	7.270
RS	43	20305	Selbach	4.911
RS	43	20321	Senador Salgado Filho	2.941
RS	43	20354	Sentinela do Sul	5.536
RS	43	20404	Serafina Corrêa	14.387
RS	43	20453	Sério	2.419
RS	43	20503	Sertão	6.726
RS	43	20552	Sertão Santana	6.077
RS	43	20578	Sete de Setembro	2.157
RS	43	20602	Severiano de Almeida	3.987
RS	43	20651	Silveira Martins	2.542
RS	43	20677	Sinimbu	10.669
RS	43	20701	Sobradinho	14.675
RS	43	20800	Soledade	30.932
RS	43	20859	Tabaí	4.269
RS	43	20909	Tapejara	18.699

U.F.		MUNICÍPIOS		POPULAÇÃO ESTIMADA
SIGLA	COD	COD	NOMES	
RS	43	21006	Tapera	10.775
RS	43	21105	Tapes	17.143
RS	43	21204	Taquara	55.270
RS	43	21303	Taquari	26.579
RS	43	21329	Taquaruçu do Sul	2.927
RS	43	21352	Tavares	5.293
RS	43	21402	Tenente Portela	14.273
RS	43	21436	Terra de Areia	10.232
RS	43	21451	Teutônia	26.670
RS	43	21469	Tio Hugo	2.705
RS	43	21477	Tiradentes do Sul	7.044
RS	43	21493	Toropi	3.146
RS	43	21501	Torres	33.686
RS	43	21600	Tramandaí	42.863
RS	43	21626	Travesseiro	2.462
RS	43	21808	Três de Maio	23.935
RS	43	21634	Três Arroios	3.088
RS	43	21667	Três Cachoeiras	10.891
RS	43	21709	Três Coroas	24.306
RS	43	21832	Três Forquilhas	3.141
RS	43	21857	Três Palmeiras	4.486
RS	43	21907	Três Passos	24.000
RS	43	21956	Trindade do Sul	6.093
RS	43	22004	Triunfo	25.096
RS	43	22103	Tucunduva	6.022
RS	43	22152	Tunas	4.532
RS	43	22186	Tupanci do Sul	1.777
RS	43	22202	Tupanciretã	23.591
RS	43	22251	Tupandi	3.843
RS	43	22301	Tuparendi	8.936
RS	43	22327	Turuçu	3.976
RS	43	22343	Ubiretama	2.474
RS	43	22350	União da Serra	1.674
RS	43	22376	Unistalda	2.421
RS	43	22400	Uruguaiana	127.138
RS	43	22509	Vacaria	62.370
RS	43	22533	Vale do Sol	11.216
RS	43	22541	Vale Real	4.971
RS	43	22525	Vale Verde	3.363
RS	43	22558	Vanini	2.032
RS	43	22608	Venâncio Aires	67.136

U.F.		MUNICÍPIOS		POPULAÇÃO ESTIMADA
SIGLA	COD	COD	NOMES	
RS	43	22707	Vera Cruz	23.702
RS	43	22806	Veranópolis	25.521
RS	43	22855	Vespasiano Correa	1.992
RS	43	22905	Viadutos	5.766
RS	43	23002	Viamão	257.844
RS	43	23101	Vicente Dutra	5.642
RS	43	23200	Victor Graeff	3.139
RS	43	23309	Vila Flores	3.288
RS	43	23358	Vila Lângaro	2.293
RS	43	23408	Vila Maria	4.291
RS	43	23457	Vila Nova do Sul	4.392
RS	43	23507	Vista Alegre	2.940
RS	43	23606	Vista Alegre do Prata	1.517
RS	43	23705	Vista Gaúcha	2.799
RS	43	23754	Vitória das Missões	3.707
RS	43	23770	Westfalia	2.824
RS	43	23804	Xangri-lá	11.402
MS	50	00203	Água Clara	13.623
MS	50	00252	Alcinópolis	4.440
MS	50	00609	Amambaí	34.501
MS	50	00708	Anastácio	23.012
MS	50	00807	Anaurilândia	8.635
MS	50	00856	Angélica	7.461
MS	50	00906	Antônio João	8.618
MS	50	01003	Aparecida do Taboado	20.433
MS	50	01102	Aquidauana	46.266
MS	50	01243	Aral Moreira	9.535
MS	50	01508	Bandeirantes	6.045
MS	50	01904	Bataguassu	19.295
MS	50	02001	Batayporã	10.870
MS	50	02100	Bela Vista	23.562
MS	50	02159	Bodoquena	8.401
MS	50	02209	Bonito	17.786
MS	50	02308	Brasilândia	12.494
MS	50	02407	Caarapó	23.437
MS	50	02605	Camapuã	13.562
MS	50	02704	Campo Grande	747.189
MS	50	02803	Caracol	5.257
MS	50	02902	Cassilândia	21.546
MS	50	02951	Chapadão do Sul	16.781
MS	50	03108	Corguinho	4.301

U.F.		MUNICÍPIOS		POPULAÇÃO ESTIMADA
SIGLA	COD	COD	NOMES	
MS	50	03157	Coronel Sapucaia	14.416
MS	50	03207	Corumbá	99.196
MS	50	03256	Costa Rica	18.884
MS	50	03306	Coxim	32.767
MS	50	03454	Deodápolis	11.586
MS	50	03488	Dois Irmãos do Buriti	9.622
MS	50	03504	Douradina	5.047
MS	50	03702	Dourados	187.601
MS	50	03751	Eldorado	12.304
MS	50	03801	Fátima do Sul	19.327
MS	50	03900	Figueirão	3.388
MS	50	04007	Glória de Dourados	9.915
MS	50	04106	Guia Lopes da Laguna	10.482
MS	50	04304	Iguatemi	15.085
MS	50	04403	Inocência	7.542
MS	50	04502	Itaporã	19.187
MS	50	04601	Itaquiraí	17.446
MS	50	04700	Ivinhema	21.138
MS	50	04809	Japorã	7.608
MS	50	04908	Jaraguari	5.745
MS	50	05004	Jardim	24.042
MS	50	05103	Jateí	3.913
MS	50	05152	Juti	5.518
MS	50	05202	Ladário	18.494
MS	50	05251	Laguna Carapã	5.989
MS	50	05400	Maracaju	31.933
MS	50	05608	Miranda	24.687
MS	50	05681	Mundo Novo	16.441
MS	50	05707	Naviraí	44.828
MS	50	05806	Nioaque	15.649
MS	50	06002	Nova Alvorada do Sul	12.430
MS	50	06200	Nova Andradina	44.971
MS	50	06259	Novo Horizonte do Sul	5.074
MS	50	06309	Paranaíba	40.118
MS	50	06358	Paranhos	11.437
MS	50	06408	Pedro Gomes	8.543
MS	50	06606	Ponta Porã	74.601
MS	50	06903	Porto Murtinho	15.333
MS	50	07109	Ribas do Rio Pardo	19.780
MS	50	07208	Rio Brilhante	27.435
MS	50	07307	Rio Negro	5.093

U.F.		MUNICÍPIOS		POPULAÇÃO ESTIMADA
SIGLA	COD	COD	NOMES	
MS	50	07406	Rio Verde de Mato Grosso	19.131
MS	50	07505	Rochedo	4.472
MS	50	07554	Santa Rita do Pardo	7.384
MS	50	07695	São Gabriel do Oeste	21.217
MS	50	07802	Selvíria	6.608
MS	50	07703	Sete Quedas	10.962
MS	50	07901	Sidrolândia	39.636
MS	50	07935	Sonora	12.991
MS	50	07950	Tacuru	9.483
MS	50	07976	Taquarussu	3.198
MS	50	08008	Terenos	14.952
MS	50	08305	Três Lagoas	88.592
MS	50	08404	Vicentina	5.787
MT	51	00102	Acorizal	5.679
MT	51	00201	Água Boa	19.777
MT	51	00250	Alta Floresta	51.136
MT	51	00300	Alto Araguaia	14.324
MT	51	00359	Alto Boa Vista	5.269
MT	51	00409	Alto Garças	9.445
MT	51	00508	Alto Paraguai	8.361
MT	51	00607	Alto Taquari	6.321
MT	51	00805	Apiacás	8.228
MT	51	01001	Araguaiana	3.038
MT	51	01209	Araguainha	1.138
MT	51	01258	Araputanga	15.878
MT	51	01308	Arenápolis	10.070
MT	51	01407	Aripuanã	19.930
MT	51	01605	Barão de Melgaço	7.842
MT	51	01704	Barra do Bugres	33.724
MT	51	01803	Barra do Garças	54.882
MT	51	01852	Bom Jesus do Araguaia	4.696
MT	51	01902	Brasnorte	14.609
MT	51	02504	Cáceres	86.805
MT	51	02603	Campinápolis	14.137
MT	51	02637	Campo Novo do Parecis	23.230
MT	51	02678	Campo Verde	27.151
MT	51	02686	Campos de Júlio	5.010
MT	51	02694	Canabrava do Norte	5.514
MT	51	02702	Canarana	17.785
MT	51	02793	Carlinda	12.032
MT	51	02850	Castanheira	8.040

U.F.		MUNICÍPIOS		POPULAÇÃO ESTIMADA
SIGLA	COD	COD	NOMES	
MT	51	03007	Chapada dos Guimarães	17.978
MT	51	03056	Cláudia	11.032
MT	51	03106	Cocalinho	6.039
MT	51	03205	Colíder	31.744
MT	51	03254	Colniza	29.636
MT	51	03304	Comodoro	18.623
MT	51	03353	Confresa	22.180
MT	51	03361	Conquista D'Oeste	3.226
MT	51	03379	Cotriguaçu	14.406
MT	51	03403	Cuiabá	544.737
MT	51	03437	Curvelândia	4.994
MT	51	03452	Denise	10.808
MT	51	03502	Diamantino	18.966
MT	51	03601	Dom Aquino	8.497
MT	51	03700	Feliz Natal	10.769
MT	51	03809	Figueirópolis D'Oeste	3.721
MT	51	03858	Gaúcha do Norte	6.052
MT	51	03908	General Carneiro	4.969
MT	51	03957	Glória D'Oeste	3.204
MT	51	04104	Guarantã do Norte	31.801
MT	51	04203	Guiratinga	14.360
MT	51	04500	Indiavaí	2.623
MT	51	04526	Ipiranga do Norte	4.376
MT	51	04542	Itanhangá	4.911
MT	51	04559	Itaúba	4.701
MT	51	04609	Itiquira	12.676
MT	51	04807	Jaciara	25.745
MT	51	04906	Jangada	8.345
MT	51	05002	Jauru	10.972
MT	51	05101	Juara	33.040
MT	51	05150	Juína	39.582
MT	51	05176	Juruena	9.182
MT	51	05200	Juscimeira	12.168
MT	51	05234	Lambari D'Oeste	5.026
MT	51	05259	Lucas do Rio Verde	32.255
MT	51	05309	Luciára	2.472
MT	51	05580	Marcelândia	14.482
MT	51	05606	Matupá	14.821
MT	51	05622	Mirassol d'Oeste	25.360
MT	51	05903	Nobres	15.296
MT	51	06000	Nortelândia	6.368

U.F.		MUNICÍPIOS		POPULAÇÃO ESTIMADA
SIGLA	COD	COD	NOMES	
MT	51	06109	Nossa Senhora do Livramento	12.766
MT	51	06158	Nova Bandeirantes	13.425
MT	51	06208	Nova Brasilândia	4.989
MT	51	06216	Nova Canaã do Norte	13.087
MT	51	08808	Nova Guarita	4.980
MT	51	06182	Nova Lacerda	5.144
MT	51	08857	Nova Marilândia	2.369
MT	51	08907	Nova Maringá	5.803
MT	51	08956	Nova Monte Verde	8.443
MT	51	06224	Nova Mutum	25.658
MT	51	06174	Nova Nazaré	2.867
MT	51	06232	Nova Olímpia	20.330
MT	51	06190	Nova Santa Helena	3.453
MT	51	06240	Nova Ubiratã	8.125
MT	51	06257	Nova Xavantina	19.268
MT	51	06273	Novo Horizonte do Norte	3.930
MT	51	06265	Novo Mundo	7.015
MT	51	06315	Novo Santo Antônio	2.221
MT	51	06281	Novo São Joaquim	7.047
MT	51	06299	Paranaíta	11.950
MT	51	06307	Paranatinga	20.874
MT	51	06372	Pedra Preta	16.208
MT	51	06422	Peixoto de Azevedo	29.995
MT	51	06455	Planalto da Serra	2.807
MT	51	06505	Poconé	32.059
MT	51	06653	Pontal do Araguaia	5.178
MT	51	06703	Ponte Branca	1.831
MT	51	06752	Pontes e Lacerda	39.071
MT	51	06802	Porto dos Gaúchos	6.321
MT	51	06778	Porto Alegre do Norte	9.979
MT	51	06828	Porto Esperidião	9.871
MT	51	06851	Porto Estrela	4.093
MT	51	07008	Poxoréo	17.985
MT	51	07040	Primavera do Leste	46.311
MT	51	07065	Querência	11.179
MT	51	07156	Reserva do Cabaçal	2.584
MT	51	07180	Ribeirão Cascalheira	9.006
MT	51	07198	Ribeirãozinho	2.176
MT	51	07206	Rio Branco	5.201
MT	51	07578	Rondolândia	3.457
MT	51	07602	Rondonópolis	179.094

U.F.		MUNICÍPIOS		POPULAÇÃO ESTIMADA
SIGLA	COD	COD	NOMES	
MT	51	07701	Rosário Oeste	18.531
MT	51	07750	Salto do Céu	3.698
MT	51	07248	Santa Carmem	4.486
MT	51	07743	Santa Cruz do Xingu	2.236
MT	51	07768	Santa Rita do Trivelato	2.615
MT	51	07776	Santa Terezinha	7.564
MT	51	07263	Santo Afonso	2.939
MT	51	07792	Santo Antônio do Leste	3.418
MT	51	07800	Santo Antônio do Leverger	19.964
MT	51	07859	São Félix do Araguaia	11.097
MT	51	07297	São José do Povo	3.417
MT	51	07305	São José do Rio Claro	18.102
MT	51	07354	São José do Xingu	4.285
MT	51	07107	São José dos Quatro Marcos	19.527
MT	51	07404	São Pedro da Cipa	4.175
MT	51	07875	Sapezal	15.014
MT	51	07883	Serra Nova Dourada	1.407
MT	51	07909	Sinop	110.513
MT	51	07925	Sorriso	57.799
MT	51	07941	Tabaporã	10.776
MT	51	07958	Tangará da Serra	79.870
MT	51	08006	Tapurah	11.020
MT	51	08055	Terra Nova do Norte	15.063
MT	51	08105	Tesouro	3.200
MT	51	08204	Torixoréu	4.184
MT	51	08303	União do Sul	4.106
MT	51	08352	Vale de São Domingos	2.955
MT	51	08402	Várzea Grande	237.925
MT	51	08501	Vera	9.468
MT	51	05507	Vila Bela da Santíssima Trindade	14.362
MT	51	08600	Vila Rica	19.672
GO	52	00050	Abadia de Goiás	6.182
GO	52	00100	Abadiânia	13.209
GO	52	00134	Acreúna	19.173
GO	52	00159	Adelândia	2.596
GO	52	00175	Água Fria de Goiás	5.243
GO	52	00209	Água Limpa	2.121
GO	52	00258	Águas Lindas de Goiás	139.804
GO	52	00308	Alexânia	20.661
GO	52	00506	Aloândia	2.121
GO	52	00555	Alto Horizonte	3.318

U.F.		MUNICÍPIOS		POPULAÇÃO ESTIMADA
SIGLA	COD	COD	NOMES	
GO	52	00605	Alto Paraíso de Goiás	6.913
GO	52	00803	Alvorada do Norte	8.570
GO	52	00829	Amaralina	3.662
GO	52	00852	Americano do Brasil	4.811
GO	52	00902	Amorinópolis	3.549
GO	52	01108	Anápolis	331.329
GO	52	01207	Anhanguera	1.007
GO	52	01306	Anicuns	18.110
GO	52	01405	Aparecida de Goiânia	494.919
GO	52	01454	Aparecida do Rio Doce	2.830
GO	52	01504	Aporé	3.684
GO	52	01603	Araçu	3.966
GO	52	01702	Aragarças	17.777
GO	52	01801	Aragoiânia	7.589
GO	52	02155	Araguapaz	7.742
GO	52	02353	Arenópolis	3.532
GO	52	02502	Aruanã	6.879
GO	52	02601	Aurilândia	3.761
GO	52	02809	Avelinópolis	2.431
GO	52	03104	Baliza	3.551
GO	52	03203	Barro Alto	6.676
GO	52	03302	Bela Vista de Goiás	21.466
GO	52	03401	Bom Jardim de Goiás	8.679
GO	52	03500	Bom Jesus de Goiás	20.668
GO	52	03559	Bonfinópolis	7.157
GO	52	03575	Bonópolis	3.340
GO	52	03609	Brazabrantes	3.294
GO	52	03807	Britânia	5.203
GO	52	03939	Buriti de Goiás	2.248
GO	52	03906	Buriti Alegre	8.485
GO	52	03962	Buritinópolis	3.558
GO	52	04003	Cabeceiras	6.796
GO	52	04201	Cachoeira de Goiás	1.441
GO	52	04102	Cachoeira Alta	8.280
GO	52	04250	Cachoeira Dourada	7.666
GO	52	04300	Caçu	11.281
GO	52	04409	Caiapônia	16.397
GO	52	04508	Caldas Novas	65.970
GO	52	04557	Caldazinha	3.299
GO	52	04607	Campestre de Goiás	3.554
GO	52	04656	Campinaçu	3.892

U.F.		MUNICÍPIOS		POPULAÇÃO ESTIMADA
SIGLA	COD	COD	NOMES	
GO	52	04706	Campinorte	10.010
GO	52	04805	Campo Alegre de Goiás	6.127
GO	52	04854	Campo Limpo de Goiás	5.903
GO	52	04904	Campos Belos	18.984
GO	52	04953	Campos Verdes	6.281
GO	52	05000	Carmo do Rio Verde	9.333
GO	52	05059	Castelândia	3.590
GO	52	05109	Catalão	79.618
GO	52	05208	Caturaí	4.639
GO	52	05307	Cavalcante	10.290
GO	52	05406	Ceres	19.114
GO	52	05455	Cezarina	7.715
GO	52	05471	Chapadão do Céu	5.673
GO	52	05497	Cidade Ocidental	51.303
GO	52	05513	Cocalzinho de Goiás	15.246
GO	52	05521	Colinas do Sul	3.999
GO	52	05703	Córrego do Ouro	2.666
GO	52	05802	Corumbá de Goiás	9.408
GO	52	05901	Corumbaíba	8.447
GO	52	06206	Cristalina	38.125
GO	52	06305	Cristianópolis	3.153
GO	52	06404	Crixás	14.986
GO	52	06503	Cromínia	3.725
GO	52	06602	Cumari	3.143
GO	52	06701	Damianópolis	3.646
GO	52	06800	Damolândia	2.789
GO	52	06909	Davinópolis	2.073
GO	52	07105	Diorama	2.269
GO	52	08301	Divinópolis de Goiás	5.537
GO	52	07253	Doverlândia	8.576
GO	52	07352	Edealina	3.822
GO	52	07402	Edéia	10.577
GO	52	07501	Estrela do Norte	3.242
GO	52	07535	Faina	7.063
GO	52	07600	Fazenda Nova	6.469
GO	52	07808	Firminópolis	10.664
GO	52	07907	Flores de Goiás	11.122
GO	52	08004	Formosa	94.717
GO	52	08103	Formoso	5.356
GO	52	08152	Gameleira de Goiás	3.492
GO	52	08400	Goianápolis	11.580

U.F.		MUNICÍPIOS		POPULAÇÃO ESTIMADA
SIGLA	COD	COD	NOMES	
GO	52	08509	Goiandira	5.074
GO	52	08608	Goianésia	56.169
GO	52	08707	Goiânia	1.265.394
GO	52	08806	Goianira	25.647
GO	52	08905	Goiás	24.859
GO	52	09101	Goiatuba	32.220
GO	52	09150	Gouvelândia	4.721
GO	52	09200	Guapó	13.974
GO	52	09291	Guaraíta	2.411
GO	52	09408	Guarani de Goiás	4.151
GO	52	09457	Guarinos	2.424
GO	52	09606	Heitoraí	3.684
GO	52	09705	Hidrolândia	14.577
GO	52	09804	Hidrolina	4.231
GO	52	09903	Iaciara	12.672
GO	52	09937	Inaciolândia	5.887
GO	52	09952	Indiara	13.230
GO	52	10000	Inhumas	46.555
GO	52	10109	Ipameri	23.911
GO	52	10158	Ipiranga de Goiás	2.900
GO	52	10208	Iporá	32.002
GO	52	10307	Israelândia	2.891
GO	52	10406	Itaberaí	31.965
GO	52	10562	Itaguari	4.369
GO	52	10604	Itaguaru	5.605
GO	52	10802	Itajá	5.544
GO	52	10901	Itapaci	16.806
GO	52	11008	Itapirapuã	8.084
GO	52	11206	Itapuranga	25.337
GO	52	11305	Itarumã	5.491
GO	52	11404	Itauçu	9.046
GO	52	11503	Itumbiara	91.843
GO	52	11602	Ivolândia	2.764
GO	52	11701	Jandaia	6.578
GO	52	11800	Jaraguá	41.014
GO	52	11909	Jataí	85.491
GO	52	12006	Jaupaci	3.070
GO	52	12055	Jesúpolis	2.281
GO	52	12105	Joviânia	6.918
GO	52	12204	Jussara	19.229
GO	52	12253	Lagoa Santa	1.308

U.F.		MUNICÍPIOS		POPULAÇÃO ESTIMADA
SIGLA	COD	COD	NOMES	
GO	52	12303	Leopoldo de Bulhões	9.362
GO	52	12501	Luziânia	203.800
GO	52	12600	Mairipotaba	2.775
GO	52	12709	Mambaí	6.891
GO	52	12808	Mara Rosa	10.516
GO	52	12907	Marzagão	2.137
GO	52	12956	Matrinchã	4.433
GO	52	13004	Maurilândia	11.367
GO	52	13053	Mimoso de Goiás	2.930
GO	52	13087	Minaçu	31.647
GO	52	13103	Mineiros	47.500
GO	52	13400	Moiporá	1.881
GO	52	13509	Monte Alegre de Goiás	7.418
GO	52	13707	Montes Claros de Goiás	8.069
GO	52	13756	Montividiu	9.766
GO	52	13772	Montividiu do Norte	4.621
GO	52	13806	Morrinhos	40.512
GO	52	13855	Morro Agudo de Goiás	2.391
GO	52	13905	Mossâmedes	4.988
GO	52	14002	Mozarlândia	13.815
GO	52	14051	Mundo Novo	6.916
GO	52	14101	Mutunópolis	4.078
GO	52	14408	Nazário	7.536
GO	52	14507	Nerópolis	20.120
GO	52	14606	Niquelândia	39.720
GO	52	14705	Nova América	2.271
GO	52	14804	Nova Aurora	2.184
GO	52	14838	Nova Crixás	13.221
GO	52	14861	Nova Glória	8.668
GO	52	14879	Nova Iguaçu de Goiás	2.687
GO	52	14903	Nova Roma	3.644
GO	52	15009	Nova Veneza	7.168
GO	52	15207	Novo Brasil	3.454
GO	52	15231	Novo Gama	87.558
GO	52	15256	Novo Planalto	4.191
GO	52	15306	Orizona	15.015
GO	52	15405	Ouro Verde de Goiás	4.580
GO	52	15504	Ouvidor	4.952
GO	52	15603	Padre Bernardo	27.429
GO	52	15652	Palestina de Goiás	3.319
GO	52	15702	Palmeiras de Goiás	22.353

U.F.		MUNICÍPIOS		POPULAÇÃO ESTIMADA
SIGLA	COD	COD	NOMES	
GO	52	15801	Palmelo	2.324
GO	52	15900	Palminópolis	3.745
GO	52	16007	Panamá	2.678
GO	52	16304	Paranaiguara	7.899
GO	52	16403	Paraúna	11.283
GO	52	16452	Perolândia	2.829
GO	52	16809	Petrolina de Goiás	10.099
GO	52	16908	Pilar de Goiás	2.872
GO	52	17104	Piracanjuba	24.008
GO	52	17203	Piranhas	11.324
GO	52	17302	Pirenópolis	20.990
GO	52	17401	Pires do Rio	27.792
GO	52	17609	Planaltina	79.162
GO	52	17708	Pontalina	16.688
GO	52	18003	Porangatu	40.420
GO	52	18052	Porteirão	3.129
GO	52	18102	Portelândia	3.359
GO	52	18300	Posse	30.812
GO	52	18391	Professor Jamil	3.386
GO	52	18508	Quirinópolis	39.485
GO	52	18607	Rialma	10.855
GO	52	18706	Rianápolis	4.269
GO	52	18789	Rio Quente	3.177
GO	52	18805	Rio Verde	158.818
GO	52	18904	Rubiataba	18.583
GO	52	19001	Sanclerlândia	7.905
GO	52	19100	Santa Bárbara de Goiás	5.936
GO	52	19209	Santa Cruz de Goiás	3.663
GO	52	19258	Santa Fé de Goiás	4.812
GO	52	19308	Santa Helena de Goiás	36.198
GO	52	19357	Santa Isabel	3.580
GO	52	19407	Santa Rita do Araguaia	6.171
GO	52	19456	Santa Rita do Novo Destino	3.528
GO	52	19506	Santa Rosa de Goiás	2.841
GO	52	19605	Santa Tereza de Goiás	4.196
GO	52	19704	Santa Terezinha de Goiás	11.856
GO	52	19712	Santo Antônio da Barra	4.276
GO	52	19738	Santo Antônio de Goiás	4.129
GO	52	19753	Santo Antônio do Descoberto	57.908
GO	52	19803	São Domingos	10.116
GO	52	19902	São Francisco de Goiás	5.847

U.F.		MUNICÍPIOS		POPULAÇÃO ESTIMADA
SIGLA	COD	COD	NOMES	
GO	52	20009	São João d'Aliança	8.642
GO	52	20058	São João da Paraúna	1.706
GO	52	20108	São Luís de Montes Belos	27.684
GO	52	20157	São Luíz do Norte	4.426
GO	52	20207	São Miguel do Araguaia	23.128
GO	52	20264	São Miguel do Passa Quatro	3.824
GO	52	20280	São Patrício	2.129
GO	52	20405	São Simão	14.308
GO	52	20454	Senador Canedo	75.297
GO	52	20504	Serranópolis	7.692
GO	52	20603	Silvânia	19.038
GO	52	20686	Simolândia	7.233
GO	52	20702	Sítio d'Abadia	3.436
GO	52	21007	Taquaral de Goiás	3.484
GO	52	21080	Teresina de Goiás	2.887
GO	52	21197	Terezópolis de Goiás	5.875
GO	52	21304	Três Ranchos	2.957
GO	52	21403	Trindade	102.870
GO	52	21452	Trombas	3.663
GO	52	21502	Turvânia	5.093
GO	52	21551	Turvelândia	4.020
GO	52	21577	Uirapuru	3.112
GO	52	21601	Uruaçu	34.411
GO	52	21700	Uruana	14.110
GO	52	21809	Urutaí	2.758
GO	52	21858	Valparaíso de Goiás	120.878
GO	52	21908	Varjão	3.782
GO	52	22005	Vianópolis	12.699
GO	52	22054	Vicentinópolis	6.091
GO	52	22203	Vila Boa	4.461
GO	52	22302	Vila Propício	5.282
DF	53	00108	Brasília	2.557.158

Fonte: IBGE/DPE/COPIS

Nota: **Inclusive o Município de Nazária, no Estado do Piauí, com instalação prevista para 1° de janeiro de 2009**
