# Supplementary material for: Forecasting dengue fever in Brazil: An assessment of climate conditions
Source: PLoS One. 2019 Aug 8;14(8):e0220106. doi: 10.1371/journal.pone.0220106 (PMC6687106; doi:10.1371/journal.pone.0220106)
Supplement: S1 File — (ZIP) [file pone.0220106.s003.zip › data and codes_submit/training dataset analysis/data/data sinan/Rio de Janeiro/Rio de Janeiro_2013.pdf]

Subsecretaria de Promoção da Saúde, Atenção Primária e Vigilância em Saúde
Superintendência de Vigilância em Saúde
Coordenação de Vigilância Epidemiológica
Gerência de Vigilância de Doenças e Agravos

Número de Casos de Dengue* por mês, Áreas de Planejamento, Regiões Administrativas e Bairros
Município do Rio de Janeiro, 2013

Dados Atualizados em 30/01/2014

Área Programática, Regiões Administrativas e Bairros	Pop. Censo 2010	Jan	Fev	Mar	Abr	Mai	Jun	Jul	Ago	Set	Out	Nov	Dez	Total
**Total**	**6.320.446**	**3.930**	**8.851**	**21.062**	**22.110**	**6.745**	**1.768**	**593**	**245**	**218**	**213**	**285**	**258**	**66.278**
**Bairro de Residência Ignorado****	**0**	**105**	**220**	**290**	**237**	**129**	**38**	**11**	**5**	**2**	**7**	**6**	**5**	**1.055**
**Área de Programática 1.0**	**297.976**	**251**	**678**	**1.368**	**1.706**	**632**	**102**	**25**	**16**	**17**	**6**	**15**	**22**	**4.838**
**I Portuária**	**48.664**	**44**	**135**	**269**	**297**	**115**	**18**	**0**	**4**	**3**	**0**	**1**	**3**	**889**
Saúde	2.749	12	14	12	6	9	2	0	0	0	0	0	0	55
Gamboa	13.108	5	23	41	38	16	2	0	0	0	0	0	0	125
Santo Cristo	12.330	14	31	57	56	26	6	0	0	0	0	0	1	191
Caju	20.477	13	67	159	197	64	8	0	4	3	0	1	2	518
**II Centro**	**41.142**	**37**	**76**	**180**	**210**	**72**	**12**	**2**	**2**	**3**	**2**	**4**	**3**	**603**
Centro	41.142	37	76	180	210	72	12	2	2	3	2	4	3	603
**III Rio Comprido**	**78.975**	**74**	**206**	**402**	**480**	**171**	**24**	**7**	**2**	**1**	**0**	**3**	**7**	**1.377**
Catumbi	12.556	28	47	50	67	11	3	0	0	1	0	1	3	211
Rio Comprido	43.764	29	95	199	247	102	16	5	1	0	0	2	4	700
Cidade Nova	5.466	3	24	46	47	11	0	0	0	0	0	0	0	131
Estácio	17.189	14	40	107	119	47	5	2	1	0	0	0	0	335
**VII São Cristóvão**	**84.908**	**49**	**113**	**246**	**378**	**192**	**29**	**15**	**8**	**2**	**1**	**3**	**1**	**1.037**
São Cristóvão	26.510	19	55	129	232	109	14	10	6	1	1	2	1	579
Mangueira	17.835	3	12	26	59	32	5	1	0	0	0	1	0	139
Benfica	25.081	27	46	91	87	51	10	4	2	1	0	0	0	319
Vasco da Gama	15.482	0	0	0	0	0	0	0	0	0	0	0	0	0
**XXI Paquetá**	**3.361**	**16**	**51**	**44**	**38**	**8**	**1**	**0**	**0**	**0**	**0**	**0**	**0**	**158**
Paquetá	3.361	16	51	44	38	8	1	0	0	0	0	0	0	158
**XXIII Santa Teresa**	**40.926**	**31**	**97**	**227**	**303**	**74**	**18**	**1**	**0**	**8**	**3**	**4**	**8**	**774**
Santa Teresa	40.926	31	97	227	303	74	18	1	0	8	3	4	8	774
**Área Programática 2.1**	**638.050**	**537**	**1.193**	**2.683**	**2.881**	**970**	**188**	**46**	**16**	**13**	**11**	**17**	**17**	**8.572**
**IV Botafogo**	**239.729**	**148**	**351**	**931**	**1.014**	**318**	**50**	**10**	**7**	**5**	**3**	**8**	**5**	**2.850**

Flamengo	50.043	26	43	126	138	44	14	2	1	2	1	2	1	400
Glória	9.661	5	26	90	75	31	3	1	1	0	1	1	0	234
Laranjeiras	45.554	26	34	127	157	37	5	1	0	0	0	1	1	389
Catete	24.057	13	44	147	135	51	8	0	0	0	0	1	0	399
Cosme Velho	7.178	7	19	52	50	13	2	0	0	0	0	0	1	144
Botafogo	82.890	66	155	317	381	119	15	5	5	3	1	3	2	1.072
Humaitá	13.285	3	22	53	43	10	3	0	0	0	0	0	0	134
Urca	7.061	2	8	19	35	13	0	1	0	0	0	0	0	78
**V Copacabana**	**161 191**	**129**	**376**	**834**	**992**	**319**	**47**	**14**	**6**	**2**	**4**	**5**	**4**	**2.732**
Leme	14.799	13	29	103	115	27	4	4	0	0	0	0	2	297
Copacabana	146.392	116	347	731	877	292	43	10	6	2	4	5	2	2.435
**VI Lagoa**	**167 774**	**82**	**157**	**457**	**534**	**142**	**27**	**10**	**2**	**1**	**4**	**4**	**6**	**1.426**
Ipanema	42.743	21	32	110	121	38	5	1	0	0	1	2	0	331
Leblon	46.044	21	26	94	107	13	5	2	1	0	2	0	1	272
Lagoa	21.198	9	18	51	69	25	3	1	0	1	0	0	0	177
Jardim Botânico	18.009	13	35	76	79	20	1	1	0	0	0	1	0	226
Gávea	16.003	9	18	58	60	14	4	2	0	0	1	0	3	169
Vidigal	12.797	4	19	50	73	27	8	2	0	0	0	1	2	186
São Conrado	10.980	5	9	18	25	5	1	1	1	0	0	0	0	65
**XXVII Rocinha**	**69 356**	**178**	**309**	**461**	**341**	**191**	**64**	**12**	**1**	**5**	**0**	**0**	**2**	**1.564**
Rocinha	69 356	178	309	461	341	191	64	12	1	5	0	0	2	1.564
**Área Programática 2.2**	**371 120**	**384**	**902**	**1.516**	**1.818**	**548**	**78**	**19**	**6**	**9**	**6**	**8**	**11**	**5.305**
**VIII Tijuca**	**181 810**	**184**	**419**	**792**	**977**	**294**	**38**	**9**	**2**	**5**	**1**	**2**	**9**	**2.732**
Praça da Bandeira	8.662	4	11	47	51	20	4	0	0	0	0	2	0	139
Tijuca	163.805	178	379	636	826	248	25	9	2	5	1	0	9	2.318
Alto da Boa Vista	9.343	2	29	109	100	26	9	0	0	0	0	0	0	275
**IX Vila Isabel**	**189 310**	**200**	**483**	**724**	**841**	**254**	**40**	**10**	**4**	**4**	**5**	**6**	**2**	**2.573**
Maracanã	25.256	30	51	90	143	37	6	3	0	0	2	0	1	363
Vila Isabel	86.018	73	202	319	319	106	16	3	0	2	2	3	0	1.045
Andaraí	39.365	55	126	142	194	54	7	0	3	0	1	0	1	583
Grajaú	38.671	42	104	173	185	57	11	4	1	2	0	3	0	582
**Área Programática 3.1**	**886 551**	**588**	**1.187**	**3.233**	**3.086**	**766**	**233**	**87**	**34**	**30**	**32**	**58**	**33**	**9.367**
**X Ramos**	**153 177**	**92**	**200**	**559**	**583**	**212**	**63**	**23**	**8**	**1**	**8**	**7**	**3**	**1.759**
Manguinhos	36.160	21	28	73	75	37	8	6	2	0	2	1	0	253
Bonsucesso	18.711	28	89	196	239	88	18	10	2	1	3	1	1	676
Ramos	40.792	25	48	177	155	53	22	3	0	0	2	3	2	490
Olaria	57.514	18	35	113	114	34	15	4	4	0	1	2	0	340

**XI Penha**	**321 887**	**130**	**515**	**1.414**	**1.045**	**162**	**51**	**17**	**3**	**1**	**1**	**8**	**6**	**3.353**
Penha	78.678	26	41	221	179	28	18	4	0	0	1	2	5	525
Penha Circular	47.816	5	27	52	47	12	4	1	0	1	0	0	0	149
Brás de Pina	59.222	12	37	148	116	26	6	2	1	0	0	2	1	351
Cordovil	45.202	16	35	177	166	30	9	5	1	0	0	0	0	439
Parada de Lucas	23.923	10	35	131	64	5	3	3	1	0	0	0	0	252
Vigário Geral	41.820	38	188	336	175	19	2	1	0	0	0	3	0	762
Jardim América	25.226	23	152	349	298	42	9	1	0	0	0	1	0	875
**XX Ilha Do Governador**	**212 574**	**322**	**433**	**824**	**1.149**	**321**	**112**	**46**	**22**	**25**	**22**	**40**	**21**	**3.337**
Ribeira	3.528	6	3	9	21	2	1	0	0	1	0	0	0	43
Zumbi	2.016	2	6	15	11	9	3	0	0	0	0	1	1	48
Cacuia	11.013	28	19	55	64	16	3	8	2	1	1	2	1	200
Pitangueiras	11.756	10	17	41	65	16	6	2	1	0	1	2	1	162
Praia da Bandeira	5.948	4	9	17	20	7	4	1	0	1	1	2	0	66
Cocotá	4.877	11	24	58	43	14	4	2	0	2	1	2	0	161
Bancários	12.512	28	27	61	93	24	10	3	0	2	2	6	0	256
Freguesia (Ilha do Governador)	19.437	19	29	66	96	29	9	3	3	3	2	6	5	270
Jardim Guanabara	32.213	43	60	92	159	34	16	5	3	2	3	4	2	423
Jardim Carioca	24.848	36	64	104	146	47	15	4	4	5	3	4	3	435
Tauá	29.567	35	67	122	141	36	9	3	2	3	4	8	4	434
Moneró	6.476	14	15	36	46	14	6	3	1	1	0	0	1	137
Portuguesa	23.856	40	36	60	109	37	17	7	3	1	3	1	3	317
Galeão	22.971	46	55	82	134	36	9	5	3	3	1	2	0	376
Cidade Universitária	1.556	0	2	6	1	0	0	0	0	0	0	0	0	9
**XXIX Complexo do Alemão**	**69 143**	**5**	**9**	**30**	**50**	**13**	**3**	**1**	**1**	**2**	**0**	**0**	**1**	**115**
Complexo do Alemão	69 143	5	9	30	50	13	3	1	1	2	0	0	1	115
**XXX Maré**	**129 770**	**39**	**30**	**406**	**259**	**58**	**4**	**0**	**0**	**1**	**1**	**3**	**2**	**803**
Maré	129 770	39	30	406	259	58	4	0	0	1	1	3	2	803
**Área Programática 3.2**	**569 970**	**264**	**608**	**1.185**	**1.343**	**566**	**194**	**53**	**23**	**19**	**16**	**31**	**30**	**4.332**
**XII Inhaúma**	**134 349**	**74**	**189**	**394**	**409**	**180**	**71**	**11**	**5**	**1**	**4**	**6**	**8**	**1.352**
Higienópolis	15.734	9	28	46	40	23	5	1	0	0	1	0	0	153
Maria da Graça	7.972	8	8	29	18	9	6	0	1	0	0	0	0	79
Del Castilho	15.610	7	32	60	65	34	11	4	1	0	0	1	3	218
Inhaúma	45.698	30	68	135	144	50	25	2	1	0	2	1	3	461
Engenho da Rainha	26.659	8	26	75	94	40	13	3	2	1	0	1	0	263
Tomas Coelho	22.676	12	27	49	48	24	11	1	0	0	1	3	2	178
**XIII Méier**	**397.782**	**187**	**408**	**779**	**913**	**372**	**121**	**40**	**18**	**18**	**12**	**25**	**20**	**2.913**

Jacaré	8.343	18	45	71	89	36	14	9	2	0	2	1	0	287
São Francisco Xavier	8.766	3	9	16	18	5	1	0	0	0	0	2	0	54
Rocha	12.653	3	18	33	37	23	6	0	1	1	0	2	0	124
Riachuelo	10.895	7	9	29	43	28	4	1	1	0	0	1	0	123
Sampaio	42.172	3	10	22	22	12	7	0	0	0	0	1	0	77
Engenho Novo	9 276	15	52	101	152	56	19	4	2	2	1	2	3	409
Lins de Vasconcelos	37.487	15	45	85	77	37	6	2	1	1	0	3	2	274
Méier	49.828	31	58	111	110	52	13	6	6	1	2	2	3	395
Todos ds Santos	24.646	7	7	22	23	16	3	2	0	0	1	1	1	83
Cachambi	42.415	16	30	65	92	30	15	3	0	3	1	1	0	256
Engenho de Dentro	45.540	19	44	72	89	27	13	3	2	2	4	3	4	282
Água Santa	8.756	1	3	4	7	3	2	0	0	0	0	0	0	20
Encantado	15.021	9	7	18	17	2	1	0	1	5	0	1	1	62
Piedade	43.378	17	25	55	55	17	6	3	0	3	1	3	3	188
Abolição	11.356	6	20	23	30	5	1	4	0	0	0	0	2	91
Pilares	27.250	17	26	52	52	23	10	3	2	0	0	2	1	188
**XVIII Jacarezinho**	**37 839**	**3**	**11**	**12**	**21**	**14**	**2**	**2**	**0**	**0**	**0**	**0**	**2**	**67**
Jacarezinho	37 839	3	11	12	21	14	2	2	0	0	0	0	2	67
**Área Programática 3.3**	**942 638**	**330**	**884**	**2.295**	**2.314**	**813**	**282**	**109**	**25**	**30**	**25**	**35**	**35**	**7.177**
**XIV Irajá**	**202 952**	**89**	**287**	**907**	**899**	**283**	**73**	**30**	**1**	**1**	**2**	**6**	**8**	**2.586**
Vila Kosmos	18.274	8	27	120	138	25	5	4	0	0	0	2	0	329
Vicente de Carvalho	24.964	6	15	72	88	33	8	0	0	0	0	0	1	223
Vila da Penha	25.465	8	31	114	98	29	5	3	0	1	2	0	2	293
Vista Alegre	8.622	3	14	68	52	20	9	2	1	0	0	0	1	170
Irajá	96.382	48	163	457	465	154	38	17	0	0	0	2	3	1.347
Colégio	29.245	16	37	76	58	22	8	4	0	0	0	2	1	224
**XV Madureira**	**372 555**	**135**	**329**	**763**	**834**	**308**	**107**	**40**	**17**	**14**	**15**	**16**	**20**	**2.598**
Campinho	10.156	12	18	56	78	16	6	3	3	0	1	0	5	198
Quintino Bocaiúva	31.185	12	29	35	31	20	10	0	3	1	5	3	3	152
Cavalcanti	16.141	8	20	17	24	14	4	3	0	0	0	1	0	91
Engenheiro Leal	6.113	1	1	6	3	1	0	0	0	0	0	1	0	13
Cascadura	34.456	18	31	86	63	41	14	2	2	1	3	1	2	264
Madureira	50.106	35	57	162	143	46	17	4	2	4	0	2	5	477
Vaz Lobo	15.167	2	9	55	46	6	7	1	0	0	1	0	0	127
Turiaçu	17.246	5	13	16	23	4	2	0	0	0	0	0	0	63
Rocha Miranda	44.188	10	32	99	92	28	8	7	0	0	0	0	0	276
Honório Gurgel	21.989	4	20	44	62	29	3	6	1	1	0	0	0	170

Oswaldo Cruz	34.040	11	23	59	66	22	6	3	2	1	3	0	1	197
Bento Ribeiro	43.707	10	38	65	102	39	14	7	2	3	1	5	2	288
Marechal Hermes	48.061	7	38	63	101	42	16	4	2	3	1	3	2	282
**XXII Anchieta**	**158 318**	**54**	**109**	**234**	**228**	**89**	**38**	**12**	**0**	**2**	**7**	**4**	**6**	**783**
Guadalupe	47.144	16	43	92	111	50	20	6	0	1	4	2	4	349
Anchieta	55.652	28	48	100	66	20	12	6	0	1	3	1	0	285
Parque Anchieta	26.212	7	7	26	19	4	0	0	0	0	0	1	0	64
Ricardo de Albuquerque	29.310	3	11	16	32	15	6	0	0	0	0	0	2	85
**XXV Pavuna**	**208 813**	**52**	**159**	**391**	**353**	**133**	**64**	**27**	**7**	**13**	**1**	**9**	**1**	**1.210**
Coelho Neto	32.423	16	28	73	73	27	9	5	0	1	0	3	0	235
Acari	27.347	6	45	75	52	15	11	4	1	2	0	0	0	211
Barros Filho	14.049	1	9	23	23	14	4	2	0	0	0	1	0	77
Costa Barros	28.442	5	11	36	28	11	7	3	1	0	0	2	1	105
Pavuna	97.350	24	66	184	177	66	33	13	5	10	1	3	0	582
Parque Columbia	9 202	0	0	0	0	0	0	0	0	0	0	0	0	0
**Área Programática 4.0**	**909.368**	**511**	**972**	**2.543**	**2.955**	**584**	**147**	**71**	**21**	**22**	**35**	**41**	**35**	**7.937**
**Xvi Jacarepagua**	**572.030**	**389**	**737**	**1.640**	**1.788**	**312**	**97**	**53**	**11**	**15**	**18**	**35**	**32**	**5.127**
Jacarepaguá	157.326	37	58	205	210	54	9	2	2	4	4	2	4	591
Anil	24.172	21	25	86	65	18	5	3	1	2	2	3	3	234
Gardênia Azul	17.715	3	22	85	91	19	5	0	0	0	0	0	1	226
Curicica	31.189	15	42	101	137	32	6	4	0	0	1	0	1	339
Freguesia (Jacarepaguá)	70.511	39	70	143	201	25	12	12	0	2	3	5	1	513
Pechincha	34.709	37	55	87	107	15	6	3	2	2	1	2	3	320
Taquara	102.126	98	226	336	443	67	24	14	2	0	2	6	4	1.222
Tanque	37.856	36	73	115	97	11	4	0	0	0	2	2	0	340
Praça Seca	64.147	72	120	373	316	47	19	9	3	4	1	9	11	984
Vila Valqueire	32.279	31	46	109	121	24	7	6	1	1	2	6	4	358
**XXXIV Cidade De Deus**	**36.515**	**7**	**19**	**41**	**44**	**14**	**2**	**2**	**0**	**3**	**0**	**0**	**0**	**132**
Cidade de Deus	36.515	7	19	41	44	14	2	2	0	3	0	0	0	132
**XXIV Barra Da Tijuca**	**300.823**	**115**	**216**	**862**	**1.123**	**258**	**48**	**16**	**10**	**4**	**17**	**6**	**3**	**2.678**
Joá	818	2	0	4	2	0	0	0	0	0	0	0	0	8
Itanhangá	38.415	12	21	83	72	27	6	2	2	0	1	3	0	229
Barra da Tijuca	135.924	56	94	309	382	54	19	4	4	0	11	2	3	938
Camorim	1.970	2	0	10	10	4	0	1	0	1	0	0	0	28
Vargem Pequena	27.250	6	11	47	108	21	1	0	0	0	0	0	0	194
Vargem Grande	14.039	9	8	63	80	24	1	0	0	0	1	1	0	187
Recreio dos Bandeirantes	82.240	28	82	346	468	127	21	9	4	3	4	0	0	1.092

Grumari	167	0	0	0	1	1	0	0	0	0	0	0	0	2
**Área Programática 5.1**	**671.041**	**429**	**1.106**	**2.587**	**2.274**	**748**	**234**	**92**	**41**	**37**	**42**	**47**	**50**	**7.687**
**XXXIII Realengo**	**243.006**	**140**	**352**	**910**	**885**	**290**	**69**	**28**	**8**	**8**	**9**	**9**	**14**	**2.722**
Deodoro	10.842	5	14	34	31	18	5	1	0	0	0	0	1	109
Vila Militar	13.184	1	4	7	7	3	0	0	0	0	0	0	0	22
Campo dos Afonsos	1.365	1	4	6	1	0	0	0	0	0	0	0	0	12
Jardim Sulacap	13.062	8	30	61	102	31	6	2	0	1	0	1	2	244
Magalhães Bastos	24.430	8	49	98	62	39	5	4	1	1	0	1	1	269
Realengo	180.123	117	251	704	682	199	53	21	7	6	9	7	10	2.066
**XVII Bangu**	**428.035**	**289**	**754**	**1.677**	**1.389**	**458**	**165**	**64**	**33**	**29**	**33**	**38**	**36**	**4.965**
Padre Miguel	64.228	64	146	272	250	72	28	15	2	3	2	7	6	867
Bangu	243.125	181	503	1.136	922	301	97	32	27	19	21	27	23	3.289
Senador Camará	105.515	44	105	269	217	85	40	17	4	7	10	4	7	809
Gericino	15.167	0	0	0	0	0	0	0	0	0	0	0	0	0
**Área Programática 5.2**	**665.198**	**394**	**772**	**2.222**	**2.549**	**624**	**144**	**40**	**40**	**13**	**13**	**15**	**9**	**6.835**
**XVIII Campo Grande**	**542.084**	**204**	**474**	**1.242**	**1.649**	**312**	**77**	**19**	**27**	**10**	**12**	**12**	**8**	**4.046**
Santíssimo	41.458	27	65	161	163	40	11	5	8	1	2	4	2	489
Campo Grande	328.370	113	269	754	1.004	202	43	10	13	8	7	6	4	2.433
Senador Vasconcelos	30.600	3	11	20	42	11	3	0	1	0	0	0	1	92
Inhoaíba	64.649	32	50	120	154	24	6	1	1	1	1	1	1	392
Cosmos	77.007	29	79	187	286	35	14	3	4	0	2	1	0	640
**XXVI Guaratiba**	**123.114**	**190**	**298**	**980**	**900**	**312**	**67**	**21**	**13**	**3**	**1**	**3**	**1**	**2.789**
Guaratiba	110.049	99	205	605	604	240	55	14	10	3	1	2	0	1.838
Barra de Guaratiba	3.577	21	12	33	44	10	7	4	1	0	0	0	0	132
Pedra de Guaratiba	9.488	70	81	342	252	62	5	3	2	0	0	1	1	819
**Área Programática 5.3**	**368.534**	**137**	**329**	**1.140**	**947**	**365**	**128**	**40**	**18**	**26**	**20**	**12**	**11**	**3.173**
**XIX Santa Cruz**	**368.534**	**137**	**329**	**1.140**	**947**	**365**	**128**	**40**	**18**	**26**	**20**	**12**	**11**	**3.173**
Paciência	94.626	52	174	337	204	53	29	10	4	4	5	3	4	879
Santa Cruz	217.333	78	141	681	627	271	87	28	12	21	14	9	6	1.975
Sepetiba	56.575	7	14	122	116	41	12	2	2	1	1	0	1	319

* Distribuição dos dados de acordo com o local de residência referido
** Sem informação de Bairro e/ou Distrito de Residência
Fonte de Informação Epidemiológica: S/SUBPAV/COORDENAÇÃO DE VIGILÂNCIA EPIDEMIOLÓGICA - SINAN - MRJ
Obs. 1: Os dados tabulados referem-se aos atendimentos realizados em unidades do Município do Rio de Janeiro.
Obs. 2: Os casos estão agrupados de acordo com o ano de notificação.
Dados Atualizados em: 30/01/2014 (dados sujeitos a alteração)
