# Supplementary material for: Forecasting dengue fever in Brazil: An assessment of climate conditions
Source: PLoS One. 2019 Aug 8;14(8):e0220106. doi: 10.1371/journal.pone.0220106 (PMC6687106; doi:10.1371/journal.pone.0220106)
Supplement: S1 File — (ZIP) [file pone.0220106.s003.zip › data and codes_submit/training dataset analysis/data/Data IBGE/pop_municipios_2009.pdf]

| ESTIMATIVAS DAS POPULAÇÕES RESIDENTES, EM 1º DE JULHO DE 2009, SEGUNDO OS MUNICÍPIOS | | | | |
|---|---|---|---|---|
| Nota: (*) EM DECORRÊNCIA DE DECISÃO JUDICIAL | | | | |
| U.F. | | MUNICÍPIOS | | POPULAÇÃO ESTIMADA |
| SIGLA | COD | COD | NOMES | |
| RO | 11 | 15 | Alta Floresta D'Oeste | 24.354 |
| RO | 11 | 379 | Alto Alegre dos Parecis | 11.875 |
| RO | 11 | 403 | Alto Paraíso | 17.569 |
| RO | 11 | 346 | Alvorada D'Oeste | 17.127 |
| RO | 11 | 23 | Ariquemes | 85.541 |
| RO | 11 | 452 | Buritis | 34.693 |
| RO | 11 | 31 | Cabixi | 6.695 |
| RO | 11 | 601 | Cacaulândia | 5.736 |
| RO | 11 | 49 | Cacoal | 78.675 |
| RO | 11 | 700 | Campo Novo de Rondônia | 12.915 |
| RO | 11 | 809 | Candeias do Jamari | 17.547 |
| RO | 11 | 908 | Castanheiras | 3.685 |
| RO | 11 | 56 | Cerejeiras | 16.622 |
| RO | 11 | 924 | Chupinguaia | 7.843 |
| RO | 11 | 64 | Colorado do Oeste | 17.822 |
| RO | 11 | 72 | Corumbiara | 9.680 |
| RO | 11 | 80 | Costa Marques | 14.452 |
| RO | 11 | 940 | Cujubim | 14.889 |
| RO | 11 | 98 | Espigão D'Oeste | 28.892 |
| RO | 11 | 1005 | Governador Jorge Teixeira | 11.593 |
| RO | 11 | 106 | Guajará-Mirim | 40.762 |
| RO | 11 | 1104 | Itapuã do Oeste | 8.235 |
| RO | 11 | 114 | Jaru | 53.943 |
| RO | 11 | 122 | Ji-Paraná | 111.010 |
| RO | 11 | 130 | Machadinho D'Oeste | 33.159 |
| RO | 11 | 1203 | Ministro Andreazza | 10.572 |
| RO | 11 | 1302 | Mirante da Serra | 12.363 |
| RO | 11 | 1401 | Monte Negro | 12.708 |
| RO | 11 | 148 | Nova Brasilândia D'Oeste | 17.698 |
| RO | 11 | 338 | Nova Mamoré | 22.337 |
| RO | 11 | 1435 | Nova União | 7.944 |
| RO | 11 | 502 | Novo Horizonte do Oeste | 9.720 |
| RO | 11 | 155 | Ouro Preto do Oeste | 36.725 |
| RO | 11 | 1450 | Parecis | 4.802 |
| RO | 11 | 189 | Pimenta Bueno | 33.984 |
| RO | 11 | 1468 | Pimenteiras do Oeste | 2.415 |
| RO | 11 | 205 | Porto Velho | 382.829 |
| RO | 11 | 254 | Presidente Médici | 22.519 |
| RO | 11 | 1476 | Primavera de Rondônia | 3.765 |
| RO | 11 | 262 | Rio Crespo | 3.290 |
| RO | 11 | 288 | Rolim de Moura | 50.499 |
| RO | 11 | 296 | Santa Luzia D'Oeste | 9.348 |
| RO | 11 | 1484 | São Felipe D'Oeste | 6.412 |
| RO | 11 | 1492 | São Francisco do Guaporé | 16.491 |
| RO | 11 | 320 | São Miguel do Guaporé | 23.201 |

| U.F. | | MUNICÍPIOS | | POPULAÇÃO ESTIMADA |
|---|---|---|---|---|
| SIGLA | COD | COD | NOMES | |
| RO | 11 | 1500 | Seringueiras | 12.117 |
| RO | 11 | 1559 | Teixeirópolis | 5.010 |
| RO | 11 | 1609 | Theobroma | 10.325 |
| RO | 11 | 1708 | Urupá | 13.661 |
| RO | 11 | 1757 | Vale do Anari | 9.100 |
| RO | 11 | 1807 | Vale do Paraíso | 8.913 |
| RO | 11 | 304 | Vilhena | 69.866 |
| AC | 12 | 13 | Acrelândia | 12.241 |
| AC | 12 | 54 | Assis Brasil | 5.662 |
| AC | 12 | 104 | Brasiléia | 20.238 |
| AC | 12 | 138 | Bujari | 6.772 |
| AC | 12 | 179 | Capixaba | 9.287 |
| AC | 12 | 203 | Cruzeiro do Sul | 77.004 |
| AC | 12 | 252 | Epitaciolândia | 14.224 |
| AC | 12 | 302 | Feijó | 32.261 |
| AC | 12 | 328 | Jordão | 6.520 |
| AC | 12 | 336 | Mâncio Lima | 14.774 |
| AC | 12 | 344 | Manoel Urbano | 7.505 |
| AC | 12 | 351 | Marechal Thaumaturgo | 14.275 |
| AC | 12 | 385 | Plácido de Castro | 18.235 |
| AC | 12 | 807 | Porto Acre | 14.682 |
| AC | 12 | 393 | Porto Walter | 8.855 |
| AC | 12 | 401 | Rio Branco | 305.954 |
| AC | 12 | 427 | Rodrigues Alves | 13.460 |
| AC | 12 | 435 | Santa Rosa do Purus | 4.358 |
| AC | 12 | 500 | Sena Madureira | 36.166 |
| AC | 12 | 450 | Senador Guiomard | 19.697 |
| AC | 12 | 609 | Tarauacá | 33.883 |
| AC | 12 | 708 | Xapuri | 15.079 |
| AM | 13 | 29 | Alvarães | 13.566 |
| AM | 13 | 60 | Amaturá | 8.828 |
| AM | 13 | 86 | Anamã | 8.673 |
| AM | 13 | 102 | Anori | 14.688 |
| AM | 13 | 144 | Apuí | 18.597 |
| AM | 13 | 201 | Atalaia do Norte | 14.715 |
| AM | 13 | 300 | Autazes | 31.774 |
| AM | 13 | 409 | Barcelos | 25.410 |
| AM | 13 | 508 | Barreirinha | 28.162 |
| AM | 13 | 607 | Benjamin Constant | 31.195 |
| AM | 13 | 631 | Beruri | 14.705 |
| AM | 13 | 680 | Boa Vista do Ramos | 13.994 |
| AM | 13 | 706 | Boca do Acre | 31.221 |
| AM | 13 | 805 | Borba | 32.496 |
| AM | 13 | 839 | Caapiranga | 11.170 |
| AM | 13 | 904 | Canutama | 11.948 |
| AM | 13 | 1001 | Carauari | 26.187 |
| AM | 13 | 1100 | Careiro | 32.638 |
| AM | 13 | 1159 | Careiro da Várzea | 24.704 |
| AM | 13 | 1209 | Coari | 66.991 |
| AM | 13 | 1308 | Codajás | 16.291 |

| U.F. | | MUNICÍPIOS | | POPULAÇÃO ESTIMADA |
|---|---|---|---|---|
| SIGLA | COD | COD | NOMES | |
| AM | 13 | 1407 | Eirunepé | 30.901 |
| AM | 13 | 1506 | Envira | 17.614 |
| AM | 13 | 1605 | Fonte Boa | 18.803 |
| AM | 13 | 1654 | Guajará | 15.031 |
| AM | 13 | 1704 | Humaitá | 40.735 |
| AM | 13 | 1803 | Ipixuna | 18.202 |
| AM | 13 | 1852 | Iranduba | 33.884 |
| AM | 13 | 1902 | Itacoatiara | 89.440 |
| AM | 13 | 1951 | Itamarati | 8.281 |
| AM | 13 | 2009 | Itapiranga | 9.733 |
| AM | 13 | 2108 | Japurá | 4.238 |
| AM | 13 | 2207 | Juruá | 9.275 |
| AM | 13 | 2306 | Jutaí | 16.791 |
| AM | 13 | 2405 | Lábrea | 39.393 |
| AM | 13 | 2504 | Manacapuru | 86.472 |
| AM | 13 | 2553 | Manaquiri | 20.836 |
| AM | 13 | 2603 | Manaus | 1.738.641 |
| AM | 13 | 2702 | Manicoré | 46.773 |
| AM | 13 | 2801 | Maraã | 18.135 |
| AM | 13 | 2900 | Maués | 49.666 |
| AM | 13 | 3007 | Nhamundá | 18.474 |
| AM | 13 | 3106 | Nova Olinda do Norte | 31.012 |
| AM | 13 | 3205 | Novo Airão | 15.915 |
| AM | 13 | 3304 | Novo Aripuanã | 18.952 |
| AM | 13 | 3403 | Parintins | 107.250 |
| AM | 13 | 3502 | Pauini | 19.111 |
| AM | 13 | 3536 | Presidente Figueiredo | 26.282 |
| AM | 13 | 3569 | Rio Preto da Eva | 26.847 |
| AM | 13 | 3601 | Santa Isabel do Rio Negro | 18.506 |
| AM | 13 | 3700 | Santo Antônio do Içá | 30.351 |
| AM | 13 | 3809 | São Gabriel da Cachoeira | 41.885 |
| AM | 13 | 3908 | São Paulo de Olivença | 32.958 |
| AM | 13 | 3957 | São Sebastião do Uatumã | 9.268 |
| AM | 13 | 4005 | Silves | 8.543 |
| AM | 13 | 4062 | Tabatinga | 47.948 |
| AM | 13 | 4104 | Tapauá | 19.884 |
| AM | 13 | 4203 | Tefé | 64.671 |
| AM | 13 | 4237 | Tonantins | 20.286 |
| AM | 13 | 4260 | Uarini | 10.108 |
| AM | 13 | 4302 | Urucará | 15.780 |
| AM | 13 | 4401 | Urucurituba | 18.541 |
| RR | 14 | 50 | Alto Alegre | 14.205 |
| RR | 14 | 27 | Amajari | 8.249 |
| RR | 14 | 100 | Boa Vista | 266.901 |
| RR | 14 | 159 | Bonfim | 10.726 |
| RR | 14 | 175 | Cantá | 11.942 |
| RR | 14 | 209 | Caracaraí | 19.235 |
| RR | 14 | 233 | Caroebe | 7.569 |
| RR | 14 | 282 | Iracema | 6.250 |
| RR | 14 | 308 | Mucajaí | 13.188 |

| U.F. | | MUNICÍPIOS | | POPULAÇÃO |
|---|---|---|---|---|
| SIGLA | COD | COD | NOMES | ESTIMADA |
| RR | 14 | 407 | Normandia | 7.527 |
| RR | 14 | 456 | Pacaraima | 9.220 |
| RR | 14 | 472 | Rorainópolis | 26.546 |
| RR | 14 | 506 | São João da Baliza | 6.028 |
| RR | 14 | 605 | São Luiz | 5.979 |
| RR | 14 | 704 | Uiramutã | 7.934 |
| PA | 15 | 107 | Abaetetuba | 139.819 |
| PA | 15 | 131 | Abel Figueiredo | 6.967 |
| PA | 15 | 206 | Acará | 48.501 |
| PA | 15 | 305 | Afuá | 32.633 |
| PA | 15 | 347 | Água Azul do Norte | 31.216 |
| PA | 15 | 404 | Alenquer | 57.067 |
| PA | 15 | 503 | Almeirim | 31.192 |
| PA | 15 | 602 | Altamira | 98.750 |
| PA | 15 | 701 | Anajás | 27.386 |
| PA | 15 | 800 | Ananindeua | 505.512 |
| PA | 15 | 859 | Anapu | 20.421 |
| PA | 15 | 909 | Augusto Corrêa | 39.317 |
| PA | 15 | 958 | Aurora do Pará | 22.315 |
| PA | 15 | 1006 | Aveiro | 20.266 |
| PA | 15 | 1105 | Bagre | 20.386 |
| PA | 15 | 1204 | Baião | 28.299 |
| PA | 15 | 1253 | Bannach | 3.947 |
| PA | 15 | 1303 | Barcarena | 92.567 |
| PA | 15 | 1402 | Belém | 1.437.600 |
| PA | 15 | 1451 | Belterra | 12.671 |
| PA | 15 | 1501 | Benevides | 46.611 |
| PA | 15 | 1576 | Bom Jesus do Tocantins | 13.593 |
| PA | 15 | 1600 | Bonito | 12.013 |
| PA | 15 | 1709 | Bragança | 107.060 |
| PA | 15 | 1725 | Brasil Novo | 19.754 |
| PA | 15 | 1758 | Brejo Grande do Araguaia | 7.688 |
| PA | 15 | 1782 | Breu Branco | 52.200 |
| PA | 15 | 1808 | Breves | 101.094 |
| PA | 15 | 1907 | Bujaru | 23.654 |
| PA | 15 | 2004 | Cachoeira do Arari | 20.411 |
| PA | 15 | 1956 | Cachoeira do Piriá | 18.777 |
| PA | 15 | 2103 | Cametá | 117.099 |
| PA | 15 | 2152 | Canaã dos Carajás | 27.675 |
| PA | 15 | 2202 | Capanema | 64.429 |
| PA | 15 | 2301 | Capitão Poço | 52.797 |
| PA | 15 | 2400 | Castanhal | 161.497 |
| PA | 15 | 2509 | Chaves | 20.506 |
| PA | 15 | 2608 | Colares | 11.433 |
| PA | 15 | 2707 | Conceição do Araguaia | 47.237 |
| PA | 15 | 2756 | Concórdia do Pará | 22.251 |
| PA | 15 | 2764 | Cumaru do Norte | 11.890 |
| PA | 15 | 2772 | Curionópolis | 17.944 |
| PA | 15 | 2806 | Curralinho | 27.543 |
| PA | 15 | 2855 | Curuá | 12.984 |

| U.F. | | MUNICÍPIOS | | POPULAÇÃO |
|---|---|---|---|---|
| SIGLA | COD | COD | NOMES | ESTIMADA |
| PA | 15 | 2905 | Curuçá | 36.748 |
| PA | 15 | 2939 | Dom Eliseu | 39.088 |
| PA | 15 | 2954 | Eldorado dos Carajás | 29.251 |
| PA | 15 | 3002 | Faro | 19.585 |
| PA | 15 | 3044 | Floresta do Araguaia | 15.629 |
| PA | 15 | 3077 | Garrafão do Norte | 25.538 |
| PA | 15 | 3093 | Goianésia do Pará | 29.164 |
| PA | 15 | 3101 | Gurupá | 25.511 |
| PA | 15 | 3200 | Igarapé-Açu | 35.241 |
| PA | 15 | 3309 | Igarapé-Miri | 57.003 |
| PA | 15 | 3408 | Inhangapi | 10.377 |
| PA | 15 | 3457 | Ipixuna do Pará | 44.396 |
| PA | 15 | 3507 | Irituia | 30.552 |
| PA | 15 | 3606 | Itaituba | 127.848 |
| PA | 15 | 3705 | Itupiranga | 41.541 |
| PA | 15 | 3754 | Jacareacanga | 41.487 |
| PA | 15 | 3804 | Jacundá | 55.900 |
| PA | 15 | 3903 | Juruti | 35.530 |
| PA | 15 | 4000 | Limoeiro do Ajuru | 24.967 |
| PA | 15 | 4059 | Mãe do Rio | 29.087 |
| PA | 15 | 4109 | Magalhães Barata | 7.895 |
| PA | 15 | 4208 | Marabá | 203.049 |
| PA | 15 | 4307 | Maracanã | 29.417 |
| PA | 15 | 4406 | Marapanim | 28.011 |
| PA | 15 | 4422 | Marituba | 101.158 |
| PA | 15 | 4455 | Medicilândia | 23.682 |
| PA | 15 | 4505 | Melgaço | 17.657 |
| PA | 15 | 4604 | Mocajuba | 24.695 |
| PA | 15 | 4703 | Moju | 68.600 |
| PA | 15 | 4802 | Monte Alegre | 63.941 |
| PA | 15 | 4901 | Muaná | 30.568 |
| PA | 15 | 4950 | Nova Esperança do Piriá | 24.062 |
| PA | 15 | 4976 | Nova Ipixuna | 15.097 |
| PA | 15 | 5007 | Nova Timboteua | 12.677 |
| PA | 15 | 5031 | Novo Progresso | 21.504 |
| PA | 15 | 5064 | Novo Repartimento | 55.762 |
| PA | 15 | 5106 | Óbidos | 48.429 |
| PA | 15 | 5205 | Oeiras do Pará | 26.796 |
| PA | 15 | 5304 | Oriximiná | 58.683 |
| PA | 15 | 5403 | Ourém | 15.841 |
| PA | 15 | 5437 | Ourilândia do Norte | 21.327 |
| PA | 15 | 5486 | Pacajá | 41.953 |
| PA | 15 | 5494 | Palestina do Pará | 7.301 |
| PA | 15 | 5502 | Paragominas | 97.350 |
| PA | 15 | 5536 | Parauapebas | 152.777 |
| PA | 15 | 5551 | Pau D'Arco | 6.522 |
| PA | 15 | 5601 | Peixe-Boi | 7.916 |
| PA | 15 | 5635 | Piçarra | 13.140 |
| PA | 15 | 5650 | Placas | 19.592 |
| PA | 15 | 5700 | Ponta de Pedras | 26.445 |

| U.F. | | MUNICÍPIOS | | POPULAÇÃO |
|---|---|---|---|---|
| SIGLA | COD | COD | NOMES | ESTIMADA |
| PA | 15 | 5809 | Portel | 48.945 |
| PA | 15 | 5908 | Porto de Moz | 28.091 |
| PA | 15 | 6005 | Prainha | 26.570 |
| PA | 15 | 6104 | Primavera | 10.993 |
| PA | 15 | 6112 | Quatipuru | 13.459 |
| PA | 15 | 6138 | Redenção | 67.064 |
| PA | 15 | 6161 | Rio Maria | 17.437 |
| PA | 15 | 6187 | Rondon do Pará | 47.772 |
| PA | 15 | 6195 | Rurópolis | 36.068 |
| PA | 15 | 6203 | Salinópolis | 39.184 |
| PA | 15 | 6302 | Salvaterra | 18.124 |
| PA | 15 | 6351 | Santa Bárbara do Pará | 14.740 |
| PA | 15 | 6401 | Santa Cruz do Arari | 6.280 |
| PA | 15 | 6500 | Santa Isabel do Pará | 55.570 |
| PA | 15 | 6559 | Santa Luzia do Pará | 18.417 |
| PA | 15 | 6583 | Santa Maria das Barreiras | 17.778 |
| PA | 15 | 6609 | Santa Maria do Pará | 23.202 |
| PA | 15 | 6708 | Santana do Araguaia | 55.033 |
| PA | 15 | 6807 | Santarém | 276.665 |
| PA | 15 | 6906 | Santarém Novo | 6.347 |
| PA | 15 | 7003 | Santo Antônio do Tauá | 26.855 |
| PA | 15 | 7102 | São Caetano de Odivelas | 16.862 |
| PA | 15 | 7151 | São Domingos do Araguaia | 22.063 |
| PA | 15 | 7201 | São Domingos do Capim | 27.923 |
| PA | 15 | 7300 | São Félix do Xingu | 67.208 |
| PA | 15 | 7409 | São Francisco do Pará | 11.743 |
| PA | 15 | 7458 | São Geraldo do Araguaia | 25.027 |
| PA | 15 | 7466 | São João da Ponta | 5.038 |
| PA | 15 | 7474 | São João de Pirabas | 19.900 |
| PA | 15 | 7508 | São João do Araguaia | 11.923 |
| PA | 15 | 7607 | São Miguel do Guamá | 44.818 |
| PA | 15 | 7706 | São Sebastião da Boa Vista | 21.874 |
| PA | 15 | 7755 | Sapucaia | 5.609 |
| PA | 15 | 7805 | Senador José Porfírio | 14.434 |
| PA | 15 | 7904 | Soure | 22.459 |
| PA | 15 | 7953 | Tailândia | 72.720 |
| PA | 15 | 7961 | Terra Alta | 10.580 |
| PA | 15 | 7979 | Terra Santa | 16.004 |
| PA | 15 | 8001 | Tomé-Açu | 48.607 |
| PA | 15 | 8035 | Tracuateua | 27.825 |
| PA | 15 | 8050 | Trairão | 17.134 |
| PA | 15 | 8084 | Tucumã | 27.691 |
| PA | 15 | 8100 | Tucuruí | 96.010 |
| PA | 15 | 8126 | Ulianópolis | 36.020 |
| PA | 15 | 8159 | Uruará | 59.881 (*) |
| PA | 15 | 8209 | Vigia | 46.205 |
| PA | 15 | 8308 | Viseu | 55.512 |
| PA | 15 | 8357 | Vitória do Xingu | 9.664 |
| PA | 15 | 8407 | Xinguara | 40.529 |
| AP | 16 | 105 | Amapá | 7.802 |

| U.F. | | MUNICÍPIOS | | POPULAÇÃO ESTIMADA |
|---|---|---|---|---|
| SIGLA | COD | COD | NOMES | |
| AP | 16 | 204 | Calçoene | 9.291 |
| AP | 16 | 212 | Cutias | 4.652 |
| AP | 16 | 238 | Ferreira Gomes | 5.475 |
| AP | 16 | 253 | Itaubal | 3.650 |
| AP | 16 | 279 | Laranjal do Jari | 40.357 |
| AP | 16 | 303 | Macapá | 366.484 |
| AP | 16 | 402 | Mazagão | 14.655 |
| AP | 16 | 501 | Oiapoque | 20.962 |
| AP | 16 | 154 | Pedra Branca do Amapari | 8.182 |
| AP | 16 | 535 | Porto Grande | 14.951 |
| AP | 16 | 550 | Pracuúba | 3.658 |
| AP | 16 | 600 | Santana | 97.220 |
| AP | 16 | 55 | Serra do Navio | 3.982 |
| AP | 16 | 709 | Tartarugalzinho | 13.769 |
| AP | 16 | 808 | Vitória do Jari | 11.519 |
| TO | 17 | 251 | Abreulândia | 2.321 |
| TO | 17 | 301 | Aguiarnópolis | 4.216 |
| TO | 17 | 350 | Aliança do Tocantins | 5.822 |
| TO | 17 | 400 | Almas | 7.605 |
| TO | 17 | 707 | Alvorada | 8.161 |
| TO | 17 | 1002 | Ananás | 9.514 |
| TO | 17 | 1051 | Angico | 3.300 |
| TO | 17 | 1101 | Aparecida do Rio Negro | 4.200 |
| TO | 17 | 1309 | Aragominas | 5.555 |
| TO | 17 | 1903 | Araguacema | 5.591 |
| TO | 17 | 2000 | Araguaçu | 9.225 |
| TO | 17 | 2109 | Araguaína | 119.637 |
| TO | 17 | 2158 | Araguanã | 5.248 |
| TO | 17 | 2208 | Araguatins | 26.771 |
| TO | 17 | 2307 | Arapoema | 7.029 |
| TO | 17 | 2406 | Arraias | 10.913 |
| TO | 17 | 2554 | Augustinópolis | 15.469 |
| TO | 17 | 2703 | Aurora do Tocantins | 3.523 |
| TO | 17 | 2901 | Axixá do Tocantins | 9.203 |
| TO | 17 | 3008 | Babaçulândia | 10.698 |
| TO | 17 | 3057 | Bandeirantes do Tocantins | 2.807 |
| TO | 17 | 3073 | Barra do Ouro | 3.691 |
| TO | 17 | 3107 | Barrolândia | 5.322 |
| TO | 17 | 3206 | Bernardo Sayão | 4.653 |
| TO | 17 | 3305 | Bom Jesus do Tocantins | 2.839 |
| TO | 17 | 3602 | Brasilândia do Tocantins | 2.208 |
| TO | 17 | 3701 | Brejinho de Nazaré | 5.506 |
| TO | 17 | 3800 | Buriti do Tocantins | 8.454 |
| TO | 17 | 3826 | Cachoeirinha | 2.255 |
| TO | 17 | 3842 | Campos Lindos | 8.079 |
| TO | 17 | 3867 | Cariri do Tocantins | 3.738 |
| TO | 17 | 3883 | Carmolândia | 2.420 |
| TO | 17 | 3891 | Carrasco Bonito | 3.428 |
| TO | 17 | 3909 | Caseara | 4.927 |
| TO | 17 | 4105 | Centenário | 2.485 |

| U.F. | | MUNICÍPIOS | | POPULAÇÃO ESTIMADA |
|---|---|---|---|---|
| SIGLA | COD | COD | NOMES | |
| TO | 17 | 5102 | Chapada da Natividade | 3.840 |
| TO | 17 | 4600 | Chapada de Areia | 1.273 |
| TO | 17 | 5508 | Colinas do Tocantins | 30.666 |
| TO | 17 | 16703 | Colméia | 8.961 |
| TO | 17 | 5557 | Combinado | 5.070 |
| TO | 17 | 5607 | Conceição do Tocantins | 4.541 |
| TO | 17 | 6001 | Couto de Magalhães | 5.102 |
| TO | 17 | 6100 | Cristalândia | 6.628 |
| TO | 17 | 6258 | Crixás do Tocantins | 1.289 |
| TO | 17 | 6506 | Darcinópolis | 5.388 |
| TO | 17 | 7009 | Dianópolis | 19.524 |
| TO | 17 | 7108 | Divinópolis do Tocantins | 6.623 |
| TO | 17 | 7207 | Dois Irmãos do Tocantins | 7.254 |
| TO | 17 | 7306 | Dueré | 4.618 |
| TO | 17 | 7405 | Esperantina | 8.445 |
| TO | 17 | 7553 | Fátima | 4.123 |
| TO | 17 | 7652 | Figueirópolis | 4.883 |
| TO | 17 | 7702 | Filadélfia | 7.978 |
| TO | 17 | 8205 | Formoso do Araguaia | 18.719 |
| TO | 17 | 8254 | Fortaleza do Tabocão | 2.149 |
| TO | 17 | 8304 | Goianorte | 5.426 |
| TO | 17 | 9005 | Goiatins | 12.068 |
| TO | 17 | 9302 | Guaraí | 22.530 |
| TO | 17 | 9500 | Gurupi | 74.357 |
| TO | 17 | 9807 | Ipueiras | 1.813 |
| TO | 17 | 10508 | Itacajá | 6.534 |
| TO | 17 | 10706 | Itaguatins | 6.226 |
| TO | 17 | 10904 | Itapiratins | 3.543 |
| TO | 17 | 11100 | Itaporã do Tocantins | 3.135 |
| TO | 17 | 11506 | Jaú do Tocantins | 3.983 |
| TO | 17 | 11803 | Juarina | 2.185 |
| TO | 17 | 11902 | Lagoa da Confusão | 8.711 |
| TO | 17 | 11951 | Lagoa do Tocantins | 3.352 |
| TO | 17 | 12009 | Lajeado | 2.204 |
| TO | 17 | 12157 | Lavandeira | 1.683 |
| TO | 17 | 12405 | Lizarda | 3.729 |
| TO | 17 | 12454 | Luzinópolis | 2.959 |
| TO | 17 | 12504 | Marianópolis do Tocantins | 4.743 |
| TO | 17 | 12702 | Mateiros | 1.802 |
| TO | 17 | 12801 | Maurilândia do Tocantins | 3.322 |
| TO | 17 | 13205 | Miracema do Tocantins | 19.740 |
| TO | 17 | 13304 | Miranorte | 12.231 |
| TO | 17 | 13601 | Monte do Carmo | 6.723 |
| TO | 17 | 13700 | Monte Santo do Tocantins | 1.914 |
| TO | 17 | 13957 | Muricilândia | 2.958 |
| TO | 17 | 14203 | Natividade | 9.396 |
| TO | 17 | 14302 | Nazaré | 4.596 |
| TO | 17 | 14880 | Nova Olinda | 10.974 |
| TO | 17 | 15002 | Nova Rosalândia | 3.956 |
| TO | 17 | 15101 | Novo Acordo | 3.950 |

| U.F. | | MUNICÍPIOS | | POPULAÇÃO ESTIMADA |
|---|---|---|---|---|
| SIGLA | COD | COD | NOMES | |
| TO | 17 | 15150 | Novo Alegre | 1.802 |
| TO | 17 | 15259 | Novo Jardim | 2.525 |
| TO | 17 | 15507 | Oliveira de Fátima | 1.129 |
| TO | 17 | 21000 | Palmas | 188.645 |
| TO | 17 | 15705 | Palmeirante | 4.959 |
| TO | 17 | 13809 | Palmeiras do Tocantins | 4.673 |
| TO | 17 | 15754 | Palmeirópolis | 8.492 |
| TO | 17 | 16109 | Paraíso do Tocantins | 42.015 |
| TO | 17 | 16208 | Paranã | 10.824 |
| TO | 17 | 16307 | Pau D'Arco | 4.964 |
| TO | 17 | 16505 | Pedro Afonso | 10.758 |
| TO | 17 | 16604 | Peixe | 9.018 |
| TO | 17 | 16653 | Pequizeiro | 4.971 |
| TO | 17 | 17008 | Pindorama do Tocantins | 4.500 |
| TO | 17 | 17206 | Piraquê | 3.127 |
| TO | 17 | 17503 | Pium | 6.701 |
| TO | 17 | 17800 | Ponte Alta do Bom Jesus | 4.664 |
| TO | 17 | 17909 | Ponte Alta do Tocantins | 6.818 |
| TO | 17 | 18006 | Porto Alegre do Tocantins | 2.968 |
| TO | 17 | 18204 | Porto Nacional | 46.722 |
| TO | 17 | 18303 | Praia Norte | 7.310 |
| TO | 17 | 18402 | Presidente Kennedy | 3.784 |
| TO | 17 | 18451 | Pugmil | 2.252 |
| TO | 17 | 18501 | Recursolândia | 3.839 |
| TO | 17 | 18550 | Riachinho | 3.808 |
| TO | 17 | 18659 | Rio da Conceição | 1.530 |
| TO | 17 | 18709 | Rio dos Bois | 2.136 |
| TO | 17 | 18758 | Rio Sono | 6.366 |
| TO | 17 | 18808 | Sampaio | 3.886 |
| TO | 17 | 18840 | Sandolândia | 3.562 |
| TO | 17 | 18865 | Santa Fé do Araguaia | 5.795 |
| TO | 17 | 18881 | Santa Maria do Tocantins | 2.807 |
| TO | 17 | 18899 | Santa Rita do Tocantins | 2.377 |
| TO | 17 | 18907 | Santa Rosa do Tocantins | 4.565 |
| TO | 17 | 19004 | Santa Tereza do Tocantins | 2.390 |
| TO | 17 | 20002 | Santa Terezinha do Tocantins | 2.343 |
| TO | 17 | 20101 | São Bento do Tocantins | 4.666 |
| TO | 17 | 20150 | São Félix do Tocantins | 1.468 |
| TO | 17 | 20200 | São Miguel do Tocantins | 10.737 |
| TO | 17 | 20259 | São Salvador do Tocantins | 3.136 |
| TO | 17 | 20309 | São Sebastião do Tocantins | 4.441 |
| TO | 17 | 20499 | São Valério da Natividade | 5.017 |
| TO | 17 | 20655 | Silvanópolis | 5.299 |
| TO | 17 | 20804 | Sítio Novo do Tocantins | 9.568 |
| TO | 17 | 20853 | Sucupira | 1.741 |
| TO | 17 | 20903 | Taguatinga | 14.655 |
| TO | 17 | 20937 | Taipas do Tocantins | 1.999 |
| TO | 17 | 20978 | Talismã | 2.663 |
| TO | 17 | 21109 | Tocantínia | 6.971 |
| TO | 17 | 21208 | Tocantinópolis | 21.826 |

| U.F. | | MUNICÍPIOS | | POPULAÇÃO ESTIMADA |
|---|---|---|---|---|
| SIGLA | COD | COD | NOMES | |
| TO | 17 | 21257 | Tupirama | 1.474 |
| TO | 17 | 21307 | Tupiratins | 2.143 |
| TO | 17 | 22081 | Wanderlândia | 9.493 |
| TO | 17 | 22107 | Xambioá | 11.099 |
| MA | 21 | 55 | Açailândia | 101.130 |
| MA | 21 | 105 | Afonso Cunha | 5.948 |
| MA | 21 | 154 | Água Doce do Maranhão | 12.460 |
| MA | 21 | 204 | Alcântara | 22.020 |
| MA | 21 | 303 | Aldeias Altas | 22.667 |
| MA | 21 | 402 | Altamira do Maranhão | 7.771 |
| MA | 21 | 436 | Alto Alegre do Maranhão | 22.914 |
| MA | 21 | 477 | Alto Alegre do Pindaré | 33.211 |
| MA | 21 | 501 | Alto Parnaíba | 10.640 |
| MA | 21 | 550 | Amapá do Maranhão | 6.451 |
| MA | 21 | 600 | Amarante do Maranhão | 37.388 |
| MA | 21 | 709 | Anajatuba | 25.063 |
| MA | 21 | 808 | Anapurus | 13.257 |
| MA | 21 | 832 | Apicum-Açu | 13.890 |
| MA | 21 | 873 | Araguanã | 10.325 |
| MA | 21 | 907 | Araioses | 39.166 |
| MA | 21 | 956 | Arame | 27.750 |
| MA | 21 | 1004 | Arari | 28.787 |
| MA | 21 | 1103 | Axixá | 15.203 |
| MA | 21 | 1202 | Bacabal | 98.489 |
| MA | 21 | 1251 | Bacabeira | 15.574 |
| MA | 21 | 1301 | Bacuri | 16.585 |
| MA | 21 | 1350 | Bacurituba | 5.687 |
| MA | 21 | 1400 | Balsas | 83.617 |
| MA | 21 | 1509 | Barão de Grajaú | 17.231 |
| MA | 21 | 1608 | Barra do Corda | 81.329 |
| MA | 21 | 1707 | Barreirinhas | 50.354 |
| MA | 21 | 1772 | Bela Vista do Maranhão | 8.855 |
| MA | 21 | 1731 | Belágua | 5.953 |
| MA | 21 | 1806 | Benedito Leite | 5.567 |
| MA | 21 | 1905 | Bequimão | 21.508 |
| MA | 21 | 1939 | Bernardo do Mearim | 6.249 |
| MA | 21 | 1970 | Boa Vista do Gurupi | 7.895 |
| MA | 21 | 2002 | Bom Jardim | 39.224 |
| MA | 21 | 2036 | Bom Jesus das Selvas | 25.473 |
| MA | 21 | 2077 | Bom Lugar | 13.384 |
| MA | 21 | 2101 | Brejo | 32.453 |
| MA | 21 | 2150 | Brejo de Areia | 5.505 |
| MA | 21 | 2200 | Buriti | 26.202 |
| MA | 21 | 2309 | Buriti Bravo | 23.074 |
| MA | 21 | 2325 | Buriticupu | 64.685 |
| MA | 21 | 2358 | Buritirana | 15.051 |
| MA | 21 | 2374 | Cachoeira Grande | 9.286 |
| MA | 21 | 2408 | Cajapió | 10.337 |
| MA | 21 | 2507 | Cajari | 13.170 |
| MA | 21 | 2556 | Campestre do Maranhão | 12.716 |

| U.F. | | MUNICÍPIOS | | POPULAÇÃO ESTIMADA |
|---|---|---|---|---|
| SIGLA | COD | COD | NOMES | |
| MA | 21 | 2606 | Cândido Mendes | 19.684 |
| MA | 21 | 2705 | Cantanhede | 19.564 |
| MA | 21 | 2754 | Capinzal do Norte | 10.688 |
| MA | 21 | 2804 | Carolina | 25.257 |
| MA | 21 | 2903 | Carutapera | 21.121 |
| MA | 21 | 3000 | Caxias | 148.072 |
| MA | 21 | 3109 | Cedral | 10.152 |
| MA | 21 | 3125 | Central do Maranhão | 9.246 |
| MA | 21 | 3158 | Centro do Guilherme | 7.432 |
| MA | 21 | 3174 | Centro Novo do Maranhão | 15.668 |
| MA | 21 | 3208 | Chapadinha | 70.537 |
| MA | 21 | 3257 | Cidelândia | 12.866 |
| MA | 21 | 3307 | Codó | 113.937 |
| MA | 21 | 3406 | Coelho Neto | 45.624 |
| MA | 21 | 3505 | Colinas | 36.787 |
| MA | 21 | 3554 | Conceição do Lago-Açu | 14.909 |
| MA | 21 | 3604 | Coroatá | 63.081 |
| MA | 21 | 3703 | Cururupu | 35.108 |
| MA | 21 | 3752 | Davinópolis | 12.091 |
| MA | 21 | 3802 | Dom Pedro | 22.086 |
| MA | 21 | 3901 | Duque Bacelar | 10.827 |
| MA | 21 | 4008 | Esperantinópolis | 18.815 |
| MA | 21 | 4057 | Estreito | 27.756 |
| MA | 21 | 4073 | Feira Nova do Maranhão | 7.899 |
| MA | 21 | 4081 | Fernando Falcão | 8.765 |
| MA | 21 | 4099 | Formosa da Serra Negra | 17.792 |
| MA | 21 | 4107 | Fortaleza dos Nogueiras | 11.972 |
| MA | 21 | 4206 | Fortuna | 14.922 |
| MA | 21 | 4305 | Godofredo Viana | 11.207 |
| MA | 21 | 4404 | Gonçalves Dias | 16.911 |
| MA | 21 | 4503 | Governador Archer | 10.330 |
| MA | 21 | 4552 | Governador Edison Lobão | 14.921 |
| MA | 21 | 4602 | Governador Eugênio Barros | 16.503 |
| MA | 21 | 4628 | Governador Luiz Rocha | 7.144 |
| MA | 21 | 4651 | Governador Newton Bello | 11.642 |
| MA | 21 | 4677 | Governador Nunes Freire | 24.520 |
| MA | 21 | 4701 | Graça Aranha | 6.470 |
| MA | 21 | 4800 | Grajaú | 56.633 |
| MA | 21 | 4909 | Guimarães | 12.740 |
| MA | 21 | 5005 | Humberto de Campos | 25.403 |
| MA | 21 | 5104 | Icatu | 25.557 |
| MA | 21 | 5153 | Igarapé do Meio | 12.291 |
| MA | 21 | 5203 | Igarapé Grande | 11.121 |
| MA | 21 | 5302 | Imperatriz | 236.691 |
| MA | 21 | 5351 | Itaipava do Grajaú | 13.964 |
| MA | 21 | 5401 | Itapecuru Mirim | 56.810 |
| MA | 21 | 5427 | Itinga do Maranhão | 26.125 |
| MA | 21 | 5450 | Jatobá | 8.909 |
| MA | 21 | 5476 | Jenipapo dos Vieiras | 15.438 |
| MA | 21 | 5500 | João Lisboa | 20.395 |

| U.F. | | MUNICÍPIOS | | POPULAÇÃO ESTIMADA |
|---|---|---|---|---|
| SIGLA | COD | COD | NOMES | |
| MA | 21 | 5609 | Joselândia | 16.188 |
| MA | 21 | 5658 | Junco do Maranhão | 3.950 |
| MA | 21 | 5708 | Lago da Pedra | 44.272 |
| MA | 21 | 5807 | Lago do Junco | 9.888 |
| MA | 21 | 5948 | Lago dos Rodrigues | 7.940 |
| MA | 21 | 5906 | Lago Verde | 15.103 |
| MA | 21 | 5922 | Lagoa do Mato | 10.639 |
| MA | 21 | 5963 | Lagoa Grande do Maranhão | 9.363 |
| MA | 21 | 5989 | Lajeado Novo | 6.937 |
| MA | 21 | 6003 | Lima Campos | 11.794 |
| MA | 21 | 6102 | Loreto | 10.701 |
| MA | 21 | 6201 | Luís Domingues | 6.997 |
| MA | 21 | 6300 | Magalhães de Almeida | 14.808 |
| MA | 21 | 6326 | Maracaçumé | 18.414 |
| MA | 21 | 6359 | Marajá do Sena | 6.954 |
| MA | 21 | 6375 | Maranhãozinho | 12.684 |
| MA | 21 | 6409 | Mata Roma | 14.491 |
| MA | 21 | 6508 | Matinha | 21.204 |
| MA | 21 | 6607 | Matões | 29.386 |
| MA | 21 | 6631 | Matões do Norte | 11.295 |
| MA | 21 | 6672 | Milagres do Maranhão | 7.854 |
| MA | 21 | 6706 | Mirador | 19.991 |
| MA | 21 | 6755 | Miranda do Norte | 18.494 |
| MA | 21 | 6805 | Mirinzal | 14.311 |
| MA | 21 | 6904 | Monção | 28.602 |
| MA | 21 | 7001 | Montes Altos | 8.914 |
| MA | 21 | 7100 | Morros | 17.916 |
| MA | 21 | 7209 | Nina Rodrigues | 10.527 |
| MA | 21 | 7258 | Nova Colinas | 5.094 |
| MA | 21 | 7308 | Nova Iorque | 5.087 |
| MA | 21 | 7357 | Nova Olinda do Maranhão | 17.835 |
| MA | 21 | 7407 | Olho d'Água das Cunhãs | 17.923 |
| MA | 21 | 7456 | Olinda Nova do Maranhão | 12.684 |
| MA | 21 | 7506 | Paço do Lumiar | 103.958 |
| MA | 21 | 7605 | Palmeirândia | 18.772 |
| MA | 21 | 7704 | Paraibano | 20.255 |
| MA | 21 | 7803 | Parnarama | 36.300 |
| MA | 21 | 7902 | Passagem Franca | 17.898 |
| MA | 21 | 8009 | Pastos Bons | 18.306 |
| MA | 21 | 8058 | Paulino Neves | 13.355 |
| MA | 21 | 8108 | Paulo Ramos | 16.236 |
| MA | 21 | 8207 | Pedreiras | 38.934 |
| MA | 21 | 8256 | Pedro do Rosário | 22.856 |
| MA | 21 | 8306 | Penalva | 34.907 |
| MA | 21 | 8405 | Peri Mirim | 12.492 |
| MA | 21 | 8454 | Peritoró | 19.817 |
| MA | 21 | 8504 | Pindaré-Mirim | 32.236 |
| MA | 21 | 8603 | Pinheiro | 77.182 |
| MA | 21 | 8702 | Pio XII | 22.220 |
| MA | 21 | 8801 | Pirapemas | 15.500 |

| U.F. | | MUNICÍPIOS | | POPULAÇÃO ESTIMADA |
|---|---|---|---|---|
| SIGLA | COD | COD | NOMES | |
| MA | 21 | 8900 | Poção de Pedras | 15.533 |
| MA | 21 | 9007 | Porto Franco | 19.503 |
| MA | 21 | 9056 | Porto Rico do Maranhão | 7.179 |
| MA | 21 | 9106 | Presidente Dutra | 41.303 |
| MA | 21 | 9205 | Presidente Juscelino | 12.382 |
| MA | 21 | 9239 | Presidente Médici | 6.363 |
| MA | 21 | 9270 | Presidente Sarney | 16.325 |
| MA | 21 | 9304 | Presidente Vargas | 10.191 |
| MA | 21 | 9403 | Primeira Cruz | 12.493 |
| MA | 21 | 9452 | Raposa | 25.837 |
| MA | 21 | 9502 | Riachão | 21.672 |
| MA | 21 | 9551 | Ribamar Fiquene | 7.477 |
| MA | 21 | 9601 | Rosário | 39.627 |
| MA | 21 | 9700 | Sambaíba | 6.038 |
| MA | 21 | 9759 | Santa Filomena do Maranhão | 5.804 |
| MA | 21 | 9809 | Santa Helena | 35.472 |
| MA | 21 | 9908 | Santa Inês | 85.701 |
| MA | 21 | 10005 | Santa Luzia | 71.455 |
| MA | 21 | 10039 | Santa Luzia do Paruá | 20.190 |
| MA | 21 | 10104 | Santa Quitéria do Maranhão | 29.551 |
| MA | 21 | 10203 | Santa Rita | 32.872 |
| MA | 21 | 10237 | Santana do Maranhão | 11.170 |
| MA | 21 | 10278 | Santo Amaro do Maranhão | 11.693 |
| MA | 21 | 10302 | Santo Antônio dos Lopes | 14.663 |
| MA | 21 | 10401 | São Benedito do Rio Preto | 17.818 |
| MA | 21 | 10500 | São Bento | 39.312 |
| MA | 21 | 10609 | São Bernardo | 26.615 |
| MA | 21 | 10658 | São Domingos do Azeitão | 7.252 |
| MA | 21 | 10708 | São Domingos do Maranhão | 33.506 |
| MA | 21 | 10807 | São Félix de Balsas | 4.442 |
| MA | 21 | 10856 | São Francisco do Brejão | 8.863 |
| MA | 21 | 10906 | São Francisco do Maranhão | 14.801 |
| MA | 21 | 11029 | São João do Carú | 12.511 |
| MA | 21 | 11052 | São João do Paraíso | 11.729 |
| MA | 21 | 11078 | São João do Soter | 17.326 |
| MA | 21 | 11102 | São João dos Patos | 24.357 |
| MA | 21 | 11003 | São João Batista | 18.570 |
| MA | 21 | 11201 | São José de Ribamar | 139.473 |
| MA | 21 | 11250 | São José dos Basílios | 7.489 |
| MA | 21 | 11300 | São Luís | 997.098 |
| MA | 21 | 11409 | São Luís Gonzaga do Maranhão | 19.877 |
| MA | 21 | 11508 | São Mateus do Maranhão | 39.622 |
| MA | 21 | 11532 | São Pedro da Água Branca | 11.481 |
| MA | 21 | 11573 | São Pedro dos Crentes | 4.144 |
| MA | 21 | 11607 | São Raimundo das Mangabeiras | 16.594 |
| MA | 21 | 11631 | São Raimundo do Doca Bezerra | 4.372 |
| MA | 21 | 11672 | São Roberto | 5.217 |
| MA | 21 | 11706 | São Vicente Ferrer | 20.463 |
| MA | 21 | 11722 | Satubinha | 8.715 |
| MA | 21 | 11748 | Senador Alexandre Costa | 9.414 |

| U.F. | | MUNICÍPIOS | | POPULAÇÃO ESTIMADA |
|---|---|---|---|---|
| SIGLA | COD | COD | NOMES | |
| MA | 21 | 11763 | Senador La Rocque | 19.359 |
| MA | 21 | 11789 | Serrano do Maranhão | 11.085 |
| MA | 21 | 11805 | Sítio Novo | 16.086 |
| MA | 21 | 11904 | Sucupira do Norte | 10.535 |
| MA | 21 | 11953 | Sucupira do Riachão | 4.869 |
| MA | 21 | 12001 | Tasso Fragoso | 6.890 |
| MA | 21 | 12100 | Timbiras | 26.909 |
| MA | 21 | 12209 | Timon | 150.635 |
| MA | 21 | 12233 | Trizidela do Vale | 19.104 |
| MA | 21 | 12274 | Tufilândia | 5.687 |
| MA | 21 | 12308 | Tuntum | 39.380 |
| MA | 21 | 12407 | Turiaçu | 33.649 |
| MA | 21 | 12456 | Turilândia | 21.102 |
| MA | 21 | 12506 | Tutóia | 48.424 |
| MA | 21 | 12605 | Urbano Santos | 22.938 |
| MA | 21 | 12704 | Vargem Grande | 45.630 |
| MA | 21 | 12803 | Viana | 49.348 |
| MA | 21 | 12852 | Vila Nova dos Martírios | 9.185 |
| MA | 21 | 12902 | Vitória do Mearim | 32.018 |
| MA | 21 | 13009 | Vitorino Freire | 31.144 |
| MA | 21 | 14007 | Zé Doca | 46.265 |
| PI | 22 | 53 | Acauã | 7.354 |
| PI | 22 | 103 | Agricolândia | 5.094 |
| PI | 22 | 202 | Água Branca | 16.518 |
| PI | 22 | 251 | Alagoinha do Piauí | 7.736 |
| PI | 22 | 277 | Alegrete do Piauí | 4.595 |
| PI | 22 | 301 | Alto Longá | 14.147 |
| PI | 22 | 400 | Altos | 39.735 |
| PI | 22 | 459 | Alvorada do Gurguéia | 5.209 |
| PI | 22 | 509 | Amarante | 17.892 |
| PI | 22 | 608 | Angical do Piauí | 6.793 |
| PI | 22 | 707 | Anísio de Abreu | 8.552 |
| PI | 22 | 806 | Antônio Almeida | 3.267 |
| PI | 22 | 905 | Aroazes | 6.019 |
| PI | 22 | 954 | Aroeiras do Itaim | 2.759 |
| PI | 22 | 1002 | Arraial | 5.165 |
| PI | 22 | 1051 | Assunção do Piauí | 8.370 |
| PI | 22 | 1101 | Avelino Lopes | 12.039 |
| PI | 22 | 1150 | Baixa Grande do Ribeiro | 10.804 |
| PI | 22 | 1176 | Barra D'Alcântara | 3.886 |
| PI | 22 | 1200 | Barras | 44.913 |
| PI | 22 | 1309 | Barreiras do Piauí | 3.485 |
| PI | 22 | 1408 | Barro Duro | 6.853 |
| PI | 22 | 1507 | Batalha | 26.681 |
| PI | 22 | 1556 | Bela Vista do Piauí | 3.762 |
| PI | 22 | 1572 | Belém do Piauí | 2.912 |
| PI | 22 | 1606 | Beneditinos | 9.837 |
| PI | 22 | 1705 | Bertolínia | 5.484 |
| PI | 22 | 1739 | Betânia do Piauí | 6.442 |
| PI | 22 | 1770 | Boa Hora | 6.369 |

| U.F. | | MUNICÍPIOS | | POPULAÇÃO ESTIMADA |
|---|---|---|---|---|
| SIGLA | COD | COD | NOMES | |
| PI | 22 | 1804 | Bocaina | 4.146 |
| PI | 22 | 1903 | Bom Jesus | 20.511 |
| PI | 22 | 1919 | Bom Princípio do Piauí | 5.506 |
| PI | 22 | 1929 | Bonfim do Piauí | 5.376 |
| PI | 22 | 1945 | Boqueirão do Piauí | 6.599 |
| PI | 22 | 1960 | Brasileira | 7.970 |
| PI | 22 | 1988 | Brejo do Piauí | 4.025 |
| PI | 22 | 2000 | Buriti dos Lopes | 19.796 |
| PI | 22 | 2026 | Buriti dos Montes | 8.117 |
| PI | 22 | 2059 | Cabeceiras do Piauí | 9.826 |
| PI | 22 | 2075 | Cajazeiras do Piauí | 3.323 |
| PI | 22 | 2083 | Cajueiro da Praia | 7.286 |
| PI | 22 | 2091 | Caldeirão Grande do Piauí | 5.809 |
| PI | 22 | 2109 | Campinas do Piauí | 5.818 |
| PI | 22 | 2117 | Campo Alegre do Fidalgo | 4.647 |
| PI | 22 | 2133 | Campo Grande do Piauí | 5.898 |
| PI | 22 | 2174 | Campo Largo do Piauí | 7.035 |
| PI | 22 | 2208 | Campo Maior | 46.068 |
| PI | 22 | 2251 | Canavieira | 4.093 |
| PI | 22 | 2307 | Canto do Buriti | 20.761 |
| PI | 22 | 2406 | Capitão de Campos | 11.246 |
| PI | 22 | 2455 | Capitão Gervásio Oliveira | 4.029 |
| PI | 22 | 2505 | Caracol | 10.838 |
| PI | 22 | 2539 | Caraúbas do Piauí | 5.595 |
| PI | 22 | 2554 | Caridade do Piauí | 4.778 |
| PI | 22 | 2604 | Castelo do Piauí | 19.142 |
| PI | 22 | 2653 | Caxingó | 5.270 |
| PI | 22 | 2703 | Cocal | 27.220 |
| PI | 22 | 2711 | Cocal de Telha | 4.689 |
| PI | 22 | 2729 | Cocal dos Alves | 5.525 |
| PI | 22 | 2737 | Coivaras | 3.955 |
| PI | 22 | 2752 | Colônia do Gurguéia | 5.947 |
| PI | 22 | 2778 | Colônia do Piauí | 7.940 |
| PI | 22 | 2802 | Conceição do Canindé | 5.077 |
| PI | 22 | 2851 | Coronel José Dias | 4.484 |
| PI | 22 | 2901 | Corrente | 25.406 |
| PI | 22 | 3008 | Cristalândia do Piauí | 8.177 |
| PI | 22 | 3107 | Cristino Castro | 9.836 |
| PI | 22 | 3206 | Curimatá | 10.765 |
| PI | 22 | 3230 | Currais | 4.710 |
| PI | 22 | 3271 | Curral Novo do Piauí | 5.119 |
| PI | 22 | 3255 | Curralinhos | 4.242 |
| PI | 22 | 3305 | Demerval Lobão | 13.232 |
| PI | 22 | 3354 | Dirceu Arcoverde | 6.996 |
| PI | 22 | 3404 | Dom Expedito Lopes | 6.793 |
| PI | 22 | 3453 | Dom Inocêncio | 10.795 |
| PI | 22 | 3420 | Domingos Mourão | 4.447 |
| PI | 22 | 3503 | Elesbão Veloso | 14.490 |
| PI | 22 | 3602 | Eliseu Martins | 4.923 |
| PI | 22 | 3701 | Esperantina | 37.520 |

| U.F. | | MUNICÍPIOS | | POPULAÇÃO ESTIMADA |
|---|---|---|---|---|
| SIGLA | COD | COD | NOMES | |
| PI | 22 | 3750 | Fartura do Piauí | 5.402 |
| PI | 22 | 3800 | Flores do Piauí | 4.615 |
| PI | 22 | 3859 | Floresta do Piauí | 2.643 |
| PI | 22 | 3909 | Floriano | 57.968 |
| PI | 22 | 4006 | Francinópolis | 5.458 |
| PI | 22 | 4105 | Francisco Ayres | 5.147 |
| PI | 22 | 4154 | Francisco Macedo | 2.315 |
| PI | 22 | 4204 | Francisco Santos | 8.315 |
| PI | 22 | 4303 | Fronteiras | 11.502 |
| PI | 22 | 4352 | Geminiano | 5.465 |
| PI | 22 | 4402 | Gilbués | 10.681 |
| PI | 22 | 4501 | Guadalupe | 9.809 |
| PI | 22 | 4550 | Guaribas | 4.491 |
| PI | 22 | 4600 | Hugo Napoleão | 3.784 |
| PI | 22 | 4659 | Ilha Grande | 8.734 |
| PI | 22 | 4709 | Inhuma | 15.490 |
| PI | 22 | 4808 | Ipiranga do Piauí | 9.739 |
| PI | 22 | 4907 | Isaías Coelho | 8.043 |
| PI | 22 | 5003 | Itainópolis | 11.498 |
| PI | 22 | 5102 | Itaueira | 10.904 |
| PI | 22 | 5151 | Jacobina do Piauí | 5.831 |
| PI | 22 | 5201 | Jaicós | 17.786 |
| PI | 22 | 5250 | Jardim do Mulato | 4.318 |
| PI | 22 | 5276 | Jatobá do Piauí | 4.683 |
| PI | 22 | 5300 | Jerumenha | 4.490 |
| PI | 22 | 5359 | João Costa | 3.315 |
| PI | 22 | 5409 | Joaquim Pires | 14.276 |
| PI | 22 | 5458 | Joca Marques | 5.614 |
| PI | 22 | 5508 | José de Freitas | 36.485 |
| PI | 22 | 5516 | Juazeiro do Piauí | 4.941 |
| PI | 22 | 5524 | Júlio Borges | 5.444 |
| PI | 22 | 5532 | Jurema | 4.559 |
| PI | 22 | 5573 | Lagoa de São Francisco | 6.796 |
| PI | 22 | 5565 | Lagoa do Barro do Piauí | 4.692 |
| PI | 22 | 5581 | Lagoa do Piauí | 3.817 |
| PI | 22 | 5599 | Lagoa do Sítio | 5.292 |
| PI | 22 | 5557 | Lagoa Alegre | 8.210 |
| PI | 22 | 5540 | Lagoinha do Piauí | 2.676 |
| PI | 22 | 5607 | Landri Sales | 5.753 |
| PI | 22 | 5706 | Luís Correia | 27.148 |
| PI | 22 | 5805 | Luzilândia | 25.099 |
| PI | 22 | 5854 | Madeiro | 8.012 |
| PI | 22 | 5904 | Manoel Emídio | 5.543 |
| PI | 22 | 5953 | Marcolândia | 7.477 |
| PI | 22 | 6001 | Marcos Parente | 4.297 |
| PI | 22 | 6050 | Massapê do Piauí | 6.672 |
| PI | 22 | 6100 | Matias Olímpio | 10.863 |
| PI | 22 | 6209 | Miguel Alves | 33.410 |
| PI | 22 | 6308 | Miguel Leão | 1.212 |
| PI | 22 | 6357 | Milton Brandão | 7.246 |

| U.F. | | MUNICÍPIOS | | POPULAÇÃO ESTIMADA |
|---|---|---|---|---|
| SIGLA | COD | COD | NOMES | |
| PI | 22 | 6407 | Monsenhor Gil | 10.639 |
| PI | 22 | 6506 | Monsenhor Hipólito | 7.425 |
| PI | 22 | 6605 | Monte Alegre do Piauí | 10.663 |
| PI | 22 | 6670 | Morro do Chapéu do Piauí | 6.611 |
| PI | 22 | 6654 | Morro Cabeça no Tempo | 4.378 |
| PI | 22 | 6696 | Murici dos Portelas | 8.024 |
| PI | 22 | 6704 | Nazaré do Piauí | 7.070 |
| PI | 22 | 6720 | Nazária | 7.895 |
| PI | 22 | 6753 | Nossa Senhora de Nazaré | 4.192 |
| PI | 22 | 6803 | Nossa Senhora dos Remédios | 8.378 |
| PI | 22 | 7959 | Nova Santa Rita | 4.199 |
| PI | 22 | 6902 | Novo Oriente do Piauí | 6.321 |
| PI | 22 | 6951 | Novo Santo Antônio | 3.547 |
| PI | 22 | 7009 | Oeiras | 36.293 |
| PI | 22 | 7108 | Olho D'Água do Piauí | 2.755 |
| PI | 22 | 7207 | Padre Marcos | 7.582 |
| PI | 22 | 7306 | Paes Landim | 4.562 |
| PI | 22 | 7355 | Pajeú do Piauí | 3.802 |
| PI | 22 | 7405 | Palmeira do Piauí | 5.116 |
| PI | 22 | 7504 | Palmeirais | 14.306 |
| PI | 22 | 7553 | Paquetá | 4.686 |
| PI | 22 | 7603 | Parnaguá | 10.731 |
| PI | 22 | 7702 | Parnaíba | 146.059 |
| PI | 22 | 7751 | Passagem Franca do Piauí | 4.246 |
| PI | 22 | 7777 | Patos do Piauí | 6.417 |
| PI | 22 | 7793 | Pau D'Arco do Piauí | 3.903 |
| PI | 22 | 7801 | Paulistana | 20.390 |
| PI | 22 | 7850 | Pavussu | 4.465 |
| PI | 22 | 7900 | Pedro II | 37.850 |
| PI | 22 | 7934 | Pedro Laurentino | 2.418 |
| PI | 22 | 8007 | Picos | 73.021 |
| PI | 22 | 8106 | Pimenteiras | 12.125 |
| PI | 22 | 8205 | Pio IX | 17.714 |
| PI | 22 | 8304 | Piracuruca | 26.499 |
| PI | 22 | 8403 | Piripiri | 62.107 |
| PI | 22 | 8502 | Porto | 11.941 |
| PI | 22 | 8551 | Porto Alegre do Piauí | 2.567 |
| PI | 22 | 8601 | Prata do Piauí | 3.260 |
| PI | 22 | 8650 | Queimada Nova | 9.116 |
| PI | 22 | 8700 | Redenção do Gurguéia | 8.567 |
| PI | 22 | 8809 | Regeneração | 18.190 |
| PI | 22 | 8858 | Riacho Frio | 5.093 |
| PI | 22 | 8874 | Ribeira do Piauí | 4.252 |
| PI | 22 | 8908 | Ribeiro Gonçalves | 6.869 |
| PI | 22 | 9005 | Rio Grande do Piauí | 6.650 |
| PI | 22 | 9104 | Santa Cruz do Piauí | 5.969 |
| PI | 22 | 9153 | Santa Cruz dos Milagres | 3.457 |
| PI | 22 | 9203 | Santa Filomena | 6.180 |
| PI | 22 | 9302 | Santa Luz | 5.495 |
| PI | 22 | 9377 | Santa Rosa do Piauí | 5.304 |

| U.F. | | MUNICÍPIOS | | POPULAÇÃO ESTIMADA |
|---|---|---|---|---|
| SIGLA | COD | COD | NOMES | |
| PI | 22 | 9351 | Santana do Piauí | 5.015 |
| PI | 22 | 9401 | Santo Antônio de Lisboa | 5.965 |
| PI | 22 | 9450 | Santo Antônio dos Milagres | 2.032 |
| PI | 22 | 9500 | Santo Inácio do Piauí | 3.756 |
| PI | 22 | 9559 | São Braz do Piauí | 4.455 |
| PI | 22 | 9609 | São Félix do Piauí | 3.201 |
| PI | 22 | 9658 | São Francisco de Assis do Piauí | 5.224 |
| PI | 22 | 9708 | São Francisco do Piauí | 6.461 |
| PI | 22 | 9757 | São Gonçalo do Gurguéia | 2.544 |
| PI | 22 | 9807 | São Gonçalo do Piauí | 4.485 |
| PI | 22 | 9856 | São João da Canabrava | 4.511 |
| PI | 22 | 9872 | São João da Fronteira | 5.174 |
| PI | 22 | 9906 | São João da Serra | 6.877 |
| PI | 22 | 9955 | São João da Varjota | 4.965 |
| PI | 22 | 9971 | São João do Arraial | 7.440 |
| PI | 22 | 10003 | São João do Piauí | 19.264 |
| PI | 22 | 10052 | São José do Divino | 5.217 |
| PI | 22 | 10102 | São José do Peixe | 3.835 |
| PI | 22 | 10201 | São José do Piauí | 7.028 |
| PI | 22 | 10300 | São Julião | 6.111 |
| PI | 22 | 10359 | São Lourenço do Piauí | 5.115 |
| PI | 22 | 10375 | São Luis do Piauí | 2.687 |
| PI | 22 | 10383 | São Miguel da Baixa Grande | 2.153 |
| PI | 22 | 10391 | São Miguel do Fidalgo | 3.182 |
| PI | 22 | 10409 | São Miguel do Tapuio | 19.831 |
| PI | 22 | 10508 | São Pedro do Piauí | 13.544 |
| PI | 22 | 10607 | São Raimundo Nonato | 32.215 |
| PI | 22 | 10623 | Sebastião Barros | 4.270 |
| PI | 22 | 10631 | Sebastião Leal | 4.231 |
| PI | 22 | 10656 | Sigefredo Pacheco | 9.846 |
| PI | 22 | 10706 | Simões | 14.167 |
| PI | 22 | 10805 | Simplício Mendes | 11.886 |
| PI | 22 | 10904 | Socorro do Piauí | 4.733 |
| PI | 22 | 10938 | Sussuapara | 5.759 |
| PI | 22 | 10953 | Tamboril do Piauí | 3.037 |
| PI | 22 | 10979 | Tanque do Piauí | 2.714 |
| PI | 22 | 11001 | Teresina | 802.537 |
| PI | 22 | 11100 | União | 43.135 |
| PI | 22 | 11209 | Uruçuí | 19.811 |
| PI | 22 | 11308 | Valença do Piauí | 20.303 |
| PI | 22 | 11357 | Várzea Branca | 5.338 |
| PI | 22 | 11407 | Várzea Grande | 4.560 |
| PI | 22 | 11506 | Vera Mendes | 3.212 |
| PI | 22 | 11605 | Vila Nova do Piauí | 3.136 |
| PI | 22 | 11704 | Wall Ferraz | 4.636 |
| CE | 23 | 101 | Abaiara | 11.077 |
| CE | 23 | 150 | Acarape | 15.627 |
| CE | 23 | 200 | Acaraú | 54.753 |
| CE | 23 | 309 | Acopiara | 50.784 |
| CE | 23 | 408 | Aiuaba | 16.423 |

| U.F. | | MUNICÍPIOS | | POPULAÇÃO ESTIMADA |
|---|---|---|---|---|
| SIGLA | COD | COD | NOMES | |
| CE | 23 | 507 | Alcântaras | 10.816 |
| CE | 23 | 606 | Altaneira | 6.834 |
| CE | 23 | 705 | Alto Santo | 20.829 |
| CE | 23 | 754 | Amontada | 40.198 |
| CE | 23 | 804 | Antonina do Norte | 7.059 |
| CE | 23 | 903 | Apuiarés | 14.414 |
| CE | 23 | 1000 | Aquiraz | 71.400 |
| CE | 23 | 1109 | Aracati | 69.616 |
| CE | 23 | 1208 | Aracoiaba | 25.351 |
| CE | 23 | 1257 | Ararendá | 11.186 |
| CE | 23 | 1307 | Araripe | 22.373 |
| CE | 23 | 1406 | Aratuba | 12.478 |
| CE | 23 | 1505 | Arneiroz | 7.486 |
| CE | 23 | 1604 | Assaré | 22.550 |
| CE | 23 | 1703 | Aurora | 25.113 |
| CE | 23 | 1802 | Baixio | 5.991 |
| CE | 23 | 1851 | Banabuiú | 18.388 |
| CE | 23 | 1901 | Barbalha | 53.011 |
| CE | 23 | 1950 | Barreira | 19.469 |
| CE | 23 | 2008 | Barro | 21.556 |
| CE | 23 | 2057 | Barroquinha | 15.558 |
| CE | 23 | 2107 | Baturité | 33.271 |
| CE | 23 | 2206 | Beberibe | 48.760 |
| CE | 23 | 2305 | Bela Cruz | 30.900 |
| CE | 23 | 2404 | Boa Viagem | 56.236 |
| CE | 23 | 2503 | Brejo Santo | 41.266 |
| CE | 23 | 2602 | Camocim | 61.278 |
| CE | 23 | 2701 | Campos Sales | 26.417 |
| CE | 23 | 2800 | Canindé | 77.552 |
| CE | 23 | 2909 | Capistrano | 17.033 |
| CE | 23 | 3006 | Caridade | 19.233 |
| CE | 23 | 3105 | Cariré | 19.132 |
| CE | 23 | 3204 | Caririaçu | 27.380 |
| CE | 23 | 3303 | Cariús | 19.338 |
| CE | 23 | 3402 | Carnaubal | 16.755 |
| CE | 23 | 3501 | Cascavel | 67.956 |
| CE | 23 | 3600 | Catarina | 18.010 |
| CE | 23 | 3659 | Catunda | 11.198 |
| CE | 23 | 3709 | Caucaia | 334.364 |
| CE | 23 | 3808 | Cedro | 25.591 |
| CE | 23 | 3907 | Chaval | 12.644 |
| CE | 23 | 3931 | Choró | 13.439 |
| CE | 23 | 3956 | Chorozinho | 18.759 |
| CE | 23 | 4004 | Coreaú | 22.215 |
| CE | 23 | 4103 | Crateús | 75.249 |
| CE | 23 | 4202 | Crato | 116.759 |
| CE | 23 | 4236 | Croatá | 18.246 |
| CE | 23 | 4251 | Cruz | 23.540 |
| CE | 23 | 4269 | Deputado Irapuan Pinheiro | 9.615 |
| CE | 23 | 4277 | Ererê | 7.332 |

| U.F. | | MUNICÍPIOS | | POPULAÇÃO ESTIMADA |
|---|---|---|---|---|
| SIGLA | COD | COD | NOMES | |
| CE | 23 | 4285 | Eusébio | 41.307 |
| CE | 23 | 4301 | Farias Brito | 19.605 |
| CE | 23 | 4350 | Forquilha | 21.599 |
| CE | 23 | 4400 | Fortaleza | 2.505.552 |
| CE | 23 | 4459 | Fortim | 15.095 |
| CE | 23 | 4509 | Frecheirinha | 13.537 |
| CE | 23 | 4608 | General Sampaio | 6.654 |
| CE | 23 | 4657 | Graça | 15.949 |
| CE | 23 | 4707 | Granja | 53.952 |
| CE | 23 | 4806 | Granjeiro | 4.999 |
| CE | 23 | 4905 | Groaíras | 9.971 |
| CE | 23 | 4954 | Guaiúba | 23.853 |
| CE | 23 | 5001 | Guaraciaba do Norte | 38.406 |
| CE | 23 | 5100 | Guaramiranga | 4.070 |
| CE | 23 | 5209 | Hidrolândia | 19.252 |
| CE | 23 | 5233 | Horizonte | 54.362 |
| CE | 23 | 5266 | Ibaretama | 13.206 |
| CE | 23 | 5308 | Ibiapina | 24.125 |
| CE | 23 | 5332 | Ibicuitinga | 11.800 |
| CE | 23 | 5357 | Icapuí | 19.385 |
| CE | 23 | 5407 | Icó | 65.612 |
| CE | 23 | 5506 | Iguatu | 97.203 |
| CE | 23 | 5605 | Independência | 26.317 |
| CE | 23 | 5654 | Ipaporanga | 11.768 |
| CE | 23 | 5704 | Ipaumirim | 11.999 |
| CE | 23 | 5803 | Ipu | 41.052 |
| CE | 23 | 5902 | Ipueiras | 39.288 |
| CE | 23 | 6009 | Iracema | 15.114 |
| CE | 23 | 6108 | Irauçuba | 23.309 |
| CE | 23 | 6207 | Itaiçaba | 7.955 |
| CE | 23 | 6256 | Itaitinga | 32.678 |
| CE | 23 | 6306 | Itapagé | 47.354 |
| CE | 23 | 6405 | Itapipoca | 114.441 |
| CE | 23 | 6504 | Itapiúna | 18.610 |
| CE | 23 | 6553 | Itarema | 36.536 |
| CE | 23 | 6603 | Itatira | 18.875 |
| CE | 23 | 6702 | Jaguaretama | 18.411 |
| CE | 23 | 6801 | Jaguaribara | 10.399 |
| CE | 23 | 6900 | Jaguaribe | 36.493 |
| CE | 23 | 7007 | Jaguaruana | 32.352 |
| CE | 23 | 7106 | Jardim | 26.578 |
| CE | 23 | 7205 | Jati | 7.518 |
| CE | 23 | 7254 | Jijoca de Jericoacoara | 16.880 |
| CE | 23 | 7304 | Juazeiro do Norte | 249.829 |
| CE | 23 | 7403 | Jucás | 23.738 |
| CE | 23 | 7502 | Lavras da Mangabeira | 30.524 |
| CE | 23 | 7601 | Limoeiro do Norte | 56.098 |
| CE | 23 | 7635 | Madalena | 17.773 |
| CE | 23 | 7650 | Maracanaú | 201.693 |
| CE | 23 | 7700 | Maranguape | 110.523 |

| U.F. | | MUNICÍPIOS | | POPULAÇÃO ESTIMADA |
|---|---|---|---|---|
| SIGLA | COD | COD | NOMES | |
| CE | 23 | 7809 | Marco | 24.622 |
| CE | 23 | 7908 | Martinópole | 11.118 |
| CE | 23 | 8005 | Massapê | 35.388 |
| CE | 23 | 8104 | Mauriti | 44.211 |
| CE | 23 | 8203 | Meruoca | 12.780 |
| CE | 23 | 8302 | Milagres | 28.077 |
| CE | 23 | 8351 | Milhã | 14.826 |
| CE | 23 | 8377 | Miraíma | 12.737 |
| CE | 23 | 8401 | Missão Velha | 35.135 |
| CE | 23 | 8500 | Mombaça | 46.728 |
| CE | 23 | 8609 | Monsenhor Tabosa | 17.178 |
| CE | 23 | 8708 | Morada Nova | 63.126 |
| CE | 23 | 8807 | Moraújo | 8.538 |
| CE | 23 | 8906 | Morrinhos | 22.695 |
| CE | 23 | 9003 | Mucambo | 14.537 |
| CE | 23 | 9102 | Mulungu | 11.914 |
| CE | 23 | 9201 | Nova Olinda | 13.659 |
| CE | 23 | 9300 | Nova Russas | 32.016 |
| CE | 23 | 9409 | Novo Oriente | 28.703 |
| CE | 23 | 9458 | Ocara | 24.636 |
| CE | 23 | 9508 | Orós | 21.784 |
| CE | 23 | 9607 | Pacajus | 59.689 |
| CE | 23 | 9706 | Pacatuba | 71.839 |
| CE | 23 | 9805 | Pacoti | 11.519 |
| CE | 23 | 9904 | Pacujá | 6.233 |
| CE | 23 | 10001 | Palhano | 9.268 |
| CE | 23 | 10100 | Palmácia | 10.838 |
| CE | 23 | 10209 | Paracuru | 32.557 |
| CE | 23 | 10258 | Paraipaba | 29.892 |
| CE | 23 | 10308 | Parambu | 32.231 |
| CE | 23 | 10407 | Paramoti | 12.130 |
| CE | 23 | 10506 | Pedra Branca | 42.152 |
| CE | 23 | 10605 | Penaforte | 8.168 |
| CE | 23 | 10704 | Pentecoste | 35.166 |
| CE | 23 | 10803 | Pereiro | 15.828 |
| CE | 23 | 10852 | Pindoretama | 18.322 |
| CE | 23 | 10902 | Piquet Carneiro | 15.673 |
| CE | 23 | 10951 | Pires Ferreira | 9.857 |
| CE | 23 | 11009 | Poranga | 12.356 |
| CE | 23 | 11108 | Porteiras | 15.058 |
| CE | 23 | 11207 | Potengi | 10.144 |
| CE | 23 | 11231 | Potiretama | 6.891 |
| CE | 23 | 11264 | Quiterianópolis | 21.230 |
| CE | 23 | 11306 | Quixadá | 80.447 |
| CE | 23 | 11355 | Quixelô | 16.272 |
| CE | 23 | 11405 | Quixeramobim | 73.517 |
| CE | 23 | 11504 | Quixeré | 19.772 |
| CE | 23 | 11603 | Redenção | 26.768 |
| CE | 23 | 11702 | Reriutaba | 19.444 |
| CE | 23 | 11801 | Russas | 67.960 |

| U.F. | | MUNICÍPIOS | | POPULAÇÃO ESTIMADA |
|---|---|---|---|---|
| SIGLA | COD | COD | NOMES | |
| CE | 23 | 11900 | Saboeiro | 16.851 |
| CE | 23 | 11959 | Salitre | 16.845 |
| CE | 23 | 12205 | Santa Quitéria | 45.080 |
| CE | 23 | 12007 | Santana do Acaraú | 30.410 |
| CE | 23 | 12106 | Santana do Cariri | 18.369 |
| CE | 23 | 12304 | São Benedito | 45.407 |
| CE | 23 | 12403 | São Gonçalo do Amarante | 42.962 |
| CE | 23 | 12502 | São João do Jaguaribe | 8.500 |
| CE | 23 | 12601 | São Luís do Curu | 12.613 |
| CE | 23 | 12700 | Senador Pompeu | 25.263 |
| CE | 23 | 12809 | Senador Sá | 6.669 |
| CE | 23 | 12908 | Sobral | 182.431 |
| CE | 23 | 13005 | Solonópole | 18.025 |
| CE | 23 | 13104 | Tabuleiro do Norte | 29.576 |
| CE | 23 | 13203 | Tamboril | 26.857 |
| CE | 23 | 13252 | Tarrafas | 8.900 |
| CE | 23 | 13302 | Tauá | 56.639 |
| CE | 23 | 13351 | Tejuçuoca | 16.180 |
| CE | 23 | 13401 | Tianguá | 68.588 |
| CE | 23 | 13500 | Trairi | 51.386 |
| CE | 23 | 13559 | Tururu | 14.308 |
| CE | 23 | 13609 | Ubajara | 31.247 |
| CE | 23 | 13708 | Umari | 7.891 |
| CE | 23 | 13757 | Umirim | 19.044 |
| CE | 23 | 13807 | Uruburetama | 20.627 |
| CE | 23 | 13906 | Uruoca | 13.820 |
| CE | 23 | 13955 | Varjota | 17.802 |
| CE | 23 | 14003 | Várzea Alegre | 39.810 |
| CE | 23 | 14102 | Viçosa do Ceará | 56.673 |
| RN | 24 | 109 | Acari | 11.215 |
| RN | 24 | 208 | Açu | 53.282 |
| RN | 24 | 307 | Afonso Bezerra | 10.594 |
| RN | 24 | 406 | Água Nova | 2.952 |
| RN | 24 | 505 | Alexandria | 14.151 |
| RN | 24 | 604 | Almino Afonso | 5.071 |
| RN | 24 | 703 | Alto do Rodrigues | 12.045 |
| RN | 24 | 802 | Angicos | 11.525 |
| RN | 24 | 901 | Antônio Martins | 7.245 |
| RN | 24 | 1008 | Apodi | 35.768 |
| RN | 24 | 1107 | Areia Branca | 25.394 |
| RN | 24 | 1206 | Arês | 12.732 |
| RN | 24 | 1305 | Augusto Severo | 9.203 |
| RN | 24 | 1404 | Baía Formosa | 8.811 |
| RN | 24 | 1453 | Baraúna | 24.347 |
| RN | 24 | 1503 | Barcelona | 4.042 |
| RN | 24 | 1602 | Bento Fernandes | 5.199 |
| RN | 24 | 1651 | Bodó | 2.592 |
| RN | 24 | 1701 | Bom Jesus | 8.725 |
| RN | 24 | 1800 | Brejinho | 11.585 |
| RN | 24 | 1859 | Caiçara do Norte | 6.652 |

| U.F. | | MUNICÍPIOS | | POPULAÇÃO ESTIMADA |
|---|---|---|---|---|
| SIGLA | COD | COD | NOMES | |
| RN | 24 | 1909 | Caiçara do Rio do Vento | 3.185 |
| RN | 24 | 2006 | Caicó | 63.006 |
| RN | 24 | 2105 | Campo Redondo | 10.947 |
| RN | 24 | 2204 | Canguaretama | 30.541 |
| RN | 24 | 2303 | Caraúbas | 20.471 |
| RN | 24 | 2402 | Carnaúba dos Dantas | 7.083 |
| RN | 24 | 2501 | Carnaubais | 9.712 |
| RN | 24 | 2600 | Ceará-Mirim | 67.869 |
| RN | 24 | 2709 | Cerro Corá | 11.235 |
| RN | 24 | 2808 | Coronel Ezequiel | 5.399 |
| RN | 24 | 2907 | Coronel João Pessoa | 4.993 |
| RN | 24 | 3004 | Cruzeta | 8.029 |
| RN | 24 | 3103 | Currais Novos | 43.536 |
| RN | 24 | 3202 | Doutor Severiano | 6.616 |
| RN | 24 | 3301 | Encanto | 5.365 |
| RN | 24 | 3400 | Equador | 6.084 |
| RN | 24 | 3509 | Espírito Santo | 10.373 |
| RN | 24 | 3608 | Extremoz | 22.751 |
| RN | 24 | 3707 | Felipe Guerra | 5.875 |
| RN | 24 | 3756 | Fernando Pedroza | 2.994 |
| RN | 24 | 3806 | Florânia | 8.487 |
| RN | 24 | 3905 | Francisco Dantas | 3.008 |
| RN | 24 | 4002 | Frutuoso Gomes | 4.468 |
| RN | 24 | 4101 | Galinhos | 2.264 |
| RN | 24 | 4200 | Goianinha | 21.321 |
| RN | 24 | 4309 | Governador Dix-Sept Rosado | 12.835 |
| RN | 24 | 4408 | Grossos | 9.886 |
| RN | 24 | 4507 | Guamaré | 12.558 |
| RN | 24 | 4606 | Ielmo Marinho | 12.189 |
| RN | 24 | 4705 | Ipanguaçu | 14.056 |
| RN | 24 | 4804 | Ipueira | 2.115 |
| RN | 24 | 4853 | Itajá | 6.629 |
| RN | 24 | 4903 | Itaú | 5.999 |
| RN | 24 | 5009 | Jaçanã | 8.045 |
| RN | 24 | 5108 | Jandaíra | 6.688 |
| RN | 24 | 5207 | Janduís | 5.562 |
| RN | 24 | 5306 | Januário Cicco | 8.629 |
| RN | 24 | 5405 | Japi | 5.693 |
| RN | 24 | 5504 | Jardim de Angicos | 2.598 |
| RN | 24 | 5603 | Jardim de Piranhas | 14.347 |
| RN | 24 | 5702 | Jardim do Seridó | 12.384 |
| RN | 24 | 5801 | João Câmara | 31.518 |
| RN | 24 | 5900 | João Dias | 2.824 |
| RN | 24 | 6007 | José da Penha | 6.177 |
| RN | 24 | 6106 | Jucurutu | 18.069 |
| RN | 24 | 6155 | Jundiá | 3.663 |
| RN | 24 | 6205 | Lagoa d'Anta | 6.164 |
| RN | 24 | 6304 | Lagoa de Pedras | 7.282 |
| RN | 24 | 6403 | Lagoa de Velhos | 2.789 |
| RN | 24 | 6502 | Lagoa Nova | 13.718 |

| U.F. | | MUNICÍPIOS | | POPULAÇÃO ESTIMADA |
|---|---|---|---|---|
| SIGLA | COD | COD | NOMES | |
| RN | 24 | 6601 | Lagoa Salgada | 7.449 |
| RN | 24 | 6700 | Lajes | 10.865 |
| RN | 24 | 6809 | Lajes Pintadas | 4.309 |
| RN | 24 | 6908 | Lucrécia | 3.550 |
| RN | 24 | 7005 | Luís Gomes | 10.144 |
| RN | 24 | 7104 | Macaíba | 66.380 |
| RN | 24 | 7203 | Macau | 28.157 |
| RN | 24 | 7252 | Major Sales | 3.631 |
| RN | 24 | 7302 | Marcelino Vieira | 8.331 |
| RN | 24 | 7401 | Martins | 8.386 |
| RN | 24 | 7500 | Maxaranguape | 9.371 |
| RN | 24 | 7609 | Messias Targino | 3.923 |
| RN | 24 | 7708 | Montanhas | 12.834 |
| RN | 24 | 7906 | Monte das Gameleiras | 2.449 |
| RN | 24 | 7807 | Monte Alegre | 21.448 |
| RN | 24 | 8003 | Mossoró | 244.287 |
| RN | 24 | 8102 | Natal | 806.203 |
| RN | 24 | 8201 | Nísia Floresta | 24.109 |
| RN | 24 | 8300 | Nova Cruz | 36.561 |
| RN | 24 | 8409 | Olho-d'Água do Borges | 4.578 |
| RN | 24 | 8508 | Ouro Branco | 5.166 |
| RN | 24 | 8607 | Paraná | 4.039 |
| RN | 24 | 8706 | Paraú | 3.974 |
| RN | 24 | 8805 | Parazinho | 4.977 |
| RN | 24 | 8904 | Parelhas | 20.676 |
| RN | 24 | 3251 | Parnamirim | 184.222 |
| RN | 24 | 9100 | Passa e Fica | 10.954 |
| RN | 24 | 9209 | Passagem | 2.703 |
| RN | 24 | 9308 | Patu | 11.671 |
| RN | 24 | 9407 | Pau dos Ferros | 27.809 |
| RN | 24 | 9506 | Pedra Grande | 4.027 |
| RN | 24 | 9605 | Pedra Preta | 2.718 |
| RN | 24 | 9704 | Pedro Avelino | 7.559 |
| RN | 24 | 9803 | Pedro Velho | 14.118 |
| RN | 24 | 9902 | Pendências | 13.034 |
| RN | 24 | 10009 | Pilões | 3.535 |
| RN | 24 | 10108 | Poço Branco | 12.673 |
| RN | 24 | 10207 | Portalegre | 7.082 |
| RN | 24 | 10256 | Porto do Mangue | 5.033 |
| RN | 24 | 10306 | Presidente Juscelino | 8.703 |
| RN | 24 | 10405 | Pureza | 8.415 |
| RN | 24 | 10504 | Rafael Fernandes | 4.797 |
| RN | 24 | 10603 | Rafael Godeiro | 3.251 |
| RN | 24 | 10702 | Riacho da Cruz | 3.165 |
| RN | 24 | 10801 | Riacho de Santana | 4.437 |
| RN | 24 | 10900 | Riachuelo | 7.171 |
| RN | 24 | 8953 | Rio do Fogo | 10.124 |
| RN | 24 | 11007 | Rodolfo Fernandes | 4.724 |
| RN | 24 | 11106 | Ruy Barbosa | 3.730 |
| RN | 24 | 11205 | Santa Cruz | 35.095 |

| U.F. | | MUNICÍPIOS | | POPULAÇÃO ESTIMADA |
|---|---|---|---|---|
| SIGLA | COD | COD | NOMES | |
| RN | 24 | 9332 | Santa Maria | 4.915 |
| RN | 24 | 11403 | Santana do Matos | 14.544 |
| RN | 24 | 11429 | Santana do Seridó | 2.858 |
| RN | 24 | 11502 | Santo Antônio | 22.071 |
| RN | 24 | 11601 | São Bento do Norte | 3.658 |
| RN | 24 | 11700 | São Bento do Trairí | 3.875 |
| RN | 24 | 11809 | São Fernando | 3.505 |
| RN | 24 | 11908 | São Francisco do Oeste | 3.807 |
| RN | 24 | 12005 | São Gonçalo do Amarante | 80.737 |
| RN | 24 | 12104 | São João do Sabugi | 5.953 |
| RN | 24 | 12203 | São José de Mipibu | 38.404 |
| RN | 24 | 12302 | São José do Campestre | 12.079 |
| RN | 24 | 12401 | São José do Seridó | 4.066 |
| RN | 24 | 12500 | São Miguel | 23.593 |
| RN | 24 | 12559 | São Miguel do Gostoso | 9.240 |
| RN | 24 | 12609 | São Paulo do Potengi | 15.017 |
| RN | 24 | 12708 | São Pedro | 6.590 |
| RN | 24 | 12807 | São Rafael | 8.357 |
| RN | 24 | 12906 | São Tomé | 11.501 |
| RN | 24 | 13003 | São Vicente | 6.023 |
| RN | 24 | 13102 | Senador Elói de Souza | 6.201 |
| RN | 24 | 13201 | Senador Georgino Avelino | 3.854 |
| RN | 24 | 13300 | Serra de São Bento | 5.973 |
| RN | 24 | 13359 | Serra do Mel | 9.627 |
| RN | 24 | 13409 | Serra Negra do Norte | 7.428 |
| RN | 24 | 13508 | Serrinha | 6.885 |
| RN | 24 | 13557 | Serrinha dos Pintos | 4.504 |
| RN | 24 | 13607 | Severiano Melo | 5.224 |
| RN | 24 | 13706 | Sítio Novo | 5.471 |
| RN | 24 | 13805 | Taboleiro Grande | 2.380 |
| RN | 24 | 13904 | Taipu | 12.165 |
| RN | 24 | 14001 | Tangará | 13.610 |
| RN | 24 | 14100 | Tenente Ananias | 9.655 |
| RN | 24 | 14159 | Tenente Laurentino Cruz | 5.369 |
| RN | 24 | 11056 | Tibau | 3.937 |
| RN | 24 | 14209 | Tibau do Sul | 11.707 |
| RN | 24 | 14308 | Timbaúba dos Batistas | 2.380 |
| RN | 24 | 14407 | Touros | 30.549 |
| RN | 24 | 14456 | Triunfo Potiguar | 3.327 |
| RN | 24 | 14506 | Umarizal | 10.913 |
| RN | 24 | 14605 | Upanema | 13.334 |
| RN | 24 | 14704 | Várzea | 5.475 |
| RN | 24 | 14753 | Venha-Ver | 3.612 |
| RN | 24 | 14803 | Vera Cruz | 10.861 |
| RN | 24 | 14902 | Viçosa | 1.856 |
| RN | 24 | 15008 | Vila Flor | 2.745 |
| PB | 25 | 106 | Água Branca | 9.581 |
| PB | 25 | 205 | Aguiar | 5.799 |
| PB | 25 | 304 | Alagoa Grande | 28.126 |
| PB | 25 | 403 | Alagoa Nova | 19.799 |

| U.F. | | MUNICÍPIOS | | POPULAÇÃO ESTIMADA |
|---|---|---|---|---|
| SIGLA | COD | COD | NOMES | |
| PB | 25 | 502 | Alagoinha | 13.470 |
| PB | 25 | 536 | Alcantil | 5.232 |
| PB | 25 | 577 | Algodão de Jandaíra | 2.425 |
| PB | 25 | 601 | Alhandra | 18.941 |
| PB | 25 | 734 | Amparo | 2.078 |
| PB | 25 | 775 | Aparecida | 7.607 |
| PB | 25 | 809 | Araçagi | 17.839 |
| PB | 25 | 908 | Arara | 12.804 |
| PB | 25 | 1005 | Araruna | 20.009 |
| PB | 25 | 1104 | Areia | 25.648 |
| PB | 25 | 1153 | Areia de Baraúnas | 2.159 |
| PB | 25 | 1203 | Areial | 6.441 |
| PB | 25 | 1302 | Aroeiras | 19.725 |
| PB | 25 | 1351 | Assunção | 3.471 |
| PB | 25 | 1401 | Baía da Traição | 7.966 |
| PB | 25 | 1500 | Bananeiras | 22.316 |
| PB | 25 | 1534 | Baraúna | 4.045 |
| PB | 25 | 1609 | Barra de Santa Rosa | 13.273 |
| PB | 25 | 1575 | Barra de Santana | 8.909 |
| PB | 25 | 1708 | Barra de São Miguel | 5.624 |
| PB | 25 | 1807 | Bayeux | 96.198 |
| PB | 25 | 1906 | Belém | 17.746 |
| PB | 25 | 2003 | Belém do Brejo do Cruz | 7.256 |
| PB | 25 | 2052 | Bernardino Batista | 3.315 |
| PB | 25 | 2102 | Boa Ventura | 5.905 |
| PB | 25 | 2151 | Boa Vista | 5.908 |
| PB | 25 | 2201 | Bom Jesus | 2.305 |
| PB | 25 | 2300 | Bom Sucesso | 5.296 |
| PB | 25 | 2409 | Bonito de Santa Fé | 10.657 |
| PB | 25 | 2508 | Boqueirão | 16.360 |
| PB | 25 | 2706 | Borborema | 5.186 |
| PB | 25 | 2805 | Brejo do Cruz | 12.852 |
| PB | 25 | 2904 | Brejo dos Santos | 5.899 |
| PB | 25 | 3001 | Caaporã | 20.064 |
| PB | 25 | 3100 | Cabaceiras | 5.112 |
| PB | 25 | 3209 | Cabedelo | 51.865 |
| PB | 25 | 3308 | Cachoeira dos Índios | 8.693 |
| PB | 25 | 3407 | Cacimba de Areia | 3.604 |
| PB | 25 | 3506 | Cacimba de Dentro | 17.654 |
| PB | 25 | 3555 | Cacimbas | 7.029 |
| PB | 25 | 3605 | Caiçara | 7.535 |
| PB | 25 | 3704 | Cajazeiras | 57.875 |
| PB | 25 | 3753 | Cajazeirinhas | 3.168 |
| PB | 25 | 3803 | Caldas Brandão | 5.544 |
| PB | 25 | 3902 | Camalaú | 5.959 |
| PB | 25 | 4009 | Campina Grande | 383.764 |
| PB | 25 | 16409 | Campo de Santana | 9.854 |
| PB | 25 | 4033 | Capim | 5.458 |
| PB | 25 | 4074 | Caraúbas | 3.964 |
| PB | 25 | 4108 | Carrapateira | 2.347 |

| U.F. | | MUNICÍPIOS | | POPULAÇÃO ESTIMADA |
|---|---|---|---|---|
| SIGLA | COD | COD | NOMES | |
| PB | 25 | 4157 | Casserengue | 6.985 |
| PB | 25 | 4207 | Catingueira | 5.006 |
| PB | 25 | 4306 | Catolé do Rocha | 28.468 |
| PB | 25 | 4355 | Caturité | 4.629 |
| PB | 25 | 4405 | Conceição | 17.988 |
| PB | 25 | 4504 | Condado | 6.925 |
| PB | 25 | 4603 | Conde | 20.849 |
| PB | 25 | 4702 | Congo | 4.930 |
| PB | 25 | 4801 | Coremas | 15.709 |
| PB | 25 | 4850 | Coxixola | 1.770 |
| PB | 25 | 4900 | Cruz do Espírito Santo | 15.854 |
| PB | 25 | 5006 | Cubati | 6.546 |
| PB | 25 | 5105 | Cuité | 20.834 |
| PB | 25 | 5238 | Cuité de Mamanguape | 6.737 |
| PB | 25 | 5204 | Cuitegi | 7.243 |
| PB | 25 | 5279 | Curral de Cima | 5.648 |
| PB | 25 | 5303 | Curral Velho | 2.886 |
| PB | 25 | 5352 | Damião | 4.998 |
| PB | 25 | 5402 | Desterro | 8.191 |
| PB | 25 | 5600 | Diamante | 6.751 |
| PB | 25 | 5709 | Dona Inês | 11.142 |
| PB | 25 | 5808 | Duas Estradas | 3.855 |
| PB | 25 | 5907 | Emas | 3.374 |
| PB | 25 | 6004 | Esperança | 30.855 |
| PB | 25 | 6103 | Fagundes | 12.183 |
| PB | 25 | 6202 | Frei Martinho | 3.025 |
| PB | 25 | 6251 | Gado Bravo | 8.461 |
| PB | 25 | 6301 | Guarabira | 56.136 |
| PB | 25 | 6400 | Gurinhém | 13.998 |
| PB | 25 | 6509 | Gurjão | 3.093 |
| PB | 25 | 6608 | Ibiara | 6.304 |
| PB | 25 | 2607 | Igaracy | 6.529 |
| PB | 25 | 6707 | Imaculada | 11.851 |
| PB | 25 | 6806 | Ingá | 18.784 |
| PB | 25 | 6905 | Itabaiana | 25.463 |
| PB | 25 | 7002 | Itaporanga | 23.224 |
| PB | 25 | 7101 | Itapororoca | 16.573 |
| PB | 25 | 7200 | Itatuba | 10.182 |
| PB | 25 | 7309 | Jacaraú | 14.087 |
| PB | 25 | 7408 | Jericó | 8.100 |
| PB | 25 | 7507 | João Pessoa | 702.235 |
| PB | 25 | 7606 | Juarez Távora | 7.603 |
| PB | 25 | 7705 | Juazeirinho | 16.476 |
| PB | 25 | 7804 | Junco do Seridó | 6.731 |
| PB | 25 | 7903 | Juripiranga | 10.605 |
| PB | 25 | 8000 | Juru | 10.548 |
| PB | 25 | 8109 | Lagoa | 4.949 |
| PB | 25 | 8208 | Lagoa de Dentro | 7.495 |
| PB | 25 | 8307 | Lagoa Seca | 25.766 |
| PB | 25 | 8406 | Lastro | 2.894 |

| U.F. | | MUNICÍPIOS | | POPULAÇÃO ESTIMADA |
|---|---|---|---|---|
| SIGLA | COD | COD | NOMES | |
| PB | 25 | 8505 | Livramento | 7.330 |
| PB | 25 | 8554 | Logradouro | 3.971 |
| PB | 25 | 8604 | Lucena | 11.383 |
| PB | 25 | 8703 | Mãe d'Água | 4.127 |
| PB | 25 | 8802 | Malta | 5.800 |
| PB | 25 | 8901 | Mamanguape | 41.677 |
| PB | 25 | 9008 | Manaíra | 11.366 |
| PB | 25 | 9057 | Marcação | 7.606 |
| PB | 25 | 9107 | Mari | 21.137 |
| PB | 25 | 9156 | Marizópolis | 6.457 |
| PB | 25 | 9206 | Massaranduba | 12.946 |
| PB | 25 | 9305 | Mataraca | 7.299 |
| PB | 25 | 9339 | Matinhas | 4.314 |
| PB | 25 | 9370 | Mato Grosso | 2.695 |
| PB | 25 | 9396 | Maturéia | 6.029 |
| PB | 25 | 9404 | Mogeiro | 12.504 |
| PB | 25 | 9503 | Montadas | 4.750 |
| PB | 25 | 9602 | Monte Horebe | 4.498 |
| PB | 25 | 9701 | Monteiro | 31.100 |
| PB | 25 | 9800 | Mulungu | 9.629 |
| PB | 25 | 9909 | Natuba | 10.495 |
| PB | 25 | 10006 | Nazarezinho | 7.248 |
| PB | 25 | 10105 | Nova Floresta | 10.392 |
| PB | 25 | 10204 | Nova Olinda | 6.455 |
| PB | 25 | 10303 | Nova Palmeira | 4.087 |
| PB | 25 | 10402 | Olho d'Água | 7.642 |
| PB | 25 | 10501 | Olivedos | 3.622 |
| PB | 25 | 10600 | Ouro Velho | 3.078 |
| PB | 25 | 10659 | Parari | 1.266 |
| PB | 25 | 10709 | Passagem | 2.202 |
| PB | 25 | 10808 | Patos | 100.732 |
| PB | 25 | 10907 | Paulista | 12.004 |
| PB | 25 | 11004 | Pedra Branca | 3.861 |
| PB | 25 | 11103 | Pedra Lavrada | 7.035 |
| PB | 25 | 11202 | Pedras de Fogo | 27.116 |
| PB | 25 | 12721 | Pedro Régis | 5.822 |
| PB | 25 | 11301 | Piancó | 16.455 |
| PB | 25 | 11400 | Picuí | 19.359 |
| PB | 25 | 11509 | Pilar | 11.737 |
| PB | 25 | 11608 | Pilões | 7.068 |
| PB | 25 | 11707 | Pilõezinhos | 5.470 |
| PB | 25 | 11806 | Pirpirituba | 10.546 |
| PB | 25 | 11905 | Pitimbu | 16.832 |
| PB | 25 | 12002 | Pocinhos | 16.538 |
| PB | 25 | 12077 | Poço de José de Moura | 4.118 |
| PB | 25 | 12036 | Poço Dantas | 4.046 |
| PB | 25 | 12101 | Pombal | 32.443 |
| PB | 25 | 12200 | Prata | 4.057 |
| PB | 25 | 12309 | Princesa Isabel | 20.017 |
| PB | 25 | 12408 | Puxinanã | 13.354 |

| U.F. | | MUNICÍPIOS | | POPULAÇÃO ESTIMADA |
|---|---|---|---|---|
| SIGLA | COD | COD | NOMES | |
| PB | 25 | 12507 | Queimadas | 40.323 |
| PB | 25 | 12606 | Quixabá | 1.488 |
| PB | 25 | 12705 | Remígio | 17.423 |
| PB | 25 | 12747 | Riachão | 3.564 |
| PB | 25 | 12754 | Riachão do Bacamarte | 4.319 |
| PB | 25 | 12762 | Riachão do Poço | 4.417 |
| PB | 25 | 12788 | Riacho de Santo Antônio | 1.588 |
| PB | 25 | 12804 | Riacho dos Cavalos | 8.301 |
| PB | 25 | 12903 | Rio Tinto | 23.788 |
| PB | 25 | 13000 | Salgadinho | 3.518 |
| PB | 25 | 13109 | Salgado de São Félix | 12.950 |
| PB | 25 | 13158 | Santa Cecília | 7.244 |
| PB | 25 | 13208 | Santa Cruz | 6.677 |
| PB | 25 | 13307 | Santa Helena | 6.198 |
| PB | 25 | 13356 | Santa Inês | 3.834 |
| PB | 25 | 13406 | Santa Luzia | 14.752 |
| PB | 25 | 13703 | Santa Rita | 126.775 |
| PB | 25 | 13802 | Santa Teresinha | 4.778 |
| PB | 25 | 13505 | Santana de Mangueira | 5.764 |
| PB | 25 | 13604 | Santana dos Garrotes | 7.817 |
| PB | 25 | 13653 | Santarém | 2.749 |
| PB | 25 | 13851 | Santo André | 2.707 |
| PB | 25 | 13927 | São Bentinho | 4.214 |
| PB | 25 | 13901 | São Bento | 30.353 |
| PB | 25 | 13968 | São Domingos de Pombal | 2.822 |
| PB | 25 | 13943 | São Domingos do Cariri | 2.341 |
| PB | 25 | 13984 | São Francisco | 3.544 |
| PB | 25 | 14008 | São João do Cariri | 4.563 |
| PB | 25 | 700 | São João do Rio do Peixe | 18.323 |
| PB | 25 | 14107 | São João do Tigre | 4.726 |
| PB | 25 | 14206 | São José da Lagoa Tapada | 8.067 |
| PB | 25 | 14305 | São José de Caiana | 6.141 |
| PB | 25 | 14404 | São José de Espinharas | 5.045 |
| PB | 25 | 14503 | São José de Piranhas | 19.566 |
| PB | 25 | 14552 | São José de Princesa | 4.756 |
| PB | 25 | 14602 | São José do Bonfim | 3.314 |
| PB | 25 | 14651 | São José do Brejo do Cruz | 1.707 |
| PB | 25 | 14701 | São José do Sabugi | 4.115 |
| PB | 25 | 14800 | São José dos Cordeiros | 4.079 |
| PB | 25 | 14453 | São José dos Ramos | 5.702 |
| PB | 25 | 14909 | São Mamede | 7.998 |
| PB | 25 | 15005 | São Miguel de Taipu | 6.812 |
| PB | 25 | 15104 | São Sebastião de Lagoa de Roça | 11.320 |
| PB | 25 | 15203 | São Sebastião do Umbuzeiro | 3.170 |
| PB | 25 | 15302 | Sapé | 47.682 |
| PB | 25 | 15401 | Seridó | 10.091 |
| PB | 25 | 15609 | Serra da Raiz | 3.198 |
| PB | 25 | 15500 | Serra Branca | 12.816 |
| PB | 25 | 15708 | Serra Grande | 3.147 |
| PB | 25 | 15807 | Serra Redonda | 7.915 |

| U.F. | | MUNICÍPIOS | | POPULAÇÃO ESTIMADA |
|---|---|---|---|---|
| SIGLA | COD | COD | NOMES | |
| PB | 25 | 15906 | Serraria | 6.798 |
| PB | 25 | 15930 | Sertãozinho | 4.411 |
| PB | 25 | 15971 | Sobrado | 7.604 |
| PB | 25 | 16003 | Solânea | 27.951 |
| PB | 25 | 16102 | Soledade | 13.623 |
| PB | 25 | 16151 | Sossêgo | 3.089 |
| PB | 25 | 16201 | Sousa | 65.930 |
| PB | 25 | 16300 | Sumé | 17.085 |
| PB | 25 | 16508 | Taperoá | 15.236 |
| PB | 25 | 16607 | Tavares | 14.487 |
| PB | 25 | 16706 | Teixeira | 14.177 |
| PB | 25 | 16755 | Tenório | 2.921 |
| PB | 25 | 16805 | Triunfo | 9.968 |
| PB | 25 | 16904 | Uiraúna | 14.963 |
| PB | 25 | 17001 | Umbuzeiro | 9.450 |
| PB | 25 | 17100 | Várzea | 2.568 |
| PB | 25 | 17209 | Vieirópolis | 4.908 |
| PB | 25 | 5501 | Vista Serrana | 3.364 |
| PB | 25 | 17407 | Zabelê | 2.101 |
| PE | 26 | 54 | Abreu e Lima | 96.266 |
| PE | 26 | 104 | Afogados da Ingazeira | 35.528 |
| PE | 26 | 203 | Afrânio | 17.445 |
| PE | 26 | 302 | Agrestina | 22.591 |
| PE | 26 | 401 | Água Preta | 30.792 |
| PE | 26 | 500 | Águas Belas | 39.672 |
| PE | 26 | 609 | Alagoinha | 14.913 |
| PE | 26 | 708 | Aliança | 35.235 |
| PE | 26 | 807 | Altinho | 22.427 |
| PE | 26 | 906 | Amaraji | 20.509 |
| PE | 26 | 1003 | Angelim | 10.385 |
| PE | 26 | 1052 | Araçoiaba | 17.484 |
| PE | 26 | 1102 | Araripina | 79.877 |
| PE | 26 | 1201 | Arcoverde | 68.000 |
| PE | 26 | 1300 | Barra de Guabiraba | 13.623 |
| PE | 26 | 1409 | Barreiros | 43.911 |
| PE | 26 | 1508 | Belém de Maria | 9.703 |
| PE | 26 | 1607 | Belém de São Francisco | 21.342 |
| PE | 26 | 1706 | Belo Jardim | 74.028 |
| PE | 26 | 1805 | Betânia | 12.011 |
| PE | 26 | 1904 | Bezerros | 58.354 |
| PE | 26 | 2001 | Bodocó | 34.988 |
| PE | 26 | 2100 | Bom Conselho | 45.250 |
| PE | 26 | 2209 | Bom Jardim | 40.924 |
| PE | 26 | 2308 | Bonito | 40.832 |
| PE | 26 | 2407 | Brejão | 9.780 |
| PE | 26 | 2506 | Brejinho | 7.369 |
| PE | 26 | 2605 | Brejo da Madre de Deus | 42.250 |
| PE | 26 | 2704 | Buenos Aires | 13.675 |
| PE | 26 | 2803 | Buíque | 53.272 |
| PE | 26 | 2902 | Cabo de Santo Agostinho | 171.583 |

| U.F. | | MUNICÍPIOS | | POPULAÇÃO ESTIMADA |
|---|---|---|---|---|
| SIGLA | COD | COD | NOMES | |
| PE | 26 | 3009 | Cabrobó | 30.432 |
| PE | 26 | 3108 | Cachoeirinha | 18.123 |
| PE | 26 | 3207 | Caetés | 26.386 |
| PE | 26 | 3306 | Calçado | 11.619 |
| PE | 26 | 3405 | Calumbi | 7.977 |
| PE | 26 | 3454 | Camaragibe | 143.210 |
| PE | 26 | 3504 | Camocim de São Félix | 16.574 |
| PE | 26 | 3603 | Camutanga | 8.214 |
| PE | 26 | 3702 | Canhotinho | 24.847 |
| PE | 26 | 3801 | Capoeiras | 19.936 |
| PE | 26 | 3900 | Carnaíba | 19.155 |
| PE | 26 | 3926 | Carnaubeira da Penha | 12.451 |
| PE | 26 | 4007 | Carpina | 68.070 |
| PE | 26 | 4106 | Caruaru | 298.501 |
| PE | 26 | 4155 | Casinhas | 14.798 |
| PE | 26 | 4205 | Catende | 35.251 |
| PE | 26 | 4304 | Cedro | 10.784 |
| PE | 26 | 4403 | Chã de Alegria | 12.185 |
| PE | 26 | 4502 | Chã Grande | 17.924 |
| PE | 26 | 4601 | Condado | 24.403 |
| PE | 26 | 4700 | Correntes | 16.686 |
| PE | 26 | 4809 | Cortês | 11.712 |
| PE | 26 | 4908 | Cumaru | 13.812 |
| PE | 26 | 5004 | Cupira | 22.783 |
| PE | 26 | 5103 | Custódia | 33.874 |
| PE | 26 | 5152 | Dormentes | 16.462 |
| PE | 26 | 5202 | Escada | 62.604 |
| PE | 26 | 5301 | Exu | 31.086 |
| PE | 26 | 5400 | Feira Nova | 20.052 |
| PE | 26 | 5459 | Fernando de Noronha | 3.108 |
| PE | 26 | 5509 | Ferreiros | 11.456 |
| PE | 26 | 5608 | Flores | 23.034 |
| PE | 26 | 5707 | Floresta | 28.100 |
| PE | 26 | 5806 | Frei Miguelinho | 14.855 |
| PE | 26 | 5905 | Gameleira | 27.823 |
| PE | 26 | 6002 | Garanhuns | 131.313 |
| PE | 26 | 6101 | Glória do Goitá | 28.289 |
| PE | 26 | 6200 | Goiana | 74.424 |
| PE | 26 | 6309 | Granito | 6.955 |
| PE | 26 | 6408 | Gravatá | 75.229 |
| PE | 26 | 6507 | Iati | 18.350 |
| PE | 26 | 6606 | Ibimirim | 29.018 |
| PE | 26 | 6705 | Ibirajuba | 7.833 |
| PE | 26 | 6804 | Igarassu | 100.191 |
| PE | 26 | 6903 | Iguaraci | 12.397 |
| PE | 26 | 7604 | Ilha de Itamaracá | 18.658 |
| PE | 26 | 7000 | Inajá | 14.729 |
| PE | 26 | 7109 | Ingazeira | 4.561 |
| PE | 26 | 7208 | Ipojuca | 75.512 |
| PE | 26 | 7307 | Ipubi | 27.353 |

| U.F. | | MUNICÍPIOS | | POPULAÇÃO ESTIMADA |
|---|---|---|---|---|
| SIGLA | COD | COD | NOMES | |
| PE | 26 | 7406 | Itacuruba | 4.358 |
| PE | 26 | 7505 | Itaíba | 27.631 |
| PE | 26 | 7653 | Itambé | 36.126 |
| PE | 26 | 7703 | Itapetim | 14.063 |
| PE | 26 | 7752 | Itapissuma | 24.406 |
| PE | 26 | 7802 | Itaquitinga | 15.507 |
| PE | 26 | 7901 | Jaboatão dos Guararapes | 687.688 |
| PE | 26 | 7950 | Jaqueira | 12.642 |
| PE | 26 | 8008 | Jataúba | 15.365 |
| PE | 26 | 8057 | Jatobá | 14.452 |
| PE | 26 | 8107 | João Alfredo | 29.875 |
| PE | 26 | 8206 | Joaquim Nabuco | 16.498 |
| PE | 26 | 8255 | Jucati | 11.086 |
| PE | 26 | 8305 | Jupi | 14.461 |
| PE | 26 | 8404 | Jurema | 15.552 |
| PE | 26 | 8453 | Lagoa do Carro | 15.230 |
| PE | 26 | 8503 | Lagoa do Itaenga | 20.618 |
| PE | 26 | 8602 | Lagoa do Ouro | 12.244 |
| PE | 26 | 8701 | Lagoa dos Gatos | 15.567 |
| PE | 26 | 8750 | Lagoa Grande | 22.408 |
| PE | 26 | 8800 | Lajedo | 34.809 |
| PE | 26 | 8909 | Limoeiro | 57.243 |
| PE | 26 | 9006 | Macaparana | 24.031 |
| PE | 26 | 9105 | Machados | 11.802 |
| PE | 26 | 9154 | Manari | 18.097 |
| PE | 26 | 9204 | Maraial | 12.303 |
| PE | 26 | 9303 | Mirandiba | 13.810 |
| PE | 26 | 14303 | Moreilândia | 10.584 |
| PE | 26 | 9402 | Moreno | 55.659 |
| PE | 26 | 9501 | Nazaré da Mata | 30.185 |
| PE | 26 | 9600 | Olinda | 397.268 |
| PE | 26 | 9709 | Orobó | 22.239 |
| PE | 26 | 9808 | Orocó | 14.279 |
| PE | 26 | 9907 | Ouricuri | 66.978 |
| PE | 26 | 10004 | Palmares | 58.819 |
| PE | 26 | 10103 | Palmeirina | 8.481 |
| PE | 26 | 10202 | Panelas | 25.500 |
| PE | 26 | 10301 | Paranatama | 12.441 |
| PE | 26 | 10400 | Parnamirim | 19.850 |
| PE | 26 | 10509 | Passira | 28.518 |
| PE | 26 | 10608 | Paudalho | 47.521 |
| PE | 26 | 10707 | Paulista | 319.373 |
| PE | 26 | 10806 | Pedra | 20.788 |
| PE | 26 | 10905 | Pesqueira | 64.454 |
| PE | 26 | 11002 | Petrolândia | 32.568 |
| PE | 26 | 11101 | Petrolina | 281.851 |
| PE | 26 | 11200 | Poção | 11.503 |
| PE | 26 | 11309 | Pombos | 22.120 |
| PE | 26 | 11408 | Primavera | 12.364 |
| PE | 26 | 11507 | Quipapá | 25.603 |

| U.F. | | MUNICÍPIOS | | POPULAÇÃO ESTIMADA |
|---|---|---|---|---|
| SIGLA | COD | COD | NOMES | |
| PE | 26 | 11533 | Quixaba | 7.116 |
| PE | 26 | 11606 | Recife | 1.561.659 |
| PE | 26 | 11705 | Riacho das Almas | 18.930 |
| PE | 26 | 11804 | Ribeirão | 39.317 |
| PE | 26 | 11903 | Rio Formoso | 21.815 |
| PE | 26 | 12000 | Sairé | 14.194 |
| PE | 26 | 12109 | Salgadinho | 8.214 |
| PE | 26 | 12208 | Salgueiro | 55.435 |
| PE | 26 | 12307 | Saloá | 15.547 |
| PE | 26 | 12406 | Sanharó | 18.723 |
| PE | 26 | 12455 | Santa Cruz | 14.782 |
| PE | 26 | 12471 | Santa Cruz da Baixa Verde | 12.209 |
| PE | 26 | 12505 | Santa Cruz do Capibaribe | 80.330 |
| PE | 26 | 12554 | Santa Filomena | 14.694 |
| PE | 26 | 12604 | Santa Maria da Boa Vista | 41.745 |
| PE | 26 | 12703 | Santa Maria do Cambucá | 12.942 |
| PE | 26 | 12802 | Santa Terezinha | 10.184 |
| PE | 26 | 12901 | São Benedito do Sul | 10.838 |
| PE | 26 | 13008 | São Bento do Una | 49.372 |
| PE | 26 | 13107 | São Caitano | 36.336 |
| PE | 26 | 13206 | São João | 22.087 |
| PE | 26 | 13305 | São Joaquim do Monte | 21.872 |
| PE | 26 | 13404 | São José da Coroa Grande | 18.555 |
| PE | 26 | 13503 | São José do Belmonte | 34.118 |
| PE | 26 | 13602 | São José do Egito | 31.792 |
| PE | 26 | 13701 | São Lourenço da Mata | 99.945 |
| PE | 26 | 13800 | São Vicente Ferrer | 17.333 |
| PE | 26 | 13909 | Serra Talhada | 80.294 |
| PE | 26 | 14006 | Serrita | 18.958 |
| PE | 26 | 14105 | Sertânia | 35.914 |
| PE | 26 | 14204 | Sirinhaém | 38.610 |
| PE | 26 | 14402 | Solidão | 6.123 |
| PE | 26 | 14501 | Surubim | 56.795 |
| PE | 26 | 14600 | Tabira | 27.219 |
| PE | 26 | 14709 | Tacaimbó | 12.273 |
| PE | 26 | 14808 | Tacaratu | 22.231 |
| PE | 26 | 14857 | Tamandaré | 18.999 |
| PE | 26 | 15003 | Taquaritinga do Norte | 22.657 |
| PE | 26 | 15102 | Terezinha | 6.774 |
| PE | 26 | 15201 | Terra Nova | 9.801 |
| PE | 26 | 15300 | Timbaúba | 51.770 |
| PE | 26 | 15409 | Toritama | 33.206 |
| PE | 26 | 15508 | Tracunhaém | 13.265 |
| PE | 26 | 15607 | Trindade | 26.250 |
| PE | 26 | 15706 | Triunfo | 15.770 |
| PE | 26 | 15805 | Tupanatinga | 19.026 |
| PE | 26 | 15904 | Tuparetama | 8.678 |
| PE | 26 | 16001 | Venturosa | 16.706 |
| PE | 26 | 16100 | Verdejante | 10.098 |
| PE | 26 | 16183 | Vertente do Lério | 7.464 |

| U.F. | | MUNICÍPIOS | | POPULAÇÃO ESTIMADA |
|---|---|---|---|---|
| SIGLA | COD | COD | NOMES | |
| PE | 26 | 16209 | Vertentes | 18.186 |
| PE | 26 | 16308 | Vicência | 27.883 |
| PE | 26 | 16407 | Vitória de Santo Antão | 126.399 |
| PE | 26 | 16506 | Xexéu | 14.887 |
| AL | 27 | 102 | Água Branca | 19.989 |
| AL | 27 | 201 | Anadia | 17.740 |
| AL | 27 | 300 | Arapiraca | 210.521 |
| AL | 27 | 409 | Atalaia | 53.023 |
| AL | 27 | 508 | Barra de Santo Antônio | 14.435 |
| AL | 27 | 607 | Barra de São Miguel | 7.572 |
| AL | 27 | 706 | Batalha | 16.919 |
| AL | 27 | 805 | Belém | 5.083 |
| AL | 27 | 904 | Belo Monte | 7.510 |
| AL | 27 | 1001 | Boca da Mata | 26.030 |
| AL | 27 | 1100 | Branquinha | 12.215 |
| AL | 27 | 1209 | Cacimbinhas | 10.182 |
| AL | 27 | 1308 | Cajueiro | 20.825 |
| AL | 27 | 1357 | Campestre | 6.178 |
| AL | 27 | 1407 | Campo Alegre | 47.209 |
| AL | 27 | 1506 | Campo Grande | 9.909 |
| AL | 27 | 1605 | Canapi | 18.397 |
| AL | 27 | 1704 | Capela | 17.366 |
| AL | 27 | 1803 | Carneiros | 8.463 |
| AL | 27 | 1902 | Chã Preta | 7.073 |
| AL | 27 | 2009 | Coité do Nóia | 11.127 |
| AL | 27 | 2108 | Colônia Leopoldina | 20.181 |
| AL | 27 | 2207 | Coqueiro Seco | 5.525 |
| AL | 27 | 2306 | Coruripe | 53.369 |
| AL | 27 | 2355 | Craíbas | 23.294 |
| AL | 27 | 2405 | Delmiro Gouveia | 48.462 |
| AL | 27 | 2504 | Dois Riachos | 11.200 |
| AL | 27 | 2553 | Estrela de Alagoas | 17.251 |
| AL | 27 | 2603 | Feira Grande | 21.824 |
| AL | 27 | 2702 | Feliz Deserto | 4.568 |
| AL | 27 | 2801 | Flexeiras | 12.290 |
| AL | 27 | 2900 | Girau do Ponciano | 36.904 |
| AL | 27 | 3007 | Ibateguara | 15.863 |
| AL | 27 | 3106 | Igaci | 25.865 |
| AL | 27 | 3205 | Igreja Nova | 23.807 |
| AL | 27 | 3304 | Inhapi | 18.166 |
| AL | 27 | 3403 | Jacaré dos Homens | 5.902 |
| AL | 27 | 3502 | Jacuípe | 7.045 |
| AL | 27 | 3601 | Japaratinga | 7.763 |
| AL | 27 | 3700 | Jaramataia | 6.140 |
| AL | 27 | 3759 | Jequiá da Praia | 11.615 |
| AL | 27 | 3809 | Joaquim Gomes | 22.436 |
| AL | 27 | 3908 | Jundiá | 4.698 |
| AL | 27 | 4005 | Junqueiro | 25.290 |
| AL | 27 | 4104 | Lagoa da Canoa | 18.196 |
| AL | 27 | 4203 | Limoeiro de Anadia | 26.415 |

| U.F. | | MUNICÍPIOS | | POPULAÇÃO ESTIMADA |
|---|---|---|---|---|
| SIGLA | COD | COD | NOMES | |
| AL | 27 | 4302 | Maceió | 936.314 |
| AL | 27 | 4401 | Major Isidoro | 19.530 |
| AL | 27 | 4906 | Mar Vermelho | 4.131 |
| AL | 27 | 4500 | Maragogi | 26.978 |
| AL | 27 | 4609 | Maravilha | 10.110 |
| AL | 27 | 4708 | Marechal Deodoro | 47.623 |
| AL | 27 | 4807 | Maribondo | 14.144 |
| AL | 27 | 5002 | Mata Grande | 25.309 |
| AL | 27 | 5101 | Matriz de Camaragibe | 25.493 |
| AL | 27 | 5200 | Messias | 15.899 |
| AL | 27 | 5309 | Minador do Negrão | 5.292 |
| AL | 27 | 5408 | Monteirópolis | 7.292 |
| AL | 27 | 5507 | Murici | 26.918 |
| AL | 27 | 5606 | Novo Lino | 12.449 |
| AL | 27 | 5705 | Olho d'Água das Flores | 20.555 |
| AL | 27 | 5804 | Olho d'Água do Casado | 8.517 |
| AL | 27 | 5903 | Olho d'Água Grande | 4.963 |
| AL | 27 | 6000 | Olivença | 10.865 |
| AL | 27 | 6109 | Ouro Branco | 11.504 |
| AL | 27 | 6208 | Palestina | 5.071 |
| AL | 27 | 6307 | Palmeira dos Índios | 72.564 |
| AL | 27 | 6406 | Pão de Açúcar | 24.534 |
| AL | 27 | 6422 | Pariconha | 10.539 |
| AL | 27 | 6448 | Paripueira | 10.222 |
| AL | 27 | 6505 | Passo de Camaragibe | 14.261 |
| AL | 27 | 6604 | Paulo Jacinto | 7.772 |
| AL | 27 | 6703 | Penedo | 61.082 |
| AL | 27 | 6802 | Piaçabuçu | 18.087 |
| AL | 27 | 6901 | Pilar | 32.655 |
| AL | 27 | 7008 | Pindoba | 3.246 |
| AL | 27 | 7107 | Piranhas | 25.107 |
| AL | 27 | 7206 | Poço das Trincheiras | 12.463 |
| AL | 27 | 7404 | Porto de Pedras | 10.649 |
| AL | 27 | 7305 | Porto Calvo | 26.044 |
| AL | 27 | 7503 | Porto Real do Colégio | 18.453 |
| AL | 27 | 7602 | Quebrangulo | 11.566 |
| AL | 27 | 7701 | Rio Largo | 67.797 |
| AL | 27 | 7800 | Roteiro | 6.864 |
| AL | 27 | 7909 | Santa Luzia do Norte | 7.572 |
| AL | 27 | 8006 | Santana do Ipanema | 43.699 |
| AL | 27 | 8105 | Santana do Mundaú | 12.039 |
| AL | 27 | 8204 | São Brás | 7.062 |
| AL | 27 | 8303 | São José da Laje | 23.112 |
| AL | 27 | 8402 | São José da Tapera | 31.361 |
| AL | 27 | 8501 | São Luís do Quitunde | 32.871 |
| AL | 27 | 8600 | São Miguel dos Campos | 54.064 |
| AL | 27 | 8709 | São Miguel dos Milagres | 7.601 |
| AL | 27 | 8808 | São Sebastião | 32.181 |
| AL | 27 | 8907 | Satuba | 14.779 |
| AL | 27 | 8956 | Senador Rui Palmeira | 13.110 |

| U.F. | | MUNICÍPIOS | | POPULAÇÃO ESTIMADA |
|---|---|---|---|---|
| SIGLA | COD | COD | NOMES | |
| AL | 27 | 9004 | Tanque d'Arca | 5.766 |
| AL | 27 | 9103 | Taquarana | 18.848 |
| AL | 27 | 9152 | Teotônio Vilela | 41.935 |
| AL | 27 | 9202 | Traipu | 25.854 |
| AL | 27 | 9301 | União dos Palmares | 62.727 |
| AL | 27 | 9400 | Viçosa | 26.830 |
| SE | 28 | 100 | Amparo de São Francisco | 2.268 |
| SE | 28 | 209 | Aquidabã | 19.890 |
| SE | 28 | 308 | Aracaju | 544.039 |
| SE | 28 | 407 | Arauá | 12.042 |
| SE | 28 | 506 | Areia Branca | 16.733 |
| SE | 28 | 605 | Barra dos Coqueiros | 19.998 |
| SE | 28 | 670 | Boquim | 25.270 |
| SE | 28 | 704 | Brejo Grande | 8.086 |
| SE | 28 | 1009 | Campo do Brito | 16.745 |
| SE | 28 | 1108 | Canhoba | 4.025 |
| SE | 28 | 1207 | Canindé de São Francisco | 23.005 |
| SE | 28 | 1306 | Capela | 28.960 |
| SE | 28 | 1405 | Carira | 19.708 |
| SE | 28 | 1504 | Carmópolis | 12.610 |
| SE | 28 | 1603 | Cedro de São João | 5.522 |
| SE | 28 | 1702 | Cristinápolis | 16.131 |
| SE | 28 | 1900 | Cumbe | 3.870 |
| SE | 28 | 2007 | Divina Pastora | 4.448 |
| SE | 28 | 2106 | Estância | 63.582 |
| SE | 28 | 2205 | Feira Nova | 5.875 |
| SE | 28 | 2304 | Frei Paulo | 13.060 |
| SE | 28 | 2403 | Gararu | 11.999 |
| SE | 28 | 2502 | General Maynard | 2.907 |
| SE | 28 | 2601 | Gracho Cardoso | 5.732 |
| SE | 28 | 2700 | Ilha das Flores | 8.906 |
| SE | 28 | 2809 | Indiaroba | 18.126 |
| SE | 28 | 2908 | Itabaiana | 86.564 |
| SE | 28 | 3005 | Itabaianinha | 38.850 |
| SE | 28 | 3104 | Itabi | 4.827 |
| SE | 28 | 3203 | Itaporanga d'Ajuda | 29.347 |
| SE | 28 | 3302 | Japaratuba | 16.046 |
| SE | 28 | 3401 | Japoatã | 14.027 |
| SE | 28 | 3500 | Lagarto | 92.474 |
| SE | 28 | 3609 | Laranjeiras | 24.714 |
| SE | 28 | 3708 | Macambira | 6.554 |
| SE | 28 | 3807 | Malhada dos Bois | 3.830 |
| SE | 28 | 3906 | Malhador | 12.124 |
| SE | 28 | 4003 | Maruim | 15.582 |
| SE | 28 | 4102 | Moita Bonita | 11.269 |
| SE | 28 | 4201 | Monte Alegre de Sergipe | 13.817 |
| SE | 28 | 4300 | Muribeca | 7.466 |
| SE | 28 | 4409 | Neópolis | 19.538 |
| SE | 28 | 4508 | Nossa Senhora da Glória | 30.804 |
| SE | 28 | 4607 | Nossa Senhora das Dores | 24.747 |

| U.F. | | MUNICÍPIOS | | POPULAÇÃO |
|---|---|---|---|---|
| SIGLA | COD | COD | NOMES | ESTIMADA |
| SE | 28 | 4706 | Nossa Senhora de Lourdes | 6.509 |
| SE | 28 | 4805 | Nossa Senhora do Socorro | 155.334 |
| SE | 28 | 4458 | Nossa Senhora Aparecida | 8.813 |
| SE | 28 | 4904 | Pacatuba | 12.870 |
| SE | 28 | 5000 | Pedra Mole | 2.878 |
| SE | 28 | 5109 | Pedrinhas | 8.709 |
| SE | 28 | 5208 | Pinhão | 5.809 |
| SE | 28 | 5307 | Pirambu | 8.608 |
| SE | 28 | 5406 | Poço Redondo | 30.249 |
| SE | 28 | 5505 | Poço Verde | 21.882 |
| SE | 28 | 5604 | Porto da Folha | 27.456 |
| SE | 28 | 5703 | Propriá | 28.520 |
| SE | 28 | 5802 | Riachão do Dantas | 19.588 |
| SE | 28 | 5901 | Riachuelo | 9.466 |
| SE | 28 | 6008 | Ribeirópolis | 16.194 |
| SE | 28 | 6107 | Rosário do Catete | 8.965 |
| SE | 28 | 6206 | Salgado | 19.101 |
| SE | 28 | 6305 | Santa Luzia do Itanhy | 13.502 |
| SE | 28 | 6503 | Santa Rosa de Lima | 3.996 |
| SE | 28 | 6404 | Santana do São Francisco | 6.861 |
| SE | 28 | 6602 | Santo Amaro das Brotas | 12.140 |
| SE | 28 | 6701 | São Cristóvão | 75.104 |
| SE | 28 | 6800 | São Domingos | 10.555 |
| SE | 28 | 6909 | São Francisco | 3.007 |
| SE | 28 | 7006 | São Miguel do Aleixo | 3.813 |
| SE | 28 | 7105 | Simão Dias | 38.343 |
| SE | 28 | 7204 | Siriri | 7.946 |
| SE | 28 | 7303 | Telha | 2.969 |
| SE | 28 | 7402 | Tobias Barreto | 49.261 |
| SE | 28 | 7501 | Tomar do Geru | 13.717 |
| SE | 28 | 7600 | Umbaúba | 21.397 |
| BA | 29 | 108 | Abaíra | 8.821 |
| BA | 29 | 207 | Abaré | 18.831 |
| BA | 29 | 306 | Acajutiba | 15.192 |
| BA | 29 | 355 | Adustina | 15.448 |
| BA | 29 | 405 | Água Fria | 15.328 |
| BA | 29 | 603 | Aiquara | 5.361 |
| BA | 29 | 702 | Alagoinhas | 137.810 |
| BA | 29 | 801 | Alcobaça | 20.242 |
| BA | 29 | 900 | Almadina | 6.621 |
| BA | 29 | 1007 | Amargosa | 35.278 |
| BA | 29 | 1106 | Amélia Rodrigues | 24.491 |
| BA | 29 | 1155 | América Dourada | 16.787 |
| BA | 29 | 1205 | Anagé | 25.262 |
| BA | 29 | 1304 | Andaraí | 14.609 |
| BA | 29 | 1353 | Andorinha | 14.201 |
| BA | 29 | 1403 | Angical | 15.191 |
| BA | 29 | 1502 | Anguera | 9.826 |
| BA | 29 | 1601 | Antas | 17.583 |
| BA | 29 | 1700 | Antônio Cardoso | 12.589 |

| U.F. | | MUNICÍPIOS | | POPULAÇÃO ESTIMADA |
|---|---|---|---|---|
| SIGLA | COD | COD | NOMES | |
| BA | 29 | 1809 | Antônio Gonçalves | 11.240 |
| BA | 29 | 1908 | Aporá | 18.738 |
| BA | 29 | 1957 | Apuarema | 7.610 |
| BA | 29 | 2054 | Araças | 12.209 |
| BA | 29 | 2005 | Aracatu | 14.316 |
| BA | 29 | 2104 | Araci | 54.713 |
| BA | 29 | 2203 | Aramari | 9.911 |
| BA | 29 | 2252 | Arataca | 10.953 |
| BA | 29 | 2302 | Aratuípe | 8.822 |
| BA | 29 | 2401 | Aurelino Leal | 14.280 |
| BA | 29 | 2500 | Baianópolis | 14.195 |
| BA | 29 | 2609 | Baixa Grande | 21.814 |
| BA | 29 | 2658 | Banzaê | 11.157 |
| BA | 29 | 2708 | Barra | 50.226 |
| BA | 29 | 2807 | Barra da Estiva | 20.537 |
| BA | 29 | 2906 | Barra do Choça | 31.527 |
| BA | 29 | 3003 | Barra do Mendes | 14.459 |
| BA | 29 | 3102 | Barra do Rocha | 5.860 |
| BA | 29 | 3201 | Barreiras | 137.832 |
| BA | 29 | 3235 | Barro Alto | 14.172 |
| BA | 29 | 3300 | Barro Preto | 6.417 |
| BA | 29 | 3276 | Barrocas | 13.868 |
| BA | 29 | 3409 | Belmonte | 22.553 |
| BA | 29 | 3508 | Belo Campo | 15.185 |
| BA | 29 | 3607 | Biritinga | 14.260 |
| BA | 29 | 3706 | Boa Nova | 15.427 |
| BA | 29 | 3805 | Boa Vista do Tupim | 18.298 |
| BA | 29 | 3904 | Bom Jesus da Lapa | 66.192 |
| BA | 29 | 3953 | Bom Jesus da Serra | 10.588 |
| BA | 29 | 4001 | Boninal | 13.857 |
| BA | 29 | 4050 | Bonito | 14.326 |
| BA | 29 | 4100 | Boquira | 22.521 |
| BA | 29 | 4209 | Botuporã | 11.048 |
| BA | 29 | 4308 | Brejões | 12.446 |
| BA | 29 | 4407 | Brejolândia | 9.791 |
| BA | 29 | 4506 | Brotas de Macaúbas | 10.949 |
| BA | 29 | 4605 | Brumado | 64.642 |
| BA | 29 | 4704 | Buerarema | 20.830 |
| BA | 29 | 4753 | Buritirama | 19.492 |
| BA | 29 | 4803 | Caatiba | 9.450 |
| BA | 29 | 4852 | Cabaceiras do Paraguaçu | 18.569 |
| BA | 29 | 4902 | Cachoeira | 33.782 |
| BA | 29 | 5008 | Caculé | 22.557 |
| BA | 29 | 5107 | Caém | 10.187 |
| BA | 29 | 5156 | Caetanos | 12.307 |
| BA | 29 | 5206 | Caetité | 48.007 |
| BA | 29 | 5305 | Cafarnaum | 18.314 |
| BA | 29 | 5404 | Cairu | 14.736 |
| BA | 29 | 5503 | Caldeirão Grande | 13.922 |
| BA | 29 | 5602 | Camacan | 31.113 |

| U.F. | | MUNICÍPIOS | | POPULAÇÃO ESTIMADA |
|---|---|---|---|---|
| SIGLA | COD | COD | NOMES | |
| BA | 29 | 5701 | Camaçari | 234.558 |
| BA | 29 | 5800 | Camamu | 32.881 |
| BA | 29 | 5909 | Campo Alegre de Lourdes | 27.670 |
| BA | 29 | 6006 | Campo Formoso | 68.101 |
| BA | 29 | 6105 | Canápolis | 11.136 |
| BA | 29 | 6204 | Canarana | 25.935 |
| BA | 29 | 6303 | Canavieiras | 37.041 |
| BA | 29 | 6402 | Candeal | 9.050 |
| BA | 29 | 6501 | Candeias | 81.699 |
| BA | 29 | 6600 | Candiba | 12.821 |
| BA | 29 | 6709 | Cândido Sales | 27.180 |
| BA | 29 | 6808 | Cansanção | 34.093 |
| BA | 29 | 6824 | Canudos | 15.366 |
| BA | 29 | 6857 | Capela do Alto Alegre | 12.824 |
| BA | 29 | 6873 | Capim Grosso | 27.158 |
| BA | 29 | 6899 | Caraíbas | 10.495 |
| BA | 29 | 6907 | Caravelas | 22.115 |
| BA | 29 | 7004 | Cardeal da Silva | 8.618 |
| BA | 29 | 7103 | Carinhanha | 30.240 |
| BA | 29 | 7202 | Casa Nova | 66.718 |
| BA | 29 | 7301 | Castro Alves | 24.978 |
| BA | 29 | 7400 | Catolândia | 4.059 |
| BA | 29 | 7509 | Catu | 50.809 |
| BA | 29 | 7558 | Caturama | 8.713 |
| BA | 29 | 7608 | Central | 18.029 |
| BA | 29 | 7707 | Chorrochó | 11.022 |
| BA | 29 | 7806 | Cícero Dantas | 31.832 |
| BA | 29 | 7905 | Cipó | 15.758 |
| BA | 29 | 8002 | Coaraci | 22.274 |
| BA | 29 | 8101 | Cocos | 17.923 |
| BA | 29 | 8200 | Conceição da Feira | 20.117 |
| BA | 29 | 8309 | Conceição do Almeida | 17.974 |
| BA | 29 | 8408 | Conceição do Coité | 62.893 |
| BA | 29 | 8507 | Conceição do Jacuípe | 28.769 |
| BA | 29 | 8606 | Conde | 23.166 |
| BA | 29 | 8705 | Condeúba | 17.210 |
| BA | 29 | 8804 | Contendas do Sincorá | 3.886 |
| BA | 29 | 8903 | Coração de Maria | 23.774 |
| BA | 29 | 9000 | Cordeiros | 8.883 |
| BA | 29 | 9109 | Coribe | 14.895 |
| BA | 29 | 9208 | Coronel João Sá | 18.408 |
| BA | 29 | 9307 | Correntina | 32.980 |
| BA | 29 | 9406 | Cotegipe | 14.191 |
| BA | 29 | 9505 | Cravolândia | 5.713 |
| BA | 29 | 9604 | Crisópolis | 20.279 |
| BA | 29 | 9703 | Cristópolis | 14.279 |
| BA | 29 | 9802 | Cruz das Almas | 57.098 |
| BA | 29 | 9901 | Curaçá | 34.421 |
| BA | 29 | 10008 | Dário Meira | 12.331 |
| BA | 29 | 10057 | Dias d'Ávila | 57.708 |

| U.F. | | MUNICÍPIOS | | POPULAÇÃO ESTIMADA |
|---|---|---|---|---|
| SIGLA | COD | COD | NOMES | |
| BA | 29 | 10107 | Dom Basílio | 11.620 |
| BA | 29 | 10206 | Dom Macedo Costa | 3.951 |
| BA | 29 | 10305 | Elísio Medrado | 8.183 |
| BA | 29 | 10404 | Encruzilhada | 20.720 |
| BA | 29 | 10503 | Entre Rios | 40.524 |
| BA | 29 | 504 | Érico Cardoso | 10.717 |
| BA | 29 | 10602 | Esplanada | 33.115 |
| BA | 29 | 10701 | Euclides da Cunha | 59.193 |
| BA | 29 | 10727 | Eunápolis | 99.553 |
| BA | 29 | 10750 | Fátima | 19.703 |
| BA | 29 | 10776 | Feira da Mata | 6.562 |
| BA | 29 | 10800 | Feira de Santana | 591.707 |
| BA | 29 | 10859 | Filadélfia | 16.215 |
| BA | 29 | 10909 | Firmino Alves | 5.864 |
| BA | 29 | 11006 | Floresta Azul | 10.364 |
| BA | 29 | 11105 | Formosa do Rio Preto | 22.171 |
| BA | 29 | 11204 | Gandu | 31.819 |
| BA | 29 | 11253 | Gavião | 4.507 |
| BA | 29 | 11303 | Gentio do Ouro | 11.988 |
| BA | 29 | 11402 | Glória | 14.176 |
| BA | 29 | 11501 | Gongogi | 6.170 |
| BA | 29 | 11600 | Governador Mangabeira | 20.668 |
| BA | 29 | 11659 | Guajeru | 9.330 |
| BA | 29 | 11709 | Guanambi | 79.886 |
| BA | 29 | 11808 | Guaratinga | 22.960 |
| BA | 29 | 11857 | Heliópolis | 14.713 |
| BA | 29 | 11907 | Iaçu | 28.602 |
| BA | 29 | 12004 | Ibiassucê | 9.008 |
| BA | 29 | 12103 | Ibicaraí | 24.569 |
| BA | 29 | 12202 | Ibicoara | 16.940 |
| BA | 29 | 12301 | Ibicuí | 16.464 |
| BA | 29 | 12400 | Ibipeba | 17.666 |
| BA | 29 | 12509 | Ibipitanga | 14.408 |
| BA | 29 | 12608 | Ibiquera | 5.300 |
| BA | 29 | 12707 | Ibirapitanga | 24.223 |
| BA | 29 | 12806 | Ibirapuã | 7.893 |
| BA | 29 | 12905 | Ibirataia | 24.544 |
| BA | 29 | 13002 | Ibitiara | 16.665 |
| BA | 29 | 13101 | Ibititá | 19.410 |
| BA | 29 | 13200 | Ibotirama | 26.419 |
| BA | 29 | 13309 | Ichu | 6.148 |
| BA | 29 | 13408 | Igaporã | 14.970 |
| BA | 29 | 13457 | Igrapiúna | 13.268 |
| BA | 29 | 13507 | Iguaí | 29.449 |
| BA | 29 | 13606 | Ilhéus | 219.266 |
| BA | 29 | 13705 | Inhambupe | 36.719 |
| BA | 29 | 13804 | Ipecaetá | 15.888 |
| BA | 29 | 13903 | Ipiaú | 43.723 |
| BA | 29 | 14000 | Ipirá | 62.197 |
| BA | 29 | 14109 | Ipupiara | 9.325 |

| U.F. | | MUNICÍPIOS | | POPULAÇÃO ESTIMADA |
|---|---|---|---|---|
| SIGLA | COD | COD | NOMES | |
| BA | 29 | 14208 | Irajuba | 7.666 |
| BA | 29 | 14307 | Iramaia | 15.045 |
| BA | 29 | 14406 | Iraquara | 24.415 |
| BA | 29 | 14505 | Irará | 25.811 |
| BA | 29 | 14604 | Irecê | 66.061 |
| BA | 29 | 14653 | Itabela | 26.704 |
| BA | 29 | 14703 | Itaberaba | 61.490 |
| BA | 29 | 14802 | Itabuna | 213.656 |
| BA | 29 | 14901 | Itacaré | 27.170 |
| BA | 29 | 15007 | Itaeté | 14.664 |
| BA | 29 | 15106 | Itagi | 14.162 |
| BA | 29 | 15205 | Itagibá | 16.590 |
| BA | 29 | 15304 | Itagimirim | 7.118 |
| BA | 29 | 15353 | Itaguaçu da Bahia | 13.269 |
| BA | 29 | 15403 | Itaju do Colônia | 7.706 |
| BA | 29 | 15502 | Itajuípe | 20.490 |
| BA | 29 | 15601 | Itamaraju | 67.803 |
| BA | 29 | 15700 | Itamari | 8.781 |
| BA | 29 | 15809 | Itambé | 35.512 |
| BA | 29 | 15908 | Itanagra | 6.884 |
| BA | 29 | 16005 | Itanhém | 21.154 |
| BA | 29 | 16104 | Itaparica | 20.796 |
| BA | 29 | 16203 | Itapé | 10.630 |
| BA | 29 | 16302 | Itapebi | 12.003 |
| BA | 29 | 16401 | Itapetinga | 66.665 |
| BA | 29 | 16500 | Itapicuru | 32.563 |
| BA | 29 | 16609 | Itapitanga | 10.376 |
| BA | 29 | 16708 | Itaquara | 7.743 |
| BA | 29 | 16807 | Itarantim | 18.375 |
| BA | 29 | 16856 | Itatim | 15.516 |
| BA | 29 | 16906 | Itiruçu | 16.827 |
| BA | 29 | 17003 | Itiúba | 36.996 |
| BA | 29 | 17102 | Itororó | 20.930 |
| BA | 29 | 17201 | Ituaçu | 18.772 |
| BA | 29 | 17300 | Ituberá | 24.169 |
| BA | 29 | 17334 | Iuiú | 12.093 |
| BA | 29 | 17359 | Jaborandi | 8.895 |
| BA | 29 | 17409 | Jacaraci | 14.837 |
| BA | 29 | 17508 | Jacobina | 79.013 |
| BA | 29 | 17607 | Jaguaquara | 48.206 |
| BA | 29 | 17706 | Jaguarari | 30.484 |
| BA | 29 | 17805 | Jaguaripe | 17.435 |
| BA | 29 | 17904 | Jandaíra | 10.018 |
| BA | 29 | 18001 | Jequié | 150.541 |
| BA | 29 | 18100 | Jeremoabo | 39.302 |
| BA | 29 | 18209 | Jiquiriçá | 13.772 |
| BA | 29 | 18308 | Jitaúna | 16.365 |
| BA | 29 | 18357 | João Dourado | 21.990 |
| BA | 29 | 18407 | Juazeiro | 243.896 |
| BA | 29 | 18456 | Jucuruçu | 10.516 |

| U.F. | | MUNICÍPIOS | | POPULAÇÃO ESTIMADA |
|---|---|---|---|---|
| SIGLA | COD | COD | NOMES | |
| BA | 29 | 18506 | Jussara | 15.204 |
| BA | 29 | 18555 | Jussari | 6.914 |
| BA | 29 | 18605 | Jussiape | 8.043 |
| BA | 29 | 18704 | Lafaiete Coutinho | 3.502 |
| BA | 29 | 18753 | Lagoa Real | 14.510 |
| BA | 29 | 18803 | Laje | 22.180 |
| BA | 29 | 18902 | Lajedão | 3.599 |
| BA | 29 | 19009 | Lajedinho | 4.468 |
| BA | 29 | 19058 | Lajedo do Tabocal | 8.999 |
| BA | 29 | 19108 | Lamarão | 12.995 |
| BA | 29 | 19157 | Lapão | 26.616 |
| BA | 29 | 19207 | Lauro de Freitas | 156.936 |
| BA | 29 | 19306 | Lençóis | 10.112 |
| BA | 29 | 19405 | Licínio de Almeida | 13.205 |
| BA | 29 | 19504 | Livramento de Nossa Senhora | 44.568 |
| BA | 29 | 19553 | Luís Eduardo Magalhães | 52.054 |
| BA | 29 | 19603 | Macajuba | 11.517 |
| BA | 29 | 19702 | Macarani | 16.940 |
| BA | 29 | 19801 | Macaúbas | 48.633 |
| BA | 29 | 19900 | Macururé | 7.834 |
| BA | 29 | 19926 | Madre de Deus | 16.783 |
| BA | 29 | 19959 | Maetinga | 7.884 |
| BA | 29 | 20007 | Maiquinique | 8.848 |
| BA | 29 | 20106 | Mairi | 19.700 |
| BA | 29 | 20205 | Malhada | 16.739 |
| BA | 29 | 20304 | Malhada de Pedras | 7.677 |
| BA | 29 | 20403 | Manoel Vitorino | 14.138 |
| BA | 29 | 20452 | Mansidão | 12.244 |
| BA | 29 | 20502 | Maracás | 35.990 |
| BA | 29 | 20601 | Maragogipe | 43.921 |
| BA | 29 | 20700 | Maraú | 17.270 |
| BA | 29 | 20809 | Marcionílio Souza | 11.061 |
| BA | 29 | 20908 | Mascote | 16.590 |
| BA | 29 | 21005 | Mata de São João | 39.585 |
| BA | 29 | 21054 | Matina | 13.245 |
| BA | 29 | 21104 | Medeiros Neto | 22.752 |
| BA | 29 | 21203 | Miguel Calmon | 27.724 |
| BA | 29 | 21302 | Milagres | 12.092 |
| BA | 29 | 21401 | Mirangaba | 18.849 |
| BA | 29 | 21450 | Mirante | 8.375 |
| BA | 29 | 21500 | Monte Santo | 53.429 |
| BA | 29 | 21609 | Morpará | 8.871 |
| BA | 29 | 21708 | Morro do Chapéu | 35.031 |
| BA | 29 | 21807 | Mortugaba | 14.692 |
| BA | 29 | 21906 | Mucugê | 14.714 |
| BA | 29 | 22003 | Mucuri | 35.501 |
| BA | 29 | 22052 | Mulungu do Morro | 13.879 |
| BA | 29 | 22102 | Mundo Novo | 25.245 |
| BA | 29 | 22201 | Muniz Ferreira | 7.236 |
| BA | 29 | 22250 | Muquém de São Francisco | 10.691 |

| U.F. | | MUNICÍPIOS | | POPULAÇÃO ESTIMADA |
|---|---|---|---|---|
| SIGLA | COD | COD | NOMES | |
| BA | 29 | 22300 | Muritiba | 27.755 |
| BA | 29 | 22409 | Mutuípe | 22.066 |
| BA | 29 | 22508 | Nazaré | 27.427 |
| BA | 29 | 22607 | Nilo Peçanha | 13.274 |
| BA | 29 | 22656 | Nordestina | 12.670 |
| BA | 29 | 22706 | Nova Canaã | 20.311 |
| BA | 29 | 22730 | Nova Fátima | 7.964 |
| BA | 29 | 22755 | Nova Ibiá | 7.029 |
| BA | 29 | 22805 | Nova Itarana | 7.875 |
| BA | 29 | 22854 | Nova Redenção | 9.318 |
| BA | 29 | 22904 | Nova Soure | 26.874 |
| BA | 29 | 23001 | Nova Viçosa | 36.407 |
| BA | 29 | 23035 | Novo Horizonte | 11.092 |
| BA | 29 | 23050 | Novo Triunfo | 14.763 |
| BA | 29 | 23100 | Olindina | 24.557 |
| BA | 29 | 23209 | Oliveira dos Brejinhos | 23.596 |
| BA | 29 | 23308 | Ouriçangas | 8.131 |
| BA | 29 | 23357 | Ourolândia | 17.080 |
| BA | 29 | 23407 | Palmas de Monte Alto | 22.061 |
| BA | 29 | 23506 | Palmeiras | 8.437 |
| BA | 29 | 23605 | Paramirim | 20.870 |
| BA | 29 | 23704 | Paratinga | 29.874 |
| BA | 29 | 23803 | Paripiranga | 29.727 |
| BA | 29 | 23902 | Pau Brasil | 12.342 |
| BA | 29 | 24009 | Paulo Afonso | 106.703 |
| BA | 29 | 24058 | Pé de Serra | 14.788 |
| BA | 29 | 24108 | Pedrão | 7.597 |
| BA | 29 | 24207 | Pedro Alexandre | 17.698 |
| BA | 29 | 24306 | Piatã | 18.470 |
| BA | 29 | 24405 | Pilão Arcado | 34.466 |
| BA | 29 | 24504 | Pindaí | 15.772 |
| BA | 29 | 24603 | Pindobaçu | 20.854 |
| BA | 29 | 24652 | Pintadas | 10.812 |
| BA | 29 | 24678 | Piraí do Norte | 8.818 |
| BA | 29 | 24702 | Piripá | 13.325 |
| BA | 29 | 24801 | Piritiba | 26.265 |
| BA | 29 | 24900 | Planaltino | 8.880 |
| BA | 29 | 25006 | Planalto | 22.151 |
| BA | 29 | 25105 | Poções | 46.390 |
| BA | 29 | 25204 | Pojuca | 32.225 |
| BA | 29 | 25253 | Ponto Novo | 14.734 |
| BA | 29 | 25303 | Porto Seguro | 122.896 |
| BA | 29 | 25402 | Potiraguá | 9.656 |
| BA | 29 | 25501 | Prado | 26.016 |
| BA | 29 | 25600 | Presidente Dutra | 14.306 |
| BA | 29 | 25709 | Presidente Jânio Quadros | 14.212 |
| BA | 29 | 25758 | Presidente Tancredo Neves | 24.249 |
| BA | 29 | 25808 | Queimadas | 28.729 |
| BA | 29 | 25907 | Quijingue | 28.143 |
| BA | 29 | 25931 | Quixabeira | 9.631 |

| U.F. | | MUNICÍPIOS | | POPULAÇÃO ESTIMADA |
|---|---|---|---|---|
| SIGLA | COD | COD | NOMES | |
| BA | 29 | 25956 | Rafael Jambeiro | 24.004 |
| BA | 29 | 26004 | Remanso | 39.705 |
| BA | 29 | 26103 | Retirolândia | 11.798 |
| BA | 29 | 26202 | Riachão das Neves | 23.431 |
| BA | 29 | 26301 | Riachão do Jacuípe | 33.666 |
| BA | 29 | 26400 | Riacho de Santana | 30.602 |
| BA | 29 | 26509 | Ribeira do Amparo | 14.621 |
| BA | 29 | 26608 | Ribeira do Pombal | 49.263 |
| BA | 29 | 26657 | Ribeirão do Largo | 14.528 |
| BA | 29 | 26707 | Rio de Contas | 13.816 |
| BA | 29 | 26806 | Rio do Antônio | 15.110 |
| BA | 29 | 26905 | Rio do Pires | 11.612 |
| BA | 29 | 27002 | Rio Real | 38.095 |
| BA | 29 | 27101 | Rodelas | 7.446 |
| BA | 29 | 27200 | Ruy Barbosa | 30.422 |
| BA | 29 | 27309 | Salinas da Margarida | 14.194 |
| BA | 29 | 27408 | Salvador | 2.998.056 |
| BA | 29 | 27507 | Santa Bárbara | 20.461 |
| BA | 29 | 27606 | Santa Brígida | 15.652 |
| BA | 29 | 27804 | Santa Cruz da Vitória | 6.443 |
| BA | 29 | 27705 | Santa Cruz Cabrália | 26.250 |
| BA | 29 | 27903 | Santa Inês | 10.803 |
| BA | 29 | 28059 | Santa Luzia | 15.613 |
| BA | 29 | 28109 | Santa Maria da Vitória | 41.758 |
| BA | 29 | 28406 | Santa Rita de Cássia | 27.528 |
| BA | 29 | 28505 | Santa Teresinha | 10.546 |
| BA | 29 | 28000 | Santaluz | 35.416 |
| BA | 29 | 28208 | Santana | 27.259 |
| BA | 29 | 28307 | Santanópolis | 9.643 |
| BA | 29 | 28604 | Santo Amaro | 58.387 |
| BA | 29 | 28703 | Santo Antônio de Jesus | 88.768 |
| BA | 29 | 28802 | Santo Estêvão | 46.855 |
| BA | 29 | 28901 | São Desidério | 27.513 |
| BA | 29 | 28950 | São Domingos | 9.730 |
| BA | 29 | 29107 | São Felipe | 20.952 |
| BA | 29 | 29008 | São Félix | 16.208 |
| BA | 29 | 29057 | São Félix do Coribe | 13.504 |
| BA | 29 | 29206 | São Francisco do Conde | 31.699 |
| BA | 29 | 29255 | São Gabriel | 19.099 |
| BA | 29 | 29305 | São Gonçalo dos Campos | 30.724 |
| BA | 29 | 29354 | São José da Vitória | 6.270 |
| BA | 29 | 29370 | São José do Jacuípe | 11.134 |
| BA | 29 | 29404 | São Miguel das Matas | 10.675 |
| BA | 29 | 29503 | São Sebastião do Passé | 41.758 |
| BA | 29 | 29602 | Sapeaçu | 17.087 |
| BA | 29 | 29701 | Sátiro Dias | 18.919 |
| BA | 29 | 29750 | Saubara | 11.632 |
| BA | 29 | 29800 | Saúde | 12.355 |
| BA | 29 | 29909 | Seabra | 42.175 |
| BA | 29 | 30006 | Sebastião Laranjeiras | 11.421 |

| U.F. | | MUNICÍPIOS | | POPULAÇÃO ESTIMADA |
|---|---|---|---|---|
| SIGLA | COD | COD | NOMES | |
| BA | 29 | 30105 | Senhor do Bonfim | 76.113 |
| BA | 29 | 30204 | Sento Sé | 38.735 |
| BA | 29 | 30154 | Serra do Ramalho | 31.809 |
| BA | 29 | 30303 | Serra Dourada | 17.858 |
| BA | 29 | 30402 | Serra Preta | 16.168 |
| BA | 29 | 30501 | Serrinha | 73.859 |
| BA | 29 | 30600 | Serrolândia | 12.665 |
| BA | 29 | 30709 | Simões Filho | 116.662 |
| BA | 29 | 30758 | Sítio do Mato | 13.187 |
| BA | 29 | 30766 | Sítio do Quinto | 13.811 |
| BA | 29 | 30774 | Sobradinho | 22.026 |
| BA | 29 | 30808 | Souto Soares | 19.407 |
| BA | 29 | 30907 | Tabocas do Brejo Velho | 12.608 |
| BA | 29 | 31004 | Tanhaçu | 20.134 |
| BA | 29 | 31053 | Tanque Novo | 16.466 |
| BA | 29 | 31103 | Tanquinho | 7.875 |
| BA | 29 | 31202 | Taperoá | 19.388 |
| BA | 29 | 31301 | Tapiramutá | 17.879 |
| BA | 29 | 31350 | Teixeira de Freitas | 125.430 |
| BA | 29 | 31400 | Teodoro Sampaio | 8.442 |
| BA | 29 | 31509 | Teofilândia | 21.461 |
| BA | 29 | 31608 | Teolândia | 12.810 |
| BA | 29 | 31707 | Terra Nova | 12.583 |
| BA | 29 | 31806 | Tremedal | 18.433 |
| BA | 29 | 31905 | Tucano | 49.827 |
| BA | 29 | 32002 | Uauá | 25.159 |
| BA | 29 | 32101 | Ubaíra | 21.428 |
| BA | 29 | 32200 | Ubaitaba | 20.333 |
| BA | 29 | 32309 | Ubatã | 26.355 |
| BA | 29 | 32408 | Uibaí | 14.203 |
| BA | 29 | 32457 | Umburanas | 17.096 |
| BA | 29 | 32507 | Una | 24.650 |
| BA | 29 | 32606 | Urandi | 16.289 |
| BA | 29 | 32705 | Uruçuca | 23.237 |
| BA | 29 | 32804 | Utinga | 20.448 |
| BA | 29 | 32903 | Valença | 89.597 |
| BA | 29 | 33000 | Valente | 22.295 |
| BA | 29 | 33059 | Várzea da Roça | 15.171 |
| BA | 29 | 33109 | Várzea do Poço | 8.992 |
| BA | 29 | 33158 | Várzea Nova | 14.365 |
| BA | 29 | 33174 | Varzedo | 9.451 |
| BA | 29 | 33208 | Vera Cruz | 37.539 |
| BA | 29 | 33257 | Vereda | 7.346 |
| BA | 29 | 33307 | Vitória da Conquista | 318.901 |
| BA | 29 | 33406 | Wagner | 8.807 |
| BA | 29 | 33455 | Wanderley | 13.252 |
| BA | 29 | 33505 | Wenceslau Guimarães | 24.802 |
| BA | 29 | 33604 | Xique-Xique | 47.470 |
| MG | 31 | 104 | Abadia dos Dourados | 6.805 |
| MG | 31 | 203 | Abaeté | 23.258 |

| U.F. | | MUNICÍPIOS | | POPULAÇÃO ESTIMADA |
|---|---|---|---|---|
| SIGLA | COD | COD | NOMES | |
| MG | 31 | 302 | Abre Campo | 13.177 |
| MG | 31 | 401 | Acaiaca | 4.234 |
| MG | 31 | 500 | Açucena | 11.409 |
| MG | 31 | 609 | Água Boa | 16.643 |
| MG | 31 | 708 | Água Comprida | 2.164 |
| MG | 31 | 807 | Aguanil | 4.315 |
| MG | 31 | 906 | Águas Formosas | 19.310 |
| MG | 31 | 1003 | Águas Vermelhas | 13.301 |
| MG | 31 | 1102 | Aimorés | 24.825 |
| MG | 31 | 1201 | Aiuruoca | 6.210 |
| MG | 31 | 1300 | Alagoa | 2.926 |
| MG | 31 | 1409 | Albertina | 2.976 |
| MG | 31 | 1508 | Além Paraíba | 34.591 |
| MG | 31 | 1607 | Alfenas | 75.214 |
| MG | 31 | 1631 | Alfredo Vasconcelos | 6.300 |
| MG | 31 | 1706 | Almenara | 38.531 |
| MG | 31 | 1805 | Alpercata | 7.252 |
| MG | 31 | 1904 | Alpinópolis | 18.619 |
| MG | 31 | 2001 | Alterosa | 13.810 |
| MG | 31 | 2050 | Alto Caparaó | 5.312 |
| MG | 31 | 53509 | Alto Jequitibá | 8.122 |
| MG | 31 | 2100 | Alto Rio Doce | 12.778 |
| MG | 31 | 2209 | Alvarenga | 4.545 |
| MG | 31 | 2308 | Alvinópolis | 15.678 |
| MG | 31 | 2407 | Alvorada de Minas | 3.588 |
| MG | 31 | 2506 | Amparo do Serra | 5.362 |
| MG | 31 | 2605 | Andradas | 36.633 |
| MG | 31 | 2803 | Andrelândia | 12.369 |
| MG | 31 | 2852 | Angelândia | 8.571 |
| MG | 31 | 2902 | Antônio Carlos | 11.624 |
| MG | 31 | 3009 | Antônio Dias | 9.598 |
| MG | 31 | 3108 | Antônio Prado de Minas | 2.070 |
| MG | 31 | 3207 | Araçaí | 2.524 |
| MG | 31 | 3306 | Aracitaba | 1.885 |
| MG | 31 | 3405 | Araçuaí | 37.388 |
| MG | 31 | 3504 | Araguari | 111.095 |
| MG | 31 | 3603 | Arantina | 2.538 |
| MG | 31 | 3702 | Araponga | 8.328 |
| MG | 31 | 3751 | Araporã | 6.522 |
| MG | 31 | 3801 | Arapuá | 2.778 |
| MG | 31 | 3900 | Araújos | 7.692 |
| MG | 31 | 4007 | Araxá | 92.927 |
| MG | 31 | 4106 | Arceburgo | 8.253 |
| MG | 31 | 4205 | Arcos | 36.455 |
| MG | 31 | 4304 | Areado | 13.864 |
| MG | 31 | 4403 | Argirita | 3.051 |
| MG | 31 | 4452 | Aricanduva | 5.140 |
| MG | 31 | 4502 | Arinos | 18.153 |
| MG | 31 | 4601 | Astolfo Dutra | 13.109 |
| MG | 31 | 4700 | Ataléia | 15.162 |

| U.F. | | MUNICÍPIOS | | POPULAÇÃO ESTIMADA |
|---|---|---|---|---|
| SIGLA | COD | COD | NOMES | |
| MG | 31 | 4809 | Augusto de Lima | 4.599 |
| MG | 31 | 4908 | Baependi | 18.745 |
| MG | 31 | 5004 | Baldim | 8.582 |
| MG | 31 | 5103 | Bambuí | 22.622 |
| MG | 31 | 5202 | Bandeira | 5.521 |
| MG | 31 | 5301 | Bandeira do Sul | 5.330 |
| MG | 31 | 5400 | Barão de Cocais | 28.074 |
| MG | 31 | 5509 | Barão de Monte Alto | 5.700 |
| MG | 31 | 5608 | Barbacena | 128.572 |
| MG | 31 | 5707 | Barra Longa | 7.050 |
| MG | 31 | 5905 | Barroso | 20.253 |
| MG | 31 | 6002 | Bela Vista de Minas | 10.333 |
| MG | 31 | 6101 | Belmiro Braga | 3.079 |
| MG | 31 | 6200 | Belo Horizonte | 2.452.617 |
| MG | 31 | 6309 | Belo Oriente | 22.555 |
| MG | 31 | 6408 | Belo Vale | 7.470 |
| MG | 31 | 6507 | Berilo | 13.717 |
| MG | 31 | 6655 | Berizal | 4.655 |
| MG | 31 | 6606 | Bertópolis | 4.780 |
| MG | 31 | 6705 | Betim | 441.748 |
| MG | 31 | 6804 | Bias Fortes | 3.881 |
| MG | 31 | 6903 | Bicas | 14.309 |
| MG | 31 | 7000 | Biquinhas | 2.621 |
| MG | 31 | 7109 | Boa Esperança | 39.254 |
| MG | 31 | 7208 | Bocaina de Minas | 5.216 |
| MG | 31 | 7307 | Bocaiúva | 46.624 |
| MG | 31 | 7406 | Bom Despacho | 44.265 |
| MG | 31 | 7505 | Bom Jardim de Minas | 6.657 |
| MG | 31 | 7604 | Bom Jesus da Penha | 3.980 |
| MG | 31 | 7703 | Bom Jesus do Amparo | 5.744 |
| MG | 31 | 7802 | Bom Jesus do Galho | 15.462 |
| MG | 31 | 7901 | Bom Repouso | 10.826 |
| MG | 31 | 8008 | Bom Sucesso | 17.805 |
| MG | 31 | 8107 | Bonfim | 6.902 |
| MG | 31 | 8206 | Bonfinópolis de Minas | 5.869 |
| MG | 31 | 8255 | Bonito de Minas | 9.315 |
| MG | 31 | 8305 | Borda da Mata | 15.507 |
| MG | 31 | 8404 | Botelhos | 15.289 |
| MG | 31 | 8503 | Botumirim | 6.550 |
| MG | 31 | 8701 | Brás Pires | 4.615 |
| MG | 31 | 8552 | Brasilândia de Minas | 13.593 |
| MG | 31 | 8602 | Brasília de Minas | 32.438 |
| MG | 31 | 8909 | Brasópolis | 14.756 |
| MG | 31 | 8800 | Braúnas | 5.332 |
| MG | 31 | 9006 | Brumadinho | 34.391 |
| MG | 31 | 9105 | Bueno Brandão | 11.212 |
| MG | 31 | 9204 | Buenópolis | 9.627 |
| MG | 31 | 9253 | Bugre | 4.095 |
| MG | 31 | 9303 | Buritis | 22.465 |
| MG | 31 | 9402 | Buritizeiro | 27.068 |

| U.F. | | MUNICÍPIOS | | POPULAÇÃO ESTIMADA |
|---|---|---|---|---|
| SIGLA | COD | COD | NOMES | |
| MG | 31 | 9451 | Cabeceira Grande | 6.600 |
| MG | 31 | 9501 | Cabo Verde | 14.042 |
| MG | 31 | 9600 | Cachoeira da Prata | 3.936 |
| MG | 31 | 9709 | Cachoeira de Minas | 11.250 |
| MG | 31 | 2704 | Cachoeira de Pajeú | 9.537 |
| MG | 31 | 9808 | Cachoeira Dourada | 2.595 |
| MG | 31 | 9907 | Caetanópolis | 10.040 |
| MG | 31 | 10004 | Caeté | 41.043 |
| MG | 31 | 10103 | Caiana | 4.733 |
| MG | 31 | 10202 | Cajuri | 4.106 |
| MG | 31 | 10301 | Caldas | 14.655 |
| MG | 31 | 10400 | Camacho | 3.229 |
| MG | 31 | 10509 | Camanducaia | 20.160 |
| MG | 31 | 10608 | Cambuí | 26.365 |
| MG | 31 | 10707 | Cambuquira | 12.936 |
| MG | 31 | 10806 | Campanário | 3.757 |
| MG | 31 | 10905 | Campanha | 15.949 |
| MG | 31 | 11002 | Campestre | 20.854 |
| MG | 31 | 11101 | Campina Verde | 19.201 |
| MG | 31 | 11309 | Campo do Meio | 11.871 |
| MG | 31 | 11150 | Campo Azul | 4.020 |
| MG | 31 | 11200 | Campo Belo | 53.653 |
| MG | 31 | 11408 | Campo Florido | 7.105 |
| MG | 31 | 11507 | Campos Altos | 13.719 |
| MG | 31 | 11606 | Campos Gerais | 27.964 |
| MG | 31 | 11903 | Cana Verde | 5.915 |
| MG | 31 | 11705 | Canaã | 4.795 |
| MG | 31 | 11804 | Canápolis | 11.865 |
| MG | 31 | 12000 | Candeias | 16.281 |
| MG | 31 | 12059 | Cantagalo | 4.132 |
| MG | 31 | 12109 | Caparaó | 4.991 |
| MG | 31 | 12208 | Capela Nova | 4.661 |
| MG | 31 | 12307 | Capelinha | 34.634 |
| MG | 31 | 12406 | Capetinga | 7.326 |
| MG | 31 | 12505 | Capim Branco | 9.276 |
| MG | 31 | 12604 | Capinópolis | 16.043 |
| MG | 31 | 12653 | Capitão Andrade | 5.087 |
| MG | 31 | 12703 | Capitão Enéas | 14.830 |
| MG | 31 | 12802 | Capitólio | 7.864 |
| MG | 31 | 12901 | Caputira | 9.157 |
| MG | 31 | 13008 | Caraí | 22.392 |
| MG | 31 | 13107 | Caranaíba | 3.553 |
| MG | 31 | 13206 | Carandaí | 23.286 |
| MG | 31 | 13305 | Carangola | 33.182 |
| MG | 31 | 13404 | Caratinga | 85.472 |
| MG | 31 | 13503 | Carbonita | 10.783 |
| MG | 31 | 13602 | Careaçu | 6.286 |
| MG | 31 | 13701 | Carlos Chagas | 21.212 |
| MG | 31 | 13800 | Carmésia | 2.712 |
| MG | 31 | 13909 | Carmo da Cachoeira | 12.061 |

| U.F. | | MUNICÍPIOS | | POPULAÇÃO ESTIMADA |
|---|---|---|---|---|
| SIGLA | COD | COD | NOMES | |
| MG | 31 | 14006 | Carmo da Mata | 11.446 |
| MG | 31 | 14105 | Carmo de Minas | 14.397 |
| MG | 31 | 14204 | Carmo do Cajuru | 20.031 |
| MG | 31 | 14303 | Carmo do Paranaíba | 32.059 |
| MG | 31 | 14402 | Carmo do Rio Claro | 20.070 |
| MG | 31 | 14501 | Carmópolis de Minas | 16.624 |
| MG | 31 | 14550 | Carneirinho | 9.143 |
| MG | 31 | 14600 | Carrancas | 4.182 |
| MG | 31 | 14709 | Carvalhópolis | 3.380 |
| MG | 31 | 14808 | Carvalhos | 4.735 |
| MG | 31 | 14907 | Casa Grande | 2.129 |
| MG | 31 | 15003 | Cascalho Rico | 2.938 |
| MG | 31 | 15102 | Cássia | 17.587 |
| MG | 31 | 15300 | Cataguases | 70.507 |
| MG | 31 | 15359 | Catas Altas | 4.795 |
| MG | 31 | 15409 | Catas Altas da Noruega | 3.573 |
| MG | 31 | 15458 | Catuji | 6.633 |
| MG | 31 | 15474 | Catuti | 5.473 |
| MG | 31 | 15508 | Caxambu | 21.431 |
| MG | 31 | 15607 | Cedro do Abaeté | 1.221 |
| MG | 31 | 15706 | Central de Minas | 6.821 |
| MG | 31 | 15805 | Centralina | 10.557 |
| MG | 31 | 15904 | Chácara | 2.762 |
| MG | 31 | 16001 | Chalé | 5.598 |
| MG | 31 | 16100 | Chapada do Norte | 16.025 |
| MG | 31 | 16159 | Chapada Gaúcha | 11.368 |
| MG | 31 | 16209 | Chiador | 2.974 |
| MG | 31 | 16308 | Cipotânea | 6.807 |
| MG | 31 | 16407 | Claraval | 4.453 |
| MG | 31 | 16506 | Claro dos Poções | 8.389 |
| MG | 31 | 16605 | Cláudio | 25.938 |
| MG | 31 | 16704 | Coimbra | 7.209 |
| MG | 31 | 16803 | Coluna | 9.570 |
| MG | 31 | 16902 | Comendador Gomes | 3.252 |
| MG | 31 | 17009 | Comercinho | 8.637 |
| MG | 31 | 17108 | Conceição da Aparecida | 10.771 |
| MG | 31 | 15201 | Conceição da Barra de Minas | 4.077 |
| MG | 31 | 17306 | Conceição das Alagoas | 21.938 |
| MG | 31 | 17207 | Conceição das Pedras | 2.821 |
| MG | 31 | 17405 | Conceição de Ipanema | 4.549 |
| MG | 31 | 17504 | Conceição do Mato Dentro | 18.534 |
| MG | 31 | 17603 | Conceição do Pará | 4.866 |
| MG | 31 | 17702 | Conceição do Rio Verde | 13.244 |
| MG | 31 | 17801 | Conceição dos Ouros | 10.869 |
| MG | 31 | 17836 | Cônego Marinho | 6.440 |
| MG | 31 | 17876 | Confins | 6.072 |
| MG | 31 | 17900 | Congonhal | 10.261 |
| MG | 31 | 18007 | Congonhas | 48.723 |
| MG | 31 | 18106 | Congonhas do Norte | 5.335 |
| MG | 31 | 18205 | Conquista | 6.922 |

| U.F. | | MUNICÍPIOS | | POPULAÇÃO |
|---|---|---|---|---|
| SIGLA | COD | COD | NOMES | ESTIMADA |
| MG | 31 | 18304 | Conselheiro Lafaiete | 114.579 |
| MG | 31 | 18403 | Conselheiro Pena | 22.539 |
| MG | 31 | 18502 | Consolação | 1.750 |
| MG | 31 | 18601 | Contagem | 625.393 |
| MG | 31 | 18700 | Coqueiral | 9.747 |
| MG | 31 | 18809 | Coração de Jesus | 27.110 |
| MG | 31 | 18908 | Cordisburgo | 9.465 |
| MG | 31 | 19005 | Cordislândia | 3.743 |
| MG | 31 | 19104 | Corinto | 23.048 |
| MG | 31 | 19203 | Coroaci | 11.131 |
| MG | 31 | 19302 | Coromandel | 28.296 |
| MG | 31 | 19401 | Coronel Fabriciano | 105.037 |
| MG | 31 | 19500 | Coronel Murta | 9.423 |
| MG | 31 | 19609 | Coronel Pacheco | 2.427 |
| MG | 31 | 19708 | Coronel Xavier Chaves | 3.303 |
| MG | 31 | 19906 | Córrego do Bom Jesus | 3.824 |
| MG | 31 | 19807 | Córrego Danta | 3.475 |
| MG | 31 | 19955 | Córrego Fundo | 5.939 |
| MG | 31 | 20003 | Córrego Novo | 3.138 |
| MG | 31 | 20102 | Couto de Magalhães de Minas | 4.560 |
| MG | 31 | 20151 | Crisólita | 5.941 |
| MG | 31 | 20201 | Cristais | 11.269 |
| MG | 31 | 20300 | Cristália | 5.961 |
| MG | 31 | 20409 | Cristiano Otoni | 5.039 |
| MG | 31 | 20508 | Cristina | 11.478 |
| MG | 31 | 20607 | Crucilândia | 4.776 |
| MG | 31 | 20706 | Cruzeiro da Fortaleza | 3.897 |
| MG | 31 | 20805 | Cruzília | 15.373 |
| MG | 31 | 20839 | Cuparaque | 4.561 |
| MG | 31 | 20870 | Curral de Dentro | 7.376 |
| MG | 31 | 20904 | Curvelo | 75.051 |
| MG | 31 | 21001 | Datas | 5.696 |
| MG | 31 | 21100 | Delfim Moreira | 8.047 |
| MG | 31 | 21209 | Delfinópolis | 6.954 |
| MG | 31 | 21258 | Delta | 7.210 |
| MG | 31 | 21308 | Descoberto | 5.126 |
| MG | 31 | 21407 | Desterro de Entre Rios | 7.173 |
| MG | 31 | 21506 | Desterro do Melo | 3.302 |
| MG | 31 | 21605 | Diamantina | 46.372 |
| MG | 31 | 21704 | Diogo de Vasconcelos | 4.066 |
| MG | 31 | 21803 | Dionísio | 10.589 |
| MG | 31 | 21902 | Divinésia | 3.409 |
| MG | 31 | 22009 | Divino | 20.099 |
| MG | 31 | 22108 | Divino das Laranjeiras | 5.092 |
| MG | 31 | 22207 | Divinolândia de Minas | 7.023 |
| MG | 31 | 22306 | Divinópolis | 216.099 |
| MG | 31 | 22355 | Divisa Alegre | 6.235 |
| MG | 31 | 22405 | Divisa Nova | 5.828 |
| MG | 31 | 22454 | Divisópolis | 8.463 |
| MG | 31 | 22470 | Dom Bosco | 3.839 |

| U.F. | | MUNICÍPIOS | | POPULAÇÃO ESTIMADA |
|---|---|---|---|---|
| SIGLA | COD | COD | NOMES | |
| MG | 31 | 22504 | Dom Cavati | 5.811 |
| MG | 31 | 22603 | Dom Joaquim | 4.640 |
| MG | 31 | 22702 | Dom Silvério | 5.475 |
| MG | 31 | 22801 | Dom Viçoso | 3.117 |
| MG | 31 | 22900 | Dona Eusébia | 5.808 |
| MG | 31 | 23007 | Dores de Campos | 9.821 |
| MG | 31 | 23106 | Dores de Guanhães | 5.751 |
| MG | 31 | 23205 | Dores do Indaiá | 14.366 |
| MG | 31 | 23304 | Dores do Turvo | 4.668 |
| MG | 31 | 23403 | Doresópolis | 1.578 |
| MG | 31 | 23502 | Douradoquara | 1.924 |
| MG | 31 | 23528 | Durandé | 7.146 |
| MG | 31 | 23601 | Elói Mendes | 25.532 |
| MG | 31 | 23700 | Engenheiro Caldas | 10.908 |
| MG | 31 | 23809 | Engenheiro Navarro | 7.315 |
| MG | 31 | 23858 | Entre Folhas | 5.065 |
| MG | 31 | 23908 | Entre Rios de Minas | 14.548 |
| MG | 31 | 24005 | Ervália | 18.855 |
| MG | 31 | 24104 | Esmeraldas | 59.408 |
| MG | 31 | 24203 | Espera Feliz | 21.612 |
| MG | 31 | 24302 | Espinosa | 32.461 |
| MG | 31 | 24401 | Espírito Santo do Dourado | 4.471 |
| MG | 31 | 24500 | Estiva | 11.426 |
| MG | 31 | 24708 | Estrela do Indaiá | 3.787 |
| MG | 31 | 24807 | Estrela do Sul | 7.439 |
| MG | 31 | 24609 | Estrela Dalva | 2.536 |
| MG | 31 | 24906 | Eugenópolis | 10.769 |
| MG | 31 | 25002 | Ewbank da Câmara | 3.676 |
| MG | 31 | 25101 | Extrema | 27.155 |
| MG | 31 | 25200 | Fama | 2.259 |
| MG | 31 | 25309 | Faria Lemos | 3.734 |
| MG | 31 | 25408 | Felício dos Santos | 5.865 |
| MG | 31 | 25606 | Felisburgo | 7.024 |
| MG | 31 | 25705 | Felixlândia | 14.287 |
| MG | 31 | 25804 | Fernandes Tourinho | 2.713 |
| MG | 31 | 25903 | Ferros | 11.531 |
| MG | 31 | 25952 | Fervedouro | 10.754 |
| MG | 31 | 26000 | Florestal | 6.199 |
| MG | 31 | 26109 | Formiga | 67.178 |
| MG | 31 | 26208 | Formoso | 6.857 |
| MG | 31 | 26307 | Fortaleza de Minas | 3.986 |
| MG | 31 | 26406 | Fortuna de Minas | 2.541 |
| MG | 31 | 26505 | Francisco Badaró | 10.604 |
| MG | 31 | 26604 | Francisco Dumont | 4.987 |
| MG | 31 | 26703 | Francisco Sá | 25.994 |
| MG | 31 | 26752 | Franciscópolis | 5.662 |
| MG | 31 | 26802 | Frei Gaspar | 6.649 |
| MG | 31 | 26901 | Frei Inocêncio | 9.347 |
| MG | 31 | 26950 | Frei Lagonegro | 3.493 |
| MG | 31 | 27008 | Fronteira | 15.706 |

| U.F. | | MUNICÍPIOS | | POPULAÇÃO ESTIMADA |
|---|---|---|---|---|
| SIGLA | COD | COD | NOMES | |
| MG | 31 | 27057 | Fronteira dos Vales | 4.980 |
| MG | 31 | 27073 | Fruta de Leite | 6.426 |
| MG | 31 | 27107 | Frutal | 54.819 |
| MG | 31 | 27206 | Funilândia | 3.852 |
| MG | 31 | 27305 | Galiléia | 7.563 |
| MG | 31 | 27339 | Gameleiras | 5.392 |
| MG | 31 | 27354 | Glaucilândia | 3.072 |
| MG | 31 | 27370 | Goiabeira | 3.239 |
| MG | 31 | 27388 | Goianá | 3.846 |
| MG | 31 | 27404 | Gonçalves | 4.450 |
| MG | 31 | 27503 | Gonzaga | 5.786 |
| MG | 31 | 27602 | Gouveia | 11.927 |
| MG | 31 | 27701 | Governador Valadares | 263.274 |
| MG | 31 | 27800 | Grão Mogol | 15.177 |
| MG | 31 | 27909 | Grupiara | 1.468 |
| MG | 31 | 28006 | Guanhães | 30.638 |
| MG | 31 | 28105 | Guapé | 13.475 |
| MG | 31 | 28204 | Guaraciaba | 10.820 |
| MG | 31 | 28253 | Guaraciama | 4.728 |
| MG | 31 | 28303 | Guaranésia | 18.635 |
| MG | 31 | 28402 | Guarani | 10.049 |
| MG | 31 | 28501 | Guarará | 4.114 |
| MG | 31 | 28600 | Guarda-Mor | 6.778 |
| MG | 31 | 28709 | Guaxupé | 49.719 |
| MG | 31 | 28808 | Guidoval | 7.523 |
| MG | 31 | 28907 | Guimarânia | 7.322 |
| MG | 31 | 29004 | Guiricema | 9.116 |
| MG | 31 | 29103 | Gurinhatã | 6.228 |
| MG | 31 | 29202 | Heliodora | 6.295 |
| MG | 31 | 29301 | Iapu | 11.501 |
| MG | 31 | 29400 | Ibertioga | 5.206 |
| MG | 31 | 29509 | Ibiá | 23.069 |
| MG | 31 | 29608 | Ibiaí | 7.907 |
| MG | 31 | 29657 | Ibiracatu | 5.936 |
| MG | 31 | 29707 | Ibiraci | 11.593 |
| MG | 31 | 29806 | Ibirité | 157.438 |
| MG | 31 | 29905 | Ibitiúra de Minas | 3.516 |
| MG | 31 | 30002 | Ibituruna | 2.938 |
| MG | 31 | 30051 | Icaraí de Minas | 10.934 |
| MG | 31 | 30101 | Igarapé | 33.773 |
| MG | 31 | 30200 | Igaratinga | 9.045 |
| MG | 31 | 30309 | Iguatama | 7.727 |
| MG | 31 | 30408 | Ijaci | 6.036 |
| MG | 31 | 30507 | Ilicínea | 11.828 |
| MG | 31 | 30556 | Imbé de Minas | 6.641 |
| MG | 31 | 30606 | Inconfidentes | 7.692 |
| MG | 31 | 30655 | Indaiabira | 7.748 |
| MG | 31 | 30705 | Indianópolis | 6.671 |
| MG | 31 | 30804 | Ingaí | 2.580 |
| MG | 31 | 30903 | Inhapim | 24.952 |

| U.F. | | MUNICÍPIOS | | POPULAÇÃO ESTIMADA |
|---|---|---|---|---|
| SIGLA | COD | COD | NOMES | |
| MG | 31 | 31000 | Inhaúma | 5.565 |
| MG | 31 | 31109 | Inimutaba | 6.713 |
| MG | 31 | 31158 | Ipaba | 15.422 |
| MG | 31 | 31208 | Ipanema | 17.916 |
| MG | 31 | 31307 | Ipatinga | 244.508 |
| MG | 31 | 31406 | Ipiaçu | 4.374 |
| MG | 31 | 31505 | Ipuiúna | 9.549 |
| MG | 31 | 31604 | Iraí de Minas | 6.605 |
| MG | 31 | 31703 | Itabira | 110.419 |
| MG | 31 | 31802 | Itabirinha | 10.819 |
| MG | 31 | 31901 | Itabirito | 43.832 |
| MG | 31 | 32008 | Itacambira | 5.303 |
| MG | 31 | 32107 | Itacarambi | 18.261 |
| MG | 31 | 32206 | Itaguara | 12.956 |
| MG | 31 | 32305 | Itaipé | 12.072 |
| MG | 31 | 32404 | Itajubá | 90.225 |
| MG | 31 | 32503 | Itamarandiba | 33.581 |
| MG | 31 | 32602 | Itamarati de Minas | 4.232 |
| MG | 31 | 32701 | Itambacuri | 23.386 |
| MG | 31 | 32800 | Itambé do Mato Dentro | 2.479 |
| MG | 31 | 32909 | Itamogi | 11.218 |
| MG | 31 | 33006 | Itamonte | 14.611 |
| MG | 31 | 33105 | Itanhandu | 15.253 |
| MG | 31 | 33204 | Itanhomi | 12.357 |
| MG | 31 | 33303 | Itaobim | 21.618 |
| MG | 31 | 33402 | Itapagipe | 15.043 |
| MG | 31 | 33501 | Itapecerica | 21.200 |
| MG | 31 | 33600 | Itapeva | 8.073 |
| MG | 31 | 33709 | Itatiaiuçu | 9.364 |
| MG | 31 | 33758 | Itaú de Minas | 15.257 |
| MG | 31 | 33808 | Itaúna | 85.838 |
| MG | 31 | 33907 | Itaverava | 5.749 |
| MG | 31 | 34004 | Itinga | 15.252 |
| MG | 31 | 34103 | Itueta | 6.074 |
| MG | 31 | 34202 | Ituiutaba | 96.759 |
| MG | 31 | 34301 | Itumirim | 6.667 |
| MG | 31 | 34400 | Iturama | 33.231 |
| MG | 31 | 34509 | Itutinga | 4.165 |
| MG | 31 | 34608 | Jaboticatubas | 16.513 |
| MG | 31 | 34707 | Jacinto | 12.923 |
| MG | 31 | 34806 | Jacuí | 7.426 |
| MG | 31 | 34905 | Jacutinga | 21.424 |
| MG | 31 | 35001 | Jaguaraçu | 2.857 |
| MG | 31 | 35050 | Jaíba | 32.190 |
| MG | 31 | 35076 | Jampruca | 5.145 |
| MG | 31 | 35100 | Janaúba | 68.527 |
| MG | 31 | 35209 | Januária | 67.516 |
| MG | 31 | 35308 | Japaraíba | 3.866 |
| MG | 31 | 35357 | Japonvar | 8.536 |
| MG | 31 | 35407 | Jeceaba | 6.036 |

| U.F. | | MUNICÍPIOS | | POPULAÇÃO ESTIMADA |
|---|---|---|---|---|
| SIGLA | COD | COD | NOMES | |
| MG | 31 | 35456 | Jenipapo de Minas | 7.242 |
| MG | 31 | 35506 | Jequeri | 13.225 |
| MG | 31 | 35605 | Jequitaí | 8.117 |
| MG | 31 | 35704 | Jequitibá | 5.756 |
| MG | 31 | 35803 | Jequitinhonha | 25.060 |
| MG | 31 | 35902 | Jesuânia | 4.983 |
| MG | 31 | 36009 | Joaíma | 15.464 |
| MG | 31 | 36108 | Joanésia | 5.567 |
| MG | 31 | 36207 | João Monlevade | 75.320 |
| MG | 31 | 36306 | João Pinheiro | 45.150 |
| MG | 31 | 36405 | Joaquim Felício | 4.086 |
| MG | 31 | 36504 | Jordânia | 11.337 |
| MG | 31 | 36520 | José Gonçalves de Minas | 4.662 |
| MG | 31 | 36553 | José Raydan | 4.412 |
| MG | 31 | 36579 | Josenópolis | 4.636 |
| MG | 31 | 36652 | Juatuba | 20.978 |
| MG | 31 | 36702 | Juiz de Fora | 526.706 |
| MG | 31 | 36801 | Juramento | 4.108 |
| MG | 31 | 36900 | Juruaia | 8.684 |
| MG | 31 | 36959 | Juvenília | 6.321 |
| MG | 31 | 37007 | Ladainha | 17.195 |
| MG | 31 | 37106 | Lagamar | 7.873 |
| MG | 31 | 37205 | Lagoa da Prata | 47.007 |
| MG | 31 | 37304 | Lagoa dos Patos | 4.596 |
| MG | 31 | 37403 | Lagoa Dourada | 12.265 |
| MG | 31 | 37502 | Lagoa Formosa | 17.134 |
| MG | 31 | 37536 | Lagoa Grande | 9.216 |
| MG | 31 | 37601 | Lagoa Santa | 48.213 |
| MG | 31 | 37700 | Lajinha | 17.678 |
| MG | 31 | 37809 | Lambari | 19.244 |
| MG | 31 | 37908 | Lamim | 3.654 |
| MG | 31 | 38005 | Laranjal | 6.568 |
| MG | 31 | 38104 | Lassance | 6.651 |
| MG | 31 | 38203 | Lavras | 92.542 |
| MG | 31 | 38302 | Leandro Ferreira | 2.986 |
| MG | 31 | 38351 | Leme do Prado | 5.145 |
| MG | 31 | 38401 | Leopoldina | 51.545 |
| MG | 31 | 38500 | Liberdade | 5.397 |
| MG | 31 | 38609 | Lima Duarte | 16.494 |
| MG | 31 | 38625 | Limeira do Oeste | 6.792 |
| MG | 31 | 38658 | Lontra | 8.332 |
| MG | 31 | 38674 | Luisburgo | 6.482 |
| MG | 31 | 38682 | Luislândia | 6.727 |
| MG | 31 | 38708 | Luminárias | 5.527 |
| MG | 31 | 38807 | Luz | 17.835 |
| MG | 31 | 38906 | Machacalis | 7.069 |
| MG | 31 | 39003 | Machado | 39.509 |
| MG | 31 | 39102 | Madre de Deus de Minas | 5.173 |
| MG | 31 | 39201 | Malacacheta | 18.181 |
| MG | 31 | 39250 | Mamonas | 6.485 |

| U.F. | | MUNICÍPIOS | | POPULAÇÃO ESTIMADA |
|---|---|---|---|---|
| SIGLA | COD | COD | NOMES | |
| MG | 31 | 39300 | Manga | 21.338 |
| MG | 31 | 39409 | Manhuaçu | 78.605 |
| MG | 31 | 39508 | Manhumirim | 20.934 |
| MG | 31 | 39607 | Mantena | 27.580 |
| MG | 31 | 39805 | Mar de Espanha | 11.658 |
| MG | 31 | 39706 | Maravilhas | 7.224 |
| MG | 31 | 39904 | Maria da Fé | 14.637 |
| MG | 31 | 40001 | Mariana | 54.689 |
| MG | 31 | 40100 | Marilac | 4.394 |
| MG | 31 | 40159 | Mário Campos | 12.029 |
| MG | 31 | 40209 | Maripá de Minas | 2.981 |
| MG | 31 | 40308 | Marliéria | 3.793 |
| MG | 31 | 40407 | Marmelópolis | 3.156 |
| MG | 31 | 40506 | Martinho Campos | 12.662 |
| MG | 31 | 40530 | Martins Soares | 6.715 |
| MG | 31 | 40555 | Mata Verde | 7.802 |
| MG | 31 | 40605 | Materlândia | 4.772 |
| MG | 31 | 40704 | Mateus Leme | 26.862 |
| MG | 31 | 71501 | Mathias Lobato | 3.526 |
| MG | 31 | 40803 | Matias Barbosa | 13.872 |
| MG | 31 | 40852 | Matias Cardoso | 11.037 |
| MG | 31 | 40902 | Matipó | 17.017 |
| MG | 31 | 41009 | Mato Verde | 12.957 |
| MG | 31 | 41108 | Matozinhos | 35.233 |
| MG | 31 | 41207 | Matutina | 3.789 |
| MG | 31 | 41306 | Medeiros | 3.398 |
| MG | 31 | 41405 | Medina | 21.115 |
| MG | 31 | 41504 | Mendes Pimentel | 6.684 |
| MG | 31 | 41603 | Mercês | 10.902 |
| MG | 31 | 41702 | Mesquita | 6.641 |
| MG | 31 | 41801 | Minas Novas | 31.647 |
| MG | 31 | 41900 | Minduri | 3.665 |
| MG | 31 | 42007 | Mirabela | 13.252 |
| MG | 31 | 42106 | Miradouro | 10.648 |
| MG | 31 | 42205 | Miraí | 13.502 |
| MG | 31 | 42254 | Miravânia | 4.998 |
| MG | 31 | 42304 | Moeda | 4.667 |
| MG | 31 | 42403 | Moema | 7.041 |
| MG | 31 | 42502 | Monjolos | 2.311 |
| MG | 31 | 42601 | Monsenhor Paulo | 7.582 |
| MG | 31 | 42700 | Montalvânia | 16.135 |
| MG | 31 | 42809 | Monte Alegre de Minas | 19.051 |
| MG | 31 | 42908 | Monte Azul | 22.838 |
| MG | 31 | 43005 | Monte Belo | 12.852 |
| MG | 31 | 43104 | Monte Carmelo | 45.975 |
| MG | 31 | 43153 | Monte Formoso | 4.943 |
| MG | 31 | 43203 | Monte Santo de Minas | 20.536 |
| MG | 31 | 43401 | Monte Sião | 20.135 |
| MG | 31 | 43302 | Montes Claros | 363.227 |
| MG | 31 | 43450 | Montezuma | 7.677 |

| U.F. | | MUNICÍPIOS | | POPULAÇÃO ESTIMADA |
|---|---|---|---|---|
| SIGLA | COD | COD | NOMES | |
| MG | 31 | 43500 | Morada Nova de Minas | 8.750 |
| MG | 31 | 43609 | Morro da Garça | 2.966 |
| MG | 31 | 43708 | Morro do Pilar | 3.524 |
| MG | 31 | 43807 | Munhoz | 6.419 |
| MG | 31 | 43906 | Muriaé | 99.628 |
| MG | 31 | 44003 | Mutum | 27.123 |
| MG | 31 | 44102 | Muzambinho | 20.426 |
| MG | 31 | 44201 | Nacip Raydan | 3.015 |
| MG | 31 | 44300 | Nanuque | 41.329 |
| MG | 31 | 44359 | Naque | 6.154 |
| MG | 31 | 44375 | Natalândia | 3.376 |
| MG | 31 | 44409 | Natércia | 4.773 |
| MG | 31 | 44508 | Nazareno | 8.096 |
| MG | 31 | 44607 | Nepomuceno | 25.152 |
| MG | 31 | 44656 | Ninheira | 11.031 |
| MG | 31 | 44672 | Nova Belém | 3.532 |
| MG | 31 | 44706 | Nova Era | 18.579 |
| MG | 31 | 44805 | Nova Lima | 76.608 |
| MG | 31 | 44904 | Nova Módica | 3.952 |
| MG | 31 | 45000 | Nova Ponte | 12.504 |
| MG | 31 | 45059 | Nova Porteirinha | 7.597 |
| MG | 31 | 45109 | Nova Resende | 14.686 |
| MG | 31 | 45208 | Nova Serrana | 67.967 |
| MG | 31 | 36603 | Nova União | 5.653 |
| MG | 31 | 45307 | Novo Cruzeiro | 31.319 |
| MG | 31 | 45356 | Novo Oriente de Minas | 10.763 |
| MG | 31 | 45372 | Novorizonte | 5.136 |
| MG | 31 | 45406 | Olaria | 2.479 |
| MG | 31 | 45455 | Olhos-d'Água | 5.338 |
| MG | 31 | 45505 | Olímpio Noronha | 2.655 |
| MG | 31 | 45604 | Oliveira | 39.214 |
| MG | 31 | 45703 | Oliveira Fortes | 1.951 |
| MG | 31 | 45802 | Onça de Pitangui | 3.129 |
| MG | 31 | 45851 | Oratórios | 4.538 |
| MG | 31 | 45877 | Orizânia | 7.078 |
| MG | 31 | 45901 | Ouro Branco | 35.475 |
| MG | 31 | 46008 | Ouro Fino | 32.639 |
| MG | 31 | 46107 | Ouro Preto | 69.495 |
| MG | 31 | 46206 | Ouro Verde de Minas | 7.215 |
| MG | 31 | 46255 | Padre Carvalho | 6.176 |
| MG | 31 | 46305 | Padre Paraíso | 18.891 |
| MG | 31 | 46552 | Pai Pedro | 6.217 |
| MG | 31 | 46404 | Paineiras | 4.672 |
| MG | 31 | 46503 | Pains | 8.476 |
| MG | 31 | 46602 | Paiva | 1.687 |
| MG | 31 | 46701 | Palma | 6.212 |
| MG | 31 | 46750 | Palmópolis | 6.810 |
| MG | 31 | 46909 | Papagaios | 15.384 |
| MG | 31 | 47105 | Pará de Minas | 84.264 |
| MG | 31 | 47006 | Paracatu | 83.560 |

| U.F. | | MUNICÍPIOS | | POPULAÇÃO |
|---|---|---|---|---|
| SIGLA | COD | COD | NOMES | ESTIMADA |
| MG | 31 | 47204 | Paraguaçu | 20.429 |
| MG | 31 | 47303 | Paraisópolis | 18.844 |
| MG | 31 | 47402 | Paraopeba | 23.410 |
| MG | 31 | 47600 | Passa Quatro | 15.907 |
| MG | 31 | 47709 | Passa Tempo | 8.783 |
| MG | 31 | 47808 | Passa-Vinte | 2.132 |
| MG | 31 | 47501 | Passabém | 1.825 |
| MG | 31 | 47907 | Passos | 107.619 |
| MG | 31 | 47956 | Patis | 5.572 |
| MG | 31 | 48004 | Patos de Minas | 139.841 |
| MG | 31 | 48103 | Patrocínio | 86.467 |
| MG | 31 | 48202 | Patrocínio do Muriaé | 5.613 |
| MG | 31 | 48301 | Paula Cândido | 9.404 |
| MG | 31 | 48400 | Paulistas | 5.002 |
| MG | 31 | 48509 | Pavão | 9.155 |
| MG | 31 | 48608 | Peçanha | 17.727 |
| MG | 31 | 48806 | Pedra do Anta | 3.731 |
| MG | 31 | 48905 | Pedra do Indaiá | 4.080 |
| MG | 31 | 48707 | Pedra Azul | 26.000 |
| MG | 31 | 48756 | Pedra Bonita | 6.751 |
| MG | 31 | 49002 | Pedra Dourada | 2.241 |
| MG | 31 | 49101 | Pedralva | 11.351 |
| MG | 31 | 49150 | Pedras de Maria da Cruz | 11.877 |
| MG | 31 | 49200 | Pedrinópolis | 3.586 |
| MG | 31 | 49309 | Pedro Leopoldo | 59.064 |
| MG | 31 | 49408 | Pedro Teixeira | 1.680 |
| MG | 31 | 49507 | Pequeri | 3.093 |
| MG | 31 | 49606 | Pequi | 4.504 |
| MG | 31 | 49705 | Perdigão | 7.961 |
| MG | 31 | 49804 | Perdizes | 14.786 |
| MG | 31 | 49903 | Perdões | 20.228 |
| MG | 31 | 49952 | Periquito | 7.161 |
| MG | 31 | 50000 | Pescador | 4.197 |
| MG | 31 | 50109 | Piau | 3.064 |
| MG | 31 | 50158 | Piedade de Caratinga | 6.937 |
| MG | 31 | 50208 | Piedade de Ponte Nova | 4.273 |
| MG | 31 | 50307 | Piedade do Rio Grande | 4.871 |
| MG | 31 | 50406 | Piedade dos Gerais | 4.762 |
| MG | 31 | 50505 | Pimenta | 8.525 |
| MG | 31 | 50539 | Pingo-d'Água | 4.201 |
| MG | 31 | 50570 | Pintópolis | 8.183 |
| MG | 31 | 50604 | Piracema | 6.785 |
| MG | 31 | 50703 | Pirajuba | 4.059 |
| MG | 31 | 50802 | Piranga | 17.836 |
| MG | 31 | 50901 | Piranguçu | 5.319 |
| MG | 31 | 51008 | Piranguinho | 8.227 |
| MG | 31 | 51107 | Pirapetinga | 10.636 |
| MG | 31 | 51206 | Pirapora | 53.708 |
| MG | 31 | 51305 | Piraúba | 10.931 |
| MG | 31 | 51404 | Pitangui | 26.038 |

| U.F. | | MUNICÍPIOS | | POPULAÇÃO |
|---|---|---|---|---|
| SIGLA | COD | COD | NOMES | ESTIMADA |
| MG | 31 | 51503 | Piumhi | 32.580 |
| MG | 31 | 51602 | Planura | 11.138 |
| MG | 31 | 51701 | Poço Fundo | 15.916 |
| MG | 31 | 51800 | Poços de Caldas | 151.449 |
| MG | 31 | 51909 | Pocrane | 8.790 |
| MG | 31 | 52006 | Pompéu | 29.929 |
| MG | 31 | 52105 | Ponte Nova | 57.654 |
| MG | 31 | 52170 | Ponto dos Volantes | 11.458 |
| MG | 31 | 52131 | Ponto Chique | 4.282 |
| MG | 31 | 52204 | Porteirinha | 37.842 |
| MG | 31 | 52303 | Porto Firme | 10.989 |
| MG | 31 | 52402 | Poté | 15.237 |
| MG | 31 | 52501 | Pouso Alegre | 127.974 |
| MG | 31 | 52600 | Pouso Alto | 6.494 |
| MG | 31 | 52709 | Prados | 8.560 |
| MG | 31 | 52808 | Prata | 26.857 |
| MG | 31 | 52907 | Pratápolis | 8.800 |
| MG | 31 | 53004 | Pratinha | 3.435 |
| MG | 31 | 53103 | Presidente Bernardes | 5.853 |
| MG | 31 | 53202 | Presidente Juscelino | 4.385 |
| MG | 31 | 53301 | Presidente Kubitschek | 3.085 |
| MG | 31 | 53400 | Presidente Olegário | 18.989 |
| MG | 31 | 53608 | Prudente de Morais | 9.335 |
| MG | 31 | 53707 | Quartel Geral | 3.353 |
| MG | 31 | 53806 | Queluzito | 1.896 |
| MG | 31 | 53905 | Raposos | 15.521 |
| MG | 31 | 54002 | Raul Soares | 24.606 |
| MG | 31 | 54101 | Recreio | 10.538 |
| MG | 31 | 54150 | Reduto | 6.663 |
| MG | 31 | 54200 | Resende Costa | 10.941 |
| MG | 31 | 54309 | Resplendor | 17.608 |
| MG | 31 | 54408 | Ressaquinha | 4.732 |
| MG | 31 | 54457 | Riachinho | 8.437 |
| MG | 31 | 54507 | Riacho dos Machados | 9.716 |
| MG | 31 | 54606 | Ribeirão das Neves | 349.307 |
| MG | 31 | 54705 | Ribeirão Vermelho | 3.938 |
| MG | 31 | 55108 | Rio do Prado | 4.412 |
| MG | 31 | 54804 | Rio Acima | 8.685 |
| MG | 31 | 54903 | Rio Casca | 14.790 |
| MG | 31 | 55009 | Rio Doce | 2.656 |
| MG | 31 | 55207 | Rio Espera | 6.728 |
| MG | 31 | 55306 | Rio Manso | 5.266 |
| MG | 31 | 55405 | Rio Novo | 9.300 |
| MG | 31 | 55504 | Rio Paranaíba | 10.990 |
| MG | 31 | 55603 | Rio Pardo de Minas | 29.947 |
| MG | 31 | 55702 | Rio Piracicaba | 14.846 |
| MG | 31 | 55801 | Rio Pomba | 17.359 |
| MG | 31 | 55900 | Rio Preto | 5.631 |
| MG | 31 | 56007 | Rio Vermelho | 15.343 |
| MG | 31 | 56106 | Ritápolis | 5.149 |

| U.F. | | MUNICÍPIOS | | POPULAÇÃO ESTIMADA |
|---|---|---|---|---|
| SIGLA | COD | COD | NOMES | |
| MG | 31 | 56205 | Rochedo de Minas | 2.137 |
| MG | 31 | 56304 | Rodeiro | 6.589 |
| MG | 31 | 56403 | Romaria | 3.636 |
| MG | 31 | 56452 | Rosário da Limeira | 4.362 |
| MG | 31 | 56502 | Rubelita | 8.097 |
| MG | 31 | 56601 | Rubim | 9.855 |
| MG | 31 | 56700 | Sabará | 126.195 |
| MG | 31 | 56809 | Sabinópolis | 16.326 |
| MG | 31 | 56908 | Sacramento | 23.112 |
| MG | 31 | 57005 | Salinas | 38.789 |
| MG | 31 | 57104 | Salto da Divisa | 7.157 |
| MG | 31 | 57203 | Santa Bárbara | 27.571 |
| MG | 31 | 57252 | Santa Bárbara do Leste | 7.762 |
| MG | 31 | 57278 | Santa Bárbara do Monte Verde | 2.999 |
| MG | 31 | 57302 | Santa Bárbara do Tugúrio | 4.574 |
| MG | 31 | 57336 | Santa Cruz de Minas | 7.671 |
| MG | 31 | 57377 | Santa Cruz de Salinas | 5.466 |
| MG | 31 | 57401 | Santa Cruz do Escalvado | 5.321 |
| MG | 31 | 57500 | Santa Efigênia de Minas | 4.568 |
| MG | 31 | 57609 | Santa Fé de Minas | 4.129 |
| MG | 31 | 57658 | Santa Helena de Minas | 6.126 |
| MG | 31 | 57708 | Santa Juliana | 11.571 |
| MG | 31 | 57807 | Santa Luzia | 231.607 |
| MG | 31 | 57906 | Santa Margarida | 14.806 |
| MG | 31 | 58003 | Santa Maria de Itabira | 10.821 |
| MG | 31 | 58102 | Santa Maria do Salto | 5.989 |
| MG | 31 | 58201 | Santa Maria do Suaçuí | 14.931 |
| MG | 31 | 59209 | Santa Rita de Caldas | 9.333 |
| MG | 31 | 59407 | Santa Rita de Ibitipoca | 3.847 |
| MG | 31 | 59308 | Santa Rita de Jacutinga | 5.869 |
| MG | 31 | 59357 | Santa Rita de Minas | 5.980 |
| MG | 31 | 59506 | Santa Rita do Itueto | 5.739 |
| MG | 31 | 59605 | Santa Rita do Sapucaí | 36.150 |
| MG | 31 | 59704 | Santa Rosa da Serra | 3.407 |
| MG | 31 | 59803 | Santa Vitória | 15.791 |
| MG | 31 | 58300 | Santana da Vargem | 7.222 |
| MG | 31 | 58409 | Santana de Cataguases | 3.785 |
| MG | 31 | 58508 | Santana de Pirapama | 8.820 |
| MG | 31 | 58607 | Santana do Deserto | 3.977 |
| MG | 31 | 58706 | Santana do Garambéu | 2.205 |
| MG | 31 | 58805 | Santana do Jacaré | 4.716 |
| MG | 31 | 58904 | Santana do Manhuaçu | 8.353 |
| MG | 31 | 58953 | Santana do Paraíso | 24.695 |
| MG | 31 | 59001 | Santana do Riacho | 4.405 |
| MG | 31 | 59100 | Santana dos Montes | 4.134 |
| MG | 31 | 59902 | Santo Antônio do Amparo | 18.125 |
| MG | 31 | 60009 | Santo Antônio do Aventureiro | 3.600 |
| MG | 31 | 60108 | Santo Antônio do Grama | 4.349 |
| MG | 31 | 60207 | Santo Antônio do Itambé | 4.651 |
| MG | 31 | 60306 | Santo Antônio do Jacinto | 11.458 |

| U.F. | | MUNICÍPIOS | | POPULAÇÃO |
|---|---|---|---|---|
| SIGLA | COD | COD | NOMES | ESTIMADA |
| MG | 31 | 60405 | Santo Antônio do Monte | 25.899 |
| MG | 31 | 60454 | Santo Antônio do Retiro | 7.087 |
| MG | 31 | 60504 | Santo Antônio do Rio Abaixo | 1.794 |
| MG | 31 | 60603 | Santo Hipólito | 3.673 |
| MG | 31 | 60702 | Santos Dumont | 47.244 |
| MG | 31 | 60801 | São Bento Abade | 4.715 |
| MG | 31 | 60900 | São Brás do Suaçuí | 3.657 |
| MG | 31 | 60959 | São Domingos das Dores | 5.418 |
| MG | 31 | 61007 | São Domingos do Prata | 17.857 |
| MG | 31 | 61056 | São Félix de Minas | 3.484 |
| MG | 31 | 61106 | São Francisco | 55.141 |
| MG | 31 | 61205 | São Francisco de Paula | 6.384 |
| MG | 31 | 61304 | São Francisco de Sales | 5.314 |
| MG | 31 | 61403 | São Francisco do Glória | 5.685 |
| MG | 31 | 61502 | São Geraldo | 9.846 |
| MG | 31 | 61601 | São Geraldo da Piedade | 4.865 |
| MG | 31 | 61650 | São Geraldo do Baixio | 3.461 |
| MG | 31 | 61700 | São Gonçalo do Abaeté | 6.546 |
| MG | 31 | 61809 | São Gonçalo do Pará | 11.246 |
| MG | 31 | 61908 | São Gonçalo do Rio Abaixo | 9.738 |
| MG | 31 | 25507 | São Gonçalo do Rio Preto | 3.269 |
| MG | 31 | 62005 | São Gonçalo do Sapucaí | 23.627 |
| MG | 31 | 62104 | São Gotardo | 32.580 |
| MG | 31 | 62252 | São João da Lagoa | 4.971 |
| MG | 31 | 62302 | São João da Mata | 2.981 |
| MG | 31 | 62401 | São João da Ponte | 26.983 |
| MG | 31 | 62450 | São João das Missões | 11.267 |
| MG | 31 | 62500 | São João del Rei | 85.503 |
| MG | 31 | 62559 | São João do Manhuaçu | 9.881 |
| MG | 31 | 62575 | São João do Manteninha | 5.132 |
| MG | 31 | 62609 | São João do Oriente | 8.128 |
| MG | 31 | 62658 | São João do Pacuí | 4.223 |
| MG | 31 | 62708 | São João do Paraíso | 22.782 |
| MG | 31 | 62203 | São João Batista do Glória | 7.198 |
| MG | 31 | 62807 | São João Evangelista | 16.254 |
| MG | 31 | 62906 | São João Nepomuceno | 26.160 |
| MG | 31 | 62922 | São Joaquim de Bicas | 23.986 |
| MG | 31 | 62948 | São José da Barra | 7.090 |
| MG | 31 | 62955 | São José da Lapa | 19.234 |
| MG | 31 | 63003 | São José da Safira | 4.069 |
| MG | 31 | 63102 | São José da Varginha | 4.070 |
| MG | 31 | 63201 | São José do Alegre | 4.066 |
| MG | 31 | 63300 | São José do Divino | 3.881 |
| MG | 31 | 63409 | São José do Goiabal | 5.743 |
| MG | 31 | 63508 | São José do Jacuri | 7.234 |
| MG | 31 | 63607 | São José do Mantimento | 2.579 |
| MG | 31 | 63706 | São Lourenço | 42.688 |
| MG | 31 | 63805 | São Miguel do Anta | 7.094 |
| MG | 31 | 63904 | São Pedro da União | 5.386 |
| MG | 31 | 64100 | São Pedro do Suaçuí | 5.925 |

| U.F. | | MUNICÍPIOS | | POPULAÇÃO ESTIMADA |
|---|---|---|---|---|
| SIGLA | COD | COD | NOMES | |
| MG | 31 | 64001 | São Pedro dos Ferros | 9.087 |
| MG | 31 | 64209 | São Romão | 9.713 |
| MG | 31 | 64308 | São Roque de Minas | 6.301 |
| MG | 31 | 64407 | São Sebastião da Bela Vista | 5.193 |
| MG | 31 | 64431 | São Sebastião da Vargem Alegre | 2.877 |
| MG | 31 | 64472 | São Sebastião do Anta | 5.723 |
| MG | 31 | 64506 | São Sebastião do Maranhão | 12.099 |
| MG | 31 | 64605 | São Sebastião do Oeste | 5.689 |
| MG | 31 | 64704 | São Sebastião do Paraíso | 64.800 |
| MG | 31 | 64803 | São Sebastião do Rio Preto | 1.737 |
| MG | 31 | 64902 | São Sebastião do Rio Verde | 2.292 |
| MG | 31 | 65206 | São Thomé das Letras | 6.943 |
| MG | 31 | 65008 | São Tiago | 10.645 |
| MG | 31 | 65107 | São Tomás de Aquino | 7.073 |
| MG | 31 | 65305 | São Vicente de Minas | 6.525 |
| MG | 31 | 65404 | Sapucaí-Mirim | 6.046 |
| MG | 31 | 65503 | Sardoá | 5.477 |
| MG | 31 | 65537 | Sarzedo | 25.583 |
| MG | 31 | 65560 | Sem-Peixe | 2.993 |
| MG | 31 | 65578 | Senador Amaral | 5.201 |
| MG | 31 | 65602 | Senador Cortes | 2.082 |
| MG | 31 | 65701 | Senador Firmino | 7.361 |
| MG | 31 | 65800 | Senador José Bento | 1.855 |
| MG | 31 | 65909 | Senador Modestino Gonçalves | 5.105 |
| MG | 31 | 66006 | Senhora de Oliveira | 5.873 |
| MG | 31 | 66105 | Senhora do Porto | 3.635 |
| MG | 31 | 66204 | Senhora dos Remédios | 10.588 |
| MG | 31 | 66303 | Sericita | 7.345 |
| MG | 31 | 66402 | Seritinga | 1.818 |
| MG | 31 | 66600 | Serra da Saudade | 890 |
| MG | 31 | 66808 | Serra do Salitre | 10.778 |
| MG | 31 | 66709 | Serra dos Aimorés | 8.667 |
| MG | 31 | 66501 | Serra Azul de Minas | 4.479 |
| MG | 31 | 66907 | Serrania | 7.584 |
| MG | 31 | 66956 | Serranópolis de Minas | 4.787 |
| MG | 31 | 67004 | Serranos | 2.130 |
| MG | 31 | 67103 | Serro | 21.525 |
| MG | 31 | 67202 | Sete Lagoas | 225.358 |
| MG | 31 | 65552 | Setubinha | 11.588 |
| MG | 31 | 67301 | Silveirânia | 2.193 |
| MG | 31 | 67400 | Silvianópolis | 6.261 |
| MG | 31 | 67509 | Simão Pereira | 2.594 |
| MG | 31 | 67608 | Simonésia | 17.933 |
| MG | 31 | 67707 | Sobrália | 6.116 |
| MG | 31 | 67806 | Soledade de Minas | 5.795 |
| MG | 31 | 67905 | Tabuleiro | 4.068 |
| MG | 31 | 68002 | Taiobeiras | 31.333 |
| MG | 31 | 68051 | Taparuba | 3.357 |
| MG | 31 | 68101 | Tapira | 3.757 |
| MG | 31 | 68200 | Tapiraí | 1.888 |

| U.F. | | MUNICÍPIOS | | POPULAÇÃO ESTIMADA |
|---|---|---|---|---|
| SIGLA | COD | COD | NOMES | |
| MG | 31 | 68309 | Taquaraçu de Minas | 3.950 |
| MG | 31 | 68408 | Tarumirim | 14.585 |
| MG | 31 | 68507 | Teixeiras | 12.187 |
| MG | 31 | 68606 | Teófilo Otoni | 130.517 |
| MG | 31 | 68705 | Timóteo | 79.813 |
| MG | 31 | 68804 | Tiradentes | 6.966 |
| MG | 31 | 68903 | Tiros | 7.626 |
| MG | 31 | 69000 | Tocantins | 16.408 |
| MG | 31 | 69059 | Tocos do Moji | 4.084 |
| MG | 31 | 69109 | Toledo | 6.038 |
| MG | 31 | 69208 | Tombos | 8.881 |
| MG | 31 | 69307 | Três Corações | 75.776 |
| MG | 31 | 69356 | Três Marias | 28.042 |
| MG | 31 | 69406 | Três Pontas | 54.149 |
| MG | 31 | 69505 | Tumiritinga | 6.198 |
| MG | 31 | 69604 | Tupaciguara | 23.841 |
| MG | 31 | 69703 | Turmalina | 18.134 |
| MG | 31 | 69802 | Turvolândia | 5.020 |
| MG | 31 | 69901 | Ubá | 99.708 |
| MG | 31 | 70008 | Ubaí | 12.499 |
| MG | 31 | 70057 | Ubaporanga | 12.561 |
| MG | 31 | 70107 | Uberaba | 296.261 |
| MG | 31 | 70206 | Uberlândia | 634.345 |
| MG | 31 | 70305 | Umburatiba | 2.844 |
| MG | 31 | 70404 | Unaí | 78.125 |
| MG | 31 | 70438 | União de Minas | 4.735 |
| MG | 31 | 70479 | Uruana de Minas | 2.747 |
| MG | 31 | 70503 | Urucânia | 10.502 |
| MG | 31 | 70529 | Urucuia | 12.203 |
| MG | 31 | 70578 | Vargem Alegre | 6.829 |
| MG | 31 | 70602 | Vargem Bonita | 2.140 |
| MG | 31 | 70651 | Vargem Grande do Rio Pardo | 4.924 |
| MG | 31 | 70701 | Varginha | 121.785 |
| MG | 31 | 70750 | Varjão de Minas | 6.520 |
| MG | 31 | 70800 | Várzea da Palma | 36.314 |
| MG | 31 | 70909 | Varzelândia | 19.771 |
| MG | 31 | 71006 | Vazante | 20.042 |
| MG | 31 | 71030 | Verdelândia | 8.514 |
| MG | 31 | 71071 | Veredinha | 6.045 |
| MG | 31 | 71105 | Veríssimo | 3.991 |
| MG | 31 | 71154 | Vermelho Novo | 4.698 |
| MG | 31 | 71204 | Vespasiano | 101.846 |
| MG | 31 | 71303 | Viçosa | 74.171 |
| MG | 31 | 71402 | Vieiras | 3.899 |
| MG | 31 | 71600 | Virgem da Lapa | 14.685 |
| MG | 31 | 71709 | Virgínia | 8.544 |
| MG | 31 | 71808 | Virginópolis | 11.273 |
| MG | 31 | 71907 | Virgolândia | 5.819 |
| MG | 31 | 72004 | Visconde do Rio Branco | 37.228 |
| MG | 31 | 72103 | Volta Grande | 5.402 |

| U.F. | | MUNICÍPIOS | | POPULAÇÃO ESTIMADA |
|---|---|---|---|---|
| SIGLA | COD | COD | NOMES | |
| MG | 31 | 72202 | Wenceslau Braz | 2.571 |
| ES | 32 | 102 | Afonso Cláudio | 31.384 |
| ES | 32 | 169 | Água Doce do Norte | 12.091 |
| ES | 32 | 136 | Águia Branca | 9.503 |
| ES | 32 | 201 | Alegre | 31.143 |
| ES | 32 | 300 | Alfredo Chaves | 14.585 |
| ES | 32 | 359 | Alto Rio Novo | 6.172 |
| ES | 32 | 409 | Anchieta | 20.226 |
| ES | 32 | 508 | Apiacá | 7.883 |
| ES | 32 | 607 | Aracruz | 78.658 |
| ES | 32 | 706 | Atilio Vivacqua | 9.361 |
| ES | 32 | 805 | Baixo Guandu | 29.891 |
| ES | 32 | 904 | Barra de São Francisco | 41.645 |
| ES | 32 | 1001 | Boa Esperança | 13.119 |
| ES | 32 | 1100 | Bom Jesus do Norte | 9.672 |
| ES | 32 | 1159 | Brejetuba | 11.097 |
| ES | 32 | 1209 | Cachoeiro de Itapemirim | 201.259 |
| ES | 32 | 1308 | Cariacica | 365.859 |
| ES | 32 | 1407 | Castelo | 33.212 |
| ES | 32 | 1506 | Colatina | 111.365 |
| ES | 32 | 1605 | Conceição da Barra | 27.059 |
| ES | 32 | 1704 | Conceição do Castelo | 11.851 |
| ES | 32 | 1803 | Divino de São Lourenço | 5.011 |
| ES | 32 | 1902 | Domingos Martins | 32.455 |
| ES | 32 | 2009 | Dores do Rio Preto | 6.293 |
| ES | 32 | 2108 | Ecoporanga | 23.891 |
| ES | 32 | 2207 | Fundão | 16.431 |
| ES | 32 | 2256 | Governador Lindenberg | 10.420 |
| ES | 32 | 2306 | Guaçuí | 26.743 |
| ES | 32 | 2405 | Guarapari | 104.534 |
| ES | 32 | 2454 | Ibatiba | 20.471 |
| ES | 32 | 2504 | Ibiraçu | 10.724 |
| ES | 32 | 2553 | Ibitirama | 9.238 |
| ES | 32 | 2603 | Iconha | 11.901 |
| ES | 32 | 2652 | Irupi | 10.735 |
| ES | 32 | 2702 | Itaguaçu | 14.171 |
| ES | 32 | 2801 | Itapemirim | 32.761 |
| ES | 32 | 2900 | Itarana | 10.667 |
| ES | 32 | 3007 | Iúna | 26.239 |
| ES | 32 | 3056 | Jaguaré | 23.472 |
| ES | 32 | 3106 | Jerônimo Monteiro | 11.235 |
| ES | 32 | 3130 | João Neiva | 14.621 |
| ES | 32 | 3163 | Laranja da Terra | 11.136 |
| ES | 32 | 3205 | Linhares | 132.664 |
| ES | 32 | 3304 | Mantenópolis | 11.630 |
| ES | 32 | 3320 | Marataízes | 32.502 |
| ES | 32 | 3346 | Marechal Floriano | 13.302 |
| ES | 32 | 3353 | Marilândia | 10.676 |
| ES | 32 | 3403 | Mimoso do Sul | 27.124 |
| ES | 32 | 3502 | Montanha | 18.856 |

| U.F. | | MUNICÍPIOS | | POPULAÇÃO |
|---|---|---|---|---|
| SIGLA | COD | COD | NOMES | ESTIMADA |
| ES | 32 | 3601 | Mucurici | 5.910 |
| ES | 32 | 3700 | Muniz Freire | 18.358 |
| ES | 32 | 3809 | Muqui | 14.377 |
| ES | 32 | 3908 | Nova Venécia | 46.354 |
| ES | 32 | 4005 | Pancas | 18.497 |
| ES | 32 | 4054 | Pedro Canário | 24.404 |
| ES | 32 | 4104 | Pinheiros | 23.874 |
| ES | 32 | 4203 | Piúma | 17.212 |
| ES | 32 | 4252 | Ponto Belo | 7.247 |
| ES | 32 | 4302 | Presidente Kennedy | 10.903 |
| ES | 32 | 4351 | Rio Bananal | 17.247 |
| ES | 32 | 4401 | Rio Novo do Sul | 11.447 |
| ES | 32 | 4500 | Santa Leopoldina | 12.743 |
| ES | 32 | 4559 | Santa Maria de Jetibá | 33.921 |
| ES | 32 | 4609 | Santa Teresa | 20.742 |
| ES | 32 | 4658 | São Domingos do Norte | 8.205 |
| ES | 32 | 4708 | São Gabriel da Palha | 30.604 |
| ES | 32 | 4807 | São José do Calçado | 10.965 |
| ES | 32 | 4906 | São Mateus | 101.613 |
| ES | 32 | 4955 | São Roque do Canaã | 10.817 |
| ES | 32 | 5002 | Serra | 404.688 |
| ES | 32 | 5010 | Sooretama | 23.761 |
| ES | 32 | 5036 | Vargem Alta | 18.637 |
| ES | 32 | 5069 | Venda Nova do Imigrante | 20.028 |
| ES | 32 | 5101 | Viana | 60.829 |
| ES | 32 | 5150 | Vila Pavão | 9.126 |
| ES | 32 | 5176 | Vila Valério | 14.048 |
| ES | 32 | 5200 | Vila Velha | 413.548 |
| ES | 32 | 5309 | Vitória | 320.156 |
| RJ | 33 | 100 | Angra dos Reis | 168.664 |
| RJ | 33 | 159 | Aperibé | 9.556 |
| RJ | 33 | 209 | Araruama | 109.705 |
| RJ | 33 | 225 | Areal | 11.982 |
| RJ | 33 | 233 | Armação dos Búzios | 28.653 |
| RJ | 33 | 258 | Arraial do Cabo | 26.896 |
| RJ | 33 | 308 | Barra do Piraí | 103.833 |
| RJ | 33 | 407 | Barra Mansa | 176.899 |
| RJ | 33 | 456 | Belford Roxo | 501.544 |
| RJ | 33 | 506 | Bom Jardim | 26.549 |
| RJ | 33 | 605 | Bom Jesus do Itabapoana | 35.303 |
| RJ | 33 | 704 | Cabo Frio | 186.004 |
| RJ | 33 | 803 | Cachoeiras de Macacu | 57.300 |
| RJ | 33 | 902 | Cambuci | 14.770 |
| RJ | 33 | 1009 | Campos dos Goytacazes | 434.008 |
| RJ | 33 | 1108 | Cantagalo | 20.540 |
| RJ | 33 | 936 | Carapebus | 11.939 |
| RJ | 33 | 1157 | Cardoso Moreira | 12.481 |
| RJ | 33 | 1207 | Carmo | 18.024 |
| RJ | 33 | 1306 | Casimiro de Abreu | 30.572 |
| RJ | 33 | 951 | Comendador Levy Gasparian | 8.839 |

| U.F. | | MUNICÍPIOS | | POPULAÇÃO ESTIMADA |
|---|---|---|---|---|
| SIGLA | COD | COD | NOMES | |
| RJ | 33 | 1405 | Conceição de Macabu | 20.687 |
| RJ | 33 | 1504 | Cordeiro | 19.902 |
| RJ | 33 | 1603 | Duas Barras | 10.891 |
| RJ | 33 | 1702 | Duque de Caxias | 872.762 |
| RJ | 33 | 1801 | Engenheiro Paulo de Frontin | 13.214 |
| RJ | 33 | 1850 | Guapimirim | 49.748 |
| RJ | 33 | 1876 | Iguaba Grande | 22.947 |
| RJ | 33 | 1900 | Itaboraí | 228.996 |
| RJ | 33 | 2007 | Itaguaí | 105.633 |
| RJ | 33 | 2056 | Italva | 14.676 |
| RJ | 33 | 2106 | Itaocara | 22.452 |
| RJ | 33 | 2205 | Itaperuna | 99.454 |
| RJ | 33 | 2254 | Itatiaia | 35.577 |
| RJ | 33 | 2270 | Japeri | 101.690 |
| RJ | 33 | 2304 | Laje do Muriaé | 7.997 |
| RJ | 33 | 2403 | Macaé | 194.413 |
| RJ | 33 | 2452 | Macuco | 5.626 |
| RJ | 33 | 2502 | Magé | 244.334 |
| RJ | 33 | 2601 | Mangaratiba | 32.533 |
| RJ | 33 | 2700 | Maricá | 123.492 |
| RJ | 33 | 2809 | Mendes | 17.880 |
| RJ | 33 | 2858 | Mesquita | 190.056 |
| RJ | 33 | 2908 | Miguel Pereira | 25.866 |
| RJ | 33 | 3005 | Miracema | 26.824 |
| RJ | 33 | 3104 | Natividade | 15.406 |
| RJ | 33 | 3203 | Nilópolis | 159.408 |
| RJ | 33 | 3302 | Niterói | 479.384 |
| RJ | 33 | 3401 | Nova Friburgo | 178.653 |
| RJ | 33 | 3500 | Nova Iguaçu | 865.089 |
| RJ | 33 | 3609 | Paracambi | 45.016 |
| RJ | 33 | 3708 | Paraíba do Sul | 41.679 |
| RJ | 33 | 3807 | Parati | 35.730 |
| RJ | 33 | 3856 | Paty do Alferes | 26.196 |
| RJ | 33 | 3906 | Petrópolis | 315.119 |
| RJ | 33 | 3955 | Pinheiral | 22.382 |
| RJ | 33 | 4003 | Piraí | 26.114 |
| RJ | 33 | 4102 | Porciúncula | 18.444 |
| RJ | 33 | 4110 | Porto Real | 16.253 |
| RJ | 33 | 4128 | Quatis | 13.137 |
| RJ | 33 | 4144 | Queimados | 139.378 |
| RJ | 33 | 4151 | Quissamã | 19.878 |
| RJ | 33 | 4201 | Resende | 130.035 |
| RJ | 33 | 4508 | Rio das Flores | 8.787 |
| RJ | 33 | 4524 | Rio das Ostras | 96.622 |
| RJ | 33 | 4557 | Rio de Janeiro | 6.186.710 |
| RJ | 33 | 4300 | Rio Bonito | 55.051 |
| RJ | 33 | 4409 | Rio Claro | 18.366 |
| RJ | 33 | 4607 | Santa Maria Madalena | 10.775 |
| RJ | 33 | 4706 | Santo Antônio de Pádua | 42.405 |
| RJ | 33 | 4805 | São Fidélis | 39.256 |

| U.F. | | MUNICÍPIOS | | POPULAÇÃO ESTIMADA |
|---|---|---|---|---|
| SIGLA | COD | COD | NOMES | |
| RJ | 33 | 4755 | São Francisco de Itabapoana | 47.832 |
| RJ | 33 | 4904 | São Gonçalo | 991.382 |
| RJ | 33 | 5000 | São João da Barra | 30.595 |
| RJ | 33 | 5109 | São João de Meriti | 469.827 |
| RJ | 33 | 5133 | São José de Ubá | 7.297 |
| RJ | 33 | 5158 | São José do Vale do Rio Preto | 20.574 |
| RJ | 33 | 5208 | São Pedro da Aldeia | 84.866 |
| RJ | 33 | 5307 | São Sebastião do Alto | 9.052 |
| RJ | 33 | 5406 | Sapucaia | 17.356 |
| RJ | 33 | 5505 | Saquarema | 69.374 |
| RJ | 33 | 5554 | Seropédica | 78.819 |
| RJ | 33 | 5604 | Silva Jardim | 22.230 |
| RJ | 33 | 5703 | Sumidouro | 15.313 |
| RJ | 33 | 5752 | Tanguá | 30.531 |
| RJ | 33 | 5802 | Teresópolis | 162.075 |
| RJ | 33 | 5901 | Trajano de Morais | 9.914 |
| RJ | 33 | 6008 | Três Rios | 76.075 |
| RJ | 33 | 6107 | Valença | 75.819 |
| RJ | 33 | 6156 | Varre-Sai | 8.852 |
| RJ | 33 | 6206 | Vassouras | 34.259 |
| RJ | 33 | 6305 | Volta Redonda | 261.403 |
| SP | 35 | 105 | Adamantina | 34.424 |
| SP | 35 | 204 | Adolfo | 3.710 |
| SP | 35 | 303 | Aguaí | 32.101 |
| SP | 35 | 402 | Águas da Prata | 7.734 |
| SP | 35 | 501 | Águas de Lindóia | 16.341 |
| SP | 35 | 550 | Águas de Santa Bárbara | 5.612 |
| SP | 35 | 600 | Águas de São Pedro | 2.613 |
| SP | 35 | 709 | Agudos | 36.188 |
| SP | 35 | 758 | Alambari | 4.391 |
| SP | 35 | 808 | Alfredo Marcondes | 4.055 |
| SP | 35 | 907 | Altair | 3.287 |
| SP | 35 | 1004 | Altinópolis | 15.554 |
| SP | 35 | 1103 | Alto Alegre | 4.267 |
| SP | 35 | 1152 | Alumínio | 16.427 |
| SP | 35 | 1202 | Álvares Florence | 3.876 |
| SP | 35 | 1301 | Álvares Machado | 23.779 |
| SP | 35 | 1400 | Álvaro de Carvalho | 4.984 |
| SP | 35 | 1509 | Alvinlândia | 2.869 |
| SP | 35 | 1608 | Americana | 205.229 |
| SP | 35 | 1806 | Américo de Campos | 5.488 |
| SP | 35 | 1707 | Américo Brasiliense | 33.255 |
| SP | 35 | 1905 | Amparo | 65.928 |
| SP | 35 | 2002 | Analândia | 4.558 |
| SP | 35 | 2101 | Andradina | 56.593 |
| SP | 35 | 2200 | Angatuba | 23.225 |
| SP | 35 | 2309 | Anhembi | 5.766 |
| SP | 35 | 2408 | Anhumas | 3.947 |
| SP | 35 | 2507 | Aparecida | 37.629 |
| SP | 35 | 2606 | Aparecida d'Oeste | 4.598 |

| U.F. | | MUNICÍPIOS | | POPULAÇÃO |
|---|---|---|---|---|
| SIGLA | COD | COD | NOMES | ESTIMADA |
| SP | 35 | 2705 | Apiaí | 25.700 |
| SP | 35 | 2754 | Araçariguama | 13.208 |
| SP | 35 | 2804 | Araçatuba | 182.204 |
| SP | 35 | 2903 | Araçoiaba da Serra | 26.626 |
| SP | 35 | 3000 | Aramina | 5.308 |
| SP | 35 | 3109 | Arandu | 6.212 |
| SP | 35 | 3158 | Arapeí | 2.582 |
| SP | 35 | 3208 | Araraquara | 200.666 |
| SP | 35 | 3307 | Araras | 114.515 |
| SP | 35 | 3356 | Arco-Íris | 2.010 |
| SP | 35 | 3406 | Arealva | 7.886 |
| SP | 35 | 3505 | Areias | 3.690 |
| SP | 35 | 3604 | Areiópolis | 11.157 |
| SP | 35 | 3703 | Ariranha | 8.876 |
| SP | 35 | 3802 | Artur Nogueira | 43.499 |
| SP | 35 | 3901 | Arujá | 80.922 |
| SP | 35 | 3950 | Aspásia | 1.826 |
| SP | 35 | 4008 | Assis | 98.715 |
| SP | 35 | 4107 | Atibaia | 126.757 |
| SP | 35 | 4206 | Auriflama | 14.366 |
| SP | 35 | 4305 | Avaí | 5.170 |
| SP | 35 | 4404 | Avanhandava | 12.112 |
| SP | 35 | 4503 | Avaré | 84.416 |
| SP | 35 | 4602 | Bady Bassitt | 14.127 |
| SP | 35 | 4701 | Balbinos | 4.697 |
| SP | 35 | 4800 | Bálsamo | 8.227 |
| SP | 35 | 4909 | Bananal | 10.822 |
| SP | 35 | 5005 | Barão de Antonina | 2.835 |
| SP | 35 | 5104 | Barbosa | 6.944 |
| SP | 35 | 5203 | Bariri | 33.267 |
| SP | 35 | 5351 | Barra do Chapéu | 5.358 |
| SP | 35 | 5401 | Barra do Turvo | 7.699 |
| SP | 35 | 5302 | Barra Bonita | 36.214 |
| SP | 35 | 5500 | Barretos | 113.618 |
| SP | 35 | 5609 | Barrinha | 27.169 |
| SP | 35 | 5708 | Barueri | 270.173 |
| SP | 35 | 5807 | Bastos | 21.380 |
| SP | 35 | 5906 | Batatais | 56.476 |
| SP | 35 | 6003 | Bauru | 359.429 |
| SP | 35 | 6102 | Bebedouro | 77.630 |
| SP | 35 | 6201 | Bento de Abreu | 2.982 |
| SP | 35 | 6300 | Bernardino de Campos | 10.776 |
| SP | 35 | 6359 | Bertioga | 44.233 |
| SP | 35 | 6409 | Bilac | 7.494 |
| SP | 35 | 6508 | Birigui | 110.911 |
| SP | 35 | 6607 | Biritiba-Mirim | 29.650 |
| SP | 35 | 6706 | Boa Esperança do Sul | 13.953 |
| SP | 35 | 6805 | Bocaina | 11.028 |
| SP | 35 | 6904 | Bofete | 9.375 |
| SP | 35 | 7001 | Boituva | 44.906 |

| U.F. | | MUNICÍPIOS | | POPULAÇÃO |
|---|---|---|---|---|
| SIGLA | COD | COD | NOMES | ESTIMADA |
| SP | 35 | 7100 | Bom Jesus dos Perdões | 17.993 |
| SP | 35 | 7159 | Bom Sucesso de Itararé | 3.968 |
| SP | 35 | 7209 | Borá | 837 |
| SP | 35 | 7308 | Boracéia | 4.439 |
| SP | 35 | 7407 | Borborema | 14.485 |
| SP | 35 | 7456 | Borebi | 2.349 |
| SP | 35 | 7506 | Botucatu | 130.348 |
| SP | 35 | 7605 | Bragança Paulista | 145.894 |
| SP | 35 | 7704 | Braúna | 5.043 |
| SP | 35 | 7753 | Brejo Alegre | 2.573 |
| SP | 35 | 7803 | Brodowski | 20.485 |
| SP | 35 | 7902 | Brotas | 22.631 |
| SP | 35 | 8009 | Buri | 18.146 |
| SP | 35 | 8108 | Buritama | 15.636 |
| SP | 35 | 8207 | Buritizal | 4.096 |
| SP | 35 | 8306 | Cabrália Paulista | 4.369 |
| SP | 35 | 8405 | Cabreúva | 42.700 |
| SP | 35 | 8504 | Caçapava | 85.181 |
| SP | 35 | 8603 | Cachoeira Paulista | 34.666 |
| SP | 35 | 8702 | Caconde | 19.304 |
| SP | 35 | 8801 | Cafelândia | 16.778 |
| SP | 35 | 8900 | Caiabu | 4.131 |
| SP | 35 | 9007 | Caieiras | 88.212 |
| SP | 35 | 9106 | Caiuá | 5.447 |
| SP | 35 | 9205 | Cajamar | 63.675 |
| SP | 35 | 9254 | Cajati | 28.936 |
| SP | 35 | 9304 | Cajobi | 10.009 |
| SP | 35 | 9403 | Cajuru | 24.313 |
| SP | 35 | 9452 | Campina do Monte Alegre | 5.560 |
| SP | 35 | 9502 | Campinas | 1.064.669 |
| SP | 35 | 9601 | Campo Limpo Paulista | 74.863 |
| SP | 35 | 9700 | Campos do Jordão | 46.505 |
| SP | 35 | 9809 | Campos Novos Paulista | 5.014 |
| SP | 35 | 9908 | Cananéia | 12.374 |
| SP | 35 | 9957 | Canas | 4.765 |
| SP | 35 | 10005 | Cândido Mota | 30.776 |
| SP | 35 | 10104 | Cândido Rodrigues | 2.770 |
| SP | 35 | 10153 | Canitar | 4.743 |
| SP | 35 | 10203 | Capão Bonito | 46.338 |
| SP | 35 | 10302 | Capela do Alto | 17.232 |
| SP | 35 | 10401 | Capivari | 46.331 |
| SP | 35 | 10500 | Caraguatatuba | 96.125 |
| SP | 35 | 10609 | Carapicuíba | 392.701 |
| SP | 35 | 10708 | Cardoso | 11.624 |
| SP | 35 | 10807 | Casa Branca | 28.189 |
| SP | 35 | 10906 | Cássia dos Coqueiros | 2.737 |
| SP | 35 | 11003 | Castilho | 16.165 |
| SP | 35 | 11102 | Catanduva | 114.812 |
| SP | 35 | 11201 | Catiguá | 7.250 |
| SP | 35 | 11300 | Cedral | 8.258 |

| U.F. | | MUNICÍPIOS | | POPULAÇÃO ESTIMADA |
|---|---|---|---|---|
| SIGLA | COD | COD | NOMES | |
| SP | 35 | 11409 | Cerqueira César | 17.337 |
| SP | 35 | 11508 | Cerquilho | 38.199 |
| SP | 35 | 11607 | Cesário Lange | 14.979 |
| SP | 35 | 11706 | Charqueada | 15.423 |
| SP | 35 | 57204 | Chavantes | 12.688 |
| SP | 35 | 11904 | Clementina | 6.582 |
| SP | 35 | 12001 | Colina | 17.745 |
| SP | 35 | 12100 | Colômbia | 6.345 |
| SP | 35 | 12209 | Conchal | 24.485 |
| SP | 35 | 12308 | Conchas | 16.274 |
| SP | 35 | 12407 | Cordeirópolis | 20.720 |
| SP | 35 | 12506 | Coroados | 5.250 |
| SP | 35 | 12605 | Coronel Macedo | 5.343 |
| SP | 35 | 12704 | Corumbataí | 4.138 |
| SP | 35 | 12803 | Cosmópolis | 59.297 |
| SP | 35 | 12902 | Cosmorama | 7.033 |
| SP | 35 | 13009 | Cotia | 182.045 |
| SP | 35 | 13108 | Cravinhos | 30.849 |
| SP | 35 | 13207 | Cristais Paulista | 7.436 |
| SP | 35 | 13306 | Cruzália | 2.356 |
| SP | 35 | 13405 | Cruzeiro | 79.957 |
| SP | 35 | 13504 | Cubatão | 129.582 |
| SP | 35 | 13603 | Cunha | 23.735 |
| SP | 35 | 13702 | Descalvado | 30.867 |
| SP | 35 | 13801 | Diadema | 397.738 |
| SP | 35 | 13850 | Dirce Reis | 1.623 |
| SP | 35 | 13900 | Divinolândia | 11.343 |
| SP | 35 | 14007 | Dobrada | 8.214 |
| SP | 35 | 14106 | Dois Córregos | 26.040 |
| SP | 35 | 14205 | Dolcinópolis | 2.272 |
| SP | 35 | 14304 | Dourado | 9.131 |
| SP | 35 | 14403 | Dracena | 44.309 |
| SP | 35 | 14502 | Duartina | 12.796 |
| SP | 35 | 14601 | Dumont | 8.346 |
| SP | 35 | 14700 | Echaporã | 6.239 |
| SP | 35 | 14809 | Eldorado | 14.514 |
| SP | 35 | 14908 | Elias Fausto | 15.312 |
| SP | 35 | 14924 | Elisiário | 3.317 |
| SP | 35 | 14957 | Embaúba | 2.442 |
| SP | 35 | 15004 | Embu | 248.722 |
| SP | 35 | 15103 | Embu-Guaçu | 62.137 |
| SP | 35 | 15129 | Emilianópolis | 3.184 |
| SP | 35 | 15152 | Engenheiro Coelho | 14.300 |
| SP | 35 | 15186 | Espírito Santo do Pinhal | 42.260 |
| SP | 35 | 15194 | Espírito Santo do Turvo | 4.428 |
| SP | 35 | 57303 | Estiva Gerbi | 9.657 |
| SP | 35 | 15202 | Estrela d'Oeste | 9.042 |
| SP | 35 | 15301 | Estrela do Norte | 2.474 |
| SP | 35 | 15350 | Euclides da Cunha Paulista | 10.168 |
| SP | 35 | 15400 | Fartura | 14.969 |

| U.F. | | MUNICÍPIOS | | POPULAÇÃO |
|---|---|---|---|---|
| SIGLA | COD | COD | NOMES | ESTIMADA |
| SP | 35 | 15608 | Fernando Prestes | 5.312 |
| SP | 35 | 15509 | Fernandópolis | 63.539 |
| SP | 35 | 15657 | Fernão | 1.521 |
| SP | 35 | 15707 | Ferraz de Vasconcelos | 179.231 |
| SP | 35 | 15806 | Flora Rica | 2.029 |
| SP | 35 | 15905 | Floreal | 2.884 |
| SP | 35 | 16002 | Flórida Paulista | 13.761 |
| SP | 35 | 16101 | Florínia | 2.856 |
| SP | 35 | 16200 | Franca | 330.938 |
| SP | 35 | 16309 | Francisco Morato | 157.294 |
| SP | 35 | 16408 | Franco da Rocha | 131.366 |
| SP | 35 | 16507 | Gabriel Monteiro | 2.698 |
| SP | 35 | 16606 | Gália | 6.635 |
| SP | 35 | 16705 | Garça | 43.380 |
| SP | 35 | 16804 | Gastão Vidigal | 4.118 |
| SP | 35 | 16853 | Gavião Peixoto | 4.244 |
| SP | 35 | 16903 | General Salgado | 10.934 |
| SP | 35 | 17000 | Getulina | 10.960 |
| SP | 35 | 17109 | Glicério | 4.547 |
| SP | 35 | 17208 | Guaiçara | 11.206 |
| SP | 35 | 17307 | Guaimbê | 4.740 |
| SP | 35 | 17406 | Guaíra | 38.676 |
| SP | 35 | 17505 | Guapiaçu | 17.938 |
| SP | 35 | 17604 | Guapiara | 20.927 |
| SP | 35 | 17703 | Guará | 19.168 |
| SP | 35 | 17802 | Guaraçaí | 8.657 |
| SP | 35 | 17901 | Guaraci | 9.459 |
| SP | 35 | 18008 | Guarani d'Oeste | 2.017 |
| SP | 35 | 18107 | Guarantã | 6.680 |
| SP | 35 | 18206 | Guararapes | 29.639 |
| SP | 35 | 18305 | Guararema | 26.974 |
| SP | 35 | 18404 | Guaratinguetá | 113.357 |
| SP | 35 | 18503 | Guareí | 14.918 |
| SP | 35 | 18602 | Guariba | 34.508 |
| SP | 35 | 18701 | Guarujá | 308.058 |
| SP | 35 | 18800 | Guarulhos | 1.299.283 |
| SP | 35 | 18859 | Guatapará | 6.382 |
| SP | 35 | 18909 | Guzolândia | 4.917 |
| SP | 35 | 19006 | Herculândia | 9.125 |
| SP | 35 | 19055 | Holambra | 10.224 |
| SP | 35 | 19071 | Hortolândia | 205.856 |
| SP | 35 | 19105 | Iacanga | 9.732 |
| SP | 35 | 19204 | Iacri | 6.879 |
| SP | 35 | 19253 | Iaras | 5.658 |
| SP | 35 | 19303 | Ibaté | 29.714 |
| SP | 35 | 19402 | Ibirá | 11.141 |
| SP | 35 | 19501 | Ibirarema | 7.235 |
| SP | 35 | 19600 | Ibitinga | 53.148 |
| SP | 35 | 19709 | Ibiúna | 67.392 |
| SP | 35 | 19808 | Icém | 6.524 |

| U.F. | | MUNICÍPIOS | | POPULAÇÃO ESTIMADA |
|---|---|---|---|---|
| SIGLA | COD | COD | NOMES | |
| SP | 35 | 19907 | Iepê | 7.856 |
| SP | 35 | 20004 | Igaraçu do Tietê | 24.124 |
| SP | 35 | 20103 | Igarapava | 28.230 |
| SP | 35 | 20202 | Igaratá | 8.950 |
| SP | 35 | 20301 | Iguape | 30.675 |
| SP | 35 | 20426 | Ilha Comprida | 10.090 |
| SP | 35 | 20442 | Ilha Solteira | 25.144 |
| SP | 35 | 20400 | Ilhabela | 26.011 |
| SP | 35 | 20509 | Indaiatuba | 183.803 |
| SP | 35 | 20608 | Indiana | 4.751 |
| SP | 35 | 20707 | Indiaporã | 3.949 |
| SP | 35 | 20806 | Inúbia Paulista | 3.841 |
| SP | 35 | 20905 | Ipaussu | 13.608 |
| SP | 35 | 21002 | Iperó | 27.526 |
| SP | 35 | 21101 | Ipeúna | 5.691 |
| SP | 35 | 21150 | Ipiguá | 4.253 |
| SP | 35 | 21200 | Iporanga | 4.789 |
| SP | 35 | 21309 | Ipuã | 15.883 |
| SP | 35 | 21408 | Iracemápolis | 19.700 |
| SP | 35 | 21507 | Irapuã | 6.978 |
| SP | 35 | 21606 | Irapuru | 7.874 |
| SP | 35 | 21705 | Itaberá | 17.674 |
| SP | 35 | 21804 | Itaí | 24.093 |
| SP | 35 | 21903 | Itajobi | 14.683 |
| SP | 35 | 22000 | Itaju | 2.714 |
| SP | 35 | 22109 | Itanhaém | 87.338 |
| SP | 35 | 22158 | Itaóca | 3.143 |
| SP | 35 | 22208 | Itapecerica da Serra | 161.983 |
| SP | 35 | 22307 | Itapetininga | 148.808 |
| SP | 35 | 22406 | Itapeva | 89.768 |
| SP | 35 | 22505 | Itapevi | 205.881 |
| SP | 35 | 22604 | Itapira | 72.657 |
| SP | 35 | 22653 | Itapirapuã Paulista | 3.919 |
| SP | 35 | 22703 | Itápolis | 40.411 |
| SP | 35 | 22802 | Itaporanga | 14.780 |
| SP | 35 | 22901 | Itapuí | 12.536 |
| SP | 35 | 23008 | Itapura | 3.941 |
| SP | 35 | 23107 | Itaquaquecetuba | 359.253 |
| SP | 35 | 23206 | Itararé | 51.412 |
| SP | 35 | 23305 | Itariri | 16.284 |
| SP | 35 | 23404 | Itatiba | 99.047 |
| SP | 35 | 23503 | Itatinga | 19.085 |
| SP | 35 | 23602 | Itirapina | 14.829 |
| SP | 35 | 23701 | Itirapuã | 5.903 |
| SP | 35 | 23800 | Itobi | 7.708 |
| SP | 35 | 23909 | Itu | 157.384 |
| SP | 35 | 24006 | Itupeva | 42.458 |
| SP | 35 | 24105 | Ituverava | 40.882 |
| SP | 35 | 24204 | Jaborandi | 6.715 |
| SP | 35 | 24303 | Jaboticabal | 73.084 |

| U.F. | | MUNICÍPIOS | | POPULAÇÃO ESTIMADA |
|---|---|---|---|---|
| SIGLA | COD | COD | NOMES | |
| SP | 35 | 24402 | Jacareí | 212.824 |
| SP | 35 | 24501 | Jaci | 5.556 |
| SP | 35 | 24600 | Jacupiranga | 16.322 |
| SP | 35 | 24709 | Jaguariúna | 41.107 |
| SP | 35 | 24808 | Jales | 49.996 |
| SP | 35 | 24907 | Jambeiro | 5.550 |
| SP | 35 | 25003 | Jandira | 112.130 |
| SP | 35 | 25102 | Jardinópolis | 37.471 |
| SP | 35 | 25201 | Jarinu | 22.822 |
| SP | 35 | 25300 | Jaú | 135.546 |
| SP | 35 | 25409 | Jeriquara | 3.216 |
| SP | 35 | 25508 | Joanópolis | 11.169 |
| SP | 35 | 25607 | João Ramalho | 4.344 |
| SP | 35 | 25706 | José Bonifácio | 32.551 |
| SP | 35 | 25805 | Júlio Mesquita | 4.539 |
| SP | 35 | 25854 | Jumirim | 2.290 |
| SP | 35 | 25904 | Jundiaí | 349.929 |
| SP | 35 | 26001 | Junqueirópolis | 19.976 |
| SP | 35 | 26100 | Juquiá | 19.585 |
| SP | 35 | 26209 | Juquitiba | 29.335 |
| SP | 35 | 26308 | Lagoinha | 4.909 |
| SP | 35 | 26407 | Laranjal Paulista | 26.296 |
| SP | 35 | 26506 | Lavínia | 9.445 |
| SP | 35 | 26605 | Lavrinhas | 7.002 |
| SP | 35 | 26704 | Leme | 88.995 |
| SP | 35 | 26803 | Lençóis Paulista | 63.314 |
| SP | 35 | 26902 | Limeira | 281.583 |
| SP | 35 | 27009 | Lindóia | 5.974 |
| SP | 35 | 27108 | Lins | 73.183 |
| SP | 35 | 27207 | Lorena | 82.770 |
| SP | 35 | 27256 | Lourdes | 2.032 |
| SP | 35 | 27306 | Louveira | 33.251 |
| SP | 35 | 27405 | Lucélia | 20.284 |
| SP | 35 | 27504 | Lucianópolis | 2.443 |
| SP | 35 | 27603 | Luís Antônio | 11.924 |
| SP | 35 | 27702 | Luiziânia | 5.138 |
| SP | 35 | 27801 | Lupércio | 4.396 |
| SP | 35 | 27900 | Lutécia | 2.855 |
| SP | 35 | 28007 | Macatuba | 16.939 |
| SP | 35 | 28106 | Macaubal | 7.672 |
| SP | 35 | 28205 | Macedônia | 3.393 |
| SP | 35 | 28304 | Magda | 3.160 |
| SP | 35 | 28403 | Mairinque | 43.658 |
| SP | 35 | 28502 | Mairiporã | 79.155 |
| SP | 35 | 28601 | Manduri | 9.123 |
| SP | 35 | 28700 | Marabá Paulista | 5.996 |
| SP | 35 | 28809 | Maracaí | 13.710 |
| SP | 35 | 28858 | Marapoama | 2.780 |
| SP | 35 | 28908 | Mariápolis | 3.896 |
| SP | 35 | 29005 | Marília | 225.938 |

| U.F. | | MUNICÍPIOS | | POPULAÇÃO ESTIMADA |
|---|---|---|---|---|
| SIGLA | COD | COD | NOMES | |
| SP | 35 | 29104 | Marinópolis | 2.158 |
| SP | 35 | 29203 | Martinópolis | 25.533 |
| SP | 35 | 29302 | Matão | 78.222 |
| SP | 35 | 29401 | Mauá | 417.458 |
| SP | 35 | 29500 | Mendonça | 4.217 |
| SP | 35 | 29609 | Meridiano | 3.929 |
| SP | 35 | 29658 | Mesópolis | 1.767 |
| SP | 35 | 29708 | Miguelópolis | 21.095 |
| SP | 35 | 29807 | Mineiros do Tietê | 12.334 |
| SP | 35 | 30003 | Mira Estrela | 2.663 |
| SP | 35 | 29906 | Miracatu | 23.801 |
| SP | 35 | 30102 | Mirandópolis | 26.761 |
| SP | 35 | 30201 | Mirante do Paranapanema | 18.131 |
| SP | 35 | 30300 | Mirassol | 54.920 |
| SP | 35 | 30409 | Mirassolândia | 4.396 |
| SP | 35 | 30508 | Mococa | 68.718 |
| SP | 35 | 30607 | Mogi das Cruzes | 375.268 |
| SP | 35 | 30706 | Mogi Guaçu | 139.836 |
| SP | 35 | 30805 | Moji Mirim | 88.373 |
| SP | 35 | 30904 | Mombuca | 3.471 |
| SP | 35 | 31001 | Monções | 2.129 |
| SP | 35 | 31100 | Mongaguá | 44.087 |
| SP | 35 | 31209 | Monte Alegre do Sul | 7.473 |
| SP | 35 | 31308 | Monte Alto | 45.895 |
| SP | 35 | 31407 | Monte Aprazível | 21.015 |
| SP | 35 | 31506 | Monte Azul Paulista | 19.741 |
| SP | 35 | 31605 | Monte Castelo | 4.131 |
| SP | 35 | 31803 | Monte Mor | 46.641 |
| SP | 35 | 31704 | Monteiro Lobato | 4.295 |
| SP | 35 | 31902 | Morro Agudo | 26.305 |
| SP | 35 | 32009 | Morungaba | 13.305 |
| SP | 35 | 32058 | Motuca | 4.691 |
| SP | 35 | 32108 | Murutinga do Sul | 4.290 |
| SP | 35 | 32157 | Nantes | 2.662 |
| SP | 35 | 32207 | Narandiba | 4.167 |
| SP | 35 | 32306 | Natividade da Serra | 7.674 |
| SP | 35 | 32405 | Nazaré Paulista | 15.232 |
| SP | 35 | 32504 | Neves Paulista | 9.114 |
| SP | 35 | 32603 | Nhandeara | 10.771 |
| SP | 35 | 32702 | Nipoã | 4.103 |
| SP | 35 | 32801 | Nova Aliança | 5.140 |
| SP | 35 | 32827 | Nova Campina | 9.267 |
| SP | 35 | 32843 | Nova Canaã Paulista | 2.172 |
| SP | 35 | 32868 | Nova Castilho | 1.122 |
| SP | 35 | 32900 | Nova Europa | 10.092 |
| SP | 35 | 33007 | Nova Granada | 18.683 |
| SP | 35 | 33106 | Nova Guataporanga | 2.184 |
| SP | 35 | 33205 | Nova Independência | 2.742 |
| SP | 35 | 33304 | Nova Luzitânia | 3.795 |
| SP | 35 | 33403 | Nova Odessa | 48.754 |

| U.F. | | MUNICÍPIOS | | POPULAÇÃO ESTIMADA |
|---|---|---|---|---|
| SIGLA | COD | COD | NOMES | |
| SP | 35 | 33254 | Novais | 3.974 |
| SP | 35 | 33502 | Novo Horizonte | 36.271 |
| SP | 35 | 33601 | Nuporanga | 7.004 |
| SP | 35 | 33700 | Ocauçu | 4.341 |
| SP | 35 | 33809 | Óleo | 2.699 |
| SP | 35 | 33908 | Olímpia | 50.602 |
| SP | 35 | 34005 | Onda Verde | 4.005 |
| SP | 35 | 34104 | Oriente | 6.382 |
| SP | 35 | 34203 | Orindiúva | 5.406 |
| SP | 35 | 34302 | Orlândia | 37.534 |
| SP | 35 | 34401 | Osasco | 718.646 |
| SP | 35 | 34500 | Oscar Bressane | 2.536 |
| SP | 35 | 34609 | Osvaldo Cruz | 31.461 |
| SP | 35 | 34708 | Ourinhos | 104.542 |
| SP | 35 | 34807 | Ouro Verde | 8.162 |
| SP | 35 | 34757 | Ouroeste | 7.598 |
| SP | 35 | 34906 | Pacaembu | 13.778 |
| SP | 35 | 35002 | Palestina | 11.354 |
| SP | 35 | 35101 | Palmares Paulista | 11.742 |
| SP | 35 | 35200 | Palmeira d'Oeste | 9.706 |
| SP | 35 | 35309 | Palmital | 22.323 |
| SP | 35 | 35408 | Panorama | 14.577 |
| SP | 35 | 35507 | Paraguaçu Paulista | 44.685 |
| SP | 35 | 35606 | Paraibuna | 16.833 |
| SP | 35 | 35705 | Paraíso | 5.816 |
| SP | 35 | 35804 | Paranapanema | 17.752 |
| SP | 35 | 35903 | Paranapuã | 3.739 |
| SP | 35 | 36000 | Parapuã | 11.402 |
| SP | 35 | 36109 | Pardinho | 5.337 |
| SP | 35 | 36208 | Pariquera-Açu | 18.918 |
| SP | 35 | 36257 | Parisi | 2.149 |
| SP | 35 | 36307 | Patrocínio Paulista | 12.943 |
| SP | 35 | 36406 | Paulicéia | 5.791 |
| SP | 35 | 36505 | Paulínia | 84.577 |
| SP | 35 | 36570 | Paulistânia | 1.909 |
| SP | 35 | 36604 | Paulo de Faria | 9.463 |
| SP | 35 | 36703 | Pederneiras | 43.245 |
| SP | 35 | 36802 | Pedra Bela | 6.142 |
| SP | 35 | 36901 | Pedranópolis | 2.834 |
| SP | 35 | 37008 | Pedregulho | 15.777 |
| SP | 35 | 37107 | Pedreira | 40.752 |
| SP | 35 | 37156 | Pedrinhas Paulista | 2.927 |
| SP | 35 | 37206 | Pedro de Toledo | 10.254 |
| SP | 35 | 37305 | Penápolis | 59.597 |
| SP | 35 | 37404 | Pereira Barreto | 24.777 |
| SP | 35 | 37503 | Pereiras | 8.076 |
| SP | 35 | 37602 | Peruíbe | 57.686 |
| SP | 35 | 37701 | Piacatu | 5.472 |
| SP | 35 | 37800 | Piedade | 49.508 |
| SP | 35 | 37909 | Pilar do Sul | 28.455 |

| U.F. | | MUNICÍPIOS | | POPULAÇÃO ESTIMADA |
|---|---|---|---|---|
| SIGLA | COD | COD | NOMES | |
| SP | 35 | 38006 | Pindamonhangaba | 144.613 |
| SP | 35 | 38105 | Pindorama | 15.377 |
| SP | 35 | 38204 | Pinhalzinho | 12.591 |
| SP | 35 | 38303 | Piquerobi | 3.781 |
| SP | 35 | 38501 | Piquete | 14.709 |
| SP | 35 | 38600 | Piracaia | 22.740 |
| SP | 35 | 38709 | Piracicaba | 368.843 |
| SP | 35 | 38808 | Piraju | 29.398 |
| SP | 35 | 38907 | Pirajuí | 22.192 |
| SP | 35 | 39004 | Pirangi | 10.807 |
| SP | 35 | 39103 | Pirapora do Bom Jesus | 15.706 |
| SP | 35 | 39202 | Pirapozinho | 25.236 |
| SP | 35 | 39301 | Pirassununga | 71.470 |
| SP | 35 | 39400 | Piratininga | 11.989 |
| SP | 35 | 39509 | Pitangueiras | 35.441 |
| SP | 35 | 39608 | Planalto | 4.302 |
| SP | 35 | 39707 | Platina | 3.407 |
| SP | 35 | 39806 | Poá | 112.481 |
| SP | 35 | 39905 | Poloni | 5.100 |
| SP | 35 | 40002 | Pompéia | 20.168 |
| SP | 35 | 40101 | Pongaí | 3.510 |
| SP | 35 | 40200 | Pontal | 39.272 |
| SP | 35 | 40259 | Pontalinda | 4.200 |
| SP | 35 | 40309 | Pontes Gestal | 2.557 |
| SP | 35 | 40408 | Populina | 4.253 |
| SP | 35 | 40507 | Porangaba | 8.946 |
| SP | 35 | 40606 | Porto Feliz | 47.964 |
| SP | 35 | 40705 | Porto Ferreira | 51.090 |
| SP | 35 | 40754 | Potim | 20.668 |
| SP | 35 | 40804 | Potirendaba | 15.128 |
| SP | 35 | 40853 | Pracinha | 3.271 |
| SP | 35 | 40903 | Pradópolis | 16.619 |
| SP | 35 | 41000 | Praia Grande | 249.551 |
| SP | 35 | 41059 | Pratânia | 4.614 |
| SP | 35 | 41109 | Presidente Alves | 4.517 |
| SP | 35 | 41208 | Presidente Bernardes | 15.382 |
| SP | 35 | 41307 | Presidente Epitácio | 40.891 |
| SP | 35 | 41406 | Presidente Prudente | 207.725 |
| SP | 35 | 41505 | Presidente Venceslau | 38.439 |
| SP | 35 | 41604 | Promissão | 37.570 |
| SP | 35 | 41653 | Quadra | 2.789 |
| SP | 35 | 41703 | Quatá | 12.539 |
| SP | 35 | 41802 | Queiroz | 2.875 |
| SP | 35 | 41901 | Queluz | 11.197 |
| SP | 35 | 42008 | Quintana | 5.948 |
| SP | 35 | 42107 | Rafard | 8.364 |
| SP | 35 | 42206 | Rancharia | 29.149 |
| SP | 35 | 42305 | Redenção da Serra | 4.245 |
| SP | 35 | 42404 | Regente Feijó | 17.725 |
| SP | 35 | 42503 | Reginópolis | 8.172 |

| U.F. | | MUNICÍPIOS | | POPULAÇÃO ESTIMADA |
|---|---|---|---|---|
| SIGLA | COD | COD | NOMES | |
| SP | 35 | 42602 | Registro | 55.168 |
| SP | 35 | 42701 | Restinga | 6.897 |
| SP | 35 | 42800 | Ribeira | 3.545 |
| SP | 35 | 43204 | Ribeirão do Sul | 4.663 |
| SP | 35 | 43238 | Ribeirão dos Índios | 2.317 |
| SP | 35 | 42909 | Ribeirão Bonito | 11.857 |
| SP | 35 | 43006 | Ribeirão Branco | 18.607 |
| SP | 35 | 43105 | Ribeirão Corrente | 4.216 |
| SP | 35 | 43253 | Ribeirão Grande | 6.992 |
| SP | 35 | 43303 | Ribeirão Pires | 112.011 |
| SP | 35 | 43402 | Ribeirão Preto | 563.107 |
| SP | 35 | 43600 | Rifaina | 3.826 |
| SP | 35 | 43709 | Rincão | 10.846 |
| SP | 35 | 43808 | Rinópolis | 9.385 |
| SP | 35 | 44004 | Rio das Pedras | 28.478 |
| SP | 35 | 43907 | Rio Claro | 191.886 |
| SP | 35 | 44103 | Rio Grande da Serra | 41.602 |
| SP | 35 | 44202 | Riolândia | 10.542 |
| SP | 35 | 43501 | Riversul | 6.520 |
| SP | 35 | 44251 | Rosana | 18.918 |
| SP | 35 | 44301 | Roseira | 9.527 |
| SP | 35 | 44400 | Rubiácea | 2.661 |
| SP | 35 | 44509 | Rubinéia | 2.611 |
| SP | 35 | 44608 | Sabino | 5.420 |
| SP | 35 | 44707 | Sagres | 2.338 |
| SP | 35 | 44806 | Sales | 5.399 |
| SP | 35 | 44905 | Sales Oliveira | 8.021 |
| SP | 35 | 45001 | Salesópolis | 16.041 |
| SP | 35 | 45100 | Salmourão | 4.779 |
| SP | 35 | 45159 | Saltinho | 7.149 |
| SP | 35 | 45209 | Salto | 109.948 |
| SP | 35 | 45308 | Salto de Pirapora | 39.616 |
| SP | 35 | 45407 | Salto Grande | 8.968 |
| SP | 35 | 45506 | Sandovalina | 3.387 |
| SP | 35 | 45605 | Santa Adélia | 14.537 |
| SP | 35 | 45704 | Santa Albertina | 4.992 |
| SP | 35 | 45803 | Santa Bárbara d'Oeste | 189.573 |
| SP | 35 | 46009 | Santa Branca | 13.881 |
| SP | 35 | 46108 | Santa Clara d'Oeste | 2.141 |
| SP | 35 | 46207 | Santa Cruz da Conceição | 4.154 |
| SP | 35 | 46256 | Santa Cruz da Esperança | 1.733 |
| SP | 35 | 46306 | Santa Cruz das Palmeiras | 33.583 |
| SP | 35 | 46405 | Santa Cruz do Rio Pardo | 43.483 |
| SP | 35 | 46504 | Santa Ernestina | 5.618 |
| SP | 35 | 46603 | Santa Fé do Sul | 29.192 |
| SP | 35 | 46702 | Santa Gertrudes | 21.028 |
| SP | 35 | 46801 | Santa Isabel | 46.902 |
| SP | 35 | 46900 | Santa Lúcia | 8.154 |
| SP | 35 | 47007 | Santa Maria da Serra | 5.920 |
| SP | 35 | 47106 | Santa Mercedes | 2.596 |

| U.F. | | MUNICÍPIOS | | POPULAÇÃO ESTIMADA |
|---|---|---|---|---|
| SIGLA | COD | COD | NOMES | |
| SP | 35 | 47403 | Santa Rita d'Oeste | 2.501 |
| SP | 35 | 47502 | Santa Rita do Passa Quatro | 27.557 |
| SP | 35 | 47601 | Santa Rosa de Viterbo | 24.049 |
| SP | 35 | 47650 | Santa Salete | 1.446 |
| SP | 35 | 47205 | Santana da Ponte Pensa | 1.616 |
| SP | 35 | 47304 | Santana de Parnaíba | 114.321 |
| SP | 35 | 47700 | Santo Anastácio | 21.222 |
| SP | 35 | 47809 | Santo André | 673.396 |
| SP | 35 | 47908 | Santo Antônio da Alegria | 6.346 |
| SP | 35 | 48005 | Santo Antônio de Posse | 21.247 |
| SP | 35 | 48054 | Santo Antônio do Aracanguá | 7.335 |
| SP | 35 | 48104 | Santo Antônio do Jardim | 5.785 |
| SP | 35 | 48203 | Santo Antônio do Pinhal | 6.896 |
| SP | 35 | 48302 | Santo Expedito | 2.956 |
| SP | 35 | 48401 | Santópolis do Aguapeí | 4.234 |
| SP | 35 | 48500 | Santos | 417.098 |
| SP | 35 | 48609 | São Bento do Sapucaí | 10.966 |
| SP | 35 | 48708 | São Bernardo do Campo | 810.979 |
| SP | 35 | 48807 | São Caetano do Sul | 152.093 |
| SP | 35 | 48906 | São Carlos | 220.463 |
| SP | 35 | 49003 | São Francisco | 2.894 |
| SP | 35 | 49102 | São João da Boa Vista | 83.909 |
| SP | 35 | 49201 | São João das Duas Pontes | 2.630 |
| SP | 35 | 49250 | São João de Iracema | 1.810 |
| SP | 35 | 49300 | São João do Pau d'Alho | 2.191 |
| SP | 35 | 49409 | São Joaquim da Barra | 46.172 |
| SP | 35 | 49508 | São José da Bela Vista | 8.513 |
| SP | 35 | 49607 | São José do Barreiro | 4.490 |
| SP | 35 | 49706 | São José do Rio Pardo | 53.281 |
| SP | 35 | 49805 | São José do Rio Preto | 419.632 |
| SP | 35 | 49904 | São José dos Campos | 615.871 |
| SP | 35 | 49953 | São Lourenço da Serra | 18.319 |
| SP | 35 | 50001 | São Luís do Paraitinga | 10.908 |
| SP | 35 | 50100 | São Manuel | 39.696 |
| SP | 35 | 50209 | São Miguel Arcanjo | 31.329 |
| SP | 35 | 50308 | São Paulo | 11.037.593 |
| SP | 35 | 50407 | São Pedro | 31.575 |
| SP | 35 | 50506 | São Pedro do Turvo | 7.439 |
| SP | 35 | 50605 | São Roque | 67.715 |
| SP | 35 | 50704 | São Sebastião | 73.631 |
| SP | 35 | 50803 | São Sebastião da Grama | 12.990 |
| SP | 35 | 50902 | São Simão | 14.329 |
| SP | 35 | 51009 | São Vicente | 330.795 |
| SP | 35 | 51108 | Sarapuí | 8.601 |
| SP | 35 | 51207 | Sarutaiá | 3.790 |
| SP | 35 | 51306 | Sebastianópolis do Sul | 3.109 |
| SP | 35 | 51405 | Serra Azul | 10.121 |
| SP | 35 | 51603 | Serra Negra | 25.912 |
| SP | 35 | 51504 | Serrana | 39.574 |
| SP | 35 | 51702 | Sertãozinho | 110.999 |

| U.F. | | MUNICÍPIOS | | POPULAÇÃO ESTIMADA |
|---|---|---|---|---|
| SIGLA | COD | COD | NOMES | |
| SP | 35 | 51801 | Sete Barras | 13.148 |
| SP | 35 | 51900 | Severínia | 15.707 |
| SP | 35 | 52007 | Silveiras | 5.841 |
| SP | 35 | 52106 | Socorro | 34.447 |
| SP | 35 | 52205 | Sorocaba | 584.313 |
| SP | 35 | 52304 | Sud Mennucci | 8.141 |
| SP | 35 | 52403 | Sumaré | 241.077 |
| SP | 35 | 52551 | Suzanápolis | 3.805 |
| SP | 35 | 52502 | Suzano | 284.356 |
| SP | 35 | 52601 | Tabapuã | 11.980 |
| SP | 35 | 52700 | Tabatinga | 14.876 |
| SP | 35 | 52809 | Taboão da Serra | 227.343 |
| SP | 35 | 52908 | Taciba | 5.675 |
| SP | 35 | 53005 | Taguaí | 10.849 |
| SP | 35 | 53104 | Taiaçu | 6.093 |
| SP | 35 | 53203 | Taiúva | 5.505 |
| SP | 35 | 53302 | Tambaú | 22.575 |
| SP | 35 | 53401 | Tanabi | 24.591 |
| SP | 35 | 53500 | Tapiraí | 7.991 |
| SP | 35 | 53609 | Tapiratiba | 12.410 |
| SP | 35 | 53658 | Taquaral | 2.974 |
| SP | 35 | 53708 | Taquaritinga | 55.662 |
| SP | 35 | 53807 | Taquarituba | 23.060 |
| SP | 35 | 53856 | Taquarivaí | 5.541 |
| SP | 35 | 53906 | Tarabai | 6.464 |
| SP | 35 | 53955 | Tarumã | 13.386 |
| SP | 35 | 54003 | Tatuí | 109.017 |
| SP | 35 | 54102 | Taubaté | 273.426 |
| SP | 35 | 54201 | Tejupá | 5.067 |
| SP | 35 | 54300 | Teodoro Sampaio | 21.202 |
| SP | 35 | 54409 | Terra Roxa | 8.619 |
| SP | 35 | 54508 | Tietê | 36.211 |
| SP | 35 | 54607 | Timburi | 2.548 |
| SP | 35 | 54656 | Torre de Pedra | 3.149 |
| SP | 35 | 54706 | Torrinha | 9.278 |
| SP | 35 | 54755 | Trabiju | 1.519 |
| SP | 35 | 54805 | Tremembé | 41.159 |
| SP | 35 | 54904 | Três Fronteiras | 5.163 |
| SP | 35 | 54953 | Tuiuti | 6.284 |
| SP | 35 | 55000 | Tupã | 64.098 |
| SP | 35 | 55109 | Tupi Paulista | 14.389 |
| SP | 35 | 55208 | Turiúba | 2.040 |
| SP | 35 | 55307 | Turmalina | 1.958 |
| SP | 35 | 55356 | Ubarana | 4.855 |
| SP | 35 | 55406 | Ubatuba | 81.096 |
| SP | 35 | 55505 | Ubirajara | 4.472 |
| SP | 35 | 55604 | Uchoa | 9.818 |
| SP | 35 | 55703 | União Paulista | 1.522 |
| SP | 35 | 55802 | Urânia | 9.007 |
| SP | 35 | 55901 | Uru | 1.395 |

| U.F. | | MUNICÍPIOS | | POPULAÇÃO ESTIMADA |
|---|---|---|---|---|
| SIGLA | COD | COD | NOMES | |
| SP | 35 | 56008 | Urupês | 12.388 |
| SP | 35 | 56107 | Valentim Gentil | 10.082 |
| SP | 35 | 56206 | Valinhos | 107.481 |
| SP | 35 | 56305 | Valparaíso | 22.514 |
| SP | 35 | 56354 | Vargem | 7.098 |
| SP | 35 | 56404 | Vargem Grande do Sul | 39.160 |
| SP | 35 | 56453 | Vargem Grande Paulista | 44.754 |
| SP | 35 | 56503 | Várzea Paulista | 107.211 |
| SP | 35 | 56602 | Vera Cruz | 9.952 |
| SP | 35 | 56701 | Vinhedo | 63.729 |
| SP | 35 | 56800 | Viradouro | 18.110 |
| SP | 35 | 56909 | Vista Alegre do Alto | 6.874 |
| SP | 35 | 56958 | Vitória Brasil | 1.662 |
| SP | 35 | 57006 | Votorantim | 105.193 |
| SP | 35 | 57105 | Votuporanga | 81.279 |
| SP | 35 | 57154 | Zacarias | 2.427 |
| PR | 41 | 103 | Abatiá | 7.928 |
| PR | 41 | 202 | Adrianópolis | 6.856 |
| PR | 41 | 301 | Agudos do Sul | 8.735 |
| PR | 41 | 400 | Almirante Tamandaré | 97.523 |
| PR | 41 | 459 | Altamira do Paraná | 3.799 |
| PR | 41 | 28625 | Alto Paraíso | 3.217 |
| PR | 41 | 608 | Alto Paraná | 13.435 |
| PR | 41 | 707 | Alto Piquiri | 10.406 |
| PR | 41 | 509 | Altônia | 20.764 |
| PR | 41 | 806 | Alvorada do Sul | 9.255 |
| PR | 41 | 905 | Amaporã | 5.447 |
| PR | 41 | 1002 | Ampére | 18.041 |
| PR | 41 | 1051 | Anahy | 2.926 |
| PR | 41 | 1101 | Andirá | 21.964 |
| PR | 41 | 1150 | Ângulo | 2.893 |
| PR | 41 | 1200 | Antonina | 17.743 |
| PR | 41 | 1309 | Antônio Olinto | 7.750 |
| PR | 41 | 1408 | Apucarana | 121.290 |
| PR | 41 | 1507 | Arapongas | 103.025 |
| PR | 41 | 1606 | Arapoti | 26.998 |
| PR | 41 | 1655 | Arapuã | 4.017 |
| PR | 41 | 1705 | Araruna | 12.962 |
| PR | 41 | 1804 | Araucária | 117.964 |
| PR | 41 | 1853 | Ariranha do Ivaí | 2.533 |
| PR | 41 | 1903 | Assaí | 16.112 |
| PR | 41 | 2000 | Assis Chateaubriand | 33.023 |
| PR | 41 | 2109 | Astorga | 25.164 |
| PR | 41 | 2208 | Atalaia | 3.645 |
| PR | 41 | 2307 | Balsa Nova | 11.252 |
| PR | 41 | 2406 | Bandeirantes | 32.994 |
| PR | 41 | 2505 | Barbosa Ferraz | 13.995 |
| PR | 41 | 2703 | Barra do Jacaré | 2.860 |
| PR | 41 | 2604 | Barracão | 9.267 |
| PR | 41 | 2752 | Bela Vista da Caroba | 4.176 |

| U.F. | | MUNICÍPIOS | | POPULAÇÃO ESTIMADA |
|---|---|---|---|---|
| SIGLA | COD | COD | NOMES | |
| PR | 41 | 2802 | Bela Vista do Paraíso | 15.496 |
| PR | 41 | 2901 | Bituruna | 16.803 |
| PR | 41 | 3008 | Boa Esperança | 4.742 |
| PR | 41 | 3024 | Boa Esperança do Iguaçu | 2.898 |
| PR | 41 | 3040 | Boa Ventura de São Roque | 6.964 |
| PR | 41 | 3057 | Boa Vista da Aparecida | 7.919 |
| PR | 41 | 3107 | Bocaiúva do Sul | 9.989 |
| PR | 41 | 3156 | Bom Jesus do Sul | 3.879 |
| PR | 41 | 3206 | Bom Sucesso | 6.676 |
| PR | 41 | 3222 | Bom Sucesso do Sul | 3.075 |
| PR | 41 | 3305 | Borrazópolis | 8.235 |
| PR | 41 | 3354 | Braganey | 6.209 |
| PR | 41 | 3370 | Brasilândia do Sul | 3.260 |
| PR | 41 | 3404 | Cafeara | 2.853 |
| PR | 41 | 3453 | Cafelândia | 14.034 |
| PR | 41 | 3479 | Cafezal do Sul | 4.313 |
| PR | 41 | 3503 | Califórnia | 7.767 |
| PR | 41 | 3602 | Cambará | 25.102 |
| PR | 41 | 3701 | Cambé | 97.329 |
| PR | 41 | 3800 | Cambira | 7.142 |
| PR | 41 | 3909 | Campina da Lagoa | 16.243 |
| PR | 41 | 3958 | Campina do Simão | 4.272 |
| PR | 41 | 4006 | Campina Grande do Sul | 36.825 |
| PR | 41 | 4105 | Campo do Tenente | 6.715 |
| PR | 41 | 4055 | Campo Bonito | 4.385 |
| PR | 41 | 4204 | Campo Largo | 112.548 |
| PR | 41 | 4253 | Campo Magro | 23.607 |
| PR | 41 | 4303 | Campo Mourão | 85.896 |
| PR | 41 | 4402 | Cândido de Abreu | 18.100 |
| PR | 41 | 4428 | Candói | 16.271 |
| PR | 41 | 4451 | Cantagalo | 12.733 |
| PR | 41 | 4501 | Capanema | 18.681 |
| PR | 41 | 4600 | Capitão Leônidas Marques | 13.870 |
| PR | 41 | 4659 | Carambeí | 17.537 |
| PR | 41 | 4709 | Carlópolis | 13.581 |
| PR | 41 | 4808 | Cascavel | 296.254 |
| PR | 41 | 4907 | Castro | 68.071 |
| PR | 41 | 5003 | Catanduvas | 9.673 |
| PR | 41 | 5102 | Centenário do Sul | 11.474 |
| PR | 41 | 5201 | Cerro Azul | 18.660 |
| PR | 41 | 5300 | Céu Azul | 11.413 |
| PR | 41 | 5409 | Chopinzinho | 19.517 |
| PR | 41 | 5508 | Cianorte | 68.629 |
| PR | 41 | 5607 | Cidade Gaúcha | 11.079 |
| PR | 41 | 5706 | Clevelândia | 17.995 |
| PR | 41 | 5805 | Colombo | 247.268 |
| PR | 41 | 5904 | Colorado | 21.789 |
| PR | 41 | 6001 | Congonhinhas | 9.035 |
| PR | 41 | 6100 | Conselheiro Mairinck | 3.700 |
| PR | 41 | 6209 | Contenda | 15.728 |

| U.F. | | MUNICÍPIOS | | POPULAÇÃO ESTIMADA |
|---|---|---|---|---|
| SIGLA | COD | COD | NOMES | |
| PR | 41 | 6308 | Corbélia | 15.850 |
| PR | 41 | 6407 | Cornélio Procópio | 48.544 |
| PR | 41 | 6456 | Coronel Domingos Soares | 7.864 |
| PR | 41 | 6506 | Coronel Vivida | 21.830 |
| PR | 41 | 6555 | Corumbataí do Sul | 4.220 |
| PR | 41 | 6803 | Cruz Machado | 19.132 |
| PR | 41 | 6571 | Cruzeiro do Iguaçu | 4.225 |
| PR | 41 | 6605 | Cruzeiro do Oeste | 20.856 |
| PR | 41 | 6704 | Cruzeiro do Sul | 4.574 |
| PR | 41 | 6852 | Cruzmaltina | 3.128 |
| PR | 41 | 6902 | Curitiba | 1.851.215 |
| PR | 41 | 7009 | Curiúva | 15.217 |
| PR | 41 | 7108 | Diamante do Norte | 5.668 |
| PR | 41 | 7124 | Diamante do Sul | 3.791 |
| PR | 41 | 7157 | Diamante D'Oeste | 5.129 |
| PR | 41 | 7207 | Dois Vizinhos | 35.707 |
| PR | 41 | 7256 | Douradina | 6.853 |
| PR | 41 | 7306 | Doutor Camargo | 5.753 |
| PR | 41 | 28633 | Doutor Ulysses | 6.145 |
| PR | 41 | 7405 | Enéas Marques | 6.066 |
| PR | 41 | 7504 | Engenheiro Beltrão | 14.280 |
| PR | 41 | 7538 | Entre Rios do Oeste | 4.113 |
| PR | 41 | 7520 | Esperança Nova | 1.836 |
| PR | 41 | 7546 | Espigão Alto do Iguaçu | 5.200 |
| PR | 41 | 7553 | Farol | 3.354 |
| PR | 41 | 7603 | Faxinal | 16.032 |
| PR | 41 | 7652 | Fazenda Rio Grande | 80.868 |
| PR | 41 | 7702 | Fênix | 5.017 |
| PR | 41 | 7736 | Fernandes Pinheiro | 5.696 |
| PR | 41 | 7751 | Figueira | 8.485 |
| PR | 41 | 7850 | Flor da Serra do Sul | 4.742 |
| PR | 41 | 7801 | Floraí | 5.158 |
| PR | 41 | 7900 | Floresta | 5.417 |
| PR | 41 | 8007 | Florestópolis | 11.795 |
| PR | 41 | 8106 | Flórida | 2.535 |
| PR | 41 | 8205 | Formosa do Oeste | 7.453 |
| PR | 41 | 8304 | Foz do Iguaçu | 325.137 |
| PR | 41 | 8452 | Foz do Jordão | 5.881 |
| PR | 41 | 8320 | Francisco Alves | 6.389 |
| PR | 41 | 8403 | Francisco Beltrão | 76.311 |
| PR | 41 | 8502 | General Carneiro | 15.275 |
| PR | 41 | 8551 | Godoy Moreira | 3.616 |
| PR | 41 | 8601 | Goioerê | 29.704 |
| PR | 41 | 8650 | Goioxim | 8.240 |
| PR | 41 | 8700 | Grandes Rios | 7.839 |
| PR | 41 | 8809 | Guaíra | 29.664 |
| PR | 41 | 8908 | Guairaçá | 5.867 |
| PR | 41 | 8957 | Guamiranga | 7.918 |
| PR | 41 | 9005 | Guapirama | 3.645 |
| PR | 41 | 9104 | Guaporema | 2.250 |

| U.F. | | MUNICÍPIOS | | POPULAÇÃO |
|---|---|---|---|---|
| SIGLA | COD | COD | NOMES | ESTIMADA |
| PR | 41 | 9203 | Guaraci | 5.183 |
| PR | 41 | 9302 | Guaraniaçu | 16.161 |
| PR | 41 | 9401 | Guarapuava | 172.728 |
| PR | 41 | 9500 | Guaraqueçaba | 7.843 |
| PR | 41 | 9609 | Guaratuba | 32.806 |
| PR | 41 | 9658 | Honório Serpa | 6.180 |
| PR | 41 | 9708 | Ibaiti | 29.441 |
| PR | 41 | 9757 | Ibema | 6.144 |
| PR | 41 | 9807 | Ibiporã | 47.514 |
| PR | 41 | 9906 | Icaraíma | 9.296 |
| PR | 41 | 10003 | Iguaraçu | 3.907 |
| PR | 41 | 10052 | Iguatu | 2.372 |
| PR | 41 | 10078 | Imbaú | 12.040 |
| PR | 41 | 10102 | Imbituva | 28.660 |
| PR | 41 | 10201 | Inácio Martins | 11.431 |
| PR | 41 | 10300 | Inajá | 2.876 |
| PR | 41 | 10409 | Indianópolis | 4.258 |
| PR | 41 | 10508 | Ipiranga | 14.655 |
| PR | 41 | 10607 | Iporã | 15.227 |
| PR | 41 | 10656 | Iracema do Oeste | 2.567 |
| PR | 41 | 10706 | Irati | 56.483 |
| PR | 41 | 10805 | Iretama | 11.510 |
| PR | 41 | 10904 | Itaguajé | 4.589 |
| PR | 41 | 10953 | Itaipulândia | 9.349 |
| PR | 41 | 11001 | Itambaracá | 7.128 |
| PR | 41 | 11100 | Itambé | 6.082 |
| PR | 41 | 11209 | Itapejara d'Oeste | 11.270 |
| PR | 41 | 11258 | Itaperuçu | 23.501 |
| PR | 41 | 11308 | Itaúna do Sul | 3.621 |
| PR | 41 | 11407 | Ivaí | 13.533 |
| PR | 41 | 11506 | Ivaiporã | 32.157 |
| PR | 41 | 11555 | Ivaté | 8.294 |
| PR | 41 | 11605 | Ivatuba | 2.786 |
| PR | 41 | 11704 | Jaboti | 5.307 |
| PR | 41 | 11803 | Jacarezinho | 40.582 |
| PR | 41 | 11902 | Jaguapitã | 12.414 |
| PR | 41 | 12009 | Jaguariaíva | 33.244 |
| PR | 41 | 12108 | Jandaia do Sul | 20.159 |
| PR | 41 | 12207 | Janiópolis | 6.983 |
| PR | 41 | 12306 | Japira | 4.797 |
| PR | 41 | 12405 | Japurá | 8.663 |
| PR | 41 | 12504 | Jardim Alegre | 14.976 |
| PR | 41 | 12603 | Jardim Olinda | 1.494 |
| PR | 41 | 12702 | Jataizinho | 11.604 |
| PR | 41 | 12751 | Jesuítas | 8.849 |
| PR | 41 | 12801 | Joaquim Távora | 10.755 |
| PR | 41 | 12900 | Jundiaí do Sul | 3.777 |
| PR | 41 | 12959 | Juranda | 7.822 |
| PR | 41 | 13007 | Jussara | 6.240 |
| PR | 41 | 13106 | Kaloré | 4.718 |

| U.F. | | MUNICÍPIOS | | POPULAÇÃO ESTIMADA |
|---|---|---|---|---|
| SIGLA | COD | COD | NOMES | |
| PR | 41 | 13205 | Lapa | 42.933 |
| PR | 41 | 13254 | Laranjal | 6.339 |
| PR | 41 | 13304 | Laranjeiras do Sul | 31.641 |
| PR | 41 | 13403 | Leópolis | 4.316 |
| PR | 41 | 13429 | Lidianópolis | 4.083 |
| PR | 41 | 13452 | Lindoeste | 5.419 |
| PR | 41 | 13502 | Loanda | 20.103 |
| PR | 41 | 13601 | Lobato | 4.405 |
| PR | 41 | 13700 | Londrina | 510.707 |
| PR | 41 | 13734 | Luiziana | 7.357 |
| PR | 41 | 13759 | Lunardelli | 5.094 |
| PR | 41 | 13809 | Lupionópolis | 4.538 |
| PR | 41 | 13908 | Mallet | 12.784 |
| PR | 41 | 14005 | Mamborê | 14.332 |
| PR | 41 | 14104 | Mandaguaçu | 19.270 |
| PR | 41 | 14203 | Mandaguari | 33.109 |
| PR | 41 | 14302 | Mandirituba | 21.885 |
| PR | 41 | 14351 | Manfrinópolis | 3.283 |
| PR | 41 | 14401 | Mangueirinha | 17.526 |
| PR | 41 | 14500 | Manoel Ribas | 13.113 |
| PR | 41 | 14609 | Marechal Cândido Rondon | 47.048 |
| PR | 41 | 14708 | Maria Helena | 6.115 |
| PR | 41 | 14807 | Marialva | 31.397 |
| PR | 41 | 14906 | Marilândia do Sul | 9.197 |
| PR | 41 | 15002 | Marilena | 6.704 |
| PR | 41 | 15101 | Mariluz | 10.861 |
| PR | 41 | 15200 | Maringá | 335.511 |
| PR | 41 | 15309 | Mariópolis | 5.944 |
| PR | 41 | 15358 | Maripá | 5.673 |
| PR | 41 | 15408 | Marmeleiro | 13.463 |
| PR | 41 | 15457 | Marquinho | 5.258 |
| PR | 41 | 15507 | Marumbi | 4.154 |
| PR | 41 | 15606 | Matelândia | 16.217 |
| PR | 41 | 15705 | Matinhos | 23.925 |
| PR | 41 | 15739 | Mato Rico | 4.205 |
| PR | 41 | 15754 | Mauá da Serra | 8.446 |
| PR | 41 | 15804 | Medianeira | 39.857 |
| PR | 41 | 15853 | Mercedes | 4.902 |
| PR | 41 | 15903 | Mirador | 2.371 |
| PR | 41 | 16000 | Miraselva | 1.946 |
| PR | 41 | 16059 | Missal | 10.760 |
| PR | 41 | 16109 | Moreira Sales | 13.238 |
| PR | 41 | 16208 | Morretes | 17.000 |
| PR | 41 | 16307 | Munhoz de Melo | 3.714 |
| PR | 41 | 16406 | Nossa Senhora das Graças | 4.055 |
| PR | 41 | 16505 | Nova Aliança do Ivaí | 1.434 |
| PR | 41 | 16604 | Nova América da Colina | 3.331 |
| PR | 41 | 16703 | Nova Aurora | 11.636 |
| PR | 41 | 16802 | Nova Cantu | 7.481 |
| PR | 41 | 16901 | Nova Esperança | 26.591 |

| U.F. | | MUNICÍPIOS | | POPULAÇÃO ESTIMADA |
|---|---|---|---|---|
| SIGLA | COD | COD | NOMES | |
| PR | 41 | 16950 | Nova Esperança do Sudoeste | 5.337 |
| PR | 41 | 17008 | Nova Fátima | 8.259 |
| PR | 41 | 17057 | Nova Laranjeiras | 11.577 |
| PR | 41 | 17107 | Nova Londrina | 12.898 |
| PR | 41 | 17206 | Nova Olímpia | 5.391 |
| PR | 41 | 17255 | Nova Prata do Iguaçu | 10.822 |
| PR | 41 | 17214 | Nova Santa Bárbara | 3.984 |
| PR | 41 | 17222 | Nova Santa Rosa | 7.965 |
| PR | 41 | 17271 | Nova Tebas | 8.283 |
| PR | 41 | 17297 | Novo Itacolomi | 2.808 |
| PR | 41 | 17305 | Ortigueira | 25.002 |
| PR | 41 | 17404 | Ourizona | 3.380 |
| PR | 41 | 17453 | Ouro Verde do Oeste | 5.648 |
| PR | 41 | 17503 | Paiçandu | 36.876 |
| PR | 41 | 17602 | Palmas | 43.409 |
| PR | 41 | 17701 | Palmeira | 32.401 |
| PR | 41 | 17800 | Palmital | 15.548 |
| PR | 41 | 17909 | Palotina | 28.966 |
| PR | 41 | 18006 | Paraíso do Norte | 11.939 |
| PR | 41 | 18105 | Paranacity | 9.946 |
| PR | 41 | 18204 | Paranaguá | 139.796 |
| PR | 41 | 18303 | Paranapoema | 2.818 |
| PR | 41 | 18402 | Paranavaí | 82.716 |
| PR | 41 | 18451 | Pato Bragado | 4.947 |
| PR | 41 | 18501 | Pato Branco | 70.160 |
| PR | 41 | 18600 | Paula Freitas | 5.751 |
| PR | 41 | 18709 | Paulo Frontin | 7.398 |
| PR | 41 | 18808 | Peabiru | 13.347 |
| PR | 41 | 18857 | Perobal | 5.163 |
| PR | 41 | 18907 | Pérola | 9.665 |
| PR | 41 | 19004 | Pérola d'Oeste | 7.202 |
| PR | 41 | 19103 | Piên | 11.811 |
| PR | 41 | 19152 | Pinhais | 118.319 |
| PR | 41 | 19251 | Pinhal de São Bento | 2.600 |
| PR | 41 | 19202 | Pinhalão | 6.005 |
| PR | 41 | 19301 | Pinhão | 30.295 |
| PR | 41 | 19400 | Piraí do Sul | 24.374 |
| PR | 41 | 19509 | Piraquara | 87.285 |
| PR | 41 | 19608 | Pitanga | 35.052 |
| PR | 41 | 19657 | Pitangueiras | 2.822 |
| PR | 41 | 19707 | Planaltina do Paraná | 3.864 |
| PR | 41 | 19806 | Planalto | 13.983 |
| PR | 41 | 19905 | Ponta Grossa | 314.681 |
| PR | 41 | 19954 | Pontal do Paraná | 17.820 |
| PR | 41 | 20002 | Porecatu | 14.189 |
| PR | 41 | 20101 | Porto Amazonas | 4.348 |
| PR | 41 | 20150 | Porto Barreiro | 3.768 |
| PR | 41 | 20200 | Porto Rico | 2.521 |
| PR | 41 | 20309 | Porto Vitória | 3.833 |
| PR | 41 | 20333 | Prado Ferreira | 3.510 |

| U.F. | | MUNICÍPIOS | | POPULAÇÃO ESTIMADA |
|---|---|---|---|---|
| SIGLA | COD | COD | NOMES | |
| PR | 41 | 20358 | Pranchita | 5.885 |
| PR | 41 | 20408 | Presidente Castelo Branco | 4.934 |
| PR | 41 | 20507 | Primeiro de Maio | 11.126 |
| PR | 41 | 20606 | Prudentópolis | 51.008 |
| PR | 41 | 20655 | Quarto Centenário | 4.881 |
| PR | 41 | 20705 | Quatiguá | 7.148 |
| PR | 41 | 20804 | Quatro Barras | 19.277 |
| PR | 41 | 20853 | Quatro Pontes | 3.800 |
| PR | 41 | 20903 | Quedas do Iguaçu | 31.976 |
| PR | 41 | 21000 | Querência do Norte | 12.306 |
| PR | 41 | 21109 | Quinta do Sol | 5.189 |
| PR | 41 | 21208 | Quitandinha | 16.608 |
| PR | 41 | 21257 | Ramilândia | 4.365 |
| PR | 41 | 21307 | Rancho Alegre | 4.083 |
| PR | 41 | 21356 | Rancho Alegre D'Oeste | 2.976 |
| PR | 41 | 21406 | Realeza | 16.288 |
| PR | 41 | 21505 | Rebouças | 14.637 |
| PR | 41 | 21604 | Renascença | 6.937 |
| PR | 41 | 21703 | Reserva | 25.148 |
| PR | 41 | 21752 | Reserva do Iguaçu | 7.449 |
| PR | 41 | 21901 | Ribeirão do Pinhal | 13.584 |
| PR | 41 | 21802 | Ribeirão Claro | 11.246 |
| PR | 41 | 22008 | Rio Azul | 13.760 |
| PR | 41 | 22107 | Rio Bom | 3.292 |
| PR | 41 | 22156 | Rio Bonito do Iguaçu | 15.121 |
| PR | 41 | 22172 | Rio Branco do Ivaí | 4.006 |
| PR | 41 | 22206 | Rio Branco do Sul | 33.142 |
| PR | 41 | 22305 | Rio Negro | 31.191 |
| PR | 41 | 22404 | Rolândia | 56.352 |
| PR | 41 | 22503 | Roncador | 12.309 |
| PR | 41 | 22602 | Rondon | 9.465 |
| PR | 41 | 22651 | Rosário do Ivaí | 5.813 |
| PR | 41 | 22701 | Sabáudia | 5.642 |
| PR | 41 | 22800 | Salgado Filho | 4.640 |
| PR | 41 | 22909 | Salto do Itararé | 5.134 |
| PR | 41 | 23006 | Salto do Lontra | 12.828 |
| PR | 41 | 23105 | Santa Amélia | 4.105 |
| PR | 41 | 23204 | Santa Cecília do Pavão | 3.695 |
| PR | 41 | 23303 | Santa Cruz de Monte Castelo | 8.015 |
| PR | 41 | 23402 | Santa Fé | 10.354 |
| PR | 41 | 23501 | Santa Helena | 24.198 |
| PR | 41 | 23600 | Santa Inês | 1.882 |
| PR | 41 | 23709 | Santa Isabel do Ivaí | 8.621 |
| PR | 41 | 23808 | Santa Izabel do Oeste | 11.747 |
| PR | 41 | 23824 | Santa Lúcia | 3.743 |
| PR | 41 | 23857 | Santa Maria do Oeste | 11.369 |
| PR | 41 | 23907 | Santa Mariana | 11.996 |
| PR | 41 | 23956 | Santa Mônica | 3.642 |
| PR | 41 | 24020 | Santa Tereza do Oeste | 9.320 |
| PR | 41 | 24053 | Santa Terezinha de Itaipu | 20.539 |

| U.F. | | MUNICÍPIOS | | POPULAÇÃO ESTIMADA |
|---|---|---|---|---|
| SIGLA | COD | COD | NOMES | |
| PR | 41 | 24004 | Santana do Itararé | 5.697 |
| PR | 41 | 24103 | Santo Antônio da Platina | 42.002 |
| PR | 41 | 24202 | Santo Antônio do Caiuá | 2.733 |
| PR | 41 | 24301 | Santo Antônio do Paraíso | 2.315 |
| PR | 41 | 24400 | Santo Antônio do Sudoeste | 19.386 |
| PR | 41 | 24509 | Santo Inácio | 4.956 |
| PR | 41 | 24608 | São Carlos do Ivaí | 5.990 |
| PR | 41 | 24707 | São Jerônimo da Serra | 11.905 |
| PR | 41 | 24806 | São João | 11.187 |
| PR | 41 | 24905 | São João do Caiuá | 6.152 |
| PR | 41 | 25001 | São João do Ivaí | 11.890 |
| PR | 41 | 25100 | São João do Triunfo | 14.399 |
| PR | 41 | 25209 | São Jorge d'Oeste | 9.195 |
| PR | 41 | 25308 | São Jorge do Ivaí | 5.382 |
| PR | 41 | 25357 | São Jorge do Patrocínio | 6.080 |
| PR | 41 | 25407 | São José da Boa Vista | 6.320 |
| PR | 41 | 25456 | São José das Palmeiras | 3.942 |
| PR | 41 | 25506 | São José dos Pinhais | 279.297 |
| PR | 41 | 25555 | São Manoel do Paraná | 2.170 |
| PR | 41 | 25605 | São Mateus do Sul | 41.188 |
| PR | 41 | 25704 | São Miguel do Iguaçu | 26.451 |
| PR | 41 | 25753 | São Pedro do Iguaçu | 6.561 |
| PR | 41 | 25803 | São Pedro do Ivaí | 9.921 |
| PR | 41 | 25902 | São Pedro do Paraná | 2.561 |
| PR | 41 | 26009 | São Sebastião da Amoreira | 9.012 |
| PR | 41 | 26108 | São Tomé | 5.522 |
| PR | 41 | 26207 | Sapopema | 6.800 |
| PR | 41 | 26256 | Sarandi | 84.651 |
| PR | 41 | 26272 | Saudade do Iguaçu | 5.187 |
| PR | 41 | 26306 | Sengés | 20.445 |
| PR | 41 | 26355 | Serranópolis do Iguaçu | 4.362 |
| PR | 41 | 26405 | Sertaneja | 5.924 |
| PR | 41 | 26504 | Sertanópolis | 16.103 |
| PR | 41 | 26603 | Siqueira Campos | 17.411 |
| PR | 41 | 26652 | Sulina | 3.434 |
| PR | 41 | 26678 | Tamarana | 11.577 |
| PR | 41 | 26702 | Tamboara | 4.804 |
| PR | 41 | 26801 | Tapejara | 15.367 |
| PR | 41 | 26900 | Tapira | 5.904 |
| PR | 41 | 27007 | Teixeira Soares | 10.548 |
| PR | 41 | 27106 | Telêmaco Borba | 69.278 |
| PR | 41 | 27205 | Terra Boa | 15.069 |
| PR | 41 | 27304 | Terra Rica | 15.060 |
| PR | 41 | 27403 | Terra Roxa | 16.734 |
| PR | 41 | 27502 | Tibagi | 19.421 |
| PR | 41 | 27601 | Tijucas do Sul | 13.762 |
| PR | 41 | 27700 | Toledo | 116.774 |
| PR | 41 | 27809 | Tomazina | 8.808 |
| PR | 41 | 27858 | Três Barras do Paraná | 12.088 |
| PR | 41 | 27882 | Tunas do Paraná | 6.753 |

| U.F. | | MUNICÍPIOS | | POPULAÇÃO ESTIMADA |
|---|---|---|---|---|
| SIGLA | COD | COD | NOMES | |
| PR | 41 | 27908 | Tuneiras do Oeste | 8.777 |
| PR | 41 | 27957 | Tupãssi | 7.947 |
| PR | 41 | 27965 | Turvo | 14.363 |
| PR | 41 | 28005 | Ubiratã | 21.558 |
| PR | 41 | 28104 | Umuarama | 99.606 |
| PR | 41 | 28203 | União da Vitória | 53.466 |
| PR | 41 | 28302 | Uniflor | 2.495 |
| PR | 41 | 28401 | Uraí | 11.774 |
| PR | 41 | 28534 | Ventania | 11.239 |
| PR | 41 | 28559 | Vera Cruz do Oeste | 9.257 |
| PR | 41 | 28609 | Verê | 8.078 |
| PR | 41 | 28658 | Virmond | 4.181 |
| PR | 41 | 28708 | Vitorino | 6.531 |
| PR | 41 | 28500 | Wenceslau Braz | 19.090 |
| PR | 41 | 28807 | Xambrê | 5.829 |
| SC | 42 | 51 | Abdon Batista | 2.805 |
| SC | 42 | 101 | Abelardo Luz | 16.899 |
| SC | 42 | 200 | Agrolândia | 9.661 |
| SC | 42 | 309 | Agronômica | 4.925 |
| SC | 42 | 408 | Água Doce | 6.959 |
| SC | 42 | 507 | Águas de Chapecó | 6.354 |
| SC | 42 | 556 | Águas Frias | 2.641 |
| SC | 42 | 606 | Águas Mornas | 4.503 |
| SC | 42 | 705 | Alfredo Wagner | 10.274 |
| SC | 42 | 754 | Alto Bela Vista | 2.071 |
| SC | 42 | 804 | Anchieta | 6.683 |
| SC | 42 | 903 | Angelina | 5.396 |
| SC | 42 | 1000 | Anita Garibaldi | 9.191 |
| SC | 42 | 1109 | Anitápolis | 3.267 |
| SC | 42 | 1208 | Antônio Carlos | 7.466 |
| SC | 42 | 1257 | Apiúna | 10.996 |
| SC | 42 | 1273 | Arabutã | 4.048 |
| SC | 42 | 1307 | Araquari | 23.080 |
| SC | 42 | 1406 | Araranguá | 59.537 |
| SC | 42 | 1505 | Armazém | 7.650 |
| SC | 42 | 1604 | Arroio Trinta | 3.638 |
| SC | 42 | 1653 | Arvoredo | 2.241 |
| SC | 42 | 1703 | Ascurra | 6.945 |
| SC | 42 | 1802 | Atalanta | 3.402 |
| SC | 42 | 1901 | Aurora | 5.560 |
| SC | 42 | 1950 | Balneário Arroio do Silva | 8.808 |
| SC | 42 | 2057 | Balneário Barra do Sul | 7.791 |
| SC | 42 | 2008 | Balneário Camboriú | 102.081 |
| SC | 42 | 2073 | Balneário Gaivota | 7.959 |
| SC | 42 | 12809 | Balneário Piçarras | 14.845 |
| SC | 42 | 2081 | Bandeirante | 3.095 |
| SC | 42 | 2099 | Barra Bonita | 2.120 |
| SC | 42 | 2107 | Barra Velha | 19.861 |
| SC | 42 | 2131 | Bela Vista do Toldo | 6.145 |
| SC | 42 | 2156 | Belmonte | 2.790 |

| U.F. | | MUNICÍPIOS | | POPULAÇÃO ESTIMADA |
|---|---|---|---|---|
| SIGLA | COD | COD | NOMES | |
| SC | 42 | 2206 | Benedito Novo | 10.335 |
| SC | 42 | 2305 | Biguaçu | 56.395 |
| SC | 42 | 2404 | Blumenau | 299.416 |
| SC | 42 | 2438 | Bocaina do Sul | 3.128 |
| SC | 42 | 2503 | Bom Jardim da Serra | 4.383 |
| SC | 42 | 2537 | Bom Jesus | 2.427 |
| SC | 42 | 2578 | Bom Jesus do Oeste | 2.065 |
| SC | 42 | 2602 | Bom Retiro | 8.594 |
| SC | 42 | 2453 | Bombinhas | 13.695 |
| SC | 42 | 2701 | Botuverá | 4.345 |
| SC | 42 | 2800 | Braço do Norte | 29.317 |
| SC | 42 | 2859 | Braço do Trombudo | 3.419 |
| SC | 42 | 2875 | Brunópolis | 2.943 |
| SC | 42 | 2909 | Brusque | 102.280 |
| SC | 42 | 3006 | Caçador | 70.720 |
| SC | 42 | 3105 | Caibi | 6.392 |
| SC | 42 | 3154 | Calmon | 4.265 |
| SC | 42 | 3204 | Camboriú | 57.793 |
| SC | 42 | 3303 | Campo Alegre | 11.713 |
| SC | 42 | 3402 | Campo Belo do Sul | 8.212 |
| SC | 42 | 3501 | Campo Erê | 9.737 |
| SC | 42 | 3600 | Campos Novos | 29.133 |
| SC | 42 | 3709 | Canelinha | 10.168 |
| SC | 42 | 3808 | Canoinhas | 54.645 |
| SC | 42 | 3253 | Capão Alto | 3.358 |
| SC | 42 | 3907 | Capinzal | 18.996 |
| SC | 42 | 3956 | Capivari de Baixo | 21.059 |
| SC | 42 | 4004 | Catanduvas | 9.119 |
| SC | 42 | 4103 | Caxambu do Sul | 4.963 |
| SC | 42 | 4152 | Celso Ramos | 2.720 |
| SC | 42 | 4178 | Cerro Negro | 4.045 |
| SC | 42 | 4194 | Chapadão do Lageado | 2.882 |
| SC | 42 | 4202 | Chapecó | 174.187 |
| SC | 42 | 4251 | Cocal do Sul | 15.229 |
| SC | 42 | 4301 | Concórdia | 70.393 |
| SC | 42 | 4350 | Cordilheira Alta | 3.531 |
| SC | 42 | 4400 | Coronel Freitas | 10.520 |
| SC | 42 | 4459 | Coronel Martins | 2.583 |
| SC | 42 | 4558 | Correia Pinto | 14.842 |
| SC | 42 | 4509 | Corupá | 13.380 |
| SC | 42 | 4608 | Criciúma | 188.557 |
| SC | 42 | 4707 | Cunha Porã | 11.079 |
| SC | 42 | 4756 | Cunhataí | 1.948 |
| SC | 42 | 4806 | Curitibanos | 39.045 |
| SC | 42 | 4905 | Descanso | 8.898 |
| SC | 42 | 5001 | Dionísio Cerqueira | 15.399 |
| SC | 42 | 5100 | Dona Emma | 3.583 |
| SC | 42 | 5159 | Doutor Pedrinho | 3.432 |
| SC | 42 | 5175 | Entre Rios | 3.104 |
| SC | 42 | 5191 | Ermo | 1.857 |

| U.F. | | MUNICÍPIOS | | POPULAÇÃO ESTIMADA |
|---|---|---|---|---|
| SIGLA | COD | COD | NOMES | |
| SC | 42 | 5209 | Erval Velho | 4.195 |
| SC | 42 | 5308 | Faxinal dos Guedes | 10.585 |
| SC | 42 | 5357 | Flor do Sertão | 1.700 |
| SC | 42 | 5407 | Florianópolis | 408.161 |
| SC | 42 | 5431 | Formosa do Sul | 2.683 |
| SC | 42 | 5456 | Forquilhinha | 21.928 |
| SC | 42 | 5506 | Fraiburgo | 36.469 |
| SC | 42 | 5555 | Frei Rogério | 2.673 |
| SC | 42 | 5605 | Galvão | 3.444 |
| SC | 42 | 5704 | Garopaba | 16.710 |
| SC | 42 | 5803 | Garuva | 14.281 |
| SC | 42 | 5902 | Gaspar | 55.489 |
| SC | 42 | 6009 | Governador Celso Ramos | 12.704 |
| SC | 42 | 6108 | Grão Pará | 6.278 |
| SC | 42 | 6207 | Gravatal | 10.793 |
| SC | 42 | 6306 | Guabiruba | 17.316 |
| SC | 42 | 6405 | Guaraciaba | 10.857 |
| SC | 42 | 6504 | Guaramirim | 31.910 |
| SC | 42 | 6603 | Guarujá do Sul | 4.870 |
| SC | 42 | 6652 | Guatambú | 4.610 |
| SC | 42 | 6702 | Herval d'Oeste | 19.323 |
| SC | 42 | 6751 | Ibiam | 2.060 |
| SC | 42 | 6801 | Ibicaré | 3.458 |
| SC | 42 | 6900 | Ibirama | 17.469 |
| SC | 42 | 7007 | Içara | 57.103 |
| SC | 42 | 7106 | Ilhota | 12.149 |
| SC | 42 | 7205 | Imaruí | 11.677 |
| SC | 42 | 7304 | Imbituba | 38.882 |
| SC | 42 | 7403 | Imbuia | 5.738 |
| SC | 42 | 7502 | Indaial | 50.917 |
| SC | 42 | 7577 | Iomerê | 2.643 |
| SC | 42 | 7601 | Ipira | 4.771 |
| SC | 42 | 7650 | Iporã do Oeste | 8.405 |
| SC | 42 | 7684 | Ipuaçu | 6.881 |
| SC | 42 | 7700 | Ipumirim | 7.400 |
| SC | 42 | 7759 | Iraceminha | 4.328 |
| SC | 42 | 7809 | Irani | 9.754 |
| SC | 42 | 7858 | Irati | 2.033 |
| SC | 42 | 7908 | Irineópolis | 10.748 |
| SC | 42 | 8005 | Itá | 6.552 |
| SC | 42 | 8104 | Itaiópolis | 20.551 |
| SC | 42 | 8203 | Itajaí | 172.081 |
| SC | 42 | 8302 | Itapema | 36.629 |
| SC | 42 | 8401 | Itapiranga | 16.015 |
| SC | 42 | 8450 | Itapoá | 11.489 |
| SC | 42 | 8500 | Ituporanga | 21.496 |
| SC | 42 | 8609 | Jaborá | 4.129 |
| SC | 42 | 8708 | Jacinto Machado | 11.051 |
| SC | 42 | 8807 | Jaguaruna | 16.418 |
| SC | 42 | 8906 | Jaraguá do Sul | 139.017 |

| U.F. | | MUNICÍPIOS | | POPULAÇÃO ESTIMADA |
|---|---|---|---|---|
| SIGLA | COD | COD | NOMES | |
| SC | 42 | 8955 | Jardinópolis | 1.881 |
| SC | 42 | 9003 | Joaçaba | 25.322 |
| SC | 42 | 9102 | Joinville | 497.331 |
| SC | 42 | 9151 | José Boiteux | 5.054 |
| SC | 42 | 9177 | Jupiá | 2.185 |
| SC | 42 | 9201 | Lacerdópolis | 2.266 |
| SC | 42 | 9300 | Lages | 167.805 |
| SC | 42 | 9409 | Laguna | 51.691 |
| SC | 42 | 9458 | Lajeado Grande | 1.485 |
| SC | 42 | 9508 | Laurentino | 5.757 |
| SC | 42 | 9607 | Lauro Muller | 14.173 |
| SC | 42 | 9706 | Lebon Régis | 12.134 |
| SC | 42 | 9805 | Leoberto Leal | 3.674 |
| SC | 42 | 9854 | Lindóia do Sul | 4.662 |
| SC | 42 | 9904 | Lontras | 9.660 |
| SC | 42 | 10001 | Luiz Alves | 9.506 |
| SC | 42 | 10035 | Luzerna | 5.528 |
| SC | 42 | 10050 | Macieira | 1.787 |
| SC | 42 | 10100 | Mafra | 52.933 |
| SC | 42 | 10209 | Major Gercino | 2.869 |
| SC | 42 | 10308 | Major Vieira | 7.675 |
| SC | 42 | 10407 | Maracajá | 6.185 |
| SC | 42 | 10506 | Maravilha | 23.099 |
| SC | 42 | 10555 | Marema | 2.275 |
| SC | 42 | 10605 | Massaranduba | 14.500 |
| SC | 42 | 10704 | Matos Costa | 2.826 |
| SC | 42 | 10803 | Meleiro | 7.063 |
| SC | 42 | 10852 | Mirim Doce | 2.583 |
| SC | 42 | 10902 | Modelo | 3.862 |
| SC | 42 | 11009 | Mondaí | 9.515 |
| SC | 42 | 11058 | Monte Carlo | 9.144 |
| SC | 42 | 11108 | Monte Castelo | 8.328 |
| SC | 42 | 11207 | Morro da Fumaça | 16.128 |
| SC | 42 | 11256 | Morro Grande | 2.775 |
| SC | 42 | 11306 | Navegantes | 57.324 |
| SC | 42 | 11405 | Nova Erechim | 4.381 |
| SC | 42 | 11454 | Nova Itaberaba | 4.222 |
| SC | 42 | 11504 | Nova Trento | 12.025 |
| SC | 42 | 11603 | Nova Veneza | 13.177 |
| SC | 42 | 11652 | Novo Horizonte | 2.954 |
| SC | 42 | 11702 | Orleans | 21.731 |
| SC | 42 | 11751 | Otacílio Costa | 16.587 |
| SC | 42 | 11801 | Ouro | 7.231 |
| SC | 42 | 11850 | Ouro Verde | 2.179 |
| SC | 42 | 11876 | Paial | 1.830 |
| SC | 42 | 11892 | Painel | 2.357 |
| SC | 42 | 11900 | Palhoça | 130.878 |
| SC | 42 | 12007 | Palma Sola | 8.145 |
| SC | 42 | 12056 | Palmeira | 2.456 |
| SC | 42 | 12106 | Palmitos | 16.596 |

| U.F. | | MUNICÍPIOS | | POPULAÇÃO ESTIMADA |
|---|---|---|---|---|
| SIGLA | COD | COD | NOMES | |
| SC | 42 | 12205 | Papanduva | 17.670 |
| SC | 42 | 12239 | Paraíso | 4.200 |
| SC | 42 | 12254 | Passo de Torres | 5.690 |
| SC | 42 | 12270 | Passos Maia | 4.555 |
| SC | 42 | 12304 | Paulo Lopes | 7.255 |
| SC | 42 | 12403 | Pedras Grandes | 4.515 |
| SC | 42 | 12502 | Penha | 22.263 |
| SC | 42 | 12601 | Peritiba | 2.977 |
| SC | 42 | 12700 | Petrolândia | 6.188 |
| SC | 42 | 12908 | Pinhalzinho | 15.692 |
| SC | 42 | 13005 | Pinheiro Preto | 3.048 |
| SC | 42 | 13104 | Piratuba | 4.446 |
| SC | 42 | 13153 | Planalto Alegre | 2.767 |
| SC | 42 | 13203 | Pomerode | 26.788 |
| SC | 42 | 13302 | Ponte Alta | 5.228 |
| SC | 42 | 13351 | Ponte Alta do Norte | 3.677 |
| SC | 42 | 13401 | Ponte Serrada | 11.724 |
| SC | 42 | 13500 | Porto Belo | 14.228 |
| SC | 42 | 13609 | Porto União | 33.408 |
| SC | 42 | 13708 | Pouso Redondo | 14.510 |
| SC | 42 | 13807 | Praia Grande | 7.318 |
| SC | 42 | 13906 | Presidente Castello Branco | 1.779 |
| SC | 42 | 14003 | Presidente Getúlio | 14.392 |
| SC | 42 | 14102 | Presidente Nereu | 2.324 |
| SC | 42 | 14151 | Princesa | 2.687 |
| SC | 42 | 14201 | Quilombo | 11.259 |
| SC | 42 | 14300 | Rancho Queimado | 2.893 |
| SC | 42 | 14409 | Rio das Antas | 6.237 |
| SC | 42 | 14508 | Rio do Campo | 6.135 |
| SC | 42 | 14607 | Rio do Oeste | 7.033 |
| SC | 42 | 14805 | Rio do Sul | 59.962 |
| SC | 42 | 14706 | Rio dos Cedros | 10.170 |
| SC | 42 | 14904 | Rio Fortuna | 4.648 |
| SC | 42 | 15000 | Rio Negrinho | 44.633 |
| SC | 42 | 15059 | Rio Rufino | 2.518 |
| SC | 42 | 15075 | Riqueza | 5.126 |
| SC | 42 | 15109 | Rodeio | 11.215 |
| SC | 42 | 15208 | Romelândia | 5.760 |
| SC | 42 | 15307 | Salete | 7.737 |
| SC | 42 | 15356 | Saltinho | 4.178 |
| SC | 42 | 15406 | Salto Veloso | 4.367 |
| SC | 42 | 15455 | Sangão | 11.121 |
| SC | 42 | 15505 | Santa Cecília | 15.928 |
| SC | 42 | 15554 | Santa Helena | 2.484 |
| SC | 42 | 15604 | Santa Rosa de Lima | 2.103 |
| SC | 42 | 15653 | Santa Rosa do Sul | 8.241 |
| SC | 42 | 15679 | Santa Terezinha | 9.363 |
| SC | 42 | 15687 | Santa Terezinha do Progresso | 3.062 |
| SC | 42 | 15695 | Santiago do Sul | 1.443 |
| SC | 42 | 15703 | Santo Amaro da Imperatriz | 18.436 |

| U.F. | | MUNICÍPIOS | | POPULAÇÃO ESTIMADA |
|---|---|---|---|---|
| SIGLA | COD | COD | NOMES | |
| SC | 42 | 15802 | São Bento do Sul | 76.514 |
| SC | 42 | 15752 | São Bernardino | 2.633 |
| SC | 42 | 15901 | São Bonifácio | 3.274 |
| SC | 42 | 16008 | São Carlos | 10.938 |
| SC | 42 | 16057 | São Cristovão do Sul | 5.087 |
| SC | 42 | 16107 | São Domingos | 9.611 |
| SC | 42 | 16206 | São Francisco do Sul | 40.030 |
| SC | 42 | 16354 | São João do Itaperiú | 3.426 |
| SC | 42 | 16255 | São João do Oeste | 6.269 |
| SC | 42 | 16404 | São João do Sul | 7.174 |
| SC | 42 | 16305 | São João Batista | 24.419 |
| SC | 42 | 16503 | São Joaquim | 25.122 |
| SC | 42 | 16602 | São José | 201.746 |
| SC | 42 | 16701 | São José do Cedro | 14.155 |
| SC | 42 | 16800 | São José do Cerrito | 10.624 |
| SC | 42 | 16909 | São Lourenço do Oeste | 23.015 |
| SC | 42 | 17006 | São Ludgero | 10.951 |
| SC | 42 | 17105 | São Martinho | 3.281 |
| SC | 42 | 17154 | São Miguel da Boa Vista | 2.026 |
| SC | 42 | 17204 | São Miguel do Oeste | 35.249 |
| SC | 42 | 17253 | São Pedro de Alcântara | 5.183 |
| SC | 42 | 17303 | Saudades | 8.929 |
| SC | 42 | 17402 | Schroeder | 14.000 |
| SC | 42 | 17501 | Seara | 17.827 |
| SC | 42 | 17550 | Serra Alta | 3.277 |
| SC | 42 | 17600 | Siderópolis | 12.967 |
| SC | 42 | 17709 | Sombrio | 25.553 |
| SC | 42 | 17758 | Sul Brasil | 3.150 |
| SC | 42 | 17808 | Taió | 17.522 |
| SC | 42 | 17907 | Tangará | 8.611 |
| SC | 42 | 17956 | Tigrinhos | 1.768 |
| SC | 42 | 18004 | Tijucas | 29.674 |
| SC | 42 | 18103 | Timbé do Sul | 5.260 |
| SC | 42 | 18202 | Timbó | 35.303 |
| SC | 42 | 18251 | Timbó Grande | 7.315 |
| SC | 42 | 18301 | Três Barras | 18.708 |
| SC | 42 | 18350 | Treviso | 3.692 |
| SC | 42 | 18400 | Treze de Maio | 6.791 |
| SC | 42 | 18509 | Treze Tílias | 6.004 |
| SC | 42 | 18608 | Trombudo Central | 6.520 |
| SC | 42 | 18707 | Tubarão | 96.529 |
| SC | 42 | 18756 | Tunápolis | 4.775 |
| SC | 42 | 18806 | Turvo | 11.427 |
| SC | 42 | 18855 | União do Oeste | 3.084 |
| SC | 42 | 18905 | Urubici | 10.825 |
| SC | 42 | 18954 | Urupema | 2.578 |
| SC | 42 | 19002 | Urussanga | 19.936 |
| SC | 42 | 19101 | Vargeão | 3.685 |
| SC | 42 | 19150 | Vargem | 3.187 |
| SC | 42 | 19176 | Vargem Bonita | 4.279 |

| U.F. | | MUNICÍPIOS | | POPULAÇÃO ESTIMADA |
|---|---|---|---|---|
| SIGLA | COD | COD | NOMES | |
| SC | 42 | 19200 | Vidal Ramos | 6.112 |
| SC | 42 | 19309 | Videira | 46.585 |
| SC | 42 | 19358 | Vitor Meireles | 5.756 |
| SC | 42 | 19408 | Witmarsum | 3.584 |
| SC | 42 | 19507 | Xanxerê | 42.174 |
| SC | 42 | 19606 | Xavantina | 4.316 |
| SC | 42 | 19705 | Xaxim | 25.444 |
| SC | 42 | 19853 | Zortéa | 3.015 |
| RS | 43 | 34 | Aceguá | 4.347 |
| RS | 43 | 59 | Água Santa | 3.619 |
| RS | 43 | 109 | Agudo | 17.063 |
| RS | 43 | 208 | Ajuricaba | 7.374 |
| RS | 43 | 307 | Alecrim | 7.259 |
| RS | 43 | 406 | Alegrete | 78.984 |
| RS | 43 | 455 | Alegria | 4.775 |
| RS | 43 | 471 | Almirante Tamandaré do Sul | 2.208 |
| RS | 43 | 505 | Alpestre | 8.880 |
| RS | 43 | 554 | Alto Alegre | 1.945 |
| RS | 43 | 570 | Alto Feliz | 3.067 |
| RS | 43 | 604 | Alvorada | 213.894 |
| RS | 43 | 638 | Amaral Ferrador | 6.602 |
| RS | 43 | 646 | Ametista do Sul | 8.538 |
| RS | 43 | 661 | André da Rocha | 1.276 |
| RS | 43 | 703 | Anta Gorda | 6.326 |
| RS | 43 | 802 | Antônio Prado | 14.274 |
| RS | 43 | 851 | Arambaré | 3.929 |
| RS | 43 | 877 | Araricá | 5.181 |
| RS | 43 | 901 | Aratiba | 6.690 |
| RS | 43 | 1008 | Arroio do Meio | 19.059 |
| RS | 43 | 1073 | Arroio do Padre | 2.882 |
| RS | 43 | 1057 | Arroio do Sal | 7.291 |
| RS | 43 | 1206 | Arroio do Tigre | 13.208 |
| RS | 43 | 1107 | Arroio dos Ratos | 14.181 |
| RS | 43 | 1305 | Arroio Grande | 18.748 |
| RS | 43 | 1404 | Arvorezinha | 10.548 |
| RS | 43 | 1503 | Augusto Pestana | 7.361 |
| RS | 43 | 1552 | Áurea | 3.789 |
| RS | 43 | 1602 | Bagé | 115.745 |
| RS | 43 | 1636 | Balneário Pinhal | 11.840 |
| RS | 43 | 1651 | Barão | 5.444 |
| RS | 43 | 1701 | Barão de Cotegipe | 6.724 |
| RS | 43 | 1750 | Barão do Triunfo | 7.246 |
| RS | 43 | 1859 | Barra do Guarita | 3.067 |
| RS | 43 | 1875 | Barra do Quaraí | 3.873 |
| RS | 43 | 1909 | Barra do Ribeiro | 11.762 |
| RS | 43 | 1925 | Barra do Rio Azul | 1.978 |
| RS | 43 | 1958 | Barra Funda | 2.453 |
| RS | 43 | 1800 | Barracão | 5.401 |
| RS | 43 | 2006 | Barros Cassal | 11.879 |
| RS | 43 | 2055 | Benjamin Constant do Sul | 2.188 |

| U.F. | | MUNICÍPIOS | | POPULAÇÃO ESTIMADA |
|---|---|---|---|---|
| SIGLA | COD | COD | NOMES | |
| RS | 43 | 2105 | Bento Gonçalves | 106.999 |
| RS | 43 | 2154 | Boa Vista das Missões | 2.100 |
| RS | 43 | 2204 | Boa Vista do Buricá | 6.655 |
| RS | 43 | 2220 | Boa Vista do Cadeado | 2.525 |
| RS | 43 | 2238 | Boa Vista do Incra | 2.583 |
| RS | 43 | 2253 | Boa Vista do Sul | 2.700 |
| RS | 43 | 2303 | Bom Jesus | 12.201 |
| RS | 43 | 2352 | Bom Princípio | 11.731 |
| RS | 43 | 2378 | Bom Progresso | 2.404 |
| RS | 43 | 2402 | Bom Retiro do Sul | 11.623 |
| RS | 43 | 2451 | Boqueirão do Leão | 8.097 |
| RS | 43 | 2501 | Bossoroca | 7.885 |
| RS | 43 | 2584 | Bozano | 2.361 |
| RS | 43 | 2600 | Braga | 3.842 |
| RS | 43 | 2659 | Brochier | 4.876 |
| RS | 43 | 2709 | Butiá | 20.213 |
| RS | 43 | 2808 | Caçapava do Sul | 33.060 |
| RS | 43 | 2907 | Cacequi | 13.578 |
| RS | 43 | 3004 | Cachoeira do Sul | 86.557 |
| RS | 43 | 3103 | Cachoeirinha | 118.089 |
| RS | 43 | 3202 | Cacique Doble | 5.008 |
| RS | 43 | 3301 | Caibaté | 5.212 |
| RS | 43 | 3400 | Caiçara | 5.240 |
| RS | 43 | 3509 | Camaquã | 62.723 |
| RS | 43 | 3558 | Camargo | 2.549 |
| RS | 43 | 3608 | Cambará do Sul | 7.238 |
| RS | 43 | 3673 | Campestre da Serra | 3.328 |
| RS | 43 | 3707 | Campina das Missões | 6.352 |
| RS | 43 | 3806 | Campinas do Sul | 5.745 |
| RS | 43 | 3905 | Campo Bom | 59.366 |
| RS | 43 | 4002 | Campo Novo | 5.419 |
| RS | 43 | 4101 | Campos Borges | 3.693 |
| RS | 43 | 4200 | Candelária | 30.423 |
| RS | 43 | 4309 | Cândido Godói | 6.722 |
| RS | 43 | 4358 | Candiota | 8.576 |
| RS | 43 | 4408 | Canela | 41.115 |
| RS | 43 | 4507 | Canguçu | 56.064 |
| RS | 43 | 4606 | Canoas | 332.056 |
| RS | 43 | 4614 | Canudos do Vale | 2.004 |
| RS | 43 | 4630 | Capão da Canoa | 40.861 |
| RS | 43 | 4655 | Capão do Cipó | 3.485 |
| RS | 43 | 4663 | Capão do Leão | 24.458 |
| RS | 43 | 4622 | Capão Bonito do Sul | 1.877 |
| RS | 43 | 4689 | Capela de Santana | 11.617 |
| RS | 43 | 4697 | Capitão | 2.694 |
| RS | 43 | 4671 | Capivari do Sul | 3.528 |
| RS | 43 | 4713 | Caraá | 7.606 |
| RS | 43 | 4705 | Carazinho | 60.388 |
| RS | 43 | 4804 | Carlos Barbosa | 25.866 |
| RS | 43 | 4853 | Carlos Gomes | 1.718 |

| U.F. | | MUNICÍPIOS | | POPULAÇÃO ESTIMADA |
|---|---|---|---|---|
| SIGLA | COD | COD | NOMES | |
| RS | 43 | 4903 | Casca | 8.654 |
| RS | 43 | 4952 | Caseiros | 3.132 |
| RS | 43 | 5009 | Catuípe | 9.611 |
| RS | 43 | 5108 | Caxias do Sul | 410.166 |
| RS | 43 | 5116 | Centenário | 3.100 |
| RS | 43 | 5124 | Cerrito | 6.767 |
| RS | 43 | 5132 | Cerro Branco | 4.673 |
| RS | 43 | 5157 | Cerro Grande | 2.659 |
| RS | 43 | 5173 | Cerro Grande do Sul | 9.854 |
| RS | 43 | 5207 | Cerro Largo | 12.861 |
| RS | 43 | 5306 | Chapada | 9.673 |
| RS | 43 | 5355 | Charqueadas | 36.045 |
| RS | 43 | 5371 | Charrua | 3.642 |
| RS | 43 | 5405 | Chiapetta | 4.094 |
| RS | 43 | 5439 | Chuí | 5.496 |
| RS | 43 | 5447 | Chuvisca | 5.159 |
| RS | 43 | 5454 | Cidreira | 11.885 |
| RS | 43 | 5504 | Ciríaco | 5.020 |
| RS | 43 | 5587 | Colinas | 2.470 |
| RS | 43 | 5603 | Colorado | 3.771 |
| RS | 43 | 5702 | Condor | 6.873 |
| RS | 43 | 5801 | Constantina | 10.191 |
| RS | 43 | 5835 | Coqueiro Baixo | 1.610 |
| RS | 43 | 5850 | Coqueiros do Sul | 3.342 |
| RS | 43 | 5871 | Coronel Barros | 2.522 |
| RS | 43 | 5900 | Coronel Bicaco | 7.971 |
| RS | 43 | 5934 | Coronel Pilar | 1.645 |
| RS | 43 | 5959 | Cotiporã | 4.887 |
| RS | 43 | 5975 | Coxilha | 2.997 |
| RS | 43 | 6007 | Crissiumal | 15.096 |
| RS | 43 | 6056 | Cristal | 7.393 |
| RS | 43 | 6072 | Cristal do Sul | 3.099 |
| RS | 43 | 6106 | Cruz Alta | 64.438 |
| RS | 43 | 6130 | Cruzaltense | 2.266 |
| RS | 43 | 6205 | Cruzeiro do Sul | 12.752 |
| RS | 43 | 6304 | David Canabarro | 4.857 |
| RS | 43 | 6320 | Derrubadas | 3.391 |
| RS | 43 | 6353 | Dezesseis de Novembro | 2.923 |
| RS | 43 | 6379 | Dilermando de Aguiar | 3.215 |
| RS | 43 | 6403 | Dois Irmãos | 26.421 |
| RS | 43 | 6429 | Dois Irmãos das Missões | 2.444 |
| RS | 43 | 6452 | Dois Lajeados | 3.484 |
| RS | 43 | 6502 | Dom Feliciano | 15.300 |
| RS | 43 | 6601 | Dom Pedrito | 38.767 |
| RS | 43 | 6551 | Dom Pedro de Alcântara | 2.851 |
| RS | 43 | 6700 | Dona Francisca | 3.593 |
| RS | 43 | 6734 | Doutor Maurício Cardoso | 5.424 |
| RS | 43 | 6759 | Doutor Ricardo | 2.101 |
| RS | 43 | 6767 | Eldorado do Sul | 33.668 |
| RS | 43 | 6809 | Encantado | 20.530 |

| U.F. | | MUNICÍPIOS | | POPULAÇÃO ESTIMADA |
|---|---|---|---|---|
| SIGLA | COD | COD | NOMES | |
| RS | 43 | 6908 | Encruzilhada do Sul | 25.154 |
| RS | 43 | 6924 | Engenho Velho | 1.467 |
| RS | 43 | 6957 | Entre Rios do Sul | 3.087 |
| RS | 43 | 6932 | Entre-Ijuís | 9.263 |
| RS | 43 | 6973 | Erebango | 2.936 |
| RS | 43 | 7005 | Erechim | 97.916 |
| RS | 43 | 7054 | Ernestina | 3.094 |
| RS | 43 | 7203 | Erval Grande | 5.367 |
| RS | 43 | 7302 | Erval Seco | 8.196 |
| RS | 43 | 7401 | Esmeralda | 3.383 |
| RS | 43 | 7450 | Esperança do Sul | 3.468 |
| RS | 43 | 7500 | Espumoso | 15.435 |
| RS | 43 | 7559 | Estação | 6.253 |
| RS | 43 | 7609 | Estância Velha | 43.906 |
| RS | 43 | 7708 | Esteio | 81.170 |
| RS | 43 | 7807 | Estrela | 30.603 |
| RS | 43 | 7815 | Estrela Velha | 3.777 |
| RS | 43 | 7831 | Eugênio de Castro | 3.083 |
| RS | 43 | 7864 | Fagundes Varela | 2.607 |
| RS | 43 | 7906 | Farroupilha | 63.375 |
| RS | 43 | 8003 | Faxinal do Soturno | 6.407 |
| RS | 43 | 8052 | Faxinalzinho | 2.607 |
| RS | 43 | 8078 | Fazenda Vilanova | 3.248 |
| RS | 43 | 8102 | Feliz | 12.198 |
| RS | 43 | 8201 | Flores da Cunha | 26.695 |
| RS | 43 | 8250 | Floriano Peixoto | 2.155 |
| RS | 43 | 8300 | Fontoura Xavier | 11.335 |
| RS | 43 | 8409 | Formigueiro | 7.214 |
| RS | 43 | 8433 | Forquetinha | 2.614 |
| RS | 43 | 8458 | Fortaleza dos Valos | 4.635 |
| RS | 43 | 8508 | Frederico Westphalen | 28.428 |
| RS | 43 | 8607 | Garibaldi | 30.518 |
| RS | 43 | 8656 | Garruchos | 3.509 |
| RS | 43 | 8706 | Gaurama | 6.232 |
| RS | 43 | 8805 | General Câmara | 9.101 |
| RS | 43 | 8854 | Gentil | 1.574 |
| RS | 43 | 8904 | Getúlio Vargas | 16.345 |
| RS | 43 | 9001 | Giruá | 17.139 |
| RS | 43 | 9050 | Glorinha | 7.531 |
| RS | 43 | 9100 | Gramado | 33.706 |
| RS | 43 | 9126 | Gramado dos Loureiros | 2.399 |
| RS | 43 | 9159 | Gramado Xavier | 4.124 |
| RS | 43 | 9209 | Gravataí | 269.446 |
| RS | 43 | 9258 | Guabiju | 1.702 |
| RS | 43 | 9308 | Guaíba | 96.603 |
| RS | 43 | 9407 | Guaporé | 22.589 |
| RS | 43 | 9506 | Guarani das Missões | 8.414 |
| RS | 43 | 9555 | Harmonia | 3.785 |
| RS | 43 | 7104 | Herval | 7.120 |
| RS | 43 | 9571 | Herveiras | 2.882 |

| U.F. | | MUNICÍPIOS | | POPULAÇÃO ESTIMADA |
|---|---|---|---|---|
| SIGLA | COD | COD | NOMES | |
| RS | 43 | 9605 | Horizontina | 19.131 |
| RS | 43 | 9654 | Hulha Negra | 6.448 |
| RS | 43 | 9704 | Humaitá | 4.999 |
| RS | 43 | 9753 | Ibarama | 4.443 |
| RS | 43 | 9803 | Ibiaçá | 4.796 |
| RS | 43 | 9902 | Ibiraiaras | 7.319 |
| RS | 43 | 9951 | Ibirapuitã | 4.315 |
| RS | 43 | 10009 | Ibirubá | 19.358 |
| RS | 43 | 10108 | Igrejinha | 33.551 |
| RS | 43 | 10207 | Ijuí | 79.719 |
| RS | 43 | 10306 | Ilópolis | 4.331 |
| RS | 43 | 10330 | Imbé | 16.301 |
| RS | 43 | 10363 | Imigrante | 3.125 |
| RS | 43 | 10405 | Independência | 6.715 |
| RS | 43 | 10413 | Inhacorá | 2.341 |
| RS | 43 | 10439 | Ipê | 6.210 |
| RS | 43 | 10462 | Ipiranga do Sul | 2.029 |
| RS | 43 | 10504 | Iraí | 8.518 |
| RS | 43 | 10538 | Itaara | 4.812 |
| RS | 43 | 10553 | Itacurubi | 3.712 |
| RS | 43 | 10579 | Itapuca | 2.465 |
| RS | 43 | 10603 | Itaqui | 36.560 |
| RS | 43 | 10652 | Itati | 2.720 |
| RS | 43 | 10702 | Itatiba do Sul | 4.521 |
| RS | 43 | 10751 | Ivorá | 2.424 |
| RS | 43 | 10801 | Ivoti | 20.160 |
| RS | 43 | 10850 | Jaboticaba | 4.204 |
| RS | 43 | 10876 | Jacuizinho | 2.765 |
| RS | 43 | 10900 | Jacutinga | 3.615 |
| RS | 43 | 11007 | Jaguarão | 28.244 |
| RS | 43 | 11106 | Jaguari | 11.762 |
| RS | 43 | 11122 | Jaquirana | 4.429 |
| RS | 43 | 11130 | Jari | 3.802 |
| RS | 43 | 11155 | Jóia | 8.566 |
| RS | 43 | 11205 | Júlio de Castilhos | 19.947 |
| RS | 43 | 11270 | Lagoa dos Três Cantos | 1.633 |
| RS | 43 | 11239 | Lagoa Bonita do Sul | 2.759 |
| RS | 43 | 11304 | Lagoa Vermelha | 28.236 |
| RS | 43 | 11254 | Lagoão | 6.702 |
| RS | 43 | 11403 | Lajeado | 72.208 |
| RS | 43 | 11429 | Lajeado do Bugre | 2.647 |
| RS | 43 | 11502 | Lavras do Sul | 8.399 |
| RS | 43 | 11601 | Liberato Salzano | 6.167 |
| RS | 43 | 11627 | Lindolfo Collor | 5.732 |
| RS | 43 | 11643 | Linha Nova | 1.516 |
| RS | 43 | 11718 | Maçambara | 4.375 |
| RS | 43 | 11700 | Machadinho | 5.623 |
| RS | 43 | 11734 | Mampituba | 2.972 |
| RS | 43 | 11759 | Manoel Viana | 6.954 |
| RS | 43 | 11775 | Maquiné | 7.652 |

| U.F. | | MUNICÍPIOS | | POPULAÇÃO |
|---|---|---|---|---|
| SIGLA | COD | COD | NOMES | ESTIMADA |
| RS | 43 | 11791 | Maratá | 2.542 |
| RS | 43 | 11809 | Marau | 36.643 |
| RS | 43 | 11908 | Marcelino Ramos | 5.329 |
| RS | 43 | 11981 | Mariana Pimentel | 4.173 |
| RS | 43 | 12005 | Mariano Moro | 2.304 |
| RS | 43 | 12054 | Marques de Souza | 4.122 |
| RS | 43 | 12104 | Mata | 5.386 |
| RS | 43 | 12138 | Mato Castelhano | 2.747 |
| RS | 43 | 12153 | Mato Leitão | 3.827 |
| RS | 43 | 12179 | Mato Queimado | 1.880 |
| RS | 43 | 12203 | Maximiliano de Almeida | 5.050 |
| RS | 43 | 12252 | Minas do Leão | 8.124 |
| RS | 43 | 12302 | Miraguaí | 4.986 |
| RS | 43 | 12351 | Montauri | 1.606 |
| RS | 43 | 12377 | Monte Alegre dos Campos | 3.256 |
| RS | 43 | 12385 | Monte Belo do Sul | 2.826 |
| RS | 43 | 12401 | Montenegro | 59.557 |
| RS | 43 | 12427 | Mormaço | 2.713 |
| RS | 43 | 12443 | Morrinhos do Sul | 3.263 |
| RS | 43 | 12450 | Morro Redondo | 6.477 |
| RS | 43 | 12476 | Morro Reuter | 5.985 |
| RS | 43 | 12500 | Mostardas | 12.392 |
| RS | 43 | 12609 | Muçum | 4.685 |
| RS | 43 | 12617 | Muitos Capões | 3.104 |
| RS | 43 | 12625 | Muliterno | 1.872 |
| RS | 43 | 12658 | Não-Me-Toque | 16.012 |
| RS | 43 | 12674 | Nicolau Vergueiro | 1.803 |
| RS | 43 | 12708 | Nonoai | 12.601 |
| RS | 43 | 12757 | Nova Alvorada | 3.258 |
| RS | 43 | 12807 | Nova Araçá | 4.075 |
| RS | 43 | 12906 | Nova Bassano | 9.249 |
| RS | 43 | 12955 | Nova Boa Vista | 2.113 |
| RS | 43 | 13003 | Nova Bréscia | 3.291 |
| RS | 43 | 13011 | Nova Candelária | 2.789 |
| RS | 43 | 13037 | Nova Esperança do Sul | 5.180 |
| RS | 43 | 13060 | Nova Hartz | 17.772 |
| RS | 43 | 13086 | Nova Pádua | 2.598 |
| RS | 43 | 13102 | Nova Palma | 6.693 |
| RS | 43 | 13201 | Nova Petrópolis | 18.631 |
| RS | 43 | 13300 | Nova Prata | 24.252 |
| RS | 43 | 13334 | Nova Ramada | 2.464 |
| RS | 43 | 13359 | Nova Roma do Sul | 3.754 |
| RS | 43 | 13375 | Nova Santa Rita | 22.818 |
| RS | 43 | 13490 | Novo Barreiro | 3.989 |
| RS | 43 | 13391 | Novo Cabrais | 3.960 |
| RS | 43 | 13409 | Novo Hamburgo | 257.746 |
| RS | 43 | 13425 | Novo Machado | 4.246 |
| RS | 43 | 13441 | Novo Tiradentes | 2.387 |
| RS | 43 | 13466 | Novo Xingu | 1.926 |
| RS | 43 | 13508 | Osório | 41.642 |

| U.F. | | MUNICÍPIOS | | POPULAÇÃO ESTIMADA |
|---|---|---|---|---|
| SIGLA | COD | COD | NOMES | |
| RS | 43 | 13607 | Paim Filho | 4.526 |
| RS | 43 | 13656 | Palmares do Sul | 11.998 |
| RS | 43 | 13706 | Palmeira das Missões | 34.225 |
| RS | 43 | 13805 | Palmitinho | 7.134 |
| RS | 43 | 13904 | Panambi | 38.794 |
| RS | 43 | 13953 | Pantano Grande | 9.794 |
| RS | 43 | 14001 | Paraí | 6.979 |
| RS | 43 | 14027 | Paraíso do Sul | 7.643 |
| RS | 43 | 14035 | Pareci Novo | 3.232 |
| RS | 43 | 14050 | Parobé | 51.634 |
| RS | 43 | 14068 | Passa Sete | 5.280 |
| RS | 43 | 14076 | Passo do Sobrado | 6.300 |
| RS | 43 | 14100 | Passo Fundo | 187.507 |
| RS | 43 | 14134 | Paulo Bento | 2.149 |
| RS | 43 | 14159 | Paverama | 7.841 |
| RS | 43 | 14175 | Pedras Altas | 2.638 |
| RS | 43 | 14209 | Pedro Osório | 8.297 |
| RS | 43 | 14308 | Pejuçara | 3.946 |
| RS | 43 | 14407 | Pelotas | 345.181 |
| RS | 43 | 14423 | Picada Café | 5.039 |
| RS | 43 | 14456 | Pinhal | 2.401 |
| RS | 43 | 14464 | Pinhal da Serra | 2.022 |
| RS | 43 | 14472 | Pinhal Grande | 4.581 |
| RS | 43 | 14498 | Pinheirinho do Vale | 4.635 |
| RS | 43 | 14506 | Pinheiro Machado | 13.152 |
| RS | 43 | 14555 | Pirapó | 2.979 |
| RS | 43 | 14605 | Piratini | 21.180 |
| RS | 43 | 14704 | Planalto | 10.734 |
| RS | 43 | 14753 | Poço das Antas | 2.098 |
| RS | 43 | 14779 | Pontão | 4.040 |
| RS | 43 | 14787 | Ponte Preta | 1.846 |
| RS | 43 | 14803 | Portão | 30.802 |
| RS | 43 | 14902 | Porto Alegre | 1.436.123 |
| RS | 43 | 15008 | Porto Lucena | 5.587 |
| RS | 43 | 15057 | Porto Mauá | 2.580 |
| RS | 43 | 15073 | Porto Vera Cruz | 2.038 |
| RS | 43 | 15107 | Porto Xavier | 11.131 |
| RS | 43 | 15131 | Pouso Novo | 1.998 |
| RS | 43 | 15149 | Presidente Lucena | 2.526 |
| RS | 43 | 15156 | Progresso | 6.420 |
| RS | 43 | 15172 | Protásio Alves | 2.188 |
| RS | 43 | 15206 | Putinga | 4.242 |
| RS | 43 | 15305 | Quaraí | 22.883 |
| RS | 43 | 15313 | Quatro Irmãos | 1.791 |
| RS | 43 | 15321 | Quevedos | 2.839 |
| RS | 43 | 15354 | Quinze de Novembro | 3.678 |
| RS | 43 | 15404 | Redentora | 10.170 |
| RS | 43 | 15453 | Relvado | 2.244 |
| RS | 43 | 15503 | Restinga Seca | 15.885 |
| RS | 43 | 15552 | Rio dos Índios | 4.192 |

| U.F. | | MUNICÍPIOS | | POPULAÇÃO ESTIMADA |
|---|---|---|---|---|
| SIGLA | COD | COD | NOMES | |
| RS | 43 | 15602 | Rio Grande | 196.337 |
| RS | 43 | 15701 | Rio Pardo | 38.989 |
| RS | 43 | 15750 | Riozinho | 4.664 |
| RS | 43 | 15800 | Roca Sales | 10.468 |
| RS | 43 | 15909 | Rodeio Bonito | 5.880 |
| RS | 43 | 15958 | Rolador | 2.869 |
| RS | 43 | 16006 | Rolante | 20.306 |
| RS | 43 | 16105 | Ronda Alta | 9.865 |
| RS | 43 | 16204 | Rondinha | 5.736 |
| RS | 43 | 16303 | Roque Gonzales | 7.394 |
| RS | 43 | 16402 | Rosário do Sul | 41.746 |
| RS | 43 | 16428 | Sagrada Família | 2.668 |
| RS | 43 | 16436 | Saldanha Marinho | 3.019 |
| RS | 43 | 16451 | Salto do Jacuí | 12.770 |
| RS | 43 | 16477 | Salvador das Missões | 2.671 |
| RS | 43 | 16501 | Salvador do Sul | 7.165 |
| RS | 43 | 16600 | Sananduva | 15.216 |
| RS | 43 | 16709 | Santa Bárbara do Sul | 9.164 |
| RS | 43 | 16733 | Santa Cecília do Sul | 1.776 |
| RS | 43 | 16758 | Santa Clara do Sul | 5.868 |
| RS | 43 | 16808 | Santa Cruz do Sul | 122.451 |
| RS | 43 | 16972 | Santa Margarida do Sul | 2.235 |
| RS | 43 | 16907 | Santa Maria | 268.969 |
| RS | 43 | 16956 | Santa Maria do Herval | 6.476 |
| RS | 43 | 17202 | Santa Rosa | 66.059 |
| RS | 43 | 17251 | Santa Tereza | 1.893 |
| RS | 43 | 17301 | Santa Vitória do Palmar | 31.605 |
| RS | 43 | 17004 | Santana da Boa Vista | 8.891 |
| RS | 43 | 17103 | Santana do Livramento | 84.079 |
| RS | 43 | 17400 | Santiago | 51.160 |
| RS | 43 | 17509 | Santo Ângelo | 75.445 |
| RS | 43 | 17608 | Santo Antônio da Patrulha | 39.500 |
| RS | 43 | 17707 | Santo Antônio das Missões | 12.016 |
| RS | 43 | 17558 | Santo Antônio do Palma | 2.295 |
| RS | 43 | 17756 | Santo Antônio do Planalto | 2.108 |
| RS | 43 | 17806 | Santo Augusto | 13.845 |
| RS | 43 | 17905 | Santo Cristo | 14.585 |
| RS | 43 | 17954 | Santo Expedito do Sul | 2.684 |
| RS | 43 | 18002 | São Borja | 63.035 |
| RS | 43 | 18051 | São Domingos do Sul | 2.961 |
| RS | 43 | 18101 | São Francisco de Assis | 19.799 |
| RS | 43 | 18200 | São Francisco de Paula | 22.503 |
| RS | 43 | 18309 | São Gabriel | 59.337 |
| RS | 43 | 18408 | São Jerônimo | 21.289 |
| RS | 43 | 18424 | São João da Urtiga | 5.123 |
| RS | 43 | 18432 | São João do Polêsine | 2.782 |
| RS | 43 | 18440 | São Jorge | 2.825 |
| RS | 43 | 18457 | São José das Missões | 3.039 |
| RS | 43 | 18465 | São José do Herval | 2.549 |
| RS | 43 | 18481 | São José do Hortêncio | 4.173 |

| U.F. | | MUNICÍPIOS | | POPULAÇÃO ESTIMADA |
|---|---|---|---|---|
| SIGLA | COD | COD | NOMES | |
| RS | 43 | 18499 | São José do Inhacorá | 2.122 |
| RS | 43 | 18507 | São José do Norte | 26.116 |
| RS | 43 | 18606 | São José do Ouro | 7.190 |
| RS | 43 | 18614 | São José do Sul | 2.010 |
| RS | 43 | 18622 | São José dos Ausentes | 3.314 |
| RS | 43 | 18705 | São Leopoldo | 211.663 |
| RS | 43 | 18804 | São Lourenço do Sul | 43.388 |
| RS | 43 | 18903 | São Luiz Gonzaga | 34.999 |
| RS | 43 | 19000 | São Marcos | 20.537 |
| RS | 43 | 19109 | São Martinho | 5.910 |
| RS | 43 | 19125 | São Martinho da Serra | 3.579 |
| RS | 43 | 19158 | São Miguel das Missões | 7.656 |
| RS | 43 | 19208 | São Nicolau | 5.959 |
| RS | 43 | 19307 | São Paulo das Missões | 6.767 |
| RS | 43 | 19356 | São Pedro da Serra | 3.311 |
| RS | 43 | 19364 | São Pedro das Missões | 2.051 |
| RS | 43 | 19372 | São Pedro do Butiá | 2.802 |
| RS | 43 | 19406 | São Pedro do Sul | 17.073 |
| RS | 43 | 19505 | São Sebastião do Caí | 21.272 |
| RS | 43 | 19604 | São Sepé | 24.353 |
| RS | 43 | 19703 | São Valentim | 3.996 |
| RS | 43 | 19711 | São Valentim do Sul | 2.336 |
| RS | 43 | 19737 | São Valério do Sul | 2.729 |
| RS | 43 | 19752 | São Vendelino | 1.891 |
| RS | 43 | 19802 | São Vicente do Sul | 8.659 |
| RS | 43 | 19901 | Sapiranga | 78.045 |
| RS | 43 | 20008 | Sapucaia do Sul | 126.316 |
| RS | 43 | 20107 | Sarandi | 21.828 |
| RS | 43 | 20206 | Seberi | 11.098 |
| RS | 43 | 20230 | Sede Nova | 2.996 |
| RS | 43 | 20263 | Segredo | 7.301 |
| RS | 43 | 20305 | Selbach | 4.912 |
| RS | 43 | 20321 | Senador Salgado Filho | 2.940 |
| RS | 43 | 20354 | Sentinela do Sul | 5.598 |
| RS | 43 | 20404 | Serafina Corrêa | 14.733 |
| RS | 43 | 20453 | Sério | 2.387 |
| RS | 43 | 20503 | Sertão | 6.644 |
| RS | 43 | 20552 | Sertão Santana | 6.155 |
| RS | 43 | 20578 | Sete de Setembro | 2.134 |
| RS | 43 | 20602 | Severiano de Almeida | 3.966 |
| RS | 43 | 20651 | Silveira Martins | 2.537 |
| RS | 43 | 20677 | Sinimbu | 10.706 |
| RS | 43 | 20701 | Sobradinho | 14.744 |
| RS | 43 | 20800 | Soledade | 31.028 |
| RS | 43 | 20859 | Tabaí | 4.338 |
| RS | 43 | 20909 | Tapejara | 19.147 |
| RS | 43 | 21006 | Tapera | 10.787 |
| RS | 43 | 21105 | Tapes | 17.216 |
| RS | 43 | 21204 | Taquara | 55.473 |
| RS | 43 | 21303 | Taquari | 26.626 |

| U.F. | | MUNICÍPIOS | | POPULAÇÃO ESTIMADA |
|---|---|---|---|---|
| SIGLA | COD | COD | NOMES | |
| RS | 43 | 21329 | Taquaruçu do Sul | 2.925 |
| RS | 43 | 21352 | Tavares | 5.283 |
| RS | 43 | 21402 | Tenente Portela | 14.253 |
| RS | 43 | 21436 | Terra de Areia | 10.389 |
| RS | 43 | 21451 | Teutônia | 27.215 |
| RS | 43 | 21469 | Tio Hugo | 2.730 |
| RS | 43 | 21477 | Tiradentes do Sul | 6.991 |
| RS | 43 | 21493 | Toropi | 3.138 |
| RS | 43 | 21501 | Torres | 33.944 |
| RS | 43 | 21600 | Tramandaí | 44.040 |
| RS | 43 | 21626 | Travesseiro | 2.471 |
| RS | 43 | 21808 | Três de Maio | 23.893 |
| RS | 43 | 21634 | Três Arroios | 3.079 |
| RS | 43 | 21667 | Três Cachoeiras | 11.022 |
| RS | 43 | 21709 | Três Coroas | 24.786 |
| RS | 43 | 21832 | Três Forquilhas | 3.128 |
| RS | 43 | 21857 | Três Palmeiras | 4.469 |
| RS | 43 | 21907 | Três Passos | 23.911 |
| RS | 43 | 21956 | Trindade do Sul | 6.105 |
| RS | 43 | 22004 | Triunfo | 25.374 |
| RS | 43 | 22103 | Tucunduva | 5.988 |
| RS | 43 | 22152 | Tunas | 4.551 |
| RS | 43 | 22186 | Tupanci do Sul | 1.781 |
| RS | 43 | 22202 | Tupanciretã | 23.842 |
| RS | 43 | 22251 | Tupandi | 3.931 |
| RS | 43 | 22301 | Tuparendi | 8.866 |
| RS | 43 | 22327 | Turuçu | 4.000 |
| RS | 43 | 22343 | Ubiretama | 2.451 |
| RS | 43 | 22350 | União da Serra | 1.649 |
| RS | 43 | 22376 | Unistalda | 2.396 |
| RS | 43 | 22400 | Uruguaiana | 127.045 |
| RS | 43 | 22509 | Vacaria | 62.831 |
| RS | 43 | 22533 | Vale do Sol | 11.273 |
| RS | 43 | 22541 | Vale Real | 5.031 |
| RS | 43 | 22525 | Vale Verde | 3.392 |
| RS | 43 | 22558 | Vanini | 2.045 |
| RS | 43 | 22608 | Venâncio Aires | 67.682 |
| RS | 43 | 22707 | Vera Cruz | 23.928 |
| RS | 43 | 22806 | Veranópolis | 26.121 |
| RS | 43 | 22855 | Vespasiano Correa | 1.967 |
| RS | 43 | 22905 | Viadutos | 5.728 |
| RS | 43 | 23002 | Viamão | 260.740 |
| RS | 43 | 23101 | Vicente Dutra | 5.587 |
| RS | 43 | 23200 | Victor Graeff | 3.120 |
| RS | 43 | 23309 | Vila Flores | 3.305 |
| RS | 43 | 23358 | Vila Lângaro | 2.293 |
| RS | 43 | 23408 | Vila Maria | 4.299 |
| RS | 43 | 23457 | Vila Nova do Sul | 4.401 |
| RS | 43 | 23507 | Vista Alegre | 2.932 |
| RS | 43 | 23606 | Vista Alegre do Prata | 1.505 |

| U.F. | | MUNICÍPIOS | | POPULAÇÃO ESTIMADA |
|---|---|---|---|---|
| SIGLA | COD | COD | NOMES | |
| RS | 43 | 23705 | Vista Gaúcha | 2.804 |
| RS | 43 | 23754 | Vitória das Missões | 3.676 |
| RS | 43 | 23770 | Westfalia | 2.843 |
| RS | 43 | 23804 | Xangri-lá | 11.721 |
| MS | 50 | 203 | Água Clara | 13.879 |
| MS | 50 | 252 | Alcinópolis | 4.515 |
| MS | 50 | 609 | Amambaí | 34.986 |
| MS | 50 | 708 | Anastácio | 23.047 |
| MS | 50 | 807 | Anaurilândia | 8.697 |
| MS | 50 | 856 | Angélica | 7.465 |
| MS | 50 | 906 | Antônio João | 8.734 |
| MS | 50 | 1003 | Aparecida do Taboado | 20.623 |
| MS | 50 | 1102 | Aquidauana | 46.515 |
| MS | 50 | 1243 | Aral Moreira | 9.679 |
| MS | 50 | 1508 | Bandeirantes | 6.001 |
| MS | 50 | 1904 | Bataguassu | 19.596 |
| MS | 50 | 2001 | Batayporã | 10.885 |
| MS | 50 | 2100 | Bela Vista | 23.726 |
| MS | 50 | 2159 | Bodoquena | 8.397 |
| MS | 50 | 2209 | Bonito | 17.856 |
| MS | 50 | 2308 | Brasilândia | 12.538 |
| MS | 50 | 2407 | Caarapó | 23.696 |
| MS | 50 | 2605 | Camapuã | 13.532 |
| MS | 50 | 2704 | Campo Grande | 755.107 |
| MS | 50 | 2803 | Caracol | 5.320 |
| MS | 50 | 2902 | Cassilândia | 21.677 |
| MS | 50 | 2951 | Chapadão do Sul | 17.293 |
| MS | 50 | 3108 | Corguinho | 4.370 |
| MS | 50 | 3157 | Coronel Sapucaia | 14.569 |
| MS | 50 | 3207 | Corumbá | 99.467 |
| MS | 50 | 3256 | Costa Rica | 19.228 |
| MS | 50 | 3306 | Coxim | 32.933 |
| MS | 50 | 3454 | Deodápolis | 11.600 |
| MS | 50 | 3488 | Dois Irmãos do Buriti | 9.643 |
| MS | 50 | 3504 | Douradina | 5.075 |
| MS | 50 | 3702 | Dourados | 189.762 |
| MS | 50 | 3751 | Eldorado | 12.421 |
| MS | 50 | 3801 | Fátima do Sul | 19.332 |
| MS | 50 | 3900 | Figueirão | 3.443 |
| MS | 50 | 4007 | Glória de Dourados | 9.894 |
| MS | 50 | 4106 | Guia Lopes da Laguna | 10.407 |
| MS | 50 | 4304 | Iguatemi | 15.222 |
| MS | 50 | 4403 | Inocência | 7.501 |
| MS | 50 | 4502 | Itaporã | 19.390 |
| MS | 50 | 4601 | Itaquiraí | 17.603 |
| MS | 50 | 4700 | Ivinhema | 21.067 |
| MS | 50 | 4809 | Japorã | 7.752 |
| MS | 50 | 4908 | Jaraguari | 5.776 |
| MS | 50 | 5004 | Jardim | 24.174 |
| MS | 50 | 5103 | Jateí | 3.895 |

| U.F. | | MUNICÍPIOS | | POPULAÇÃO ESTIMADA |
|---|---|---|---|---|
| SIGLA | COD | COD | NOMES | |
| MS | 50 | 5152 | Juti | 5.569 |
| MS | 50 | 5202 | Ladário | 18.805 |
| MS | 50 | 5251 | Laguna Carapã | 6.031 |
| MS | 50 | 5400 | Maracaju | 32.492 |
| MS | 50 | 5608 | Miranda | 24.838 |
| MS | 50 | 5681 | Mundo Novo | 16.506 |
| MS | 50 | 5707 | Naviraí | 45.627 |
| MS | 50 | 5806 | Nioaque | 15.693 |
| MS | 50 | 6002 | Nova Alvorada do Sul | 12.673 |
| MS | 50 | 6200 | Nova Andradina | 45.916 |
| MS | 50 | 6259 | Novo Horizonte do Sul | 4.932 |
| MS | 50 | 6309 | Paranaíba | 40.259 |
| MS | 50 | 6358 | Paranhos | 11.553 |
| MS | 50 | 6408 | Pedro Gomes | 8.537 |
| MS | 50 | 6606 | Ponta Porã | 75.941 |
| MS | 50 | 6903 | Porto Murtinho | 15.527 |
| MS | 50 | 7109 | Ribas do Rio Pardo | 20.077 |
| MS | 50 | 7208 | Rio Brilhante | 27.903 |
| MS | 50 | 7307 | Rio Negro | 5.054 |
| MS | 50 | 7406 | Rio Verde de Mato Grosso | 19.216 |
| MS | 50 | 7505 | Rochedo | 4.480 |
| MS | 50 | 7554 | Santa Rita do Pardo | 7.454 |
| MS | 50 | 7695 | São Gabriel do Oeste | 21.650 |
| MS | 50 | 7802 | Selvíria | 6.656 |
| MS | 50 | 7703 | Sete Quedas | 10.955 |
| MS | 50 | 7901 | Sidrolândia | 41.261 |
| MS | 50 | 7935 | Sonora | 13.334 |
| MS | 50 | 7950 | Tacuru | 9.554 |
| MS | 50 | 7976 | Taquarussu | 3.165 |
| MS | 50 | 8008 | Terenos | 15.276 |
| MS | 50 | 8305 | Três Lagoas | 89.493 |
| MS | 50 | 8404 | Vicentina | 5.783 |
| MT | 51 | 102 | Acorizal | 5.659 |
| MT | 51 | 201 | Água Boa | 20.276 |
| MT | 51 | 250 | Alta Floresta | 51.414 |
| MT | 51 | 300 | Alto Araguaia | 14.611 |
| MT | 51 | 359 | Alto Boa Vista | 5.475 |
| MT | 51 | 409 | Alto Garças | 9.550 |
| MT | 51 | 508 | Alto Paraguai | 8.329 |
| MT | 51 | 607 | Alto Taquari | 6.505 |
| MT | 51 | 805 | Apiacás | 8.381 |
| MT | 51 | 1001 | Araguaiana | 2.996 |
| MT | 51 | 1209 | Araguainha | 1.115 |
| MT | 51 | 1258 | Araputanga | 16.090 |
| MT | 51 | 1308 | Arenápolis | 9.903 |
| MT | 51 | 1407 | Aripuanã | 20.511 |
| MT | 51 | 1605 | Barão de Melgaço | 7.851 |
| MT | 51 | 1704 | Barra do Bugres | 34.349 |
| MT | 51 | 1803 | Barra do Garças | 55.120 |
| MT | 51 | 1852 | Bom Jesus do Araguaia | 4.792 |

| U.F. | | MUNICÍPIOS | | POPULAÇÃO ESTIMADA |
|---|---|---|---|---|
| SIGLA | COD | COD | NOMES | |
| MT | 51 | 1902 | Brasnorte | 15.089 |
| MT | 51 | 2504 | Cáceres | 87.261 |
| MT | 51 | 2603 | Campinápolis | 14.301 |
| MT | 51 | 2637 | Campo Novo do Parecis | 23.784 |
| MT | 51 | 2678 | Campo Verde | 28.147 |
| MT | 51 | 2686 | Campos de Júlio | 5.223 |
| MT | 51 | 2694 | Canabrava do Norte | 5.563 |
| MT | 51 | 2702 | Canarana | 18.014 |
| MT | 51 | 2793 | Carlinda | 12.097 |
| MT | 51 | 2850 | Castanheira | 8.059 |
| MT | 51 | 3007 | Chapada dos Guimarães | 18.190 |
| MT | 51 | 3056 | Cláudia | 11.148 |
| MT | 51 | 3106 | Cocalinho | 6.103 |
| MT | 51 | 3205 | Colíder | 32.096 |
| MT | 51 | 3254 | Colniza | 31.597 |
| MT | 51 | 3304 | Comodoro | 18.974 |
| MT | 51 | 3353 | Confresa | 22.606 |
| MT | 51 | 3361 | Conquista D'Oeste | 3.289 |
| MT | 51 | 3379 | Cotriguaçu | 14.965 |
| MT | 51 | 3403 | Cuiabá | 550.562 |
| MT | 51 | 3437 | Curvelândia | 5.039 |
| MT | 51 | 3452 | Denise | 11.142 |
| MT | 51 | 3502 | Diamantino | 18.989 |
| MT | 51 | 3601 | Dom Aquino | 8.498 |
| MT | 51 | 3700 | Feliz Natal | 11.170 |
| MT | 51 | 3809 | Figueirópolis D'Oeste | 3.656 |
| MT | 51 | 3858 | Gaúcha do Norte | 6.195 |
| MT | 51 | 3908 | General Carneiro | 5.028 |
| MT | 51 | 3957 | Glória D'Oeste | 3.185 |
| MT | 51 | 4104 | Guarantã do Norte | 32.142 |
| MT | 51 | 4203 | Guiratinga | 14.523 |
| MT | 51 | 4500 | Indiavaí | 2.679 |
| MT | 51 | 4526 | Ipiranga do Norte | 4.641 |
| MT | 51 | 4542 | Itanhangá | 5.061 |
| MT | 51 | 4559 | Itaúba | 4.585 |
| MT | 51 | 4609 | Itiquira | 13.022 |
| MT | 51 | 4807 | Jaciara | 25.922 |
| MT | 51 | 4906 | Jangada | 8.462 |
| MT | 51 | 5002 | Jauru | 10.748 |
| MT | 51 | 5101 | Juara | 33.246 |
| MT | 51 | 5150 | Juína | 39.708 |
| MT | 51 | 5176 | Juruena | 9.595 |
| MT | 51 | 5200 | Juscimeira | 12.168 |
| MT | 51 | 5234 | Lambari D'Oeste | 5.060 |
| MT | 51 | 5259 | Lucas do Rio Verde | 33.556 |
| MT | 51 | 5309 | Luciára | 2.467 |
| MT | 51 | 5580 | Marcelândia | 14.473 |
| MT | 51 | 5606 | Matupá | 15.170 |
| MT | 51 | 5622 | Mirassol d'Oeste | 25.605 |
| MT | 51 | 5903 | Nobres | 15.315 |

| U.F. | | MUNICÍPIOS | | POPULAÇÃO |
|---|---|---|---|---|
| SIGLA | COD | COD | NOMES | ESTIMADA |
| MT | 51 | 6000 | Nortelândia | 6.272 |
| MT | 51 | 6109 | Nossa Senhora do Livramento | 12.819 |
| MT | 51 | 6158 | Nova Bandeirantes | 14.078 |
| MT | 51 | 6208 | Nova Brasilândia | 4.902 |
| MT | 51 | 6216 | Nova Canaã do Norte | 13.237 |
| MT | 51 | 8808 | Nova Guarita | 4.907 |
| MT | 51 | 6182 | Nova Lacerda | 5.252 |
| MT | 51 | 8857 | Nova Marilândia | 2.345 |
| MT | 51 | 8907 | Nova Maringá | 5.989 |
| MT | 51 | 8956 | Nova Monte Verde | 8.602 |
| MT | 51 | 6224 | Nova Mutum | 26.874 |
| MT | 51 | 6174 | Nova Nazaré | 2.955 |
| MT | 51 | 6232 | Nova Olímpia | 20.944 |
| MT | 51 | 6190 | Nova Santa Helena | 3.473 |
| MT | 51 | 6240 | Nova Ubiratã | 8.372 |
| MT | 51 | 6257 | Nova Xavantina | 19.398 |
| MT | 51 | 6273 | Novo Horizonte do Norte | 3.970 |
| MT | 51 | 6265 | Novo Mundo | 7.216 |
| MT | 51 | 6315 | Novo Santo Antônio | 2.325 |
| MT | 51 | 6281 | Novo São Joaquim | 6.985 |
| MT | 51 | 6299 | Paranaíta | 12.113 |
| MT | 51 | 6307 | Paranatinga | 21.424 |
| MT | 51 | 6372 | Pedra Preta | 16.461 |
| MT | 51 | 6422 | Peixoto de Azevedo | 30.363 |
| MT | 51 | 6455 | Planalto da Serra | 2.797 |
| MT | 51 | 6505 | Poconé | 32.162 |
| MT | 51 | 6653 | Pontal do Araguaia | 5.322 |
| MT | 51 | 6703 | Ponte Branca | 1.804 |
| MT | 51 | 6752 | Pontes e Lacerda | 39.228 |
| MT | 51 | 6802 | Porto dos Gaúchos | 6.383 |
| MT | 51 | 6778 | Porto Alegre do Norte | 10.109 |
| MT | 51 | 6828 | Porto Esperidião | 9.850 |
| MT | 51 | 6851 | Porto Estrela | 4.027 |
| MT | 51 | 7008 | Poxoréo | 17.758 |
| MT | 51 | 7040 | Primavera do Leste | 46.933 |
| MT | 51 | 7065 | Querência | 11.570 |
| MT | 51 | 7156 | Reserva do Cabaçal | 2.598 |
| MT | 51 | 7180 | Ribeirão Cascalheira | 9.172 |
| MT | 51 | 7198 | Ribeirãozinho | 2.194 |
| MT | 51 | 7206 | Rio Branco | 5.208 |
| MT | 51 | 7578 | Rondolândia | 3.484 |
| MT | 51 | 7602 | Rondonópolis | 181.902 |
| MT | 51 | 7701 | Rosário Oeste | 18.497 |
| MT | 51 | 7750 | Salto do Céu | 3.584 |
| MT | 51 | 7248 | Santa Carmem | 4.573 |
| MT | 51 | 7743 | Santa Cruz do Xingu | 2.357 |
| MT | 51 | 7768 | Santa Rita do Trivelato | 2.751 |
| MT | 51 | 7776 | Santa Terezinha | 7.690 |
| MT | 51 | 7263 | Santo Afonso | 2.944 |
| MT | 51 | 7792 | Santo Antônio do Leste | 3.573 |

| U.F. | | MUNICÍPIOS | | POPULAÇÃO ESTIMADA |
|---|---|---|---|---|
| SIGLA | COD | COD | NOMES | |
| MT | 51 | 7800 | Santo Antônio do Leverger | 20.412 |
| MT | 51 | 7859 | São Félix do Araguaia | 11.257 |
| MT | 51 | 7297 | São José do Povo | 3.451 |
| MT | 51 | 7305 | São José do Rio Claro | 18.637 |
| MT | 51 | 7354 | São José do Xingu | 4.218 |
| MT | 51 | 7107 | São José dos Quatro Marcos | 19.493 |
| MT | 51 | 7404 | São Pedro da Cipa | 4.241 |
| MT | 51 | 7875 | Sapezal | 15.735 |
| MT | 51 | 7883 | Serra Nova Dourada | 1.447 |
| MT | 51 | 7909 | Sinop | 114.051 |
| MT | 51 | 7925 | Sorriso | 60.028 |
| MT | 51 | 7941 | Tabaporã | 10.760 |
| MT | 51 | 7958 | Tangará da Serra | 81.960 |
| MT | 51 | 8006 | Tapurah | 11.517 |
| MT | 51 | 8055 | Terra Nova do Norte | 15.190 |
| MT | 51 | 8105 | Tesouro | 3.205 |
| MT | 51 | 8204 | Torixoréu | 4.113 |
| MT | 51 | 8303 | União do Sul | 4.093 |
| MT | 51 | 8352 | Vale de São Domingos | 2.955 |
| MT | 51 | 8402 | Várzea Grande | 240.038 |
| MT | 51 | 8501 | Vera | 9.502 |
| MT | 51 | 5507 | Vila Bela da Santíssima Trindade | 14.523 |
| MT | 51 | 8600 | Vila Rica | 20.075 |
| GO | 52 | 50 | Abadia de Goiás | 6.301 |
| GO | 52 | 100 | Abadiânia | 13.378 |
| GO | 52 | 134 | Acreúna | 19.246 |
| GO | 52 | 159 | Adelândia | 2.608 |
| GO | 52 | 175 | Água Fria de Goiás | 5.318 |
| GO | 52 | 209 | Água Limpa | 2.111 |
| GO | 52 | 258 | Águas Lindas de Goiás | 143.179 |
| GO | 52 | 308 | Alexânia | 20.706 |
| GO | 52 | 506 | Aloândia | 2.118 |
| GO | 52 | 555 | Alto Horizonte | 3.392 |
| GO | 52 | 605 | Alto Paraíso de Goiás | 6.982 |
| GO | 52 | 803 | Alvorada do Norte | 8.666 |
| GO | 52 | 829 | Amaralina | 3.719 |
| GO | 52 | 852 | Americano do Brasil | 4.795 |
| GO | 52 | 902 | Amorinópolis | 3.484 |
| GO | 52 | 1108 | Anápolis | 335.960 |
| GO | 52 | 1207 | Anhanguera | 1.018 |
| GO | 52 | 1306 | Anicuns | 18.027 |
| GO | 52 | 1405 | Aparecida de Goiânia | 510.770 |
| GO | 52 | 1454 | Aparecida do Rio Doce | 2.871 |
| GO | 52 | 1504 | Aporé | 3.708 |
| GO | 52 | 1603 | Araçu | 3.946 |
| GO | 52 | 1702 | Aragarças | 17.883 |
| GO | 52 | 1801 | Aragoiânia | 7.702 |
| GO | 52 | 2155 | Araguapaz | 7.780 |
| GO | 52 | 2353 | Arenópolis | 3.481 |
| GO | 52 | 2502 | Aruanã | 7.056 |

| U.F. | | MUNICÍPIOS | | POPULAÇÃO ESTIMADA |
|---|---|---|---|---|
| SIGLA | COD | COD | NOMES | |
| GO | 52 | 2601 | Aurilândia | 3.709 |
| GO | 52 | 2809 | Avelinópolis | 2.421 |
| GO | 52 | 3104 | Baliza | 3.679 |
| GO | 52 | 3203 | Barro Alto | 6.714 |
| GO | 52 | 3302 | Bela Vista de Goiás | 21.679 |
| GO | 52 | 3401 | Bom Jardim de Goiás | 8.734 |
| GO | 52 | 3500 | Bom Jesus de Goiás | 21.103 |
| GO | 52 | 3559 | Bonfinópolis | 7.336 |
| GO | 52 | 3575 | Bonópolis | 3.413 |
| GO | 52 | 3609 | Brazabrantes | 3.345 |
| GO | 52 | 3807 | Britânia | 5.190 |
| GO | 52 | 3939 | Buriti de Goiás | 2.203 |
| GO | 52 | 3906 | Buriti Alegre | 8.454 |
| GO | 52 | 3962 | Buritinópolis | 3.573 |
| GO | 52 | 4003 | Cabeceiras | 6.794 |
| GO | 52 | 4201 | Cachoeira de Goiás | 1.434 |
| GO | 52 | 4102 | Cachoeira Alta | 8.235 |
| GO | 52 | 4250 | Cachoeira Dourada | 7.571 |
| GO | 52 | 4300 | Caçu | 11.343 |
| GO | 52 | 4409 | Caiapônia | 16.559 |
| GO | 52 | 4508 | Caldas Novas | 67.588 |
| GO | 52 | 4557 | Caldazinha | 3.341 |
| GO | 52 | 4607 | Campestre de Goiás | 3.591 |
| GO | 52 | 4656 | Campinaçu | 3.908 |
| GO | 52 | 4706 | Campinorte | 10.039 |
| GO | 52 | 4805 | Campo Alegre de Goiás | 6.286 |
| GO | 52 | 4854 | Campo Limpo de Goiás | 6.022 |
| GO | 52 | 4904 | Campos Belos | 19.166 |
| GO | 52 | 4953 | Campos Verdes | 6.093 |
| GO | 52 | 5000 | Carmo do Rio Verde | 9.481 |
| GO | 52 | 5059 | Castelândia | 3.557 |
| GO | 52 | 5109 | Catalão | 81.109 |
| GO | 52 | 5208 | Caturaí | 4.667 |
| GO | 52 | 5307 | Cavalcante | 10.398 |
| GO | 52 | 5406 | Ceres | 19.069 |
| GO | 52 | 5455 | Cezarina | 7.832 |
| GO | 52 | 5471 | Chapadão do Céu | 5.863 |
| GO | 52 | 5497 | Cidade Ocidental | 52.380 |
| GO | 52 | 5513 | Cocalzinho de Goiás | 15.296 |
| GO | 52 | 5521 | Colinas do Sul | 4.026 |
| GO | 52 | 5703 | Córrego do Ouro | 2.632 |
| GO | 52 | 5802 | Corumbá de Goiás | 9.372 |
| GO | 52 | 5901 | Corumbaíba | 8.624 |
| GO | 52 | 6206 | Cristalina | 38.504 |
| GO | 52 | 6305 | Cristianópolis | 3.174 |
| GO | 52 | 6404 | Crixás | 15.005 |
| GO | 52 | 6503 | Cromínia | 3.729 |
| GO | 52 | 6602 | Cumari | 3.145 |
| GO | 52 | 6701 | Damianópolis | 3.678 |
| GO | 52 | 6800 | Damolândia | 2.809 |

| U.F. | | MUNICÍPIOS | | POPULAÇÃO |
|---|---|---|---|---|
| SIGLA | COD | COD | NOMES | ESTIMADA |
| GO | 52 | 6909 | Davinópolis | 2.075 |
| GO | 52 | 7105 | Diorama | 2.243 |
| GO | 52 | 8301 | Divinópolis de Goiás | 5.570 |
| GO | 52 | 7253 | Doverlândia | 8.570 |
| GO | 52 | 7352 | Edealina | 3.821 |
| GO | 52 | 7402 | Edéia | 10.604 |
| GO | 52 | 7501 | Estrela do Norte | 3.223 |
| GO | 52 | 7535 | Faina | 7.020 |
| GO | 52 | 7600 | Fazenda Nova | 6.399 |
| GO | 52 | 7808 | Firminópolis | 10.732 |
| GO | 52 | 7907 | Flores de Goiás | 11.483 |
| GO | 52 | 8004 | Formosa | 96.284 |
| GO | 52 | 8103 | Formoso | 5.327 |
| GO | 52 | 8152 | Gameleira de Goiás | 3.582 |
| GO | 52 | 8400 | Goianápolis | 11.663 |
| GO | 52 | 8509 | Goiandira | 5.081 |
| GO | 52 | 8608 | Goianésia | 56.839 |
| GO | 52 | 8707 | Goiânia | 1.281.975 |
| GO | 52 | 8806 | Goianira | 26.336 |
| GO | 52 | 8905 | Goiás | 24.605 |
| GO | 52 | 9101 | Goiatuba | 32.304 |
| GO | 52 | 9150 | Gouvelândia | 4.790 |
| GO | 52 | 9200 | Guapó | 13.973 |
| GO | 52 | 9291 | Guaraíta | 2.370 |
| GO | 52 | 9408 | Guarani de Goiás | 4.093 |
| GO | 52 | 9457 | Guarinos | 2.379 |
| GO | 52 | 9606 | Heitoraí | 3.706 |
| GO | 52 | 9705 | Hidrolândia | 14.718 |
| GO | 52 | 9804 | Hidrolina | 4.195 |
| GO | 52 | 9903 | Iaciara | 12.855 |
| GO | 52 | 9937 | Inaciolândia | 5.949 |
| GO | 52 | 9952 | Indiara | 13.364 |
| GO | 52 | 10000 | Inhumas | 46.786 |
| GO | 52 | 10109 | Ipameri | 24.021 |
| GO | 52 | 10158 | Ipiranga de Goiás | 2.906 |
| GO | 52 | 10208 | Iporá | 32.045 |
| GO | 52 | 10307 | Israelândia | 2.876 |
| GO | 52 | 10406 | Itaberaí | 32.356 |
| GO | 52 | 10562 | Itaguari | 4.364 |
| GO | 52 | 10604 | Itaguaru | 5.591 |
| GO | 52 | 10802 | Itajá | 5.528 |
| GO | 52 | 10901 | Itapaci | 17.086 |
| GO | 52 | 11008 | Itapirapuã | 7.792 |
| GO | 52 | 11206 | Itapuranga | 25.170 |
| GO | 52 | 11305 | Itarumã | 5.490 |
| GO | 52 | 11404 | Itauçu | 9.117 |
| GO | 52 | 11503 | Itumbiara | 92.832 |
| GO | 52 | 11602 | Ivolândia | 2.738 |
| GO | 52 | 11701 | Jandaia | 6.596 |
| GO | 52 | 11800 | Jaraguá | 41.772 |

| U.F. | | MUNICÍPIOS | | POPULAÇÃO ESTIMADA |
|---|---|---|---|---|
| SIGLA | COD | COD | NOMES | |
| GO | 52 | 11909 | Jataí | 86.447 |
| GO | 52 | 12006 | Jaupaci | 3.059 |
| GO | 52 | 12055 | Jesúpolis | 2.296 |
| GO | 52 | 12105 | Joviânia | 6.914 |
| GO | 52 | 12204 | Jussara | 19.130 |
| GO | 52 | 12253 | Lagoa Santa | 1.346 |
| GO | 52 | 12303 | Leopoldo de Bulhões | 9.518 |
| GO | 52 | 12501 | Luziânia | 210.064 |
| GO | 52 | 12600 | Mairipotaba | 2.811 |
| GO | 52 | 12709 | Mambaí | 7.096 |
| GO | 52 | 12808 | Mara Rosa | 10.360 |
| GO | 52 | 12907 | Marzagão | 2.157 |
| GO | 52 | 12956 | Matrinchã | 4.420 |
| GO | 52 | 13004 | Maurilândia | 11.604 |
| GO | 52 | 13053 | Mimoso de Goiás | 2.941 |
| GO | 52 | 13087 | Minaçu | 31.417 |
| GO | 52 | 13103 | Mineiros | 48.329 |
| GO | 52 | 13400 | Moiporá | 1.865 |
| GO | 52 | 13509 | Monte Alegre de Goiás | 7.466 |
| GO | 52 | 13707 | Montes Claros de Goiás | 8.070 |
| GO | 52 | 13756 | Montividiu | 9.965 |
| GO | 52 | 13772 | Montividiu do Norte | 4.702 |
| GO | 52 | 13806 | Morrinhos | 40.838 |
| GO | 52 | 13855 | Morro Agudo de Goiás | 2.379 |
| GO | 52 | 13905 | Mossâmedes | 4.901 |
| GO | 52 | 14002 | Mozarlândia | 14.073 |
| GO | 52 | 14051 | Mundo Novo | 6.787 |
| GO | 52 | 14101 | Mutunópolis | 4.086 |
| GO | 52 | 14408 | Nazário | 7.622 |
| GO | 52 | 14507 | Nerópolis | 20.260 |
| GO | 52 | 14606 | Niquelândia | 39.803 |
| GO | 52 | 14705 | Nova América | 2.278 |
| GO | 52 | 14804 | Nova Aurora | 2.209 |
| GO | 52 | 14838 | Nova Crixás | 13.432 |
| GO | 52 | 14861 | Nova Glória | 8.632 |
| GO | 52 | 14879 | Nova Iguaçu de Goiás | 2.678 |
| GO | 52 | 14903 | Nova Roma | 3.633 |
| GO | 52 | 15009 | Nova Veneza | 7.240 |
| GO | 52 | 15207 | Novo Brasil | 3.377 |
| GO | 52 | 15231 | Novo Gama | 88.835 |
| GO | 52 | 15256 | Novo Planalto | 4.266 |
| GO | 52 | 15306 | Orizona | 15.201 |
| GO | 52 | 15405 | Ouro Verde de Goiás | 4.599 |
| GO | 52 | 15504 | Ouvidor | 5.017 |
| GO | 52 | 15603 | Padre Bernardo | 28.012 |
| GO | 52 | 15652 | Palestina de Goiás | 3.317 |
| GO | 52 | 15702 | Palmeiras de Goiás | 22.798 |
| GO | 52 | 15801 | Palmelo | 2.324 |
| GO | 52 | 15900 | Palminópolis | 3.761 |
| GO | 52 | 16007 | Panamá | 2.665 |

| U.F. | | MUNICÍPIOS | | POPULAÇÃO |
|---|---|---|---|---|
| SIGLA | COD | COD | NOMES | ESTIMADA |
| GO | 52 | 16304 | Paranaiguara | 7.862 |
| GO | 52 | 16403 | Paraúna | 11.319 |
| GO | 52 | 16452 | Perolândia | 2.830 |
| GO | 52 | 16809 | Petrolina de Goiás | 10.061 |
| GO | 52 | 16908 | Pilar de Goiás | 2.821 |
| GO | 52 | 17104 | Piracanjuba | 24.033 |
| GO | 52 | 17203 | Piranhas | 11.215 |
| GO | 52 | 17302 | Pirenópolis | 20.945 |
| GO | 52 | 17401 | Pires do Rio | 27.928 |
| GO | 52 | 17609 | Planaltina | 79.651 |
| GO | 52 | 17708 | Pontalina | 16.687 |
| GO | 52 | 18003 | Porangatu | 40.469 |
| GO | 52 | 18052 | Porteirão | 3.158 |
| GO | 52 | 18102 | Portelândia | 3.321 |
| GO | 52 | 18300 | Posse | 31.257 |
| GO | 52 | 18391 | Professor Jamil | 3.381 |
| GO | 52 | 18508 | Quirinópolis | 39.756 |
| GO | 52 | 18607 | Rialma | 10.911 |
| GO | 52 | 18706 | Rianápolis | 4.256 |
| GO | 52 | 18789 | Rio Quente | 3.285 |
| GO | 52 | 18805 | Rio Verde | 163.021 |
| GO | 52 | 18904 | Rubiataba | 18.618 |
| GO | 52 | 19001 | Sanclerlândia | 7.936 |
| GO | 52 | 19100 | Santa Bárbara de Goiás | 6.031 |
| GO | 52 | 19209 | Santa Cruz de Goiás | 3.680 |
| GO | 52 | 19258 | Santa Fé de Goiás | 4.882 |
| GO | 52 | 19308 | Santa Helena de Goiás | 36.336 |
| GO | 52 | 19357 | Santa Isabel | 3.575 |
| GO | 52 | 19407 | Santa Rita do Araguaia | 6.277 |
| GO | 52 | 19456 | Santa Rita do Novo Destino | 3.576 |
| GO | 52 | 19506 | Santa Rosa de Goiás | 2.765 |
| GO | 52 | 19605 | Santa Tereza de Goiás | 4.141 |
| GO | 52 | 19704 | Santa Terezinha de Goiás | 11.829 |
| GO | 52 | 19712 | Santo Antônio da Barra | 4.295 |
| GO | 52 | 19738 | Santo Antônio de Goiás | 4.230 |
| GO | 52 | 19753 | Santo Antônio do Descoberto | 58.474 |
| GO | 52 | 19803 | São Domingos | 10.156 |
| GO | 52 | 19902 | São Francisco de Goiás | 5.823 |
| GO | 52 | 20009 | São João d'Aliança | 8.830 |
| GO | 52 | 20058 | São João da Paraúna | 1.672 |
| GO | 52 | 20108 | São Luís de Montes Belos | 27.793 |
| GO | 52 | 20157 | São Luíz do Norte | 4.456 |
| GO | 52 | 20207 | São Miguel do Araguaia | 23.142 |
| GO | 52 | 20264 | São Miguel do Passa Quatro | 3.856 |
| GO | 52 | 20280 | São Patrício | 2.144 |
| GO | 52 | 20405 | São Simão | 14.373 |
| GO | 52 | 20454 | Senador Canedo | 77.511 |
| GO | 52 | 20504 | Serranópolis | 7.813 |
| GO | 52 | 20603 | Silvânia | 19.154 |
| GO | 52 | 20686 | Simolândia | 7.331 |

| U.F. | | MUNICÍPIOS | | POPULAÇÃO ESTIMADA |
|---|---|---|---|---|
| SIGLA | COD | COD | NOMES | |
| GO | 52 | 20702 | Sítio d'Abadia | 3.510 |
| GO | 52 | 21007 | Taquaral de Goiás | 3.471 |
| GO | 52 | 21080 | Teresina de Goiás | 2.915 |
| GO | 52 | 21197 | Terezópolis de Goiás | 5.951 |
| GO | 52 | 21304 | Três Ranchos | 2.967 |
| GO | 52 | 21403 | Trindade | 104.979 |
| GO | 52 | 21452 | Trombas | 3.683 |
| GO | 52 | 21502 | Turvânia | 5.085 |
| GO | 52 | 21551 | Turvelândia | 4.068 |
| GO | 52 | 21577 | Uirapuru | 3.117 |
| GO | 52 | 21601 | Uruaçu | 34.470 |
| GO | 52 | 21700 | Uruana | 14.115 |
| GO | 52 | 21809 | Urutaí | 2.720 |
| GO | 52 | 21858 | Valparaíso de Goiás | 123.444 |
| GO | 52 | 21908 | Varjão | 3.806 |
| GO | 52 | 22005 | Vianópolis | 12.831 |
| GO | 52 | 22054 | Vicentinópolis | 6.093 |
| GO | 52 | 22203 | Vila Boa | 4.578 |
| GO | 52 | 22302 | Vila Propício | 5.359 |
| DF | 53 | 108 | Brasília | 2.606.885 |

**Fonte: IBGE.Diretoria de Pesquisas - DPE**
**Coordenação de População e Indicadores Socias - COPIS.**
